# hBN/TiO_2_ water-based nanolubricants: a solution for stick–slip mitigation in tribological applications†

**DOI:** 10.1039/d4na01049c

**Published:** 2025-02-06

**Authors:** Afshana Morshed, Fei Lin, Hui Wu, Zhao Xing, Sihai Jiao, Md Mahadi Hasan, Zhengyi Jiang

**Affiliations:** a School of Mechanical, Materials, Mechatronic and Biomedical Engineering, University of Wollongong Wollongong NSW 2522 Australia jiang@uow.edu.au; b Baosteel Research Institute (R&D Centre), Baoshan Iron & Steel Co., Ltd Shanghai 200431 China; c Department of Industrial and Production Engineering, American International University-Bangladesh Dhaka 1229 Bangladesh

## Abstract

In this study, the stick–slip behaviour of synthesised water-based nanolubricants was investigated *via* an Rtec ball-on-disk tribometer. By varying the lubricating conditions, including the concentration of hBN/TiO_2_ as nanoadditives, the tribological properties and lubrication mechanisms were analysed, especially the stick–slip phenomenon. Compared with dry and wet conditions, the hBN/TiO_2_ nanolubricant presented better efficiency in mitigating stick–slip and achieving friction stability. The relationship between anti-stick–slip properties and lubrication assisted in the selection of high-performance water-based nanoadditives. At a concentration of 0.5 wt% hBN/TiO_2_, the nanolubricant achieved the lowest average coefficient of friction (COF) of up to 78% compared to that under dry conditions. Additionally, the 0.5 wt% hBN/TiO_2_ nanolubricant showed an excellent anti-stick–slip effect, with the overall stick–slip phenomenon and threshold speed reduced by 77% and 72%, respectively, compared with those under dry conditions. Moreover, the findings indicate that the anti-stick–slip effect under wet conditions is superior to that under dry conditions. The mechanism of hBN/TiO_2_ nanoadditives in inhibiting stick–slip behaviour involves trapping wear debris and forming uniform tribofilms. It can be predicted that an optimal concentration of hBN/TiO_2_ (0.5 wt%) can eliminate the stick–slip phenomenon and effectively improve the friction state of the sliding interface.

## Abstract 2

Stick–slip phenomenon can be significantly reduced with the application of hBN/TiO_2_ water-based lubricants during sliding process, where hBN/TiO_2_ participates in trapping wear debris and forming well-distributed tribofilms between the contact zones.

## Graphical abstract

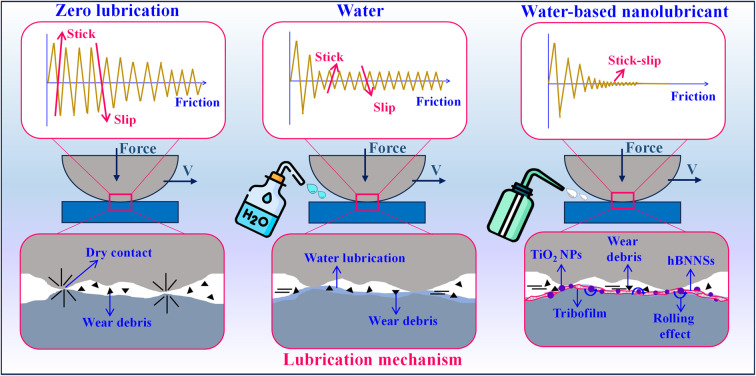

## 1 Introduction

With the ongoing advancements in mechanical devices for engineering applications, an in-depth understanding of friction and wear could contribute to energy savings, extending service life, and environmental protection. Certainly, a major aspect of their manufacturing process involves attempts to reduce friction-wear and eliminate the stick–slip phenomenon.^1–3^ The stick–slip phenomenon is generally observed under heavy loads and at low speeds when the sliding motion between two contact surfaces does not generate a constant friction motion. This motion alternates between adhesion and sliding in a periodic manner, leading to jerky and unstable vibratory friction or stick–slip behaviour.^4–6^ However, the stick–slip phenomenon repeats rapidly until the sliding speed reaches a threshold, where the friction begins to stabilise and ultimately disappears once the critical speed is reached.^4,7–9^ Additionally, this stick–slip behaviour presents tribological characteristics and typically occurs under conditions such as dry friction, boundary lubrication, and mixed lubrication rather than hydrodynamic lubrication.^10–13^ Shoaib *et al.*^14^ reported that the stick–slip characteristics of hydrogel lubricants are influenced by their microstructure, applied load, and sliding speed, which are governed by two different boundary lubrication mechanisms. Few studies have been conducted to understand the tribological behaviour, along with the stick–slip phenomenon. For example, Jeong *et al.*^15^ experimentally investigated the stick–slip phenomenon to study the influence of the slip regime transition on friction and wear conditions between steel surfaces under fretting conditions. Lu *et al.*^16^ showed that adding damping components made of styrene-butadiene rubber (SBR) or Mu-Cu damping alloy (DA) materials to friction systems significantly reduces friction-induced stick–slip behaviour. Notably, the stick–slip phenomenon is unacceptable because of the negative impact of stick–slip motion on the performance and functionality of different mechanical systems.^11,17–19^ The stick–slip phenomenon in certain mechanical systems, particularly in machine tools, must be eliminated or reduced; otherwise, it can lead to vibrations and unavoidable positioning errors. Thus, overcoming the undesirable effects of friction-wear and stick–slip during the sliding process is crucial.^1,20^

Lubricants such as neat oils and oil-in-water emulsions can effectively reduce friction and wear.^21^ Some researchers have investigated the influence of lubrication on stick–slip motion. The stick–slip behaviour of micro-dimpled and micro-bulged textured discs was explored by He *et al.*^11^ through friction dynamics tests *via* a pin-on-disc tribometer. Their findings indicated that, compared to untextured discs, both types of textured discs presented a lower intensity of stick–slip behaviour under oil lubrication, demonstrating their ability to inhibit stick–slip. However, the use of lubricants containing oil requires labour-intensive maintenance of the oil nozzles and has detrimental environmental effects, especially when they are ejected and burned.^21^ In light of this, water is an ideal substitute for these conventional oils, as it is clean, readily available and environmentally friendly. However, its low lubricity, poor anti-wear properties, and corrosive nature limit its application.^22–25^ In order to expand their potential applications, different nanoadditives have been added to improve the tribological and lubrication properties.^24–26^ These nanoadditives include metals, metal oxides, metal sulphides, non-metallic oxides, carbon-based materials, composites, and other nanoadditives such as nitrides, carbides, and metal salts.^21,27^ Some researchers have used water-based lubricants for tribological studies. Dong *et al.*^28^ studied the tribological behaviour of polymer materials in water lubrication, which demonstrated reduced stick–slip and frictional vibrations. In addition, there have been numerous studies on surfactants as lubricity enhancers for both oil-based^29,30^ and water-based^23–25,31,32^ systems. For example, Zhang *et al.*^26^ carried out tribological studies on steel surfaces using SDS (sodium dodecyl sulphate) in aqueous lubrication to correlate the boundary lubrication behaviour, particularly the stick–slip phenomenon. The results showed that with an increasing SDS concentration in the solution, both the static and kinetic friction decreased until stable sliding was attained. Although the aforementioned studies have proposed methods for reducing stick–slip behaviour, there is a lack of systematic investigations on the application of nanoparticles (NPs) as nanoadditives in water-based lubricants to influence the stick–slip behaviour.

In this study, hBN/TiO_2_ nanoadditives were incorporated into water-based lubricants as nanoadditives with the target of reducing the stick–slip phenomenon. The unique characteristics of hBN nanosheets (hBNNSs), including their high-temperature stability, chemical inertness, non-toxicity, and environmental friendliness, have gained attention in engineering applications, particularly when hBNNSs are used as nanoadditives in lubricants. Besides, our earlier studies have shown that TiO_2_ NPs possess considerable potential as nanoadditives in water-based lubricants for tribological applications.^33–35^ Furthermore, our previous tribological tests indicate that combining hBN and TiO_2_ significantly reduces friction and wear compared with using either hBN or TiO_2_ individually.^27^ Considering this, tribological tests were conducted using a ball-on-disk tribometer with hBN/TiO_2_ water-based nanolubricants to analyse the stick–slip phenomenon during the sliding process.

## 2 Experimental

### 2.1 Materials

**Fig. 1:**
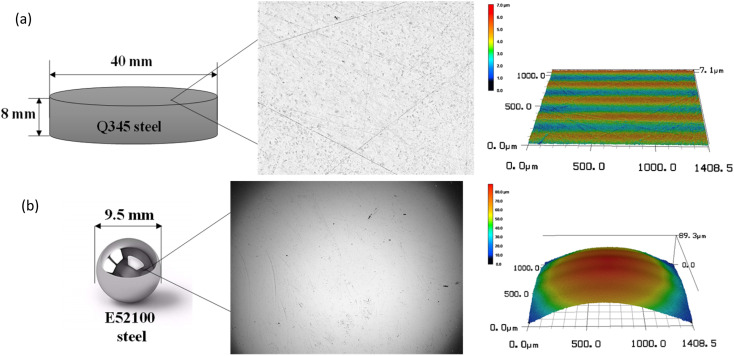
Schematic, optical image, and 3D profile of the (a) disk and (b) ball.^27^

The material used for the disk in the tribological tests was mild steel, specifically Q345, with a yield stress of 345 MPa and a Vickers hardness of 136 HV. The Q345 disk was machined to a diameter of 40.0 mm and a thickness of 8.0 mm. A surface roughness (*R*_a_) of 0.5 μm was obtained after grinding and polishing the disk surface. The ball was made of E52100 steel with a diameter of 9.5 mm. The surface roughness (*R*_a_) and Vickers hardness of the ball were 0.5 μm and 780 HV, respectively. Fig. 1 presents the schematic diagram, optical image and 3D profile of the disk and ball. The chemical compositions of the disk Q345 steel and ball E52100 steel are listed in Table 1. Both the ball and disk were cleaned with acetone prior to conducting the tribological tests to remove any residual contaminants from the machining process.

**Table 1:** Chemical compositions of the disk Q345 and ball E52100 (wt%)

Materials	C	Si	Mn	Mo	Ni	Cr	Cu	P + S	Nb + V + Ti
Disk-Q345	0.16	0.25	1.5	0.007	0.006	0.02	—	0.019	<0.02
Ball-E52100	1.0	0.25	0.35	0.10	—	1.5	0.30	<0.03	—

### 2.2 Lubricant preparation

Commercially available hBNNSs and P25 TiO_2_ NPs, along with glycerol and surfactant sodium dodecyl benzene sulfonate (SDBS), all sourced from Sigma-Aldrich were used to prepare the water-based lubricants. The hBNNSs exhibited a layered structure and flat sheet-like morphology with a honeycomb-like arrangement. P25 TiO_2_ NPs were composed of 75% anatase and 25% rutile and had a spherical shape. Glycerol is a viscous, odorless, and colourless liquid that enhances the viscosity and wettability of water-based lubricants.^36^ SDBS is an anionic surfactant with a hydrophilic head and a hydrophobic tail, which promotes wettability and viscosity by effectively dispersing NPs in water.^37^ The water-based nanolubricants were prepared by mechanically stirring all the additives into distilled water, followed by ultrasonication to ensure uniform dispersion. Table 2 provides the chemical composition of the synthesised nanolubricants and the concentrations of each nanoadditive. Dry and distilled water conditions were chosen as reference benchmarks for comparison purposes.

**Table 2:** Chemical compositions of different lubricating conditions

Lubrication type	Description
1	Dry
2	Water
3	0.25 wt% hBN + 0.25 wt% TiO_2_ + 10.0 wt% glycerol + 0.2 wt% SDBS
4	0.5 wt% hBN + 0.5 wt% TiO_2_ + 10.0 wt% glycerol + 0.2 wt% SDBS
5	1.0 wt% hBN + 1.0 wt% TiO_2_ + 10.0 wt% glycerol + 0.2 wt% SDBS
6	2.0 wt% hBN + 2.0 wt% TiO_2_ + 10.0 wt% glycerol + 0.2 wt% SDBS

### 2.3 Tribological tests

**Fig. 2:**
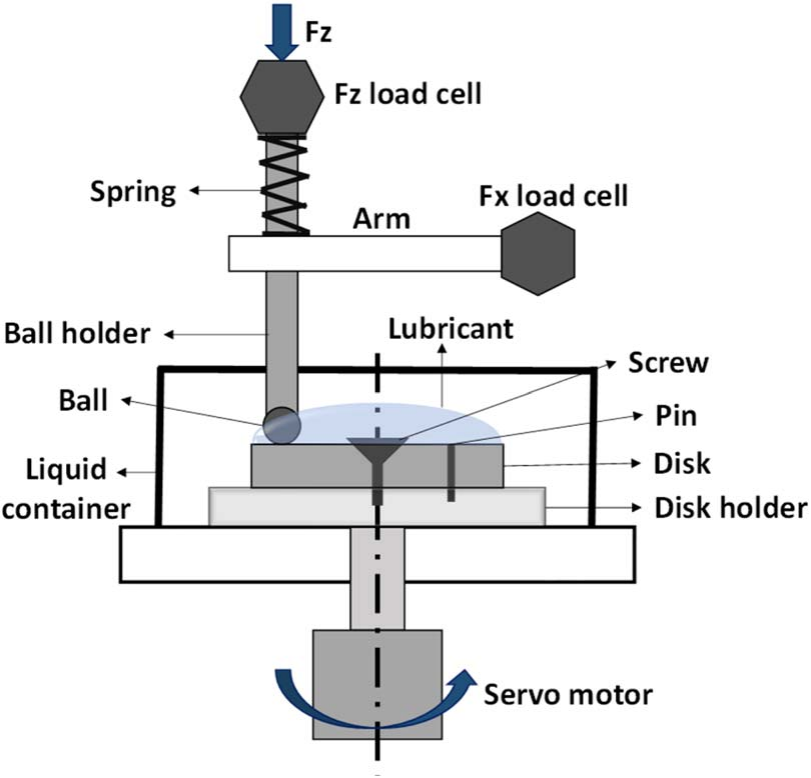
Schematic illustration of the ball-on-disk tribometer setup.^27^

An Rtec MFT-5000 multi-functional tribometer (Rtec Instruments, San Jose, CA, USA) equipped with a ball-on-disk configuration was used to conduct the tribological tests. Fig. 2 presents a schematic representation of the ball-on-disk setup at room temperature; more details were reported in our previous study.^27^ Prior to each test in the liquid lubrication setup, a consistent amount of 5.0 ml of lubricant was evenly applied to the disk surface. The E52100 steel ball was placed 14.0 mm from the centre of the disk, and a normal force of 50.0 N was applied using the ball on the disk. The rotational speed of the disk ranged from 0–25 rpm with an increment of 0.5 rpm every minute; thus, the total duration of each test was 50 min.

### 2.4 Appraisal techniques

The phase characterisation of the hBNNSs and P25 TiO_2_ NPs was conducted using X-ray diffraction (XRD) measurements on a Philips PW1730 conventional diffraction meter equipped with Cu–K radiation. The morphology of the hBNNSs and TiO_2_ NPs was observed using a JEOL JEM-ARM200F Transmission Electron Microscope (TEM) coupled with an energy-dispersive spectrometer (EDS). The wettability of the lubricants was evaluated *via* contact angle measurements on the surface of the Q345 steel disk surface using a Rame-hart 290 Goniometer (Rame-hart Instrument Co., Succasunna, NJ, USA). Each measurement was conducted five times to determine an average value. The disk wear tracks were observed under a JSM-7001F Scanning Electron Microscope (SEM) equipped with an energy dispersive spectrometer (EDS) to understand the lubrication mechanism.

## 3 Results and discussion

### 3.1 Nanoparticle characterisation

**Fig. 3:**
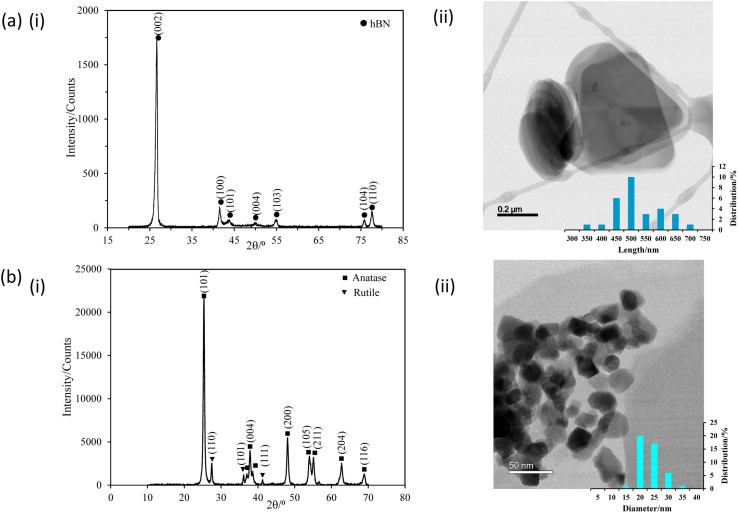
(i) XRD patterns and (ii) TEM images with size distributions of (a) hBNNSs and (b) P25 TiO_2_ NPs.

Fig. 3(a(i)) presents the XRD patterns of the hBN nanopowders, which are consistent with those of high-purity hexagonal boron nitride according to the standard JCPDS database (JCPDS no. 034-0421). The TEM image of hBNNSs in Fig. 3(a(ii)) shows thin plate-like morphologies and rounded surfaces, with an average length of 500 nm and a thickness of 50 nm.

Fig. 3(b(i)) displays the XRD patterns of the TiO_2_ nanopowders, revealing that the NPs consisted of typical P25 TiO_2_ with 75% anatase and 25% rutile, as confirmed by the XRD standard atlas (JCPDS 00-021-1272 and JCPDS 01-070-7347). The TEM image of P25 TiO_2_ NPs in Fig. 3(b(ii)) shows that the NPs have an average diameter of 20 nm and a nearly spherical shape.^33^ Further details regarding the characterisation of the NPs were covered in our previous studies.^27^

### 3.2 Stick–slip phenomenon

**Fig. 4:**
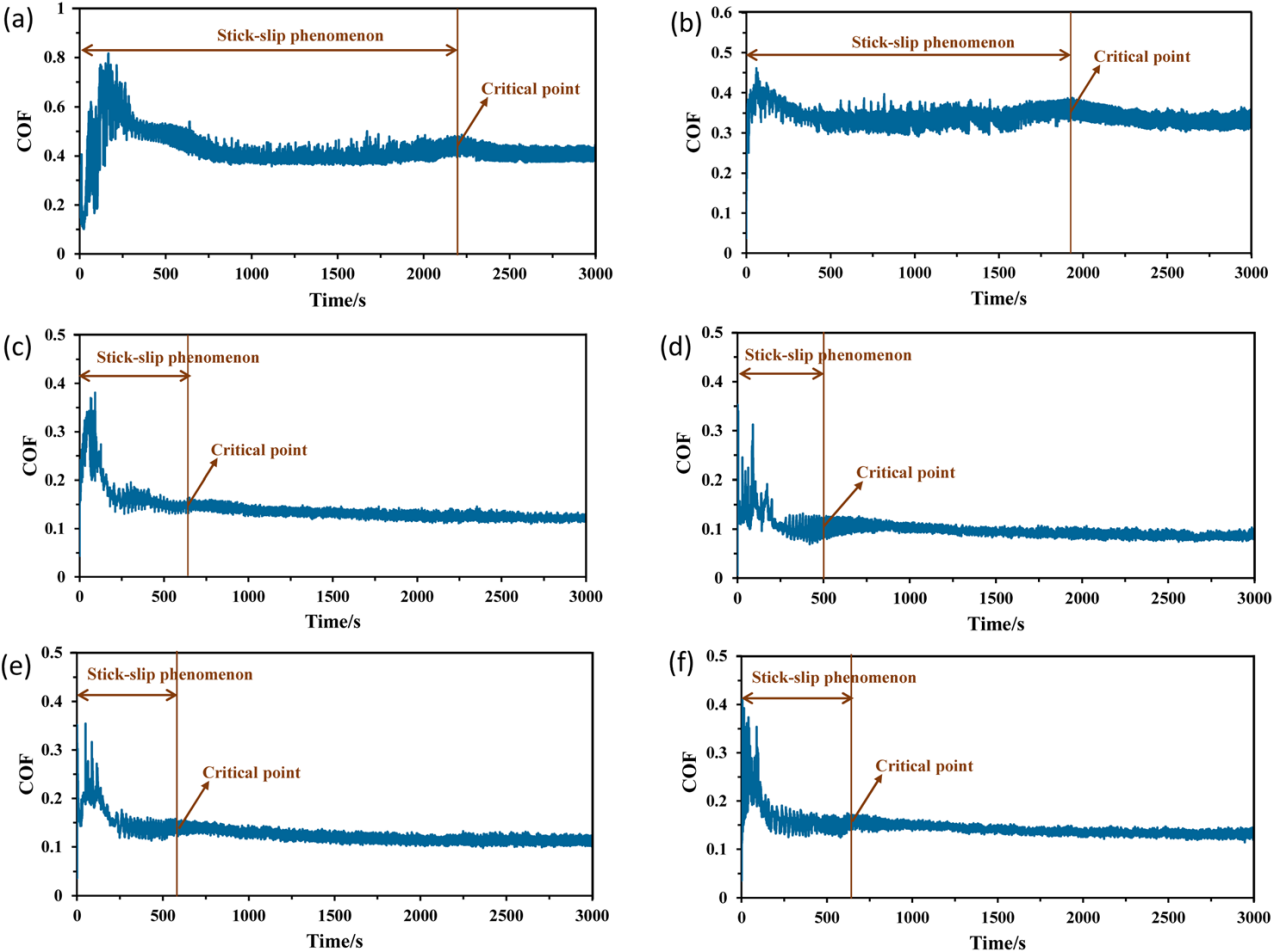
COF *vs.* time measured during tribological tests under different lubricating conditions, (a) dry, (b) water, (c) 0.25 wt% hBN/TiO_2_, (d) 0.5 wt% hBN/TiO_2_, (e) 1.0 wt% hBN/TiO_2_, and (f) 2.0 wt% hBN/TiO_2_, revealing the stick–slip phenomenon (50 N, 0–25 rpm).

**Fig. 5:**
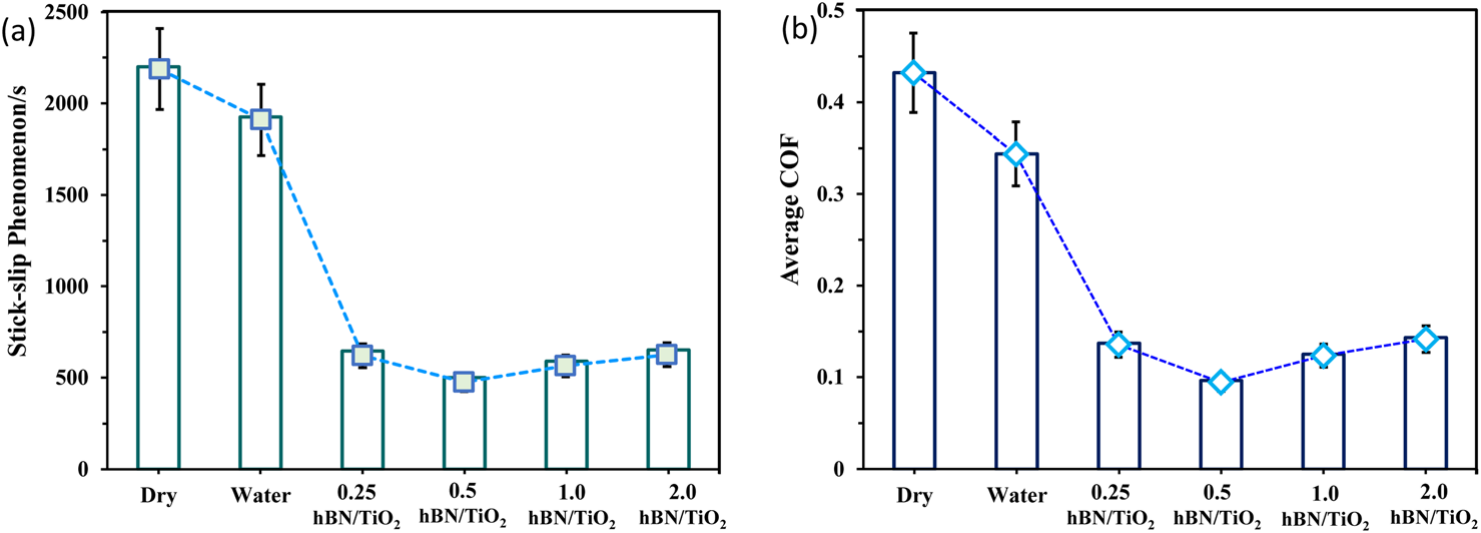
(a) Overall stick–slip phenomenon, and (b) average COF values under different lubrication conditions (50 N, 0–25 rpm, 50 min).

The stick–slip phenomenon typically occurs at the beginning of the sliding process due to the difference between the coefficients of kinetic and static friction. Fig. 4 shows the COF curves over time for different lubricating conditions to explore the relationship between the stick–slip behaviour and tribological properties. During the time interval from 0 to 3000 s, the COF increases sharply to a peak value before decreasing rapidly to a stable level. For dry and wet conditions, the stick–slip behaviour is relatively significant, whereas the water-based lubricants result in less pronounced stick–slip. The highest COF of up to 0.43 and longest stick–slip duration of 2200 s are observed under dry conditions. A comparatively lower COF is noted for wet conditions, with a slight increase in the COF curve observed before reaching the critical point and stabilising. In the case of all the water-based lubricants, the stick–slip behaviour is quite similar, with a less visible difference, representing a very high peak of the COF at the beginning and then settling soon. Fig. 5 presents the overall stick–slip phenomenon and the average COF for all the lubricating conditions. The average COF is calculated after 100 s, when a little prominent wear track is formed on the disk. Among the water-based lubricants, the average COF and stick–slip for 0.5 wt% hBN/TiO_2_ are lower than those for 0.25, 1.0 and 2.0 wt% hBN/TiO_2_ at different rotational speeds. Therefore, the lowest COF of 0.09 and the shortest total stick–slip phenomenon for 500 s are obtained for 0.5 wt% hBN/TiO_2_.

Fig. 4 shows that the COFs for all the conditions sharply decrease when the sliding speed increases with time and reach a certain stage. Thus, the COF fluctuations at specific speeds of 1, 5, and 10 rpm are analysed for each lubrication condition. The specific speeds are chosen to highlight the significant variations in the stick–slip phenomenon across the overall testing period. In order to further evaluate the intensity of stick–slip behaviour, friction force data during the stick–slip phase should be obtained. The friction force is calculated using the given formula.

**Fig. 6:**
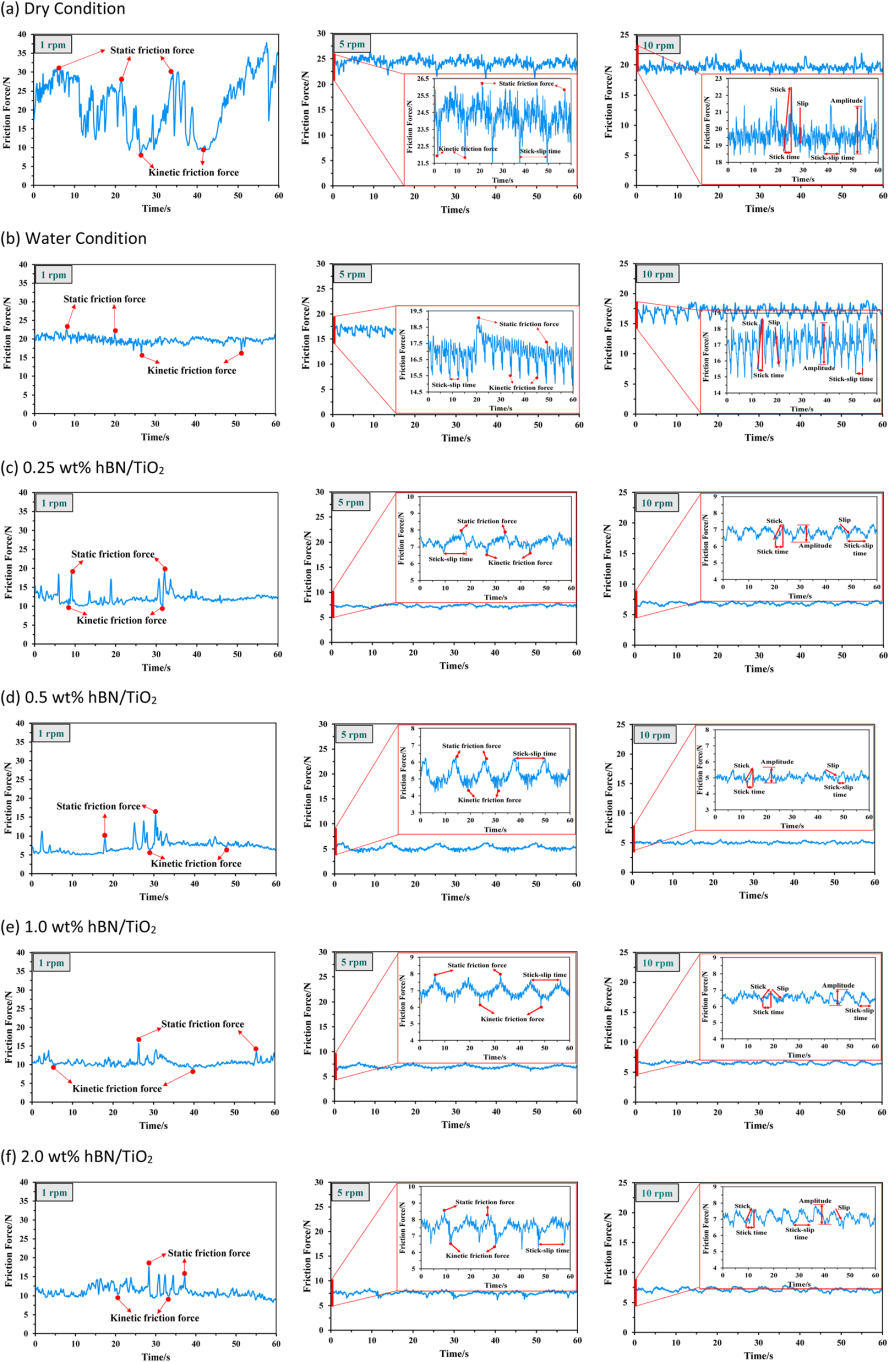
The variation of friction forces over time at 1, 5, and 10 rpm under 50 N force for different conditions, (a) dry, (b) water, (c) 0.25 wt% hBN/TiO_2_, (d) 0.5 wt% hBN/TiO_2_, (e) 1.0 wt% hBN/TiO_2_, and (f) 2.0 wt% hBN/TiO_2_.

1 Friction force (N), *F*_f_ = *F*_n_*μ*

where *F*_n_ is the load force (50 N) and *μ* is the COF. Fig. 6 represents both the kinetic and static friction forces with respect to time at specific speeds of 1, 5, and 10 rpm. The friction force gradually increases to the static friction force and then drops to the kinetic friction force.^38^ Based on the friction forces, the stick–slip amplitude and time are the key indicators of the stick–slip intensity.^39,40^ A greater intensity of stick–slip behaviour is typically associated with greater stick–slip amplitude and longer stick–slip time.^41^ At the beginning of the tribo-test at 1 rpm, fluctuations with a sawtooth appearance are observed for all six lubricating conditions (Fig. 6). The rotational displacement exhibits an irregular stick–slip motion, with the amplitude during the stick phase being significantly higher than that during the slip phase. In addition, overcoming the static friction force to initiate movement is more challenging, which indeed indicates that the stick–slip phenomenon is more likely to occur. However, the highest friction force, *i.e.*, static friction, decreases once sliding begins. When the speed increases to 5 rpm, a slight decrease in stick is observed, resulting in both the stick and slip phases having nearly equal amplitudes and times. The friction force during the stick phase does not increase linearly, as some minor slips are observed in this stick phase. During the period from 1140–1200 s, particularly at 10 rpm, the friction curve becomes steady and periodic. Both the frequency and amplitude of the stick–slip events decrease with increasing speed. Fig. 6(a) shows that the friction force amplitude is much greater for dry conditions than that for water and water-based lubricants. Even after reaching 10 rpm, the stick peaks are prominent under dry conditions. Fig. 6(b) shows that both the static and kinetic friction forces are still high after water is used, indicating a high stick–slip amplitude with a high frequency of stick–slip repetitions. In contrast, for water-based nanolubricants, comparatively reduced stick–slip and friction force curves are obtained. With the addition of hBN/TiO_2_, the friction force starts to decrease, reducing the static friction that needs to be overcome and thereby helping to control the stick–slip phenomenon. For 0.25 wt% hBN/TiO_2_ as shown in Fig. 6(c), a comparatively improved and steady friction force curve is achieved, with a decrease in both the stick–slip amplitude and time compared with those of the dry and wet conditions. As the hBN/TiO_2_ concentration reached 0.5 wt%, the friction force decreased even further (Fig. 6(d)). However, as shown in Fig. 6(e) and (f), the friction force begins to increase with the further increase in the hBN/TiO_2_ concentration to 1.0 and 2.0 wt%. Therefore, the most consistent and lowest static and kinetic friction forces are observed at a concentration of 0.5 wt% hBN/TiO_2_.

**Fig. 7:**
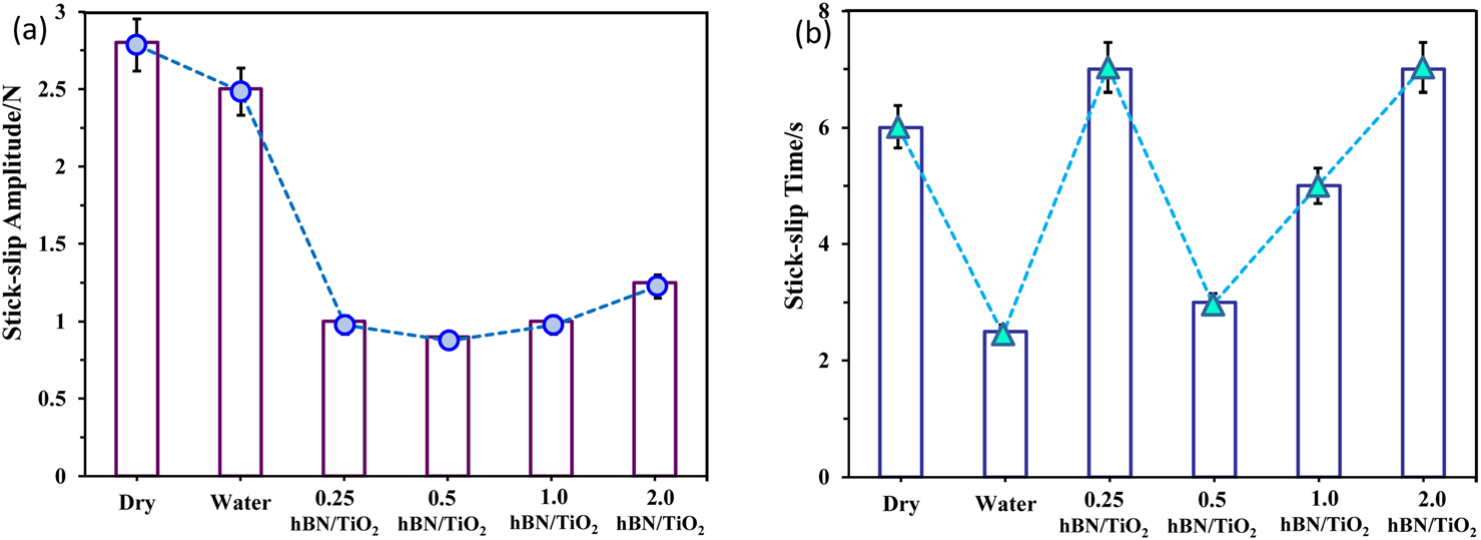
Intensities under different lubricating conditions at 10 rpm sliding speed under 50 N normal force: (a) stick–slip amplitude and (b) stick–slip time.

To compare the intensity of stick–slip behaviour across different lubricants accurately, Fig. 7 shows the stick–slip amplitude and time for all six conditions at 10 rpm. The stick–slip amplitude of the water-based lubricants is approximately half that of both water and dry sliding, which indicates that water-based lubricants exhibit superior anti-stick–slip properties compared with that under dry and wet conditions. Fig. 7(a) reveals the following amplitude relationship among the six lubricating conditions: dry > water > 2.0 wt% hBN/TiO_2_ > 0.25 wt% hBN/TiO_2_ > 1.0 wt% hBN/TiO_2_ > 0.5 wt% hBN/TiO_2_. The dry stick–slip has the highest amplitude of 2.8 N with a stick–slip time of 6 s, followed by water with 2.5 N for 2.5 s, whereas 0.5 wt% hBN/TiO_2_ has the lowest amplitude of 0.9 N for 3 s. As shown in Fig. 7(b), the 0.25 wt% and 2.0 wt% hBN/TiO_2_ samples exhibit the longest stick–slip time of 7 s and 1.0 wt% hBN/TiO_2_ has a stick–slip time of 5 s. Although the stick–slip time for dry and wet conditions is shorter than that for water-based lubrication, the frequency of stick–slip occurrence is significantly higher. It is noted that water-based lubricants have half the number of stick–slip repetitions compared with both dry and wet conditions. This phenomenon occurs because the rough surfaces in contact, with numerous small asperities and protrusions, interact and lead to frequent initiation and termination of stick–slip cycles. Each interaction between asperities causes a brief stick followed by a slip, resulting in high-frequency but short-duration events. Among the water-based lubricants, 0.5 wt% hBN/TiO_2_ resulted in shorter stick–slip times because of its effective participation during the sliding process. Therefore, it can be concluded that water-based lubricants help reduce the intensity of stick–slip behaviour, with 0.5 wt% hBN/TiO_2_ proving to be the most effective.

### 3.3 Threshold and critical speed

**Fig. 8:**
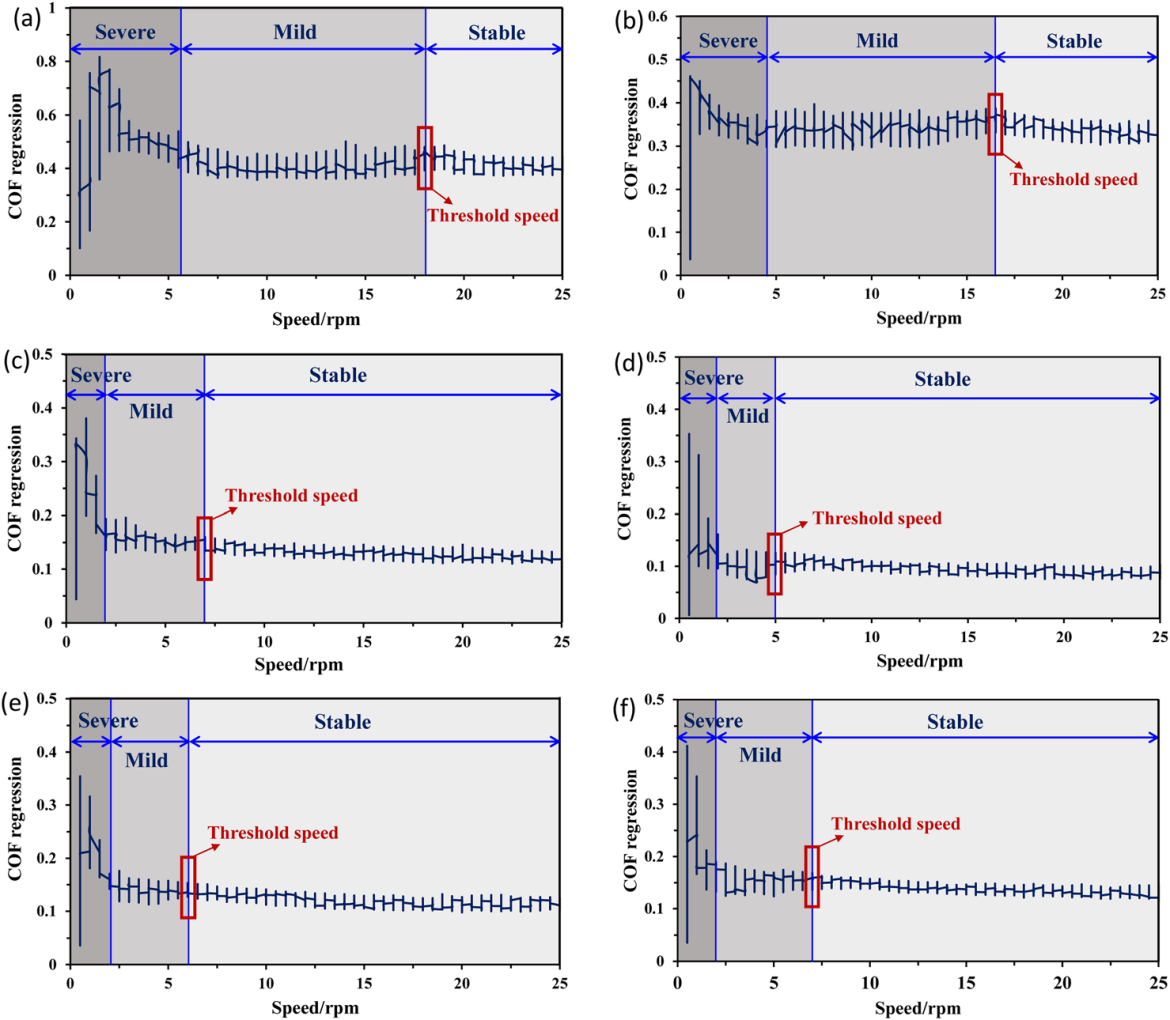
COF regression *vs.* speed under 50 N force for 50 min using different conditions: (a) dry, (b) water, (c) 0.25 wt% hBN/TiO_2_, (d) 0.5 wt% hBN/TiO_2_, (e) 1.0 wt% hBN/TiO_2_, and (f) 2.0 wt% hBN/TiO_2_.

Fig. 8 presents the COF regression for all the lubricating conditions under a load of 50 N and at a rotational speed ranging from 0–25 rpm. The COF regression displays randomness or quasiperiodicity, corresponding to different motion stages, as illustrated in Fig. 8. Thus, the motion of the samples can be categorised into three phases: severe, mild, and stable. The severe phase duration for water-based lubricants is approximately half of that for dry and wet conditions. These findings indicate that water-based lubricants can effectively suppress stick–slip behaviour. When the sliding speed is very low, for example, at 1 rpm, the stick–slip phenomenon is noted for all conditions, also observed in Fig. 6. The rotating speed plays a crucial role in influencing the stick–slip phenomenon.^42–44^ For different lubricating conditions, the ratio of the stick–slip regime to the continuous sliding regime changes with the rotating speed, which is defined by the threshold speed. At threshold speed, the stick–slip phenomenon ceases and stability is attained for different lubricants. The threshold speed varies with different lubricating conditions and rotating speeds, as demonstrated in Fig. 8. With the addition of nanoadditives to water-based lubricants, the threshold speed decreases. The COF regression shows considerable variation for dry conditions until the rotational speed reaches 18 rpm. In other words, the threshold speed for the stick–slip phenomenon, in this case, is 18 rpm. For wet conditions, the threshold speed depicted in Fig. 8(b) is slightly lower at 16.5 rpm, which aligns with the findings presented in Fig. 4. Compared with both dry and wet conditions, all water-based lubricants exhibit a noticeable reduction in threshold speed. The threshold speeds at which stick–slip behaviour stabilises for 0.25, 0.5, 1.0, and 2.0 wt% hBN/TiO_2_ water-based lubrication are 7, 5, 6, and 7 rpm, respectively.

**Fig. 9:**
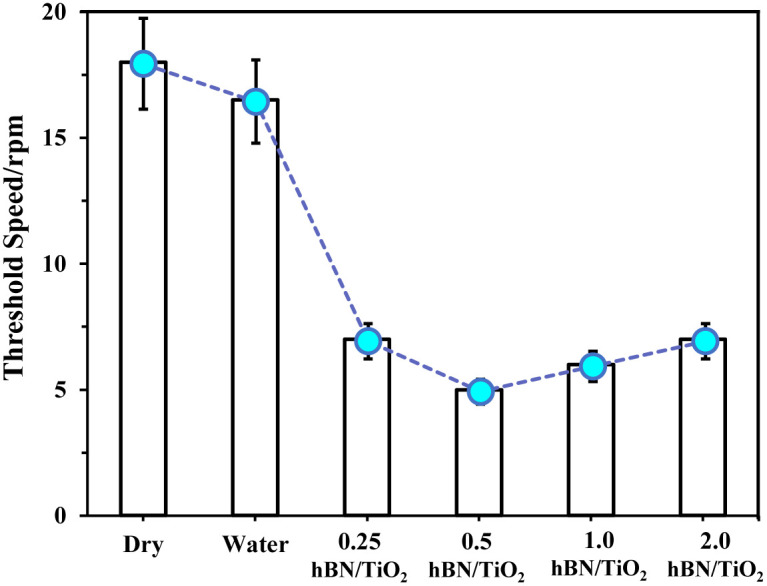
Threshold speed for the stick–slip phenomenon under different lubrication conditions (50 N, 0–25 rpm, 50 min).

COF regression for dry and wet conditions has been high at different speeds and with significant variations, which indicates that the stick–slip phenomenon persists for both dry and wet conditions within the speed range of 0–25 rpm. However, for water-based nanolubricants, the stick–slip phenomenon rarely occurs during the entire sliding process, and the degree of COF regression remains low after a specific stage is reached. When the hBN/TiO_2_ concentration is either insufficient or excessive, both the COF and the threshold speeds for the stick–slip phenomenon at different rotational speeds are higher. While the hBN/TiO_2_ concentration is 0.5 wt%, both the COF at different rotating speeds and the threshold speeds for the stick–slip phenomenon are the lowest. Therefore, the concentration of hBN/TiO_2_ has a minor influence on the threshold speed. Fig. 9 shows the threshold speed when the stick–slip phenomenon stabilises for different lubricating conditions. It can be concluded that, even at 5 rpm, stable sliding with a 0.09 average COF can be obtained with the 0.5 wt% hBN/TiO_2_ nanolubricant. Thus, 0.5 wt% hBN/TiO_2_ can be selected as the optimal concentration for stick–slip reduction.

**Fig. 10:**
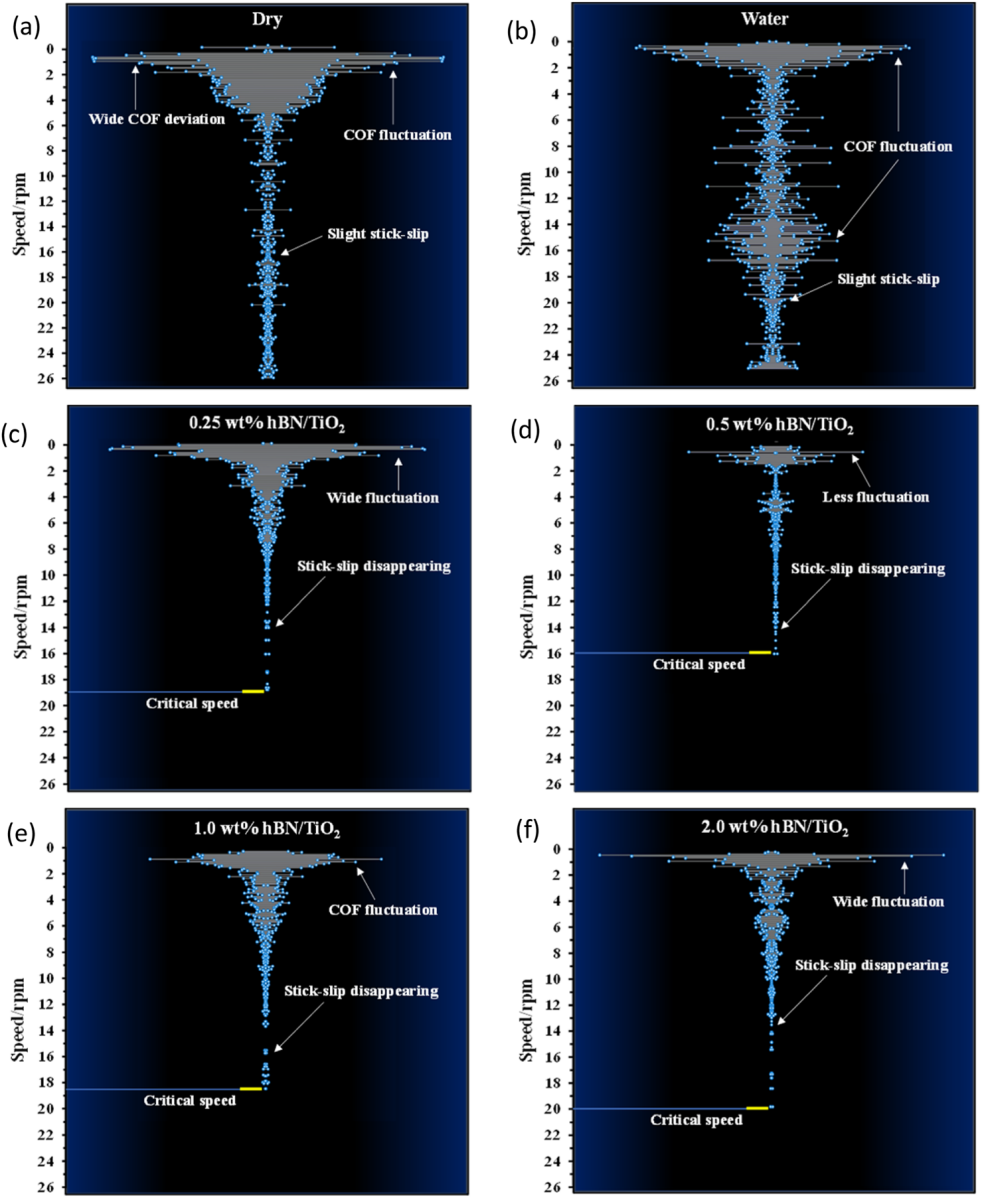
COF deviation *vs.* speed under 50 N for 50 min when lubricated with (a) dry, (b) water, (c) 0.25 wt% hBN/TiO_2_, (d) 0.5 wt% hBN/TiO_2_, (e) 1.0 wt% hBN/TiO_2_, and (f) 2.0 wt% hBN/TiO_2_.

Fig. 10 presents the COF deviation at varying speeds to obtain a better visual representation of the stick–slip motion. The funnel diagrams show the COF deviations for the six lubricating conditions across different speeds, revealing a decreasing trend as the rotational speed increases. According to the stick–slip phenomenon rules, continuous sliding occurs with respect to the motion state, and the stick–slip phenomenon disappears once the rotational speed exceeds the critical value. For dry conditions (Fig. 10(a)), before reaching the threshold speed at 18 rpm, the COF fluctuates regularly from a higher value to a lower value because of the periodic alternation between adhesion (static) and sliding in the stick–slip process. Although the fluctuation is reduced, slight stick–slip is observed until the end of the test. For wet conditions in Fig. 10(b), an increase in COF fluctuation is observed in the middle of the test at approximately 16 rpm, and the COF again decreases. This phenomenon occurs because the presence of water can initiate and accelerate corrosion, which degrades surface quality, produces abrasive particles, and leads to varying wear conditions. This ultimately causes an increase in COF fluctuations with increasing speed during long-duration tests. With the addition of nanoadditives, the dependence of COF deviation on the hBN/TiO_2_ concentration at different speeds was analysed, as shown in Fig. 10(c)–(f). Moreover, the COF deviations diminish once the rotational speed exceeds the critical speed, which is observed for all water-based lubricants. As shown in Fig. 10(c), with 0.25 wt% hBN/TiO_2_, the severe COF fluctuations reduce as the rotating speed increases, leading to the complete disappearance of stick–slip at 19 rpm. Thus, improved lubrication can effectively mitigate the stick–slip phenomenon. Increasing the hBN/TiO_2_ concentration to 0.5 wt% results in a shift to continuous sliding with less COF fluctuations, and the stick–slip disappears sooner at 16 rpm (Fig. 10(d)). For 1.0 and 2.0 wt% hBN/TiO_2_, wide COF fluctuations are observed until the critical speeds are attained at 18.5 and 20 rpm, respectively, as shown in Fig. 10(e) and (f). Generally, before reaching the critical speed, the COF deviation is relatively large due to the strong vibration of the friction force; however, the stick–slip disappears completely as the adhesion phenomenon diminishes with increasing speed. Accordingly, the COF deviation is reduced to a relatively lower value, which is characterised by a decrease in friction force, as shown in Fig. 6. Therefore, it is clear that water-based nanolubricants can successfully eradicate the stick–slip phenomenon.

### 3.4 Wettability

**Fig. 11:**
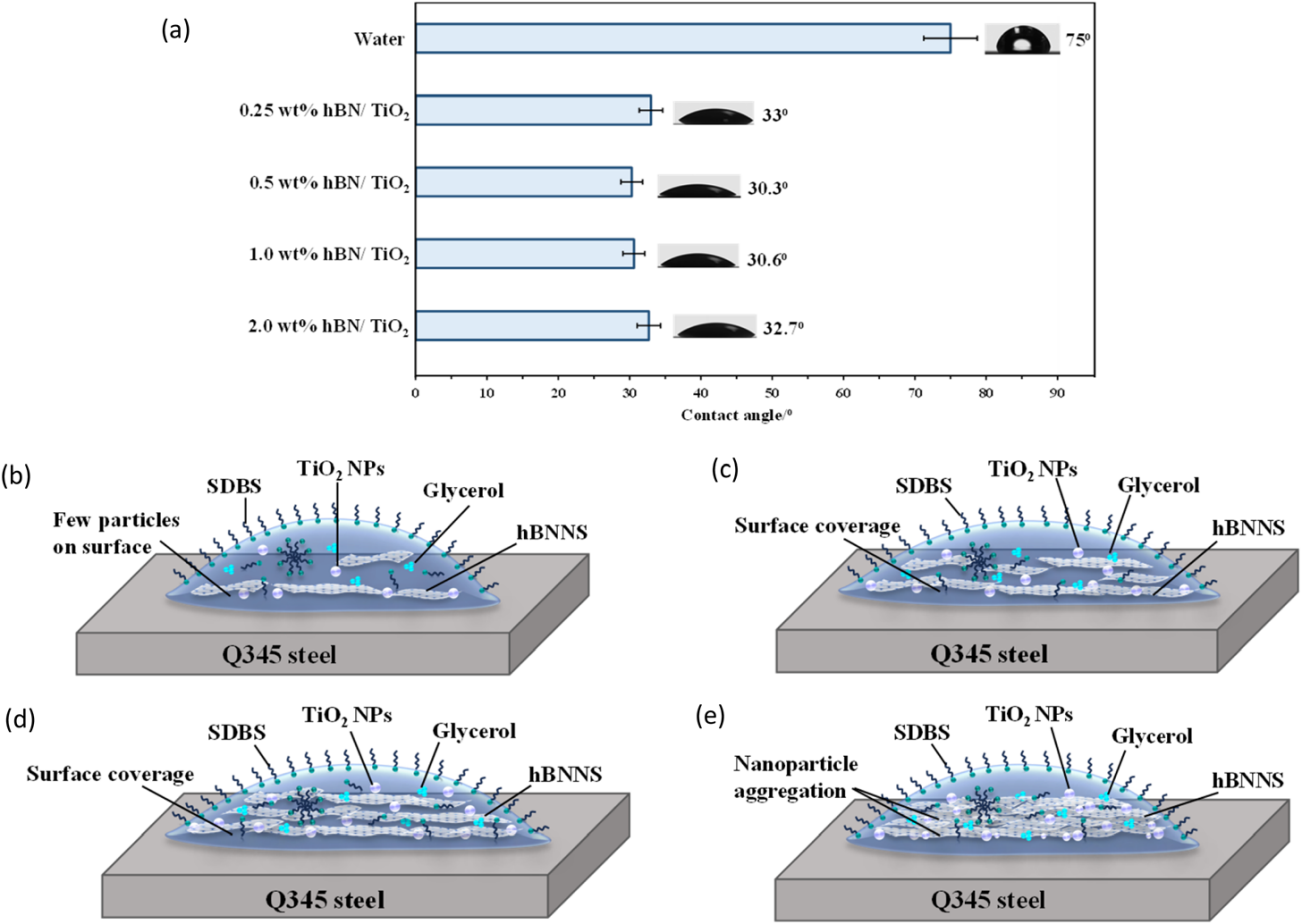
(a) Contact angle measured on the Q345 steel surface using various lubricants; the schematic diagram of contact angles when lubricated with (b) 0.25 wt% hBN/TiO_2_, (c) 0.5 wt% hBN/TiO_2_, (d) 1.0 wt% hBN/TiO_2_, and (e) 2.0 wt% hBN/TiO_2_.

The degree of liquid affinity and its ability to adhere to a solid surface are indicated by wettability.^45,46^ Proper wettability is essential for creating an adhesive and effective lubricating film on a metal surface by preventing direct contact between friction pairs.^47,48^ Wettability is typically assessed by contact angle measurements, and improved wettability is associated with a smaller contact angle.^49^ The contact angles for various lubricants were measured using the sessile drop method.^50,51^Fig. 11(a) presents the contact angles for water and water-based hBN/TiO_2_ droplets on Q345 steel. It is observed that the contact angles vary with the concentration of hBN/TiO_2_ in the water-based lubricant. The error bars indicate the range between the average contact angle for five similar droplets and the corresponding maximum and minimum angles in each series. Potential sources of error include measurement inaccuracies, substrate non-homogeneity, surface roughness, and local variations in the hBN/TiO_2_ concentration within the lubricant. For a droplet of pure water, the contact angle is significantly higher. The results show that pure water generates the largest contact angle (75°), indicating the poorest wettability among all tested lubricants. The addition of hBN/TiO_2_ to the water-based lubricant is capable of reducing the contact angle. Owing to its smaller size and higher surface area, hBN/TiO_2_ can effectively reduce surface tension, allowing improved wettability. For 0.25 wt% hBN/TiO_2_ in water, the contact angle decreased to 33°, which indicates that even a very low concentration of hBN/TiO_2_ can notably alter the surface-wetting properties. Additionally, the contact angle decreases further as the hBN/TiO_2_ concentration gradually increases, reaching 30.3° at 0.5 wt%. In contrast, 1.0 wt% hBN/TiO_2_ showed no significant change in the contact angle compared to 0.5 wt% (30.3° *versus* 30.6°). Initially, the contact angle decreases significantly with an increasing hBN/TiO_2_ concentration, reaching a minimum for 1.0% hBN/TiO_2_. Beyond this concentration, the contact angle starts to increase slightly. Thus, for 2.0 wt% hBN/TiO_2_, the contact angle increased slightly to 32.7°, as shown in Fig. 11(a). This indicates that the wettability between the lubricant and the disk surface improves with an increasing hBN/TiO_2_ concentration up to a certain point, after which it begins to decrease. The addition of 0.5 wt% hBN/TiO_2_ to water-based lubricants was the most effective for achieving optimal adhesion with the steel surface.

Enhanced wettability between lubricants and the disk surface increases the adhesive force of the lubricant, which optimises its effectiveness and influences the tribological behaviour.^52,53^Fig. 11(b)–(e) display the schematic diagrams of how the contact angle varies with the hBN/TiO_2_ concentration on Q345 steel samples. It can be observed that hBN/TiO_2_ acts as an anchor, which promotes adhesion between the lubricant and the sample surface. When hBN/TiO_2_ is more evenly distributed, providing proper surface coverage, the lubricant spreads uniformly across the sample surface, leading to enhanced wetting (Fig. 11(c) and (d)). However, at low concentrations, Fig. 11(a) shows that an insufficient amount of hBN/TiO_2_ leads to an uneven distribution, leaving parts of the surface exposed, which reduces surface coverage and affects wettability. As highlighted in our previous study,^54^ lubricants with improved wettability tend to accommodate an effective number of NPs adhering to the sample surface, thereby reducing stick–slip by lowering friction in the contact zone. Fig. 11(e) shows that at higher concentrations, excess hBN/TiO_2_ may not interact with the steel surface, either due to NP agglomeration or limited availability of adsorption sites. This limited interaction can obstruct further reduction in the contact angle and lead to a slight increase. Moreover, our previous study demonstrated the effect of SDBS on wettability.^37^ SDBS can significantly reduce the surface tension of water by orienting its hydrophilic head in water and its hydrophobic tail in air. A lower surface tension enables water to spread more easily on the steel surface, improving its ability to wet and cover the surface by distributing hBN/TiO_2_. To explain this phenomenon, the dissociation of SDBS in water creates phenyl sulfonic groups that adsorb around the NPs, increasing their net negative charge and enhancing repulsive forces between them.^55^ For hBN/TiO_2_, this behaviour helps the particles disperse in water. Hydrophobic or poorly water-soluble hBN/TiO_2_ can adsorb onto SDBS micelles, helping to stabilise the NPs by reducing their tendency to agglomerate.^51^ The hydrophilic heads of SDBS interact with water, while the hydrophobic tails can interact with the surface of hydrophobic hBN/TiO_2_. This dual interaction improves the overall wettability and dispersion of hBN/TiO_2_ in aqueous solution. However, as the concentration of hBN/TiO_2_ increases (Fig. 11(e)), they may start to aggregate and form clusters due to van der Waals forces or insufficient stabilisation even with the presence of SDBS. Thus, when the concentration of NPs exceeds the ability of surfactant SDBS to cover and stabilise their surfaces effectively, it leads to agglomeration, as the surfactant can no longer prevent particle–particle interactions.

### 3.5 SEM analysis

**Fig. 12:**
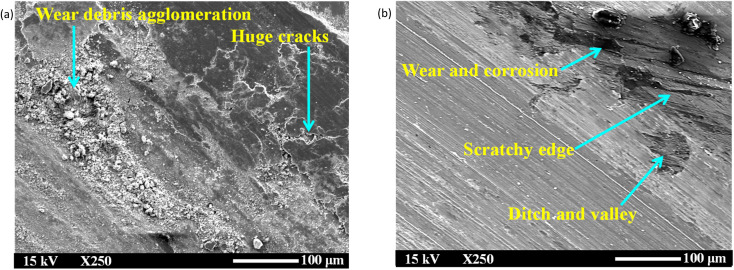
SEM images of the disk wear under (a) dry and (b) wet conditions at room temperature (50 N, 0–25 rpm, 50 min).

**Fig. 13:**
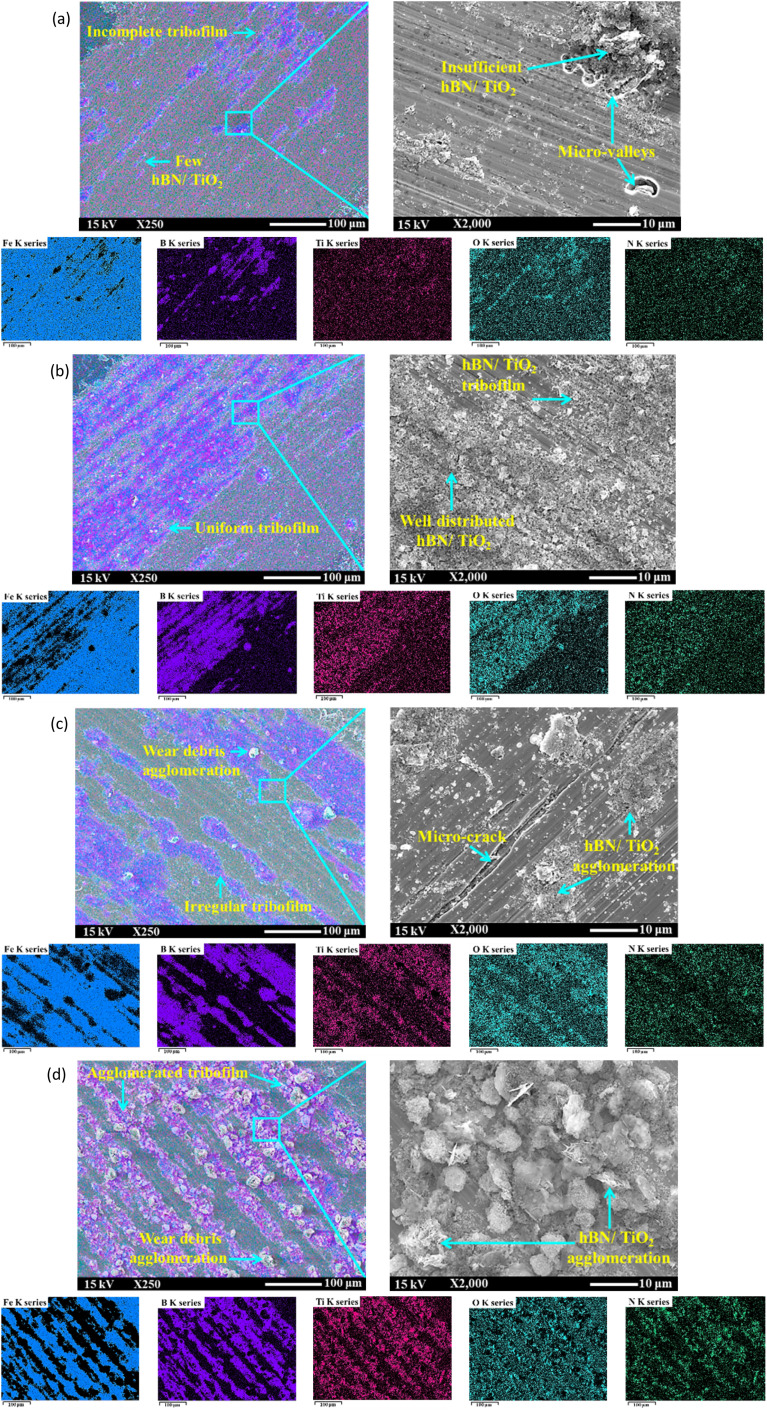
SEM images with EDS mappings of the disk wear under lubricating conditions of (a) 0.25 wt% hBN/TiO_2_, (b) 0.5 wt% hBN/TiO_2_, (c) 1.0 wt% hBN/TiO_2_, and (d) 2.0 wt% hBN/TiO_2_ (50 N, 0–25 rpm, 50 min).

Fig. 12 and 13 show SEM images of disk wear under various lubrication conditions. Specifically, in Fig. 12(a), significant cracks and substantial wear agglomeration are visible on the track under dry sliding. Due to lack of lubrication, the wear particles agglomerated on the wear track, resulting in the highest COF and intensified stick–slip behaviour (Fig. 4(a) and 10(a)). As shown in Fig. 12(b), multiple scratches and corroded wear particles along the edge of the wear track are observed under wet conditions, leading to a rough surface marked by several ditches and valleys. These corroded wear particles contributed to the sudden increase in COF fluctuations in the middle of the test, as evident in Fig. 10(b). This occurred because the water remained on the disk surface for an extended period, which facilitated corrosion and led to these fluctuations. However, water's ability to rinse the surface and remove little wear debris from the track slightly enhances tribological and stick–slip behaviour compared with dry conditions (Fig. 5).

**Fig. 14:**
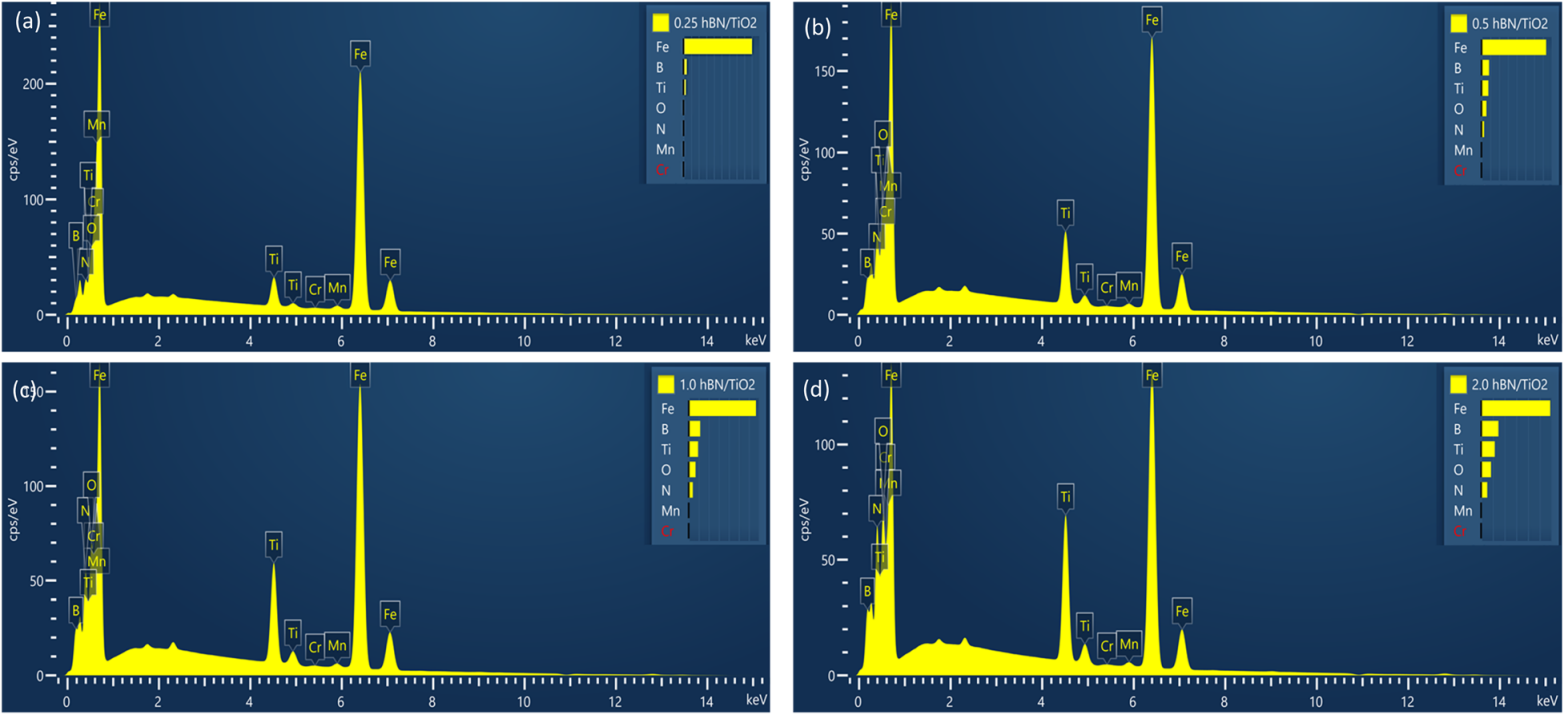
EDS spectra of disk wear when lubricated with (a) 0.25 wt% hBN/TiO_2_, (b) 0.5 wt% hBN/TiO_2_, (c) 1.0 wt% hBN/TiO_2_, and (d) 2.0 wt% hBN/TiO_2_ (50 N, 0–25 rpm, 50 min).

With the addition of nanoadditives, the friction stability significantly increases, and the intensity of the stick–slip phenomenon decreases. Fig. 13 and 14 present the EDS mappings and spectra of the wear tracks lubricated with four different hBN/TiO_2_ concentrations in water-based lubricants. The bright regions in the Fe, B, Ti, O and N mappings provide direct evidence supporting the performance of nanoadditives on the wear track. In addition, the EDS spectra indicate the quantity of nanoadditives involved during the tribological test, which corresponds to their concentration. When the hBN/TiO_2_ concentration is insufficient (0.25 wt%), the contact surfaces experience inadequate lubrication, resulting in a higher static friction force than that of other water-based lubricants, as shown in Fig. 6(c). The SEM images of the disk wear track in Fig. 13(a) show significant micro-valleys; few hBN/TiO_2_ resulted in poor and incomplete tribofilms, allowing direct metal–metal contact and leading to substantial wear. At a concentration of 0.5 wt% hBN/TiO_2_, the presence of sufficient additives led to a decrease in static friction force, causing the kinetic and static friction forces to become more similar, ultimately resulting in the earlier disappearance of stick–slip behaviour shown in Fig. 10(d). Besides, Fig. 13(b) illustrates that uniform tribofilms are formed on the wear track, characterised by well-distributed layers of hBN/TiO_2_. The lubricating film formed at a 0.5 wt% concentration ensures effective surface coverage, preventing direct contact between the sliding surfaces. When the concentration is increased to 1.0 wt%, the SEM images and EDS mappings in Fig. 13(c) reveal signs of an irregular tribofilm with the presence of wear clusters and slight NP agglomeration on the track. Although there is an improvement in surface coverage compared with 0.25 wt%, the excessive concentration can hinder the lubricant's effectiveness. The wear track shows some micro-valleys; this indicates that while lubrication is better than lower concentrations, it still does not match the performance of the 0.5 wt% concentration. Moreover, the results presented in Fig. 6(e) and 10(e) also indicate a comparatively higher stick–slip intensity and critical speed, respectively. With a further increase in the hBN/TiO_2_ concentration to 2.0 wt%, Fig. 13(d) reveals a highly agglomerated surface with substantial clusters of wear debris and hBN/TiO_2_. The dense clustering of hBN/TiO_2_ disrupts the ability of water-based lubricants to provide a consistent film, resulting in a pronounced stick–slip phenomenon. The overall surface condition appears rougher than that of both 0.5 wt% and 1.0 wt%, leading to higher COFs and wear rates. Due to uneven particle distribution and increased wettability, the lubrication performance of 2.0 wt% hBN/TiO_2_ is compromised. Therefore, the optimal concentration of the water-based nanolubricant for tribological applications is 0.5 wt% hBN/TiO_2_.

### 3.6 Lubrication mechanism

According to the Stribeck curve, there are three lubrication regimes, *i.e.* boundary, mixed, and hydrodynamic lubrication, categorised by friction variations relative to speed. The COF results in Fig. 4 clearly show that the lubrication type falls in the boundary lubrication regime, where the COF typically ranges from *μ* ≈ 0.08–0.15.^56^ Some studies have confirmed that all lubrication types (boundary, mixed, and hydrodynamic) can occur under starved conditions, and the COF serves as an effective indicator to detect changes in the lubricated state.^57,58^ It has long been understood that stick–slip occurs in the low relative speed regime, especially under boundary lubrication.^43,44^ Since there is sufficient time for adhesion to increase at low speed, this results in a high static friction force. As the speed increases, there is less time for adhesion to develop, causing the static friction force to decrease until reaching the critical speed. According to the relationship between friction stability and lubrication, stick–slip motion does not occur in hydrodynamic lubrication.^59^ Moreover, friction stability is linked to the interfacial properties of sliding surfaces, particularly the contact area.^60–62^ Several studies indicate that frictional instability is a key trigger for stick–slip behaviour.^40,63^ This is because stick–slip behaviour involves complex friction dynamics, which lead to contact instability, causing oscillations and intermittent movements.^64^

**Fig. 15:**
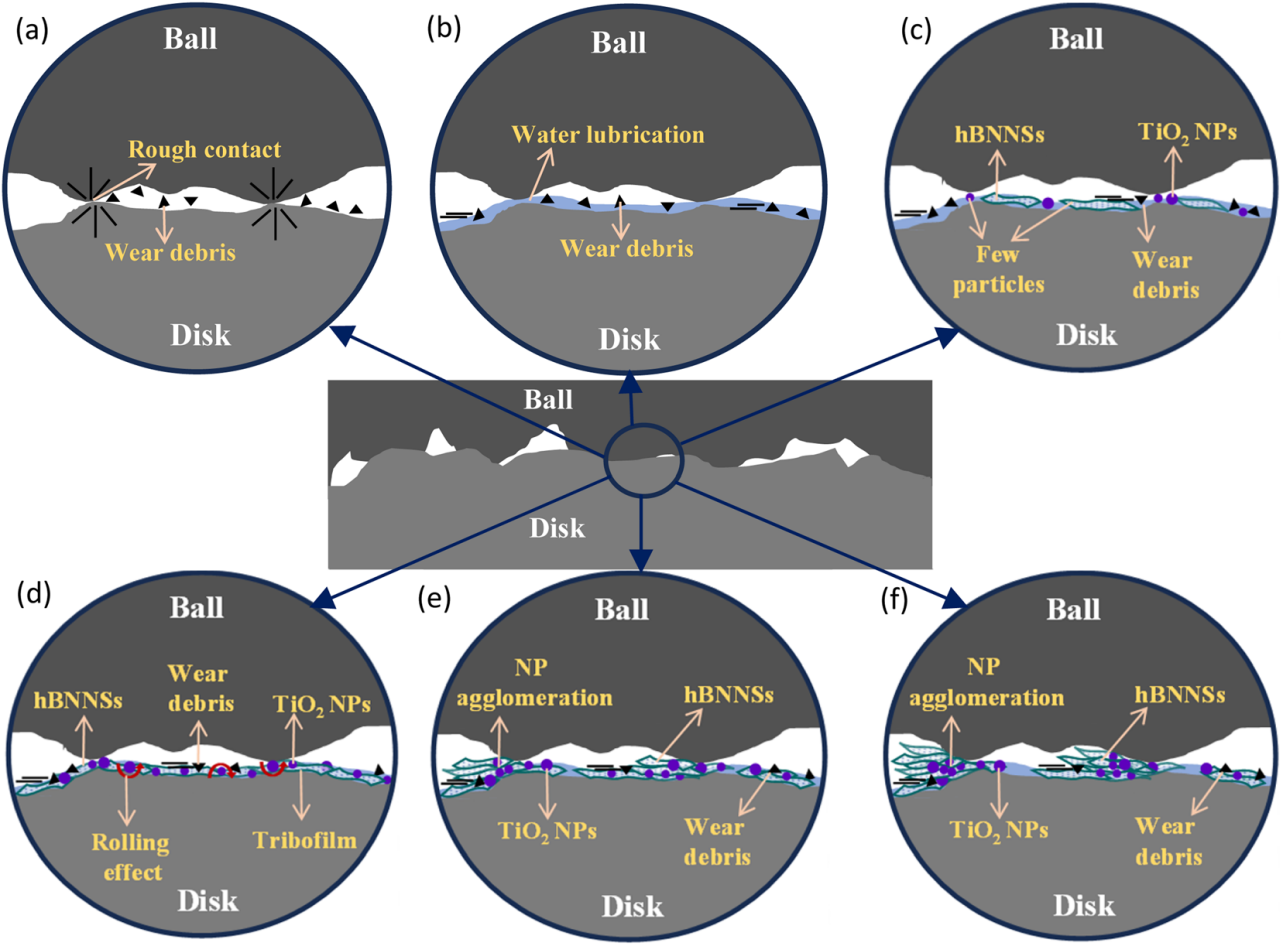
Schematics of lubrication mechanisms during ball-on-disk tribological testing under (a) dry, (b) water, (c) 0.25 wt% hBN/TiO_2_, (d) 0.5 wt% hBN/TiO_2_, (e) 1.0 wt% hBN/TiO_2_, and (f) 2.0 wt% hBN/TiO_2_.

In the boundary lubrication regime, friction and wear are influenced primarily by the interactions between the contact surface and the lubricating film.^65^Fig. 15 presents schematics of the lubrication mechanisms during ball-on-disk tests under different lubrication conditions. With zero lubrication, *i.e.*, for dry conditions in Fig. 15(a), a very rough contact between the sliding surfaces is observed, along with the significant amount of wear debris in Fig. 12(a). Fig. 4(b) shows that when pure water was used, the COF ranged from 0.5 to 0.6, as no adsorbed lubricating layer formed. Water is capable of carrying a small amount of wear debris from the contact area, as shown in Fig. 15(b), but cannot provide proper lubrication without the presence of NPs to fill the cracks and valleys (Fig. 12(b)). Water-based lubricants show significantly less wear, with few visible micro-valleys and micro-cracks in Fig. 13(a) and (c), respectively. With the addition of hBN/TiO_2_, the stick–slip behaviour and COF are significantly influenced by the adsorption of hBN/TiO_2_ on the Q345 steel surface. When the ball and disk come into contact and move relative to each other, a layer of lubricating film exists between them. However, at low concentrations (0.25 wt% hBN/TiO_2_), the load-bearing capability and ability to reduce contact between the asperities of the sliding surfaces are limited. Fig. 15(c) shows that the limited surface coverage by NPs results in non-uniform tribofilm formation at the contact zones, which is also evident in Fig. 11(b) and 13(a). On the other hand, it is clear from Fig. 11(c) that at 0.5 wt% hBN/TiO_2_, there is an ideal balance between the number of nanoadditives and their distribution in the lubricant.^66–68^Fig. 15(d) and 13(b) show that the synergistic effect of hBN and TiO_2_ contributes to tribofilm formation and rolling and mending effects by effectively minimising stick–slip; thus, a well-maintained surface with minimal wear features is obtained.^69,70^ As smooth sliding is facilitated by this protective film, the COF is significantly lower than that of other concentrations (Fig. 4(d) and 6(d)), while maintaining sufficient strength to resist wear. A further increase in the hBN/TiO_2_ concentration to 1.0 and 2.0 wt% led to significant NP agglomeration, as evident from Fig. 15(e) and (f), respectively. Instead of forming a uniform lubricating layer, these aggregated NP clusters disrupt smooth sliding, leading to a higher friction force and longer stick–slip as shown in Fig. 6 and 10, respectively. Thus, the comparison of lubrication mechanisms across different hBN/TiO_2_ concentrations revealed that all four types of lubricated surfaces effectively inhibited the stick–slip phenomenon. The inhibition effectiveness and lubrication mechanisms of the nanoadditives are ranked as follows: 0.5 wt%, 0.25 wt%, 1.0 wt%, and 2.0 wt% hBN/TiO_2_.

## 4 Conclusions

The stick–slip phenomenon, a common issue in tribological applications, especially at low speeds and high loads, should be mitigated to improve the operational efficiency of mechanical parts. Ball-on-disk tests were carried out to examine the influence of different lubrication conditions on the stick–slip phenomenon, along with the gradual increase in the sliding speed over time. The following conclusions can be summarised:

(1) Water-based lubricants exhibited superior anti-stick–slip properties compared with both dry and wet conditions. Compared with other water-based lubricants, the 0.5 wt% hBN/TiO_2_ lubricant was the first to eliminate stick–slip behaviour and start stable sliding.

(2) At a sliding speed of 10 rpm, the stick–slip amplitudes for dry, water, 0.25, 0.5, 1.0 and 2.0 wt% hBN/TiO_2_ water-based lubricants were 2.8, 2.5, 1, 0.9, 1 and, 1.25 N and the stick–slip times were 6, 2.5, 7, 3, 5 and, 7 s, respectively.

(3) The test process was categorised into three phases: severe, mild, and stable. Throughout these phases, both the kinetic and static friction progressively decreased until continuous sliding was achieved. For different lubricating conditions, the threshold speeds at which the friction reached stability are as follows: 0.5 wt% (5 rpm) > 1.0 wt% (6 rpm) > 0.25 and 2.0 wt% hBN/TiO_2_ (7 rpm)> water (16.5 rpm)> dry (18 rpm).

(4) Critical speed was attained for water-based lubrication, where the stick–slip phenomenon disappeared. However, for both dry and wet conditions, slight stick–slip persisted throughout the test, and critical speed was never achieved.

(5) Wettability of water (contact angle 75°) improved with the addition of 0.25, 0.5, 1.0, and 2.0 wt% hBN/TiO_2_ combined with glycerol and SDBS which reduced the contact angles to 33°, 30.3°, 30.6°, and 32° (hydrophilic, 0° < *θ* < 90°), respectively.

(6) This study provides a perspective on the lubrication mechanism in the boundary lubrication regime, highlighting the influence of water-based nanolubricants on the stick–slip phenomenon. The lubrication mechanisms of hBN/TiO_2_ were governed primarily by the rolling and mending effects, along with the protective film formation and synergistic effects of the hBNNSs and TiO_2_ NPs.

## Data availability

The data supporting this article have been included as part of the ESI.†

## Author contributions

Afshana Morshed: methodology, formal analysis, investigation, writing—original draft preparation, writing—review and editing, visualization. Fei Lin: methodology, investigation. Hui Wu: conceptualization, writing—review and editing. Zhao Xing: conceptualization, resources, project administration. Sihai Jiao: conceptualization, resources, project administration. Md Mahadi Hasan: writing—review and editing, supervision. Zhengyi Jiang: conceptualization, writing—review and editing, supervision, funding acquisition.

## Conflicts of interest

There are no conflicts to declare.

## Supplementary Material

NA-OLF-D4NA01049C-s001
