# Supplementary material for: hBN/TiO2 water-based nanolubricants: a solution for stick–slip mitigation in tribological applications
Source: Nanoscale Adv. 2025 Feb 6;7(7):1972–88. doi: 10.1039/d4na01049c (PMC11826335; doi:10.1039/d4na01049c)
Supplement: NA-007-D4NA01049C-s001 [file NA-007-D4NA01049C-s001.pdf]

Supplementary Information (SI) for Nanoscale Advances.
This journal is © The Royal Society of Chemistry 2025

Data availability statement

Supplementary Information

hBN/TiO_2 water-based nanolubricants: A solution for stick-slip mitigation in tribological applications

Afshana Morshed[a], Fei Lin[a], Hui Wu[a], Zhao Xing[b], Sihai Jiao[b], Md Mahadi Hasan*[c] and Zhengyi Jiang*[a]

[a.]School of Mechanical, Materials, Mechatronic and Biomedical Engineering, University of Wollongong, Wollongong, NSW 2522, Australia.

[b.]Baosteel Research Institute (R&D Centre), Baoshan Iron & Steel Co., Ltd., Shanghai 200431, China.

[c.]Department of Industrial and Production Engineering, American International University, Dhaka 1229, Bangladesh.

Table S1. In-situ co-efficient of friction (COF) for six lubrication conditions

Time/s	Speed/rpm	Dry (COF)	Water (COF)	0.25 wt% hBN/TiO_2 (COF)	0.5 wt% hBN/TiO_2 (COF)	1.0 wt% hBN/TiO_2 (COF)	2.0 wt% hBN/TiO_2 (COF)
0.148	0.5	0.169875	0.03720447	0.04383186	0.04140505	0.0359528	0.03567162
0.248	0.5	0.170262	0.08618055	0.1015394	0.00754394	0.07256539	0.084853
0.348	0.5	0.171403	0.1273328	0.1451846	0.01427503	0.1075508	0.1285745
0.448	0.5	0.172034	0.1827732	0.1682818	0.0349243	0.1514512	0.149557
0.548	0.5	0.166453	0.2254635	0.1966105	0.0608214	0.1975542	0.1707029
0.648	0.5	0.159528	0.2354499	0.2232577	0.0901772	0.2383539	0.1998357
0.748	0.5	0.156372	0.199322	0.2376616	0.1216115	0.2576663	0.2290301
0.848	0.5	0.156019	0.1981921	0.2077842	0.1427711	0.2869552	0.2547375
0.948	0.5	0.156196	0.201892	0.1806703	0.1615757	0.3061355	0.2688274
1.048	0.5	0.155381	0.2080752	0.1722466	0.1863078	0.313594	0.2695174
1.148	0.5	0.153887	0.2203405	0.1652724	0.2086114	0.327616	0.2416418
1.248	0.5	0.153342	0.2359625	0.1598928	0.2291109	0.3381395	0.2095063
1.348	0.5	0.151927	0.2524202	0.1563404	0.2524293	0.3516238	0.1605685
1.448	0.5	0.149738	0.2646069	0.1555785	0.2774727	0.331099	0.1297062
1.548	0.5	0.149654	0.273682	0.1547903	0.3045744	0.21099	0.1162877
1.648	0.5	0.150079	0.2782291	0.1549017	0.3322759	0.190268	0.1123351
1.748	0.5	0.152325	0.282114	0.1558294	0.3527351	0.1917353	0.116024
1.848	0.5	0.158012	0.2889227	0.1565127	0.3365405	0.194778	0.1262059
1.948	0.5	0.162977	0.2918603	0.1564763	0.3159971	0.2001162	0.1410111
2.048	0.5	0.165775	0.2762815	0.1567573	0.3098213	0.2033233	0.1576559
2.148	0.5	0.168215	0.2301531	0.1634431	0.2750441	0.2056266	0.1704689
2.248	0.5	0.166135	0.1967208	0.1655571	0.2429674	0.2053346	0.178746
2.348	0.5	0.164474	0.1881265	0.1687595	0.2207405	0.2091921	0.1799225
2.448	0.5	0.164239	0.1875253	0.1735857	0.1990592	0.2219495	0.1751869
2.548	0.5	0.16675	0.1958851	0.1837933	0.17064	0.2318383	0.1556263
2.648	0.5	0.168525	0.2088239	0.1883089	0.1435125	0.2303845	0.1343531
2.748	0.5	0.167988	0.2273259	0.1883863	0.1361255	0.2301898	0.1265936
2.848	0.5	0.16267	0.2515944	0.1843437	0.1347825	0.2415019	0.1239147
2.948	0.5	0.155382	0.2784223	0.1739392	0.1328793	0.2571808	0.119964
3.048	0.5	0.151505	0.2985317	0.1715434	0.1294339	0.2722677	0.1183815
3.148	0.5	0.149861	0.2799855	0.1885605	0.127337	0.2853977	0.1189675
3.248	0.5	0.153422	0.2911657	0.206832	0.1156072	0.2904429	0.1307886
3.348	0.5	0.161891	0.3138817	0.2236264	0.1131099	0.2973688	0.1583009
3.448	0.5	0.169638	0.3254739	0.2377219	0.1322384	0.3019963	0.1898928
3.548	0.5	0.178455	0.3308476	0.2407447	0.1626463	0.2780698	0.2187772
3.648	0.5	0.188158	0.317419	0.2199028	0.1951018	0.242729	0.2491819
3.748	0.5	0.194177	0.2860059	0.1949911	0.2260022	0.2224528	0.2773867
3.848	0.5	0.196708	0.2981781	0.1798469	0.2611658	0.2152276	0.3044633
3.948	0.5	0.194794	0.3068579	0.1728107	0.2919941	0.2197951	0.330361
4.048	0.5	0.190051	0.3293092	0.1720687	0.322068	0.2310802	0.3576197
4.148	0.5	0.18249	0.3506587	0.1718888	0.339886	0.2365523	0.3788005
4.248	0.5	0.17312	0.3578043	0.1724355	0.318445	0.2402483	0.3959232
4.348	0.5	0.165148	0.3549297	0.1697957	0.3010686	0.25065	0.4114968
4.448	0.5	0.16189	0.3462855	0.1679693	0.2781478	0.2479864	0.369368
4.548	0.5	0.163524	0.3458715	0.1685743	0.2500922	0.2320433	0.1875232
4.648	0.5	0.168783	0.346628	0.1672836	0.2341237	0.2284833	0.1643263
4.748	0.5	0.17734	0.3485428	0.1663151	0.2283646	0.2200145	0.1561399
4.848	0.5	0.184978	0.3542514	0.1635837	0.1928275	0.2179598	0.1532356
4.948	0.5	0.190175	0.3523388	0.1629097	0.1485444	0.2103256	0.1505835
5.048	0.5	0.196215	0.3448286	0.1632083	0.1470922	0.2071756	0.150087
5.148	0.5	0.199929	0.3431389	0.1630521	0.1440111	0.2123482	0.1514578
5.248	0.5	0.200882	0.3353791	0.1608828	0.1365426	0.2131444	0.153163
5.348	0.5	0.201518	0.3393208	0.1589048	0.1328862	0.21228	0.1542813
5.448	0.5	0.201049	0.3539045	0.1604137	0.1317819	0.2146207	0.1536455
5.548	0.5	0.203413	0.3635709	0.1626853	0.1311066	0.2198762	0.1464367
5.648	0.5	0.206939	0.3495999	0.1653824	0.13024	0.2165666	0.1390971
5.748	0.5	0.210223	0.3422913	0.1652304	0.130562	0.2196626	0.1360935
5.848	0.5	0.214698	0.3169172	0.1645538	0.1281468	0.2245071	0.1357707
5.948	0.5	0.215875	0.3112615	0.1651977	0.1279946	0.2266121	0.1377849
6.048	0.5	0.213014	0.3205439	0.16579	0.1316118	0.2177824	0.1392262
6.148	0.5	0.211388	0.3257235	0.168955	0.1346288	0.2144889	0.1394538
6.248	0.5	0.209232	0.3405355	0.172113	0.1389208	0.2145186	0.1360112
6.348	0.5	0.208112	0.3412349	0.1704126	0.1365279	0.218444	0.140708

6.448	0.5	0.20926	0.3378876	0.172557	0.1290484	0.2264057	0.1496317
6.548	0.5	0.22034	0.3402832	0.1784682	0.1214487	0.2323858	0.1503129
6.648	0.5	0.231415	0.3435868	0.1780344	0.1205019	0.2356738	0.1457822
6.748	0.5	0.236989	0.3455239	0.1774348	0.1177075	0.2205242	0.1438811
6.848	0.5	0.242111	0.3418775	0.1776638	0.1163347	0.2026573	0.1404109
6.948	0.5	0.243949	0.3566244	0.1797747	0.1159607	0.1937965	0.1362658
7.048	0.5	0.244252	0.3630309	0.1842953	0.1178985	0.1926169	0.1409748
7.148	0.5	0.258086	0.3683548	0.1882724	0.120879	0.1951014	0.1514867
7.248	0.5	0.276473	0.3691206	0.1914518	0.1195659	0.1967459	0.1615954
7.348	0.5	0.28658	0.3749797	0.1928832	0.1148502	0.1959621	0.1622829
7.448	0.5	0.286518	0.380331	0.1912866	0.1114883	0.1999614	0.1786544
7.548	0.5	0.294331	0.373505	0.1891887	0.112425	0.2002622	0.2000058
7.648	0.5	0.324225	0.3683987	0.1895787	0.1140062	0.1927634	0.2177938
7.748	0.5	0.353335	0.3531012	0.1917011	0.1167429	0.1891592	0.2358124
7.848	0.5	0.363257	0.3515916	0.1958009	0.1188324	0.1902506	0.2615786
7.948	0.5	0.346346	0.3484595	0.1982411	0.1169137	0.1893957	0.2903681
8.048	0.5	0.341216	0.3470427	0.1996398	0.1173748	0.1852921	0.3168338
8.148	0.5	0.338421	0.3490092	0.2000593	0.1188598	0.1812381	0.3378941
8.248	0.5	0.338324	0.3512666	0.2029528	0.1184235	0.1799601	0.3390955
8.348	0.5	0.326265	0.3517985	0.2050101	0.1162308	0.1789474	0.2997281
8.448	0.5	0.222649	0.3433154	0.2043146	0.1164532	0.179408	0.1891221
8.548	0.5	0.123971	0.3361861	0.2037592	0.1154096	0.1780587	0.1747895
8.648	0.5	0.1227	0.3425371	0.2026718	0.1152871	0.1767691	0.1666976
8.748	0.5	0.13539	0.3435889	0.2004477	0.1123068	0.1764865	0.1609275
8.848	0.5	0.150348	0.3453633	0.1997547	0.1126734	0.1745461	0.1563382
8.948	0.5	0.165025	0.3504268	0.2006493	0.1149668	0.1781183	0.1560514
9.048	0.5	0.181523	0.3610838	0.2029379	0.1155012	0.1825162	0.1570814
9.148	0.5	0.196571	0.3655762	0.208063	0.1179765	0.1880463	0.1558989
9.248	0.5	0.218882	0.3646734	0.2131364	0.1212175	0.1898298	0.1557063
9.348	0.5	0.247709	0.3644015	0.2113442	0.1248668	0.1857578	0.1553815
9.448	0.5	0.275392	0.3699617	0.2117142	0.123507	0.180168	0.1543393
9.548	0.5	0.298774	0.3767449	0.2157714	0.1219615	0.1759009	0.153566
9.648	0.5	0.322145	0.3814143	0.2213547	0.1213845	0.1707734	0.1543403
9.748	0.5	0.338006	0.3834718	0.224479	0.119712	0.1692807	0.1550756
9.848	0.5	0.352124	0.3800514	0.2193506	0.1193698	0.1684646	0.1545391
9.948	0.5	0.376101	0.3657704	0.2246585	0.1195312	0.1691719	0.1537903
10.048	0.5	0.3854	0.3521908	0.2334386	0.1190442	0.1690568	0.1537363
10.148	0.5	0.402001	0.3535578	0.2335611	0.1184798	0.1669474	0.1533937
10.248	0.5	0.405742	0.3575826	0.2342609	0.1206998	0.1684157	0.1533574
10.348	0.5	0.310077	0.366883	0.2286218	0.1219742	0.1724258	0.1530999
10.448	0.5	0.292009	0.3766357	0.2209429	0.1248775	0.1751266	0.1528351
10.548	0.5	0.315938	0.3673073	0.2146738	0.1333584	0.1773507	0.1519329
10.648	0.5	0.34214	0.359522	0.2125319	0.1382022	0.179867	0.1512487
10.748	0.5	0.362793	0.3518171	0.2118114	0.1383357	0.1776523	0.1526347
10.848	0.5	0.357918	0.3479856	0.211291	0.1348991	0.1719702	0.1542284
10.948	0.5	0.304392	0.3537004	0.2097312	0.1311353	0.1711166	0.155973
11.048	0.5	0.26399	0.3597611	0.2069069	0.1329924	0.1686195	0.1556319
11.148	0.5	0.255416	0.3530029	0.2023062	0.1350848	0.1659519	0.1547259
11.248	0.5	0.238223	0.3582848	0.1998523	0.135825	0.1666802	0.1532119
11.348	0.5	0.218383	0.3580303	0.1975975	0.1334069	0.1652463	0.1503747
11.448	0.5	0.198831	0.3479117	0.1971112	0.127496	0.1655995	0.151326
11.548	0.5	0.19116	0.3435553	0.198996	0.1238507	0.1639275	0.1535821
11.648	0.5	0.189382	0.3387936	0.2036994	0.1265884	0.1643835	0.1555273
11.748	0.5	0.187328	0.3407186	0.2103828	0.1282259	0.1620677	0.1552642
11.848	0.5	0.184789	0.3358679	0.2171299	0.1267707	0.1613329	0.1546534
11.948	0.5	0.187084	0.3409497	0.2202903	0.1250834	0.1624926	0.1534861
12.048	0.5	0.192705	0.3462386	0.2205348	0.11854	0.1628213	0.1532795
12.148	0.5	0.196474	0.3425283	0.2162308	0.1202444	0.1601463	0.1522108
12.248	0.5	0.197573	0.3371117	0.2167907	0.1189061	0.1608927	0.1528047
12.348	0.5	0.196911	0.3162895	0.2172968	0.1142839	0.1617937	0.1543472
12.448	0.5	0.197719	0.3048131	0.216337	0.1159622	0.1603235	0.1573427
12.548	0.5	0.197254	0.3123004	0.216538	0.1175249	0.1615244	0.1611163
12.648	0.5	0.193797	0.3213695	0.2126599	0.1184945	0.1623304	0.1590861
12.748	0.5	0.194656	0.3242481	0.2138219	0.118659	0.1591652	0.1556777
12.848	0.5	0.194253	0.3198846	0.2192114	0.1181287	0.1588645	0.1541509
12.948	0.5	0.1899	0.323034	0.2271331	0.114759	0.1585336	0.1616731
13.048	0.5	0.181195	0.3359155	0.233806	0.1150558	0.1592973	0.1776129
13.148	0.5	0.175537	0.347726	0.2400071	0.1153728	0.1631104	0.1965179
13.248	0.5	0.173981	0.3552278	0.2493819	0.1157771	0.1683222	0.2154098
13.348	0.5	0.173732	0.3547493	0.2577339	0.1241994	0.1690242	0.2396393
13.448	0.5	0.171143	0.3176352	0.2632115	0.1308844	0.1717794	0.2673189
13.548	0.5	0.166467	0.2559955	0.2575008	0.1361111	0.1739138	0.2978981
13.648	0.5	0.159422	0.2509538	0.2381107	0.1340095	0.1746078	0.3276812
13.748	0.5	0.155089	0.2587045	0.2089408	0.1328786	0.1743148	0.3560143
13.848	0.5	0.146118	0.2734883	0.196522	0.1348258	0.1714494	0.3804888
13.948	0.5	0.134973	0.2843895	0.1965379	0.1439258	0.1688975	0.3911872
14.048	0.5	0.126055	0.2920323	0.201787	0.1525311	0.1683778	0.32037
14.148	0.5	0.121481	0.2937063	0.2144769	0.1568724	0.1717819	0.2161213
14.248	0.5	0.119096	0.2992732	0.2270877	0.1492843	0.1691332	0.1988966
14.348	0.5	0.117435	0.2943881	0.2396482	0.1446481	0.1621357	0.1931515
14.448	0.5	0.115635	0.2994359	0.2476082	0.1415932	0.1569902	0.1908188
14.548	0.5	0.114362	0.3042982	0.2494858	0.1421494	0.1569532	0.1920777
14.648	0.5	0.113185	0.3039996	0.2402751	0.1365936	0.156113	0.1941518
14.748	0.5	0.11274	0.3071149	0.2272339	0.1328661	0.1548535	0.1916936
14.848	0.5	0.115805	0.3096371	0.2242551	0.1280007	0.1552755	0.1956617
14.948	0.5	0.120718	0.3138632	0.2266022	0.1247334	0.1521697	0.1943059
15.048	0.5	0.127107	0.3126329	0.2296098	0.1237005	0.1516718	0.1942342
15.148	0.5	0.133369	0.3126931	0.2331688	0.1218582	0.151252	0.1889942
15.248	0.5	0.138904	0.315734	0.2379715	0.1232205	0.1510449	0.182146
15.348	0.5	0.144354	0.3184828	0.2467669	0.1241071	0.1554064	0.1743205
15.448	0.5	0.149706	0.3205357	0.2546431	0.1220222	0.1579677	0.1715563
15.548	0.5	0.153466	0.3236074	0.2551623	0.1215574	0.1552335	0.1697798
15.648	0.5	0.156359	0.3259003	0.2514961	0.1224521	0.1566106	0.1663564
15.748	0.5	0.158956	0.3331991	0.2439134	0.1232833	0.1573513	0.165893
15.848	0.5	0.157818	0.3292621	0.2401211	0.1207472	0.1550974	0.1638031

15.948	0.5	0.156381	0.305363	0.238424	0.1193399	0.1542729	0.1614838
16.048	0.5	0.158466	0.3000356	0.2319172	0.1171185	0.159798	0.1629055
16.148	0.5	0.161787	0.3140341	0.2282091	0.113834	0.1621393	0.1645144
16.248	0.5	0.165826	0.3326774	0.2346837	0.1137747	0.161686	0.1638216
16.348	0.5	0.168599	0.3288997	0.2469707	0.1125773	0.171322	0.1640263
16.448	0.5	0.16807	0.3063861	0.2613524	0.1109784	0.1676503	0.1650142
16.548	0.5	0.169198	0.3191174	0.2740695	0.1106668	0.168931	0.1667807
16.648	0.5	0.169491	0.3382392	0.2718534	0.1116969	0.1616131	0.1692267
16.748	0.5	0.166418	0.3527798	0.267753	0.1147893	0.1589271	0.1731831
16.848	0.5	0.168248	0.3570739	0.2598267	0.1186387	0.1586686	0.1734138
16.948	0.5	0.171584	0.3496595	0.2350603	0.1177061	0.159015	0.1724644
17.048	0.5	0.173431	0.3373148	0.2223644	0.1196757	0.15747	0.1713352
17.148	0.5	0.170136	0.3456622	0.2356422	0.1208701	0.1547189	0.1681487
17.248	0.5	0.165246	0.3585304	0.2557165	0.1214324	0.1549095	0.1661569
17.348	0.5	0.15873	0.3654354	0.2619434	0.1207941	0.15603	0.1682401
17.448	0.5	0.15241	0.3706113	0.2623543	0.1220443	0.1552439	0.169339
17.548	0.5	0.147582	0.3724976	0.261246	0.1233315	0.154681	0.1696135
17.648	0.5	0.138671	0.3742788	0.2456786	0.1248243	0.1583415	0.1692119
17.748	0.5	0.127524	0.375562	0.2183575	0.1240901	0.1598386	0.1679748
17.848	0.5	0.123207	0.3804817	0.2058107	0.1196588	0.1560829	0.1708137
17.948	0.5	0.126351	0.3850727	0.2093532	0.1172549	0.1546301	0.1710157
18.048	0.5	0.13128	0.382478	0.2236219	0.1164934	0.155116	0.1705639
18.148	0.5	0.136137	0.3713649	0.2408298	0.116173	0.1564305	0.16723
18.248	0.5	0.139128	0.3749569	0.251021	0.1150968	0.1560035	0.1662806
18.348	0.5	0.139696	0.3777446	0.2608593	0.1147681	0.1535156	0.1647567
18.448	0.5	0.137553	0.3838554	0.2656272	0.1160047	0.1529808	0.1634562
18.548	0.5	0.135941	0.3837439	0.2558328	0.1153449	0.1532195	0.1641713
18.648	0.5	0.136871	0.3797734	0.2516871	0.1161071	0.1516122	0.1676266
18.748	0.5	0.13778	0.3724519	0.2527143	0.1159691	0.1499194	0.1759135
18.848	0.5	0.137936	0.3700345	0.2561979	0.1170131	0.149122	0.1821027
18.948	0.5	0.138195	0.3834071	0.2580843	0.1180087	0.1499573	0.1790347
19.048	0.5	0.139384	0.3738611	0.2619021	0.1145565	0.1473851	0.1747772
19.148	0.5	0.139977	0.3741066	0.2650295	0.1116392	0.1469774	0.1737613
19.248	0.5	0.139249	0.3767085	0.2628797	0.1098941	0.144419	0.1807262
19.348	0.5	0.132222	0.3895989	0.2580012	0.1105423	0.1426348	0.1853964
19.448	0.5	0.125859	0.3886023	0.2516871	0.1133592	0.1441186	0.1847877
19.548	0.5	0.120119	0.3916229	0.2459197	0.1146864	0.1504818	0.1846729
19.648	0.5	0.115157	0.3934168	0.2370639	0.1169626	0.1569372	0.1823014
19.748	0.5	0.111176	0.3955137	0.23265	0.1175183	0.1554334	0.1808844
19.848	0.5	0.1089	0.3907399	0.2399773	0.120846	0.1552892	0.1867348
19.948	0.5	0.108363	0.3908371	0.2522291	0.1204891	0.1557263	0.1858014
20.048	0.5	0.107888	0.3923602	0.2588166	0.1180534	0.1542653	0.1867359
20.148	0.5	0.10783	0.3902051	0.2671098	0.117835	0.1572571	0.1917331
20.248	0.5	0.108851	0.3894973	0.275951	0.1204952	0.1565288	0.1980627
20.348	0.5	0.109404	0.3925407	0.2718303	0.1202273	0.1588994	0.1976608
20.448	0.5	0.109064	0.3883355	0.2579616	0.1239502	0.1637267	0.1930028
20.548	0.5	0.108914	0.38862	0.2428253	0.1268513	0.165475	0.1904564
20.648	0.5	0.111117	0.3864257	0.2323494	0.12763	0.1592673	0.1836811
20.748	0.5	0.113186	0.3856005	0.2331578	0.1301755	0.1628017	0.1804954
20.848	0.5	0.112372	0.3871602	0.2419174	0.1287029	0.1618442	0.1774381
20.948	0.5	0.112438	0.3837406	0.2564955	0.1317548	0.1624912	0.1769954
21.048	0.5	0.114501	0.3812666	0.2739331	0.1279924	0.157768	0.1782483
21.148	0.5	0.119133	0.3909111	0.2886231	0.1246057	0.1628704	0.1819371
21.248	0.5	0.124158	0.3912281	0.2939844	0.12244	0.1650845	0.1861583
21.348	0.5	0.129005	0.3786102	0.2912137	0.121479	0.1568666	0.187144
21.448	0.5	0.130725	0.3800254	0.2611479	0.1175065	0.1548164	0.1914974
21.548	0.5	0.12619	0.375863	0.2496034	0.1169343	0.160641	0.1938716
21.648	0.5	0.119825	0.3718952	0.2442157	0.1172716	0.1630906	0.1962268
21.748	0.5	0.115388	0.3755778	0.2462913	0.1171661	0.1635829	0.2050885
21.848	0.5	0.112652	0.3799959	0.2599505	0.1189615	0.161782	0.2063835
21.948	0.5	0.110506	0.3824551	0.2701555	0.1189603	0.1598384	0.2013057
22.048	0.5	0.109178	0.3862944	0.2774272	0.1166402	0.1659569	0.19149
22.148	0.5	0.107274	0.3840533	0.275683	0.1221961	0.1697377	0.1858447
22.248	0.5	0.105559	0.3772877	0.265031	0.1289584	0.1670091	0.1813505
22.348	0.5	0.103345	0.3712301	0.2647073	0.1297957	0.1607375	0.1736991
22.448	0.5	0.102635	0.3734669	0.2630528	0.1254483	0.1563739	0.1712742
22.548	0.5	0.103517	0.3783917	0.2695593	0.1229786	0.1531216	0.1660155
22.648	0.5	0.105528	0.3809048	0.279296	0.1190902	0.1518091	0.1672141
22.748	0.5	0.106354	0.3827171	0.2844083	0.1147614	0.1526592	0.169299
22.848	0.5	0.111248	0.3800698	0.2900026	0.1149762	0.1520414	0.1698398
22.948	0.5	0.117332	0.3777363	0.287007	0.1190076	0.151559	0.1738191
23.048	0.5	0.120488	0.3844402	0.2586378	0.1216571	0.1497929	0.1746554
23.148	0.5	0.123697	0.391084	0.2389621	0.1173257	0.1490687	0.1748061
23.248	0.5	0.126562	0.389005	0.2350745	0.1155455	0.151723	0.1830931
23.348	0.5	0.129622	0.3935293	0.2438901	0.1161924	0.149828	0.1952772
23.448	0.5	0.130476	0.3969265	0.2570112	0.1183538	0.1522083	0.2099667
23.548	0.5	0.130149	0.3914916	0.2715999	0.1198554	0.1521532	0.2278123
23.648	0.5	0.128904	0.3890365	0.2796278	0.1227316	0.1503933	0.2419495
23.748	0.5	0.125163	0.3867881	0.2853935	0.1215397	0.1498294	0.2617639
23.848	0.5	0.11893	0.3877107	0.2754492	0.1206228	0.1479219	0.2830877
23.948	0.5	0.112812	0.3859502	0.2541156	0.1188703	0.1470488	0.3005137
24.048	0.5	0.106652	0.3892155	0.2369512	0.121188	0.1458927	0.3227547
24.148	0.5	0.102799	0.3815103	0.2433366	0.1224435	0.1447759	0.3403279
24.248	0.5	0.101339	0.3803425	0.2587305	0.1205275	0.1451284	0.3534276
24.348	0.5	0.104537	0.3860257	0.2744641	0.1211581	0.1460966	0.3269603
24.448	0.5	0.109727	0.3852373	0.2896867	0.1229337	0.1462375	0.2497921
24.548	0.5	0.114925	0.3815783	0.2963853	0.1254224	0.1454744	0.2178758
24.648	0.5	0.116805	0.3843269	0.2966588	0.1257805	0.1440658	0.2006482
24.748	0.5	0.11527	0.3901424	0.2831323	0.1262463	0.1430945	0.1991971
24.848	0.5	0.112176	0.3910914	0.2576534	0.1261395	0.1446531	0.1978265
24.948	0.5	0.109491	0.3888604	0.2463954	0.1214542	0.1462946	0.1910508
25.048	0.5	0.110256	0.3938518	0.2492407	0.1180049	0.1473369	0.1926262
25.148	0.5	0.109789	0.4004185	0.2574725	0.1179366	0.1454904	0.1926176
25.248	0.5	0.109592	0.4068132	0.2680877	0.1166343	0.1450828	0.194544
25.348	0.5	0.108704	0.4030944	0.2789159	0.1154443	0.1436359	0.1903106

25.448	0.5	0.108051	0.3914126	0.2879318	0.1163647	0.1457232	0.1832353
25.548	0.5	0.106975	0.3900736	0.2919632	0.1151099	0.1567689	0.1821267
25.648	0.5	0.10673	0.3900384	0.2946769	0.1151972	0.1714559	0.1778818
25.748	0.5	0.106643	0.380402	0.2826358	0.1175197	0.1846005	0.1758858
25.848	0.5	0.107364	0.3812603	0.2574451	0.1189744	0.1971978	0.1734192
25.948	0.5	0.111763	0.3781699	0.2557728	0.1181953	0.1976918	0.1752077
26.048	0.5	0.11749	0.3830801	0.2684569	0.1170342	0.1918586	0.1738895
26.148	0.5	0.122517	0.3901443	0.2788848	0.1154883	0.1893123	0.1742155
26.248	0.5	0.128151	0.3834755	0.2912754	0.1167415	0.178617	0.1744991
26.348	0.5	0.133735	0.3804795	0.291948	0.1180475	0.1649808	0.1778586
26.448	0.5	0.137176	0.3859691	0.2794747	0.1165934	0.1593027	0.1823107
26.548	0.5	0.1366	0.3957901	0.2586887	0.1234329	0.1539889	0.1824151
26.648	0.5	0.13634	0.3924221	0.2612352	0.1293424	0.1530593	0.1808662
26.748	0.5	0.136621	0.3813299	0.2670048	0.135486	0.1593756	0.1771013
26.848	0.5	0.131601	0.3847313	0.2760915	0.1420926	0.1643668	0.1746391
26.948	0.5	0.127385	0.382109	0.2876451	0.1464184	0.1634825	0.1737835
27.048	0.5	0.127867	0.3697893	0.2925749	0.1449383	0.1622711	0.1784677
27.148	0.5	0.131127	0.3662773	0.2965405	0.143388	0.1626651	0.1821443
27.248	0.5	0.135485	0.3677772	0.291104	0.1401081	0.1631988	0.1837868
27.348	0.5	0.138667	0.3726029	0.2798903	0.1368997	0.1633968	0.1779194
27.448	0.5	0.140559	0.3722286	0.2609794	0.1424478	0.1645568	0.1778347
27.548	0.5	0.142415	0.3723605	0.2484407	0.1447527	0.1641081	0.1783532
27.648	0.5	0.142042	0.3682131	0.252056	0.1415609	0.1649376	0.1775658
27.748	0.5	0.139679	0.3704311	0.2639855	0.1466896	0.1654958	0.1765645
27.848	0.5	0.137228	0.3725267	0.2784892	0.14633	0.1673433	0.1828781
27.948	0.5	0.136411	0.3671223	0.2922834	0.1398512	0.1671911	0.1908355
28.048	0.5	0.13664	0.3695783	0.299677	0.1361095	0.167698	0.1934334
28.148	0.5	0.135376	0.3671138	0.2854818	0.1299504	0.1691213	0.195514
28.248	0.5	0.135673	0.3565391	0.2560614	0.1209439	0.1696017	0.1983332
28.348	0.5	0.13396	0.3607773	0.2440303	0.1179051	0.1674786	0.2033469
28.448	0.5	0.131464	0.3701428	0.2538986	0.1317456	0.1674808	0.2073782
28.548	0.5	0.131453	0.3751925	0.2735084	0.1501585	0.1691938	0.2088033
28.648	0.5	0.131933	0.3785486	0.2928053	0.162687	0.1705218	0.2155232
28.748	0.5	0.131471	0.374195	0.311132	0.174889	0.1711049	0.2217126
28.848	0.5	0.129537	0.3642827	0.323337	0.1863597	0.1711421	0.220046
28.948	0.5	0.129367	0.3653691	0.3232757	0.2012852	0.1633182	0.223229
29.048	0.5	0.132836	0.3715571	0.2944033	0.2168863	0.1627817	0.2250277
29.148	0.5	0.137274	0.3721494	0.2706947	0.2334598	0.1674107	0.2224905
29.248	0.5	0.141002	0.3667147	0.2728229	0.2453525	0.1665201	0.213246
29.348	0.5	0.143329	0.3717262	0.2810808	0.2379925	0.1624835	0.2060956
29.448	0.5	0.144831	0.3791463	0.2912353	0.2262566	0.1590212	0.1946723
29.548	0.5	0.14706	0.3773629	0.2972324	0.2155971	0.1588177	0.1854352
29.648	0.5	0.145587	0.3719895	0.2999353	0.1864515	0.1607471	0.1885269
29.748	0.5	0.142888	0.3714884	0.3035711	0.1590238	0.1614858	0.2024958
29.848	0.5	0.141783	0.3681498	0.2870904	0.145217	0.1618828	0.2199663
29.948	0.5	0.141384	0.3679552	0.266475	0.1339893	0.1628843	0.2335849
30.048	0.5	0.141519	0.3580434	0.2556883	0.1305574	0.1637781	0.2511833
30.148	0.5	0.143246	0.352361	0.2557499	0.1271006	0.1695141	0.2732978
30.248	0.5	0.144349	0.3524329	0.2637037	0.12369	0.1715717	0.2981054
30.348	0.5	0.143387	0.3575813	0.2753411	0.1233176	0.1759772	0.3184149
30.448	0.5	0.142052	0.360154	0.2831891	0.1218983	0.1786731	0.3376279
30.548	0.5	0.142465	0.3640027	0.2892723	0.1219817	0.1824632	0.3523444
30.648	0.5	0.142692	0.3600485	0.2909398	0.1196884	0.1851065	0.3614327
30.748	0.5	0.14194	0.3609387	0.2883646	0.1203993	0.1851409	0.3513905
30.848	0.5	0.142744	0.3682806	0.283322	0.121463	0.1853777	0.3026327
30.948	0.5	0.144607	0.367288	0.2817706	0.1200079	0.1872131	0.2284972
31.048	0.5	0.145897	0.3556523	0.2645087	0.1193374	0.1894794	0.2087982
31.148	0.5	0.146335	0.3523616	0.2550763	0.1198609	0.1907566	0.210458
31.248	0.5	0.149389	0.3518456	0.2637243	0.117943	0.193473	0.2122312
31.348	0.5	0.155067	0.3522319	0.2713244	0.1154152	0.1935731	0.2080127
31.448	0.5	0.162083	0.3438717	0.2777914	0.1170885	0.193776	0.2063307
31.548	0.5	0.170666	0.3426405	0.2844748	0.1185044	0.1941076	0.2105772
31.648	0.5	0.179001	0.3423364	0.2770051	0.1178578	0.1920157	0.2161919
31.748	0.5	0.187771	0.3418349	0.2505148	0.1163896	0.1884445	0.2176671
31.848	0.5	0.194284	0.3322821	0.2295581	0.1161577	0.188899	0.2164056
31.948	0.5	0.198323	0.3300089	0.2308238	0.1161644	0.1878726	0.2114477
32.048	0.5	0.196531	0.3386393	0.242325	0.1150464	0.1880087	0.21043
32.148	0.5	0.187659	0.3478355	0.2550446	0.1179044	0.1901202	0.211984
32.248	0.5	0.182902	0.3531516	0.2666227	0.1188968	0.1931268	0.2134141
32.348	0.5	0.181068	0.3415682	0.2763138	0.1191225	0.1926434	0.2130264
32.448	0.5	0.180743	0.3400746	0.2799265	0.1178788	0.1943547	0.2111436
32.548	0.5	0.178795	0.3449936	0.2786723	0.1168795	0.1950785	0.2011128
32.648	0.5	0.17515	0.3499857	0.2780816	0.1156739	0.197406	0.1969639
32.748	0.5	0.169156	0.3442391	0.2800547	0.1160191	0.1992486	0.2010478
32.848	0.5	0.16469	0.3308138	0.2848007	0.1197427	0.2008921	0.2081384
32.948	0.5	0.162398	0.3372295	0.2905726	0.1170804	0.2034219	0.2265426
33.048	0.5	0.160727	0.34527	0.2906337	0.113256	0.2050113	0.2483731
33.148	0.5	0.160203	0.3478221	0.2916038	0.1138906	0.2022235	0.2645338
33.248	0.5	0.160451	0.3409525	0.2921896	0.1165453	0.2015047	0.2844161
33.348	0.5	0.161004	0.334602	0.2938723	0.1225027	0.2001381	0.3037312
33.448	0.5	0.162769	0.3266757	0.2999558	0.1267224	0.1991837	0.326706
33.548	0.5	0.167858	0.3275847	0.2999022	0.1301427	0.1987669	0.346862
33.648	0.5	0.175711	0.3411629	0.3024099	0.1341505	0.2017103	0.3550081
33.748	0.5	0.186256	0.3550748	0.3009416	0.1325508	0.2055622	0.334277
33.848	0.5	0.196932	0.359791	0.298231	0.1281907	0.205465	0.2611705
33.948	0.5	0.20496	0.3493632	0.2930307	0.1260928	0.2008783	0.2299379
34.048	0.5	0.206817	0.3429739	0.2845112	0.1237156	0.1948855	0.2338758
34.148	0.5	0.201613	0.3524192	0.282279	0.1239532	0.1934239	0.2307087
34.248	0.5	0.199057	0.350328	0.2833843	0.1230736	0.1938525	0.2264983
34.348	0.5	0.199737	0.3480031	0.2869628	0.1236192	0.1945727	0.2273116
34.448	0.5	0.20341	0.3541529	0.2980174	0.1204393	0.1985274	0.2286405
34.548	0.5	0.206886	0.3701915	0.3102363	0.1162094	0.200016	0.211111
34.648	0.5	0.206278	0.3750302	0.3186807	0.1178249	0.2003615	0.1933837
34.748	0.5	0.202838	0.3651488	0.2992563	0.1205835	0.2015542	0.1866069
34.848	0.5	0.199625	0.3598666	0.2719184	0.1232587	0.2001275	0.183837

34.948	0.5	0.196867	0.3631867	0.2754183	0.1330469	0.2003352	0.1812997
35.048	0.5	0.195944	0.3635742	0.2813709	0.1377813	0.2027323	0.1794991
35.148	0.5	0.195388	0.3619618	0.2930723	0.1416286	0.2044374	0.177426
35.248	0.5	0.195557	0.3648979	0.3071019	0.1455479	0.2051717	0.1799718
35.348	0.5	0.196349	0.3732045	0.3088048	0.1477371	0.2017163	0.1837382
35.448	0.5	0.198809	0.3643565	0.3088834	0.1437051	0.2006168	0.1911667
35.548	0.5	0.201877	0.3578166	0.3020722	0.139951	0.2049571	0.191386
35.648	0.5	0.209687	0.3612813	0.2828366	0.1424931	0.2069401	0.1904717
35.748	0.5	0.218009	0.3676345	0.273571	0.141701	0.204969	0.1854319
35.848	0.5	0.227132	0.367839	0.276976	0.1346729	0.2031574	0.1812255
35.948	0.5	0.233692	0.3724791	0.2858458	0.1253079	0.1991936	0.1806308
36.048	0.5	0.235763	0.375755	0.2959801	0.1223675	0.1978334	0.1803386
36.148	0.5	0.242183	0.369897	0.3016917	0.1238965	0.1954662	0.1902135
36.248	0.5	0.249998	0.365895	0.3055524	0.1226637	0.1955023	0.1968669
36.348	0.5	0.251474	0.361347	0.3052988	0.123331	0.1962158	0.2067714
36.448	0.5	0.250328	0.3618829	0.2907538	0.1226623	0.1960088	0.220087
36.548	0.5	0.252141	0.3642105	0.2844418	0.1226515	0.1958655	0.2299079
36.648	0.5	0.250409	0.3694145	0.288127	0.121826	0.1966503	0.2382081
36.748	0.5	0.235353	0.3750114	0.2941516	0.1182707	0.2003783	0.2455208
36.848	0.5	0.22083	0.3751109	0.2997777	0.1228908	0.2042831	0.2505908
36.948	0.5	0.223934	0.369639	0.3054045	0.1275231	0.1986201	0.2559997
37.048	0.5	0.236493	0.37446	0.3060552	0.1291399	0.1954002	0.2599242
37.148	0.5	0.253292	0.3846074	0.3083409	0.1262238	0.1971961	0.2664341
37.248	0.5	0.267586	0.3843105	0.3017986	0.1213047	0.2004849	0.2759632
37.348	0.5	0.275413	0.3894872	0.2679344	0.1152769	0.2031954	0.2868994
37.448	0.5	0.275006	0.3847507	0.2588492	0.1140164	0.2031043	0.2934532
37.548	0.5	0.261628	0.3838527	0.2670581	0.1162737	0.2002653	0.2768947
37.648	0.5	0.255739	0.3717403	0.2844675	0.1176895	0.196543	0.2636386
37.748	0.5	0.250774	0.3742658	0.3011169	0.1188577	0.1971145	0.2538752
37.848	0.5	0.253456	0.3844704	0.3137296	0.1193034	0.2006676	0.2417689
37.948	0.5	0.267525	0.3940767	0.3196877	0.1223216	0.203712	0.2300154
38.048	0.5	0.283806	0.3891376	0.3287111	0.1240041	0.2024969	0.2167796
38.148	0.5	0.294985	0.3849979	0.3241453	0.1248939	0.2025744	0.2130094
38.248	0.5	0.299874	0.384003	0.2995633	0.1242776	0.2051505	0.2146374
38.348	0.5	0.293268	0.389956	0.2755404	0.1284097	0.2120589	0.2109553
38.448	0.5	0.274261	0.3922208	0.2795697	0.1264548	0.2061793	0.2037051
38.548	0.5	0.220922	0.3936385	0.2864919	0.1247019	0.2026832	0.1945367
38.648	0.5	0.185522	0.4013459	0.3010758	0.1233028	0.2003891	0.1968302
38.748	0.5	0.190278	0.4031567	0.3115304	0.1222301	0.20148	0.205272
38.848	0.5	0.204386	0.3998683	0.3167679	0.1242656	0.2012545	0.2132953
38.948	0.5	0.21701	0.3752851	0.3080373	0.1255727	0.2023942	0.2208909
39.048	0.5	0.213883	0.3741843	0.2939757	0.1252727	0.2010821	0.2294603
39.148	0.5	0.172316	0.3808947	0.2811247	0.1312692	0.2017406	0.2381789
39.248	0.5	0.15931	0.3845452	0.2777484	0.1399687	0.202939	0.2548935
39.348	0.5	0.165342	0.3728488	0.2911521	0.1476211	0.201291	0.2727745
39.448	0.5	0.17768	0.3486162	0.3040513	0.144135	0.2025473	0.2936163
39.548	0.5	0.190368	0.3560826	0.3160472	0.1485385	0.2006902	0.3110189
39.648	0.5	0.200124	0.369543	0.3226795	0.1527653	0.1993239	0.3248233
39.748	0.5	0.206228	0.377884	0.3269005	0.1491387	0.199364	0.3443099
39.848	0.5	0.211233	0.3815148	0.3073231	0.1379613	0.1954431	0.3553739
39.948	0.5	0.213321	0.3874448	0.2721647	0.1350131	0.1936637	0.367164
40.048	0.5	0.209391	0.3959272	0.2678247	0.1314165	0.1945972	0.3732851
40.148	0.5	0.208868	0.3995641	0.2788085	0.1248579	0.1930744	0.3727644
40.248	0.5	0.21568	0.391391	0.2953431	0.1341321	0.1973833	0.351323
40.348	0.5	0.223768	0.3859588	0.312001	0.1487094	0.2055275	0.3118244
40.448	0.5	0.238479	0.3905459	0.3218316	0.1591346	0.2058767	0.3019306
40.548	0.5	0.259963	0.3961939	0.3227428	0.1666312	0.2041301	0.2962366
40.648	0.5	0.281887	0.4000524	0.3182476	0.1701411	0.2032287	0.2829273
40.748	0.5	0.300308	0.4025589	0.3081231	0.1699361	0.204165	0.2771634
40.848	0.5	0.3161	0.4029654	0.3078108	0.1685999	0.2024764	0.2765799
40.948	0.5	0.329029	0.3930736	0.3129629	0.1565649	0.2030644	0.2817737
41.048	0.5	0.340328	0.4015152	0.3166658	0.146546	0.201759	0.2908683
41.148	0.5	0.347276	0.4046481	0.3213304	0.131769	0.202137	0.2862078
41.248	0.5	0.352502	0.4054726	0.2981365	0.1313304	0.2011503	0.2842836
41.348	0.5	0.351146	0.4088173	0.2827318	0.129721	0.1985689	0.2915696
41.448	0.5	0.343981	0.41087	0.2878631	0.1264864	0.1981991	0.3015408
41.548	0.5	0.321377	0.4105539	0.2922679	0.1221057	0.1970939	0.3038225
41.648	0.5	0.265325	0.4040959	0.3021066	0.1203632	0.1978386	0.3049325
41.748	0.5	0.188914	0.4034169	0.3064551	0.12049	0.1997749	0.3121333
41.848	0.5	0.165562	0.4046852	0.3088411	0.1260253	0.2011928	0.3113455
41.948	0.5	0.157421	0.4068392	0.3153089	0.1306048	0.2037945	0.2923104
42.048	0.5	0.160254	0.3986953	0.320309	0.1352059	0.2018196	0.2781164
42.148	0.5	0.16517	0.3951765	0.3174096	0.1350065	0.2015711	0.2762508
42.248	0.5	0.171875	0.3910197	0.313648	0.1349962	0.2012294	0.26604
42.348	0.5	0.185267	0.3809994	0.3155679	0.1412549	0.2048576	0.2569883
42.448	0.5	0.206773	0.3601559	0.3225478	0.1379144	0.2072264	0.2509399
42.548	0.5	0.234762	0.3524235	0.3316674	0.1282655	0.2047816	0.253132
42.648	0.5	0.264425	0.3588799	0.3373117	0.1206801	0.2036625	0.2527549
42.748	0.5	0.293829	0.3729747	0.340825	0.1213237	0.2035645	0.2553746
42.848	0.5	0.321541	0.3899238	0.3326653	0.1225718	0.2027087	0.2586862
42.948	0.5	0.340682	0.3973063	0.3081633	0.1216553	0.2038708	0.2619054
43.048	0.5	0.3495	0.4013048	0.3072045	0.1246515	0.2062604	0.2655522
43.148	0.5	0.356738	0.3926041	0.3110017	0.1231405	0.2020266	0.2727352
43.248	0.5	0.361867	0.3932257	0.3151419	0.1233205	0.1977308	0.2704905
43.348	0.5	0.375449	0.4015569	0.3159703	0.1254524	0.1990041	0.2542935
43.448	0.5	0.393965	0.402054	0.3210343	0.125964	0.196784	0.256096
43.548	0.5	0.411308	0.4024674	0.3237706	0.1212567	0.1977015	0.2704266
43.648	0.5	0.424111	0.4020649	0.3232458	0.1212957	0.1995867	0.2827826
43.748	0.5	0.423633	0.4057405	0.3206958	0.1208638	0.1969355	0.2902226
43.848	0.5	0.408752	0.4060836	0.3147448	0.1202054	0.1967844	0.2950214
43.948	0.5	0.390873	0.4114443	0.3157216	0.1230348	0.1981294	0.2884574
44.048	0.5	0.395112	0.4053288	0.3188912	0.1246018	0.1967253	0.2754249
44.148	0.5	0.40091	0.4130968	0.3153903	0.1248242	0.1963072	0.2937715
44.248	0.5	0.408927	0.4195332	0.3147268	0.1261943	0.1980656	0.3172859
44.348	0.5	0.395925	0.411569	0.3126985	0.12977	0.1949476	0.3303418

44.448	0.5	0.386616	0.4122488	0.3084179	0.1310448	0.1940155	0.3393805
44.548	0.5	0.39095	0.4148138	0.3138796	0.1369003	0.1957068	0.3514241
44.648	0.5	0.412129	0.4141231	0.3088857	0.1383728	0.1974021	0.3378011
44.748	0.5	0.42236	0.4180446	0.3007333	0.1358205	0.2017724	0.3159341
44.848	0.5	0.428062	0.4144952	0.2994908	0.1330539	0.2009192	0.3042201
44.948	0.5	0.416784	0.409914	0.3097868	0.1301149	0.1990305	0.2940954
45.048	0.5	0.403193	0.4058342	0.3177246	0.1321878	0.1990004	0.2891243
45.148	0.5	0.428818	0.4100671	0.3189508	0.1390715	0.20293	0.2886656
45.248	0.5	0.452947	0.4156388	0.3172719	0.1451455	0.2042479	0.2832204
45.348	0.5	0.466654	0.3993731	0.3167834	0.1402001	0.2001883	0.2756217
45.448	0.5	0.468964	0.4067052	0.3157693	0.1377277	0.198648	0.2740062
45.548	0.5	0.451143	0.407241	0.318809	0.146111	0.2014357	0.2749769
45.648	0.5	0.400187	0.4004897	0.3163635	0.1631989	0.2044114	0.2768637
45.748	0.5	0.415703	0.4005209	0.3220063	0.1800051	0.2050105	0.273459
45.848	0.5	0.448587	0.4067661	0.3217132	0.1978	0.2025044	0.267575
45.948	0.5	0.477434	0.4102389	0.3225492	0.2102687	0.2037286	0.2717884
46.048	0.5	0.480312	0.4060388	0.3151557	0.2173417	0.2034488	0.272837
46.148	0.5	0.479828	0.4080829	0.3136819	0.2114547	0.2047663	0.2639127
46.248	0.5	0.44231	0.3998456	0.3137412	0.1880367	0.2056852	0.2620495
46.348	0.5	0.431281	0.4109191	0.3149229	0.1721276	0.1963835	0.2523546
46.448	0.5	0.440288	0.4157233	0.312842	0.1540864	0.1947258	0.2381086
46.548	0.5	0.453396	0.408143	0.308459	0.1443526	0.2006522	0.2365573
46.648	0.5	0.461783	0.403653	0.3091851	0.1351567	0.2090592	0.2452904
46.748	0.5	0.461276	0.3955062	0.308751	0.1331169	0.2046677	0.2480295
46.848	0.5	0.449133	0.390567	0.3090735	0.1322675	0.2019702	0.2468113
46.948	0.5	0.434512	0.3884376	0.3051407	0.1308572	0.2028908	0.2461971
47.048	0.5	0.407451	0.3891654	0.3136156	0.1310375	0.2020855	0.2551469
47.148	0.5	0.38595	0.3918124	0.3183951	0.1437115	0.2039669	0.2642674
47.248	0.5	0.36582	0.3900639	0.3149484	0.1637939	0.2057524	0.2596952
47.348	0.5	0.367163	0.3849425	0.3104276	0.1791304	0.2008782	0.2635248
47.448	0.5	0.378233	0.3840573	0.3067486	0.1840528	0.2004678	0.2685606
47.548	0.5	0.390871	0.3840794	0.3095815	0.187849	0.2008686	0.274553
47.648	0.5	0.394211	0.3916177	0.3133756	0.1918874	0.2048743	0.2764789
47.748	0.5	0.369864	0.3909099	0.3175641	0.188159	0.2068966	0.2866082
47.848	0.5	0.308209	0.3868966	0.3178912	0.1761967	0.2079511	0.2858317
47.948	0.5	0.258553	0.3910486	0.3137076	0.1727931	0.2073867	0.2791611
48.048	0.5	0.255623	0.3926791	0.30326	0.1612903	0.2063507	0.273299
48.148	0.5	0.253917	0.4017646	0.2964268	0.1423001	0.206562	0.2684596
48.248	0.5	0.260596	0.3946443	0.2988659	0.1355763	0.2023244	0.2638906
48.348	0.5	0.273014	0.3865226	0.2971942	0.1378401	0.1993842	0.2635642
48.448	0.5	0.28383	0.3750538	0.3020506	0.1401951	0.2008884	0.256501
48.548	0.5	0.294812	0.3774824	0.3035106	0.1394857	0.2018363	0.2485553
48.648	0.5	0.303173	0.3880527	0.3010818	0.145344	0.197209	0.2477916
48.748	0.5	0.313151	0.3918373	0.3016422	0.1603484	0.1996103	0.239533
48.848	0.5	0.325405	0.393267	0.302792	0.1690269	0.2000933	0.2275146
48.948	0.5	0.322527	0.3806126	0.3081405	0.1745699	0.1973239	0.2242786
49.048	0.5	0.290995	0.3673417	0.3029992	0.1810137	0.1993945	0.2277651
49.148	0.5	0.294075	0.3542733	0.3020371	0.1839255	0.2008051	0.2216769
49.248	0.5	0.310075	0.3613799	0.3002002	0.1867993	0.1977686	0.2176857
49.348	0.5	0.329845	0.368207	0.3018082	0.1848945	0.1971552	0.2185744
49.448	0.5	0.358286	0.3707508	0.3041	0.169117	0.1886364	0.2176317
49.548	0.5	0.378273	0.3612881	0.30665	0.1665677	0.1877335	0.2273105
49.648	0.5	0.393119	0.3719286	0.3085683	0.1633452	0.2106488	0.2380485
49.748	0.5	0.407165	0.3834864	0.3103525	0.1649009	0.2433829	0.2540549
49.848	0.5	0.417411	0.3899553	0.3101052	0.1602202	0.2743202	0.2688699
49.948	0.5	0.437244	0.3945924	0.3111613	0.159246	0.3030439	0.2797218
50.048	0.5	0.462101	0.4038683	0.3105697	0.1565332	0.331174	0.2949174
50.148	0.5	0.476259	0.4084236	0.3112289	0.1479122	0.3543178	0.3102577
50.248	0.5	0.47891	0.410996	0.3131716	0.1340632	0.3299095	0.3138083
50.348	0.5	0.464296	0.405362	0.314386	0.1277324	0.298065	0.2919987
50.448	0.5	0.448551	0.3940217	0.3150388	0.1246574	0.2749836	0.2555082
50.548	0.5	0.455023	0.401462	0.3123749	0.1236048	0.2581363	0.2477256
50.648	0.5	0.439769	0.4011482	0.3125289	0.1226224	0.2525654	0.2470214
50.748	0.5	0.447797	0.3976851	0.3186464	0.1225791	0.2476099	0.2368209
50.848	0.5	0.470475	0.4019497	0.3223615	0.1253581	0.2441539	0.2246123
50.948	0.5	0.498003	0.4030291	0.3237801	0.1276299	0.2443245	0.2213213
51.048	0.5	0.520994	0.4016882	0.3221516	0.1320995	0.2417723	0.220059
51.148	0.5	0.533404	0.402639	0.3219608	0.1303519	0.2311753	0.221511
51.248	0.5	0.525525	0.3955393	0.3228232	0.1319551	0.2334534	0.2277702
51.348	0.5	0.461313	0.3785079	0.3229462	0.1304466	0.2341121	0.2340636
51.448	0.5	0.438091	0.3803132	0.3226399	0.1275949	0.2207405	0.2372869
51.548	0.5	0.467899	0.4017807	0.3268288	0.1253004	0.2174622	0.2351442
51.648	0.5	0.492811	0.4075623	0.33161	0.1261411	0.2182065	0.2311362
51.748	0.5	0.51499	0.4078429	0.3361413	0.1259412	0.2138299	0.2223471
51.848	0.5	0.533084	0.4042462	0.3394914	0.1255113	0.2075596	0.2151776
51.948	0.5	0.527534	0.3912659	0.3403102	0.1248725	0.211419	0.2141118
52.048	0.5	0.444311	0.3716687	0.3253335	0.1237582	0.215544	0.2186808
52.148	0.5	0.388118	0.3603849	0.3216911	0.1260684	0.2200133	0.2222076
52.248	0.5	0.424899	0.3460864	0.3227597	0.1266703	0.2223695	0.2278947
52.348	0.5	0.455857	0.3494469	0.3205424	0.1283971	0.2265085	0.2299841
52.448	0.5	0.482968	0.3694443	0.3246441	0.1287446	0.2336607	0.2343172
52.548	0.5	0.509701	0.3734516	0.3289711	0.1280098	0.2390318	0.2359159
52.648	0.5	0.533926	0.3756759	0.3313448	0.1291273	0.2447255	0.2335326
52.748	0.5	0.550906	0.3735358	0.3327983	0.1301339	0.2357331	0.2343856
52.848	0.5	0.55607	0.3756133	0.3343687	0.1318276	0.2312998	0.2404822
52.948	0.5	0.518721	0.3779233	0.3283951	0.1306236	0.2253603	0.2502741
53.048	0.5	0.514878	0.3523029	0.3201307	0.1301419	0.2184655	0.2538402
53.148	0.5	0.523007	0.3609262	0.3099869	0.1300724	0.2148271	0.2557449
53.248	0.5	0.54859	0.3753606	0.3122038	0.1276907	0.210491	0.2522805
53.348	0.5	0.569363	0.3917617	0.3150927	0.1267382	0.2054132	0.246783
53.448	0.5	0.579622	0.4034033	0.3221565	0.1271841	0.2046431	0.2437017
53.548	0.5	0.574522	0.3992008	0.3273623	0.1265344	0.205743	0.2429155
53.648	0.5	0.502307	0.4031118	0.3274927	0.1266539	0.2092137	0.2350295
53.748	0.5	0.422565	0.4104981	0.3247158	0.1290784	0.2072272	0.2393897
53.848	0.5	0.450606	0.4189062	0.3218492	0.1290088	0.2050714	0.2455224

53.948	0.5	0.483906	0.4179657	0.3249759	0.1280045	0.2017874	0.2436495
54.048	0.5	0.51509	0.4103102	0.3260036	0.1266724	0.1991964	0.2462084
54.148	0.5	0.541264	0.4173088	0.3165824	0.1247369	0.2007357	0.2542112
54.248	0.5	0.550556	0.4186091	0.3141931	0.1265804	0.1971899	0.2604811
54.348	0.5	0.546137	0.4163595	0.3149768	0.1270697	0.1966759	0.2611611
54.448	0.5	0.550661	0.4194429	0.3078113	0.1281707	0.1995394	0.268864
54.548	0.5	0.568573	0.4145197	0.3112131	0.1270783	0.1996259	0.2720191
54.648	0.5	0.574136	0.414803	0.3156523	0.1257487	0.1995641	0.2654738
54.748	0.5	0.549042	0.4063336	0.3197929	0.1246758	0.1987162	0.2650112
54.848	0.5	0.458008	0.4105166	0.3242284	0.1281864	0.1962792	0.2692719
54.948	0.5	0.36602	0.4198652	0.3270038	0.1256659	0.1959781	0.277435
55.048	0.5	0.336494	0.4220155	0.3318224	0.1227781	0.1968138	0.2743061
55.148	0.5	0.274876	0.4238622	0.3384981	0.1233376	0.1995179	0.2696394
55.248	0.5	0.251001	0.4234058	0.3393435	0.1214357	0.1966988	0.2701167
55.348	0.5	0.243171	0.4150445	0.3290704	0.1198501	0.1959415	0.2680213
55.448	0.5	0.233629	0.4156615	0.3299148	0.1260175	0.1972157	0.2735699
55.548	0.5	0.230818	0.4190833	0.336925	0.1277864	0.1976947	0.2656251
55.648	0.5	0.231488	0.4081785	0.3429123	0.129632	0.2068954	0.2557946
55.748	0.5	0.23284	0.4197653	0.3297786	0.1328529	0.2186756	0.2578108
55.848	0.5	0.229587	0.4200958	0.3171719	0.129192	0.2327485	0.2582021
55.948	0.5	0.225148	0.4202372	0.3166568	0.1248904	0.2257816	0.2492393
56.048	0.5	0.219261	0.4199707	0.3133878	0.1213422	0.2167701	0.2333521
56.148	0.5	0.214332	0.4201843	0.3114911	0.1199378	0.2130203	0.2299673
56.248	0.5	0.212765	0.4171084	0.3094741	0.115938	0.2095561	0.2242485
56.348	0.5	0.21316	0.4257782	0.3119911	0.1144759	0.2152321	0.2269624
56.448	0.5	0.219081	0.4312319	0.3081653	0.1144419	0.2288309	0.2174494
56.548	0.5	0.229316	0.436099	0.3096558	0.1161149	0.2316825	0.2044773
56.648	0.5	0.238601	0.4444618	0.3166959	0.1176149	0.2302768	0.1991513
56.748	0.5	0.245635	0.4448399	0.3075417	0.1210175	0.2274793	0.2010722
56.848	0.5	0.245043	0.4416237	0.3022772	0.1256542	0.224361	0.2126399
56.948	0.5	0.236342	0.433346	0.3055032	0.1225326	0.2217297	0.2245008
57.048	0.5	0.224445	0.4252343	0.3082702	0.1233199	0.2227229	0.2351742
57.148	0.5	0.221663	0.424006	0.3095756	0.1262248	0.2204008	0.2371873
57.248	0.5	0.221788	0.423795	0.3144623	0.128134	0.21769	0.2411156
57.348	0.5	0.225602	0.4236999	0.327291	0.1256192	0.2161637	0.2390831
57.448	0.5	0.233387	0.428102	0.3310459	0.1212783	0.2195083	0.2264508
57.548	0.5	0.238988	0.4315775	0.3286679	0.1208865	0.2248522	0.224308
57.648	0.5	0.250309	0.4385691	0.3220871	0.1244422	0.2325741	0.2208387
57.748	0.5	0.259008	0.4358354	0.3118407	0.1260387	0.2459469	0.218498
57.848	0.5	0.261745	0.4310221	0.3108283	0.1320334	0.2595007	0.216896
57.948	0.5	0.26603	0.4124986	0.3107006	0.1346236	0.2631907	0.2117859
58.048	0.5	0.26475	0.3872531	0.3118708	0.1290538	0.2606342	0.2054402
58.148	0.5	0.25934	0.399303	0.3149163	0.1219403	0.2415567	0.2026987
58.248	0.5	0.254981	0.4054717	0.3162916	0.1212973	0.2302715	0.2142739
58.348	0.5	0.243504	0.4183386	0.3179261	0.1218996	0.2193243	0.2328855
58.448	0.5	0.253408	0.421607	0.313672	0.1227903	0.2144594	0.248019
58.548	0.5	0.259638	0.4190941	0.304856	0.1186591	0.2204784	0.2640389
58.648	0.5	0.26312	0.4181454	0.2991158	0.1157992	0.2317751	0.2813169
58.748	0.5	0.271151	0.4257909	0.3053483	0.1152682	0.2492494	0.2887493
58.848	0.5	0.273639	0.42262	0.3048898	0.115396	0.2634388	0.2861618
58.948	0.5	0.273979	0.4247249	0.3085892	0.1148362	0.2622243	0.2879028
59.048	0.5	0.277308	0.4223033	0.2914922	0.1137577	0.2587324	0.2883263
59.148	0.5	0.2841	0.4305746	0.2923698	0.1141722	0.2495093	0.2801127
59.248	0.5	0.28992	0.4349251	0.2994572	0.1121207	0.2300468	0.2602097
59.348	0.5	0.28676	0.4446302	0.3028591	0.1108099	0.2185692	0.2341515
59.448	0.5	0.238543	0.4523173	0.302066	0.1115135	0.212336	0.2390433
59.548	0.5	0.248386	0.4579319	0.3048046	0.109905	0.2066208	0.2446917
59.648	0.5	0.267814	0.4613709	0.3064518	0.1095475	0.2046193	0.2402141
59.748	0.5	0.278346	0.4534343	0.3119754	0.1100403	0.2059976	0.2396058
59.848	0.5	0.288642	0.4535645	0.3222429	0.1144921	0.2063247	0.2305669
59.948	0.5	0.314642	0.4577899	0.3326724	0.1220406	0.2091016	0.2281356
60.064	1	0.345499	0.4264183	0.3094947	0.1426769	0.2127222	0.2413634
60.164	1	0.374786	0.3952891	0.2730922	0.1436087	0.2133262	0.2361839
60.264	1	0.401444	0.3923125	0.269894	0.1332364	0.2107263	0.2321023
60.364	1	0.432395	0.3997749	0.2721448	0.1229619	0.2111902	0.2358315
60.464	1	0.464503	0.4184804	0.2778127	0.1144326	0.2121209	0.2417892
60.564	1	0.509796	0.415902	0.2796756	0.1108372	0.2182568	0.2430689
60.664	1	0.530462	0.4204774	0.2698093	0.1089106	0.2118334	0.2355136
60.764	1	0.539783	0.4225523	0.2732956	0.1173937	0.2088943	0.219517
60.864	1	0.491143	0.4207836	0.2681138	0.1193622	0.2112609	0.2240308
60.964	1	0.449807	0.3931696	0.2456757	0.1176257	0.2125647	0.2304693
61.064	1	0.509594	0.4009424	0.2425059	0.1181754	0.2188317	0.2231487
61.164	1	0.483528	0.4032295	0.2494983	0.1150047	0.2221814	0.2166551
61.264	1	0.43842	0.401718	0.2603885	0.11133	0.2239256	0.2141109
61.364	1	0.495903	0.3932926	0.2762027	0.1090307	0.2208192	0.2180478
61.464	1	0.544002	0.4134523	0.3046148	0.1082529	0.2159975	0.2135029
61.564	1	0.469603	0.408313	0.3064243	0.1064963	0.2321302	0.2212523
61.664	1	0.443682	0.4179421	0.2840177	0.1071432	0.2452244	0.2311896
61.764	1	0.506243	0.4298451	0.2680838	0.1138562	0.2456554	0.2356117
61.864	1	0.517273	0.4335015	0.25135	0.1144688	0.2372365	0.2276055
61.964	1	0.467664	0.4206423	0.2442619	0.1153035	0.2245957	0.2116684
62.064	1	0.496722	0.4162257	0.2497667	0.118576	0.2121162	0.2090787
62.164	1	0.553398	0.4157431	0.2438542	0.1456445	0.2070087	0.2108265
62.264	1	0.588412	0.4184068	0.2349919	0.1855867	0.2083216	0.2068347
62.364	1	0.486064	0.4210906	0.2281293	0.2091617	0.2071591	0.2060461
62.464	1	0.486206	0.425586	0.2280503	0.2242857	0.2040435	0.1952282
62.564	1	0.532578	0.4129236	0.2339637	0.2187239	0.2062643	0.192242
62.664	1	0.527164	0.3925899	0.2444931	0.1747678	0.2054185	0.1919009
62.764	1	0.508251	0.4133313	0.2630571	0.1318679	0.2233163	0.1923692
62.864	1	0.537324	0.4306814	0.2765172	0.123051	0.2542228	0.1963278
62.964	1	0.53427	0.430224	0.279979	0.1187592	0.2616917	0.1971103
63.064	1	0.515432	0.4165812	0.2695437	0.117054	0.2374707	0.2054925
63.164	1	0.520018	0.4217783	0.2650752	0.1156148	0.224958	0.2225313
63.264	1	0.534796	0.412388	0.2593241	0.1145845	0.2249586	0.2269415
63.364	1	0.525494	0.4065235	0.2480222	0.1106413	0.2268368	0.2151072

63.464	1	0.505699	0.406291	0.243407	0.1095668	0.2352069	0.2119032
63.564	1	0.525208	0.4144976	0.2463413	0.1151609	0.241341	0.2085306
63.664	1	0.547734	0.4062885	0.2499464	0.1119116	0.2390494	0.2078381
63.764	1	0.557434	0.4144421	0.2527157	0.1107823	0.2453174	0.2162463
63.864	1	0.559722	0.3965646	0.2547998	0.1101065	0.2495436	0.2245795
63.964	1	0.535097	0.4083162	0.2594174	0.1127482	0.2686926	0.2257845
64.064	1	0.483665	0.4139587	0.2460315	0.1148994	0.2759635	0.209763
64.164	1	0.500782	0.4140618	0.2364168	0.1292845	0.2434842	0.1971403
64.264	1	0.552531	0.4011879	0.2344146	0.1422551	0.2220705	0.1965555
64.364	1	0.567521	0.4053257	0.2398552	0.1506215	0.2174073	0.1969691
64.464	1	0.521876	0.4042923	0.2464369	0.1493808	0.2181199	0.1963132
64.564	1	0.513584	0.4079954	0.2530515	0.1325053	0.2210123	0.2024122
64.664	1	0.568074	0.4059552	0.2593073	0.1173408	0.2260029	0.2047811
64.764	1	0.607688	0.3904276	0.263403	0.1124637	0.2248632	0.2107139
64.864	1	0.597357	0.415114	0.2622278	0.1113283	0.2192825	0.2177993
64.964	1	0.580591	0.4275071	0.2615258	0.1137508	0.2114901	0.2259946
65.064	1	0.599095	0.4347278	0.2592063	0.1167989	0.2102639	0.2275827
65.164	1	0.614553	0.4377971	0.2633785	0.1169274	0.204976	0.226244
65.264	1	0.599893	0.4404196	0.2638323	0.1192823	0.1994839	0.2108639
65.364	1	0.610294	0.4387009	0.2631461	0.1184289	0.20059	0.2063384
65.464	1	0.603063	0.429434	0.2588874	0.1181426	0.2019856	0.2042769
65.564	1	0.606754	0.4249165	0.2561464	0.1151591	0.205778	0.211529
65.664	1	0.619369	0.4321244	0.2690513	0.1164556	0.202456	0.2116764
65.764	1	0.588336	0.4279448	0.3201121	0.1184159	0.1970212	0.2059852
65.864	1	0.548684	0.4270173	0.3699428	0.1205455	0.2013747	0.2076267
65.964	1	0.583515	0.4178905	0.3066242	0.1205949	0.2024754	0.2069577
66.064	1	0.570409	0.402317	0.2226657	0.1206019	0.2045262	0.2170576
66.164	1	0.528727	0.4073074	0.2150838	0.1254511	0.2052536	0.2192918
66.264	1	0.530618	0.407038	0.2107258	0.1210799	0.2074153	0.2331272
66.364	1	0.584918	0.4212799	0.2123614	0.1167125	0.2062365	0.235133
66.464	1	0.605624	0.4320673	0.2163887	0.1158434	0.2038939	0.2249819
66.564	1	0.595023	0.4258857	0.2197379	0.118418	0.2051973	0.2197627
66.664	1	0.60825	0.4042639	0.2215501	0.1222735	0.2086098	0.2174722
66.764	1	0.577304	0.4006185	0.2214427	0.129049	0.2089416	0.2253705
66.864	1	0.543755	0.401821	0.2189229	0.1339505	0.2090462	0.2291737
66.964	1	0.601561	0.3902002	0.217463	0.1361202	0.2067225	0.2331002
67.064	1	0.607379	0.4097899	0.2140987	0.1224739	0.2067678	0.2160457
67.164	1	0.593288	0.4171019	0.2147779	0.1161013	0.2124453	0.2055457
67.264	1	0.559517	0.4095725	0.2207729	0.1136734	0.2120072	0.2003798
67.364	1	0.551622	0.40475	0.2210968	0.1112758	0.2114925	0.1985964
67.464	1	0.513637	0.3976858	0.2239115	0.1123471	0.2100716	0.1986962
67.564	1	0.493558	0.4016669	0.2252161	0.1132209	0.2093645	0.1975419
67.664	1	0.511844	0.4118118	0.2223372	0.116174	0.2072657	0.1996852
67.764	1	0.531297	0.4330608	0.2233196	0.119677	0.2080106	0.2001074
67.864	1	0.557703	0.4494884	0.2321015	0.1232199	0.2045343	0.195077
67.964	1	0.568668	0.4503329	0.2238923	0.1176977	0.2022819	0.1951161
68.064	1	0.596314	0.4418196	0.2163952	0.1154859	0.2030585	0.1998734
68.164	1	0.597415	0.4230524	0.2139623	0.1078943	0.2092251	0.2014046
68.264	1	0.590483	0.4174637	0.2091396	0.1087617	0.2118897	0.1939942
68.364	1	0.600614	0.4125481	0.2375913	0.1124952	0.2073196	0.1939006
68.464	1	0.57658	0.416283	0.2334867	0.1118873	0.2059199	0.1977213
68.564	1	0.472168	0.4148889	0.2165206	0.1156385	0.2109303	0.1987027
68.664	1	0.517421	0.4085901	0.21402	0.1154849	0.2122391	0.2033516
68.764	1	0.568878	0.4064872	0.2530597	0.1206208	0.2174309	0.2089985
68.864	1	0.570581	0.4188441	0.3056377	0.1206352	0.2288104	0.2088573
68.964	1	0.563101	0.3757877	0.3549181	0.1218413	0.2173093	0.2203006
69.064	1	0.570671	0.3918196	0.3674672	0.1176399	0.2173557	0.2098932
69.164	1	0.571773	0.405746	0.3074999	0.11094	0.2175821	0.2055942
69.264	1	0.570111	0.4001036	0.2526055	0.1069491	0.2154802	0.2070036
69.364	1	0.570925	0.4147111	0.2303585	0.105012	0.2100318	0.2109791
69.464	1	0.563317	0.4265701	0.2266705	0.1024814	0.2042604	0.217351
69.564	1	0.543592	0.4166365	0.2341602	0.1024908	0.2031406	0.2053997
69.664	1	0.564836	0.4102868	0.2322409	0.1020627	0.1996595	0.2006954
69.764	1	0.581156	0.4026323	0.2296101	0.1022797	0.1976325	0.2023528
69.864	1	0.579555	0.4188212	0.2213574	0.1047478	0.1978683	0.1957908
69.964	1	0.5776	0.4161645	0.2167628	0.1104133	0.1963008	0.2004715
70.064	1	0.582361	0.4216038	0.2130989	0.1130363	0.1992509	0.2007451
70.164	1	0.559673	0.4161219	0.2087222	0.1146611	0.2045389	0.1965567
70.264	1	0.566738	0.4023307	0.2101944	0.1139791	0.205921	0.1981776
70.364	1	0.578521	0.385391	0.2097939	0.1104805	0.2118391	0.1949617
70.464	1	0.567765	0.4082793	0.2101829	0.1088631	0.2152546	0.1976431
70.564	1	0.547823	0.3772343	0.2079393	0.105429	0.2097552	0.1936311
70.664	1	0.560096	0.4036257	0.2059002	0.1021979	0.2054987	0.1989527
70.764	1	0.581058	0.4269827	0.202561	0.1015457	0.2132097	0.2017485
70.864	1	0.567286	0.43643	0.2017708	0.1020875	0.2237777	0.2061387
70.964	1	0.551803	0.436008	0.2019849	0.1022987	0.2300126	0.2050308
71.064	1	0.567797	0.4120852	0.2037967	0.1050694	0.2237945	0.2051973
71.164	1	0.406027	0.388548	0.2047979	0.1083119	0.2302082	0.2014046
71.264	1	0.362707	0.4000021	0.2041403	0.1059822	0.218319	0.1992195
71.364	1	0.394767	0.4126545	0.2024695	0.1021111	0.2146788	0.2126262
71.464	1	0.430735	0.4141284	0.2044872	0.1005869	0.2116778	0.2229852
71.564	1	0.429168	0.3969958	0.2053054	0.1025798	0.2059589	0.2290648
71.664	1	0.296666	0.4158684	0.205195	0.1049421	0.207233	0.2143327
71.764	1	0.282747	0.413503	0.2030669	0.103722	0.2079342	0.1971171
71.864	1	0.286619	0.4030672	0.2008034	0.1006555	0.205957	0.1978042
71.964	1	0.265384	0.4090967	0.2019314	0.1001983	0.2063602	0.2086921
72.064	1	0.287704	0.4147598	0.2020182	0.101368	0.2149874	0.2245501
72.164	1	0.321781	0.4050021	0.2038096	0.1027877	0.2131329	0.2215425
72.264	1	0.346822	0.3969615	0.204492	0.1030185	0.2264686	0.211264
72.364	1	0.368388	0.4106999	0.2052628	0.1041551	0.2266825	0.2092718
72.464	1	0.372462	0.4181027	0.2077845	0.1064511	0.2237825	0.2048517
72.564	1	0.332372	0.4299768	0.2090711	0.1045074	0.2201349	0.2072529
72.664	1	0.257412	0.4096864	0.2071446	0.1039219	0.2157822	0.2099565
72.764	1	0.2729	0.4109306	0.2063149	0.1037223	0.2102288	0.2245196
72.864	1	0.314079	0.418037	0.2061055	0.1021811	0.2073722	0.229949

72.964	1	0.362747	0.4122926	0.2104396	0.1010404	0.2008252	0.2346169
73.064	1	0.39291	0.3891706	0.2125718	0.101679	0.1921862	0.2365717
73.164	1	0.317275	0.3977402	0.214691	0.1075359	0.1920473	0.2480342
73.264	1	0.283989	0.3928464	0.2285572	0.1082112	0.1948931	0.2604242
73.364	1	0.349052	0.3986985	0.264116	0.1063757	0.1948148	0.2539678
73.464	1	0.417523	0.4246699	0.2799636	0.1028984	0.1988198	0.2469877
73.564	1	0.463286	0.4095256	0.2625597	0.101601	0.1984456	0.2360301
73.664	1	0.450046	0.3921431	0.2396379	0.1042436	0.2012439	0.2328866
73.764	1	0.395989	0.3940273	0.2270082	0.1049026	0.2040535	0.2371101
73.864	1	0.438028	0.3983333	0.2211212	0.1065826	0.2043832	0.2455352
73.964	1	0.499186	0.4181849	0.2178075	0.1052932	0.203383	0.2564856
74.064	1	0.526963	0.4091955	0.2191207	0.1043276	0.206845	0.2669775
74.164	1	0.45465	0.3762809	0.2215354	0.1043261	0.205345	0.2585766
74.264	1	0.44981	0.3985089	0.2206483	0.102992	0.2058728	0.2661497
74.364	1	0.503352	0.4121439	0.2192941	0.1022257	0.2014143	0.2629248
74.464	1	0.502004	0.4252867	0.2218149	0.1047273	0.1999486	0.2599859
74.564	1	0.41085	0.4208609	0.2241103	0.1023236	0.1997312	0.2519329
74.664	1	0.328711	0.4005682	0.2264304	0.1022436	0.1980132	0.25261
74.764	1	0.297594	0.4042806	0.2273376	0.1023276	0.1965543	0.2504252
74.864	1	0.279476	0.4085554	0.2264438	0.1016523	0.1953219	0.239086
74.964	1	0.254993	0.4039634	0.2197769	0.1024169	0.1958921	0.2468844
75.064	1	0.243677	0.415535	0.2220915	0.105083	0.1969526	0.2636778
75.164	1	0.244169	0.4122737	0.2271743	0.1047086	0.1979302	0.2793257
75.264	1	0.268147	0.3899859	0.2152501	0.1047517	0.2001695	0.2831039
75.364	1	0.314818	0.3863322	0.2101867	0.1045061	0.1993824	0.2834281
75.464	1	0.371326	0.3879487	0.2093304	0.1051697	0.2002354	0.2832541
75.564	1	0.420764	0.3802665	0.2093726	0.105899	0.2066917	0.2446916
75.664	1	0.422987	0.4032034	0.2162859	0.1088005	0.2130438	0.2187421
75.764	1	0.313792	0.4060107	0.210868	0.1086556	0.2098517	0.209052
75.864	1	0.290308	0.411515	0.2105521	0.106818	0.2090869	0.2293276
75.964	1	0.301347	0.4163516	0.2119934	0.106352	0.2078597	0.2588086
76.064	1	0.325807	0.4054388	0.2233839	0.1096381	0.2024041	0.2786277
76.164	1	0.353803	0.3895259	0.2363664	0.1082675	0.1921484	0.2900886
76.264	1	0.375119	0.3836428	0.2463957	0.1098596	0.198983	0.2840027
76.364	1	0.420046	0.3919739	0.241515	0.1140529	0.2027308	0.2858765
76.464	1	0.452721	0.3869327	0.2284697	0.1122256	0.2021258	0.2699896
76.564	1	0.439817	0.3903814	0.2177702	0.1117304	0.2099899	0.2606425
76.664	1	0.397575	0.3995428	0.2049392	0.1095981	0.20273	0.2582014
76.764	1	0.42261	0.3882095	0.2061889	0.1134091	0.2031274	0.2397678
76.864	1	0.460249	0.4009581	0.210445	0.1134267	0.2000079	0.2257373
76.964	1	0.477919	0.4151201	0.2219991	0.1112511	0.2032375	0.2289922
77.064	1	0.470842	0.3709833	0.2430558	0.111383	0.2007611	0.2376267
77.164	1	0.468461	0.3887912	0.2491485	0.1087428	0.195921	0.2484575
77.264	1	0.405709	0.3950168	0.2315956	0.1080344	0.1911711	0.250752
77.364	1	0.285452	0.4016863	0.2207778	0.108098	0.1929657	0.2538732
77.464	1	0.29961	0.4133908	0.2207845	0.1071479	0.1946901	0.2406707
77.564	1	0.334178	0.405851	0.2190566	0.1165238	0.1961	0.2469971
77.664	1	0.376895	0.408537	0.2205025	0.1402919	0.1895314	0.2505448
77.764	1	0.416525	0.3875056	0.2217264	0.1627757	0.1944505	0.2405223
77.864	1	0.453889	0.3864013	0.2172642	0.1811511	0.2237638	0.2503584
77.964	1	0.490491	0.3840328	0.2119491	0.1869799	0.2244869	0.2594136
78.064	1	0.519213	0.3788113	0.2111287	0.1686557	0.2276374	0.2677207
78.164	1	0.522662	0.3963118	0.2157705	0.1394695	0.2158016	0.2594345
78.264	1	0.494815	0.3931004	0.2175326	0.1213068	0.203158	0.2323533
78.364	1	0.47135	0.385492	0.2222492	0.1170478	0.2047305	0.2250417
78.464	1	0.482197	0.3958175	0.2270167	0.1178634	0.210131	0.2351428
78.564	1	0.505941	0.4039167	0.2588677	0.1210275	0.216855	0.2459859
78.664	1	0.518609	0.4079149	0.3068102	0.1215948	0.2232626	0.2582566
78.764	1	0.517886	0.4046712	0.3427519	0.1237985	0.2352332	0.2652497
78.864	1	0.512797	0.3688225	0.3302385	0.1231892	0.2397889	0.2705461
78.964	1	0.52039	0.3732852	0.2553146	0.1224069	0.2210864	0.2792844
79.064	1	0.534854	0.3960569	0.2233788	0.125803	0.2256503	0.27385
79.164	1	0.52702	0.4093294	0.2176425	0.1264978	0.2306439	0.2699008
79.264	1	0.513187	0.3835735	0.2124077	0.1301741	0.2026056	0.2715755
79.364	1	0.499494	0.3765167	0.2095346	0.132893	0.1923691	0.2749068
79.464	1	0.504229	0.3914258	0.2101355	0.1258218	0.1893641	0.2661375
79.564	1	0.517368	0.382848	0.2097781	0.1333031	0.1962381	0.2617454
79.664	1	0.512069	0.3806803	0.2090723	0.1323722	0.2103448	0.2432487
79.764	1	0.493804	0.4019191	0.2173419	0.1258334	0.209616	0.2264018
79.864	1	0.511381	0.4275042	0.2226764	0.1228516	0.2105592	0.2251984
79.964	1	0.51042	0.4208137	0.2212739	0.1236769	0.2039295	0.2352144
80.064	1	0.502314	0.4049928	0.2230601	0.1249821	0.2118836	0.2340345
80.164	1	0.496555	0.4035161	0.2266448	0.1256817	0.2185052	0.2359274
80.264	1	0.481841	0.3746662	0.2366317	0.1235573	0.2037491	0.2348063
80.364	1	0.393002	0.3648035	0.2459227	0.1235844	0.1904664	0.2124147
80.464	1	0.312147	0.3868822	0.2396358	0.1206456	0.1875151	0.2082024
80.564	1	0.357641	0.3798026	0.2209818	0.1194639	0.1876108	0.2076869
80.664	1	0.420254	0.3864955	0.2176879	0.1202163	0.1936821	0.2121072
80.764	1	0.478206	0.3898204	0.2210484	0.1250025	0.2046893	0.2111026
80.864	1	0.501542	0.3922778	0.2246323	0.1269983	0.2011844	0.2197251
80.964	1	0.498053	0.3805141	0.2273488	0.1240658	0.1981197	0.2279605
81.064	1	0.51996	0.3763232	0.2313911	0.1228699	0.198395	0.24527
81.164	1	0.54226	0.390849	0.2316094	0.1210533	0.2130159	0.2580683
81.264	1	0.545731	0.4005956	0.2355181	0.1258578	0.2227521	0.2558127
81.364	1	0.549939	0.3956955	0.2375205	0.1252156	0.2116942	0.2536761
81.464	1	0.547961	0.3675714	0.2321732	0.1266747	0.2073376	0.2513263
81.564	1	0.537944	0.3878954	0.2240265	0.128394	0.2108226	0.2467559
81.664	1	0.471161	0.4011555	0.2178472	0.1282108	0.2135346	0.2464843
81.764	1	0.407292	0.3909877	0.2106588	0.1308753	0.2176484	0.2472968
81.864	1	0.368702	0.3853965	0.2103527	0.131819	0.2135345	0.2629302
81.964	1	0.397048	0.3816051	0.208786	0.1318826	0.1996844	0.2563385
82.064	1	0.436525	0.3894016	0.2106511	0.1295356	0.1944201	0.2594762
82.164	1	0.470933	0.3753439	0.2139298	0.129542	0.1920341	0.2600032
82.264	1	0.459965	0.366593	0.2203461	0.1299742	0.1885833	0.2750441
82.364	1	0.432909	0.3711816	0.2200299	0.1313508	0.1916107	0.26425

82.464	1	0.428633	0.3763306	0.2259353	0.1306571	0.1956019	0.2664551
82.564	1	0.396336	0.3852029	0.2300833	0.1307273	0.1947836	0.2847706
82.664	1	0.324171	0.373771	0.2300027	0.1326934	0.1920955	0.2908935
82.764	1	0.251981	0.3795943	0.2279815	0.1350654	0.1885838	0.2710816
82.864	1	0.225271	0.3703921	0.2283621	0.1363248	0.1867204	0.2445602
82.964	1	0.226892	0.3674957	0.2305986	0.1326459	0.1916917	0.2363861
83.064	1	0.21843	0.3759621	0.2330444	0.1326717	0.1940569	0.2233527
83.164	1	0.207025	0.3881811	0.2336294	0.1323523	0.1920678	0.219146
83.264	1	0.196615	0.3791373	0.2333403	0.1350006	0.1959425	0.2211521
83.364	1	0.186224	0.3765465	0.232617	0.134179	0.2052089	0.2342546
83.464	1	0.193083	0.3956552	0.2351575	0.1346676	0.211001	0.2488451
83.564	1	0.204085	0.4025249	0.2362809	0.1379999	0.2039226	0.2630935
83.664	1	0.219949	0.3854564	0.2382732	0.1391949	0.199742	0.284002
83.764	1	0.230791	0.3784455	0.235337	0.1348159	0.1964156	0.2944308
83.864	1	0.234787	0.4005525	0.2347435	0.1310067	0.204755	0.2833373
83.964	1	0.237627	0.4026913	0.2342073	0.1299315	0.2213393	0.2656811
84.064	1	0.242383	0.3791622	0.2387151	0.1282697	0.2278967	0.2555015
84.164	1	0.260159	0.3819508	0.245847	0.1304796	0.2308058	0.248138
84.264	1	0.308285	0.3840082	0.2456431	0.1321176	0.2241484	0.2336647
84.364	1	0.368545	0.3818245	0.2479839	0.1353222	0.2061805	0.2403083
84.464	1	0.408455	0.3733996	0.2484568	0.1369431	0.2018994	0.2507338
84.564	1	0.403445	0.3390794	0.2410029	0.1373652	0.2072611	0.2375499
84.664	1	0.3177	0.3588139	0.2340014	0.1346195	0.2164356	0.234154
84.764	1	0.338064	0.3868871	0.2303746	0.131033	0.2152284	0.2266401
84.864	1	0.384107	0.3761085	0.2339405	0.1704013	0.2043809	0.2167588
84.964	1	0.421826	0.3818501	0.241624	0.2123866	0.201745	0.2267976
85.064	1	0.40004	0.3966161	0.2447782	0.244995	0.2066258	0.2327915
85.164	1	0.305388	0.393939	0.246478	0.2691267	0.2124732	0.2270064
85.264	1	0.255337	0.3753216	0.2420683	0.2420755	0.2081146	0.2264376
85.364	1	0.243649	0.3642795	0.2382764	0.2214175	0.1991438	0.2272513
85.464	1	0.257368	0.3661273	0.2372941	0.2053293	0.2046063	0.2262258
85.564	1	0.298686	0.3638817	0.2406839	0.1745989	0.2062744	0.2243089
85.664	1	0.29932	0.3903134	0.239743	0.1641972	0.2058757	0.2219437
85.764	1	0.256118	0.4025659	0.2331909	0.1626019	0.2131128	0.2130152
85.864	1	0.217551	0.3814703	0.2330761	0.1599349	0.2081759	0.2167797
85.964	1	0.178932	0.3785267	0.2362531	0.144283	0.202462	0.2299986
86.064	1	0.1719	0.3710528	0.2407298	0.1335303	0.2271587	0.2235572
86.164	1	0.16685	0.3751269	0.2457873	0.1291048	0.2709615	0.2223919
86.264	1	0.171353	0.3730854	0.2423475	0.1298367	0.3158934	0.2273594
86.364	1	0.179918	0.3665738	0.2377201	0.1285139	0.2940234	0.2363207
86.464	1	0.19284	0.3334335	0.2274248	0.1291515	0.2389544	0.2475889
86.564	1	0.204874	0.3284363	0.2258188	0.1306877	0.2116183	0.2440366
86.664	1	0.211102	0.3694109	0.231507	0.132657	0.204606	0.2282772
86.764	1	0.212507	0.391335	0.2327468	0.1355672	0.208102	0.2251516
86.864	1	0.21544	0.3935528	0.232652	0.1358085	0.2092227	0.2274152
86.964	1	0.209684	0.3780736	0.2296464	0.1436714	0.2059956	0.2307312
87.064	1	0.202033	0.3729043	0.232511	0.1594566	0.2042083	0.2353314
87.164	1	0.198364	0.3721613	0.2310131	0.1839472	0.1979244	0.2424879
87.264	1	0.196835	0.3703465	0.2325003	0.2157384	0.196604	0.2386293
87.364	1	0.192881	0.3723747	0.2368085	0.2612618	0.1976885	0.2456189
87.464	1	0.190273	0.3857146	0.2478228	0.2791895	0.198363	0.2420398
87.564	1	0.190721	0.3898343	0.2540328	0.2538652	0.2029072	0.248828
87.664	1	0.198548	0.3789093	0.242113	0.1972662	0.2009984	0.242856
87.764	1	0.206448	0.3679044	0.2364791	0.1698451	0.2013858	0.2466917
87.864	1	0.213798	0.3809437	0.2291326	0.1649872	0.2124826	0.2464719
87.964	1	0.22152	0.393341	0.2284976	0.1712989	0.2270446	0.2487617
88.064	1	0.224826	0.3957658	0.2318712	0.1950461	0.233728	0.2630895
88.164	1	0.229405	0.3777704	0.232211	0.2128457	0.2268131	0.3137114
88.264	1	0.242127	0.3805356	0.2311575	0.2173018	0.2302808	0.3535049
88.364	1	0.251117	0.3828589	0.2243949	0.1726752	0.2453897	0.299957
88.464	1	0.246277	0.3826762	0.2257718	0.1470569	0.249244	0.2265244
88.564	1	0.233271	0.3885936	0.2290456	0.1372807	0.229926	0.2107619
88.664	1	0.229206	0.3924915	0.2294691	0.1340073	0.231321	0.1965601
88.764	1	0.242074	0.3904255	0.2328356	0.1308711	0.2222888	0.1968268
88.864	1	0.282521	0.3821123	0.2307914	0.1288206	0.2105429	0.1997938
88.964	1	0.326847	0.3607871	0.2287827	0.1298981	0.2113207	0.2032015
89.064	1	0.369824	0.3584684	0.2319263	0.1278295	0.2089465	0.2044738
89.164	1	0.373212	0.3718195	0.2335797	0.1325138	0.2118068	0.1918836
89.264	1	0.247233	0.3734468	0.2310953	0.133582	0.2159665	0.1901195
89.364	1	0.190167	0.366962	0.2236553	0.1347427	0.2157919	0.1888643
89.464	1	0.19283	0.3668201	0.2301184	0.1365477	0.2021857	0.1913406
89.564	1	0.207957	0.3739533	0.2324337	0.1360457	0.1989939	0.191921
89.664	1	0.229937	0.3760385	0.2418093	0.1464773	0.203123	0.1896264
89.764	1	0.249834	0.376725	0.2443989	0.1535201	0.2073165	0.190419
89.864	1	0.227331	0.3768064	0.2351801	0.1604485	0.2043049	0.1946091
89.964	1	0.220414	0.3882111	0.2312563	0.155885	0.2088707	0.197263
90.064	1	0.232605	0.3996544	0.2347444	0.1751492	0.2123508	0.1987141
90.164	1	0.246272	0.3885802	0.2420203	0.2261748	0.2412729	0.197
90.264	1	0.259275	0.3914369	0.2516319	0.2790938	0.2579601	0.2058693
90.364	1	0.263964	0.3787494	0.2835137	0.3121451	0.2691982	0.2381344
90.464	1	0.275535	0.3776624	0.3167934	0.2659919	0.2736478	0.2603495
90.564	1	0.295315	0.3985132	0.3352391	0.2020075	0.2814972	0.281146
90.664	1	0.300391	0.4023973	0.3440588	0.2202493	0.275124	0.3116813
90.764	1	0.283223	0.3850965	0.312929	0.2417237	0.2661784	0.3095901
90.864	1	0.292247	0.3989663	0.2840054	0.243817	0.2444696	0.3080088
90.964	1	0.308177	0.4066796	0.2256409	0.1797702	0.2343757	0.2469001
91.064	1	0.335169	0.4111457	0.2157433	0.1881497	0.2328646	0.2111855
91.164	1	0.349733	0.4127845	0.2097412	0.1809532	0.2322296	0.1993627
91.264	1	0.342357	0.4061685	0.2083993	0.1789134	0.2364623	0.1989079
91.364	1	0.345609	0.3986794	0.2067533	0.1934991	0.2408074	0.2042761
91.464	1	0.363216	0.3768593	0.1994279	0.205096	0.2463206	0.2102291
91.564	1	0.3864	0.3672137	0.2036211	0.2171461	0.2564767	0.2118505
91.664	1	0.397722	0.3750313	0.2658474	0.2237338	0.2418898	0.2090551
91.764	1	0.343323	0.3907478	0.3244909	0.1909763	0.2453325	0.2071841
91.864	1	0.342611	0.3800299	0.3574403	0.1575273	0.2515873	0.2113523

91.964	1	0.392326	0.388309	0.3797396	0.1493736	0.2545767	0.2421256
92.064	1	0.431291	0.3902045	0.3805929	0.1682143	0.2328745	0.2710771
92.164	1	0.402131	0.3960379	0.3379395	0.1684452	0.2390054	0.286896
92.264	1	0.327351	0.4047532	0.3098947	0.1519165	0.2421491	0.3098299
92.364	1	0.363468	0.40024	0.2887001	0.1608429	0.2470115	0.2950097
92.464	1	0.404241	0.4034036	0.2692192	0.1749159	0.2417997	0.235264
92.564	1	0.422155	0.4015969	0.2686144	0.1749164	0.2403173	0.2141447
92.664	1	0.393896	0.3978876	0.278109	0.1855568	0.2349375	0.2146858
92.764	1	0.38497	0.3977585	0.2720571	0.1964064	0.2310153	0.2191688
92.864	1	0.420266	0.3953717	0.2642242	0.1984984	0.2332576	0.206186
92.964	1	0.449606	0.3933213	0.2623405	0.2095919	0.2360837	0.2002607
93.064	1	0.463528	0.3851267	0.2677734	0.2117818	0.23445	0.2011382
93.164	1	0.486698	0.3883463	0.2873831	0.1994541	0.2238591	0.2003707
93.264	1	0.498607	0.3830648	0.2935194	0.1821336	0.2223885	0.1984324
93.364	1	0.516875	0.3802008	0.3139698	0.1750353	0.2167846	0.2052863
93.464	1	0.535463	0.3755045	0.3417448	0.1629033	0.2218897	0.21541
93.564	1	0.563404	0.3762899	0.334327	0.1655427	0.2152	0.23255
93.664	1	0.561958	0.3857409	0.3043761	0.1690441	0.2150386	0.2358554
93.764	1	0.573141	0.3929341	0.2978737	0.1597188	0.2202735	0.2381114
93.864	1	0.565945	0.3961487	0.2917591	0.1478758	0.220937	0.2319764
93.964	1	0.593441	0.3997507	0.2934248	0.1527501	0.2214946	0.2395151
94.064	1	0.587153	0.3987554	0.2903814	0.1622748	0.2135526	0.2643776
94.164	1	0.513622	0.3904379	0.280057	0.1626322	0.2097868	0.2852634
94.264	1	0.38341	0.3890428	0.2656688	0.1644143	0.212501	0.3070857
94.364	1	0.265245	0.3908493	0.2602827	0.1587704	0.2149853	0.2979083
94.464	1	0.303271	0.3823037	0.2575716	0.1602477	0.2140679	0.2349487
94.564	1	0.37223	0.3857826	0.2509248	0.1622652	0.2121051	0.2169727
94.664	1	0.451103	0.3954208	0.2433259	0.1590275	0.2097579	0.2143115
94.764	1	0.512292	0.3990218	0.2430385	0.1542835	0.2088398	0.2111285
94.864	1	0.541516	0.4008459	0.2467738	0.1555734	0.2034528	0.2069448
94.964	1	0.565332	0.4043519	0.2532811	0.1593217	0.1974157	0.2082612
95.064	1	0.594354	0.4057566	0.254385	0.1579971	0.1980388	0.2084521
95.164	1	0.593586	0.3922458	0.2571945	0.1583996	0.2086056	0.2104951
95.264	1	0.601087	0.3882277	0.2533131	0.1576174	0.2066116	0.2089637
95.364	1	0.594059	0.3931594	0.2561376	0.1591194	0.2071439	0.2061987
95.464	1	0.578685	0.3848153	0.2601275	0.1639645	0.2086885	0.207765
95.564	1	0.53067	0.3848501	0.2541281	0.165988	0.2056809	0.2143609
95.664	1	0.462095	0.3820915	0.2511765	0.1641333	0.2009855	0.2270436
95.764	1	0.439529	0.378464	0.2504489	0.1656221	0.1986729	0.2372933
95.864	1	0.387581	0.3828488	0.2456879	0.1646749	0.1995189	0.2418534
95.964	1	0.335425	0.3841776	0.2455509	0.1670406	0.1970887	0.2461732
96.064	1	0.364071	0.3857259	0.2423342	0.1689129	0.1942747	0.2510371
96.164	1	0.415529	0.387661	0.2408473	0.1663925	0.1909175	0.2399095
96.264	1	0.460225	0.3859616	0.2440768	0.1668604	0.1889596	0.2321108
96.364	1	0.499774	0.3790094	0.2453248	0.1622778	0.1925379	0.2460489
96.464	1	0.530958	0.3749145	0.246888	0.1649891	0.1967387	0.2530786
96.564	1	0.546571	0.3741847	0.2530793	0.1670825	0.2002902	0.2535804
96.664	1	0.537004	0.3796918	0.253271	0.1648446	0.1920411	0.2408899
96.764	1	0.554782	0.3846054	0.24921	0.1581405	0.1905143	0.2406241
96.864	1	0.555169	0.3834744	0.2359002	0.1543789	0.1911005	0.2452867
96.964	1	0.490974	0.3718685	0.2284172	0.1586908	0.1926173	0.2729998
97.064	1	0.360927	0.3617225	0.2350385	0.1654922	0.1976658	0.2808114
97.164	1	0.273968	0.3678682	0.2407951	0.1740173	0.1963674	0.3001634
97.264	1	0.259978	0.373012	0.2430687	0.1763347	0.19441	0.2804744
97.364	1	0.244886	0.3678023	0.2374498	0.1677962	0.1924407	0.2366886
97.464	1	0.235783	0.3668742	0.2391597	0.1590645	0.1958126	0.2389807
97.564	1	0.226637	0.3685295	0.2427052	0.1590619	0.1995156	0.2359672
97.664	1	0.213733	0.3673411	0.2493009	0.1560882	0.189631	0.2353074
97.764	1	0.210742	0.3758368	0.254252	0.1578151	0.1884727	0.2336797
97.864	1	0.213687	0.3823799	0.2517492	0.1554678	0.1924651	0.2235925
97.964	1	0.22304	0.3797672	0.2477078	0.1627835	0.1902915	0.2223115
98.064	1	0.240742	0.3793062	0.2509909	0.1649208	0.1946758	0.2180533
98.164	1	0.266141	0.3690891	0.2532162	0.1654462	0.2013483	0.2122239
98.264	1	0.298631	0.3753439	0.2541395	0.1671394	0.2054247	0.2165781
98.364	1	0.330512	0.3754605	0.250159	0.1616426	0.2045521	0.2169511
98.464	1	0.365414	0.3883743	0.2435648	0.1596875	0.2040966	0.2211851
98.564	1	0.391395	0.3920763	0.2749502	0.151356	0.1998427	0.2248904
98.664	1	0.413651	0.3916657	0.288702	0.1505852	0.1920092	0.2299551
98.764	1	0.414283	0.3925389	0.2727843	0.1471183	0.1913181	0.2287624
98.864	1	0.404604	0.4020242	0.2538174	0.1505215	0.1899586	0.2266871
98.964	1	0.357348	0.4030294	0.2438992	0.1515039	0.1887012	0.21521
99.064	1	0.281464	0.3995874	0.2467847	0.152379	0.1895995	0.2118566
99.164	1	0.261521	0.405064	0.2515911	0.1546705	0.1854655	0.2020389
99.264	1	0.247319	0.4126909	0.2480754	0.1550737	0.1832766	0.2099089
99.364	1	0.239252	0.4099385	0.2445051	0.1510201	0.1816258	0.2100231
99.464	1	0.237265	0.3999387	0.2457843	0.1516856	0.1821039	0.2061127
99.564	1	0.234807	0.3987313	0.2452726	0.1552281	0.1809875	0.2145117
99.664	1	0.226359	0.3962332	0.2674815	0.1555055	0.1817426	0.2144315
99.764	1	0.22226	0.4031284	0.2788541	0.1515678	0.1821035	0.2219401
99.864	1	0.214736	0.4079396	0.2661702	0.1508716	0.184505	0.220377
99.964	1	0.205668	0.4104914	0.2548909	0.1523114	0.1937628	0.2159829
100.064	1	0.197476	0.4105434	0.2502969	0.1585413	0.1943189	0.207571
100.164	1	0.191742	0.4113563	0.2440983	0.161187	0.1932906	0.2114182
100.264	1	0.19003	0.4039325	0.2382946	0.1563328	0.1904111	0.2098699
100.364	1	0.190626	0.4083057	0.236345	0.1509054	0.1991637	0.207536
100.464	1	0.190447	0.4078136	0.239456	0.1531667	0.1998783	0.2001744
100.564	1	0.192831	0.4055139	0.2416803	0.1652445	0.1986364	0.2025543
100.664	1	0.197315	0.4119302	0.2401389	0.164525	0.1952852	0.2049575
100.764	1	0.198928	0.4054014	0.2419372	0.1533604	0.1901364	0.2046474
100.864	1	0.196231	0.4009793	0.2472547	0.1501832	0.1905128	0.2045196
100.964	1	0.188067	0.4044517	0.2493666	0.1503216	0.1940188	0.2057075
101.064	1	0.185064	0.4075887	0.2481485	0.1490622	0.1970423	0.2127014
101.164	1	0.184338	0.4087165	0.2495931	0.1500884	0.1974908	0.2102198
101.264	1	0.186213	0.3985958	0.2472762	0.1545292	0.2006845	0.2016588
101.364	1	0.191091	0.3976344	0.252108	0.1586845	0.2009393	0.2005998

101.464	1	0.197071	0.3983653	0.2459395	0.1599679	0.1953292	0.1969337
101.564	1	0.201226	0.4028585	0.2327964	0.1607432	0.19513	0.2013554
101.664	1	0.205475	0.40679	0.2328363	0.1566871	0.1963428	0.2010276
101.764	1	0.208626	0.4129238	0.2368277	0.1520921	0.1949141	0.1949089
101.864	1	0.207467	0.4101297	0.2336334	0.1521665	0.1893791	0.2000542
101.964	1	0.200188	0.4051714	0.2324204	0.1602969	0.191157	0.205381
102.064	1	0.191862	0.4009882	0.2355284	0.1578131	0.1892269	0.2223302
102.164	1	0.190355	0.4007909	0.2405886	0.1508243	0.1878223	0.2216209
102.264	1	0.190944	0.3997338	0.2378843	0.1479558	0.1887774	0.2186147
102.364	1	0.19073	0.3967478	0.2313301	0.1500875	0.1863218	0.2161421
102.464	1	0.190819	0.3957303	0.2318535	0.1549471	0.1867641	0.2180778
102.564	1	0.190386	0.3947816	0.2343682	0.1589723	0.1878798	0.2138526
102.664	1	0.190844	0.4039302	0.2368967	0.1521799	0.1879655	0.209999
102.764	1	0.191019	0.4043362	0.2400643	0.150269	0.1838627	0.2123958
102.864	1	0.192284	0.401251	0.2367273	0.1477634	0.1875249	0.2182807
102.964	1	0.195493	0.3926585	0.2381576	0.1522852	0.1866556	0.2168222
103.064	1	0.202231	0.3877865	0.2372317	0.1613179	0.191895	0.2018771
103.164	1	0.210822	0.3916093	0.2324627	0.1617367	0.194036	0.1995177
103.264	1	0.223373	0.394819	0.2331761	0.1581469	0.1929605	0.2044961
103.364	1	0.231327	0.4017413	0.2335698	0.1665216	0.1946194	0.2126112
103.464	1	0.231177	0.4004078	0.2343833	0.1765407	0.1970833	0.2195298
103.564	1	0.22814	0.4019734	0.2293761	0.1795041	0.1956767	0.2149087
103.664	1	0.230192	0.395878	0.2249232	0.1812112	0.1930351	0.2194677
103.764	1	0.237105	0.3901928	0.2264446	0.179825	0.1937606	0.2168921
103.864	1	0.248473	0.3871208	0.2304623	0.1620892	0.1957357	0.2116061
103.964	1	0.249364	0.3961244	0.2320956	0.155796	0.1980148	0.2164385
104.064	1	0.240928	0.3859476	0.2245513	0.1511372	0.1910634	0.2223514
104.164	1	0.237149	0.377138	0.2273642	0.1475617	0.1936309	0.2180699
104.264	1	0.244785	0.3829697	0.231847	0.1447501	0.1972347	0.2108202
104.364	1	0.244498	0.3969971	0.231703	0.1542908	0.2004755	0.2158653
104.464	1	0.247737	0.3959391	0.2299521	0.1600295	0.2002433	0.2128416
104.564	1	0.253912	0.3892569	0.2339044	0.1656268	0.1986133	0.2056597
104.664	1	0.263611	0.387905	0.2369194	0.1757638	0.1934603	0.2027474
104.764	1	0.273676	0.3918371	0.2276588	0.1858341	0.1946486	0.1934375
104.864	1	0.28747	0.3964356	0.2294751	0.1908288	0.1951849	0.1898277
104.964	1	0.299998	0.3930981	0.2293281	0.1974095	0.191636	0.1967208
105.064	1	0.306771	0.3967001	0.232687	0.1913561	0.1906257	0.2048551
105.164	1	0.327156	0.3925331	0.2389725	0.1847178	0.1960665	0.2175416
105.264	1	0.339061	0.3803779	0.2369784	0.1775226	0.1984392	0.2315262
105.364	1	0.344148	0.385044	0.2329545	0.1667943	0.199534	0.236304
105.464	1	0.349991	0.3908857	0.2299607	0.1592793	0.1993585	0.2217277
105.564	1	0.358414	0.3946996	0.2387745	0.1646642	0.2049675	0.2072527
105.664	1	0.359141	0.3919212	0.2569882	0.162366	0.2053137	0.204426
105.764	1	0.331807	0.3919624	0.2484404	0.1609086	0.2120156	0.2030725
105.864	1	0.345532	0.3812976	0.2220819	0.1583598	0.2147632	0.20248
105.964	1	0.375718	0.3770422	0.216834	0.1577989	0.2081813	0.2083708
106.064	1	0.411075	0.3843771	0.222458	0.1587696	0.2026302	0.2084716
106.164	1	0.433219	0.3880006	0.2284754	0.1676264	0.2037828	0.2098173
106.264	1	0.436646	0.3830597	0.2325221	0.1641693	0.2008541	0.206197
106.364	1	0.423044	0.382874	0.2328961	0.1613043	0.1969913	0.2043036
106.464	1	0.436499	0.3816795	0.2337073	0.1629946	0.1976039	0.2049102
106.564	1	0.468884	0.3787448	0.2341675	0.1621227	0.2023628	0.2090028
106.664	1	0.462264	0.3822781	0.2386795	0.157836	0.2057823	0.2155123
106.764	1	0.411678	0.3827576	0.2356497	0.1574586	0.2105428	0.2150858
106.864	1	0.440502	0.362951	0.2314219	0.1520618	0.2052412	0.2075173
106.964	1	0.458948	0.3615994	0.2316197	0.1500039	0.2097014	0.2128938
107.064	1	0.423297	0.3653531	0.2370806	0.1515013	0.2132922	0.2134762
107.164	1	0.441737	0.3718537	0.2376182	0.1507797	0.2129758	0.2104042
107.264	1	0.478975	0.375394	0.2365609	0.1504795	0.2101476	0.2085741
107.364	1	0.478031	0.3854161	0.2331499	0.1457762	0.2100522	0.2016126
107.464	1	0.432941	0.3903648	0.2308615	0.1459747	0.2075363	0.1998629
107.564	1	0.470566	0.3846861	0.2350884	0.1431506	0.2057665	0.1993755
107.664	1	0.500078	0.3868914	0.2409888	0.1453767	0.2038953	0.2006857
107.764	1	0.496123	0.3949023	0.2395975	0.1429457	0.206361	0.1982386
107.864	1	0.510923	0.3905607	0.2364902	0.1481044	0.2075433	0.1948757
107.964	1	0.510434	0.386003	0.237886	0.156699	0.210537	0.1916969
108.064	1	0.535786	0.4004352	0.2365808	0.1633762	0.2174718	0.1940358
108.164	1	0.529193	0.4113707	0.2339334	0.1668363	0.2072406	0.1927854
108.264	1	0.468071	0.4055258	0.2315858	0.1620249	0.207335	0.199569
108.364	1	0.459349	0.4055724	0.2316657	0.1538438	0.2109422	0.2079875
108.464	1	0.465437	0.4078771	0.2348355	0.1520433	0.2116899	0.2113373
108.564	1	0.473851	0.4065534	0.2291491	0.1561985	0.2097367	0.2104094
108.664	1	0.502196	0.3984445	0.2282961	0.1575577	0.2073228	0.2180452
108.764	1	0.506266	0.4043453	0.2337072	0.155699	0.2067803	0.2309714
108.864	1	0.517516	0.4105318	0.2380193	0.150546	0.2089399	0.2284742
108.964	1	0.522346	0.402037	0.2375784	0.1486309	0.2100987	0.229085
109.064	1	0.50054	0.4040242	0.2336332	0.1505653	0.2086949	0.2391117
109.164	1	0.478455	0.4076247	0.2298607	0.1558107	0.2203789	0.2378322
109.264	1	0.458816	0.3973264	0.2296733	0.1566191	0.2247187	0.2197049
109.364	1	0.470812	0.3974616	0.2315817	0.1584952	0.2227411	0.2064024
109.464	1	0.478963	0.3941385	0.2312256	0.1489216	0.2167203	0.2013717
109.564	1	0.486555	0.3882026	0.232633	0.1501312	0.2080763	0.210005
109.664	1	0.498846	0.3913692	0.2333701	0.1536835	0.2083994	0.2197543
109.764	1	0.515779	0.3821734	0.2333339	0.1597334	0.2110084	0.2136672
109.864	1	0.542998	0.386878	0.2315668	0.1607361	0.2160076	0.2190778
109.964	1	0.549329	0.3981498	0.2368633	0.1697246	0.2202818	0.2183807
110.064	1	0.542306	0.4046225	0.2382097	0.1728965	0.2123003	0.2343674
110.164	1	0.518226	0.4090269	0.2368726	0.1644855	0.2148654	0.2243997
110.264	1	0.544311	0.4064827	0.2381772	0.1553158	0.2143463	0.2174469
110.364	1	0.567918	0.4032243	0.2384085	0.1515377	0.2171392	0.2059266
110.464	1	0.552966	0.4066821	0.2406113	0.1624832	0.2092584	0.1998542
110.564	1	0.508891	0.4050733	0.2389983	0.1673762	0.2048478	0.1967084
110.664	1	0.535717	0.3973624	0.234826	0.1619382	0.2049659	0.2041039
110.764	1	0.561596	0.400877	0.2423462	0.1465134	0.2099279	0.2088427
110.864	1	0.566141	0.3992026	0.2460035	0.1424478	0.2081414	0.1996728

110.964	1	0.566272	0.400612	0.2423908	0.1414773	0.2136012	0.1987723
111.064	1	0.562449	0.3991765	0.2411269	0.1447089	0.2161044	0.1993212
111.164	1	0.551809	0.4014457	0.238387	0.1498128	0.2152177	0.2023889
111.264	1	0.538564	0.3828053	0.2331832	0.1504935	0.2075876	0.2017048
111.364	1	0.479406	0.3407477	0.2347884	0.1536566	0.2079048	0.2014312
111.464	1	0.506968	0.3524325	0.235918	0.1483837	0.2149447	0.2095772
111.564	1	0.538981	0.3756496	0.2389355	0.147019	0.2177124	0.2074841
111.664	1	0.565778	0.3814157	0.2422797	0.1483442	0.2138295	0.2058741
111.764	1	0.573108	0.3971419	0.2434574	0.1479545	0.2160308	0.201755
111.864	1	0.586193	0.4047275	0.241597	0.1450658	0.2127785	0.2040679
111.964	1	0.613409	0.3933142	0.2420657	0.1434899	0.2064321	0.2011434
112.064	1	0.629836	0.3822793	0.2447146	0.1484582	0.2082511	0.2013233
112.164	1	0.631771	0.358477	0.2356851	0.1518085	0.2134584	0.2062348
112.264	1	0.641841	0.3716779	0.237243	0.14953	0.2145231	0.2144969
112.364	1	0.660198	0.3918739	0.2465984	0.1549489	0.2123668	0.2019009
112.464	1	0.677971	0.4100615	0.2451596	0.1559373	0.2158168	0.205698
112.564	1	0.678693	0.4074943	0.2479907	0.1599394	0.2098773	0.2014468
112.664	1	0.637698	0.4021139	0.2427414	0.146576	0.2104643	0.2036406
112.764	1	0.630497	0.4014721	0.2413462	0.1473369	0.2173652	0.2141075
112.864	1	0.651306	0.4022597	0.2420363	0.160945	0.2050151	0.2183091
112.964	1	0.649292	0.4065962	0.243443	0.1683937	0.2032571	0.2270274
113.064	1	0.627345	0.4047841	0.2432254	0.1636521	0.2079511	0.2291311
113.164	1	0.60615	0.4037948	0.2405753	0.1715497	0.2126266	0.2157403
113.264	1	0.595581	0.4025677	0.2395245	0.1647134	0.2139599	0.2108835
113.364	1	0.592587	0.4011899	0.2410559	0.1597061	0.2183665	0.2112039
113.464	1	0.613866	0.3941986	0.242026	0.1526291	0.2169601	0.2031785
113.564	1	0.618104	0.3963504	0.2438463	0.1487299	0.2156665	0.1975776
113.664	1	0.563317	0.403845	0.2448643	0.150278	0.215052	0.1980751
113.764	1	0.566562	0.402649	0.2461595	0.1500919	0.2114004	0.1995205
113.864	1	0.570048	0.3941891	0.244044	0.1555961	0.2055655	0.2078376
113.964	1	0.5901	0.4016136	0.2408748	0.1555989	0.2047009	0.218075
114.064	1	0.59601	0.3976726	0.2418098	0.1559	0.2077148	0.2218093
114.164	1	0.611764	0.3957894	0.2418635	0.1594268	0.2054768	0.2249093
114.264	1	0.596142	0.3893992	0.2423839	0.1503361	0.204002	0.2213833
114.364	1	0.597858	0.3868057	0.2460485	0.1452928	0.2086273	0.2175651
114.464	1	0.629937	0.3936262	0.2460853	0.1431788	0.2077961	0.2061779
114.564	1	0.63084	0.396033	0.2499583	0.1465801	0.2012272	0.2065175
114.664	1	0.630235	0.3896132	0.2563914	0.1431979	0.1958335	0.1918583
114.764	1	0.649033	0.3882706	0.2582945	0.1508393	0.2007564	0.1803628
114.864	1	0.676577	0.3860914	0.2549524	0.1480941	0.2126672	0.177646
114.964	1	0.670266	0.3852506	0.2583502	0.150928	0.2226707	0.1831342
115.064	1	0.62403	0.3925171	0.2542681	0.1532338	0.2313652	0.1867699
115.164	1	0.637149	0.4000302	0.2584976	0.1538876	0.2382156	0.1964848
115.264	1	0.641647	0.4011207	0.2591315	0.1503092	0.2552887	0.1997156
115.364	1	0.661553	0.3927302	0.2554395	0.1499607	0.2717333	0.2027072
115.464	1	0.670419	0.3902372	0.2565706	0.1522863	0.2639042	0.1973229
115.564	1	0.675263	0.3861994	0.2514436	0.1458751	0.2338015	0.1962947
115.664	1	0.667634	0.3841936	0.249423	0.1453673	0.2166711	0.202993
115.764	1	0.668587	0.3887255	0.2444932	0.1467727	0.2149319	0.2115836
115.864	1	0.668979	0.3924102	0.2445463	0.1490181	0.2091113	0.2132581
115.964	1	0.65516	0.3925718	0.2450226	0.1440159	0.2135544	0.197028
116.064	1	0.661588	0.3975109	0.2540686	0.1397401	0.2119654	0.1892856
116.164	1	0.691699	0.3922017	0.2585654	0.1440239	0.2129558	0.1907922
116.264	1	0.703907	0.3807211	0.2528556	0.1474337	0.216984	0.1918179
116.364	1	0.70313	0.3790904	0.2524011	0.1444118	0.2346669	0.2071349
116.464	1	0.69859	0.3729201	0.2530236	0.1435909	0.2480227	0.2074732
116.564	1	0.721354	0.3724648	0.2581183	0.1412087	0.2389216	0.2036545
116.664	1	0.734726	0.3785812	0.256377	0.1409571	0.2249227	0.1959182
116.764	1	0.737344	0.3872615	0.2568117	0.1402711	0.2173583	0.1857154
116.864	1	0.742618	0.3950107	0.2521785	0.1367602	0.2070741	0.1803912
116.964	1	0.754978	0.3945513	0.2511637	0.1351713	0.2083998	0.1861384
117.064	1	0.703991	0.3956599	0.2532249	0.1372639	0.2091606	0.1959626
117.164	1	0.724831	0.3957137	0.2535503	0.1413461	0.2139434	0.1897269
117.264	1	0.704319	0.3962233	0.2565202	0.1362666	0.2112657	0.182435
117.364	1	0.60387	0.394941	0.2544669	0.1366095	0.2125971	0.1798652
117.464	1	0.345379	0.3966402	0.2553689	0.1359653	0.214898	0.1778333
117.564	1	0.36917	0.400896	0.2538547	0.1365549	0.2162244	0.1795495
117.664	1	0.403254	0.3998589	0.2490844	0.1419902	0.2197404	0.1811859
117.764	1	0.436829	0.3959807	0.24843	0.1416304	0.2158081	0.1807026
117.864	1	0.479864	0.3963402	0.2473596	0.1434521	0.2286461	0.1801386
117.964	1	0.51646	0.3948226	0.2462626	0.1379772	0.2327822	0.1828712
118.064	1	0.542128	0.3941342	0.2448727	0.1385233	0.232149	0.1880069
118.164	1	0.550762	0.3973464	0.2465538	0.1333634	0.2191593	0.1877257
118.264	1	0.518552	0.4047691	0.2496278	0.1316404	0.2143454	0.185886
118.364	1	0.529025	0.407771	0.2534381	0.1305492	0.2154828	0.1809083
118.464	1	0.5521	0.4031658	0.2453835	0.129413	0.2128539	0.1771068
118.564	1	0.587799	0.3977483	0.2391606	0.1330063	0.2178251	0.1809339
118.664	1	0.613631	0.3999185	0.2394806	0.1348042	0.2148268	0.1863273
118.764	1	0.633418	0.4035703	0.2469648	0.12923	0.2124845	0.1819071
118.864	1	0.633691	0.4052044	0.2499577	0.1283439	0.2174426	0.1727993
118.964	1	0.584221	0.3996317	0.2491714	0.1297867	0.2362996	0.1743174
119.064	1	0.544644	0.3967223	0.2454427	0.1289401	0.2472636	0.1664415
119.164	1	0.593236	0.4022475	0.2478961	0.1282867	0.2428448	0.1665763
119.264	1	0.643931	0.4062684	0.2433253	0.131371	0.2274333	0.1721773
119.364	1	0.65109	0.4032468	0.244549	0.1327082	0.2220964	0.1792747
119.464	1	0.685623	0.400546	0.2508227	0.1317853	0.2305163	0.181148
119.564	1	0.702515	0.4077207	0.2481092	0.1313027	0.2441708	0.1793104
119.664	1	0.691554	0.4156726	0.2480771	0.1267903	0.2605866	0.183339
119.764	1	0.70117	0.4242547	0.2448889	0.1254803	0.2623609	0.1826625
119.864	1	0.704918	0.422299	0.2410629	0.1227973	0.2449785	0.1785761
120.049	1.5	0.684585	0.3905306	0.2376036	0.1315684	0.2105527	0.1784706
120.149	1.5	0.674964	0.3951388	0.2412924	0.1322613	0.2101627	0.17866
120.249	1.5	0.7069	0.4024985	0.2393543	0.1314886	0.2105593	0.1787394
120.349	1.5	0.657462	0.4044719	0.236196	0.1304431	0.2064056	0.1814356
120.449	1.5	0.655025	0.3998795	0.2344458	0.1328946	0.204345	0.1852945

120.549	1.5	0.710632	0.3930482	0.2391098	0.1322833	0.2054934	0.1877981
120.649	1.5	0.690498	0.3957185	0.2423747	0.1308316	0.2092966	0.1878011
120.749	1.5	0.715146	0.3966763	0.2446425	0.127853	0.2047972	0.1914807
120.849	1.5	0.728903	0.3912768	0.2477238	0.1285783	0.2012118	0.1898212
120.949	1.5	0.725914	0.3992204	0.2453759	0.129164	0.207653	0.1832804
121.049	1.5	0.748885	0.4088169	0.2362615	0.1337996	0.2107189	0.1862383
121.149	1.5	0.739236	0.4076613	0.2318808	0.128967	0.2196373	0.1847901
121.249	1.5	0.769441	0.4042206	0.2263742	0.1264291	0.2300544	0.1840042
121.349	1.5	0.765507	0.4050041	0.2312624	0.1327559	0.2340258	0.1897973
121.449	1.5	0.750245	0.4085127	0.2317541	0.1322955	0.2234868	0.1916737
121.549	1.5	0.762376	0.4030413	0.2374888	0.1342166	0.2221192	0.1926039
121.649	1.5	0.766459	0.3918658	0.2453786	0.140033	0.2084795	0.1918708
121.749	1.5	0.766539	0.396565	0.2419944	0.1463517	0.2117738	0.1893817
121.849	1.5	0.743311	0.4024927	0.2407341	0.1426642	0.2215099	0.1853957
121.949	1.5	0.734485	0.4001191	0.2396106	0.1346745	0.2289882	0.1837197
122.049	1.5	0.726093	0.4021865	0.229544	0.1281044	0.2252399	0.1860413
122.149	1.5	0.734535	0.3955469	0.2255283	0.12457	0.2227779	0.1870999
122.249	1.5	0.749706	0.3966084	0.2282528	0.1255108	0.2271615	0.1866795
122.349	1.5	0.733318	0.3954714	0.2279459	0.1276983	0.2235192	0.1864011
122.449	1.5	0.757746	0.4002157	0.2262589	0.1252253	0.2135874	0.1867241
122.549	1.5	0.694158	0.3960017	0.2247104	0.1252429	0.2191739	0.1925118
122.649	1.5	0.696504	0.3967327	0.2249848	0.1316893	0.2245765	0.1896125
122.749	1.5	0.664563	0.3975866	0.2280604	0.1333868	0.221959	0.1917477
122.849	1.5	0.672422	0.4006187	0.2296608	0.1310604	0.2230322	0.1910315
122.949	1.5	0.661618	0.3981237	0.2293757	0.1369177	0.2229098	0.1867946
123.049	1.5	0.695363	0.3968946	0.2310509	0.1357191	0.2154867	0.1829892
123.149	1.5	0.728153	0.4023539	0.2326022	0.1303724	0.2141453	0.1841027
123.249	1.5	0.72767	0.4090074	0.2312655	0.1280234	0.2184331	0.1881725
123.349	1.5	0.740436	0.4061115	0.2326203	0.1271959	0.2148534	0.1854298
123.449	1.5	0.68637	0.4095677	0.2307024	0.1314737	0.2116808	0.1882603
123.549	1.5	0.682331	0.4096435	0.2262412	0.1275523	0.2145508	0.186241
123.649	1.5	0.602041	0.4094799	0.2195984	0.1252183	0.2250737	0.1864068
123.749	1.5	0.580769	0.406608	0.217362	0.124897	0.2199102	0.1872039
123.849	1.5	0.623059	0.407271	0.2178907	0.124259	0.2147328	0.1858357
123.949	1.5	0.641787	0.4011565	0.2150432	0.1253486	0.2108898	0.1826416
124.049	1.5	0.617091	0.40398	0.216336	0.1278099	0.2112731	0.1822823
124.149	1.5	0.57735	0.4006618	0.2215873	0.1269516	0.2142225	0.1879069
124.249	1.5	0.575046	0.4002053	0.2229499	0.1284503	0.2139759	0.1845622
124.349	1.5	0.633016	0.3987614	0.2292658	0.1266487	0.2123097	0.1849886
124.449	1.5	0.627691	0.3922087	0.2260289	0.1252812	0.2151285	0.1863313
124.549	1.5	0.675956	0.4004241	0.2281042	0.1259628	0.214738	0.188127
124.649	1.5	0.692702	0.4004282	0.2302146	0.1255115	0.2166846	0.1924956
124.749	1.5	0.713166	0.3959345	0.2276063	0.1262664	0.219773	0.1947874
124.849	1.5	0.682843	0.3994722	0.2255825	0.124834	0.2159053	0.1930526
124.949	1.5	0.682553	0.3995211	0.2279269	0.1246387	0.2118963	0.1927958
125.049	1.5	0.703679	0.4181836	0.2241492	0.1261468	0.2128937	0.1948924
125.149	1.5	0.709902	0.4155369	0.2223209	0.1271859	0.2141377	0.1960764
125.249	1.5	0.686005	0.412003	0.2250545	0.1266532	0.2078635	0.1956929
125.349	1.5	0.675253	0.407729	0.2216233	0.1260511	0.2099757	0.198505
125.449	1.5	0.633893	0.4119161	0.2169385	0.1227197	0.2107947	0.1957227
125.549	1.5	0.638946	0.4095111	0.218563	0.1214243	0.2125726	0.1990324
125.649	1.5	0.665923	0.396785	0.2204818	0.1252044	0.2072194	0.1966189
125.749	1.5	0.638809	0.3942337	0.2419231	0.1296991	0.2201019	0.1951266
125.849	1.5	0.65926	0.3873622	0.2585248	0.1336696	0.2201072	0.1927198
125.949	1.5	0.698728	0.3931353	0.2734719	0.1389091	0.2177984	0.1906786
126.049	1.5	0.724706	0.4000331	0.259082	0.1336608	0.214037	0.1911895
126.149	1.5	0.733626	0.404184	0.2320686	0.1303013	0.2126662	0.1892438
126.249	1.5	0.757394	0.4013588	0.2205652	0.1208958	0.2149446	0.1917819
126.349	1.5	0.762169	0.4007858	0.2214127	0.1231672	0.2126355	0.1931332
126.449	1.5	0.72957	0.403203	0.2228733	0.1211153	0.208986	0.1907724
126.549	1.5	0.677839	0.4063278	0.219623	0.1209212	0.2105635	0.1897447
126.649	1.5	0.660908	0.3903766	0.2186259	0.1214043	0.2157017	0.1930695
126.749	1.5	0.699172	0.3891327	0.2141753	0.1235419	0.2127143	0.1921841
126.849	1.5	0.706628	0.3855756	0.2107123	0.1218489	0.209552	0.1918016
126.949	1.5	0.636558	0.3990578	0.2108756	0.1201661	0.207114	0.190002
127.049	1.5	0.639802	0.4152617	0.2125169	0.1201686	0.2060549	0.1894983
127.149	1.5	0.703713	0.4053122	0.2096124	0.1201811	0.2029379	0.1892838
127.249	1.5	0.730314	0.3899283	0.2092189	0.1210743	0.210052	0.1877141
127.349	1.5	0.719134	0.3960108	0.2110166	0.1205066	0.2090106	0.1880016
127.449	1.5	0.728536	0.3992269	0.212493	0.1210861	0.2107524	0.1820468
127.549	1.5	0.748006	0.4012595	0.2143373	0.1243452	0.2166753	0.1860518
127.649	1.5	0.746612	0.4065804	0.2116664	0.1249951	0.2094163	0.190209
127.749	1.5	0.713726	0.4027869	0.2098131	0.1244297	0.2116238	0.1895432
127.849	1.5	0.712006	0.4058241	0.2108858	0.1251117	0.2149966	0.1856949
127.949	1.5	0.71658	0.399546	0.2112657	0.1228483	0.2058834	0.1865969
128.049	1.5	0.706574	0.4000673	0.2131571	0.1226726	0.2072004	0.1863771
128.149	1.5	0.66853	0.410261	0.2129544	0.1227268	0.2108203	0.1834745
128.249	1.5	0.670723	0.4031629	0.2150647	0.1243393	0.2089583	0.1788259
128.349	1.5	0.624272	0.4075073	0.2134733	0.1208543	0.2096521	0.1712703
128.449	1.5	0.640964	0.4064182	0.2140631	0.1203885	0.2059608	0.1740434
128.549	1.5	0.636714	0.3934442	0.2114185	0.1202687	0.2031918	0.1770786
128.649	1.5	0.637188	0.3962656	0.2091381	0.121879	0.2054641	0.1671968
128.749	1.5	0.609603	0.4012076	0.2126136	0.1230539	0.213926	0.1709385
128.849	1.5	0.636872	0.4004118	0.2151031	0.1228974	0.2049064	0.1834256
128.949	1.5	0.655726	0.3932601	0.2149943	0.1223397	0.2034452	0.1785094
129.049	1.5	0.68426	0.3951776	0.2149485	0.1246766	0.2029863	0.1773123
129.149	1.5	0.701479	0.4096425	0.2131388	0.1254871	0.203617	0.1699836
129.249	1.5	0.721281	0.3878817	0.2111349	0.1252302	0.2017625	0.1681323
129.349	1.5	0.70644	0.4004725	0.2129794	0.127491	0.2019068	0.1677475
129.449	1.5	0.682835	0.4137077	0.213595	0.1262977	0.2021828	0.1714748
129.549	1.5	0.670089	0.4036275	0.21604	0.1255459	0.202039	0.1753723
129.649	1.5	0.657747	0.4053128	0.2156189	0.1239005	0.201133	0.1713289
129.749	1.5	0.656815	0.3951313	0.2127252	0.1237901	0.2015188	0.1717113
129.849	1.5	0.639924	0.4046226	0.2114147	0.1222966	0.1988699	0.1671278
129.949	1.5	0.593647	0.3907001	0.2106205	0.1242486	0.2026615	0.1693487

130.049	1.5	0.617709	0.3904207	0.2104102	0.1244062	0.2038067	0.1701258
130.149	1.5	0.631605	0.3849741	0.206117	0.1236171	0.1979689	0.1735891
130.249	1.5	0.650585	0.3972321	0.2046574	0.1234259	0.1970413	0.1832993
130.349	1.5	0.649835	0.3985169	0.2056272	0.1232726	0.1995233	0.1812417
130.449	1.5	0.658284	0.407329	0.2080966	0.1249295	0.2036465	0.1755108
130.549	1.5	0.655408	0.3946946	0.207546	0.126502	0.2043765	0.1708448
130.649	1.5	0.650211	0.3839324	0.2075309	0.124771	0.1972438	0.169792
130.749	1.5	0.638693	0.3861781	0.205403	0.1291452	0.1933773	0.1704369
130.849	1.5	0.645152	0.3859088	0.2014083	0.1275628	0.2049818	0.1762196
130.949	1.5	0.634274	0.3840204	0.2049618	0.1243968	0.1996899	0.1817987
131.049	1.5	0.650985	0.3966972	0.2137708	0.1253039	0.1988472	0.1861528
131.149	1.5	0.656849	0.3854553	0.2224422	0.1265177	0.198788	0.1867348
131.249	1.5	0.680095	0.385642	0.2205498	0.1266627	0.1997783	0.183928
131.349	1.5	0.694323	0.395182	0.2091333	0.1252574	0.200946	0.1799293
131.449	1.5	0.691944	0.3990007	0.202915	0.1295328	0.2016638	0.1734998
131.549	1.5	0.679337	0.3982117	0.2062777	0.1311408	0.2005354	0.1737292
131.649	1.5	0.713539	0.3885344	0.2092208	0.1316615	0.1987948	0.1803741
131.749	1.5	0.714617	0.3848844	0.2049872	0.1314944	0.1917112	0.1785437
131.849	1.5	0.696329	0.3875753	0.2066195	0.1307094	0.2046216	0.1772396
131.949	1.5	0.691523	0.3884574	0.2019444	0.1313258	0.2003791	0.1844998
132.049	1.5	0.66828	0.3841766	0.2011957	0.1333096	0.1968201	0.1893424
132.149	1.5	0.643081	0.3904747	0.2014869	0.131142	0.1964094	0.1857585
132.249	1.5	0.622818	0.3998478	0.198121	0.1295433	0.1945146	0.1772023
132.349	1.5	0.667622	0.3830277	0.2013776	0.1305359	0.196652	0.1724042
132.449	1.5	0.689448	0.3777441	0.2062296	0.1309325	0.1946779	0.1757975
132.549	1.5	0.679636	0.4018956	0.2021475	0.1319115	0.1943366	0.1774742
132.649	1.5	0.670841	0.39075	0.20062	0.1342919	0.1936794	0.1742262
132.749	1.5	0.673049	0.3887812	0.2014949	0.132342	0.1991528	0.1765621
132.849	1.5	0.67839	0.3887943	0.2003537	0.1341378	0.2020577	0.179392
132.949	1.5	0.667326	0.3832768	0.1997327	0.1332189	0.1961215	0.1839135
133.049	1.5	0.685307	0.3965103	0.203584	0.1355853	0.194679	0.1780016
133.149	1.5	0.677743	0.4045388	0.2081718	0.1373255	0.1949804	0.171001
133.249	1.5	0.662202	0.4017906	0.2071147	0.1378211	0.1975133	0.1718979
133.349	1.5	0.694427	0.3858845	0.2084864	0.1344789	0.2034327	0.1743574
133.449	1.5	0.691833	0.3811008	0.2107725	0.1322106	0.199276	0.1720131
133.549	1.5	0.679288	0.379146	0.2087939	0.1333151	0.1955818	0.1751626
133.649	1.5	0.695132	0.3858755	0.2077631	0.1336977	0.1966885	0.1773157
133.749	1.5	0.645529	0.3867509	0.2111607	0.1359713	0.1905262	0.169534
133.849	1.5	0.686414	0.3754431	0.2130808	0.1369351	0.1922267	0.1706311
133.949	1.5	0.723022	0.3867806	0.2142229	0.1356433	0.1895951	0.1721247
134.049	1.5	0.704697	0.3874194	0.2191242	0.1331665	0.1928366	0.1783431
134.149	1.5	0.708994	0.3752944	0.2165958	0.1341288	0.2059143	0.1752492
134.249	1.5	0.634431	0.3889086	0.2161337	0.1354104	0.2044587	0.1774845
134.349	1.5	0.668596	0.3938255	0.211571	0.1396417	0.1940241	0.1739971
134.449	1.5	0.675776	0.3796369	0.2094787	0.1428957	0.199185	0.1697273
134.549	1.5	0.669081	0.3807812	0.2079805	0.1411705	0.1974011	0.1777611
134.649	1.5	0.662269	0.368846	0.2112807	0.1405832	0.1991888	0.1794565
134.749	1.5	0.656322	0.3677361	0.2063802	0.1401672	0.1970643	0.1781465
134.849	1.5	0.629328	0.3727407	0.2117815	0.141136	0.1962519	0.1776667
134.949	1.5	0.579627	0.3770649	0.2120359	0.1415675	0.1981555	0.1877985
135.049	1.5	0.554987	0.3794948	0.2124576	0.1408658	0.2004522	0.1882203
135.149	1.5	0.600086	0.3746974	0.2150071	0.1410492	0.2025377	0.1855131
135.249	1.5	0.621005	0.3806566	0.214811	0.1401727	0.2007839	0.1867627
135.349	1.5	0.623864	0.3820259	0.2135314	0.13733	0.1992497	0.1853332
135.449	1.5	0.615058	0.3829238	0.2111238	0.1399283	0.1984743	0.1794142
135.549	1.5	0.579318	0.3903963	0.2143767	0.1420653	0.2004492	0.1784863
135.649	1.5	0.557328	0.3819571	0.2137126	0.1410985	0.2070385	0.1796359
135.749	1.5	0.59381	0.3939802	0.2189835	0.1409765	0.2071692	0.1824489
135.849	1.5	0.627269	0.3922351	0.2165616	0.1415079	0.1979323	0.1809719
135.949	1.5	0.636525	0.3831271	0.2191753	0.1404366	0.1990666	0.1832719
136.049	1.5	0.642275	0.3819631	0.2186406	0.1420099	0.2020577	0.1794859
136.149	1.5	0.661761	0.3603021	0.2196876	0.1452194	0.2090704	0.1809868
136.249	1.5	0.691967	0.354196	0.2170966	0.1443645	0.214393	0.1874909
136.349	1.5	0.689709	0.3831017	0.21528	0.1395853	0.2087018	0.1933922
136.449	1.5	0.690327	0.3778979	0.2170005	0.1408129	0.2030687	0.1922612
136.549	1.5	0.734476	0.3837369	0.2170253	0.1433771	0.2049448	0.1916272
136.649	1.5	0.744634	0.3804089	0.2185879	0.1431926	0.204367	0.1952363
136.749	1.5	0.745976	0.3705894	0.2200254	0.1502387	0.2008617	0.1959106
136.849	1.5	0.71222	0.3696397	0.218799	0.1584978	0.1955144	0.1961211
136.949	1.5	0.689344	0.3778887	0.2206051	0.1595461	0.1926347	0.1907087
137.049	1.5	0.685862	0.3811832	0.2163966	0.1676212	0.1905838	0.1918387
137.149	1.5	0.65133	0.3743586	0.2209032	0.171092	0.1918626	0.1966878
137.249	1.5	0.578844	0.3702749	0.2225728	0.1577003	0.1902149	0.2011118
137.349	1.5	0.442883	0.3655456	0.2183447	0.1602113	0.1951807	0.1911573
137.449	1.5	0.364939	0.3713631	0.2146166	0.160895	0.192724	0.1883913
137.549	1.5	0.374116	0.3474278	0.2146633	0.1496817	0.202023	0.1877515
137.649	1.5	0.396706	0.3582218	0.2188635	0.1410028	0.2138348	0.187538
137.749	1.5	0.42065	0.3717196	0.2174897	0.1360086	0.2217982	0.1864372
137.849	1.5	0.424194	0.3712537	0.2160745	0.1366817	0.2102826	0.1861157
137.949	1.5	0.432935	0.373149	0.215487	0.137304	0.2006525	0.1849273
138.049	1.5	0.411672	0.3758147	0.2168183	0.1478626	0.2075172	0.1877646
138.149	1.5	0.386933	0.380414	0.2207826	0.1677918	0.2042862	0.1903269
138.249	1.5	0.371004	0.3845537	0.2221503	0.1599307	0.1979096	0.1918693
138.349	1.5	0.382655	0.3685067	0.2204458	0.151866	0.2029104	0.187617
138.449	1.5	0.423577	0.3746043	0.2175706	0.1424008	0.1973977	0.1802314
138.549	1.5	0.471826	0.3847058	0.2174448	0.1541458	0.1999122	0.181665
138.649	1.5	0.524672	0.3872466	0.2207747	0.1678402	0.2020186	0.1866961
138.749	1.5	0.583111	0.382134	0.2212038	0.172708	0.1993654	0.194125
138.849	1.5	0.638305	0.3784486	0.2226762	0.1546838	0.2050668	0.1978284
138.949	1.5	0.670296	0.3817286	0.2211478	0.1554847	0.2025304	0.1956067
139.049	1.5	0.702484	0.3845267	0.2209728	0.1586532	0.2034623	0.2013568
139.149	1.5	0.709869	0.3616147	0.2175145	0.1594647	0.2021418	0.1958669
139.249	1.5	0.721336	0.3654163	0.2189241	0.1476632	0.1946141	0.1953961
139.349	1.5	0.72782	0.3670301	0.217043	0.142437	0.1911548	0.1970047
139.449	1.5	0.719576	0.3712656	0.2195509	0.1465357	0.193955	0.1952835

139.549	1.5	0.695566	0.3701488	0.2179992	0.1447781	0.1968022	0.1988884
139.649	1.5	0.506188	0.3746454	0.2185113	0.1344113	0.1948458	0.1982885
139.749	1.5	0.447661	0.3709285	0.2167064	0.1357065	0.1958178	0.191287
139.849	1.5	0.493498	0.387194	0.2194264	0.1353193	0.1947339	0.1899303
139.949	1.5	0.525097	0.3953555	0.2157857	0.1477594	0.1932986	0.190016
140.049	1.5	0.56246	0.3930251	0.2036252	0.1527438	0.1944395	0.1875032
140.149	1.5	0.60854	0.376309	0.1958007	0.1628394	0.191646	0.1708159
140.249	1.5	0.635172	0.3716754	0.1963298	0.1597641	0.1768336	0.1756007
140.349	1.5	0.674741	0.3706741	0.1972521	0.1614226	0.1851377	0.193092
140.449	1.5	0.679991	0.3795029	0.1907742	0.1382275	0.1886449	0.2118338
140.549	1.5	0.685368	0.3898859	0.1893473	0.127574	0.1851978	0.2096767
140.649	1.5	0.683855	0.3894821	0.1921212	0.1173675	0.1847448	0.2054776
140.749	1.5	0.668922	0.3873863	0.1957612	0.1173372	0.1851426	0.2066364
140.849	1.5	0.687312	0.3769411	0.1910502	0.1154384	0.1870188	0.2038195
140.949	1.5	0.703749	0.364159	0.1973612	0.1133386	0.1820054	0.1974487
141.049	1.5	0.684235	0.3705112	0.1991855	0.1177042	0.1825377	0.1981664
141.149	1.5	0.689263	0.3737903	0.2060781	0.0977257	0.1822717	0.1761436
141.249	1.5	0.706019	0.3777953	0.2036739	0.0965514	0.1806748	0.1758379
141.349	1.5	0.716799	0.3871039	0.2049438	0.0961953	0.1813215	0.1733963
141.449	1.5	0.730172	0.3850233	0.1986322	0.105414	0.1795469	0.1787704
141.549	1.5	0.737727	0.3823142	0.1927712	0.1085037	0.1823069	0.1790663
141.649	1.5	0.726724	0.3759498	0.1918671	0.1071356	0.1791413	0.181706
141.749	1.5	0.641796	0.3787109	0.1871152	0.1026052	0.180381	0.184608
141.849	1.5	0.639744	0.3714763	0.18477	0.1009864	0.1789621	0.184625
141.949	1.5	0.622191	0.36498	0.1811209	0.1065655	0.1793124	0.1886766
142.049	1.5	0.653989	0.366088	0.1839498	0.1079015	0.1866157	0.1857067
142.149	1.5	0.682982	0.3654891	0.1852473	0.1084866	0.1862372	0.1785523
142.249	1.5	0.632155	0.3717939	0.1850401	0.1160508	0.1829554	0.1787206
142.349	1.5	0.619302	0.3810194	0.1830431	0.1118935	0.183241	0.1712618
142.449	1.5	0.626261	0.3782169	0.1821957	0.1138649	0.1842491	0.161559
142.549	1.5	0.604986	0.3755887	0.1803591	0.1184743	0.1850627	0.1624143
142.649	1.5	0.609714	0.3684381	0.1807637	0.1187859	0.1880165	0.1714236
142.749	1.5	0.631887	0.3726188	0.1847576	0.117885	0.1863308	0.1709413
142.849	1.5	0.600053	0.3795891	0.1853288	0.1222794	0.1851533	0.1746068
142.949	1.5	0.61975	0.3881524	0.1855988	0.1222831	0.1855965	0.1752526
143.049	1.5	0.660896	0.3935813	0.1853271	0.1232363	0.1892779	0.1733849
143.149	1.5	0.661477	0.3966624	0.1865029	0.1247472	0.193133	0.1743441
143.249	1.5	0.663736	0.3898699	0.1859595	0.1253284	0.1912105	0.1744541
143.349	1.5	0.650266	0.3824967	0.1869057	0.1229629	0.1902573	0.1762681
143.449	1.5	0.590395	0.3782727	0.1861787	0.1261044	0.1890226	0.1779184
143.549	1.5	0.594145	0.3723944	0.1872276	0.1256414	0.1890471	0.1757903
143.649	1.5	0.554881	0.3750922	0.1866801	0.1271138	0.1905347	0.1730303
143.749	1.5	0.543737	0.3809081	0.1883616	0.1295648	0.1898982	0.1697241
143.849	1.5	0.591389	0.3800462	0.1868074	0.1257774	0.1876743	0.1690206
143.949	1.5	0.63716	0.3841203	0.1868223	0.1273209	0.1909789	0.168138
144.049	1.5	0.628655	0.3718384	0.1873882	0.1264016	0.1902577	0.1644038
144.149	1.5	0.663691	0.3676051	0.1881556	0.1274713	0.1899682	0.1592347
144.249	1.5	0.679845	0.3687533	0.1861169	0.1275644	0.1913933	0.1621838
144.349	1.5	0.604502	0.3786925	0.187009	0.1275894	0.1908188	0.1595934
144.449	1.5	0.564453	0.3757517	0.1885741	0.1290017	0.1912501	0.1620625
144.549	1.5	0.55564	0.3650547	0.1918907	0.1299891	0.1890002	0.1666421
144.649	1.5	0.540987	0.3720554	0.1921257	0.1274267	0.1867544	0.1602011
144.749	1.5	0.506791	0.3722724	0.1909686	0.1245962	0.1928165	0.1643175
144.849	1.5	0.497794	0.372751	0.1917669	0.126874	0.1915687	0.1643495
144.949	1.5	0.451065	0.3678156	0.1913221	0.1266493	0.1875947	0.1635534
145.049	1.5	0.445968	0.3720261	0.1912861	0.1269183	0.1901007	0.1629208
145.149	1.5	0.436444	0.3714869	0.1911685	0.1271744	0.1909035	0.1678693
145.249	1.5	0.474134	0.367845	0.1916974	0.1307093	0.1910473	0.1722322
145.349	1.5	0.459837	0.3732015	0.1913162	0.1348441	0.1911113	0.1682332
145.449	1.5	0.478294	0.3774665	0.1927162	0.1354287	0.1874791	0.1655095
145.549	1.5	0.52877	0.3874385	0.1917689	0.137391	0.189287	0.1663878
145.649	1.5	0.579539	0.3883693	0.1871523	0.1403755	0.1912195	0.1692575
145.749	1.5	0.612167	0.3930016	0.1881314	0.1380537	0.1889098	0.1656061
145.849	1.5	0.614286	0.3951221	0.1874208	0.1352726	0.1927003	0.1632873
145.949	1.5	0.623037	0.3955722	0.1895792	0.1363114	0.1909968	0.1601829
146.049	1.5	0.602699	0.3874909	0.1916976	0.1361852	0.1896584	0.1649214
146.149	1.5	0.5327	0.3867968	0.1907191	0.1347801	0.188797	0.1618924
146.249	1.5	0.485049	0.3869303	0.1898922	0.1313394	0.1914428	0.1670999
146.349	1.5	0.471772	0.403937	0.192074	0.1298134	0.1914048	0.1674915
146.449	1.5	0.445225	0.4005445	0.1907306	0.1293377	0.1858898	0.165133
146.549	1.5	0.444242	0.3996585	0.1886886	0.1296314	0.1817475	0.1609379
146.649	1.5	0.411981	0.3924277	0.1919209	0.1299197	0.186498	0.1647642
146.749	1.5	0.365333	0.3905539	0.1937591	0.1283945	0.1841678	0.166368
146.849	1.5	0.359183	0.3971651	0.192876	0.1292137	0.1854205	0.1620331
146.949	1.5	0.382835	0.3911579	0.1918932	0.1291799	0.1817486	0.1658097
147.049	1.5	0.415755	0.3966067	0.1895989	0.1319596	0.1849011	0.160603
147.149	1.5	0.43109	0.40163	0.1911255	0.1317008	0.1837561	0.1601584
147.249	1.5	0.43045	0.3905127	0.1939082	0.1288888	0.1866907	0.1631956
147.349	1.5	0.428495	0.3910953	0.1933364	0.1277553	0.1925568	0.1640279
147.449	1.5	0.454541	0.3958228	0.1924513	0.127782	0.1913643	0.1615342
147.549	1.5	0.473079	0.3985601	0.1943737	0.131359	0.1886339	0.1564038
147.649	1.5	0.50844	0.3984925	0.1931147	0.1321283	0.1890069	0.153638
147.749	1.5	0.493504	0.393208	0.1974312	0.1297799	0.1882441	0.1546777
147.849	1.5	0.497115	0.3937092	0.1974955	0.1286795	0.1873051	0.1535713
147.949	1.5	0.500368	0.388243	0.1965764	0.1291378	0.186767	0.1571318
148.049	1.5	0.487635	0.3876098	0.1961927	0.1323176	0.1846279	0.1570952
148.149	1.5	0.499512	0.3857192	0.1959984	0.1308118	0.1834708	0.1576104
148.249	1.5	0.482101	0.3950945	0.1964692	0.1324228	0.1837192	0.1561158
148.349	1.5	0.455918	0.386296	0.1959601	0.1302728	0.1868786	0.1556017
148.449	1.5	0.467058	0.3821541	0.200195	0.1297308	0.1867639	0.1583779
148.549	1.5	0.451128	0.383346	0.198921	0.1283483	0.1846564	0.1542714
148.649	1.5	0.453672	0.387621	0.1966828	0.1285685	0.1881188	0.1572026
148.749	1.5	0.447497	0.3855757	0.1989021	0.1283438	0.1919373	0.156714
148.849	1.5	0.450677	0.3861309	0.1993688	0.1260935	0.1928911	0.1601792
148.949	1.5	0.49516	0.3789604	0.1989423	0.1218827	0.2001864	0.1591154

149.049	1.5	0.522573	0.3918035	0.2002547	0.1254708	0.2046686	0.1601669
149.149	1.5	0.530224	0.3820499	0.19738	0.1300877	0.1989838	0.1619325
149.249	1.5	0.546051	0.3804258	0.2023274	0.1336487	0.1977481	0.1611955
149.349	1.5	0.542099	0.3894639	0.201203	0.1384352	0.1971624	0.1568072
149.449	1.5	0.53156	0.3860295	0.1976912	0.1302037	0.1965821	0.1592379
149.549	1.5	0.581201	0.3834441	0.197177	0.1271112	0.1923302	0.1581945
149.649	1.5	0.60322	0.3840031	0.1951561	0.1210828	0.1944952	0.1590625
149.749	1.5	0.590067	0.3928649	0.1990743	0.121209	0.1961138	0.1590651
149.849	1.5	0.586	0.3933559	0.1983643	0.1203069	0.1932977	0.1568395
149.949	1.5	0.594103	0.3809766	0.1970164	0.1151926	0.1927688	0.1552802
150.049	1.5	0.648426	0.385095	0.1960102	0.1185406	0.1927058	0.1597665
150.149	1.5	0.668558	0.3784197	0.1951464	0.12275	0.1932727	0.1595728
150.249	1.5	0.641041	0.3910246	0.1978516	0.1240772	0.1883398	0.1556374
150.349	1.5	0.660843	0.382496	0.1952147	0.1225587	0.1931799	0.1600796
150.449	1.5	0.681685	0.3826638	0.1946512	0.1217498	0.1984164	0.1603819
150.549	1.5	0.692678	0.3766303	0.1935325	0.1244362	0.1966232	0.160751
150.649	1.5	0.700246	0.3829752	0.1945042	0.1257586	0.1925668	0.159276
150.749	1.5	0.723338	0.3724147	0.1962603	0.1226823	0.186674	0.1594841
150.849	1.5	0.736686	0.3811656	0.201317	0.1237847	0.1882638	0.1574745
150.949	1.5	0.759841	0.3712871	0.1986935	0.128759	0.1889393	0.1501945
151.049	1.5	0.775731	0.3697705	0.1984062	0.1525999	0.1874737	0.1918902
151.149	1.5	0.770704	0.3687455	0.1994285	0.1507167	0.1909548	0.1927477
151.249	1.5	0.717789	0.3705975	0.1996158	0.1507123	0.1926615	0.1945861
151.349	1.5	0.713079	0.3844436	0.2014714	0.150024	0.1928754	0.1949517
151.449	1.5	0.730884	0.3801806	0.2007017	0.1459792	0.1919384	0.1914566
151.549	1.5	0.748245	0.3840133	0.20298	0.1472654	0.1935685	0.1897224
151.649	1.5	0.741351	0.3830033	0.198921	0.1467621	0.1953718	0.1900399
151.749	1.5	0.741844	0.3818734	0.1995334	0.1439818	0.1953354	0.1909854
151.849	1.5	0.765309	0.401573	0.1997284	0.1431058	0.1942305	0.1924024
151.949	1.5	0.760459	0.3931012	0.199163	0.1453158	0.1889825	0.1922949
152.049	1.5	0.725322	0.3952768	0.2002981	0.1449465	0.1880593	0.189089
152.149	1.5	0.676177	0.3926477	0.1982095	0.1420435	0.1883314	0.189537
152.249	1.5	0.668389	0.3935966	0.1972426	0.1466407	0.1901134	0.1902533
152.349	1.5	0.666338	0.3932987	0.1967961	0.1494714	0.1860129	0.1898272
152.449	1.5	0.62617	0.399244	0.1969364	0.1414922	0.1853097	0.1887192
152.549	1.5	0.662006	0.3920926	0.1974018	0.1376614	0.1826693	0.1893302
152.649	1.5	0.710415	0.3856388	0.197148	0.1443418	0.190144	0.1903079
152.749	1.5	0.654553	0.3783698	0.1996348	0.1438557	0.1885234	0.1885342
152.849	1.5	0.614911	0.3834642	0.1987633	0.1430679	0.1907309	0.1894192
152.949	1.5	0.664537	0.378238	0.1962404	0.1434449	0.1884465	0.192372
153.049	1.5	0.638087	0.3863684	0.1983055	0.1426332	0.1891576	0.1950817
153.149	1.5	0.639924	0.3967209	0.2010756	0.1424944	0.1917419	0.1964798
153.249	1.5	0.667954	0.393878	0.2025518	0.1409369	0.1873379	0.1966643
153.349	1.5	0.688712	0.3995945	0.199013	0.1428394	0.1855287	0.1949141
153.449	1.5	0.702049	0.3938125	0.1972001	0.1395882	0.1889244	0.1939328
153.549	1.5	0.66662	0.3895365	0.1983422	0.1397293	0.187535	0.1942434
153.649	1.5	0.687943	0.3956124	0.1991363	0.1418015	0.186356	0.1943105
153.749	1.5	0.686837	0.3887056	0.200408	0.1389654	0.1855568	0.1924016
153.849	1.5	0.716147	0.391429	0.2027201	0.1402527	0.1863429	0.1899852
153.949	1.5	0.746743	0.3873071	0.200343	0.1393505	0.1825105	0.1885661
154.049	1.5	0.764014	0.3744946	0.1997713	0.1403494	0.1844223	0.1879749
154.149	1.5	0.725635	0.3681253	0.1986189	0.1436165	0.186769	0.1887197
154.249	1.5	0.766824	0.3852945	0.1979866	0.1444174	0.1886665	0.1914562
154.349	1.5	0.745661	0.3810508	0.2029749	0.1449377	0.1885357	0.1909486
154.449	1.5	0.764968	0.3846606	0.2067567	0.144115	0.1842446	0.1937364
154.549	1.5	0.734787	0.3576593	0.2000154	0.1465275	0.1807779	0.1929979
154.649	1.5	0.7243	0.3526	0.2023546	0.1501092	0.1812498	0.1926136
154.749	1.5	0.749674	0.3615096	0.1996703	0.1467212	0.178032	0.194365
154.849	1.5	0.750072	0.3728375	0.200116	0.1469015	0.1786352	0.1930978
154.949	1.5	0.654779	0.369577	0.1982459	0.1515817	0.1773511	0.194039
155.049	1.5	0.655814	0.3787601	0.2024034	0.154755	0.1779906	0.19432
155.149	1.5	0.688366	0.3762693	0.2040425	0.1534637	0.1765802	0.1944008
155.249	1.5	0.673402	0.3830554	0.1996021	0.1513975	0.1786922	0.1938201
155.349	1.5	0.678663	0.3890212	0.2006144	0.1527915	0.1802702	0.1921908
155.449	1.5	0.714297	0.3829845	0.2036715	0.1538507	0.1819117	0.1898368
155.549	1.5	0.736072	0.3961563	0.20555	0.1555271	0.1813784	0.1893386
155.649	1.5	0.745254	0.3818606	0.2037843	0.1541029	0.1878922	0.1907094
155.749	1.5	0.756962	0.3813906	0.2060234	0.1487877	0.184748	0.19149
155.849	1.5	0.761298	0.381876	0.206114	0.1512698	0.1813151	0.1948348
155.949	1.5	0.757108	0.3786526	0.2038546	0.1480071	0.1834642	0.1993287
156.049	1.5	0.705233	0.3763607	0.2058341	0.1505544	0.1837429	0.2032564
156.149	1.5	0.731611	0.3779363	0.2064106	0.1519922	0.1877856	0.2002326
156.249	1.5	0.749188	0.3755168	0.2074299	0.1525363	0.1830018	0.1968181
156.349	1.5	0.737841	0.375521	0.2065577	0.1518692	0.1778246	0.1969737
156.449	1.5	0.728973	0.3730895	0.2039986	0.1511271	0.176341	0.1999713
156.549	1.5	0.706355	0.3819883	0.2060301	0.152528	0.1709272	0.1998588
156.649	1.5	0.620281	0.3782936	0.2073582	0.1534023	0.1773042	0.202039
156.749	1.5	0.653661	0.3697498	0.2084902	0.1544422	0.1800493	0.2003746
156.849	1.5	0.675172	0.3733939	0.2115604	0.1579343	0.1776395	0.1945219
156.949	1.5	0.703376	0.3716997	0.2101786	0.1564153	0.1774127	0.1886592
157.049	1.5	0.70492	0.3831016	0.2065819	0.1567648	0.1792349	0.1845753
157.149	1.5	0.694615	0.3902754	0.2047049	0.1537555	0.1822718	0.1855576
157.249	1.5	0.706571	0.3772415	0.2067759	0.1495804	0.18267	0.1885548
157.349	1.5	0.670313	0.3702297	0.2060385	0.1481446	0.1820049	0.1895633
157.449	1.5	0.598255	0.3602117	0.2094229	0.1526054	0.1832055	0.1882297
157.549	1.5	0.612672	0.3682076	0.2099469	0.1557135	0.1811988	0.1896219
157.649	1.5	0.61793	0.3818484	0.2090498	0.1571354	0.1795695	0.1893908
157.749	1.5	0.622287	0.3835614	0.2060376	0.1585212	0.1774472	0.1907737
157.849	1.5	0.648337	0.3852605	0.2109087	0.1559131	0.1758274	0.1928681
157.949	1.5	0.551776	0.3852142	0.2109954	0.1561754	0.1778911	0.1907798
158.049	1.5	0.44438	0.3832659	0.207907	0.1556035	0.1778176	0.1903782
158.149	1.5	0.460203	0.3899549	0.2067396	0.1579074	0.1776071	0.1929601
158.249	1.5	0.49657	0.38082	0.2043135	0.1563799	0.1757301	0.1927135
158.349	1.5	0.528235	0.3834993	0.2048853	0.1525218	0.1781699	0.1915606
158.449	1.5	0.536811	0.384365	0.2039162	0.14563	0.1801745	0.1908662

158.549	1.5	0.557987	0.3900141	0.2002452	0.1475451	0.178786	0.193902
158.649	1.5	0.549733	0.3948179	0.2046981	0.1495263	0.1753518	0.1941981
158.749	1.5	0.555893	0.39607	0.2066191	0.1532253	0.1757881	0.1936958
158.849	1.5	0.611194	0.3845624	0.2032419	0.1552256	0.1735669	0.1925998
158.949	1.5	0.652215	0.3896874	0.2013333	0.15319	0.1719468	0.1915386
159.049	1.5	0.625391	0.3939322	0.1982883	0.1543051	0.1723458	0.1875545
159.149	1.5	0.556107	0.3785708	0.194621	0.155444	0.1724555	0.1895135
159.249	1.5	0.611085	0.3861244	0.1966508	0.1594415	0.1709064	0.1884518
159.349	1.5	0.607066	0.3874584	0.1976156	0.1605282	0.1723572	0.1913272
159.449	1.5	0.642147	0.3837788	0.1974709	0.1557792	0.1771406	0.1940851
159.549	1.5	0.654193	0.395651	0.2019382	0.1500104	0.1744085	0.197871
159.649	1.5	0.655866	0.4041999	0.203227	0.1511769	0.1700352	0.1971056
159.749	1.5	0.661729	0.4060329	0.1953594	0.1500969	0.1688861	0.1903949
159.849	1.5	0.660678	0.4008082	0.1960071	0.1518144	0.1707686	0.1849748
159.949	1.5	0.705235	0.384147	0.1968429	0.1528337	0.1733142	0.1830098
160.049	1.5	0.715993	0.3855049	0.1958641	0.1516635	0.1720252	0.1793898
160.149	1.5	0.722809	0.3938218	0.192877	0.1545598	0.1767307	0.1683034
160.249	1.5	0.713107	0.4005787	0.1940753	0.1544369	0.1744473	0.1636064
160.349	1.5	0.718273	0.3931634	0.196584	0.1541044	0.1756235	0.1489695
160.449	1.5	0.697872	0.3940686	0.1965621	0.152337	0.1739081	0.1497162
160.549	1.5	0.684795	0.3879984	0.1964979	0.1505345	0.171644	0.1748317
160.649	1.5	0.709326	0.3946117	0.1941085	0.1492996	0.1711884	0.1687814
160.749	1.5	0.72456	0.3906099	0.1936921	0.1512075	0.1724342	0.1594552
160.849	1.5	0.724549	0.3877779	0.1964351	0.1500147	0.1712596	0.1591636
160.949	1.5	0.698383	0.3897302	0.1973519	0.1531308	0.1697687	0.1551573
161.049	1.5	0.701718	0.3955065	0.1976931	0.1572949	0.1708502	0.142889
161.149	1.5	0.708927	0.3945349	0.1965275	0.1537311	0.1732315	0.1468921
161.249	1.5	0.723217	0.3919711	0.1953471	0.1508557	0.1749956	0.156422
161.349	1.5	0.721809	0.3927906	0.1960942	0.153776	0.1746226	0.1500488
161.449	1.5	0.733907	0.4054327	0.1959842	0.1563379	0.1702203	0.1774284
161.549	1.5	0.718052	0.4023673	0.1964351	0.1575875	0.1701522	0.1493419
161.649	1.5	0.704983	0.389186	0.1979738	0.1603309	0.1719166	0.14735
161.749	1.5	0.65099	0.3898072	0.1958072	0.1585025	0.1719807	0.1549631
161.849	1.5	0.662236	0.3977382	0.1942871	0.1558853	0.1736394	0.1475496
161.949	1.5	0.674824	0.3915151	0.1904418	0.1579677	0.1709899	0.1547857
162.049	1.5	0.673609	0.3891397	0.1923795	0.1580118	0.1704183	0.1633296
162.149	1.5	0.688265	0.3916698	0.1949484	0.1565195	0.1711799	0.1498419
162.249	1.5	0.701039	0.3907921	0.1947235	0.157833	0.1738361	0.1556101
162.349	1.5	0.689405	0.3907831	0.1937428	0.1567603	0.1745308	0.149624
162.449	1.5	0.69836	0.386589	0.1937478	0.1574714	0.1731024	0.1493472
162.549	1.5	0.716555	0.3898964	0.194306	0.159254	0.1709876	0.1574399
162.649	1.5	0.725125	0.3894741	0.1947822	0.1590838	0.1719018	0.152809
162.749	1.5	0.714486	0.3881118	0.1967485	0.1566219	0.1720055	0.1541177
162.849	1.5	0.718608	0.390945	0.1952861	0.15872	0.1743825	0.14904
162.949	1.5	0.701335	0.3892409	0.1955001	0.1603116	0.168576	0.1526862
163.049	1.5	0.715518	0.3882343	0.1970511	0.160549	0.1704867	0.1557006
163.149	1.5	0.706914	0.3938203	0.1961783	0.1591989	0.1710004	0.1534077
163.249	1.5	0.704694	0.4015259	0.1966832	0.1557582	0.1693525	0.1706557
163.349	1.5	0.708439	0.3981614	0.197478	0.1540071	0.1679073	0.1515341
163.449	1.5	0.719909	0.3986436	0.1955341	0.158917	0.1722012	0.1482038
163.549	1.5	0.713332	0.4025262	0.1866737	0.1587777	0.1699654	0.1521132
163.649	1.5	0.662343	0.4002312	0.1824546	0.1594166	0.170517	0.1548383
163.749	1.5	0.638101	0.3934833	0.1886521	0.1587733	0.1695089	0.1593277
163.849	1.5	0.648345	0.3920218	0.1890479	0.1599747	0.1679138	0.1589051
163.949	1.5	0.670904	0.3913926	0.1882077	0.1559815	0.1696083	0.1499214
164.049	1.5	0.680927	0.3949653	0.1889485	0.1549874	0.1704968	0.1647933
164.149	1.5	0.690724	0.3927136	0.1887333	0.1595155	0.1728944	0.1707384
164.249	1.5	0.68476	0.3850335	0.1903877	0.1636996	0.1723279	0.1733415
164.349	1.5	0.67755	0.3908802	0.1901885	0.1658673	0.1724025	0.166797
164.449	1.5	0.692834	0.3903873	0.191227	0.1703407	0.1714447	0.1555952
164.549	1.5	0.713427	0.3910788	0.1935379	0.1661963	0.1707501	0.1446374
164.649	1.5	0.74851	0.3940622	0.1931234	0.1619814	0.1707936	0.1523775
164.749	1.5	0.754916	0.3872659	0.1942928	0.1629214	0.1711562	0.1456845
164.849	1.5	0.763309	0.3873022	0.1941139	0.1616329	0.1716508	0.1493489
164.949	1.5	0.718688	0.3936329	0.1922056	0.1603026	0.1724887	0.1479062
165.049	1.5	0.753126	0.4049717	0.190479	0.1639317	0.1720534	0.1542971
165.149	1.5	0.775338	0.4071889	0.190686	0.1653266	0.173176	0.1516242
165.249	1.5	0.756686	0.397756	0.1885349	0.1692705	0.1720381	0.1580264
165.349	1.5	0.753548	0.3958233	0.1838891	0.166904	0.1722646	0.1500424
165.449	1.5	0.749611	0.3994205	0.182674	0.1671021	0.1732213	0.150892
165.549	1.5	0.72244	0.3979288	0.1776213	0.1727785	0.1740396	0.1649435
165.649	1.5	0.732961	0.3910766	0.1694131	0.176683	0.1744667	0.1530749
165.749	1.5	0.729967	0.390887	0.1683006	0.175822	0.1728314	0.1459443
165.849	1.5	0.744159	0.3813854	0.1708038	0.1761261	0.1716084	0.1458201
165.949	1.5	0.77608	0.3873362	0.173183	0.1735633	0.1659717	0.1477048
166.049	1.5	0.793077	0.3879702	0.1735342	0.1723796	0.1645818	0.1418156
166.149	1.5	0.791067	0.3962227	0.1738027	0.1667371	0.1690067	0.1444086
166.249	1.5	0.811536	0.3893296	0.1749772	0.1595267	0.1699023	0.154131
166.349	1.5	0.804263	0.3907054	0.1786934	0.1576716	0.169896	0.1491043
166.449	1.5	0.814466	0.3954577	0.1791582	0.1566495	0.1707664	0.1480541
166.549	1.5	0.81619	0.3946108	0.1803041	0.1569697	0.1728635	0.1545873
166.649	1.5	0.712256	0.3847001	0.1822556	0.1579648	0.1762068	0.1677787
166.749	1.5	0.706443	0.3808342	0.1825873	0.159844	0.1704915	0.1736212
166.849	1.5	0.718949	0.3823339	0.1830573	0.1621133	0.1677666	0.1697823
166.949	1.5	0.715941	0.3907256	0.1835869	0.1618865	0.169813	0.1639868
167.049	1.5	0.721239	0.3974065	0.1856215	0.1629088	0.1692773	0.1526501
167.149	1.5	0.718168	0.3926828	0.1841127	0.1620902	0.1672646	0.1618506
167.249	1.5	0.701713	0.3809884	0.1852365	0.1641776	0.1691327	0.1640183
167.349	1.5	0.690115	0.3858153	0.1856402	0.1648393	0.1687402	0.1600319
167.449	1.5	0.700205	0.3884651	0.1848781	0.1632073	0.167676	0.1631039
167.549	1.5	0.698986	0.3924099	0.1851811	0.1637449	0.1668519	0.1572107
167.649	1.5	0.716995	0.3947394	0.1843659	0.1656467	0.1673676	0.1569106
167.749	1.5	0.722065	0.3979234	0.1811406	0.1711971	0.1693165	0.156731
167.849	1.5	0.702866	0.3985529	0.1821928	0.1746487	0.1673066	0.1592725
167.949	1.5	0.684812	0.3890295	0.1838811	0.1700287	0.167799	0.1746815

168.049	1.5	0.700145	0.3919401	0.1855142	0.1693899	0.167666	0.1742909
168.149	1.5	0.7188	0.3991281	0.1849583	0.169648	0.1664574	0.170033
168.249	1.5	0.72501	0.3922231	0.1842625	0.1742265	0.1660167	0.1630526
168.349	1.5	0.712537	0.3991569	0.1854017	0.1766829	0.1674282	0.1633932
168.449	1.5	0.697623	0.3955194	0.1854244	0.1710563	0.1669277	0.1527452
168.549	1.5	0.662605	0.3893756	0.1830659	0.1657817	0.1685967	0.1684812
168.649	1.5	0.646804	0.3939571	0.1789984	0.1645544	0.174152	0.1715394
168.749	1.5	0.594133	0.3971799	0.1779166	0.1622554	0.1719507	0.1607813
168.849	1.5	0.61505	0.392604	0.1800766	0.1583302	0.16784	0.1637505
168.949	1.5	0.654813	0.3882336	0.1806154	0.1557921	0.1641051	0.1687769
169.049	1.5	0.689774	0.3940813	0.1821228	0.1610637	0.1669026	0.1619084
169.149	1.5	0.70329	0.4045765	0.1816883	0.1614577	0.1680304	0.1558294
169.249	1.5	0.716398	0.3850072	0.1827513	0.1641325	0.1693251	0.1622225
169.349	1.5	0.710396	0.3977466	0.183336	0.1682969	0.1704118	0.1466191
169.449	1.5	0.692946	0.4037507	0.1848325	0.1712212	0.1712419	0.1551222
169.549	1.5	0.649446	0.3918594	0.1844187	0.1678333	0.1724303	0.1559311
169.649	1.5	0.629193	0.3960579	0.182691	0.1691869	0.1711258	0.1471708
169.749	1.5	0.649198	0.3855327	0.1808677	0.1719414	0.1728779	0.1395618
169.849	1.5	0.666737	0.3940535	0.1797951	0.1763123	0.175474	0.1509964
169.949	1.5	0.667735	0.38726	0.1805901	0.1745158	0.1712727	0.1615636
170.049	1.5	0.636294	0.3851874	0.1810035	0.175772	0.1721119	0.1457965
170.149	1.5	0.595655	0.3843871	0.1825654	0.1792815	0.1715676	0.1403881
170.249	1.5	0.631939	0.3938851	0.1831227	0.1768086	0.175214	0.1406363
170.349	1.5	0.664676	0.394331	0.183806	0.1751808	0.1774664	0.1527417
170.449	1.5	0.692276	0.3994438	0.1835327	0.1739701	0.174119	0.1508532
170.549	1.5	0.685057	0.3909887	0.1818937	0.1752475	0.1764162	0.1488918
170.649	1.5	0.638858	0.383164	0.1816774	0.1736184	0.1812985	0.1507215
170.749	1.5	0.610428	0.386261	0.1792361	0.1665934	0.1721222	0.1530323
170.849	1.5	0.641708	0.393176	0.1816358	0.1640842	0.1703407	0.1426535
170.949	1.5	0.64544	0.3922583	0.1823348	0.1665121	0.1735604	0.1396424
171.049	1.5	0.652575	0.3960767	0.1822748	0.1687963	0.1683942	0.1385401
171.149	1.5	0.652961	0.380752	0.1769523	0.1689345	0.1752798	0.1367362
171.249	1.5	0.668533	0.3829407	0.1783159	0.1718139	0.1747077	0.1367761
171.349	1.5	0.671306	0.3954868	0.1789415	0.1713019	0.175535	0.139418
171.449	1.5	0.650335	0.3956894	0.1797598	0.1756911	0.1760864	0.1501584
171.549	1.5	0.632582	0.3937958	0.1794267	0.1790423	0.178156	0.1599923
171.649	1.5	0.637618	0.3810136	0.1780178	0.1772367	0.1774249	0.1520715
171.749	1.5	0.643182	0.3761797	0.1776304	0.1721117	0.1749105	0.1465006
171.849	1.5	0.636357	0.3827162	0.1799135	0.1722631	0.1691699	0.1432512
171.949	1.5	0.637267	0.3829775	0.1783945	0.1796134	0.1685629	0.1586675
172.049	1.5	0.616613	0.3825189	0.1742111	0.1915995	0.1730246	0.1744078
172.149	1.5	0.615508	0.3852828	0.1673729	0.18966	0.1756368	0.1572633
172.249	1.5	0.6268	0.3858784	0.1695885	0.1868521	0.1739836	0.1466601
172.349	1.5	0.619234	0.3744395	0.1714488	0.1830171	0.1746426	0.1490335
172.449	1.5	0.629887	0.3823626	0.174903	0.181184	0.1760941	0.1488936
172.549	1.5	0.651451	0.3909019	0.1776884	0.1573034	0.1753457	0.146112
172.649	1.5	0.679662	0.3847027	0.1787034	0.1533764	0.1729707	0.1475059
172.749	1.5	0.717341	0.3883836	0.1787186	0.1562378	0.1727763	0.1465435
172.849	1.5	0.737347	0.3782821	0.1756594	0.1573416	0.1752691	0.1567324
172.949	1.5	0.733793	0.3838333	0.1741175	0.1603246	0.1733289	0.1576919
173.049	1.5	0.740476	0.3943917	0.1738349	0.159404	0.176686	0.1704618
173.149	1.5	0.74881	0.3965986	0.1742509	0.1620198	0.1791426	0.1694004
173.249	1.5	0.740269	0.393399	0.1749582	0.1587044	0.1762449	0.1700211
173.349	1.5	0.730029	0.3724343	0.174144	0.1612259	0.1718627	0.1715242
173.449	1.5	0.695021	0.3742833	0.1736422	0.1670223	0.1709319	0.1594597
173.549	1.5	0.690816	0.3729045	0.1756048	0.1671567	0.1717859	0.1617509
173.649	1.5	0.694949	0.3770138	0.1782894	0.1625623	0.1759199	0.1679817
173.749	1.5	0.675237	0.375368	0.1781549	0.1631387	0.1707967	0.1611654
173.849	1.5	0.683351	0.3726324	0.1790394	0.1613622	0.1748908	0.1503137
173.949	1.5	0.626528	0.3824006	0.1815908	0.161961	0.1790967	0.1558482
174.049	1.5	0.558631	0.3733695	0.1792471	0.1632515	0.1774816	0.1731697
174.149	1.5	0.536538	0.3742941	0.1805783	0.1662864	0.1799263	0.1685852
174.249	1.5	0.527519	0.3826809	0.1820331	0.1640027	0.1792125	0.1435822
174.349	1.5	0.569609	0.3922874	0.1822393	0.1646834	0.1816746	0.1475241
174.449	1.5	0.596449	0.3846869	0.1812661	0.1623057	0.1812239	0.1647497
174.549	1.5	0.612445	0.3842452	0.182306	0.1648296	0.1798881	0.1670268
174.649	1.5	0.60432	0.3718799	0.1773937	0.164188	0.179876	0.169845
174.749	1.5	0.55264	0.3658009	0.180011	0.1645962	0.1816865	0.1565694
174.849	1.5	0.542109	0.366724	0.1794603	0.1575253	0.181453	0.1661878
174.949	1.5	0.595366	0.3715913	0.1802524	0.1610825	0.1805603	0.1683044
175.049	1.5	0.613829	0.3792475	0.1805911	0.1656507	0.1866101	0.1637057
175.149	1.5	0.589259	0.3780667	0.1810769	0.1645934	0.1868377	0.1659487
175.249	1.5	0.602452	0.3813467	0.1828666	0.1645268	0.1837223	0.1628106
175.349	1.5	0.614516	0.3795689	0.1823809	0.1620433	0.1800796	0.168443
175.449	1.5	0.614455	0.3830035	0.1838918	0.1593658	0.1784883	0.1706821
175.549	1.5	0.64075	0.3757836	0.1827643	0.1603944	0.1785898	0.1563353
175.649	1.5	0.647331	0.3848176	0.1832456	0.1618994	0.1768386	0.1739064
175.749	1.5	0.647264	0.3845148	0.1840263	0.1617439	0.178731	0.1707402
175.849	1.5	0.657567	0.3753689	0.1848698	0.1564821	0.1781741	0.1620402
175.949	1.5	0.675816	0.3759864	0.1842652	0.1538192	0.1766643	0.1689481
176.049	1.5	0.70236	0.360898	0.1813531	0.1565177	0.1801273	0.1749289
176.149	1.5	0.693455	0.3401017	0.1796732	0.1578879	0.1793015	0.1776032
176.249	1.5	0.669964	0.3663555	0.182451	0.1599519	0.1807894	0.1621821
176.349	1.5	0.685241	0.3713163	0.1841045	0.1570874	0.1754041	0.1692946
176.449	1.5	0.677132	0.3771724	0.1833989	0.1530639	0.1762661	0.1805373
176.549	1.5	0.662352	0.3771367	0.1834666	0.1552106	0.1768314	0.1739298
176.649	1.5	0.677644	0.3681614	0.1842701	0.1548544	0.1832949	0.1697002
176.749	1.5	0.688059	0.3673061	0.1850596	0.1449592	0.1795772	0.166993
176.849	1.5	0.673412	0.3639081	0.1834157	0.1350685	0.1771615	0.1682494
176.949	1.5	0.620678	0.3763353	0.1800631	0.1356762	0.1796672	0.1701194
177.049	1.5	0.641444	0.3705938	0.1816587	0.1400962	0.1752112	0.1767105
177.149	1.5	0.681082	0.3661299	0.1823541	0.1430245	0.1763446	0.1795441
177.249	1.5	0.600219	0.3612263	0.1812366	0.149354	0.1765859	0.1829608
177.349	1.5	0.470477	0.3630196	0.1818267	0.1463032	0.1707914	0.1860528
177.449	1.5	0.511391	0.3534645	0.1831566	0.1439277	0.1639852	0.1827938

177.549	1.5	0.495854	0.3542323	0.1836285	0.1361022	0.1763027	0.1840608
177.649	1.5	0.480061	0.360722	0.1829615	0.1352013	0.1830134	0.1852458
177.749	1.5	0.488218	0.3625199	0.1803823	0.1391087	0.1842679	0.1865703
177.849	1.5	0.493945	0.3617035	0.1805742	0.1426728	0.1797067	0.1868436
177.949	1.5	0.489615	0.3639823	0.1829425	0.1426051	0.176782	0.1892035
178.049	1.5	0.497559	0.3696063	0.183626	0.1438346	0.1778937	0.1870077
178.149	1.5	0.524726	0.3762364	0.1819038	0.1462684	0.1793499	0.1866259
178.249	1.5	0.542624	0.3704611	0.1805854	0.1460388	0.1790042	0.1872297
178.349	1.5	0.546597	0.365546	0.1834607	0.1437683	0.1833971	0.186351
178.449	1.5	0.564969	0.3788279	0.1831049	0.1427245	0.180638	0.1850313
178.549	1.5	0.614439	0.3759883	0.1834843	0.1453066	0.1755972	0.1845729
178.649	1.5	0.619272	0.3825718	0.1835315	0.1496015	0.1762676	0.1848282
178.749	1.5	0.615955	0.3741349	0.1843496	0.1478132	0.1725363	0.1809926
178.849	1.5	0.639368	0.3740969	0.1847307	0.1350426	0.1786902	0.1831357
178.949	1.5	0.648579	0.3800324	0.1846336	0.1322891	0.1737728	0.1842499
179.049	1.5	0.689208	0.3705947	0.1852636	0.1386622	0.1715423	0.1826149
179.149	1.5	0.731643	0.3609318	0.1803276	0.1442966	0.1743411	0.1794634
179.249	1.5	0.763557	0.3611977	0.1796663	0.1392873	0.1771313	0.1852524
179.349	1.5	0.762029	0.3607216	0.1799139	0.1343649	0.1738886	0.1867635
179.449	1.5	0.719876	0.3666937	0.1833856	0.1365038	0.1760682	0.183983
179.549	1.5	0.679917	0.3713441	0.1842224	0.1397474	0.1764414	0.1874591
179.649	1.5	0.667189	0.3712161	0.1846638	0.1431174	0.1722588	0.1876952
179.749	1.5	0.695651	0.3704852	0.1849144	0.1480897	0.1705052	0.1873532
179.849	1.5	0.744819	0.3813693	0.1843165	0.1446462	0.1713089	0.1863963
180.002	2	0.766287	0.3534875	0.1607611	0.119507	0.1601181	0.1845778
180.102	2	0.769714	0.3611544	0.1666781	0.1215892	0.1599236	0.1861745
180.202	2	0.734704	0.3752747	0.1693035	0.1260066	0.1646585	0.1837161
180.302	2	0.751791	0.3777548	0.1696349	0.1238851	0.1622727	0.1823865
180.402	2	0.742253	0.3722921	0.1701075	0.1186972	0.1615355	0.1828022
180.502	2	0.650075	0.3632829	0.1600309	0.1205111	0.1612037	0.180487
180.602	2	0.629403	0.360237	0.1461626	0.1220064	0.1581953	0.1793659
180.702	2	0.634142	0.3661814	0.1567001	0.121055	0.1645025	0.1798516
180.802	2	0.639768	0.3716823	0.1621524	0.1186131	0.1630597	0.1810342
180.902	2	0.667758	0.3693076	0.1603427	0.1203783	0.1634708	0.1820954
181.002	2	0.659455	0.3732997	0.1630042	0.1225139	0.1624681	0.1798625
181.102	2	0.655103	0.3643967	0.1636388	0.1217863	0.1606655	0.179083
181.202	2	0.679108	0.3608075	0.1621297	0.1259403	0.1603001	0.1770605
181.302	2	0.67215	0.3592779	0.1579583	0.1287293	0.157624	0.1761332
181.402	2	0.623969	0.3570596	0.155931	0.1251706	0.1598812	0.1760431
181.502	2	0.594673	0.3652219	0.1590311	0.1258249	0.1597254	0.1783094
181.602	2	0.557685	0.3710598	0.1621931	0.1233558	0.1585305	0.1784822
181.702	2	0.587888	0.3699629	0.161576	0.1221309	0.1592415	0.1795688
181.802	2	0.654079	0.3620452	0.1612142	0.1222324	0.158181	0.1766371
181.902	2	0.664766	0.3639354	0.1624508	0.1229561	0.1605605	0.1755833
182.002	2	0.662025	0.3745763	0.1623311	0.1247493	0.1579486	0.1744906
182.102	2	0.684987	0.3827038	0.1628802	0.1248916	0.1567604	0.1742039
182.202	2	0.709179	0.3823417	0.1622231	0.1283039	0.1606511	0.174431
182.302	2	0.693573	0.3747876	0.1620888	0.1278575	0.1603123	0.169414
182.402	2	0.701083	0.3699137	0.1611173	0.1255053	0.1593333	0.167405
182.502	2	0.691567	0.3602167	0.1617032	0.1267271	0.1600804	0.1739738
182.602	2	0.681808	0.3666999	0.1658161	0.127104	0.1637979	0.1766642
182.702	2	0.694441	0.3727261	0.1639952	0.1268073	0.1608599	0.1758851
182.802	2	0.705864	0.3735209	0.16452	0.1269534	0.161	0.175605
182.902	2	0.7142	0.3651547	0.1634165	0.1248681	0.1608936	0.1755338
183.002	2	0.710143	0.3660138	0.1620547	0.1272898	0.1613457	0.1740452
183.102	2	0.581384	0.372243	0.163315	0.1292445	0.1587844	0.1710265
183.202	2	0.533663	0.366763	0.165438	0.1314341	0.161311	0.1695585
183.302	2	0.547341	0.3643076	0.1652153	0.128826	0.1622004	0.1720412
183.402	2	0.585347	0.3633126	0.1646985	0.1271463	0.1609847	0.1751345
183.502	2	0.637118	0.368522	0.1684526	0.1284695	0.163348	0.1737362
183.602	2	0.654472	0.3688434	0.1668406	0.132563	0.1631352	0.1756011
183.702	2	0.667483	0.3648396	0.1661534	0.1341246	0.1623002	0.1741702
183.802	2	0.694838	0.3653224	0.1660007	0.1307082	0.1619277	0.1746513
183.902	2	0.638899	0.3669516	0.1673659	0.1297015	0.1603456	0.1765889
184.002	2	0.637515	0.3754323	0.1609326	0.1302203	0.1598794	0.1774734
184.102	2	0.683588	0.3789453	0.1615371	0.1300054	0.15988	0.176651
184.202	2	0.711978	0.3829082	0.1659025	0.1309603	0.1620118	0.1735248
184.302	2	0.711977	0.383496	0.1652695	0.1316016	0.1609693	0.175138
184.402	2	0.694698	0.3735519	0.163978	0.128942	0.1601344	0.1757649
184.502	2	0.654998	0.3692378	0.1642414	0.1312437	0.1607593	0.1742418
184.602	2	0.624149	0.3882281	0.1618719	0.1298395	0.1628261	0.1741975
184.702	2	0.608755	0.3853438	0.1633979	0.1319412	0.1636448	0.1715313
184.802	2	0.643389	0.3820744	0.1651476	0.1331995	0.1627999	0.1731828
184.902	2	0.502288	0.3816251	0.1665661	0.1303809	0.1617214	0.1750111
185.002	2	0.464111	0.38432	0.1658566	0.1305253	0.1630626	0.1731447
185.102	2	0.509761	0.376105	0.1662429	0.1304584	0.1629063	0.1741604
185.202	2	0.513078	0.3889332	0.1647949	0.1305784	0.1627265	0.1734536
185.302	2	0.51764	0.378108	0.1656843	0.1315528	0.1618854	0.1730183
185.402	2	0.531365	0.3767714	0.1680164	0.1322122	0.1648134	0.1710455
185.502	2	0.538135	0.3816517	0.1683076	0.131308	0.1646522	0.1724145
185.602	2	0.578499	0.3827314	0.166547	0.1312503	0.1647947	0.1720245
185.702	2	0.529745	0.3790172	0.1684333	0.130055	0.1662581	0.1737785
185.802	2	0.488531	0.3768076	0.1670063	0.131453	0.1674056	0.172664
185.902	2	0.504384	0.3777936	0.1661589	0.131562	0.1655118	0.173958
186.002	2	0.535048	0.3834059	0.1671506	0.1328201	0.1661737	0.172901
186.102	2	0.534564	0.3744723	0.1686414	0.1306522	0.1661514	0.173212
186.202	2	0.548209	0.3686252	0.1659595	0.1310271	0.1685978	0.1703804
186.302	2	0.588821	0.3694077	0.1666882	0.1291687	0.1649854	0.1716043
186.402	2	0.599917	0.3713535	0.1666461	0.1299905	0.1636995	0.1714624
186.502	2	0.570293	0.3672185	0.1670312	0.1315733	0.1640852	0.1708979
186.602	2	0.620659	0.3692864	0.1713386	0.1313693	0.1628306	0.1726486
186.702	2	0.636814	0.3767211	0.1708895	0.1301862	0.165774	0.1707382
186.802	2	0.658083	0.3751369	0.1713037	0.1282096	0.1653331	0.1709617
186.902	2	0.680908	0.3727039	0.1718753	0.1265035	0.1696436	0.1728874
187.002	2	0.690681	0.3703069	0.1705829	0.1264906	0.1705311	0.1722165

187.102	2	0.66807	0.3733065	0.1709423	0.1273516	0.1671823	0.17321
187.202	2	0.680148	0.3847042	0.1702312	0.1290516	0.1689153	0.1737016
187.302	2	0.715274	0.3728857	0.1705261	0.128683	0.1697962	0.1753715
187.402	2	0.734431	0.3742903	0.1697515	0.1325768	0.168141	0.1731992
187.502	2	0.722163	0.3697623	0.1647397	0.1334973	0.1699884	0.1728509
187.602	2	0.731473	0.3785761	0.165295	0.1288543	0.1651239	0.1724633
187.702	2	0.719436	0.3710333	0.168924	0.1331045	0.1653806	0.173306
187.802	2	0.669646	0.3663402	0.1698657	0.1313567	0.1681082	0.1732244
187.902	2	0.69096	0.3723962	0.16921	0.1304064	0.1643578	0.1715779
188.002	2	0.716422	0.3755786	0.1691757	0.1304772	0.1636599	0.1712099
188.102	2	0.71241	0.3617285	0.168197	0.1300254	0.1653788	0.1722244
188.202	2	0.702918	0.367511	0.1695013	0.131341	0.1633282	0.1710595
188.302	2	0.701111	0.3620533	0.1709678	0.1311774	0.1633897	0.1706932
188.402	2	0.657219	0.3690791	0.1699563	0.1254388	0.1628305	0.1706677
188.502	2	0.631706	0.3740155	0.1703954	0.1261557	0.1621258	0.1764818
188.602	2	0.684493	0.3769287	0.1725349	0.1264668	0.1614369	0.1744889
188.702	2	0.686247	0.3764153	0.1712241	0.1237033	0.1623036	0.1726257
188.802	2	0.683577	0.383677	0.171492	0.1251372	0.1612094	0.1715044
188.902	2	0.706288	0.3850197	0.1721924	0.1214734	0.1577942	0.172755
189.002	2	0.724779	0.3770812	0.1727631	0.1215897	0.1608045	0.1718148
189.102	2	0.695294	0.3883381	0.1734394	0.1218819	0.159443	0.1724198
189.202	2	0.70887	0.3870403	0.1729766	0.118759	0.160709	0.1716836
189.302	2	0.728614	0.3774114	0.1760563	0.1274146	0.1556922	0.1744828
189.402	2	0.74154	0.3730307	0.1744627	0.1272555	0.1561166	0.1735459
189.502	2	0.743237	0.3722119	0.1746266	0.1249008	0.1580491	0.1730253
189.602	2	0.706386	0.3733842	0.1718706	0.1240639	0.1591441	0.1742548
189.702	2	0.690137	0.384905	0.173484	0.1232922	0.1574447	0.1769411
189.802	2	0.672458	0.3824333	0.1750764	0.1223772	0.1561426	0.1763887
189.902	2	0.696639	0.3880955	0.1752538	0.1219438	0.1556289	0.1754409
190.002	2	0.70443	0.3786119	0.1722212	0.1246792	0.1583743	0.1776309
190.102	2	0.696628	0.3846377	0.1728616	0.1245771	0.1602014	0.1753116
190.202	2	0.693026	0.3858117	0.1737006	0.1236528	0.1555133	0.1756006
190.302	2	0.723076	0.3819954	0.1731353	0.123992	0.1546306	0.1727604
190.402	2	0.737831	0.3766012	0.1745748	0.1276009	0.1549604	0.1766319
190.502	2	0.70258	0.3672457	0.1737459	0.1264302	0.1573609	0.1759541
190.602	2	0.692213	0.3760142	0.1742983	0.1250648	0.161307	0.1732262
190.702	2	0.687237	0.372777	0.1733052	0.1277487	0.1616957	0.1737568
190.802	2	0.636611	0.3683971	0.1787944	0.1279774	0.1589338	0.1749384
190.902	2	0.641762	0.3622029	0.176372	0.1288515	0.1573553	0.1769407
191.002	2	0.646872	0.3776289	0.1735365	0.1314726	0.1533676	0.176231
191.102	2	0.649451	0.3723022	0.1750225	0.1326909	0.1533958	0.1762459
191.202	2	0.66403	0.3723432	0.1730485	0.1330826	0.1508988	0.1751255
191.302	2	0.673446	0.3726341	0.1732204	0.1348408	0.1547117	0.1754244
191.402	2	0.683973	0.375995	0.1723171	0.1369133	0.1532083	0.1750782
191.502	2	0.682315	0.3835936	0.1745771	0.1356623	0.1572998	0.1761373
191.602	2	0.683732	0.3687686	0.1765439	0.130829	0.1605918	0.1759677
191.702	2	0.645779	0.3731123	0.1768657	0.1314196	0.1552307	0.1771011
191.802	2	0.6591	0.3657592	0.178054	0.1323617	0.1561131	0.1750405
191.902	2	0.705907	0.3672938	0.1753687	0.1319196	0.1543542	0.175477
192.002	2	0.684659	0.3650872	0.175646	0.1325303	0.1556423	0.1778029
192.102	2	0.66888	0.3627381	0.1767251	0.132291	0.1558857	0.1768163
192.202	2	0.665483	0.3673141	0.1744939	0.1322299	0.1442878	0.1779554
192.302	2	0.659699	0.3673931	0.174586	0.1371095	0.1493208	0.1777701
192.402	2	0.677676	0.3626698	0.1779273	0.1412937	0.1542804	0.1629184
192.502	2	0.693449	0.3588997	0.1759807	0.140005	0.1525866	0.1566975
192.602	2	0.699275	0.3667658	0.1774635	0.1381359	0.15341	0.1597861
192.702	2	0.68951	0.3772408	0.1767572	0.1376487	0.1553129	0.1561027
192.802	2	0.663584	0.3629425	0.1779325	0.1359329	0.1554009	0.1456135
192.902	2	0.659961	0.3556529	0.17471	0.1420957	0.153441	0.1439174
193.002	2	0.67572	0.3585258	0.1754589	0.1423807	0.1539063	0.1510868
193.102	2	0.698308	0.3655384	0.1781612	0.1399525	0.1517489	0.1469357
193.202	2	0.697793	0.3690795	0.1766613	0.1385551	0.150702	0.1453618
193.302	2	0.479563	0.3730581	0.1786118	0.1385619	0.1517887	0.145158
193.402	2	0.475174	0.377039	0.1746995	0.140642	0.152928	0.1446797
193.502	2	0.528632	0.3757042	0.1755003	0.1389783	0.1489648	0.1524213
193.602	2	0.594118	0.3703308	0.1748087	0.1346028	0.1502015	0.152166
193.702	2	0.617062	0.3702717	0.1743621	0.136349	0.1510901	0.1615175
193.802	2	0.60596	0.3774945	0.1733314	0.1411562	0.1468509	0.1488049
193.902	2	0.636464	0.3773684	0.1747918	0.1403759	0.1481581	0.1708599
194.002	2	0.650324	0.3779649	0.1761576	0.1404797	0.1470484	0.1422247
194.102	2	0.616619	0.3873031	0.175469	0.1417012	0.1486915	0.1533592
194.202	2	0.600775	0.3688204	0.1724455	0.1423365	0.1461142	0.1472063
194.302	2	0.638045	0.3751688	0.1686329	0.1414849	0.1488081	0.1448097
194.402	2	0.621888	0.3690567	0.1691032	0.1399391	0.1523933	0.1436182
194.502	2	0.637409	0.3843057	0.1711922	0.1382472	0.1469653	0.156435
194.602	2	0.599924	0.3934985	0.1734391	0.1396367	0.1464149	0.1537977
194.702	2	0.616777	0.3927868	0.1720339	0.1363493	0.1478754	0.149209
194.802	2	0.644977	0.3768479	0.172324	0.1361521	0.1474697	0.1399132
194.902	2	0.650171	0.3748206	0.1727063	0.1376472	0.1494977	0.1437117
195.002	2	0.65065	0.3821027	0.1697089	0.1364636	0.1481991	0.1487871
195.102	2	0.640141	0.3799656	0.1713675	0.1362384	0.1499151	0.1490001
195.202	2	0.674699	0.3802292	0.1716943	0.1403971	0.1506449	0.1461366
195.302	2	0.663588	0.3850143	0.1687144	0.1408496	0.1480821	0.1576638
195.402	2	0.658503	0.3803016	0.169486	0.1387588	0.1501933	0.1634586
195.502	2	0.652299	0.3758647	0.1668628	0.140764	0.1470416	0.1472203
195.602	2	0.625313	0.385612	0.1675774	0.1420281	0.1466786	0.151551
195.702	2	0.648956	0.3829805	0.1679664	0.138291	0.1496488	0.1615445
195.802	2	0.667624	0.3798485	0.1661747	0.1401025	0.1493914	0.1650336
195.902	2	0.66373	0.3935383	0.1689856	0.1423014	0.1461355	0.1512587
196.002	2	0.640716	0.3832398	0.1695959	0.143447	0.1480462	0.1414938
196.102	2	0.575507	0.3869951	0.1716827	0.1408374	0.1460199	0.1537398
196.202	2	0.618005	0.3832212	0.1671523	0.1407946	0.1450571	0.1489054
196.302	2	0.636783	0.3793926	0.1653479	0.1445836	0.1450257	0.1500519
196.402	2	0.645515	0.378631	0.1658553	0.1409328	0.1468015	0.1520441
196.502	2	0.609282	0.3823148	0.1680702	0.1427939	0.1473345	0.1521145

196.602	2	0.596418	0.3768009	0.1688078	0.1422264	0.1490479	0.1449122
196.702	2	0.624023	0.3766248	0.1692277	0.1432889	0.1482546	0.1466301
196.802	2	0.615541	0.3777187	0.1687036	0.1421951	0.1489942	0.1452563
196.902	2	0.629739	0.3796667	0.1695225	0.1425952	0.1516926	0.145162
197.002	2	0.635088	0.3802235	0.1704282	0.142173	0.1506664	0.1488428
197.102	2	0.605082	0.3792827	0.1703542	0.1445043	0.1499048	0.1452726
197.202	2	0.628484	0.3856042	0.1694792	0.1441134	0.1492376	0.15026
197.302	2	0.634018	0.388655	0.1682679	0.1417871	0.1488036	0.1483485
197.402	2	0.602826	0.3876187	0.1666827	0.1432568	0.1513693	0.1504212
197.502	2	0.579369	0.3918589	0.1599386	0.1448964	0.1515424	0.1623685
197.602	2	0.604461	0.3836544	0.1588707	0.1444497	0.1517512	0.1719035
197.702	2	0.646321	0.3850315	0.1673748	0.146894	0.1508207	0.1641833
197.802	2	0.621855	0.382561	0.1685704	0.1440174	0.1523945	0.1579243
197.902	2	0.589727	0.387839	0.1707416	0.1450255	0.1530234	0.1588045
198.002	2	0.611992	0.3792274	0.1690397	0.1475678	0.1529533	0.1634928
198.102	2	0.617672	0.3825316	0.1715988	0.1506158	0.1537982	0.1552374
198.202	2	0.625091	0.379377	0.1699087	0.1482547	0.1525146	0.1559226
198.302	2	0.612492	0.3840963	0.1687393	0.1440835	0.1512192	0.1541805
198.402	2	0.63251	0.3791853	0.1689704	0.1451903	0.1506841	0.153198
198.502	2	0.625149	0.3799627	0.1676235	0.142647	0.1516978	0.1593388
198.602	2	0.619856	0.3913299	0.1649685	0.1479596	0.1508406	0.1603926
198.702	2	0.640941	0.3908813	0.1643576	0.1477736	0.1519357	0.1573822
198.802	2	0.667398	0.3816124	0.1642521	0.1471144	0.1523011	0.152889
198.902	2	0.665357	0.3925912	0.1619392	0.1483823	0.1522141	0.1514688
199.002	2	0.663298	0.3890255	0.1594403	0.1559246	0.1499117	0.1566696
199.102	2	0.657425	0.381749	0.1511898	0.1610569	0.1513764	0.1511113
199.202	2	0.668916	0.3745922	0.151	0.1571618	0.1518154	0.1461594
199.302	2	0.675436	0.3777234	0.1551495	0.1563454	0.1494366	0.1492391
199.402	2	0.65461	0.3798958	0.1566156	0.1549904	0.1517204	0.145569
199.502	2	0.667382	0.3798207	0.1593423	0.1505357	0.1520459	0.1462016
199.602	2	0.66015	0.3812446	0.1593093	0.1501218	0.1509392	0.146767
199.702	2	0.628551	0.3866485	0.1632467	0.1520446	0.1514436	0.1488715
199.802	2	0.617638	0.3779505	0.1659634	0.1491612	0.1524787	0.1426623
199.902	2	0.617785	0.3748673	0.1670239	0.1484001	0.1501247	0.1471026
200.002	2	0.650586	0.3747961	0.1673237	0.1495355	0.1523285	0.1522545
200.102	2	0.635107	0.3847229	0.1668713	0.151458	0.150743	0.1396217
200.202	2	0.623745	0.3802359	0.1682263	0.1508215	0.1506108	0.1481594
200.302	2	0.642581	0.3761092	0.1678692	0.15132	0.1494785	0.1606826
200.402	2	0.634608	0.3816826	0.1694533	0.1502439	0.1507992	0.1562102
200.502	2	0.637094	0.3811924	0.1687745	0.15072	0.1519771	0.1505222
200.602	2	0.619595	0.3810561	0.1687075	0.1529419	0.1547544	0.1577228
200.702	2	0.603061	0.3903617	0.1678824	0.1565699	0.1532876	0.1499132
200.802	2	0.628937	0.3809813	0.1695964	0.1589864	0.1504549	0.1440309
200.902	2	0.639444	0.3824076	0.1714641	0.157236	0.1531919	0.1326787
201.002	2	0.630691	0.3894764	0.1795355	0.1193903	0.1520816	0.1322971
201.102	2	0.615903	0.3903801	0.181458	0.1191873	0.1529991	0.1375177
201.202	2	0.596463	0.3796856	0.1808215	0.1187344	0.1517509	0.1438544
201.302	2	0.581575	0.383768	0.18132	0.1178881	0.1541691	0.1398356
201.402	2	0.568992	0.3835657	0.1802439	0.1187773	0.1512029	0.1343665
201.502	2	0.592875	0.3758456	0.18072	0.1248684	0.148058	0.1465452
201.602	2	0.631403	0.3811834	0.1829419	0.1209274	0.1467673	0.1485267
201.702	2	0.634793	0.390168	0.1865699	0.1170199	0.1474987	0.1369636
201.802	2	0.630905	0.3772146	0.1889864	0.1167874	0.1489144	0.1464781
201.902	2	0.60672	0.3911482	0.187236	0.1187841	0.1474708	0.1453805
202.002	2	0.582764	0.3845211	0.1822443	0.1175599	0.1468596	0.1456723
202.102	2	0.583052	0.384588	0.181916	0.1166945	0.1476471	0.1536466
202.202	2	0.592222	0.386115	0.1753389	0.1160854	0.1523789	0.1542243
202.302	2	0.554028	0.3786657	0.1726964	0.1141937	0.150447	0.1667337
202.402	2	0.546429	0.3820254	0.1714771	0.1137797	0.1489553	0.1636693
202.502	2	0.557204	0.3819087	0.168199	0.1129969	0.1485488	0.1642407
202.602	2	0.566587	0.3819587	0.1694451	0.1131276	0.1468973	0.1603252
202.702	2	0.591504	0.3861125	0.1725547	0.1141808	0.1459754	0.1531919
202.802	2	0.597086	0.3798797	0.1742637	0.116158	0.1512291	0.1605897
202.902	2	0.609894	0.3769673	0.1777232	0.1137038	0.1444634	0.1523505
203.002	2	0.619328	0.3746555	0.1800377	0.1098933	0.1500795	0.1527539
203.102	2	0.618813	0.3714137	0.1849652	0.1103571	0.1506309	0.1634813
203.202	2	0.604719	0.381213	0.1864811	0.1132048	0.1540097	0.1413087
203.302	2	0.577765	0.3706554	0.1859337	0.1151867	0.1529184	0.1498553
203.402	2	0.561107	0.3831285	0.1899806	0.1149254	0.1545751	0.1608278
203.502	2	0.570303	0.3798143	0.1935597	0.1159376	0.1555204	0.159872
203.602	2	0.583472	0.3756371	0.1919896	0.1103189	0.1551617	0.1610784
203.702	2	0.595598	0.3678892	0.1910263	0.1101936	0.1486926	0.1642481
203.802	2	0.56489	0.3728779	0.1926262	0.1106674	0.1539681	0.1545213
203.902	2	0.563622	0.3713637	0.1873947	0.108479	0.1575938	0.1568515
204.002	2	0.617264	0.3780013	0.1778192	0.1070287	0.1558527	0.1553231
204.102	2	0.638425	0.37274	0.1779887	0.109867	0.1560559	0.1545466
204.202	2	0.639439	0.3643384	0.1769947	0.1116238	0.1551641	0.1492495
204.302	2	0.67786	0.3692749	0.1771151	0.1063192	0.1543929	0.1587798
204.402	2	0.685941	0.3716062	0.1799217	0.1060835	0.1564834	0.1524935
204.502	2	0.664284	0.3740202	0.1859645	0.1096777	0.1567395	0.1576419
204.602	2	0.664792	0.3753251	0.1848324	0.1111008	0.1576594	0.1654963
204.702	2	0.635415	0.3778869	0.1792587	0.1094945	0.1581882	0.1593676
204.802	2	0.641502	0.3830345	0.1813856	0.1094628	0.1547614	0.1614684
204.902	2	0.645247	0.3660821	0.1878849	0.1126795	0.1554225	0.1668201
205.002	2	0.668009	0.3658816	0.1886804	0.1134119	0.1564605	0.1621368
205.102	2	0.678767	0.3691425	0.1900005	0.1105216	0.15772	0.1704927
205.202	2	0.63961	0.3686837	0.1880195	0.109955	0.1583975	0.1747784
205.302	2	0.640566	0.3748172	0.1705226	0.1100663	0.1587226	0.1824954
205.402	2	0.632945	0.3691118	0.170453	0.1121804	0.156643	0.1876826
205.502	2	0.655487	0.368382	0.1694501	0.1126133	0.1567319	0.1858569
205.602	2	0.677371	0.3786234	0.1696529	0.1084788	0.1572809	0.1859626
205.702	2	0.642661	0.3753362	0.1707935	0.114932	0.1563613	0.1854867
205.802	2	0.643341	0.3747497	0.1694545	0.1135468	0.1561389	0.1882774
205.902	2	0.587476	0.3594806	0.1742671	0.1110758	0.1549764	0.1862567
206.002	2	0.598667	0.3617255	0.1790372	0.1074384	0.1556395	0.1844893

206.102	2	0.641114	0.3630609	0.1741205	0.1093932	0.1540345	0.183144
206.202	2	0.633745	0.3667713	0.1734997	0.1107784	0.1561372	0.1839578
206.302	2	0.63566	0.3698315	0.1730719	0.108876	0.1545064	0.1831982
206.402	2	0.629338	0.3657427	0.1745265	0.1104384	0.1539608	0.183368
206.502	2	0.584313	0.3686641	0.1753559	0.1155279	0.1520292	0.180909
206.602	2	0.592016	0.3691199	0.1718553	0.1173679	0.1511413	0.1801334
206.702	2	0.640647	0.3708303	0.1730894	0.1148455	0.1527025	0.1788043
206.802	2	0.665267	0.3608106	0.1725115	0.10768	0.150038	0.1753301
206.902	2	0.649839	0.3662298	0.1733901	0.1102222	0.1516981	0.1771398
207.002	2	0.627861	0.3299572	0.172299	0.1115401	0.1524121	0.1778818
207.102	2	0.57644	0.3563547	0.1778005	0.1140549	0.15058	0.1774767
207.202	2	0.602749	0.3629064	0.1746752	0.1116228	0.1510152	0.1797715
207.302	2	0.613306	0.3707153	0.171521	0.1135482	0.1507664	0.1801769
207.402	2	0.582098	0.3546415	0.1700952	0.1129447	0.1490319	0.1826347
207.502	2	0.608445	0.3454284	0.1709503	0.110586	0.1496079	0.1843094
207.602	2	0.595885	0.3531937	0.1733757	0.1083992	0.1502482	0.1818293
207.702	2	0.55755	0.3514543	0.1705646	0.1102277	0.1505908	0.1793542
207.802	2	0.518553	0.3498619	0.1667338	0.1109902	0.150738	0.1812142
207.902	2	0.4991	0.3455713	0.1671841	0.108832	0.1482632	0.1801347
208.002	2	0.468667	0.3378153	0.1686891	0.1059664	0.1516623	0.1811614
208.102	2	0.488795	0.3428112	0.1696707	0.1081619	0.1528237	0.1799465
208.202	2	0.543791	0.3427298	0.1631769	0.1086738	0.1503162	0.180231
208.302	2	0.574068	0.3505348	0.1701303	0.109083	0.1497269	0.1805971
208.402	2	0.528089	0.355084	0.1711705	0.110681	0.1501654	0.1825292
208.502	2	0.544027	0.3588832	0.1599865	0.110512	0.1518345	0.1813195
208.602	2	0.53173	0.3545284	0.1627497	0.1085026	0.1521822	0.181669
208.702	2	0.556061	0.3572815	0.1664382	0.1080337	0.1520667	0.1823087
208.802	2	0.561979	0.3619595	0.1710269	0.108475	0.1573332	0.1818362
208.902	2	0.583809	0.3668897	0.1688029	0.1108574	0.1546192	0.1802373
209.002	2	0.607708	0.3633704	0.1604067	0.1058351	0.1560664	0.1787891
209.102	2	0.632914	0.3684078	0.1606729	0.1029046	0.1567826	0.1785791
209.202	2	0.652207	0.3477849	0.1648408	0.1103352	0.1579016	0.1802819
209.302	2	0.662287	0.3558752	0.1670414	0.1146976	0.1525614	0.1799314
209.402	2	0.684118	0.3510813	0.169004	0.1145608	0.1522537	0.1805825
209.502	2	0.690624	0.3569942	0.17139	0.1116613	0.1535491	0.1810905
209.602	2	0.701584	0.3555741	0.170463	0.1127257	0.152781	0.1775032
209.702	2	0.68943	0.3541397	0.1692791	0.1165853	0.1517806	0.1778645
209.802	2	0.683563	0.3580937	0.1723361	0.1162342	0.1510469	0.1775512
209.902	2	0.686146	0.3540891	0.1714721	0.1135033	0.1499426	0.1763086
210.002	2	0.691335	0.351005	0.1624911	0.1158019	0.1449194	0.1771899
210.102	2	0.665668	0.3519574	0.164415	0.1144734	0.1501639	0.1743336
210.202	2	0.652478	0.3605358	0.169282	0.1159365	0.1494697	0.1777675
210.302	2	0.631861	0.3691023	0.1594854	0.1038265	0.1504846	0.1781787
210.402	2	0.648093	0.3623796	0.1587197	0.1070348	0.1487233	0.1761358
210.502	2	0.642675	0.3568676	0.1614754	0.1163502	0.148793	0.1754325
210.602	2	0.634226	0.3590238	0.1637545	0.1171363	0.1519831	0.1721824
210.702	2	0.656265	0.3598067	0.1645229	0.1185167	0.1545813	0.1719985
210.802	2	0.671808	0.3703919	0.1581082	0.1194569	0.1498817	0.1762092
210.902	2	0.654434	0.3794811	0.1354771	0.1173805	0.1485002	0.1758634
211.002	2	0.672933	0.3795563	0.1497425	0.1090701	0.1493756	0.1758
211.102	2	0.647619	0.3739389	0.1576989	0.1070209	0.149319	0.1730534
211.202	2	0.679389	0.3663791	0.1573175	0.1160824	0.1494028	0.1713987
211.302	2	0.685849	0.3626173	0.1520152	0.1188263	0.1476379	0.1711647
211.402	2	0.692865	0.3607515	0.1495906	0.1189324	0.1488029	0.1718446
211.502	2	0.665502	0.3641418	0.1538602	0.1204785	0.145859	0.173079
211.602	2	0.641495	0.3756338	0.1539606	0.1181009	0.145477	0.1757673
211.702	2	0.639377	0.3773658	0.1520056	0.1183379	0.1475239	0.1788903
211.802	2	0.617528	0.3677067	0.1529559	0.1179592	0.1482556	0.1765265
211.902	2	0.616585	0.366273	0.1526596	0.1136158	0.1462782	0.1764644
212.002	2	0.615888	0.3723396	0.151706	0.1022132	0.14465	0.1774678
212.102	2	0.656973	0.3838179	0.1521799	0.1048754	0.1457204	0.1769723
212.202	2	0.663337	0.3829831	0.1606537	0.1130574	0.1471587	0.175173
212.302	2	0.641231	0.3802931	0.1569385	0.1171262	0.1440911	0.1714924
212.402	2	0.621213	0.371206	0.1528194	0.117168	0.1447463	0.1696423
212.502	2	0.629948	0.3626712	0.1550144	0.1190179	0.1434291	0.1741853
212.602	2	0.634276	0.3675372	0.1563284	0.1157011	0.1433635	0.1710869
212.702	2	0.639778	0.3739125	0.1557291	0.1177723	0.1474415	0.1714493
212.802	2	0.660457	0.3753954	0.1534527	0.1210361	0.1468846	0.1709839
212.902	2	0.653491	0.3662346	0.1559392	0.1189414	0.1485321	0.1703084
213.002	2	0.64273	0.3658057	0.1580793	0.1159731	0.1491814	0.1691938
213.102	2	0.623564	0.3711812	0.1593658	0.1168139	0.1483456	0.1664805
213.202	2	0.609863	0.3753264	0.160181	0.116545	0.1494326	0.1675622
213.302	2	0.582321	0.3718982	0.1582077	0.1160616	0.1479884	0.1680531
213.402	2	0.602176	0.370722	0.156963	0.1061479	0.148328	0.1691568
213.502	2	0.638642	0.3667192	0.1590196	0.108601	0.1487601	0.1688352
213.602	2	0.662507	0.3693911	0.1568674	0.1048983	0.1500514	0.1700924
213.702	2	0.67228	0.3663781	0.1569753	0.1103159	0.1504145	0.1703884
213.802	2	0.660996	0.365059	0.1576352	0.1099576	0.1471722	0.1692052
213.902	2	0.635588	0.3686104	0.1570781	0.1094313	0.1464701	0.1694057
214.002	2	0.53806	0.375182	0.1586841	0.1128761	0.1450943	0.1688876
214.102	2	0.554337	0.3726801	0.1608056	0.1163622	0.1465202	0.1675915
214.202	2	0.596406	0.378822	0.1652419	0.1135698	0.1496918	0.1666483
214.302	2	0.619302	0.3791184	0.1607772	0.1128065	0.1497627	0.1690908
214.402	2	0.619735	0.3718872	0.1543039	0.1111588	0.14819	0.1698066
214.502	2	0.522042	0.3712697	0.1566289	0.110525	0.1508012	0.169554
214.602	2	0.511429	0.3861527	0.1602026	0.1122907	0.1496345	0.1658557
214.702	2	0.562273	0.3838213	0.1611205	0.1128154	0.1474456	0.1621849
214.802	2	0.650809	0.3814392	0.156397	0.1098132	0.1475795	0.1662684
214.902	2	0.689907	0.374043	0.1581561	0.1114457	0.1479613	0.1643822
215.002	2	0.71804	0.3845226	0.1580567	0.1105418	0.1501496	0.164297
215.102	2	0.721669	0.3761284	0.1573453	0.1093047	0.1488022	0.1660809
215.202	2	0.742576	0.3846064	0.158501	0.1137449	0.1492219	0.1643594
215.302	2	0.767846	0.3776979	0.1583369	0.1128677	0.1473869	0.1634831
215.402	2	0.735849	0.3738385	0.1560763	0.1117808	0.147432	0.1634383
215.502	2	0.684682	0.3784518	0.157783	0.1120677	0.1476898	0.1643841

215.602	2	0.685553	0.3783944	0.1577804	0.1117028	0.1478542	0.1630494
215.702	2	0.709752	0.3745995	0.1615263	0.1131249	0.1491593	0.1643007
215.802	2	0.719156	0.3708284	0.1578251	0.1141871	0.1511768	0.1640573
215.902	2	0.738566	0.3631991	0.1550171	0.1052735	0.1531091	0.1662001
216.002	2	0.686388	0.3641879	0.1554248	0.1030673	0.1516488	0.1638142
216.102	2	0.70758	0.3615094	0.1566198	0.1140403	0.1489504	0.1637996
216.202	2	0.733482	0.3548886	0.1587344	0.1161613	0.1498502	0.1621435
216.302	2	0.72569	0.3438701	0.1587139	0.1142775	0.149504	0.1630472
216.402	2	0.647725	0.3403855	0.1596222	0.1145738	0.152312	0.1616041
216.502	2	0.641757	0.3282363	0.1581338	0.1168672	0.1491409	0.161309
216.602	2	0.646977	0.324913	0.1559241	0.1187591	0.1476494	0.1624614
216.702	2	0.627524	0.337219	0.1568813	0.1170506	0.1482165	0.1606289
216.802	2	0.606438	0.3333242	0.1607287	0.1192039	0.1515801	0.1613699
216.902	2	0.638605	0.3350736	0.1615837	0.1151562	0.1552502	0.1601996
217.002	2	0.637289	0.3286539	0.160794	0.1165368	0.1526029	0.1602543
217.102	2	0.637499	0.341833	0.1563602	0.1134544	0.1512811	0.1613998
217.202	2	0.653982	0.351053	0.1580416	0.114252	0.1545591	0.1628669
217.302	2	0.665679	0.3463399	0.1590602	0.1142636	0.1511827	0.1642609
217.402	2	0.671849	0.3509913	0.1626004	0.114871	0.1502852	0.1636195
217.502	2	0.66442	0.3470059	0.164022	0.1066535	0.1523716	0.1626459
217.602	2	0.659812	0.3554224	0.1619088	0.1065658	0.1479204	0.163485
217.702	2	0.668601	0.3531139	0.1619533	0.1153554	0.1524511	0.1640815
217.802	2	0.63157	0.3480803	0.1619086	0.1166919	0.1533363	0.1618406
217.902	2	0.641642	0.3565744	0.1610656	0.115177	0.1495127	0.1622328
218.002	2	0.661908	0.3546208	0.1605128	0.1185628	0.1513014	0.1604758
218.102	2	0.667251	0.3501258	0.1631078	0.1175209	0.1506951	0.1610095
218.202	2	0.672176	0.3539857	0.161536	0.1180144	0.1498057	0.1626332
218.302	2	0.690633	0.3544695	0.1619918	0.1087858	0.152744	0.1611863
218.402	2	0.659671	0.3625989	0.164635	0.114449	0.1529471	0.1631702
218.502	2	0.659127	0.3707463	0.1591879	0.1140633	0.1493235	0.1632037
218.602	2	0.67174	0.3724763	0.156836	0.1108185	0.1490693	0.1634353
218.702	2	0.636402	0.3685194	0.1619358	0.1151979	0.1500386	0.1610674
218.802	2	0.624069	0.3786818	0.1617169	0.1162754	0.1500339	0.1595964
218.902	2	0.614891	0.3780347	0.1594445	0.1202866	0.1472365	0.1611773
219.002	2	0.631325	0.3742178	0.1598404	0.1193758	0.1473835	0.1628178
219.102	2	0.624953	0.3776739	0.159735	0.1159367	0.1464747	0.1637411
219.202	2	0.638507	0.3802955	0.1633945	0.1141196	0.1489343	0.1635612
219.302	2	0.643029	0.3812906	0.1664973	0.1119151	0.1444214	0.1643738
219.402	2	0.66899	0.3717324	0.1603835	0.1092367	0.1420805	0.1652715
219.502	2	0.643314	0.3663125	0.157533	0.1081563	0.1445931	0.1659191
219.602	2	0.662625	0.3723254	0.1586652	0.1079076	0.1462792	0.1649572
219.702	2	0.666712	0.370453	0.1545377	0.109547	0.1443364	0.1621879
219.802	2	0.676936	0.3796064	0.1536699	0.1073987	0.1448609	0.1626416
219.902	2	0.678375	0.3756566	0.1527786	0.1024194	0.1441522	0.1630312
220.002	2	0.663223	0.3764854	0.1559007	0.1070963	0.1458701	0.1620287
220.102	2	0.655218	0.3724345	0.1547	0.1110172	0.1477685	0.1613353
220.202	2	0.665543	0.3794969	0.148483	0.1098367	0.145214	0.159413
220.302	2	0.652947	0.3772785	0.1594454	0.1098004	0.1442198	0.1615155
220.402	2	0.632013	0.3810369	0.1624729	0.109425	0.1446569	0.1629903
220.502	2	0.63605	0.3657342	0.158421	0.1092575	0.1452692	0.1614253
220.602	2	0.653959	0.370295	0.1553045	0.1125761	0.1469979	0.1624886
220.702	2	0.637946	0.3702314	0.1547717	0.1115894	0.1473448	0.1618628
220.802	2	0.57161	0.3750038	0.1532881	0.111382	0.1490723	0.1626834
220.902	2	0.574629	0.3674654	0.1528775	0.1048774	0.1460077	0.1631219
221.002	2	0.57495	0.3704103	0.157668	0.1045206	0.145308	0.1629629
221.102	2	0.585624	0.3776772	0.1589234	0.1133781	0.145365	0.1624895
221.202	2	0.599115	0.3732102	0.1575366	0.1131784	0.147274	0.1620898
221.302	2	0.614888	0.3739383	0.1566086	0.1122319	0.149252	0.1621345
221.402	2	0.604745	0.3746393	0.1605484	0.1107725	0.1489	0.1636778
221.502	2	0.604113	0.370728	0.1600325	0.111534	0.148465	0.1643238
221.602	2	0.615142	0.3701264	0.1570162	0.1114153	0.149679	0.1644624
221.702	2	0.601832	0.3703485	0.1612871	0.1121706	0.149105	0.1647594
221.802	2	0.597156	0.3668568	0.1590732	0.1117851	0.148744	0.1643652
221.902	2	0.599212	0.3600466	0.156267	0.1128957	0.150142	0.1648574
222.002	2	0.600233	0.3624159	0.1585634	0.1107004	0.152682	0.164663
222.102	2	0.597871	0.3640798	0.1575948	0.1132664	0.151241	0.1630794
222.202	2	0.605956	0.3605763	0.1577413	0.1121138	0.154397	0.1631752
222.302	2	0.581805	0.365018	0.1624715	0.1140923	0.151103	0.1539423
222.402	2	0.590368	0.3621921	0.1654851	0.1089549	0.148045	0.1495237
222.502	2	0.602049	0.358041	0.1615874	0.1090651	0.14487	0.1541427
222.602	2	0.607627	0.3590664	0.1599888	0.1116614	0.146305	0.1473894
222.702	2	0.604944	0.3688907	0.1603191	0.1110968	0.14711	0.1438266
222.802	2	0.590961	0.3699055	0.1600541	0.109205	0.146165	0.1405829
222.902	2	0.591735	0.3575006	0.15856	0.1086754	0.147549	0.1451829
223.002	2	0.578058	0.351126	0.1591956	0.1078648	0.149477	0.1371872
223.102	2	0.615311	0.3533789	0.1585655	0.104496	0.15033	0.1346286
223.202	2	0.60457	0.3611308	0.1590544	0.1029186	0.153592	0.1399379
223.302	2	0.528624	0.365278	0.1620376	0.1062625	0.150833	0.1438132
223.402	2	0.539253	0.3623944	0.1644712	0.1042107	0.146686	0.1387679
223.502	2	0.605693	0.3749686	0.1603157	0.1044689	0.150368	0.1404117
223.602	2	0.629411	0.3634751	0.160662	0.1047694	0.150336	0.1456655
223.702	2	0.621959	0.362245	0.1598349	0.105009	0.146867	0.1344236
223.802	2	0.589183	0.365652	0.1587728	0.1077854	0.147117	0.1373675
223.902	2	0.55707	0.3767905	0.1648616	0.1073368	0.148015	0.1412893
224.002	2	0.579744	0.3691863	0.167308	0.1073212	0.147875	0.1341555
224.102	2	0.581482	0.3805287	0.1654154	0.1063657	0.146472	0.1416176
224.202	2	0.612004	0.3727292	0.16253	0.1061223	0.15238	0.1390111
224.302	2	0.62707	0.3655497	0.1612285	0.1060683	0.152512	0.1329606
224.402	2	0.624198	0.3698947	0.163094	0.1054768	0.15304	0.1383654
224.502	2	0.599738	0.3722402	0.1581693	0.1020364	0.150042	0.1397289
224.602	2	0.582926	0.3816976	0.1562167	0.1025906	0.150536	0.1422914
224.702	2	0.587702	0.3887866	0.1623012	0.1047047	0.150479	0.1365337
224.802	2	0.587349	0.3810959	0.1661353	0.1045486	0.15176	0.1355303
224.902	2	0.577046	0.3670836	0.1633048	0.104846	0.150397	0.1327264
225.002	2	0.583663	0.3717666	0.1626991	0.1060177	0.149342	0.1416123

225.102	2	0.586028	0.375937	0.1632961	0.1065791	0.149712	0.13874
225.202	2	0.587434	0.3741017	0.1653898	0.1080123	0.152169	0.1366423
225.302	2	0.579218	0.3709333	0.1625458	0.108818	0.154368	0.1426664
225.402	2	0.568737	0.3751405	0.1598432	0.1087982	0.153978	0.1453729
225.502	2	0.544703	0.3703919	0.1599292	0.1056579	0.151609	0.1329939
225.602	2	0.549693	0.3704298	0.1622886	0.1078446	0.152599	0.1465936
225.702	2	0.558561	0.3850474	0.1622227	0.1052794	0.154701	0.1450353
225.802	2	0.559016	0.3795554	0.1634294	0.1067277	0.154073	0.1477831
225.902	2	0.552431	0.3784201	0.1632403	0.107145	0.152896	0.141354
226.002	2	0.517226	0.3781626	0.1583791	0.1060961	0.153211	0.1373136
226.102	2	0.542316	0.370126	0.1595517	0.1070325	0.152949	0.1399768
226.202	2	0.562344	0.3791346	0.1630574	0.1087154	0.151456	0.1437646
226.302	2	0.578647	0.3756355	0.1637865	0.1080678	0.152344	0.1473819
226.402	2	0.561954	0.3737029	0.1584882	0.1092043	0.15514	0.1433007
226.502	2	0.543979	0.3730287	0.1619599	0.1084032	0.154553	0.1421623
226.602	2	0.567363	0.3735457	0.162365	0.1060979	0.154549	0.1380257
226.702	2	0.550353	0.3688087	0.1598251	0.1072511	0.153978	0.141771
226.802	2	0.573042	0.3702955	0.1621096	0.1078602	0.153776	0.1361829
226.902	2	0.567786	0.3677608	0.1697399	0.1103741	0.155113	0.1392711
227.002	2	0.546734	0.3742782	0.167197	0.1092046	0.156769	0.1397423
227.102	2	0.547665	0.3711213	0.162913	0.1111973	0.156882	0.1397524
227.202	2	0.544574	0.3725138	0.1659989	0.1105593	0.158078	0.140404
227.302	2	0.556257	0.3784503	0.1688538	0.1084154	0.158168	0.144133
227.402	2	0.578076	0.3781551	0.1634518	0.110471	0.160834	0.1465288
227.502	2	0.555445	0.3811322	0.1670539	0.1070133	0.158958	0.1485164
227.602	2	0.579119	0.3834327	0.165671	0.1056131	0.159605	0.160169
227.702	2	0.572339	0.3751335	0.1617846	0.1078773	0.160207	0.1626504
227.802	2	0.537578	0.3777352	0.1610475	0.1073845	0.164433	0.1569614
227.902	2	0.560968	0.3786481	0.1619753	0.1065935	0.162669	0.1581834
228.002	2	0.563434	0.3759783	0.1639351	0.1070481	0.160209	0.1626273
228.102	2	0.571199	0.3742598	0.1656841	0.105755	0.160451	0.1543558
228.202	2	0.579748	0.3731692	0.1628666	0.1056337	0.16196	0.1554344
228.302	2	0.602206	0.3800354	0.1649249	0.1071826	0.162079	0.1537439
228.402	2	0.589672	0.3729059	0.1655782	0.104103	0.162237	0.1502644
228.502	2	0.59307	0.3721332	0.1649071	0.1036238	0.16392	0.1565272
228.602	2	0.597498	0.3771301	0.1651938	0.106735	0.162285	0.1531525
228.702	2	0.616884	0.3893049	0.165529	0.1050415	0.162332	0.1515122
228.802	2	0.614249	0.378799	0.1646977	0.104732	0.161687	0.1509801
228.902	2	0.618991	0.3790386	0.1692898	0.1077108	0.162328	0.1520238
229.002	2	0.626692	0.3855109	0.1686679	0.106792	0.160356	0.1569846
229.102	2	0.64834	0.3734697	0.1676486	0.10773	0.160808	0.1500457
229.202	2	0.668008	0.3742758	0.1631153	0.1049216	0.16156	0.1474668
229.302	2	0.654685	0.3736361	0.1630429	0.1049457	0.161544	0.1525671
229.402	2	0.641692	0.3706804	0.1641267	0.1071455	0.162913	0.1446293
229.502	2	0.620869	0.3800958	0.1655426	0.1064116	0.163104	0.1472931
229.602	2	0.587653	0.3748522	0.1682073	0.1073861	0.16402	0.1474828
229.702	2	0.581336	0.377378	0.1689337	0.1058034	0.165087	0.1485825
229.802	2	0.60011	0.3756505	0.1672871	0.1066327	0.16511	0.1411059
229.902	2	0.644673	0.3640369	0.1707842	0.1093158	0.162361	0.1399884
230.002	2	0.667249	0.3672346	0.1789634	0.1068089	0.163971	0.1491229
230.102	2	0.652662	0.3733063	0.1793281	0.1082943	0.164298	0.1432243
230.202	2	0.628412	0.375144	0.1770304	0.1095054	0.166477	0.149317
230.302	2	0.589684	0.3671362	0.1756609	0.1088447	0.166106	0.1574823
230.402	2	0.564014	0.3725702	0.171189	0.1073492	0.166559	0.1569221
230.502	2	0.562514	0.372383	0.1729747	0.1079406	0.170621	0.155875
230.602	2	0.559227	0.3741946	0.1738688	0.1082979	0.169889	0.1525816
230.702	2	0.575371	0.3804587	0.1723608	0.1105302	0.168033	0.1538927
230.802	2	0.599917	0.3801929	0.1673901	0.1083775	0.166254	0.1506666
230.902	2	0.605589	0.3715127	0.1691924	0.1084809	0.167835	0.1445177
231.002	2	0.599403	0.3808255	0.1706844	0.1091809	0.169615	0.1373513
231.102	2	0.561853	0.3754461	0.1722803	0.108461	0.165345	0.141693
231.202	2	0.547321	0.3756108	0.173013	0.1081837	0.163083	0.1447538
231.302	2	0.539376	0.3695868	0.1734392	0.108931	0.161085	0.1375105
231.402	2	0.558907	0.3735275	0.1725873	0.1089948	0.160974	0.1378637
231.502	2	0.577992	0.3756651	0.1733422	0.1096009	0.162079	0.1409236
231.602	2	0.599319	0.37392	0.1726129	0.1077635	0.155739	0.1453909
231.702	2	0.6157	0.3785262	0.1780985	0.1070074	0.155222	0.1408579
231.802	2	0.59422	0.3727491	0.1811412	0.1077269	0.157239	0.1357506
231.902	2	0.593546	0.3808527	0.1775559	0.1088103	0.158206	0.1398564
232.002	2	0.612937	0.3874499	0.1763403	0.1093057	0.160313	0.1432735
232.102	2	0.598605	0.3809431	0.1737436	0.1056642	0.158192	0.1444045
232.202	2	0.571041	0.3757706	0.1681858	0.1014256	0.156948	0.145369
232.302	2	0.551747	0.3746818	0.1674788	0.1032743	0.155975	0.1496229
232.402	2	0.552979	0.3709137	0.1673835	0.1045661	0.157295	0.1605442
232.502	2	0.592172	0.3724059	0.1662376	0.1022532	0.158708	0.1562916
232.602	2	0.623252	0.3740629	0.1699237	0.1045482	0.157483	0.1591134
232.702	2	0.638537	0.3812258	0.1723718	0.1016695	0.157616	0.1571732
232.802	2	0.64926	0.3763985	0.1759616	0.105065	0.158457	0.1585594
232.902	2	0.643063	0.3702048	0.1783882	0.1057529	0.156688	0.1598033
233.002	2	0.641936	0.3681479	0.1781217	0.1016577	0.156366	0.1490452
233.102	2	0.643759	0.3632977	0.1792364	0.1014862	0.157094	0.15904
233.202	2	0.654151	0.3732834	0.1800548	0.1042988	0.155565	0.1544698
233.302	2	0.653271	0.3602943	0.1773324	0.1041508	0.153655	0.1448632
233.402	2	0.649297	0.3737206	0.1824935	0.1049116	0.15499	0.157266
233.502	2	0.657805	0.373078	0.1822143	0.1046427	0.153417	0.1570798
233.602	2	0.64715	0.3638086	0.1804145	0.1009125	0.155166	0.1563789
233.702	2	0.653884	0.3552885	0.1780907	0.1011991	0.155502	0.1614901
233.802	2	0.661563	0.3616267	0.1782752	0.1022274	0.157158	0.1610868
233.902	2	0.673071	0.3595167	0.1691541	0.1000951	0.160767	0.1563914
234.002	2	0.698209	0.3599445	0.1679683	0.09814953	0.157508	0.1553255
234.102	2	0.693838	0.363744	0.1701703	0.1007873	0.159472	0.1512076
234.202	2	0.69093	0.3493035	0.1670245	0.1030673	0.158706	0.1494088
234.302	2	0.689611	0.3533335	0.1679957	0.1014093	0.1585	0.1520272
234.402	2	0.67124	0.3515216	0.1728197	0.100722	0.16077	0.1518499
234.502	2	0.681096	0.3574127	0.1757806	0.1011047	0.161088	0.1528002

234.602	2	0.688744	0.3584264	0.1735857	0.1030791	0.159569	0.155821
234.702	2	0.662704	0.3673798	0.1709384	0.1008726	0.158134	0.1577793
234.802	2	0.645809	0.3672786	0.1758949	0.1036144	0.156393	0.1566135
234.902	2	0.653025	0.3557847	0.1816046	0.1033978	0.15718	0.1666305
235.002	2	0.663269	0.353058	0.1827007	0.1039389	0.162583	0.1541281
235.102	2	0.657283	0.364857	0.1807207	0.1034052	0.163045	0.1621562
235.202	2	0.64424	0.3635697	0.1744566	0.1020008	0.165016	0.170986
235.302	2	0.622913	0.3684211	0.1622663	0.1041053	0.16454	0.1780394
235.402	2	0.626309	0.3661948	0.1637059	0.1039311	0.165675	0.1860762
235.502	2	0.657416	0.361518	0.1632016	0.1051836	0.164256	0.1893914
235.602	2	0.623928	0.3683349	0.1633746	0.1027889	0.167729	0.1865581
235.702	2	0.628906	0.3690411	0.1620226	0.1029925	0.161688	0.1894039
235.802	2	0.62458	0.3745959	0.163534	0.1089567	0.162376	0.1869008
235.902	2	0.639852	0.3640123	0.1726545	0.1071851	0.164076	0.1903261
236.002	2	0.590175	0.3610854	0.1729857	0.102735	0.161328	0.18698
236.102	2	0.612092	0.3597926	0.1683883	0.1029981	0.161355	0.186552
236.202	2	0.641756	0.3655537	0.1663554	0.1043352	0.163504	0.1832853
236.302	2	0.665233	0.3643363	0.1659971	0.102644	0.164363	0.1839634
236.402	2	0.657784	0.3632249	0.1678539	0.1031157	0.165314	0.1830683
236.502	2	0.665677	0.3679489	0.1706587	0.1077641	0.167829	0.185355
236.602	2	0.671932	0.3656925	0.1684728	0.1093064	0.163137	0.1822153
236.702	2	0.676678	0.3659401	0.166416	0.1076972	0.15836	0.1836037
236.802	2	0.661868	0.3598909	0.16855	0.1008675	0.158305	0.181985
236.902	2	0.629836	0.3621208	0.1695952	0.1031908	0.155969	0.1786279
237.002	2	0.627052	0.340032	0.1666151	0.1073387	0.156943	0.1772119
237.102	2	0.631925	0.3574007	0.1736427	0.1083743	0.160169	0.1785088
237.202	2	0.636126	0.3613398	0.1689167	0.1053036	0.15981	0.1792925
237.302	2	0.646545	0.3697052	0.1675044	0.1055609	0.159494	0.1797032
237.402	2	0.668221	0.3594463	0.1657018	0.1051929	0.164162	0.1797317
237.502	2	0.660367	0.3477859	0.1678282	0.1064453	0.163971	0.1814802
237.602	2	0.660362	0.3512619	0.1688943	0.1043223	0.163654	0.1822288
237.702	2	0.669495	0.3490324	0.1620527	0.1052688	0.157581	0.179437
237.802	2	0.679575	0.3512736	0.1597997	0.1050887	0.155399	0.1799538
237.902	2	0.661524	0.3480142	0.1608223	0.1039106	0.155582	0.1786243
238.002	2	0.650709	0.3462359	0.1645995	0.1014986	0.152085	0.1790295
238.102	2	0.645849	0.3434417	0.1693094	0.1014211	0.151746	0.1800504
238.202	2	0.58994	0.3440482	0.1653923	0.100795	0.152096	0.179052
238.302	2	0.597026	0.3470225	0.1700333	0.1018195	0.153902	0.1785013
238.402	2	0.626457	0.34987	0.1680646	0.103632	0.151189	0.1776963
238.502	2	0.636874	0.3536416	0.153986	0.1023879	0.151812	0.1757285
238.602	2	0.637604	0.355443	0.1578756	0.103049	0.148995	0.1759233
238.702	2	0.674866	0.3557764	0.1650768	0.1065738	0.147722	0.1740524
238.802	2	0.690244	0.3549702	0.1740344	0.1058188	0.152493	0.1766064
238.902	2	0.68563	0.3614692	0.1708506	0.1049161	0.150672	0.1764433
239.002	2	0.648892	0.3626939	0.1612979	0.09669448	0.15147	0.1765036
239.102	2	0.651256	0.3637798	0.1590952	0.09603779	0.154647	0.1716319
239.202	2	0.645954	0.3570114	0.1659595	0.1015114	0.154345	0.174428
239.302	2	0.668789	0.351739	0.1667685	0.104002	0.156035	0.1755272
239.402	2	0.65729	0.3497881	0.1652933	0.1036978	0.156176	0.1744694
239.502	2	0.648213	0.3568528	0.1669202	0.1014526	0.156338	0.1753123
239.602	2	0.650601	0.3639421	0.1661207	0.1040228	0.152242	0.174348
239.702	2	0.637796	0.3597056	0.1635701	0.1066823	0.149399	0.1753451
239.802	2	0.629007	0.3662002	0.1645127	0.1050124	0.147603	0.175504
239.921	2.5	0.641346	0.3461719	0.1664047	0.1059405	0.146147	0.1747018
240.021	2.5	0.604985	0.3550109	0.1598412	0.1052185	0.145803	0.1747303
240.121	2.5	0.599165	0.3618345	0.1584427	0.1070998	0.147958	0.1739058
240.221	2.5	0.611736	0.3607948	0.1712717	0.103343	0.150085	0.1736892
240.321	2.5	0.593511	0.3525104	0.1672653	0.1069949	0.153067	0.173988
240.421	2.5	0.584963	0.3560288	0.1596892	0.1078851	0.150159	0.17405
240.521	2.5	0.571522	0.3639339	0.1591924	0.1101177	0.147755	0.1728907
240.621	2.5	0.604997	0.3670143	0.1597428	0.1046409	0.147287	0.1723073
240.721	2.5	0.608817	0.3632525	0.1585195	0.09752938	0.146628	0.1686703
240.821	2.5	0.61358	0.3560113	0.1517824	0.1081216	0.143698	0.1724037
240.921	2.5	0.615935	0.3559931	0.151629	0.1099196	0.130793	0.1687095
241.021	2.5	0.626063	0.3596303	0.1512214	0.1115364	0.13008	0.1696869
241.121	2.5	0.618588	0.358917	0.1419852	0.1110446	0.138275	0.1691913
241.221	2.5	0.60645	0.3649944	0.1435773	0.1116312	0.136285	0.1687346
241.321	2.5	0.593777	0.360706	0.1471808	0.110446	0.13728	0.1646189
241.421	2.5	0.613814	0.3563504	0.1459482	0.100603	0.132909	0.1668364
241.521	2.5	0.607529	0.372631	0.1485535	0.09725647	0.130175	0.1695874
241.621	2.5	0.623581	0.3725085	0.1488834	0.104368	0.134578	0.1694373
241.721	2.5	0.614055	0.3682061	0.1488316	0.1091012	0.139214	0.1695004
241.821	2.5	0.62729	0.3602255	0.1566505	0.1111113	0.137881	0.1665501
241.921	2.5	0.642755	0.3577852	0.149697	0.1111527	0.138043	0.1678571
242.021	2.5	0.674903	0.3637237	0.1494987	0.1119244	0.136515	0.1674954
242.121	2.5	0.652091	0.367312	0.1526503	0.1112807	0.138607	0.164687
242.221	2.5	0.62181	0.3584845	0.1518893	0.1088651	0.14084	0.1633591
242.321	2.5	0.623793	0.3624165	0.1498782	0.1098733	0.139163	0.1657495
242.421	2.5	0.604938	0.3681387	0.150064	0.1058165	0.137095	0.1662649
242.521	2.5	0.602088	0.3643342	0.1522909	0.1004787	0.135717	0.1630529
242.621	2.5	0.573274	0.3605322	0.1532797	0.09920043	0.136752	0.1622433
242.721	2.5	0.574694	0.3615678	0.1516486	0.09840961	0.135764	0.166919
242.821	2.5	0.582965	0.36121	0.1505078	0.1033169	0.137531	0.1645894
242.921	2.5	0.56309	0.3581451	0.1511454	0.1017039	0.139662	0.1643241
243.021	2.5	0.527333	0.3599234	0.1527567	0.1039119	0.140306	0.1631147
243.121	2.5	0.558009	0.3697532	0.1533146	0.1076975	0.140708	0.1655731
243.221	2.5	0.611333	0.3679051	0.1524735	0.1047605	0.137777	0.1656405
243.321	2.5	0.661343	0.3720068	0.1543193	0.1042931	0.139315	0.1646011
243.421	2.5	0.681425	0.3648403	0.1583763	0.103516	0.137533	0.1658774
243.521	2.5	0.688868	0.3644492	0.1536329	0.105616	0.138311	0.1647177
243.621	2.5	0.640378	0.3764709	0.151394	0.1043931	0.139315	0.1656826
243.721	2.5	0.604724	0.3676111	0.1534033	0.1020241	0.140777	0.1662536
243.821	2.5	0.603857	0.3657111	0.155558	0.1028253	0.141647	0.1640061
243.921	2.5	0.577401	0.3673702	0.1520503	0.1030972	0.140105	0.1642126
244.021	2.5	0.576587	0.3701703	0.15165	0.1067391	0.139733	0.1631089

244.121	2.5	0.577794	0.3680037	0.1508509	0.1056102	0.138598	0.1639013
244.221	2.5	0.593354	0.3660401	0.1517164	0.1044616	0.138784	0.1640577
244.321	2.5	0.584742	0.3686753	0.1515751	0.1023603	0.138169	0.1639927
244.421	2.5	0.601567	0.3645477	0.1505463	0.1061583	0.137239	0.1620997
244.521	2.5	0.619721	0.3627448	0.15012	0.1023257	0.139335	0.1630565
244.621	2.5	0.626645	0.360007	0.1540641	0.09810405	0.139937	0.1669326
244.721	2.5	0.620778	0.3628753	0.1509462	0.1053712	0.14001	0.167022
244.821	2.5	0.654526	0.3564036	0.1505156	0.1056475	0.140208	0.1663061
244.921	2.5	0.677581	0.3579872	0.1521225	0.1049612	0.140976	0.164829
245.021	2.5	0.692975	0.3581966	0.1530757	0.1087851	0.142144	0.1658
245.121	2.5	0.687967	0.3472745	0.1533688	0.1073918	0.141045	0.1647709
245.221	2.5	0.657279	0.353997	0.1516419	0.1096048	0.141178	0.1635786
245.321	2.5	0.653069	0.3531097	0.1499995	0.1090553	0.140821	0.1640096
245.421	2.5	0.657206	0.3560161	0.1533104	0.1087863	0.140615	0.1653201
245.521	2.5	0.642751	0.3480561	0.1562194	0.1088185	0.141549	0.1627483
245.621	2.5	0.663128	0.3615227	0.1578592	0.1081948	0.13988	0.1652164
245.721	2.5	0.635853	0.3562082	0.1497353	0.1073398	0.13884	0.1670597
245.821	2.5	0.622234	0.3619788	0.1501563	0.1012898	0.139162	0.1647933
245.921	2.5	0.638389	0.3648447	0.1536119	0.1003713	0.140529	0.1649318
246.021	2.5	0.623175	0.3601612	0.1559052	0.1099793	0.140963	0.1647052
246.121	2.5	0.62966	0.3552108	0.1541197	0.1072132	0.141088	0.1651266
246.221	2.5	0.610672	0.3582428	0.1541578	0.1072097	0.140169	0.1634433
246.321	2.5	0.589433	0.3538081	0.1544706	0.112074	0.141676	0.1653313
246.421	2.5	0.592593	0.3554546	0.1524118	0.108886	0.140407	0.1647984
246.521	2.5	0.588149	0.3525935	0.1551941	0.1043904	0.140373	0.1668297
246.621	2.5	0.615012	0.3548639	0.1556424	0.111764	0.140828	0.1636241
246.721	2.5	0.601969	0.3566813	0.1574109	0.1083782	0.140715	0.1632853
246.821	2.5	0.582802	0.3612333	0.1576198	0.1119754	0.140325	0.1651517
246.921	2.5	0.571737	0.3665021	0.151602	0.1102492	0.139118	0.1637898
247.021	2.5	0.589744	0.3644079	0.1547595	0.1138758	0.139011	0.1620603
247.121	2.5	0.614139	0.363901	0.1515361	0.1095377	0.140878	0.1657242
247.221	2.5	0.607351	0.3672756	0.1523129	0.1080113	0.141398	0.1638299
247.321	2.5	0.594346	0.3610296	0.1521026	0.109765	0.142962	0.1629
247.421	2.5	0.582713	0.3558792	0.1536115	0.1068685	0.142734	0.1644282
247.521	2.5	0.587169	0.3600343	0.1541461	0.1064479	0.140615	0.1672606
247.621	2.5	0.60665	0.3579286	0.1498224	0.1052918	0.142486	0.1677641
247.721	2.5	0.602522	0.3602087	0.1499027	0.1040002	0.141341	0.16639
247.821	2.5	0.591292	0.361312	0.1473559	0.1003532	0.141563	0.1653515
247.921	2.5	0.546067	0.3607772	0.146911	0.1078613	0.140696	0.1651751
248.021	2.5	0.547605	0.3642116	0.1462958	0.1066249	0.14019	0.1650677
248.121	2.5	0.55375	0.363472	0.1447814	0.1043918	0.137856	0.1648172
248.221	2.5	0.562131	0.3567205	0.1438871	0.1014132	0.137484	0.1632705
248.321	2.5	0.566294	0.357728	0.1482433	0.1040567	0.138907	0.165168
248.421	2.5	0.558726	0.3600124	0.1519628	0.1052272	0.130153	0.1658598
248.521	2.5	0.541702	0.358346	0.1494273	0.1048751	0.118821	0.1619662
248.621	2.5	0.547266	0.3553494	0.1470973	0.1029929	0.137749	0.1624776
248.721	2.5	0.546272	0.3649203	0.1434016	0.1062174	0.135631	0.151869
248.821	2.5	0.539127	0.3611782	0.14773	0.1086404	0.13138	0.1570767
248.921	2.5	0.539167	0.3585832	0.151954	0.1090449	0.129293	0.1543702
249.021	2.5	0.552566	0.3588345	0.1517314	0.1063458	0.1299	0.1470215
249.121	2.5	0.552356	0.3611175	0.1509908	0.1075904	0.130065	0.1455499
249.221	2.5	0.522797	0.3561451	0.1545344	0.1060696	0.129347	0.1496018
249.321	2.5	0.549349	0.3519088	0.1514427	0.1041473	0.130137	0.14094
249.421	2.5	0.536228	0.354896	0.1516874	0.1046975	0.127597	0.1462221
249.521	2.5	0.529216	0.3543411	0.1501477	0.1077555	0.129031	0.1493511
249.621	2.5	0.532072	0.3534052	0.150478	0.1097822	0.131417	0.1486353
249.721	2.5	0.551601	0.3524247	0.1500396	0.1086193	0.12865	0.152635
249.821	2.5	0.551181	0.3515667	0.1496858	0.1049518	0.130019	0.1520555
249.921	2.5	0.54626	0.3493611	0.1539032	0.1078968	0.129532	0.1599159
250.021	2.5	0.568145	0.3568393	0.1526561	0.108947	0.12678	0.1358373
250.121	2.5	0.588738	0.3486382	0.1481947	0.1059768	0.126151	0.1394338
250.221	2.5	0.59834	0.3432072	0.1517331	0.1048904	0.127029	0.1364941
250.321	2.5	0.587537	0.3504309	0.1520228	0.104899	0.131411	0.1337951
250.421	2.5	0.543992	0.3506209	0.1516383	0.1006341	0.130877	0.1341279
250.521	2.5	0.563069	0.3543824	0.1524408	0.1013048	0.127771	0.1370206
250.621	2.5	0.57205	0.3616029	0.1528226	0.1021392	0.127085	0.1335738
250.721	2.5	0.584923	0.3549866	0.1563579	0.10392	0.126909	0.1339638
250.821	2.5	0.57149	0.3541539	0.1553012	0.1036351	0.130384	0.1354265
250.921	2.5	0.56383	0.3640977	0.152707	0.1032673	0.127889	0.1339923
251.021	2.5	0.567193	0.3578257	0.1495443	0.1033267	0.128543	0.1339688
251.121	2.5	0.55235	0.3674709	0.152585	0.1061862	0.128778	0.1409631
251.221	2.5	0.536514	0.3590823	0.1607231	0.1041845	0.130167	0.1308948
251.321	2.5	0.547939	0.3586302	0.158766	0.1039878	0.131782	0.1426151
251.421	2.5	0.56245	0.3573065	0.1539961	0.1022991	0.133457	0.1445674
251.521	2.5	0.555086	0.3707387	0.1540245	0.09941795	0.131457	0.1453755
251.621	2.5	0.544361	0.373242	0.1550625	0.1032797	0.136353	0.1379697
251.721	2.5	0.543604	0.3591285	0.1477088	0.1029429	0.1338	0.1383152
251.821	2.5	0.549744	0.3617739	0.1504786	0.1017349	0.130753	0.1370001
251.921	2.5	0.557095	0.3649799	0.1575163	0.1036843	0.133773	0.1441269
252.021	2.5	0.540988	0.3632748	0.1561871	0.1047248	0.137298	0.1414908
252.121	2.5	0.522764	0.3690825	0.1598102	0.1059281	0.136007	0.1375389
252.221	2.5	0.560511	0.3624895	0.1611384	0.1052306	0.138736	0.1400385
252.321	2.5	0.57004	0.3639534	0.1553043	0.1063684	0.140985	0.134238
252.421	2.5	0.553243	0.3671962	0.1509552	0.1040757	0.13566	0.1361438
252.521	2.5	0.564279	0.3642719	0.1548706	0.1025546	0.133764	0.1361812
252.621	2.5	0.558932	0.369198	0.1592403	0.1041745	0.137537	0.1372127
252.721	2.5	0.571206	0.3629673	0.161089	0.1037259	0.137102	0.1435714
252.821	2.5	0.559938	0.3655246	0.1590372	0.1079433	0.138132	0.1467637
252.921	2.5	0.567474	0.360768	0.1541699	0.1082001	0.138199	0.1608611
253.021	2.5	0.562023	0.362765	0.1586262	0.1102693	0.138692	0.1637745
253.121	2.5	0.562922	0.361557	0.1589216	0.1069477	0.135725	0.1590661
253.221	2.5	0.571126	0.3585601	0.1571408	0.1055762	0.135879	0.161986
253.321	2.5	0.569124	0.3575701	0.161264	0.1081306	0.136004	0.1535547
253.421	2.5	0.577312	0.3619382	0.1559425	0.1094351	0.135377	0.1559096
253.521	2.5	0.5465	0.3612455	0.1564964	0.110993	0.137077	0.1521552

253.621	2.5	0.571308	0.3642226	0.163115	0.1084809	0.136196	0.1582544
253.721	2.5	0.559466	0.3676318	0.161079	0.1080158	0.133806	0.1568209
253.821	2.5	0.581617	0.3670796	0.1606572	0.1090259	0.134356	0.1500612
253.921	2.5	0.580339	0.3724717	0.1608805	0.1092106	0.134683	0.1533275
254.021	2.5	0.584705	0.3687752	0.1600876	0.1074731	0.137364	0.1535696
254.121	2.5	0.587747	0.3672827	0.1644643	0.1078357	0.136474	0.1507457
254.221	2.5	0.581788	0.3667937	0.1602384	0.1089745	0.139446	0.152564
254.321	2.5	0.592186	0.3630213	0.1580124	0.1100992	0.139532	0.1521713
254.421	2.5	0.579704	0.3624115	0.159791	0.1078249	0.138354	0.1466296
254.521	2.5	0.59409	0.3642138	0.162771	0.1082681	0.13751	0.1475912
254.621	2.5	0.62455	0.363566	0.1578866	0.1044319	0.138344	0.1464785
254.721	2.5	0.60571	0.3653487	0.1593925	0.1074957	0.140735	0.1422044
254.821	2.5	0.583853	0.375292	0.1618407	0.1070711	0.139925	0.1466915
254.921	2.5	0.537036	0.3665701	0.1609644	0.1062805	0.139633	0.1403103
255.021	2.5	0.545502	0.3737303	0.1601206	0.1076865	0.139604	0.1475922
255.121	2.5	0.551639	0.3670408	0.1584222	0.1071016	0.137954	0.1529146
255.221	2.5	0.546513	0.3596232	0.1660866	0.1061958	0.141385	0.1520903
255.321	2.5	0.563481	0.3614171	0.167412	0.1067428	0.141799	0.1544975
255.421	2.5	0.545074	0.3626117	0.1618168	0.1065745	0.142672	0.1474727
255.521	2.5	0.543087	0.3609399	0.1583623	0.1061781	0.14393	0.1418299
255.621	2.5	0.542692	0.3679202	0.1580807	0.1062783	0.144331	0.1361074
255.721	2.5	0.540463	0.3612676	0.1646477	0.1071651	0.14561	0.1431257
255.821	2.5	0.554464	0.3588043	0.1681239	0.1060898	0.145642	0.1428836
255.921	2.5	0.575732	0.360108	0.1658159	0.1064024	0.142251	0.136997
256.021	2.5	0.557541	0.3616112	0.1738923	0.1065189	0.144294	0.1406273
256.121	2.5	0.527848	0.3585692	0.1815774	0.1101445	0.144118	0.1403527
256.221	2.5	0.523698	0.3619094	0.1763243	0.107656	0.145429	0.135117
256.321	2.5	0.525215	0.3605659	0.1704399	0.1097553	0.147677	0.1357459
256.421	2.5	0.536202	0.3663933	0.1646621	0.1121873	0.148398	0.1361069
256.521	2.5	0.545677	0.3633813	0.1671258	0.1122763	0.146108	0.1404406
256.621	2.5	0.545645	0.3662777	0.1664143	0.1119648	0.148072	0.1438725
256.721	2.5	0.547277	0.3627993	0.164123	0.1107958	0.147085	0.1525492
256.821	2.5	0.522146	0.3623548	0.1663409	0.1128951	0.149344	0.1544772
256.921	2.5	0.499579	0.3611395	0.1696771	0.1130653	0.150616	0.1615341
257.021	2.5	0.49064	0.3687095	0.1714878	0.1124109	0.153501	0.1568444
257.121	2.5	0.523901	0.3645871	0.1702173	0.1099747	0.154564	0.1557747
257.221	2.5	0.555461	0.373271	0.1651549	0.105725	0.154117	0.1461824
257.321	2.5	0.571783	0.3624399	0.165204	0.1079505	0.154391	0.1547753
257.421	2.5	0.576715	0.3748923	0.1697517	0.1067021	0.157086	0.147472
257.521	2.5	0.55955	0.3717922	0.1714345	0.1039701	0.155497	0.1499899
257.621	2.5	0.592378	0.3681865	0.1720666	0.09908554	0.156434	0.1536317
257.721	2.5	0.578674	0.3621432	0.1689548	0.1017877	0.158548	0.1543753
257.821	2.5	0.580674	0.3608769	0.1625094	0.10359	0.157177	0.1616886
257.921	2.5	0.587329	0.3625486	0.1631939	0.1010692	0.153232	0.15615
258.021	2.5	0.551573	0.367334	0.1619062	0.09895348	0.152964	0.1551565
258.121	2.5	0.555128	0.360425	0.1638348	0.1035591	0.1547	0.1550685
258.221	2.5	0.568397	0.3583408	0.1696887	0.1011011	0.156241	0.1508723
258.321	2.5	0.588389	0.3524915	0.1766113	0.09626226	0.156965	0.1556572
258.421	2.5	0.599243	0.3595966	0.1770084	0.09821073	0.158482	0.1513268
258.521	2.5	0.596357	0.3572737	0.1747902	0.101843	0.159736	0.153464
258.621	2.5	0.610509	0.3631578	0.1744998	0.104921	0.15855	0.1538891
258.721	2.5	0.561783	0.355262	0.1763558	0.1039107	0.159877	0.1606661
258.821	2.5	0.521891	0.3507834	0.1809987	0.1010313	0.160708	0.158603
258.921	2.5	0.52175	0.3566661	0.1773065	0.1022161	0.158478	0.161203
259.021	2.5	0.498054	0.3578793	0.1761268	0.09819541	0.158761	0.1705012
259.121	2.5	0.507937	0.354387	0.1700964	0.09716937	0.159951	0.1770867
259.221	2.5	0.533966	0.348098	0.1633952	0.1022994	0.160595	0.1863336
259.321	2.5	0.517021	0.3504594	0.1629618	0.1037007	0.161014	0.1845791
259.421	2.5	0.518143	0.3571549	0.1625773	0.1007447	0.162575	0.1868632
259.521	2.5	0.533178	0.3543963	0.1642493	0.09974495	0.162825	0.1862932
259.621	2.5	0.555815	0.3632442	0.1707085	0.09696316	0.164938	0.1865681
259.721	2.5	0.565827	0.3554085	0.1644867	0.0986919	0.166881	0.1840795
259.821	2.5	0.565468	0.3502676	0.1617204	0.1025967	0.166349	0.1793167
259.921	2.5	0.589463	0.3572257	0.1698692	0.1027596	0.165996	0.1808489
260.021	2.5	0.579698	0.356072	0.1695808	0.09970651	0.167371	0.1815597
260.121	2.5	0.61105	0.3630481	0.170533	0.1026512	0.165446	0.1800004
260.221	2.5	0.633095	0.3493597	0.1548688	0.1022187	0.168019	0.1814474
260.321	2.5	0.646292	0.3568841	0.1566098	0.1028115	0.165268	0.1776517
260.421	2.5	0.634989	0.3553319	0.1577678	0.1004817	0.16966	0.1730251
260.521	2.5	0.649472	0.3496004	0.155018	0.1048297	0.176313	0.1752607
260.621	2.5	0.647412	0.351359	0.1602721	0.1015178	0.170825	0.1768926
260.721	2.5	0.655341	0.3599884	0.1625695	0.09880885	0.17099	0.1769436
260.821	2.5	0.661631	0.3569903	0.1649199	0.1011909	0.174209	0.1783252
260.921	2.5	0.667277	0.3553287	0.1632188	0.1019255	0.175102	0.1788083
261.021	2.5	0.664805	0.3559623	0.1623993	0.1013845	0.17515	0.174365
261.121	2.5	0.671719	0.3636508	0.1621455	0.1035279	0.170928	0.1770369
261.221	2.5	0.658908	0.3567146	0.1625125	0.1039783	0.165919	0.1756103
261.321	2.5	0.666961	0.3543511	0.1627223	0.0993879	0.164934	0.175942
261.421	2.5	0.626	0.3320191	0.1660314	0.1007183	0.165155	0.1754245
261.521	2.5	0.588951	0.3411423	0.1671745	0.1041319	0.168091	0.1746377
261.621	2.5	0.606739	0.3570289	0.1632809	0.1044374	0.158757	0.1743683
261.721	2.5	0.646001	0.3523482	0.1683754	0.1007595	0.152333	0.171298
261.821	2.5	0.645535	0.3404732	0.1627338	0.1016257	0.153448	0.169667
261.921	2.5	0.636865	0.3403188	0.1602644	0.1007527	0.155272	0.171505
262.021	2.5	0.589057	0.3462902	0.1623779	0.09852265	0.158148	0.1721807
262.121	2.5	0.616673	0.3426708	0.16368	0.100382	0.156043	0.1699164
262.221	2.5	0.658097	0.334761	0.1569691	0.09988021	0.158207	0.1700891
262.321	2.5	0.646198	0.3359238	0.1579964	0.09756881	0.156673	0.1695302
262.421	2.5	0.598956	0.338205	0.1625464	0.09798235	0.158133	0.1679986
262.521	2.5	0.589829	0.342806	0.1658457	0.09713778	0.160058	0.1688003
262.621	2.5	0.582987	0.3495782	0.1675219	0.1000598	0.161414	0.1694798
262.721	2.5	0.580574	0.3476614	0.1652054	0.09737159	0.161698	0.1708373
262.821	2.5	0.597307	0.3462107	0.1468114	0.09930281	0.159782	0.1688818
262.921	2.5	0.607496	0.3492098	0.1566847	0.1006991	0.158065	0.1696935
263.021	2.5	0.601859	0.3531043	0.1689142	0.1002776	0.158318	0.1700711

263.121	2.5	0.640096	0.3524521	0.1666163	0.09194252	0.158566	0.1689462
263.221	2.5	0.65812	0.3423882	0.157048	0.09221183	0.157473	0.1690122
263.321	2.5	0.667174	0.3447972	0.1585	0.09928754	0.156498	0.1681275
263.421	2.5	0.652673	0.3509822	0.1634652	0.09665643	0.154682	0.1658601
263.521	2.5	0.673153	0.3540914	0.1620565	0.09709083	0.15261	0.1662647
263.621	2.5	0.641485	0.355495	0.1656806	0.09911882	0.153136	0.1706795
263.721	2.5	0.625719	0.3578474	0.1650325	0.1022079	0.153533	0.1684345
263.821	2.5	0.644587	0.3501909	0.1623844	0.09986764	0.155568	0.1682508
263.921	2.5	0.663207	0.3488934	0.165732	0.1022214	0.160048	0.1670229
264.021	2.5	0.683599	0.3569524	0.1574457	0.1014514	0.156707	0.1689698
264.121	2.5	0.668532	0.3568543	0.1631916	0.0893582	0.160897	0.1705781
264.221	2.5	0.640891	0.3496345	0.1741224	0.08983263	0.160427	0.1727063
264.321	2.5	0.610085	0.3443963	0.1611906	0.09964553	0.15637	0.1695128
264.421	2.5	0.633797	0.3547965	0.1604674	0.1017452	0.158571	0.1727038
264.521	2.5	0.643981	0.3598513	0.1622234	0.1026852	0.155856	0.170132
264.621	2.5	0.645039	0.3603011	0.157529	0.1029941	0.154516	0.1717659
264.721	2.5	0.656462	0.3531039	0.1393834	0.09603596	0.153719	0.1650248
264.821	2.5	0.672634	0.3498027	0.1311299	0.09799703	0.153666	0.1625933
264.921	2.5	0.69193	0.3498254	0.1443856	0.102786	0.154552	0.1661469
265.021	2.5	0.696023	0.3515931	0.1530057	0.1041283	0.160413	0.1651505
265.121	2.5	0.683415	0.3583966	0.145647	0.1047364	0.159847	0.1665352
265.221	2.5	0.617113	0.3569086	0.1489186	0.1058431	0.160846	0.1644605
265.321	2.5	0.608635	0.3470463	0.1528998	0.1053242	0.159398	0.1617154
265.421	2.5	0.644118	0.3537175	0.152942	0.1022574	0.15879	0.1608701
265.521	2.5	0.621919	0.3654862	0.1538158	0.09170637	0.16043	0.162532
265.621	2.5	0.63612	0.3690285	0.1553098	0.09438108	0.167559	0.1636414
265.721	2.5	0.614587	0.3546617	0.1567469	0.102648	0.160013	0.1635492
265.821	2.5	0.616267	0.350909	0.1712192	0.1007852	0.159386	0.1612064
265.921	2.5	0.653445	0.3559214	0.1639649	0.102453	0.161693	0.1601667
266.021	2.5	0.667039	0.3624896	0.1604653	0.1028245	0.157729	0.1618523
266.121	2.5	0.670713	0.360051	0.1591701	0.1053539	0.156802	0.1593554
266.221	2.5	0.622383	0.3553399	0.152843	0.1054769	0.158954	0.1574209
266.321	2.5	0.630828	0.3560368	0.1506439	0.1030788	0.160461	0.1591185
266.421	2.5	0.630228	0.3589934	0.1529011	0.1021456	0.163141	0.1590619
266.521	2.5	0.584997	0.3604988	0.1538416	0.0978414	0.164875	0.1580308
266.621	2.5	0.554338	0.3588776	0.1571885	0.09036927	0.164608	0.1557149
266.721	2.5	0.568054	0.3578177	0.1578627	0.0926748	0.158405	0.1584074
266.821	2.5	0.556352	0.3545237	0.1545285	0.09390276	0.153104	0.1567225
266.921	2.5	0.559052	0.3558519	0.1543457	0.0946741	0.149612	0.1583361
267.021	2.5	0.599184	0.365029	0.1537976	0.09716187	0.152712	0.1567482
267.121	2.5	0.60912	0.3637443	0.1597017	0.1004504	0.158423	0.1583504
267.221	2.5	0.615718	0.366663	0.1553508	0.1008329	0.157386	0.1593823
267.321	2.5	0.611306	0.361064	0.1590397	0.1016612	0.156263	0.1585283
267.421	2.5	0.620339	0.3609567	0.1660689	0.09941571	0.158376	0.1593326
267.521	2.5	0.566419	0.3703721	0.1607276	0.1002452	0.160193	0.1590576
267.621	2.5	0.552355	0.3672626	0.1486658	0.1023025	0.161056	0.1600357
267.721	2.5	0.523772	0.3605025	0.1549954	0.09884321	0.15625	0.1612787
267.821	2.5	0.527108	0.3623067	0.1592512	0.09964298	0.154221	0.1578135
267.921	2.5	0.502052	0.3632463	0.1544012	0.09993175	0.153811	0.1591295
268.021	2.5	0.505701	0.3676336	0.1542263	0.1032959	0.148575	0.1594531
268.121	2.5	0.505711	0.3588458	0.1564212	0.103663	0.148984	0.1589687
268.221	2.5	0.492857	0.3629699	0.1585168	0.1033134	0.149366	0.1572208
268.321	2.5	0.52377	0.3625457	0.1606187	0.1021853	0.150053	0.15691
268.421	2.5	0.587433	0.3628764	0.1572352	0.1020888	0.151048	0.1569591
268.521	2.5	0.603072	0.360577	0.1541497	0.1011415	0.153292	0.1591157
268.621	2.5	0.615814	0.3639682	0.1564374	0.09371449	0.144778	0.1603089
268.721	2.5	0.621208	0.3603975	0.1590527	0.09851593	0.142991	0.161761
268.821	2.5	0.642478	0.3577198	0.1526071	0.1009847	0.145759	0.1602798
268.921	2.5	0.625311	0.3605651	0.1532128	0.09922134	0.14661	0.1588664
269.021	2.5	0.610284	0.3635437	0.1560093	0.1030477	0.150268	0.1572523
269.121	2.5	0.597464	0.3582023	0.1589032	0.1046001	0.154705	0.1574095
269.221	2.5	0.567322	0.3683648	0.1608946	0.1048594	0.157062	0.158064
269.321	2.5	0.579987	0.3676452	0.1584591	0.1085355	0.159283	0.1576669
269.421	2.5	0.558566	0.3634485	0.153712	0.1079359	0.157218	0.1585766
269.521	2.5	0.566586	0.361425	0.155382	0.1103121	0.158216	0.1555391
269.621	2.5	0.582196	0.3738985	0.163376	0.1057824	0.152071	0.1572285
269.721	2.5	0.557229	0.3640419	0.1650849	0.105095	0.148758	0.1589264
269.821	2.5	0.572239	0.3648098	0.1552197	0.1017972	0.148522	0.1571737
269.921	2.5	0.572163	0.3665588	0.1574784	0.09862772	0.145118	0.1573474
270.021	2.5	0.554923	0.3607265	0.1607622	0.1075489	0.143772	0.1574194
270.121	2.5	0.543547	0.354826	0.161008	0.1063112	0.145032	0.160462
270.221	2.5	0.536979	0.358867	0.1581495	0.1020046	0.147	0.1582629
270.321	2.5	0.547835	0.3466325	0.1610741	0.10249	0.15067	0.1607023
270.421	2.5	0.533577	0.3509933	0.1592476	0.104228	0.148301	0.1607234
270.521	2.5	0.548303	0.3474633	0.1596242	0.09849007	0.149031	0.160804
270.621	2.5	0.533659	0.3558792	0.1654362	0.107429	0.147679	0.1602886
270.721	2.5	0.547463	0.3582384	0.1669033	0.101358	0.149218	0.1623297
270.821	2.5	0.548028	0.3630698	0.1725598	0.1059915	0.147952	0.1620999
270.921	2.5	0.541267	0.3671097	0.1655124	0.103357	0.135666	0.1593248
271.021	2.5	0.546674	0.3646373	0.1588877	0.1040967	0.137359	0.1589672
271.121	2.5	0.544843	0.365844	0.1589243	0.1005928	0.133018	0.1620247
271.221	2.5	0.533465	0.3682143	0.1565833	0.1004083	0.131237	0.1607543
271.321	2.5	0.534694	0.3611003	0.1567203	0.1021484	0.138764	0.1600515
271.421	2.5	0.552747	0.3564694	0.1536202	0.1027176	0.13945	0.1590607
271.521	2.5	0.547293	0.3593875	0.1560198	0.1011704	0.139227	0.1594677
271.621	2.5	0.578384	0.3617322	0.1537778	0.1037538	0.1374	0.1591327
271.721	2.5	0.559879	0.3629854	0.1536695	0.09875709	0.139806	0.1611847
271.821	2.5	0.542834	0.3605813	0.1548327	0.09468357	0.140568	0.1606418
271.921	2.5	0.526334	0.3622267	0.1505423	0.1001194	0.140049	0.1599388
272.021	2.5	0.522318	0.3679538	0.1515893	0.1004053	0.137947	0.1598766
272.121	2.5	0.526723	0.3683183	0.1510402	0.1016167	0.139805	0.1598067
272.221	2.5	0.532985	0.3571463	0.1469902	0.1002035	0.138888	0.1602357
272.321	2.5	0.525189	0.3595076	0.1489962	0.1010599	0.140409	0.1641418
272.421	2.5	0.523407	0.3603824	0.1555985	0.1010016	0.14222	0.1635135
272.521	2.5	0.513348	0.3612429	0.1535697	0.1031033	0.13999	0.1608212

272.621	2.5	0.535963	0.3555907	0.154327	0.09743444	0.141415	0.1626876
272.721	2.5	0.500437	0.3638885	0.1502424	0.1046051	0.139122	0.1461474
272.821	2.5	0.501403	0.3598945	0.1468011	0.1062912	0.138789	0.1482043
272.921	2.5	0.498888	0.3549513	0.1521334	0.1068969	0.138039	0.1483352
273.021	2.5	0.509294	0.3592874	0.156097	0.1023079	0.143168	0.1411756
273.121	2.5	0.501768	0.3571864	0.1537256	0.1019492	0.141654	0.1386107
273.221	2.5	0.465024	0.3537318	0.1573584	0.1015314	0.141071	0.1409382
273.321	2.5	0.502926	0.3502674	0.1630502	0.1014867	0.139788	0.1357486
273.421	2.5	0.501602	0.3532009	0.1549753	0.1000097	0.139267	0.13976
273.521	2.5	0.493867	0.354845	0.1549591	0.1009087	0.137809	0.137067
273.621	2.5	0.501973	0.3499754	0.1527609	0.1046843	0.141045	0.1337699
273.721	2.5	0.544734	0.3489529	0.1549041	0.1010883	0.138986	0.1375051
273.821	2.5	0.541817	0.3478061	0.1539853	0.09973653	0.140391	0.1329357
273.921	2.5	0.545554	0.3484875	0.1535352	0.1031911	0.140972	0.1358225
274.021	2.5	0.550967	0.3558985	0.1575914	0.1056403	0.141792	0.1286916
274.121	2.5	0.558427	0.3498845	0.1535854	0.1057262	0.141404	0.1365646
274.221	2.5	0.567853	0.342666	0.1517384	0.1039801	0.14087	0.1332846
274.321	2.5	0.555284	0.3473454	0.1549754	0.1030042	0.142557	0.1283837
274.421	2.5	0.535744	0.3492976	0.1575994	0.09792207	0.144694	0.1321293
274.521	2.5	0.532263	0.3544521	0.157553	0.09964464	0.143089	0.1317892
274.621	2.5	0.542798	0.3615823	0.1573362	0.09853084	0.140239	0.1274881
274.721	2.5	0.556812	0.3552943	0.1596487	0.09670455	0.142053	0.126394
274.821	2.5	0.56208	0.3528269	0.165023	0.09986874	0.14241	0.1302365
274.921	2.5	0.552707	0.3630512	0.1615504	0.1020351	0.14215	0.1295404
275.021	2.5	0.548419	0.358046	0.1562769	0.1016885	0.143933	0.1247117
275.121	2.5	0.547166	0.3684182	0.1577655	0.1072839	0.143056	0.1310379
275.221	2.5	0.52756	0.3593915	0.1679435	0.1034176	0.141489	0.1281115
275.321	2.5	0.533619	0.357051	0.1702713	0.1051632	0.14124	0.1355984
275.421	2.5	0.536784	0.3577575	0.1635691	0.1034018	0.142215	0.1368183
275.521	2.5	0.537415	0.3692625	0.1597317	0.0979568	0.140419	0.1362861
275.621	2.5	0.535519	0.3742765	0.1671175	0.09962457	0.139321	0.1305153
275.721	2.5	0.535718	0.359823	0.1581461	0.1011261	0.138939	0.1363703
275.821	2.5	0.545894	0.3588887	0.1514426	0.09955752	0.137815	0.1310885
275.921	2.5	0.552706	0.3658068	0.1596044	0.1013097	0.138359	0.1397597
276.021	2.5	0.543675	0.3623547	0.1652055	0.1015675	0.138025	0.1366328
276.121	2.5	0.526775	0.3638963	0.1644504	0.1030791	0.13745	0.1365299
276.221	2.5	0.55018	0.3586611	0.1662631	0.1036648	0.135298	0.1390081
276.321	2.5	0.56154	0.3640469	0.1625091	0.1052583	0.1348	0.1354443
276.421	2.5	0.547381	0.3661231	0.1558262	0.1063759	0.132915	0.1377426
276.521	2.5	0.551982	0.3611602	0.1574237	0.1046286	0.129633	0.1371445
276.621	2.5	0.552011	0.3645809	0.1605658	0.1074384	0.132935	0.1396126
276.721	2.5	0.54612	0.3568446	0.1634813	0.1086696	0.121168	0.1392138
276.821	2.5	0.551935	0.3604354	0.1672459	0.1083894	0.122827	0.1465744
276.921	2.5	0.54352	0.3586641	0.1598805	0.1104282	0.131039	0.1560568
277.021	2.5	0.541717	0.3591864	0.1611384	0.1118555	0.125914	0.154578
277.121	2.5	0.558244	0.3590975	0.1632582	0.111437	0.124649	0.1536722
277.221	2.5	0.572945	0.3579176	0.1596237	0.1069779	0.12299	0.1624648
277.321	2.5	0.563599	0.3560959	0.1648954	0.1101819	0.123458	0.1561687
277.421	2.5	0.582208	0.362039	0.1595789	0.1113793	0.121506	0.1577876
277.521	2.5	0.55919	0.3586906	0.1598499	0.1136056	0.120595	0.156215
277.621	2.5	0.550649	0.363674	0.1672036	0.1123479	0.123826	0.157934
277.721	2.5	0.551478	0.3658454	0.164544	0.1111494	0.123849	0.1607611
277.821	2.5	0.570755	0.36726	0.1647557	0.1129349	0.122507	0.1529077
277.921	2.5	0.583057	0.366178	0.1682302	0.1130635	0.122269	0.1593425
278.021	2.5	0.575071	0.3681146	0.1666515	0.1121047	0.119719	0.15911
278.121	2.5	0.575439	0.369836	0.1710562	0.1122613	0.123156	0.1553446
278.221	2.5	0.566339	0.366805	0.1679662	0.1121361	0.125372	0.1567588
278.321	2.5	0.587622	0.3670046	0.166297	0.114265	0.125256	0.1551547
278.421	2.5	0.586549	0.3632907	0.1663887	0.1143231	0.121303	0.1519779
278.521	2.5	0.577937	0.3635891	0.1706256	0.1135027	0.122046	0.1527523
278.621	2.5	0.58861	0.3597825	0.166895	0.1121687	0.12476	0.1540544
278.721	2.5	0.605891	0.3647599	0.1643569	0.1126953	0.123519	0.1517851
278.821	2.5	0.595855	0.3702255	0.1684247	0.1137531	0.122551	0.1520533
278.921	2.5	0.574839	0.3655698	0.1665984	0.1130702	0.125234	0.1482376
279.021	2.5	0.559213	0.373718	0.1664353	0.1150349	0.127262	0.1526983
279.121	2.5	0.56409	0.362955	0.1621118	0.113493	0.129422	0.1495555
279.221	2.5	0.58084	0.3575139	0.1707837	0.1144456	0.128312	0.1464583
279.321	2.5	0.595152	0.3602678	0.1755878	0.1160249	0.129677	0.1499765
279.421	2.5	0.590964	0.3614293	0.1725672	0.1145066	0.125624	0.1443943
279.521	2.5	0.598001	0.3620053	0.1646885	0.1138861	0.127346	0.1403459
279.621	2.5	0.600268	0.372414	0.1606854	0.1121913	0.1291	0.1364057
279.721	2.5	0.583104	0.3598311	0.1647061	0.1117728	0.13006	0.1384396
279.821	2.5	0.561188	0.362196	0.1712605	0.1138669	0.131805	0.1374759
279.921	2.5	0.560523	0.3696073	0.172713	0.1130332	0.128806	0.1380409
280.021	2.5	0.574373	0.3621998	0.1822391	0.1155354	0.126274	0.1419515
280.121	2.5	0.552952	0.3624934	0.1919825	0.1176761	0.130916	0.1370768
280.221	2.5	0.526281	0.3620292	0.1850997	0.1151059	0.131233	0.1378021
280.321	2.5	0.539964	0.3601001	0.1760938	0.1189362	0.130729	0.1370021
280.421	2.5	0.544743	0.3688131	0.1668975	0.1188894	0.133409	0.1350283
280.521	2.5	0.5567	0.3620701	0.1672432	0.1195654	0.132006	0.1413214
280.621	2.5	0.554399	0.3643555	0.1654657	0.117271	0.132284	0.1474186
280.721	2.5	0.543702	0.3673736	0.1642522	0.1171361	0.132714	0.1549725
280.821	2.5	0.534466	0.3539565	0.1692661	0.1214075	0.13542	0.1578629
280.921	2.5	0.516249	0.3624084	0.1725567	0.1234248	0.134503	0.1648997
281.021	2.5	0.503625	0.3672455	0.1731702	0.1213991	0.134237	0.1565172
281.121	2.5	0.52655	0.3638901	0.1717335	0.1172918	0.135463	0.1551603
281.221	2.5	0.560576	0.3697191	0.1679452	0.1115774	0.138155	0.1508918
281.321	2.5	0.579058	0.3625965	0.1697549	0.1097488	0.138225	0.1596712
281.421	2.5	0.566503	0.3711803	0.175659	0.114946	0.13928	0.1489822
281.521	2.5	0.579351	0.3734819	0.1740369	0.1114815	0.137714	0.1509288
281.621	2.5	0.591524	0.368001	0.1701571	0.1034632	0.136938	0.1569548
281.721	2.5	0.592742	0.3579254	0.1702353	0.1035036	0.138909	0.1565461
281.821	2.5	0.60093	0.3592819	0.1600713	0.1055744	0.139853	0.1617864
281.921	2.5	0.611349	0.3610584	0.1596299	0.1034008	0.138059	0.1542629
282.021	2.5	0.606914	0.3651069	0.1610149	0.1008543	0.140222	0.154103

282.121	2.5	0.606601	0.3583571	0.1641693	0.1021729	0.137522	0.1526234
282.221	2.5	0.606418	0.3581544	0.170502	0.1024042	0.140785	0.1548039
282.321	2.5	0.608148	0.3546321	0.1785772	0.09852126	0.142086	0.1525033
282.421	2.5	0.604905	0.3541281	0.1793809	0.1001564	0.146238	0.152994
282.521	2.5	0.597549	0.3585208	0.1796505	0.1051705	0.145732	0.1520716
282.621	2.5	0.596095	0.3573702	0.1795663	0.1059347	0.144816	0.1546088
282.721	2.5	0.599352	0.3523545	0.1781374	0.1059906	0.143609	0.1612354
282.821	2.5	0.541752	0.3499563	0.1823178	0.1043107	0.145213	0.1559459
282.921	2.5	0.537061	0.354808	0.1784362	0.1049555	0.144898	0.1633396
283.021	2.5	0.521974	0.3586994	0.1765691	0.1033385	0.145709	0.1681236
283.121	2.5	0.510752	0.3545895	0.173878	0.1019586	0.146955	0.1751344
283.221	2.5	0.536615	0.3496074	0.1639271	0.1074396	0.146131	0.1809129
283.321	2.5	0.555298	0.3470587	0.1682488	0.1086089	0.145992	0.1801226
283.421	2.5	0.548956	0.351229	0.1675748	0.1035195	0.146131	0.1800189
283.521	2.5	0.536786	0.3543382	0.1662739	0.1012764	0.147931	0.1835613
283.621	2.5	0.529312	0.3599251	0.1713352	0.1004456	0.149424	0.1807547
283.721	2.5	0.536175	0.3500935	0.1739668	0.100411	0.15177	0.1789534
283.821	2.5	0.566322	0.3508644	0.1688815	0.1011311	0.150311	0.1776361
283.921	2.5	0.582914	0.3532363	0.179606	0.1033385	0.155031	0.1767701
284.021	2.5	0.561225	0.3534764	0.1840356	0.1013625	0.153029	0.1781734
284.121	2.5	0.560663	0.3623515	0.1857301	0.1014547	0.154001	0.1748504
284.221	2.5	0.574847	0.3527283	0.1739055	0.1016323	0.158396	0.174806
284.321	2.5	0.573963	0.3554597	0.1589275	0.1025941	0.152317	0.1733508
284.421	2.5	0.588633	0.3545986	0.1637245	0.09635397	0.152015	0.1730333
284.521	2.5	0.608752	0.3432496	0.1606933	0.1001552	0.15407	0.1748949
284.621	2.5	0.616359	0.3510833	0.1639433	0.1015802	0.155117	0.1751401
284.721	2.5	0.611966	0.3553716	0.1677724	0.09974936	0.155574	0.1746752
284.821	2.5	0.618995	0.3526711	0.1749171	0.1000526	0.156037	0.1746828
284.921	2.5	0.582068	0.349933	0.1742909	0.09879889	0.154946	0.1727669
285.021	2.5	0.584082	0.3539607	0.1700957	0.1003621	0.155664	0.1740838
285.121	2.5	0.590518	0.3581409	0.168809	0.09994083	0.153929	0.1731029
285.221	2.5	0.604666	0.3522148	0.1712085	0.1019022	0.154217	0.1735746
285.321	2.5	0.564014	0.3538731	0.1694213	0.0969801	0.155334	0.1742621
285.421	2.5	0.582277	0.3274185	0.1707695	0.09620486	0.155227	0.1734838
285.521	2.5	0.607155	0.3372536	0.1710693	0.09995486	0.156393	0.1725524
285.621	2.5	0.610586	0.3536216	0.1689171	0.1031058	0.157854	0.1704609
285.721	2.5	0.637344	0.3485022	0.173545	0.1003494	0.159894	0.1682345
285.821	2.5	0.641737	0.3388511	0.1708368	0.1009523	0.160317	0.1664464
285.921	2.5	0.620778	0.3346318	0.1681349	0.1002171	0.159673	0.1703521
286.021	2.5	0.603986	0.34015	0.1639961	0.09890477	0.159409	0.1696119
286.121	2.5	0.596515	0.3399793	0.1681608	0.1029462	0.159282	0.1671363
286.221	2.5	0.599613	0.33277	0.1626074	0.1030413	0.15707	0.1680057
286.321	2.5	0.614293	0.3333539	0.1638546	0.1000179	0.161377	0.1678773
286.421	2.5	0.572842	0.3321986	0.1673002	0.09896395	0.167758	0.167959
286.521	2.5	0.580747	0.339931	0.1745085	0.09808149	0.162577	0.1680411
286.621	2.5	0.577208	0.3452105	0.1714891	0.09860227	0.166284	0.167351
286.721	2.5	0.549854	0.3422389	0.174301	0.09728252	0.167929	0.1647909
286.821	2.5	0.580067	0.3414113	0.1596608	0.09900533	0.166491	0.1683519
286.921	2.5	0.582874	0.3472479	0.1568022	0.1002268	0.164415	0.1686433
287.021	2.5	0.545918	0.3492106	0.1659368	0.09889251	0.160713	0.1672582
287.121	2.5	0.578681	0.3523992	0.1727835	0.09099503	0.159362	0.1671131
287.221	2.5	0.592129	0.3439889	0.1682914	0.09342013	0.160387	0.1674489
287.321	2.5	0.605367	0.3459243	0.1630643	0.1050621	0.150863	0.1659593
287.421	2.5	0.591721	0.3500424	0.1664758	0.09673153	0.145756	0.1647276
287.521	2.5	0.607977	0.3536477	0.1665469	0.09690198	0.148041	0.1693536
287.621	2.5	0.59005	0.3587369	0.1713935	0.09901793	0.150871	0.1684081
287.721	2.5	0.557322	0.3569098	0.172497	0.1002106	0.152795	0.1672907
287.821	2.5	0.580935	0.3473512	0.171027	0.1001626	0.152382	0.1648876
287.921	2.5	0.578133	0.346572	0.1732792	0.1022275	0.152516	0.166532
288.021	2.5	0.586391	0.3582169	0.1646469	0.1002519	0.154734	0.1680884
288.121	2.5	0.581187	0.3587899	0.1649971	0.0837013	0.157009	0.1710073
288.221	2.5	0.582658	0.3517485	0.1808357	0.08469594	0.156424	0.1701191
288.321	2.5	0.54501	0.3470843	0.1706397	0.09566872	0.153999	0.1709684
288.421	2.5	0.546328	0.3554376	0.1648148	0.09755377	0.152216	0.1729947
288.521	2.5	0.55403	0.3642607	0.1674186	0.09957626	0.153461	0.1683934
288.621	2.5	0.576616	0.3627198	0.1644695	0.09929284	0.153903	0.167477
288.721	2.5	0.574866	0.3579731	0.147728	0.09251781	0.150305	0.1603591
288.821	2.5	0.583988	0.3526816	0.1326378	0.09294823	0.148893	0.1675092
288.921	2.5	0.586082	0.3489308	0.1454142	0.1003937	0.14991	0.1670958
289.021	2.5	0.587719	0.3548672	0.1564669	0.1000626	0.151455	0.1647713
289.121	2.5	0.564491	0.3638656	0.1525917	0.09956405	0.155036	0.1647115
289.221	2.5	0.542004	0.3594916	0.1501744	0.1006548	0.153322	0.1646683
289.321	2.5	0.552564	0.3485453	0.1546647	0.1007732	0.156284	0.1592357
289.421	2.5	0.573331	0.3606411	0.1574804	0.09603697	0.152506	0.1591692
289.521	2.5	0.572637	0.3677645	0.1608712	0.08819714	0.153229	0.1609988
289.621	2.5	0.569513	0.3679206	0.1617096	0.09106832	0.150335	0.1611959
289.721	2.5	0.559458	0.3566285	0.160116	0.09536895	0.149197	0.1616542
289.821	2.5	0.554871	0.355477	0.1750077	0.09661527	0.146345	0.1605466
289.921	2.5	0.568746	0.3558666	0.1674163	0.09954453	0.148804	0.1615261
290.021	2.5	0.57804	0.3625532	0.1684329	0.1008218	0.153157	0.1614722
290.121	2.5	0.575832	0.3572205	0.1710231	0.1058148	0.153287	0.1598067
290.221	2.5	0.57573	0.3537357	0.1702186	0.1047849	0.151326	0.1577979
290.321	2.5	0.574507	0.3579757	0.1621443	0.1021584	0.151144	0.1591752
290.421	2.5	0.557149	0.3605834	0.1598834	0.09863152	0.152555	0.1577182
290.521	2.5	0.552617	0.3604662	0.1646083	0.09266534	0.155455	0.1578091
290.621	2.5	0.543181	0.3603421	0.1683335	0.08494911	0.150419	0.1551191
290.721	2.5	0.535024	0.3615295	0.1640084	0.09039161	0.15471	0.1585214
290.821	2.5	0.553593	0.3561666	0.160156	0.09104729	0.152163	0.1556001
290.921	2.5	0.558196	0.3569989	0.1591465	0.09325118	0.150136	0.1567574
291.021	2.5	0.542546	0.3635184	0.1598265	0.09160221	0.149348	0.1565442
291.121	2.5	0.558146	0.3651145	0.16369	0.09769253	0.150644	0.1559057
291.221	2.5	0.58721	0.368702	0.160204	0.1001812	0.152427	0.1568706
291.321	2.5	0.582207	0.366798	0.1607098	0.09792037	0.150361	0.1556398
291.421	2.5	0.608668	0.360608	0.1688234	0.09779871	0.147257	0.1570939
291.521	2.5	0.600016	0.371179	0.1576341	0.09556416	0.144774	0.1572245

291.621	2.5	0.576113	0.366062	0.1505418	0.09515388	0.146946	0.1563502
291.721	2.5	0.592141	0.3652883	0.1570729	0.09465559	0.150759	0.1574943
291.821	2.5	0.595181	0.3633598	0.1603666	0.09479941	0.149359	0.1542574
291.921	2.5	0.575811	0.3669277	0.1526687	0.0972894	0.152975	0.1550083
292.021	2.5	0.571836	0.3655684	0.1549737	0.09836897	0.154999	0.1568572
292.121	2.5	0.586151	0.3662789	0.1570351	0.102746	0.153594	0.1544326
292.221	2.5	0.586288	0.3657318	0.1615737	0.1017503	0.149612	0.1535732
292.321	2.5	0.578829	0.3636085	0.1651451	0.09963807	0.152344	0.152729
292.421	2.5	0.588766	0.3641166	0.1611001	0.1001026	0.148778	0.152142
292.521	2.5	0.56643	0.3589824	0.1592518	0.1025333	0.145732	0.1563828
292.621	2.5	0.565167	0.3676462	0.1653925	0.09519172	0.145268	0.1567332
292.721	2.5	0.560331	0.3564763	0.1622148	0.09128638	0.144717	0.1554569
292.821	2.5	0.553118	0.3557605	0.1577057	0.09824847	0.14768	0.1543789
292.921	2.5	0.560138	0.3564232	0.1592119	0.09683084	0.143303	0.1532662
293.021	2.5	0.553025	0.35464	0.1640499	0.09695388	0.141477	0.1534736
293.121	2.5	0.566951	0.3691361	0.1656284	0.0988552	0.143802	0.1537867
293.221	2.5	0.552858	0.3703911	0.1648619	0.100164	0.148231	0.1561491
293.321	2.5	0.55805	0.3688252	0.1594634	0.1013016	0.151309	0.1561652
293.421	2.5	0.5606	0.3623219	0.1545605	0.1002107	0.154147	0.1566448
293.521	2.5	0.5558	0.3612478	0.1598487	0.1022733	0.157382	0.1518113
293.621	2.5	0.549319	0.3681649	0.1670972	0.099342	0.154482	0.1539052
293.721	2.5	0.532793	0.3653331	0.1610877	0.09963407	0.150759	0.1560627
293.821	2.5	0.54078	0.3598542	0.1584259	0.100228	0.146805	0.156332
293.921	2.5	0.545741	0.3616584	0.1600472	0.09137872	0.144634	0.1570624
294.021	2.5	0.543636	0.3528634	0.1604538	0.0944966	0.145837	0.1570532
294.121	2.5	0.547842	0.3536426	0.1598051	0.1015165	0.144776	0.1591447
294.221	2.5	0.517798	0.3506274	0.1621916	0.09748992	0.146375	0.1592826
294.321	2.5	0.531093	0.3416652	0.1638769	0.09937933	0.146074	0.1592006
294.421	2.5	0.525006	0.3397101	0.1595619	0.1005208	0.144327	0.160289
294.521	2.5	0.533353	0.3344684	0.1640357	0.09614554	0.146667	0.1605754
294.621	2.5	0.535644	0.3380337	0.1684609	0.09611432	0.146792	0.1599258
294.721	2.5	0.539413	0.3388679	0.1729527	0.09900809	0.143062	0.1586625
294.821	2.5	0.531407	0.3450607	0.1721943	0.09760923	0.129369	0.1561489
294.921	2.5	0.534045	0.3367277	0.1598576	0.1003429	0.130535	0.1563445
295.021	2.5	0.533961	0.3294119	0.1616343	0.09933325	0.134245	0.1559386
295.121	2.5	0.536423	0.3278376	0.1568178	0.09943651	0.135563	0.1591692
295.221	2.5	0.54498	0.3273945	0.1573282	0.09697323	0.129551	0.1549717
295.321	2.5	0.525833	0.326361	0.1540101	0.09517514	0.133683	0.1538244
295.421	2.5	0.527242	0.3198782	0.1553264	0.09871867	0.139568	0.1538766
295.521	2.5	0.561269	0.3236842	0.1544114	0.09809191	0.138728	0.1546283
295.621	2.5	0.555033	0.3248859	0.1527774	0.09649535	0.138252	0.1553308
295.721	2.5	0.543592	0.3252491	0.1553256	0.0972771	0.139324	0.1565049
295.821	2.5	0.527825	0.321906	0.1524133	0.09112589	0.142578	0.1575212
295.921	2.5	0.512498	0.3276315	0.1492342	0.09755515	0.140855	0.1577197
296.021	2.5	0.516145	0.327576	0.1509216	0.09739313	0.139289	0.1583306
296.121	2.5	0.522524	0.3256071	0.1493439	0.09538453	0.140437	0.1593611
296.221	2.5	0.523496	0.3252226	0.1438629	0.09487636	0.140685	0.1590684
296.321	2.5	0.531156	0.3400337	0.1476893	0.0974984	0.140062	0.1622123
296.421	2.5	0.520606	0.3537924	0.1524678	0.09579875	0.140813	0.1644999
296.521	2.5	0.522672	0.35627	0.1504754	0.09763396	0.143048	0.1606514
296.621	2.5	0.521237	0.3619305	0.1561948	0.0924466	0.139743	0.1608328
296.721	2.5	0.532612	0.3642043	0.1504681	0.09015504	0.142889	0.1445451
296.821	2.5	0.505336	0.3536867	0.1476449	0.09878342	0.141656	0.143788
296.921	2.5	0.509066	0.3578097	0.1531617	0.09633934	0.141071	0.1433046
297.021	2.5	0.510041	0.3595625	0.1567069	0.09745692	0.144193	0.1398288
297.121	2.5	0.517714	0.3519132	0.1514479	0.09628525	0.146719	0.1375886
297.221	2.5	0.504254	0.3511994	0.1565528	0.09432478	0.143201	0.1390111
297.321	2.5	0.511886	0.3511328	0.159392	0.09396413	0.143104	0.1369004
297.421	2.5	0.506462	0.3510411	0.1554508	0.09368332	0.143628	0.140596
297.521	2.5	0.50305	0.3510843	0.1549183	0.09512063	0.142158	0.1390012
297.621	2.5	0.514798	0.3546601	0.1548171	0.1003183	0.143223	0.1364827
297.721	2.5	0.541132	0.347362	0.1545381	0.09866805	0.144312	0.139975
297.821	2.5	0.543772	0.3438543	0.1543875	0.09463362	0.142911	0.1346593
297.921	2.5	0.538116	0.3468849	0.1532649	0.09495113	0.141913	0.1411248
298.021	2.5	0.554058	0.3509188	0.1568728	0.1017381	0.143246	0.1346078
298.121	2.5	0.554554	0.3477911	0.1520351	0.1016659	0.143564	0.1411263
298.221	2.5	0.547427	0.3425679	0.1532753	0.09959072	0.142556	0.1404082
298.321	2.5	0.534294	0.3467497	0.158074	0.100363	0.143388	0.1420388
298.421	2.5	0.514517	0.3517746	0.1584489	0.0965938	0.144467	0.1394768
298.521	2.5	0.521295	0.3534976	0.1591837	0.09903517	0.144945	0.1385393
298.621	2.5	0.531484	0.3586392	0.1579328	0.09864953	0.141638	0.1325576
298.721	2.5	0.52905	0.3527084	0.1631066	0.097952	0.142879	0.1320571
298.821	2.5	0.534713	0.3496258	0.165131	0.0995507	0.145307	0.1323858
298.921	2.5	0.5164	0.3628435	0.1626219	0.1010317	0.145265	0.1323974
299.021	2.5	0.522304	0.3554665	0.1588253	0.100105	0.144059	0.1318958
299.121	2.5	0.522062	0.3628959	0.1607468	0.1038154	0.146643	0.1336429
299.221	2.5	0.527602	0.3497716	0.1698525	0.1026625	0.143012	0.1286907
299.321	2.5	0.531875	0.3502003	0.1704277	0.1028367	0.143788	0.1377275
299.421	2.5	0.528315	0.3580721	0.1617873	0.1040791	0.143343	0.1382588
299.521	2.5	0.527221	0.3640072	0.161184	0.09886431	0.142152	0.1329788
299.621	2.5	0.51825	0.366754	0.1671116	0.09915288	0.139506	0.1308281
299.721	2.5	0.528988	0.3560806	0.1560276	0.1005057	0.142396	0.1306733
299.904	3	0.533137	0.3543749	0.1536763	0.09987093	0.141451	0.1336916
300.004	3	0.529212	0.3534692	0.1623098	0.1004451	0.140433	0.1387993
300.104	3	0.524399	0.3563015	0.1655012	0.1032933	0.142359	0.1376039
300.204	3	0.52231	0.3505183	0.1698267	0.102802	0.144092	0.1407663
300.304	3	0.524814	0.356518	0.1697951	0.1063641	0.143067	0.1395407
300.404	3	0.519976	0.3556858	0.1632408	0.1060963	0.142311	0.1394556
300.504	3	0.519961	0.3525799	0.1619262	0.1092062	0.142687	0.1409418
300.604	3	0.517493	0.351975	0.1640408	0.1146039	0.139586	0.1443472
300.704	3	0.537066	0.3510301	0.1650439	0.1115285	0.136621	0.1437607
300.804	3	0.527043	0.3505283	0.169822	0.1101348	0.136685	0.1467068
300.904	3	0.517901	0.3501401	0.1644667	0.1139265	0.117649	0.1584699
301.004	3	0.516202	0.3525705	0.1660484	0.1179628	0.126818	0.1582374
301.104	3	0.518763	0.3506971	0.1664042	0.1145183	0.126883	0.1613418

301.204	3	0.536019	0.355949	0.1637866	0.1126458	0.123774	0.1582762
301.304	3	0.530495	0.3550189	0.170872	0.1159587	0.125392	0.1558016
301.404	3	0.533762	0.3532631	0.1685166	0.1160853	0.127839	0.1598845
301.504	3	0.520986	0.3543687	0.1719324	0.116501	0.128458	0.1559002
301.604	3	0.540218	0.3551274	0.1678742	0.1171921	0.126639	0.158297
301.704	3	0.527503	0.356223	0.1725893	0.1215022	0.126543	0.1551252
301.804	3	0.538643	0.3530349	0.1734465	0.1199763	0.128448	0.1555935
301.904	3	0.53886	0.3521646	0.168939	0.1196329	0.126939	0.1559899
302.004	3	0.525745	0.3522652	0.1703797	0.1197238	0.125515	0.1520839
302.104	3	0.519291	0.3578022	0.170925	0.1214296	0.12192	0.1527209
302.204	3	0.538005	0.3513577	0.1668242	0.1171156	0.125212	0.1516791
302.304	3	0.520586	0.3582405	0.1685855	0.1191759	0.125897	0.1538056
302.404	3	0.525815	0.3515021	0.171056	0.1178408	0.127164	0.1484142
302.504	3	0.520129	0.3485191	0.1667085	0.1194648	0.122192	0.1540429
302.604	3	0.521546	0.3512945	0.1715536	0.1203589	0.123451	0.1496412
302.704	3	0.515671	0.3483922	0.1766643	0.120888	0.123668	0.1419096
302.804	3	0.522074	0.3587838	0.1742959	0.1211403	0.126243	0.1415579
302.904	3	0.527146	0.3481405	0.1675768	0.1226686	0.126198	0.1438269
303.004	3	0.530597	0.3490244	0.1657317	0.1209042	0.125982	0.1384881
303.104	3	0.524035	0.3527081	0.1734809	0.1254303	0.126308	0.1433003
303.204	3	0.510732	0.3497784	0.1748908	0.1287592	0.127965	0.1408731
303.304	3	0.508064	0.3505368	0.1878602	0.1273693	0.125535	0.1376071
303.404	3	0.511053	0.3525459	0.1959159	0.1258003	0.12535	0.134446
303.504	3	0.502316	0.3530252	0.1857878	0.1260232	0.125751	0.1408327
303.604	3	0.492193	0.3527395	0.1715661	0.1225657	0.126341	0.1445088
303.704	3	0.490482	0.3572268	0.1708073	0.1229357	0.126534	0.1556929
303.804	3	0.477897	0.3493559	0.1681182	0.1270956	0.128811	0.1546479
303.904	3	0.486498	0.3528638	0.165562	0.1275176	0.130874	0.1560751
304.004	3	0.523357	0.3529002	0.1693713	0.1248268	0.125885	0.1533629
304.104	3	0.528733	0.3627891	0.1719615	0.1177164	0.123707	0.1510954
304.204	3	0.521486	0.3568831	0.172102	0.1126353	0.130159	0.1548907
304.304	3	0.517184	0.3613049	0.1679884	0.1149579	0.129988	0.143947
304.404	3	0.54028	0.3599296	0.1740293	0.1068716	0.129509	0.1534334
304.504	3	0.527584	0.3546947	0.1784994	0.1036071	0.131017	0.1559474
304.604	3	0.525067	0.3551169	0.172873	0.1069525	0.129183	0.1615054
304.704	3	0.516639	0.3530335	0.1683616	0.1032041	0.130018	0.1526511
304.804	3	0.529297	0.3549776	0.161153	0.1026584	0.129149	0.1529902
304.904	3	0.542487	0.3498198	0.1600088	0.107909	0.13261	0.1499916
305.004	3	0.51742	0.3471647	0.1619144	0.1009789	0.131569	0.1547403
305.104	3	0.525184	0.3452776	0.1684909	0.1021924	0.129655	0.1540097
305.204	3	0.510098	0.3455425	0.1740798	0.1023761	0.130051	0.1548773
305.304	3	0.528249	0.3454863	0.1754945	0.1048991	0.132847	0.1585039
305.404	3	0.5046	0.3416208	0.1784306	0.1042654	0.131411	0.1594797
305.504	3	0.498016	0.3413248	0.1785737	0.1022855	0.13453	0.1574983
305.604	3	0.524391	0.3441814	0.1825739	0.1026675	0.134667	0.1663795
305.704	3	0.522396	0.3410547	0.1787302	0.103534	0.134114	0.1716477
305.804	3	0.514631	0.3420148	0.1759427	0.1072687	0.136412	0.1767508
305.904	3	0.529663	0.341989	0.1667016	0.1008917	0.138668	0.1769307
306.004	3	0.509952	0.3440623	0.1632539	0.09868143	0.135791	0.1780082
306.104	3	0.512938	0.353185	0.1639898	0.09835511	0.138127	0.1771468
306.204	3	0.546352	0.3441593	0.1646202	0.09926208	0.137136	0.1770512
306.304	3	0.537955	0.3389652	0.1707733	0.09969572	0.139833	0.1751105
306.404	3	0.525331	0.3444435	0.1702819	0.0983553	0.140228	0.175721
306.504	3	0.534326	0.3487019	0.1771992	0.09857311	0.141113	0.178645
306.604	3	0.5261	0.3425122	0.1831618	0.1010596	0.141271	0.1772813
306.704	3	0.540706	0.3457403	0.1852726	0.09827567	0.144266	0.1734807
306.804	3	0.543588	0.344183	0.1683133	0.09911734	0.142727	0.1772375
306.904	3	0.537504	0.3348401	0.1620616	0.1022562	0.145086	0.1771909
307.004	3	0.538913	0.3430729	0.162187	0.09822717	0.145208	0.174939
307.104	3	0.530334	0.3456301	0.1655173	0.09718282	0.14612	0.1764211
307.204	3	0.529071	0.3422702	0.1684726	0.09666693	0.147926	0.1722238
307.304	3	0.531971	0.343073	0.1735784	0.1009197	0.145309	0.1735645
307.404	3	0.534942	0.3475381	0.170082	0.09972037	0.145131	0.1746031
307.504	3	0.540155	0.3426894	0.1658119	0.09616014	0.145217	0.1762713
307.604	3	0.562989	0.3287189	0.1687903	0.09450529	0.146959	0.1733659
307.704	3	0.553087	0.3314972	0.1666821	0.1000125	0.148367	0.1745625
307.804	3	0.577448	0.3465126	0.1656615	0.09841183	0.149284	0.1720309
307.904	3	0.564682	0.3398754	0.1651494	0.09953548	0.148596	0.1689871
308.004	3	0.546952	0.3305959	0.1658318	0.09769535	0.154641	0.1684336
308.104	3	0.536447	0.3334641	0.1674172	0.09727191	0.153108	0.1688955
308.204	3	0.557191	0.3353871	0.1650869	0.09984687	0.155053	0.1646117
308.304	3	0.532459	0.3293513	0.164356	0.09608985	0.157015	0.1681528
308.404	3	0.530191	0.3292005	0.1623289	0.09442722	0.152122	0.1712246
308.504	3	0.539589	0.3315565	0.1603458	0.09401035	0.149209	0.1727331
308.604	3	0.51201	0.3408009	0.164273	0.09346715	0.150574	0.1709004
308.704	3	0.512105	0.3401511	0.1719948	0.09139408	0.151139	0.170375
308.804	3	0.535236	0.3391266	0.1709251	0.09634649	0.150776	0.1692109
308.904	3	0.522776	0.344604	0.1675476	0.09498437	0.151672	0.1704838
309.004	3	0.530139	0.3455871	0.154805	0.08765802	0.150882	0.1694427
309.104	3	0.540945	0.3420896	0.1626305	0.08863115	0.151334	0.1682843
309.204	3	0.535877	0.3397282	0.1699048	0.09432459	0.149742	0.1641953
309.304	3	0.535168	0.3464912	0.1643463	0.09337121	0.148895	0.1637937
309.404	3	0.532748	0.3469179	0.1655895	0.09693168	0.150071	0.1680934
309.504	3	0.531947	0.3540258	0.1691152	0.09710255	0.149602	0.1650084
309.604	3	0.542406	0.3437149	0.1722783	0.09606446	0.150296	0.163009
309.704	3	0.534475	0.3420857	0.1730329	0.09788786	0.151485	0.1648761
309.804	3	0.534512	0.3528196	0.171579	0.08479685	0.152115	0.1676243
309.904	3	0.51572	0.3550842	0.1738279	0.08117982	0.153057	0.1713013
310.004	3	0.510745	0.3462732	0.1686375	0.09274663	0.153864	0.1702262
310.104	3	0.515513	0.3467747	0.1797204	0.09362664	0.154139	0.1725207
310.204	3	0.545564	0.3575555	0.1711805	0.09397481	0.15316	0.1694652
310.304	3	0.537313	0.3603263	0.1650863	0.08824614	0.152699	0.1670199
310.404	3	0.520864	0.3516542	0.1680542	0.08919483	0.154994	0.1625549
310.504	3	0.528874	0.3464286	0.1641859	0.09755134	0.162121	0.1654171
310.604	3	0.532413	0.3457286	0.136316	0.09765361	0.155407	0.1625444

310.704	3	0.529927	0.3531523	0.1450413	0.09903839	0.15616	0.1627368
310.804	3	0.53583	0.3576035	0.1604121	0.09804159	0.15742	0.1607802
310.904	3	0.522611	0.3427476	0.1551477	0.08846392	0.157285	0.1560301
311.004	3	0.524281	0.3531612	0.1566775	0.08027396	0.153397	0.1598829
311.104	3	0.518645	0.3595243	0.1610606	0.08433371	0.150766	0.160504
311.204	3	0.521409	0.3534286	0.1613661	0.08639793	0.151595	0.1599721
311.304	3	0.526207	0.3457372	0.164747	0.09091868	0.150665	0.1589499
311.404	3	0.530756	0.3494617	0.168241	0.09458269	0.145117	0.1608309
311.504	3	0.528561	0.355804	0.1770599	0.09985452	0.139453	0.1584911
311.604	3	0.533267	0.3502877	0.171743	0.09706496	0.142723	0.1567104
311.704	3	0.523751	0.3464791	0.1760588	0.09212796	0.146891	0.158107
311.804	3	0.519655	0.3526927	0.1770212	0.08423221	0.146468	0.1541892
311.904	3	0.517639	0.3584503	0.166719	0.07977553	0.14867	0.1551541
312.004	3	0.53642	0.3504431	0.1634171	0.08346397	0.14829	0.1558868
312.104	3	0.529429	0.3489885	0.1709699	0.08734843	0.150921	0.154583
312.204	3	0.532844	0.350659	0.1708199	0.08800762	0.150692	0.1540258
312.304	3	0.538142	0.3552646	0.1637813	0.094104	0.151769	0.1540572
312.404	3	0.554226	0.3564703	0.1635689	0.09555851	0.146396	0.1549262
312.504	3	0.566709	0.3630898	0.166682	0.09476958	0.145636	0.1531394
312.604	3	0.553177	0.3583315	0.1656441	0.09224252	0.146853	0.1546843
312.704	3	0.542652	0.3570974	0.1689552	0.09386817	0.148172	0.1543627
312.804	3	0.554154	0.3655155	0.1771618	0.09308302	0.143626	0.1560097
312.904	3	0.54207	0.3603237	0.1579831	0.0921755	0.141702	0.1553795
313.004	3	0.531824	0.3624718	0.1576452	0.09203959	0.14446	0.1529272
313.104	3	0.544685	0.3617723	0.1664046	0.09827343	0.143977	0.1521843
313.204	3	0.553941	0.3574787	0.1585812	0.09829142	0.152308	0.1524943
313.304	3	0.55643	0.3592661	0.1597703	0.09662288	0.150485	0.1527765
313.404	3	0.539134	0.3572776	0.1597383	0.09743226	0.154167	0.1539673
313.504	3	0.524718	0.3555053	0.1646477	0.094892	0.15045	0.1531425
313.604	3	0.530274	0.3550089	0.1598545	0.08659979	0.149255	0.1527894
313.704	3	0.517135	0.3568565	0.158048	0.09510054	0.148557	0.1538434
313.804	3	0.517872	0.3491475	0.162778	0.09430298	0.145908	0.1528619
313.904	3	0.526878	0.3505991	0.156967	0.09555265	0.146058	0.154157
314.004	3	0.523666	0.3503838	0.1559792	0.0964678	0.143447	0.1526755
314.104	3	0.529835	0.3553697	0.1614917	0.09653395	0.149282	0.1526077
314.204	3	0.529833	0.3603843	0.1648076	0.09806163	0.151796	0.153014
314.304	3	0.535295	0.3597631	0.1649362	0.1001624	0.149718	0.1522894
314.404	3	0.510301	0.354609	0.1583256	0.09708125	0.148042	0.1523901
314.504	3	0.518118	0.3609483	0.1569634	0.09684747	0.148578	0.1555748
314.604	3	0.517311	0.3524039	0.1687522	0.09081301	0.154137	0.1560594
314.704	3	0.523001	0.3508522	0.1621746	0.08900042	0.148716	0.1537762
314.804	3	0.514213	0.3493238	0.1602659	0.09608068	0.150839	0.1537005
314.904	3	0.519211	0.3460205	0.1619875	0.09410766	0.147636	0.1546442
315.004	3	0.512903	0.3451518	0.1621735	0.09692431	0.147374	0.1542617
315.104	3	0.528855	0.3404632	0.1631425	0.09521536	0.148031	0.153342
315.204	3	0.521497	0.3421232	0.1626519	0.09176719	0.148368	0.1536362
315.304	3	0.521802	0.3352418	0.1614413	0.09623053	0.150785	0.1523822
315.404	3	0.52112	0.3361521	0.1692371	0.09657479	0.150663	0.1507193
315.504	3	0.517941	0.345816	0.16897	0.09851147	0.146835	0.1509151
315.604	3	0.524031	0.3337751	0.1682471	0.09762242	0.143675	0.1492934
315.704	3	0.521307	0.3257914	0.1588155	0.09298182	0.146612	0.1497059
315.804	3	0.516701	0.3351644	0.1572869	0.09189028	0.152716	0.1481167
315.904	3	0.52501	0.3367623	0.1568225	0.09481978	0.150565	0.1476214
316.004	3	0.537116	0.3373711	0.1577323	0.09359681	0.152532	0.1505941
316.104	3	0.520547	0.3369121	0.1577163	0.09335737	0.150748	0.1508031
316.204	3	0.525859	0.3310558	0.1539092	0.08823864	0.150627	0.1533241
316.304	3	0.513995	0.329925	0.1537345	0.08983974	0.148165	0.1550323
316.404	3	0.520361	0.3427998	0.1558636	0.09458842	0.150827	0.1560262
316.504	3	0.528898	0.347195	0.1521119	0.09365194	0.144874	0.154918
316.604	3	0.523617	0.3393339	0.1538747	0.09337692	0.146064	0.1554506
316.704	3	0.519644	0.339826	0.1494836	0.09441312	0.146754	0.1580339
316.804	3	0.523349	0.3457209	0.1436466	0.09457257	0.144871	0.1603824
316.904	3	0.534721	0.3536082	0.1523916	0.09160524	0.148832	0.1588799
317.004	3	0.509147	0.3583722	0.1525048	0.08950613	0.143156	0.15865
317.104	3	0.512075	0.3558377	0.1569312	0.0961135	0.143166	0.1403436
317.204	3	0.517547	0.3554655	0.1500668	0.09277435	0.143204	0.1421988
317.304	3	0.511843	0.3624426	0.1494391	0.09246245	0.147733	0.1399145
317.404	3	0.511591	0.3633572	0.1564174	0.09082589	0.153643	0.1391718
317.504	3	0.505189	0.3562812	0.151478	0.08940703	0.154637	0.1381112
317.604	3	0.512292	0.355929	0.1586336	0.08937289	0.156796	0.1383256
317.704	3	0.519494	0.3591124	0.1558925	0.09088653	0.15637	0.1408467
317.804	3	0.536059	0.3555981	0.1568616	0.094318	0.153558	0.138896
317.904	3	0.525131	0.3546605	0.155576	0.09274957	0.147539	0.1416115
318.004	3	0.54574	0.353716	0.1551155	0.09206606	0.147833	0.1357785
318.104	3	0.527237	0.356411	0.1527677	0.09965007	0.147021	0.138552
318.204	3	0.528506	0.3577993	0.1524745	0.09692436	0.146621	0.1407613
318.304	3	0.512803	0.348908	0.1556698	0.09692764	0.148407	0.138457
318.404	3	0.51297	0.3597	0.1517383	0.09597974	0.147624	0.1394284
318.504	3	0.523849	0.3581206	0.1554389	0.09732381	0.144412	0.1420179
318.604	3	0.514818	0.3650123	0.1558506	0.0952875	0.143795	0.1379298
318.704	3	0.51549	0.3606611	0.1566138	0.09593052	0.145975	0.1370295
318.804	3	0.515804	0.3564521	0.1575496	0.0944315	0.143014	0.1369859
318.904	3	0.505512	0.3666613	0.1617316	0.09605752	0.122417	0.1351442
319.004	3	0.503209	0.3636712	0.1596685	0.09830975	0.127843	0.1365802
319.104	3	0.514445	0.36579	0.1573342	0.09895302	0.135196	0.1338449
319.204	3	0.517065	0.3611865	0.1635751	0.09941219	0.136073	0.1352077
319.304	3	0.517757	0.3640487	0.1698287	0.0971778	0.130403	0.139479
319.404	3	0.524091	0.3736648	0.1631699	0.09182996	0.135103	0.131651
319.504	3	0.514258	0.372559	0.1612046	0.09592692	0.141372	0.1342112
319.604	3	0.520216	0.3629557	0.1629445	0.09368174	0.141082	0.1303228
319.704	3	0.512133	0.3687374	0.1496262	0.09502619	0.139596	0.1380219
319.804	3	0.521688	0.3627543	0.1555414	0.09706623	0.141251	0.1373215
319.904	3	0.523456	0.3652853	0.1621165	0.09915143	0.1441	0.1433964
320.004	3	0.514144	0.3641504	0.1655119	0.0961896	0.141946	0.142553
320.104	3	0.517187	0.3727187	0.1668682	0.09922979	0.140126	0.14745

320.204	3	0.510445	0.3702029	0.1615433	0.09845718	0.14109	0.1498248
320.304	3	0.513529	0.3678759	0.1588207	0.09970994	0.141279	0.1536046
320.404	3	0.517843	0.3631714	0.1600346	0.1018142	0.141964	0.1547285
320.504	3	0.513327	0.3650542	0.1647909	0.1054016	0.141824	0.1636281
320.604	3	0.514178	0.3657416	0.1650932	0.1109854	0.144558	0.1623552
320.704	3	0.521872	0.3636673	0.1593034	0.1087984	0.139465	0.1568297
320.804	3	0.520279	0.3585667	0.1621825	0.1078927	0.142406	0.1645127
320.904	3	0.504328	0.3569134	0.1590681	0.1119283	0.141273	0.1606174
321.004	3	0.518078	0.3594661	0.1648762	0.1158052	0.139246	0.1592182
321.104	3	0.502629	0.3597289	0.1610334	0.1153884	0.143923	0.1589793
321.204	3	0.525155	0.3679651	0.1641755	0.1122796	0.145462	0.1625414
321.304	3	0.516425	0.3690644	0.1696839	0.1155807	0.140286	0.15928
321.404	3	0.523756	0.3687821	0.1653531	0.1180217	0.138373	0.1612779
321.504	3	0.514193	0.3668763	0.1681551	0.1179213	0.139174	0.1552498
321.604	3	0.513418	0.364994	0.1672727	0.119373	0.138424	0.1567678
321.704	3	0.521463	0.3670863	0.1716123	0.1230077	0.139319	0.1598483
321.804	3	0.523647	0.3646537	0.169834	0.1198614	0.139375	0.1556647
321.904	3	0.52194	0.3625	0.1666075	0.1201844	0.140819	0.1531586
322.004	3	0.519269	0.342916	0.1690041	0.118731	0.140239	0.1489497
322.104	3	0.514072	0.3440759	0.1675893	0.1176833	0.140794	0.1526447
322.204	3	0.536936	0.3411574	0.1656616	0.1146547	0.141805	0.1491254
322.304	3	0.509389	0.3482365	0.1670432	0.1151094	0.141061	0.1564126
322.404	3	0.518478	0.3434688	0.1714574	0.1169191	0.142668	0.1527485
322.504	3	0.509154	0.3437508	0.1657836	0.1191365	0.142405	0.1592501
322.604	3	0.514535	0.3431572	0.1722624	0.1191092	0.142857	0.154798
322.704	3	0.511824	0.3404455	0.1756713	0.1203896	0.138568	0.1445153
322.804	3	0.521583	0.3450911	0.1712156	0.1214498	0.139033	0.1445511
322.904	3	0.523045	0.3318089	0.1639256	0.122851	0.141063	0.1451997
323.004	3	0.518152	0.3352537	0.1634365	0.1212068	0.140534	0.1393145
323.104	3	0.512612	0.3303012	0.1712894	0.1226745	0.138867	0.142193
323.204	3	0.504914	0.3289286	0.1715782	0.1263773	0.140822	0.1413618
323.304	3	0.504298	0.334134	0.1847881	0.1267003	0.139473	0.1402313
323.404	3	0.503928	0.3359133	0.1864345	0.126943	0.13948	0.1379951
323.504	3	0.496071	0.3388382	0.1778596	0.1274171	0.138684	0.1407914
323.604	3	0.479747	0.341127	0.1714509	0.1250799	0.138409	0.1430481
323.704	3	0.480255	0.3420184	0.1703345	0.1232929	0.134981	0.1533224
323.804	3	0.476692	0.3362351	0.1661976	0.1244342	0.136257	0.1516358
323.904	3	0.493263	0.3427649	0.1640435	0.1248965	0.137563	0.1545029
324.004	3	0.520104	0.3405417	0.165304	0.1234415	0.136151	0.1536739
324.104	3	0.512458	0.3501883	0.1689092	0.1184165	0.137773	0.147608
324.204	3	0.507094	0.3480249	0.1692908	0.1171516	0.13848	0.1523099
324.304	3	0.505992	0.3472374	0.1650403	0.1133908	0.138408	0.1408626
324.404	3	0.526885	0.3473577	0.1678924	0.102934	0.137963	0.1518615
324.504	3	0.50776	0.3463464	0.173804	0.1038943	0.1387	0.1551403
324.604	3	0.50798	0.349868	0.1709191	0.1071373	0.138992	0.1579793
324.704	3	0.501148	0.343919	0.1709179	0.1046614	0.134288	0.1497957
324.804	3	0.539617	0.3430463	0.1614746	0.1061302	0.136288	0.1509835
324.904	3	0.517588	0.3444361	0.1612162	0.1067607	0.125597	0.1483574
325.004	3	0.506921	0.3417846	0.1611357	0.1010558	0.123346	0.1538486
325.104	3	0.509734	0.3431979	0.1667634	0.1015073	0.133176	0.1517879
325.204	3	0.482758	0.3414178	0.1751822	0.1051349	0.128274	0.1499766
325.304	3	0.476431	0.3458211	0.1758678	0.1036393	0.126861	0.1482356
325.404	3	0.474218	0.3405994	0.1759823	0.1030294	0.128123	0.1548418
325.504	3	0.494286	0.3409391	0.1764402	0.1039949	0.127805	0.1513931
325.604	3	0.500481	0.3422212	0.1801977	0.1024547	0.128923	0.165648
325.704	3	0.49729	0.3441738	0.1766804	0.1042248	0.125987	0.1679624
325.804	3	0.50907	0.3369989	0.1773774	0.1049327	0.129873	0.1720896
325.904	3	0.504515	0.3388667	0.1700383	0.1008644	0.12912	0.1733237
326.004	3	0.505146	0.340678	0.1664469	0.09969653	0.127712	0.174213
326.104	3	0.521526	0.3444899	0.1652096	0.09903612	0.125054	0.1767273
326.204	3	0.524654	0.3431506	0.1644461	0.09921724	0.12447	0.1768151
326.304	3	0.516737	0.3379524	0.1716414	0.1011333	0.129013	0.1717383
326.404	3	0.494062	0.3411396	0.1713962	0.09820294	0.13006	0.1716265
326.504	3	0.513196	0.3419139	0.1755205	0.09986491	0.128761	0.1712783
326.604	3	0.516387	0.3433049	0.181952	0.1005201	0.128787	0.1745452
326.704	3	0.507866	0.3420277	0.1837922	0.09773072	0.128373	0.169899
326.804	3	0.522616	0.3432449	0.1688716	0.1010053	0.133685	0.1680054
326.904	3	0.511005	0.3377093	0.1625153	0.1015727	0.13048	0.1686051
327.004	3	0.516942	0.336158	0.1622469	0.09697819	0.131334	0.1698073
327.104	3	0.505229	0.3454898	0.1643863	0.09918834	0.130884	0.1721135
327.204	3	0.518468	0.3399729	0.167552	0.098779	0.132586	0.1709665
327.304	3	0.507316	0.3448995	0.1718456	0.10079	0.131893	0.1699119
327.404	3	0.525386	0.3487235	0.1694919	0.09939175	0.131663	0.1694061
327.504	3	0.52855	0.343262	0.1662945	0.09248725	0.132373	0.1708582
327.604	3	0.538834	0.3424087	0.1684038	0.09644987	0.131969	0.1701986
327.704	3	0.54394	0.3177229	0.1667068	0.1011747	0.133721	0.1672583
327.804	3	0.538414	0.3318887	0.1676859	0.09807654	0.134414	0.1681318
327.904	3	0.517491	0.3392778	0.1662352	0.09814312	0.136254	0.1662833
328.004	3	0.517994	0.3302416	0.1670721	0.09608953	0.133301	0.1645603
328.104	3	0.51477	0.328597	0.168972	0.09837829	0.129132	0.1665434
328.204	3	0.522085	0.3337981	0.1662644	0.1006563	0.131667	0.1607774
328.304	3	0.512647	0.3260939	0.1671805	0.09502386	0.134564	0.1614607
328.404	3	0.515666	0.3240171	0.1652463	0.09656768	0.13368	0.1631794
328.504	3	0.518322	0.3304152	0.1620964	0.09523734	0.135556	0.1613168
328.604	3	0.497826	0.334414	0.1670035	0.0931185	0.136333	0.1629382
328.704	3	0.514211	0.3390555	0.1739732	0.09443554	0.137208	0.167402
328.804	3	0.525843	0.3389353	0.1733056	0.09676646	0.136154	0.1670874
328.904	3	0.525888	0.3442101	0.1678456	0.093647	0.136482	0.1633553
329.004	3	0.524199	0.3460947	0.1575033	0.08435801	0.139394	0.1638972
329.104	3	0.533008	0.3436365	0.1655772	0.09441528	0.135648	0.1647061
329.204	3	0.528845	0.3375276	0.1698827	0.09504833	0.135175	0.1638728
329.304	3	0.514712	0.3403028	0.1654053	0.09732588	0.137355	0.158255
329.404	3	0.523757	0.3475287	0.1690569	0.09911869	0.135105	0.1663371
329.504	3	0.521741	0.3557907	0.169141	0.0960554	0.137938	0.1660488
329.604	3	0.519719	0.3484417	0.1719063	0.09676165	0.1367	0.1656301

329.704	3	0.527767	0.3443038	0.1722787	0.0959981	0.1373	0.1645077
329.804	3	0.519321	0.3482211	0.173661	0.07845704	0.137283	0.1635441
329.904	3	0.506486	0.3560603	0.1747488	0.08495208	0.14015	0.1663182
330.004	3	0.50506	0.3427199	0.1687568	0.08901094	0.138611	0.1676467
330.104	3	0.520375	0.3425259	0.1800631	0.09304116	0.138954	0.1671447
330.204	3	0.535235	0.3523664	0.1725513	0.09246881	0.139105	0.1655336
330.304	3	0.529919	0.3571801	0.1699488	0.0858013	0.13923	0.1704561
330.404	3	0.515312	0.3497108	0.1706832	0.09161791	0.14047	0.1577556
330.504	3	0.523847	0.346087	0.1641769	0.09482069	0.138795	0.162218
330.604	3	0.527935	0.3456493	0.1384201	0.09709494	0.140989	0.1618101
330.704	3	0.523878	0.3501252	0.1465692	0.09960223	0.141768	0.1591852
330.804	3	0.530715	0.3571077	0.1617002	0.09531322	0.138195	0.1585507
330.904	3	0.519747	0.3448041	0.1592608	0.08486293	0.13768	0.1529783
331.004	3	0.514047	0.3517267	0.15887	0.0828044	0.136417	0.1553265
331.104	3	0.517377	0.3592033	0.162542	0.08646248	0.137585	0.1583929
331.204	3	0.510925	0.3544193	0.1662185	0.08898116	0.140783	0.1574026
331.304	3	0.515518	0.3432894	0.1694416	0.09213787	0.143514	0.15659
331.404	3	0.504942	0.347988	0.1765593	0.09445439	0.144917	0.1606835
331.504	3	0.518372	0.3530833	0.1766226	0.09940957	0.145315	0.1594498
331.604	3	0.507002	0.3473142	0.1743015	0.09821365	0.147456	0.1571462
331.704	3	0.514517	0.3417366	0.1783319	0.09286543	0.148248	0.1582853
331.804	3	0.516154	0.3506585	0.1787997	0.08539714	0.151024	0.1549742
331.904	3	0.512966	0.3557883	0.1700135	0.08430681	0.145822	0.153497
332.004	3	0.52572	0.3446535	0.1704166	0.0865519	0.144362	0.1524929
332.104	3	0.531296	0.3438559	0.1766236	0.08800949	0.147134	0.1550427
332.204	3	0.527777	0.3440989	0.1728195	0.08765765	0.146803	0.1548783
332.304	3	0.529957	0.3454483	0.1695619	0.09467342	0.147319	0.1540672
332.404	3	0.543989	0.3484568	0.1665398	0.09508288	0.147159	0.1553232
332.504	3	0.553374	0.3536938	0.1719839	0.0971271	0.146882	0.1536951
332.604	3	0.543904	0.349593	0.1702686	0.09398602	0.145098	0.1533149
332.704	3	0.539487	0.3489041	0.1731703	0.09474947	0.146254	0.152977
332.804	3	0.546552	0.3513564	0.1821449	0.09192656	0.147079	0.1521818
332.904	3	0.532142	0.3482859	0.1628149	0.09218022	0.14778	0.1534205
333.004	3	0.521313	0.3500849	0.1615105	0.09434328	0.147926	0.1524473
333.104	3	0.539258	0.3494721	0.1688791	0.09768952	0.150237	0.1524527
333.204	3	0.550123	0.3471294	0.1605955	0.09744014	0.150815	0.1524138
333.304	3	0.546714	0.346132	0.1642231	0.09513389	0.149625	0.1525472
333.404	3	0.523361	0.3451506	0.1655378	0.09737216	0.150364	0.1529268
333.504	3	0.520927	0.347133	0.1708156	0.09572701	0.150499	0.1521997
333.604	3	0.52668	0.3453843	0.1666168	0.08621418	0.158708	0.1525265
333.704	3	0.505621	0.3479709	0.1636241	0.09493634	0.154764	0.1527049
333.804	3	0.512474	0.3432924	0.1689175	0.09464857	0.155322	0.1530785
333.904	3	0.515183	0.3449607	0.162965	0.09351535	0.157083	0.1543678
334.004	3	0.516489	0.3461228	0.1613883	0.0947157	0.153328	0.1545803
334.104	3	0.522709	0.3454435	0.1656319	0.09440741	0.148655	0.1545629
334.204	3	0.522769	0.3493988	0.168215	0.09827414	0.149312	0.1531971
334.304	3	0.524306	0.3523275	0.1685699	0.09962771	0.148288	0.1522631
334.404	3	0.504249	0.3482759	0.1625565	0.09718788	0.139275	0.1510832
334.504	3	0.51401	0.3507793	0.1611966	0.09590098	0.139201	0.156655
334.604	3	0.507401	0.3483332	0.1737433	0.08694335	0.146793	0.1537736
334.704	3	0.519084	0.3421698	0.1739229	0.08765265	0.147518	0.1551062
334.804	3	0.513869	0.3433461	0.167817	0.09360023	0.148223	0.1558337
334.904	3	0.510974	0.3404116	0.1707745	0.09101045	0.148001	0.1550117
335.004	3	0.504182	0.3400588	0.1698007	0.09502757	0.149856	0.1543339
335.104	3	0.522606	0.3395848	0.168267	0.09219471	0.147561	0.1525887
335.204	3	0.514042	0.3445493	0.169975	0.0898502	0.142336	0.1531539
335.304	3	0.513844	0.3401155	0.1663907	0.09338183	0.142457	0.1501802
335.404	3	0.51651	0.3388227	0.1713984	0.09558547	0.146047	0.1497009
335.504	3	0.517784	0.3486342	0.1736217	0.09853911	0.144354	0.1484928
335.604	3	0.522645	0.3439748	0.1772047	0.09863576	0.139918	0.1471838
335.704	3	0.512891	0.343311	0.1676512	0.09519225	0.143042	0.1473873
335.804	3	0.516173	0.3467892	0.162956	0.09047662	0.144422	0.1469025
335.904	3	0.519813	0.346705	0.1599162	0.09379014	0.148859	0.1462841
336.004	3	0.537327	0.3432662	0.1618225	0.09318728	0.150272	0.1477442
336.104	3	0.516748	0.3400488	0.1594166	0.0937218	0.149511	0.1488008
336.204	3	0.526646	0.3456042	0.1600643	0.08579001	0.148281	0.1501271
336.304	3	0.515694	0.3426979	0.1554113	0.08719442	0.145247	0.1493607
336.404	3	0.517475	0.3388427	0.1584495	0.08894646	0.144158	0.1515205
336.504	3	0.530511	0.3447182	0.1565606	0.09232693	0.14115	0.14963
336.604	3	0.524892	0.3418648	0.1580617	0.09263609	0.143532	0.1487303
336.704	3	0.521768	0.3412208	0.1583855	0.09278531	0.146549	0.1506025
336.804	3	0.522422	0.344463	0.1497817	0.09210272	0.144459	0.1521274
336.904	3	0.532321	0.3453183	0.1496476	0.0875431	0.142621	0.1510355
337.004	3	0.508908	0.3445673	0.1534045	0.09015663	0.146422	0.1458643
337.104	3	0.504551	0.3487818	0.1588714	0.09249642	0.144221	0.1365754
337.204	3	0.514632	0.3440615	0.1573085	0.09051158	0.145536	0.1380612
337.304	3	0.509888	0.343934	0.1513194	0.09325216	0.142822	0.1367527
337.404	3	0.506127	0.3449748	0.1575254	0.09459834	0.144134	0.1391079
337.504	3	0.506468	0.3406957	0.1603937	0.09264878	0.143349	0.1373083
337.604	3	0.508648	0.3360556	0.1573269	0.0897963	0.147114	0.1399948
337.704	3	0.523384	0.3365364	0.1634711	0.09283888	0.146997	0.1421604
337.804	3	0.5264	0.3343543	0.1611323	0.09397798	0.142187	0.1421366
337.904	3	0.523562	0.3366171	0.1609746	0.0884957	0.140895	0.1451285
338.004	3	0.536527	0.3348293	0.1596193	0.09180912	0.147209	0.1399349
338.104	3	0.51755	0.3373183	0.1593486	0.09824371	0.148743	0.1390591
338.204	3	0.514862	0.3417599	0.1558727	0.09509336	0.15104	0.1422923
338.304	3	0.490173	0.3323792	0.1558079	0.09615938	0.148911	0.1407886
338.404	3	0.52163	0.3372669	0.1540162	0.09379365	0.147068	0.1418475
338.504	3	0.511358	0.3400448	0.156457	0.09467021	0.147252	0.1434576
338.604	3	0.510254	0.3405928	0.1600679	0.09496776	0.145798	0.1404237
338.704	3	0.5022	0.3438139	0.1604185	0.09314314	0.142834	0.1386334
338.804	3	0.507856	0.3411972	0.1582022	0.09405688	0.144814	0.1366832
338.904	3	0.504688	0.3490654	0.1635972	0.09528322	0.145295	0.1363515
339.004	3	0.506137	0.347159	0.163826	0.09872707	0.145073	0.1374514
339.104	3	0.509933	0.3481461	0.1608375	0.09879567	0.139779	0.135069

339.204	3	0.512794	0.3455765	0.1606868	0.1004952	0.139936	0.137644
339.304	3	0.512111	0.3454268	0.1692309	0.09715635	0.146577	0.1390084
339.404	3	0.526107	0.3508097	0.1688482	0.09678364	0.151171	0.1345163
339.504	3	0.512639	0.3512437	0.1608103	0.09660271	0.153599	0.1331393
339.604	3	0.505798	0.3455477	0.1650292	0.0959959	0.150131	0.1283626
339.704	3	0.513579	0.3494432	0.1537921	0.09562784	0.147252	0.1350995
339.804	3	0.515642	0.3466159	0.1522775	0.09830833	0.14399	0.1336037
339.904	3	0.5169	0.3468564	0.160072	0.09818027	0.143758	0.1375644
340.004	3	0.504083	0.3440723	0.1623338	0.0990332	0.142063	0.1370643
340.104	3	0.51124	0.3503473	0.165821	0.1013299	0.143366	0.1405072
340.204	3	0.509031	0.3468219	0.1625107	0.1011393	0.143929	0.1433012
340.304	3	0.505673	0.3492174	0.1580601	0.1017571	0.140136	0.1502061
340.404	3	0.519373	0.3454545	0.1590821	0.1042313	0.139489	0.1506508
340.504	3	0.50167	0.348803	0.1624618	0.1088073	0.141239	0.1602479
340.604	3	0.52003	0.3452436	0.1674859	0.1150603	0.122609	0.1608871
340.704	3	0.506583	0.3468966	0.1618035	0.1122248	0.12339	0.1550191
340.804	3	0.51274	0.3451816	0.1649733	0.1120081	0.130699	0.161823
340.904	3	0.498183	0.3455799	0.1616676	0.1162601	0.130387	0.1555938
341.004	3	0.512712	0.3450987	0.1630978	0.1181784	0.127986	0.1586825
341.104	3	0.521215	0.3461351	0.1620664	0.1171677	0.136933	0.1567987
341.204	3	0.510703	0.3485866	0.1617243	0.1131299	0.137807	0.1545058
341.304	3	0.507151	0.3511643	0.1688326	0.1156388	0.136857	0.1489844
341.404	3	0.517498	0.3477404	0.1640122	0.1182086	0.141903	0.156689
341.504	3	0.515628	0.3493021	0.1671676	0.1183577	0.141717	0.1548359
341.604	3	0.511517	0.3483569	0.1649719	0.1195368	0.139132	0.1520843
341.704	3	0.512384	0.3489945	0.1697574	0.1228377	0.141276	0.1558761
341.804	3	0.515313	0.347147	0.1701619	0.1212009	0.140122	0.152362
341.904	3	0.511752	0.3459512	0.1686409	0.1249146	0.140728	0.155255
342.004	3	0.512219	0.3457764	0.1707644	0.122293	0.143643	0.1520823
342.104	3	0.51135	0.3499599	0.1697241	0.122263	0.139263	0.1524495
342.204	3	0.523363	0.3444157	0.1657409	0.1208889	0.141731	0.1463811
342.304	3	0.503251	0.3508853	0.1680825	0.1200472	0.141097	0.1498001
342.404	3	0.51374	0.3439614	0.1715786	0.1205578	0.140182	0.1495187
342.504	3	0.501603	0.3441223	0.1659424	0.1217998	0.146033	0.1484513
342.604	3	0.510925	0.344988	0.1686019	0.1214156	0.142717	0.1500391
342.704	3	0.504982	0.3440621	0.1717862	0.119934	0.139746	0.1452027
342.804	3	0.513762	0.3496527	0.1678924	0.1228932	0.13964	0.1400079
342.904	3	0.516372	0.3415836	0.1627249	0.1242118	0.137681	0.1448325
343.004	3	0.509322	0.3416195	0.1606506	0.1213442	0.138044	0.1428871
343.104	3	0.503255	0.3442268	0.1689109	0.1218792	0.13811	0.1430861
343.204	3	0.504459	0.3438453	0.1684753	0.1234282	0.13872	0.1450329
343.304	3	0.502212	0.3459382	0.1764994	0.125308	0.1406	0.140908
343.404	3	0.497892	0.3466868	0.1812005	0.1255608	0.140133	0.1428142
343.504	3	0.483853	0.3514962	0.1756172	0.1270357	0.142765	0.1374041
343.604	3	0.474129	0.3501694	0.1695107	0.1250463	0.143281	0.1429203
343.704	3	0.474283	0.350641	0.1683353	0.1246249	0.14123	0.1488743
343.804	3	0.474494	0.346402	0.1656386	0.1278167	0.140169	0.1523565
343.904	3	0.5083	0.3502027	0.1653018	0.1264491	0.142465	0.1538442
344.004	3	0.511079	0.3484646	0.1687032	0.1243422	0.140938	0.1514059
344.104	3	0.504427	0.3545136	0.171957	0.1162152	0.140067	0.1493742
344.204	3	0.494902	0.34949	0.1722422	0.117727	0.142552	0.1479692
344.304	3	0.505687	0.3519068	0.1686473	0.115835	0.139889	0.1448423
344.404	3	0.518813	0.3482213	0.1717935	0.1097087	0.14098	0.1443882
344.504	3	0.507092	0.3490298	0.1759221	0.105493	0.13946	0.1537502
344.604	3	0.504612	0.3487616	0.1722926	0.1078929	0.135997	0.1559793
344.704	3	0.502909	0.3465962	0.1713557	0.1049124	0.139148	0.1565787
344.804	3	0.534994	0.3495726	0.162163	0.1026962	0.1377	0.1495586
344.904	3	0.507903	0.3430458	0.1622221	0.1089627	0.139063	0.1548429
345.004	3	0.496049	0.3417505	0.1633198	0.1019946	0.140258	0.1526591
345.104	3	0.501567	0.3406514	0.1692009	0.1029113	0.138633	0.1514003
345.204	3	0.491829	0.3445813	0.1773401	0.1055505	0.138275	0.1512804
345.304	3	0.477496	0.3445029	0.1785148	0.106955	0.141506	0.1478955
345.404	3	0.468683	0.3405802	0.176214	0.1034181	0.136292	0.1566147
345.504	3	0.478975	0.3395984	0.1752905	0.1042813	0.136706	0.1488903
345.604	3	0.485737	0.339339	0.1792537	0.1030516	0.120604	0.1577673
345.704	3	0.474582	0.3425461	0.1771281	0.1036393	0.131833	0.1657818
345.804	3	0.492992	0.3366957	0.175577	0.1072232	0.127697	0.1748552
345.904	3	0.479768	0.3355944	0.1687091	0.1021197	0.125754	0.1711036
346.004	3	0.492783	0.3375256	0.1643022	0.09897691	0.126255	0.1738442
346.104	3	0.521312	0.3462491	0.1648381	0.09645028	0.126659	0.1781942
346.204	3	0.513085	0.3391362	0.1644069	0.09570824	0.126031	0.1812441
346.304	3	0.484153	0.33448	0.1694031	0.09747878	0.125858	0.1740686
346.404	3	0.506164	0.3381395	0.1668496	0.09603911	0.129105	0.1750127
346.504	3	0.51282	0.3384096	0.1716252	0.09797637	0.126722	0.1753966
346.604	3	0.515349	0.3364503	0.1788387	0.1003505	0.124694	0.1767067
346.704	3	0.511602	0.3394879	0.1795505	0.09870468	0.126813	0.1753254
346.804	3	0.513623	0.3389739	0.167064	0.09554464	0.127886	0.169658
346.904	3	0.504271	0.3312431	0.1601672	0.1014714	0.129083	0.1733449
347.004	3	0.51221	0.3359265	0.1571706	0.09618668	0.126687	0.1761257
347.104	3	0.50982	0.339641	0.1586706	0.0962687	0.126784	0.1760814
347.204	3	0.521117	0.3406915	0.1639922	0.0960831	0.128137	0.1766028
347.304	3	0.514879	0.3403163	0.1700417	0.1019246	0.127316	0.1720393
347.404	3	0.516443	0.3466308	0.1662197	0.1013971	0.127811	0.1764368
347.504	3	0.527587	0.3385236	0.163082	0.09606639	0.131335	0.1717982
347.604	3	0.524504	0.333754	0.1652626	0.09488367	0.130818	0.1727679
347.704	3	0.531862	0.3227355	0.1651077	0.100029	0.130925	0.1722921
347.804	3	0.523929	0.3363758	0.1652869	0.09876449	0.131053	0.1756847
347.904	3	0.507535	0.336417	0.1653986	0.09807105	0.131903	0.1761642
348.004	3	0.508626	0.3288701	0.1633782	0.09473348	0.135978	0.1798186
348.104	3	0.516167	0.3279578	0.1651061	0.09728619	0.137524	0.1777525
348.204	3	0.51236	0.3359158	0.1630442	0.09895786	0.130823	0.1716238
348.304	3	0.515775	0.3302197	0.1618314	0.09547692	0.131048	0.166786
348.404	3	0.506426	0.3250778	0.1629833	0.09535148	0.133139	0.1685758
348.504	3	0.512386	0.33002	0.1591428	0.09536223	0.13374	0.1651967
348.604	3	0.494855	0.3366881	0.1601034	0.09592778	0.132526	0.1660781

348.704	3	0.51253	0.338629	0.1690369	0.09286649	0.134283	0.167043
348.804	3	0.514611	0.3335556	0.1686008	0.09435896	0.135889	0.1672344
348.904	3	0.51911	0.3432343	0.1668685	0.09466974	0.131925	0.1663428
349.004	3	0.525853	0.3433947	0.153681	0.08803818	0.136632	0.1713472
349.104	3	0.526547	0.3383229	0.1551742	0.08624481	0.135242	0.1750204
349.204	3	0.518483	0.3398079	0.1654399	0.0934606	0.133228	0.1706062
349.304	3	0.511607	0.3435317	0.1647517	0.09428389	0.135415	0.1690171
349.404	3	0.515652	0.3501291	0.1626777	0.09662615	0.134689	0.1749868
349.504	3	0.518442	0.3589514	0.1649769	0.09574702	0.136607	0.1726021
349.604	3	0.514148	0.3461592	0.1669626	0.09625804	0.136468	0.1719491
349.704	3	0.5241	0.3416191	0.1656768	0.09964065	0.136533	0.1672121
349.804	3	0.516677	0.3544533	0.1664621	0.0845993	0.138312	0.1686807
349.904	3	0.508243	0.3597682	0.1692095	0.08022103	0.136668	0.1734286
350.004	3	0.503461	0.3432721	0.1646732	0.08899532	0.136597	0.176225
350.104	3	0.522014	0.3440954	0.1726303	0.08987781	0.135728	0.1731541
350.204	3	0.51655	0.3561828	0.1695307	0.09306184	0.138001	0.1711635
350.304	3	0.509571	0.357864	0.1654958	0.09034298	0.137971	0.1715121
350.404	3	0.510371	0.3514453	0.16658	0.08939498	0.14048	0.1635178
350.504	3	0.516353	0.3473083	0.1628448	0.09529102	0.141434	0.1703296
350.604	3	0.523907	0.3483843	0.1447311	0.09833048	0.138977	0.1723312
350.704	3	0.51914	0.3505713	0.1408228	0.100585	0.137753	0.1683469
350.804	3	0.523276	0.357253	0.1534936	0.09984643	0.139873	0.1639603
350.904	3	0.51504	0.3457279	0.1576346	0.09128918	0.143612	0.1570281
351.004	3	0.51248	0.3518755	0.1565913	0.08402529	0.138731	0.1576338
351.104	3	0.513653	0.3584284	0.1594011	0.08659637	0.138968	0.1627757
351.204	3	0.506163	0.3545665	0.1602934	0.08905548	0.140276	0.1626469
351.304	3	0.51562	0.3448458	0.1647459	0.09104041	0.144004	0.1610626
351.404	3	0.501052	0.351075	0.1649095	0.09505384	0.147151	0.1611267
351.504	3	0.517483	0.3536572	0.1742217	0.09893122	0.145995	0.1622958
351.604	3	0.495661	0.3471701	0.1685982	0.09580313	0.151297	0.160771
351.704	3	0.515483	0.3512339	0.1702713	0.09269878	0.150319	0.1610249
351.804	3	0.518551	0.3532636	0.1704017	0.08609731	0.153905	0.1589594
351.904	3	0.516621	0.3581142	0.1649574	0.08093426	0.149581	0.1583382
352.004	3	0.526399	0.3495716	0.1635081	0.08734525	0.143885	0.1577916
352.104	3	0.519756	0.3480704	0.1695468	0.08947163	0.147903	0.1590795
352.204	3	0.520667	0.3462445	0.1703124	0.08815954	0.148119	0.1591196
352.304	3	0.526546	0.3532591	0.1671457	0.09356218	0.14859	0.157522
352.404	3	0.543746	0.3539525	0.1646788	0.0940192	0.1498	0.1564584
352.504	3	0.542843	0.3585421	0.1672086	0.09477267	0.149365	0.1580546
352.604	3	0.526097	0.3524357	0.1668132	0.0929592	0.145661	0.1568417
352.704	3	0.536422	0.3560167	0.1678587	0.09470202	0.148634	0.1567448
352.804	3	0.537207	0.3585967	0.1736181	0.09374062	0.149201	0.155091
352.904	3	0.522345	0.3538421	0.1668712	0.09405638	0.149052	0.1568796
353.004	3	0.514596	0.3547796	0.1569184	0.09330885	0.150127	0.1546892
353.104	3	0.545081	0.3540217	0.1647719	0.09819217	0.153119	0.1558957
353.204	3	0.533754	0.3518875	0.1601719	0.09862636	0.153369	0.1558258
353.304	3	0.531384	0.3513207	0.1580907	0.09683164	0.153301	0.1559057
353.404	3	0.518302	0.3494671	0.1605323	0.09624533	0.15316	0.1573064
353.504	3	0.510906	0.3509745	0.1660813	0.09751767	0.152476	0.1562485
353.604	3	0.518415	0.3477574	0.164529	0.08822992	0.159527	0.1575205
353.704	3	0.501715	0.3522218	0.158236	0.09377269	0.157287	0.1588339
353.804	3	0.503056	0.345771	0.162793	0.0983731	0.156645	0.1575162
353.904	3	0.515272	0.3504723	0.1620602	0.09716737	0.159512	0.1576438
354.004	3	0.507048	0.3502854	0.1575911	0.09782795	0.155785	0.156085
354.104	3	0.512984	0.3583829	0.1594797	0.0984595	0.151338	0.1581847
354.204	3	0.526402	0.3546189	0.1645885	0.1018158	0.151229	0.1564059
354.304	3	0.51348	0.3505442	0.1671903	0.1006597	0.150099	0.1546974
354.404	3	0.50222	0.3501063	0.1629512	0.09951555	0.142841	0.1499375
354.504	3	0.50304	0.3509467	0.157867	0.0993938	0.141364	0.1557831
354.604	3	0.507386	0.349114	0.1674069	0.09072833	0.149167	0.1555367
354.704	3	0.517499	0.3500756	0.1689956	0.08637369	0.150162	0.1565912
354.804	3	0.509877	0.3430184	0.1639417	0.09437585	0.151507	0.1579624
354.904	3	0.509764	0.3441695	0.1661753	0.09283175	0.150353	0.1562332
355.004	3	0.501504	0.3430154	0.1665634	0.09355525	0.153595	0.1551652
355.104	3	0.521695	0.3428755	0.166173	0.09560473	0.151604	0.1545163
355.204	3	0.50784	0.3437094	0.1659551	0.09033798	0.146858	0.1551743
355.304	3	0.510367	0.3426024	0.164098	0.09388752	0.144674	0.1548779
355.404	3	0.513836	0.3409973	0.1674649	0.09513965	0.147938	0.1534391
355.504	3	0.512012	0.3484543	0.1698376	0.09943078	0.147261	0.1547336
355.604	3	0.512231	0.344185	0.1738813	0.09925199	0.142147	0.1541371
355.704	3	0.505377	0.3477601	0.1695659	0.09726314	0.144871	0.1527756
355.804	3	0.510914	0.352674	0.1647425	0.09308168	0.147604	0.1551038
355.904	3	0.515734	0.3460011	0.16007	0.09673896	0.152165	0.1542427
356.004	3	0.525083	0.3443502	0.1600074	0.0967005	0.152856	0.1544821
356.104	3	0.515337	0.3442003	0.1580926	0.09740081	0.150548	0.1548646
356.204	3	0.524619	0.3502204	0.156682	0.09454162	0.149582	0.1569464
356.304	3	0.514525	0.3421968	0.1533871	0.08804852	0.146805	0.1563253
356.404	3	0.513707	0.3430011	0.1579284	0.09214606	0.14741	0.1563554
356.504	3	0.520259	0.3449624	0.1571677	0.09272981	0.146459	0.156196
356.604	3	0.522361	0.3440665	0.1601819	0.09475186	0.151656	0.154849
356.704	3	0.510829	0.3434928	0.1593887	0.09679363	0.154028	0.1546378
356.804	3	0.51126	0.3442656	0.1520279	0.09469085	0.15028	0.1565121
356.904	3	0.536799	0.3426827	0.1500737	0.09206182	0.149058	0.1542598
357.004	3	0.50362	0.3478582	0.1521018	0.08587457	0.153684	0.1527911
357.104	3	0.504739	0.3476411	0.1551036	0.09216403	0.150465	0.1353903
357.204	3	0.50164	0.3432837	0.1568047	0.08920874	0.150928	0.1383441
357.304	3	0.500289	0.3463072	0.1522238	0.08998558	0.147855	0.1345895
357.404	3	0.50799	0.3459788	0.1559514	0.09156472	0.150632	0.1321185
357.504	3	0.502896	0.3423231	0.1580769	0.0892028	0.149798	0.1340479
357.604	3	0.507827	0.336945	0.1546001	0.08810451	0.153787	0.1323574
357.704	3	0.509132	0.3371028	0.1621766	0.09106136	0.152448	0.1373022
357.804	3	0.520621	0.3405239	0.1603208	0.09484204	0.146654	0.1341285
357.904	3	0.513713	0.3388125	0.1605633	0.09001221	0.144908	0.1379222
358.004	3	0.533638	0.3350895	0.1589831	0.08936413	0.151637	0.1337706
358.104	3	0.516424	0.3414887	0.1582391	0.09724739	0.151578	0.1345221

358.204	3	0.514591	0.3420229	0.153572	0.09691513	0.155946	0.137448
358.304	3	0.495946	0.3330325	0.1560904	0.09886834	0.151232	0.1351352
358.404	3	0.508977	0.3371601	0.1530188	0.09675671	0.147495	0.1349976
358.504	3	0.516568	0.3402998	0.1563767	0.09575979	0.150051	0.1395828
358.604	3	0.504922	0.3448673	0.1603319	0.09753589	0.146398	0.1375277
358.704	3	0.501366	0.3419503	0.1596252	0.09554362	0.145908	0.1359158
358.804	3	0.499908	0.3401537	0.1589543	0.09423959	0.147017	0.136969
358.904	3	0.501934	0.3488979	0.1616405	0.09811776	0.149557	0.1356118
359.004	3	0.508025	0.3464434	0.1645697	0.1008109	0.146324	0.1362218
359.104	3	0.50906	0.3504163	0.1629141	0.1010427	0.141716	0.132832
359.204	3	0.522118	0.3443123	0.161657	0.1005728	0.144926	0.1356492
359.304	3	0.507864	0.3464158	0.1694546	0.100245	0.149746	0.1373781
359.404	3	0.505934	0.3538129	0.1715477	0.0953386	0.151574	0.129362
359.504	3	0.513547	0.3523897	0.1623149	0.09823985	0.154061	0.1324236
359.604	3	0.514322	0.3466796	0.1661296	0.09668566	0.153724	0.1310408
359.704	3	0.508468	0.3499969	0.1608326	0.09785754	0.147254	0.1370921
359.859	3.5	0.511194	0.3458112	0.1526133	0.09893606	0.146571	0.1332571
359.959	3.5	0.508112	0.3502863	0.1595006	0.09872055	0.145418	0.1379029
360.059	3.5	0.50335	0.3465716	0.1610875	0.098675	0.14432	0.1347731
360.159	3.5	0.508659	0.3487129	0.1663379	0.1029304	0.144358	0.1392798
360.259	3.5	0.50524	0.3457487	0.1658282	0.1083721	0.144069	0.1431001
360.359	3.5	0.513456	0.3447782	0.1605615	0.1132671	0.14336	0.1486241
360.459	3.5	0.51002	0.3439797	0.1583122	0.1094149	0.144345	0.1497227
360.559	3.5	0.500217	0.3448362	0.1602566	0.1140265	0.13753	0.1612047
360.659	3.5	0.503231	0.3434605	0.1667806	0.1131308	0.11872	0.1607645
360.759	3.5	0.519599	0.3445957	0.1624954	0.1145291	0.12799	0.1573882
360.859	3.5	0.501529	0.3492943	0.158775	0.1103666	0.13364	0.1617671
360.959	3.5	0.510002	0.3518567	0.1614651	0.1137939	0.130243	0.1492569
361.059	3.5	0.505999	0.3478188	0.1613096	0.1154654	0.131016	0.1558548
361.159	3.5	0.502082	0.3470968	0.1660044	0.1150041	0.140146	0.1536235
361.259	3.5	0.509914	0.3478831	0.1628617	0.1189353	0.140002	0.1514647
361.359	3.5	0.51215	0.3467644	0.1620858	0.1155501	0.138805	0.1533882
361.459	3.5	0.506154	0.3444616	0.1670042	0.1187539	0.144247	0.1529707
361.559	3.5	0.502629	0.3430825	0.1671813	0.1165875	0.139606	0.155872
361.659	3.5	0.519579	0.3509548	0.1635389	0.1180182	0.139296	0.1525059
361.759	3.5	0.501133	0.3422245	0.1671621	0.1181942	0.141411	0.1449306
361.859	3.5	0.510732	0.3495602	0.1634853	0.1185228	0.139641	0.1483333
361.959	3.5	0.508507	0.3415522	0.1637613	0.1191693	0.140528	0.1471366
362.059	3.5	0.502457	0.3454971	0.1673683	0.1192264	0.143128	0.1556983
362.159	3.5	0.509261	0.3412615	0.1638571	0.1184469	0.13875	0.147804
362.259	3.5	0.505668	0.3491924	0.1681354	0.12148	0.139801	0.1413231
362.359	3.5	0.506647	0.338838	0.1705506	0.1200651	0.13885	0.1432368
362.459	3.5	0.503883	0.343535	0.164376	0.1204018	0.140445	0.1422172
362.559	3.5	0.503692	0.3402059	0.1601585	0.1226791	0.143782	0.14526
362.659	3.5	0.496089	0.3442273	0.1651702	0.1268403	0.141779	0.1424463
362.759	3.5	0.480041	0.3432939	0.1681384	0.1271564	0.13908	0.1456929
362.859	3.5	0.470385	0.3466786	0.1773629	0.1269419	0.138838	0.1393043
362.959	3.5	0.466215	0.3465479	0.1775389	0.125139	0.138302	0.1444128
363.059	3.5	0.474814	0.3475524	0.170355	0.1297165	0.138432	0.1484483
363.159	3.5	0.50059	0.3415041	0.1657728	0.1282468	0.139885	0.1478857
363.259	3.5	0.501294	0.3465898	0.1617225	0.1231537	0.138485	0.1488142
363.359	3.5	0.491295	0.3494192	0.1594214	0.1125565	0.139654	0.1454221
363.459	3.5	0.506854	0.3476157	0.1646868	0.1149319	0.138122	0.1479429
363.559	3.5	0.507049	0.3504628	0.169354	0.1081184	0.141432	0.1411424
363.659	3.5	0.504993	0.3459657	0.1697829	0.1029034	0.142214	0.1444518
363.759	3.5	0.499115	0.3455674	0.1691896	0.1068028	0.140159	0.1505935
363.859	3.5	0.531896	0.3431919	0.1731636	0.1013097	0.138443	0.155057
363.959	3.5	0.505872	0.3444467	0.1723002	0.1027787	0.140305	0.1444068
364.059	3.5	0.505398	0.3384817	0.1682036	0.1019713	0.140056	0.148034
364.159	3.5	0.501323	0.3385298	0.1601817	0.09708948	0.139742	0.149305
364.259	3.5	0.486494	0.3397874	0.1609772	0.09976672	0.14072	0.1455741
364.359	3.5	0.471158	0.3455011	0.1642372	0.101592	0.13887	0.1448337
364.459	3.5	0.485092	0.3402202	0.1704288	0.09974334	0.139493	0.1444485
364.559	3.5	0.482584	0.336622	0.1731088	0.09881788	0.138142	0.1496899
364.659	3.5	0.472632	0.3358903	0.1764228	0.09916059	0.135927	0.150463
364.759	3.5	0.498504	0.332787	0.1795135	0.09959631	0.137755	0.160283
364.859	3.5	0.486274	0.3365315	0.1781444	0.09560571	0.133835	0.1671922
364.959	3.5	0.501962	0.3310705	0.1763001	0.09354197	0.134411	0.1656338
365.059	3.5	0.508151	0.3386626	0.1664123	0.09113052	0.136388	0.1700572
365.159	3.5	0.494574	0.3347029	0.1627258	0.09248514	0.135586	0.1713266
365.259	3.5	0.50806	0.333149	0.1614146	0.09335749	0.134564	0.1676781
365.359	3.5	0.508816	0.3336343	0.1624611	0.09286706	0.136611	0.1651746
365.459	3.5	0.501541	0.3356445	0.1652546	0.09664307	0.131539	0.1687978
365.559	3.5	0.507709	0.3355814	0.1679847	0.09721271	0.133974	0.1698726
365.659	3.5	0.50258	0.3339148	0.1745695	0.09388496	0.117807	0.164412
365.759	3.5	0.504358	0.3261987	0.175857	0.09695729	0.130415	0.1606531
365.859	3.5	0.50538	0.3344671	0.1602829	0.09175855	0.127944	0.1633357
365.959	3.5	0.510473	0.3371812	0.1601189	0.09266067	0.126043	0.1648659
366.059	3.5	0.511092	0.336371	0.1595042	0.09582248	0.12598	0.1657801
366.159	3.5	0.512042	0.3416421	0.1633295	0.1001409	0.127341	0.1627452
366.259	3.5	0.525144	0.3353994	0.1656063	0.0953237	0.125781	0.1631808
366.359	3.5	0.519176	0.3237892	0.1627939	0.09407014	0.124832	0.1657471
366.459	3.5	0.514078	0.3282214	0.1639438	0.09708472	0.127145	0.1639084
366.559	3.5	0.50287	0.3337508	0.1646128	0.09416209	0.127097	0.1618043
366.659	3.5	0.506522	0.3254309	0.1650306	0.09144602	0.124491	0.1586081
366.759	3.5	0.514384	0.320226	0.1625316	0.09223536	0.126786	0.1596426
366.859	3.5	0.503804	0.3253833	0.1605887	0.09502962	0.128378	0.1607963
366.959	3.5	0.507514	0.3239081	0.163002	0.09124738	0.128728	0.1582557
367.059	3.5	0.509853	0.3211374	0.1622781	0.09159409	0.127219	0.1614844
367.159	3.5	0.499017	0.326566	0.1657359	0.09286437	0.1292	0.1626146
367.259	3.5	0.513508	0.3324759	0.1575864	0.09108148	0.130358	0.1617576
367.359	3.5	0.514907	0.3261484	0.1603436	0.09308461	0.128664	0.1630657
367.459	3.5	0.518922	0.3344269	0.1679584	0.09223554	0.12944	0.1619237
367.559	3.5	0.526222	0.3342131	0.1677662	0.0843094	0.129344	0.16013
367.659	3.5	0.510871	0.3321631	0.1572929	0.08691992	0.12919	0.1608669

367.759	3.5	0.510142	0.3324902	0.1547733	0.09116238	0.130349	0.1619991
367.859	3.5	0.510438	0.3427311	0.1645091	0.09308838	0.130393	0.155452
367.959	3.5	0.515293	0.3513209	0.162413	0.09281215	0.129022	0.1618638
368.059	3.5	0.525577	0.3409858	0.161275	0.09331934	0.131834	0.1649038
368.159	3.5	0.515484	0.3385137	0.1640952	0.09637863	0.131012	0.1604516
368.259	3.5	0.511577	0.3519333	0.1677759	0.08049642	0.129042	0.1601056
368.359	3.5	0.507591	0.3400798	0.1655826	0.08068006	0.130045	0.1653217
368.459	3.5	0.518635	0.3363322	0.1689936	0.08834181	0.132335	0.1661553
368.559	3.5	0.511099	0.3500294	0.1637401	0.09142446	0.130658	0.1657042
368.659	3.5	0.50982	0.3533215	0.1709192	0.08828969	0.132989	0.168308
368.759	3.5	0.512065	0.3462236	0.163553	0.08664052	0.133525	0.1664728
368.859	3.5	0.522808	0.3418809	0.1626488	0.09527163	0.134062	0.1562103
368.959	3.5	0.518422	0.3442183	0.1638421	0.09721067	0.132463	0.1627338
369.059	3.5	0.515114	0.3509187	0.1428308	0.09910085	0.137723	0.1592768
369.159	3.5	0.516576	0.338075	0.1396133	0.09321344	0.134936	0.1595771
369.259	3.5	0.512919	0.3469398	0.1571045	0.08413605	0.134423	0.1525044
369.359	3.5	0.503994	0.3510277	0.1567928	0.08431547	0.134215	0.1553952
369.459	3.5	0.509268	0.3435961	0.1562237	0.08901653	0.134003	0.1585557
369.559	3.5	0.502228	0.3425752	0.1586253	0.09277005	0.135505	0.1580067
369.659	3.5	0.507692	0.3494793	0.1634678	0.09479076	0.134077	0.1581208
369.759	3.5	0.507509	0.3459277	0.1650648	0.09420135	0.136091	0.158087
369.859	3.5	0.512975	0.3463625	0.1703158	0.09069325	0.136762	0.1570086
369.959	3.5	0.510061	0.3499611	0.166358	0.08385563	0.134968	0.1574627
370.059	3.5	0.515817	0.3517056	0.1710386	0.08267059	0.136572	0.1530598
370.159	3.5	0.517413	0.3485292	0.1664577	0.08694205	0.138543	0.1515925
370.259	3.5	0.517	0.3441643	0.161148	0.08865451	0.139722	0.1546326
370.359	3.5	0.528531	0.3471002	0.1683259	0.09008515	0.14075	0.1553431
370.459	3.5	0.536466	0.3504612	0.167242	0.09593698	0.142102	0.1526823
370.559	3.5	0.524902	0.3536524	0.1644579	0.09535312	0.141777	0.1550442
370.659	3.5	0.529461	0.3462191	0.1620215	0.09279242	0.140626	0.155229
370.759	3.5	0.52464	0.3525724	0.1644342	0.09624745	0.14007	0.1544928
370.859	3.5	0.512852	0.3514752	0.1625054	0.09240833	0.140608	0.1534917
370.959	3.5	0.525739	0.3505642	0.1681877	0.09365293	0.140898	0.1541708
371.059	3.5	0.542359	0.3480031	0.155277	0.09708794	0.136979	0.1529267
371.159	3.5	0.525435	0.3464711	0.1565558	0.09847242	0.138445	0.1536108
371.259	3.5	0.514583	0.3493713	0.1610152	0.09707597	0.139424	0.1532376
371.359	3.5	0.514459	0.3459229	0.158204	0.09640615	0.142422	0.1521388
371.459	3.5	0.510645	0.3407846	0.1617521	0.08984809	0.142885	0.1518164
371.559	3.5	0.50481	0.3472077	0.1664761	0.0891427	0.14503	0.1543244
371.659	3.5	0.509977	0.3427306	0.1630863	0.0954432	0.147447	0.1544177
371.759	3.5	0.508345	0.347746	0.1598576	0.09517967	0.147682	0.1539981
371.859	3.5	0.50961	0.3478353	0.1631983	0.09724683	0.150219	0.1532614
371.959	3.5	0.527748	0.3625081	0.1573288	0.1004214	0.143701	0.1533186
372.059	3.5	0.502181	0.357731	0.1595021	0.1020874	0.141478	0.1544507
372.159	3.5	0.505586	0.3494388	0.1630453	0.1000845	0.144687	0.1550874
372.259	3.5	0.505932	0.3552373	0.163975	0.09772653	0.144502	0.1533314
372.359	3.5	0.514604	0.3509582	0.1580765	0.08398316	0.145793	0.1557553
372.459	3.5	0.506999	0.3518921	0.1577723	0.0899911	0.14363	0.1564543
372.559	3.5	0.504585	0.3421934	0.16745	0.09225767	0.143168	0.1595064
372.659	3.5	0.511061	0.3466908	0.1617172	0.09288857	0.141863	0.1579947
372.759	3.5	0.505242	0.3403032	0.1642168	0.092747	0.143773	0.1576495
372.859	3.5	0.512919	0.3484001	0.1662543	0.08771405	0.144716	0.1570249
372.959	3.5	0.516863	0.3450178	0.1651988	0.09333602	0.14464	0.157244
373.059	3.5	0.509709	0.3437145	0.1650763	0.09721778	0.144616	0.1561834
373.159	3.5	0.519365	0.3483126	0.1666956	0.09834146	0.149678	0.1571826
373.259	3.5	0.507326	0.3462375	0.1713232	0.09588475	0.150767	0.1532972
373.359	3.5	0.515668	0.3491948	0.1736826	0.09222028	0.149119	0.1487897
373.459	3.5	0.523111	0.3475269	0.1670871	0.0946872	0.149544	0.1478821
373.559	3.5	0.517144	0.3384973	0.1648315	0.09484819	0.149897	0.1494215
373.659	3.5	0.523868	0.3414018	0.1610929	0.09454282	0.15693	0.1524315
373.759	3.5	0.512515	0.3481785	0.1596037	0.08704886	0.153212	0.151972
373.859	3.5	0.519999	0.340313	0.1570048	0.08978488	0.152283	0.1480268
373.959	3.5	0.520434	0.3417159	0.151456	0.09261463	0.153819	0.1478043
374.059	3.5	0.512861	0.3416661	0.1541782	0.09339321	0.1523	0.1473499
374.159	3.5	0.522608	0.3411642	0.1551422	0.0940892	0.147214	0.1449609
374.259	3.5	0.522974	0.3402133	0.1604465	0.09216854	0.148246	0.1472588
374.359	3.5	0.505632	0.3417322	0.1530104	0.08506642	0.146858	0.1464542
374.459	3.5	0.501883	0.3442607	0.1472291	0.08986889	0.140391	0.1431849
374.559	3.5	0.503993	0.3401408	0.1475203	0.0885449	0.141539	0.1291828
374.659	3.5	0.50513	0.343771	0.1556389	0.08890192	0.148002	0.1322578
374.759	3.5	0.486074	0.3425291	0.1541187	0.08964498	0.147891	0.1264847
374.859	3.5	0.480006	0.3342219	0.1523817	0.08838639	0.149534	0.1298704
374.959	3.5	0.509565	0.3319087	0.1590796	0.0893288	0.146789	0.1308918
375.059	3.5	0.508835	0.3354902	0.1565618	0.09376086	0.149504	0.1352835
375.159	3.5	0.516964	0.335472	0.1636438	0.08958203	0.150287	0.1330269
375.259	3.5	0.50785	0.3349546	0.1606566	0.09063532	0.145162	0.1346353
375.359	3.5	0.512034	0.3384694	0.160479	0.09808986	0.144455	0.1346651
375.459	3.5	0.492933	0.3357381	0.1592321	0.09719154	0.147686	0.1318714
375.559	3.5	0.51654	0.3323696	0.154471	0.09952093	0.145592	0.1317037
375.659	3.5	0.499985	0.3357432	0.1550362	0.09541745	0.139612	0.1328176
375.759	3.5	0.502027	0.3422247	0.1523322	0.09688113	0.141852	0.1360415
375.859	3.5	0.502399	0.3393916	0.1566018	0.09423756	0.143092	0.1329723
375.959	3.5	0.501814	0.3428867	0.1569281	0.09504134	0.150188	0.1335006
376.059	3.5	0.50479	0.3450468	0.1587561	0.09781227	0.15003	0.1355358
376.159	3.5	0.511548	0.3527422	0.1619506	0.1016164	0.150796	0.1348442
376.259	3.5	0.508661	0.3461459	0.1661338	0.1005433	0.148909	0.1329819
376.359	3.5	0.509303	0.3441254	0.1627488	0.0961908	0.145419	0.1338388
376.459	3.5	0.512966	0.353611	0.1655015	0.09503672	0.144179	0.1316983
376.559	3.5	0.507343	0.3500466	0.1700687	0.0931106	0.14216	0.1258553
376.659	3.5	0.508083	0.3482534	0.1625593	0.09673469	0.144858	0.1290709
376.759	3.5	0.509969	0.3503796	0.1659959	0.09775423	0.148846	0.1318101
376.859	3.5	0.508776	0.3460526	0.1575253	0.09785383	0.146646	0.1342669
376.959	3.5	0.503999	0.3444864	0.1544837	0.09912743	0.145969	0.1371882
377.059	3.5	0.504665	0.3513829	0.1615263	0.1017609	0.14955	0.1344176
377.159	3.5	0.503271	0.3457455	0.16548	0.1016163	0.145959	0.1373697

377.259	3.5	0.510738	0.3484797	0.165263	0.1014536	0.147252	0.1442911
377.359	3.5	0.501069	0.3484789	0.1600472	0.1057408	0.145984	0.1473487
377.459	3.5	0.519199	0.3434303	0.1582327	0.1099689	0.147662	0.1560844
377.559	3.5	0.506291	0.3442672	0.1604708	0.1071734	0.14538	0.1559348
377.659	3.5	0.499065	0.3445323	0.1636204	0.1069588	0.147793	0.1546145
377.759	3.5	0.509789	0.3419364	0.160617	0.1118914	0.150332	0.1569478
377.859	3.5	0.503187	0.3423727	0.1618959	0.1114593	0.145353	0.1560228
377.959	3.5	0.513992	0.3491371	0.1603614	0.1102426	0.141741	0.1579527
378.059	3.5	0.499241	0.3504143	0.1615188	0.1105236	0.147059	0.1548522
378.159	3.5	0.503428	0.350669	0.1621985	0.1159303	0.148079	0.1513861
378.259	3.5	0.510345	0.3482472	0.1658421	0.1150996	0.151363	0.1580894
378.359	3.5	0.504734	0.3473422	0.1633019	0.1180906	0.148675	0.1513114
378.459	3.5	0.506921	0.3464808	0.1619875	0.1178886	0.147111	0.1544625
378.559	3.5	0.501466	0.3449434	0.1667076	0.1174424	0.146339	0.1528926
378.659	3.5	0.502728	0.3405339	0.168371	0.1197357	0.14624	0.157879
378.759	3.5	0.508695	0.3525907	0.1652382	0.1188799	0.142372	0.1532352
378.859	3.5	0.511185	0.347351	0.1689538	0.118664	0.144437	0.1507228
378.959	3.5	0.50274	0.3521896	0.1661732	0.1181346	0.144405	0.1473642
379.059	3.5	0.49726	0.3444016	0.1672263	0.1216525	0.144073	0.1469684
379.159	3.5	0.501609	0.3462771	0.1688302	0.1226809	0.140591	0.1481462
379.259	3.5	0.500012	0.3434337	0.1645826	0.1217088	0.139597	0.1542903
379.359	3.5	0.508131	0.3504741	0.1687104	0.1216313	0.144955	0.1486416
379.459	3.5	0.50492	0.3389901	0.1718377	0.1248129	0.147938	0.1479094
379.559	3.5	0.502754	0.3443508	0.1689663	0.1238507	0.150537	0.1448622
379.659	3.5	0.503368	0.341499	0.1615532	0.1216728	0.14833	0.1444861
379.759	3.5	0.49701	0.3431113	0.1666678	0.1233216	0.147586	0.1469979
379.859	3.5	0.489296	0.3454049	0.1680191	0.1266064	0.142389	0.1449011
379.959	3.5	0.47033	0.3479572	0.1760375	0.1266897	0.141871	0.1417321
380.059	3.5	0.469797	0.3448231	0.176832	0.125793	0.140298	0.143824
380.159	3.5	0.469778	0.3488293	0.1705444	0.1276615	0.141373	0.149817
380.259	3.5	0.479809	0.3403006	0.1665043	0.1317704	0.139599	0.1515345
380.359	3.5	0.506673	0.3487836	0.164541	0.1258806	0.13796	0.1488162
380.459	3.5	0.494924	0.3445557	0.1625361	0.1167764	0.13781	0.1459524
380.559	3.5	0.484168	0.3489484	0.1651117	0.111952	0.138987	0.143848
380.659	3.5	0.506361	0.3516229	0.1684651	0.1118419	0.117963	0.1459324
380.759	3.5	0.494127	0.3450828	0.1676382	0.1026827	0.122268	0.1392449
380.859	3.5	0.503813	0.3448501	0.1667059	0.1031602	0.128297	0.1447711
380.959	3.5	0.492567	0.3473105	0.173053	0.103087	0.127171	0.1507614
381.059	3.5	0.525211	0.3449247	0.1698819	0.1016473	0.123468	0.1458398
381.159	3.5	0.493644	0.3381728	0.1662119	0.1052384	0.135102	0.1466514
381.259	3.5	0.500456	0.3375435	0.1576395	0.09981179	0.136669	0.1476393
381.359	3.5	0.491314	0.3418556	0.1554905	0.09729406	0.135319	0.1448118
381.459	3.5	0.476924	0.3409413	0.1587857	0.1013132	0.140739	0.1458227
381.559	3.5	0.468201	0.337796	0.1658503	0.09875266	0.136973	0.1418274
381.659	3.5	0.485481	0.3375059	0.1701996	0.0971899	0.133147	0.1483433
381.759	3.5	0.467708	0.3349153	0.1734806	0.0993491	0.135592	0.1462988
381.859	3.5	0.469155	0.339986	0.1752499	0.1012027	0.134664	0.1530796
381.959	3.5	0.481414	0.3348804	0.1803448	0.09741436	0.134892	0.1655003
382.059	3.5	0.485162	0.3348213	0.1745222	0.09128412	0.136712	0.1641059
382.159	3.5	0.497951	0.3340028	0.1698136	0.08914597	0.134662	0.1675252
382.259	3.5	0.500781	0.3407276	0.1633821	0.09050058	0.137563	0.1695305
382.359	3.5	0.496633	0.3346899	0.1635061	0.09104985	0.135417	0.1691991
382.459	3.5	0.500296	0.3353269	0.1631987	0.08962729	0.133877	0.167225
382.559	3.5	0.501063	0.3347355	0.1672392	0.08969852	0.141245	0.1709782
382.659	3.5	0.503444	0.3327647	0.1639662	0.09164181	0.136861	0.1696928
382.759	3.5	0.505017	0.3324267	0.1689431	0.09012812	0.134427	0.1651843
382.859	3.5	0.499331	0.3296716	0.1744257	0.09386011	0.136081	0.1600286
382.959	3.5	0.497156	0.3277354	0.1649168	0.08916955	0.133627	0.165606
383.059	3.5	0.505406	0.3343809	0.1557197	0.09100112	0.134698	0.1660808
383.159	3.5	0.502751	0.3315914	0.1536646	0.09021456	0.137065	0.1673859
383.259	3.5	0.504714	0.3326939	0.1579642	0.09337073	0.136526	0.166814
383.359	3.5	0.519487	0.3373285	0.1651648	0.09214375	0.138034	0.1626374
383.459	3.5	0.511288	0.3304578	0.1648939	0.08932107	0.137235	0.1643829
383.559	3.5	0.511338	0.3175009	0.1617307	0.09395206	0.139983	0.1661419
383.659	3.5	0.504229	0.3293202	0.1650756	0.09163624	0.139821	0.1638855
383.759	3.5	0.510391	0.3314656	0.164504	0.08947039	0.139301	0.1582189
383.859	3.5	0.504627	0.3265511	0.1667938	0.09026558	0.137072	0.1558795
383.959	3.5	0.498951	0.3211781	0.1656736	0.0928598	0.137408	0.1581964
384.059	3.5	0.512814	0.3278361	0.1670422	0.0885145	0.138263	0.1512156
384.159	3.5	0.511166	0.3215942	0.1643619	0.08866453	0.135533	0.1560072
384.259	3.5	0.497231	0.321561	0.163347	0.08777398	0.137485	0.157668
384.359	3.5	0.502491	0.3273169	0.1611316	0.08689582	0.13499	0.1581415
384.459	3.5	0.510521	0.3299097	0.1590088	0.08608989	0.135172	0.1581665
384.559	3.5	0.516961	0.3287572	0.1662773	0.08492573	0.133518	0.1602042
384.659	3.5	0.523833	0.3359217	0.166925	0.08053499	0.132707	0.1570249
384.759	3.5	0.516791	0.3335281	0.1647448	0.08500411	0.135397	0.1562839
384.859	3.5	0.508047	0.3329476	0.15215	0.08773468	0.131977	0.1595746
384.959	3.5	0.50982	0.3315512	0.1598598	0.08939978	0.134025	0.1558543
385.059	3.5	0.514939	0.3428281	0.1630673	0.09043688	0.134577	0.1644222
385.159	3.5	0.516429	0.3461789	0.1603509	0.09325133	0.13491	0.1652977
385.259	3.5	0.518034	0.3394783	0.1638277	0.09713718	0.133844	0.1618343
385.359	3.5	0.510589	0.3390754	0.1642202	0.07968581	0.135104	0.1589797
385.459	3.5	0.50865	0.3468851	0.1658021	0.07657987	0.132805	0.1629584
385.559	3.5	0.514183	0.3380121	0.1667718	0.0861226	0.134892	0.1644325
385.659	3.5	0.503508	0.3371819	0.163899	0.08971837	0.119283	0.1632843
385.759	3.5	0.505779	0.3476948	0.1695951	0.08429695	0.129912	0.1698833
385.859	3.5	0.505234	0.3507524	0.16726	0.08310956	0.129988	0.169369
385.959	3.5	0.520129	0.3446769	0.16341	0.09298929	0.126731	0.1578283
386.059	3.5	0.517168	0.3388044	0.1648087	0.0948769	0.127499	0.1593524
386.159	3.5	0.51885	0.341132	0.1537632	0.09717612	0.128518	0.1581703
386.259	3.5	0.515851	0.3469687	0.140781	0.09172733	0.128268	0.1581714
386.359	3.5	0.507109	0.3378974	0.1538245	0.08144907	0.127202	0.1511905
386.459	3.5	0.504312	0.3489502	0.1579266	0.0822434	0.131879	0.1536217
386.559	3.5	0.503371	0.3484244	0.1547759	0.08796456	0.128696	0.1566261
386.659	3.5	0.491985	0.3398812	0.1561051	0.09472015	0.128183	0.1571675

386.759	3.5	0.511043	0.3445889	0.1606039	0.09558739	0.130153	0.1562297
386.859	3.5	0.497043	0.3492513	0.1618394	0.09334859	0.130147	0.1576265
386.959	3.5	0.510651	0.3408998	0.1703538	0.08974747	0.135857	0.1560541
387.059	3.5	0.507907	0.3442813	0.1674289	0.08406071	0.131629	0.1565392
387.159	3.5	0.523674	0.3489412	0.1679635	0.08039325	0.132443	0.1540318
387.259	3.5	0.504425	0.3449048	0.1638639	0.08311351	0.133082	0.1506856
387.359	3.5	0.506979	0.3466046	0.1594569	0.08833607	0.12962	0.1521325
387.459	3.5	0.508964	0.3431261	0.1665239	0.08581686	0.127826	0.1541937
387.559	3.5	0.535376	0.3458598	0.1690306	0.09381893	0.1284	0.1517707
387.659	3.5	0.526493	0.3509954	0.1655812	0.09277371	0.129309	0.1508242
387.759	3.5	0.517987	0.3504318	0.1643112	0.09214109	0.12947	0.1527487
387.859	3.5	0.525758	0.3463116	0.1675656	0.0940984	0.13092	0.1537515
387.959	3.5	0.51739	0.3527181	0.1644303	0.09145683	0.131337	0.1544432
388.059	3.5	0.511855	0.3505944	0.1707468	0.09102467	0.132804	0.1535835
388.159	3.5	0.539697	0.3509475	0.1607843	0.09486768	0.13208	0.1513848
388.259	3.5	0.526216	0.3507296	0.1548712	0.09811675	0.12969	0.1537525
388.359	3.5	0.508785	0.3497579	0.159327	0.09518474	0.126137	0.1523178
388.459	3.5	0.513774	0.3475065	0.1548337	0.09435134	0.133041	0.1518016
388.559	3.5	0.517072	0.3516898	0.1578938	0.09016631	0.131587	0.1499852
388.659	3.5	0.505108	0.3400635	0.1607904	0.08419193	0.131009	0.1503547
388.759	3.5	0.50255	0.3474197	0.1602012	0.09355544	0.129903	0.1517249
388.859	3.5	0.51343	0.3439425	0.1591295	0.09268087	0.129958	0.1517685
388.959	3.5	0.512741	0.3452644	0.1634672	0.09680012	0.128785	0.1505103
389.059	3.5	0.516769	0.3520579	0.1574558	0.09703917	0.130909	0.1508863
389.159	3.5	0.518011	0.3568478	0.1574709	0.1006965	0.129879	0.1514691
389.259	3.5	0.507152	0.3520947	0.1608249	0.1019582	0.129382	0.1527515
389.359	3.5	0.503754	0.3531481	0.1621429	0.09964886	0.130181	0.1499082
389.459	3.5	0.509692	0.3497643	0.1564392	0.09345961	0.129312	0.1521991
389.559	3.5	0.512637	0.3472093	0.1546707	0.0840781	0.129343	0.151547
389.659	3.5	0.506112	0.3458293	0.163434	0.09156191	0.130288	0.1515901
389.759	3.5	0.498508	0.3414726	0.1585853	0.09105218	0.129177	0.1514629
389.859	3.5	0.517256	0.3433305	0.1587988	0.09250522	0.133272	0.149709
389.959	3.5	0.508872	0.3440024	0.1592458	0.08841255	0.129378	0.1517967
390.059	3.5	0.511037	0.3426088	0.1614567	0.09147716	0.129386	0.1525652
390.159	3.5	0.52096	0.3438207	0.1611676	0.09226738	0.131141	0.1503354
390.259	3.5	0.516613	0.3489608	0.1615756	0.09575226	0.134143	0.1503673
390.359	3.5	0.50509	0.3440461	0.1651192	0.09826121	0.135874	0.1484272
390.459	3.5	0.50804	0.3463678	0.1678245	0.09372022	0.138162	0.1459989
390.559	3.5	0.510863	0.3508861	0.1626667	0.09302492	0.140844	0.1457037
390.659	3.5	0.52399	0.3432188	0.1607127	0.09355281	0.140872	0.1442288
390.759	3.5	0.52436	0.3375808	0.1547414	0.09506319	0.139814	0.1448996
390.859	3.5	0.518762	0.3479799	0.1550315	0.08909976	0.138197	0.1458139
390.959	3.5	0.510052	0.3456945	0.1553468	0.08664626	0.138235	0.1482325
391.059	3.5	0.520481	0.3422465	0.1526229	0.08931459	0.140875	0.1462215
391.159	3.5	0.509494	0.3427447	0.1541562	0.09271038	0.144125	0.1482778
391.259	3.5	0.511747	0.3406734	0.1537213	0.09241996	0.143547	0.1472089
391.359	3.5	0.535939	0.3437996	0.1571707	0.09236462	0.14748	0.1498207
391.459	3.5	0.500501	0.3390009	0.1520516	0.08816904	0.147801	0.1490301
391.559	3.5	0.499841	0.3406104	0.151352	0.08625679	0.148502	0.1497381
391.659	3.5	0.503062	0.339453	0.1493123	0.08807605	0.142257	0.1359618
391.759	3.5	0.499023	0.3414782	0.1527181	0.08620657	0.143785	0.1346336
391.859	3.5	0.494195	0.343307	0.1521178	0.08785124	0.145958	0.1291663
391.959	3.5	0.472904	0.3367577	0.1508825	0.08696905	0.145984	0.1288116
392.059	3.5	0.483342	0.3331433	0.1574425	0.08427412	0.144974	0.1286633
392.159	3.5	0.509964	0.3335074	0.153614	0.08967651	0.142015	0.133499
392.259	3.5	0.521442	0.3318761	0.1595372	0.08932221	0.141498	0.1318893
392.359	3.5	0.511053	0.332963	0.1579349	0.08803786	0.143605	0.135637
392.459	3.5	0.509982	0.3322513	0.1583593	0.0949531	0.142997	0.1336982
392.559	3.5	0.501188	0.3378426	0.1573818	0.09835909	0.143244	0.1331525
392.659	3.5	0.51522	0.32902	0.1540833	0.09802789	0.147634	0.1358813
392.759	3.5	0.506701	0.3378628	0.1538238	0.09469587	0.147145	0.1335719
392.859	3.5	0.509444	0.3382408	0.1535214	0.09395829	0.146117	0.1384484
392.959	3.5	0.500433	0.3415269	0.1535496	0.09513877	0.146927	0.1360329
393.059	3.5	0.499795	0.3362103	0.1565435	0.09234212	0.153072	0.1361528
393.159	3.5	0.497907	0.3416045	0.1570614	0.09392881	0.149541	0.1394266
393.259	3.5	0.506368	0.3409594	0.1576723	0.09654251	0.152138	0.1368904
393.359	3.5	0.507206	0.3435451	0.1641688	0.09938087	0.151807	0.1389165
393.459	3.5	0.502698	0.3392513	0.1617569	0.09638678	0.148681	0.1323772
393.559	3.5	0.514977	0.3447534	0.162078	0.09182426	0.145722	0.1369456
393.659	3.5	0.504994	0.3529787	0.1691052	0.09439649	0.146128	0.1274559
393.759	3.5	0.504964	0.3431019	0.1650855	0.0939952	0.138224	0.1313292
393.859	3.5	0.510296	0.3485011	0.1611163	0.09498829	0.141377	0.1302121
393.959	3.5	0.503871	0.3416434	0.1619903	0.09609438	0.149192	0.1356134
394.059	3.5	0.503608	0.3405858	0.1536541	0.09674013	0.149242	0.1335914
394.159	3.5	0.498654	0.3455561	0.1601697	0.1012337	0.146861	0.1348116
394.259	3.5	0.499444	0.3493496	0.1620791	0.1021224	0.14781	0.136794
394.359	3.5	0.507836	0.3483587	0.165567	0.1026079	0.148309	0.1403857
394.459	3.5	0.491696	0.3444071	0.1610112	0.1016584	0.143056	0.1457599
394.559	3.5	0.512651	0.344545	0.1575282	0.1076057	0.143188	0.1512515
394.659	3.5	0.503732	0.3451483	0.159305	0.1075939	0.144046	0.1557966
394.759	3.5	0.503079	0.3409286	0.1655754	0.1036786	0.136632	0.1492536
394.859	3.5	0.509348	0.3400552	0.1624189	0.1107749	0.137242	0.1559102
394.959	3.5	0.500899	0.3409237	0.1641103	0.1095058	0.141542	0.1510894
395.059	3.5	0.512758	0.3414031	0.1600554	0.1088473	0.146978	0.1520788
395.159	3.5	0.496749	0.3475764	0.1632546	0.1069142	0.149366	0.1565021
395.259	3.5	0.500587	0.3483563	0.1593726	0.1142662	0.147567	0.1488801
395.359	3.5	0.502657	0.346411	0.1666823	0.114857	0.147481	0.1550709
395.459	3.5	0.497368	0.3461885	0.162671	0.1167789	0.143956	0.1501654
395.559	3.5	0.500479	0.3467928	0.1645035	0.1196181	0.141856	0.1521624
395.659	3.5	0.49677	0.3444823	0.1633671	0.119543	0.143168	0.1529285
395.759	3.5	0.504474	0.3438746	0.1666785	0.1196607	0.14823	0.1544243
395.859	3.5	0.511252	0.3423771	0.1637797	0.119404	0.145062	0.1537869
395.959	3.5	0.511644	0.3533337	0.1641512	0.1180868	0.147091	0.1522431
396.059	3.5	0.505006	0.34768	0.1648012	0.1192253	0.149471	0.1422945
396.159	3.5	0.497327	0.3538596	0.1614443	0.1177969	0.146168	0.1460207

396.259	3.5	0.497679	0.3474123	0.1636224	0.1204428	0.142975	0.1423501
396.359	3.5	0.496743	0.3477362	0.1637022	0.1215603	0.146008	0.1538766
396.459	3.5	0.506708	0.3406115	0.1626688	0.121042	0.145441	0.151599
396.559	3.5	0.503335	0.3494082	0.1677918	0.1239457	0.149263	0.1486977
396.659	3.5	0.503402	0.3384715	0.1657051	0.124049	0.145111	0.1497404
396.759	3.5	0.503052	0.3448032	0.160095	0.1225386	0.140993	0.1437193
396.859	3.5	0.499574	0.3411845	0.1600031	0.1233596	0.145903	0.1466943
396.959	3.5	0.492234	0.3429276	0.1651324	0.1250175	0.145785	0.1436137
397.059	3.5	0.471599	0.344799	0.1678078	0.1239195	0.148947	0.1507481
397.159	3.5	0.46527	0.346309	0.1746546	0.12438	0.145273	0.140243
397.259	3.5	0.465457	0.3453652	0.1698519	0.1248575	0.143238	0.1428514
397.359	3.5	0.481205	0.3496854	0.1640004	0.126707	0.144809	0.1504312
397.459	3.5	0.498904	0.3416169	0.1623341	0.1237245	0.138487	0.143283
397.559	3.5	0.493168	0.3441433	0.1599178	0.1186269	0.139285	0.1412797
397.659	3.5	0.479802	0.3486705	0.1623532	0.1110165	0.142735	0.1394244
397.759	3.5	0.507191	0.3508927	0.1653596	0.1099124	0.13977	0.1439098
397.859	3.5	0.496774	0.352762	0.164275	0.1030337	0.133722	0.1396334
397.959	3.5	0.501932	0.346616	0.1623667	0.1022504	0.137538	0.1435341
398.059	3.5	0.486008	0.3453891	0.1686901	0.1066748	0.144029	0.1459684
398.159	3.5	0.518538	0.3455227	0.1701316	0.09946834	0.145656	0.1519725
398.259	3.5	0.491577	0.3451234	0.1712067	0.1059568	0.145809	0.143558
398.359	3.5	0.496457	0.3377283	0.1599953	0.1010232	0.140836	0.1488553
398.459	3.5	0.485331	0.3356009	0.1583234	0.09983769	0.139734	0.1475907
398.559	3.5	0.479452	0.3382227	0.1603645	0.1044032	0.13785	0.1449471
398.659	3.5	0.474138	0.338489	0.1671373	0.1022046	0.13825	0.1435022
398.759	3.5	0.486969	0.3312703	0.1693345	0.1010094	0.137582	0.1424317
398.859	3.5	0.4818	0.3301747	0.171054	0.09982865	0.134718	0.1462824
398.959	3.5	0.467788	0.3335162	0.1740388	0.1003052	0.135254	0.1466503
399.059	3.5	0.491589	0.3279971	0.1776945	0.1003924	0.126466	0.1560216
399.159	3.5	0.490555	0.3335326	0.1750124	0.0934387	0.116601	0.1637735
399.259	3.5	0.49497	0.3321955	0.1716554	0.09308177	0.124855	0.1614372
399.359	3.5	0.500653	0.340512	0.1642766	0.09211501	0.124922	0.1646403
399.459	3.5	0.492497	0.338128	0.1620852	0.09118289	0.124326	0.1657036
399.559	3.5	0.503877	0.3304413	0.1612239	0.09229997	0.133363	0.1622307
399.659	3.5	0.49722	0.3304295	0.1633783	0.09244113	0.132855	0.1620456
399.759	3.5	0.498688	0.3316924	0.1605079	0.09172923	0.132744	0.1665767
399.859	3.5	0.503487	0.3341603	0.166327	0.08821891	0.136242	0.1674056
399.959	3.5	0.492963	0.3325138	0.1700761	0.09137753	0.132569	0.1605205
400.059	3.5	0.496709	0.3236324	0.1657335	0.08909452	0.133306	0.1569916
400.159	3.5	0.495224	0.3274731	0.1562971	0.08876092	0.132975	0.1607515
400.259	3.5	0.497394	0.3293713	0.1548145	0.09045286	0.133105	0.1640536
400.359	3.5	0.503132	0.3323402	0.1559143	0.09369466	0.133354	0.1646335
400.459	3.5	0.502733	0.3336704	0.1606287	0.09332111	0.133339	0.1595665
400.559	3.5	0.514305	0.3315745	0.1628658	0.08744712	0.134179	0.1619072
400.659	3.5	0.508401	0.3235111	0.1581719	0.09203364	0.133292	0.1648837
400.759	3.5	0.504992	0.324564	0.159438	0.08825514	0.13732	0.1614597
400.859	3.5	0.502807	0.3533499	0.1577247	0.08892581	0.134189	0.1563905
400.959	3.5	0.507402	0.3483737	0.1589141	0.08887775	0.132132	0.1522705
401.059	3.5	0.505129	0.3440615	0.1592049	0.09086758	0.130343	0.149987
401.159	3.5	0.504084	0.3499134	0.1616039	0.08999328	0.13069	0.1511545
401.259	3.5	0.507673	0.3521953	0.1557419	0.08811062	0.131687	0.1454418
401.359	3.5	0.50834	0.3477599	0.1528746	0.08710107	0.13072	0.1516852
401.459	3.5	0.499912	0.3352952	0.1538548	0.08981123	0.132801	0.1544749
401.559	3.5	0.509141	0.3473763	0.1528869	0.08597291	0.132309	0.1541661
401.659	3.5	0.511102	0.3483714	0.1582361	0.08712127	0.135417	0.1556804
401.759	3.5	0.516243	0.3272542	0.161524	0.08575417	0.135509	0.1547816
401.859	3.5	0.522053	0.3299957	0.162253	0.08072122	0.134623	0.151982
401.959	3.5	0.50763	0.3432264	0.1504029	0.08694106	0.135387	0.1519524
402.059	3.5	0.510105	0.3402294	0.1723517	0.08807152	0.132367	0.1693559
402.159	3.5	0.504549	0.3394251	0.1708054	0.09118076	0.135204	0.1683733
402.259	3.5	0.507341	0.3561272	0.170026	0.09217025	0.133833	0.169681
402.359	3.5	0.520956	0.3498593	0.1695758	0.09645274	0.134379	0.1669481
402.459	3.5	0.515325	0.3473014	0.1679139	0.08599292	0.133002	0.1615649
402.559	3.5	0.509616	0.3462745	0.16819	0.07500547	0.133266	0.1648783
402.659	3.5	0.50811	0.3470658	0.1673032	0.08238821	0.13353	0.1689397
402.759	3.5	0.514385	0.3432396	0.1691121	0.08848916	0.131837	0.1706123
402.859	3.5	0.508327	0.3441704	0.1704297	0.087167	0.134102	0.1716231
402.959	3.5	0.505839	0.3463589	0.1699335	0.07748444	0.13499	0.1714926
403.059	3.5	0.514098	0.333865	0.1677853	0.08924389	0.135034	0.1726627
403.159	3.5	0.518102	0.3462768	0.1680042	0.09356035	0.13669	0.1703371
403.259	3.5	0.514878	0.3516035	0.1734885	0.09494121	0.135157	0.1674875
403.359	3.5	0.512541	0.352882	0.1732401	0.09117968	0.127926	0.1679516
403.459	3.5	0.511843	0.3333586	0.171124	0.08385396	0.128619	0.1690743
403.559	3.5	0.510159	0.3365282	0.171094	0.08089171	0.131399	0.1691209
403.659	3.5	0.495937	0.3404066	0.171227	0.08714963	0.128866	0.1709727
403.759	3.5	0.50064	0.3395188	0.1706084	0.09079263	0.130344	0.1729972
403.859	3.5	0.502371	0.3346744	0.1714061	0.09169418	0.129784	0.1723444
403.959	3.5	0.500391	0.3279135	0.1737709	0.0927628	0.129293	0.172555
404.059	3.5	0.510432	0.337932	0.1722947	0.08941398	0.13087	0.1720895
404.159	3.5	0.507859	0.3512942	0.1707478	0.08309545	0.128271	0.1701399
404.259	3.5	0.518385	0.355727	0.1740557	0.07777631	0.126693	0.1703104
404.359	3.5	0.509351	0.3648587	0.1731714	0.07753915	0.130037	0.1666074
404.459	3.5	0.507708	0.3614046	0.1743171	0.08523774	0.132766	0.1686222
404.559	3.5	0.513446	0.3482558	0.1728718	0.08693114	0.130857	0.1698429
404.659	3.5	0.527923	0.3407344	0.1714808	0.09402532	0.130868	0.1699667
404.759	3.5	0.527052	0.3420131	0.1752303	0.09217744	0.132493	0.1691799
404.859	3.5	0.518913	0.3381265	0.1784144	0.09424342	0.130797	0.1706415
404.959	3.5	0.521347	0.342536	0.1761689	0.09243084	0.127983	0.1685622
405.059	3.5	0.520349	0.3406468	0.1751182	0.09075122	0.13007	0.1585642
405.159	3.5	0.515521	0.3462167	0.1731496	0.0891804	0.131082	0.1614891
405.259	3.5	0.525994	0.3487877	0.1734135	0.09435938	0.133324	0.1642678
405.359	3.5	0.529586	0.3466417	0.1746631	0.09920462	0.130876	0.1662637
405.459	3.5	0.510964	0.3287044	0.1759215	0.09513637	0.132766	0.1664436
405.559	3.5	0.513597	0.3430518	0.1759152	0.09393541	0.13289	0.1668239
405.659	3.5	0.514867	0.346458	0.1751999	0.09168012	0.129154	0.165673

405.759	3.5	0.502294	0.3247416	0.1763981	0.08408936	0.133489	0.1650571
405.859	3.5	0.501012	0.319935	0.1790188	0.09430695	0.131916	0.1648234
405.959	3.5	0.51307	0.336105	0.1794767	0.09330685	0.130964	0.1631854
406.059	3.5	0.50398	0.3329649	0.1788579	0.09528588	0.131495	0.1636684
406.159	3.5	0.510886	0.3319703	0.1795851	0.09587751	0.131087	0.1656112
406.259	3.5	0.515742	0.3523265	0.1780519	0.09706559	0.130666	0.1651865
406.359	3.5	0.503612	0.3468647	0.1802534	0.0995429	0.129595	0.166393
406.459	3.5	0.503514	0.3435679	0.1761856	0.09816099	0.133501	0.1644874
406.559	3.5	0.508115	0.3419366	0.1762279	0.09370578	0.134509	0.1646215
406.659	3.5	0.51096	0.3436972	0.1768396	0.0837045	0.132902	0.1652827
406.759	3.5	0.506554	0.3411272	0.1751224	0.09267498	0.132845	0.1657444
406.859	3.5	0.492903	0.340272	0.1746053	0.0924028	0.132368	0.1670017
406.959	3.5	0.51254	0.3420336	0.1748743	0.0929134	0.13458	0.1669877
407.059	3.5	0.504711	0.3329726	0.1761995	0.08908594	0.132214	0.16579
407.159	3.5	0.50536	0.3451759	0.1783772	0.09239313	0.131096	0.1667708
407.259	3.5	0.516226	0.3475648	0.1808119	0.09100521	0.132906	0.1693082
407.359	3.5	0.50794	0.3507519	0.1781231	0.09541447	0.134177	0.1690768
407.459	3.5	0.498949	0.3284034	0.1780083	0.09661333	0.138259	0.1689834
407.559	3.5	0.501808	0.331318	0.1782983	0.09315402	0.14082	0.1698447
407.659	3.5	0.505397	0.3349782	0.1804154	0.09067018	0.140036	0.1702667
407.759	3.5	0.521102	0.3358482	0.1818353	0.09086816	0.139729	0.1705484
407.859	3.5	0.509733	0.3288085	0.1800804	0.09099527	0.143747	0.1721357
407.959	3.5	0.523034	0.3238849	0.1786208	0.08535437	0.14064	0.1721759
408.059	3.5	0.504934	0.3320218	0.179259	0.0844796	0.139052	0.1707309
408.159	3.5	0.515833	0.3471208	0.1796158	0.08766519	0.140417	0.1728641
408.259	3.5	0.504583	0.3540432	0.1804324	0.09182012	0.144261	0.1743978
408.359	3.5	0.511476	0.3656515	0.1786739	0.09042554	0.144458	0.1730167
408.459	3.5	0.530391	0.3591681	0.1778708	0.09103867	0.147808	0.1736461
408.559	3.5	0.504206	0.3494489	0.1778096	0.09042866	0.150147	0.1752017
408.659	3.5	0.497321	0.3442368	0.1795188	0.08449247	0.151896	0.1719845
408.759	3.5	0.499248	0.3410076	0.1774472	0.08810929	0.147204	0.1719139
408.859	3.5	0.502812	0.3347517	0.17686	0.0871279	0.143631	0.171862
408.959	3.5	0.497344	0.336975	0.1756503	0.08795221	0.145466	0.1706789
409.059	3.5	0.479943	0.3370347	0.1724043	0.08603797	0.145071	0.1707435
409.159	3.5	0.475324	0.3443896	0.1712742	0.08384707	0.145575	0.1693989
409.259	3.5	0.502407	0.3477064	0.1744082	0.08733456	0.143497	0.1676709
409.359	3.5	0.509998	0.3467925	0.1719617	0.09040156	0.141517	0.1686349
409.459	3.5	0.51283	0.3248193	0.1722927	0.08562244	0.144093	0.172087
409.559	3.5	0.503219	0.3410441	0.1712406	0.09269131	0.144952	0.1696511
409.659	3.5	0.50564	0.3460868	0.1785172	0.09674004	0.144237	0.1698929
409.759	3.5	0.503062	0.3223	0.1803893	0.0956389	0.145311	0.1720648
409.859	3.5	0.504935	0.3209823	0.1784211	0.09505051	0.146903	0.1729521
409.959	3.5	0.506635	0.3331622	0.1742447	0.09294417	0.147467	0.1718677
410.059	3.5	0.49845	0.3274387	0.1772159	0.09463753	0.148037	0.170395
410.159	3.5	0.502552	0.3268439	0.1758678	0.09198975	0.149826	0.1699633
410.259	3.5	0.502813	0.3485634	0.1715692	0.09456367	0.160342	0.1686855
410.359	3.5	0.507482	0.3439352	0.1643377	0.09667162	0.153481	0.1664518
410.459	3.5	0.503636	0.3404334	0.1692776	0.09835108	0.157987	0.1674631
410.559	3.5	0.505451	0.340159	0.1712644	0.09737608	0.155054	0.1707877
410.659	3.5	0.515705	0.3411438	0.1694231	0.09324625	0.148628	0.1686301
410.759	3.5	0.501291	0.3406401	0.165115	0.09213441	0.148362	0.1674263
410.859	3.5	0.500667	0.3387606	0.1659294	0.09227902	0.148001	0.1677424
410.959	3.5	0.517058	0.3422295	0.1703555	0.09275484	0.138949	0.1599714
411.059	3.5	0.505543	0.3347652	0.1704002	0.09501395	0.148168	0.1467472
411.159	3.5	0.501141	0.3437931	0.1683675	0.09586515	0.148144	0.151924
411.259	3.5	0.503406	0.3406962	0.1679319	0.09787123	0.14901	0.1572316
411.359	3.5	0.499837	0.3453529	0.1689063	0.1000555	0.1481	0.1579137
411.459	3.5	0.501974	0.321819	0.1706982	0.1010373	0.150756	0.157665
411.559	3.5	0.502887	0.3250977	0.1703229	0.1003689	0.147964	0.1453164
411.659	3.5	0.502514	0.3304312	0.1676542	0.1025748	0.143118	0.1326594
411.759	3.5	0.511899	0.3345405	0.1528812	0.1084588	0.146652	0.142229
411.859	3.5	0.500972	0.3275923	0.1621507	0.1012622	0.146475	0.1474685
411.959	3.5	0.499026	0.3183169	0.1654855	0.1038242	0.142039	0.1474404
412.059	3.5	0.498927	0.3251361	0.1620824	0.109516	0.141716	0.1492111
412.159	3.5	0.519365	0.3481047	0.1506163	0.1078532	0.148938	0.1516054
412.259	3.5	0.494169	0.3522908	0.1544681	0.1077137	0.148692	0.1534335
412.359	3.5	0.512334	0.3623576	0.1530863	0.1112816	0.149552	0.1485894
412.459	3.5	0.497012	0.3569375	0.1576905	0.1157113	0.146537	0.1457516
412.559	3.5	0.497156	0.3463714	0.1587668	0.1156246	0.144019	0.1495869
412.659	3.5	0.498625	0.3385494	0.1504632	0.1205846	0.142027	0.1510065
412.759	3.5	0.498215	0.335995	0.1563895	0.1203472	0.141721	0.1500143
412.859	3.5	0.494661	0.3339265	0.1602814	0.1195235	0.146908	0.1506343
412.959	3.5	0.492761	0.3344186	0.1626046	0.1180319	0.144489	0.1516346
413.059	3.5	0.521812	0.3336217	0.1599289	0.1178045	0.14495	0.1529135
413.159	3.5	0.490408	0.342476	0.1596701	0.1196095	0.14926	0.1538002
413.259	3.5	0.501205	0.3472624	0.1636334	0.1184148	0.145133	0.1540925
413.359	3.5	0.494408	0.3446491	0.1612127	0.1184525	0.14321	0.1541675
413.459	3.5	0.496325	0.3217698	0.1636274	0.1198649	0.143318	0.1536286
413.559	3.5	0.502319	0.3362633	0.168295	0.1206869	0.142797	0.1529896
413.659	3.5	0.505147	0.3454652	0.1653369	0.120375	0.14756	0.1583671
413.759	3.5	0.502189	0.3236914	0.1690806	0.1200286	0.146855	0.1598327
413.859	3.5	0.49695	0.3173479	0.1666034	0.1204032	0.140462	0.1594555
413.959	3.5	0.501556	0.3268723	0.1665181	0.1207209	0.144874	0.1609735
414.059	3.5	0.494322	0.3249933	0.1718066	0.1225335	0.146164	0.1597543
414.159	3.5	0.475008	0.3265117	0.1716623	0.1240659	0.150813	0.1574632
414.259	3.5	0.463102	0.3479056	0.1691047	0.1260889	0.146455	0.1580895
414.359	3.5	0.46168	0.3418701	0.1704469	0.1221555	0.141138	0.1563792
414.459	3.5	0.471015	0.3390945	0.1708488	0.1238333	0.140873	0.1580751
414.559	3.5	0.497882	0.3377185	0.171351	0.1240808	0.13706	0.1583698
414.659	3.5	0.488629	0.3397332	0.1690617	0.1221006	0.139308	0.1592759
414.759	3.5	0.478491	0.3376765	0.1682598	0.1119435	0.140801	0.1597756
414.859	3.5	0.496874	0.3352859	0.1645624	0.1112076	0.139022	0.159486
414.959	3.5	0.501177	0.3417823	0.1654117	0.1066765	0.1344	0.1568222
415.059	3.5	0.5027	0.3329442	0.1687009	0.09885336	0.136196	0.1531461
415.159	3.5	0.49019	0.3400643	0.1670339	0.1028274	0.141608	0.1542644

415.259	3.5	0.512535	0.3309662	0.1648084	0.09982125	0.143764	0.1554857
415.359	3.5	0.496948	0.3400577	0.1666622	0.1022455	0.141956	0.1553135
415.459	3.5	0.497001	0.3195081	0.1658248	0.1037827	0.137625	0.1561659
415.559	3.5	0.493843	0.3257668	0.1670889	0.09540524	0.136533	0.1566147
415.659	3.5	0.485107	0.3252766	0.16879	0.0981643	0.135189	0.153683
415.759	3.5	0.479383	0.3330306	0.1643414	0.1025481	0.136296	0.1562127
415.859	3.5	0.486499	0.3226382	0.1657109	0.1001324	0.136349	0.1567535
415.959	3.5	0.480877	0.3134545	0.161189	0.09972729	0.133601	0.1563315
416.059	3.5	0.46478	0.3171127	0.1615186	0.1004561	0.134776	0.1558549
416.159	3.5	0.485855	0.3477168	0.1643109	0.1009571	0.125857	0.1552957
416.259	3.5	0.482565	0.3471947	0.166318	0.09758144	0.116369	0.152808
416.359	3.5	0.491028	0.3585387	0.1657988	0.09189383	0.128001	0.1531558
416.459	3.5	0.494683	0.3517052	0.162892	0.09216546	0.127071	0.1544376
416.559	3.5	0.479011	0.3410791	0.1623424	0.0914327	0.127775	0.1557224
416.659	3.5	0.502194	0.3341715	0.1623117	0.09094743	0.136438	0.15517
416.759	3.5	0.494445	0.332173	0.1637716	0.09083243	0.133879	0.156421
416.859	3.5	0.493308	0.3313628	0.1619058	0.09276343	0.1341	0.1555922
416.959	3.5	0.497775	0.3323074	0.16107	0.09051301	0.134413	0.1537633
417.059	3.5	0.494404	0.3302884	0.1649785	0.08681372	0.130015	0.1562027
417.159	3.5	0.497739	0.3398526	0.165842	0.09054627	0.131855	0.1549492
417.259	3.5	0.498449	0.3451264	0.1653205	0.08760711	0.130498	0.1531637
417.359	3.5	0.497876	0.3427971	0.1648679	0.09124772	0.132698	0.1538758
417.459	3.5	0.501599	0.3198982	0.1640888	0.08984493	0.132181	0.1535043
417.559	3.5	0.499128	0.332935	0.1659065	0.09243996	0.133114	0.1548093
417.659	3.5	0.513179	0.3434258	0.1638217	0.08833762	0.133447	0.1561174
417.759	3.5	0.507534	0.3147163	0.1662344	0.0878676	0.133078	0.155724
417.859	3.5	0.503935	0.3057889	0.1652915	0.0907031	0.137359	0.1560543
417.959	3.5	0.495746	0.3217764	0.1652295	0.08866626	0.134303	0.156908
418.059	3.5	0.508509	0.3233442	0.1678316	0.08919328	0.131347	0.1556031
418.159	3.5	0.503671	0.3247488	0.1671192	0.08978838	0.130182	0.1549444
418.259	3.5	0.495707	0.3450394	0.164738	0.09235836	0.132161	0.1545599
418.359	3.5	0.504669	0.3393891	0.1658602	0.08840865	0.133262	0.1544759
418.459	3.5	0.511634	0.3371751	0.1603979	0.08662525	0.13275	0.1529637
418.559	3.5	0.491576	0.3339099	0.1613097	0.08756571	0.135014	0.1501636
418.659	3.5	0.505336	0.3368346	0.1645217	0.08963507	0.134	0.1516093
418.759	3.5	0.509518	0.3357373	0.1672453	0.08874092	0.137659	0.1550212
418.859	3.5	0.515419	0.3333797	0.165624	0.08802549	0.138255	0.1523667
418.959	3.5	0.518454	0.3383808	0.1624041	0.08069243	0.135575	0.1520572
419.059	3.5	0.511884	0.3304079	0.1575743	0.08426926	0.135329	0.1515081
419.159	3.5	0.507931	0.3368745	0.1594946	0.08755295	0.132571	0.1543303
419.259	3.5	0.513267	0.3254244	0.1609115	0.08784995	0.13443	0.1537343
419.359	3.5	0.506699	0.3371203	0.1612319	0.09244195	0.134686	0.1546248
419.459	3.5	0.503851	0.3156172	0.1647239	0.09430332	0.135329	0.1557648
419.559	3.5	0.512528	0.3200644	0.1611	0.09776404	0.132359	0.1556188
419.659	3.5	0.516822	0.3194188	0.1608818	0.07865441	0.133609	0.1553467
419.785	4	0.51623	0.3064626	0.1629262	0.06904478	0.135537	0.1580918
419.885	4	0.511533	0.3162989	0.1622701	0.07647484	0.133512	0.1569945
419.985	4	0.507506	0.3454549	0.164582	0.08045008	0.135458	0.1549337
420.085	4	0.493323	0.3405677	0.158784	0.08815202	0.135728	0.1573084
420.185	4	0.489055	0.3523338	0.1565394	0.09363946	0.136046	0.1581865
420.285	4	0.50845	0.3471407	0.1562701	0.09368446	0.137336	0.1570615
420.385	4	0.498758	0.3369455	0.1579159	0.08423301	0.133383	0.1590249
420.485	4	0.514301	0.328633	0.1562566	0.07848366	0.133139	0.1591035
420.585	4	0.513497	0.3298357	0.1563464	0.08677533	0.116884	0.1597073
420.685	4	0.516368	0.331392	0.1557808	0.09164034	0.127139	0.1588984
420.785	4	0.504329	0.329713	0.1463309	0.09238723	0.128797	0.1579452
420.885	4	0.512183	0.3313964	0.148328	0.08764622	0.131027	0.1610565
420.985	4	0.52728	0.3455608	0.1436111	0.08332534	0.132814	0.1612435
421.085	4	0.515957	0.3430029	0.1522865	0.07621926	0.134315	0.1580114
421.185	4	0.520738	0.328624	0.153063	0.07634782	0.134304	0.159422
421.285	4	0.513503	0.3202463	0.1576536	0.08158721	0.134913	0.1604493
421.385	4	0.512617	0.3354134	0.1494111	0.08683074	0.13265	0.1609196
421.485	4	0.521376	0.326723	0.1570776	0.09143096	0.130942	0.162188
421.585	4	0.533629	0.3135804	0.1587613	0.09048557	0.133372	0.1632835
421.685	4	0.506117	0.3146188	0.1594689	0.09158029	0.137805	0.1623878
421.785	4	0.510475	0.3217805	0.1598511	0.08935771	0.137384	0.1607626
421.885	4	0.509723	0.3198776	0.1598421	0.08724158	0.13949	0.1629826
421.985	4	0.506035	0.3415072	0.1577479	0.09274934	0.136803	0.1691425
422.085	4	0.508113	0.3403283	0.1635451	0.09669697	0.134381	0.1708452
422.185	4	0.50491	0.3381781	0.1594503	0.09305844	0.130384	0.1697758
422.285	4	0.50712	0.3359089	0.1611278	0.09243024	0.131357	0.1695904
422.385	4	0.510905	0.3364973	0.1613584	0.08395468	0.133548	0.167595
422.485	4	0.504116	0.3363452	0.1637917	0.08838984	0.134137	0.1645395
422.585	4	0.502402	0.3343825	0.1617039	0.09102277	0.130791	0.1644147
422.685	4	0.512577	0.3366795	0.1619005	0.09317434	0.133663	0.166791
422.785	4	0.510862	0.3284708	0.1628132	0.09630023	0.131117	0.1685494
422.885	4	0.504901	0.3368114	0.1618668	0.0975955	0.12889	0.1697479
422.985	4	0.518224	0.3261902	0.16225	0.09893432	0.132369	0.1708487
423.085	4	0.507428	0.3325237	0.1591288	0.09532499	0.132837	0.1694052
423.185	4	0.514453	0.3123563	0.1594303	0.08339854	0.130414	0.1717974
423.285	4	0.512627	0.3179267	0.1590012	0.09003568	0.131559	0.1718153
423.385	4	0.516146	0.3164374	0.1586656	0.09213786	0.131044	0.1677101
423.485	4	0.505326	0.325049	0.1579643	0.0942279	0.128437	0.1649946
423.585	4	0.506161	0.3188961	0.1613513	0.08810365	0.129577	0.1645673
423.685	4	0.519721	0.3027139	0.1601484	0.09311868	0.13435	0.1654055
423.785	4	0.508803	0.3187242	0.1615312	0.0950872	0.13332	0.1663867
423.885	4	0.522098	0.3467422	0.1612534	0.09757769	0.132077	0.1674304
423.985	4	0.510181	0.3442791	0.1596148	0.09322567	0.134214	0.1689715
424.085	4	0.515994	0.3506282	0.158066	0.09124974	0.135597	0.1662202
424.185	4	0.510492	0.3438035	0.1572591	0.09078171	0.134252	0.1660883
424.285	4	0.521822	0.3313242	0.1602829	0.08712547	0.133173	0.1658769
424.385	4	0.506383	0.3285851	0.1579855	0.08091041	0.134172	0.1640524
424.485	4	0.501191	0.3326064	0.1578007	0.08677658	0.13683	0.1677632
424.585	4	0.50027	0.3323999	0.1577446	0.09219632	0.137986	0.1663414
424.685	4	0.495802	0.3283615	0.1595205	0.09006588	0.142198	0.1650479

424.785	4	0.492042	0.3355398	0.1591978	0.0898445	0.142154	0.1632547
424.885	4	0.480367	0.3454484	0.1592266	0.0836496	0.140579	0.1634787
424.985	4	0.500572	0.3446846	0.1591496	0.08430333	0.142862	0.1623471
425.085	4	0.508539	0.3217802	0.1579897	0.08597943	0.144181	0.1640648
425.185	4	0.506075	0.3299836	0.1589658	0.08718318	0.141085	0.1650205
425.285	4	0.506419	0.3431295	0.160398	0.08496739	0.141054	0.1636448
425.385	4	0.49933	0.3241693	0.1606091	0.08180536	0.142613	0.1616003
425.485	4	0.520534	0.3139213	0.1590841	0.08753406	0.144466	0.1585846
425.585	4	0.506835	0.3251857	0.1570332	0.08564569	0.145807	0.1601759
425.685	4	0.50376	0.3228941	0.1584746	0.08731236	0.14815	0.1611463
425.785	4	0.504408	0.3269619	0.1562731	0.09439152	0.14902	0.1631902
425.885	4	0.506382	0.3436134	0.1579656	0.09288634	0.14814	0.1615191
425.985	4	0.506189	0.3385745	0.1546087	0.09311507	0.1429	0.1619743
426.085	4	0.511983	0.3363749	0.1531088	0.09004905	0.144517	0.1634002
426.185	4	0.51437	0.3341723	0.1557729	0.09150755	0.143787	0.1588022
426.285	4	0.496785	0.3373155	0.1575711	0.09155479	0.144802	0.1603425
426.385	4	0.498625	0.3350553	0.1544512	0.0947051	0.145778	0.1625587
426.485	4	0.515348	0.333176	0.1529641	0.09819153	0.143359	0.1585663
426.585	4	0.503205	0.3360315	0.1538699	0.096886	0.14419	0.1570682
426.685	4	0.503593	0.3252626	0.1531378	0.09297097	0.145731	0.1561563
426.785	4	0.500307	0.3321628	0.1514494	0.09452824	0.145625	0.1570359
426.885	4	0.498021	0.3283288	0.1542311	0.09300709	0.145748	0.1548956
426.985	4	0.504109	0.3229869	0.1544954	0.09393825	0.147752	0.1546396
427.085	4	0.492614	0.3092912	0.1548083	0.09455943	0.147441	0.1572625
427.185	4	0.508494	0.3164981	0.1550133	0.09641557	0.148489	0.1596358
427.285	4	0.492497	0.3209341	0.1577887	0.09852035	0.148578	0.1592524
427.385	4	0.505217	0.3243738	0.1576401	0.1016844	0.156186	0.1526712
427.485	4	0.501338	0.3194537	0.1551989	0.09973213	0.154168	0.1508133
427.585	4	0.508985	0.3046404	0.1547404	0.1036161	0.159337	0.1548237
427.685	4	0.509971	0.3244222	0.1550738	0.1044497	0.156725	0.1577467
427.785	4	0.501013	0.3434169	0.1621222	0.1035596	0.150529	0.1574985
427.885	4	0.499769	0.3463426	0.1597096	0.1091991	0.148722	0.1585226
427.985	4	0.495928	0.3451734	0.153808	0.1066372	0.152178	0.1579819
428.085	4	0.498473	0.3391212	0.1527127	0.1063362	0.142685	0.1582485
428.185	4	0.496195	0.3300096	0.1534884	0.1153509	0.146607	0.156615
428.285	4	0.515876	0.3267488	0.1526121	0.1174629	0.148331	0.1576485
428.385	4	0.493962	0.3303981	0.1522541	0.118555	0.1496	0.1572323
428.485	4	0.503035	0.3321553	0.1540332	0.121162	0.150013	0.1585884
428.585	4	0.496216	0.3273292	0.1516054	0.1200995	0.153183	0.1575897
428.685	4	0.498772	0.3362474	0.1505693	0.1199903	0.151438	0.1492419
428.785	4	0.506882	0.3437658	0.1521794	0.1190153	0.145826	0.1505761
428.885	4	0.503532	0.3399587	0.1520558	0.1190251	0.146327	0.1562039
428.985	4	0.503126	0.3168119	0.1519736	0.1157019	0.147147	0.1576835
429.085	4	0.496897	0.3312124	0.1477286	0.1180523	0.141861	0.1602183
429.185	4	0.498602	0.341976	0.1499191	0.1180633	0.144235	0.160405
429.285	4	0.478223	0.3116808	0.1519524	0.1180621	0.147499	0.1591784
429.385	4	0.460913	0.3077003	0.1532963	0.1160601	0.148769	0.1567608
429.485	4	0.4623	0.3231374	0.1536339	0.1178492	0.152182	0.1584622
429.585	4	0.474338	0.3172082	0.1518674	0.118613	0.148685	0.1595394
429.685	4	0.491506	0.329323	0.1514856	0.1212963	0.145639	0.1559567
429.785	4	0.48652	0.3394282	0.1449278	0.1237714	0.145631	0.15382
429.885	4	0.489196	0.3387709	0.1469528	0.1253923	0.143761	0.1532899
429.985	4	0.499	0.3345699	0.1537148	0.1213089	0.14913	0.1506113
430.085	4	0.498917	0.3343705	0.1521614	0.125821	0.147471	0.1469712
430.185	4	0.48717	0.3357646	0.1502488	0.1227949	0.143746	0.144074
430.285	4	0.513409	0.3339353	0.1525866	0.1135116	0.14471	0.1379433
430.385	4	0.490051	0.3328591	0.1529443	0.1087728	0.144293	0.1474073
430.485	4	0.497182	0.3321127	0.1511025	0.09910588	0.144576	0.1462272
430.585	4	0.49057	0.3262492	0.1531897	0.09380811	0.141016	0.1458707
430.685	4	0.48642	0.3254808	0.1528248	0.0982917	0.14179	0.1457673
430.785	4	0.495389	0.3335226	0.15351	0.09239912	0.144516	0.1499643
430.885	4	0.484205	0.3179821	0.15499	0.0986949	0.145926	0.1511251
430.985	4	0.481536	0.3059428	0.154605	0.09357496	0.141769	0.1532759
431.085	4	0.501734	0.3151735	0.1593559	0.09601298	0.141409	0.152986
431.185	4	0.492533	0.3227274	0.1611502	0.1027542	0.149352	0.1526724
431.285	4	0.497297	0.3192932	0.1611377	0.0978791	0.149908	0.1547729
431.385	4	0.494747	0.3100543	0.1594282	0.09976107	0.152278	0.1542973
431.485	4	0.499511	0.3059562	0.158065	0.1006045	0.145877	0.1570883
431.585	4	0.4947	0.3350897	0.1594114	0.1012569	0.142988	0.1558325
431.685	4	0.493961	0.3401907	0.156784	0.09139012	0.140455	0.156859
431.785	4	0.493946	0.3503367	0.1564571	0.09122419	0.139934	0.1556975
431.885	4	0.49773	0.3465266	0.1573564	0.09241384	0.139644	0.1566707
431.985	4	0.486944	0.3333586	0.1591832	0.0898413	0.141973	0.1584101
432.085	4	0.48212	0.3289932	0.1585156	0.08768509	0.132107	0.1581834
432.185	4	0.494594	0.3271931	0.1581695	0.08958093	0.133144	0.1566431
432.285	4	0.503283	0.3298175	0.1588393	0.08664411	0.14284	0.157414
432.385	4	0.505785	0.3312288	0.156418	0.08660147	0.144165	0.1578627
432.485	4	0.50191	0.3266188	0.1560006	0.08374325	0.144021	0.1492163
432.585	4	0.497934	0.3397959	0.1564901	0.08634771	0.142273	0.1517583
432.685	4	0.505647	0.3434462	0.156401	0.089517	0.137405	0.1536276
432.785	4	0.501812	0.3371027	0.1545785	0.08890678	0.136722	0.1581559
432.885	4	0.506263	0.3343686	0.1576397	0.08629236	0.137002	0.1595731
432.985	4	0.512534	0.348571	0.1564363	0.09056795	0.137203	0.1597345
433.085	4	0.502704	0.345327	0.1558007	0.089282	0.135652	0.1612035
433.185	4	0.502214	0.3337764	0.1556581	0.08962635	0.136987	0.1593304
433.285	4	0.508406	0.3404644	0.1578906	0.08906625	0.136801	0.1565927
433.385	4	0.514916	0.3385094	0.1579653	0.08955717	0.112989	0.15695
433.485	4	0.517816	0.3519545	0.1564335	0.08669164	0.12507	0.1587451
433.585	4	0.499888	0.3636318	0.1511733	0.08843546	0.128724	0.1597269
433.685	4	0.509599	0.356693	0.1531839	0.09086911	0.126652	0.1595193
433.785	4	0.507429	0.3517673	0.1569542	0.09055226	0.136555	0.1609108
433.885	4	0.509794	0.3525115	0.157269	0.08733802	0.135299	0.1631284
433.985	4	0.514752	0.353298	0.1574139	0.07833111	0.133521	0.1617958
434.085	4	0.515273	0.3495273	0.1546102	0.08321139	0.138917	0.1624938
434.185	4	0.508243	0.3469976	0.1551906	0.08685375	0.133887	0.164849

434.285	4	0.510536	0.3508447	0.1546021	0.09014739	0.134064	0.1639518
434.385	4	0.502882	0.3493757	0.1520881	0.09382861	0.133403	0.1549819
434.485	4	0.503203	0.3581311	0.1558196	0.09676883	0.136885	0.1551563
434.585	4	0.512015	0.3681118	0.1570021	0.07759207	0.139279	0.1578792
434.685	4	0.51694	0.3654588	0.1580213	0.07538787	0.136151	0.157492
434.785	4	0.504874	0.3545251	0.1569458	0.07785223	0.137698	0.1562955
434.885	4	0.512999	0.3560133	0.1569085	0.07839797	0.133861	0.1589966
434.985	4	0.507001	0.3537285	0.1582776	0.07169551	0.140029	0.1590931
435.085	4	0.492455	0.3467448	0.1546283	0.08475538	0.138979	0.1520001
435.185	4	0.491856	0.3429394	0.1538123	0.09149516	0.136718	0.1516798
435.285	4	0.508737	0.3311709	0.1540608	0.09315237	0.136234	0.1529701
435.385	4	0.498532	0.3355294	0.1531884	0.08527225	0.135889	0.1555655
435.485	4	0.512025	0.367963	0.1563008	0.07724069	0.136067	0.1579616
435.585	4	0.516888	0.3718288	0.1562968	0.08474092	0.135985	0.1608138
435.685	4	0.503023	0.3684203	0.1563642	0.09189628	0.137279	0.1597098
435.785	4	0.50449	0.3728828	0.1576096	0.09441999	0.135588	0.1585441
435.885	4	0.516246	0.3726661	0.1566284	0.09031224	0.140134	0.1592376
435.985	4	0.529919	0.3678046	0.1575632	0.0883036	0.140963	0.1580613
436.085	4	0.512254	0.3643218	0.1560629	0.07878873	0.136712	0.1578281
436.185	4	0.518998	0.3645562	0.1558677	0.07726936	0.137989	0.1600543
436.285	4	0.514961	0.3644007	0.1545687	0.0811501	0.136677	0.1616474
436.385	4	0.509064	0.3623273	0.153901	0.08342744	0.136765	0.1624666
436.485	4	0.516694	0.3670586	0.1542171	0.09038082	0.137064	0.1626496
436.585	4	0.528286	0.3596072	0.1563628	0.09272166	0.137978	0.1580697
436.685	4	0.510071	0.3561406	0.1570754	0.09258176	0.136054	0.1602563
436.785	4	0.513637	0.3648779	0.1562324	0.0898298	0.13455	0.1578772
436.885	4	0.508555	0.3657184	0.1559588	0.08916069	0.138421	0.1589861
436.985	4	0.504673	0.3486977	0.1578387	0.09306584	0.136103	0.158796
437.085	4	0.5105	0.349684	0.1556928	0.09792604	0.138696	0.1587601
437.185	4	0.505812	0.3541888	0.1555951	0.09473427	0.140246	0.1568292
437.285	4	0.510712	0.3468906	0.1572431	0.09275233	0.138356	0.1599801
437.385	4	0.509246	0.3588323	0.157024	0.08550717	0.14093	0.160899
437.485	4	0.508631	0.3703922	0.1551076	0.09037238	0.134789	0.1602708
437.585	4	0.504556	0.3624371	0.1566932	0.09396083	0.134684	0.161879
437.685	4	0.512162	0.3564801	0.1582693	0.09385737	0.117921	0.1635356
437.785	4	0.509455	0.3542589	0.1601354	0.09752361	0.129024	0.1658892
437.885	4	0.504735	0.3523407	0.1577772	0.09927138	0.128099	0.1664584
437.985	4	0.513418	0.3508506	0.158597	0.09974343	0.130865	0.1661011
438.085	4	0.50563	0.3490444	0.1597344	0.09575994	0.135396	0.1622963
438.185	4	0.508929	0.3540417	0.1573679	0.08335419	0.134825	0.1662384
438.285	4	0.512218	0.3519848	0.1583407	0.0895272	0.133505	0.1696503
438.385	4	0.51466	0.358446	0.1592428	0.09367636	0.135646	0.1683645
438.485	4	0.503496	0.3646272	0.1595039	0.09623785	0.132627	0.1703501
438.585	4	0.508486	0.3519957	0.1613316	0.08504713	0.133335	0.1737923
438.685	4	0.522104	0.3430248	0.1642645	0.0934057	0.132688	0.1679552
438.785	4	0.514321	0.3416912	0.1608859	0.09403807	0.138524	0.1685469
438.885	4	0.522545	0.3468392	0.1607747	0.09911123	0.139115	0.1710908
438.985	4	0.511222	0.336251	0.161465	0.09389752	0.138838	0.1692294
439.085	4	0.519831	0.3359949	0.1618323	0.08873221	0.139962	0.1709413
439.185	4	0.509078	0.330576	0.16238	0.09082308	0.139315	0.1703298
439.285	4	0.515599	0.3415439	0.1634241	0.09065533	0.136514	0.1666169
439.385	4	0.523984	0.3660483	0.1645882	0.08331523	0.138055	0.1685812
439.485	4	0.498763	0.3676507	0.1609872	0.0883725	0.139897	0.1687524
439.585	4	0.49982	0.3604737	0.1623297	0.09140351	0.142058	0.1647151
439.685	4	0.499863	0.3647834	0.1641444	0.08989789	0.135949	0.1617366
439.785	4	0.498198	0.3585206	0.1615493	0.09151983	0.138192	0.1647343
439.885	4	0.472599	0.3551033	0.163114	0.08787331	0.13727	0.1678417
439.985	4	0.485132	0.3525243	0.165925	0.08799925	0.132667	0.1671094
440.085	4	0.497494	0.3517638	0.1656917	0.09103895	0.136175	0.1669451
440.185	4	0.514634	0.3513771	0.1653658	0.09184194	0.137206	0.1655824
440.285	4	0.498656	0.3487902	0.163443	0.08910451	0.136458	0.1658582
440.385	4	0.497496	0.3491917	0.1615256	0.08265724	0.136372	0.1655113
440.485	4	0.508253	0.3491575	0.1613221	0.08806143	0.136007	0.1622694
440.585	4	0.492198	0.3527163	0.161092	0.08856463	0.134745	0.1653652
440.685	4	0.501681	0.3622638	0.1598592	0.08603977	0.137243	0.1629981
440.785	4	0.505214	0.3560415	0.1597372	0.09682837	0.140887	0.1617974
440.885	4	0.493473	0.339916	0.1597594	0.09464707	0.142664	0.1598026
440.985	4	0.502858	0.347203	0.161306	0.09614448	0.138473	0.1479024
441.085	4	0.502	0.3409934	0.1597805	0.09216642	0.13901	0.1518461
441.185	4	0.507156	0.3469876	0.1594542	0.09276368	0.139863	0.1533045
441.285	4	0.508312	0.3560711	0.157437	0.09181535	0.14099	0.1475451
441.385	4	0.5032	0.3605921	0.158899	0.0948175	0.140578	0.1321799
441.485	4	0.509962	0.3566697	0.1611254	0.0993742	0.13758	0.1403712
441.585	4	0.499232	0.3506109	0.1605224	0.1012817	0.138999	0.143352
441.685	4	0.502649	0.3471118	0.1588199	0.09649637	0.141632	0.1455885
441.785	4	0.500501	0.3496105	0.1577965	0.09478125	0.145385	0.1487869
441.885	4	0.497696	0.3446685	0.1573921	0.09545342	0.146989	0.1497466
441.985	4	0.513856	0.3460098	0.1579454	0.09408434	0.143252	0.139271
442.085	4	0.483567	0.3486447	0.1595818	0.09496313	0.142386	0.1417337
442.185	4	0.516132	0.3522789	0.1587453	0.0953	0.147915	0.1444649
442.285	4	0.488634	0.3551432	0.1571895	0.09924107	0.143434	0.1445228
442.385	4	0.499305	0.3614611	0.1576751	0.1030255	0.143377	0.1458488
442.485	4	0.502987	0.3460269	0.156073	0.1023812	0.143524	0.1463727
442.585	4	0.513872	0.3314859	0.1529842	0.1074672	0.145525	0.1468055
442.685	4	0.498628	0.333661	0.1452785	0.1098911	0.144682	0.146314
442.785	4	0.494361	0.3457915	0.1480625	0.105327	0.149894	0.1466385
442.885	4	0.497334	0.3314196	0.1494278	0.1132024	0.148879	0.1450412
442.985	4	0.498256	0.3324776	0.1498487	0.1120572	0.149665	0.1475442
443.085	4	0.496015	0.327015	0.1493409	0.1093741	0.145194	0.1489157
443.185	4	0.497139	0.3474897	0.1505191	0.1162436	0.14425	0.1501882
443.285	4	0.514547	0.360856	0.1503437	0.1197268	0.146132	0.1498814
443.385	4	0.49969	0.3649549	0.1516607	0.1204725	0.145571	0.1484088
443.485	4	0.501186	0.3578295	0.1492081	0.1245234	0.145683	0.1492385
443.585	4	0.489948	0.3632419	0.1426816	0.1227714	0.144419	0.1487829
443.685	4	0.496315	0.3581387	0.1483302	0.1210544	0.14218	0.1497596

443.785	4	0.497167	0.3533207	0.1506205	0.1211359	0.14386	0.1501831
443.885	4	0.498771	0.3479806	0.1469641	0.1199352	0.145565	0.150551
443.985	4	0.502965	0.3504228	0.1439573	0.1156984	0.145062	0.1497461
444.085	4	0.495786	0.3462113	0.146251	0.1204943	0.146992	0.1457093
444.185	4	0.497098	0.3445483	0.1453004	0.1176261	0.147758	0.1477469
444.285	4	0.474651	0.3460515	0.1460343	0.1187014	0.147246	0.1485959
444.385	4	0.459743	0.3489578	0.1491784	0.1188365	0.1477	0.1485838
444.485	4	0.460774	0.3493043	0.1507116	0.1219958	0.151003	0.1480518
444.585	4	0.467636	0.3529368	0.1493285	0.1230689	0.155967	0.1463694
444.685	4	0.488603	0.3451914	0.1369128	0.1275054	0.151626	0.1483849
444.785	4	0.487565	0.3354311	0.1412138	0.1282183	0.156023	0.1471004
444.885	4	0.483688	0.3461439	0.1486278	0.1264123	0.151837	0.1469361
444.985	4	0.493157	0.3352698	0.1351542	0.1260092	0.148595	0.1451742
445.085	4	0.494066	0.3406014	0.1375708	0.1281448	0.147751	0.1457265
445.185	4	0.488861	0.3561422	0.1426137	0.1251071	0.145075	0.1463176
445.285	4	0.514441	0.3535239	0.1498602	0.1146132	0.14215	0.1459573
445.385	4	0.488715	0.3500796	0.1448343	0.1136093	0.149142	0.1459085
445.485	4	0.49962	0.3438325	0.1418943	0.09966712	0.147834	0.1455943
445.585	4	0.492345	0.3467413	0.1517645	0.09982026	0.14767	0.1452469
445.685	4	0.486276	0.3438478	0.1514055	0.09930687	0.147485	0.1469597
445.785	4	0.486483	0.3415816	0.1483218	0.09908068	0.151352	0.1471077
445.885	4	0.478032	0.3426597	0.1513042	0.09655108	0.146543	0.1467308
445.985	4	0.474525	0.3471891	0.1523098	0.09360934	0.142215	0.1472674
446.085	4	0.492042	0.348467	0.1532627	0.1024474	0.145491	0.1488625
446.185	4	0.491417	0.3525577	0.1563781	0.1023819	0.145194	0.1512265
446.285	4	0.489898	0.3524017	0.1507511	0.09844173	0.142258	0.1508121
446.385	4	0.492754	0.3393266	0.1531131	0.1020936	0.143963	0.1515065
446.485	4	0.498047	0.3297467	0.1533148	0.1045902	0.150653	0.1500043
446.585	4	0.493476	0.333955	0.1551935	0.1012446	0.151079	0.1501235
446.685	4	0.489539	0.3396587	0.1557606	0.09412821	0.151141	0.1503158
446.785	4	0.496009	0.328319	0.1554402	0.09813192	0.148189	0.1498851
446.885	4	0.494622	0.3240076	0.1545015	0.09547146	0.145825	0.1464236
446.985	4	0.488146	0.3244922	0.1543002	0.09265528	0.145076	0.151981
447.085	4	0.482925	0.3533527	0.1551217	0.09635743	0.145572	0.1500834
447.185	4	0.495051	0.3602318	0.1530403	0.09231436	0.151344	0.1498272
447.285	4	0.502825	0.3599367	0.1548309	0.08941447	0.149725	0.1508083
447.385	4	0.507233	0.3567477	0.1524038	0.09046222	0.148693	0.1510502
447.485	4	0.49765	0.3547422	0.1532553	0.08904535	0.152091	0.1505427
447.585	4	0.496287	0.352635	0.1587576	0.09086844	0.148769	0.1515848
447.685	4	0.503845	0.3474064	0.1554913	0.09439643	0.146111	0.1512444
447.785	4	0.504879	0.3459383	0.1547824	0.09289811	0.146809	0.154748
447.885	4	0.499417	0.3451178	0.1523438	0.08900452	0.145659	0.1533336
447.985	4	0.509242	0.3410216	0.1532076	0.09316856	0.149697	0.1529105
448.085	4	0.504833	0.3444857	0.1529308	0.092425	0.144187	0.1545752
448.185	4	0.505336	0.3447452	0.1530449	0.08928744	0.139521	0.1538076
448.285	4	0.505833	0.3451209	0.1566075	0.09189748	0.144665	0.1543739
448.385	4	0.509508	0.3474938	0.1542827	0.09343814	0.147257	0.1542429
448.485	4	0.515592	0.3534414	0.1554421	0.08924315	0.150366	0.1546123
448.585	4	0.499782	0.3402744	0.1551753	0.09075705	0.147137	0.1529314
448.685	4	0.511402	0.339405	0.1552838	0.09124446	0.141578	0.1558509
448.785	4	0.507776	0.344247	0.1544081	0.09045874	0.142963	0.1573556
448.885	4	0.509637	0.3316235	0.1527957	0.08825316	0.139598	0.1567301
448.985	4	0.511939	0.343878	0.1548724	0.07851752	0.141158	0.1576639
449.085	4	0.511084	0.3574521	0.1530567	0.08441559	0.142862	0.1563854
449.185	4	0.503267	0.3477658	0.1524608	0.08779023	0.138446	0.1583531
449.285	4	0.518767	0.3442671	0.1543892	0.09237763	0.132612	0.15751
449.385	4	0.506432	0.342461	0.1551036	0.09415297	0.138495	0.1581025
449.485	4	0.505083	0.3433788	0.1547926	0.09545873	0.144983	0.1596429
449.585	4	0.508044	0.3414219	0.1541725	0.07677546	0.148578	0.1637723
449.685	4	0.516595	0.3387847	0.15359	0.07610836	0.146781	0.1646405
449.785	4	0.516588	0.3432462	0.1565575	0.07941712	0.140766	0.1650902
449.885	4	0.512508	0.3404844	0.1565047	0.08047143	0.137003	0.1631027
449.985	4	0.509924	0.3460045	0.1538351	0.07598033	0.135395	0.1598945
450.085	4	0.491337	0.3525549	0.1538455	0.08690839	0.137615	0.1626216
450.185	4	0.4935	0.3453183	0.1600371	0.09352054	0.137641	0.1624773
450.285	4	0.501492	0.3315654	0.163906	0.09759226	0.134905	0.1619072
450.385	4	0.493903	0.3263198	0.1594904	0.08850517	0.138886	0.1630565
450.485	4	0.509321	0.3347172	0.1540996	0.07976883	0.1259	0.1644315
450.585	4	0.50947	0.3287404	0.157634	0.08635286	0.118982	0.1647323
450.685	4	0.512087	0.3230978	0.1566367	0.09415625	0.125821	0.160927
450.785	4	0.506325	0.317897	0.1553933	0.0977807	0.13658	0.1613953
450.885	4	0.503314	0.3248438	0.1583235	0.09401693	0.135255	0.1639256
450.985	4	0.523861	0.3563673	0.1586029	0.09061769	0.139754	0.1637378
451.085	4	0.513235	0.357662	0.1582609	0.07978261	0.135616	0.1618
451.185	4	0.517971	0.3509137	0.1567671	0.07943187	0.134152	0.162927
451.285	4	0.513282	0.3536201	0.1583071	0.08310693	0.135262	0.1639964
451.385	4	0.512652	0.3536724	0.1614941	0.08565838	0.140157	0.1639208
451.485	4	0.513791	0.3475237	0.1580483	0.09490054	0.139413	0.163144
451.585	4	0.527649	0.3442065	0.1555081	0.09581239	0.139392	0.1644641
451.685	4	0.51076	0.3439412	0.1543606	0.0944949	0.13665	0.1607371
451.785	4	0.513803	0.3411567	0.1573777	0.09282568	0.14046	0.1592892
451.885	4	0.505137	0.3379678	0.156726	0.09095464	0.140358	0.1592297
451.985	4	0.505291	0.3428267	0.1581926	0.09454262	0.139488	0.1589517
452.085	4	0.511866	0.3455128	0.1560311	0.1004338	0.138295	0.1609227
452.185	4	0.50696	0.3403361	0.1576924	0.09679475	0.139681	0.159635
452.285	4	0.504982	0.3441718	0.1574881	0.0946501	0.141223	0.1556568
452.385	4	0.505918	0.3458737	0.1563773	0.08659665	0.14022	0.1545939
452.485	4	0.506991	0.331281	0.1586394	0.09425694	0.140851	0.1560175
452.585	4	0.495218	0.3350881	0.1553503	0.09543963	0.145116	0.1592644
452.685	4	0.510101	0.3331165	0.1570352	0.09683585	0.145485	0.1573536
452.785	4	0.51338	0.3297881	0.1590952	0.1001311	0.141316	0.1590668
452.885	4	0.503653	0.3433935	0.1583761	0.1019142	0.141308	0.1558989
452.985	4	0.515172	0.3523185	0.157739	0.1016905	0.138911	0.1573419
453.085	4	0.504301	0.3440993	0.1542172	0.09560858	0.142504	0.1589933
453.185	4	0.507346	0.3408604	0.1583308	0.08406236	0.14105	0.1501884

453.285	4	0.507038	0.3398996	0.1610456	0.09406349	0.140033	0.1523327
453.385	4	0.510287	0.3415789	0.1620814	0.09605474	0.13685	0.1521194
453.485	4	0.499979	0.3373765	0.1612535	0.09780885	0.139682	0.1512305
453.585	4	0.50097	0.3380007	0.1597115	0.08945445	0.138473	0.1516302
453.685	4	0.525099	0.3401662	0.1595999	0.09495796	0.141629	0.1530295
453.785	4	0.512679	0.3379446	0.1518051	0.09531321	0.142671	0.1542968
453.885	4	0.515886	0.3411142	0.1591848	0.100035	0.144215	0.1488938
453.985	4	0.504304	0.3527621	0.158831	0.09598015	0.140436	0.1497658
454.085	4	0.510053	0.3394774	0.1554968	0.0904715	0.136263	0.1530575
454.185	4	0.512656	0.3199041	0.1542261	0.09110253	0.119584	0.1526207
454.285	4	0.525328	0.320138	0.1582088	0.0923541	0.128357	0.1529679
454.385	4	0.5118	0.3330986	0.1598463	0.08283672	0.128479	0.1543104
454.485	4	0.498613	0.3242164	0.1561058	0.08960253	0.133675	0.1530156
454.585	4	0.495732	0.3158377	0.156791	0.09210081	0.135352	0.1520457
454.685	4	0.500484	0.3133417	0.1584254	0.08990049	0.135275	0.1519693
454.785	4	0.4983	0.3244369	0.1609628	0.09177431	0.136458	0.151682
454.885	4	0.480523	0.3512653	0.1629786	0.08796573	0.134566	0.1490192
454.985	4	0.495334	0.3545366	0.1618646	0.090418	0.134355	0.1455975
455.085	4	0.511641	0.3497261	0.1626532	0.08916387	0.136758	0.1517126
455.185	4	0.500073	0.3521827	0.1650012	0.09119722	0.136219	0.1525199
455.285	4	0.504113	0.348888	0.1656111	0.08722756	0.136822	0.1537333
455.385	4	0.494291	0.344182	0.1637818	0.08176488	0.136767	0.1526788
455.485	4	0.515117	0.3402293	0.1604813	0.08922258	0.133021	0.1525506
455.585	4	0.49757	0.3420762	0.1610117	0.08771296	0.1333	0.1521143
455.685	4	0.496283	0.3376182	0.1585309	0.08763471	0.136607	0.1526837
455.785	4	0.498861	0.337167	0.1585088	0.09787181	0.141753	0.1512804
455.885	4	0.500485	0.3417055	0.1591279	0.0963042	0.135043	0.1488767
455.985	4	0.500246	0.3433039	0.1592982	0.09443823	0.137184	0.1474317
456.085	4	0.504682	0.3339853	0.1587587	0.09207594	0.134635	0.1445217
456.185	4	0.512462	0.3432593	0.1582108	0.09336709	0.130792	0.1378309
456.285	4	0.499765	0.3413185	0.1588699	0.0942452	0.135253	0.1391558
456.385	4	0.501546	0.3295876	0.159857	0.09946874	0.134054	0.1456744
456.485	4	0.510581	0.3389131	0.1579571	0.1011388	0.134015	0.1437305
456.585	4	0.496865	0.3245113	0.1567124	0.1012232	0.134134	0.1443795
456.685	4	0.502767	0.3316641	0.1554714	0.09622823	0.133315	0.1477429
456.785	4	0.49849	0.3477714	0.1537446	0.09544937	0.136705	0.1501642
456.885	4	0.496668	0.349184	0.1544624	0.09745394	0.138896	0.1509758
456.985	4	0.508993	0.343678	0.1534326	0.09653836	0.136834	0.1507732
457.085	4	0.485779	0.3405573	0.154388	0.09613716	0.136412	0.1520035
457.185	4	0.511349	0.3396986	0.1530325	0.0952951	0.136295	0.1514911
457.285	4	0.489076	0.3401235	0.1536867	0.09644365	0.138251	0.1525953
457.385	4	0.509936	0.3363491	0.1550759	0.0999724	0.137868	0.152939
457.485	4	0.4995	0.3363579	0.1505855	0.1025717	0.135017	0.1497195
457.585	4	0.5088	0.3343273	0.1448177	0.106609	0.136899	0.1529748
457.685	4	0.508736	0.3376395	0.1477112	0.1085871	0.141055	0.1552337
457.785	4	0.500614	0.3393124	0.149325	0.1049957	0.145349	0.1529927
457.885	4	0.493052	0.3466307	0.1494979	0.1142353	0.142223	0.1530944
457.985	4	0.490678	0.3311788	0.1501072	0.112897	0.141853	0.1471442
458.085	4	0.493313	0.3196707	0.1487638	0.1114945	0.143669	0.1476148
458.185	4	0.491713	0.32435	0.1489354	0.1164572	0.138754	0.1510736
458.285	4	0.508537	0.3322081	0.1502934	0.1166558	0.138865	0.1566697
458.385	4	0.49369	0.3233089	0.1488015	0.1178863	0.14187	0.1552223
458.485	4	0.501697	0.3196014	0.1512224	0.1190412	0.142863	0.1563738
458.585	4	0.490245	0.3179642	0.15208	0.1185681	0.144987	0.1542734
458.685	4	0.495037	0.3357366	0.1524066	0.1191484	0.146286	0.1509663
458.785	4	0.50116	0.3493751	0.1518889	0.1200448	0.148205	0.1529846
458.885	4	0.498753	0.3543851	0.1525333	0.1200351	0.140727	0.155543
458.985	4	0.501545	0.3527562	0.1541148	0.1173886	0.142972	0.154468
459.085	4	0.49308	0.3476547	0.1514145	0.1215672	0.144186	0.1579035
459.185	4	0.49294	0.3468122	0.1505436	0.120388	0.144635	0.1587149
459.285	4	0.47021	0.3418926	0.1505531	0.1213251	0.141724	0.1572912
459.385	4	0.459335	0.3390682	0.1515426	0.1214914	0.137499	0.1591429
459.485	4	0.456885	0.3429624	0.1549358	0.1202937	0.140806	0.1541891
459.585	4	0.466621	0.3358506	0.1545112	0.1226197	0.141573	0.151833
459.685	4	0.485107	0.3397753	0.1522042	0.1260706	0.142085	0.1528229
459.785	4	0.484061	0.3398773	0.1552009	0.1271084	0.147268	0.151186
459.885	4	0.480354	0.34117	0.1528127	0.1246883	0.147298	0.1517595
459.985	4	0.489033	0.3345015	0.1525506	0.1241188	0.147749	0.1525404
460.085	4	0.492614	0.3407256	0.1498677	0.1240619	0.150324	0.1461428
460.185	4	0.483921	0.3367427	0.149111	0.1210004	0.154857	0.149571
460.285	4	0.508761	0.3308885	0.1475522	0.1138369	0.153098	0.1547986
460.385	4	0.489288	0.3349119	0.1484243	0.113391	0.152625	0.1548238
460.485	4	0.496033	0.323164	0.1484189	0.09910331	0.149648	0.1562459
460.585	4	0.494897	0.3395001	0.1510551	0.09814663	0.14816	0.1564248
460.685	4	0.477722	0.3527458	0.1506013	0.09988356	0.146714	0.1549279
460.785	4	0.488752	0.3427515	0.152002	0.1013715	0.142012	0.153534
460.885	4	0.470975	0.3415299	0.1532132	0.09897196	0.14862	0.1546426
460.985	4	0.474464	0.3388585	0.1535595	0.09413293	0.148488	0.1548739
461.085	4	0.494853	0.3400333	0.1537143	0.1047002	0.146064	0.1547126
461.185	4	0.491856	0.337196	0.1525584	0.1001823	0.147915	0.15759
461.285	4	0.490752	0.336964	0.1544535	0.09707477	0.149664	0.1549335
461.385	4	0.492556	0.3377793	0.1545617	0.1022838	0.143044	0.1565745
461.485	4	0.49696	0.3336868	0.1526349	0.1010024	0.143697	0.1543973
461.585	4	0.491932	0.3369518	0.1511798	0.09605324	0.144086	0.1544387
461.685	4	0.489688	0.3404447	0.1535275	0.0946208	0.139538	0.1542692
461.785	4	0.49221	0.3416283	0.1570263	0.09885535	0.14072	0.1521938
461.885	4	0.497372	0.324997	0.1541366	0.0953025	0.146001	0.1530482
461.985	4	0.4931	0.3175933	0.1534375	0.09030127	0.14877	0.1552625
462.085	4	0.491135	0.3257271	0.1571133	0.09388163	0.146554	0.155877
462.185	4	0.496654	0.3279363	0.1542918	0.08977108	0.14266	0.1561242
462.285	4	0.504644	0.3199084	0.15503	0.0915123	0.141724	0.1577146
462.385	4	0.505545	0.3103663	0.1559413	0.09202756	0.143141	0.15857
462.485	4	0.504779	0.3141558	0.1563652	0.09155917	0.148203	0.1562922
462.585	4	0.496042	0.3433892	0.1584883	0.09158993	0.147684	0.1605155
462.685	4	0.502134	0.3486894	0.159639	0.09549472	0.145765	0.1599055

462.785	4	0.500556	0.3489681	0.1554076	0.08914474	0.145852	0.1617615
462.885	4	0.504606	0.3492251	0.1571029	0.08911186	0.142525	0.1606876
462.985	4	0.504095	0.3471034	0.1559093	0.09169726	0.140986	0.1552877
463.085	4	0.501574	0.3432128	0.15697	0.09045999	0.142433	0.1610683
463.185	4	0.510731	0.3419344	0.1564222	0.08916253	0.145437	0.1601471
463.285	4	0.504623	0.3371273	0.1570076	0.09357864	0.139588	0.1603508
463.385	4	0.512355	0.3378621	0.1573482	0.09016277	0.136477	0.1598816
463.485	4	0.513113	0.3349364	0.154887	0.08978014	0.142188	0.1559535
463.585	4	0.497319	0.3401265	0.1553602	0.09120946	0.145062	0.1592421
463.685	4	0.505863	0.3411175	0.1540249	0.09105939	0.145268	0.157626
463.785	4	0.506487	0.3350259	0.152368	0.08639198	0.1407	0.1567434
463.885	4	0.506385	0.3381261	0.1577851	0.08660206	0.14088	0.16095
463.985	4	0.503888	0.3432666	0.1594863	0.07936286	0.138656	0.1608252
464.085	4	0.50701	0.3295579	0.1603476	0.08609214	0.139307	0.1617109
464.185	4	0.502805	0.3297744	0.1574385	0.08862343	0.13913	0.1590602
464.285	4	0.512681	0.3265857	0.158469	0.09262566	0.135699	0.1602489
464.385	4	0.498301	0.3259089	0.1581321	0.09539858	0.136587	0.1573643
464.485	4	0.503552	0.3392055	0.1582424	0.09362157	0.144558	0.1613069
464.585	4	0.510539	0.3510457	0.1557077	0.07356905	0.147562	0.1597984
464.685	4	0.510036	0.3410893	0.1557196	0.08049817	0.146662	0.158135
464.785	4	0.511322	0.3393734	0.1551295	0.08375602	0.138326	0.151602
464.885	4	0.509743	0.3378712	0.1547939	0.08028919	0.13835	0.1434337
464.985	4	0.507459	0.3397621	0.1519394	0.08133232	0.138316	0.1482213
465.085	4	0.487017	0.3363328	0.1521407	0.09000074	0.140176	0.1532162
465.185	4	0.483388	0.3365965	0.1493006	0.09722215	0.137014	0.1541854
465.285	4	0.50348	0.3373482	0.1503986	0.09882899	0.139608	0.1415695
465.385	4	0.496125	0.3325432	0.1518837	0.08624248	0.124943	0.1342319
465.485	4	0.507533	0.3343384	0.1520772	0.08225678	0.121643	0.142057
465.585	4	0.511189	0.3458322	0.1526024	0.09115304	0.125823	0.142893
465.685	4	0.507607	0.3351496	0.1519047	0.0966941	0.123973	0.146816
465.785	4	0.504994	0.320463	0.1495775	0.09962176	0.132528	0.1489342
465.885	4	0.505419	0.3145015	0.1486517	0.09270449	0.131454	0.1496672
465.985	4	0.521127	0.3272939	0.1492577	0.09016521	0.134218	0.1414042
466.085	4	0.505104	0.320747	0.1466082	0.08140072	0.134032	0.1433939
466.185	4	0.514303	0.3163913	0.1483161	0.08288486	0.133034	0.1443355
466.285	4	0.509264	0.3078879	0.1472716	0.08617537	0.133869	0.1450624
466.385	4	0.512723	0.3153029	0.1464842	0.09136862	0.137284	0.1460603
466.485	4	0.517207	0.3445777	0.1453696	0.09647747	0.139562	0.1468704
466.585	4	0.525737	0.3514231	0.141002	0.09793668	0.139365	0.1468393
466.685	4	0.510618	0.3453452	0.1393479	0.09533839	0.139555	0.1460316
466.785	4	0.514067	0.3494391	0.1422182	0.09216398	0.140949	0.1464373
466.885	4	0.501363	0.3475489	0.1435005	0.09257388	0.139835	0.145791
466.985	4	0.504909	0.3397911	0.1412571	0.09747645	0.138714	0.1477812
467.085	4	0.512014	0.3372944	0.1416465	0.1004962	0.137376	0.1477195
467.185	4	0.502428	0.3371998	0.141515	0.09656972	0.137755	0.1499147
467.285	4	0.504976	0.3347993	0.1420803	0.09411106	0.139377	0.1484152
467.385	4	0.50157	0.3362717	0.1420293	0.0851529	0.138912	0.1491622
467.485	4	0.506631	0.3385427	0.1358301	0.09243561	0.138799	0.1485861
467.585	4	0.500766	0.3432258	0.1412082	0.09333632	0.142762	0.1489238
467.685	4	0.511654	0.3290295	0.1437768	0.09439035	0.143349	0.1494454
467.785	4	0.509677	0.3385449	0.1405159	0.09926388	0.141043	0.1503565
467.885	4	0.501393	0.3371161	0.140785	0.1014715	0.142171	0.1504951
467.985	4	0.51584	0.3225835	0.1427857	0.0983917	0.138499	0.1497919
468.085	4	0.504095	0.3336004	0.1400724	0.09745821	0.140059	0.1466373
468.185	4	0.502012	0.3193831	0.1402289	0.08510658	0.138575	0.1497377
468.285	4	0.503701	0.3261087	0.1432469	0.09442804	0.138282	0.1490237
468.385	4	0.509156	0.3403195	0.1432385	0.09762041	0.137337	0.1479181
468.485	4	0.503759	0.3461855	0.1466224	0.09815563	0.141575	0.1476185
468.585	4	0.507312	0.3405164	0.1380256	0.08952537	0.13947	0.1470889
468.685	4	0.521952	0.336362	0.1394224	0.09476151	0.142495	0.1492814
468.785	4	0.511583	0.3374413	0.146318	0.09704053	0.141129	0.1473237
468.885	4	0.524019	0.3381405	0.1363775	0.09988651	0.14126	0.1478979
468.985	4	0.514852	0.3352463	0.1401931	0.09664238	0.140908	0.1459713
469.085	4	0.514579	0.3344719	0.1426162	0.09066178	0.137311	0.1475643
469.185	4	0.512855	0.3338318	0.1444573	0.09319185	0.122168	0.1488779
469.285	4	0.528985	0.3326302	0.1432815	0.09381009	0.128802	0.1478515
469.385	4	0.501328	0.3359552	0.1382852	0.08444433	0.12851	0.147651
469.485	4	0.499247	0.3449828	0.1486778	0.09130432	0.134687	0.1484615
469.585	4	0.501412	0.328452	0.145474	0.09400431	0.136173	0.1482626
469.685	4	0.491345	0.3098304	0.1412105	0.09027902	0.135033	0.1483009
469.785	4	0.497167	0.3156258	0.1427989	0.09181642	0.137432	0.1496322
469.885	4	0.489623	0.3253011	0.1466804	0.08907276	0.13554	0.1514842
469.985	4	0.500122	0.3168993	0.1454441	0.09106363	0.134119	0.1514274
470.085	4	0.519447	0.3169471	0.1479586	0.08926933	0.138709	0.1519407
470.185	4	0.496999	0.3109154	0.1450692	0.09146345	0.137317	0.152725
470.285	4	0.505251	0.3332515	0.1465985	0.08786311	0.13585	0.1534059
470.385	4	0.493497	0.3424387	0.1466726	0.08227728	0.13653	0.1553715
470.485	4	0.505033	0.3484489	0.1466971	0.09088924	0.133308	0.1542943
470.585	4	0.501024	0.3453172	0.1485936	0.08892249	0.132581	0.1534936
470.685	4	0.49937	0.3464437	0.1496055	0.0882434	0.134554	0.1544157
470.785	4	0.497146	0.3470783	0.14866	0.0985306	0.138877	0.154178
470.885	4	0.499056	0.3419849	0.1511638	0.0980969	0.134718	0.1494026
470.985	4	0.493748	0.3390191	0.1504929	0.09524042	0.135833	0.1539846
471.085	4	0.504882	0.3399366	0.148243	0.09292895	0.135313	0.1520935
471.185	4	0.52044	0.3372859	0.1525193	0.09469399	0.131233	0.1524935
471.285	4	0.493621	0.3415543	0.1447737	0.0930852	0.135233	0.1541919
471.385	4	0.498254	0.3428713	0.1445128	0.09741105	0.135254	0.1536245
471.485	4	0.508203	0.3429112	0.1483269	0.102088	0.133033	0.1532791
471.585	4	0.500368	0.3354065	0.151236	0.1013941	0.136247	0.1547271
471.685	4	0.504041	0.3359289	0.1502963	0.09647159	0.132197	0.1547763
471.785	4	0.5007	0.3358821	0.1490605	0.09615435	0.133585	0.1554114
471.885	4	0.502917	0.3207127	0.150739	0.09668085	0.137894	0.156065
471.985	4	0.503266	0.3376123	0.1506351	0.09759828	0.136868	0.1562053
472.085	4	0.502814	0.3184367	0.1482748	0.09711666	0.136026	0.1557614
472.185	4	0.497748	0.3296049	0.1495698	0.09771618	0.136658	0.1557659

472.285	4	0.483047	0.3454495	0.1497004	0.09865043	0.137566	0.1569976
472.385	4	0.505244	0.3438987	0.1536825	0.1005089	0.137606	0.1561924
472.485	4	0.506446	0.3394324	0.1552706	0.1021395	0.136189	0.1567486
472.585	4	0.492804	0.3372547	0.1517813	0.1072887	0.136878	0.1542251
472.685	4	0.503403	0.3389517	0.1530429	0.1084609	0.138732	0.1585525
472.785	4	0.490266	0.3371961	0.151282	0.1069997	0.144843	0.1596654
472.885	4	0.492948	0.3332146	0.1557609	0.1147377	0.142214	0.158385
472.985	4	0.487987	0.3333858	0.1535724	0.1125003	0.139203	0.1583735
473.085	4	0.483909	0.3315645	0.1527299	0.1113072	0.145739	0.1577186
473.185	4	0.480986	0.3347513	0.1529652	0.1144432	0.141852	0.1604786
473.285	4	0.521912	0.3332339	0.1538559	0.1162952	0.141018	0.1595008
473.385	4	0.484203	0.3404123	0.1547208	0.1201969	0.14172	0.1605653
473.485	4	0.495273	0.3225505	0.1547334	0.11608	0.143842	0.1618575
473.585	4	0.495008	0.3102677	0.1545477	0.1176294	0.145924	0.1618301
473.685	4	0.501593	0.3161345	0.1541927	0.1195882	0.14635	0.1611365
473.785	4	0.498575	0.3221354	0.1556453	0.1202699	0.150084	0.1615377
473.885	4	0.503601	0.3143216	0.1541391	0.1213646	0.144927	0.1602876
473.985	4	0.497718	0.313352	0.1514209	0.1192486	0.144799	0.1587439
474.085	4	0.495754	0.3075942	0.1542326	0.1201728	0.144685	0.1614048
474.185	4	0.492553	0.3405657	0.1539433	0.1203796	0.14615	0.1619267
474.285	4	0.468996	0.347192	0.1564474	0.123712	0.145541	0.1637456
474.385	4	0.455767	0.3453376	0.1531762	0.1210911	0.140557	0.1623466
474.485	4	0.457442	0.3466965	0.1504277	0.1184717	0.142617	0.1637371
474.585	4	0.475074	0.3476618	0.1546403	0.12168	0.144549	0.1629217
474.685	4	0.48262	0.3462223	0.1526249	0.127235	0.145195	0.1600677
474.785	4	0.475454	0.3384912	0.1522209	0.1270085	0.149389	0.1618526
474.885	4	0.493184	0.3375002	0.155412	0.1217138	0.145998	0.1659562
474.985	4	0.488303	0.3377904	0.1527273	0.1221934	0.145502	0.1632331
475.085	4	0.497226	0.337895	0.1544065	0.1228704	0.150136	0.1642307
475.185	4	0.487842	0.3456349	0.1547886	0.1231221	0.154429	0.1644643
475.285	4	0.502478	0.3434429	0.1603571	0.1131781	0.153153	0.1671641
475.385	4	0.483701	0.3423443	0.1591848	0.1121526	0.153211	0.1671223
475.485	4	0.498012	0.3388017	0.156725	0.09877788	0.148655	0.1680382
475.585	4	0.483983	0.3400739	0.1552594	0.09877315	0.146845	0.1649901
475.685	4	0.48033	0.3326649	0.1507344	0.1006396	0.144609	0.1612171
475.785	4	0.492176	0.3301155	0.1535598	0.1025628	0.14009	0.1597247
475.885	4	0.468314	0.3343438	0.1539349	0.1051748	0.1467	0.1592126
475.985	4	0.48328	0.3240439	0.1556116	0.09717046	0.144987	0.1586583
476.085	4	0.495306	0.3335055	0.1528403	0.1046023	0.145762	0.1593131
476.185	4	0.491766	0.3497712	0.1547519	0.1049508	0.149244	0.156866
476.285	4	0.496147	0.3414032	0.1559606	0.09961955	0.146215	0.1541472
476.385	4	0.489666	0.3414344	0.1573186	0.1017521	0.142095	0.1559177
476.485	4	0.495928	0.3395154	0.1582758	0.1043813	0.145138	0.1553245
476.585	4	0.493159	0.3383738	0.1549231	0.09996745	0.142435	0.1571891
476.685	4	0.487567	0.3362678	0.1558218	0.09678259	0.140779	0.1569649
476.785	4	0.489386	0.3350377	0.1584665	0.09946065	0.143076	0.1587008
476.885	4	0.493288	0.3350318	0.1562627	0.0958534	0.145668	0.1562175
476.985	4	0.49288	0.3301353	0.1549005	0.09158622	0.149852	0.1599196
477.085	4	0.4835	0.3363374	0.1526914	0.09216332	0.147546	0.157345
477.185	4	0.498364	0.3399424	0.1562974	0.09187711	0.142523	0.1488115
477.285	4	0.505003	0.3316704	0.1588072	0.09090745	0.141648	0.1511417
477.385	4	0.499044	0.3191268	0.1591932	0.0918757	0.145274	0.1514576
477.485	4	0.497919	0.3103673	0.1593287	0.09104074	0.1471	0.1501445
477.585	4	0.49147	0.320863	0.1571949	0.09245486	0.145092	0.1525395
477.685	4	0.501563	0.3220945	0.1592839	0.09491021	0.147795	0.1548383
477.785	4	0.500034	0.3153505	0.1533855	0.09541542	0.145165	0.1540902
477.885	4	0.502362	0.3108288	0.1594012	0.08903421	0.141634	0.148886
477.985	4	0.501728	0.3136142	0.1615494	0.09175771	0.142956	0.1529708
478.085	4	0.499874	0.3473426	0.1588173	0.09229105	0.143355	0.1552177
478.185	4	0.504716	0.3438098	0.1619831	0.08964097	0.144391	0.1550738
478.285	4	0.503909	0.3384038	0.1674853	0.09339523	0.137537	0.1560718
478.385	4	0.509792	0.3530663	0.1694952	0.09214248	0.13812	0.1570221
478.485	4	0.512778	0.3505768	0.1698472	0.08769041	0.142315	0.1531139
478.585	4	0.498291	0.3406116	0.1684742	0.09029453	0.146858	0.1528556
478.685	4	0.510209	0.33878	0.1680458	0.09312426	0.142804	0.1526866
478.785	4	0.507159	0.3324978	0.1679555	0.09412477	0.141135	0.1536376
478.885	4	0.508364	0.3342463	0.1663878	0.08961473	0.140709	0.1451764
478.985	4	0.512187	0.3336531	0.1656559	0.08253384	0.139548	0.1456448
479.085	4	0.505981	0.3402101	0.166717	0.0856833	0.139626	0.1484268
479.185	4	0.500532	0.3409416	0.1645537	0.08840912	0.139005	0.1511464
479.285	4	0.511672	0.336641	0.1642969	0.09231313	0.134939	0.1549038
479.385	4	0.517498	0.3362752	0.1645061	0.09575126	0.138908	0.1543884
479.485	4	0.506205	0.3401103	0.1595869	0.09796286	0.144897	0.1537176
479.585	4	0.508987	0.3250695	0.1599877	0.07854256	0.142965	0.1542701
479.778	4.5	0.487203	0.3373968	0.1579167	0.07928532	0.139938	0.1535928
479.878	4.5	0.484265	0.3480415	0.1568256	0.08396636	0.133751	0.1501576
479.978	4.5	0.49855	0.3403537	0.1595715	0.08493973	0.135442	0.1492865
480.078	4.5	0.503182	0.3359864	0.1571442	0.0988614	0.13373	0.1469377
480.178	4.5	0.503226	0.3361233	0.1554699	0.0821982	0.133104	0.1410843
480.278	4.5	0.51249	0.3355303	0.1558785	0.0852766	0.133864	0.1350469
480.378	4.5	0.504364	0.3328212	0.1562425	0.09618414	0.13508	0.1422687
480.478	4.5	0.50482	0.3336303	0.1553658	0.09946623	0.117503	0.142108
480.578	4.5	0.521624	0.3315054	0.1540086	0.09551519	0.124889	0.1423676
480.678	4.5	0.508391	0.3359509	0.1537876	0.09498906	0.127452	0.1438995
480.778	4.5	0.513416	0.3338391	0.151519	0.08585129	0.125823	0.1467583
480.878	4.5	0.50919	0.3381416	0.1510892	0.08630656	0.134584	0.1501189
480.978	4.5	0.504418	0.3206447	0.1482203	0.09066273	0.132452	0.1509389
481.078	4.5	0.532758	0.3094183	0.143773	0.09943768	0.135032	0.1525975
481.178	4.5	0.512922	0.3201606	0.1473748	0.09932312	0.132699	0.1506056
481.278	4.5	0.516079	0.320584	0.1458374	0.0991747	0.132568	0.1536819
481.378	4.5	0.50318	0.3124538	0.1473471	0.09530742	0.133342	0.1564596
481.478	4.5	0.502911	0.3046919	0.1457087	0.09632782	0.137951	0.1551888
481.578	4.5	0.510071	0.3091188	0.1472128	0.1006242	0.137068	0.1525882
481.678	4.5	0.501484	0.3425027	0.1452138	0.1022276	0.137289	0.1548817
481.778	4.5	0.511539	0.341503	0.1494303	0.09705217	0.135858	0.1545375

481.878	4.5	0.502559	0.3363023	0.1485079	0.09280079	0.140043	0.1544946
481.978	4.5	0.502668	0.3525291	0.150873	0.09113599	0.139155	0.1535556
482.078	4.5	0.512706	0.3465584	0.1505922	0.09656785	0.138487	0.1474248
482.178	4.5	0.509579	0.3376098	0.1508085	0.09635355	0.136585	0.1494601
482.278	4.5	0.498981	0.3346575	0.1463035	0.09845416	0.137631	0.1532726
482.378	4.5	0.509882	0.332375	0.1458551	0.1016696	0.13844	0.1565072
482.478	4.5	0.503481	0.3299236	0.1463598	0.09885545	0.136747	0.1558583
482.578	4.5	0.50712	0.3339604	0.1494453	0.0959521	0.135965	0.1580084
482.678	4.5	0.50843	0.3379306	0.1520585	0.08840368	0.139756	0.1561263
482.778	4.5	0.500046	0.3415592	0.1524925	0.09915659	0.139663	0.1564336
482.878	4.5	0.503223	0.3326546	0.156144	0.100396	0.135824	0.1580477
482.978	4.5	0.515724	0.3332619	0.1559625	0.09395138	0.136851	0.1595594
483.078	4.5	0.513538	0.3375447	0.1542805	0.09722684	0.135242	0.1604568
483.178	4.5	0.524266	0.328122	0.151535	0.09872089	0.136708	0.1657919
483.278	4.5	0.515591	0.3405416	0.1523654	0.1031693	0.13606	0.1644621
483.378	4.5	0.506928	0.3283608	0.1542857	0.09840207	0.135985	0.1624004
483.478	4.5	0.520657	0.3309746	0.1536687	0.09524807	0.137387	0.1633529
483.578	4.5	0.52331	0.3488423	0.1540669	0.09754939	0.139248	0.1553235
483.678	4.5	0.504196	0.3408876	0.155904	0.0892821	0.136628	0.1540269
483.778	4.5	0.501946	0.3367061	0.1576742	0.09247357	0.13942	0.152583
483.878	4.5	0.495523	0.3336986	0.1580868	0.09341192	0.137365	0.1516331
483.978	4.5	0.50192	0.3354874	0.1548099	0.09185979	0.140474	0.1546447
484.078	4.5	0.499202	0.3322576	0.1571454	0.09384301	0.136853	0.1511338
484.178	4.5	0.508832	0.3313143	0.1552618	0.08789705	0.135011	0.1453703
484.278	4.5	0.512275	0.3318074	0.1536662	0.09151419	0.124277	0.1495586
484.378	4.5	0.498961	0.3282287	0.1543603	0.09279557	0.13042	0.1567328
484.478	4.5	0.491401	0.3303421	0.1541878	0.09378812	0.129912	0.1570175
484.578	4.5	0.508143	0.3378077	0.1540928	0.08666664	0.135607	0.1561948
484.678	4.5	0.492952	0.3248545	0.1534626	0.08869317	0.136137	0.155428
484.778	4.5	0.500669	0.3100591	0.156425	0.09142141	0.135936	0.1552889
484.878	4.5	0.50175	0.306764	0.1536481	0.09008504	0.135802	0.153049
484.978	4.5	0.495388	0.3214242	0.1540654	0.0989108	0.133309	0.1546043
485.078	4.5	0.494624	0.3109596	0.154917	0.0986288	0.133216	0.1561034
485.178	4.5	0.510136	0.3080735	0.1556025	0.09253727	0.138155	0.1575399
485.278	4.5	0.501337	0.2981769	0.154715	0.09536701	0.135033	0.1600177
485.378	4.5	0.494746	0.3234589	0.1540581	0.09543014	0.134879	0.1606056
485.478	4.5	0.516732	0.3379746	0.1552838	0.09710312	0.135294	0.1617071
485.578	4.5	0.494723	0.3444652	0.1542092	0.1026241	0.131651	0.1585728
485.678	4.5	0.501085	0.3421913	0.1535293	0.09980468	0.130962	0.159654
485.778	4.5	0.501164	0.3448247	0.154352	0.09569335	0.133032	0.1573794
485.878	4.5	0.505924	0.342507	0.1534803	0.09728341	0.135246	0.1539928
485.978	4.5	0.481058	0.3343183	0.1537827	0.09903868	0.132002	0.1557088
486.078	4.5	0.507719	0.3313559	0.1522737	0.09935308	0.13549	0.1547838
486.178	4.5	0.482403	0.329287	0.1504918	0.09762392	0.132935	0.1552167
486.278	4.5	0.50164	0.3293595	0.1516303	0.1013182	0.133014	0.1593715
486.378	4.5	0.500802	0.3369377	0.1519362	0.1015452	0.136412	0.1626737
486.478	4.5	0.495026	0.3378552	0.1537256	0.1015564	0.135714	0.1645275
486.578	4.5	0.503524	0.3371365	0.1543746	0.1080518	0.135496	0.1615517
486.678	4.5	0.488838	0.328854	0.1550552	0.107674	0.135905	0.1633628
486.778	4.5	0.490703	0.3344785	0.1525373	0.1095045	0.134199	0.1646455
486.878	4.5	0.488667	0.323563	0.1509909	0.1130055	0.135903	0.1678384
486.978	4.5	0.48705	0.321674	0.1515981	0.1111379	0.137934	0.1702449
487.078	4.5	0.519334	0.3323655	0.1517946	0.1119079	0.136719	0.1615507
487.178	4.5	0.489137	0.3219434	0.1504854	0.1137309	0.136613	0.1613519
487.278	4.5	0.493807	0.3313789	0.1487638	0.1157124	0.137754	0.1641095
487.378	4.5	0.491476	0.3463008	0.1444189	0.1196857	0.138602	0.163185
487.478	4.5	0.503431	0.3364166	0.1478328	0.1162758	0.138019	0.164009
487.578	4.5	0.492306	0.3314617	0.1476602	0.1176052	0.135836	0.1586909
487.678	4.5	0.49633	0.3318373	0.1473508	0.1195907	0.138497	0.1614312
487.778	4.5	0.489986	0.3326283	0.1461838	0.1169943	0.141346	0.158866
487.878	4.5	0.498153	0.331236	0.1460647	0.119193	0.145619	0.1596005
487.978	4.5	0.464882	0.3292475	0.1457025	0.1196083	0.14008	0.1627404
488.078	4.5	0.454358	0.327503	0.1455425	0.1221044	0.138171	0.1643422
488.178	4.5	0.45073	0.3336206	0.146415	0.12072	0.143191	0.1640175
488.278	4.5	0.479928	0.3431429	0.1460854	0.1166321	0.138694	0.1609449
488.378	4.5	0.48154	0.3515222	0.1450746	0.1206309	0.139331	0.1619165
488.478	4.5	0.479816	0.3337708	0.1448799	0.1233034	0.141821	0.157856
488.578	4.5	0.485794	0.3191805	0.138621	0.1228911	0.14259	0.1607679
488.678	4.5	0.491526	0.3211028	0.1406837	0.1212739	0.144619	0.1612184
488.778	4.5	0.490666	0.3225253	0.1405923	0.122463	0.145076	0.1594275
488.878	4.5	0.493808	0.3178972	0.141411	0.1234343	0.147985	0.1512755
488.978	4.5	0.494441	0.3111274	0.1416145	0.1154698	0.143414	0.1413066
489.078	4.5	0.489919	0.3092258	0.140565	0.111221	0.146081	0.1439954
489.178	4.5	0.480692	0.336365	0.1407246	0.09785717	0.146636	0.1516147
489.278	4.5	0.485314	0.3425127	0.137096	0.1017825	0.146356	0.1538896
489.378	4.5	0.467543	0.3405466	0.1426431	0.09645258	0.143908	0.1459723
489.478	4.5	0.47737	0.3482959	0.1408032	0.1057777	0.141227	0.13024
489.578	4.5	0.494011	0.3446102	0.1380076	0.09835375	0.143368	0.1379811
489.678	4.5	0.490656	0.3366651	0.1400293	0.1027067	0.14447	0.1398282
489.778	4.5	0.487561	0.3329905	0.1386427	0.1023873	0.144604	0.1401979
489.878	4.5	0.491908	0.3342998	0.1400235	0.09937524	0.14892	0.1424794
489.978	4.5	0.490261	0.3369121	0.1416827	0.1033819	0.147546	0.1453171
490.078	4.5	0.487893	0.3379291	0.145006	0.1030213	0.145691	0.1388202
490.178	4.5	0.497159	0.3422228	0.1392414	0.09608868	0.149619	0.1392874
490.278	4.5	0.498347	0.3429909	0.142983	0.0973144	0.154268	0.1426431
490.378	4.5	0.496833	0.3356891	0.1404937	0.09660988	0.151707	0.1425945
490.478	4.5	0.493256	0.3301158	0.1379279	0.09223796	0.152795	0.1441404
490.578	4.5	0.5024	0.3320411	0.1436505	0.09365408	0.147571	0.1455172
490.678	4.5	0.507298	0.3252819	0.1443618	0.08998824	0.144381	0.1443766
490.778	4.5	0.501066	0.3346334	0.14616	0.092609	0.144909	0.144146
490.878	4.5	0.497009	0.3232869	0.1495892	0.08920477	0.142467	0.1450429
490.978	4.5	0.500623	0.3294553	0.1506627	0.0917528	0.146608	0.1452571
491.078	4.5	0.502386	0.3492698	0.1417284	0.09251277	0.144673	0.1453003
491.178	4.5	0.512292	0.3411902	0.1410981	0.09370048	0.143943	0.1469857
491.278	4.5	0.511175	0.3387055	0.1436951	0.09083021	0.146309	0.1482847

491.378	4.5	0.501244	0.3374093	0.1432565	0.09311628	0.144173	0.1460062
491.478	4.5	0.506053	0.3358526	0.142605	0.09274182	0.140346	0.1464746
491.578	4.5	0.510547	0.3333305	0.1428862	0.09020367	0.141858	0.1467508
491.678	4.5	0.514425	0.329792	0.143736	0.09328414	0.138554	0.1468546
491.778	4.5	0.497861	0.3334386	0.1432322	0.08767335	0.139508	0.1487062
491.878	4.5	0.508052	0.3290094	0.1451029	0.08870908	0.142369	0.1496
491.978	4.5	0.509182	0.3373739	0.146927	0.08997919	0.144656	0.1490994
492.078	4.5	0.513169	0.3500639	0.1470411	0.09333877	0.146842	0.1482805
492.178	4.5	0.508314	0.339373	0.1460983	0.08690896	0.143696	0.1444146
492.278	4.5	0.503089	0.322061	0.1453617	0.08093815	0.13984	0.1464579
492.378	4.5	0.51578	0.3186527	0.1443373	0.08818451	0.138645	0.1476916
492.478	4.5	0.502178	0.3204142	0.1427377	0.09100622	0.142939	0.1469601
492.578	4.5	0.500777	0.3163719	0.1444823	0.09332424	0.142571	0.1474226
492.678	4.5	0.511951	0.3160948	0.1478956	0.09681536	0.143701	0.1470904
492.778	4.5	0.512373	0.3049657	0.1496449	0.08200365	0.146015	0.1488735
492.878	4.5	0.508276	0.3231769	0.1488531	0.08179463	0.144485	0.1481447
492.978	4.5	0.502821	0.3386388	0.1510079	0.08771576	0.138612	0.1463366
493.078	4.5	0.493538	0.3434693	0.1473853	0.08489981	0.140409	0.1465124
493.178	4.5	0.485027	0.3405167	0.1480181	0.08721346	0.139726	0.1455284
493.278	4.5	0.495007	0.3432553	0.1484995	0.09550095	0.138974	0.1470978
493.378	4.5	0.486508	0.3403258	0.1507389	0.101755	0.133464	0.1473829
493.478	4.5	0.50241	0.3302104	0.1511399	0.09846682	0.136931	0.1454983
493.578	4.5	0.513645	0.3315331	0.1513713	0.08279566	0.14049	0.1458368
493.678	4.5	0.506814	0.33376	0.1501952	0.09295955	0.144697	0.1434952
493.778	4.5	0.504783	0.334257	0.1542078	0.1029717	0.140834	0.1467889
493.878	4.5	0.50233	0.3412378	0.1535004	0.1041939	0.139357	0.1472344
493.978	4.5	0.511571	0.3417351	0.1527292	0.09786237	0.138751	0.1495502
494.078	4.5	0.510768	0.3396193	0.151057	0.09195641	0.13815	0.1487247
494.178	4.5	0.507521	0.3364014	0.1490224	0.08331368	0.137783	0.1505523
494.278	4.5	0.509156	0.3434168	0.1496525	0.08740153	0.133138	0.1518564
494.378	4.5	0.513242	0.3348715	0.150462	0.09139803	0.129322	0.1512547
494.478	4.5	0.516894	0.3373375	0.1537486	0.1012173	0.136222	0.1526584
494.578	4.5	0.511264	0.34072	0.151762	0.1013844	0.137999	0.1515831
494.678	4.5	0.514346	0.3359325	0.1515049	0.09896398	0.136168	0.1509747
494.778	4.5	0.496897	0.3418293	0.1524697	0.09346015	0.132942	0.1522037
494.878	4.5	0.504753	0.3537443	0.1523449	0.09559613	0.134056	0.1526165
494.978	4.5	0.507794	0.3433815	0.1562652	0.1003661	0.132954	0.1489532
495.078	4.5	0.500459	0.3398006	0.1529077	0.09752335	0.133334	0.149922
495.178	4.5	0.503567	0.3384224	0.1569612	0.09700285	0.131806	0.1495864
495.278	4.5	0.512008	0.3387693	0.1533771	0.09244002	0.1319	0.1508714
495.378	4.5	0.500768	0.3323718	0.1522646	0.09779739	0.128954	0.1532409
495.478	4.5	0.508889	0.3317411	0.1533341	0.09928297	0.11408	0.1525693
495.578	4.5	0.508944	0.3307329	0.1512979	0.09883501	0.127826	0.152961
495.678	4.5	0.50353	0.3375216	0.1525932	0.1027869	0.124028	0.1548304
495.778	4.5	0.505956	0.3448097	0.1532158	0.1019917	0.130696	0.1551425
495.878	4.5	0.498192	0.3480414	0.1523967	0.09780276	0.131387	0.1573666
495.978	4.5	0.493986	0.3276182	0.1524986	0.0885276	0.127074	0.1575756
496.078	4.5	0.505446	0.319073	0.1513736	0.09633712	0.13289	0.157072
496.178	4.5	0.501135	0.3154272	0.1539639	0.102081	0.130627	0.1570208
496.278	4.5	0.507544	0.3154125	0.1541186	0.1012645	0.131051	0.1560809
496.378	4.5	0.505514	0.310947	0.1511435	0.09676135	0.130974	0.1562101
496.478	4.5	0.518116	0.3117115	0.1519474	0.1018605	0.133865	0.158446
496.578	4.5	0.512827	0.3051024	0.1532473	0.1038664	0.130993	0.1591232
496.678	4.5	0.513273	0.3361261	0.1538231	0.1023341	0.134295	0.1572771
496.778	4.5	0.507159	0.3395359	0.1563491	0.09505119	0.131633	0.1596612
496.878	4.5	0.532031	0.3355665	0.1549275	0.09852389	0.134435	0.1639664
496.978	4.5	0.504512	0.3426494	0.1580075	0.09510978	0.13387	0.162182
497.078	4.5	0.497166	0.3429209	0.1563008	0.09050034	0.132881	0.1610643
497.178	4.5	0.499057	0.3349316	0.1559092	0.0947201	0.130745	0.1593761
497.278	4.5	0.495442	0.3326574	0.1543593	0.09123681	0.131569	0.1604817
497.378	4.5	0.490718	0.3307859	0.1569983	0.09084083	0.134485	0.1599648
497.478	4.5	0.494538	0.3343528	0.1585199	0.09077135	0.133542	0.1589252
497.578	4.5	0.520448	0.3362382	0.1582353	0.09366585	0.133703	0.1641919
497.678	4.5	0.50099	0.3390822	0.157681	0.09171223	0.139496	0.1642285
497.778	4.5	0.503425	0.3426477	0.1586684	0.09479476	0.137478	0.1640902
497.878	4.5	0.500861	0.335305	0.1540907	0.08883302	0.136306	0.1639416
497.978	4.5	0.492139	0.3401998	0.1592168	0.08734084	0.136714	0.1614033
498.078	4.5	0.494056	0.3367617	0.1604316	0.0915724	0.132521	0.1570482
498.178	4.5	0.500798	0.3298435	0.1538315	0.08919398	0.13367	0.1600066
498.278	4.5	0.495698	0.3371319	0.1554514	0.09761821	0.134622	0.1620993
498.378	4.5	0.488866	0.331158	0.1616141	0.09904071	0.134214	0.1641795
498.478	4.5	0.496857	0.331658	0.1577078	0.09515147	0.137246	0.1633212
498.578	4.5	0.514149	0.3488608	0.1578584	0.09807933	0.139617	0.1645138
498.678	4.5	0.500503	0.3429114	0.1603792	0.1003719	0.13789	0.1646418
498.778	4.5	0.508717	0.3389508	0.1616908	0.09868303	0.138053	0.1602801
498.878	4.5	0.496625	0.336763	0.1602533	0.1026504	0.136834	0.1620343
498.978	4.5	0.501856	0.3377161	0.162049	0.1033663	0.138496	0.1655246
499.078	4.5	0.499739	0.3351521	0.1655094	0.09874173	0.134589	0.1658785
499.178	4.5	0.487061	0.3327955	0.1636112	0.09627984	0.130823	0.1637147
499.278	4.5	0.498407	0.3335885	0.1605173	0.1008372	0.119086	0.1653266
499.378	4.5	0.506899	0.3291685	0.159688	0.1022183	0.127549	0.1646935
499.478	4.5	0.487553	0.3371063	0.1559811	0.09900443	0.128966	0.167202
499.578	4.5	0.491616	0.3531128	0.155341	0.1012077	0.133239	0.1665626
499.678	4.5	0.49449	0.3381229	0.1568934	0.1030547	0.132853	0.1699489
499.778	4.5	0.508331	0.3187739	0.1543712	0.103136	0.133388	0.1638923
499.878	4.5	0.505366	0.3114202	0.153281	0.1081358	0.133858	0.1584782
499.978	4.5	0.488315	0.3144398	0.1540164	0.1084589	0.132851	0.1594189
500.078	4.5	0.49181	0.3133439	0.1519734	0.1068788	0.133733	0.1577583
500.178	4.5	0.489649	0.3170858	0.1515013	0.1146481	0.137942	0.1624137
500.278	4.5	0.485804	0.3046707	0.1514034	0.1093563	0.13756	0.1600964
500.378	4.5	0.499631	0.3205559	0.153403	0.1103034	0.137597	0.15456
500.478	4.5	0.507227	0.3377491	0.1527158	0.1160203	0.137089	0.1546059
500.578	4.5	0.496907	0.3450641	0.1500912	0.1178652	0.134343	0.1542133
500.678	4.5	0.491434	0.3398113	0.1489529	0.1216675	0.133501	0.1583038
500.778	4.5	0.501383	0.3407839	0.1476388	0.116747	0.137076	0.1580176

500.878	4.5	0.49745	0.3412506	0.1487266	0.1162302	0.139262	0.1612698
500.978	4.5	0.503383	0.3303964	0.146599	0.1196197	0.132789	0.1501785
501.078	4.5	0.497558	0.330375	0.1418596	0.1193168	0.132912	0.1502936
501.178	4.5	0.491129	0.3307711	0.1460814	0.1195117	0.131339	0.1523297
501.278	4.5	0.473884	0.3319049	0.1473063	0.1190282	0.128925	0.1509356
501.378	4.5	0.456494	0.3393303	0.1471936	0.1192315	0.133836	0.1531153
501.478	4.5	0.451587	0.3406969	0.1452897	0.120413	0.13335	0.1559829
501.578	4.5	0.461959	0.3379427	0.1472578	0.117873	0.129942	0.1492167
501.678	4.5	0.476281	0.3325019	0.1448528	0.1181697	0.132618	0.1527856
501.778	4.5	0.470672	0.3407437	0.1469481	0.1229192	0.128863	0.1542546
501.878	4.5	0.48838	0.3306764	0.1474549	0.1239198	0.132116	0.1545772
501.978	4.5	0.488409	0.33098	0.1475682	0.1171821	0.136488	0.1561789
502.078	4.5	0.478814	0.3375422	0.1466809	0.1197699	0.133189	0.1520675
502.178	4.5	0.500525	0.3280641	0.1480262	0.1223294	0.13358	0.1513065
502.278	4.5	0.482121	0.3324315	0.1431872	0.1169061	0.134075	0.1520578
502.378	4.5	0.492824	0.3512307	0.14161	0.1133853	0.135805	0.1502672
502.478	4.5	0.484442	0.3390193	0.1432457	0.103513	0.134735	0.1449025
502.578	4.5	0.479353	0.3353262	0.1462894	0.1025616	0.134864	0.1489063
502.678	4.5	0.491732	0.3332297	0.1474899	0.09937881	0.137115	0.1524629
502.778	4.5	0.473337	0.3337436	0.1495078	0.101871	0.141507	0.1536465
502.878	4.5	0.495001	0.3308851	0.1500732	0.09970282	0.145392	0.1527194
502.978	4.5	0.490818	0.3299461	0.1499105	0.09907571	0.141477	0.1526221
503.078	4.5	0.495277	0.3300683	0.1506177	0.1061071	0.142168	0.151762
503.178	4.5	0.491975	0.3325861	0.1492677	0.1057515	0.146464	0.1480832
503.278	4.5	0.496429	0.3401687	0.1476467	0.1063503	0.140066	0.1463534
503.378	4.5	0.490274	0.3462674	0.1472728	0.1070567	0.139083	0.1439532
503.478	4.5	0.491982	0.3259001	0.1467462	0.1005171	0.143135	0.1329492
503.578	4.5	0.493983	0.3105828	0.1507191	0.0984932	0.143833	0.1387083
503.678	4.5	0.493668	0.3084182	0.1538583	0.09838832	0.147001	0.1429198
503.778	4.5	0.490404	0.3131801	0.1530155	0.094757	0.147051	0.1427805
503.878	4.5	0.503005	0.3160634	0.1545983	0.09152674	0.147934	0.1450776
503.978	4.5	0.502268	0.3084114	0.1505919	0.09108289	0.143073	0.1483934
504.078	4.5	0.500104	0.3022548	0.1547374	0.0884698	0.147253	0.1476325
504.178	4.5	0.489031	0.33217	0.1535696	0.08893476	0.146151	0.1513302
504.278	4.5	0.493675	0.3375036	0.1527595	0.08892539	0.144719	0.1510778
504.378	4.5	0.49735	0.3315696	0.1534418	0.08941329	0.142822	0.1524078
504.478	4.5	0.486505	0.3347626	0.1535909	0.09114837	0.141502	0.1526172
504.578	4.5	0.508715	0.3388693	0.1514296	0.08736743	0.142221	0.1514761
504.678	4.5	0.501161	0.3325958	0.152957	0.0902817	0.14266	0.1537216
504.778	4.5	0.506855	0.3277093	0.1570245	0.09101385	0.142662	0.1508106
504.878	4.5	0.502805	0.3282388	0.1546241	0.08998557	0.145714	0.1502481
504.978	4.5	0.507621	0.3303368	0.1541199	0.09447702	0.143367	0.1467905
505.078	4.5	0.506923	0.3332626	0.1545977	0.09178889	0.14436	0.1491983
505.178	4.5	0.507267	0.3387235	0.1558903	0.09130325	0.150332	0.1520241
505.278	4.5	0.503621	0.3415057	0.1561672	0.09296966	0.150712	0.152132
505.378	4.5	0.505832	0.3295222	0.1545285	0.09460794	0.152093	0.1564667
505.478	4.5	0.506553	0.3368218	0.1527173	0.0936491	0.149611	0.1533311
505.578	4.5	0.507267	0.3324347	0.1511789	0.08431535	0.146628	0.1540328
505.678	4.5	0.50507	0.322671	0.1496706	0.09074181	0.144522	0.1572778
505.778	4.5	0.503711	0.3334584	0.1523533	0.09136669	0.142046	0.1566089
505.878	4.5	0.500946	0.3233739	0.1501687	0.09624832	0.142616	0.1608822
505.978	4.5	0.500182	0.3222601	0.150324	0.09962562	0.149438	0.1610481
506.078	4.5	0.507314	0.3444225	0.1506525	0.0856789	0.14977	0.1600869
506.178	4.5	0.50991	0.3374976	0.147806	0.08360458	0.147946	0.1566769
506.278	4.5	0.502968	0.3355699	0.1501606	0.09052242	0.149124	0.1531601
506.378	4.5	0.504346	0.3317198	0.1499512	0.08743189	0.145695	0.1515378
506.478	4.5	0.48375	0.3324001	0.1511392	0.08917438	0.140744	0.1507258
506.578	4.5	0.484441	0.3309466	0.1497397	0.1002687	0.14394	0.1508542
506.678	4.5	0.49107	0.3289463	0.1511122	0.1060193	0.137903	0.1442131
506.778	4.5	0.500173	0.3289157	0.1503426	0.1014209	0.140275	0.1474493
506.878	4.5	0.500889	0.3251566	0.1502301	0.08725791	0.142511	0.1538827
506.978	4.5	0.508613	0.3313237	0.1488198	0.09591147	0.145992	0.154558
507.078	4.5	0.505341	0.3453449	0.1459427	0.1048362	0.148973	0.1509731
507.178	4.5	0.498243	0.3348934	0.1483932	0.1063188	0.146368	0.1496509
507.278	4.5	0.510987	0.3090952	0.1480964	0.1012539	0.143087	0.1496419
507.378	4.5	0.505551	0.3046699	0.1465832	0.09562647	0.141357	0.1502156
507.478	4.5	0.505902	0.308309	0.1485648	0.08686787	0.145078	0.1531696
507.578	4.5	0.504668	0.3100268	0.1500871	0.09368279	0.144214	0.154851
507.678	4.5	0.509344	0.3081726	0.1469669	0.09876481	0.144622	0.1543826
507.778	4.5	0.52426	0.3080001	0.1464612	0.1068232	0.149185	0.1568861
507.878	4.5	0.507291	0.3191919	0.14652	0.1030322	0.145146	0.1547993
507.978	4.5	0.516651	0.3315148	0.1459786	0.09951399	0.140611	0.1526906
508.078	4.5	0.494939	0.3327678	0.1456327	0.09572605	0.140591	0.153761
508.178	4.5	0.504867	0.3306831	0.1472259	0.09481959	0.140394	0.1515941
508.278	4.5	0.511076	0.3317425	0.1476073	0.09874813	0.143269	0.1513172
508.378	4.5	0.499207	0.3371432	0.1470453	0.09875909	0.13707	0.1513964
508.478	4.5	0.506396	0.3248739	0.1453979	0.1006331	0.135542	0.1554859
508.578	4.5	0.508424	0.3251247	0.1413741	0.0916434	0.141056	0.1581219
508.678	4.5	0.503803	0.3264932	0.1417154	0.09719887	0.146466	0.1577893
508.778	4.5	0.50968	0.3293467	0.1426962	0.1001588	0.144734	0.1587646
508.878	4.5	0.51323	0.338256	0.1429297	0.09971697	0.141276	0.1607511
508.978	4.5	0.496704	0.3407668	0.1433458	0.1033086	0.141083	0.1671244
509.078	4.5	0.499776	0.3324113	0.1408736	0.1043757	0.13941	0.1645579
509.178	4.5	0.498584	0.3264317	0.1402704	0.09990019	0.139251	0.1594555
509.278	4.5	0.493912	0.3376934	0.1374449	0.09293762	0.137589	0.1615969
509.378	4.5	0.509406	0.3177857	0.1413564	0.09662668	0.130278	0.1611548
509.478	4.5	0.495255	0.3214796	0.1435998	0.1029954	0.134064	0.1604667
509.578	4.5	0.498534	0.3268829	0.1417375	0.1039772	0.141039	0.1578435
509.678	4.5	0.509108	0.318387	0.1419226	0.09572929	0.138419	0.1615701
509.778	4.5	0.512513	0.3266677	0.1415863	0.09929228	0.135707	0.1577582
509.878	4.5	0.520651	0.3451556	0.1430631	0.1021115	0.131814	0.1580538
509.978	4.5	0.516068	0.3375332	0.1426957	0.1060668	0.13429	0.1561304
510.078	4.5	0.507613	0.334515	0.1429624	0.0980342	0.1336	0.1559383
510.178	4.5	0.519197	0.3343412	0.1382426	0.09973576	0.133163	0.1592412
510.278	4.5	0.512887	0.3344253	0.1395336	0.09986698	0.131252	0.1544144

510.378	4.5	0.501381	0.3290905	0.1413683	0.09053382	0.135163	0.1578138
510.478	4.5	0.502487	0.3263526	0.1343619	0.09586252	0.115755	0.1579039
510.578	4.5	0.490763	0.3248159	0.1400492	0.094937	0.124155	0.1529218
510.678	4.5	0.495208	0.3297706	0.1398617	0.09475853	0.128948	0.1383431
510.778	4.5	0.497418	0.3350613	0.140164	0.09497934	0.132674	0.139532
510.878	4.5	0.510939	0.3426154	0.140115	0.09053164	0.130057	0.1497761
510.978	4.5	0.503496	0.3168307	0.1459247	0.09308967	0.129764	0.1512297
511.078	4.5	0.49787	0.3055805	0.1409346	0.09273935	0.131072	0.1373226
511.178	4.5	0.493913	0.3059588	0.1393678	0.09043232	0.132077	0.1309945
511.278	4.5	0.495155	0.3099772	0.1429434	0.08816337	0.130592	0.1380097
511.378	4.5	0.496314	0.3109882	0.1421509	0.09261011	0.130509	0.1382435
511.478	4.5	0.493203	0.311176	0.1453051	0.09145681	0.132691	0.1398324
511.578	4.5	0.486166	0.3056237	0.1431672	0.0945586	0.132046	0.1412376
511.678	4.5	0.483031	0.3309231	0.1442909	0.0996595	0.131288	0.1344318
511.778	4.5	0.495961	0.3412986	0.1434222	0.09758954	0.133225	0.1389372
511.878	4.5	0.509774	0.343345	0.1439665	0.09587238	0.133472	0.1392045
511.978	4.5	0.493733	0.3416891	0.1466046	0.09945155	0.131935	0.1369493
512.078	4.5	0.497237	0.3421643	0.1478034	0.0967163	0.132962	0.1362508
512.178	4.5	0.502389	0.3361195	0.1471457	0.1016705	0.136252	0.1349876
512.278	4.5	0.49825	0.3313853	0.1467749	0.1046328	0.136308	0.1389706
512.378	4.5	0.493301	0.3326952	0.144686	0.102563	0.136082	0.1375695
512.478	4.5	0.488024	0.3347117	0.1437952	0.09875461	0.133513	0.137322
512.578	4.5	0.504793	0.3388632	0.1420868	0.1008389	0.131631	0.138877
512.678	4.5	0.490541	0.34422	0.144705	0.1029077	0.131663	0.1400785
512.778	4.5	0.503351	0.341998	0.1452125	0.1014921	0.131145	0.1403017
512.878	4.5	0.479796	0.3262507	0.1472977	0.1018686	0.129694	0.1399944
512.978	4.5	0.492197	0.3353285	0.1486901	0.1011894	0.13376	0.1424505
513.078	4.5	0.511178	0.3299043	0.1449901	0.1035011	0.13331	0.1426731
513.178	4.5	0.49268	0.3269611	0.1462641	0.1071808	0.135044	0.1443459
513.278	4.5	0.492512	0.3416681	0.1455271	0.111982	0.133826	0.1440715
513.378	4.5	0.488903	0.33547	0.1484386	0.1057154	0.135403	0.1430006
513.478	4.5	0.486734	0.3296423	0.1493442	0.1136032	0.133888	0.1426293
513.578	4.5	0.482391	0.3429687	0.1471407	0.1122224	0.12249	0.1464907
513.678	4.5	0.491312	0.3521425	0.1459447	0.110153	0.129581	0.1448506
513.778	4.5	0.510934	0.3452299	0.1477055	0.1150587	0.13144	0.1431105
513.878	4.5	0.493095	0.3379491	0.1466337	0.118567	0.134322	0.145108
513.978	4.5	0.486886	0.3393177	0.1481441	0.1218353	0.134825	0.1441858
514.078	4.5	0.491202	0.3332925	0.1477533	0.116735	0.13606	0.1419145
514.178	4.5	0.496407	0.334051	0.1430609	0.1180159	0.135426	0.142531
514.278	4.5	0.491859	0.3377673	0.1427903	0.1196923	0.134976	0.142874
514.378	4.5	0.499768	0.334957	0.1435668	0.1222673	0.136524	0.1443307
514.478	4.5	0.490839	0.3393914	0.1475941	0.1229739	0.138827	0.1446772
514.578	4.5	0.474868	0.3515096	0.1479118	0.1183521	0.139439	0.1464041
514.678	4.5	0.456575	0.3364686	0.1453629	0.1178306	0.139392	0.1436547
514.778	4.5	0.44884	0.3125367	0.1464323	0.119201	0.135817	0.1447887
514.878	4.5	0.450016	0.3169995	0.1485409	0.1173813	0.133666	0.1464902
514.978	4.5	0.470699	0.3251045	0.1497827	0.1185335	0.139003	0.1488778
515.078	4.5	0.470675	0.3163666	0.1477758	0.1241158	0.138133	0.150388
515.178	4.5	0.48338	0.3155334	0.1479379	0.1268426	0.136492	0.1512194
515.278	4.5	0.481231	0.3093653	0.1452386	0.1218421	0.135353	0.150532
515.378	4.5	0.480954	0.3167576	0.1458444	0.1251064	0.137203	0.1530202
515.478	4.5	0.498222	0.3353613	0.1461261	0.1249144	0.138911	0.1518524
515.578	4.5	0.482755	0.3448609	0.1446062	0.1176494	0.138108	0.1531295
515.678	4.5	0.490003	0.3472973	0.1450763	0.1124203	0.135566	0.1535393
515.778	4.5	0.484041	0.345936	0.1468571	0.09968904	0.132409	0.1504044
515.878	4.5	0.473613	0.3454514	0.1508428	0.1010578	0.133383	0.1531996
515.978	4.5	0.487023	0.3379055	0.1486831	0.09906669	0.138148	0.1513991
516.078	4.5	0.468911	0.3330629	0.1495699	0.1018351	0.13857	0.1518306
516.178	4.5	0.497477	0.3336011	0.1511193	0.09838033	0.136803	0.1534202
516.278	4.5	0.487376	0.332874	0.148569	0.09836274	0.135104	0.1553946
516.378	4.5	0.484871	0.3410565	0.1462229	0.1068263	0.138143	0.1561747
516.478	4.5	0.488929	0.3401626	0.1509012	0.1037573	0.138823	0.1575378
516.578	4.5	0.496745	0.3365238	0.1529253	0.1045611	0.138453	0.1583124
516.678	4.5	0.486719	0.3236813	0.1540728	0.1074329	0.141923	0.1585398
516.778	4.5	0.490961	0.3439601	0.1549669	0.09959985	0.145749	0.157912
516.878	4.5	0.491643	0.3268103	0.1542952	0.1003542	0.145247	0.1580247
516.978	4.5	0.496091	0.3367271	0.1572007	0.1017795	0.141149	0.158698
517.078	4.5	0.489309	0.3452755	0.1549357	0.09556201	0.147839	0.1601262
517.178	4.5	0.498644	0.3349139	0.1572457	0.092724	0.141953	0.1589979
517.278	4.5	0.502972	0.333822	0.1529334	0.09219922	0.142437	0.1651802
517.378	4.5	0.502031	0.3500186	0.1556934	0.08900801	0.14579	0.1626768
517.478	4.5	0.492969	0.3460184	0.1571254	0.08744147	0.143561	0.1600616
517.578	4.5	0.490906	0.3381488	0.1544691	0.08572561	0.146536	0.1602174
517.678	4.5	0.49093	0.3360187	0.1542031	0.08671062	0.149017	0.1612223
517.778	4.5	0.495956	0.3363726	0.1545565	0.09150668	0.14456	0.1588527
517.878	4.5	0.503129	0.3309838	0.1505983	0.08720075	0.146567	0.1636532
517.978	4.5	0.50361	0.3372943	0.1574461	0.09100866	0.144917	0.1665403
518.078	4.5	0.499627	0.3345879	0.1569367	0.09457174	0.143279	0.1614613
518.178	4.5	0.493154	0.3415331	0.1538532	0.0938804	0.141396	0.1578726
518.278	4.5	0.505038	0.3431234	0.1564971	0.09773223	0.140692	0.1545429
518.378	4.5	0.508782	0.3504484	0.1570596	0.09609356	0.143153	0.1541237
518.478	4.5	0.495684	0.3267845	0.1590899	0.0948858	0.143294	0.1565292
518.578	4.5	0.506653	0.3190303	0.1597668	0.09671097	0.1461	0.1575318
518.678	4.5	0.500091	0.3245497	0.1607528	0.09568675	0.147568	0.1572526
518.778	4.5	0.504428	0.3237048	0.1614917	0.09698325	0.147857	0.1602884
518.878	4.5	0.505819	0.3218247	0.1588477	0.09032893	0.153456	0.1561139
518.978	4.5	0.494586	0.3150599	0.1611529	0.09132351	0.152583	0.1579331
519.078	4.5	0.510773	0.3088229	0.1614877	0.09433451	0.156448	0.1618247
519.178	4.5	0.49775	0.3343081	0.1586728	0.09955815	0.151552	0.1607792
519.278	4.5	0.502775	0.3434392	0.1586978	0.1009253	0.146282	0.1605853
519.378	4.5	0.51236	0.3500243	0.1574681	0.09594669	0.144869	0.1614158
519.478	4.5	0.511431	0.3484409	0.1554385	0.08011441	0.142668	0.1612482
519.578	4.5	0.501329	0.3427745	0.1566149	0.08953951	0.146558	0.1616221
519.678	4.5	0.505038	0.3394452	0.157502	0.09332068	0.146633	0.1626607
519.778	4.5	0.481167	0.3330271	0.1564382	0.07998508	0.147083	0.1569435

519.878	4.5	0.476443	0.3319246	0.1560181	0.09506593	0.15084	0.1559748
519.978	4.5	0.49265	0.335116	0.1544485	0.1033996	0.145	0.1554364
520.078	4.5	0.488854	0.3368076	0.1502824	0.1044175	0.143441	0.1579858
520.178	4.5	0.500247	0.3388096	0.1509788	0.08977163	0.144786	0.1547504
520.278	4.5	0.514728	0.3430345	0.1486613	0.09221318	0.139747	0.1508156
520.378	4.5	0.501461	0.325189	0.1488645	0.1008599	0.141315	0.1517936
520.478	4.5	0.497673	0.3346721	0.1474343	0.1058132	0.146018	0.1557257
520.578	4.5	0.512674	0.3371727	0.1473585	0.1028295	0.14683	0.1556497
520.678	4.5	0.503758	0.3334086	0.1467087	0.09946425	0.142689	0.156113
520.778	4.5	0.507098	0.3452718	0.1442939	0.08715911	0.141271	0.1549441
520.878	4.5	0.501485	0.3373467	0.1446784	0.08982805	0.140515	0.1606168
520.978	4.5	0.501242	0.3296087	0.142605	0.09651829	0.143187	0.1538937
521.078	4.5	0.527135	0.3453718	0.1416238	0.1020695	0.143719	0.1513901
521.178	4.5	0.503949	0.3415979	0.144804	0.09863891	0.146496	0.1520415
521.278	4.5	0.515009	0.338378	0.1468937	0.1009296	0.144198	0.148285
521.378	4.5	0.495068	0.3304029	0.1462695	0.09909087	0.137891	0.1532477
521.478	4.5	0.501969	0.3301585	0.1433486	0.101001	0.138754	0.1559239
521.578	4.5	0.511827	0.3287883	0.1448176	0.09915233	0.140584	0.148081
521.678	4.5	0.499509	0.329113	0.1437815	0.09799623	0.139209	0.151912
521.778	4.5	0.507994	0.3330353	0.1458863	0.09756859	0.136417	0.1538617
521.878	4.5	0.504512	0.3308036	0.1455605	0.09480636	0.140047	0.155066
521.978	4.5	0.50344	0.3375772	0.1447337	0.09136144	0.143233	0.1581442
522.078	4.5	0.5085	0.3507077	0.1451825	0.09795365	0.139594	0.1530041
522.178	4.5	0.508912	0.3409844	0.1470855	0.09910215	0.139394	0.150543
522.278	4.5	0.49502	0.315406	0.1425142	0.100142	0.137124	0.1494647
522.378	4.5	0.503589	0.3154822	0.1411883	0.1026687	0.137798	0.1504916
522.478	4.5	0.492826	0.3227508	0.1425683	0.09836596	0.136118	0.1427374
522.578	4.5	0.495025	0.3143303	0.1451463	0.09187066	0.131456	0.1475002
522.678	4.5	0.507366	0.3169225	0.148159	0.0936308	0.13542	0.1488688
522.778	4.5	0.495953	0.3115279	0.1482855	0.1006522	0.13894	0.1498974
522.878	4.5	0.498666	0.3139091	0.1489199	0.1026677	0.137572	0.1504635
522.978	4.5	0.507603	0.336018	0.1473226	0.09815191	0.132663	0.1514338
523.078	4.5	0.509999	0.3487409	0.148998	0.0997249	0.133485	0.1498616
523.178	4.5	0.527145	0.3500221	0.1501067	0.1026856	0.133532	0.1457476
523.278	4.5	0.508804	0.3429378	0.1488279	0.1057434	0.13417	0.1440438
523.378	4.5	0.505209	0.3417852	0.1504395	0.09966823	0.131951	0.1408654
523.478	4.5	0.519087	0.3331501	0.1462352	0.1007941	0.13515	0.1322345
523.578	4.5	0.512694	0.3310809	0.1508583	0.1019037	0.118494	0.134119
523.678	4.5	0.503191	0.3306368	0.1535626	0.09211211	0.128322	0.1365563
523.778	4.5	0.498777	0.3321704	0.1529977	0.09652935	0.128282	0.1364935
523.878	4.5	0.495548	0.3380652	0.1529356	0.09653921	0.130261	0.1385134
523.978	4.5	0.501109	0.3390633	0.1515925	0.09546709	0.131293	0.1425783
524.078	4.5	0.49763	0.3316115	0.1526286	0.09565808	0.132079	0.144089
524.178	4.5	0.512197	0.3253961	0.155567	0.09206031	0.132735	0.1468375
524.278	4.5	0.502547	0.3407945	0.1520282	0.09442868	0.129291	0.1472516
524.378	4.5	0.502402	0.3298241	0.1525001	0.09378229	0.129518	0.1493311
524.478	4.5	0.491326	0.3352894	0.150722	0.09271729	0.133604	0.1489308
524.578	4.5	0.501915	0.338824	0.1496602	0.08997367	0.132026	0.1468422
524.678	4.5	0.488209	0.3261648	0.149785	0.09168068	0.132708	0.1474139
524.778	4.5	0.492762	0.325562	0.1525614	0.09059603	0.130145	0.1465446
524.878	4.5	0.484915	0.3436904	0.1524078	0.0931559	0.130037	0.1460191
524.978	4.5	0.484584	0.3386041	0.1508864	0.09865334	0.129048	0.1431646
525.078	4.5	0.494491	0.3320637	0.1488327	0.09880384	0.129333	0.1443106
525.178	4.5	0.513472	0.3296085	0.1497005	0.09595954	0.130016	0.1487372
525.278	4.5	0.488058	0.3303806	0.150937	0.1002859	0.132258	0.1503265
525.378	4.5	0.492796	0.3281954	0.1500772	0.09747653	0.133384	0.1511687
525.478	4.5	0.504223	0.3271488	0.1483743	0.1028296	0.134421	0.1510188
525.578	4.5	0.494797	0.325614	0.1487682	0.1055475	0.137017	0.1502071
525.678	4.5	0.496194	0.3336087	0.1477519	0.1017557	0.134994	0.1529322
525.778	4.5	0.489524	0.3404621	0.1466719	0.09821321	0.134361	0.1531031
525.878	4.5	0.500243	0.3450057	0.1473611	0.09851442	0.12894	0.1575268
525.978	4.5	0.480352	0.3228384	0.1468929	0.1011771	0.130653	0.1593361
526.078	4.5	0.511342	0.3112015	0.1487157	0.1018012	0.130391	0.1562058
526.178	4.5	0.48097	0.3131518	0.1492964	0.1018602	0.129384	0.1554783
526.278	4.5	0.495085	0.3134615	0.1478233	0.1016829	0.13299	0.1494289
526.378	4.5	0.507198	0.3111342	0.1463036	0.10075	0.132456	0.1491382
526.478	4.5	0.482024	0.3088142	0.1495244	0.1003821	0.134486	0.148365
526.578	4.5	0.496068	0.3033662	0.1512398	0.108001	0.136189	0.1516629
526.678	4.5	0.489939	0.3264484	0.1518906	0.1038198	0.134594	0.1486326
526.778	4.5	0.48218	0.3387572	0.1512282	0.1113134	0.133356	0.1465631
526.878	4.5	0.48495	0.3463311	0.1528997	0.1135878	0.13061	0.1525604
526.978	4.5	0.488558	0.3436746	0.1544883	0.1113689	0.123908	0.1537603
527.078	4.5	0.528423	0.3398166	0.150214	0.1148147	0.129349	0.1528235
527.178	4.5	0.484317	0.3339787	0.1510106	0.1158195	0.132724	0.1526752
527.278	4.5	0.497176	0.3295986	0.151008	0.1177554	0.135225	0.1516257
527.378	4.5	0.489052	0.3283198	0.1508399	0.118878	0.135478	0.1546008
527.478	4.5	0.439998	0.3304779	0.1496776	0.1195028	0.136697	0.1549568
527.578	4.5	0.475817	0.3334926	0.1553908	0.1212357	0.135213	0.1569618
527.678	4.5	0.498294	0.3379288	0.1523928	0.1227401	0.134278	0.1593022
527.778	4.5	0.487724	0.3418489	0.1474804	0.1200227	0.139353	0.1629039
527.878	4.5	0.479354	0.3210805	0.1454253	0.1227919	0.139121	0.1587178
527.978	4.5	0.457865	0.3295424	0.146718	0.1218306	0.138862	0.1570542
528.078	4.5	0.453945	0.3292467	0.1469047	0.1204301	0.136739	0.1593477
528.178	4.5	0.444534	0.3248616	0.1480314	0.1176877	0.132515	0.1565721
528.278	4.5	0.454611	0.3389234	0.1468057	0.1184372	0.138019	0.156997
528.378	4.5	0.478892	0.3246955	0.146126	0.1226267	0.140345	0.1547337
528.478	4.5	0.467487	0.3146792	0.1431018	0.1250601	0.133747	0.1578739
528.578	4.5	0.473711	0.3333369	0.1419245	0.125336	0.133603	0.1601406
528.678	4.5	0.484169	0.3345366	0.1390072	0.1191128	0.131904	0.1610507
528.778	4.5	0.479334	0.3384459	0.1387546	0.1189899	0.137827	0.1612009
528.878	4.5	0.488302	0.3325493	0.1397299	0.1190403	0.13893	0.1654302
528.978	4.5	0.48455	0.33061	0.1414759	0.1122352	0.137154	0.1690522
529.078	4.5	0.481241	0.3311975	0.1424318	0.1078365	0.138162	0.167126
529.178	4.5	0.476121	0.3306184	0.1424669	0.09940146	0.13441	0.161009
529.278	4.5	0.490119	0.3376007	0.1398019	0.1047624	0.137595	0.1631172

529.378	4.5	0.470475	0.3384233	0.1377899	0.09730183	0.140207	0.1615355
529.478	4.5	0.484819	0.3417464	0.1444374	0.1013978	0.13755	0.1587759
529.578	4.5	0.492485	0.346949	0.1396388	0.09722738	0.137389	0.1577825
529.678	4.5	0.485244	0.3367249	0.1420699	0.1050152	0.139994	0.1607113
529.778	4.5	0.483717	0.3186259	0.1402585	0.1060616	0.140019	0.1573472
529.878	4.5	0.481064	0.3143484	0.14194	0.1018709	0.136503	0.1577058
529.978	4.5	0.489992	0.314625	0.1416088	0.1075696	0.139814	0.1560159
530.078	4.5	0.484812	0.313282	0.1415125	0.1058917	0.143508	0.1536293
530.178	4.5	0.487686	0.3250403	0.1399976	0.0985346	0.146738	0.1571423
530.278	4.5	0.494443	0.3167279	0.1314902	0.09982622	0.139534	0.1552638
530.378	4.5	0.496599	0.3243161	0.1421737	0.09865804	0.144395	0.1572402
530.478	4.5	0.487036	0.3429209	0.1342996	0.09513848	0.141703	0.157025
530.578	4.5	0.501083	0.344949	0.137484	0.09133281	0.14051	0.1547138
530.678	4.5	0.503136	0.3447875	0.1382063	0.09135884	0.145791	0.1425231
530.778	4.5	0.502778	0.3373747	0.1388815	0.08911495	0.147447	0.1395313
530.878	4.5	0.489664	0.3351792	0.1333139	0.08861545	0.149362	0.1519177
530.978	4.5	0.495918	0.339559	0.1454882	0.08758036	0.149531	0.1533896
531.078	4.5	0.496155	0.3373612	0.1469962	0.09226792	0.149476	0.1372314
531.178	4.5	0.504292	0.3411029	0.1422927	0.09214587	0.14796	0.1277902
531.278	4.5	0.502491	0.3440396	0.145426	0.08990354	0.148057	0.1367125
531.378	4.5	0.493677	0.3519036	0.1411653	0.09684224	0.14681	0.1371811
531.478	4.5	0.497054	0.3512067	0.1441905	0.09781462	0.144152	0.1399326
531.578	4.5	0.503356	0.3342852	0.1403701	0.09545317	0.142853	0.1376963
531.678	4.5	0.510795	0.3217415	0.1410702	0.09715239	0.145687	0.1311636
531.778	4.5	0.495184	0.3265892	0.1415047	0.09352478	0.146561	0.1358949
531.878	4.5	0.506391	0.3198836	0.1417156	0.09579096	0.149633	0.1378683
531.978	4.5	0.503484	0.3262721	0.1440828	0.09522574	0.149219	0.1379685
532.078	4.5	0.506621	0.3326485	0.1466287	0.09481934	0.149186	0.1385899
532.178	4.5	0.502989	0.3286163	0.1465662	0.09306414	0.151573	0.1358372
532.278	4.5	0.487852	0.3261703	0.145579	0.09182561	0.156285	0.1382378
532.378	4.5	0.510624	0.3475254	0.1445877	0.09648339	0.15567	0.1379732
532.478	4.5	0.491328	0.351197	0.1438726	0.09746377	0.154673	0.1400253
532.578	4.5	0.500475	0.3460799	0.1433301	0.1012603	0.150171	0.1411308
532.678	4.5	0.508513	0.3402263	0.1451703	0.1025135	0.1468	0.1414548
532.778	4.5	0.501799	0.3399437	0.1468123	0.08239061	0.146526	0.1410373
532.878	4.5	0.495469	0.3358901	0.1466437	0.08882283	0.149261	0.1439669
532.978	4.5	0.49988	0.3367189	0.1482162	0.09661558	0.149585	0.1442318
533.078	4.5	0.483721	0.3455361	0.1468865	0.08592007	0.149966	0.1452136
533.178	4.5	0.479763	0.3472465	0.1448008	0.08423615	0.152803	0.1456514
533.278	4.5	0.491984	0.3459927	0.1470024	0.0950986	0.149737	0.1440173
533.378	4.5	0.483906	0.3492459	0.1500487	0.1002812	0.145167	0.1412555
533.478	4.5	0.494957	0.3259625	0.1506608	0.09315065	0.149042	0.1444834
533.578	4.5	0.512321	0.3212355	0.1489108	0.08662202	0.143293	0.1457817
533.678	4.5	0.495776	0.3221063	0.1482844	0.09783053	0.143886	0.1448745
533.778	4.5	0.497443	0.3247929	0.1480518	0.1028693	0.148596	0.1432869
533.878	4.5	0.506882	0.325084	0.1465845	0.1038579	0.150819	0.1464579
533.978	4.5	0.510699	0.3203759	0.1474968	0.09974199	0.14944	0.1458481
534.078	4.5	0.515142	0.3226836	0.1489558	0.09061661	0.142782	0.1454091
534.178	4.5	0.49814	0.3411981	0.1471486	0.08463195	0.142562	0.1447377
534.278	4.5	0.506192	0.3462681	0.1455089	0.09151351	0.144662	0.1445824
534.378	4.5	0.507468	0.3528959	0.146519	0.09648383	0.145754	0.1463369
534.478	4.5	0.515479	0.3505177	0.1516552	0.1020178	0.147702	0.1475721
534.578	4.5	0.507743	0.3465376	0.1504006	0.09907587	0.146388	0.1478293
534.678	4.5	0.503494	0.3435591	0.1509582	0.09824631	0.140755	0.1460414
534.778	4.5	0.49025	0.3440773	0.1515964	0.09853158	0.143224	0.1461137
534.878	4.5	0.49653	0.3416772	0.1537366	0.09706695	0.143651	0.1481752
534.978	4.5	0.502002	0.3422551	0.1526715	0.09728047	0.145924	0.1502058
535.078	4.5	0.498391	0.3438601	0.1493825	0.09585957	0.138633	0.1517224
535.178	4.5	0.495695	0.3466697	0.1494958	0.09791718	0.144686	0.1515186
535.278	4.5	0.508043	0.3475313	0.150082	0.08839951	0.144093	0.1529903
535.378	4.5	0.500217	0.3345207	0.1455082	0.09451011	0.145545	0.1533779
535.478	4.5	0.502691	0.3410621	0.1511624	0.09728234	0.141396	0.1530698
535.578	4.5	0.500515	0.3374489	0.1478306	0.09664208	0.13997	0.154073
535.678	4.5	0.503531	0.3336909	0.145951	0.09980854	0.140536	0.1526376
535.778	4.5	0.493881	0.3375441	0.1457803	0.09966211	0.139681	0.1504286
535.878	4.5	0.483905	0.3404254	0.148673	0.09527519	0.136519	0.1514004
535.978	4.5	0.482152	0.3240879	0.147546	0.08846994	0.1347	0.1506702
536.078	4.5	0.499531	0.3426909	0.147242	0.09731877	0.14177	0.1532364
536.178	4.5	0.493151	0.3537256	0.146945	0.09904498	0.14148	0.1532438
536.278	4.5	0.507993	0.3557379	0.1443188	0.1001031	0.13653	0.154859
536.378	4.5	0.497239	0.3439484	0.1407509	0.09614087	0.135792	0.1559452
536.478	4.5	0.513565	0.3459803	0.1439002	0.1006863	0.137767	0.158476
536.578	4.5	0.512902	0.3438781	0.1472981	0.1078955	0.136927	0.160696
536.678	4.5	0.508124	0.3416222	0.1481401	0.1053599	0.136524	0.1613037
536.778	4.5	0.508825	0.3474233	0.1487216	0.09798253	0.141811	0.1588584
536.878	4.5	0.527066	0.349067	0.1467639	0.1033154	0.126743	0.1588952
536.978	4.5	0.51044	0.35095	0.1492468	0.1005358	0.128926	0.1613559
537.078	4.5	0.500057	0.3545535	0.1500521	0.09144104	0.133086	0.1614084
537.178	4.5	0.498079	0.339616	0.151367	0.09674816	0.132457	0.1591469
537.278	4.5	0.498041	0.3193745	0.147103	0.09857846	0.136375	0.167147
537.378	4.5	0.506812	0.3191289	0.1497835	0.1022713	0.134926	0.162707
537.478	4.5	0.494836	0.3253521	0.1527666	0.1006943	0.140472	0.1618069
537.578	4.5	0.525757	0.3191336	0.1516168	0.09584703	0.135876	0.1629313
537.678	4.5	0.492862	0.3253046	0.1491662	0.09457584	0.136052	0.1637918
537.778	4.5	0.49594	0.3171287	0.1514214	0.09667789	0.139704	0.1635183
537.878	4.5	0.499896	0.3342108	0.1476939	0.09461837	0.13664	0.1696571
537.978	4.5	0.482148	0.3477582	0.1552824	0.09566276	0.137667	0.1706975
538.078	4.5	0.48483	0.3569437	0.1554922	0.0955025	0.136018	0.1652409
538.178	4.5	0.496191	0.3593917	0.1520198	0.09319785	0.137836	0.1623912
538.278	4.5	0.48995	0.3523117	0.1513799	0.1010583	0.13655	0.1584177
538.378	4.5	0.477538	0.345741	0.1538021	0.1011634	0.135378	0.1586478
538.478	4.5	0.495233	0.3488857	0.1513589	0.0987213	0.135561	0.1612244
538.578	4.5	0.5119	0.3416814	0.1547883	0.09940092	0.138283	0.1620284
538.678	4.5	0.492018	0.3402398	0.154107	0.1047904	0.13826	0.1600255
538.778	4.5	0.505752	0.3380842	0.1561528	0.1037126	0.137721	0.1611266

538.878	4.5	0.490908	0.3442734	0.1549181	0.1091738	0.139783	0.155407
538.978	4.5	0.492206	0.3451541	0.1561333	0.1092651	0.137536	0.1565584
539.078	4.5	0.479067	0.3372074	0.1570615	0.1043425	0.138	0.1619734
539.178	4.5	0.511559	0.3381222	0.1556187	0.100899	0.134922	0.1631409
539.278	4.5	0.476285	0.3486417	0.1533522	0.1010619	0.136191	0.1628378
539.378	4.5	0.494402	0.3374156	0.1528313	0.1024511	0.135348	0.1614917
539.478	4.5	0.502142	0.3347608	0.1513702	0.1031637	0.13479	0.1639203
539.578	4.5	0.494938	0.3431441	0.1511098	0.1025315	0.136586	0.164569
539.74	5	0.485371	0.3463366	0.1536631	0.1031904	0.136356	0.1626936
539.84	5	0.480874	0.3529155	0.1518784	0.1031499	0.137578	0.1570636
539.94	5	0.480678	0.3462193	0.1518708	0.1069786	0.1385	0.1557087
540.04	5	0.488504	0.3446567	0.1518868	0.1143804	0.136648	0.1564734
540.14	5	0.516924	0.3425186	0.1501083	0.109885	0.134048	0.1592361
540.24	5	0.48413	0.3393733	0.1477221	0.11141	0.130962	0.1560094
540.34	5	0.486776	0.3420939	0.1465579	0.1159175	0.124759	0.1522552
540.44	5	0.474136	0.3487307	0.1467454	0.1207756	0.132771	0.1512637
540.54	5	0.480779	0.3504665	0.1450781	0.1187498	0.134169	0.1550932
540.64	5	0.490712	0.3521944	0.1443122	0.1188792	0.138403	0.1558495
540.74	5	0.490303	0.3390117	0.1459968	0.120654	0.135704	0.1559797
540.84	5	0.484102	0.3224737	0.1434488	0.1191868	0.140989	0.1544985
540.94	5	0.45835	0.3176481	0.1391677	0.1187764	0.13685	0.1602958
541.04	5	0.44847	0.321128	0.1439496	0.1181803	0.135716	0.1522096
541.14	5	0.437609	0.3147581	0.1437583	0.1198724	0.141639	0.1495851
541.24	5	0.452322	0.3221036	0.1412288	0.1190631	0.13709	0.1506332
541.34	5	0.466318	0.3107688	0.1420735	0.117526	0.136655	0.1484213
541.44	5	0.472561	0.3292786	0.1417232	0.1225753	0.136745	0.1536235
541.54	5	0.481139	0.3488029	0.1434089	0.1251764	0.13175	0.1568493
541.64	5	0.468647	0.3589384	0.1426617	0.121716	0.134171	0.1490896
541.74	5	0.494182	0.3591896	0.1421843	0.1240082	0.140851	0.1526569
541.84	5	0.479694	0.3501024	0.1420168	0.1230052	0.135725	0.1544096
541.94	5	0.478449	0.3467744	0.1403381	0.116831	0.135892	0.1586418
542.04	5	0.472781	0.3455895	0.1400881	0.1143882	0.134088	0.159329
542.14	5	0.479957	0.3400707	0.1417924	0.10461	0.134021	0.1528826
542.24	5	0.449778	0.3386494	0.145171	0.1098231	0.139213	0.1505881
542.34	5	0.477723	0.3381828	0.1447489	0.107644	0.135463	0.1514337
542.44	5	0.491047	0.341654	0.1463955	0.1083253	0.139248	0.1504926
542.54	5	0.494942	0.3455859	0.1440146	0.1038872	0.132818	0.1449554
542.64	5	0.486396	0.3295364	0.1458971	0.1110961	0.13494	0.1481805
542.74	5	0.482241	0.3472623	0.14573	0.1088176	0.140841	0.1530691
542.84	5	0.48013	0.3416393	0.146765	0.1116508	0.139459	0.1532319
542.94	5	0.488708	0.3321405	0.1453848	0.1121295	0.138174	0.1535007
543.04	5	0.493082	0.3423939	0.1469574	0.1025748	0.138906	0.1549509
543.14	5	0.495098	0.3378211	0.1502145	0.1028798	0.141761	0.1506358
543.24	5	0.493274	0.3234247	0.1500517	0.1023821	0.138668	0.148924
543.34	5	0.493954	0.338664	0.1492335	0.09937514	0.139328	0.1469995
543.44	5	0.495096	0.3460758	0.1484361	0.09851253	0.141216	0.1413821
543.54	5	0.495191	0.3479374	0.1501877	0.09520664	0.145267	0.1305974
543.64	5	0.490016	0.3415101	0.1478352	0.09657218	0.142879	0.1352605
543.74	5	0.499269	0.3415959	0.1478699	0.09501019	0.142648	0.1376671
543.84	5	0.494244	0.3406548	0.1452407	0.09541797	0.144982	0.137571
543.94	5	0.499642	0.3371755	0.1464163	0.09871689	0.142425	0.1439372
544.04	5	0.491131	0.3438059	0.1473316	0.09330869	0.1445	0.1460828
544.14	5	0.491516	0.3451755	0.1464077	0.09589816	0.146691	0.1464159
544.24	5	0.50313	0.3472953	0.1464418	0.09607133	0.145619	0.1481348
544.34	5	0.509483	0.346636	0.1456361	0.09522156	0.144574	0.1480196
544.44	5	0.508291	0.3217204	0.1444412	0.09464183	0.14665	0.1504323
544.54	5	0.502851	0.315189	0.1436358	0.09239136	0.143612	0.1498325
544.64	5	0.500767	0.3157121	0.142188	0.09631975	0.145046	0.1474682
544.74	5	0.50163	0.3178594	0.1424648	0.0938553	0.146393	0.1497609
544.84	5	0.48334	0.3231905	0.1433324	0.09377951	0.146382	0.1471671
544.94	5	0.507651	0.3121135	0.1407954	0.09017357	0.143239	0.1454973
545.04	5	0.49466	0.3137314	0.1393661	0.09729695	0.147251	0.1420016
545.14	5	0.496029	0.3429512	0.1417052	0.09721348	0.14811	0.1446258
545.24	5	0.500524	0.3535055	0.1429898	0.09809885	0.150721	0.1494616
545.34	5	0.508169	0.3532191	0.1445727	0.09350949	0.152999	0.1512117
545.44	5	0.498752	0.3495522	0.1406894	0.08445684	0.150665	0.1543155
545.54	5	0.493102	0.3442608	0.1443847	0.09446613	0.151961	0.1521038
545.64	5	0.479089	0.345643	0.1458065	0.09045389	0.157112	0.152227
545.74	5	0.493724	0.3382066	0.1476527	0.08863039	0.155586	0.1564041
545.84	5	0.479394	0.3368241	0.1450325	0.09638291	0.155516	0.1547677
545.94	5	0.484246	0.334196	0.1459169	0.1004648	0.151272	0.1589687
546.04	5	0.504741	0.3390893	0.1440477	0.08949406	0.147998	0.1576335
546.14	5	0.490706	0.3421355	0.1445412	0.09133892	0.146191	0.1567458
546.24	5	0.489486	0.3339798	0.1456587	0.1015424	0.148637	0.1543938
546.34	5	0.513537	0.3375902	0.142584	0.1032395	0.148922	0.1494944
546.44	5	0.509369	0.3559749	0.1479056	0.0970019	0.148604	0.1505085
546.54	5	0.492664	0.3340512	0.1470485	0.09308875	0.150007	0.1490776
546.64	5	0.501537	0.3375405	0.147416	0.08592712	0.148054	0.1516157
546.74	5	0.513343	0.3431742	0.1440394	0.09280604	0.145206	0.1484088
546.84	5	0.508181	0.3266755	0.1466018	0.1022786	0.148177	0.1476725
546.94	5	0.514352	0.3276809	0.1458799	0.1017782	0.141976	0.1540292
547.04	5	0.496099	0.3477642	0.1471421	0.1013758	0.14013	0.1541171
547.14	5	0.49895	0.353419	0.1469662	0.09772716	0.146479	0.1543222
547.24	5	0.506289	0.3440418	0.1441859	0.09750061	0.149305	0.1545546
547.34	5	0.497091	0.3411121	0.1399996	0.09924284	0.147296	0.1537012
547.44	5	0.492866	0.339786	0.1391565	0.09567128	0.142945	0.1557787
547.54	5	0.506722	0.3347599	0.1419464	0.09658135	0.142601	0.1573835
547.64	5	0.503798	0.3406917	0.1424706	0.08976238	0.143393	0.1577837
547.74	5	0.505488	0.3417693	0.1428486	0.09731923	0.142926	0.1612583
547.84	5	0.494911	0.3437313	0.1431182	0.09729378	0.144679	0.1648581
547.94	5	0.503645	0.3505155	0.1401341	0.09716044	0.145477	0.161718
548.04	5	0.488001	0.3336529	0.1409194	0.1016668	0.139929	0.160494
548.14	5	0.49017	0.3133278	0.1414933	0.09752036	0.139234	0.1613234
548.24	5	0.504178	0.3140216	0.1380123	0.08927459	0.138976	0.15829
548.34	5	0.494162	0.313746	0.140508	0.09645632	0.141574	0.157487

548.44	5	0.504702	0.3116616	0.1392612	0.1000423	0.13535	0.1559505
548.54	5	0.485802	0.317808	0.1394366	0.09864776	0.141503	0.1595279
548.64	5	0.521434	0.3054676	0.1405132	0.09882716	0.143045	0.1608223
548.74	5	0.505794	0.3329868	0.1360676	0.1041988	0.143914	0.1618115
548.84	5	0.5085	0.343136	0.1350452	0.1059355	0.141098	0.1634559
548.94	5	0.520824	0.3551663	0.1350335	0.1021381	0.142201	0.1636672
549.04	5	0.507578	0.347598	0.1330681	0.1040749	0.137875	0.1691409
549.14	5	0.503184	0.3467326	0.1361016	0.1014363	0.138884	0.1668394
549.24	5	0.496436	0.3470415	0.1348608	0.09511966	0.137103	0.1603605
549.34	5	0.496295	0.3408775	0.1368345	0.09956305	0.13531	0.1607139
549.44	5	0.503653	0.3373764	0.1436002	0.09965044	0.142052	0.1590272
549.54	5	0.498328	0.3354859	0.1379296	0.1008855	0.143939	0.1580575
549.64	5	0.512369	0.3363216	0.1409674	0.09792572	0.138595	0.1579801
549.74	5	0.50182	0.3404277	0.1427764	0.09631629	0.13441	0.1612597
549.84	5	0.484568	0.3379428	0.1435993	0.09451463	0.138828	0.1559038
549.94	5	0.488366	0.3313684	0.1420191	0.09405676	0.136869	0.1575731
550.04	5	0.477608	0.3500637	0.1425878	0.09577548	0.136361	0.1558912
550.14	5	0.488422	0.3337342	0.1427389	0.0965546	0.138523	0.1548892
550.24	5	0.487122	0.3272088	0.1465766	0.09399621	0.131521	0.1580805
550.34	5	0.470562	0.3332907	0.1461523	0.1005301	0.126489	0.1551744
550.44	5	0.498891	0.3334849	0.1456577	0.1010876	0.13176	0.1582717
550.54	5	0.496597	0.3228756	0.1460859	0.09783749	0.129707	0.1566534
550.64	5	0.491543	0.3356327	0.1432555	0.1019068	0.136274	0.1521661
550.74	5	0.503714	0.3502048	0.1434394	0.1025808	0.13098	0.1444321
550.84	5	0.501733	0.3441752	0.1457785	0.1074115	0.135954	0.1420877
550.94	5	0.491576	0.3415952	0.1451454	0.1107735	0.13216	0.1566595
551.04	5	0.484141	0.3414153	0.14711	0.1051249	0.133121	0.1582721
551.14	5	0.487026	0.3367101	0.1462844	0.103016	0.135174	0.1404159
551.24	5	0.51171	0.3363305	0.1457614	0.1008363	0.136896	0.1302666
551.34	5	0.466465	0.3419339	0.1473384	0.1031235	0.134101	0.1395364
551.44	5	0.489751	0.3439596	0.1486253	0.1033162	0.133311	0.1398695
551.54	5	0.488325	0.3433174	0.1510799	0.1041836	0.13753	0.1423136
551.64	5	0.487371	0.3334926	0.1487653	0.1041551	0.133614	0.1415552
551.74	5	0.493512	0.3158539	0.1450243	0.1040023	0.133279	0.1312098
551.84	5	0.484142	0.3102652	0.1485965	0.1092621	0.134324	0.137031
551.94	5	0.477397	0.3118407	0.1481979	0.1058543	0.133617	0.1371641
552.04	5	0.487832	0.3127289	0.1483774	0.1133869	0.133985	0.1391141
552.14	5	0.512161	0.3148705	0.1473948	0.1101997	0.134999	0.1420045
552.24	5	0.481346	0.30591	0.1458635	0.1136067	0.137577	0.1414772
552.34	5	0.480816	0.31875	0.149035	0.1164573	0.137487	0.1446632
552.44	5	0.481376	0.3384917	0.1523406	0.1208487	0.135831	0.1435018
552.54	5	0.49515	0.350572	0.1492327	0.1184529	0.132898	0.1451881
552.64	5	0.48957	0.3470181	0.1505008	0.1207777	0.131974	0.1456856
552.74	5	0.492983	0.340221	0.1538891	0.1230907	0.133025	0.1472113
552.84	5	0.489768	0.3446192	0.1547935	0.1221771	0.134539	0.1464624
552.94	5	0.460518	0.3427427	0.1481363	0.1205527	0.13712	0.1492196
553.04	5	0.446778	0.3327701	0.1497378	0.1206317	0.139334	0.1503419
553.14	5	0.436362	0.3330371	0.1467309	0.1221382	0.138094	0.1506164
553.24	5	0.455569	0.3346991	0.1520635	0.1195279	0.13965	0.1502549
553.34	5	0.46567	0.3390933	0.1485465	0.1190006	0.137911	0.1504951
553.44	5	0.465215	0.3406515	0.14794	0.1218087	0.136547	0.1462576
553.54	5	0.479071	0.3267844	0.1513083	0.124837	0.13424	0.1483059
553.64	5	0.470224	0.3395191	0.1532954	0.1229072	0.125166	0.1506062
553.74	5	0.490782	0.3332968	0.1525845	0.123721	0.130544	0.1509304
553.84	5	0.482616	0.3211674	0.1510461	0.1262404	0.132671	0.1485753
553.94	5	0.475311	0.3260812	0.1518589	0.11879	0.136892	0.1508608
554.04	5	0.473841	0.3309698	0.148747	0.1137043	0.136733	0.1495302
554.14	5	0.484002	0.318503	0.1533457	0.1122949	0.139018	0.1475106
554.24	5	0.462774	0.3293902	0.1524782	0.115412	0.13923	0.1456166
554.34	5	0.495467	0.3368855	0.1515979	0.1120218	0.13924	0.1444723
554.44	5	0.487661	0.3431719	0.1503259	0.1071118	0.141048	0.1448278
554.54	5	0.483808	0.3382798	0.1522921	0.1063429	0.141917	0.1471185
554.64	5	0.486383	0.3385265	0.1518963	0.112466	0.141606	0.14522
554.74	5	0.481322	0.3339985	0.1509283	0.1076376	0.139965	0.1466028
554.84	5	0.480914	0.3346756	0.1504725	0.1142806	0.132167	0.1477817
554.94	5	0.48277	0.3392127	0.1544109	0.1134296	0.133952	0.1500771
555.04	5	0.486927	0.342486	0.1528736	0.1068274	0.140985	0.147917
555.14	5	0.487628	0.3391048	0.1505065	0.109415	0.137898	0.1512313
555.24	5	0.492916	0.3393221	0.1512668	0.1070862	0.135876	0.1506212
555.34	5	0.494441	0.3178872	0.1486336	0.1050239	0.136591	0.1534793
555.44	5	0.495735	0.309484	0.1529661	0.1023255	0.137131	0.1520494
555.54	5	0.479294	0.3096989	0.1504199	0.1012747	0.140655	0.152382
555.64	5	0.490066	0.3119643	0.1483651	0.09873179	0.137505	0.1537096
555.74	5	0.492356	0.3187547	0.1505792	0.09904159	0.13918	0.1510143
555.84	5	0.496311	0.3126583	0.150385	0.1040988	0.135687	0.152042
555.94	5	0.496897	0.3038112	0.1550808	0.09589282	0.136219	0.1486433
556.04	5	0.490146	0.3338369	0.1580289	0.0956988	0.140216	0.1496786
556.14	5	0.482247	0.3464485	0.1567068	0.09946772	0.142699	0.1518101
556.24	5	0.496349	0.3454856	0.1561684	0.09794661	0.141214	0.1506466
556.34	5	0.500989	0.3454676	0.1577411	0.09704949	0.140949	0.1527995
556.44	5	0.509954	0.3336277	0.1560496	0.09278481	0.142569	0.1529211
556.54	5	0.491894	0.3430538	0.1540656	0.09364225	0.142687	0.1567292
556.64	5	0.492926	0.333606	0.1536867	0.09642244	0.14286	0.1577487
556.74	5	0.498113	0.3333133	0.1538376	0.09525141	0.145011	0.1576947
556.84	5	0.478687	0.3298927	0.155296	0.0904128	0.149454	0.1566682
556.94	5	0.506321	0.3365833	0.153116	0.09353543	0.145036	0.1581488
557.04	5	0.498047	0.3399086	0.152496	0.09904084	0.14286	0.1580628
557.14	5	0.49668	0.333241	0.1519364	0.100318	0.148954	0.154529
557.24	5	0.500387	0.3299304	0.1493262	0.1038025	0.144138	0.1574998
557.34	5	0.506085	0.3458622	0.1479082	0.09374396	0.145401	0.1595233
557.44	5	0.493142	0.3191757	0.1474709	0.09287164	0.148096	0.1587049
557.54	5	0.48752	0.3151996	0.1472747	0.1008864	0.1488	0.1582557
557.64	5	0.474052	0.3235804	0.1456214	0.09207349	0.149116	0.1629536
557.74	5	0.490148	0.321713	0.1460755	0.09533378	0.151571	0.1616665
557.84	5	0.47591	0.3139046	0.1439614	0.09991709	0.145825	0.1610092

557.94	5	0.481398	0.3347192	0.1419615	0.09925292	0.148957	0.1637518
558.04	5	0.498797	0.3448005	0.1369318	0.08951768	0.147085	0.1657225
558.14	5	0.489134	0.3421579	0.1430141	0.09573312	0.144449	0.1630178
558.24	5	0.494707	0.3422431	0.1444676	0.1019151	0.143427	0.1599714
558.34	5	0.512036	0.3401	0.1410464	0.1044649	0.144407	0.1537923
558.44	5	0.504145	0.3391041	0.1404952	0.09781368	0.147956	0.1581439
558.54	5	0.49323	0.3423203	0.1420368	0.09149288	0.1442	0.1593196
558.64	5	0.506632	0.3545054	0.1416021	0.08584179	0.146543	0.1555793
558.74	5	0.51447	0.3505314	0.1440119	0.09102907	0.147631	0.1558916
558.84	5	0.498041	0.3510043	0.1432101	0.09900737	0.148009	0.1558286
558.94	5	0.51182	0.3397439	0.1450637	0.09861682	0.154399	0.1511516
559.04	5	0.49354	0.327098	0.142184	0.1003804	0.149638	0.1517682
559.14	5	0.499144	0.3296444	0.140763	0.09820928	0.156011	0.157417
559.24	5	0.500859	0.3360406	0.1399196	0.0963059	0.150268	0.154223
559.34	5	0.493871	0.3234351	0.141025	0.09858222	0.145835	0.1553024
559.44	5	0.493094	0.3299681	0.1447357	0.09236313	0.144794	0.1573401
559.54	5	0.504468	0.3403809	0.1468447	0.09537024	0.141793	0.1578628
559.64	5	0.504108	0.3632235	0.1453227	0.08933318	0.146352	0.1641298
559.74	5	0.500086	0.3711762	0.1446714	0.09532503	0.147056	0.1578438
559.84	5	0.492803	0.373075	0.1457112	0.09837575	0.145625	0.1552236
559.94	5	0.49505	0.3723472	0.1463851	0.09987875	0.14898	0.1525587
560.04	5	0.479735	0.368985	0.1457777	0.1005685	0.144188	0.1562291
560.14	5	0.478707	0.3694047	0.1470406	0.09587637	0.145815	0.1576056
560.24	5	0.492244	0.3682972	0.1513177	0.09002262	0.143275	0.1537306
560.34	5	0.488063	0.3701955	0.1521051	0.0972115	0.14002	0.1532221
560.44	5	0.502065	0.375456	0.1533444	0.1019582	0.143945	0.1522695
560.54	5	0.483097	0.3772359	0.1511844	0.1011938	0.145811	0.1558799
560.64	5	0.523194	0.3812452	0.1534854	0.1000566	0.146883	0.1576036
560.74	5	0.502347	0.363844	0.1495005	0.1034628	0.143212	0.1545316
560.84	5	0.501674	0.3689932	0.1492345	0.1064677	0.140592	0.157118
560.94	5	0.519678	0.3701155	0.1471559	0.1029009	0.141206	0.1521647
561.04	5	0.500922	0.3662977	0.1470168	0.1052507	0.141399	0.1496342
561.14	5	0.494275	0.3633276	0.1483275	0.1046011	0.14115	0.1524809
561.24	5	0.492928	0.3658006	0.1456329	0.09814799	0.143307	0.154005
561.34	5	0.493005	0.363601	0.1420086	0.100587	0.141185	0.1478616
561.44	5	0.492352	0.3643401	0.1439544	0.1004415	0.137346	0.1525469
561.54	5	0.504146	0.3723753	0.14532	0.1012274	0.137165	0.1535511
561.64	5	0.500708	0.3667425	0.1433678	0.09765216	0.138412	0.1597514
561.74	5	0.500916	0.3643628	0.1391854	0.09859367	0.134285	0.1531951
561.84	5	0.489563	0.3608918	0.1390498	0.09602395	0.133521	0.1485562
561.94	5	0.476205	0.3569233	0.1401128	0.09379878	0.135294	0.1498519
562.04	5	0.481156	0.3519027	0.1391686	0.09888586	0.141731	0.1478973
562.14	5	0.488194	0.3485307	0.1396981	0.09799337	0.137714	0.1452039
562.24	5	0.480218	0.3535466	0.1383473	0.09596781	0.137186	0.147145
562.34	5	0.471127	0.3615768	0.1383782	0.1002951	0.136773	0.150741
562.44	5	0.503299	0.3633685	0.1405347	0.1002354	0.136977	0.1495525
562.54	5	0.483873	0.3605434	0.1414365	0.09703712	0.136326	0.1506718
562.64	5	0.494147	0.3405235	0.1410844	0.1025073	0.133342	0.1486494
562.74	5	0.491571	0.3488108	0.1418121	0.1021867	0.134714	0.1469294
562.84	5	0.496804	0.3527404	0.1446409	0.1080193	0.138588	0.14383
562.94	5	0.483829	0.3330299	0.1421099	0.1083882	0.138265	0.1331976
563.04	5	0.483628	0.3282492	0.1423065	0.1018489	0.134181	0.1323807
563.14	5	0.4711	0.3266423	0.1397354	0.1047847	0.136237	0.1359694
563.24	5	0.510214	0.3468236	0.1411899	0.1050267	0.135553	0.1380045
563.34	5	0.465384	0.3621879	0.14336	0.1063141	0.135384	0.1439416
563.44	5	0.481185	0.3629905	0.1425716	0.1089277	0.135568	0.1485757
563.54	5	0.491503	0.3602475	0.1425511	0.1084673	0.137048	0.1506602
563.64	5	0.495562	0.3561724	0.1464826	0.1076426	0.122911	0.1502634
563.74	5	0.483576	0.3498642	0.1464937	0.1090472	0.1338	0.1513481
563.84	5	0.480438	0.3497664	0.1447464	0.1106031	0.132237	0.1538748
563.94	5	0.477265	0.3493161	0.1445677	0.1078523	0.134871	0.1514699
564.04	5	0.480713	0.351896	0.1438907	0.1125566	0.133058	0.1514569
564.14	5	0.516098	0.3522798	0.1443975	0.1102632	0.13521	0.1486541
564.24	5	0.477405	0.3586585	0.1445224	0.1125985	0.134979	0.144501
564.34	5	0.485315	0.3554662	0.1430987	0.1143601	0.132673	0.1443848
564.44	5	0.481522	0.3596602	0.1402324	0.1186819	0.132682	0.1501482
564.54	5	0.483561	0.361146	0.1400809	0.1166496	0.137677	0.1523054
564.64	5	0.487221	0.3535061	0.1411424	0.1194336	0.13559	0.1556865
564.74	5	0.489843	0.3540353	0.1416764	0.1205959	0.134992	0.1536916
564.84	5	0.484879	0.3570387	0.1398581	0.1196441	0.135341	0.1564116
564.94	5	0.456736	0.3547966	0.140734	0.1209749	0.135841	0.1563772
565.04	5	0.445179	0.3514954	0.1387827	0.1210077	0.134204	0.1591477
565.14	5	0.430542	0.3574675	0.1395403	0.1199542	0.130618	0.1577075
565.24	5	0.451011	0.3542993	0.1417039	0.116576	0.133822	0.1588894
565.34	5	0.46138	0.3571058	0.139174	0.1168723	0.136617	0.1539154
565.44	5	0.458638	0.3553594	0.1398378	0.1177467	0.134208	0.1512827
565.54	5	0.473244	0.3516299	0.1398894	0.121558	0.135752	0.1510971
565.64	5	0.460315	0.3478462	0.1403149	0.1194875	0.138712	0.1530958
565.74	5	0.484477	0.3443715	0.1404451	0.1227056	0.137778	0.1478069
565.84	5	0.47124	0.3481218	0.1349657	0.1233624	0.137992	0.150841
565.94	5	0.476476	0.3496917	0.133957	0.1198325	0.135125	0.1537814
566.04	5	0.467739	0.3496787	0.1367955	0.109089	0.136376	0.1528308
566.14	5	0.477306	0.3604811	0.1311247	0.1096388	0.1346	0.1546348
566.24	5	0.461373	0.3400234	0.1361656	0.1124807	0.134603	0.1548886
566.34	5	0.480958	0.323407	0.1353975	0.1102848	0.138101	0.1568577
566.44	5	0.488469	0.3384438	0.1329445	0.1089143	0.139191	0.1590415
566.54	5	0.478943	0.3348583	0.1439345	0.1064179	0.138642	0.1611423
566.64	5	0.489773	0.3172931	0.1368626	0.1105949	0.139375	0.1654933
566.74	5	0.477103	0.3171079	0.1397356	0.1091957	0.140245	0.1621239
566.84	5	0.477844	0.3297475	0.1418294	0.1140241	0.138402	0.1594855
566.94	5	0.481463	0.3547434	0.1445185	0.11537	0.135031	0.1598846
567.04	5	0.485743	0.3588793	0.1421103	0.1098641	0.127013	0.1582714
567.14	5	0.491385	0.3574807	0.1428322	0.1089445	0.132997	0.1521197
567.24	5	0.496851	0.3526659	0.1432973	0.1082819	0.134493	0.1554469
567.34	5	0.494891	0.3427227	0.1450646	0.1082817	0.138632	0.1584762

567.44	5	0.491786	0.3396048	0.144559	0.1065608	0.13585	0.157666
567.54	5	0.481068	0.3391978	0.1446459	0.1042217	0.139614	0.160302
567.64	5	0.494662	0.3414868	0.1445461	0.1042986	0.13678	0.1630816
567.74	5	0.494127	0.3406506	0.1419117	0.1018895	0.135593	0.1653742
567.84	5	0.491223	0.3437843	0.1428259	0.106029	0.140618	0.1600284
567.94	5	0.494305	0.3463694	0.1443126	0.09791863	0.13939	0.1597356
568.04	5	0.487625	0.3510896	0.1447134	0.09949208	0.139784	0.1567207
568.14	5	0.48432	0.3602553	0.1471987	0.09890264	0.136121	0.1557606
568.24	5	0.496044	0.3555729	0.149168	0.09781849	0.131939	0.1574541
568.34	5	0.501858	0.3450929	0.1471586	0.09924635	0.136191	0.1563842
568.44	5	0.506953	0.3492277	0.1459601	0.09702809	0.141016	0.157344
568.54	5	0.487508	0.3482175	0.1463127	0.09966111	0.133545	0.1567463
568.64	5	0.490302	0.3482339	0.149931	0.1019128	0.134891	0.1523299
568.74	5	0.490905	0.3478077	0.1466468	0.100256	0.136684	0.1539288
568.84	5	0.478153	0.3522555	0.1458088	0.0944164	0.140391	0.1507557
568.94	5	0.503277	0.3542581	0.1437762	0.09848773	0.143343	0.1537393
569.04	5	0.491184	0.3538041	0.1422216	0.1022782	0.140109	0.1495425
569.14	5	0.490452	0.3523539	0.1454689	0.1035959	0.143199	0.141318
569.24	5	0.493303	0.3474403	0.1454547	0.1034311	0.138203	0.1412499
569.34	5	0.49176	0.3424912	0.1453598	0.08936992	0.140096	0.154666
569.44	5	0.485588	0.3407949	0.1457443	0.09470543	0.141573	0.1495715
569.54	5	0.476231	0.3441234	0.1502207	0.1016741	0.140738	0.1270234
569.64	5	0.46927	0.3477041	0.1484825	0.09047835	0.141508	0.1377846
569.74	5	0.481711	0.3578551	0.146056	0.0976722	0.143722	0.1410512
569.84	5	0.471136	0.3474676	0.1495555	0.1016967	0.145018	0.1433213
569.94	5	0.479713	0.3260641	0.1508231	0.1003073	0.143469	0.1366453
570.04	5	0.497475	0.3276547	0.147259	0.08949191	0.144145	0.1309849
570.14	5	0.48831	0.3389447	0.1469497	0.09773478	0.146251	0.1334143
570.24	5	0.490001	0.3212746	0.1514654	0.1006846	0.146999	0.1340778
570.34	5	0.502751	0.3171781	0.1492713	0.1034734	0.141196	0.1361491
570.44	5	0.505001	0.3223357	0.152016	0.09896405	0.144274	0.1373982
570.54	5	0.493569	0.3473936	0.145779	0.08852375	0.142211	0.1378812
570.64	5	0.506888	0.3542253	0.1461798	0.08716924	0.141626	0.1381782
570.74	5	0.5152	0.3589278	0.1506852	0.09647328	0.146812	0.1379918
570.84	5	0.494806	0.354389	0.1498921	0.1039366	0.145847	0.1421904
570.94	5	0.51774	0.3458807	0.1468732	0.1006304	0.142422	0.1419028
571.04	5	0.480476	0.3404072	0.1503747	0.1006445	0.145611	0.1441881
571.14	5	0.495752	0.3391668	0.1474105	0.09789155	0.144189	0.1430452
571.24	5	0.488086	0.3401836	0.1508111	0.0992879	0.144369	0.1436994
571.34	5	0.492876	0.3450486	0.1528768	0.09859881	0.144793	0.1443917
571.44	5	0.490337	0.3447389	0.1517273	0.09307536	0.147526	0.1406601
571.54	5	0.487625	0.348528	0.1496316	0.09260803	0.148902	0.1389869
571.64	5	0.494488	0.3502112	0.151157	0.09147426	0.150043	0.142825
571.74	5	0.501343	0.3546809	0.1522156	0.09732081	0.14929	0.1420562
571.84	5	0.483984	0.3559008	0.1521862	0.1000629	0.147917	0.1418763
571.94	5	0.489211	0.3462932	0.1492236	0.1002324	0.157486	0.1428398
572.04	5	0.47182	0.3442797	0.150421	0.1004176	0.151642	0.1425505
572.14	5	0.478542	0.3470111	0.1525993	0.09673691	0.154864	0.1413227
572.24	5	0.489055	0.3428328	0.149412	0.09072608	0.151216	0.1415807
572.34	5	0.483002	0.3415732	0.148217	0.1007497	0.14665	0.1430305
572.44	5	0.497424	0.3497052	0.1455976	0.1010375	0.143942	0.1438836
572.54	5	0.481243	0.3530363	0.1511911	0.1001664	0.145462	0.1436873
572.64	5	0.515105	0.3553645	0.1526036	0.1006311	0.145742	0.1448846
572.74	5	0.508812	0.3532783	0.1501485	0.1035351	0.146538	0.1451238
572.84	5	0.500304	0.3511761	0.1514692	0.107788	0.149373	0.1459937
572.94	5	0.51661	0.3452594	0.1493114	0.1022992	0.14507	0.1484807
573.04	5	0.501006	0.3405113	0.1515636	0.1067635	0.146759	0.1482327
573.14	5	0.493907	0.3446795	0.1537029	0.104959	0.142523	0.1512401
573.24	5	0.484869	0.3509815	0.156423	0.09599727	0.139421	0.1488035
573.34	5	0.490713	0.3466089	0.1552752	0.09969422	0.143089	0.1507344
573.44	5	0.486838	0.3525985	0.1559889	0.09967458	0.145854	0.1502772
573.54	5	0.502611	0.330159	0.1542644	0.09908639	0.141412	0.147376
573.64	5	0.494704	0.321334	0.1540853	0.0979558	0.138814	0.1492024
573.74	5	0.493285	0.3337826	0.1535342	0.09752336	0.14282	0.1505958
573.84	5	0.485574	0.3305072	0.1530855	0.09827612	0.146244	0.1505658
573.94	5	0.470457	0.3102044	0.1533556	0.09700118	0.144386	0.1492313
574.04	5	0.477133	0.3124917	0.1528073	0.1014189	0.144028	0.151601
574.14	5	0.480154	0.3295431	0.1514125	0.09960959	0.139767	0.1539791
574.24	5	0.47367	0.3518334	0.1520865	0.09738837	0.140093	0.1547455
574.34	5	0.472784	0.3564847	0.150184	0.1029269	0.14115	0.155082
574.44	5	0.497106	0.3521796	0.1493998	0.1031123	0.13842	0.1556746
574.54	5	0.477044	0.3464188	0.1459722	0.09897715	0.135325	0.1580611
574.64	5	0.495166	0.3413379	0.1451684	0.09993149	0.138642	0.1576239
574.74	5	0.48523	0.3396119	0.1452736	0.1022078	0.142151	0.1556298
574.84	5	0.498945	0.3374189	0.1464433	0.1066957	0.139906	0.1603039
574.94	5	0.474222	0.3408483	0.1426771	0.1089138	0.139582	0.1559957
575.04	5	0.485419	0.3389663	0.1421534	0.102555	0.137541	0.1535313
575.14	5	0.461714	0.3458014	0.1374207	0.1057876	0.139203	0.1548113
575.24	5	0.507729	0.3452115	0.1401141	0.1063693	0.136961	0.1562609
575.34	5	0.463874	0.3457194	0.1446695	0.1052582	0.135607	0.1602175
575.44	5	0.475342	0.3550701	0.1426274	0.10551	0.140641	0.1622856
575.54	5	0.494095	0.3507569	0.1424914	0.1088834	0.138389	0.1581721
575.64	5	0.491001	0.3403056	0.144564	0.1079732	0.134594	0.1537383
575.74	5	0.47776	0.3420954	0.1422851	0.109648	0.136113	0.1532904
575.84	5	0.478357	0.3429741	0.144896	0.1118075	0.135182	0.1565488
575.94	5	0.476983	0.3387223	0.1464821	0.1096513	0.135287	0.156237
576.04	5	0.483974	0.3443787	0.1466102	0.1148982	0.137246	0.1535504
576.14	5	0.515603	0.3476895	0.1455346	0.1135172	0.130229	0.1533749
576.24	5	0.481946	0.3533037	0.1426983	0.1155776	0.128446	0.1506959
576.34	5	0.470383	0.3533769	0.1422272	0.116839	0.128479	0.1524318
576.44	5	0.478857	0.3501911	0.1426175	0.1193588	0.132798	0.1523533
576.54	5	0.474651	0.3428867	0.1471628	0.1174505	0.13424	0.1535097
576.64	5	0.492433	0.3358912	0.146835	0.121135	0.1373	0.1570896
576.74	5	0.482279	0.3386795	0.1466554	0.122937	0.131918	0.1571237
576.84	5	0.479143	0.3423774	0.1465989	0.1222984	0.131209	0.1576618

576.94	5	0.447216	0.3391909	0.1462857	0.122472	0.133951	0.1491194
577.04	5	0.440314	0.355165	0.1462676	0.1220299	0.135059	0.1480563
577.14	5	0.424996	0.3352352	0.1466942	0.1240359	0.134876	0.1512902
577.24	5	0.454113	0.3163089	0.1456828	0.1218616	0.134248	0.1546321
577.34	5	0.455186	0.320883	0.1497048	0.1201208	0.135473	0.150888
577.44	5	0.462446	0.3323148	0.1537654	0.1217419	0.133223	0.1506743
577.54	5	0.475503	0.3083342	0.1514763	0.1250435	0.129921	0.1535266
577.64	5	0.459977	0.3065783	0.1505481	0.1232004	0.13168	0.1545592
577.74	5	0.484177	0.3152678	0.1520085	0.1209392	0.131199	0.153299
577.84	5	0.47004	0.3420651	0.1531796	0.1225691	0.13313	0.152514
577.94	5	0.472963	0.3508659	0.149152	0.117009	0.136764	0.1533937
578.04	5	0.461443	0.3538816	0.1507619	0.1115081	0.135189	0.1464311
578.14	5	0.468896	0.3462834	0.1467685	0.1091677	0.135277	0.1497866
578.24	5	0.450292	0.3415089	0.1501576	0.1127181	0.131968	0.1485561
578.34	5	0.48185	0.3364404	0.1479065	0.1140228	0.131302	0.1539131
578.44	5	0.47756	0.3334354	0.1448597	0.1137791	0.130272	0.1502343
578.54	5	0.478796	0.3368587	0.1420744	0.1133157	0.132007	0.1511392
578.64	5	0.479477	0.3374741	0.1467665	0.1134447	0.133019	0.1547378
578.74	5	0.4685	0.3412956	0.1465938	0.1114162	0.13479	0.1562906
578.84	5	0.474799	0.3456556	0.144518	0.1201192	0.137173	0.1579255
578.94	5	0.476959	0.3450437	0.1409131	0.1197936	0.136686	0.1519006
579.04	5	0.482592	0.3479806	0.1399144	0.1107573	0.133159	0.1516473
579.14	5	0.480621	0.3519937	0.14073	0.1079808	0.128882	0.1538309
579.24	5	0.49885	0.3385446	0.1426425	0.1057414	0.135639	0.1446003
579.34	5	0.506546	0.3398287	0.143089	0.1071314	0.130142	0.1485729
579.44	5	0.497467	0.3417038	0.1408426	0.1063061	0.134068	0.1506312
579.54	5	0.481766	0.3353448	0.1396491	0.1048436	0.136009	0.1490134
579.64	5	0.487668	0.3376204	0.1412691	0.1036821	0.137382	0.1484463
579.74	5	0.493694	0.3426817	0.1408758	0.1034066	0.133891	0.1493931
579.84	5	0.486364	0.3461785	0.1462168	0.106999	0.132722	0.1454423
579.94	5	0.490607	0.3469098	0.1431725	0.1012477	0.138172	0.1450588
580.04	5	0.48454	0.3472383	0.1418308	0.09917645	0.136245	0.1389344
580.14	5	0.47911	0.3423213	0.1400023	0.1018426	0.138022	0.12387
580.24	5	0.494386	0.3375391	0.1406832	0.1039735	0.131388	0.1340649
580.34	5	0.497268	0.3312056	0.1376686	0.1055462	0.131432	0.1370429
580.44	5	0.502873	0.3349853	0.1402671	0.1014504	0.136119	0.1401693
580.54	5	0.482626	0.3405561	0.1416572	0.1026081	0.134106	0.1486072
580.64	5	0.486569	0.3478957	0.1413518	0.1041646	0.136433	0.1487284
580.74	5	0.485595	0.3366636	0.1458481	0.1027504	0.136756	0.1518426
580.84	5	0.47346	0.3203341	0.1469297	0.09836137	0.138744	0.1520002
580.94	5	0.499648	0.3131224	0.1465557	0.0997527	0.138621	0.1544873
581.04	5	0.498106	0.3313479	0.1453833	0.1033501	0.136623	0.1501652
581.14	5	0.49817	0.3147995	0.1447639	0.1037852	0.138248	0.1531565
581.24	5	0.49879	0.310359	0.1454306	0.1049287	0.135323	0.150951
581.34	5	0.496879	0.3103155	0.1454074	0.1000204	0.140422	0.1492261
581.44	5	0.483866	0.333798	0.1455308	0.09351469	0.141223	0.1451197
581.54	5	0.486584	0.3445199	0.1426676	0.1026402	0.140776	0.1492597
581.64	5	0.466322	0.3509236	0.1385694	0.09770007	0.140397	0.1528518
581.74	5	0.476632	0.3501964	0.1378049	0.09557654	0.142909	0.1550745
581.84	5	0.466338	0.3410245	0.1420301	0.1027666	0.140095	0.1518698
581.94	5	0.481663	0.3360592	0.1414642	0.1052161	0.142337	0.152901
582.04	5	0.493376	0.3337052	0.1435502	0.09315553	0.144701	0.1567548
582.14	5	0.476401	0.3349453	0.1470243	0.09608501	0.143762	0.1594897
582.24	5	0.485049	0.3388516	0.1416038	0.1057277	0.138889	0.1588528
582.34	5	0.502817	0.3395246	0.1420178	0.1059754	0.143008	0.1560386
582.44	5	0.503968	0.3443488	0.1425994	0.1023761	0.141196	0.1556886
582.54	5	0.486369	0.3460318	0.1412212	0.09669533	0.143906	0.1492764
582.64	5	0.497613	0.3439565	0.14355	0.08724415	0.145136	0.1500279
582.74	5	0.507226	0.3476141	0.1443144	0.09280756	0.147275	0.1496902
582.84	5	0.492678	0.3388057	0.142908	0.1051425	0.1449	0.1493545
582.94	5	0.507664	0.3323957	0.1422873	0.1060449	0.141994	0.1435542
583.04	5	0.478096	0.3441103	0.1325128	0.1061744	0.145475	0.149961
583.14	5	0.494419	0.3343772	0.1383845	0.1028512	0.144431	0.1482434
583.24	5	0.490771	0.3318504	0.1351482	0.1040303	0.144722	0.149047
583.34	5	0.485818	0.3417857	0.1329538	0.1041247	0.144086	0.1514545
583.44	5	0.486573	0.3423441	0.1355684	0.09646806	0.146049	0.1531209
583.54	5	0.492373	0.3460013	0.135114	0.09913544	0.148711	0.153647
583.64	5	0.487599	0.3430396	0.1352714	0.09381702	0.149506	0.1552151
583.74	5	0.499542	0.3409213	0.1425154	0.100028	0.149395	0.1579889
583.84	5	0.478249	0.336181	0.1372286	0.100897	0.147253	0.159612
583.94	5	0.483826	0.326324	0.1414479	0.1012021	0.152145	0.1583744
584.04	5	0.47061	0.329963	0.1416414	0.1050881	0.151084	0.1552356
584.14	5	0.474023	0.337118	0.1433723	0.09905604	0.151518	0.1541206
584.24	5	0.487035	0.3327352	0.1421007	0.09098778	0.145898	0.15281
584.34	5	0.484005	0.3465219	0.1422668	0.09965464	0.144696	0.1492228
584.44	5	0.498106	0.3192566	0.1416346	0.1031995	0.142364	0.1537708
584.54	5	0.47872	0.314255	0.1444919	0.09844595	0.145995	0.1562873
584.64	5	0.517765	0.326966	0.1445306	0.1002958	0.145465	0.154045
584.74	5	0.496847	0.3216768	0.1450095	0.1032647	0.145796	0.1568393
584.84	5	0.500712	0.3083574	0.1407209	0.1036777	0.147949	0.162319
584.94	5	0.509918	0.3062691	0.1416032	0.1001696	0.143448	0.1618467
585.04	5	0.492883	0.3188384	0.1393159	0.1059749	0.146089	0.1583399
585.14	5	0.484132	0.3379514	0.1415042	0.1041524	0.140264	0.1562369
585.24	5	0.487774	0.3447326	0.1447604	0.09949756	0.140994	0.1546206
585.34	5	0.485534	0.3457741	0.1456541	0.1034471	0.145387	0.1526625
585.44	5	0.489903	0.3377745	0.1455693	0.1031561	0.145917	0.156138
585.54	5	0.498969	0.3359386	0.1435945	0.1020436	0.143235	0.1537973
585.64	5	0.495603	0.3330588	0.1463708	0.1003744	0.139666	0.154906
585.74	5	0.488328	0.3324562	0.1478061	0.1002958	0.141149	0.1550478
585.84	5	0.480496	0.3358183	0.1454344	0.1000846	0.139326	0.1546543
585.94	5	0.467159	0.3352391	0.1440644	0.09921325	0.141473	0.1536161
586.04	5	0.468004	0.3423396	0.1427875	0.1024221	0.142723	0.1524841
586.14	5	0.47282	0.3432945	0.1445435	0.1001613	0.138447	0.1509845
586.24	5	0.467957	0.3412433	0.1442879	0.0989125	0.138317	0.1496703
586.34	5	0.462538	0.3448966	0.1465427	0.1042656	0.140018	0.1407116

586.44	5	0.491997	0.3434519	0.1445467	0.1062865	0.13837	0.1463477
586.54	5	0.469243	0.3312856	0.1451519	0.1044503	0.137496	0.1528884
586.64	5	0.486609	0.3371733	0.1477088	0.1041597	0.13852	0.1449126
586.74	5	0.488606	0.3349895	0.1498141	0.1046387	0.142332	0.1264624
586.84	5	0.492284	0.3273713	0.1468206	0.1103435	0.137197	0.1407215
586.94	5	0.467203	0.3337544	0.1447556	0.1110284	0.13644	0.1455728
587.04	5	0.475274	0.3399709	0.1473943	0.104638	0.134487	0.1478639
587.14	5	0.467578	0.3428337	0.1468999	0.1052463	0.135316	0.1364534
587.24	5	0.509581	0.3432747	0.1425393	0.106966	0.133405	0.1357314
587.34	5	0.456952	0.3415333	0.1496108	0.1068506	0.136094	0.1359423
587.44	5	0.473313	0.3382768	0.1473041	0.1069889	0.140466	0.1385044
587.54	5	0.486096	0.3317201	0.1481409	0.1069215	0.13648	0.1429807
587.64	5	0.481842	0.3268197	0.1466898	0.1070743	0.132622	0.1453989
587.74	5	0.486	0.3303365	0.1445144	0.1087625	0.134351	0.1436964
587.84	5	0.478495	0.3229112	0.1479648	0.1113501	0.13311	0.1443516
587.94	5	0.471261	0.3420148	0.1486382	0.1074884	0.131977	0.1460686
588.04	5	0.473072	0.324036	0.1479644	0.1147267	0.1331	0.1487374
588.14	5	0.505214	0.3133776	0.1483319	0.1135619	0.121991	0.1471465
588.24	5	0.476518	0.3094398	0.1477325	0.114871	0.131361	0.14996
588.34	5	0.463999	0.327071	0.1472007	0.1151448	0.130278	0.1498485
588.44	5	0.467905	0.3076451	0.1500713	0.1171618	0.134962	0.1503992
588.54	5	0.480883	0.3005093	0.1498647	0.1160759	0.12987	0.1478419
588.64	5	0.469516	0.3075647	0.1497367	0.1183802	0.135589	0.1461977
588.74	5	0.488475	0.329804	0.1482895	0.1193632	0.130406	0.1453018
588.84	5	0.472	0.3382034	0.1514808	0.1188867	0.130647	0.1467953
588.94	5	0.454279	0.3511161	0.1514913	0.1211193	0.135045	0.1453352
589.04	5	0.44335	0.3459562	0.1497558	0.1206603	0.133845	0.1461692
589.14	5	0.426073	0.3367887	0.1467411	0.1195447	0.13263	0.1447955
589.24	5	0.435145	0.3372833	0.1518066	0.1190555	0.135719	0.1445996
589.34	5	0.457432	0.3326282	0.1512882	0.1197708	0.133886	0.1436331
589.44	5	0.459435	0.3340673	0.148919	0.1233272	0.133011	0.1449443
589.54	5	0.464561	0.3381036	0.1483602	0.1247526	0.132293	0.1460427
589.64	5	0.45625	0.3382003	0.1452855	0.1232459	0.132053	0.1472687
589.74	5	0.477786	0.3447341	0.1529281	0.1220088	0.131599	0.1460188
589.84	5	0.465245	0.3462141	0.1503152	0.1223232	0.132928	0.1458155
589.94	5	0.46947	0.3402292	0.147995	0.1188835	0.135623	0.1476922
590.04	5	0.465222	0.3460925	0.1491276	0.1150235	0.131877	0.1475343
590.14	5	0.473662	0.3302266	0.1477432	0.1070233	0.132972	0.1507193
590.24	5	0.459755	0.3289129	0.1503854	0.1119466	0.130236	0.1508535
590.34	5	0.473985	0.3336905	0.151317	0.1101495	0.130363	0.1531819
590.44	5	0.477987	0.3288341	0.1519877	0.1108287	0.129793	0.1504319
590.54	5	0.472071	0.324479	0.152623	0.1075258	0.128984	0.1527557
590.64	5	0.468696	0.3373308	0.1541434	0.1126166	0.130741	0.1511898
590.74	5	0.459646	0.3395757	0.1526087	0.1104123	0.132025	0.1514876
590.84	5	0.462091	0.3430398	0.1520577	0.1148131	0.133168	0.1504951
590.94	5	0.480715	0.341427	0.1511063	0.1188828	0.134414	0.1513884
591.04	5	0.478987	0.3404616	0.1515979	0.110484	0.133618	0.150768
591.14	5	0.483001	0.3355968	0.1533864	0.1087626	0.127755	0.1514809
591.24	5	0.486563	0.3276314	0.1509703	0.107657	0.131192	0.1515883
591.34	5	0.490176	0.3278115	0.1500592	0.1101896	0.132085	0.1557742
591.44	5	0.475023	0.3414633	0.1508445	0.108911	0.134003	0.1545521
591.54	5	0.476893	0.3369316	0.1496185	0.1087391	0.135458	0.1541176
591.64	5	0.476342	0.3387444	0.1510302	0.1085768	0.137575	0.1569295
591.74	5	0.488343	0.3206303	0.1464658	0.1045179	0.134596	0.1581977
591.84	5	0.477956	0.3114593	0.1456506	0.1057646	0.133262	0.1562725
591.94	5	0.487754	0.3249297	0.1457283	0.1042157	0.136782	0.1611164
592.04	5	0.480356	0.313403	0.1456055	0.1003119	0.135932	0.1608622
592.14	5	0.477816	0.3039866	0.1441868	0.1026383	0.139588	0.1564643
592.24	5	0.490876	0.3010342	0.143772	0.1022713	0.134268	0.1569156
592.34	5	0.497609	0.3232602	0.1369569	0.1036126	0.133804	0.1564859
592.44	5	0.497032	0.3355081	0.1416337	0.1006257	0.138575	0.1592047
592.54	5	0.483133	0.3512152	0.1458093	0.1000956	0.135254	0.1633439
592.64	5	0.484958	0.3471149	0.1429074	0.1026305	0.136939	0.163247
592.74	5	0.476565	0.3356098	0.1416325	0.1023391	0.137927	0.1605263
592.84	5	0.463998	0.3380553	0.1427277	0.1000404	0.140231	0.1534467
592.94	5	0.49806	0.3348389	0.1445627	0.1014327	0.140771	0.1585078
593.04	5	0.48565	0.3343598	0.1484892	0.1052441	0.138833	0.1587156
593.14	5	0.488069	0.3381626	0.1486858	0.1053744	0.136724	0.157131
593.24	5	0.480459	0.3377227	0.1492756	0.1045544	0.139079	0.160906
593.34	5	0.490153	0.3433624	0.1464517	0.09862181	0.142705	0.155556
593.44	5	0.47787	0.3444502	0.1445142	0.09419881	0.140558	0.1575835
593.54	5	0.478391	0.3373528	0.1434406	0.1025298	0.140927	0.1630826
593.64	5	0.467863	0.3416156	0.1430957	0.1014571	0.142187	0.1584101
593.74	5	0.477598	0.3359685	0.1470329	0.09842432	0.144127	0.1607785
593.84	5	0.467718	0.3247507	0.1476714	0.1049233	0.142446	0.1621979
593.94	5	0.464822	0.334524	0.1459986	0.1025153	0.144624	0.1634887
594.04	5	0.481758	0.3308874	0.1469097	0.09236524	0.147702	0.1609461
594.14	5	0.472846	0.3216617	0.149079	0.09670209	0.143854	0.1501855
594.24	5	0.472201	0.3315726	0.1510924	0.1055578	0.142192	0.1490056
594.34	5	0.502578	0.3342105	0.1485088	0.1072741	0.143864	0.1539015
594.44	5	0.494669	0.3408392	0.1483314	0.1033143	0.142095	0.1551921
594.54	5	0.487994	0.3385792	0.1528523	0.09915944	0.1456	0.1524461
594.64	5	0.503558	0.3435154	0.1563618	0.09000697	0.145669	0.1525589
594.74	5	0.495766	0.3400204	0.1560678	0.09378725	0.148358	0.157113
594.84	5	0.496097	0.3324459	0.1527323	0.1011741	0.14587	0.1591182
594.94	5	0.501667	0.3273052	0.1575199	0.1028963	0.142109	0.1547719
595.04	5	0.480647	0.3366208	0.1552689	0.104887	0.145697	0.1590034
595.14	5	0.494891	0.3354557	0.1539954	0.1018116	0.142062	0.1558523
595.24	5	0.496759	0.3499818	0.1545358	0.1041353	0.145228	0.1527094
595.34	5	0.483286	0.3214397	0.1509048	0.1036449	0.143346	0.1530056
595.44	5	0.486688	0.3140394	0.1548103	0.09700213	0.143669	0.1532567
595.54	5	0.493747	0.3130013	0.1517258	0.09797619	0.146113	0.156425
595.64	5	0.485848	0.319748	0.1478239	0.09122995	0.146746	0.1498454
595.74	5	0.485164	0.3082913	0.1493393	0.09868755	0.145859	0.1571513
595.84	5	0.486097	0.2993551	0.1525205	0.1017383	0.145882	0.1581541

595.94	5	0.480476	0.312842	0.1543006	0.1044111	0.152709	0.1623542
596.04	5	0.466418	0.3334936	0.1511736	0.1071001	0.149174	0.1589355
596.14	5	0.47377	0.3459563	0.1478421	0.1011551	0.14855	0.152067
596.24	5	0.488107	0.3470505	0.1475574	0.0952635	0.144793	0.1542481
596.34	5	0.47637	0.339008	0.1489067	0.1005945	0.141319	0.1545665
596.44	5	0.491602	0.3360092	0.1482995	0.1049382	0.139976	0.1490907
596.54	5	0.471044	0.3385266	0.1483154	0.1028263	0.144649	0.1532982
596.64	5	0.503479	0.3328813	0.1483149	0.09972796	0.144835	0.1568355
596.74	5	0.501947	0.3364292	0.1493225	0.1040561	0.14431	0.1531572
596.84	5	0.496522	0.3356049	0.1482343	0.1060579	0.143825	0.1520662
596.94	5	0.515881	0.3442448	0.149322	0.09954677	0.140766	0.1515792
597.04	5	0.501599	0.3449417	0.1507338	0.1018699	0.14264	0.14876
597.14	5	0.491143	0.3400194	0.1490611	0.102712	0.139419	0.1459847
597.24	5	0.482022	0.3390402	0.1473188	0.09935039	0.139887	0.139497
597.34	5	0.481035	0.3407717	0.1455825	0.09977953	0.141938	0.1313462
597.44	5	0.481581	0.3275051	0.1430205	0.1007286	0.144164	0.1383028
597.54	5	0.490702	0.3297042	0.1425107	0.1015995	0.141773	0.1412746
597.64	5	0.502188	0.3310405	0.1451468	0.1014774	0.138967	0.146769
597.74	5	0.487829	0.3236409	0.1451569	0.09929487	0.140815	0.1515449
597.84	5	0.466396	0.3295929	0.1476819	0.09747849	0.140015	0.1556657
597.94	5	0.477049	0.3345015	0.1501618	0.09773005	0.142436	0.1551882
598.04	5	0.465003	0.338929	0.1498858	0.1013379	0.142012	0.1549576
598.14	5	0.468639	0.3416899	0.1462125	0.100527	0.137442	0.1540726
598.24	5	0.47149	0.3402703	0.1473654	0.09896274	0.138559	0.1518957
598.34	5	0.455617	0.3391148	0.1436466	0.1036259	0.139589	0.1531829
598.44	5	0.485627	0.3339488	0.1476287	0.1036148	0.136173	0.151402
598.54	5	0.470643	0.3280526	0.1468486	0.1023358	0.135595	0.1482524
598.64	5	0.484085	0.329473	0.1462975	0.1051817	0.135315	0.1475167
598.74	5	0.486585	0.3331619	0.14503	0.1047784	0.140834	0.1532614
598.84	5	0.49518	0.3453504	0.1417473	0.1093613	0.135135	0.1547024
598.94	5	0.466183	0.333843	0.1454591	0.1115502	0.137703	0.156188
599.04	5	0.501136	0.3114836	0.1506	0.105539	0.136646	0.1536401
599.14	5	0.455683	0.3062744	0.1517059	0.1060447	0.136382	0.1540399
599.24	5	0.468251	0.3221287	0.147643	0.1048003	0.133662	0.1529698
599.34	5	0.479807	0.3075984	0.1497182	0.1058058	0.138312	0.1574302
599.44	5	0.490209	0.2978838	0.1411644	0.1066797	0.142429	0.1561282
599.54	5	0.4796	0.3054297	0.1500705	0.1093672	0.139648	0.1590119
599.669	5.5	0.468569	0.3463395	0.1422446	0.1083574	0.136641	0.1544337
599.769	5.5	0.467156	0.3399906	0.1441182	0.1119037	0.138113	0.1532118
599.869	5.5	0.499916	0.3343245	0.1430682	0.1128784	0.137519	0.1493708
599.969	5.5	0.471613	0.3312255	0.1458117	0.1134946	0.136655	0.1526952
600.069	5.5	0.469906	0.3279635	0.1441979	0.1167876	0.139092	0.1499345
600.169	5.5	0.480686	0.3312375	0.1431674	0.1169975	0.125704	0.153924
600.269	5.5	0.47316	0.3345918	0.1353991	0.1178776	0.132151	0.1545125
600.369	5.5	0.485939	0.3385556	0.1440879	0.1193008	0.134347	0.1515267
600.469	5.5	0.470279	0.3429806	0.1322596	0.1175338	0.142713	0.1490723
600.569	5.5	0.461128	0.340554	0.1353649	0.1193959	0.136505	0.154305
600.669	5.5	0.441954	0.3368133	0.1388851	0.1204006	0.142106	0.1557504
600.769	5.5	0.424257	0.3421178	0.1332705	0.1170628	0.136347	0.1603846
600.869	5.5	0.4397	0.3251161	0.140866	0.118478	0.135353	0.1598333
600.969	5.5	0.45226	0.3294195	0.143205	0.1170424	0.141663	0.1651595
601.069	5.5	0.452549	0.3313297	0.1457968	0.121634	0.138549	0.1635502
601.169	5.5	0.467783	0.3248159	0.1427255	0.1212192	0.138511	0.1606401
601.269	5.5	0.463592	0.332073	0.1444121	0.1229446	0.139309	0.1596812
601.369	5.5	0.471953	0.3335788	0.1433558	0.1208094	0.139295	0.1571577
601.469	5.5	0.470339	0.3404942	0.1465859	0.1199266	0.136686	0.1522141
601.569	5.5	0.469122	0.3393164	0.1456986	0.1195515	0.136405	0.1551443
601.669	5.5	0.466547	0.3418273	0.1447168	0.1160721	0.137463	0.1591855
601.769	5.5	0.461897	0.3381031	0.1427996	0.1071717	0.139798	0.1600599
601.869	5.5	0.481749	0.3307267	0.1420414	0.110431	0.140549	0.1618768
601.969	5.5	0.481377	0.3234748	0.1418069	0.1132266	0.141551	0.1635211
602.069	5.5	0.477151	0.3336568	0.1467042	0.1085694	0.138636	0.1690734
602.169	5.5	0.475893	0.3303969	0.1464766	0.1100966	0.140094	0.1613075
602.269	5.5	0.461215	0.3426833	0.1454894	0.1115881	0.136793	0.1628694
602.369	5.5	0.477668	0.3153586	0.1472498	0.1111197	0.134679	0.1587354
602.469	5.5	0.472246	0.3073886	0.1465305	0.1157743	0.135534	0.1566621
602.569	5.5	0.477434	0.3095751	0.147491	0.1091839	0.136086	0.157019
602.669	5.5	0.477207	0.3150628	0.144532	0.1089615	0.136301	0.1574412
602.769	5.5	0.497417	0.2978565	0.1421735	0.1080139	0.13802	0.1583116
602.869	5.5	0.483528	0.2969944	0.1442588	0.1074795	0.139503	0.1591369
602.969	5.5	0.47442	0.3130546	0.1455639	0.1075685	0.139066	0.1584051
603.069	5.5	0.484328	0.3307778	0.1470714	0.1061391	0.134254	0.1586674
603.169	5.5	0.480387	0.3416719	0.1457122	0.1053049	0.127551	0.1562333
603.269	5.5	0.475904	0.3405569	0.1454584	0.1046312	0.131561	0.1579855
603.369	5.5	0.476923	0.3296518	0.1506579	0.107	0.132516	0.1541426
603.469	5.5	0.476505	0.3340204	0.1469302	0.1009642	0.13574	0.1479647
603.569	5.5	0.472674	0.3301096	0.1473766	0.1040913	0.134533	0.1435154
603.669	5.5	0.496281	0.3292653	0.151489	0.1039546	0.13767	0.1563517
603.769	5.5	0.49196	0.3365139	0.1473464	0.106491	0.135891	0.1551052
603.869	5.5	0.488476	0.3376815	0.1476669	0.1020789	0.134654	0.1319413
603.969	5.5	0.479759	0.3441038	0.1486647	0.1028646	0.139394	0.1400595
604.069	5.5	0.474077	0.3443611	0.1477234	0.1049006	0.137263	0.1471383
604.169	5.5	0.471641	0.3361534	0.1452112	0.1017066	0.138759	0.150851
604.269	5.5	0.489897	0.3407798	0.1471241	0.09498854	0.132802	0.1444626
604.369	5.5	0.481543	0.3293177	0.1504493	0.1020332	0.133937	0.1384084
604.469	5.5	0.484925	0.3280486	0.1501288	0.100274	0.140475	0.1404071
604.569	5.5	0.489687	0.3312039	0.1500717	0.103783	0.13656	0.1410732
604.669	5.5	0.471596	0.3230812	0.1499525	0.09682763	0.138577	0.1434064
604.769	5.5	0.477594	0.3236085	0.1483406	0.09322824	0.139151	0.1484525
604.869	5.5	0.45803	0.3325951	0.149124	0.1020319	0.14091	0.1498792
604.969	5.5	0.473276	0.3464125	0.1488565	0.09821045	0.141134	0.1490676
605.069	5.5	0.45542	0.345687	0.1469989	0.1015547	0.13844	0.149475
605.169	5.5	0.463052	0.3438126	0.147537	0.1026512	0.135549	0.1528968
605.269	5.5	0.480104	0.3409637	0.1505915	0.09776251	0.135949	0.1527652
605.369	5.5	0.472946	0.3359496	0.1489897	0.09275251	0.140086	0.1528259

605.469	5.5	0.492408	0.3349404	0.1475139	0.1034565	0.139328	0.1534898
605.569	5.5	0.490838	0.3450341	0.1523934	0.1028627	0.139142	0.1506326
605.669	5.5	0.48798	0.3316226	0.1500476	0.09897143	0.140131	0.1523081
605.769	5.5	0.497845	0.3497027	0.1496736	0.09324688	0.141296	0.148401
605.869	5.5	0.496786	0.3262114	0.1459584	0.08785616	0.139537	0.146883
605.969	5.5	0.483506	0.31634	0.1533558	0.09560314	0.143038	0.1472544
606.069	5.5	0.500124	0.3218457	0.1506245	0.1044683	0.146634	0.148056
606.169	5.5	0.462447	0.3275468	0.1499498	0.1022267	0.143925	0.1483249
606.269	5.5	0.487977	0.3125511	0.1488704	0.09916851	0.142673	0.1461051
606.369	5.5	0.479578	0.3060033	0.1490179	0.1007695	0.144461	0.14616
606.469	5.5	0.484747	0.3112991	0.1512552	0.10079	0.142668	0.1462605
606.569	5.5	0.485335	0.3308999	0.1521011	0.09627525	0.144627	0.1477903
606.669	5.5	0.47898	0.3502321	0.1527264	0.09658866	0.144494	0.148971
606.769	5.5	0.482016	0.3503895	0.1530134	0.09367777	0.147721	0.1503066
606.869	5.5	0.478085	0.3446703	0.1505014	0.09927762	0.147676	0.1483485
606.969	5.5	0.480104	0.3372243	0.1502856	0.09828141	0.142909	0.1488101
607.069	5.5	0.454995	0.3427464	0.1497642	0.1012371	0.146257	0.1503674
607.169	5.5	0.471178	0.3423387	0.1508541	0.101622	0.144378	0.1509127
607.269	5.5	0.470321	0.3482928	0.1501457	0.09742428	0.143321	0.1519258
607.369	5.5	0.476371	0.3503343	0.1491094	0.09503955	0.141267	0.1545205
607.469	5.5	0.473781	0.3597227	0.1498214	0.1027735	0.144524	0.1577539
607.569	5.5	0.494706	0.3551658	0.148516	0.1026158	0.145888	0.1545634
607.669	5.5	0.498712	0.3425074	0.1473958	0.09887127	0.147843	0.1572695
607.769	5.5	0.497746	0.351224	0.1455775	0.09985334	0.146583	0.1570679
607.869	5.5	0.507719	0.3504862	0.1467728	0.1030666	0.146815	0.1545721
607.969	5.5	0.484567	0.3402677	0.1478482	0.09852376	0.154865	0.1554385
608.069	5.5	0.479354	0.3515704	0.1467208	0.1036745	0.15149	0.1576699
608.169	5.5	0.472605	0.354614	0.1451231	0.1001239	0.151334	0.1577183
608.269	5.5	0.485841	0.3561737	0.1406873	0.1010371	0.147224	0.1585686
608.369	5.5	0.478906	0.3584947	0.146138	0.1011605	0.146667	0.1601229
608.469	5.5	0.50996	0.3679511	0.1441645	0.1006475	0.142659	0.1610828
608.569	5.5	0.477427	0.3703586	0.1425358	0.09833652	0.143689	0.1639203
608.669	5.5	0.46847	0.3646205	0.1427328	0.09861507	0.142602	0.1613359
608.769	5.5	0.47435	0.3642387	0.1431656	0.09994209	0.142733	0.1621609
608.869	5.5	0.459893	0.3587975	0.1477438	0.09853204	0.142927	0.1624136
608.969	5.5	0.471941	0.3546499	0.1471119	0.1013884	0.1394	0.1612877
609.069	5.5	0.458793	0.3556917	0.1483929	0.09998788	0.142572	0.1624026
609.169	5.5	0.458971	0.3635371	0.1452064	0.1018893	0.137971	0.1674735
609.269	5.5	0.476302	0.3632616	0.1425762	0.104004	0.137497	0.1629343
609.369	5.5	0.46945	0.3653631	0.1423643	0.1032104	0.141431	0.1613225
609.469	5.5	0.488704	0.3512969	0.1447475	0.1052731	0.141776	0.1627678
609.569	5.5	0.478374	0.344284	0.146551	0.1092476	0.139277	0.1631209
609.669	5.5	0.470543	0.348644	0.1435947	0.1107861	0.13645	0.1670122
609.769	5.5	0.460858	0.3276788	0.1439462	0.1124519	0.138564	0.1686032
609.869	5.5	0.465227	0.3394898	0.1461791	0.1074908	0.13972	0.1656254
609.969	5.5	0.493932	0.343905	0.1484827	0.1097533	0.139988	0.1588044
610.069	5.5	0.447822	0.3568751	0.1454341	0.1097287	0.139584	0.1609329
610.169	5.5	0.457743	0.3690552	0.1512913	0.1070712	0.136462	0.1620449
610.269	5.5	0.480882	0.3650291	0.1529951	0.1085906	0.136069	0.1598425
610.369	5.5	0.483528	0.3580572	0.1522271	0.10916	0.139718	0.1650371
610.469	5.5	0.474096	0.3545845	0.1509071	0.1098742	0.137243	0.1598416
610.569	5.5	0.465798	0.3574284	0.1547614	0.1146257	0.13636	0.1601717
610.669	5.5	0.474164	0.3620588	0.150415	0.1132179	0.137912	0.1647736
610.769	5.5	0.484918	0.3685395	0.1527132	0.117865	0.14156	0.1624556
610.869	5.5	0.469579	0.3691006	0.1481958	0.1173274	0.136123	0.1642039
610.969	5.5	0.465338	0.3796503	0.1506591	0.1191106	0.137913	0.1644041
611.069	5.5	0.470113	0.3722483	0.1499789	0.118613	0.135621	0.1649781
611.169	5.5	0.465447	0.3533978	0.1486522	0.1191994	0.13581	0.1642652
611.269	5.5	0.485064	0.3629484	0.151197	0.1194237	0.132585	0.1550045
611.369	5.5	0.468764	0.3623483	0.1517262	0.1196964	0.134016	0.1528091
611.469	5.5	0.466223	0.3608103	0.1514373	0.1219366	0.14025	0.1589503
611.569	5.5	0.440441	0.3621958	0.149104	0.1241774	0.137629	0.1604299
611.669	5.5	0.41894	0.3683902	0.1486431	0.1211755	0.133439	0.1579264
611.769	5.5	0.429232	0.3708655	0.1477678	0.1218656	0.134154	0.1572156
611.869	5.5	0.449302	0.3741107	0.1479351	0.1198725	0.134275	0.1598207
611.969	5.5	0.452716	0.3751495	0.1475771	0.1207954	0.133629	0.1626138
612.069	5.5	0.457931	0.3779328	0.1496298	0.1243264	0.135832	0.1595685
612.169	5.5	0.457454	0.376936	0.1498249	0.1254312	0.127395	0.1656636
612.269	5.5	0.472024	0.3727613	0.1500418	0.1208419	0.127129	0.1609266
612.369	5.5	0.466861	0.368154	0.1510476	0.1210089	0.129048	0.158679
612.469	5.5	0.47088	0.3603914	0.1490045	0.1200593	0.135411	0.1571714
612.569	5.5	0.46541	0.3577872	0.1475509	0.116135	0.131633	0.1559557
612.669	5.5	0.450181	0.3542276	0.1488081	0.1097646	0.133426	0.1594686
612.769	5.5	0.469664	0.352502	0.1448239	0.1101021	0.130678	0.1549135
612.869	5.5	0.47552	0.3513673	0.1479064	0.1109678	0.131545	0.1609402
612.969	5.5	0.459554	0.341586	0.1442366	0.107558	0.134184	0.1631401
613.069	5.5	0.471903	0.3370114	0.1501384	0.1123396	0.134206	0.1678225
613.169	5.5	0.452322	0.3382786	0.1514462	0.1141348	0.134116	0.1658538
613.269	5.5	0.466157	0.3230186	0.1496784	0.1130291	0.13363	0.1593252
613.369	5.5	0.471164	0.3205941	0.1495457	0.1169736	0.135129	0.1580424
613.469	5.5	0.470237	0.3320911	0.1467898	0.1106054	0.133137	0.1591433
613.569	5.5	0.478565	0.3437415	0.1500141	0.1105478	0.130595	0.1521548
613.669	5.5	0.493964	0.3564497	0.1494163	0.1081045	0.130695	0.1568812
613.769	5.5	0.48603	0.3597407	0.1467905	0.1078764	0.130573	0.1603816
613.869	5.5	0.475681	0.3550344	0.1424418	0.1074882	0.130732	0.1575774
613.969	5.5	0.484162	0.345623	0.1427368	0.1089689	0.134226	0.1581547
614.069	5.5	0.47638	0.34341	0.14557	0.1063888	0.131322	0.1594061
614.169	5.5	0.48503	0.3412549	0.1463552	0.1049244	0.132914	0.1552001
614.269	5.5	0.474189	0.3452444	0.1453151	0.1081812	0.131196	0.153594
614.369	5.5	0.470413	0.3457302	0.1462198	0.1016367	0.131016	0.1454884
614.469	5.5	0.466896	0.3463156	0.1455266	0.1049065	0.131431	0.1333677
614.569	5.5	0.494584	0.3500053	0.1491638	0.1064199	0.130925	0.1424102
614.669	5.5	0.492632	0.3502113	0.1447478	0.1075165	0.133297	0.1471014
614.769	5.5	0.48658	0.3552128	0.1436588	0.1031993	0.133861	0.1515939
614.869	5.5	0.483528	0.3517658	0.1463831	0.1040843	0.136368	0.1589125

614.969	5.5	0.470268	0.3448549	0.1437852	0.105715	0.136036	0.1589851
615.069	5.5	0.470154	0.3439162	0.1405562	0.1043286	0.134475	0.1601552
615.169	5.5	0.485876	0.3421161	0.1368086	0.09856194	0.129367	0.1584064
615.269	5.5	0.478156	0.3426153	0.1387002	0.102487	0.131711	0.1619442
615.369	5.5	0.481098	0.3419931	0.1352382	0.1009531	0.131466	0.1560887
615.469	5.5	0.477095	0.3461531	0.1414477	0.103138	0.134873	0.1591629
615.569	5.5	0.46882	0.3479874	0.1383662	0.09580455	0.134332	0.1569102
615.669	5.5	0.477098	0.3457502	0.1456545	0.09535991	0.136214	0.1552542
615.769	5.5	0.454604	0.3445683	0.1439771	0.10212	0.133678	0.1532087
615.869	5.5	0.471621	0.3422228	0.1428923	0.09713883	0.133465	0.1573788
615.969	5.5	0.451986	0.3393466	0.144515	0.1008445	0.13956	0.1607324
616.069	5.5	0.459063	0.3429155	0.14497	0.1048923	0.138461	0.1633196
616.169	5.5	0.476617	0.3416786	0.1437453	0.1001589	0.138854	0.1584962
616.269	5.5	0.470274	0.3487709	0.145874	0.0955424	0.135258	0.1581882
616.369	5.5	0.487786	0.354947	0.1478983	0.1035061	0.136777	0.1622365
616.469	5.5	0.480704	0.343192	0.1491642	0.1071338	0.140293	0.1627054
616.569	5.5	0.49187	0.3326035	0.1499149	0.1041406	0.136514	0.1659767
616.669	5.5	0.504047	0.3333405	0.1471889	0.09692299	0.137121	0.164182
616.769	5.5	0.489298	0.3287474	0.1450767	0.08916039	0.136919	0.1664861
616.869	5.5	0.482374	0.3156579	0.1440855	0.09666862	0.140198	0.1578229
616.969	5.5	0.493486	0.3279991	0.1448996	0.1046326	0.139626	0.1601934
617.069	5.5	0.46114	0.3342135	0.1497319	0.1027613	0.138201	0.1559658
617.169	5.5	0.487195	0.3476531	0.1486724	0.0993441	0.137215	0.1589151
617.269	5.5	0.476246	0.3575263	0.147461	0.1016738	0.138928	0.1561044
617.369	5.5	0.474571	0.3533715	0.1488277	0.1030658	0.142348	0.1630338
617.469	5.5	0.478817	0.3453172	0.1484677	0.1019928	0.139923	0.1595409
617.569	5.5	0.47325	0.340636	0.1492061	0.09962112	0.140876	0.1572208
617.669	5.5	0.474771	0.3370439	0.1480321	0.09367965	0.142808	0.1575907
617.769	5.5	0.478794	0.338339	0.1474377	0.1012122	0.142603	0.160764
617.869	5.5	0.473577	0.3411672	0.147465	0.1025214	0.142107	0.1645885
617.969	5.5	0.448542	0.3400982	0.1493311	0.1044626	0.143961	0.1638561
618.069	5.5	0.463541	0.3446449	0.1482752	0.1035627	0.14658	0.1661434
618.169	5.5	0.468833	0.3468531	0.1480826	0.09716458	0.142019	0.1671883
618.269	5.5	0.471024	0.348515	0.1498793	0.09743472	0.140952	0.1669796
618.369	5.5	0.471315	0.3541196	0.1500592	0.1047059	0.143597	0.1644708
618.469	5.5	0.513859	0.3422408	0.1467722	0.1043965	0.139383	0.1633009
618.569	5.5	0.494145	0.3411628	0.1480837	0.1024388	0.141231	0.1599444
618.669	5.5	0.491784	0.3384704	0.1504336	0.1011762	0.144322	0.1567805
618.769	5.5	0.500304	0.3391269	0.1435786	0.1043241	0.146534	0.1630981
618.869	5.5	0.484665	0.3380804	0.1443841	0.09980954	0.146825	0.1664262
618.969	5.5	0.472312	0.3399459	0.1452467	0.1044069	0.143513	0.1651183
619.069	5.5	0.477587	0.3445854	0.1468344	0.1006702	0.145069	0.1686715
619.169	5.5	0.482208	0.3441189	0.145043	0.1006272	0.142514	0.1745769
619.269	5.5	0.474873	0.3418497	0.1478606	0.1018514	0.140905	0.1687552
619.369	5.5	0.510922	0.3422958	0.1492572	0.1033009	0.138842	0.164553
619.469	5.5	0.480808	0.3381549	0.1480094	0.1020818	0.141462	0.1596073
619.569	5.5	0.464237	0.3391749	0.1476917	0.1013934	0.142856	0.1585824
619.669	5.5	0.460244	0.3381526	0.1499936	0.1007103	0.144956	0.1584713
619.769	5.5	0.454259	0.346792	0.1497017	0.1030106	0.14707	0.163027
619.869	5.5	0.46701	0.3480156	0.1504456	0.105049	0.143417	0.1629425
619.969	5.5	0.449496	0.3467325	0.1493891	0.1012037	0.151301	0.1639781
620.069	5.5	0.454029	0.3285335	0.1478743	0.1013269	0.149361	0.163223
620.169	5.5	0.467775	0.3273868	0.1509554	0.1053294	0.152369	0.1647684
620.269	5.5	0.462421	0.3314944	0.152365	0.1052104	0.146417	0.1612896
620.369	5.5	0.494813	0.3089833	0.1509568	0.1065757	0.144718	0.1649457
620.469	5.5	0.471203	0.3218801	0.148447	0.1083821	0.141927	0.1594164
620.569	5.5	0.460731	0.3274983	0.1528629	0.1094874	0.142724	0.1543135
620.669	5.5	0.462889	0.3385357	0.1513765	0.1121581	0.14229	0.1469244
620.769	5.5	0.448725	0.3496298	0.1499967	0.1078216	0.141129	0.1574161
620.869	5.5	0.487298	0.3533882	0.1469503	0.1093039	0.144333	0.1411134
620.969	5.5	0.447237	0.3481061	0.1576617	0.1109216	0.139012	0.1513693
621.069	5.5	0.458172	0.3401519	0.15061	0.1086532	0.139197	0.1535085
621.169	5.5	0.474778	0.33608	0.1514469	0.109857	0.138435	0.1411168
621.269	5.5	0.480254	0.3360831	0.1496418	0.1102323	0.13685	0.1428479
621.369	5.5	0.478862	0.3417051	0.1513458	0.109571	0.141807	0.1470068
621.469	5.5	0.461251	0.3392934	0.1539069	0.1138411	0.143652	0.1488565
621.569	5.5	0.468426	0.3474406	0.1535208	0.1119453	0.140971	0.1517534
621.669	5.5	0.48968	0.3462048	0.1535348	0.1165538	0.137474	0.1538121
621.769	5.5	0.461319	0.3440867	0.154821	0.1133918	0.137804	0.1512539
621.869	5.5	0.461071	0.3537717	0.1543412	0.1172169	0.142144	0.1532498
621.969	5.5	0.470359	0.3424252	0.1521717	0.117616	0.140054	0.1551998
622.069	5.5	0.458518	0.3392569	0.1536762	0.1209259	0.139042	0.1535813
622.169	5.5	0.482455	0.3390569	0.1540662	0.1197777	0.135951	0.1552473
622.269	5.5	0.465848	0.3381172	0.1528355	0.1197075	0.134892	0.153879
622.369	5.5	0.459593	0.3360409	0.1510563	0.1196863	0.135104	0.1549751
622.469	5.5	0.435462	0.3376751	0.1516996	0.1195644	0.134915	0.1531409
622.569	5.5	0.414326	0.3403607	0.1514474	0.1188631	0.132525	0.1511109
622.669	5.5	0.426241	0.342075	0.1516717	0.1183946	0.134532	0.1538761
622.769	5.5	0.44423	0.340207	0.1498765	0.1189289	0.137519	0.1528202
622.869	5.5	0.445639	0.3410946	0.1503147	0.1198199	0.133962	0.1510909
622.969	5.5	0.448786	0.3383915	0.1507878	0.1218342	0.1354	0.1526167
623.069	5.5	0.449537	0.3368272	0.1497133	0.1239561	0.133591	0.1505148
623.169	5.5	0.471718	0.3361937	0.147072	0.119237	0.134179	0.1510569
623.269	5.5	0.458765	0.343158	0.1431976	0.1181448	0.133038	0.1544466
623.369	5.5	0.461753	0.3418579	0.1505015	0.1160453	0.132766	0.1552769
623.469	5.5	0.451915	0.3420092	0.1474317	0.1134048	0.138376	0.1524134
623.569	5.5	0.44785	0.3293094	0.1473926	0.1068537	0.137338	0.1550189
623.669	5.5	0.467056	0.3184139	0.1476355	0.1109661	0.134866	0.1551851
623.769	5.5	0.469202	0.3290581	0.1481895	0.1076769	0.135038	0.1566575
623.869	5.5	0.458115	0.3102078	0.1522464	0.1090652	0.134123	0.1593785
623.969	5.5	0.465627	0.3114177	0.1497694	0.1074168	0.132362	0.1610366
624.069	5.5	0.447625	0.3209867	0.1487674	0.1112174	0.132773	0.1606497
624.169	5.5	0.460512	0.3316385	0.1456665	0.1076469	0.126215	0.1615918
624.269	5.5	0.468182	0.3371803	0.1447556	0.1123838	0.128289	0.1579135
624.369	5.5	0.465948	0.3511239	0.1457348	0.1070607	0.127094	0.1577932

624.469	5.5	0.468084	0.3483178	0.1457793	0.1049094	0.133088	0.1565718
624.569	5.5	0.484971	0.3408561	0.1476555	0.1052913	0.130553	0.1565231
624.669	5.5	0.476017	0.3342153	0.1457852	0.1037437	0.134416	0.1580324
624.769	5.5	0.472677	0.331399	0.1465937	0.1039737	0.131637	0.1605355
624.869	5.5	0.480996	0.336996	0.148582	0.1041397	0.131162	0.1613911
624.969	5.5	0.465003	0.3381097	0.1480923	0.1029232	0.132621	0.1607615
625.069	5.5	0.481855	0.3419169	0.1476267	0.09940176	0.131735	0.160372
625.169	5.5	0.478119	0.3460154	0.1533877	0.1030991	0.130715	0.1609716
625.269	5.5	0.467608	0.3416135	0.154998	0.09734093	0.129823	0.1602898
625.369	5.5	0.462132	0.3481844	0.1534671	0.1006205	0.132085	0.1680889
625.469	5.5	0.486464	0.3460732	0.1537611	0.1019302	0.129731	0.1652519
625.569	5.5	0.486748	0.3365804	0.1550623	0.1009177	0.129416	0.1621461
625.669	5.5	0.485027	0.3368106	0.1533109	0.09953813	0.130905	0.1637534
625.769	5.5	0.480218	0.3379288	0.1517385	0.1006975	0.13073	0.1629399
625.869	5.5	0.464427	0.3358328	0.1505741	0.1011217	0.131389	0.1669045
625.969	5.5	0.46057	0.3364629	0.1515391	0.1002157	0.132409	0.1703255
626.069	5.5	0.479812	0.3391864	0.1504607	0.09485336	0.130916	0.1657131
626.169	5.5	0.478789	0.3413469	0.149847	0.1007427	0.131643	0.1569991
626.269	5.5	0.483316	0.341139	0.1520155	0.09995335	0.130108	0.1623667
626.369	5.5	0.48584	0.3386384	0.1511735	0.1014518	0.130059	0.16355
626.469	5.5	0.463962	0.33694	0.1497512	0.09920321	0.132158	0.1618871
626.569	5.5	0.475283	0.3353063	0.1513415	0.08973242	0.130721	0.1617266
626.669	5.5	0.445331	0.3360365	0.1510684	0.09852982	0.131541	0.1609616
626.769	5.5	0.46633	0.3363868	0.1498864	0.0963627	0.130622	0.1668648
626.869	5.5	0.450079	0.3418634	0.1510074	0.09841763	0.133782	0.1640791
626.969	5.5	0.447278	0.3427165	0.1501809	0.1034366	0.132839	0.1662755
627.069	5.5	0.464013	0.3269673	0.1505076	0.09805709	0.132058	0.1690228
627.169	5.5	0.46327	0.3201745	0.1488124	0.09469945	0.130417	0.1722081
627.269	5.5	0.489469	0.3170462	0.1530003	0.101102	0.128434	0.1595267
627.369	5.5	0.485782	0.3153254	0.1539631	0.1046879	0.131357	0.1550636
627.469	5.5	0.49198	0.3069693	0.154633	0.103429	0.134389	0.1613564
627.569	5.5	0.505478	0.3171878	0.1503624	0.09759577	0.133515	0.1604692
627.669	5.5	0.482008	0.3279628	0.1489187	0.09091105	0.136371	0.1590185
627.769	5.5	0.47938	0.3359597	0.1479289	0.09395653	0.136999	0.1591866
627.869	5.5	0.48912	0.3496365	0.1506133	0.1030238	0.135839	0.1626857
627.969	5.5	0.464124	0.351428	0.1475955	0.1037477	0.138693	0.1625167
628.069	5.5	0.476184	0.3434457	0.1537384	0.1031464	0.13936	0.1670598
628.169	5.5	0.470849	0.3385331	0.1532019	0.1005728	0.140496	0.1618363
628.269	5.5	0.478905	0.3328627	0.14909	0.1026536	0.138719	0.1588094
628.369	5.5	0.472403	0.3362204	0.1499483	0.1016901	0.138138	0.1589915
628.469	5.5	0.462776	0.3399689	0.1467633	0.1000263	0.141592	0.1621078
628.569	5.5	0.470449	0.3400729	0.1482598	0.09407044	0.140397	0.1591839
628.669	5.5	0.470282	0.3447202	0.1494706	0.09925713	0.140861	0.1593445
628.769	5.5	0.473625	0.3457614	0.1458192	0.1021479	0.141449	0.1634018
628.869	5.5	0.454959	0.3401327	0.1415416	0.1045117	0.145137	0.1673928
628.969	5.5	0.459354	0.3459269	0.1433985	0.1042679	0.14543	0.1604185
629.069	5.5	0.470238	0.3379344	0.1471756	0.09835192	0.142619	0.1571025
629.169	5.5	0.469984	0.3372219	0.1469017	0.09297656	0.142058	0.1601189
629.269	5.5	0.481742	0.3369105	0.1440613	0.1035128	0.140807	0.1570246
629.369	5.5	0.47733	0.3372137	0.1456967	0.1071341	0.143268	0.1586565
629.469	5.5	0.4924	0.3343432	0.1418007	0.1008798	0.142155	0.1622467
629.569	5.5	0.481033	0.3374909	0.1448171	0.1014527	0.142853	0.1615461
629.669	5.5	0.494454	0.3417484	0.1447228	0.1053467	0.145154	0.1620627
629.769	5.5	0.491926	0.3406525	0.1434671	0.09948572	0.145316	0.1580004
629.869	5.5	0.473898	0.3404938	0.1470038	0.1027228	0.143309	0.1573624
629.969	5.5	0.462355	0.338801	0.1438406	0.1020141	0.143994	0.1500896
630.069	5.5	0.476603	0.3346537	0.1413245	0.09769044	0.148279	0.1398378
630.169	5.5	0.470551	0.3354634	0.1325386	0.09950512	0.145827	0.1460907
630.269	5.5	0.496718	0.3354397	0.138888	0.1027261	0.143145	0.1506917
630.369	5.5	0.473534	0.3412376	0.1330882	0.1002798	0.146832	0.1599041
630.469	5.5	0.456269	0.3454408	0.1414515	0.09961936	0.141854	0.1616898
630.569	5.5	0.464174	0.332139	0.137381	0.1002565	0.144856	0.1626438
630.669	5.5	0.436407	0.3137504	0.1437615	0.09827485	0.146463	0.1585155
630.769	5.5	0.452822	0.3189071	0.1465481	0.102327	0.143397	0.1618984
630.869	5.5	0.454766	0.3250505	0.1449711	0.1035518	0.14619	0.1508364
630.969	5.5	0.441293	0.3035747	0.1449331	0.1006838	0.144633	0.1495984
631.069	5.5	0.466747	0.3109258	0.1441184	0.1020794	0.144983	0.1521252
631.169	5.5	0.456129	0.3237791	0.1437292	0.1050697	0.145513	0.1492747
631.269	5.5	0.474639	0.3322714	0.144605	0.106539	0.145941	0.1528769
631.369	5.5	0.472648	0.3417425	0.1448039	0.1072966	0.147271	0.1570921
631.469	5.5	0.458318	0.3499971	0.1465105	0.1078047	0.147868	0.1564759
631.569	5.5	0.443395	0.3465702	0.1480996	0.1125587	0.146564	0.1543819
631.669	5.5	0.458403	0.3361367	0.1451968	0.1099516	0.154596	0.1558601
631.769	5.5	0.48346	0.3325153	0.1438593	0.1073887	0.154073	0.1573094
631.869	5.5	0.442016	0.3319857	0.1452638	0.1104866	0.153834	0.1619631
631.969	5.5	0.446017	0.3358749	0.1462536	0.1098912	0.147677	0.1618297
632.069	5.5	0.466364	0.3353654	0.1484319	0.1084513	0.148528	0.1645982
632.169	5.5	0.47007	0.3430514	0.1496795	0.1113628	0.146264	0.1576102
632.269	5.5	0.466038	0.342793	0.1464042	0.1111285	0.145096	0.1599083
632.369	5.5	0.461585	0.3354641	0.1473803	0.116475	0.146435	0.1587774
632.469	5.5	0.457161	0.3484878	0.1482596	0.1161505	0.149474	0.1539825
632.569	5.5	0.475214	0.3396674	0.1471171	0.1173692	0.145159	0.1597669
632.669	5.5	0.464307	0.3374906	0.1456914	0.1158533	0.145601	0.1588752
632.769	5.5	0.452237	0.338452	0.1462517	0.1174412	0.140473	0.1563189
632.869	5.5	0.461577	0.3369549	0.1472432	0.1160816	0.14235	0.1570274
632.969	5.5	0.455991	0.3347446	0.1484539	0.1194632	0.147912	0.1617015
633.069	5.5	0.466953	0.3354848	0.146418	0.1181617	0.146464	0.1635861
633.169	5.5	0.455163	0.3399312	0.1448136	0.1205388	0.141227	0.164485
633.269	5.5	0.462854	0.3398991	0.1484285	0.1220183	0.140732	0.1675501
633.369	5.5	0.432396	0.3393966	0.1466334	0.1212067	0.14385	0.1662461
633.469	5.5	0.415839	0.3400495	0.1436116	0.121385	0.145164	0.163764
633.569	5.5	0.405691	0.3378856	0.1468116	0.1201384	0.147813	0.1624733
633.669	5.5	0.438944	0.3339733	0.1478703	0.1217395	0.142002	0.1553154
633.769	5.5	0.439555	0.3352107	0.1425585	0.1203293	0.142378	0.1572255
633.869	5.5	0.445012	0.3398487	0.1441014	0.1212965	0.143315	0.1641804

633.969	5.5	0.444448	0.3407772	0.1424065	0.1230321	0.137682	0.1637552
634.069	5.5	0.470862	0.3344646	0.1438803	0.1200857	0.141074	0.1631463
634.169	5.5	0.454085	0.317681	0.1413594	0.1184655	0.140614	0.1674651
634.269	5.5	0.457087	0.3123686	0.1455515	0.1193233	0.139503	0.1663586
634.369	5.5	0.442884	0.3233687	0.1471437	0.1185551	0.140574	0.1661809
634.469	5.5	0.447286	0.3069136	0.1458461	0.1086102	0.137917	0.1624298
634.569	5.5	0.458264	0.3093549	0.1472078	0.1107873	0.137254	0.1600274
634.669	5.5	0.467838	0.3197666	0.145117	0.1074982	0.134694	0.1616244
634.769	5.5	0.461583	0.3263628	0.145809	0.1108195	0.137488	0.1588528
634.869	5.5	0.462736	0.3328989	0.146312	0.107439	0.14175	0.1599502
634.969	5.5	0.447715	0.3481833	0.1471846	0.1087021	0.138326	0.1590602
635.069	5.5	0.457083	0.3441804	0.1469423	0.1059969	0.137283	0.1612318
635.169	5.5	0.465571	0.3357613	0.1484549	0.1098359	0.136628	0.1578851
635.269	5.5	0.461776	0.331231	0.1513743	0.1087738	0.134994	0.1620608
635.369	5.5	0.463126	0.3292448	0.1476558	0.1063917	0.137591	0.1583282
635.469	5.5	0.476791	0.3355905	0.1480653	0.1063982	0.128839	0.1527254
635.569	5.5	0.477551	0.3357119	0.1528029	0.1049905	0.128324	0.151521
635.669	5.5	0.475359	0.3406055	0.151007	0.1055827	0.131013	0.1616727
635.769	5.5	0.474628	0.3443261	0.1497482	0.1048759	0.139468	0.1472711
635.869	5.5	0.477202	0.3391045	0.1460793	0.1023498	0.136353	0.1399311
635.969	5.5	0.467973	0.3444016	0.1552531	0.09990007	0.14003	0.148313
636.069	5.5	0.462657	0.3422013	0.1505516	0.1026354	0.136106	0.150813
636.169	5.5	0.462273	0.3350272	0.1518113	0.09963256	0.13817	0.1459509
636.269	5.5	0.45732	0.3364879	0.1479868	0.1021246	0.137232	0.142987
636.369	5.5	0.476213	0.3365893	0.1506458	0.1051849	0.137342	0.1465978
636.469	5.5	0.472271	0.3339924	0.1532703	0.1057021	0.137345	0.1495381
636.569	5.5	0.488094	0.3344109	0.1530648	0.1021232	0.136045	0.1512704
636.669	5.5	0.473664	0.3376112	0.1546051	0.1006948	0.133	0.1538539
636.769	5.5	0.477239	0.3385758	0.1545524	0.1022617	0.131674	0.1506384
636.869	5.5	0.537998	0.3397694	0.152372	0.1003388	0.13472	0.1531867
636.969	5.5	0.529192	0.3382744	0.15075	0.09842227	0.133979	0.1600408
637.069	5.5	0.513943	0.3376718	0.1528711	0.09731185	0.135099	0.1572507
637.169	5.5	0.51508	0.3353185	0.1539112	0.1014572	0.135217	0.1568918
637.269	5.5	0.514736	0.3347954	0.1516477	0.1022993	0.134023	0.1549899
637.369	5.5	0.499379	0.335417	0.1506625	0.1047833	0.132826	0.1568489
637.469	5.5	0.515464	0.3397769	0.1499093	0.0922863	0.131871	0.1552208
637.569	5.5	0.482322	0.3388404	0.1496162	0.0977615	0.133283	0.1530434
637.669	5.5	0.462792	0.3186814	0.1496304	0.09875016	0.132793	0.1542765
637.769	5.5	0.467682	0.319367	0.1481898	0.09803634	0.133802	0.1541611
637.869	5.5	0.460721	0.3147731	0.1498393	0.1018881	0.133632	0.1518887
637.969	5.5	0.489465	0.311573	0.1511277	0.1027201	0.135482	0.1530999
638.069	5.5	0.472021	0.3041036	0.1486959	0.09428094	0.133177	0.1517806
638.169	5.5	0.478579	0.3190641	0.1483338	0.09855034	0.129352	0.1508946
638.269	5.5	0.496292	0.3246672	0.142439	0.1011058	0.124163	0.1543172
638.369	5.5	0.493616	0.3256669	0.149399	0.1042012	0.130801	0.1559731
638.469	5.5	0.497999	0.3431823	0.1469017	0.104018	0.137047	0.1525535
638.569	5.5	0.515351	0.3453386	0.1480208	0.09646892	0.135259	0.1553874
638.669	5.5	0.495581	0.3341084	0.1481221	0.09569786	0.136492	0.1573796
638.769	5.5	0.476201	0.3344956	0.1501682	0.1037766	0.134367	0.1589473
638.869	5.5	0.483426	0.3273417	0.1549839	0.1042695	0.134812	0.1630855
638.969	5.5	0.487751	0.3330858	0.1553709	0.1024585	0.136382	0.1631862
639.069	5.5	0.483394	0.3378558	0.1535435	0.09789017	0.13792	0.1615702
639.169	5.5	0.484627	0.3375704	0.1484646	0.09978296	0.138372	0.1641853
639.269	5.5	0.486773	0.3446684	0.148294	0.09998158	0.136053	0.159522
639.369	5.5	0.476226	0.3441777	0.1492726	0.09791481	0.139258	0.1571704
639.469	5.5	0.49164	0.3389079	0.1508215	0.09796204	0.138975	0.157197
639.569	5.5	0.496398	0.343724	0.153459	0.1004081	0.138134	0.1581165
639.669	5.5	0.484809	0.3349132	0.1496406	0.1048226	0.138112	0.1593442
639.769	5.5	0.470792	0.3362189	0.1502309	0.1070331	0.141996	0.1617874
639.869	5.5	0.462718	0.3364135	0.1512226	0.1085878	0.141804	0.1634931
639.969	5.5	0.482877	0.3347187	0.1522216	0.1017491	0.14175	0.164801
640.069	5.5	0.469445	0.3333933	0.1519107	0.09585091	0.137942	0.1644199
640.169	5.5	0.480553	0.336738	0.1587914	0.1042408	0.14149	0.1652454
640.269	5.5	0.511609	0.339763	0.1623103	0.1037074	0.140747	0.1644313
640.369	5.5	0.539729	0.3389272	0.1610749	0.1012901	0.138477	0.1675928
640.469	5.5	0.51453	0.3403707	0.1558751	0.1021916	0.138968	0.1699869
640.569	5.5	0.511683	0.3383135	0.1598697	0.1078906	0.139963	0.1659886
640.669	5.5	0.51049	0.3344235	0.1546312	0.1021493	0.139679	0.1665635
640.769	5.5	0.505066	0.3369294	0.1572245	0.1037246	0.14291	0.1650061
640.869	5.5	0.502236	0.3341807	0.1536901	0.1026862	0.146809	0.170266
640.969	5.5	0.517322	0.3397193	0.1574962	0.09679306	0.1415	0.1706159
641.069	5.5	0.517208	0.3435805	0.1567311	0.09828438	0.145315	0.1691718
641.169	5.5	0.512306	0.3180066	0.1545285	0.1026858	0.143332	0.1612976
641.269	5.5	0.496541	0.3152477	0.1556102	0.1015926	0.144422	0.1638792
641.369	5.5	0.47451	0.314444	0.1563181	0.1018999	0.144501	0.162911
641.469	5.5	0.483014	0.3150792	0.1561536	0.09923047	0.146937	0.1611551
641.569	5.5	0.472237	0.3045337	0.1539061	0.09738853	0.145557	0.1615452
641.669	5.5	0.485468	0.3145629	0.1542926	0.1002054	0.144194	0.161248
641.769	5.5	0.494085	0.3227711	0.1524266	0.09944344	0.146684	0.1673224
641.869	5.5	0.473337	0.3258388	0.1510309	0.1010559	0.144157	0.1648135
641.969	5.5	0.485325	0.341972	0.1539657	0.1020965	0.143545	0.1664654
642.069	5.5	0.474859	0.345129	0.1511435	0.1049742	0.140966	0.1673645
642.169	5.5	0.492593	0.3388265	0.1506813	0.1036536	0.145535	0.1697241
642.269	5.5	0.480766	0.3341945	0.154802	0.1103589	0.148651	0.159033
642.369	5.5	0.471576	0.3286264	0.1556418	0.1114054	0.149081	0.1548451
642.469	5.5	0.468867	0.3306388	0.1547333	0.1143322	0.146244	0.1627228
642.569	5.5	0.47788	0.336174	0.1532717	0.1088428	0.152454	0.1633793
642.669	5.5	0.501826	0.3377176	0.153461	0.1075488	0.149617	0.1621194
642.769	5.5	0.462762	0.3452778	0.1483305	0.1113394	0.150594	0.1605642
642.869	5.5	0.465697	0.3445171	0.1497288	0.1104514	0.1438	0.1634856
642.969	5.5	0.475675	0.3347926	0.1449603	0.1112791	0.144896	0.164069
643.069	5.5	0.476434	0.3431358	0.1534492	0.1136279	0.144139	0.1672973
643.169	5.5	0.475698	0.3381907	0.1552197	0.1106915	0.144703	0.1660772
643.269	5.5	0.461597	0.3360173	0.1507721	0.1144955	0.144769	0.1620091
643.369	5.5	0.465941	0.3366474	0.1524429	0.1137991	0.145865	0.1567958

643.469	5.5	0.479631	0.3357234	0.1483957	0.1174185	0.14031	0.1578342
643.569	5.5	0.480387	0.3336231	0.1498865	0.1158556	0.142467	0.161409
643.669	5.5	0.460607	0.3344489	0.1487342	0.1173955	0.13831	0.1617924
643.769	5.5	0.458386	0.3388722	0.1451596	0.1162609	0.140224	0.1672505
643.869	5.5	0.458156	0.3396485	0.1428663	0.1166711	0.142797	0.1700643
643.969	5.5	0.450317	0.3385444	0.1448288	0.1159735	0.142718	0.163965
644.069	5.5	0.461718	0.3383234	0.1509207	0.1155596	0.137963	0.159679
644.169	5.5	0.462833	0.3357518	0.1488838	0.1170709	0.140359	0.1627307
644.269	5.5	0.436622	0.3344657	0.1461786	0.1164154	0.141606	0.1625693
644.369	5.5	0.425748	0.3351356	0.1468015	0.118413	0.141926	0.1607993
644.469	5.5	0.415003	0.3372831	0.1415187	0.1203531	0.142817	0.1631953
644.569	5.5	0.440203	0.3398363	0.1450726	0.1201906	0.137734	0.1626201
644.669	5.5	0.425023	0.3284931	0.1450624	0.1192449	0.13801	0.1638529
644.769	5.5	0.444582	0.3109943	0.1432955	0.1218583	0.138303	0.1633418
644.869	5.5	0.440223	0.3104888	0.1465818	0.1247525	0.13432	0.1603803
644.969	5.5	0.475759	0.3183599	0.1430331	0.121215	0.136736	0.1562572
645.069	5.5	0.461897	0.30387	0.1414301	0.121717	0.139348	0.146966
645.169	5.5	0.457126	0.3102877	0.1324558	0.1210255	0.135431	0.1477125
645.269	5.5	0.449778	0.3182809	0.1393475	0.1190019	0.135919	0.1502969
645.369	5.5	0.472543	0.3211832	0.1331034	0.1092246	0.135354	0.1568365
645.469	5.5	0.458499	0.3294861	0.1414861	0.1099462	0.132577	0.164339
645.569	5.5	0.493377	0.3431513	0.1374306	0.1063818	0.131954	0.1658543
645.669	5.5	0.450095	0.3410457	0.1387776	0.1080669	0.137313	0.1621387
645.769	5.5	0.452874	0.3338963	0.1473271	0.1078929	0.137474	0.165644
645.869	5.5	0.462896	0.329147	0.1442835	0.1080454	0.134252	0.1578504
645.969	5.5	0.459279	0.3262084	0.1448609	0.1066506	0.134192	0.159207
646.069	5.5	0.469134	0.336314	0.1450939	0.1129967	0.133865	0.1569611
646.169	5.5	0.463806	0.3349381	0.1435601	0.1069824	0.131861	0.1556848
646.269	5.5	0.463721	0.3417128	0.1446077	0.1078472	0.133192	0.1533319
646.369	5.5	0.476695	0.3425469	0.1439405	0.108422	0.125189	0.1594353
646.469	5.5	0.484084	0.3366228	0.1479408	0.1054681	0.131615	0.1601189
646.569	5.5	0.478794	0.3399856	0.1473372	0.1036186	0.130811	0.1559882
646.669	5.5	0.475915	0.337667	0.1451501	0.1033366	0.136315	0.1567569
646.769	5.5	0.461413	0.3309222	0.1416187	0.1028158	0.133747	0.1616767
646.869	5.5	0.463009	0.3350093	0.1422638	0.09982181	0.134681	0.1655844
646.969	5.5	0.457061	0.334743	0.1435333	0.1033678	0.129917	0.1665387
647.069	5.5	0.455268	0.3308032	0.1499163	0.09812904	0.131639	0.1653521
647.169	5.5	0.450697	0.333982	0.1505852	0.103556	0.132133	0.1610441
647.269	5.5	0.475816	0.3365062	0.1479068	0.1054448	0.133195	0.1602243
647.369	5.5	0.467608	0.33854	0.1472121	0.1019623	0.13485	0.1580683
647.469	5.5	0.483325	0.3366853	0.1487371	0.09921555	0.13426	0.1564988
647.569	5.5	0.467856	0.3386216	0.14975	0.1018817	0.133946	0.1584736
647.669	5.5	0.476554	0.335526	0.1474896	0.1040924	0.132953	0.1608893
647.769	5.5	0.472994	0.333552	0.1451185	0.1009193	0.133095	0.1579086
647.869	5.5	0.474431	0.3350767	0.1456546	0.09678016	0.132554	0.1602141
647.969	5.5	0.468659	0.3361244	0.1489801	0.09813268	0.134362	0.1597675
648.069	5.5	0.465575	0.3373209	0.1471358	0.0981462	0.134603	0.1630532
648.169	5.5	0.46418	0.3336081	0.148097	0.1019596	0.133994	0.1637705
648.269	5.5	0.459238	0.3172855	0.1506439	0.1032225	0.131177	0.166988
648.369	5.5	0.472846	0.3150041	0.1513531	0.09203903	0.130049	0.1629002
648.469	5.5	0.42809	0.31922	0.147598	0.09810449	0.130665	0.1599231
648.569	5.5	0.448468	0.3108204	0.1504345	0.1011248	0.130008	0.1588871
648.669	5.5	0.440599	0.314515	0.1553404	0.09946328	0.13331	0.1577476
648.769	5.5	0.425289	0.3232847	0.1497546	0.1052859	0.136289	0.1564528
648.869	5.5	0.454335	0.3315133	0.1488443	0.1045466	0.136693	0.1631371
648.969	5.5	0.452465	0.3424248	0.1492399	0.0949368	0.135298	0.1628248
649.069	5.5	0.466666	0.3501418	0.1509448	0.102001	0.131862	0.1630805
649.169	5.5	0.483626	0.3552397	0.1492612	0.1062339	0.126847	0.1706228
649.269	5.5	0.487153	0.3432075	0.151682	0.1097444	0.129585	0.1702117
649.369	5.5	0.486815	0.3435848	0.1587096	0.1063101	0.134232	0.1708142
649.469	5.5	0.488152	0.3415633	0.1581101	0.09480449	0.136424	0.1669206
649.569	5.5	0.480397	0.3498764	0.1557846	0.09664612	0.138993	0.1622349
649.669	5.5	0.476225	0.3530321	0.1567125	0.1057674	0.137516	0.1616952
649.769	5.5	0.467417	0.3566831	0.1574803	0.1058822	0.137233	0.1592629
649.869	5.5	0.464883	0.3611932	0.1581125	0.1041668	0.138059	0.162319
649.969	5.5	0.48025	0.3539478	0.1595924	0.09921913	0.13713	0.1618139
650.069	5.5	0.471718	0.3580418	0.1588677	0.0995822	0.138854	0.1615368
650.169	5.5	0.46533	0.3534866	0.1611436	0.1013061	0.136347	0.157075
650.269	5.5	0.456099	0.3469506	0.1641755	0.09970738	0.141491	0.1596257
650.369	5.5	0.469043	0.3462955	0.1583812	0.1006523	0.139959	0.1557825
650.469	5.5	0.47436	0.3500171	0.1534934	0.1020993	0.138128	0.1510579
650.569	5.5	0.460288	0.3523977	0.1592383	0.1056883	0.13706	0.1516346
650.669	5.5	0.444801	0.3533557	0.1561109	0.1048261	0.138706	0.1594591
650.769	5.5	0.443243	0.3597765	0.1538106	0.1078672	0.136352	0.1450835
650.869	5.5	0.455822	0.3643096	0.1510641	0.1006639	0.136318	0.1415721
650.969	5.5	0.449946	0.3630177	0.1605356	0.09710835	0.133333	0.1488458
651.069	5.5	0.460685	0.3655374	0.1545919	0.1008602	0.140573	0.1501915
651.169	5.5	0.466514	0.3607571	0.1542715	0.1007287	0.14127	0.1444584
651.269	5.5	0.502388	0.3591968	0.1506317	0.09950867	0.139828	0.1408336
651.369	5.5	0.472469	0.3573456	0.1531618	0.1002541	0.141047	0.1469444
651.469	5.5	0.475411	0.3488501	0.1542397	0.104943	0.142351	0.150683
651.569	5.5	0.478711	0.3499744	0.1551539	0.1010392	0.142438	0.152576
651.669	5.5	0.467896	0.3507042	0.1549943	0.1013842	0.146248	0.1554761
651.769	5.5	0.44585	0.3223069	0.1536351	0.1046778	0.147729	0.1522866
651.869	5.5	0.465686	0.3201745	0.1531027	0.09839197	0.143802	0.152489
651.969	5.5	0.45187	0.3251957	0.1508697	0.09901475	0.147064	0.1554512
652.069	5.5	0.488817	0.3188294	0.1525717	0.1043657	0.143462	0.1563082
652.169	5.5	0.462361	0.3111347	0.1529739	0.1033508	0.144934	0.1577464
652.269	5.5	0.457274	0.3230683	0.1502247	0.1022126	0.147256	0.1537338
652.369	5.5	0.457798	0.328937	0.1495172	0.09963483	0.148003	0.152949
652.469	5.5	0.426316	0.338488	0.1491992	0.09954447	0.147356	0.153008
652.569	5.5	0.441117	0.3533826	0.1478744	0.1005989	0.145283	0.1516017
652.669	5.5	0.440969	0.3585998	0.1499733	0.0985722	0.147571	0.1516077
652.769	5.5	0.419215	0.3485171	0.1468887	0.0990186	0.14456	0.1517473
652.869	5.5	0.453647	0.3447616	0.1487642	0.1016736	0.143427	0.1502649

652.969	5.5	0.432746	0.3378995	0.1498279	0.1038712	0.144164	0.1531785
653.069	5.5	0.462731	0.3435122	0.1488091	0.1041692	0.146888	0.1525645
653.169	5.5	0.458393	0.3463886	0.1479483	0.1079873	0.148386	0.1502945
653.269	5.5	0.449336	0.3487004	0.1392957	0.1085789	0.149575	0.153836
653.369	5.5	0.432135	0.3501304	0.1493448	0.1121189	0.14648	0.1544944
653.469	5.5	0.460138	0.350653	0.1462501	0.1086827	0.15165	0.1530327
653.569	5.5	0.467117	0.3505897	0.1462192	0.1053253	0.148261	0.1544221
653.669	5.5	0.430204	0.3485388	0.1474046	0.1094693	0.149614	0.1557933
653.769	5.5	0.436307	0.3400196	0.1480976	0.1078375	0.145315	0.1557436
653.869	5.5	0.457363	0.3404674	0.1527324	0.1061634	0.142747	0.1570729
653.969	5.5	0.461811	0.3382849	0.1532289	0.1090591	0.142104	0.1583778
654.069	5.5	0.457977	0.3408451	0.1527965	0.1092504	0.146256	0.1563579
654.169	5.5	0.461107	0.3384808	0.1471505	0.1139019	0.145493	0.1599645
654.269	5.5	0.442482	0.3422411	0.1479022	0.1133476	0.14819	0.1571753
654.369	5.5	0.463855	0.3455454	0.1488071	0.1174572	0.143894	0.1550752
654.469	5.5	0.4576	0.3437242	0.1507452	0.1159744	0.145063	0.153234
654.569	5.5	0.450446	0.3443366	0.1523283	0.1147977	0.14199	0.1571771
654.669	5.5	0.448628	0.3434559	0.1498027	0.1157282	0.140659	0.1586266
654.769	5.5	0.451149	0.3410132	0.1501817	0.1192845	0.145169	0.1614583
654.869	5.5	0.447012	0.3411515	0.1516564	0.1164534	0.145318	0.1613284
654.969	5.5	0.452853	0.3393596	0.1532502	0.1177322	0.139871	0.1629706
655.069	5.5	0.461185	0.342539	0.1502268	0.118247	0.139136	0.1611419
655.169	5.5	0.423907	0.3499659	0.1571235	0.1199934	0.141241	0.1627064
655.269	5.5	0.407528	0.3363832	0.1600356	0.1204872	0.141226	0.1625509
655.369	5.5	0.403324	0.3166644	0.1579767	0.1213683	0.144104	0.1615901
655.469	5.5	0.430776	0.3201694	0.1525122	0.1202371	0.139715	0.1684029
655.569	5.5	0.421986	0.3242734	0.1571311	0.1186936	0.137633	0.1649721
655.669	5.5	0.436549	0.3100347	0.1521299	0.1209878	0.137563	0.1655517
655.769	5.5	0.431724	0.3194717	0.1550147	0.1246861	0.134828	0.1640646
655.869	5.5	0.459979	0.3213425	0.1507923	0.1198811	0.136404	0.1671924
655.969	5.5	0.45643	0.3348031	0.1547223	0.1223373	0.137783	0.1667147
656.069	5.5	0.448376	0.3478278	0.1529293	0.1205461	0.136774	0.1661731
656.169	5.5	0.433048	0.3567206	0.1516699	0.1177796	0.134619	0.161124
656.269	5.5	0.443998	0.3557918	0.1532292	0.1113144	0.134199	0.1622683
656.369	5.5	0.43849	0.3434396	0.1543486	0.1128872	0.133564	0.1638275
656.469	5.5	0.471377	0.3415968	0.1541767	0.1097167	0.131377	0.1606023
656.569	5.5	0.450138	0.339273	0.1519371	0.1106936	0.133458	0.1647303
656.669	5.5	0.453993	0.3421751	0.1491455	0.108738	0.138556	0.1605664
656.769	5.5	0.446518	0.3435716	0.1494286	0.110418	0.136846	0.1647196
656.869	5.5	0.439557	0.3476151	0.1506184	0.1087776	0.133133	0.1621875
656.969	5.5	0.458315	0.3477082	0.149596	0.1133811	0.134094	0.166051
657.069	5.5	0.459271	0.3458902	0.1480589	0.1075413	0.133016	0.1672741
657.169	5.5	0.464252	0.3507596	0.1465226	0.1072342	0.132353	0.170137
657.269	5.5	0.465702	0.3407041	0.1512978	0.104763	0.130284	0.1631254
657.369	5.5	0.459826	0.3373767	0.1534308	0.1029902	0.12855	0.1555257
657.469	5.5	0.469969	0.3357962	0.1520626	0.1053326	0.128476	0.1595779
657.569	5.5	0.473645	0.3364561	0.1494767	0.1074841	0.133268	0.162525
657.669	5.5	0.463362	0.3365878	0.1516985	0.1030701	0.13035	0.1594602
657.769	5.5	0.467742	0.3378933	0.1455261	0.09883811	0.134787	0.1581911
657.869	5.5	0.468898	0.3422285	0.1495931	0.1035319	0.130045	0.1605999
657.969	5.5	0.460461	0.3406098	0.1463001	0.09788942	0.130698	0.1615678
658.069	5.5	0.451574	0.3405099	0.1482239	0.1006254	0.132309	0.1636535
658.169	5.5	0.476367	0.3415349	0.1550168	0.1019057	0.129275	0.1652175
658.269	5.5	0.476032	0.3375996	0.1525673	0.1010753	0.129859	0.1608594
658.369	5.5	0.485835	0.3364205	0.1508586	0.09959118	0.133084	0.1590189
658.469	5.5	0.471793	0.3356737	0.1508949	0.09840149	0.131195	0.1576931
658.569	5.5	0.461003	0.3392832	0.1515885	0.09961361	0.128935	0.1590775
658.669	5.5	0.462539	0.3441337	0.1517815	0.09879771	0.13227	0.1593547
658.769	5.5	0.469551	0.3402237	0.1509272	0.09780717	0.132783	0.1661561
658.869	5.5	0.457009	0.3166756	0.1484254	0.09914307	0.135155	0.1685877
658.969	5.5	0.456555	0.316965	0.1431557	0.1031388	0.135091	0.1639579
659.069	5.5	0.426578	0.3220324	0.1531498	0.1047843	0.132769	0.1601702
659.169	5.5	0.450822	0.3166314	0.1541606	0.1058809	0.131482	0.1645131
659.269	5.5	0.432882	0.3118683	0.1506273	0.09483048	0.132764	0.1632865
659.369	5.5	0.434515	0.3217107	0.1522145	0.09991959	0.133642	0.1599756
659.469	5.5	0.436214	0.3322919	0.1429814	0.1028044	0.133489	0.1611272
659.57	6	0.451769	0.3470429	0.148723	0.09936775	0.135685	0.1624293
659.67	6	0.481654	0.3417374	0.1460312	0.1047782	0.136897	0.1631158
659.77	6	0.478057	0.3350258	0.1465735	0.1050567	0.136834	0.163239
659.87	6	0.498963	0.3398736	0.1486419	0.1067614	0.136797	0.1605317
659.97	6	0.488725	0.339526	0.147996	0.1011094	0.13461	0.1552957
660.07	6	0.470827	0.3445516	0.1460052	0.0900941	0.130426	0.1482845
660.17	6	0.477053	0.3467567	0.1403235	0.09652569	0.129747	0.1502276
660.27	6	0.446549	0.3462564	0.1465713	0.1036471	0.133161	0.1515964
660.37	6	0.46763	0.3491445	0.1357657	0.1015782	0.134069	0.1598092
660.47	6	0.464344	0.3389577	0.1417108	0.09770886	0.13647	0.1646097
660.57	6	0.459607	0.3350463	0.1433288	0.09992693	0.133414	0.1638384
660.67	6	0.447658	0.3343619	0.1382951	0.09995054	0.135123	0.1631382
660.77	6	0.46527	0.3358552	0.1447471	0.1009892	0.136812	0.1673693
660.87	6	0.460956	0.3344382	0.1431884	0.1002113	0.137035	0.161319
660.97	6	0.454254	0.3373632	0.1462891	0.1003544	0.136583	0.162551
661.07	6	0.434701	0.3409048	0.1473582	0.1043779	0.133292	0.1596956
661.17	6	0.453243	0.3398486	0.150244	0.1078382	0.138862	0.1567318
661.27	6	0.455811	0.3386427	0.1483456	0.1057262	0.140597	0.1573404
661.37	6	0.457119	0.3403862	0.1434686	0.09920414	0.137888	0.1614725
661.47	6	0.448748	0.3372882	0.1478559	0.1005655	0.137809	0.1613512
661.57	6	0.485327	0.3367563	0.1454596	0.1003627	0.138903	0.1604157
661.67	6	0.465695	0.3345515	0.1497495	0.09922405	0.140613	0.1634243
661.77	6	0.460962	0.3416578	0.148366	0.1009224	0.140824	0.1648161
661.87	6	0.470392	0.3488218	0.1462377	0.1033398	0.141001	0.1661743
661.97	6	0.457459	0.3263763	0.1493449	0.0987179	0.144702	0.1635018
662.07	6	0.438218	0.3160194	0.1499776	0.1016497	0.14691	0.1660354
662.17	6	0.459195	0.3165522	0.1478896	0.102256	0.144222	0.1609065
662.27	6	0.446998	0.3214298	0.1456796	0.09749451	0.14534	0.159873
662.37	6	0.48984	0.3035874	0.1474714	0.09832555	0.146963	0.1607804

662.47	6	0.464992	0.3130769	0.1505732	0.09904443	0.144965	0.1581875
662.57	6	0.45032	0.3279401	0.1499016	0.09735073	0.146507	0.1591688
662.67	6	0.432282	0.3394571	0.1495951	0.09530078	0.148822	0.1612834
662.77	6	0.437657	0.3533074	0.1541496	0.09720163	0.145853	0.1580585
662.87	6	0.44024	0.3562281	0.1510208	0.09963945	0.146182	0.1577414
662.97	6	0.415017	0.346964	0.1523074	0.1011108	0.146696	0.160889
663.07	6	0.445297	0.3451678	0.154285	0.1028368	0.145335	0.1632923
663.17	6	0.428456	0.3356066	0.14901	0.1027059	0.148703	0.1606553
663.27	6	0.453654	0.3397834	0.1491528	0.1049237	0.148473	0.1627261
663.37	6	0.447121	0.3403495	0.1527034	0.1054787	0.146914	0.1623182
663.47	6	0.440104	0.3434976	0.1512028	0.1066503	0.145904	0.1603612
663.57	6	0.436398	0.3482904	0.1499021	0.1065596	0.147685	0.1595722
663.67	6	0.432894	0.3455213	0.1521883	0.1082056	0.144915	0.1585416
663.77	6	0.460806	0.3455032	0.1529428	0.1021208	0.142195	0.1561867
663.87	6	0.438179	0.3392266	0.1528309	0.1060744	0.139348	0.1637939
663.97	6	0.439688	0.33314	0.1524049	0.1090656	0.144548	0.1655996
664.07	6	0.458866	0.3327431	0.1516988	0.1072905	0.145483	0.163392
664.17	6	0.456767	0.3362042	0.1538599	0.1102318	0.146232	0.1663648
664.27	6	0.4556	0.3335821	0.1538897	0.1103899	0.144701	0.170024
664.37	6	0.448169	0.3360793	0.1567965	0.1137026	0.148882	0.1646312
664.47	6	0.459317	0.3387083	0.1573735	0.112767	0.149071	0.1647559
664.57	6	0.455009	0.3404525	0.1583809	0.1177528	0.150206	0.1625896
664.67	6	0.435514	0.3404944	0.1537267	0.1148033	0.146478	0.1624662
664.77	6	0.451822	0.3406878	0.1564035	0.1146831	0.141706	0.1607199
664.87	6	0.440071	0.3366864	0.1522045	0.1158137	0.140271	0.1612865
664.97	6	0.463224	0.3337494	0.1539391	0.1171677	0.144656	0.1622273
665.07	6	0.450864	0.3320656	0.1529398	0.1150974	0.143888	0.1644125
665.17	6	0.42661	0.336263	0.1527206	0.1174447	0.144232	0.1616112
665.27	6	0.413775	0.344553	0.1534392	0.118544	0.143328	0.1632172
665.37	6	0.42909	0.3290469	0.1532101	0.1191037	0.140458	0.1601833
665.47	6	0.429368	0.3097889	0.15341	0.1200772	0.141224	0.1559861
665.57	6	0.42682	0.3121175	0.1564994	0.120696	0.139931	0.1513865
665.67	6	0.432187	0.3189782	0.1558616	0.1194006	0.142453	0.1593559
665.77	6	0.433786	0.3092372	0.1573257	0.119969	0.144236	0.1478352
665.87	6	0.45752	0.309328	0.1568286	0.123009	0.141225	0.1404458
665.97	6	0.450727	0.322301	0.1548028	0.1189465	0.139117	0.1501141
666.07	6	0.444437	0.3320532	0.1534533	0.1219003	0.141249	0.1554023
666.17	6	0.450724	0.3491711	0.154949	0.1186223	0.141137	0.151227
666.27	6	0.423585	0.3553459	0.1540485	0.113298	0.142947	0.1468152
666.37	6	0.457668	0.3512739	0.1522429	0.1101857	0.139319	0.151609
666.47	6	0.44223	0.3437585	0.1531	0.1063779	0.136364	0.1547512
666.57	6	0.447904	0.3318563	0.150714	0.1079874	0.137587	0.1564188
666.67	6	0.436219	0.3347722	0.150309	0.10759	0.135437	0.1623014
666.77	6	0.444863	0.337073	0.1498965	0.1079903	0.134593	0.1571592
666.87	6	0.447788	0.3395478	0.1531313	0.1079307	0.138553	0.1564289
666.97	6	0.45107	0.3431309	0.1524434	0.1102194	0.141669	0.1602842
667.07	6	0.466196	0.3456448	0.1504921	0.1046605	0.138963	0.1606919
667.17	6	0.475357	0.3406036	0.1414074	0.106441	0.139537	0.1628135
667.27	6	0.466031	0.3399	0.1513447	0.101648	0.138388	0.1585232
667.37	6	0.468221	0.3345029	0.1474279	0.1018634	0.137452	0.1603347
667.47	6	0.456973	0.3352762	0.149641	0.1018509	0.136305	0.1590383
667.57	6	0.462513	0.3357465	0.1509819	0.09889676	0.140493	0.1556891
667.67	6	0.447527	0.3354405	0.1538033	0.09798038	0.140879	0.1583413
667.77	6	0.448899	0.3340966	0.1550005	0.1027033	0.137334	0.1559073
667.87	6	0.440337	0.3391229	0.152904	0.09800514	0.137877	0.1572769
667.97	6	0.473794	0.3424348	0.1496112	0.1010597	0.1374	0.1595306
668.07	6	0.472439	0.3416166	0.1486319	0.1017571	0.136197	0.1553961
668.17	6	0.461375	0.3415912	0.1522829	0.1010863	0.137159	0.1564711
668.27	6	0.451029	0.3373823	0.1530922	0.09683481	0.132578	0.1577947
668.37	6	0.463357	0.3344713	0.1505454	0.09767576	0.134153	0.1578831
668.47	6	0.456728	0.3345934	0.1494391	0.09835298	0.134147	0.1566235
668.57	6	0.461509	0.3333922	0.1499323	0.0983681	0.135574	0.159698
668.67	6	0.463091	0.3458751	0.1532056	0.09694327	0.136704	0.1594307
668.77	6	0.46165	0.3375637	0.1522288	0.09996662	0.133976	0.1598151
668.87	6	0.458754	0.3175949	0.1580793	0.1017254	0.13368	0.1632721
668.97	6	0.462417	0.3126912	0.1583205	0.1011315	0.136054	0.1655442
669.07	6	0.423307	0.3198424	0.1530352	0.09562496	0.132763	0.1629108
669.17	6	0.444678	0.3131666	0.1573762	0.1011307	0.133426	0.164695
669.27	6	0.4358	0.3072586	0.1533348	0.09714539	0.138526	0.1599551
669.37	6	0.427605	0.3194867	0.1532375	0.100985	0.135227	0.1595736
669.47	6	0.442967	0.3272472	0.1500389	0.1058616	0.130626	0.1578952
669.57	6	0.447444	0.3431447	0.1519195	0.0970462	0.130558	0.1605031
669.67	6	0.477659	0.3545853	0.1503874	0.09884377	0.131328	0.162239
669.77	6	0.471305	0.3558941	0.1503836	0.1053869	0.131965	0.1631207
669.87	6	0.470457	0.3456287	0.1512774	0.1069743	0.136544	0.1639606
669.97	6	0.475467	0.3347356	0.1479439	0.1051316	0.134172	0.163908
670.07	6	0.46361	0.334104	0.1499539	0.09509883	0.134559	0.1637188
670.17	6	0.478946	0.3378223	0.1490136	0.09752095	0.135744	0.1646947
670.27	6	0.456698	0.3388536	0.1472541	0.10763	0.13518	0.1633349
670.37	6	0.463818	0.3447034	0.1466734	0.107866	0.135094	0.1667609
670.47	6	0.463344	0.3440258	0.1490389	0.1023834	0.134869	0.1671186
670.57	6	0.457485	0.3410587	0.1476239	0.1055401	0.135097	0.1621575
670.67	6	0.453907	0.3426634	0.149028	0.1055784	0.137737	0.1640893
670.77	6	0.450681	0.3360268	0.1532775	0.1031927	0.136976	0.1619731
670.87	6	0.456713	0.3356025	0.1530195	0.1042395	0.13345	0.1648165
670.97	6	0.44899	0.3352585	0.1507059	0.1028209	0.127356	0.1658311
671.07	6	0.427431	0.3363394	0.1512553	0.1106886	0.128022	0.1620924
671.17	6	0.436086	0.3349065	0.1505315	0.1100975	0.132469	0.1569597
671.27	6	0.450219	0.3379194	0.1494774	0.1098095	0.135352	0.1628433
671.37	6	0.45519	0.3426034	0.150671	0.1027674	0.133825	0.163745
671.47	6	0.445058	0.3424415	0.1592069	0.1003559	0.131509	0.1624336
671.57	6	0.472932	0.3423842	0.157034	0.1039643	0.130039	0.1623477
671.67	6	0.464346	0.3405813	0.1516819	0.1031026	0.133768	0.16119
671.77	6	0.454415	0.3369727	0.1493029	0.1007447	0.135642	0.1663223
671.87	6	0.46374	0.3403685	0.1499333	0.1055727	0.136346	0.1617669

671.97	6	0.460122	0.3382646	0.1513326	0.1035521	0.130014	0.1640284
672.07	6	0.435996	0.3455716	0.1482092	0.1035631	0.133089	0.1662308
672.17	6	0.455846	0.3487495	0.1439042	0.1063499	0.135675	0.1679237
672.27	6	0.439893	0.3247102	0.1536461	0.09974214	0.132962	0.1597313
672.37	6	0.487607	0.315446	0.1553385	0.1017598	0.134277	0.156165
672.47	6	0.452294	0.3206409	0.1533826	0.09936671	0.137049	0.1610658
672.57	6	0.43827	0.3218577	0.152131	0.09851901	0.138259	0.1596089
672.67	6	0.43974	0.3106813	0.146416	0.09870916	0.137292	0.1598461
672.77	6	0.425159	0.3181419	0.1434556	0.1029385	0.135844	0.1607507
672.87	6	0.44012	0.3282274	0.1469546	0.1008126	0.139044	0.1616732
672.97	6	0.412038	0.3398119	0.1477068	0.1018984	0.139619	0.1601916
673.07	6	0.437118	0.3522871	0.1469035	0.1017539	0.138153	0.1655201
673.17	6	0.424989	0.3571712	0.1464258	0.1015953	0.142399	0.1645178
673.27	6	0.444984	0.3515235	0.1448004	0.103407	0.144926	0.1586628
673.37	6	0.444567	0.3468447	0.1458027	0.1055658	0.143565	0.1559772
673.47	6	0.437413	0.3397315	0.1390218	0.1063131	0.144656	0.1579589
673.57	6	0.429756	0.3440447	0.1479568	0.1059586	0.147086	0.1584516
673.67	6	0.427246	0.3427441	0.1412274	0.1064964	0.143091	0.1589988
673.77	6	0.444205	0.3471656	0.1497986	0.0988679	0.144219	0.1631114
673.87	6	0.427698	0.349515	0.1506636	0.1028096	0.145629	0.1667137
673.97	6	0.426016	0.3420299	0.1488606	0.1048002	0.14347	0.1621427
674.07	6	0.44868	0.3451748	0.1508444	0.1055837	0.146908	0.1565518
674.17	6	0.45224	0.3386303	0.1483501	0.1096353	0.147443	0.1606588
674.27	6	0.453651	0.3332379	0.150568	0.1090168	0.147406	0.1576384
674.37	6	0.447189	0.3372718	0.1518488	0.111636	0.146522	0.1578275
674.47	6	0.45154	0.336748	0.1555801	0.1115276	0.146531	0.1597257
674.57	6	0.448016	0.3383128	0.1539527	0.1147472	0.144701	0.1607479
674.67	6	0.438552	0.3396931	0.1497955	0.1118707	0.142304	0.1623802
674.77	6	0.441255	0.3438803	0.1506789	0.1131456	0.138489	0.1586246
674.87	6	0.427791	0.3463666	0.1487368	0.1140583	0.141555	0.157611
674.97	6	0.450288	0.3466832	0.1514769	0.1156878	0.143371	0.1541058
675.07	6	0.43741	0.3467176	0.153598	0.1140479	0.144186	0.1433046
675.17	6	0.427141	0.3406689	0.152906	0.1163081	0.143561	0.1486804
675.27	6	0.404255	0.3395992	0.1536211	0.1153664	0.148972	0.1496451
675.37	6	0.415713	0.3393822	0.1555293	0.1169994	0.150618	0.1554883
675.47	6	0.422963	0.3433057	0.1530931	0.115579	0.151209	0.159331
675.57	6	0.412168	0.3465642	0.1500508	0.1166197	0.144763	0.1597109
675.67	6	0.43294	0.3282887	0.1504573	0.1147339	0.143813	0.156907
675.77	6	0.419709	0.3107032	0.1525074	0.115362	0.141773	0.1607992
675.87	6	0.457205	0.3163917	0.1512355	0.1195204	0.144675	0.1552162
675.97	6	0.438519	0.3174547	0.1533127	0.1170172	0.145889	0.1576466
676.07	6	0.436842	0.314069	0.1567531	0.1189702	0.145985	0.1564994
676.17	6	0.446328	0.3184673	0.153917	0.1160236	0.145869	0.1531142
676.27	6	0.422783	0.3219333	0.1525831	0.1117094	0.143346	0.1532283
676.37	6	0.448603	0.3325028	0.156852	0.1092373	0.142328	0.1560121
676.47	6	0.445534	0.3438553	0.1542971	0.1079009	0.14048	0.1561617
676.57	6	0.44258	0.3515738	0.1522233	0.109235	0.144349	0.1552556
676.67	6	0.440095	0.351319	0.1524509	0.1075965	0.143999	0.1577976
676.77	6	0.439231	0.3452731	0.1519683	0.111155	0.140947	0.15846
676.87	6	0.458277	0.3401989	0.1520161	0.109156	0.13865	0.1601874
676.97	6	0.439628	0.341688	0.1540107	0.1116587	0.140725	0.1589514
677.07	6	0.456599	0.3421637	0.1549875	0.1079058	0.141228	0.1612465
677.17	6	0.46292	0.3389625	0.1533158	0.1075633	0.144434	0.1566581
677.27	6	0.456374	0.3462671	0.1559329	0.1036087	0.142787	0.1574408
677.37	6	0.460793	0.3482069	0.1526558	0.1041059	0.141379	0.1574181
677.47	6	0.460579	0.3438228	0.1538847	0.1043868	0.141319	0.1550978
677.57	6	0.462664	0.3411977	0.1535794	0.1034869	0.138201	0.1573748
677.67	6	0.445495	0.3361371	0.1557599	0.1022465	0.13766	0.1577738
677.77	6	0.443263	0.3369247	0.1548806	0.1050133	0.138743	0.1565282
677.87	6	0.435304	0.3400956	0.1585944	0.09894124	0.141579	0.156695
677.97	6	0.465566	0.3408929	0.1550312	0.1013335	0.138427	0.1591361
678.07	6	0.462619	0.3406043	0.1585865	0.1043142	0.136192	0.1613871
678.17	6	0.45568	0.3438309	0.1560868	0.1043764	0.134688	0.1623612
678.27	6	0.452613	0.3466769	0.1565637	0.1001202	0.134097	0.1634243
678.37	6	0.453905	0.3457149	0.1549962	0.1010692	0.135379	0.1615907
678.47	6	0.456866	0.3450961	0.1570026	0.1012105	0.140192	0.1586608
678.57	6	0.461777	0.338306	0.1558192	0.1017484	0.136491	0.1579459
678.67	6	0.461832	0.3374759	0.1558005	0.09755106	0.131837	0.1551549
678.77	6	0.454264	0.3375731	0.1528163	0.102518	0.134328	0.1557019
678.87	6	0.455665	0.340112	0.1566662	0.1045294	0.132239	0.1614948
678.97	6	0.453556	0.3454501	0.1584899	0.1024503	0.131247	0.1610609
679.07	6	0.421223	0.3351899	0.1591959	0.0962378	0.1299	0.1615304
679.17	6	0.426952	0.3161488	0.1589612	0.103621	0.127437	0.1656565
679.27	6	0.424582	0.3150074	0.1565983	0.09944779	0.129595	0.1635812
679.37	6	0.416822	0.3210869	0.1551798	0.1030763	0.132055	0.1605375
679.47	6	0.428461	0.312954	0.1556441	0.1043497	0.132543	0.1596858
679.57	6	0.448265	0.3127436	0.1555755	0.09845857	0.135476	0.1573388
679.67	6	0.479442	0.3225058	0.1528856	0.1009721	0.132417	0.1586898
679.77	6	0.468145	0.331172	0.1539714	0.1071852	0.132673	0.156089
679.87	6	0.469325	0.3469981	0.1518249	0.1092233	0.13601	0.1585785
679.97	6	0.466913	0.3553782	0.1498353	0.1073275	0.133463	0.1569402
680.07	6	0.454031	0.3555799	0.15025	0.09333573	0.13382	0.1581314
680.17	6	0.458688	0.350218	0.1527323	0.09418167	0.135899	0.1548246
680.27	6	0.448438	0.3439231	0.1506187	0.104294	0.134177	0.1579145
680.37	6	0.464847	0.3407247	0.1498583	0.1074623	0.129003	0.156302
680.47	6	0.464638	0.3408065	0.141551	0.104117	0.1312	0.1526454
680.57	6	0.457526	0.3405484	0.1514738	0.106147	0.132797	0.154272
680.67	6	0.453299	0.3432765	0.1476076	0.1040171	0.132841	0.159109
680.77	6	0.452163	0.3460995	0.1498392	0.1010609	0.136493	0.1501633
680.87	6	0.462899	0.3431922	0.1490818	0.1023734	0.133695	0.1509389
680.97	6	0.444937	0.3429478	0.1516419	0.1016407	0.133223	0.1524859
681.07	6	0.431465	0.3356686	0.1567845	0.1051342	0.135053	0.1545179
681.17	6	0.439506	0.3356504	0.1543102	0.1079687	0.135764	0.155165
681.27	6	0.449192	0.3342432	0.1483497	0.1091444	0.135224	0.1540681
681.37	6	0.453067	0.3354487	0.1475371	0.1020137	0.137151	0.1568498

681.47	6	0.440506	0.3354285	0.153294	0.1019231	0.138978	0.1595955
681.57	6	0.46368	0.3388073	0.1533504	0.1060729	0.140717	0.1597239
681.67	6	0.468476	0.3407723	0.1536739	0.1054905	0.140833	0.1638179
681.77	6	0.459	0.3398066	0.1498771	0.1040266	0.136855	0.157272
681.87	6	0.448795	0.341799	0.147028	0.104833	0.13233	0.156773
681.97	6	0.452871	0.3383278	0.151423	0.09901746	0.132902	0.1568016
682.07	6	0.436728	0.3341623	0.1493099	0.09927619	0.136596	0.1592901
682.17	6	0.448017	0.3368018	0.1576183	0.1026709	0.139122	0.1602612
682.27	6	0.448894	0.337182	0.1598464	0.09448227	0.137373	0.1642058
682.37	6	0.480653	0.3462401	0.1551888	0.09792346	0.135904	0.1637297
682.47	6	0.453903	0.3439096	0.1582787	0.09724417	0.133241	0.1647425
682.57	6	0.440177	0.316066	0.1549012	0.09610905	0.134945	0.1654737
682.67	6	0.440235	0.3161634	0.1575836	0.1000822	0.133257	0.1651616
682.77	6	0.426379	0.3153165	0.1519745	0.09873321	0.133294	0.1704439
682.87	6	0.437774	0.3198762	0.1535756	0.09876255	0.12936	0.1648652
682.97	6	0.419872	0.3129803	0.151801	0.09997135	0.133957	0.1659689
683.07	6	0.426678	0.3193153	0.1510185	0.09929939	0.1401	0.1659753
683.17	6	0.427828	0.329306	0.1538778	0.1013111	0.135316	0.1700157
683.27	6	0.436792	0.3388835	0.1512324	0.1037868	0.135988	0.1670408
683.37	6	0.435156	0.3512142	0.1500295	0.1021727	0.137077	0.1616198
683.47	6	0.432118	0.3588807	0.147922	0.1056649	0.137563	0.1621793
683.57	6	0.423413	0.3532306	0.1466024	0.105493	0.136988	0.1638402
683.67	6	0.440018	0.344093	0.1482758	0.1077788	0.136279	0.1612771
683.77	6	0.461498	0.3372034	0.1502895	0.1013112	0.139143	0.1605338
683.87	6	0.428627	0.3417529	0.145067	0.1036986	0.140028	0.1603753
683.97	6	0.442678	0.336479	0.1452229	0.1062171	0.139532	0.1647192
684.07	6	0.46126	0.3419606	0.1528534	0.1052027	0.142612	0.1606254
684.17	6	0.443856	0.343248	0.1508524	0.1080828	0.142768	0.1635689
684.27	6	0.452646	0.3425584	0.1481773	0.1075253	0.140427	0.1676613
684.37	6	0.444129	0.343126	0.1503185	0.1106645	0.141823	0.1616524
684.47	6	0.437866	0.3354037	0.1457704	0.1112111	0.147548	0.1558605
684.57	6	0.439482	0.3330875	0.1493589	0.1138703	0.144774	0.1616001
684.67	6	0.426823	0.3343514	0.1451709	0.1114581	0.143793	0.1601662
684.77	6	0.426964	0.335072	0.1513203	0.1126221	0.146454	0.1599555
684.87	6	0.418763	0.3371263	0.1509441	0.1157319	0.144507	0.1617944
684.97	6	0.445379	0.3364866	0.1456962	0.1170096	0.147113	0.1643227
685.07	6	0.436058	0.3409491	0.1451491	0.1149513	0.148045	0.1647396
685.17	6	0.424798	0.3396937	0.1480053	0.1166927	0.146552	0.1644055
685.27	6	0.402236	0.3425576	0.1471641	0.118996	0.14614	0.1598187
685.37	6	0.404504	0.3415419	0.1455115	0.1206551	0.147929	0.1592462
685.47	6	0.417642	0.3363652	0.1417778	0.1200888	0.145501	0.1592579
685.57	6	0.413378	0.3348781	0.1465504	0.1168294	0.144466	0.1584536
685.67	6	0.427696	0.3360347	0.1512563	0.1163414	0.14098	0.1633396
685.77	6	0.423224	0.3413982	0.1481504	0.1176665	0.145622	0.1651343
685.87	6	0.447461	0.348745	0.1501586	0.1201671	0.146573	0.166006
685.97	6	0.435587	0.323514	0.1428701	0.1187808	0.148269	0.1585899
686.07	6	0.433698	0.3103049	0.1433373	0.1192244	0.144092	0.1614803
686.17	6	0.443187	0.3140309	0.1467719	0.1163733	0.151077	0.1577275
686.27	6	0.424036	0.3190827	0.1463517	0.1117129	0.150826	0.1572873
686.37	6	0.443492	0.3090963	0.147772	0.1132061	0.153384	0.1607679
686.47	6	0.443516	0.3126158	0.1436237	0.1108108	0.147879	0.1632206
686.57	6	0.442518	0.324773	0.1407559	0.1113045	0.145264	0.163625
686.67	6	0.432323	0.3313038	0.1481954	0.1075267	0.143749	0.1582654
686.77	6	0.437137	0.3460165	0.1381066	0.1082139	0.146839	0.1552047
686.87	6	0.452376	0.354875	0.144416	0.1099216	0.145372	0.1474487
686.97	6	0.438241	0.3497336	0.1403065	0.1142018	0.149257	0.1535233
687.07	6	0.46091	0.3444406	0.1450357	0.1092962	0.146245	0.1528362
687.17	6	0.475824	0.3370502	0.1485962	0.110838	0.146173	0.1604987
687.27	6	0.450929	0.3377136	0.1434799	0.1091284	0.142987	0.1620685
687.37	6	0.462511	0.3386368	0.1442349	0.1088414	0.142315	0.1614893
687.47	6	0.459477	0.3384247	0.1401579	0.1097928	0.146678	0.1631947
687.57	6	0.450091	0.3437711	0.1437723	0.1083447	0.146798	0.1593088
687.67	6	0.44965	0.3429068	0.1482878	0.1055133	0.144299	0.1599181
687.77	6	0.448189	0.3416969	0.1519782	0.1080226	0.144046	0.1563566
687.87	6	0.431462	0.3394682	0.1510886	0.1013475	0.14577	0.1529723
687.97	6	0.463772	0.3334024	0.1460139	0.1053938	0.144464	0.1557041
688.07	6	0.460411	0.3368867	0.1474797	0.1067341	0.144763	0.1571388
688.17	6	0.454698	0.3353359	0.1458971	0.1048233	0.143074	0.1568724
688.27	6	0.451098	0.3358415	0.1458741	0.1024866	0.141891	0.1561167
688.37	6	0.446369	0.3360396	0.1487505	0.1046894	0.142571	0.1604146
688.47	6	0.44612	0.3389664	0.1489207	0.1008748	0.141164	0.1605772
688.57	6	0.450433	0.3394088	0.1494306	0.09915826	0.14285	0.1589554
688.67	6	0.455913	0.3417761	0.1491948	0.09886093	0.144365	0.1642216
688.77	6	0.449674	0.3416862	0.1494107	0.1036657	0.1444	0.1564481
688.87	6	0.447036	0.3372165	0.1476839	0.1045175	0.140435	0.1564671
688.97	6	0.447259	0.333061	0.1480193	0.1019654	0.140537	0.1576355
689.07	6	0.417985	0.3367402	0.148219	0.09898051	0.140727	0.1517863
689.17	6	0.428478	0.3393512	0.1460841	0.1037643	0.138638	0.1567137
689.27	6	0.4152	0.3461839	0.1480394	0.1008291	0.138345	0.1560376
689.37	6	0.412297	0.3353921	0.1489665	0.1036889	0.142652	0.1558254
689.47	6	0.42222	0.3104585	0.148753	0.1078848	0.141115	0.1583608
689.57	6	0.438251	0.3089044	0.1477654	0.09856398	0.137361	0.1597447
689.67	6	0.463773	0.3158253	0.1529588	0.1010514	0.137422	0.1613974
689.77	6	0.467221	0.3105025	0.1506703	0.1074722	0.136065	0.1613268
689.87	6	0.467107	0.314136	0.1433968	0.1078543	0.135789	0.1593659
689.97	6	0.455994	0.3189695	0.1477896	0.1073454	0.136405	0.1609706
690.07	6	0.452971	0.3268141	0.1489721	0.09634934	0.130899	0.159373
690.17	6	0.450585	0.3397384	0.1501562	0.09449637	0.134355	0.1564495
690.27	6	0.434052	0.347627	0.1497352	0.1071666	0.139638	0.1635496
690.37	6	0.456624	0.3518749	0.1529129	0.1086229	0.137433	0.1639263
690.47	6	0.44784	0.3461168	0.150657	0.1042181	0.139626	0.1615396
690.57	6	0.443213	0.3397777	0.1536611	0.1067836	0.134747	0.1664582
690.67	6	0.432013	0.3366972	0.1491239	0.108515	0.135114	0.1621101
690.77	6	0.446844	0.3385929	0.1531985	0.1045122	0.137705	0.1611604
690.87	6	0.447865	0.3397368	0.1515065	0.1045892	0.133925	0.1578644

690.97	6	0.437612	0.3423537	0.1505106	0.1034667	0.131232	0.1577895
691.07	6	0.423036	0.3445029	0.1541258	0.1089682	0.134465	0.1587282
691.17	6	0.435688	0.3441153	0.1544115	0.1119931	0.135804	0.1601645
691.27	6	0.432944	0.3412506	0.153632	0.1111711	0.139471	0.1593657
691.37	6	0.441694	0.3347737	0.1529947	0.1034882	0.1361	0.1602833
691.47	6	0.440219	0.337417	0.1545484	0.1037566	0.134561	0.1583514
691.57	6	0.466955	0.3356425	0.1525151	0.106668	0.133807	0.1560191
691.67	6	0.453649	0.3372235	0.1510827	0.1053474	0.133584	0.1526395
691.77	6	0.441942	0.3356906	0.1547443	0.104207	0.133067	0.1485796
691.87	6	0.44861	0.3391529	0.1525634	0.106752	0.133658	0.1564852
691.97	6	0.44688	0.3408856	0.1561702	0.1036739	0.1354	0.1419287
692.07	6	0.4312	0.3415709	0.1508211	0.1023352	0.137549	0.1434016
692.17	6	0.447868	0.3413661	0.1535207	0.1045976	0.137379	0.1492875
692.27	6	0.432373	0.338553	0.1571842	0.09890876	0.131307	0.1509279
692.37	6	0.477507	0.3334185	0.1561067	0.1007378	0.128583	0.1435765
692.47	6	0.445529	0.3336472	0.153642	0.1011709	0.132181	0.1493276
692.57	6	0.4363	0.3375979	0.1543778	0.1004527	0.136663	0.1529237
692.67	6	0.434536	0.3432252	0.1513285	0.1009956	0.13617	0.1538944
692.77	6	0.430805	0.334255	0.1523153	0.09910897	0.135275	0.1544851
692.87	6	0.437838	0.3111138	0.1545087	0.09944212	0.134246	0.1506236
692.97	6	0.414385	0.3107543	0.1508029	0.1008241	0.137844	0.1542192
693.07	6	0.426512	0.3124047	0.1512653	0.1012404	0.134059	0.1556284
693.17	6	0.424815	0.3108025	0.1500844	0.1018706	0.133183	0.1580722
693.27	6	0.429879	0.3086174	0.1499762	0.1060837	0.133346	0.159221
693.37	6	0.433686	0.3194174	0.1477579	0.1075515	0.139491	0.1588582
693.47	6	0.426853	0.3206387	0.1501124	0.1058196	0.137	0.1539604
693.57	6	0.422652	0.3303886	0.1506799	0.109959	0.138165	0.1545452
693.67	6	0.422648	0.3431195	0.1468484	0.1110481	0.138965	0.1561781
693.77	6	0.440134	0.3526984	0.1438449	0.1032828	0.139686	0.1544032
693.87	6	0.425854	0.3450009	0.146757	0.1075043	0.138726	0.1549684
693.97	6	0.422096	0.3399086	0.1489065	0.1104612	0.137262	0.1540083
694.07	6	0.448908	0.3361864	0.1441042	0.1118012	0.142068	0.1521035
694.17	6	0.435569	0.3377891	0.1483388	0.1154288	0.142456	0.1558578
694.27	6	0.442677	0.335134	0.1484951	0.1141949	0.14003	0.1577979
694.37	6	0.439218	0.3428032	0.1529605	0.1173686	0.14191	0.1565512
694.47	6	0.437276	0.3426215	0.1543589	0.1174836	0.143961	0.1574695
694.57	6	0.433453	0.3401994	0.1470687	0.1179644	0.141774	0.1571896
694.67	6	0.426816	0.3414732	0.1454289	0.1168283	0.145732	0.1608849
694.77	6	0.424009	0.3384969	0.145924	0.1180397	0.147314	0.163741
694.87	6	0.423942	0.3368452	0.1496652	0.118822	0.143527	0.1601006
694.97	6	0.446624	0.3375276	0.1500985	0.1202901	0.14602	0.1640919
695.07	6	0.43462	0.3403675	0.1442928	0.1199372	0.142728	0.1612964
695.17	6	0.420628	0.3408347	0.1416353	0.1191026	0.145089	0.1581042
695.27	6	0.396174	0.343024	0.1458136	0.1196593	0.145775	0.1596175
695.37	6	0.386469	0.3482139	0.145185	0.1221329	0.146743	0.1625791
695.47	6	0.415384	0.349537	0.1479157	0.1214205	0.144625	0.1621954
695.57	6	0.402574	0.354332	0.1518695	0.1203383	0.147288	0.1641213
695.67	6	0.423456	0.3518629	0.150428	0.119561	0.144902	0.1648654
695.77	6	0.414012	0.3457208	0.1495257	0.1206173	0.145478	0.1630927
695.87	6	0.440832	0.3439453	0.1517391	0.1229419	0.144186	0.1636321
695.97	6	0.431057	0.3418862	0.150253	0.1192085	0.146393	0.1641956
696.07	6	0.439211	0.341312	0.1485005	0.1206441	0.148248	0.168634
696.17	6	0.43735	0.3447812	0.1500534	0.1172931	0.148364	0.1670482
696.27	6	0.424615	0.3242621	0.1506266	0.1123192	0.149817	0.1654897
696.37	6	0.441832	0.3207695	0.1478227	0.1145557	0.151508	0.1663558
696.47	6	0.436186	0.3396291	0.1505909	0.1114489	0.151338	0.169209
696.57	6	0.43874	0.3348629	0.1509054	0.1118434	0.147561	0.1691311
696.67	6	0.429884	0.322922	0.1484968	0.1103112	0.145698	0.1653348
696.77	6	0.432154	0.3297112	0.1462698	0.1132516	0.146932	0.1581468
696.87	6	0.449906	0.3523819	0.1457352	0.1131652	0.144179	0.1628961
696.97	6	0.439461	0.3691301	0.1446823	0.1128725	0.143454	0.1627052
697.07	6	0.453439	0.3700672	0.1446139	0.1087866	0.145097	0.1630872
697.17	6	0.455933	0.3691395	0.144257	0.1116406	0.143349	0.1612155
697.27	6	0.458565	0.3634148	0.1432573	0.1107991	0.143297	0.1639541
697.37	6	0.463657	0.3641433	0.1477141	0.1104021	0.140218	0.161237
697.47	6	0.459802	0.3638349	0.1486317	0.1112239	0.143483	0.1608066
697.57	6	0.457356	0.3711489	0.144231	0.1078978	0.141428	0.1650686
697.67	6	0.449396	0.3707205	0.1440229	0.1032527	0.136644	0.1672826
697.77	6	0.445055	0.3817467	0.1431514	0.1079287	0.137428	0.1563305
697.87	6	0.432639	0.3800568	0.1472108	0.1024267	0.138275	0.1553187
697.97	6	0.460336	0.3569679	0.1457633	0.1072184	0.138708	0.1603468
698.07	6	0.458242	0.3654288	0.1465045	0.1074733	0.136508	0.1576935
698.17	6	0.444385	0.3616037	0.1521307	0.1049011	0.134157	0.1580413
698.27	6	0.44435	0.3607049	0.1475822	0.1022474	0.136801	0.1599138
698.37	6	0.442292	0.3628411	0.1469616	0.1072432	0.136826	0.1582395
698.47	6	0.445389	0.3698314	0.1449538	0.1049438	0.136215	0.1632442
698.57	6	0.457755	0.3737188	0.1465767	0.1020197	0.137449	0.160607
698.67	6	0.457442	0.3786654	0.1470522	0.1020964	0.137071	0.159539
698.77	6	0.448943	0.381241	0.1460763	0.1061044	0.134906	0.1563397
698.87	6	0.450104	0.3840698	0.1433392	0.1061537	0.134776	0.1589102
698.97	6	0.431707	0.3859496	0.150132	0.1033318	0.136884	0.1559255
699.07	6	0.412634	0.3841381	0.1497985	0.09940236	0.134986	0.1583204
699.17	6	0.422338	0.3763444	0.1457367	0.1040711	0.137664	0.1648903
699.27	6	0.409996	0.3685379	0.1463714	0.09874553	0.139112	0.1586874
699.37	6	0.407039	0.3622311	0.1416108	0.1055786	0.136151	0.1579459
699.47	6	0.422377	0.3575924	0.1433574	0.1069452	0.135788	0.1619306
699.57	6	0.443206	0.3544387	0.1464053	0.09941805	0.134906	0.1561227
699.67	6	0.468578	0.3536695	0.1477565	0.1040449	0.134111	0.1587317
699.77	6	0.459559	0.3472973	0.1468585	0.1098694	0.131348	0.16121
699.87	6	0.470726	0.3558569	0.1449328	0.1102024	0.135255	0.1606551
699.97	6	0.451208	0.3500463	0.1418995	0.1086393	0.131599	0.1567367
700.07	6	0.453017	0.3230675	0.1430461	0.09526612	0.135453	0.1550439
700.17	6	0.449372	0.3295121	0.1390278	0.09687119	0.132354	0.1481171
700.27	6	0.428584	0.3369227	0.1458296	0.1069184	0.133809	0.1471357
700.37	6	0.450381	0.3489358	0.1387876	0.109188	0.134697	0.1498173

700.47	6	0.447333	0.3534724	0.1478798	0.1039089	0.138012	0.1538558
700.57	6	0.439228	0.3557689	0.1472851	0.1045597	0.134455	0.1579714
700.67	6	0.428802	0.3506135	0.1433592	0.1068327	0.134623	0.1569177
700.77	6	0.447701	0.3471144	0.1439109	0.1049276	0.135425	0.1564016
700.87	6	0.449259	0.3431173	0.1414289	0.1060693	0.133742	0.1581804
700.97	6	0.434637	0.3476256	0.1482428	0.1052733	0.132653	0.1548779
701.07	6	0.423195	0.3518806	0.1523485	0.1087034	0.135153	0.1588965
701.17	6	0.436641	0.3546062	0.1544198	0.1111936	0.133601	0.1556378
701.27	6	0.436249	0.3590266	0.1507853	0.1088262	0.136648	0.1537854
701.37	6	0.443739	0.3519486	0.145171	0.103874	0.133824	0.1587948
701.47	6	0.428116	0.3500054	0.1479144	0.1003572	0.132687	0.1596464
701.57	6	0.482359	0.346574	0.1455495	0.1057629	0.13123	0.1567611
701.67	6	0.466445	0.3374558	0.1523039	0.1049152	0.132442	0.1589399
701.77	6	0.454808	0.3370628	0.1549357	0.1043468	0.132846	0.1572146
701.87	6	0.44392	0.3405053	0.1535821	0.1067638	0.133647	0.1616462
701.97	6	0.444031	0.3427761	0.1540051	0.1023152	0.135385	0.1638794
702.07	6	0.424601	0.3455789	0.1554922	0.1033043	0.136904	0.1576916
702.17	6	0.448554	0.3494944	0.1525251	0.107218	0.136234	0.1553366
702.27	6	0.442246	0.351728	0.1499724	0.1002853	0.131831	0.1553503
702.37	6	0.484259	0.3533288	0.1516002	0.1039745	0.130605	0.1528932
702.47	6	0.451453	0.350927	0.1523216	0.1056402	0.135075	0.1570855
702.57	6	0.446527	0.3478465	0.1488761	0.1016832	0.139528	0.1546732
702.67	6	0.428937	0.3419275	0.1488441	0.09994982	0.138294	0.1571538
702.77	6	0.429746	0.3437759	0.1517359	0.1014485	0.137675	0.1593108
702.87	6	0.43606	0.3418234	0.151504	0.1015271	0.135166	0.1614702
702.97	6	0.408462	0.3444273	0.1537833	0.1039742	0.140616	0.1608371
703.07	6	0.427149	0.3463705	0.1553693	0.1027311	0.139531	0.1621302
703.17	6	0.42378	0.3406266	0.1457734	0.1035693	0.136667	0.1587923
703.27	6	0.428582	0.3410126	0.146198	0.1069338	0.134157	0.156526
703.37	6	0.434682	0.3454556	0.1488035	0.1074262	0.13916	0.1571902
703.47	6	0.426391	0.3120922	0.1487679	0.1079455	0.134975	0.1512374
703.57	6	0.422202	0.3184398	0.1514759	0.1085167	0.135817	0.1572845
703.67	6	0.416466	0.3231936	0.155964	0.1085305	0.137608	0.161759
703.77	6	0.431754	0.3364604	0.1531892	0.1043143	0.140034	0.1625338
703.87	6	0.423849	0.3459376	0.1535966	0.1083525	0.141107	0.1674958
703.97	6	0.424862	0.3534348	0.1521607	0.1102554	0.139237	0.1641931
704.07	6	0.435372	0.3482296	0.152868	0.1105204	0.140834	0.1592917
704.17	6	0.432279	0.3426182	0.1554014	0.1122913	0.141595	0.1570671
704.27	6	0.439863	0.3369641	0.1521745	0.1116107	0.140355	0.1536387
704.37	6	0.431897	0.3388608	0.1561207	0.112187	0.141562	0.1546391
704.47	6	0.444969	0.3413393	0.1540539	0.1103305	0.14268	0.1548411
704.57	6	0.436455	0.3412247	0.1553652	0.1124148	0.143052	0.1571867
704.67	6	0.422278	0.3448205	0.1538835	0.1124686	0.146166	0.155813
704.77	6	0.421803	0.3465397	0.1569744	0.1149818	0.147723	0.1525066
704.87	6	0.421954	0.3468318	0.1526223	0.1176304	0.145299	0.154693
704.97	6	0.439339	0.3473973	0.1525547	0.1192088	0.148166	0.1536195
705.07	6	0.43859	0.3353483	0.1555581	0.1172196	0.147539	0.1490326
705.17	6	0.415375	0.3346787	0.1539963	0.1187551	0.148353	0.1537508
705.27	6	0.394275	0.3347459	0.1562891	0.1191875	0.14755	0.1508674
705.37	6	0.421914	0.3381139	0.1555263	0.1195282	0.146761	0.1360557
705.47	6	0.40684	0.3375592	0.1560022	0.1196874	0.146413	0.1467151
705.57	6	0.411366	0.3406717	0.157477	0.120352	0.147723	0.1543199
705.67	6	0.425864	0.3420856	0.156441	0.121469	0.146275	0.1487196
705.77	6	0.416166	0.3416058	0.1567678	0.1230375	0.144538	0.1467719
705.87	6	0.436071	0.3432493	0.1559508	0.1259696	0.14482	0.1492103
705.97	6	0.426768	0.3398151	0.1532572	0.1220386	0.145306	0.1518345
706.07	6	0.436689	0.3354818	0.1553939	0.1220016	0.146605	0.1543978
706.17	6	0.437432	0.338665	0.1566263	0.1203319	0.146514	0.1532155
706.27	6	0.423834	0.3340807	0.1558037	0.1131266	0.149049	0.1506232
706.37	6	0.438606	0.339615	0.1531023	0.1145684	0.148445	0.155809
706.47	6	0.433203	0.3460689	0.1531342	0.1112826	0.149541	0.1525559
706.57	6	0.436365	0.3381261	0.1523095	0.1124178	0.143186	0.1551655
706.67	6	0.430784	0.3375283	0.1503653	0.1102301	0.144945	0.154333
706.77	6	0.429086	0.3464753	0.1518054	0.1131972	0.145431	0.1546273
706.87	6	0.443273	0.3225811	0.1551155	0.1130771	0.144343	0.1522065
706.97	6	0.447403	0.3125781	0.1527287	0.1160609	0.142356	0.1535001
707.07	6	0.456881	0.3183099	0.1485429	0.1092904	0.145704	0.1545378
707.17	6	0.452625	0.3294541	0.1431938	0.112429	0.143662	0.1534173
707.27	6	0.472138	0.3394162	0.1531781	0.1073083	0.14307	0.156945
707.37	6	0.451739	0.3536188	0.1491198	0.1058496	0.13753	0.1550056
707.47	6	0.451094	0.3489742	0.1532081	0.1071347	0.139246	0.156732
707.57	6	0.451565	0.3411699	0.1520647	0.1066901	0.14036	0.1589091
707.67	6	0.443741	0.3355986	0.1545413	0.1039973	0.137271	0.158387
707.77	6	0.43747	0.3336171	0.1569182	0.1066835	0.138274	0.1599162
707.87	6	0.427816	0.3389454	0.1532815	0.1025471	0.139271	0.1599619
707.97	6	0.459817	0.3368129	0.1484589	0.1044362	0.137183	0.1596616
708.07	6	0.454423	0.3414846	0.1488902	0.1071063	0.139481	0.1629067
708.17	6	0.443926	0.343704	0.1505221	0.106935	0.136265	0.1635081
708.27	6	0.446094	0.3455955	0.1509943	0.1030411	0.136532	0.1640301
708.37	6	0.438907	0.3507669	0.1526422	0.1020652	0.137583	0.1659974
708.47	6	0.425803	0.338765	0.1494163	0.1005028	0.137316	0.1562351
708.57	6	0.434811	0.336881	0.1503059	0.09928492	0.139113	0.1597814
708.67	6	0.440696	0.3356245	0.1528761	0.09680095	0.14059	0.1620796
708.77	6	0.440341	0.3353981	0.1519106	0.1016194	0.139105	0.167311
708.87	6	0.444101	0.3370229	0.1576412	0.1042852	0.136812	0.1673245
708.97	6	0.438025	0.3378934	0.1582187	0.1049498	0.136425	0.1677326
709.07	6	0.410218	0.3400986	0.1564604	0.0959221	0.13312	0.1637022
709.17	6	0.422537	0.3402583	0.1583063	0.1021605	0.136739	0.1650661
709.27	6	0.40559	0.3403997	0.1541489	0.1000651	0.138437	0.1667936
709.37	6	0.400463	0.3400391	0.156778	0.1021641	0.13641	0.1668723
709.47	6	0.409015	0.3371564	0.1511272	0.1070193	0.135461	0.170331
709.57	6	0.430111	0.3346587	0.1542367	0.09981824	0.135084	0.1637015
709.67	6	0.463908	0.3350053	0.1535875	0.101749	0.134421	0.1662197
709.77	6	0.459455	0.3348948	0.1521125	0.1072204	0.132504	0.1678258
709.87	6	0.471016	0.3448581	0.1541223	0.1083967	0.128464	0.1710628

709.97	6	0.449908	0.339103	0.1538406	0.1067301	0.127382	0.1651211
710.07	6	0.445589	0.3320403	0.1488325	0.09836969	0.134075	0.1586521
710.17	6	0.448951	0.3378301	0.1479078	0.09929407	0.135208	0.1602518
710.27	6	0.423294	0.3287629	0.1481123	0.1085499	0.13428	0.1613538
710.37	6	0.443814	0.3076494	0.1484742	0.1105219	0.133736	0.1628078
710.47	6	0.448974	0.3125673	0.1479296	0.1049115	0.136986	0.1619523
710.57	6	0.449578	0.324129	0.1455801	0.1068781	0.132987	0.1586361
710.67	6	0.432288	0.3370689	0.1457581	0.1075103	0.13375	0.1629699
710.77	6	0.445191	0.3447047	0.1517942	0.104715	0.137176	0.1607141
710.87	6	0.447516	0.356752	0.1490102	0.1053283	0.135752	0.1640469
710.97	6	0.435269	0.35024	0.1448075	0.1045941	0.131784	0.1668415
711.07	6	0.422016	0.3493501	0.1484564	0.1083229	0.136298	0.1601608
711.17	6	0.433871	0.3435377	0.14452	0.1105547	0.13712	0.1555902
711.27	6	0.434225	0.3467234	0.1482351	0.1091284	0.140261	0.1630033
711.37	6	0.441617	0.3461287	0.1438263	0.1054633	0.140706	0.1600711
711.47	6	0.428087	0.3442559	0.1528636	0.1002249	0.13979	0.1579621
711.57	6	0.447124	0.3497676	0.1507708	0.1040026	0.137246	0.158913
711.67	6	0.441437	0.3451242	0.1461976	0.1038381	0.135759	0.1593493
711.77	6	0.446839	0.3493421	0.1470666	0.1038101	0.135288	0.1563446
711.87	6	0.441659	0.3434293	0.1495936	0.1064509	0.13861	0.160603
711.97	6	0.437335	0.3353614	0.1504322	0.1020107	0.139862	0.1570162
712.07	6	0.423663	0.3381489	0.148028	0.1024112	0.139523	0.1540543
712.17	6	0.436464	0.3376914	0.1443246	0.1063063	0.137473	0.1563248
712.27	6	0.428295	0.3384946	0.1486059	0.1028091	0.133517	0.1571548
712.37	6	0.47182	0.3393583	0.1491211	0.1060674	0.131784	0.1610578
712.47	6	0.441333	0.3415233	0.1452599	0.1061002	0.134779	0.1642713
712.57	6	0.428401	0.3429188	0.1489301	0.1036528	0.138696	0.1642576
712.67	6	0.428023	0.3425196	0.1453234	0.1028701	0.137753	0.1547475
712.77	6	0.418502	0.3426506	0.1438703	0.1038778	0.134933	0.1584057
712.87	6	0.428935	0.3371644	0.1498098	0.1041863	0.134247	0.1572712
712.97	6	0.401231	0.3331422	0.1477009	0.1061832	0.138809	0.1535802
713.07	6	0.422579	0.3328358	0.1487993	0.1030002	0.138217	0.155023
713.17	6	0.420607	0.3343024	0.1466534	0.1026132	0.135632	0.1570011
713.27	6	0.43666	0.3388032	0.1430776	0.1061541	0.13506	0.1582674
713.37	6	0.447906	0.3419105	0.1481287	0.1069997	0.139942	0.1533313
713.47	6	0.44475	0.3280904	0.1371162	0.1064549	0.137558	0.1521048
713.57	6	0.422195	0.3278665	0.1450526	0.1064339	0.137989	0.1424101
713.67	6	0.41992	0.3350743	0.1422234	0.1090596	0.138818	0.1488914
713.77	6	0.428919	0.3023205	0.1466298	0.1050796	0.141083	0.1527215
713.87	6	0.41588	0.309074	0.1511186	0.1043436	0.1386	0.16124
713.97	6	0.413476	0.3209984	0.1435025	0.1068628	0.1389	0.1610472
714.07	6	0.428988	0.3293142	0.1445335	0.106152	0.140569	0.1600509
714.17	6	0.425037	0.3361817	0.1426491	0.10885	0.141832	0.1631629
714.27	6	0.434172	0.3520505	0.1436068	0.1082399	0.141881	0.1573954
714.37	6	0.427582	0.3480384	0.1479592	0.1115986	0.140707	0.1587455
714.47	6	0.433603	0.3410636	0.1524609	0.1119043	0.142813	0.1576558
714.57	6	0.445256	0.3382124	0.1511771	0.1139349	0.143587	0.1534181
714.67	6	0.430796	0.3405996	0.1454408	0.1135061	0.148946	0.1538424
714.77	6	0.440061	0.3445918	0.1457097	0.1143104	0.148284	0.1569221
714.87	6	0.438	0.3436041	0.1449199	0.1157381	0.145097	0.1574607
714.97	6	0.456114	0.3465663	0.1462681	0.1191178	0.146043	0.1540195
715.07	6	0.451949	0.3474862	0.1512618	0.1182699	0.142036	0.1575779
715.17	6	0.425956	0.3492878	0.1508939	0.1186573	0.14458	0.160127
715.27	6	0.403055	0.3474054	0.1507518	0.1186785	0.14505	0.1597917
715.37	6	0.416202	0.3363646	0.1526959	0.1195179	0.146972	0.1610802
715.47	6	0.420561	0.3370827	0.1500375	0.1174816	0.146365	0.1529552
715.57	6	0.415157	0.3366877	0.1465402	0.1185659	0.147205	0.1529346
715.67	6	0.431688	0.3388492	0.145863	0.1183606	0.144962	0.1549921
715.77	6	0.422211	0.3389664	0.1473498	0.1214499	0.144728	0.1567976
715.87	6	0.451738	0.3418527	0.1463556	0.1222475	0.145255	0.1575896
715.97	6	0.444039	0.3423653	0.1465297	0.1188196	0.145557	0.153824
716.07	6	0.43721	0.3429744	0.1484706	0.1212173	0.148056	0.1565856
716.17	6	0.437585	0.3435351	0.1506771	0.1183802	0.14445	0.1591093
716.27	6	0.41997	0.3398924	0.1464415	0.1120103	0.14863	0.1578352
716.37	6	0.435503	0.3340694	0.1523352	0.1109938	0.145805	0.1596091
716.47	6	0.428599	0.333582	0.1499479	0.1096954	0.145061	0.1579696
716.57	6	0.433557	0.3362928	0.1404996	0.1122162	0.141639	0.1583424
716.67	6	0.41784	0.3396786	0.1461121	0.1091791	0.143158	0.156805
716.77	6	0.424501	0.3400665	0.1467682	0.1107068	0.144859	0.1534102
716.87	6	0.433543	0.3327459	0.1480728	0.108925	0.144172	0.1563111
716.97	6	0.43085	0.3274159	0.1499977	0.1105133	0.143471	0.161365
717.07	6	0.447096	0.3371642	0.1497104	0.1062172	0.145717	0.1604712
717.17	6	0.447285	0.3063306	0.1457414	0.1096194	0.145825	0.1615181
717.27	6	0.4445	0.3083825	0.1510371	0.1055715	0.144053	0.1645089
717.37	6	0.451341	0.3140624	0.1457686	0.104502	0.141164	0.1595693
717.47	6	0.445703	0.3229857	0.1509727	0.1067108	0.143061	0.159607
717.57	6	0.451307	0.3284432	0.1518681	0.105155	0.144583	0.15613
717.67	6	0.443346	0.3481393	0.1488168	0.1059827	0.140686	0.1577543
717.77	6	0.437524	0.3494098	0.1521017	0.1068486	0.140042	0.1566738
717.87	6	0.426168	0.3404673	0.1516792	0.1028446	0.141193	0.1567923
717.97	6	0.455834	0.3364632	0.1476769	0.1043918	0.143102	0.1571766
718.07	6	0.449536	0.335412	0.1491242	0.1074551	0.13999	0.1565441
718.17	6	0.442384	0.3416398	0.1490905	0.1049585	0.136663	0.1583694
718.27	6	0.446505	0.3387499	0.1486486	0.1022674	0.140275	0.1564151
718.37	6	0.431112	0.3451904	0.1470622	0.1043358	0.138118	0.1525049
718.47	6	0.426079	0.3456374	0.1533103	0.1029159	0.136644	0.1518438
718.57	6	0.438083	0.3444345	0.1518588	0.1002514	0.13773	0.1503263
718.67	6	0.442275	0.346835	0.1542021	0.09999556	0.137287	0.1395849
718.77	6	0.428394	0.3353095	0.1491093	0.1056731	0.138838	0.1462304
718.87	6	0.412234	0.3354585	0.1535404	0.1057087	0.137692	0.1522767
718.97	6	0.435928	0.3343706	0.1561919	0.1093513	0.136695	0.1476004
719.07	6	0.405991	0.3356929	0.1541609	0.09846071	0.134716	0.1471391
719.17	6	0.392952	0.3369011	0.1525625	0.1045522	0.136899	0.1508937
719.27	6	0.422602	0.3370997	0.1532758	0.100565	0.138458	0.152728
719.37	6	0.448177	0.3412659	0.1506244	0.1024309	0.134014	0.1539035

719.542	6.5	0.459	0.3392205	0.1519197	0.1060518	0.132714	0.154781
719.642	6.5	0.474972	0.3338867	0.1537916	0.09814468	0.131967	0.1530665
719.742	6.5	0.461839	0.3343194	0.1502166	0.09957835	0.13171	0.1587441
719.842	6.5	0.447756	0.3344094	0.1513584	0.1067183	0.130903	0.1542495
719.942	6.5	0.453042	0.3388005	0.1481608	0.09314662	0.131866	0.1546523
720.042	6.5	0.438911	0.3384554	0.147196	0.09709961	0.131096	0.1545938
720.142	6.5	0.441398	0.3251889	0.1462908	0.1083664	0.1359	0.1571154
720.242	6.5	0.444898	0.3222081	0.1502613	0.1101312	0.133324	0.1555127
720.342	6.5	0.445858	0.3366434	0.14871	0.1052747	0.131997	0.1542129
720.442	6.5	0.430124	0.3096219	0.1483116	0.1095574	0.129708	0.1543108
720.542	6.5	0.441904	0.3059581	0.1411843	0.1071375	0.133129	0.1540681
720.642	6.5	0.433847	0.3122021	0.1477762	0.1096252	0.131538	0.154615
720.742	6.5	0.420942	0.3219678	0.1467744	0.1049057	0.13238	0.1525735
720.842	6.5	0.413504	0.3304618	0.1497354	0.1074982	0.133777	0.1538107
720.942	6.5	0.426238	0.3504614	0.1507573	0.1103911	0.131749	0.1553663
721.042	6.5	0.437316	0.3438556	0.1464247	0.1103604	0.129156	0.1550604
721.142	6.5	0.426155	0.3381743	0.144008	0.1051968	0.132498	0.155459
721.242	6.5	0.436341	0.3343311	0.1461603	0.1024299	0.132522	0.1569392
721.342	6.5	0.435922	0.3356559	0.1468093	0.1066802	0.13517	0.1555908
721.442	6.5	0.439859	0.3392408	0.1448229	0.1038031	0.135793	0.1586188
721.542	6.5	0.437583	0.3376301	0.1415341	0.1064622	0.137744	0.1602093
721.642	6.5	0.434542	0.3406746	0.1424391	0.1081798	0.136198	0.1593987
721.742	6.5	0.420396	0.3433928	0.1449169	0.1027235	0.137928	0.1627336
721.842	6.5	0.432499	0.3482998	0.147587	0.1070786	0.136774	0.1562783
721.942	6.5	0.446696	0.3413923	0.1526065	0.1016723	0.138319	0.1559257
722.042	6.5	0.451415	0.3334659	0.1510297	0.1008029	0.138624	0.1571247
722.142	6.5	0.432023	0.3348837	0.1482277	0.10086	0.13923	0.1585391
722.242	6.5	0.436557	0.3367727	0.1489078	0.1002174	0.136372	0.160257
722.342	6.5	0.421608	0.3364229	0.1495345	0.1045543	0.131741	0.1615075
722.442	6.5	0.429044	0.3392439	0.1467285	0.1050314	0.129108	0.1612074
722.542	6.5	0.40855	0.3400558	0.1495395	0.1046494	0.133967	0.1617976
722.642	6.5	0.42795	0.3398066	0.1472851	0.104569	0.140226	0.1634119
722.742	6.5	0.427695	0.3418454	0.1460399	0.1030093	0.139639	0.1632696
722.842	6.5	0.43267	0.3402379	0.1474032	0.1036468	0.136426	0.1698087
722.942	6.5	0.446325	0.3369195	0.1468179	0.1037175	0.134275	0.1647876
723.042	6.5	0.417091	0.3327141	0.1441571	0.1065795	0.140029	0.1652653
723.142	6.5	0.420513	0.3329115	0.1431561	0.1064239	0.137	0.1650697
723.242	6.5	0.422224	0.3380972	0.1417214	0.1080716	0.132802	0.1686859
723.342	6.5	0.414848	0.3388543	0.1420817	0.104121	0.131753	0.1656708
723.442	6.5	0.413898	0.3309624	0.1416081	0.1048495	0.134526	0.1593909
723.542	6.5	0.428048	0.319163	0.1428574	0.1052102	0.131735	0.158774
723.642	6.5	0.429496	0.3267795	0.1460289	0.1057275	0.137143	0.1627507
723.742	6.5	0.436097	0.3230155	0.1451956	0.1079215	0.1361	0.1602793
723.842	6.5	0.426324	0.3036619	0.1431655	0.1104352	0.136217	0.159943
723.942	6.5	0.454688	0.3052482	0.1402549	0.1112215	0.136739	0.1547627
724.042	6.5	0.436787	0.3178335	0.1431097	0.1146819	0.137208	0.1585707
724.142	6.5	0.416322	0.3210334	0.1396938	0.1147681	0.139732	0.1553014
724.242	6.5	0.420451	0.3428076	0.1455186	0.1133708	0.142499	0.1614967
724.342	6.5	0.425856	0.3517583	0.1439725	0.1154436	0.143005	0.1634037
724.442	6.5	0.419751	0.3389051	0.1399806	0.1181837	0.143351	0.1582727
724.542	6.5	0.424722	0.3364588	0.1424431	0.1166054	0.143282	0.1530074
724.642	6.5	0.389874	0.3348729	0.1431553	0.1172384	0.143086	0.1573685
724.742	6.5	0.407589	0.3412147	0.14202	0.1166607	0.145503	0.156107
724.842	6.5	0.400631	0.3369764	0.1394959	0.1168967	0.144091	0.1529461
724.942	6.5	0.407979	0.3444601	0.1382339	0.1178005	0.143364	0.1545415
725.042	6.5	0.429031	0.3428128	0.146598	0.1200533	0.142202	0.1576043
725.142	6.5	0.420801	0.3438959	0.1426303	0.1210813	0.140799	0.1553734
725.242	6.5	0.445646	0.3426594	0.1438022	0.1228687	0.144412	0.1603791
725.342	6.5	0.443704	0.3345932	0.1406723	0.1201517	0.145581	0.1558052
725.442	6.5	0.426149	0.3335326	0.1438359	0.1216108	0.145147	0.1540879
725.542	6.5	0.422349	0.3330469	0.1440566	0.12094	0.144536	0.1561385
725.642	6.5	0.422414	0.335192	0.1439257	0.11467	0.146374	0.1538664
725.742	6.5	0.4297	0.3365136	0.1425589	0.114045	0.143173	0.1556897
725.842	6.5	0.429608	0.338395	0.1386132	0.112465	0.139475	0.1587584
725.942	6.5	0.429399	0.3405753	0.1449685	0.1134932	0.142265	0.161194
726.042	6.5	0.425793	0.3403026	0.1383786	0.1101876	0.144759	0.1562235
726.142	6.5	0.431918	0.3403668	0.1462831	0.1103363	0.146755	0.1569658
726.242	6.5	0.432653	0.3379754	0.1381975	0.1117894	0.143618	0.1557687
726.342	6.5	0.452214	0.3347525	0.146411	0.1108404	0.148161	0.1539097
726.442	6.5	0.442424	0.3341441	0.1458974	0.1108591	0.146631	0.1558926
726.542	6.5	0.450279	0.3382872	0.1424454	0.1092891	0.146109	0.158195
726.642	6.5	0.458653	0.3375905	0.1401397	0.1080951	0.140776	0.1599215
726.742	6.5	0.43394	0.3343733	0.1411048	0.1076828	0.141313	0.1542345
726.842	6.5	0.443108	0.3237443	0.1462911	0.1077126	0.143181	0.1545013
726.942	6.5	0.429253	0.3213682	0.1491001	0.104527	0.141932	0.1435155
727.042	6.5	0.422884	0.333102	0.1499311	0.1071245	0.140427	0.1442925
727.142	6.5	0.458091	0.2994666	0.1437771	0.104543	0.14212	0.1481008
727.242	6.5	0.441841	0.3059608	0.14533	0.1077875	0.14134	0.1563264
727.342	6.5	0.439986	0.3102272	0.1450685	0.1063462	0.138995	0.1572029
727.442	6.5	0.43677	0.3196505	0.1499899	0.1051108	0.136842	0.1567438
727.542	6.5	0.425398	0.3277465	0.150078	0.1046622	0.139328	0.1568042
727.642	6.5	0.452092	0.3492854	0.1493516	0.1031749	0.139005	0.1541458
727.742	6.5	0.449873	0.3364486	0.1548767	0.1014599	0.134035	0.1543442
727.842	6.5	0.445054	0.3358619	0.1507999	0.100495	0.136387	0.1542944
727.942	6.5	0.448534	0.3308378	0.1469119	0.1066625	0.135864	0.1519455
728.042	6.5	0.442698	0.3360543	0.147537	0.1071726	0.139782	0.1530093
728.142	6.5	0.416787	0.3383678	0.1488008	0.1069258	0.137057	0.1565244
728.242	6.5	0.423199	0.340557	0.1494243	0.1002324	0.133952	0.1576776
728.342	6.5	0.404375	0.3449129	0.1485104	0.1081136	0.137101	0.1544072
728.442	6.5	0.424065	0.3418061	0.1510696	0.1006288	0.136918	0.157602
728.542	6.5	0.438379	0.3476077	0.1484229	0.1059352	0.138028	0.1559881
728.642	6.5	0.449196	0.3367174	0.1511977	0.1057369	0.140718	0.1589592
728.742	6.5	0.474255	0.3348315	0.1518116	0.09739986	0.139293	0.1616428
728.842	6.5	0.466366	0.3342221	0.1413177	0.1068096	0.13834	0.1534965
728.942	6.5	0.462728	0.333492	0.1489304	0.1099849	0.137285	0.1511945

729.042	6.5	0.450359	0.3358182	0.1492006	0.106387	0.13679	0.15471
729.142	6.5	0.442879	0.3366939	0.1502839	0.09390526	0.134369	0.1525925
729.242	6.5	0.432662	0.3393779	0.1520263	0.09598079	0.138477	0.1552955
729.342	6.5	0.443684	0.3399943	0.1485294	0.1063642	0.136889	0.1523687
729.442	6.5	0.447314	0.3410248	0.1487582	0.1063823	0.13318	0.1533903
729.542	6.5	0.437112	0.3383511	0.1477608	0.1042924	0.134608	0.1575332
729.642	6.5	0.42127	0.3351032	0.1514559	0.1084039	0.133621	0.15748
729.742	6.5	0.440853	0.3345472	0.1513717	0.107227	0.134037	0.1577364
729.842	6.5	0.436785	0.3361312	0.1500669	0.1050569	0.129075	0.15894
729.942	6.5	0.429	0.3373754	0.1516799	0.1027844	0.132247	0.1570635
730.042	6.5	0.412926	0.3390919	0.1520825	0.1107111	0.132005	0.156575
730.142	6.5	0.429908	0.3249671	0.1494084	0.1123046	0.135609	0.1544222
730.242	6.5	0.437392	0.3235883	0.1539321	0.1140998	0.136525	0.1523769
730.342	6.5	0.425401	0.3290289	0.149559	0.107472	0.13325	0.1586534
730.442	6.5	0.430794	0.3119218	0.151101	0.1014927	0.132275	0.1569301
730.542	6.5	0.450839	0.3044692	0.156927	0.1039313	0.134863	0.1560946
730.642	6.5	0.428425	0.3093024	0.1538839	0.1039942	0.131367	0.1629151
730.742	6.5	0.419312	0.3157325	0.1565063	0.1025705	0.133432	0.1567221
730.842	6.5	0.420905	0.3222651	0.1509529	0.1050604	0.133802	0.1569701
730.942	6.5	0.407253	0.3456797	0.152967	0.09883319	0.131858	0.1566442
731.042	6.5	0.430365	0.3443248	0.154669	0.1031807	0.130779	0.158122
731.142	6.5	0.420076	0.3329235	0.1548218	0.09976031	0.135607	0.1566131
731.242	6.5	0.445809	0.3301153	0.1530525	0.1000627	0.135323	0.159161
731.342	6.5	0.429912	0.3344816	0.1492181	0.101232	0.138682	0.1588897
731.442	6.5	0.421841	0.3376591	0.1515261	0.09882731	0.137681	0.1604846
731.542	6.5	0.396721	0.3381239	0.1525082	0.1009738	0.136811	0.1593865
731.642	6.5	0.418754	0.3412805	0.147695	0.1032663	0.135659	0.1592145
731.742	6.5	0.402703	0.3441472	0.148457	0.1006703	0.136136	0.1581035
731.842	6.5	0.409701	0.3468459	0.1492063	0.1036379	0.137113	0.1506188
731.942	6.5	0.408813	0.3416297	0.1479018	0.1026208	0.136858	0.1560326
732.042	6.5	0.415026	0.332059	0.1467723	0.1032394	0.137255	0.1414414
732.142	6.5	0.432247	0.3337907	0.1492373	0.1062821	0.137524	0.1413003
732.242	6.5	0.418755	0.3350529	0.14603	0.1076552	0.135892	0.1466658
732.342	6.5	0.412673	0.3349259	0.1414941	0.1061176	0.131917	0.1494686
732.442	6.5	0.412092	0.3395987	0.1437825	0.1088673	0.127498	0.148002
732.542	6.5	0.416762	0.3407797	0.1468786	0.1051418	0.130364	0.1492142
732.642	6.5	0.398725	0.3398975	0.1488217	0.1046606	0.135318	0.1504461
732.742	6.5	0.426881	0.3411148	0.1512387	0.1075905	0.135746	0.1505686
732.842	6.5	0.428446	0.3414264	0.1521498	0.1058187	0.13331	0.1532239
732.942	6.5	0.434449	0.3398479	0.1516642	0.107057	0.131241	0.1525186
733.042	6.5	0.426623	0.3358451	0.1485201	0.1086482	0.137263	0.1573358
733.142	6.5	0.431511	0.3369171	0.1449037	0.1122146	0.135017	0.1559688
733.242	6.5	0.431328	0.3367827	0.1446576	0.112841	0.134647	0.1550129
733.342	6.5	0.412595	0.3408812	0.1466664	0.1160877	0.134918	0.1537898
733.442	6.5	0.420342	0.3277499	0.1467042	0.1157608	0.140043	0.1549323
733.542	6.5	0.409777	0.3247972	0.1444661	0.1162576	0.137802	0.1554389
733.642	6.5	0.413575	0.3293962	0.1452734	0.1167216	0.138674	0.1519042
733.742	6.5	0.421061	0.3190195	0.1478448	0.1155741	0.142325	0.153445
733.842	6.5	0.387513	0.3104572	0.1510718	0.1162762	0.143995	0.1527381
733.942	6.5	0.393849	0.3032327	0.1568926	0.1179464	0.143387	0.1543242
734.042	6.5	0.398199	0.3158767	0.1542095	0.1206588	0.144098	0.1498573
734.142	6.5	0.402209	0.3166786	0.1514003	0.1195371	0.145042	0.1510344
734.242	6.5	0.414282	0.3371148	0.1521248	0.1193353	0.14697	0.1534808
734.342	6.5	0.406204	0.346258	0.1537999	0.1200319	0.147206	0.1530226
734.442	6.5	0.433447	0.3347699	0.1484887	0.1208758	0.147627	0.1542096
734.542	6.5	0.424799	0.3299115	0.1500275	0.1198209	0.145734	0.1556183
734.642	6.5	0.424014	0.330389	0.1471941	0.1196084	0.143212	0.1542642
734.742	6.5	0.417257	0.3357525	0.1488171	0.1178102	0.147603	0.1594282
734.842	6.5	0.423593	0.3362747	0.1496718	0.1107639	0.145995	0.163167
734.942	6.5	0.42509	0.3420626	0.1471543	0.1085914	0.146025	0.1607208
735.042	6.5	0.42398	0.3423052	0.1452269	0.1058568	0.146038	0.163261
735.142	6.5	0.424144	0.3434029	0.1455259	0.10739	0.144644	0.1564009
735.242	6.5	0.419002	0.3459927	0.1450348	0.105943	0.148853	0.1551039
735.342	6.5	0.428463	0.3344581	0.1453335	0.1098838	0.147833	0.1578338
735.442	6.5	0.426699	0.3336512	0.1445833	0.111351	0.147417	0.1592299
735.542	6.5	0.44093	0.3322029	0.1470398	0.1117733	0.147034	0.1613197
735.642	6.5	0.442178	0.3327513	0.1504519	0.1099063	0.147728	0.1633652
735.742	6.5	0.442097	0.3345154	0.1472503	0.1090842	0.145952	0.1620567
735.842	6.5	0.457956	0.3358426	0.1470008	0.1061699	0.144786	0.1622714
735.942	6.5	0.429388	0.3401456	0.1428319	0.1066768	0.145503	0.1655235
736.042	6.5	0.442513	0.3381363	0.1461657	0.1073871	0.145923	0.164209
736.142	6.5	0.437917	0.3375539	0.1432439	0.1051226	0.146669	0.1705324
736.242	6.5	0.431934	0.3369344	0.1484112	0.1070076	0.144545	0.1662822
736.342	6.5	0.445409	0.3334517	0.1482553	0.102117	0.149237	0.165949
736.442	6.5	0.440662	0.3336325	0.146734	0.1047304	0.147098	0.1639993
736.542	6.5	0.436222	0.3383574	0.1464098	0.1064714	0.149449	0.16592
736.642	6.5	0.440428	0.3396235	0.1465339	0.1055291	0.143418	0.1656081
736.742	6.5	0.428119	0.3352837	0.1465188	0.104737	0.145692	0.1592368
736.842	6.5	0.427222	0.3241506	0.1431504	0.1067555	0.147042	0.1573008
736.942	6.5	0.435179	0.3217621	0.1447645	0.1028184	0.144057	0.1632011
737.042	6.5	0.442897	0.3302802	0.1476265	0.1005043	0.144017	0.1596835
737.142	6.5	0.44146	0.3065305	0.1460713	0.1040686	0.140541	0.160687
737.242	6.5	0.426551	0.3045803	0.1458277	0.1051753	0.138641	0.1529809
737.342	6.5	0.409781	0.3120617	0.1391599	0.1063199	0.136866	0.1594921
737.442	6.5	0.415151	0.3166	0.1433244	0.09821577	0.134662	0.1576746
737.542	6.5	0.399483	0.327219	0.1459653	0.1028636	0.136887	0.1640715
737.642	6.5	0.396826	0.3519734	0.1481409	0.1012064	0.138536	0.168119
737.742	6.5	0.402376	0.3404862	0.1449754	0.1047837	0.135931	0.1656831
737.842	6.5	0.428707	0.3352195	0.1388444	0.1049969	0.137246	0.1562811
737.942	6.5	0.460171	0.3316333	0.1417318	0.09901916	0.138146	0.161755
738.042	6.5	0.457048	0.3358018	0.1361992	0.1038951	0.138786	0.1627675
738.142	6.5	0.457905	0.3406776	0.1442069	0.1067085	0.137984	0.1604093
738.242	6.5	0.439517	0.3464308	0.1400872	0.1091299	0.135337	0.1598867
738.342	6.5	0.427402	0.3513373	0.1458953	0.1005844	0.137797	0.1619746
738.442	6.5	0.436495	0.3449549	0.1471342	0.09509282	0.138636	0.1608474

738.542	6.5	0.435692	0.3531925	0.1444041	0.1033814	0.138333	0.1666223
738.642	6.5	0.441857	0.3441268	0.1417882	0.1055479	0.139943	0.159531
738.742	6.5	0.448347	0.3449462	0.1429153	0.1031552	0.1407	0.1582569
738.842	6.5	0.420443	0.3475373	0.1496727	0.1023086	0.139776	0.1574921
738.942	6.5	0.438406	0.3522743	0.1544027	0.1058395	0.140191	0.1590543
739.042	6.5	0.436818	0.3605468	0.1522152	0.1046565	0.139443	0.1593296
739.142	6.5	0.430882	0.3673374	0.1470386	0.1018112	0.139039	0.1621991
739.242	6.5	0.419239	0.3735642	0.1489146	0.1047227	0.143595	0.1658761
739.342	6.5	0.4279	0.3757003	0.1476602	0.1090032	0.142603	0.1561294
739.442	6.5	0.421176	0.3793513	0.1510268	0.113704	0.138057	0.1566065
739.542	6.5	0.42979	0.3732505	0.1535089	0.1066152	0.138773	0.1610902
739.642	6.5	0.412629	0.3679481	0.1516344	0.09836433	0.136048	0.1575874
739.742	6.5	0.443445	0.3642052	0.1523945	0.1030035	0.136559	0.1578283
739.842	6.5	0.430777	0.3553734	0.1508057	0.1042392	0.132493	0.1614216
739.942	6.5	0.422606	0.3489727	0.1492113	0.1015185	0.129967	0.1594917
740.042	6.5	0.424778	0.3490913	0.1502783	0.1065464	0.131998	0.1564732
740.142	6.5	0.412545	0.3304285	0.153439	0.1020276	0.137151	0.1565973
740.242	6.5	0.428814	0.3362994	0.1520693	0.1034186	0.135822	0.1471557
740.342	6.5	0.417268	0.3399893	0.1520995	0.1018045	0.133698	0.1460098
740.442	6.5	0.445459	0.3210436	0.1550041	0.09913787	0.132505	0.1523872
740.542	6.5	0.432104	0.3142898	0.1507918	0.1019741	0.135974	0.1573807
740.642	6.5	0.422022	0.3173073	0.1540498	0.1000651	0.132454	0.1607828
740.742	6.5	0.395338	0.3246588	0.1549468	0.09630627	0.131787	0.1571617
740.842	6.5	0.412782	0.3497435	0.1442765	0.09776884	0.134449	0.1552159
740.942	6.5	0.404085	0.363794	0.1504598	0.09808706	0.132104	0.1554403
741.042	6.5	0.406003	0.360258	0.1516221	0.1003398	0.132198	0.1521984
741.142	6.5	0.406445	0.3557993	0.1529004	0.09954257	0.135417	0.1550698
741.242	6.5	0.41048	0.3591107	0.1560261	0.1013271	0.134925	0.1566979
741.342	6.5	0.41901	0.361774	0.1512318	0.1082056	0.138017	0.1543265
741.442	6.5	0.417893	0.3680599	0.1515522	0.1074716	0.136139	0.157415
741.542	6.5	0.412206	0.3705631	0.1501778	0.10715	0.137494	0.1569888
741.642	6.5	0.405674	0.3758357	0.1553451	0.1109357	0.137489	0.1587931
741.742	6.5	0.418813	0.358142	0.155404	0.1106128	0.135621	0.1613328
741.842	6.5	0.402598	0.3553577	0.1526716	0.1049473	0.133749	0.1548555
741.942	6.5	0.42859	0.3529508	0.1522497	0.1042002	0.136099	0.1522
742.042	6.5	0.422599	0.3477618	0.1559453	0.104686	0.138133	0.1518725
742.142	6.5	0.431565	0.3501365	0.1514808	0.1064409	0.137901	0.1571917
742.242	6.5	0.436286	0.3549656	0.1555792	0.1083362	0.13748	0.1532881
742.342	6.5	0.426051	0.3587712	0.1528554	0.1096891	0.133126	0.1545857
742.442	6.5	0.436221	0.3637914	0.1529392	0.1099293	0.130612	0.1552022
742.542	6.5	0.420215	0.3670366	0.1547078	0.1150424	0.133776	0.1583795
742.642	6.5	0.420913	0.3700371	0.1536102	0.1144206	0.137674	0.1587235
742.742	6.5	0.41799	0.3714367	0.1570036	0.114207	0.135681	0.1578319
742.842	6.5	0.434926	0.3681231	0.1553522	0.116322	0.135812	0.1552356
742.942	6.5	0.426772	0.3615839	0.1578137	0.1176933	0.133866	0.1553422
743.042	6.5	0.406064	0.3520861	0.1574111	0.1177633	0.141256	0.1508195
743.142	6.5	0.389725	0.3468108	0.1555698	0.1178913	0.14013	0.1562596
743.242	6.5	0.407471	0.3446735	0.1540262	0.1187056	0.139981	0.1595301
743.342	6.5	0.388719	0.348709	0.151044	0.118865	0.139239	0.1580833
743.442	6.5	0.417198	0.3392139	0.152702	0.1183342	0.141467	0.164469
743.542	6.5	0.408693	0.3369586	0.1546417	0.1181282	0.138204	0.1607487
743.642	6.5	0.425418	0.3352355	0.1488003	0.1182198	0.139204	0.1594261
743.742	6.5	0.424259	0.3285712	0.1494312	0.1204887	0.139219	0.1579073
743.842	6.5	0.427787	0.319281	0.1495325	0.1164021	0.142062	0.1594568
743.942	6.5	0.419506	0.3166756	0.1482813	0.1174828	0.14103	0.1580914
744.042	6.5	0.418781	0.3249376	0.1495614	0.1133707	0.139743	0.1606739
744.142	6.5	0.455768	0.3375992	0.1532944	0.1067383	0.140022	0.1601993
744.242	6.5	0.435342	0.3540045	0.1484213	0.1062227	0.142758	0.1577383
744.342	6.5	0.437112	0.3518132	0.1467236	0.1066101	0.146839	0.1552882
744.442	6.5	0.425109	0.3409982	0.1497618	0.1052118	0.147006	0.1511377
744.542	6.5	0.436427	0.3405464	0.1477835	0.1080463	0.146207	0.1471494
744.642	6.5	0.419638	0.3406141	0.1490734	0.1079174	0.144106	0.1531665
744.742	6.5	0.441925	0.3462379	0.1498078	0.112578	0.145892	0.1362716
744.842	6.5	0.458944	0.346555	0.1532143	0.1070037	0.146604	0.140851
744.942	6.5	0.434705	0.3541681	0.1538061	0.1094112	0.141884	0.1475012
745.042	6.5	0.442302	0.3497967	0.1514893	0.104545	0.145075	0.149176
745.142	6.5	0.448705	0.3475654	0.1474128	0.1044782	0.140655	0.1484206
745.242	6.5	0.439159	0.348644	0.1480609	0.107552	0.144183	0.1505851
745.342	6.5	0.436096	0.3365429	0.1507967	0.1040118	0.147537	0.1510589
745.442	6.5	0.441309	0.3355373	0.1495977	0.1064931	0.14686	0.1530977
745.542	6.5	0.428344	0.3342215	0.146268	0.1050286	0.145301	0.1527973
745.642	6.5	0.440545	0.3390734	0.146744	0.1054596	0.14528	0.1555949
745.742	6.5	0.43161	0.3393818	0.1501893	0.1088763	0.1442	0.1538552
745.842	6.5	0.442558	0.3445956	0.1521481	0.106856	0.142399	0.1548119
745.942	6.5	0.420394	0.3468243	0.1589895	0.1031229	0.143743	0.1538635
746.042	6.5	0.414207	0.3491812	0.1578002	0.1050843	0.146661	0.1539369
746.142	6.5	0.434282	0.348542	0.1537138	0.1038157	0.149084	0.1513668
746.242	6.5	0.441279	0.3445494	0.1576985	0.101404	0.148947	0.1525047
746.342	6.5	0.434014	0.3391534	0.1550876	0.1025933	0.149123	0.1530367
746.442	6.5	0.42553	0.3387676	0.150211	0.1052278	0.150506	0.1534064
746.542	6.5	0.411687	0.3374632	0.1507059	0.106056	0.15118	0.1512442
746.642	6.5	0.405971	0.3403764	0.150808	0.1020117	0.147049	0.1514378
746.742	6.5	0.41357	0.3398299	0.1483014	0.1028109	0.145704	0.1524805
746.842	6.5	0.393942	0.3313244	0.150379	0.1045627	0.144505	0.1512626
746.942	6.5	0.397059	0.3289948	0.1490185	0.1025663	0.144033	0.1517945
747.042	6.5	0.415322	0.3359249	0.1474465	0.1065345	0.143455	0.1526882
747.142	6.5	0.434664	0.3097025	0.1473641	0.1018253	0.146672	0.1539772
747.242	6.5	0.455286	0.3122782	0.1464424	0.1013302	0.143071	0.1567242
747.342	6.5	0.449848	0.3134524	0.1451841	0.1072976	0.144876	0.1558574
747.442	6.5	0.441701	0.3249468	0.1450341	0.1087077	0.141655	0.1560388
747.542	6.5	0.432369	0.3505304	0.1456311	0.1086048	0.142611	0.1542114
747.642	6.5	0.429486	0.3500949	0.1479702	0.0967247	0.142428	0.152285
747.742	6.5	0.418057	0.3413921	0.1449192	0.1008416	0.139192	0.1553004
747.842	6.5	0.441002	0.3371835	0.1427186	0.1051153	0.1397	0.1580908
747.942	6.5	0.439566	0.3353744	0.1430394	0.1071984	0.140161	0.1593936

748.042	6.5	0.418897	0.3398543	0.1446309	0.1049208	0.141278	0.1608274
748.142	6.5	0.418315	0.3414821	0.1422067	0.1051642	0.141579	0.1588777
748.242	6.5	0.438385	0.3449981	0.1453102	0.1040986	0.137054	0.1621673
748.342	6.5	0.429934	0.3463436	0.1463364	0.1054763	0.136434	0.1619885
748.442	6.5	0.424658	0.3465563	0.1437169	0.1040631	0.135838	0.1671329
748.542	6.5	0.41408	0.352879	0.1442772	0.110488	0.133697	0.1615575
748.642	6.5	0.415355	0.3434536	0.1443377	0.1126937	0.136787	0.1620456
748.742	6.5	0.428266	0.3345958	0.1446048	0.1108633	0.138386	0.1639644
748.842	6.5	0.412494	0.3341337	0.1429669	0.1050622	0.13615	0.1642449
748.942	6.5	0.48451	0.3326628	0.1385862	0.1031335	0.134592	0.1605998
749.042	6.5	0.473484	0.3336307	0.1461258	0.1062329	0.134511	0.1548671
749.142	6.5	0.473262	0.3362162	0.1456226	0.1044979	0.13372	0.1596539
749.242	6.5	0.45368	0.339484	0.1449328	0.1035785	0.1377	0.1583331
749.342	6.5	0.480714	0.3402998	0.138364	0.1052871	0.139519	0.1555826
749.442	6.5	0.450642	0.3431951	0.1424927	0.1005423	0.135217	0.1535762
749.542	6.5	0.478225	0.34	0.1451692	0.1062165	0.133865	0.1589722
749.642	6.5	0.478599	0.3355439	0.1461037	0.1014192	0.132997	0.1605092
749.742	6.5	0.452521	0.3349789	0.1428744	0.1028117	0.133598	0.161085
749.842	6.5	0.419181	0.3339034	0.1410165	0.1043415	0.12972	0.1596992
749.942	6.5	0.416263	0.3360218	0.1428832	0.1001389	0.128998	0.1498806
750.042	6.5	0.409566	0.3415741	0.1398271	0.09852672	0.131374	0.154681
750.142	6.5	0.415053	0.3281603	0.1461198	0.09972674	0.135275	0.1585606
750.242	6.5	0.416243	0.3306138	0.1412429	0.1020875	0.132832	0.1563604
750.342	6.5	0.46875	0.3325572	0.1451623	0.1003385	0.132558	0.1556137
750.442	6.5	0.426137	0.3173544	0.1509615	0.09995777	0.131114	0.1572696
750.542	6.5	0.446873	0.3118888	0.1436079	0.1020983	0.130928	0.1603454
750.642	6.5	0.434435	0.3063709	0.1432585	0.10565	0.132919	0.1580548
750.742	6.5	0.411336	0.3141623	0.1421989	0.1082595	0.128962	0.1550458
750.842	6.5	0.426881	0.3386645	0.1478447	0.1079127	0.127686	0.1543725
750.942	6.5	0.445642	0.3522496	0.151487	0.1098155	0.129933	0.155475
751.042	6.5	0.412006	0.344061	0.1496888	0.1050088	0.130749	0.1556779
751.142	6.5	0.418023	0.3334441	0.1429949	0.1037068	0.131549	0.1602789
751.242	6.5	0.431501	0.3319673	0.1432877	0.1078719	0.132108	0.1578328
751.342	6.5	0.422138	0.3332871	0.1434509	0.1063009	0.13128	0.1538935
751.442	6.5	0.429665	0.3390246	0.1490197	0.1081559	0.13253	0.1593229
751.542	6.5	0.434133	0.3405624	0.1501929	0.1065304	0.132172	0.1560854
751.642	6.5	0.44908	0.3424969	0.1461261	0.107619	0.136378	0.1532293
751.742	6.5	0.431664	0.3443306	0.1460053	0.1117932	0.136989	0.1562186
751.842	6.5	0.410101	0.3497841	0.1476107	0.112843	0.138345	0.156869
751.942	6.5	0.415317	0.3457348	0.1463009	0.1125452	0.136919	0.152463
752.042	6.5	0.426529	0.334733	0.1484712	0.1129893	0.13085	0.1516337
752.142	6.5	0.429188	0.3348072	0.1495816	0.1172427	0.132078	0.1413578
752.242	6.5	0.420821	0.3337657	0.1454001	0.1169383	0.135473	0.1468673
752.342	6.5	0.393038	0.3338849	0.1456214	0.1176121	0.13702	0.1512527
752.442	6.5	0.422989	0.3350761	0.1520891	0.1190646	0.138182	0.1590472
752.542	6.5	0.420343	0.3386684	0.1497453	0.1186423	0.13867	0.1593181
752.642	6.5	0.425749	0.3393247	0.1514181	0.1162569	0.142555	0.1559297
752.742	6.5	0.425276	0.3413672	0.1537929	0.1150189	0.141292	0.1541976
752.842	6.5	0.427551	0.3394021	0.1439821	0.1177549	0.142181	0.1522939
752.942	6.5	0.424914	0.3375873	0.147785	0.1207949	0.140383	0.1547002
753.042	6.5	0.418923	0.3332525	0.147675	0.1167803	0.142498	0.1519811
753.142	6.5	0.425783	0.3318463	0.1484774	0.1174935	0.138634	0.1531392
753.242	6.5	0.418419	0.3348323	0.1519506	0.118015	0.139568	0.1532266
753.342	6.5	0.423016	0.3403693	0.1501607	0.1113316	0.141218	0.1537853
753.442	6.5	0.42838	0.3317696	0.1496854	0.1083609	0.140852	0.156312
753.542	6.5	0.42685	0.3235053	0.1492519	0.1060522	0.141335	0.1568202
753.642	6.5	0.448568	0.3242917	0.1519094	0.1058833	0.140287	0.1569582
753.742	6.5	0.435376	0.3241686	0.1524073	0.106016	0.143379	0.1575413
753.842	6.5	0.44129	0.3082224	0.1506151	0.1078895	0.144523	0.1528111
753.942	6.5	0.459393	0.301335	0.1524314	0.1094506	0.143626	0.1509755
754.042	6.5	0.433555	0.3088678	0.1545988	0.1078424	0.144175	0.1509959
754.142	6.5	0.429775	0.3228385	0.1513646	0.1084133	0.145257	0.1497457
754.242	6.5	0.426002	0.3501599	0.155155	0.1082016	0.147167	0.1528162
754.342	6.5	0.431648	0.3478707	0.1524078	0.106517	0.145751	0.1536059
754.442	6.5	0.413878	0.3353192	0.1514589	0.1036791	0.147042	0.1547968
754.542	6.5	0.440766	0.3343343	0.1517119	0.1022722	0.147423	0.1567308
754.642	6.5	0.423082	0.3329211	0.1517498	0.1029976	0.148988	0.154232
754.742	6.5	0.424101	0.3400979	0.1558654	0.1054905	0.15064	0.157484
754.842	6.5	0.464568	0.3384419	0.1512297	0.1010126	0.148902	0.156099
754.942	6.5	0.467138	0.343893	0.155307	0.1037897	0.147449	0.1558417
755.042	6.5	0.453091	0.3439892	0.1565993	0.1019196	0.14794	0.1520077
755.142	6.5	0.44487	0.3472408	0.1555593	0.1015377	0.145626	0.1532612
755.242	6.5	0.441767	0.3527359	0.1540449	0.1025924	0.142807	0.1578204
755.342	6.5	0.447364	0.3396008	0.1516114	0.1041628	0.144529	0.1560168
755.442	6.5	0.440028	0.3363711	0.1535927	0.1048919	0.147458	0.1591661
755.542	6.5	0.436067	0.3334805	0.1544769	0.09963264	0.148946	0.1558744
755.642	6.5	0.446938	0.3337419	0.1520722	0.1027546	0.148206	0.1538222
755.742	6.5	0.432343	0.334339	0.1533289	0.1042235	0.151476	0.1516804
755.842	6.5	0.421097	0.3387635	0.1510036	0.1034491	0.151156	0.1526516
755.942	6.5	0.446654	0.3383432	0.1505542	0.0986402	0.145108	0.1524622
756.042	6.5	0.414395	0.3400946	0.1494775	0.1027193	0.143274	0.1536243
756.142	6.5	0.422878	0.3404913	0.1537772	0.09883178	0.14064	0.153614
756.242	6.5	0.441628	0.3393317	0.1499361	0.1025808	0.140718	0.1533036
756.342	6.5	0.455268	0.3337295	0.1458032	0.1018517	0.140475	0.1562406
756.442	6.5	0.460789	0.3342845	0.1466275	0.09817115	0.142117	0.1538992
756.542	6.5	0.465589	0.3357982	0.1479104	0.1055294	0.14099	0.1458544
756.642	6.5	0.464456	0.3385395	0.1489861	0.1072758	0.138941	0.1501247
756.742	6.5	0.450521	0.3402023	0.1512294	0.1050541	0.138151	0.1406983
756.842	6.5	0.440999	0.3263199	0.1531751	0.09668364	0.138869	0.1431444
756.942	6.5	0.421276	0.3235675	0.1544691	0.09496413	0.137681	0.1463312
757.042	6.5	0.427486	0.336095	0.1504772	0.102565	0.137737	0.1449551
757.142	6.5	0.42821	0.3123578	0.1457143	0.1040499	0.139556	0.1447973
757.242	6.5	0.416584	0.309063	0.1465314	0.1007066	0.14126	0.1478997
757.342	6.5	0.410981	0.3061593	0.1501497	0.1027489	0.142276	0.149646
757.442	6.5	0.41915	0.3182313	0.1494095	0.1003352	0.137695	0.1545019

757.542	6.5	0.430651	0.3387209	0.1453143	0.1016631	0.136476	0.1538577
757.642	6.5	0.414446	0.3488795	0.1447452	0.09689254	0.136054	0.1582638
757.742	6.5	0.39932	0.3366513	0.1479613	0.1032196	0.137852	0.1566964
757.842	6.5	0.410959	0.3349072	0.1492213	0.1027544	0.138492	0.1570904
757.942	6.5	0.418285	0.3339549	0.1565686	0.1056319	0.136889	0.1538937
758.042	6.5	0.413386	0.3378869	0.1569224	0.1020524	0.137495	0.1532244
758.142	6.5	0.414423	0.3389524	0.1512403	0.1011735	0.135544	0.149748
758.242	6.5	0.431878	0.3418373	0.1548185	0.102438	0.134352	0.1489459
758.342	6.5	0.420101	0.3459618	0.1529148	0.1018956	0.135729	0.1498154
758.442	6.5	0.425634	0.3466834	0.1470355	0.1017688	0.137821	0.1506183
758.542	6.5	0.418251	0.3534404	0.1485016	0.1043988	0.134444	0.1499965
758.642	6.5	0.404013	0.343308	0.1474948	0.09929503	0.133701	0.1499991
758.742	6.5	0.432233	0.3394729	0.1462365	0.1034004	0.132446	0.1538146
758.842	6.5	0.419348	0.3390927	0.1495441	0.09924591	0.13267	0.1544516
758.942	6.5	0.446275	0.3393773	0.148778	0.1005338	0.129443	0.1569027
759.042	6.5	0.418661	0.3422828	0.1445165	0.102646	0.133848	0.1576157
759.142	6.5	0.408521	0.3447373	0.1430691	0.09976499	0.132183	0.1562074
759.242	6.5	0.395745	0.3483572	0.1424547	0.09542859	0.133519	0.15911
759.342	6.5	0.41263	0.3493948	0.1409522	0.09794356	0.134341	0.1562493
759.442	6.5	0.402079	0.3519018	0.1430485	0.1004308	0.131658	0.1586668
759.542	6.5	0.425424	0.3489507	0.1443946	0.1004303	0.131398	0.1568899
759.642	6.5	0.416146	0.3428799	0.1489741	0.09771302	0.132384	0.1547048
759.742	6.5	0.417741	0.3407227	0.1493246	0.09839737	0.130235	0.1560902
759.842	6.5	0.419404	0.3392596	0.1443849	0.1037864	0.132645	0.1576521
759.942	6.5	0.412753	0.3406951	0.1453129	0.1063706	0.12981	0.159291
760.042	6.5	0.414031	0.3423728	0.1448946	0.1066716	0.126344	0.1632695
760.142	6.5	0.41213	0.3278146	0.1418115	0.1094006	0.130013	0.15953
760.242	6.5	0.407074	0.3224477	0.1452601	0.1060515	0.131342	0.1604119
760.342	6.5	0.402464	0.3330111	0.1492774	0.1028055	0.134917	0.1595947
760.442	6.5	0.432321	0.3119394	0.1424484	0.1058545	0.133564	0.1629346
760.542	6.5	0.421853	0.3065075	0.1438722	0.1057852	0.132482	0.1626284
760.642	6.5	0.428415	0.3060507	0.1450169	0.1065933	0.133354	0.1634621
760.742	6.5	0.420905	0.3142204	0.1483835	0.104818	0.135443	0.1615805
760.842	6.5	0.433116	0.3288326	0.1458109	0.1064215	0.137021	0.1638404
760.942	6.5	0.416297	0.352477	0.1402439	0.1088193	0.13687	0.1638771
761.042	6.5	0.405714	0.348006	0.1483239	0.115053	0.139184	0.156561
761.142	6.5	0.42553	0.3428084	0.1476205	0.112041	0.136707	0.1592473
761.242	6.5	0.413051	0.3442774	0.1464442	0.1124924	0.131853	0.1566004
761.342	6.5	0.420008	0.3429476	0.1430252	0.1151687	0.129564	0.1569423
761.442	6.5	0.420299	0.3473054	0.1428058	0.1154483	0.133084	0.1517636
761.542	6.5	0.387333	0.3458399	0.1450425	0.1144821	0.137671	0.1578811
761.642	6.5	0.394568	0.3490551	0.1470329	0.1161906	0.139062	0.155141
761.742	6.5	0.391772	0.3494979	0.1443257	0.1182952	0.136361	0.1576275
761.842	6.5	0.39464	0.351501	0.1423133	0.1180302	0.138651	0.1612972
761.942	6.5	0.414997	0.3495363	0.136123	0.1153029	0.138402	0.1540683
762.042	6.5	0.401353	0.3409904	0.1411192	0.114957	0.138029	0.1528171
762.142	6.5	0.426711	0.3405997	0.1437778	0.115261	0.136907	0.1558184
762.242	6.5	0.425029	0.3429701	0.1444585	0.1153146	0.140142	0.1536455
762.342	6.5	0.424122	0.3433051	0.141739	0.1159327	0.138569	0.152425
762.442	6.5	0.413724	0.3430357	0.1505729	0.1180084	0.13781	0.1525234
762.542	6.5	0.42193	0.3461385	0.1445975	0.1135702	0.139517	0.151669
762.642	6.5	0.42528	0.3483431	0.1438025	0.1135855	0.141946	0.1544102
762.742	6.5	0.422338	0.3497802	0.1388407	0.1103567	0.14157	0.150465
762.842	6.5	0.432609	0.3504243	0.1441517	0.1098338	0.140555	0.1489609
762.942	6.5	0.419632	0.3455954	0.147645	0.1097004	0.143786	0.1511226
763.042	6.5	0.430767	0.3404264	0.1488066	0.1100077	0.142672	0.1527797
763.142	6.5	0.419543	0.3368368	0.1430235	0.110495	0.143736	0.1572337
763.242	6.5	0.437529	0.3389719	0.1429097	0.1113411	0.14567	0.1580211
763.342	6.5	0.433224	0.3442466	0.1437552	0.1108222	0.14543	0.1504387
763.442	6.5	0.438067	0.3320266	0.1464572	0.1088862	0.147543	0.1543146
763.542	6.5	0.445941	0.3204384	0.1480902	0.1076347	0.147623	0.150162
763.642	6.5	0.414942	0.3256502	0.146974	0.1053081	0.145529	0.1463485
763.742	6.5	0.433071	0.3239623	0.1481388	0.1064509	0.146375	0.1492718
763.842	6.5	0.423939	0.3085105	0.1466779	0.1061727	0.144331	0.1507775
763.942	6.5	0.427355	0.3051181	0.1470158	0.1065539	0.145022	0.1502732
764.042	6.5	0.433482	0.3115374	0.146825	0.1024991	0.146625	0.1466539
764.142	6.5	0.448346	0.3222339	0.1490834	0.10342	0.145448	0.1390719
764.242	6.5	0.444897	0.3452318	0.1489718	0.1047676	0.143372	0.1425982
764.342	6.5	0.433861	0.3481749	0.1472368	0.1058109	0.143271	0.1474251
764.442	6.5	0.407048	0.3369157	0.1506144	0.1031285	0.141367	0.1557998
764.542	6.5	0.422718	0.3385351	0.150858	0.10512	0.142708	0.1565329
764.642	6.5	0.436863	0.335076	0.1516351	0.1030922	0.144729	0.1537307
764.742	6.5	0.436231	0.3424184	0.1507158	0.09925469	0.146732	0.1543298
764.842	6.5	0.435823	0.3399051	0.1416509	0.1018207	0.143694	0.1502995
764.942	6.5	0.428967	0.346244	0.1463147	0.1051123	0.147925	0.1514043
765.042	6.5	0.40269	0.3465848	0.1483527	0.1064819	0.146284	0.1487268
765.142	6.5	0.415783	0.3452521	0.1497599	0.09951132	0.143643	0.1509575
765.242	6.5	0.402127	0.3533999	0.1525281	0.103798	0.140601	0.1525833
765.342	6.5	0.391331	0.3430685	0.1488994	0.1019307	0.139138	0.1518879
765.442	6.5	0.384257	0.3404901	0.1488017	0.1044745	0.140308	0.1521035
765.542	6.5	0.420433	0.3365045	0.1482406	0.1067667	0.138128	0.1514122
765.642	6.5	0.444213	0.3378079	0.1510439	0.1003452	0.140068	0.1521982
765.742	6.5	0.441999	0.3363459	0.152277	0.1059796	0.139406	0.1569462
765.842	6.5	0.451519	0.3383332	0.1505287	0.1096098	0.137687	0.1503285
765.942	6.5	0.423564	0.3411542	0.1493253	0.1083407	0.135964	0.1485053
766.042	6.5	0.427198	0.3408128	0.1527477	0.1003358	0.139029	0.1500615
766.142	6.5	0.419862	0.3440114	0.1487893	0.09627431	0.136219	0.1504276
766.242	6.5	0.416445	0.3422954	0.1547694	0.1043195	0.134566	0.153365
766.342	6.5	0.43205	0.3367778	0.1515534	0.1047578	0.135934	0.1549429
766.442	6.5	0.422012	0.3357385	0.1518332	0.1022156	0.137123	0.1542113
766.542	6.5	0.399808	0.3338655	0.151335	0.1025969	0.142496	0.1545481
766.642	6.5	0.403664	0.3372988	0.1507635	0.1064216	0.14028	0.1526899
766.742	6.5	0.418563	0.3344892	0.1533178	0.102564	0.139377	0.1531193
766.842	6.5	0.411423	0.3226559	0.1510049	0.100613	0.138919	0.1503994
766.942	6.5	0.401828	0.3224522	0.1545733	0.1035423	0.136872	0.1523189

767.042	6.5	0.409782	0.3336186	0.1545906	0.1073663	0.140736	0.1524424
767.142	6.5	0.407001	0.3097577	0.151832	0.1098546	0.1401	0.155388
767.242	6.5	0.411475	0.3047427	0.1540789	0.1051583	0.139698	0.1572124
767.342	6.5	0.408924	0.3053052	0.1524315	0.1004309	0.136341	0.1563636
767.442	6.5	0.435201	0.3131236	0.1543597	0.1039051	0.136186	0.1613121
767.542	6.5	0.413332	0.3334364	0.1554781	0.1037789	0.135459	0.1565665
767.642	6.5	0.413254	0.3466617	0.1508104	0.1019476	0.138659	0.15839
767.742	6.5	0.412143	0.3393949	0.15136	0.105215	0.137619	0.1582151
767.842	6.5	0.398902	0.3381908	0.1503236	0.1028629	0.137992	0.1583464
767.942	6.5	0.440409	0.3357021	0.1494373	0.1051176	0.135749	0.1565381
768.042	6.5	0.418671	0.3388649	0.1503429	0.1019412	0.136436	0.1577153
768.142	6.5	0.45683	0.3391296	0.153511	0.09951957	0.134884	0.1567243
768.242	6.5	0.431327	0.3419427	0.1489171	0.1027821	0.132642	0.1564222
768.342	6.5	0.424548	0.3466329	0.1455153	0.1014068	0.132134	0.1569469
768.442	6.5	0.403364	0.3467078	0.1478547	0.0973871	0.138108	0.1574171
768.542	6.5	0.421132	0.3552993	0.1489233	0.09849271	0.135012	0.1505681
768.642	6.5	0.412081	0.3446294	0.1500912	0.09859588	0.13259	0.1529851
768.742	6.5	0.407945	0.3397141	0.1515762	0.09896065	0.132684	0.1366913
768.842	6.5	0.411651	0.336704	0.152767	0.1002629	0.132955	0.1408104
768.942	6.5	0.40743	0.3359082	0.1564513	0.1004538	0.133906	0.1462315
769.042	6.5	0.41901	0.3364321	0.1485087	0.1053066	0.134914	0.1465779
769.142	6.5	0.412952	0.3377216	0.1423047	0.1040277	0.133208	0.1460936
769.242	6.5	0.407679	0.3424786	0.1441998	0.1049861	0.129872	0.1479919
769.342	6.5	0.402222	0.3408316	0.1502961	0.1090139	0.131916	0.1506755
769.442	6.5	0.405821	0.3453462	0.1510646	0.1060862	0.134086	0.153304
769.542	6.5	0.402241	0.3433875	0.148126	0.103927	0.134874	0.1517479
769.642	6.5	0.418829	0.3388072	0.1461011	0.1059003	0.135737	0.1557603
769.742	6.5	0.417505	0.3368249	0.1470887	0.1045358	0.133672	0.1538863
769.842	6.5	0.422969	0.334272	0.1498143	0.1088883	0.133155	0.1543915
769.942	6.5	0.413466	0.3391336	0.1594768	0.110045	0.133138	0.1530945
770.042	6.5	0.418727	0.3414769	0.1579137	0.1095093	0.13339	0.1567715
770.142	6.5	0.420595	0.3233813	0.1530861	0.1095649	0.136795	0.154582
770.242	6.5	0.407365	0.3208253	0.152294	0.1146528	0.138913	0.1555667
770.342	6.5	0.422357	0.3278191	0.150873	0.1121029	0.139714	0.1549692
770.442	6.5	0.404729	0.3119409	0.1451842	0.1124609	0.136114	0.1551339
770.542	6.5	0.412386	0.3070139	0.1483381	0.1135445	0.132034	0.1529605
770.642	6.5	0.409254	0.3057539	0.1462286	0.1134677	0.134748	0.1531619
770.742	6.5	0.389622	0.3108215	0.1473042	0.1138114	0.137604	0.1546562
770.842	6.5	0.381479	0.3258063	0.149186	0.1174102	0.136215	0.1533512
770.942	6.5	0.393937	0.3504147	0.1475076	0.1165826	0.136503	0.1546489
771.042	6.5	0.380369	0.3447841	0.1460071	0.1165023	0.137159	0.1534175
771.142	6.5	0.412886	0.3363226	0.1462851	0.1164485	0.139204	0.1537956
771.242	6.5	0.398569	0.3339547	0.14431	0.1158958	0.137387	0.1599253
771.342	6.5	0.42601	0.3350284	0.1425739	0.1179733	0.13535	0.1567237
771.442	6.5	0.413228	0.3379443	0.1420718	0.1190786	0.137269	0.1599835
771.542	6.5	0.420878	0.3387167	0.1424786	0.1156381	0.138673	0.1576987
771.642	6.5	0.416687	0.3412677	0.1458417	0.1172535	0.137005	0.1554732
771.742	6.5	0.408735	0.3452238	0.1424042	0.1129022	0.136379	0.1592711
771.842	6.5	0.420485	0.3532073	0.1424523	0.1070774	0.138958	0.1596652
771.942	6.5	0.420076	0.3491689	0.1416978	0.1066483	0.138153	0.1623168
772.042	6.5	0.423984	0.3391014	0.1419543	0.1077302	0.13707	0.1607407
772.142	6.5	0.419973	0.3371747	0.1394611	0.1068903	0.140657	0.1593352
772.242	6.5	0.429581	0.3347897	0.147703	0.1106664	0.140499	0.1618369
772.342	6.5	0.413351	0.3347493	0.1440275	0.1093223	0.139991	0.1627166
772.442	6.5	0.431065	0.3359868	0.1392082	0.1116161	0.140883	0.1675176
772.542	6.5	0.443892	0.3395582	0.1385856	0.108186	0.140543	0.1639785
772.642	6.5	0.424995	0.3394625	0.14012	0.110155	0.143576	0.1648903
772.742	6.5	0.434199	0.344494	0.1427624	0.1048344	0.145996	0.1659546
772.842	6.5	0.424544	0.3432668	0.14122	0.1055662	0.14186	0.165552
772.942	6.5	0.424562	0.3410304	0.1395739	0.107937	0.144146	0.1619288
773.042	6.5	0.414145	0.3369739	0.144538	0.1029455	0.140939	0.1585517
773.142	6.5	0.426057	0.3346957	0.1427701	0.1028815	0.145883	0.1617693
773.242	6.5	0.416091	0.3375273	0.1429942	0.1011373	0.145886	0.1564088
773.342	6.5	0.431663	0.3429443	0.1392266	0.1011067	0.145629	0.1542993
773.442	6.5	0.42635	0.3304309	0.1420991	0.1035852	0.145761	0.1543489
773.542	6.5	0.428572	0.3181061	0.1430393	0.1020395	0.14502	0.1580585
773.642	6.5	0.403553	0.3230114	0.1436941	0.09982405	0.141443	0.1573298
773.742	6.5	0.422323	0.3236183	0.1400774	0.100904	0.137618	0.1583264
773.842	6.5	0.428978	0.3067299	0.1384943	0.0992352	0.140123	0.1595443
773.942	6.5	0.434641	0.304482	0.1424399	0.0997088	0.142456	0.1528165
774.042	6.5	0.424962	0.3076569	0.1387384	0.1030104	0.144482	0.1556785
774.142	6.5	0.419071	0.3191922	0.1469779	0.10609	0.146379	0.1575441
774.242	6.5	0.401715	0.3442433	0.1396591	0.1065373	0.146107	0.1554152
774.342	6.5	0.409176	0.349016	0.1443813	0.09908142	0.145459	0.1531141
774.442	6.5	0.397596	0.336229	0.1474257	0.1019545	0.138641	0.1534574
774.542	6.5	0.389413	0.3345266	0.1433174	0.1031501	0.138663	0.1531627
774.642	6.5	0.387162	0.3309755	0.1431349	0.1046967	0.139982	0.1536008
774.742	6.5	0.404751	0.3351457	0.1416446	0.1081615	0.139589	0.1542608
774.842	6.5	0.430358	0.3350829	0.1468298	0.1005122	0.140104	0.1531647
774.942	6.5	0.437546	0.342375	0.1509975	0.1039349	0.137523	0.1542593
775.042	6.5	0.448577	0.3436825	0.1507535	0.1097988	0.139027	0.1559233
775.142	6.5	0.432484	0.3471723	0.1437081	0.1106798	0.137159	0.1566196
775.242	6.5	0.429424	0.353403	0.1439474	0.1030771	0.139216	0.1589114
775.342	6.5	0.41882	0.3413929	0.1446176	0.09628357	0.142044	0.1528986
775.442	6.5	0.405175	0.3372666	0.148911	0.101186	0.136387	0.1567799
775.542	6.5	0.422318	0.3338987	0.1506002	0.1046587	0.135866	0.1526356
775.642	6.5	0.42203	0.3348704	0.1477077	0.10453	0.137696	0.1513956
775.742	6.5	0.399813	0.333697	0.1477049	0.1037156	0.13876	0.1568852
775.842	6.5	0.403028	0.336623	0.1467171	0.1066751	0.136686	0.155808
775.942	6.5	0.419238	0.3371836	0.1441402	0.10245	0.134134	0.1539757
776.042	6.5	0.414765	0.3390396	0.1459726	0.1026595	0.135121	0.1533251
776.142	6.5	0.410684	0.3403664	0.1482326	0.1038933	0.133363	0.1420495
776.242	6.5	0.407562	0.3387859	0.1460662	0.1075712	0.134253	0.1415707
776.342	6.5	0.409113	0.3344062	0.1459459	0.1117773	0.136372	0.1494253
776.442	6.5	0.419499	0.3311124	0.1485059	0.1095499	0.133784	0.1569862

776.542	6.5	0.415785	0.3326092	0.147056	0.1026904	0.132092	0.1561573
776.642	6.5	0.442818	0.3373592	0.1464366	0.107069	0.132137	0.1535555
776.742	6.5	0.426286	0.3348623	0.1486462	0.108647	0.131266	0.1561974
776.842	6.5	0.422192	0.320121	0.1386542	0.1049819	0.134176	0.153499
776.942	6.5	0.430701	0.3154451	0.1459442	0.1056602	0.132253	0.1532575
777.042	6.5	0.419598	0.3316025	0.1472318	0.1048107	0.128144	0.1486876
777.142	6.5	0.418911	0.308925	0.1472672	0.1035315	0.130311	0.1491003
777.242	6.5	0.418937	0.3103786	0.1518622	0.1041906	0.128223	0.15316
777.342	6.5	0.433737	0.3033414	0.1504001	0.09934158	0.129077	0.153574
777.442	6.5	0.430875	0.3135886	0.1479395	0.1022888	0.123521	0.1536255
777.542	6.5	0.414584	0.3361387	0.1456973	0.1038422	0.126766	0.1549887
777.642	6.5	0.400422	0.3489496	0.1495698	0.09762605	0.130392	0.1563792
777.742	6.5	0.410025	0.339592	0.1507803	0.09748916	0.129026	0.157287
777.842	6.5	0.406667	0.3351416	0.1502597	0.096811	0.128101	0.1516364
777.942	6.5	0.395809	0.331843	0.151529	0.0980077	0.12742	0.1495752
778.042	6.5	0.41635	0.3335227	0.1529825	0.1012157	0.130288	0.1502096
778.142	6.5	0.403894	0.3350354	0.1500575	0.1007063	0.128384	0.1491902
778.242	6.5	0.414783	0.3387175	0.1568191	0.1046849	0.130592	0.1523397
778.342	6.5	0.409861	0.3417179	0.152523	0.1061009	0.130458	0.1532057
778.442	6.5	0.397217	0.3436762	0.1512633	0.1078125	0.12979	0.1538072
778.542	6.5	0.4138	0.3534606	0.1527472	0.1072205	0.130712	0.1552816
778.642	6.5	0.447	0.343597	0.1527463	0.1083438	0.131375	0.1546027
778.742	6.5	0.429912	0.3355613	0.1566938	0.1032643	0.130093	0.1553788
778.842	6.5	0.422408	0.3327764	0.1505633	0.1048173	0.130754	0.1524139
778.942	6.5	0.416177	0.3319138	0.154298	0.1061176	0.129709	0.1515687
779.042	6.5	0.418786	0.3331205	0.1555897	0.1040901	0.128595	0.1503587
779.142	6.5	0.412689	0.3342713	0.1539171	0.1082849	0.130252	0.1538651
779.242	6.5	0.40282	0.3366569	0.1527034	0.1094208	0.131091	0.1524592
779.342	6.5	0.425122	0.3365798	0.1524553	0.1112592	0.132129	0.1530892
779.487	7	0.412874	0.3357334	0.1549213	0.1152504	0.132399	0.1588331
779.587	7	0.410181	0.3344685	0.154169	0.1154648	0.131528	0.1538214
779.687	7	0.40828	0.3318515	0.1507195	0.115833	0.131801	0.1541005
779.787	7	0.374785	0.3343867	0.1503143	0.1170914	0.129403	0.1542193
779.887	7	0.403114	0.3366718	0.1489871	0.1196335	0.13198	0.1542254
779.987	7	0.392319	0.3229873	0.1458776	0.1192489	0.134552	0.1539465
780.087	7	0.407659	0.3190081	0.148205	0.1164558	0.13473	0.1554765
780.187	7	0.393357	0.3278937	0.1514038	0.1167463	0.135778	0.1554318
780.287	7	0.424412	0.3010888	0.147273	0.1147505	0.133808	0.156011
780.387	7	0.403578	0.3076037	0.1418872	0.1179267	0.136913	0.1558715
780.487	7	0.413543	0.307405	0.147404	0.1165343	0.135573	0.1532803
780.587	7	0.408956	0.3173888	0.1462597	0.1159588	0.135196	0.1464297
780.687	7	0.408428	0.3408221	0.1517412	0.1158213	0.135011	0.1498506
780.787	7	0.410816	0.3487603	0.1524786	0.1091871	0.136394	0.1373602
780.887	7	0.415653	0.3494467	0.1461526	0.1099105	0.134133	0.1442144
780.987	7	0.419648	0.3487016	0.1444449	0.1068578	0.135065	0.1494545
781.087	7	0.418254	0.3489306	0.1493574	0.104473	0.137408	0.1481762
781.187	7	0.417833	0.356535	0.1500947	0.1098052	0.138598	0.1486813
781.287	7	0.420186	0.3602625	0.1470544	0.1081106	0.138612	0.1500508
781.387	7	0.440453	0.3689505	0.1451434	0.1103042	0.138825	0.1510097
781.487	7	0.433061	0.3645492	0.1496284	0.1053259	0.139369	0.1517716
781.587	7	0.437451	0.3469248	0.1543428	0.1064547	0.142678	0.1509541
781.687	7	0.424774	0.3508317	0.1600038	0.1079335	0.142319	0.1522003
781.787	7	0.420046	0.3487543	0.1571181	0.1069247	0.142534	0.1509881
781.887	7	0.411432	0.3492976	0.1576172	0.1068586	0.1424	0.1548147
781.987	7	0.423267	0.3528427	0.1533613	0.1025039	0.145014	0.1553236
782.087	7	0.426624	0.357978	0.1500142	0.103512	0.144783	0.1549652
782.187	7	0.437025	0.3622244	0.1488928	0.09786417	0.143762	0.152067
782.287	7	0.428959	0.3672423	0.1466954	0.1026484	0.141249	0.1537934
782.387	7	0.431894	0.3719206	0.1486951	0.10332	0.144994	0.1513785
782.487	7	0.424837	0.3764597	0.1523239	0.1002932	0.146362	0.1545032
782.587	7	0.437115	0.3761777	0.1477753	0.1000195	0.145629	0.1535289
782.687	7	0.437527	0.3723679	0.14659	0.1023011	0.143975	0.1552548
782.787	7	0.422672	0.3632748	0.1438418	0.09916221	0.144511	0.1548844
782.887	7	0.421984	0.3592301	0.1425479	0.09664731	0.142901	0.1543769
782.987	7	0.417023	0.3530364	0.1416878	0.1009224	0.140655	0.1564436
783.087	7	0.406072	0.3530429	0.1408346	0.1014892	0.141324	0.1560069
783.187	7	0.407844	0.3294587	0.1476395	0.09495771	0.142909	0.1566226
783.287	7	0.400762	0.3243566	0.1466643	0.1009456	0.143121	0.1599636
783.387	7	0.404122	0.3289251	0.1442819	0.1006423	0.141462	0.1566339
783.487	7	0.418524	0.3164767	0.1422406	0.1049972	0.14672	0.1584616
783.587	7	0.439364	0.3121414	0.1426023	0.1035363	0.144409	0.1531888
783.687	7	0.437055	0.3097868	0.139472	0.0995719	0.141449	0.1544487
783.787	7	0.429991	0.3185177	0.1466504	0.1099847	0.139588	0.1592419
783.887	7	0.419791	0.3351129	0.1409156	0.1101837	0.140732	0.1601064
783.987	7	0.4185	0.3486879	0.1382936	0.1044146	0.141315	0.161497
784.087	7	0.401598	0.3518074	0.1384892	0.09258283	0.141202	0.1628081
784.187	7	0.420504	0.3472428	0.1377449	0.09994092	0.14115	0.1621758
784.287	7	0.425391	0.3497328	0.1378812	0.1041436	0.139095	0.16474
784.387	7	0.398254	0.3516771	0.1350762	0.1032981	0.138409	0.1641142
784.487	7	0.40145	0.3578889	0.1421686	0.1014657	0.138083	0.1689417
784.587	7	0.421502	0.3625193	0.1384047	0.1025092	0.140905	0.1648387
784.687	7	0.418964	0.3677942	0.1386363	0.1019623	0.140974	0.1660015
784.787	7	0.414278	0.3536614	0.1381513	0.1012651	0.138208	0.1666057
784.887	7	0.432946	0.3549754	0.1430531	0.1048926	0.139903	0.1632796
784.987	7	0.415423	0.3509894	0.1432834	0.1073263	0.139108	0.1587423
785.087	7	0.414321	0.3471822	0.1420234	0.109843	0.140298	0.1543411
785.187	7	0.437024	0.3497813	0.141051	0.1049204	0.136399	0.157815
785.287	7	0.429449	0.3549927	0.1412052	0.1041527	0.135854	0.1535545
785.387	7	0.416358	0.3584634	0.1380325	0.1045822	0.137522	0.1535227
785.487	7	0.42421	0.3609686	0.1423007	0.102515	0.137589	0.1515885
785.587	7	0.419165	0.3657436	0.1373455	0.105322	0.139393	0.1545609
785.687	7	0.41808	0.3657573	0.1450632	0.1040374	0.13668	0.1542712
785.787	7	0.422586	0.3675703	0.1441076	0.1037171	0.13608	0.1559929
785.887	7	0.438722	0.3623557	0.1410146	0.1002731	0.135868	0.1560946
785.987	7	0.425567	0.3545607	0.1380647	0.1001197	0.134141	0.1497557

786.087	7	0.409721	0.3475925	0.144332	0.1017101	0.135023	0.1534015
786.187	7	0.393553	0.3449799	0.1496411	0.0990521	0.135813	0.1522403
786.287	7	0.404793	0.3441771	0.1515897	0.09911615	0.133663	0.1513587
786.387	7	0.395658	0.3388201	0.1429127	0.09738994	0.133514	0.1519279
786.487	7	0.425016	0.3285732	0.1437538	0.09819879	0.133383	0.1530034
786.587	7	0.400667	0.3308484	0.1461698	0.09689142	0.131874	0.1557441
786.687	7	0.400594	0.335557	0.1514767	0.09774404	0.128671	0.1537509
786.787	7	0.416451	0.3121608	0.1517737	0.1018798	0.128817	0.1527804
786.887	7	0.392533	0.3100056	0.1516979	0.1008497	0.128667	0.1518545
786.987	7	0.395738	0.3065461	0.150393	0.1005596	0.133218	0.1501689
787.087	7	0.401268	0.3228706	0.1490003	0.1022553	0.133479	0.1547702
787.187	7	0.397108	0.3411734	0.1477173	0.1043908	0.130269	0.1579383
787.287	7	0.410802	0.3478283	0.1492467	0.09919017	0.131614	0.1585877
787.387	7	0.413461	0.3402573	0.148134	0.09818342	0.133907	0.1536181
787.487	7	0.425937	0.3396881	0.1485511	0.09606419	0.132739	0.1560192
787.587	7	0.40998	0.3393939	0.1515404	0.09957553	0.135574	0.150781
787.687	7	0.420294	0.3461335	0.14898	0.102093	0.133389	0.1501902
787.787	7	0.410063	0.3479505	0.1507124	0.1067032	0.130905	0.1548257
787.887	7	0.401845	0.3558907	0.1486944	0.1091468	0.131695	0.1556849
787.987	7	0.41761	0.3505314	0.1488071	0.10859	0.132053	0.1524695
788.087	7	0.408303	0.351804	0.1474691	0.1122816	0.134522	0.1483443
788.187	7	0.403917	0.3504324	0.147483	0.1150698	0.133715	0.1391899
788.287	7	0.40133	0.3358843	0.1508717	0.1132643	0.13288	0.1441144
788.387	7	0.376627	0.3343838	0.1501974	0.111966	0.133793	0.1473739
788.487	7	0.400457	0.3362978	0.148445	0.1140423	0.134023	0.1556367
788.587	7	0.391916	0.3385216	0.1487048	0.1131632	0.135132	0.1552236
788.687	7	0.414607	0.3421636	0.1507294	0.1112114	0.135337	0.1540934
788.787	7	0.392902	0.3446831	0.1515222	0.1097834	0.135648	0.1530866
788.887	7	0.429595	0.3461902	0.1543219	0.1121445	0.134962	0.1517293
788.987	7	0.402174	0.3503039	0.1563324	0.1146711	0.131899	0.151174
789.087	7	0.408108	0.3471422	0.1553651	0.1133906	0.131117	0.1487606
789.187	7	0.410466	0.3424875	0.157732	0.115053	0.133394	0.1516606
789.287	7	0.419248	0.3357286	0.1574374	0.1139674	0.135909	0.1535078
789.387	7	0.418559	0.3326765	0.1542093	0.1068702	0.138129	0.1521248
789.487	7	0.422892	0.3354348	0.1549959	0.1051408	0.137668	0.1560927
789.587	7	0.419448	0.3436385	0.1531883	0.1042095	0.138776	0.1565775
789.687	7	0.421751	0.3212398	0.1556551	0.1029067	0.138917	0.1569161
789.787	7	0.407387	0.3312133	0.1528049	0.1060962	0.138652	0.1582944
789.887	7	0.41935	0.329585	0.1585351	0.1055567	0.134202	0.1509085
789.987	7	0.440456	0.3222691	0.1562744	0.1082178	0.138441	0.1472558
790.087	7	0.422191	0.3111548	0.151688	0.1065587	0.13869	0.147621
790.187	7	0.419543	0.3046877	0.1510051	0.1047279	0.140133	0.147572
790.287	7	0.407731	0.3148749	0.150492	0.1036753	0.140953	0.1505513
790.387	7	0.417306	0.3261745	0.1523553	0.1022606	0.141264	0.1514365
790.487	7	0.415624	0.3445507	0.1488178	0.1041853	0.142153	0.1503132
790.587	7	0.415265	0.3426659	0.1480933	0.1022088	0.140552	0.1523486
790.687	7	0.439389	0.3376083	0.1453768	0.1040896	0.142273	0.1524357
790.787	7	0.425439	0.3349156	0.1455597	0.1004745	0.142519	0.1537222
790.887	7	0.428554	0.337615	0.1485825	0.102461	0.143997	0.1530253
790.987	7	0.41687	0.3401728	0.1491611	0.1008301	0.143948	0.1538548
791.087	7	0.407086	0.3416123	0.149659	0.09784375	0.144505	0.1519895
791.187	7	0.42546	0.3443173	0.1396675	0.1005296	0.142632	0.1527744
791.287	7	0.426331	0.3460578	0.1489211	0.1004613	0.144111	0.1567908
791.387	7	0.422456	0.3517851	0.1448714	0.09616719	0.141852	0.1557008
791.487	7	0.415005	0.337381	0.1491422	0.09801231	0.142731	0.1583273
791.587	7	0.401016	0.3335015	0.1524944	0.1015629	0.144163	0.1549482
791.687	7	0.411179	0.3326207	0.1544929	0.1027136	0.146233	0.1542871
791.787	7	0.391098	0.3336796	0.1500074	0.09847265	0.145756	0.1528558
791.887	7	0.404534	0.3337994	0.1459096	0.1028411	0.146468	0.1532084
791.987	7	0.419439	0.336807	0.1489727	0.09737486	0.148346	0.1538368
792.087	7	0.441924	0.3395334	0.1541309	0.1046177	0.147396	0.1562922
792.187	7	0.450003	0.3386054	0.1506418	0.1001572	0.144561	0.15465
792.287	7	0.44278	0.340368	0.1474146	0.1018139	0.14458	0.1532328
792.387	7	0.437788	0.3366032	0.1501706	0.1053327	0.144615	0.1521356
792.487	7	0.414371	0.3313299	0.1497701	0.1057435	0.14691	0.149568
792.587	7	0.411248	0.3287967	0.157394	0.09893052	0.145627	0.1450056
792.687	7	0.3956	0.3307098	0.1567689	0.09261698	0.148144	0.1492739
792.787	7	0.42457	0.3351465	0.1543627	0.09946471	0.148002	0.136051
792.887	7	0.421291	0.3337467	0.1533441	0.1010859	0.146865	0.1387891
792.987	7	0.397765	0.3304918	0.1521585	0.09686128	0.142423	0.1463182
793.087	7	0.409344	0.3219374	0.1477442	0.0987801	0.141931	0.1470879
793.187	7	0.417701	0.3288973	0.147904	0.09771852	0.144	0.1461526
793.287	7	0.413061	0.3164045	0.1469825	0.1005757	0.140809	0.1480804
793.387	7	0.395801	0.3079599	0.1522905	0.09973523	0.144059	0.1507176
793.487	7	0.401823	0.3074742	0.1489099	0.10148	0.140938	0.1539779
793.587	7	0.408701	0.3144408	0.1473135	0.1030595	0.140748	0.1531138
793.687	7	0.407673	0.3352051	0.1467176	0.1016462	0.139518	0.1554935
793.787	7	0.435751	0.3452401	0.1460628	0.09533712	0.144917	0.1534388
793.887	7	0.475099	0.337187	0.1445661	0.09802771	0.142811	0.1548238
793.987	7	0.440586	0.334207	0.142191	0.1008954	0.138908	0.1546816
794.087	7	0.439072	0.3328327	0.1472638	0.09870622	0.139833	0.1549858
794.187	7	0.438907	0.3380482	0.1458965	0.09952069	0.140619	0.1527751
794.287	7	0.435416	0.3370872	0.1432352	0.09584023	0.144535	0.1523827
794.387	7	0.444311	0.3423021	0.1442443	0.09814554	0.142998	0.1518058
794.487	7	0.448945	0.3452721	0.1431457	0.09689526	0.141919	0.1539894
794.587	7	0.423522	0.3511894	0.1395809	0.09684151	0.14177	0.1507813
794.687	7	0.406263	0.3441969	0.1458225	0.09849738	0.140978	0.1527109
794.787	7	0.400942	0.3352414	0.1437492	0.09464059	0.144252	0.156614
794.887	7	0.412823	0.3359488	0.1416606	0.09292269	0.142563	0.1550396
794.987	7	0.406172	0.3377153	0.1403386	0.09556977	0.140678	0.1577972
795.087	7	0.420007	0.3373791	0.1419431	0.09657425	0.139986	0.1570681
795.187	7	0.406626	0.3388335	0.1413916	0.09439726	0.139414	0.1540553
795.287	7	0.404897	0.3411921	0.1352748	0.09365667	0.140144	0.156928
795.387	7	0.409317	0.3420449	0.1396142	0.09778357	0.142387	0.1540953
795.487	7	0.408859	0.3431051	0.1394814	0.1008764	0.138808	0.1577703

795.587	7	0.416117	0.3395631	0.1428001	0.1017701	0.136555	0.1523675
795.687	7	0.421412	0.3361675	0.1360879	0.103837	0.135298	0.1519975
795.787	7	0.406707	0.332808	0.1380251	0.09957081	0.132493	0.1562014
795.887	7	0.42815	0.3283855	0.1401066	0.09689457	0.129126	0.1601975
795.987	7	0.4183	0.3356467	0.1419104	0.09807337	0.129661	0.1615571
796.087	7	0.418381	0.3407109	0.1423139	0.1019912	0.129777	0.1610063
796.187	7	0.413551	0.3234172	0.1368392	0.1062799	0.132574	0.1580047
796.287	7	0.413856	0.3284633	0.1396773	0.1089026	0.13368	0.1584203
796.387	7	0.405835	0.3319469	0.1396159	0.1084351	0.13117	0.1592868
796.487	7	0.413359	0.3184519	0.139971	0.1147524	0.131243	0.165317
796.587	7	0.412808	0.3131191	0.1423634	0.114026	0.133102	0.1618477
796.687	7	0.397067	0.3016903	0.1441327	0.1156888	0.132345	0.1639145
796.787	7	0.400485	0.3129176	0.1427966	0.1155635	0.131828	0.1629633
796.887	7	0.38743	0.3196811	0.140878	0.1139539	0.131934	0.1597252
796.987	7	0.396752	0.3401387	0.1425339	0.1146989	0.131589	0.1561635
797.087	7	0.388835	0.3398706	0.147834	0.1152025	0.131683	0.1528818
797.187	7	0.405357	0.3346913	0.1499886	0.1168867	0.132895	0.1587034
797.287	7	0.410895	0.3313044	0.1479754	0.1178433	0.134338	0.1548929
797.387	7	0.403006	0.3366819	0.1451383	0.1181149	0.132562	0.15421
797.487	7	0.41739	0.3354915	0.1485229	0.119854	0.131249	0.1537422
797.587	7	0.40879	0.340931	0.1496914	0.1215704	0.131706	0.1560907
797.687	7	0.398316	0.3435093	0.151198	0.1179138	0.130154	0.1551304
797.787	7	0.400698	0.3481995	0.1489337	0.116479	0.130615	0.1555806
797.887	7	0.405334	0.3533089	0.1497573	0.1095979	0.132675	0.1588981
797.987	7	0.411317	0.3379271	0.1473733	0.1059865	0.133678	0.1509824
798.087	7	0.421325	0.3386716	0.1466976	0.1034116	0.133289	0.1561967
798.187	7	0.413162	0.3382196	0.1509657	0.10305	0.130309	0.157474
798.287	7	0.419139	0.339422	0.1482408	0.1035205	0.125485	0.1542457
798.387	7	0.400722	0.3392408	0.1467143	0.1061224	0.125537	0.1548815
798.487	7	0.422285	0.3397023	0.1490611	0.1080013	0.130141	0.1561277
798.587	7	0.431644	0.3415485	0.1480612	0.1043523	0.131128	0.1565134
798.687	7	0.418666	0.3407045	0.149498	0.1074928	0.128256	0.1549988
798.787	7	0.429529	0.341652	0.1505317	0.1020707	0.130842	0.1521885
798.887	7	0.416849	0.3388726	0.1464445	0.1018838	0.133715	0.1526958
798.987	7	0.417007	0.3319115	0.1505202	0.1021939	0.134512	0.153975
799.087	7	0.422445	0.3310975	0.1475891	0.1005077	0.132562	0.1543534
799.187	7	0.41101	0.3348401	0.1489543	0.1004192	0.135939	0.1570183
799.287	7	0.43702	0.3390476	0.1519827	0.09972425	0.140467	0.1576146
799.387	7	0.422227	0.3284276	0.1500047	0.1001903	0.138806	0.1515576
799.487	7	0.429064	0.3240266	0.1491351	0.1022281	0.140738	0.1545714
799.587	7	0.404895	0.3245353	0.1522306	0.1014545	0.141941	0.1532984
799.687	7	0.408198	0.3242657	0.1529341	0.1000262	0.140801	0.1516863
799.787	7	0.417974	0.3142125	0.1550474	0.1006631	0.138879	0.1544516
799.887	7	0.420009	0.3024098	0.1521799	0.1007338	0.140964	0.153679
799.987	7	0.4265	0.3182242	0.1531079	0.09737002	0.141446	0.1498211
800.087	7	0.410464	0.3213983	0.1533862	0.09885583	0.143009	0.1501515
800.187	7	0.395228	0.3416005	0.1534051	0.09710469	0.144235	0.1412951
800.287	7	0.403548	0.3456658	0.1525275	0.09617005	0.144538	0.1470236
800.387	7	0.389406	0.3395078	0.1543027	0.09710865	0.147008	0.1483524
800.487	7	0.381441	0.3372574	0.153282	0.1000329	0.150136	0.1544938
800.587	7	0.40202	0.338136	0.1565059	0.1013745	0.146523	0.1541196
800.687	7	0.442467	0.3412449	0.1518893	0.104328	0.145174	0.151567
800.787	7	0.443962	0.3422424	0.1553553	0.1015062	0.142624	0.1519758
800.887	7	0.448218	0.3441886	0.1530976	0.1070074	0.143214	0.1490501
800.987	7	0.412808	0.3461245	0.1505963	0.1115078	0.144631	0.1507632
801.087	7	0.413384	0.3544407	0.1516068	0.1073348	0.14386	0.1509231
801.187	7	0.400514	0.3444844	0.1497667	0.09983591	0.143957	0.1481683
801.287	7	0.411348	0.3367216	0.154174	0.09910608	0.145424	0.1501707
801.387	7	0.418558	0.3376797	0.1513028	0.1041194	0.145704	0.1521286
801.487	7	0.406905	0.3390282	0.1512216	0.1053603	0.144524	0.155434
801.587	7	0.40136	0.3381081	0.147407	0.1026531	0.144493	0.1506632
801.687	7	0.417051	0.3381189	0.1458571	0.1028679	0.146591	0.1483712
801.787	7	0.41149	0.34252	0.1483899	0.09976412	0.146561	0.1486124
801.887	7	0.402387	0.3409959	0.1520564	0.1014444	0.150753	0.1506846
801.987	7	0.399723	0.3437725	0.1509419	0.1052601	0.149438	0.1489174
802.087	7	0.413908	0.341479	0.1421317	0.1067509	0.148581	0.1504719
802.187	7	0.425256	0.3364407	0.1509924	0.1078596	0.144651	0.1507385
802.287	7	0.412956	0.3342598	0.1460643	0.1019272	0.145567	0.1517734
802.387	7	0.422358	0.3306136	0.1506306	0.09740189	0.144221	0.1546599
802.487	7	0.407667	0.3367296	0.151647	0.1018919	0.145136	0.1534661
802.587	7	0.412218	0.3385835	0.1555906	0.1004916	0.146008	0.1547938
802.687	7	0.409272	0.3196801	0.1515359	0.101886	0.142362	0.1517366
802.787	7	0.413972	0.3250795	0.1464008	0.104784	0.14482	0.1526124
802.887	7	0.4517	0.3261854	0.1473294	0.1011418	0.141926	0.1543401
802.987	7	0.441507	0.3122381	0.1511642	0.1039688	0.145509	0.1559284
803.087	7	0.451295	0.3116248	0.1501364	0.09948567	0.146518	0.159819
803.187	7	0.423296	0.3070599	0.1464121	0.102879	0.142436	0.1541274
803.287	7	0.419132	0.3192211	0.1482594	0.1030666	0.143179	0.1533179
803.387	7	0.413423	0.3270373	0.1480521	0.0986404	0.143202	0.1543909
803.487	7	0.422719	0.3448295	0.1567893	0.09840375	0.144081	0.1550104
803.587	7	0.421061	0.3403118	0.1569128	0.09878738	0.139996	0.156466
803.687	7	0.422299	0.337771	0.1521328	0.1003788	0.140138	0.1558923
803.787	7	0.421003	0.3359423	0.1584874	0.09845426	0.141857	0.1536456
803.887	7	0.4281	0.3392459	0.1545558	0.1011802	0.138616	0.1531314
803.987	7	0.430608	0.3396233	0.1488777	0.1023674	0.141087	0.1511094
804.087	7	0.427824	0.3449949	0.1514379	0.1022402	0.142505	0.1478618
804.187	7	0.414592	0.3442855	0.1480807	0.1022515	0.141208	0.1477232
804.287	7	0.407769	0.350038	0.1498118	0.1025878	0.139123	0.1394534
804.387	7	0.418701	0.3540221	0.1490732	0.0977888	0.140123	0.1429058
804.487	7	0.429548	0.3395663	0.1445307	0.0988989	0.138908	0.1471314
804.587	7	0.420177	0.3362448	0.1460213	0.09862883	0.141882	0.1466248
804.687	7	0.42675	0.3352603	0.1446222	0.1012884	0.140992	0.1462031
804.787	7	0.415085	0.3364692	0.1418813	0.1046833	0.137604	0.1476885
804.887	7	0.410174	0.3380826	0.1416419	0.1087945	0.135733	0.1522894
804.987	7	0.402616	0.340929	0.1424447	0.1102949	0.134536	0.1509849

805.087	7	0.416942	0.3416926	0.1430735	0.1123766	0.136058	0.1552981
805.187	7	0.400871	0.3430472	0.1425283	0.111641	0.131753	0.1553728
805.287	7	0.401189	0.3418122	0.1431345	0.1147198	0.131985	0.1574072
805.387	7	0.39931	0.341024	0.1396849	0.1153836	0.135932	0.1569217
805.487	7	0.376498	0.3358147	0.138712	0.1148586	0.134962	0.1545654
805.587	7	0.397937	0.3337613	0.1393613	0.1156155	0.13357	0.1534917
805.687	7	0.383411	0.3384467	0.1472912	0.1158252	0.132542	0.1516377
805.787	7	0.415078	0.3446709	0.1433567	0.1156025	0.134918	0.1517954
805.887	7	0.396954	0.327153	0.1413007	0.1164811	0.134046	0.1524766
805.987	7	0.419752	0.3229364	0.1406251	0.1165386	0.135548	0.1541748
806.087	7	0.407018	0.317867	0.1419276	0.1181931	0.134028	0.1558865
806.187	7	0.403553	0.3257364	0.1380243	0.1185819	0.130808	0.1564887
806.287	7	0.400213	0.3140041	0.1402259	0.1190169	0.13094	0.1586935
806.387	7	0.402673	0.3079364	0.1422643	0.1173811	0.131974	0.1567955
806.487	7	0.404322	0.3148444	0.1412245	0.1090848	0.133247	0.1585576
806.587	7	0.410266	0.3208402	0.1367689	0.1096041	0.135679	0.1582817
806.687	7	0.417245	0.3447848	0.1371972	0.1082618	0.133063	0.1609023
806.787	7	0.414039	0.3472977	0.1416149	0.1071362	0.134808	0.155874
806.887	7	0.416826	0.3373915	0.1429088	0.1093179	0.134849	0.1547915
806.987	7	0.405198	0.3342355	0.1399083	0.1060348	0.135158	0.156189
807.087	7	0.433344	0.3347877	0.1361148	0.1061399	0.136758	0.1593722
807.187	7	0.424943	0.3366998	0.1410918	0.1043997	0.13778	0.160568
807.287	7	0.43396	0.3380992	0.1381412	0.1053555	0.136937	0.1587063
807.387	7	0.421954	0.3407138	0.139457	0.1036765	0.134023	0.1603637
807.487	7	0.422515	0.3471293	0.1394615	0.102133	0.130086	0.1599085
807.587	7	0.404288	0.3608336	0.1461947	0.1017047	0.132774	0.1657064
807.687	7	0.418605	0.3449805	0.1437146	0.09964625	0.134413	0.1622863
807.787	7	0.41203	0.3360239	0.1442472	0.1027306	0.133461	0.1618836
807.887	7	0.423969	0.3366854	0.1442386	0.1009779	0.133649	0.1628672
807.987	7	0.414091	0.3345959	0.1485893	0.1033097	0.131598	0.1608128
808.087	7	0.416133	0.3366118	0.1501196	0.1046376	0.13578	0.1596407
808.187	7	0.394318	0.3370258	0.1482498	0.1046081	0.134365	0.1607668
808.287	7	0.415065	0.3410864	0.1449528	0.1044797	0.133151	0.1576534
808.387	7	0.420417	0.3389482	0.1467593	0.1042955	0.132752	0.1563734
808.487	7	0.412828	0.3409804	0.1486023	0.1034538	0.137184	0.1533918
808.587	7	0.414854	0.3395771	0.1502848	0.1031609	0.136511	0.1572279
808.687	7	0.395155	0.335765	0.1479778	0.104806	0.139265	0.1560056
808.787	7	0.394184	0.3330163	0.1489183	0.1045945	0.141987	0.1588336
808.887	7	0.391618	0.334088	0.148329	0.09705291	0.140752	0.1586578
808.987	7	0.384628	0.342274	0.1478583	0.1008886	0.140019	0.1503852
809.087	7	0.381256	0.3411652	0.151864	0.1012479	0.139798	0.1560244
809.187	7	0.402369	0.3241921	0.1511759	0.1052689	0.142385	0.1536864
809.287	7	0.425829	0.3165124	0.1475193	0.1064416	0.141223	0.1528025
809.387	7	0.423269	0.3303504	0.1490525	0.1028765	0.143469	0.1540274
809.487	7	0.4248	0.3146217	0.1489664	0.1080287	0.143614	0.1528739
809.587	7	0.411509	0.3087384	0.1488338	0.1078071	0.144022	0.1548339
809.687	7	0.402724	0.3084497	0.1487562	0.1042206	0.144599	0.1525739
809.787	7	0.396248	0.3181173	0.14279	0.09592347	0.143671	0.1521318
809.887	7	0.413577	0.3307544	0.1460796	0.09988198	0.145394	0.1535431
809.987	7	0.414854	0.3489949	0.1452806	0.1044329	0.142045	0.1569027
810.087	7	0.400809	0.3401458	0.147655	0.1024934	0.14408	0.1579579
810.187	7	0.40642	0.3368806	0.1505835	0.09999404	0.145555	0.1542139
810.287	7	0.41814	0.3334627	0.1453393	0.1035682	0.147075	0.1540074
810.387	7	0.41237	0.3377833	0.1481472	0.1023348	0.14535	0.1514109
810.487	7	0.396021	0.3351261	0.150445	0.1021466	0.144342	0.1510428
810.587	7	0.407627	0.3418889	0.1501961	0.1069071	0.144729	0.153027
810.687	7	0.404132	0.3431862	0.1505972	0.1093414	0.147555	0.1524782
810.787	7	0.41285	0.3562786	0.1509889	0.1113161	0.146232	0.1495718
810.887	7	0.415181	0.3547885	0.1539046	0.1075624	0.148496	0.1457738
810.987	7	0.411897	0.3382157	0.1502775	0.108033	0.147846	0.1410577
811.087	7	0.410049	0.3396283	0.151997	0.1095872	0.147851	0.1515423
811.187	7	0.410009	0.3368028	0.1486136	0.107061	0.143512	0.1563595
811.287	7	0.404782	0.3383697	0.1493968	0.108576	0.142316	0.1568662
811.387	7	0.416819	0.3389566	0.1470964	0.1074882	0.141036	0.155018
811.487	7	0.414118	0.3402715	0.1503917	0.1052935	0.139905	0.154939
811.587	7	0.422991	0.3406174	0.1468077	0.1048299	0.140015	0.1520823
811.687	7	0.420311	0.3413314	0.1518645	0.1002911	0.139269	0.15221
811.787	7	0.410615	0.3402575	0.1511851	0.1047607	0.13494	0.1488282
811.887	7	0.402806	0.3377064	0.1489833	0.1038634	0.135854	0.1511445
811.987	7	0.41638	0.3331429	0.1510737	0.1019778	0.138297	0.1544634
812.087	7	0.400195	0.3324504	0.1472324	0.1014942	0.135617	0.1530808
812.187	7	0.419917	0.3389267	0.1507872	0.1034717	0.134697	0.1574131
812.287	7	0.410236	0.343462	0.1480518	0.09908356	0.134618	0.1602898
812.387	7	0.4051	0.3225203	0.147335	0.0975171	0.136328	0.1613457
812.487	7	0.424265	0.3214937	0.1452793	0.1034002	0.132407	0.1555494
812.587	7	0.411471	0.3201135	0.146925	0.1041307	0.131886	0.1535984
812.687	7	0.419608	0.3226076	0.1483413	0.104814	0.13405	0.1559245
812.787	7	0.412452	0.3151472	0.1509243	0.1072318	0.133039	0.1537339
812.887	7	0.40117	0.303484	0.1490985	0.1072979	0.135718	0.1502211
812.987	7	0.412084	0.3148778	0.1382049	0.1038859	0.13566	0.1487691
813.087	7	0.416737	0.3219378	0.1458063	0.1051946	0.135005	0.1495801
813.187	7	0.417634	0.3431646	0.1423294	0.1031377	0.133439	0.1514745
813.287	7	0.409806	0.345533	0.1472548	0.1082433	0.130238	0.1545359
813.387	7	0.415956	0.3337778	0.149369	0.1074233	0.132164	0.1535783
813.487	7	0.409452	0.3308078	0.1536852	0.1092831	0.131878	0.1528347
813.587	7	0.401403	0.3341792	0.1499774	0.112362	0.13154	0.1503554
813.687	7	0.409741	0.3346158	0.1459027	0.1122556	0.13119	0.15343
813.787	7	0.392309	0.3364287	0.1512036	0.1109445	0.132319	0.1572217
813.887	7	0.391075	0.3413122	0.1549023	0.1138053	0.131633	0.1593337
813.987	7	0.396451	0.3472577	0.1510521	0.1139007	0.131369	0.1632505
814.087	7	0.374098	0.360039	0.1483461	0.1138552	0.127832	0.1571347
814.187	7	0.388862	0.3454314	0.1515532	0.1153378	0.129994	0.1558727
814.287	7	0.39168	0.3387778	0.1505103	0.1163597	0.132304	0.1547296
814.387	7	0.414263	0.3356289	0.1566077	0.1155526	0.12976	0.1524843
814.487	7	0.397405	0.3343708	0.1561802	0.1160452	0.133157	0.1536357

814.587	7	0.423857	0.3351202	0.152575	0.1168649	0.132517	0.153248
814.687	7	0.405512	0.3366946	0.1555816	0.1180315	0.132654	0.1517694
814.787	7	0.41205	0.3400934	0.155942	0.1149038	0.133497	0.1538122
814.887	7	0.400674	0.3389387	0.1498935	0.1152218	0.129945	0.153026
814.987	7	0.402063	0.3415899	0.1512883	0.1149177	0.131118	0.1512803
815.087	7	0.408881	0.3384174	0.1472155	0.1091845	0.132474	0.1459197
815.187	7	0.410595	0.3345438	0.1508165	0.1096666	0.131863	0.1412059
815.287	7	0.414599	0.3323294	0.1489555	0.1078644	0.130118	0.1463811
815.387	7	0.418609	0.3350598	0.1463913	0.1064535	0.130436	0.1475166
815.487	7	0.405682	0.3405465	0.1460497	0.1087994	0.131126	0.1474302
815.587	7	0.411456	0.3339643	0.1477994	0.1087563	0.131574	0.1492399
815.687	7	0.427754	0.3231871	0.1456016	0.1121782	0.134214	0.1507076
815.787	7	0.429384	0.3123375	0.1436127	0.1080792	0.135316	0.1547041
815.887	7	0.435627	0.3311758	0.145959	0.1088667	0.136824	0.1547032
815.987	7	0.409511	0.3302044	0.146369	0.1094532	0.135455	0.1573152
816.087	7	0.422065	0.3171048	0.1441293	0.1095788	0.132128	0.1547699
816.187	7	0.404916	0.3318869	0.145015	0.108193	0.130158	0.1544958
816.287	7	0.411679	0.348273	0.1422145	0.1051515	0.134887	0.1551731
816.387	7	0.427385	0.3534034	0.1407487	0.1070179	0.135976	0.1538543
816.487	7	0.429853	0.3667063	0.1424203	0.1043177	0.137093	0.1535648
816.587	7	0.419723	0.3617589	0.1451069	0.1072226	0.138039	0.1535374
816.687	7	0.414103	0.362544	0.1410821	0.1066922	0.139651	0.1548185
816.787	7	0.391317	0.3643427	0.1382706	0.1051601	0.139964	0.1499031
816.887	7	0.414045	0.3739475	0.1413492	0.1064015	0.137175	0.1520083
816.987	7	0.420193	0.3758565	0.1415753	0.1073289	0.142744	0.1533097
817.087	7	0.412617	0.3824208	0.1338447	0.1020152	0.138421	0.1524779
817.187	7	0.406903	0.3705937	0.1379291	0.1019389	0.13865	0.155604
817.287	7	0.391925	0.3620301	0.140061	0.1046682	0.139158	0.1530261
817.387	7	0.399513	0.3710214	0.1414981	0.1045608	0.139452	0.156067
817.487	7	0.380288	0.3682575	0.1375389	0.0996271	0.141418	0.1542284
817.587	7	0.380903	0.3705499	0.1379258	0.1033809	0.142156	0.1591032
817.687	7	0.385422	0.3775429	0.1428069	0.1006642	0.141521	0.1547368
817.787	7	0.407527	0.3835548	0.1420334	0.1061392	0.141191	0.1533857
817.887	7	0.43036	0.3887242	0.1391698	0.1045823	0.141297	0.1537807
817.987	7	0.434053	0.3915531	0.1361229	0.1020298	0.143289	0.1565276
818.087	7	0.431897	0.3941783	0.1384172	0.1072323	0.143298	0.1570972
818.187	7	0.415482	0.3966821	0.1360022	0.1064306	0.144463	0.1558209
818.287	7	0.411425	0.3931158	0.1390495	0.101987	0.143929	0.1563705
818.387	7	0.399518	0.3868444	0.1388881	0.09846702	0.142225	0.1567937
818.487	7	0.422025	0.3799092	0.1455017	0.1017149	0.144621	0.1629659
818.587	7	0.415662	0.3759738	0.1427251	0.1011802	0.144856	0.1598445
818.687	7	0.398525	0.3695306	0.1434698	0.1010657	0.143503	0.1591035
818.787	7	0.410108	0.3639909	0.1414558	0.1056736	0.140724	0.1587699
818.887	7	0.416296	0.3399755	0.1476016	0.102841	0.140332	0.1584875
818.987	7	0.41429	0.3409305	0.1487144	0.1016843	0.140652	0.1562732
819.087	7	0.398133	0.3379246	0.1452512	0.100045	0.14063	0.1546698
819.187	7	0.404114	0.3184978	0.1413122	0.1029054	0.140849	0.153155
819.287	7	0.406468	0.3257531	0.1422069	0.1090998	0.141748	0.1533597
819.387	7	0.408575	0.3315008	0.1449403	0.1110001	0.141636	0.1519541
819.487	7	0.421391	0.3531174	0.1504123	0.1043491	0.143248	0.1561479
819.587	7	0.414591	0.36033	0.148373	0.1074031	0.139344	0.1555766
819.687	7	0.417523	0.359128	0.1491104	0.1073724	0.137609	0.1590033
819.787	7	0.410815	0.3537262	0.1481005	0.1018545	0.135638	0.157427
819.887	7	0.40642	0.3517756	0.1460438	0.1050957	0.13692	0.1491521
819.987	7	0.424046	0.3568341	0.1486647	0.102213	0.135674	0.1528198
820.087	7	0.418851	0.3622289	0.1497038	0.1039195	0.135574	0.1511471
820.187	7	0.434225	0.3669021	0.1437147	0.1025182	0.135287	0.1509473
820.287	7	0.413384	0.3742534	0.1473143	0.1007983	0.136866	0.1545138
820.387	7	0.402531	0.3762436	0.1485835	0.1046565	0.132634	0.1538444
820.487	7	0.392115	0.3520614	0.1487477	0.1031049	0.133144	0.1590684
820.587	7	0.401939	0.3536299	0.1518509	0.1024409	0.132967	0.1569985
820.687	7	0.395133	0.3473993	0.1449161	0.103282	0.131135	0.1538019
820.787	7	0.408765	0.3500444	0.1464575	0.1053141	0.130023	0.1545616
820.887	7	0.399573	0.3530419	0.1450929	0.1019837	0.133209	0.1568342
820.987	7	0.390941	0.3594186	0.1477766	0.1024009	0.134109	0.1598226
821.087	7	0.416865	0.363091	0.1523892	0.103732	0.132984	0.1556527
821.187	7	0.4057	0.3677318	0.1478983	0.1047816	0.132168	0.1541098
821.287	7	0.395935	0.3718148	0.1482067	0.1051078	0.132857	0.1550092
821.387	7	0.40202	0.372229	0.1498758	0.105942	0.1329	0.1518005
821.487	7	0.395142	0.36927	0.1503542	0.105377	0.132587	0.1551739
821.587	7	0.411479	0.3647656	0.1511638	0.1021201	0.132752	0.1561449
821.687	7	0.409991	0.3527333	0.1501458	0.1052989	0.129307	0.1525588
821.787	7	0.425144	0.3444309	0.1524424	0.1034908	0.12935	0.1509021
821.887	7	0.412238	0.3412726	0.1501749	0.1045754	0.128467	0.1434133
821.987	7	0.422516	0.3445285	0.1532892	0.1064073	0.131228	0.1482501
822.087	7	0.403866	0.3467664	0.1500854	0.1064035	0.128576	0.152349
822.187	7	0.409678	0.334726	0.152867	0.1117596	0.129115	0.1547361
822.287	7	0.405146	0.3253488	0.1499269	0.1104278	0.128077	0.153991
822.387	7	0.386389	0.3248717	0.1515438	0.1117882	0.124979	0.1543322
822.487	7	0.400627	0.3105511	0.149959	0.1120224	0.120922	0.1506763
822.587	7	0.389405	0.3109972	0.1541297	0.1108924	0.122036	0.1519676
822.687	7	0.384038	0.343683	0.1531422	0.1125154	0.125544	0.1490395
822.787	7	0.38292	0.3418117	0.1507324	0.1138297	0.125153	0.1502077
822.887	7	0.405787	0.3462676	0.1513837	0.1148386	0.125339	0.1524249
822.987	7	0.41592	0.344703	0.1488347	0.1140593	0.123822	0.1510974
823.087	7	0.404885	0.3385211	0.1529238	0.1119082	0.128839	0.1535215
823.187	7	0.425445	0.3398941	0.1481733	0.1143233	0.127085	0.1539447
823.287	7	0.414813	0.3430322	0.1465273	0.1185487	0.127753	0.1537496
823.387	7	0.40392	0.347632	0.1442736	0.1172701	0.129306	0.1506322
823.487	7	0.407799	0.3510568	0.1461197	0.1172755	0.125343	0.1494448
823.587	7	0.408936	0.3551271	0.145556	0.1144108	0.128321	0.1494112
823.687	7	0.414852	0.3504868	0.1469062	0.1095726	0.130291	0.1506552
823.787	7	0.413633	0.3516556	0.1471564	0.1095771	0.133463	0.1504163
823.887	7	0.419075	0.3450407	0.1376525	0.1087122	0.130588	0.1513975
823.987	7	0.429034	0.3416681	0.1471491	0.1071424	0.13044	0.1519171

824.087	7	0.40742	0.3434855	0.1441196	0.1084082	0.13246	0.153339
824.187	7	0.421819	0.3470125	0.1492601	0.1087013	0.133234	0.1550953
824.287	7	0.427631	0.3482722	0.1526445	0.1093978	0.133273	0.1523504
824.387	7	0.42601	0.3501123	0.153843	0.1097073	0.13486	0.152853
824.487	7	0.432683	0.3529651	0.150339	0.10573	0.135259	0.1493332
824.587	7	0.40732	0.355108	0.1468148	0.1069763	0.131189	0.1507842
824.687	7	0.418411	0.3565333	0.148956	0.1065257	0.128405	0.1530525
824.787	7	0.418935	0.3531778	0.1536918	0.1040268	0.130667	0.1539722
824.887	7	0.411839	0.3487582	0.1497998	0.1039885	0.135065	0.1572393
824.987	7	0.424296	0.3467656	0.1487148	0.1044579	0.136161	0.1539888
825.087	7	0.421056	0.3399124	0.1511944	0.1020958	0.134908	0.1543475
825.187	7	0.422862	0.34093	0.1503411	0.1046536	0.137696	0.1533482
825.287	7	0.405636	0.3420342	0.1567186	0.1061675	0.139661	0.1533064
825.387	7	0.396376	0.3331325	0.1556021	0.1023328	0.136826	0.1553841
825.487	7	0.413919	0.3289592	0.1508036	0.1049789	0.13325	0.153378
825.587	7	0.416393	0.3300911	0.1513932	0.1055163	0.137219	0.151711
825.687	7	0.415139	0.3071394	0.1524784	0.1022038	0.135932	0.1505435
825.787	7	0.412706	0.3090425	0.1492426	0.1035551	0.138026	0.1504673
825.887	7	0.391782	0.3174867	0.1521344	0.1060231	0.142312	0.1498897
825.987	7	0.401773	0.3400981	0.1469573	0.1066484	0.139655	0.1475162
826.087	7	0.379585	0.3453146	0.150538	0.1023712	0.138379	0.1421289
826.187	7	0.377341	0.3473105	0.1488393	0.106043	0.140838	0.1439076
826.287	7	0.38717	0.338697	0.1443455	0.1062907	0.140172	0.1460621
826.387	7	0.412797	0.3366428	0.1448129	0.1095475	0.140397	0.1451585
826.487	7	0.433715	0.3358853	0.1444488	0.1042009	0.141466	0.1463499
826.587	7	0.427621	0.3387003	0.1433204	0.1057468	0.140399	0.1493929
826.687	7	0.411227	0.3400272	0.1422723	0.1099499	0.143914	0.1528491
826.787	7	0.415343	0.347678	0.1449251	0.1089643	0.143589	0.1527494
826.887	7	0.400747	0.3472632	0.1437634	0.1015479	0.1418	0.1552238
826.987	7	0.398783	0.3461381	0.1413668	0.09796075	0.140926	0.1551452
827.087	7	0.411478	0.3462502	0.1444052	0.1025563	0.140913	0.1556657
827.187	7	0.409074	0.3358595	0.1426255	0.1051951	0.140745	0.1581256
827.287	7	0.393514	0.3351687	0.1415924	0.1008381	0.139912	0.1567801
827.387	7	0.410069	0.3360492	0.1436451	0.1025966	0.137636	0.1521726
827.487	7	0.409644	0.3381677	0.1427953	0.09983198	0.136467	0.1521642
827.587	7	0.405258	0.3386591	0.1404734	0.1016125	0.134306	0.1536561
827.687	7	0.394557	0.3420063	0.1405569	0.1012531	0.135954	0.1530104
827.787	7	0.403375	0.3427828	0.1426293	0.1049835	0.135999	0.1548907
827.887	7	0.411683	0.3445765	0.1415775	0.1093208	0.1365	0.1564125
827.987	7	0.404292	0.3444346	0.1338538	0.1054799	0.138533	0.1556963
828.087	7	0.419502	0.3400348	0.1377711	0.1027559	0.140839	0.1570188
828.187	7	0.422995	0.3353553	0.1377326	0.1059405	0.136721	0.1547847
828.287	7	0.420524	0.3335005	0.1385215	0.1066504	0.133617	0.157967
828.387	7	0.420699	0.3339542	0.134245	0.1054873	0.136205	0.1575156
828.487	7	0.413896	0.3411551	0.1368816	0.1070444	0.137297	0.162587
828.587	7	0.428935	0.335638	0.1376229	0.1030484	0.136178	0.1568755
828.687	7	0.413102	0.3193013	0.1404783	0.1068284	0.137659	0.1578476
828.787	7	0.419271	0.3161771	0.1387682	0.1040688	0.137279	0.158514
828.887	7	0.410153	0.3158999	0.1342728	0.103061	0.136879	0.1604616
828.987	7	0.403768	0.3074934	0.1380172	0.1042132	0.135907	0.1600327
829.087	7	0.401971	0.3037104	0.136964	0.1002092	0.136531	0.1556499
829.187	7	0.40967	0.3364312	0.1357069	0.1025248	0.132825	0.1573362
829.287	7	0.400737	0.3306121	0.1337451	0.1024338	0.132541	0.1587359
829.387	7	0.407208	0.3456458	0.1404979	0.10227	0.133831	0.1614626
829.487	7	0.401886	0.3398844	0.1372457	0.1017677	0.134938	0.1584494
829.587	7	0.400846	0.3349607	0.1383583	0.1026698	0.133913	0.1592312
829.687	7	0.419014	0.3364444	0.1405043	0.106487	0.132078	0.1589655
829.787	7	0.408162	0.3366945	0.1465059	0.1069333	0.132571	0.159174
829.887	7	0.397812	0.3407421	0.1468461	0.1085767	0.130799	0.1547825
829.987	7	0.397106	0.3424127	0.1464164	0.1097348	0.12992	0.1532144
830.087	7	0.399743	0.3452947	0.1410695	0.103562	0.131171	0.1517314
830.187	7	0.413973	0.3458818	0.1431037	0.103947	0.130017	0.1520276
830.287	7	0.413252	0.346637	0.1453339	0.1052888	0.128777	0.1494286
830.387	7	0.420217	0.3406179	0.1470318	0.1066357	0.128941	0.1525852
830.487	7	0.411748	0.334166	0.1454176	0.1060181	0.129982	0.1527002
830.587	7	0.41868	0.3366239	0.1458207	0.1076207	0.130242	0.1567062
830.687	7	0.408314	0.337228	0.1444807	0.1080566	0.128519	0.1569095
830.787	7	0.4088	0.3376536	0.1423496	0.1138298	0.13074	0.1501333
830.887	7	0.399177	0.3401192	0.145738	0.1134125	0.130056	0.1546381
830.987	7	0.387949	0.3429023	0.150059	0.1154979	0.131659	0.1538289
831.087	7	0.406548	0.3410362	0.1472627	0.1163509	0.126826	0.1534799
831.187	7	0.379762	0.3444728	0.1471592	0.1162405	0.128249	0.1559907
831.287	7	0.396128	0.3403538	0.1452839	0.1151873	0.130874	0.1538082
831.387	7	0.382536	0.3356797	0.1459688	0.1156223	0.129386	0.1544215
831.487	7	0.416062	0.3340831	0.149786	0.1143024	0.12807	0.1523357
831.587	7	0.412547	0.33204	0.141871	0.1139086	0.13008	0.1520499
831.687	7	0.408073	0.3354812	0.1435779	0.1126312	0.131947	0.1532426
831.787	7	0.419262	0.3383078	0.1433348	0.1147223	0.132355	0.1579991
831.887	7	0.408729	0.3226018	0.1451445	0.1185593	0.13339	0.1594764
831.987	7	0.403763	0.3188853	0.1500638	0.1174674	0.131106	0.156694
832.087	7	0.404107	0.31948	0.1457741	0.1156582	0.130776	0.1540782
832.187	7	0.406005	0.3057855	0.1482875	0.1107804	0.133982	0.1535138
832.287	7	0.411739	0.306574	0.1468879	0.1078204	0.132996	0.1514565
832.387	7	0.417454	0.3190906	0.147782	0.1061903	0.132443	0.1554931
832.487	7	0.417162	0.3344368	0.1506712	0.1067744	0.132579	0.1548944
832.587	7	0.426958	0.3351536	0.1502057	0.1075559	0.131948	0.1506623
832.687	7	0.404509	0.343536	0.1515817	0.1066322	0.133015	0.1512115
832.787	7	0.426845	0.3327969	0.1491887	0.107775	0.133066	0.1427333
832.887	7	0.427371	0.335368	0.1526297	0.1061579	0.134047	0.1490979
832.987	7	0.426897	0.3336916	0.1475154	0.1081317	0.135378	0.1526544
833.087	7	0.419129	0.3382402	0.149012	0.1069523	0.134252	0.1565683
833.187	7	0.409292	0.3373965	0.1476146	0.1068482	0.130713	0.1561515
833.287	7	0.400444	0.3450613	0.1474837	0.1056901	0.128649	0.1559975
833.387	7	0.416546	0.3425319	0.1473232	0.1033181	0.131317	0.1529666
833.487	7	0.407347	0.3440024	0.1503792	0.1079595	0.132914	0.153453

833.587	7	0.42556	0.3472268	0.1504335	0.1042795	0.133971	0.1496741
833.687	7	0.413013	0.3356093	0.1491157	0.1050151	0.131485	0.1491974
833.787	7	0.418344	0.3363538	0.148561	0.1074075	0.135086	0.1536919
833.887	7	0.395394	0.338622	0.1459366	0.1074405	0.136383	0.1512078
833.987	7	0.399939	0.3398411	0.150397	0.1055021	0.135146	0.1539107
834.087	7	0.43662	0.3422262	0.1495705	0.1055591	0.136005	0.152252
834.187	7	0.415612	0.3446693	0.1447344	0.105114	0.138807	0.1534822
834.287	7	0.418564	0.3428214	0.1438759	0.1017731	0.137706	0.1521977
834.387	7	0.425274	0.3454739	0.146197	0.1033485	0.140395	0.149165
834.487	7	0.403121	0.3421885	0.1440039	0.1053782	0.141341	0.1518405
834.587	7	0.396332	0.3396845	0.1497009	0.1001085	0.140366	0.1479983
834.687	7	0.378086	0.3344103	0.1481462	0.1013297	0.138354	0.1505985
834.787	7	0.393559	0.3296498	0.1414596	0.1024989	0.141417	0.1527728
834.887	7	0.40584	0.3327915	0.1478948	0.1050972	0.139618	0.1523418
834.987	7	0.438818	0.3393249	0.1434927	0.1064354	0.139209	0.15314
835.087	7	0.435966	0.3258183	0.1477251	0.1026596	0.137533	0.1548079
835.187	7	0.440754	0.3155774	0.1510352	0.1067174	0.138982	0.1533926
835.287	7	0.414923	0.3179789	0.1524156	0.1097726	0.140641	0.1538409
835.387	7	0.403716	0.3096524	0.1476121	0.1041789	0.138523	0.1530944
835.487	7	0.40558	0.3078135	0.1418731	0.0974874	0.139356	0.1529686
835.587	7	0.414952	0.3031384	0.1443356	0.09932011	0.139583	0.1547558
835.687	7	0.419928	0.3273942	0.1499761	0.1059029	0.142585	0.1539507
835.787	7	0.400759	0.3284427	0.1458589	0.1055349	0.142059	0.1604753
835.887	7	0.396712	0.3420095	0.144449	0.1023366	0.141451	0.1568023
835.987	7	0.411989	0.3430411	0.1490461	0.1040021	0.140943	0.1555559
836.087	7	0.405046	0.3363821	0.149168	0.09912416	0.141135	0.1543692
836.187	7	0.39284	0.3378214	0.1570531	0.09892134	0.139284	0.1544513
836.287	7	0.395125	0.3399757	0.1572371	0.1028693	0.141455	0.1560516
836.387	7	0.395914	0.3416409	0.1509302	0.102286	0.143453	0.1557553
836.487	7	0.406561	0.3456027	0.1557029	0.104903	0.141441	0.1545285
836.587	7	0.397552	0.3486513	0.1536652	0.1009078	0.144745	0.1528307
836.687	7	0.412477	0.3452995	0.1486101	0.09931585	0.147649	0.1493379
836.787	7	0.401166	0.348885	0.1504356	0.09977905	0.14488	0.1484964
836.887	7	0.405346	0.3384871	0.1462284	0.1005674	0.137025	0.1491781
836.987	7	0.403977	0.3364694	0.1483456	0.1024321	0.134687	0.1416929
837.087	7	0.402214	0.3362787	0.1482849	0.10473	0.134652	0.1453531
837.187	7	0.412385	0.3367892	0.1435833	0.09975026	0.136767	0.1502429
837.287	7	0.417128	0.3404099	0.1450165	0.1039115	0.13761	0.1459228
837.387	7	0.415985	0.3432002	0.1440075	0.1007281	0.137332	0.1474778
837.487	7	0.402432	0.3449411	0.1423771	0.1030196	0.13461	0.1493521
837.587	7	0.401572	0.3450138	0.1395584	0.102309	0.136347	0.1518801
837.687	7	0.398213	0.3456383	0.1394488	0.09692007	0.137345	0.1518987
837.787	7	0.395221	0.3422403	0.1435841	0.1008446	0.132347	0.1556418
837.887	7	0.398972	0.3347002	0.1428748	0.1019808	0.133017	0.1540298
837.987	7	0.397153	0.3334772	0.1455259	0.1010658	0.13324	0.1544524
838.087	7	0.389351	0.3315612	0.1423405	0.09917395	0.136259	0.1529837
838.187	7	0.407007	0.3355795	0.1408739	0.1018537	0.135841	0.1496858
838.287	7	0.408615	0.33681	0.1383684	0.1037604	0.135681	0.1487746
838.387	7	0.397594	0.3166899	0.1436598	0.1056728	0.138184	0.1488263
838.487	7	0.391984	0.3145122	0.1394475	0.1058659	0.13678	0.1501829
838.587	7	0.388901	0.3179289	0.1405248	0.106349	0.13782	0.1514193
838.687	7	0.414761	0.3079865	0.1407394	0.1016159	0.135259	0.152447
838.787	7	0.408927	0.3013197	0.1428638	0.1036872	0.135547	0.1537456
838.887	7	0.404208	0.318043	0.1389433	0.1024805	0.134891	0.153669
838.987	7	0.403715	0.3266575	0.1372755	0.1037639	0.132595	0.1572831
839.087	7	0.395943	0.3317007	0.1410817	0.105031	0.135709	0.156106
839.187	7	0.384338	0.3456885	0.1386995	0.1074631	0.136986	0.159027
839.287	7	0.393806	0.3368855	0.1351802	0.108888	0.134306	0.1591509
839.401	7.5	0.375248	0.3397636	0.1349782	0.1117583	0.132865	0.160706
839.501	7.5	0.388946	0.3441803	0.1393516	0.1085439	0.13193	0.1580285
839.601	7.5	0.381052	0.344486	0.1409061	0.1105856	0.130272	0.1556333
839.701	7.5	0.409172	0.3493032	0.1378486	0.1133048	0.13043	0.1551523
839.801	7.5	0.4067	0.3439791	0.1356444	0.1123536	0.129896	0.1570916
839.901	7.5	0.419085	0.3335674	0.1400388	0.1121199	0.129184	0.1582193
840.001	7.5	0.41563	0.336357	0.1348007	0.1101393	0.130841	0.1600266
840.101	7.5	0.404989	0.3371821	0.1383103	0.1096333	0.12593	0.1587161
840.201	7.5	0.407354	0.3389555	0.134157	0.1140648	0.12855	0.159564
840.301	7.5	0.405162	0.3406676	0.1433689	0.114151	0.128159	0.1630131
840.401	7.5	0.41268	0.3413993	0.1368904	0.1138201	0.1296	0.1598666
840.501	7.5	0.412644	0.3387036	0.1391278	0.1073825	0.128846	0.1579635
840.601	7.5	0.412233	0.3407019	0.1489637	0.1048313	0.1284	0.159193
840.701	7.5	0.419621	0.3403937	0.1454836	0.1044482	0.128751	0.1613778
840.801	7.5	0.400991	0.334601	0.1421698	0.1035365	0.132128	0.1608548
840.901	7.5	0.429149	0.3309022	0.1430335	0.1061933	0.133539	0.1553773
841.001	7.5	0.428297	0.330836	0.1479306	0.1075587	0.132034	0.1576046
841.101	7.5	0.432009	0.3350139	0.1488106	0.1108973	0.131506	0.1554221
841.201	7.5	0.427686	0.3361713	0.1467779	0.109612	0.13198	0.1541348
841.301	7.5	0.421175	0.3133073	0.1478774	0.1079291	0.132033	0.1560505
841.401	7.5	0.405463	0.3116271	0.1469768	0.1064362	0.13403	0.1540979
841.501	7.5	0.421898	0.3158927	0.1480091	0.1055852	0.134807	0.1587219
841.601	7.5	0.425129	0.3072919	0.1502629	0.104838	0.134146	0.1620482
841.701	7.5	0.468675	0.2963409	0.1474811	0.1059735	0.133343	0.152706
841.801	7.5	0.431336	0.3263916	0.1492093	0.1035051	0.129171	0.1536948
841.901	7.5	0.409863	0.3211038	0.1483989	0.1052227	0.128539	0.1536049
842.001	7.5	0.40246	0.3397076	0.1494182	0.1076544	0.133397	0.1516697
842.101	7.5	0.418744	0.3452586	0.1470515	0.1042738	0.132956	0.1526532
842.201	7.5	0.407028	0.3364502	0.13899	0.101938	0.134038	0.1499807
842.301	7.5	0.421215	0.3346483	0.1424135	0.1042028	0.134574	0.1554912
842.401	7.5	0.405673	0.3350883	0.14334	0.1017291	0.135122	0.1547504
842.501	7.5	0.406137	0.3362547	0.1495484	0.1009941	0.13642	0.1529823
842.601	7.5	0.396605	0.3396509	0.1470834	0.102228	0.133382	0.1545947
842.701	7.5	0.388014	0.3423485	0.1481604	0.1012028	0.137466	0.1578287
842.801	7.5	0.400357	0.3450795	0.1496838	0.1011045	0.133801	0.1579825
842.901	7.5	0.420048	0.3479954	0.1525246	0.1047472	0.133093	0.1577123
843.001	7.5	0.436793	0.3369482	0.152293	0.1068397	0.138001	0.1544065

843.101	7.5	0.43655	0.3358511	0.1513983	0.1075101	0.1364	0.156818
843.201	7.5	0.410788	0.3352892	0.1527959	0.1048531	0.136238	0.1537462
843.301	7.5	0.409795	0.3382086	0.1530193	0.1067705	0.138921	0.1560925
843.401	7.5	0.40583	0.3390332	0.1515323	0.1075617	0.140183	0.1557671
843.501	7.5	0.414906	0.3407052	0.1483273	0.105666	0.139935	0.153708
843.601	7.5	0.424981	0.3385549	0.1492993	0.0993369	0.138836	0.1530875
843.701	7.5	0.410462	0.3393723	0.148518	0.1042581	0.141809	0.1458457
843.801	7.5	0.40555	0.3387257	0.1466745	0.108504	0.143104	0.1482189
843.901	7.5	0.411051	0.3359346	0.1489019	0.1051492	0.14346	0.1521993
844.001	7.5	0.404363	0.3321492	0.1531483	0.1065917	0.142522	0.1535666
844.101	7.5	0.387996	0.327861	0.1500584	0.1045334	0.142673	0.1553622
844.201	7.5	0.398201	0.3328474	0.1501903	0.1058932	0.144663	0.1567153
844.301	7.5	0.404901	0.3388101	0.1468128	0.1038482	0.143893	0.1522013
844.401	7.5	0.394836	0.3212431	0.1509029	0.1065986	0.142448	0.1547081
844.501	7.5	0.411358	0.3144711	0.1466224	0.1123813	0.141721	0.1487777
844.601	7.5	0.407378	0.3164212	0.1460658	0.1055946	0.142119	0.1500427
844.701	7.5	0.400658	0.3080407	0.1456964	0.1025471	0.14115	0.1522063
844.801	7.5	0.400793	0.3048719	0.1435414	0.1025339	0.139491	0.1512617
844.901	7.5	0.399339	0.3067954	0.1469645	0.1018394	0.141897	0.1539661
845.001	7.5	0.414061	0.3258507	0.145269	0.101278	0.143456	0.1538145
845.101	7.5	0.412589	0.3317378	0.1393087	0.1006951	0.143804	0.1541982
845.201	7.5	0.411488	0.3455258	0.1439726	0.09867948	0.146685	0.1526004
845.301	7.5	0.39582	0.3401241	0.1449572	0.09964532	0.144093	0.1492508
845.401	7.5	0.403075	0.3362364	0.1497373	0.09913747	0.139535	0.1495057
845.501	7.5	0.415746	0.3327939	0.1545495	0.1037887	0.137265	0.146228
845.601	7.5	0.390716	0.3360397	0.1542813	0.09978564	0.139228	0.1506695
845.701	7.5	0.402782	0.3376808	0.1498372	0.09934178	0.140406	0.1502431
845.801	7.5	0.395278	0.3429793	0.1469668	0.09965944	0.140472	0.150072
845.901	7.5	0.388886	0.3431169	0.1515939	0.09852751	0.139105	0.1520114
846.001	7.5	0.403369	0.3464367	0.1534062	0.09681305	0.139742	0.1535227
846.101	7.5	0.397613	0.3474361	0.1498526	0.09815201	0.135134	0.1543622
846.201	7.5	0.385261	0.3368064	0.1516174	0.1008056	0.139279	0.1518665
846.301	7.5	0.3921	0.3374508	0.1522427	0.103282	0.141908	0.1523675
846.401	7.5	0.393071	0.3387534	0.1568363	0.1009064	0.137634	0.1482705
846.501	7.5	0.4098	0.3402639	0.1572893	0.1002779	0.138568	0.1540647
846.601	7.5	0.414747	0.3431731	0.1530087	0.09913813	0.137107	0.1549742
846.701	7.5	0.421384	0.343147	0.1551874	0.1032305	0.139695	0.1599215
846.801	7.5	0.408612	0.341988	0.151963	0.1040183	0.137086	0.1571101
846.901	7.5	0.407096	0.3440119	0.1498102	0.1074143	0.135518	0.1539822
847.001	7.5	0.402893	0.3429724	0.1496194	0.1093858	0.135441	0.1519982
847.101	7.5	0.399158	0.3385906	0.1490379	0.1101138	0.134841	0.1528056
847.201	7.5	0.399581	0.3356666	0.1494162	0.1158276	0.13687	0.1537657
847.301	7.5	0.413704	0.3312229	0.1486426	0.1132395	0.135171	0.1536908
847.401	7.5	0.384737	0.336155	0.1467772	0.1132335	0.135222	0.1515495
847.501	7.5	0.400038	0.3331585	0.1451292	0.113439	0.133674	0.1499831
847.601	7.5	0.406256	0.3149878	0.1431612	0.1141657	0.130705	0.1484343
847.701	7.5	0.420555	0.3071277	0.1420759	0.1141783	0.13494	0.1458093
847.801	7.5	0.41886	0.314511	0.1476956	0.1129502	0.131475	0.1492566
847.901	7.5	0.415442	0.3037845	0.1477504	0.1139511	0.129194	0.140355
848.001	7.5	0.441707	0.2966333	0.145404	0.1113847	0.127697	0.1435193
848.101	7.5	0.425091	0.3161606	0.1431932	0.1114892	0.126057	0.1472951
848.201	7.5	0.410642	0.3187408	0.138884	0.1135959	0.123495	0.1454569
848.301	7.5	0.433332	0.3329486	0.1357754	0.1141981	0.12598	0.14767
848.401	7.5	0.437561	0.3452843	0.1416014	0.1148522	0.125991	0.1514094
848.501	7.5	0.438448	0.3350839	0.1387537	0.1098381	0.124352	0.1542355
848.601	7.5	0.433492	0.3369342	0.1381363	0.1049972	0.12636	0.1537413
848.701	7.5	0.434637	0.3351083	0.1399048	0.1029619	0.125138	0.1562019
848.801	7.5	0.418023	0.3384854	0.1397128	0.1050211	0.128168	0.1532757
848.901	7.5	0.442558	0.3424185	0.1312757	0.1065396	0.128116	0.1537465
849.001	7.5	0.439293	0.3439453	0.1355782	0.1067969	0.128835	0.1535486
849.101	7.5	0.433018	0.3467503	0.1353538	0.1088601	0.127772	0.1541151
849.201	7.5	0.448571	0.354286	0.1350994	0.109322	0.126389	0.1502874
849.301	7.5	0.431365	0.3437863	0.1319129	0.1072394	0.128833	0.1492701
849.401	7.5	0.421236	0.3381757	0.1357146	0.1051841	0.13101	0.1491168
849.501	7.5	0.420678	0.3382981	0.1396765	0.1060733	0.13294	0.1504307
849.601	7.5	0.426819	0.3403574	0.1369572	0.1043977	0.132083	0.1487152
849.701	7.5	0.436244	0.3418175	0.1331833	0.1045273	0.130794	0.1525283
849.801	7.5	0.425465	0.3421428	0.1380038	0.1034846	0.130219	0.1518601
849.901	7.5	0.402246	0.3442889	0.1346883	0.1025584	0.131918	0.15417
850.001	7.5	0.39939	0.342409	0.1343724	0.1050187	0.135051	0.1537825
850.101	7.5	0.41105	0.3425657	0.1383677	0.1030088	0.134655	0.156581
850.201	7.5	0.409675	0.340427	0.1389351	0.1029211	0.135456	0.158273
850.301	7.5	0.418434	0.333039	0.1370996	0.1061438	0.131547	0.1581453
850.401	7.5	0.407061	0.3291089	0.1364654	0.1031072	0.126085	0.1571389
850.501	7.5	0.405796	0.3300663	0.1408962	0.1025631	0.128242	0.1541445
850.601	7.5	0.401779	0.3377395	0.1445358	0.1050572	0.132746	0.1546438
850.701	7.5	0.397347	0.323522	0.1438804	0.1023718	0.13214	0.1548888
850.801	7.5	0.402502	0.3106348	0.1400926	0.09864292	0.132055	0.155209
850.901	7.5	0.412342	0.30628	0.1396178	0.100703	0.133426	0.157575
851.001	7.5	0.427994	0.3085037	0.1430272	0.1041109	0.13324	0.1572674
851.101	7.5	0.425555	0.3026023	0.1437549	0.1054949	0.134973	0.1592713
851.201	7.5	0.405475	0.3000298	0.142131	0.1009633	0.131589	0.1609582
851.301	7.5	0.407197	0.3221917	0.1438742	0.1035309	0.135755	0.1608387
851.401	7.5	0.400899	0.3217182	0.144673	0.1070297	0.132214	0.1609915
851.501	7.5	0.413253	0.3418369	0.1448541	0.1034477	0.133826	0.1604265
851.601	7.5	0.422692	0.3421673	0.1485133	0.09994617	0.136916	0.1596725
851.701	7.5	0.416903	0.3375407	0.1466666	0.1051927	0.137748	0.1589521
851.801	7.5	0.401848	0.3352544	0.148359	0.1088368	0.136181	0.1512589
851.901	7.5	0.418963	0.3362865	0.1476775	0.1046791	0.137962	0.1519726
852.001	7.5	0.400371	0.3353866	0.1480861	0.1046543	0.13916	0.1498137
852.101	7.5	0.383935	0.3423641	0.1465972	0.102846	0.138923	0.1508378
852.201	7.5	0.395138	0.3451486	0.1370785	0.1042638	0.138267	0.1525489
852.301	7.5	0.398997	0.3473927	0.1418291	0.1037761	0.139704	0.1509063
852.401	7.5	0.394431	0.3521719	0.1462109	0.1055792	0.140642	0.1552121
852.501	7.5	0.398312	0.3383812	0.1508203	0.1118458	0.141272	0.1592391

852.601	7.5	0.398269	0.3346948	0.1482671	0.1060816	0.139825	0.1514788
852.701	7.5	0.402487	0.3351268	0.1480782	0.1035262	0.13898	0.1512459
852.801	7.5	0.401054	0.3376734	0.146261	0.1025716	0.139896	0.1520528
852.901	7.5	0.39542	0.3394601	0.1493419	0.1052499	0.138113	0.1518836
853.001	7.5	0.404381	0.3404786	0.1503163	0.1050792	0.142044	0.1526877
853.101	7.5	0.405621	0.3396295	0.1477484	0.1061494	0.140747	0.1541942
853.201	7.5	0.409111	0.3403487	0.1479718	0.1043916	0.139072	0.154697
853.301	7.5	0.403837	0.3379936	0.1489989	0.1016283	0.135437	0.1507741
853.401	7.5	0.395393	0.3341622	0.149219	0.1010951	0.137337	0.1505424
853.501	7.5	0.400217	0.3324319	0.1469921	0.1021474	0.13943	0.1512701
853.601	7.5	0.389056	0.3272844	0.1491938	0.09806428	0.142106	0.1520631
853.701	7.5	0.402465	0.333967	0.1471603	0.1015892	0.138771	0.1542709
853.801	7.5	0.392864	0.3378856	0.1461027	0.1014222	0.144169	0.1570237
853.901	7.5	0.405946	0.3181057	0.1486298	0.1015579	0.145735	0.1520095
854.001	7.5	0.412455	0.3115568	0.1500892	0.09940949	0.142471	0.1557691
854.101	7.5	0.3973	0.3179005	0.1473538	0.09878474	0.140119	0.152941
854.201	7.5	0.38495	0.3069811	0.1488094	0.1028023	0.138672	0.1532037
854.301	7.5	0.388704	0.3061892	0.1467089	0.1023358	0.137119	0.1538291
854.401	7.5	0.394031	0.309713	0.1506703	0.1028692	0.139726	0.1529073
854.501	7.5	0.406222	0.3217076	0.1466944	0.103861	0.138287	0.1521682
854.601	7.5	0.410046	0.3229684	0.1452518	0.1006909	0.139789	0.1431644
854.701	7.5	0.42171	0.3414096	0.1472071	0.1023972	0.134835	0.1455753
854.801	7.5	0.407493	0.3375437	0.1435274	0.1027507	0.137734	0.1494366
854.901	7.5	0.407765	0.3343191	0.1481571	0.106516	0.135848	0.1540131
855.001	7.5	0.400124	0.333692	0.1455922	0.1087416	0.135148	0.1550006
855.101	7.5	0.404484	0.3362575	0.1399227	0.108217	0.13654	0.1575278
855.201	7.5	0.398111	0.3431489	0.1471	0.1113402	0.137568	0.1521787
855.301	7.5	0.403775	0.348563	0.1448526	0.1093859	0.138353	0.1513368
855.401	7.5	0.38129	0.3483718	0.1489372	0.1093343	0.13526	0.1475592
855.501	7.5	0.407622	0.3556047	0.1530513	0.1134378	0.136466	0.1481562
855.601	7.5	0.406164	0.3483929	0.1549366	0.1134441	0.135666	0.1515756
855.701	7.5	0.415389	0.3379502	0.1501611	0.1139205	0.137682	0.1513795
855.801	7.5	0.416454	0.3404877	0.1482751	0.1136147	0.13852	0.1522077
855.901	7.5	0.418258	0.3439623	0.1531111	0.1136317	0.135	0.1505428
856.001	7.5	0.416779	0.3470443	0.1510034	0.1121718	0.134842	0.1529142
856.101	7.5	0.41275	0.3540271	0.1475329	0.1104497	0.135634	0.1542754
856.201	7.5	0.410336	0.3579576	0.1493802	0.1129567	0.13467	0.1489297
856.301	7.5	0.409539	0.3505994	0.1488469	0.1117854	0.13844	0.1497444
856.401	7.5	0.433603	0.3537961	0.1547317	0.1125734	0.135425	0.1468968
856.501	7.5	0.439195	0.3514792	0.1536644	0.1100928	0.133093	0.1503125
856.601	7.5	0.424508	0.3464349	0.1509394	0.1048894	0.131264	0.1486763
856.701	7.5	0.42912	0.3429213	0.1500281	0.1050035	0.131187	0.1503565
856.801	7.5	0.400988	0.3424304	0.1458894	0.1052641	0.127879	0.1496854
856.901	7.5	0.422107	0.3429881	0.1448285	0.1045716	0.129418	0.1523554
857.001	7.5	0.419034	0.3309119	0.1462068	0.1061271	0.132081	0.1512937
857.101	7.5	0.430629	0.3221825	0.1454675	0.1101523	0.13238	0.1503511
857.201	7.5	0.420152	0.3236276	0.1478599	0.1094298	0.131049	0.1505364
857.301	7.5	0.428434	0.3222086	0.146407	0.1080189	0.129332	0.1492752
857.401	7.5	0.418964	0.3136136	0.1444546	0.1059182	0.130477	0.1546256
857.501	7.5	0.42021	0.3230807	0.1423358	0.1042866	0.130361	0.1537473
857.601	7.5	0.416233	0.3542602	0.1408529	0.1033721	0.13008	0.1591716
857.701	7.5	0.433237	0.3578867	0.1406337	0.1048948	0.130482	0.1561349
857.801	7.5	0.418678	0.3685265	0.1439507	0.1039388	0.127377	0.1541997
857.901	7.5	0.400701	0.3627065	0.141154	0.1021531	0.130336	0.1521017
858.001	7.5	0.391867	0.3551702	0.1411269	0.1056055	0.131489	0.1519925
858.101	7.5	0.403712	0.3602585	0.1405016	0.1046027	0.133054	0.1540566
858.201	7.5	0.407294	0.3626726	0.1400652	0.1032007	0.131192	0.152883
858.301	7.5	0.41093	0.3679954	0.1388163	0.104417	0.131205	0.1523231
858.401	7.5	0.407941	0.3750036	0.1440018	0.1029718	0.132562	0.1522479
858.501	7.5	0.397707	0.3760703	0.1401339	0.1021273	0.132817	0.1519172
858.601	7.5	0.394687	0.3568346	0.1396416	0.1039852	0.133065	0.1476895
858.701	7.5	0.388642	0.3562043	0.1416121	0.1023346	0.134921	0.1495978
858.801	7.5	0.387048	0.3502542	0.1400298	0.1023291	0.134089	0.1377658
858.901	7.5	0.405124	0.3516741	0.1319127	0.103727	0.131163	0.1452517
859.001	7.5	0.430195	0.3591224	0.1397621	0.1029167	0.126323	0.1481878
859.101	7.5	0.430149	0.3672883	0.1401815	0.1055716	0.128477	0.1444943
859.201	7.5	0.405552	0.3735681	0.1400105	0.1023796	0.130581	0.1463144
859.301	7.5	0.409871	0.3772754	0.1353533	0.1045948	0.131113	0.1496542
859.401	7.5	0.404008	0.3797863	0.1393303	0.1049011	0.129809	0.1521377
859.501	7.5	0.41726	0.380083	0.1421583	0.1030685	0.13474	0.1516395
859.601	7.5	0.421764	0.3785776	0.1381741	0.09991099	0.132347	0.1542358
859.701	7.5	0.412372	0.3740426	0.1338288	0.1021673	0.131802	0.1527813
859.801	7.5	0.395259	0.3655756	0.1364012	0.1044445	0.132109	0.1521497
859.901	7.5	0.412969	0.3568648	0.1346243	0.1025834	0.137323	0.1520035
860.001	7.5	0.396244	0.3501382	0.1338661	0.1033852	0.135083	0.1538613
860.101	7.5	0.384267	0.3462497	0.1400865	0.100509	0.136902	0.1505159
860.201	7.5	0.387652	0.3313411	0.1395268	0.1003344	0.140264	0.1505223
860.301	7.5	0.394481	0.3268973	0.1381669	0.1028717	0.139453	0.1471909
860.401	7.5	0.394079	0.3335269	0.1380174	0.106369	0.137279	0.1481768
860.501	7.5	0.399095	0.3134133	0.1439784	0.106711	0.135984	0.1456779
860.601	7.5	0.40766	0.3109211	0.1477805	0.1028693	0.137307	0.1514195
860.701	7.5	0.411248	0.3237557	0.14589	0.1024471	0.135019	0.1556293
860.801	7.5	0.404011	0.3398607	0.1422022	0.1012517	0.134657	0.1554951
860.901	7.5	0.394224	0.3450365	0.1434028	0.1029882	0.133986	0.1593989
861.001	7.5	0.405152	0.3478964	0.1443087	0.1047766	0.138505	0.1563832
861.101	7.5	0.395621	0.3405627	0.1470957	0.1042711	0.135169	0.1550302
861.201	7.5	0.400421	0.3424008	0.147199	0.1022418	0.135624	0.1559609
861.301	7.5	0.390835	0.3419258	0.146359	0.1031035	0.133506	0.1563906
861.401	7.5	0.38622	0.3466535	0.1433739	0.1026787	0.137497	0.158301
861.501	7.5	0.39319	0.3497292	0.1454991	0.1045936	0.138438	0.1567956
861.601	7.5	0.387208	0.3549762	0.1477494	0.1018418	0.138867	0.1599472
861.701	7.5	0.397838	0.3498277	0.1452878	0.1006765	0.137935	0.1617952
861.801	7.5	0.394994	0.3535951	0.1478755	0.09988405	0.135924	0.1594198
861.901	7.5	0.391372	0.3477013	0.1473319	0.1007236	0.135262	0.1576759
862.001	7.5	0.402105	0.3394884	0.147446	0.1001795	0.137734	0.1573527

862.101	7.5	0.386379	0.3406537	0.1452095	0.0995929	0.139335	0.1602098
862.201	7.5	0.389334	0.3440053	0.1429837	0.1053021	0.14019	0.156103
862.301	7.5	0.392636	0.3448721	0.1480085	0.1058843	0.14101	0.1567037
862.401	7.5	0.388462	0.3484958	0.1488992	0.1059989	0.140525	0.1546131
862.501	7.5	0.407982	0.3475982	0.1561362	0.1057926	0.140532	0.152713
862.601	7.5	0.408155	0.3464325	0.1525342	0.1014919	0.135341	0.1547866
862.701	7.5	0.419472	0.3473481	0.1518821	0.1022417	0.134853	0.1539143
862.801	7.5	0.402737	0.3448926	0.1544397	0.1044531	0.135544	0.1557117
862.901	7.5	0.405565	0.338655	0.1548644	0.1079853	0.132792	0.1572868
863.001	7.5	0.397368	0.3361815	0.1531069	0.1084074	0.136168	0.1480734
863.101	7.5	0.396656	0.3325483	0.1498605	0.1118844	0.135838	0.153911
863.201	7.5	0.389977	0.3365522	0.1501516	0.1176824	0.134546	0.1536767
863.301	7.5	0.404608	0.3419965	0.1498182	0.1161974	0.13465	0.1525336
863.401	7.5	0.372285	0.3245533	0.1479847	0.1164681	0.138652	0.1529898
863.501	7.5	0.393783	0.31609	0.1468499	0.1173322	0.137275	0.1548
863.601	7.5	0.397612	0.3134314	0.1488175	0.116325	0.136926	0.15147
863.701	7.5	0.402551	0.3061997	0.148429	0.117722	0.137138	0.1509259
863.801	7.5	0.404454	0.3023064	0.1473683	0.1179273	0.137294	0.1513348
863.901	7.5	0.412798	0.3338003	0.1495617	0.1169229	0.13644	0.1525393
864.001	7.5	0.40765	0.3323	0.152332	0.1133733	0.135196	0.1535708
864.101	7.5	0.405463	0.3421328	0.1474768	0.1135692	0.134137	0.1510155
864.201	7.5	0.401179	0.346578	0.1457977	0.1139754	0.129866	0.1508365
864.301	7.5	0.394735	0.3368873	0.1446822	0.112661	0.130463	0.1526588
864.401	7.5	0.411453	0.3362873	0.1486462	0.113209	0.132453	0.1495488
864.501	7.5	0.409794	0.3369164	0.1470083	0.108083	0.130752	0.1502995
864.601	7.5	0.406482	0.3413672	0.1437402	0.1067525	0.13133	0.148099
864.701	7.5	0.405889	0.344597	0.1439714	0.1066433	0.133356	0.1482747
864.801	7.5	0.387893	0.3475344	0.1407314	0.1032536	0.134099	0.1418472
864.901	7.5	0.409202	0.3489683	0.1461029	0.1056571	0.134983	0.1472978
865.001	7.5	0.398972	0.3520668	0.1454783	0.1073409	0.13134	0.147708
865.101	7.5	0.405449	0.3407367	0.1383243	0.1124546	0.131957	0.1521554
865.201	7.5	0.407089	0.3362764	0.1462543	0.109434	0.131977	0.152266
865.301	7.5	0.414568	0.3367311	0.141696	0.10771	0.131491	0.1524877
865.401	7.5	0.400533	0.3402292	0.1439553	0.1065928	0.127723	0.1511507
865.501	7.5	0.404757	0.340775	0.1470187	0.1062186	0.128883	0.1507789
865.601	7.5	0.407584	0.3434405	0.1458683	0.1068476	0.133541	0.1463215
865.701	7.5	0.421069	0.3419654	0.142422	0.1082547	0.131798	0.1505135
865.801	7.5	0.414426	0.3431131	0.1422086	0.1074356	0.12806	0.1512977
865.901	7.5	0.395614	0.340877	0.1487	0.1067686	0.128981	0.1512026
866.001	7.5	0.39553	0.3420507	0.1459392	0.1078125	0.13215	0.150479
866.101	7.5	0.394595	0.3354757	0.1441865	0.1063587	0.130825	0.1507597
866.201	7.5	0.394702	0.3321409	0.1452896	0.1044397	0.132757	0.1511826
866.301	7.5	0.402078	0.3321613	0.1474016	0.1056484	0.129088	0.1474033
866.401	7.5	0.395228	0.3412426	0.1518382	0.1035226	0.130493	0.1496901
866.501	7.5	0.382639	0.3356409	0.1505184	0.1020665	0.131774	0.1496988
866.601	7.5	0.382788	0.3140101	0.1511531	0.1057903	0.132116	0.1501886
866.701	7.5	0.375457	0.3159388	0.1493529	0.1080234	0.132116	0.1502312
866.801	7.5	0.385373	0.3102439	0.1451375	0.1057092	0.1321	0.1504154
866.901	7.5	0.410053	0.3004825	0.1456857	0.1078051	0.131774	0.1514318
867.001	7.5	0.432319	0.3138921	0.1421898	0.1075614	0.130316	0.1515049
867.101	7.5	0.431558	0.3310113	0.1441907	0.1087139	0.130503	0.1508034
867.201	7.5	0.398529	0.3327323	0.1474949	0.1061163	0.131568	0.1496933
867.301	7.5	0.413679	0.3418497	0.1439884	0.1054984	0.133036	0.1512139
867.401	7.5	0.392065	0.344044	0.1429142	0.1062088	0.132782	0.1533513
867.501	7.5	0.405563	0.3378702	0.1405311	0.1058891	0.128204	0.1546528
867.601	7.5	0.424364	0.3387871	0.1404193	0.1025351	0.124697	0.157618
867.701	7.5	0.413122	0.3408192	0.1416847	0.1027096	0.128457	0.1537376
867.801	7.5	0.417742	0.3438236	0.1432657	0.1052983	0.131727	0.153694
867.901	7.5	0.428022	0.3497414	0.1409509	0.1040454	0.130933	0.1522649
868.001	7.5	0.409564	0.3491718	0.1425758	0.1056572	0.130103	0.1512898
868.101	7.5	0.385811	0.3512857	0.1396838	0.104081	0.135327	0.153068
868.201	7.5	0.398139	0.3485743	0.1392717	0.1063338	0.136803	0.1511095
868.301	7.5	0.403945	0.3381771	0.139711	0.1065938	0.13414	0.1480728
868.401	7.5	0.393714	0.3389805	0.14462	0.1071233	0.134682	0.1468857
868.501	7.5	0.398003	0.340659	0.1408684	0.1104205	0.131821	0.1459842
868.601	7.5	0.39636	0.3420423	0.1397923	0.1024595	0.128525	0.1448732
868.701	7.5	0.401633	0.3450919	0.1414319	0.1000165	0.13118	0.1437809
868.801	7.5	0.393299	0.3450672	0.1411043	0.1024836	0.131597	0.1453681
868.901	7.5	0.389593	0.3422122	0.1337059	0.1054775	0.133305	0.145544
869.001	7.5	0.407575	0.3443173	0.1417765	0.105869	0.134191	0.1464721
869.101	7.5	0.401955	0.3449099	0.1414661	0.1028665	0.133776	0.1499126
869.201	7.5	0.403999	0.3410901	0.1382864	0.1032539	0.133673	0.1494495
869.301	7.5	0.394002	0.3374044	0.1322666	0.1017379	0.135868	0.1481868
869.401	7.5	0.400025	0.3335555	0.1321981	0.1033026	0.133871	0.1517333
869.501	7.5	0.40587	0.3378008	0.1371399	0.1041707	0.13491	0.1513216
869.601	7.5	0.395714	0.3379163	0.1348401	0.1001648	0.136594	0.1494909
869.701	7.5	0.405473	0.3294965	0.1326765	0.09934402	0.135041	0.151405
869.801	7.5	0.39032	0.3187906	0.1349044	0.09901343	0.137372	0.1488718
869.901	7.5	0.396669	0.3272706	0.136004	0.1010298	0.136739	0.1504647
870.001	7.5	0.408454	0.3093158	0.1351918	0.1005646	0.137067	0.1486212
870.101	7.5	0.387407	0.3068261	0.1418595	0.101825	0.136313	0.1505132
870.201	7.5	0.387269	0.3310673	0.1415784	0.1052391	0.138438	0.1487718
870.301	7.5	0.384207	0.3436174	0.1399077	0.1042773	0.14099	0.1502303
870.401	7.5	0.383743	0.3507801	0.139732	0.1062216	0.140324	0.1488136
870.501	7.5	0.39884	0.3540152	0.1445729	0.1056056	0.139122	0.1489731
870.601	7.5	0.402896	0.3467695	0.1469855	0.1031332	0.1411	0.1486817
870.701	7.5	0.406951	0.3487709	0.1452337	0.1042564	0.140621	0.152799
870.801	7.5	0.405102	0.3526874	0.1406382	0.10285	0.138855	0.1497017
870.901	7.5	0.398313	0.3568387	0.1412233	0.1026433	0.14013	0.1529831
871.001	7.5	0.382194	0.3618832	0.144498	0.1038891	0.140685	0.1493795
871.101	7.5	0.393574	0.3670703	0.1444031	0.1051817	0.139309	0.1515527
871.201	7.5	0.384354	0.3543862	0.1445285	0.1138986	0.139429	0.1556331
871.301	7.5	0.386549	0.3557967	0.1447702	0.1129958	0.137626	0.1551023
871.401	7.5	0.366497	0.3480409	0.142005	0.1142121	0.135058	0.1571084
871.501	7.5	0.378565	0.347327	0.14359	0.1119962	0.136944	0.1555018

871.601	7.5	0.381663	0.3496304	0.1461409	0.1122636	0.138842	0.1577087
871.701	7.5	0.393777	0.3553108	0.143479	0.1125565	0.137137	0.1583706
871.801	7.5	0.394707	0.3591379	0.1455576	0.1118308	0.138247	0.1548063
871.901	7.5	0.40371	0.3632697	0.1471188	0.1120891	0.13621	0.155669
872.001	7.5	0.403968	0.3647359	0.1488196	0.1104092	0.135947	0.1578626
872.101	7.5	0.40165	0.3629579	0.1449172	0.1103018	0.133242	0.1589191
872.201	7.5	0.398164	0.3632021	0.1423075	0.1140958	0.131123	0.156057
872.301	7.5	0.392289	0.3606718	0.1440389	0.1112073	0.129236	0.1554645
872.401	7.5	0.410508	0.3523402	0.1451577	0.1131779	0.130954	0.1535343
872.501	7.5	0.405311	0.3452806	0.150978	0.1086601	0.130679	0.1548296
872.601	7.5	0.406547	0.3434551	0.1489098	0.1079571	0.130287	0.1564181
872.701	7.5	0.407835	0.3484851	0.1476146	0.1060953	0.128046	0.1551683
872.801	7.5	0.385553	0.3396076	0.1486685	0.1034246	0.12708	0.1551879
872.901	7.5	0.407587	0.3225272	0.1477878	0.1038523	0.128955	0.1540781
873.001	7.5	0.399702	0.3281456	0.1461526	0.1051344	0.127717	0.1470854
873.101	7.5	0.409259	0.3104104	0.1445381	0.1099793	0.125342	0.1517248
873.201	7.5	0.400617	0.3033375	0.1457622	0.1084118	0.125883	0.149942
873.301	7.5	0.408815	0.3167258	0.1469362	0.1054667	0.12556	0.1469157
873.401	7.5	0.397638	0.3452505	0.1453903	0.1047025	0.122169	0.1488935
873.501	7.5	0.397417	0.3442905	0.1464705	0.1046666	0.126003	0.1526532
873.601	7.5	0.412489	0.3558861	0.150645	0.1024241	0.127127	0.1494355
873.701	7.5	0.407505	0.3461876	0.1500869	0.1017611	0.125527	0.1482725
873.801	7.5	0.418013	0.3391801	0.1494527	0.1028892	0.125115	0.1490572
873.901	7.5	0.391886	0.3420859	0.1501411	0.103872	0.126368	0.1499795
874.001	7.5	0.399531	0.3455621	0.1511017	0.104657	0.127911	0.1510191
874.101	7.5	0.40423	0.3489872	0.1480305	0.1039037	0.126809	0.1502786
874.201	7.5	0.41803	0.3593361	0.1482958	0.1024444	0.127558	0.1483399
874.301	7.5	0.40808	0.3552433	0.1464354	0.1033555	0.127412	0.1475157
874.401	7.5	0.403765	0.3526769	0.1490965	0.09774222	0.128263	0.1463702
874.501	7.5	0.383476	0.3544996	0.1459868	0.1002029	0.128025	0.1477824
874.601	7.5	0.376769	0.3398041	0.1438377	0.1049616	0.130321	0.1479838
874.701	7.5	0.38518	0.3375893	0.1445277	0.1038273	0.129049	0.147562
874.801	7.5	0.386797	0.3395956	0.1415729	0.1007706	0.12719	0.1398298
874.901	7.5	0.416798	0.343583	0.1471671	0.1029644	0.126103	0.1426974
875.001	7.5	0.433702	0.3455525	0.1455324	0.1046576	0.127179	0.1481154
875.101	7.5	0.430192	0.3478227	0.1395582	0.104484	0.128964	0.1515567
875.201	7.5	0.408867	0.3468383	0.1464867	0.1029233	0.129323	0.1523744
875.301	7.5	0.400108	0.347418	0.1438272	0.1040356	0.131701	0.152621
875.401	7.5	0.405353	0.347439	0.1467681	0.1063247	0.12828	0.151788
875.501	7.5	0.403108	0.3434548	0.1499123	0.1052221	0.128372	0.1489762
875.601	7.5	0.396941	0.3383944	0.1501716	0.09989072	0.13174	0.1486534
875.701	7.5	0.396809	0.3320345	0.1465454	0.1003525	0.130504	0.151049
875.801	7.5	0.393034	0.3359064	0.1456462	0.1027379	0.131103	0.1489227
875.901	7.5	0.416872	0.3422599	0.149019	0.1000089	0.133087	0.148853
876.001	7.5	0.39934	0.3270073	0.1492112	0.1014721	0.133746	0.1477709
876.101	7.5	0.382675	0.313017	0.1456465	0.1000916	0.133302	0.1491971
876.201	7.5	0.387926	0.3212844	0.1479765	0.1009361	0.131631	0.1479422
876.301	7.5	0.403932	0.305489	0.1483503	0.1006428	0.131921	0.1459957
876.401	7.5	0.400971	0.3022007	0.1558332	0.1041118	0.130455	0.1466877
876.501	7.5	0.412315	0.323961	0.1566518	0.1088614	0.132076	0.1479957
876.601	7.5	0.405997	0.3443085	0.1531118	0.1017902	0.134028	0.1473626
876.701	7.5	0.406719	0.3461705	0.1512026	0.09922048	0.133611	0.1496561
876.801	7.5	0.401513	0.3486478	0.150586	0.1007005	0.13631	0.1511591
876.901	7.5	0.393314	0.3397807	0.1504319	0.1025971	0.139239	0.1531661
877.001	7.5	0.400139	0.3385723	0.1471387	0.1027891	0.13752	0.1528693
877.101	7.5	0.404635	0.3386011	0.1476759	0.1030949	0.138714	0.1515156
877.201	7.5	0.409415	0.3438215	0.1498205	0.1022509	0.139874	0.1498127
877.301	7.5	0.394798	0.3466334	0.1462577	0.1030935	0.14252	0.1498155
877.401	7.5	0.388988	0.3528791	0.1460109	0.1025702	0.141933	0.1521281
877.501	7.5	0.388918	0.3493196	0.1469915	0.1033181	0.141746	0.1532954
877.601	7.5	0.38413	0.3535471	0.1459324	0.1011624	0.139674	0.1550763
877.701	7.5	0.399666	0.3466786	0.1427796	0.1000992	0.140358	0.151888
877.801	7.5	0.389008	0.3363592	0.1482156	0.0997504	0.140434	0.1508529
877.901	7.5	0.393046	0.3371944	0.1464884	0.1018628	0.141729	0.1517089
878.001	7.5	0.39696	0.3402573	0.1467681	0.1012542	0.140855	0.1523649
878.101	7.5	0.384989	0.3412367	0.145559	0.1000612	0.13825	0.1524172
878.201	7.5	0.383497	0.3429364	0.1460261	0.1025063	0.136001	0.1528593
878.301	7.5	0.382994	0.3440475	0.1458393	0.1036511	0.136895	0.1523886
878.401	7.5	0.383419	0.3417097	0.1504087	0.1068324	0.138606	0.1502256
878.501	7.5	0.396977	0.3441725	0.1458512	0.1066472	0.13684	0.1478362
878.601	7.5	0.403509	0.3434748	0.1444003	0.1027141	0.141799	0.1476922
878.701	7.5	0.405288	0.3377393	0.1462532	0.1035051	0.140909	0.140161
878.801	7.5	0.39961	0.3343747	0.1456799	0.1035135	0.136258	0.1449876
878.901	7.5	0.397859	0.3322404	0.1371817	0.1042273	0.135177	0.1478806
879.001	7.5	0.388174	0.3375665	0.1422995	0.104884	0.139523	0.1456312
879.101	7.5	0.389975	0.3411443	0.1409628	0.1075677	0.137748	0.1482018
879.201	7.5	0.379812	0.3103964	0.1398229	0.1110412	0.138041	0.1500096
879.301	7.5	0.387983	0.3154832	0.1358794	0.1087863	0.134602	0.1487337
879.401	7.5	0.364429	0.3185737	0.1360584	0.1087855	0.134859	0.1521742
879.501	7.5	0.3847	0.3107562	0.1392557	0.1074349	0.134948	0.1510486
879.601	7.5	0.388123	0.3009204	0.1376816	0.1050517	0.139375	0.1516223
879.701	7.5	0.400455	0.3369229	0.1316278	0.1084629	0.132721	0.1516816
879.801	7.5	0.39857	0.3396001	0.1355851	0.111089	0.134051	0.1488831
879.901	7.5	0.405278	0.3495718	0.1366798	0.1127266	0.132603	0.1522319
880.001	7.5	0.401235	0.3473752	0.1379126	0.1131597	0.131744	0.1506839
880.101	7.5	0.409876	0.3339109	0.1406561	0.1145711	0.1313	0.151174
880.201	7.5	0.396498	0.3397219	0.1429576	0.1165138	0.1326	0.1475745
880.301	7.5	0.395329	0.3389745	0.1416971	0.1158694	0.13063	0.1506649
880.401	7.5	0.409437	0.3433673	0.1431985	0.1169233	0.132889	0.1485718
880.501	7.5	0.411972	0.3468957	0.1488382	0.1107003	0.131911	0.1518405
880.601	7.5	0.412077	0.3502085	0.1510721	0.1066818	0.130741	0.151596
880.701	7.5	0.411801	0.3508738	0.1491614	0.1069573	0.130615	0.1551535
880.801	7.5	0.387795	0.3585651	0.1443597	0.1047916	0.130077	0.155023
880.901	7.5	0.410208	0.3414066	0.1444715	0.1047371	0.132239	0.15681
881.001	7.5	0.407233	0.338711	0.1462322	0.104151	0.130693	0.1550954

881.101	7.5	0.403972	0.3369659	0.1463493	0.1060429	0.12686	0.1556737
881.201	7.5	0.406755	0.3406487	0.1471646	0.1060485	0.126356	0.1566282
881.301	7.5	0.40887	0.3406095	0.1472601	0.1071649	0.127006	0.1568169
881.401	7.5	0.396525	0.3442396	0.1458316	0.107452	0.124241	0.1597165
881.501	7.5	0.404639	0.3426739	0.1473635	0.105273	0.125603	0.1567702
881.601	7.5	0.405112	0.3441386	0.1495709	0.1046035	0.12782	0.1571052
881.701	7.5	0.406851	0.3401534	0.1470229	0.1042049	0.128223	0.158048
881.801	7.5	0.407868	0.3421696	0.1488782	0.1044059	0.128451	0.1561413
881.901	7.5	0.396715	0.3372419	0.149093	0.1055852	0.129722	0.1567741
882.001	7.5	0.392645	0.3324374	0.1478958	0.1074901	0.130915	0.1563488
882.101	7.5	0.402538	0.3340211	0.1471016	0.1082548	0.128906	0.1575741
882.201	7.5	0.397683	0.3388256	0.1416098	0.1047662	0.128379	0.1533179
882.301	7.5	0.403902	0.322447	0.1442558	0.106222	0.126712	0.1539091
882.401	7.5	0.395866	0.3062031	0.1467649	0.1019258	0.129068	0.1526189
882.501	7.5	0.380315	0.3144752	0.1522381	0.1010072	0.131367	0.1526333
882.601	7.5	0.375831	0.3079144	0.1506729	0.1024543	0.134678	0.1553033
882.701	7.5	0.376006	0.3065183	0.148829	0.1040672	0.133462	0.1541425
882.801	7.5	0.380845	0.3139006	0.1485281	0.1009878	0.132271	0.1550944
882.901	7.5	0.399274	0.3417939	0.1484746	0.1013651	0.130424	0.1550904
883.001	7.5	0.420166	0.3482775	0.1485258	0.1002744	0.132162	0.1482819
883.101	7.5	0.417945	0.3479778	0.1470762	0.1021484	0.133246	0.1522782
883.201	7.5	0.406753	0.3420664	0.147342	0.09936875	0.134323	0.1491677
883.301	7.5	0.398847	0.3368302	0.1469893	0.1004637	0.130359	0.1477703
883.401	7.5	0.39177	0.3357737	0.1466129	0.1044881	0.124707	0.1509961
883.501	7.5	0.396408	0.3377418	0.1443679	0.1037278	0.124785	0.1545135
883.601	7.5	0.401863	0.3406712	0.1463927	0.09969446	0.130158	0.1522646
883.701	7.5	0.404071	0.3465436	0.1450948	0.1022541	0.130423	0.1516357
883.801	7.5	0.393475	0.346359	0.147119	0.1044758	0.131345	0.1516658
883.901	7.5	0.417041	0.3567272	0.1465321	0.100415	0.133545	0.1536516
884.001	7.5	0.410972	0.3523056	0.1485274	0.1016598	0.13533	0.1540661
884.101	7.5	0.397375	0.3392933	0.1474927	0.09804542	0.135215	0.1536366
884.201	7.5	0.407172	0.3383681	0.1484554	0.1005615	0.133406	0.1502689
884.301	7.5	0.41068	0.3401013	0.1477904	0.1036272	0.135352	0.1510908
884.401	7.5	0.413551	0.3403493	0.1514796	0.1047153	0.132113	0.1509688
884.501	7.5	0.465635	0.3415454	0.1484067	0.1093869	0.133367	0.1522697
884.601	7.5	0.472373	0.3434201	0.146742	0.1039047	0.134055	0.1511854
884.701	7.5	0.452028	0.3402332	0.1485889	0.1021454	0.134441	0.1515866
884.801	7.5	0.453344	0.3418004	0.145988	0.0998852	0.133131	0.1457872
884.901	7.5	0.451984	0.3424081	0.1518873	0.1000904	0.137155	0.1439206
885.001	7.5	0.449507	0.33628	0.1514275	0.09944623	0.137276	0.1481094
885.101	7.5	0.441714	0.3347605	0.1434009	0.09971072	0.141181	0.1541778
885.201	7.5	0.422208	0.3298275	0.1486771	0.1002719	0.14269	0.155981
885.301	7.5	0.408207	0.3322996	0.1468737	0.09990495	0.141769	0.1559736
885.401	7.5	0.402362	0.3350166	0.1499383	0.1019474	0.141172	0.15468
885.501	7.5	0.421285	0.3158277	0.1544009	0.1027313	0.140973	0.1540682
885.601	7.5	0.431802	0.3121483	0.1528851	0.1003913	0.139737	0.1496977
885.701	7.5	0.422595	0.3121215	0.1467248	0.09798378	0.141914	0.1520769
885.801	7.5	0.41284	0.3071995	0.1470397	0.09699076	0.140672	0.1534433
885.901	7.5	0.397266	0.3026906	0.1521963	0.09899385	0.143068	0.1527865
886.001	7.5	0.398211	0.3272833	0.1524858	0.1010292	0.14225	0.1525867
886.101	7.5	0.406271	0.3348888	0.1485655	0.101588	0.138864	0.1525398
886.201	7.5	0.421693	0.3392349	0.1511642	0.102966	0.138579	0.1526702
886.301	7.5	0.416474	0.3449125	0.1513974	0.1035773	0.141217	0.1484245
886.401	7.5	0.411404	0.3349347	0.1565529	0.1058187	0.142259	0.149277
886.501	7.5	0.424287	0.3357162	0.1551928	0.1063884	0.142367	0.1488419
886.601	7.5	0.412707	0.3341958	0.1527367	0.1046112	0.144197	0.1486786
886.701	7.5	0.403561	0.3371194	0.151935	0.105948	0.145101	0.1506026
886.801	7.5	0.403407	0.3411971	0.14998	0.1065661	0.138621	0.1518116
886.901	7.5	0.39482	0.3444984	0.1496793	0.1060354	0.138659	0.1521131
887.001	7.5	0.387286	0.3476438	0.1472681	0.1058431	0.138873	0.1520751
887.101	7.5	0.400487	0.35549	0.1467626	0.1082987	0.141207	0.1502317
887.201	7.5	0.390146	0.3472039	0.1465867	0.1103925	0.139806	0.1485343
887.301	7.5	0.396267	0.340745	0.1421937	0.1072056	0.138487	0.1477946
887.401	7.5	0.377623	0.3405221	0.1409469	0.1073183	0.136638	0.1515823
887.501	7.5	0.402544	0.343418	0.1430043	0.1057495	0.134673	0.1525138
887.601	7.5	0.404765	0.3433435	0.1426979	0.1064228	0.140741	0.1566644
887.701	7.5	0.413246	0.3451694	0.1426492	0.109837	0.137657	0.1543564
887.801	7.5	0.404175	0.3439897	0.1466465	0.11044	0.136513	0.1536893
887.901	7.5	0.410722	0.3445857	0.1467021	0.1082752	0.136631	0.1541722
888.001	7.5	0.417596	0.3430654	0.1434396	0.1095339	0.140176	0.1537428
888.101	7.5	0.419483	0.3431992	0.1420047	0.109831	0.136526	0.1555323
888.201	7.5	0.408006	0.3380987	0.1420417	0.111845	0.136937	0.1541338
888.301	7.5	0.399353	0.3338201	0.1396254	0.1152542	0.134918	0.1519928
888.401	7.5	0.407763	0.3334149	0.1463857	0.1139231	0.136284	0.1515864
888.501	7.5	0.400408	0.33858	0.1432793	0.1085797	0.137544	0.1496789
888.601	7.5	0.404075	0.3250337	0.1419356	0.1034644	0.134048	0.1495336
888.701	7.5	0.402694	0.3111599	0.1428949	0.1036924	0.135384	0.1427755
888.801	7.5	0.389092	0.3120925	0.1444586	0.1037447	0.135351	0.1451797
888.901	7.5	0.41207	0.3049997	0.1357528	0.103316	0.137541	0.1470703
889.001	7.5	0.409307	0.3029492	0.1422673	0.1043715	0.137773	0.1458876
889.101	7.5	0.404939	0.3062194	0.1397838	0.1066632	0.13431	0.1473644
889.201	7.5	0.40265	0.3353317	0.1409302	0.1077737	0.133964	0.1504169
889.301	7.5	0.409352	0.3359934	0.1342852	0.1070421	0.133172	0.1507375
889.401	7.5	0.395353	0.3416101	0.1385835	0.106378	0.129644	0.1542448
889.501	7.5	0.406637	0.3426826	0.1414864	0.1039669	0.131653	0.1537743
889.601	7.5	0.405461	0.3363521	0.139696	0.1026048	0.13106	0.1523549
889.701	7.5	0.406291	0.3371255	0.1341653	0.1042164	0.131716	0.1553468
889.801	7.5	0.405699	0.3376574	0.1370603	0.1047316	0.131759	0.1519804
889.901	7.5	0.397916	0.3389769	0.1352283	0.1052955	0.131888	0.1528767
890.001	7.5	0.388866	0.3469556	0.1359639	0.1078713	0.13227	0.1513896
890.101	7.5	0.402659	0.3462973	0.1401903	0.1074957	0.130995	0.1522739
890.201	7.5	0.39813	0.3525866	0.1411548	0.1040846	0.132864	0.1502052
890.301	7.5	0.401407	0.3572148	0.1393067	0.1051118	0.129201	0.1519753
890.401	7.5	0.394041	0.3424507	0.1418108	0.1026423	0.12905	0.1515294
890.501	7.5	0.379097	0.3406498	0.1451244	0.1007766	0.130633	0.1518627

890.601	7.5	0.375544	0.3425343	0.1467536	0.1044488	0.13484	0.1511625
890.701	7.5	0.372376	0.3425576	0.1465453	0.1058695	0.134098	0.1524862
890.801	7.5	0.381671	0.3441394	0.1420889	0.1039502	0.133651	0.1528467
890.901	7.5	0.397014	0.3444214	0.1419864	0.1065994	0.132523	0.1544024
891.001	7.5	0.417118	0.3420051	0.1431998	0.1062837	0.132916	0.1519522
891.101	7.5	0.414217	0.3434902	0.1434025	0.1078698	0.133798	0.151581
891.201	7.5	0.405102	0.3414404	0.1452937	0.1056441	0.13349	0.154609
891.301	7.5	0.399756	0.3386172	0.1441321	0.1060697	0.130661	0.1569729
891.401	7.5	0.395319	0.3356974	0.1425552	0.1092611	0.128758	0.1572748
891.501	7.5	0.394952	0.3318349	0.1435946	0.1073987	0.130601	0.1544929
891.601	7.5	0.400042	0.3346986	0.1467715	0.1028691	0.134879	0.1575598
891.701	7.5	0.40311	0.3331785	0.145021	0.1068222	0.136382	0.1578034
891.801	7.5	0.388555	0.314927	0.1460403	0.1082991	0.135657	0.1555885
891.901	7.5	0.410811	0.307499	0.1453667	0.1050744	0.136621	0.1554794
892.001	7.5	0.395414	0.3140734	0.1472062	0.1062282	0.135703	0.1566063
892.101	7.5	0.384921	0.3042537	0.1448664	0.1035757	0.134951	0.1565492
892.201	7.5	0.395414	0.305486	0.1407077	0.1054753	0.131483	0.1524339
892.301	7.5	0.405498	0.3218014	0.1441709	0.1070105	0.133935	0.1526086
892.401	7.5	0.399025	0.334319	0.1460967	0.1079678	0.131391	0.152475
892.501	7.5	0.424272	0.3411521	0.1496973	0.1106082	0.134955	0.1536086
892.601	7.5	0.410605	0.3479932	0.1491082	0.1060937	0.136837	0.1554277
892.701	7.5	0.393326	0.3384012	0.1486102	0.1038594	0.137526	0.1543095
892.801	7.5	0.398929	0.3347623	0.1480332	0.1039513	0.138279	0.1560623
892.901	7.5	0.403697	0.3352335	0.1477952	0.1057551	0.139675	0.1586139
893.001	7.5	0.404995	0.3400008	0.1476947	0.1072083	0.138138	0.1515936
893.101	7.5	0.40907	0.3437033	0.1448322	0.1058601	0.137353	0.1565171
893.201	7.5	0.411101	0.34746	0.1454378	0.1046121	0.138111	0.1559857
893.301	7.5	0.400035	0.3471013	0.1461286	0.1033375	0.136998	0.1538696
893.401	7.5	0.388372	0.3539896	0.1459567	0.1044961	0.138322	0.1545468
893.501	7.5	0.394288	0.3524021	0.1448485	0.1061127	0.138468	0.1553323
893.601	7.5	0.393609	0.3384666	0.1457407	0.1045572	0.134649	0.1534099
893.701	7.5	0.400785	0.3375949	0.1465744	0.1037635	0.137297	0.1527573
893.801	7.5	0.39655	0.3405695	0.1474447	0.102162	0.137936	0.1525382
893.901	7.5	0.393602	0.3437634	0.1487589	0.1017138	0.136775	0.1547318
894.001	7.5	0.400225	0.3444067	0.1507847	0.1017866	0.136243	0.1548926
894.101	7.5	0.38899	0.3451687	0.1504985	0.1031127	0.134477	0.1546384
894.201	7.5	0.395887	0.3423664	0.1507652	0.1046912	0.134496	0.1529333
894.301	7.5	0.392852	0.3440252	0.1492388	0.10354	0.136116	0.1527191
894.401	7.5	0.389884	0.3434788	0.1517451	0.1046995	0.137533	0.1508718
894.501	7.5	0.404627	0.3367444	0.1486642	0.1050543	0.139435	0.1524233
894.601	7.5	0.412035	0.3336336	0.1466011	0.1048054	0.142403	0.151023
894.701	7.5	0.408119	0.3335911	0.1474394	0.1062603	0.143226	0.1512571
894.801	7.5	0.40109	0.3369632	0.1453696	0.107048	0.140044	0.1426303
894.901	7.5	0.402652	0.3246812	0.1504371	0.1094086	0.139889	0.1477384
895.001	7.5	0.395903	0.3034014	0.1487949	0.1109428	0.140887	0.1502212
895.101	7.5	0.403038	0.3137017	0.1432365	0.110235	0.139852	0.1546654
895.201	7.5	0.391036	0.3062967	0.1480873	0.1121506	0.140116	0.1559656
895.301	7.5	0.39841	0.3055956	0.1468592	0.1116972	0.137457	0.1563868
895.401	7.5	0.37778	0.3058516	0.1498465	0.1104716	0.136025	0.1557598
895.501	7.5	0.401535	0.3319435	0.1540669	0.1086967	0.135728	0.1538959
895.601	7.5	0.405015	0.3270224	0.1541814	0.1094913	0.137732	0.1501331
895.701	7.5	0.412361	0.3379896	0.1485433	0.1112842	0.135158	0.1520526
895.801	7.5	0.412831	0.3456158	0.1490193	0.1130383	0.136031	0.1516737
895.901	7.5	0.41652	0.3361058	0.1530004	0.1128954	0.13763	0.1538672
896.001	7.5	0.416953	0.336089	0.1509915	0.1123896	0.140176	0.152691
896.101	7.5	0.413423	0.3361893	0.1476025	0.1133138	0.137734	0.1527243
896.201	7.5	0.408334	0.3385338	0.1496189	0.1136846	0.137351	0.1527261
896.301	7.5	0.397991	0.3460203	0.1473753	0.114213	0.13397	0.1498421
896.401	7.5	0.410131	0.3466693	0.1553671	0.1155747	0.13432	0.1514149
896.501	7.5	0.409197	0.3473529	0.1537401	0.1105312	0.132871	0.1486327
896.601	7.5	0.405391	0.3574195	0.1524163	0.1062314	0.130306	0.1495874
896.701	7.5	0.407775	0.3425719	0.1503889	0.1051609	0.128323	0.1496761
896.801	7.5	0.390105	0.3387637	0.1477231	0.104512	0.127644	0.1498999
896.901	7.5	0.416596	0.3389367	0.1488051	0.1043868	0.12863	0.1492687
897.001	7.5	0.415377	0.3413988	0.1446954	0.1072999	0.12613	0.149793
897.101	7.5	0.402557	0.3416826	0.1458628	0.1090715	0.123864	0.1512492
897.201	7.5	0.39513	0.3436324	0.1472676	0.1076477	0.122528	0.1502264
897.301	7.5	0.40566	0.3407146	0.1447952	0.1070085	0.123228	0.1489857
897.401	7.5	0.400446	0.3410975	0.1445239	0.1059959	0.12215	0.1525002
897.501	7.5	0.407812	0.3406879	0.1442626	0.1038008	0.124028	0.1522127
897.601	7.5	0.415717	0.3388349	0.1443537	0.1033439	0.126498	0.1547254
897.701	7.5	0.410204	0.3337011	0.1432901	0.1061801	0.127075	0.149935
897.801	7.5	0.414523	0.3285758	0.1462752	0.1064603	0.127397	0.1493184
897.901	7.5	0.389008	0.3333319	0.144633	0.1059243	0.125649	0.1500052
898.001	7.5	0.386848	0.3320277	0.1434886	0.1071644	0.126363	0.1511367
898.101	7.5	0.397618	0.315947	0.1418473	0.1064496	0.126379	0.1512959
898.201	7.5	0.393017	0.3068489	0.14393	0.1039485	0.12895	0.1512851
898.301	7.5	0.402783	0.3098278	0.1425164	0.1057303	0.126787	0.1508005
898.401	7.5	0.383188	0.3061308	0.1476627	0.1022621	0.128094	0.1485118
898.501	7.5	0.376485	0.3059678	0.1429109	0.1009932	0.1292	0.14744
898.601	7.5	0.382936	0.3112204	0.1432151	0.1031473	0.130785	0.1472408
898.701	7.5	0.396396	0.3263318	0.1430091	0.1061784	0.128762	0.1390279
898.801	7.5	0.408078	0.3298778	0.1425336	0.1033561	0.127547	0.1418609
898.901	7.5	0.408337	0.3409617	0.1335219	0.103687	0.126189	0.1446388
899.001	7.5	0.394608	0.3379821	0.1400043	0.1045804	0.127752	0.1441184
899.101	7.5	0.392786	0.3359385	0.139354	0.1047925	0.130445	0.1468839
899.201	7.5	0.401382	0.3319906	0.1383478	0.1030212	0.128987	0.149259
899.383	8	0.404545	0.344642	0.1343052	0.1043526	0.128158	0.1508072
899.483	8	0.399576	0.3472248	0.1379492	0.1073778	0.125126	0.1543677
899.583	8	0.389911	0.3559449	0.1406927	0.1038957	0.126496	0.1540963
899.683	8	0.4133	0.3518653	0.1381911	0.1054319	0.130525	0.1558183
899.783	8	0.398108	0.3404434	0.1316557	0.1023305	0.1313	0.1544142
899.883	8	0.386935	0.3377649	0.1364575	0.1049351	0.130105	0.1511624
899.983	8	0.399431	0.339663	0.1362815	0.1069426	0.132566	0.1515138
900.083	8	0.401248	0.3406695	0.1345017	0.1079776	0.133545	0.1492906

900.183	8	0.406059	0.3419717	0.1414516	0.1099972	0.130714	0.1494006
900.283	8	0.429997	0.3426512	0.1457889	0.1024025	0.12973	0.1475403
900.383	8	0.413247	0.3428206	0.1431163	0.1023839	0.135168	0.149429
900.483	8	0.399222	0.3405991	0.1410132	0.1030123	0.131921	0.1488831
900.583	8	0.405881	0.3398421	0.1442211	0.1031904	0.13304	0.1523962
900.683	8	0.414271	0.3350636	0.1435342	0.1045624	0.136286	0.1510521
900.783	8	0.419079	0.330311	0.1465563	0.1034174	0.136243	0.1537926
900.883	8	0.418944	0.3308809	0.1458803	0.1056261	0.136475	0.1538246
900.983	8	0.406687	0.3317308	0.1465494	0.1042731	0.137204	0.156236
901.083	8	0.394798	0.3178601	0.1462515	0.1056173	0.137607	0.1532491
901.183	8	0.397623	0.3129304	0.1447074	0.1045008	0.138972	0.1535208
901.283	8	0.395662	0.3106148	0.1465006	0.1049246	0.137523	0.1544663
901.383	8	0.402134	0.3045663	0.1431068	0.1054202	0.139007	0.155134
901.483	8	0.391085	0.3063007	0.1439249	0.1055325	0.136672	0.1564667
901.583	8	0.387762	0.313032	0.145982	0.1034449	0.140001	0.1544471
901.683	8	0.391194	0.3334354	0.1469544	0.104363	0.14009	0.1550862
901.783	8	0.381797	0.3371167	0.1437645	0.1041752	0.142303	0.1592399
901.883	8	0.391143	0.3481042	0.1447849	0.1027586	0.141829	0.157886
901.983	8	0.388296	0.3394188	0.1450953	0.102966	0.140659	0.1570066
902.083	8	0.392146	0.3334245	0.1476508	0.1012985	0.139615	0.1569547
902.183	8	0.396526	0.3327466	0.1511778	0.1019617	0.137035	0.1597444
902.283	8	0.4089	0.3347282	0.1486099	0.1047215	0.132562	0.1544027
902.383	8	0.403665	0.3397261	0.1496388	0.1070576	0.134361	0.1583659
902.483	8	0.394002	0.3446088	0.1493187	0.1074329	0.138396	0.1559181
902.583	8	0.39362	0.3497656	0.1496879	0.1046253	0.138449	0.1564963
902.683	8	0.396589	0.3585969	0.1495539	0.1092355	0.142044	0.1571097
902.783	8	0.389417	0.3509034	0.1481033	0.1083287	0.142907	0.1544051
902.883	8	0.385984	0.341274	0.1497543	0.1117503	0.137942	0.1563856
902.983	8	0.370783	0.3381853	0.1479615	0.1124018	0.139651	0.1574252
903.083	8	0.394435	0.3407748	0.1461945	0.1125461	0.138723	0.1497604
903.183	8	0.395494	0.3400251	0.147908	0.1134619	0.135844	0.1535931
903.283	8	0.405108	0.3433762	0.1506675	0.1128246	0.137587	0.1524012
903.383	8	0.389604	0.3420476	0.1490489	0.1138538	0.13636	0.1513161
903.483	8	0.407834	0.3418499	0.1497294	0.1108482	0.135453	0.1519218
903.583	8	0.394805	0.3421882	0.1494481	0.111836	0.135354	0.1527974
903.683	8	0.396264	0.3401814	0.1502568	0.1109173	0.138334	0.1510012
903.783	8	0.399384	0.3346946	0.1482969	0.1136595	0.133824	0.1514583
903.883	8	0.394089	0.3281016	0.1498297	0.1107408	0.134761	0.1506312
903.983	8	0.412139	0.3321882	0.1462403	0.1063245	0.132935	0.1487731
904.083	8	0.404641	0.3306307	0.1432506	0.1063438	0.134245	0.1510532
904.183	8	0.410245	0.316819	0.1440943	0.1043731	0.132244	0.1515455
904.283	8	0.401116	0.3094869	0.1428092	0.1065681	0.134849	0.1496381
904.383	8	0.419005	0.3100616	0.1444659	0.1062098	0.133944	0.1515972
904.483	8	0.410793	0.3024999	0.1444691	0.1065623	0.135706	0.1486835
904.583	8	0.410374	0.3012873	0.139214	0.1063542	0.135869	0.1508629
904.683	8	0.397191	0.3220842	0.1428453	0.1016284	0.134267	0.1487113
904.783	8	0.403951	0.3299893	0.1453357	0.1012965	0.133685	0.1506827
904.883	8	0.41258	0.3417914	0.1497174	0.1016125	0.132742	0.143818
904.983	8	0.403941	0.3519388	0.1500961	0.1006769	0.136045	0.1434406
905.083	8	0.42314	0.3371018	0.1492369	0.1036635	0.135792	0.148203
905.183	8	0.41197	0.3355969	0.1478763	0.1021435	0.135235	0.1525337
905.283	8	0.411046	0.3314922	0.1518459	0.1041108	0.134584	0.1524622
905.383	8	0.380371	0.3358892	0.1497497	0.1018839	0.133712	0.1519884
905.483	8	0.397514	0.340259	0.1490008	0.09920397	0.130372	0.1508466
905.583	8	0.392092	0.3480346	0.14904	0.101119	0.129711	0.151254
905.683	8	0.399558	0.3474503	0.1491861	0.09920703	0.133108	0.1479204
905.783	8	0.390615	0.3587568	0.1520891	0.09952944	0.133017	0.1503484
905.883	8	0.382264	0.3436678	0.1489898	0.1021194	0.132873	0.1513211
905.983	8	0.388969	0.3378505	0.1482122	0.1007021	0.131867	0.151773
906.083	8	0.378055	0.3352862	0.1467076	0.10097	0.133424	0.1512978
906.183	8	0.391551	0.3379608	0.1423027	0.09905761	0.131978	0.151671
906.283	8	0.398322	0.3387654	0.1436273	0.1006103	0.129086	0.1514247
906.383	8	0.402678	0.3403765	0.145202	0.1001774	0.125755	0.1506463
906.483	8	0.403933	0.338958	0.1456913	0.100192	0.128313	0.1507556
906.583	8	0.392898	0.3406805	0.1415835	0.1018679	0.131092	0.1508728
906.683	8	0.395064	0.3388778	0.1427006	0.1011557	0.134508	0.1481983
906.783	8	0.393435	0.3365462	0.1420645	0.09762947	0.132644	0.148429
906.883	8	0.394032	0.3332071	0.1439931	0.09874294	0.133248	0.1475014
906.983	8	0.396338	0.3285874	0.1436654	0.1049474	0.133535	0.1472844
907.083	8	0.38427	0.331862	0.1437297	0.1018672	0.132706	0.1485271
907.183	8	0.410413	0.3263014	0.1424073	0.1004565	0.13145	0.1485924
907.283	8	0.393144	0.3058842	0.1419107	0.0980043	0.132065	0.1478844
907.383	8	0.382043	0.3063062	0.1407596	0.1022515	0.131974	0.149085
907.483	8	0.389196	0.3055327	0.1387782	0.1041412	0.125689	0.1498221
907.583	8	0.396764	0.304193	0.1442936	0.1053042	0.124474	0.1527706
907.683	8	0.398204	0.30235	0.1393568	0.1055616	0.127909	0.1533365
907.783	8	0.416804	0.3281049	0.141075	0.09841046	0.130678	0.1496928
907.883	8	0.402676	0.3257789	0.1421764	0.1012273	0.13314	0.1516275
907.983	8	0.401297	0.3384722	0.1390542	0.1040936	0.134294	0.1506731
908.083	8	0.409441	0.34565	0.1361445	0.1039018	0.133775	0.1510549
908.183	8	0.432999	0.3386292	0.1374303	0.1043595	0.134943	0.1524076
908.283	8	0.436871	0.3371565	0.1380451	0.1018683	0.132518	0.1512801
908.383	8	0.452536	0.3401158	0.1334623	0.1033286	0.133567	0.1520082
908.483	8	0.421981	0.3410105	0.1365764	0.1056031	0.130239	0.1506922
908.583	8	0.41724	0.3503763	0.1389263	0.105637	0.131101	0.149075
908.683	8	0.426472	0.3489181	0.1362148	0.102431	0.132154	0.1455555
908.783	8	0.421925	0.3512569	0.1291548	0.1003379	0.133552	0.1419721
908.883	8	0.411577	0.35907	0.134187	0.09652741	0.132317	0.1440332
908.983	8	0.406018	0.3419695	0.1340655	0.09857509	0.136476	0.1446459
909.083	8	0.410174	0.3410802	0.1301466	0.09954249	0.136005	0.147222
909.183	8	0.41758	0.3419008	0.1390073	0.1006793	0.137332	0.1491427
909.283	8	0.4029	0.3434686	0.1408219	0.1005962	0.138135	0.1496316
909.383	8	0.39528	0.3462546	0.1385123	0.1011051	0.13906	0.1497943
909.483	8	0.396079	0.3513508	0.1422538	0.1020775	0.138284	0.1530109
909.583	8	0.400062	0.3480003	0.14461	0.09984989	0.138226	0.1534024

909.683	8	0.407637	0.3510316	0.1437523	0.1011877	0.136337	0.15414
909.783	8	0.410748	0.3495634	0.140534	0.1037245	0.139218	0.1524858
909.883	8	0.403019	0.3429618	0.1398654	0.1059209	0.138875	0.1507012
909.983	8	0.400145	0.339751	0.1399103	0.1053531	0.139678	0.150663
910.083	8	0.391978	0.3366781	0.1411499	0.1065964	0.137908	0.1471863
910.183	8	0.400128	0.338035	0.1417378	0.1108705	0.138736	0.1481571
910.283	8	0.389446	0.3250447	0.1424412	0.109989	0.135397	0.1460817
910.383	8	0.390234	0.3037838	0.1414719	0.1104947	0.135816	0.1500451
910.483	8	0.376817	0.3079284	0.1445833	0.1106185	0.137544	0.1493529
910.583	8	0.39937	0.3045834	0.1429199	0.111424	0.136309	0.1531015
910.683	8	0.398408	0.3034865	0.1415969	0.1113781	0.139497	0.1525098
910.783	8	0.407831	0.301183	0.1453169	0.1114947	0.142838	0.1544928
910.883	8	0.39819	0.3241481	0.1451589	0.1076818	0.137917	0.1539514
910.983	8	0.412949	0.328847	0.145206	0.1068098	0.137524	0.1562818
911.083	8	0.396658	0.3487831	0.1416758	0.1085752	0.136315	0.1519561
911.183	8	0.390867	0.3453221	0.1441279	0.1079524	0.134969	0.1516765
911.283	8	0.394934	0.3435363	0.1465903	0.112187	0.137031	0.1545905
911.383	8	0.391221	0.3376343	0.1512796	0.1113894	0.136133	0.1558731
911.483	8	0.406735	0.3405337	0.1482529	0.1042753	0.136839	0.1584518
911.583	8	0.398173	0.3448517	0.1483967	0.1004931	0.137219	0.1564413
911.683	8	0.393508	0.3511905	0.149162	0.09957502	0.139833	0.1584161
911.783	8	0.384982	0.3484685	0.1491305	0.1006173	0.138193	0.1597971
911.883	8	0.409573	0.3570558	0.1471869	0.1006066	0.139018	0.1567363
911.983	8	0.412486	0.3581873	0.1467517	0.1034462	0.137949	0.1580119
912.083	8	0.403104	0.346502	0.1446744	0.1032629	0.140639	0.1601931
912.183	8	0.391035	0.3463765	0.1470588	0.1026776	0.137338	0.1620787
912.283	8	0.399338	0.349294	0.1426383	0.09948529	0.139182	0.1556425
912.383	8	0.404156	0.3523851	0.1443733	0.0998619	0.135589	0.155774
912.483	8	0.405896	0.3549897	0.1454111	0.09834976	0.134612	0.15418
912.583	8	0.42698	0.3575898	0.1463687	0.1025968	0.136522	0.1540102
912.683	8	0.414012	0.3567417	0.1488159	0.1038806	0.134824	0.1563991
912.783	8	0.412552	0.357138	0.1486122	0.1021268	0.135697	0.1551224
912.883	8	0.381866	0.355997	0.1472802	0.1015711	0.135377	0.1594335
912.983	8	0.397589	0.3466787	0.1451996	0.0999827	0.135526	0.1591568
913.083	8	0.394287	0.3405697	0.1454153	0.1001334	0.1362	0.1533488
913.183	8	0.399007	0.338708	0.1453504	0.09864759	0.133873	0.1587371
913.283	8	0.388789	0.3411937	0.1422875	0.1004323	0.131585	0.1551362
913.383	8	0.378395	0.3210936	0.1422694	0.1059951	0.132207	0.154569
913.483	8	0.372626	0.3051308	0.139183	0.1031943	0.131411	0.1550669
913.583	8	0.372772	0.3047315	0.1450343	0.1031421	0.130712	0.1554894
913.683	8	0.380911	0.305328	0.142979	0.1032239	0.133703	0.1518982
913.783	8	0.396964	0.3017165	0.1372632	0.1036117	0.133985	0.1502654
913.883	8	0.400276	0.3065018	0.1449697	0.10134	0.132721	0.151
913.983	8	0.412846	0.3241131	0.1446075	0.1026453	0.132875	0.1515036
914.083	8	0.41416	0.3334929	0.1469409	0.103772	0.136298	0.1552077
914.183	8	0.402673	0.3489253	0.1509956	0.1027689	0.134133	0.1529993
914.283	8	0.398748	0.3477846	0.1474767	0.1005302	0.134229	0.1529652
914.383	8	0.402221	0.3467255	0.1446608	0.09831941	0.132789	0.1532006
914.483	8	0.395824	0.3408617	0.1462318	0.1050026	0.13221	0.1528529
914.583	8	0.387528	0.3435456	0.1461141	0.1006472	0.131831	0.1524346
914.683	8	0.403427	0.3479256	0.1460352	0.1016376	0.134614	0.1518267
914.783	8	0.402298	0.3529807	0.147598	0.09760266	0.13174	0.1520066
914.883	8	0.389118	0.3517408	0.1461773	0.09946806	0.131225	0.142975
914.983	8	0.385847	0.3615738	0.151348	0.1023173	0.131911	0.1493791
915.083	8	0.397114	0.3539462	0.1500557	0.103542	0.130438	0.1501162
915.183	8	0.40106	0.3460154	0.1486636	0.1044726	0.130406	0.1531989
915.283	8	0.446296	0.3435763	0.1467291	0.09779755	0.131202	0.1533397
915.383	8	0.448392	0.3465726	0.1434672	0.09851747	0.129199	0.1548064
915.483	8	0.431081	0.3482312	0.1473393	0.1014199	0.123656	0.1540378
915.583	8	0.437976	0.3524236	0.1437178	0.1002515	0.123099	0.1528091
915.683	8	0.439483	0.3511957	0.1438138	0.1002438	0.129496	0.1506195
915.783	8	0.423822	0.3540221	0.1415304	0.09800462	0.128769	0.1530065
915.883	8	0.429979	0.3537516	0.1434827	0.1008775	0.130699	0.1547589
915.983	8	0.40936	0.3500639	0.1426559	0.098493	0.133567	0.152974
916.083	8	0.395254	0.3448212	0.1410492	0.1010647	0.133352	0.1523426
916.183	8	0.405335	0.3372774	0.1400998	0.1014543	0.13624	0.1519446
916.283	8	0.413411	0.3373139	0.143643	0.09810254	0.133526	0.1505261
916.383	8	0.406847	0.3387901	0.1413306	0.09555156	0.137203	0.1487424
916.483	8	0.397604	0.321839	0.1409849	0.09681079	0.133124	0.1513678
916.583	8	0.396573	0.3065982	0.140445	0.095246	0.132889	0.1513644
916.683	8	0.394971	0.3181052	0.1407169	0.09513737	0.135633	0.1496496
916.783	8	0.394462	0.308373	0.1454361	0.09631394	0.133801	0.149822
916.883	8	0.398592	0.3068267	0.1415602	0.09688907	0.131102	0.1505488
916.983	8	0.392546	0.3193961	0.1409878	0.09970706	0.133518	0.1515464
917.083	8	0.401346	0.3294485	0.1404505	0.09954074	0.135243	0.1520048
917.183	8	0.397584	0.3482197	0.1413945	0.09981731	0.139468	0.1512153
917.283	8	0.409137	0.3513634	0.1339589	0.09953163	0.138534	0.1496206
917.383	8	0.408263	0.3471076	0.1380255	0.101945	0.137511	0.1477019
917.483	8	0.391828	0.3494705	0.1397963	0.1025892	0.137729	0.1501386
917.583	8	0.383115	0.3504483	0.1390667	0.1043143	0.137142	0.1493376
917.683	8	0.394174	0.3553938	0.1373395	0.1060467	0.135457	0.1522086
917.783	8	0.382833	0.3602841	0.1359524	0.1061999	0.135905	0.1502804
917.883	8	0.386767	0.3660404	0.1402161	0.1066344	0.136825	0.1497742
917.983	8	0.371061	0.3517453	0.1359634	0.1061679	0.136241	0.1496434
918.083	8	0.389854	0.3593577	0.1338228	0.1061019	0.13661	0.1497476
918.183	8	0.388277	0.3516782	0.134335	0.1091967	0.137785	0.1525164
918.283	8	0.401106	0.3490522	0.1349416	0.1114756	0.133206	0.1511357
918.383	8	0.392315	0.3541002	0.1373843	0.1089873	0.133853	0.1511393
918.483	8	0.402749	0.3604513	0.1424137	0.1086554	0.137112	0.1493133
918.583	8	0.392636	0.362715	0.1408107	0.1091752	0.137135	0.1466771
918.683	8	0.39063	0.3677101	0.1387627	0.1069795	0.140156	0.1449314
918.783	8	0.392111	0.3666274	0.1414331	0.1093739	0.141685	0.1423803
918.883	8	0.386504	0.3688478	0.1430325	0.108448	0.138062	0.1446039
918.983	8	0.406824	0.367096	0.1398951	0.1044326	0.133883	0.1461399
919.083	8	0.406618	0.3600508	0.1410564	0.1034179	0.13506	0.1469125

919.183	8	0.401191	0.352941	0.1391365	0.1024131	0.132785	0.1479732
919.283	8	0.39464	0.3451351	0.1428321	0.1034519	0.13461	0.1495026
919.383	8	0.400998	0.343127	0.1424844	0.1034314	0.134487	0.1508157
919.483	8	0.416106	0.3393385	0.1425987	0.1051067	0.136258	0.1543037
919.583	8	0.405405	0.3214963	0.1421176	0.1063802	0.136624	0.1526927
919.683	8	0.391674	0.3092445	0.1428547	0.1040121	0.13893	0.1539464
919.783	8	0.399321	0.3176337	0.1450641	0.1019544	0.137801	0.1536931
919.883	8	0.394716	0.3103056	0.1396028	0.1019987	0.137582	0.1510879
919.983	8	0.411769	0.3018666	0.1435103	0.09863209	0.135356	0.1516631
920.083	8	0.420916	0.3253613	0.1449362	0.1014801	0.131959	0.1496513
920.183	8	0.426388	0.3207753	0.1474007	0.1040506	0.129354	0.1490219
920.283	8	0.424244	0.3506401	0.1398417	0.1053797	0.133556	0.1474365
920.383	8	0.38894	0.347105	0.1397015	0.1031676	0.132752	0.149672
920.483	8	0.390892	0.3433315	0.1434673	0.1024321	0.134589	0.1511747
920.583	8	0.403896	0.3444785	0.1484057	0.102975	0.136403	0.1539468
920.683	8	0.412011	0.3468334	0.1488723	0.1021881	0.135043	0.1534344
920.783	8	0.421045	0.3495441	0.1475831	0.1020082	0.134807	0.1557331
920.883	8	0.40568	0.3578599	0.1470788	0.1054943	0.134382	0.1539929
920.983	8	0.401004	0.3553915	0.1480074	0.1009055	0.137513	0.1545041
921.083	8	0.393747	0.3542518	0.149702	0.1026105	0.137677	0.1553907
921.183	8	0.403665	0.3577597	0.1475409	0.1024031	0.132938	0.1565054
921.283	8	0.443455	0.3452247	0.145557	0.1027626	0.131031	0.1556144
921.383	8	0.439801	0.3456154	0.1475581	0.1026985	0.131807	0.1567057
921.483	8	0.462656	0.3493575	0.1435432	0.1032224	0.130057	0.1556506
921.583	8	0.438822	0.3528155	0.1420232	0.1051165	0.127979	0.1554068
921.683	8	0.422368	0.356115	0.1452426	0.1047226	0.129001	0.1556587
921.783	8	0.45791	0.3568688	0.1462374	0.1023404	0.128218	0.1570495
921.883	8	0.418059	0.3560878	0.1449625	0.1029524	0.128162	0.1586859
921.983	8	0.409567	0.3565764	0.1478096	0.1064684	0.127971	0.1527067
922.083	8	0.397561	0.3536295	0.1471268	0.1039548	0.129359	0.1527707
922.183	8	0.405764	0.34732	0.149692	0.1020352	0.127658	0.1510269
922.283	8	0.387386	0.3434325	0.1500854	0.09855448	0.12813	0.1490801
922.383	8	0.37877	0.3369507	0.1529923	0.09938791	0.126238	0.1509534
922.483	8	0.380414	0.3382694	0.1480358	0.1004953	0.127712	0.1514201
922.583	8	0.393025	0.3377951	0.1460643	0.1007944	0.130093	0.1536479
922.683	8	0.39378	0.3168117	0.1462051	0.1034659	0.129644	0.1474345
922.783	8	0.439732	0.3127309	0.1476507	0.09655228	0.126968	0.1489342
922.883	8	0.426841	0.3100087	0.1470051	0.09786832	0.122979	0.148976
922.983	8	0.438785	0.3039588	0.1414589	0.09891888	0.124353	0.1493545
923.083	8	0.444336	0.299387	0.1406175	0.09972278	0.125247	0.1513258
923.183	8	0.428109	0.3184347	0.1430329	0.1016081	0.12458	0.1512982
923.283	8	0.41893	0.3345797	0.1471349	0.1007871	0.125591	0.1512942
923.383	8	0.428095	0.3491255	0.150918	0.1060123	0.125646	0.1488689
923.483	8	0.396548	0.3459174	0.150762	0.1048818	0.119986	0.1485704
923.583	8	0.382792	0.3398466	0.1457681	0.1038297	0.119461	0.1504433
923.683	8	0.391911	0.338086	0.1469127	0.09979082	0.124138	0.1517059
923.783	8	0.40191	0.3412384	0.1502891	0.0987056	0.123981	0.1487703
923.883	8	0.401166	0.3449157	0.1475271	0.09717624	0.124376	0.1508685
923.983	8	0.396786	0.354097	0.1452893	0.09954812	0.128431	0.1483569
924.083	8	0.403105	0.3495439	0.1470015	0.09659627	0.13181	0.1508316
924.183	8	0.400038	0.3517902	0.1513242	0.09833598	0.131825	0.1502618
924.283	8	0.393885	0.3509574	0.1542673	0.1008179	0.130279	0.1500504
924.383	8	0.410409	0.3408492	0.1518496	0.1025134	0.135757	0.1394029
924.483	8	0.404625	0.3391585	0.1538033	0.1051225	0.131739	0.1448688
924.583	8	0.414633	0.3413489	0.1502744	0.1033385	0.132907	0.14955
924.683	8	0.402375	0.3423018	0.150226	0.1043297	0.133523	0.150345
924.783	8	0.401779	0.3446114	0.1478168	0.1065552	0.131846	0.1533769
924.883	8	0.401987	0.3457194	0.1485682	0.1068782	0.130676	0.1505585
924.983	8	0.400206	0.3441775	0.1484456	0.1091467	0.132466	0.1519191
925.083	8	0.386837	0.3478937	0.1463899	0.1099109	0.131865	0.1506032
925.183	8	0.407767	0.3446477	0.1463287	0.112384	0.134957	0.152497
925.283	8	0.390779	0.3382824	0.1442171	0.1108299	0.135881	0.1498072
925.383	8	0.392122	0.3342194	0.1418404	0.1101674	0.135854	0.1490775
925.483	8	0.37922	0.3318945	0.1454123	0.1087904	0.134892	0.1494288
925.583	8	0.401323	0.33748	0.1445463	0.1115693	0.136275	0.1504437
925.683	8	0.397986	0.3292639	0.1424475	0.1126723	0.133923	0.1478538
925.783	8	0.403278	0.3108612	0.1422443	0.1109797	0.13566	0.1462682
925.883	8	0.385409	0.3112874	0.1440586	0.1094369	0.135084	0.1446355
925.983	8	0.406021	0.3103633	0.1443375	0.1087434	0.134044	0.1463903
926.083	8	0.402544	0.3065626	0.1445394	0.1134325	0.134274	0.1473135
926.183	8	0.392653	0.3008817	0.1402796	0.1088621	0.133344	0.1467163
926.283	8	0.38914	0.3169879	0.1401487	0.1105426	0.131479	0.1472313
926.383	8	0.387141	0.334743	0.1421326	0.109894	0.133942	0.1466534
926.483	8	0.40518	0.3462845	0.1375223	0.1075623	0.134273	0.1468652
926.583	8	0.398527	0.3419261	0.1391536	0.1061637	0.130394	0.1449532
926.683	8	0.395599	0.3361753	0.1383957	0.1043889	0.134596	0.1464481
926.783	8	0.384482	0.3329527	0.1388134	0.1065089	0.135224	0.1488894
926.883	8	0.402943	0.3367177	0.1343236	0.1040239	0.132751	0.1505723
926.983	8	0.405688	0.3416023	0.1377873	0.1071807	0.131869	0.1507463
927.083	8	0.391308	0.3487751	0.1397799	0.1091928	0.130698	0.1499995
927.183	8	0.388503	0.3481269	0.1373951	0.1064318	0.12703	0.1499948
927.283	8	0.396022	0.351464	0.1322294	0.1036001	0.128195	0.1498611
927.383	8	0.399895	0.349125	0.1349622	0.1043801	0.126808	0.1520741
927.483	8	0.402563	0.3416609	0.1379341	0.102807	0.127965	0.1514071
927.583	8	0.424288	0.3405653	0.1345629	0.1034004	0.128671	0.1511995
927.683	8	0.405703	0.3415959	0.1425859	0.1028717	0.130161	0.1483959
927.783	8	0.408872	0.3419577	0.1401143	0.10498	0.127011	0.1466433
927.883	8	0.375789	0.3443153	0.1367438	0.1025285	0.125442	0.143742
927.983	8	0.392856	0.3442318	0.1401208	0.1024689	0.125811	0.1400516
928.083	8	0.388817	0.3423455	0.1413795	0.1044072	0.127581	0.1434759
928.183	8	0.396831	0.3439657	0.1415204	0.1024398	0.124084	0.1430769
928.283	8	0.396376	0.342293	0.1416111	0.1016879	0.124991	0.144485
928.383	8	0.37624	0.3393044	0.1434732	0.1048271	0.124841	0.1469849
928.483	8	0.366978	0.3330385	0.1434437	0.1010244	0.126383	0.1492298
928.583	8	0.369639	0.3331558	0.1433214	0.1022612	0.129165	0.1471842

928.683	8	0.387899	0.3343	0.1442437	0.09950196	0.128717	0.1486129
928.783	8	0.397417	0.323458	0.1432266	0.1040929	0.128501	0.1479579
928.883	8	0.399054	0.3063319	0.1414155	0.1035056	0.12572	0.1491315
928.983	8	0.411853	0.3062419	0.1456075	0.1031054	0.126832	0.1511438
929.083	8	0.397542	0.3049676	0.1447618	0.1056767	0.127209	0.1517286
929.183	8	0.401126	0.3006012	0.1424385	0.105874	0.125373	0.1484321
929.283	8	0.418082	0.3018864	0.1434502	0.1020532	0.124772	0.1481418
929.383	8	0.393923	0.3159159	0.1436973	0.1004448	0.128123	0.1462136
929.483	8	0.394376	0.3380027	0.1390953	0.1027807	0.127108	0.1495881
929.583	8	0.384894	0.3449889	0.1353327	0.1017302	0.125397	0.1494364
929.683	8	0.400102	0.3397418	0.1400766	0.102316	0.126253	0.1518116
929.783	8	0.388282	0.334681	0.1439475	0.09938736	0.12715	0.1529038
929.883	8	0.387719	0.33393	0.1485056	0.09883964	0.123944	0.155838
929.983	8	0.417768	0.335898	0.1450699	0.09985898	0.124489	0.1576744
930.083	8	0.424707	0.3414869	0.1462349	0.1003131	0.126536	0.1536627
930.183	8	0.407548	0.3482882	0.1433882	0.1043758	0.123867	0.1532692
930.283	8	0.41458	0.348561	0.1437947	0.09614023	0.12164	0.1556488
930.383	8	0.416404	0.3527177	0.1455125	0.09824494	0.135206	0.1577985
930.483	8	0.389815	0.3446924	0.1459586	0.1010856	0.136506	0.1601509
930.583	8	0.400062	0.3413652	0.1446705	0.1018134	0.134813	0.1602959
930.683	8	0.407335	0.3403803	0.1444758	0.1016468	0.134622	0.1570503
930.783	8	0.398853	0.341041	0.1404306	0.1004353	0.134646	0.1605686
930.883	8	0.403242	0.341753	0.1437736	0.1003406	0.137281	0.1593732
930.983	8	0.39823	0.3450781	0.1450175	0.1010838	0.134411	0.1597328
931.083	8	0.388836	0.3408782	0.1454141	0.1012556	0.134717	0.1609281
931.183	8	0.397106	0.3442497	0.1470686	0.09697564	0.133782	0.1576789
931.283	8	0.393271	0.3455817	0.1476823	0.09690017	0.132237	0.1554199
931.383	8	0.399599	0.3430045	0.1481438	0.09767193	0.135952	0.1532617
931.483	8	0.383352	0.340115	0.1467472	0.09859092	0.136284	0.1523781
931.583	8	0.387988	0.3321756	0.1494379	0.09963105	0.135406	0.1540252
931.683	8	0.393683	0.3329912	0.1461002	0.1024306	0.138744	0.1515487
931.783	8	0.380431	0.333705	0.1432982	0.1018107	0.141897	0.1543887
931.883	8	0.386299	0.3211845	0.144741	0.103558	0.136073	0.1471639
931.983	8	0.387011	0.3050807	0.1440817	0.1038107	0.13736	0.1455813
932.083	8	0.392555	0.3141	0.1464325	0.1034826	0.134106	0.1476812
932.183	8	0.395929	0.3062375	0.1451243	0.1053817	0.135462	0.1483372
932.283	8	0.399664	0.3018736	0.140037	0.1021655	0.135798	0.150172
932.383	8	0.398184	0.3112543	0.1439238	0.1037407	0.134382	0.153239
932.483	8	0.393871	0.316142	0.1452977	0.1058076	0.131557	0.1518016
932.583	8	0.384219	0.3471551	0.1483879	0.1063801	0.136095	0.1503782
932.683	8	0.393796	0.3428279	0.1468533	0.1100868	0.132961	0.1524234
932.783	8	0.379235	0.3374378	0.1421398	0.1093229	0.132133	0.1525891
932.883	8	0.380878	0.333166	0.1426065	0.1105655	0.133135	0.1554182
932.983	8	0.367723	0.3312905	0.1478046	0.1105602	0.134688	0.1512605
933.083	8	0.38519	0.3367425	0.1463564	0.1106715	0.131813	0.153363
933.183	8	0.38589	0.3428449	0.1452107	0.1123254	0.133438	0.1504752
933.283	8	0.394293	0.3474581	0.1469343	0.1109882	0.132587	0.1522383
933.383	8	0.38323	0.3479896	0.1511922	0.1094904	0.133157	0.1530471
933.483	8	0.397762	0.3540214	0.1548214	0.1097174	0.134442	0.1530095
933.583	8	0.39149	0.3439302	0.1507598	0.1124767	0.134456	0.1477222
933.683	8	0.386775	0.3395692	0.1506196	0.1096474	0.133134	0.1500653
933.783	8	0.385491	0.3405876	0.14791	0.1094641	0.129942	0.1530151
933.883	8	0.38528	0.3418543	0.1467181	0.1055706	0.1328	0.1562923
933.983	8	0.394666	0.3438669	0.1451365	0.1024905	0.129283	0.1530321
934.083	8	0.398121	0.3445491	0.1421825	0.1025503	0.126936	0.153048
934.183	8	0.386426	0.3420698	0.1434099	0.100671	0.127098	0.1541083
934.283	8	0.387554	0.3444861	0.1434241	0.1056133	0.129771	0.1527077
934.383	8	0.399083	0.3461811	0.1460138	0.107231	0.126532	0.1523703
934.483	8	0.402933	0.3403402	0.1437874	0.1084141	0.127209	0.1535137
934.583	8	0.398676	0.3371902	0.1410004	0.1082057	0.128578	0.151657
934.683	8	0.390178	0.3324471	0.141556	0.1061788	0.128743	0.1520069
934.783	8	0.395959	0.3313133	0.1435376	0.1031129	0.128285	0.1526975
934.883	8	0.403665	0.3328262	0.1414163	0.1058562	0.12663	0.1524318
934.983	8	0.415675	0.3082316	0.1419614	0.1066751	0.126526	0.1466867
935.083	8	0.434469	0.30373	0.143166	0.1067884	0.126701	0.1474084
935.183	8	0.425473	0.3105678	0.1406117	0.103576	0.124558	0.1481152
935.283	8	0.4174	0.3046084	0.1437559	0.1039555	0.123668	0.1482858
935.383	8	0.379803	0.2988809	0.1392894	0.1013506	0.123278	0.1486229
935.483	8	0.397771	0.3081108	0.1403967	0.1003142	0.123613	0.1495678
935.583	8	0.389804	0.3253678	0.1427398	0.103411	0.123643	0.1492951
935.683	8	0.402423	0.34365	0.139929	0.1029707	0.12277	0.1515565
935.783	8	0.406597	0.3396097	0.1352194	0.101221	0.123717	0.1515221
935.883	8	0.384433	0.3331867	0.1402241	0.104928	0.12647	0.1495061
935.983	8	0.386587	0.3319786	0.1397198	0.1023153	0.127721	0.1515529
936.083	8	0.389093	0.3301624	0.1350009	0.102372	0.125351	0.1509681
936.183	8	0.409124	0.3369156	0.1383494	0.1006059	0.12085	0.1524476
936.283	8	0.402104	0.3461539	0.1400479	0.104147	0.12155	0.1494706
936.383	8	0.408347	0.3452021	0.1374594	0.1018133	0.126599	0.1488108
936.483	8	0.408836	0.3492872	0.1321475	0.101041	0.126862	0.1480727
936.583	8	0.404248	0.3502865	0.1356042	0.1026228	0.126441	0.1517096
936.683	8	0.396498	0.3401717	0.1365741	0.1039056	0.127562	0.1494482
936.783	8	0.412292	0.3380247	0.1338909	0.0964027	0.126694	0.147527
936.883	8	0.399755	0.3401035	0.1405596	0.09783754	0.125667	0.1457328
936.983	8	0.403621	0.3411949	0.1400283	0.09786278	0.130558	0.1446283
937.083	8	0.397829	0.3429077	0.1375737	0.09604985	0.132363	0.1440907
937.183	8	0.415324	0.3422036	0.1391081	0.09971935	0.133582	0.1421123
937.283	8	0.386921	0.3413626	0.140213	0.09809144	0.134136	0.145504
937.383	8	0.387036	0.3445828	0.1398086	0.09792122	0.131304	0.1447633
937.483	8	0.413715	0.3440269	0.1379319	0.1009436	0.13206	0.1455419
937.583	8	0.418209	0.3387741	0.1378452	0.1022458	0.133452	0.1486489
937.683	8	0.404776	0.3340268	0.1381948	0.10413	0.133006	0.1508507
937.783	8	0.416675	0.3287847	0.1414001	0.09677283	0.137766	0.1520268
937.883	8	0.403907	0.3319466	0.1417002	0.0948167	0.138034	0.1535868
937.983	8	0.389723	0.3270988	0.1406903	0.09537992	0.13625	0.1517429
938.083	8	0.401396	0.3067068	0.1395393	0.09375516	0.135664	0.1514836

938.183	8	0.401768	0.3108683	0.142302	0.09383562	0.137901	0.1501734
938.283	8	0.392468	0.3077005	0.1418749	0.09195179	0.135765	0.1517383
938.383	8	0.399133	0.3020456	0.1422311	0.09501191	0.140246	0.1500887
938.483	8	0.392005	0.297041	0.1445088	0.09560951	0.140179	0.1512648
938.583	8	0.385345	0.3124819	0.1476803	0.09826784	0.137598	0.1483546
938.683	8	0.392796	0.3271046	0.1450497	0.09689995	0.137981	0.1521325
938.783	8	0.391371	0.3432716	0.1389512	0.09760661	0.134266	0.1516593
938.883	8	0.398619	0.3436262	0.1408499	0.09666123	0.135786	0.1523053
938.983	8	0.380872	0.340113	0.1420151	0.09797043	0.138435	0.1522631
939.083	8	0.384224	0.3374883	0.1472581	0.0972	0.141657	0.1542776
939.183	8	0.38962	0.34055	0.1457876	0.1000121	0.141451	0.1555422
939.283	8	0.379106	0.3455152	0.148504	0.1009613	0.139765	0.1542523
939.383	8	0.388265	0.3510888	0.1484136	0.1027754	0.133225	0.1539611
939.483	8	0.389058	0.3471404	0.1483581	0.1002885	0.12981	0.1555397
939.583	8	0.394316	0.3500514	0.1481039	0.09829913	0.132195	0.1575475
939.683	8	0.399185	0.3511024	0.1485278	0.09951888	0.134772	0.1583544
939.783	8	0.403913	0.3438048	0.1473997	0.09959206	0.134393	0.1564596
939.883	8	0.399676	0.3428457	0.1489863	0.1029195	0.132747	0.1576582
939.983	8	0.387826	0.3460902	0.1457857	0.1047873	0.132433	0.1602055
940.083	8	0.382164	0.3481516	0.145375	0.1060635	0.135022	0.157737
940.183	8	0.393196	0.3503099	0.1452858	0.1084086	0.131827	0.157355
940.283	8	0.378369	0.3515384	0.1459466	0.1069221	0.127502	0.1593642
940.383	8	0.384367	0.349991	0.1465017	0.1077091	0.124324	0.1607451
940.483	8	0.371429	0.3506479	0.1471488	0.1073374	0.12986	0.1553323
940.583	8	0.38771	0.3471036	0.1461128	0.1073344	0.126012	0.1549678
940.683	8	0.388848	0.3404294	0.1446811	0.1097898	0.124108	0.1550544
940.783	8	0.3996	0.3346293	0.1472166	0.1101336	0.124225	0.1551505
940.883	8	0.387075	0.3328996	0.1472323	0.1085744	0.12688	0.1537899
940.983	8	0.402698	0.3358961	0.1443694	0.1073068	0.128911	0.1558464
941.083	8	0.394206	0.3226444	0.1460284	0.1100722	0.128282	0.1553378
941.183	8	0.393788	0.3086433	0.1441993	0.1100477	0.127717	0.150482
941.283	8	0.389289	0.3135269	0.1478078	0.1108944	0.125666	0.1531153
941.383	8	0.383709	0.3097409	0.1453778	0.1080589	0.129596	0.1519609
941.483	8	0.396401	0.3004006	0.1370445	0.103568	0.126155	0.1558709
941.583	8	0.39703	0.3002881	0.1438946	0.1014478	0.123699	0.155102
941.683	8	0.386169	0.3096672	0.1435857	0.09990554	0.125223	0.1551726
941.783	8	0.388162	0.3298252	0.1446505	0.1043062	0.125222	0.151856
941.883	8	0.39788	0.3418978	0.1482402	0.1030868	0.124737	0.1522266
941.983	8	0.399955	0.3392772	0.1436966	0.1029779	0.126073	0.1535682
942.083	8	0.399463	0.3335894	0.1435616	0.1042663	0.123264	0.1580626
942.183	8	0.386162	0.3302448	0.1475029	0.1028306	0.120454	0.1549125
942.283	8	0.395404	0.3337528	0.1477907	0.1010274	0.121017	0.154079
942.383	8	0.392697	0.3429827	0.1450853	0.1002751	0.122955	0.152811
942.483	8	0.399461	0.3470227	0.146248	0.09879816	0.124944	0.1528216
942.583	8	0.419044	0.3461612	0.1458913	0.1003562	0.123831	0.1551823
942.683	8	0.407427	0.3488763	0.1489292	0.09809258	0.12385	0.1539349
942.783	8	0.413435	0.3456071	0.1472529	0.1011115	0.125146	0.1501281
942.883	8	0.378589	0.3396721	0.1478744	0.1015906	0.125553	0.14672
942.983	8	0.388661	0.3399616	0.1474565	0.09899887	0.124299	0.1509601
943.083	8	0.396271	0.3445831	0.1433063	0.1008095	0.124621	0.1540266
943.183	8	0.39849	0.3462834	0.1433686	0.09932598	0.124125	0.1538786
943.283	8	0.401201	0.3488105	0.1424215	0.09774146	0.125552	0.154004
943.383	8	0.378541	0.3465239	0.146908	0.10105	0.124573	0.1545254
943.483	8	0.372958	0.3471763	0.1437249	0.1008777	0.125349	0.1535437
943.583	8	0.371499	0.345321	0.142394	0.1003488	0.126558	0.1528221
943.683	8	0.38145	0.3434263	0.1405823	0.09956758	0.124468	0.155271
943.783	8	0.403837	0.3370809	0.1405336	0.1005124	0.121194	0.1537462
943.883	8	0.407662	0.3300008	0.1415802	0.1017486	0.124963	0.1547916
943.983	8	0.412253	0.3326415	0.1439497	0.09967197	0.125576	0.1556399
944.083	8	0.404716	0.3327681	0.1419182	0.1047801	0.124761	0.1553599
944.183	8	0.397226	0.3208346	0.1436019	0.1061917	0.127862	0.1516122
944.283	8	0.411435	0.3042073	0.1423489	0.09965606	0.125857	0.1512566
944.383	8	0.39349	0.3178979	0.1400087	0.09981088	0.126025	0.1504778
944.483	8	0.39309	0.3080283	0.145518	0.1024568	0.128327	0.1492209
944.583	8	0.384767	0.2961608	0.1408342	0.1000446	0.128	0.1489114
944.683	8	0.405832	0.3108702	0.1409639	0.1044	0.130183	0.1488571
944.783	8	0.386999	0.3084126	0.1420105	0.1012952	0.134399	0.1488689
944.883	8	0.38614	0.340072	0.1395798	0.1022764	0.132807	0.1493695
944.983	8	0.39856	0.341708	0.1347445	0.1039499	0.130216	0.1497812
945.083	8	0.409008	0.3343431	0.1388976	0.1032999	0.129673	0.1463764
945.183	8	0.403077	0.3291355	0.1376735	0.1044487	0.132842	0.1496055
945.283	8	0.408429	0.3302432	0.1336454	0.09875735	0.134448	0.1497838
945.383	8	0.401334	0.3344569	0.1354482	0.100862	0.134591	0.1548847
945.483	8	0.388665	0.3410392	0.13354	0.101344	0.135511	0.1509854
945.583	8	0.395658	0.3479844	0.1342051	0.1024747	0.134785	0.1489598
945.683	8	0.397368	0.3431218	0.1314686	0.1027822	0.133027	0.1493027
945.783	8	0.395476	0.3488737	0.133857	0.102745	0.132238	0.1510839
945.883	8	0.403004	0.3426422	0.1325456	0.1057158	0.133428	0.1531433
945.983	8	0.392761	0.3379703	0.1329375	0.1025613	0.134237	0.152112
946.083	8	0.38571	0.3382743	0.1391723	0.1025778	0.135531	0.1505632
946.183	8	0.393108	0.3403601	0.1409739	0.1017117	0.135261	0.1494294
946.283	8	0.393575	0.3426233	0.1383352	0.1010036	0.130847	0.1491378
946.383	8	0.398314	0.3445597	0.1385924	0.1025849	0.130111	0.1415534
946.483	8	0.381812	0.3410254	0.1394556	0.1022488	0.131862	0.146459
946.583	8	0.385975	0.3441565	0.1390946	0.1022435	0.13234	0.1467281
946.683	8	0.389998	0.3434926	0.1375722	0.1014094	0.134867	0.1456124
946.783	8	0.378041	0.3393267	0.1386724	0.1018159	0.135549	0.1474046
946.883	8	0.390667	0.3367092	0.1394571	0.1027802	0.131527	0.1491255
946.983	8	0.399358	0.32844	0.1396554	0.1026112	0.130536	0.1497793
947.083	8	0.399707	0.3284632	0.1382563	0.1024592	0.130334	0.1523469
947.183	8	0.40105	0.3313747	0.1366599	0.1009805	0.13205	0.1532818
947.283	8	0.406748	0.3134595	0.1372824	0.1044563	0.133256	0.151715
947.383	8	0.404025	0.3059697	0.1385195	0.1063822	0.131122	0.1505944
947.483	8	0.387984	0.311756	0.1402188	0.1044679	0.126977	0.1517677
947.583	8	0.384105	0.3058665	0.1382105	0.106086	0.128353	0.1518004

947.683	8	0.392794	0.2971689	0.1422279	0.1084177	0.129166	0.1523833
947.783	8	0.387075	0.312297	0.1426859	0.1077967	0.130252	0.1498919
947.883	8	0.382484	0.3172503	0.1440182	0.1103056	0.129279	0.1510096
947.983	8	0.372735	0.3376268	0.1385852	0.1122387	0.131066	0.1511488
948.083	8	0.385411	0.3434606	0.1408171	0.1102038	0.129675	0.1492267
948.183	8	0.390742	0.3383765	0.142068	0.1097877	0.128047	0.149761
948.283	8	0.397325	0.3359356	0.1460612	0.1106541	0.127661	0.1549031
948.383	8	0.392643	0.3389034	0.1478539	0.1091818	0.129851	0.1566688
948.483	8	0.399806	0.3419005	0.1469178	0.1088166	0.126402	0.1570983
948.583	8	0.391826	0.3516567	0.1480276	0.109907	0.125769	0.1531224
948.683	8	0.402651	0.3488919	0.1475197	0.1099235	0.125977	0.1535487
948.783	8	0.397131	0.3469313	0.1481407	0.1082113	0.123444	0.1550022
948.883	8	0.382546	0.3485987	0.1478372	0.1076494	0.124883	0.1561135
948.983	8	0.398889	0.3422816	0.1469864	0.102803	0.123722	0.1538698
949.083	8	0.403607	0.3398893	0.1507926	0.1026493	0.122432	0.1571648
949.183	8	0.395594	0.3417373	0.1476778	0.1011003	0.121878	0.1588294
949.283	8	0.390152	0.3430355	0.1448982	0.1060202	0.120726	0.1573648
949.383	8	0.398976	0.345716	0.1434372	0.1061752	0.120164	0.1582445
949.483	8	0.401721	0.3491507	0.1458303	0.1059632	0.121079	0.1616537
949.583	8	0.402024	0.3458824	0.1457159	0.1056204	0.118521	0.1604303
949.683	8	0.387366	0.347324	0.147014	0.1030723	0.117431	0.1529183
949.783	8	0.394928	0.3469411	0.1467977	0.101829	0.117659	0.1516286
949.883	8	0.38859	0.3385287	0.1460865	0.1039415	0.119621	0.1514181
949.983	8	0.399499	0.3340251	0.1446306	0.1009926	0.118289	0.1523081
950.083	8	0.414466	0.3308797	0.1492115	0.102174	0.120486	0.1538167
950.183	8	0.397382	0.3337329	0.1450939	0.1002398	0.121115	0.1536731
950.283	8	0.43167	0.3290382	0.144246	0.1018891	0.124158	0.1565131
950.383	8	0.408986	0.3070132	0.1413535	0.1035878	0.126805	0.1496155
950.483	8	0.413454	0.308814	0.1439157	0.1027348	0.127835	0.1540501
950.583	8	0.408903	0.309675	0.1436262	0.104239	0.12626	0.1530264
950.683	8	0.399476	0.303868	0.138034	0.1022742	0.126475	0.1534759
950.783	8	0.408924	0.2988947	0.1398968	0.09985623	0.126774	0.1541379
950.883	8	0.405249	0.308772	0.1395011	0.1036359	0.126532	0.153848
950.983	8	0.379905	0.3247254	0.1414882	0.1042525	0.128867	0.1515635
951.083	8	0.380109	0.3382236	0.1458814	0.1045212	0.125737	0.1517246
951.183	8	0.393832	0.3403711	0.1459222	0.1031096	0.121244	0.1533045
951.283	8	0.412889	0.3343582	0.1405963	0.1056104	0.117754	0.1553601
951.383	8	0.418056	0.3316605	0.1427284	0.105482	0.124305	0.1562158
951.483	8	0.418484	0.3341163	0.1466873	0.1009438	0.125493	0.1532673
951.583	8	0.413893	0.338434	0.1436414	0.1054903	0.125378	0.1538106
951.683	8	0.406302	0.3468331	0.1434397	0.107153	0.127388	0.1510714
951.783	8	0.421051	0.3461585	0.1434449	0.1011935	0.12953	0.1533404
951.883	8	0.399044	0.3464144	0.1463825	0.09999128	0.127056	0.152739
951.983	8	0.392978	0.3476049	0.1476612	0.1042491	0.128631	0.1522178
952.083	8	0.386255	0.3411216	0.1456892	0.1008639	0.129571	0.1458028
952.183	8	0.394984	0.3376366	0.1442533	0.1020334	0.132338	0.1496953
952.283	8	0.385972	0.3392054	0.1425631	0.09897681	0.134584	0.1508899
952.383	8	0.384464	0.3425174	0.1442325	0.100811	0.134517	0.1530805
952.483	8	0.392539	0.3444724	0.1384728	0.1019186	0.132385	0.1525304
952.583	8	0.401537	0.3451762	0.1388948	0.1021522	0.131203	0.1514629
952.683	8	0.400741	0.345173	0.139468	0.1026772	0.129196	0.1536944
952.783	8	0.406516	0.3448786	0.1404301	0.09674607	0.13027	0.149637
952.883	8	0.401962	0.3434563	0.1402599	0.09626267	0.130927	0.1520732
952.983	8	0.389264	0.3355229	0.1404985	0.09833208	0.132182	0.1510562
953.083	8	0.39334	0.3336002	0.1386362	0.1010998	0.130624	0.1516933
953.183	8	0.390562	0.3312327	0.1425337	0.1023707	0.129009	0.1530256
953.283	8	0.387332	0.3399128	0.1417893	0.1002141	0.128624	0.1530446
953.383	8	0.396392	0.3241314	0.1427097	0.1018918	0.132213	0.1486526
953.483	8	0.393002	0.31037	0.139263	0.09846143	0.134146	0.1477458
953.583	8	0.380334	0.3164483	0.1408685	0.09857301	0.134191	0.1472837
953.683	8	0.389132	0.3135344	0.1414622	0.09843872	0.136015	0.1491479
953.783	8	0.398859	0.3012842	0.1411722	0.1004494	0.135439	0.1494817
953.883	8	0.403554	0.3017535	0.1396318	0.1003552	0.137132	0.1501715
953.983	8	0.384803	0.3194689	0.1391567	0.1012411	0.136174	0.1507253
954.083	8	0.396082	0.3389002	0.1410353	0.1005732	0.136432	0.1496399
954.183	8	0.393865	0.3457194	0.1366309	0.09984322	0.136678	0.149928
954.283	8	0.380493	0.3427565	0.1401918	0.09987341	0.136614	0.1475583
954.383	8	0.387758	0.343581	0.1393063	0.09912393	0.134978	0.1489962
954.483	8	0.393464	0.3440097	0.1396741	0.09702531	0.135579	0.1499674
954.583	8	0.402423	0.351711	0.1354245	0.09761436	0.137247	0.1523082
954.683	8	0.407112	0.3573633	0.1360877	0.09378491	0.136096	0.1513451
954.783	8	0.4125	0.3636991	0.1394473	0.0971194	0.136449	0.1495751
954.883	8	0.407171	0.3545141	0.13882	0.09929892	0.136576	0.1501816
954.983	8	0.393531	0.3496841	0.1345519	0.1011925	0.131307	0.1512701
955.083	8	0.383655	0.3538463	0.1360976	0.1015036	0.133422	0.1507426
955.183	8	0.397998	0.3523045	0.1360254	0.1056139	0.131316	0.1500247
955.283	8	0.387503	0.3523557	0.133537	0.1052128	0.131652	0.1480637
955.383	8	0.383286	0.3571844	0.1416894	0.1101631	0.130137	0.1462396
955.483	8	0.369712	0.3598855	0.1378741	0.1114543	0.132516	0.1498592
955.583	8	0.389615	0.3643971	0.1345829	0.1094592	0.13074	0.1475974
955.683	8	0.392175	0.3624805	0.1374136	0.1096813	0.129425	0.1473231
955.783	8	0.401417	0.36486	0.1419083	0.1077337	0.127764	0.1497901
955.883	8	0.385959	0.3615094	0.1407467	0.1086593	0.128923	0.1458697
955.983	8	0.398513	0.358799	0.1406868	0.1093227	0.130336	0.1454979
956.083	8	0.39119	0.3511897	0.1393682	0.1112241	0.129056	0.1480652
956.183	8	0.397689	0.3461063	0.1423026	0.1058916	0.127874	0.1498147
956.283	8	0.39515	0.3431968	0.1401552	0.1058934	0.12598	0.1509787
956.383	8	0.385571	0.3415257	0.1392744	0.1052167	0.128809	0.1513942
956.483	8	0.39599	0.3264217	0.1387464	0.1021409	0.127573	0.1522109
956.583	8	0.400102	0.3339981	0.1392161	0.1022142	0.126671	0.1516645
956.683	8	0.389844	0.3500845	0.1443088	0.1038386	0.125908	0.1486506
956.783	8	0.387909	0.3276513	0.1424926	0.1061025	0.126131	0.149827
956.883	8	0.40071	0.3056014	0.1420623	0.1036093	0.124281	0.1487336
956.983	8	0.400021	0.3362453	0.1425539	0.1033933	0.124592	0.1472374
957.083	8	0.403158	0.3439128	0.144104	0.1031889	0.12543	0.1480685

957.183	8	0.386214	0.3562025	0.1408629	0.1002114	0.124609	0.1485029
957.283	8	0.395578	0.3578834	0.1407242	0.1009728	0.126912	0.1487118
957.383	8	0.393034	0.3525332	0.1443408	0.101705	0.125811	0.1501634
957.483	8	0.401301	0.3554347	0.1487153	0.09918603	0.122651	0.1519877
957.583	8	0.411877	0.356558	0.1524123	0.09915312	0.122122	0.1537231
957.683	8	0.395024	0.3610372	0.148674	0.09778745	0.123771	0.1570105
957.783	8	0.420446	0.3659729	0.1489826	0.1023265	0.126918	0.154665
957.883	8	0.391859	0.3701358	0.150278	0.1045689	0.12749	0.1556342
957.983	8	0.405373	0.3552592	0.1520574	0.1046134	0.127403	0.1559519
958.083	8	0.40072	0.3587866	0.1500767	0.1060599	0.126079	0.155899
958.183	8	0.406107	0.3522966	0.1488478	0.1031565	0.124857	0.1565908
958.283	8	0.422383	0.3521014	0.147715	0.09870999	0.125461	0.1577761
958.383	8	0.433761	0.3549994	0.1474555	0.09907069	0.123714	0.1587022
958.483	8	0.409849	0.3595677	0.1467952	0.09680124	0.125962	0.1593232
958.583	8	0.44423	0.3629287	0.1465484	0.09995286	0.124099	0.1564752
958.683	8	0.445241	0.3658519	0.1488477	0.1003418	0.119698	0.1573516
958.783	8	0.440898	0.3649541	0.1491108	0.09934542	0.118313	0.1584776
958.883	8	0.436248	0.3677294	0.1496278	0.09714749	0.120519	0.1551706
958.983	8	0.408787	0.3638302	0.149454	0.09522875	0.122139	0.1560481
959.083	8	0.419847	0.3579151	0.1483108	0.0967985	0.122864	0.155084
959.183	8	0.410799	0.3505199	0.1470355	0.09945009	0.125211	0.1551472
959.335	8.5	0.400333	0.3382738	0.1493242	0.09412783	0.127344	0.1572513
959.435	8.5	0.398018	0.3247219	0.1476444	0.09708775	0.126922	0.1558147
959.535	8.5	0.409764	0.3148581	0.1454715	0.1004505	0.129211	0.1573828
959.635	8.5	0.402834	0.3166257	0.1460801	0.0980882	0.127423	0.1493749
959.735	8.5	0.398062	0.2956465	0.1461229	0.09975881	0.129576	0.1485558
959.835	8.5	0.409323	0.308781	0.1501932	0.1009552	0.130866	0.1507373
959.935	8.5	0.403369	0.3283723	0.1487134	0.1003364	0.13351	0.1494776
960.035	8.5	0.416125	0.3546734	0.1394874	0.1010307	0.134198	0.1495676
960.135	8.5	0.409134	0.3497983	0.1458024	0.09551423	0.135898	0.1526698
960.235	8.5	0.39518	0.351133	0.1466712	0.09495131	0.13245	0.1502231
960.335	8.5	0.399161	0.3496608	0.1484188	0.09779477	0.132773	0.1481572
960.435	8.5	0.389238	0.3534363	0.1493204	0.09891378	0.133291	0.1469453
960.535	8.5	0.393014	0.3594468	0.1491674	0.09954315	0.136881	0.149692
960.635	8.5	0.397209	0.3660874	0.1453035	0.1018388	0.135332	0.1535682
960.735	8.5	0.388773	0.3732431	0.1427265	0.09831873	0.136451	0.1487854
960.835	8.5	0.379013	0.3595231	0.1438342	0.09852244	0.134356	0.1516856
960.935	8.5	0.389824	0.3529924	0.1479741	0.09693052	0.134965	0.1507517
961.035	8.5	0.404008	0.3492639	0.1468043	0.09658124	0.134301	0.1534228
961.135	8.5	0.397865	0.3511529	0.1443548	0.09689922	0.133929	0.1524238
961.235	8.5	0.395442	0.3541763	0.1451223	0.09771786	0.135473	0.1493142
961.335	8.5	0.403328	0.3591118	0.1438089	0.09776949	0.132419	0.143845
961.435	8.5	0.384891	0.3634529	0.144712	0.09932521	0.134979	0.1442284
961.535	8.5	0.391196	0.3688119	0.144419	0.09970249	0.137925	0.1468502
961.635	8.5	0.397462	0.3671604	0.1453178	0.1001839	0.138329	0.1495143
961.735	8.5	0.404644	0.3692853	0.1430443	0.09849282	0.141508	0.1493593
961.835	8.5	0.413139	0.3679272	0.1427504	0.09733705	0.143165	0.1490518
961.935	8.5	0.410996	0.3613744	0.1416954	0.09598383	0.136304	0.1515116
962.035	8.5	0.402718	0.3515742	0.1414314	0.0974853	0.135218	0.1497544
962.135	8.5	0.386534	0.3423373	0.1448598	0.1002083	0.134432	0.150925
962.235	8.5	0.383117	0.3395515	0.1431761	0.1012194	0.133768	0.1525288
962.335	8.5	0.399532	0.3340926	0.1438571	0.1021003	0.13196	0.1532539
962.435	8.5	0.395768	0.3326843	0.1425767	0.1032781	0.133254	0.1536214
962.535	8.5	0.390747	0.3312766	0.1405158	0.1041304	0.129896	0.1534158
962.635	8.5	0.391884	0.3357925	0.1411548	0.1067272	0.132655	0.1522052
962.735	8.5	0.4012	0.3117847	0.1414825	0.108622	0.130765	0.149911
962.835	8.5	0.403704	0.3192751	0.1411401	0.1085088	0.130457	0.1499123
962.935	8.5	0.394744	0.3342363	0.1417289	0.1084133	0.131516	0.1497447
963.035	8.5	0.393494	0.3493232	0.1402898	0.1094853	0.131778	0.1492218
963.135	8.5	0.394068	0.3483593	0.1402463	0.111159	0.129397	0.147691
963.235	8.5	0.403666	0.3466827	0.1414811	0.1128169	0.125607	0.148676
963.335	8.5	0.389301	0.3464634	0.1399685	0.108108	0.125185	0.1493164
963.435	8.5	0.386374	0.349889	0.1364031	0.1073188	0.127274	0.1469246
963.535	8.5	0.390211	0.3549961	0.1358389	0.1011741	0.130111	0.1472974
963.635	8.5	0.390759	0.3598568	0.1380909	0.09943464	0.131139	0.1441287
963.735	8.5	0.387609	0.3687487	0.1365946	0.1011355	0.130883	0.1487265
963.835	8.5	0.386168	0.357228	0.1351509	0.1027099	0.130257	0.147834
963.935	8.5	0.401404	0.3511012	0.135019	0.1021828	0.131933	0.1508408
964.035	8.5	0.398949	0.3433007	0.1352111	0.1050131	0.127302	0.1474838
964.135	8.5	0.387287	0.3428191	0.1378215	0.1037779	0.128245	0.1482104
964.235	8.5	0.3875	0.3432444	0.1391499	0.1011189	0.127107	0.1485685
964.335	8.5	0.382749	0.3470142	0.1370704	0.1012571	0.126419	0.1479567
964.435	8.5	0.399452	0.3518372	0.1375563	0.1030878	0.125848	0.1473726
964.535	8.5	0.406783	0.3568447	0.1411961	0.09960028	0.126641	0.1470605
964.635	8.5	0.391498	0.3568554	0.1409748	0.09837832	0.127023	0.1471516
964.735	8.5	0.414586	0.359576	0.1395784	0.0995269	0.12636	0.1458509
964.835	8.5	0.385058	0.3577039	0.1388972	0.1022283	0.126764	0.1470447
964.935	8.5	0.398038	0.3523706	0.1415702	0.1017939	0.127168	0.1423566
965.035	8.5	0.392825	0.3432387	0.138432	0.1032885	0.124567	0.1461739
965.135	8.5	0.401006	0.3363027	0.1399611	0.1046393	0.122845	0.1467741
965.235	8.5	0.394705	0.3362068	0.1416973	0.09875827	0.121609	0.147228
965.335	8.5	0.382173	0.3316793	0.1464981	0.1006193	0.127013	0.1486577
965.435	8.5	0.376967	0.3250926	0.1469031	0.1020747	0.125646	0.1501969
965.535	8.5	0.386056	0.3210885	0.1445649	0.09953064	0.127703	0.1475622
965.635	8.5	0.398678	0.3273835	0.1453869	0.1029988	0.127862	0.1504225
965.735	8.5	0.416973	0.2980332	0.146581	0.1028241	0.127067	0.1528167
965.835	8.5	0.410973	0.3045225	0.143709	0.103297	0.129008	0.1530273
965.935	8.5	0.419487	0.3322062	0.142085	0.1019086	0.129064	0.1533584
966.035	8.5	0.399773	0.337752	0.1461832	0.1043932	0.130654	0.1530768
966.135	8.5	0.408666	0.3413569	0.148923	0.1048294	0.128174	0.1511723
966.235	8.5	0.395729	0.3401885	0.149989	0.0977094	0.122625	0.1538017
966.335	8.5	0.386781	0.3382898	0.148578	0.09967281	0.121749	0.1489755
966.435	8.5	0.379476	0.3435335	0.1482864	0.1011599	0.125931	0.1511147
966.535	8.5	0.396134	0.3487332	0.1476994	0.09821523	0.128064	0.1511284
966.635	8.5	0.38778	0.3545917	0.1458682	0.09964871	0.126647	0.1525919

966.735	8.5	0.381986	0.3647094	0.1452286	0.09777158	0.128483	0.1504266
966.835	8.5	0.390869	0.3528976	0.1470044	0.09838507	0.128885	0.1513196
966.935	8.5	0.400988	0.3482073	0.1482523	0.1003994	0.129784	0.1522232
967.035	8.5	0.390402	0.3418497	0.144533	0.1038962	0.134528	0.1525435
967.135	8.5	0.401761	0.3403991	0.1437953	0.09788667	0.132783	0.1513141
967.235	8.5	0.38615	0.340333	0.1474211	0.09718813	0.135996	0.1530693
967.335	8.5	0.386641	0.3453106	0.1480372	0.09822624	0.134766	0.1555247
967.435	8.5	0.388828	0.3473527	0.1518489	0.1000583	0.136618	0.1549522
967.535	8.5	0.376457	0.3531148	0.1498981	0.09942213	0.1339	0.156148
967.635	8.5	0.387225	0.3550476	0.1481685	0.09944297	0.133274	0.15597
967.735	8.5	0.385136	0.3569242	0.1480451	0.100873	0.131764	0.1588075
967.835	8.5	0.378331	0.3558479	0.1513324	0.09968844	0.134755	0.1566865
967.935	8.5	0.377163	0.3505373	0.1478775	0.1002157	0.134257	0.1575792
968.035	8.5	0.383185	0.3419299	0.1488499	0.09585159	0.135162	0.159611
968.135	8.5	0.388583	0.335554	0.1462092	0.09611107	0.134589	0.1587729
968.235	8.5	0.378182	0.3350677	0.1512416	0.09667623	0.134012	0.1533359
968.335	8.5	0.385486	0.3330112	0.1478228	0.09485011	0.135402	0.1535203
968.435	8.5	0.386561	0.3312327	0.1411025	0.09396899	0.13827	0.1547019
968.535	8.5	0.377936	0.3184998	0.1448229	0.09700552	0.13527	0.1553817
968.635	8.5	0.389888	0.3239112	0.1414059	0.09717727	0.133046	0.1542821
968.735	8.5	0.382866	0.3016617	0.1445685	0.0984082	0.133573	0.1563264
968.835	8.5	0.402022	0.3212309	0.1457128	0.09911695	0.130287	0.1556923
968.935	8.5	0.41012	0.3355264	0.1436459	0.09790196	0.133642	0.1517603
969.035	8.5	0.406415	0.3516812	0.1466458	0.09864177	0.138464	0.1543783
969.135	8.5	0.394132	0.3522889	0.1489061	0.1012042	0.139936	0.1524344
969.235	8.5	0.379482	0.3563713	0.145648	0.1009808	0.139337	0.1534256
969.335	8.5	0.388212	0.3558196	0.1453363	0.103502	0.139758	0.1536117
969.435	8.5	0.391394	0.3601965	0.1458218	0.1074182	0.136964	0.1535811
969.535	8.5	0.389557	0.3672685	0.148729	0.1068724	0.136537	0.1501893
969.635	8.5	0.376784	0.3701037	0.1458298	0.1083236	0.136259	0.1506183
969.735	8.5	0.389264	0.3775377	0.1458217	0.1087475	0.134672	0.152248
969.835	8.5	0.395031	0.3624268	0.1468852	0.1066041	0.13505	0.1558811
969.935	8.5	0.405032	0.3537056	0.1463492	0.1095017	0.131213	0.1548798
970.035	8.5	0.388443	0.3535137	0.1459527	0.1096281	0.126046	0.1540624
970.135	8.5	0.404476	0.3527488	0.1464553	0.1110461	0.132634	0.1537758
970.235	8.5	0.411221	0.3549363	0.1450668	0.11153	0.132155	0.154487
970.335	8.5	0.419474	0.361183	0.1424908	0.1118338	0.131895	0.1561572
970.435	8.5	0.383247	0.3651549	0.1424022	0.1108377	0.130152	0.1536748
970.535	8.5	0.397506	0.3701856	0.1414562	0.1073357	0.133678	0.149995
970.635	8.5	0.394717	0.3722034	0.1387131	0.102624	0.130538	0.1460996
970.735	8.5	0.386522	0.3728289	0.1436212	0.1009536	0.128755	0.149598
970.835	8.5	0.387024	0.3713233	0.14182	0.1001153	0.129724	0.1524687
970.935	8.5	0.390429	0.3678514	0.1430373	0.1026082	0.133846	0.1530164
971.035	8.5	0.397148	0.3574461	0.1412295	0.1034155	0.135358	0.1521794
971.135	8.5	0.395985	0.3494371	0.1393568	0.1046917	0.134842	0.1519898
971.235	8.5	0.382554	0.3472478	0.1424219	0.1046744	0.134346	0.1492655
971.335	8.5	0.388056	0.3403527	0.1402287	0.1029642	0.132206	0.1506087
971.435	8.5	0.390909	0.3261306	0.1398347	0.1040823	0.135229	0.1535867
971.535	8.5	0.396534	0.3220938	0.1393234	0.1020492	0.131013	0.151516
971.635	8.5	0.411681	0.3322788	0.1366702	0.09882518	0.129554	0.1528844
971.735	8.5	0.395026	0.337325	0.1382532	0.09872253	0.127669	0.1535185
971.835	8.5	0.405391	0.3393185	0.1367513	0.1028927	0.127949	0.1526395
971.935	8.5	0.381285	0.3434606	0.1351375	0.1038027	0.127446	0.1472192
972.035	8.5	0.396425	0.3524399	0.134956	0.1028745	0.131143	0.1489385
972.135	8.5	0.39161	0.3490939	0.1333663	0.1054405	0.129923	0.1475504
972.235	8.5	0.404907	0.3487412	0.1355968	0.1039309	0.129437	0.1480027
972.335	8.5	0.387405	0.3501883	0.1300242	0.1015952	0.128569	0.1458992
972.435	8.5	0.377533	0.354066	0.1305666	0.1041647	0.129886	0.1463977
972.535	8.5	0.378742	0.3614618	0.1326722	0.101616	0.126645	0.1501547
972.635	8.5	0.381147	0.365639	0.1279804	0.1030082	0.125896	0.1465575
972.735	8.5	0.400927	0.3720466	0.136135	0.1009515	0.12621	0.1472163
972.835	8.5	0.413912	0.3587385	0.1349058	0.1017281	0.127974	0.1450502
972.935	8.5	0.414754	0.352653	0.1322764	0.09888723	0.130129	0.1491237
973.035	8.5	0.408444	0.3467532	0.1354689	0.0980373	0.132954	0.1493229
973.135	8.5	0.394187	0.3448571	0.1387692	0.1015191	0.131771	0.1522287
973.235	8.5	0.401725	0.3442236	0.1361466	0.09947192	0.127866	0.1495328
973.335	8.5	0.391461	0.3501618	0.1367224	0.09623226	0.128295	0.1497906
973.435	8.5	0.391844	0.3540162	0.1361587	0.09710027	0.128367	0.150121
973.535	8.5	0.382095	0.3593231	0.139102	0.09875063	0.126361	0.1524371
973.635	8.5	0.391454	0.3621372	0.1381589	0.09597654	0.125289	0.1514367
973.735	8.5	0.382341	0.3619392	0.1384024	0.09463836	0.123169	0.1513038
973.835	8.5	0.412939	0.3605678	0.1374918	0.09276021	0.121953	0.1504491
973.935	8.5	0.432721	0.3544736	0.1384538	0.09288222	0.126624	0.1484064
974.035	8.5	0.420767	0.3434832	0.1372372	0.09715746	0.127722	0.1496525
974.135	8.5	0.445423	0.3364682	0.136421	0.1006231	0.125035	0.1425853
974.235	8.5	0.428689	0.3361616	0.138803	0.09655525	0.125991	0.1456128
974.335	8.5	0.403413	0.3385248	0.139705	0.1006611	0.126258	0.1487361
974.435	8.5	0.423876	0.3229887	0.1380215	0.102083	0.124772	0.1466204
974.535	8.5	0.407161	0.3187458	0.13901	0.103571	0.127197	0.1488228
974.635	8.5	0.403522	0.3291936	0.1404444	0.1018451	0.128437	0.1495437
974.735	8.5	0.418534	0.3370004	0.1424671	0.100628	0.128621	0.1501182
974.835	8.5	0.401987	0.3463116	0.1417178	0.09879448	0.128223	0.1516066
974.935	8.5	0.396428	0.343766	0.1429673	0.09746122	0.126815	0.1498191
975.035	8.5	0.397879	0.3494864	0.1434817	0.09671099	0.126633	0.1504413
975.135	8.5	0.406312	0.3472514	0.1430771	0.09594936	0.130548	0.1521737
975.235	8.5	0.393874	0.3489566	0.1416575	0.09796122	0.131945	0.1524925
975.335	8.5	0.399758	0.3511544	0.1420809	0.09995348	0.132067	0.1508289
975.435	8.5	0.406396	0.3590401	0.1427857	0.09862932	0.133954	0.1515667
975.535	8.5	0.400085	0.365885	0.1434648	0.09813102	0.13414	0.1504129
975.635	8.5	0.395725	0.3705545	0.1405435	0.1014251	0.134743	0.154099
975.735	8.5	0.3963	0.3776412	0.1405661	0.1030257	0.135991	0.1525376
975.835	8.5	0.402622	0.3641519	0.1438691	0.1057399	0.134271	0.1547945
975.935	8.5	0.412925	0.3583276	0.1446474	0.1079673	0.134404	0.1531518
976.035	8.5	0.41492	0.3525553	0.1477283	0.1064685	0.133231	0.1559949
976.135	8.5	0.407014	0.3559231	0.1476382	0.1067532	0.132926	0.1549116

976.235	8.5	0.38539	0.3582639	0.1445058	0.1094685	0.133673	0.1530061
976.335	8.5	0.377297	0.36616	0.1455369	0.1096806	0.134389	0.1506019
976.435	8.5	0.392746	0.3692893	0.1436226	0.1094696	0.134902	0.1528267
976.535	8.5	0.388083	0.3741249	0.1435142	0.1101091	0.135279	0.1534341
976.635	8.5	0.389872	0.3756301	0.1456122	0.1094446	0.136504	0.1558503
976.735	8.5	0.3733	0.3751615	0.146264	0.108405	0.138713	0.1539567
976.835	8.5	0.39578	0.373793	0.1474169	0.1101599	0.136162	0.157517
976.935	8.5	0.394655	0.3681933	0.1424778	0.1127353	0.134361	0.1565645
977.035	8.5	0.406298	0.3565249	0.1420828	0.1132515	0.134093	0.155643
977.135	8.5	0.396778	0.3491896	0.1402475	0.1104908	0.135009	0.154913
977.235	8.5	0.392423	0.346635	0.14023	0.1089624	0.136415	0.1588526
977.335	8.5	0.395718	0.3415523	0.1445215	0.1103472	0.135934	0.1596942
977.435	8.5	0.390638	0.3259973	0.143453	0.1104533	0.13107	0.1529974
977.535	8.5	0.376764	0.3196591	0.142553	0.1100622	0.128197	0.1536453
977.635	8.5	0.39749	0.3310364	0.144942	0.1042772	0.132339	0.1540892
977.735	8.5	0.390516	0.3441476	0.1451604	0.1029385	0.133936	0.1527348
977.835	8.5	0.388203	0.3421674	0.1446573	0.102753	0.132732	0.1545405
977.935	8.5	0.382853	0.3419727	0.1452334	0.1037639	0.130045	0.1543993
978.035	8.5	0.393418	0.3407623	0.1467074	0.1044954	0.130949	0.155807
978.135	8.5	0.391249	0.3410985	0.1470381	0.1052001	0.129332	0.1485446
978.235	8.5	0.398359	0.3386291	0.1463956	0.1036979	0.130075	0.151081
978.335	8.5	0.384268	0.3375103	0.1466097	0.1034702	0.12907	0.1481544
978.435	8.5	0.387333	0.3404766	0.1448136	0.1018797	0.130306	0.1490562
978.535	8.5	0.4046	0.3461824	0.14608	0.1034299	0.133517	0.1511603
978.635	8.5	0.401249	0.3517124	0.1426421	0.1016107	0.13144	0.1533099
978.735	8.5	0.410183	0.358278	0.1444267	0.1027401	0.130358	0.1511154
978.835	8.5	0.411545	0.3518553	0.1427358	0.1009991	0.128119	0.1488169
978.935	8.5	0.426439	0.3540232	0.141052	0.1030113	0.131888	0.1490019
979.035	8.5	0.420515	0.3440453	0.1381283	0.102396	0.128576	0.1517451
979.135	8.5	0.410166	0.3434561	0.1369923	0.1002922	0.127566	0.153437
979.235	8.5	0.402159	0.3441885	0.1391737	0.1025382	0.129125	0.151309
979.335	8.5	0.418498	0.3478386	0.1398522	0.1016066	0.128741	0.1522557
979.435	8.5	0.415284	0.3494978	0.1382441	0.1019712	0.126089	0.151086
979.535	8.5	0.401354	0.3537398	0.1369803	0.1038821	0.125011	0.1525696
979.635	8.5	0.399655	0.3537599	0.1380735	0.1018265	0.12533	0.1515263
979.735	8.5	0.404115	0.3527503	0.1392123	0.1038212	0.123328	0.1497739
979.835	8.5	0.418165	0.3525536	0.1397183	0.1018103	0.121639	0.1426207
979.935	8.5	0.413596	0.3475922	0.1397241	0.103949	0.122538	0.1480893
980.035	8.5	0.420372	0.3421684	0.13901	0.100636	0.119384	0.1523797
980.135	8.5	0.427784	0.3399951	0.1384835	0.1015549	0.117078	0.1532154
980.235	8.5	0.406867	0.338727	0.1343459	0.1032944	0.121924	0.1548692
980.335	8.5	0.416993	0.340839	0.1368613	0.09840139	0.124528	0.1519954
980.435	8.5	0.400861	0.3244761	0.134775	0.09635925	0.1249	0.1526423
980.535	8.5	0.386279	0.3240175	0.1334641	0.1003781	0.124826	0.1504601
980.635	8.5	0.401475	0.3342419	0.1343171	0.09848247	0.122752	0.1508414
980.735	8.5	0.405715	0.3434455	0.1345643	0.1017705	0.121486	0.1534392
980.835	8.5	0.395694	0.3445665	0.1350348	0.09932586	0.125104	0.154978
980.935	8.5	0.406907	0.3402656	0.1266381	0.09784146	0.124531	0.1503888
981.035	8.5	0.416031	0.3441411	0.1287831	0.09970876	0.125394	0.1497777
981.135	8.5	0.40119	0.3475461	0.1311154	0.1017406	0.12339	0.1508617
981.235	8.5	0.42063	0.3434407	0.130532	0.09986492	0.117763	0.1497889
981.335	8.5	0.392622	0.3396063	0.1321939	0.0995344	0.117733	0.1481921
981.435	8.5	0.386122	0.3448608	0.1312598	0.1000869	0.124719	0.1484367
981.535	8.5	0.388523	0.3496425	0.1310157	0.09784201	0.124153	0.1497124
981.635	8.5	0.388643	0.35371	0.136149	0.09690532	0.126873	0.1469072
981.735	8.5	0.389547	0.3605541	0.138501	0.09673583	0.130262	0.1469262
981.835	8.5	0.393017	0.3518997	0.1351368	0.0981997	0.127673	0.1498531
981.935	8.5	0.38767	0.3508816	0.1336284	0.09770597	0.125549	0.1512718
982.035	8.5	0.376021	0.3420996	0.1370971	0.09809706	0.126316	0.1545325
982.135	8.5	0.385009	0.340833	0.1374061	0.09436351	0.126281	0.1496401
982.235	8.5	0.393497	0.3393947	0.1373795	0.09435179	0.129151	0.1515484
982.335	8.5	0.377216	0.3436547	0.1378084	0.09488942	0.13108	0.1513359
982.435	8.5	0.378542	0.3463067	0.1366734	0.09549603	0.131316	0.1513229
982.535	8.5	0.386018	0.35115	0.1404404	0.0937742	0.130662	0.1495636
982.635	8.5	0.379283	0.3524093	0.1386921	0.09564525	0.130904	0.1505601
982.735	8.5	0.391648	0.3521092	0.1408667	0.09806651	0.13058	0.149638
982.835	8.5	0.390278	0.351217	0.1398339	0.101216	0.132717	0.1484156
982.935	8.5	0.400897	0.347184	0.1403556	0.101578	0.134028	0.1424791
983.035	8.5	0.400937	0.3414521	0.1363533	0.09932366	0.134922	0.1449312
983.135	8.5	0.400574	0.3375964	0.1402092	0.1015598	0.132782	0.1473644
983.235	8.5	0.388303	0.3373313	0.142288	0.1036261	0.13087	0.1451759
983.335	8.5	0.383096	0.3392897	0.1441236	0.1052086	0.130296	0.147125
983.435	8.5	0.381241	0.3204424	0.142426	0.108233	0.133938	0.1492169
983.535	8.5	0.392961	0.3198784	0.1445299	0.1115004	0.132135	0.148619
983.635	8.5	0.382952	0.3308617	0.1440043	0.1105561	0.13281	0.1513044
983.735	8.5	0.377806	0.3375221	0.1444281	0.1090992	0.132775	0.1507909
983.835	8.5	0.379034	0.3324675	0.143803	0.1096286	0.131978	0.1509772
983.935	8.5	0.393648	0.335083	0.1434286	0.1098589	0.132544	0.1507402
984.035	8.5	0.398073	0.3456846	0.1456291	0.1116909	0.134329	0.149706
984.135	8.5	0.390594	0.3416094	0.1439	0.1118976	0.135924	0.1490843
984.235	8.5	0.395265	0.3379058	0.1420383	0.111734	0.137332	0.1495028
984.335	8.5	0.398925	0.3361354	0.1443198	0.1128873	0.136509	0.1501212
984.435	8.5	0.398768	0.3387764	0.1449765	0.1126485	0.132025	0.1482031
984.535	8.5	0.385578	0.3455227	0.1461495	0.109576	0.13307	0.149591
984.635	8.5	0.392204	0.3498292	0.1490748	0.1096035	0.135504	0.1501231
984.735	8.5	0.395837	0.3565662	0.148553	0.101908	0.134665	0.1533299
984.835	8.5	0.398727	0.3490871	0.1439432	0.1010516	0.134664	0.1534091
984.935	8.5	0.394166	0.3495634	0.1460608	0.1010481	0.131431	0.1529874
985.035	8.5	0.393715	0.3409877	0.1458237	0.1039378	0.128086	0.1529228
985.135	8.5	0.397323	0.3393564	0.1448467	0.1039182	0.13163	0.1563213
985.235	8.5	0.396322	0.3384428	0.1443331	0.1051375	0.128726	0.1554044
985.335	8.5	0.391666	0.3414316	0.1446971	0.1070089	0.129058	0.1557134
985.435	8.5	0.393205	0.3429463	0.1448057	0.104884	0.129338	0.1547499
985.535	8.5	0.393323	0.3469539	0.1416612	0.1051395	0.131388	0.1550641
985.635	8.5	0.400956	0.3475794	0.1430719	0.1033268	0.129762	0.1565661

985.735	8.5	0.415815	0.346949	0.1429471	0.1013674	0.129432	0.1555986
985.835	8.5	0.404819	0.347353	0.1452748	0.1034361	0.127688	0.1581002
985.935	8.5	0.421539	0.3459733	0.1479696	0.1055136	0.128416	0.1580116
986.035	8.5	0.388665	0.3382878	0.1439682	0.1089125	0.131976	0.1543259
986.135	8.5	0.403878	0.33748	0.1410208	0.1063066	0.131233	0.1557922
986.235	8.5	0.406174	0.3387107	0.1460708	0.1062234	0.130004	0.1559161
986.335	8.5	0.420566	0.3393951	0.1454511	0.104765	0.128366	0.155146
986.435	8.5	0.406479	0.3221437	0.142893	0.1021966	0.130152	0.1521347
986.535	8.5	0.396468	0.3147663	0.1443831	0.1039541	0.127691	0.1533493
986.635	8.5	0.383717	0.3312116	0.1485729	0.1000006	0.125232	0.1515028
986.735	8.5	0.401273	0.3362207	0.1480223	0.09986677	0.124147	0.1511632
986.835	8.5	0.425459	0.3366643	0.1450768	0.101261	0.124425	0.1523978
986.935	8.5	0.433135	0.3366036	0.1478299	0.1023049	0.121569	0.1529059
987.035	8.5	0.430056	0.3410577	0.1485947	0.1000633	0.122248	0.1552541
987.135	8.5	0.428469	0.3445351	0.1464205	0.09986344	0.121934	0.154718
987.235	8.5	0.410075	0.339281	0.1425559	0.1021341	0.121853	0.1528137
987.335	8.5	0.412136	0.3343229	0.1439093	0.1009275	0.123673	0.151193
987.435	8.5	0.395869	0.3360722	0.1398845	0.09450286	0.124651	0.1500796
987.535	8.5	0.38996	0.3404735	0.1400921	0.09894679	0.123668	0.154068
987.635	8.5	0.37801	0.3468711	0.1395858	0.09776575	0.119087	0.1525474
987.735	8.5	0.394238	0.3551022	0.1392	0.09760632	0.119431	0.1534813
987.835	8.5	0.383123	0.3500765	0.1432468	0.09832806	0.121402	0.1525606
987.935	8.5	0.393195	0.3514593	0.1412181	0.09732305	0.122354	0.1509999
988.035	8.5	0.39338	0.3430023	0.1385627	0.09845855	0.122085	0.1513527
988.135	8.5	0.39736	0.3394247	0.1386821	0.09923248	0.120633	0.1497408
988.235	8.5	0.414165	0.3379664	0.1384325	0.1003738	0.119786	0.1448117
988.335	8.5	0.411786	0.341622	0.1429105	0.09551195	0.120327	0.1500625
988.435	8.5	0.3895	0.3420246	0.141239	0.09623093	0.119871	0.1529737
988.535	8.5	0.39925	0.3455968	0.1411918	0.09615981	0.119864	0.1532865
988.635	8.5	0.395432	0.3452608	0.1414353	0.09446763	0.121436	0.1528804
988.735	8.5	0.387305	0.3442461	0.1371669	0.09603202	0.116816	0.1508452
988.835	8.5	0.398427	0.3451967	0.1356668	0.09955616	0.117949	0.1505358
988.935	8.5	0.382655	0.3431453	0.1350298	0.09858447	0.120588	0.1519091
989.035	8.5	0.377352	0.3353836	0.1339284	0.1005674	0.12295	0.1538538
989.135	8.5	0.386579	0.3341467	0.1325136	0.09956063	0.124497	0.1498432
989.235	8.5	0.390849	0.3376317	0.1367147	0.0959031	0.12645	0.153085
989.335	8.5	0.395833	0.3335221	0.1371285	0.0963301	0.126449	0.1535737
989.435	8.5	0.383975	0.3192473	0.1339249	0.09721535	0.1216	0.1512914
989.535	8.5	0.389	0.3078797	0.1298965	0.09687107	0.122855	0.1494552
989.635	8.5	0.387504	0.3288307	0.1321811	0.09820504	0.123648	0.1492223
989.735	8.5	0.385422	0.3328436	0.1307921	0.102414	0.127348	0.1491452
989.835	8.5	0.393347	0.3301603	0.1332819	0.1019991	0.131553	0.1493603
989.935	8.5	0.385358	0.3337614	0.1346335	0.1020007	0.129941	0.1476034
990.035	8.5	0.402699	0.334225	0.1325171	0.1027661	0.129215	0.1485131
990.135	8.5	0.405556	0.3413676	0.1301812	0.09953102	0.129968	0.1483778
990.235	8.5	0.402676	0.3401158	0.1350053	0.1015971	0.12974	0.1472416
990.335	8.5	0.390688	0.3350938	0.1364221	0.104864	0.137369	0.1477358
990.435	8.5	0.380856	0.3350895	0.1340039	0.1039231	0.137318	0.1519506
990.535	8.5	0.395375	0.3370984	0.135127	0.1065273	0.135761	0.151917
990.635	8.5	0.384489	0.3413788	0.1401377	0.106255	0.134973	0.1547949
990.735	8.5	0.389256	0.3504227	0.1366484	0.1069185	0.134864	0.151193
990.835	8.5	0.37129	0.3485793	0.1373988	0.1086064	0.134095	0.1511763
990.935	8.5	0.396018	0.3540766	0.1371191	0.1088202	0.13221	0.1495585
991.035	8.5	0.388438	0.3439095	0.1369545	0.1096911	0.131384	0.1487468
991.135	8.5	0.39621	0.3408676	0.1385514	0.1100195	0.130452	0.1494066
991.235	8.5	0.386377	0.3401792	0.1355278	0.1108857	0.131912	0.1504007
991.335	8.5	0.3856	0.3425499	0.1387459	0.1096287	0.134283	0.1475056
991.435	8.5	0.39346	0.3432146	0.1385129	0.1122228	0.137261	0.1482156
991.535	8.5	0.386347	0.3464709	0.1384871	0.1096436	0.138239	0.1413909
991.635	8.5	0.37774	0.3448711	0.1382568	0.1101981	0.133121	0.1441783
991.735	8.5	0.394416	0.3454086	0.141101	0.1071395	0.132163	0.143347
991.835	8.5	0.399162	0.3466177	0.139733	0.1030535	0.132725	0.1428136
991.935	8.5	0.390969	0.3450592	0.1419134	0.1008757	0.134168	0.1462606
992.035	8.5	0.381652	0.3387161	0.1400301	0.102249	0.133962	0.1494669
992.135	8.5	0.395888	0.3373232	0.1403794	0.1055911	0.132223	0.1481242
992.235	8.5	0.396038	0.3378259	0.1394843	0.1081669	0.134689	0.1499219
992.335	8.5	0.39572	0.3349975	0.1380462	0.1055701	0.137134	0.1506879
992.435	8.5	0.3813	0.3181907	0.137465	0.1067924	0.132251	0.1533775
992.535	8.5	0.384661	0.3064962	0.1371469	0.1070774	0.13183	0.1501183
992.635	8.5	0.404778	0.3248932	0.1432743	0.107567	0.132011	0.1507571
992.735	8.5	0.404779	0.3256076	0.1415242	0.104641	0.13194	0.1498169
992.835	8.5	0.406039	0.3245963	0.141404	0.1034912	0.130452	0.1496824
992.935	8.5	0.404558	0.3273248	0.1418034	0.1017896	0.131251	0.1501378
993.035	8.5	0.407257	0.3330023	0.1433281	0.1046222	0.130915	0.1491242
993.135	8.5	0.391757	0.3393966	0.1452659	0.1039669	0.130327	0.1484899
993.235	8.5	0.39708	0.3389329	0.1454941	0.1018615	0.130458	0.1490274
993.335	8.5	0.398156	0.333948	0.1435653	0.1036405	0.130667	0.1528714
993.435	8.5	0.416756	0.3322873	0.1410786	0.1007165	0.128695	0.1543853
993.535	8.5	0.381753	0.3349696	0.1436266	0.09775257	0.128631	0.1514249
993.635	8.5	0.384125	0.3414018	0.1415677	0.1009712	0.126218	0.1503038
993.735	8.5	0.384529	0.349351	0.1425216	0.09895021	0.125941	0.1521177
993.835	8.5	0.401478	0.346397	0.1404488	0.09832958	0.123931	0.1536016
993.935	8.5	0.410465	0.3551487	0.1430184	0.0976266	0.12288	0.1541339
994.035	8.5	0.412118	0.3435028	0.1440151	0.1002878	0.12152	0.1552711
994.135	8.5	0.4148	0.3395046	0.1383586	0.09933369	0.12362	0.1547124
994.235	8.5	0.404182	0.3383359	0.1419951	0.1009401	0.124613	0.1545041
994.335	8.5	0.395853	0.3414946	0.1413141	0.1021428	0.123516	0.1537015
994.435	8.5	0.399486	0.3413525	0.1444096	0.0980913	0.126098	0.1564713
994.535	8.5	0.395353	0.3445326	0.1459669	0.09648696	0.124195	0.1538468
994.635	8.5	0.394383	0.3435342	0.1416405	0.1013456	0.123468	0.1502042
994.735	8.5	0.410101	0.3434208	0.1406126	0.1010174	0.122594	0.1529475
994.835	8.5	0.409638	0.3447907	0.1469716	0.1040268	0.123977	0.1525782
994.935	8.5	0.396568	0.3421883	0.1446127	0.1005641	0.126682	0.1514386
995.035	8.5	0.409952	0.3373508	0.1432443	0.1002487	0.126684	0.1502401
995.135	8.5	0.401548	0.3345358	0.1440768	0.1002473	0.125194	0.1548648

995.235	8.5	0.403612	0.3375109	0.1471614	0.1011952	0.125933	0.1491611
995.335	8.5	0.411129	0.337575	0.1455078	0.09987152	0.126832	0.1499187
995.435	8.5	0.387229	0.3091765	0.1465421	0.09754969	0.125708	0.1494401
995.535	8.5	0.381764	0.3057658	0.1469441	0.09810106	0.125026	0.1506062
995.635	8.5	0.382648	0.3222872	0.1442991	0.0982102	0.125003	0.1513036
995.735	8.5	0.386091	0.3248538	0.1406916	0.09641591	0.121616	0.1511771
995.835	8.5	0.382823	0.3224785	0.1389513	0.09579625	0.12664	0.1488631
995.935	8.5	0.386695	0.3272048	0.1411541	0.09697773	0.131116	0.1482625
996.035	8.5	0.380763	0.3303985	0.1373967	0.09571394	0.129332	0.149536
996.135	8.5	0.372927	0.3419828	0.1359792	0.09826242	0.131635	0.1528142
996.235	8.5	0.381302	0.3385368	0.136869	0.09639561	0.134548	0.1505522
996.335	8.5	0.390353	0.3333709	0.1379164	0.09600974	0.134332	0.1516137
996.435	8.5	0.378975	0.3307872	0.1402525	0.09944478	0.134093	0.1470206
996.535	8.5	0.37627	0.3353513	0.1401246	0.09883408	0.137348	0.1483435
996.635	8.5	0.387726	0.3400291	0.1389956	0.09511855	0.13711	0.1483174
996.735	8.5	0.379736	0.3489245	0.1383711	0.09843099	0.138942	0.149233
996.835	8.5	0.386361	0.3460417	0.1366115	0.1006972	0.140554	0.1408674
996.935	8.5	0.385334	0.3508025	0.1421479	0.1008096	0.140647	0.1469257
997.035	8.5	0.391768	0.3421565	0.1385952	0.1031468	0.141739	0.1529969
997.135	8.5	0.395082	0.338109	0.1390128	0.1036757	0.142686	0.1524324
997.235	8.5	0.402425	0.3372269	0.1392157	0.1025788	0.144457	0.1534818
997.335	8.5	0.395851	0.3414534	0.1358443	0.1017923	0.14323	0.1522958
997.435	8.5	0.385505	0.342028	0.1349627	0.1051979	0.143318	0.1494346
997.535	8.5	0.378475	0.3459144	0.1364339	0.1062255	0.140756	0.1492307
997.635	8.5	0.39598	0.3448111	0.1353141	0.1078651	0.140325	0.1489592
997.735	8.5	0.392623	0.3436214	0.1330279	0.1088832	0.142237	0.1478761
997.835	8.5	0.392081	0.3457505	0.1340747	0.1106601	0.142252	0.1484715
997.935	8.5	0.381643	0.3434962	0.1353727	0.1099053	0.144096	0.1508257
998.035	8.5	0.403403	0.3367623	0.1305111	0.1100531	0.142883	0.1468319
998.135	8.5	0.40041	0.3337651	0.1298784	0.1113945	0.139696	0.1452548
998.235	8.5	0.40016	0.3371773	0.1299384	0.1108655	0.142885	0.1465514
998.335	8.5	0.394368	0.3355394	0.1268132	0.1095481	0.143793	0.1465599
998.435	8.5	0.398779	0.3117052	0.1333835	0.1087486	0.144067	0.1454978
998.535	8.5	0.395378	0.3022202	0.1346325	0.1111718	0.141996	0.1450606
998.635	8.5	0.389243	0.3221225	0.1331457	0.1099244	0.140952	0.1498517
998.735	8.5	0.389572	0.3246558	0.1342911	0.1101439	0.135355	0.1449965
998.835	8.5	0.393862	0.3246553	0.1369904	0.1047423	0.13489	0.145964
998.935	8.5	0.395325	0.3296518	0.1353245	0.1042007	0.138774	0.1464916
999.035	8.5	0.38784	0.3316219	0.1352962	0.1021512	0.139487	0.1485178
999.135	8.5	0.387662	0.3409986	0.1329782	0.1048225	0.138124	0.1490624
999.235	8.5	0.396445	0.3393066	0.1376598	0.1052559	0.136458	0.1504654
999.335	8.5	0.394652	0.3326915	0.134801	0.105775	0.135141	0.1492115
999.435	8.5	0.393295	0.3295066	0.1352868	0.1042996	0.137314	0.1496557
999.535	8.5	0.399265	0.3331882	0.1367756	0.1038591	0.133395	0.1478421
999.635	8.5	0.38368	0.3392178	0.1391489	0.1073282	0.131719	0.149507
999.735	8.5	0.399803	0.3486971	0.1388297	0.1063265	0.132417	0.1505631
999.835	8.5	0.403236	0.3463938	0.1376071	0.1019697	0.130629	0.1491701
999.935	8.5	0.391456	0.3531865	0.1391452	0.1026561	0.132375	0.1458269
1000.035	8.5	0.414982	0.3440334	0.1392076	0.1043596	0.131413	0.146477
1000.135	8.5	0.399632	0.3384305	0.1354999	0.1042338	0.130573	0.1379059
1000.235	8.5	0.413767	0.3389635	0.1352621	0.1025426	0.127333	0.1423078
1000.335	8.5	0.398049	0.3416125	0.1385615	0.1044493	0.125955	0.1424727
1000.435	8.5	0.414371	0.3434695	0.1420277	0.1053392	0.126241	0.1432357
1000.535	8.5	0.405019	0.3466107	0.1430003	0.1014957	0.124077	0.1452872
1000.635	8.5	0.384883	0.3468936	0.1421224	0.1029667	0.125257	0.1460527
1000.735	8.5	0.375671	0.3454588	0.1399398	0.1018618	0.123962	0.146625
1000.835	8.5	0.384093	0.3465979	0.1380838	0.09849919	0.12256	0.1493482
1000.935	8.5	0.409257	0.3441262	0.140057	0.09889052	0.123208	0.1520661
1001.035	8.5	0.439771	0.3352006	0.1391677	0.09962951	0.120717	0.1509549
1001.135	8.5	0.426377	0.3312742	0.1384346	0.1005087	0.120408	0.1496003
1001.235	8.5	0.430819	0.3353669	0.1413641	0.09928863	0.122002	0.1480532
1001.335	8.5	0.418252	0.3317622	0.1389908	0.1022209	0.123263	0.1467588
1001.435	8.5	0.410705	0.3157326	0.1412728	0.1028202	0.123636	0.1465079
1001.535	8.5	0.407056	0.2986132	0.1408018	0.09784526	0.123193	0.1482057
1001.635	8.5	0.395132	0.3218046	0.1407893	0.09970696	0.121463	0.1450358
1001.735	8.5	0.388979	0.3239755	0.1455005	0.1007225	0.121251	0.1461477
1001.835	8.5	0.396062	0.3259333	0.1471604	0.09923159	0.122602	0.1477977
1001.935	8.5	0.391457	0.3286789	0.1443837	0.1016131	0.125273	0.1523072
1002.035	8.5	0.39815	0.3277307	0.143411	0.09932003	0.126873	0.1534458
1002.135	8.5	0.397818	0.3385232	0.1437533	0.1002422	0.12682	0.1505815
1002.235	8.5	0.407347	0.339077	0.1429473	0.1020015	0.124954	0.1520586
1002.335	8.5	0.402251	0.3323044	0.1421148	0.104115	0.124311	0.1550674
1002.435	8.5	0.408659	0.3295463	0.1408594	0.1003295	0.124095	0.1572637
1002.535	8.5	0.397527	0.3313765	0.1454571	0.1010406	0.122936	0.1558892
1002.635	8.5	0.39159	0.339049	0.1438067	0.1028661	0.125138	0.1558386
1002.735	8.5	0.392643	0.3477882	0.1412672	0.1004679	0.122492	0.1542815
1002.835	8.5	0.385381	0.3450497	0.1420721	0.09948008	0.123934	0.1530863
1002.935	8.5	0.409384	0.3542512	0.1432871	0.09880281	0.126709	0.1541979
1003.035	8.5	0.394264	0.3453943	0.1463033	0.09994432	0.128285	0.1580075
1003.135	8.5	0.382206	0.3362658	0.1447621	0.09785952	0.12671	0.1540544
1003.235	8.5	0.38478	0.3370639	0.1392641	0.09871265	0.128028	0.1532639
1003.335	8.5	0.402169	0.3397093	0.1421055	0.09308679	0.127491	0.154387
1003.435	8.5	0.399888	0.3423789	0.1435887	0.09322838	0.126389	0.1529018
1003.535	8.5	0.394654	0.34444	0.1422282	0.09578604	0.129193	0.1538831
1003.635	8.5	0.404017	0.3422733	0.1441188	0.09471428	0.131874	0.1539347
1003.735	8.5	0.402563	0.3444786	0.1440609	0.09573488	0.132628	0.1546862
1003.835	8.5	0.396764	0.3449096	0.1459513	0.09594796	0.132717	0.1477234
1003.935	8.5	0.404303	0.343791	0.1432569	0.09482004	0.134577	0.1509703
1004.035	8.5	0.392641	0.3347323	0.1454107	0.0957123	0.134088	0.1487136
1004.135	8.5	0.408152	0.3313445	0.1408844	0.0945261	0.134024	0.1490014
1004.235	8.5	0.404408	0.3344733	0.1404014	0.09673037	0.132471	0.1486117
1004.335	8.5	0.401475	0.3340355	0.1357094	0.09607312	0.133887	0.1463608
1004.435	8.5	0.393134	0.312051	0.1393694	0.09758145	0.137052	0.1440509
1004.535	8.5	0.381111	0.3000781	0.1391953	0.100553	0.139028	0.1441844
1004.635	8.5	0.391203	0.3192238	0.1370009	0.1015713	0.137639	0.1492558

1004.735	8.5	0.401011	0.3225115	0.1358646	0.1049822	0.134889	0.1513932
1004.835	8.5	0.398086	0.3238308	0.1350462	0.1017936	0.136216	0.1486062
1004.935	8.5	0.385844	0.3289087	0.1378265	0.1032248	0.133835	0.1474733
1005.035	8.5	0.394789	0.3273975	0.1426593	0.1034875	0.132916	0.1453656
1005.135	8.5	0.405037	0.3400212	0.1387577	0.107172	0.137522	0.147928
1005.235	8.5	0.413756	0.3377379	0.1380358	0.1079528	0.135017	0.1485453
1005.335	8.5	0.388864	0.3299471	0.1376124	0.1058789	0.136477	0.1459828
1005.435	8.5	0.400885	0.3271568	0.1378256	0.1070762	0.135662	0.1421269
1005.535	8.5	0.410669	0.3301127	0.1423586	0.1079309	0.131383	0.1469127
1005.635	8.5	0.39875	0.3401369	0.1405679	0.1089863	0.136285	0.1493341
1005.735	8.5	0.384543	0.3460706	0.140923	0.1092002	0.135901	0.1494627
1005.835	8.5	0.399759	0.344162	0.1417713	0.1092318	0.133304	0.1503793
1005.935	8.5	0.399494	0.3531954	0.1350156	0.1040849	0.134643	0.1515127
1006.035	8.5	0.39414	0.3421167	0.1376611	0.1041387	0.134871	0.1503115
1006.135	8.5	0.389752	0.3377751	0.1367837	0.1024676	0.135024	0.1533331
1006.235	8.5	0.396413	0.3369851	0.1336707	0.1048575	0.133159	0.153877
1006.335	8.5	0.400179	0.3395126	0.1342338	0.1046313	0.131849	0.1515367
1006.435	8.5	0.394202	0.3412122	0.1349704	0.1051749	0.134014	0.1491462
1006.535	8.5	0.389221	0.3427688	0.1340177	0.1051134	0.132509	0.1471124
1006.635	8.5	0.390612	0.3402135	0.1292449	0.1026904	0.131573	0.1439827
1006.735	8.5	0.390454	0.3421307	0.1313825	0.1028391	0.132265	0.1434977
1006.835	8.5	0.400974	0.3420125	0.1332278	0.09985552	0.13358	0.1431294
1006.935	8.5	0.407229	0.340263	0.1316552	0.1001873	0.130861	0.1444543
1007.035	8.5	0.395269	0.3334212	0.1376742	0.09928504	0.130848	0.1439445
1007.135	8.5	0.416034	0.3298855	0.1355163	0.1026711	0.129556	0.1461942
1007.235	8.5	0.388311	0.3329225	0.1340386	0.1028031	0.129837	0.1469118
1007.335	8.5	0.421412	0.3328648	0.1360084	0.09913986	0.125924	0.1439898
1007.435	8.5	0.400548	0.3100199	0.1369054	0.1004159	0.124918	0.144894
1007.535	8.5	0.420449	0.2981823	0.1335733	0.09882315	0.122017	0.1459941
1007.635	8.5	0.401091	0.3175963	0.1347062	0.09820053	0.124715	0.1474105
1007.735	8.5	0.402211	0.3208619	0.135285	0.1019516	0.12341	0.1498668
1007.835	8.5	0.400009	0.3185562	0.1372629	0.1000867	0.123853	0.1495352
1007.935	8.5	0.412797	0.3270517	0.1356125	0.1003018	0.122954	0.147919
1008.035	8.5	0.413299	0.3303051	0.1352639	0.09909529	0.122098	0.1480946
1008.135	8.5	0.42484	0.3377736	0.1337686	0.1009414	0.120813	0.1491196
1008.235	8.5	0.419738	0.3350972	0.1354442	0.09923796	0.12314	0.1491503
1008.335	8.5	0.404508	0.3286635	0.1331933	0.09844692	0.121064	0.1471714
1008.435	8.5	0.396305	0.3265862	0.1367194	0.1023497	0.119853	0.1466778
1008.535	8.5	0.40213	0.3321491	0.1387838	0.1002626	0.121568	0.147518
1008.635	8.5	0.419301	0.3443566	0.1414571	0.09644007	0.122062	0.1448583
1008.735	8.5	0.409989	0.3524325	0.1363962	0.09730212	0.120999	0.1422537
1008.835	8.5	0.412555	0.3482007	0.1389568	0.09996742	0.1208	0.1438054
1008.935	8.5	0.411743	0.3558759	0.1397158	0.100618	0.121185	0.1445289
1009.035	8.5	0.402943	0.3446771	0.1444168	0.09809119	0.1186	0.1464362
1009.135	8.5	0.408262	0.3415809	0.1426751	0.09938725	0.122635	0.146855
1009.235	8.5	0.408035	0.3415751	0.1400326	0.1014464	0.122811	0.148045
1009.335	8.5	0.407297	0.345247	0.1396027	0.1018513	0.121122	0.1480989
1009.435	8.5	0.403264	0.3486271	0.1416165	0.1024578	0.118031	0.1488191
1009.535	8.5	0.388998	0.3519481	0.1394876	0.09867054	0.119318	0.1495895
1009.635	8.5	0.385295	0.3473951	0.1371411	0.09944208	0.120879	0.1474233
1009.735	8.5	0.398669	0.3483326	0.1403098	0.09878474	0.121572	0.1492806
1009.835	8.5	0.399789	0.3472168	0.1410266	0.09751971	0.118244	0.1480543
1009.935	8.5	0.394352	0.3462764	0.1396062	0.09675469	0.120395	0.1486771
1010.035	8.5	0.425926	0.3390091	0.1411166	0.09664743	0.127831	0.1474024
1010.135	8.5	0.428713	0.3353114	0.1401913	0.09412405	0.130174	0.146651
1010.235	8.5	0.389614	0.3361285	0.1421729	0.09594873	0.130535	0.147806
1010.335	8.5	0.388398	0.3327416	0.1447243	0.09659702	0.128485	0.1479183
1010.435	8.5	0.39876	0.3114496	0.1444765	0.09575615	0.127555	0.151274
1010.535	8.5	0.387295	0.2997815	0.1422499	0.09600998	0.12613	0.1523527
1010.635	8.5	0.383693	0.3200626	0.1422168	0.09680022	0.128706	0.1535971
1010.735	8.5	0.393569	0.3239907	0.1404412	0.09424413	0.129098	0.151179
1010.835	8.5	0.392945	0.3145746	0.1404125	0.09686132	0.129739	0.1534468
1010.935	8.5	0.392161	0.3289964	0.1419225	0.1017203	0.131582	0.1560253
1011.035	8.5	0.392294	0.3273151	0.1431594	0.1010977	0.135512	0.1544515
1011.135	8.5	0.397728	0.3366584	0.1434753	0.1019136	0.138211	0.1535213
1011.235	8.5	0.409854	0.3355487	0.1386103	0.09954735	0.134731	0.1557906
1011.335	8.5	0.406429	0.328677	0.1381032	0.09774776	0.13521	0.1590696
1011.435	8.5	0.397095	0.3273218	0.1353032	0.09916876	0.135727	0.152911
1011.535	8.5	0.390403	0.3314214	0.139134	0.1023474	0.137832	0.1542731
1011.635	8.5	0.376323	0.3411936	0.1427793	0.1026172	0.137821	0.1557865
1011.735	8.5	0.406221	0.3491423	0.1394994	0.1032993	0.138786	0.1527724
1011.835	8.5	0.402467	0.3444563	0.1372036	0.1021874	0.139189	0.1531507
1011.935	8.5	0.397092	0.3487198	0.1413485	0.106586	0.136366	0.1537461
1012.035	8.5	0.370206	0.3442779	0.1415653	0.1079872	0.133007	0.1522676
1012.135	8.5	0.395298	0.3381069	0.1411942	0.1094423	0.134118	0.1543504
1012.235	8.5	0.389687	0.3376548	0.1417275	0.111173	0.132971	0.1541114
1012.335	8.5	0.390231	0.3392242	0.1451352	0.1116002	0.133031	0.1546874
1012.435	8.5	0.390755	0.3409953	0.1459343	0.1100146	0.133681	0.1521435
1012.535	8.5	0.395326	0.3425097	0.1449454	0.1096314	0.131702	0.1542047
1012.635	8.5	0.401137	0.3420239	0.144489	0.1129331	0.131312	0.1531052
1012.735	8.5	0.395484	0.343408	0.1413863	0.1112896	0.13225	0.1549502
1012.835	8.5	0.389669	0.3459692	0.1410104	0.1091065	0.130767	0.1557639
1012.935	8.5	0.40229	0.3446933	0.1385017	0.1036128	0.132922	0.1529858
1013.035	8.5	0.391871	0.3373119	0.1386127	0.1015088	0.135104	0.1505285
1013.135	8.5	0.394148	0.3342439	0.1364065	0.1006294	0.134664	0.1523857
1013.235	8.5	0.383452	0.3336506	0.1360312	0.09981386	0.133942	0.1562283
1013.335	8.5	0.403311	0.3298313	0.1367569	0.101748	0.132143	0.156982
1013.435	8.5	0.397996	0.3113159	0.1375254	0.1045458	0.132076	0.1544099
1013.535	8.5	0.398319	0.2941844	0.1395791	0.1059528	0.132228	0.154032
1013.635	8.5	0.394902	0.3154331	0.1410434	0.1059927	0.129069	0.1521312
1013.735	8.5	0.380127	0.3186866	0.1387993	0.106018	0.129974	0.1525448
1013.835	8.5	0.403047	0.3115866	0.1386051	0.1089683	0.130608	0.151981
1013.935	8.5	0.408355	0.3271347	0.1374231	0.1058962	0.131889	0.1486952
1014.035	8.5	0.400246	0.3229859	0.1390943	0.1050842	0.130448	0.1489065
1014.135	8.5	0.409869	0.3321142	0.1371792	0.105479	0.128275	0.1476098

1014.235	8.5	0.392824	0.3338154	0.1372373	0.1067667	0.129414	0.1483503
1014.335	8.5	0.394332	0.3270197	0.1360131	0.1052948	0.129893	0.1475349
1014.435	8.5	0.392954	0.3257633	0.136399	0.1060535	0.126808	0.1452503
1014.535	8.5	0.402777	0.3330435	0.1331638	0.1074115	0.128399	0.1458295
1014.635	8.5	0.402974	0.3407062	0.1369921	0.1040456	0.126412	0.1443841
1014.735	8.5	0.374707	0.3484805	0.1371504	0.1071671	0.127866	0.1465823
1014.835	8.5	0.375589	0.3447791	0.1346196	0.1085404	0.124811	0.146571
1014.935	8.5	0.372733	0.3472224	0.1347196	0.1033073	0.126748	0.1488301
1015.035	8.5	0.393319	0.3442155	0.1347337	0.1036178	0.127129	0.1484863
1015.135	8.5	0.425491	0.3369821	0.1327724	0.104017	0.122695	0.1442253
1015.235	8.5	0.429724	0.3392864	0.1280501	0.1032986	0.124331	0.1433387
1015.335	8.5	0.428477	0.3409778	0.1301437	0.09991465	0.128427	0.1439595
1015.435	8.5	0.425994	0.3432027	0.1323803	0.1022148	0.128637	0.1432593
1015.535	8.5	0.40948	0.3456023	0.134304	0.1052498	0.126893	0.1402911
1015.635	8.5	0.392658	0.3442628	0.1381099	0.09868354	0.127251	0.1419775
1015.735	8.5	0.397059	0.3459567	0.1348248	0.09679099	0.125375	0.1439999
1015.835	8.5	0.393177	0.3477952	0.1337117	0.09825296	0.124242	0.1438562
1015.935	8.5	0.393307	0.3447662	0.1379013	0.09723505	0.123101	0.1450371
1016.035	8.5	0.383646	0.3359084	0.1375237	0.09907971	0.125074	0.1447433
1016.135	8.5	0.394217	0.3339092	0.1345446	0.09765832	0.124603	0.1508155
1016.235	8.5	0.387448	0.3351402	0.1333276	0.09963872	0.125134	0.1510206
1016.335	8.5	0.409405	0.3306437	0.1348182	0.09973033	0.12301	0.1533228
1016.435	8.5	0.393287	0.3094913	0.1351652	0.1024404	0.122559	0.1475581
1016.535	8.5	0.400782	0.2963941	0.1364144	0.0999887	0.121225	0.149186
1016.635	8.5	0.388225	0.3155821	0.1348612	0.1030636	0.122643	0.1478357
1016.735	8.5	0.380557	0.3214068	0.1358374	0.1046671	0.125846	0.151501
1016.835	8.5	0.389349	0.3149995	0.1386347	0.1028876	0.123591	0.1486391
1016.935	8.5	0.385941	0.3294628	0.1376441	0.1010625	0.121709	0.145274
1017.035	8.5	0.386297	0.3268029	0.1408253	0.0984007	0.126183	0.1433
1017.135	8.5	0.396494	0.335156	0.1401458	0.09875	0.127492	0.1445981
1017.235	8.5	0.384908	0.3328843	0.1417684	0.09811999	0.126317	0.1403201
1017.335	8.5	0.398118	0.32545	0.1356339	0.09957016	0.126538	0.1414127
1017.435	8.5	0.438042	0.3241138	0.1387623	0.09488485	0.127322	0.1443808
1017.535	8.5	0.427537	0.3280913	0.1409316	0.09538332	0.127634	0.1443488
1017.635	8.5	0.418281	0.3385791	0.1431875	0.09933126	0.130531	0.148096
1017.735	8.5	0.417585	0.3457848	0.1405882	0.09970272	0.128516	0.1489055
1017.835	8.5	0.426241	0.3440084	0.1400982	0.09841166	0.130102	0.1485906
1017.935	8.5	0.421341	0.3513689	0.1405876	0.10115	0.131859	0.1510532
1018.035	8.5	0.426914	0.3442442	0.1424916	0.1045327	0.132706	0.1479244
1018.135	8.5	0.415944	0.3381274	0.1407159	0.1034054	0.133128	0.1482759
1018.235	8.5	0.429823	0.3366794	0.1385507	0.1018298	0.134241	0.1481162
1018.335	8.5	0.411617	0.337905	0.1421397	0.1024086	0.137465	0.1502063
1018.435	8.5	0.414199	0.341839	0.1407966	0.1022155	0.137822	0.150559
1018.535	8.5	0.418234	0.3443985	0.1404311	0.1020737	0.139292	0.1491411
1018.635	8.5	0.409961	0.3428902	0.1406315	0.1039827	0.141604	0.1508754
1018.735	8.5	0.391241	0.3434352	0.1426786	0.1052959	0.139756	0.1505094
1018.835	8.5	0.404042	0.3431071	0.1447309	0.106439	0.136819	0.1523131
1018.935	8.5	0.401553	0.3430652	0.1471944	0.1040489	0.135588	0.1514054
1019.035	8.5	0.402055	0.3374592	0.1466458	0.107084	0.129633	0.1525425
1019.135	8.5	0.396556	0.3329465	0.1440053	0.110747	0.131277	0.153567
1019.253	9	0.390512	0.3098123	0.1468349	0.1128344	0.133541	0.1540347
1019.353	9	0.395674	0.2964289	0.1443292	0.1122142	0.135869	0.1523786
1019.453	9	0.386816	0.3179784	0.1443241	0.1113229	0.135784	0.1524553
1019.553	9	0.381258	0.3164676	0.1446719	0.1138408	0.138338	0.1533202
1019.653	9	0.390753	0.3202848	0.1453496	0.1123516	0.137752	0.1543729
1019.753	9	0.387361	0.3225549	0.1430036	0.1096674	0.142756	0.1561808
1019.853	9	0.380874	0.3222595	0.138169	0.1057014	0.140544	0.1560413
1019.953	9	0.387862	0.3323196	0.1405309	0.1049692	0.137921	0.154102
1020.053	9	0.392039	0.3302925	0.1408093	0.1026887	0.138553	0.1524144
1020.153	9	0.38788	0.3295691	0.1437822	0.1039502	0.137135	0.152832
1020.253	9	0.389057	0.3290794	0.1464491	0.1061661	0.137111	0.1520652
1020.353	9	0.383419	0.3390324	0.1414258	0.1082279	0.135825	0.1497882
1020.453	9	0.398538	0.3487864	0.1430041	0.1049048	0.132571	0.1506693
1020.553	9	0.396843	0.353485	0.1422918	0.1036828	0.132865	0.1517024
1020.653	9	0.406618	0.3477753	0.1437109	0.1050203	0.131353	0.1526867
1020.753	9	0.407743	0.3533472	0.1481158	0.1026345	0.132714	0.1511692
1020.853	9	0.392597	0.3457714	0.1450477	0.1021713	0.133105	0.1549457
1020.953	9	0.387661	0.343611	0.144992	0.1044051	0.133474	0.1506331
1021.053	9	0.388751	0.3473236	0.1431554	0.104718	0.132783	0.1504522
1021.153	9	0.400507	0.3500564	0.1423687	0.1025917	0.131296	0.1508531
1021.253	9	0.396203	0.3539601	0.1374249	0.1048187	0.129072	0.1495977
1021.353	9	0.376179	0.3558615	0.1405382	0.1037231	0.130508	0.1502315
1021.453	9	0.369875	0.3535587	0.1398274	0.1042909	0.128186	0.1512627
1021.553	9	0.372302	0.3513266	0.1395904	0.1072307	0.125523	0.1475137
1021.653	9	0.391688	0.3481168	0.1396774	0.1039532	0.125956	0.1458869
1021.753	9	0.40792	0.3390296	0.1375137	0.1043123	0.129835	0.1491242
1021.853	9	0.418013	0.3323729	0.1408374	0.1031016	0.12585	0.152079
1021.953	9	0.406091	0.3362654	0.1388597	0.1059289	0.124658	0.1518684
1022.053	9	0.398791	0.3341158	0.1351466	0.1026167	0.126333	0.1521221
1022.153	9	0.401973	0.318887	0.1352496	0.1061339	0.127119	0.1480764
1022.253	9	0.38762	0.2917153	0.1352197	0.1078992	0.122952	0.1507789
1022.353	9	0.38951	0.3104097	0.1387248	0.1001373	0.123469	0.1495352
1022.453	9	0.38436	0.3158896	0.1359474	0.1000757	0.121591	0.1480989
1022.553	9	0.383437	0.3106585	0.1360323	0.1034442	0.121004	0.1412887
1022.653	9	0.390476	0.3191622	0.1392466	0.1020491	0.123966	0.1470282
1022.753	9	0.390313	0.3129868	0.1349687	0.1033482	0.124462	0.1493115
1022.853	9	0.398196	0.3248993	0.1372133	0.1018939	0.124111	0.1496951
1022.953	9	0.389835	0.3311915	0.1375718	0.1029887	0.121281	0.1509417
1023.053	9	0.396818	0.3253787	0.1355043	0.1036367	0.121053	0.1507549
1023.153	9	0.391216	0.3246472	0.1377923	0.1046317	0.122128	0.1470461
1023.253	9	0.41089	0.3306528	0.1380854	0.09964731	0.124339	0.1479512
1023.353	9	0.415605	0.3392841	0.1328832	0.09921494	0.122958	0.1490736
1023.453	9	0.399212	0.3493143	0.1296172	0.09938779	0.123392	0.1463649
1023.553	9	0.414538	0.3453648	0.1333095	0.09787259	0.124669	0.1493277
1023.653	9	0.401314	0.354563	0.129612	0.09674936	0.124908	0.1514203

1023.753	9	0.394161	0.3478147	0.135911	0.09997978	0.124037	0.1489499
1023.853	9	0.414311	0.3403941	0.1325598	0.09591007	0.123343	0.1466313
1023.953	9	0.422306	0.3394308	0.1325737	0.09986855	0.11989	0.1450891
1024.053	9	0.403917	0.3427863	0.1357032	0.09686242	0.119853	0.1439711
1024.153	9	0.397224	0.345857	0.1362508	0.09615829	0.119678	0.1448887
1024.253	9	0.405767	0.3475175	0.1322241	0.09804194	0.117527	0.1441418
1024.353	9	0.40099	0.3446934	0.1324178	0.1005975	0.117823	0.1456808
1024.453	9	0.405017	0.3480849	0.1355241	0.1023795	0.123155	0.1476875
1024.553	9	0.400699	0.3470008	0.1343708	0.1053652	0.125953	0.1495632
1024.653	9	0.405611	0.3390986	0.1352823	0.1045607	0.121117	0.1475571
1024.753	9	0.403965	0.3338822	0.1336756	0.1013713	0.125386	0.1488758
1024.853	9	0.411233	0.3366566	0.1368635	0.1007715	0.126608	0.1507803
1024.953	9	0.387185	0.3365611	0.1357425	0.1028939	0.127979	0.152409
1025.053	9	0.381897	0.3241993	0.1346202	0.1016129	0.126451	0.1491769
1025.153	9	0.406436	0.2965896	0.1371216	0.1019512	0.125259	0.1502093
1025.253	9	0.398748	0.3023445	0.1418748	0.1013383	0.127137	0.1505868
1025.353	9	0.398665	0.3185849	0.1379859	0.102415	0.128273	0.151108
1025.453	9	0.370524	0.3175427	0.1418322	0.1011507	0.13212	0.1489391
1025.553	9	0.396802	0.3270147	0.1408989	0.1045385	0.134385	0.1487558
1025.653	9	0.393526	0.3247611	0.1444804	0.1044379	0.135476	0.1484225
1025.753	9	0.390456	0.3286722	0.14162	0.1081356	0.136802	0.1499638
1025.853	9	0.385809	0.3363706	0.1418838	0.1099649	0.136028	0.1422998
1025.953	9	0.389842	0.3276705	0.1433773	0.1110445	0.135132	0.1430593
1026.053	9	0.39316	0.3251493	0.1452404	0.1092061	0.135181	0.1434995
1026.153	9	0.381254	0.3280985	0.1440828	0.1113631	0.131682	0.145878
1026.253	9	0.393844	0.3364516	0.1445802	0.1132525	0.136156	0.1479437
1026.353	9	0.398079	0.3507071	0.1469283	0.1117391	0.137751	0.1474777
1026.453	9	0.38795	0.3474538	0.1433992	0.1106328	0.136982	0.1499347
1026.553	9	0.386933	0.3528953	0.1434842	0.1083519	0.140314	0.1479785
1026.653	9	0.398955	0.3564129	0.1446835	0.1063257	0.140551	0.1476445
1026.753	9	0.39207	0.3455312	0.1457034	0.1066899	0.138982	0.1489733
1026.853	9	0.396689	0.3428483	0.1468805	0.105156	0.139147	0.1474371
1026.953	9	0.393682	0.3462996	0.1456663	0.1066975	0.136307	0.1492117
1027.053	9	0.384067	0.3515441	0.1436028	0.1080177	0.136975	0.1501839
1027.153	9	0.402556	0.3572231	0.1460203	0.1060463	0.137922	0.1479178
1027.253	9	0.400659	0.3599066	0.1449525	0.1066736	0.135417	0.1511195
1027.353	9	0.396004	0.3600284	0.1439743	0.1063545	0.134119	0.1513272
1027.453	9	0.406035	0.358166	0.1396136	0.1036217	0.132744	0.152096
1027.553	9	0.383371	0.3539867	0.1400609	0.1002794	0.129774	0.1503863
1027.653	9	0.387684	0.3500325	0.1376839	0.1029716	0.131117	0.1508529
1027.753	9	0.387397	0.3442569	0.132656	0.1057311	0.128788	0.1538474
1027.853	9	0.405252	0.3389625	0.1373872	0.1029206	0.128226	0.1529088
1027.953	9	0.385717	0.3377078	0.1388232	0.105209	0.130736	0.1542822
1028.053	9	0.375547	0.3035052	0.1422833	0.104194	0.131122	0.155205
1028.153	9	0.371982	0.3131054	0.1396977	0.1036828	0.132338	0.1550332
1028.253	9	0.375777	0.3271474	0.1354456	0.1061368	0.132935	0.1525316
1028.353	9	0.389787	0.3216237	0.1400405	0.1002076	0.134667	0.1548854
1028.453	9	0.40043	0.3312362	0.1400322	0.1005702	0.134416	0.155762
1028.553	9	0.407725	0.3317361	0.1403099	0.1032054	0.132228	0.1557262
1028.653	9	0.399847	0.3331772	0.1424075	0.1019194	0.133081	0.155507
1028.753	9	0.39796	0.3379462	0.1460934	0.09801978	0.13119	0.156824
1028.853	9	0.395301	0.3415986	0.1427296	0.1025326	0.132824	0.1565319
1028.953	9	0.393662	0.3428063	0.1419196	0.1038721	0.131271	0.1544019
1029.053	9	0.384293	0.3455758	0.1395766	0.09701965	0.129986	0.1535626
1029.153	9	0.38968	0.3538467	0.13952	0.09820281	0.127816	0.1517843
1029.253	9	0.384697	0.3622503	0.1360123	0.1009662	0.124861	0.152604
1029.353	9	0.397654	0.3631766	0.1374478	0.09819832	0.125133	0.1535212
1029.453	9	0.385827	0.3500592	0.1378684	0.09623599	0.127739	0.1573993
1029.553	9	0.39838	0.3522331	0.1371543	0.09622166	0.125807	0.150548
1029.653	9	0.420969	0.3509133	0.1359006	0.09841259	0.123127	0.1515986
1029.753	9	0.422742	0.3546135	0.1353376	0.09994993	0.124559	0.1488274
1029.853	9	0.408785	0.3623853	0.1384723	0.09660995	0.124725	0.1482362
1029.953	9	0.433731	0.3674138	0.1356858	0.09612047	0.126255	0.150263
1030.053	9	0.414905	0.3737346	0.1347483	0.09752256	0.125239	0.1486394
1030.153	9	0.406511	0.3776799	0.1354524	0.09654306	0.126127	0.1454153
1030.253	9	0.403983	0.3788084	0.1343656	0.09832282	0.126391	0.1457772
1030.353	9	0.395209	0.3788538	0.1364192	0.09548788	0.128499	0.1498378
1030.453	9	0.39432	0.3723967	0.1354348	0.09709043	0.128813	0.1538395
1030.553	9	0.393517	0.3605294	0.1376904	0.09512875	0.127803	0.1498132
1030.653	9	0.399687	0.3522255	0.1396747	0.09692255	0.126681	0.1514629
1030.753	9	0.391229	0.3484362	0.1362501	0.09598698	0.128826	0.147299
1030.853	9	0.400381	0.3372588	0.1384158	0.09377645	0.127409	0.1502369
1030.953	9	0.391029	0.3195915	0.1392159	0.09613253	0.125769	0.1496857
1031.053	9	0.380534	0.3116975	0.1346047	0.09811949	0.124858	0.1486858
1031.153	9	0.388091	0.3328474	0.1349484	0.0985534	0.126029	0.1447703
1031.253	9	0.382294	0.3268791	0.1340057	0.100092	0.128192	0.1493615
1031.353	9	0.395412	0.3222231	0.1301118	0.1017745	0.128752	0.1515724
1031.453	9	0.403505	0.3338627	0.1281705	0.1012608	0.127312	0.1504255
1031.553	9	0.399129	0.3348959	0.130592	0.0984863	0.126836	0.1512585
1031.653	9	0.382114	0.3413993	0.1264878	0.09918206	0.126796	0.1525915
1031.753	9	0.378802	0.3429065	0.1326837	0.101246	0.126248	0.150537
1031.853	9	0.391728	0.3425489	0.1303064	0.1032497	0.128455	0.1508001
1031.953	9	0.382566	0.342649	0.1312604	0.1050884	0.130759	0.151135
1032.053	9	0.391714	0.3476908	0.1341058	0.1083901	0.131378	0.1503354
1032.153	9	0.369661	0.3553582	0.1357856	0.1065764	0.132159	0.1519975
1032.253	9	0.388292	0.3591821	0.1329473	0.1060553	0.133301	0.151568
1032.353	9	0.395256	0.3461031	0.1326197	0.1043025	0.135172	0.147271
1032.453	9	0.386736	0.3447238	0.1353326	0.1063765	0.135497	0.1471566
1032.553	9	0.389927	0.3404278	0.135357	0.1070234	0.137302	0.1461834
1032.653	9	0.400069	0.338907	0.1360055	0.107404	0.138793	0.1453605
1032.753	9	0.390223	0.3406687	0.1346627	0.1057446	0.141732	0.1460286
1032.853	9	0.385367	0.3441741	0.1344327	0.1057457	0.140268	0.1454656
1032.953	9	0.394346	0.3473601	0.1353405	0.1055713	0.13864	0.1475856
1033.053	9	0.391215	0.3519742	0.1337008	0.1049149	0.13766	0.1459374
1033.153	9	0.379479	0.3510046	0.1339021	0.1051214	0.133866	0.1460146

1033.253	9	0.380396	0.3524267	0.1369495	0.1043703	0.135725	0.1468527
1033.353	9	0.395974	0.3489555	0.1358991	0.1023415	0.135004	0.1510731
1033.453	9	0.383467	0.3406213	0.1393515	0.1072587	0.135178	0.1542578
1033.553	9	0.393213	0.3348719	0.1359532	0.1047029	0.138398	0.153935
1033.653	9	0.391226	0.3314133	0.1388946	0.1037784	0.13857	0.1480467
1033.753	9	0.381028	0.3291895	0.1384227	0.1037044	0.13928	0.1454792
1033.853	9	0.39987	0.3285543	0.1378162	0.1013027	0.141169	0.1437397
1033.953	9	0.403669	0.3007762	0.1378941	0.1017851	0.140091	0.1469728
1034.053	9	0.39315	0.3269895	0.1406454	0.1001719	0.133963	0.147176
1034.153	9	0.405432	0.3244211	0.1412145	0.09891585	0.135645	0.1468184
1034.253	9	0.383349	0.3134735	0.143154	0.09763196	0.133263	0.1455706
1034.353	9	0.390254	0.331149	0.1449252	0.1008425	0.133761	0.1490027
1034.453	9	0.387689	0.3298045	0.1419357	0.1006466	0.133503	0.1435568
1034.553	9	0.40447	0.3385348	0.1422868	0.1007763	0.131951	0.1459416
1034.653	9	0.377521	0.3455695	0.1449595	0.1053602	0.129342	0.1447018
1034.753	9	0.375856	0.3369919	0.1461887	0.1039924	0.128031	0.1448703
1034.853	9	0.374614	0.3368579	0.1475461	0.1041154	0.125823	0.145722
1034.953	9	0.385129	0.3391719	0.1461327	0.1028055	0.12712	0.1467856
1035.053	9	0.396171	0.344563	0.141904	0.1022553	0.124663	0.1487842
1035.153	9	0.400823	0.3518448	0.1433899	0.1038299	0.125582	0.1492519
1035.253	9	0.403973	0.3457178	0.1427203	0.1022167	0.128512	0.1496134
1035.353	9	0.394685	0.3425385	0.1420083	0.09989373	0.127939	0.1496896
1035.453	9	0.395074	0.3387316	0.141257	0.1010575	0.130085	0.1501921
1035.553	9	0.380308	0.3369794	0.1445273	0.1031312	0.130186	0.1524777
1035.653	9	0.393664	0.3360824	0.144299	0.100377	0.126361	0.1513451
1035.753	9	0.384506	0.3401822	0.1420731	0.1000686	0.127498	0.1480132
1035.853	9	0.391475	0.341546	0.1433414	0.1011203	0.127118	0.1495356
1035.953	9	0.382996	0.3450355	0.1421058	0.09824368	0.128389	0.1472539
1036.053	9	0.393045	0.3426601	0.1440302	0.09637093	0.12635	0.1488403
1036.153	9	0.388034	0.3447099	0.1407895	0.09484459	0.124908	0.1495593
1036.253	9	0.399749	0.3436165	0.1401402	0.09585638	0.125621	0.150318
1036.353	9	0.389456	0.3385322	0.1452841	0.09859233	0.121067	0.1533218
1036.453	9	0.393286	0.3341145	0.1428409	0.1024674	0.122786	0.1481119
1036.553	9	0.385454	0.3291382	0.1425035	0.097125	0.121964	0.1494447
1036.653	9	0.398773	0.3302075	0.1430913	0.09733188	0.122056	0.1519953
1036.753	9	0.393811	0.3272581	0.1455563	0.09924022	0.12284	0.1537461
1036.853	9	0.395631	0.3051897	0.1455668	0.1027993	0.121924	0.1550841
1036.953	9	0.392192	0.3177686	0.1452003	0.09917494	0.123205	0.1570686
1037.053	9	0.381173	0.327691	0.1418169	0.09896542	0.119841	0.1602358
1037.153	9	0.397173	0.3165409	0.1411329	0.09834217	0.122489	0.1559906
1037.253	9	0.393179	0.3246871	0.138958	0.1020453	0.124578	0.1561522
1037.353	9	0.390579	0.3368588	0.1413091	0.09872685	0.123445	0.1550914
1037.453	9	0.382209	0.3398771	0.1393209	0.0963164	0.122845	0.1518754
1037.553	9	0.392559	0.3475994	0.1380445	0.09532119	0.122142	0.1534941
1037.653	9	0.388552	0.3424137	0.1360791	0.09655958	0.122357	0.1531722
1037.753	9	0.378639	0.3467031	0.1352714	0.09684658	0.124424	0.1524047
1037.853	9	0.382018	0.3471927	0.1376098	0.1000152	0.124064	0.1542892
1037.953	9	0.387961	0.3534604	0.1363174	0.09960041	0.124098	0.1553908
1038.053	9	0.389544	0.3629535	0.1363211	0.1005539	0.120738	0.1569674
1038.153	9	0.39379	0.355114	0.1368416	0.1016678	0.121756	0.1514787
1038.253	9	0.388707	0.3418663	0.1351768	0.103149	0.126416	0.1536303
1038.353	9	0.381656	0.3423029	0.13708	0.104048	0.124317	0.151605
1038.453	9	0.380025	0.3389984	0.1365686	0.106569	0.122491	0.1547027
1038.553	9	0.387375	0.3445378	0.138124	0.1051698	0.125555	0.155784
1038.653	9	0.384232	0.3495598	0.1388899	0.1066718	0.126956	0.1539465
1038.753	9	0.372755	0.3542175	0.1344128	0.102802	0.124357	0.1511969
1038.853	9	0.379319	0.3586653	0.1361796	0.105254	0.127672	0.1504254
1038.953	9	0.388081	0.3605081	0.1371333	0.1053241	0.126165	0.1533641
1039.053	9	0.390961	0.3591332	0.1343711	0.1058869	0.12953	0.1535781
1039.153	9	0.383952	0.3578663	0.1349262	0.1089932	0.134403	0.1487392
1039.253	9	0.379932	0.3517804	0.1355421	0.1066917	0.136004	0.1511552
1039.353	9	0.389778	0.3421706	0.1300211	0.1065438	0.137309	0.1498399
1039.453	9	0.386582	0.3375361	0.1302769	0.1069637	0.136389	0.1509856
1039.553	9	0.381218	0.3357518	0.1314899	0.1087557	0.135644	0.1499913
1039.653	9	0.390128	0.334459	0.1258469	0.1074772	0.137142	0.1482307
1039.753	9	0.3889	0.3150147	0.1314035	0.1074791	0.13633	0.1467837
1039.853	9	0.380597	0.3249121	0.1304138	0.1049607	0.133303	0.1492443
1039.953	9	0.390477	0.3304994	0.1303686	0.1055724	0.130883	0.1495561
1040.053	9	0.392673	0.3219946	0.1346614	0.1040594	0.131409	0.1476278
1040.153	9	0.386757	0.3207937	0.133837	0.104992	0.12985	0.1467508
1040.253	9	0.388174	0.3411831	0.1289307	0.105187	0.127168	0.1474301
1040.353	9	0.386043	0.343209	0.1313507	0.1058369	0.125527	0.1467229
1040.453	9	0.392224	0.3551811	0.1370789	0.1025849	0.127876	0.1513909
1040.553	9	0.394394	0.3511995	0.134492	0.1020552	0.129944	0.1501913
1040.653	9	0.400209	0.3506531	0.1339607	0.1036544	0.131891	0.145488
1040.753	9	0.397914	0.3528761	0.1333067	0.1010565	0.134345	0.1444825
1040.853	9	0.403365	0.3609672	0.1348934	0.100642	0.133397	0.1450629
1040.953	9	0.404717	0.3663054	0.1346652	0.102386	0.136173	0.144712
1041.053	9	0.409985	0.3681572	0.1335241	0.1053949	0.134463	0.1432031
1041.153	9	0.411765	0.3442014	0.1350401	0.1020137	0.133089	0.1463055
1041.253	9	0.408746	0.3468035	0.1373726	0.1032134	0.132625	0.1468541
1041.353	9	0.394455	0.3456315	0.1348661	0.1021815	0.132549	0.1473972
1041.453	9	0.392652	0.3458424	0.1358578	0.1004819	0.133414	0.1504228
1041.553	9	0.40314	0.3504139	0.1356509	0.1017972	0.131893	0.1521274
1041.653	9	0.413518	0.354522	0.1405067	0.09998502	0.128284	0.1556614
1041.753	9	0.43141	0.3601772	0.1364453	0.1004146	0.128009	0.1492527
1041.853	9	0.429896	0.3633368	0.1390249	0.09925491	0.125474	0.1490144
1041.953	9	0.416712	0.3624652	0.139817	0.1023081	0.123734	0.1486449
1042.053	9	0.398783	0.3639525	0.1404651	0.09954148	0.126275	0.1520573
1042.153	9	0.402651	0.3592552	0.140366	0.1002313	0.128115	0.1509605
1042.253	9	0.393639	0.3502937	0.1409463	0.1009148	0.127637	0.1495754
1042.353	9	0.389244	0.3419714	0.1409974	0.09784757	0.127335	0.1472415
1042.453	9	0.386696	0.3391607	0.1416997	0.09860574	0.126391	0.1482613
1042.553	9	0.392723	0.3308582	0.1454669	0.09869726	0.129758	0.1401017
1042.653	9	0.400029	0.316523	0.146142	0.09685651	0.127385	0.1433535

1042.753	9	0.39639	0.3034692	0.1475588	0.09904163	0.127476	0.1423605
1042.853	9	0.399418	0.3288992	0.1492933	0.09970522	0.127967	0.1417967
1042.953	9	0.401499	0.3170251	0.1454879	0.09858949	0.130701	0.1427965
1043.053	9	0.405711	0.3088496	0.1431113	0.1001567	0.127636	0.1453305
1043.153	9	0.394986	0.328736	0.1451461	0.0976786	0.126081	0.1472221
1043.253	9	0.395652	0.3387691	0.143271	0.09516315	0.127833	0.1475754
1043.353	9	0.400178	0.3431101	0.1431767	0.0967146	0.126392	0.1455879
1043.453	9	0.40962	0.3441804	0.1420339	0.09840608	0.125015	0.1456727
1043.553	9	0.429115	0.3392799	0.1446622	0.09860961	0.12649	0.1466967
1043.653	9	0.401347	0.3403691	0.1422537	0.09844665	0.124937	0.1483107
1043.753	9	0.398714	0.3450708	0.1423604	0.100085	0.122894	0.1477023
1043.853	9	0.411172	0.3526309	0.141559	0.09962835	0.126892	0.1516684
1043.953	9	0.436187	0.355112	0.1417763	0.1006885	0.126747	0.1484757
1044.053	9	0.407103	0.3445879	0.1438027	0.09528264	0.127124	0.1487553
1044.153	9	0.4008	0.3402305	0.1414447	0.09272697	0.126809	0.1498715
1044.253	9	0.402712	0.338414	0.1406516	0.09541445	0.125637	0.1518384
1044.353	9	0.397545	0.3386198	0.145086	0.09570027	0.123949	0.1553411
1044.453	9	0.399087	0.3399707	0.1420964	0.09709729	0.126616	0.1524034
1044.553	9	0.401545	0.3425041	0.1430789	0.09890024	0.12614	0.1541041
1044.653	9	0.413069	0.3459595	0.1438925	0.09971764	0.124718	0.1562963
1044.753	9	0.413328	0.3479463	0.1460291	0.09874367	0.124472	0.1577315
1044.853	9	0.410173	0.3454062	0.1460078	0.09701559	0.124606	0.1584448
1044.953	9	0.388514	0.3488879	0.1432594	0.09765159	0.124468	0.1565687
1045.053	9	0.384186	0.3461846	0.1404308	0.09866968	0.124336	0.1563521
1045.153	9	0.404674	0.3409101	0.1391481	0.102519	0.122259	0.1551938
1045.253	9	0.400445	0.3356237	0.1382562	0.1021329	0.123124	0.157291
1045.353	9	0.390827	0.3329366	0.1402333	0.1038998	0.125559	0.1553006
1045.453	9	0.378133	0.3312084	0.1377035	0.1026401	0.126374	0.1557014
1045.553	9	0.400681	0.3243183	0.1366587	0.1021799	0.128432	0.1540498
1045.653	9	0.40753	0.3020447	0.1357758	0.1026731	0.129976	0.1534903
1045.753	9	0.393131	0.3426817	0.136099	0.1074066	0.12945	0.1531139
1045.853	9	0.392413	0.3202693	0.1371762	0.109864	0.132125	0.1532329
1045.953	9	0.404402	0.3113593	0.135287	0.1086079	0.135421	0.1506048
1046.053	9	0.399531	0.3263945	0.1354912	0.1065739	0.136629	0.1498526
1046.153	9	0.381077	0.3377773	0.1348924	0.1078331	0.138998	0.1512519
1046.253	9	0.387045	0.3457407	0.1351413	0.1079984	0.137801	0.1503685
1046.353	9	0.390457	0.346139	0.1378703	0.1089413	0.137154	0.1511712
1046.453	9	0.385154	0.3426335	0.1354676	0.1068795	0.138072	0.1507008
1046.553	9	0.380571	0.3445088	0.1356885	0.1051092	0.138375	0.1505696
1046.653	9	0.394072	0.347668	0.1381605	0.1056278	0.139344	0.1492309
1046.753	9	0.381498	0.3542101	0.1328569	0.1048977	0.136467	0.1523283
1046.853	9	0.38903	0.3634684	0.1353504	0.1037924	0.134862	0.1522406
1046.953	9	0.384105	0.3509905	0.1342952	0.104785	0.136324	0.150009
1047.053	9	0.380177	0.3413492	0.1332307	0.1049492	0.136951	0.1490283
1047.153	9	0.407367	0.3415664	0.1335586	0.1009276	0.137371	0.1475931
1047.253	9	0.404258	0.3414473	0.1317116	0.102677	0.135257	0.1485169
1047.353	9	0.392871	0.3414341	0.1273089	0.1023926	0.133434	0.1476284
1047.453	9	0.407931	0.3465821	0.1274354	0.1019552	0.132846	0.1449752
1047.553	9	0.382628	0.3507655	0.1297781	0.09990447	0.132509	0.1464574
1047.653	9	0.393227	0.3545676	0.1258904	0.1000426	0.134538	0.1498763
1047.753	9	0.392688	0.3554673	0.1311607	0.1031359	0.133904	0.1484489
1047.853	9	0.407249	0.3561656	0.1295424	0.09993469	0.132171	0.1486814
1047.953	9	0.387338	0.3528936	0.1285937	0.1013511	0.131693	0.1494923
1048.053	9	0.374721	0.3472076	0.1304443	0.1002828	0.132353	0.1451791
1048.153	9	0.370807	0.3385142	0.1311817	0.09964313	0.129478	0.1487614
1048.253	9	0.380429	0.331622	0.1285438	0.1026219	0.128281	0.1484746
1048.353	9	0.390456	0.3324656	0.1290313	0.09851173	0.124198	0.1493329
1048.453	9	0.402458	0.328817	0.1350981	0.09984116	0.127526	0.1505701
1048.553	9	0.408306	0.3078219	0.1363244	0.09900606	0.129345	0.1489682
1048.653	9	0.395672	0.3259466	0.1352397	0.09920259	0.12872	0.1451899
1048.753	9	0.389595	0.3291889	0.1334498	0.09889917	0.129613	0.1441517
1048.853	9	0.389627	0.3134257	0.1344837	0.1027518	0.129272	0.1454117
1048.953	9	0.395227	0.3220133	0.1322244	0.1007248	0.12821	0.1459328
1049.053	9	0.38295	0.3427088	0.1306523	0.09642484	0.127062	0.1459245
1049.153	9	0.390305	0.3417741	0.1310954	0.09783166	0.127373	0.1477848
1049.253	9	0.385408	0.3463004	0.1336633	0.09834491	0.12728	0.1464464
1049.353	9	0.394226	0.3366773	0.1328312	0.09723365	0.127313	0.1459648
1049.453	9	0.386812	0.3337075	0.1360179	0.09679753	0.128038	0.1469989
1049.553	9	0.395655	0.3356732	0.1361686	0.09749749	0.129169	0.1499468
1049.653	9	0.39676	0.3406717	0.1386227	0.09753978	0.128152	0.1539594
1049.753	9	0.401375	0.349586	0.1359019	0.09933439	0.127437	0.14943
1049.853	9	0.398615	0.3483996	0.1363594	0.09990843	0.130798	0.148124
1049.953	9	0.405671	0.3403214	0.1371882	0.09700336	0.12928	0.1465501
1050.053	9	0.394842	0.3405322	0.1375618	0.09837392	0.130175	0.1480755
1050.153	9	0.396148	0.3361936	0.1383582	0.09878748	0.125255	0.1478335
1050.253	9	0.394573	0.3343753	0.1389801	0.0995758	0.122747	0.1465694
1050.353	9	0.383641	0.3378344	0.1395999	0.09869964	0.126691	0.1446042
1050.453	9	0.391754	0.3417929	0.1385852	0.09846495	0.12648	0.1449544
1050.553	9	0.392022	0.3417006	0.1401785	0.09891196	0.127113	0.1411522
1050.653	9	0.396453	0.343444	0.1418396	0.09798352	0.126641	0.1455472
1050.753	9	0.385428	0.3445493	0.1406876	0.09657364	0.123781	0.1449803
1050.853	9	0.385239	0.3438261	0.1423376	0.09442569	0.125255	0.1447321
1050.953	9	0.388185	0.3428863	0.1424557	0.09715041	0.122873	0.1455925
1051.053	9	0.380743	0.3368137	0.1389821	0.1003071	0.12312	0.146612
1051.153	9	0.382112	0.3277335	0.1391303	0.1030659	0.124259	0.1471493
1051.253	9	0.379457	0.3280763	0.1378779	0.1033686	0.125961	0.1464395
1051.353	9	0.395392	0.3296392	0.1368322	0.1066286	0.121045	0.145767
1051.453	9	0.396984	0.3192613	0.136202	0.1050726	0.119049	0.1428761
1051.553	9	0.393366	0.3140872	0.1391307	0.1046996	0.12129	0.1457779
1051.653	9	0.38147	0.3306476	0.1388604	0.104704	0.122647	0.1455692
1051.753	9	0.378983	0.3230489	0.1385175	0.1078655	0.11954	0.1431464
1051.853	9	0.392266	0.3176005	0.1373825	0.1072004	0.11726	0.1456975
1051.953	9	0.383179	0.3400072	0.1387627	0.1071971	0.122554	0.1462165
1052.053	9	0.385565	0.3472302	0.1418957	0.1100006	0.126423	0.1491027
1052.153	9	0.364607	0.3464924	0.1394036	0.1095512	0.126826	0.1506681

1052.253	9	0.383541	0.3396913	0.1372617	0.1097806	0.129978	0.1512321
1052.353	9	0.400561	0.3336484	0.1422593	0.1071935	0.131853	0.1525071
1052.453	9	0.382449	0.3322862	0.1392551	0.1099414	0.130078	0.1484759
1052.553	9	0.384096	0.3382172	0.1393858	0.1113379	0.136076	0.1525211
1052.653	9	0.396963	0.3452393	0.1412266	0.1120003	0.1371	0.1535552
1052.753	9	0.387886	0.3482832	0.143156	0.1084367	0.137121	0.1542285
1052.853	9	0.382289	0.3428182	0.1438738	0.109351	0.136558	0.1558233
1052.953	9	0.390109	0.3422514	0.1433523	0.1109698	0.13306	0.1583406
1053.053	9	0.385637	0.3360096	0.141685	0.1108248	0.13396	0.1560041
1053.153	9	0.378404	0.3332449	0.1389827	0.105419	0.133963	0.1548495
1053.253	9	0.38087	0.3355771	0.1341353	0.1056886	0.135626	0.1571921
1053.353	9	0.3973	0.3378514	0.1366789	0.1047106	0.133809	0.1531854
1053.453	9	0.383159	0.3403708	0.1349253	0.1048865	0.134181	0.1515334
1053.553	9	0.3903	0.3426186	0.134116	0.1020431	0.134738	0.1488864
1053.653	9	0.385283	0.3415518	0.1341352	0.1009816	0.13575	0.1478871
1053.753	9	0.379069	0.343294	0.132981	0.0993113	0.135584	0.1503607
1053.853	9	0.400399	0.341779	0.1356339	0.1004403	0.134107	0.1533291
1053.953	9	0.408323	0.3394829	0.1350136	0.1003293	0.132527	0.1503649
1054.053	9	0.392948	0.3337783	0.1360424	0.09725653	0.134831	0.1469715
1054.153	9	0.404861	0.3327735	0.1360095	0.09902456	0.135926	0.1483987
1054.253	9	0.384766	0.3302624	0.1339836	0.09853625	0.133375	0.1471081
1054.353	9	0.391317	0.3235086	0.1360128	0.1006819	0.130653	0.1494473
1054.453	9	0.387892	0.3041393	0.1361737	0.09961412	0.131344	0.1514044
1054.553	9	0.401115	0.3330935	0.1348265	0.09692477	0.130711	0.15011
1054.653	9	0.374024	0.3226128	0.1376282	0.09826273	0.130235	0.1473215
1054.753	9	0.368075	0.3143714	0.1340404	0.09705804	0.1283	0.1526472
1054.853	9	0.368756	0.3353932	0.1370347	0.09722737	0.126195	0.1525086
1054.953	9	0.382622	0.3426544	0.137053	0.09815156	0.129767	0.1479242
1055.053	9	0.394351	0.3443715	0.1348763	0.09672585	0.129354	0.1465988
1055.153	9	0.40217	0.3445551	0.1369294	0.09756959	0.128285	0.144985
1055.253	9	0.398121	0.3352251	0.135459	0.09856743	0.13036	0.1488241
1055.353	9	0.387449	0.3344032	0.1300002	0.09887548	0.127808	0.1485741
1055.453	9	0.39095	0.3370745	0.12929	0.09944206	0.128867	0.1446922
1055.553	9	0.389827	0.3435124	0.1307177	0.1028322	0.12963	0.1497944
1055.653	9	0.396369	0.3491071	0.1277017	0.1005768	0.129496	0.1517684
1055.753	9	0.376538	0.3440901	0.1329596	0.09611626	0.128649	0.1502321
1055.853	9	0.406851	0.3403113	0.1318253	0.09892397	0.128558	0.1481794
1055.953	9	0.390328	0.3363091	0.1303986	0.09760623	0.127765	0.1492076
1056.053	9	0.401704	0.3332358	0.1343965	0.09651789	0.127777	0.1471052
1056.153	9	0.398722	0.3336467	0.1348673	0.09447263	0.127238	0.1495216
1056.253	9	0.408907	0.336738	0.1298142	0.09537676	0.129295	0.1507945
1056.353	9	0.387638	0.3403245	0.1316911	0.09643018	0.127532	0.151316
1056.453	9	0.387411	0.3408277	0.1369435	0.09797117	0.126669	0.15071
1056.553	9	0.382653	0.3422289	0.136684	0.09375656	0.123493	0.1518008
1056.653	9	0.390019	0.3445778	0.1356458	0.09592876	0.122099	0.1466756
1056.753	9	0.388553	0.3433481	0.1344548	0.09751451	0.123436	0.1475574
1056.853	9	0.384862	0.3417159	0.1362336	0.09949429	0.124028	0.1473794
1056.953	9	0.383852	0.336313	0.1375189	0.101473	0.127236	0.1458005
1057.053	9	0.376958	0.330998	0.1344186	0.1014615	0.125152	0.143638
1057.153	9	0.391072	0.3318312	0.1358153	0.1013884	0.12328	0.1453266
1057.253	9	0.388741	0.3258026	0.1369325	0.1006412	0.125565	0.1471672
1057.353	9	0.391629	0.3076242	0.1367603	0.1003218	0.123666	0.1478936
1057.453	9	0.383399	0.3310018	0.1380074	0.1005154	0.125024	0.1496872
1057.553	9	0.383211	0.3253492	0.1365937	0.09754449	0.124263	0.1501871
1057.653	9	0.384109	0.3183568	0.1392297	0.09822348	0.12384	0.1519125
1057.753	9	0.378686	0.3253872	0.1353632	0.09811081	0.121372	0.1498244
1057.853	9	0.378929	0.3438406	0.1354944	0.1007976	0.121668	0.1500225
1057.953	9	0.385441	0.3384401	0.1375972	0.1021707	0.121951	0.1478224
1058.053	9	0.396588	0.3472908	0.1400259	0.1057095	0.12177	0.1507085
1058.153	9	0.395086	0.335941	0.1394343	0.1071237	0.122528	0.1493965
1058.253	9	0.395504	0.3337635	0.138774	0.1052228	0.122682	0.148969
1058.353	9	0.382671	0.3348641	0.1426276	0.1055065	0.124186	0.1474147
1058.453	9	0.380393	0.3397319	0.1401859	0.1071762	0.120398	0.1492841
1058.553	9	0.395094	0.3479567	0.1411879	0.1049972	0.121725	0.1420944
1058.653	9	0.388693	0.3460675	0.14172	0.1038709	0.125429	0.1433849
1058.753	9	0.388379	0.3461498	0.1433437	0.10214	0.126122	0.142465
1058.853	9	0.383595	0.3421181	0.1456745	0.106193	0.129054	0.1414329
1058.953	9	0.396715	0.3342281	0.1460101	0.103515	0.129892	0.1424837
1059.053	9	0.40683	0.3333054	0.1431912	0.102294	0.133616	0.1447573
1059.153	9	0.399755	0.3359728	0.1433588	0.1044883	0.130619	0.1490494
1059.253	9	0.394302	0.3383976	0.1418446	0.1029939	0.133108	0.1482884
1059.353	9	0.427109	0.3415419	0.141381	0.1040003	0.131924	0.1480166
1059.453	9	0.425539	0.3428387	0.1413306	0.1041364	0.131003	0.1466693
1059.553	9	0.397653	0.3440674	0.1440076	0.1078921	0.131869	0.1490717
1059.653	9	0.411216	0.3445668	0.1442064	0.1075022	0.134658	0.1470867
1059.753	9	0.404639	0.3419954	0.1408345	0.1092164	0.132928	0.1440805
1059.853	9	0.40524	0.3369257	0.1399992	0.1041893	0.12893	0.1453166
1059.953	9	0.427991	0.3312267	0.1398427	0.103303	0.131897	0.1468983
1060.053	9	0.41518	0.3304759	0.1421623	0.1018238	0.132062	0.147921
1060.153	9	0.403432	0.3295868	0.1401507	0.1034624	0.134895	0.1489992
1060.253	9	0.413009	0.3119702	0.1383383	0.1040291	0.135032	0.1491174
1060.353	9	0.409797	0.3274655	0.1408391	0.1024352	0.132104	0.1515598
1060.453	9	0.401303	0.3269398	0.1409161	0.09955233	0.134931	0.1510691
1060.553	9	0.410082	0.3217467	0.1420454	0.1005519	0.133209	0.1557447
1060.653	9	0.413721	0.3305306	0.1427852	0.101828	0.129716	0.155656
1060.753	9	0.396278	0.3419398	0.1453126	0.09835402	0.129752	0.1557795
1060.853	9	0.401779	0.3492629	0.1453626	0.09757846	0.131652	0.1571362
1060.953	9	0.387289	0.3560331	0.1450943	0.0982547	0.132895	0.159234
1061.053	9	0.396823	0.3468091	0.1434061	0.09918476	0.131941	0.1589654
1061.153	9	0.393011	0.3478137	0.1410259	0.09880845	0.134911	0.158599
1061.253	9	0.411048	0.3548642	0.1376856	0.09962522	0.133191	0.1601501
1061.353	9	0.386545	0.3600276	0.1384519	0.09717161	0.131293	0.1562896
1061.453	9	0.383524	0.367614	0.1365537	0.09728866	0.132176	0.1547997
1061.553	9	0.383779	0.3678873	0.1362518	0.1018223	0.133238	0.1524802
1061.653	9	0.401236	0.3466266	0.1350604	0.09884408	0.134475	0.1517291

1061.753	9	0.404721	0.3484457	0.1345124	0.0991939	0.131912	0.1529254
1061.853	9	0.40802	0.3501405	0.1368872	0.09956691	0.129812	0.1529054
1061.953	9	0.395071	0.3565965	0.1379297	0.1009423	0.132197	0.1500702
1062.053	9	0.390199	0.3632921	0.1363641	0.0976974	0.134436	0.1469633
1062.153	9	0.390509	0.368653	0.1365804	0.1000574	0.131685	0.1476986
1062.253	9	0.40019	0.3715899	0.1366477	0.101112	0.131474	0.1465007
1062.353	9	0.390812	0.3760481	0.1376784	0.09639956	0.129798	0.14797
1062.453	9	0.381052	0.3758526	0.1363524	0.09575741	0.129709	0.1488431
1062.553	9	0.381845	0.3764924	0.1360073	0.09848286	0.128853	0.1473227
1062.653	9	0.389374	0.3732929	0.138075	0.09589794	0.131054	0.1462387
1062.753	9	0.389898	0.359672	0.1331354	0.09558389	0.131189	0.1505817
1062.853	9	0.387288	0.3484565	0.1342523	0.09393948	0.128934	0.1534931
1062.953	9	0.394256	0.3445849	0.1339967	0.09713694	0.128281	0.1492018
1063.053	9	0.390612	0.3345139	0.1339358	0.1013876	0.129071	0.1505153
1063.153	9	0.385237	0.3203433	0.1360528	0.09890008	0.128324	0.1469723
1063.253	9	0.386172	0.3231996	0.1343009	0.09522468	0.12617	0.1491296
1063.353	9	0.38754	0.328979	0.1292361	0.09780157	0.123959	0.1476953
1063.453	9	0.381456	0.3263603	0.1266255	0.09853007	0.122163	0.1436389
1063.553	9	0.382981	0.3272466	0.1288352	0.09762498	0.120078	0.1492522
1063.653	9	0.376592	0.337102	0.1252411	0.09625258	0.12278	0.1513995
1063.753	9	0.378151	0.3504836	0.1343008	0.0974789	0.122633	0.1521328
1063.853	9	0.388104	0.3457715	0.1332668	0.0954451	0.121892	0.1496179
1063.953	9	0.399838	0.3413885	0.1324276	0.09741803	0.12175	0.1494438
1064.053	9	0.400242	0.3360889	0.1352322	0.09360148	0.122202	0.1472296
1064.153	9	0.381735	0.3369358	0.1369443	0.09339822	0.124691	0.1487272
1064.253	9	0.39037	0.3401931	0.1336774	0.09682535	0.124248	0.1490069
1064.353	9	0.377027	0.3463681	0.1334049	0.0966811	0.125096	0.1481179
1064.453	9	0.379901	0.3518976	0.1363931	0.09722228	0.125717	0.1500127
1064.553	9	0.374036	0.3452433	0.135185	0.09951837	0.125143	0.1490029
1064.653	9	0.387327	0.3420382	0.1352211	0.09975582	0.124105	0.1452095
1064.753	9	0.388282	0.337162	0.1347222	0.09959932	0.125523	0.1459241
1064.853	9	0.399974	0.3360732	0.1363807	0.0994411	0.126458	0.1461002
1064.953	9	0.39222	0.3381921	0.1363346	0.09818127	0.127674	0.1452758
1065.053	9	0.389845	0.3423985	0.1349006	0.09727169	0.126039	0.144983
1065.153	9	0.393295	0.3443984	0.1367732	0.09928656	0.120141	0.1480136
1065.253	9	0.395249	0.34648	0.1411307	0.1004177	0.120203	0.1467339
1065.353	9	0.40211	0.3464151	0.1377296	0.1025228	0.122868	0.1451465
1065.453	9	0.366893	0.348949	0.138006	0.1026831	0.124135	0.1452925
1065.553	9	0.385454	0.3495302	0.1394145	0.1045134	0.123755	0.1459131
1065.653	9	0.389858	0.3415347	0.1409798	0.105255	0.126427	0.1483112
1065.753	9	0.39696	0.3353109	0.13906	0.1055309	0.127785	0.1489599
1065.853	9	0.388993	0.3315889	0.1402891	0.107442	0.132693	0.1493635
1065.953	9	0.400421	0.3342047	0.1415955	0.1083563	0.132399	0.1507128
1066.053	9	0.396646	0.328308	0.1423472	0.1070239	0.130652	0.1497051
1066.153	9	0.381149	0.3150083	0.1420655	0.1047391	0.129863	0.1468019
1066.253	9	0.394029	0.3286219	0.140804	0.1071443	0.130181	0.1440844
1066.353	9	0.385725	0.3323171	0.1413593	0.1103808	0.135071	0.1426964
1066.453	9	0.376122	0.3180938	0.1400326	0.1092689	0.134638	0.145215
1066.553	9	0.395535	0.3439046	0.1413537	0.1059069	0.135425	0.1400451
1066.653	9	0.397337	0.3489558	0.143181	0.1043747	0.130799	0.1418792
1066.753	9	0.391143	0.3481472	0.1469553	0.1031435	0.131337	0.1409284
1066.853	9	0.39902	0.3440637	0.1480888	0.1046231	0.132532	0.1417638
1066.953	9	0.386137	0.3343509	0.1451614	0.1051357	0.132357	0.1415734
1067.053	9	0.385283	0.3343264	0.1418724	0.1031712	0.134824	0.1447124
1067.153	9	0.403104	0.3362786	0.141724	0.09923422	0.135472	0.1468106
1067.253	9	0.411335	0.3415594	0.1409096	0.1028597	0.131155	0.1464971
1067.353	9	0.419918	0.3493319	0.1408892	0.1013067	0.129401	0.1453619
1067.453	9	0.416151	0.3468204	0.1392439	0.1007687	0.133608	0.1438385
1067.553	9	0.396423	0.3428929	0.1424728	0.09784636	0.13031	0.1437209
1067.653	9	0.392883	0.3357968	0.1416541	0.1011616	0.131404	0.1437583
1067.753	9	0.392026	0.3337685	0.1409417	0.09924682	0.13082	0.1442988
1067.853	9	0.413033	0.3353927	0.1403066	0.09719825	0.132704	0.1466544
1067.953	9	0.396524	0.3388211	0.1396167	0.1003524	0.130431	0.1471593
1068.053	9	0.375782	0.3412617	0.144412	0.1001383	0.128306	0.148809
1068.153	9	0.381208	0.3424379	0.1427947	0.09900638	0.128991	0.1506855
1068.253	9	0.384894	0.3434177	0.140919	0.09922002	0.130282	0.1496295
1068.353	9	0.386243	0.34467	0.1421721	0.09855819	0.130765	0.1511718
1068.453	9	0.400968	0.3445247	0.1407389	0.09907562	0.132336	0.1464748
1068.553	9	0.40845	0.3433448	0.1417953	0.09854305	0.132807	0.1490728
1068.653	9	0.393655	0.3370556	0.143103	0.09855545	0.134073	0.149748
1068.753	9	0.392565	0.3337167	0.1468138	0.09994692	0.131817	0.1526734
1068.853	9	0.390708	0.33806	0.1493949	0.102584	0.130963	0.1551597
1068.953	9	0.407596	0.3335723	0.1481046	0.1003817	0.128455	0.1547443
1069.053	9	0.38978	0.3177629	0.1439754	0.09865291	0.12751	0.1536754
1069.153	9	0.390061	0.3261267	0.1410948	0.1011252	0.128352	0.1530807
1069.253	9	0.38223	0.3321156	0.1390704	0.09811953	0.126074	0.1540795
1069.353	9	0.394345	0.3236524	0.1422334	0.09871493	0.128345	0.1506822
1069.453	9	0.387886	0.3356015	0.1410942	0.09608974	0.126575	0.1511861
1069.553	9	0.390116	0.3468294	0.1407838	0.09976485	0.126683	0.1486593
1069.653	9	0.394876	0.3530549	0.1415118	0.09945427	0.1248	0.1476727
1069.753	9	0.423373	0.3443901	0.1405765	0.09992007	0.128001	0.148875
1069.853	9	0.41319	0.334694	0.1412644	0.09927057	0.12594	0.150194
1069.953	9	0.441403	0.3319539	0.1426264	0.09878752	0.124823	0.1498492
1070.053	9	0.428718	0.3326595	0.1417315	0.09934288	0.123871	0.1479076
1070.153	9	0.421587	0.3363964	0.1428843	0.09784582	0.11998	0.1466628
1070.253	9	0.405014	0.3461083	0.1401545	0.09958056	0.122618	0.1455941
1070.353	9	0.381835	0.3461091	0.1407484	0.09928993	0.125734	0.148095
1070.453	9	0.393943	0.3425235	0.1394437	0.09775925	0.125059	0.1496311
1070.553	9	0.393005	0.3371755	0.1394009	0.09957176	0.12558	0.1473015
1070.653	9	0.409174	0.3320848	0.141389	0.09990195	0.125656	0.1454914
1070.753	9	0.393027	0.3334612	0.1369492	0.09800367	0.123251	0.1479214
1070.853	9	0.405561	0.3374965	0.1372349	0.09842128	0.123016	0.1498441
1070.953	9	0.412893	0.3394597	0.1378926	0.09998626	0.122815	0.1459795
1071.053	9	0.394159	0.3415239	0.1362603	0.0993914	0.123632	0.1458146
1071.153	9	0.402432	0.3434699	0.1378981	0.1005702	0.12589	0.1451955

1071.253	9	0.400558	0.3437232	0.1354546	0.1019374	0.123323	0.147383
1071.353	9	0.415274	0.3451879	0.1319829	0.1028952	0.124604	0.1464552
1071.453	9	0.407993	0.3440738	0.1322353	0.102129	0.125701	0.1415429
1071.553	9	0.401709	0.3410126	0.1334908	0.1013545	0.125262	0.146469
1071.653	9	0.381312	0.3354135	0.1307251	0.1006772	0.123203	0.1498323
1071.753	9	0.390482	0.3363408	0.138374	0.1018579	0.11863	0.1498976
1071.853	9	0.407924	0.3343519	0.1344673	0.1017072	0.115337	0.1481021
1071.953	9	0.397558	0.317788	0.1351227	0.1017852	0.118396	0.1494132
1072.053	9	0.390741	0.3237879	0.1384277	0.1043889	0.119936	0.146921
1072.153	9	0.368154	0.3272074	0.1374364	0.1059141	0.122815	0.1498561
1072.253	9	0.383925	0.3193241	0.1337252	0.1060329	0.126233	0.1480951
1072.353	9	0.385322	0.3216745	0.1335143	0.1070886	0.125824	0.1465145
1072.453	9	0.382689	0.339491	0.1347043	0.1080333	0.126793	0.1471922
1072.553	9	0.382968	0.3537965	0.1353879	0.1072334	0.131661	0.148913
1072.653	9	0.391212	0.3409867	0.1358057	0.1090732	0.136121	0.1443467
1072.753	9	0.390133	0.3371216	0.1355661	0.1107599	0.134392	0.145967
1072.853	9	0.38877	0.329466	0.1375861	0.1106543	0.135436	0.1439458
1072.953	9	0.400182	0.330319	0.1388586	0.1094007	0.134657	0.1435342
1073.053	9	0.389333	0.3350585	0.1365961	0.1121	0.135966	0.1436234
1073.153	9	0.390536	0.3439416	0.1357333	0.1094067	0.136798	0.1460369
1073.253	9	0.388131	0.3485483	0.1374347	0.106631	0.136015	0.1479178
1073.353	9	0.403585	0.3422389	0.1337682	0.1052041	0.13629	0.1479963
1073.453	9	0.392221	0.3426559	0.1363125	0.1070167	0.135941	0.1450832
1073.553	9	0.39461	0.3334119	0.1354659	0.1082457	0.137227	0.1469068
1073.653	9	0.394131	0.3341995	0.1379234	0.1091072	0.135163	0.1481599
1073.753	9	0.381374	0.3375486	0.1362194	0.108036	0.137212	0.1476931
1073.853	9	0.397814	0.3390596	0.1387419	0.1058106	0.136322	0.1477155
1073.953	9	0.402626	0.3424437	0.141548	0.106762	0.135188	0.1476688
1074.053	9	0.387541	0.3438483	0.1420588	0.1047528	0.133396	0.1491159
1074.153	9	0.399922	0.3433888	0.1428202	0.1025798	0.132049	0.1469393
1074.253	9	0.375938	0.3459336	0.1423023	0.1016331	0.132514	0.1472082
1074.353	9	0.39132	0.3442179	0.1414149	0.1031408	0.135331	0.1486154
1074.453	9	0.390809	0.3387706	0.1382434	0.1032947	0.139831	0.1483694
1074.553	9	0.40225	0.3349273	0.1393156	0.1012721	0.139749	0.1423481
1074.653	9	0.375879	0.334904	0.1377649	0.1007739	0.135546	0.1442784
1074.753	9	0.369401	0.3343718	0.1400551	0.09932055	0.13557	0.1425736
1074.853	9	0.366811	0.3263499	0.1423124	0.09768443	0.134106	0.1417007
1074.953	9	0.378789	0.3229267	0.1431332	0.09753474	0.134848	0.1442648
1075.053	9	0.395385	0.3290302	0.1403127	0.09734667	0.133576	0.1468557
1075.153	9	0.404951	0.3317368	0.1426533	0.09622203	0.130822	0.1487174
1075.253	9	0.394303	0.3131986	0.1400981	0.09607698	0.133674	0.1486658
1075.353	9	0.389959	0.3340879	0.1421039	0.09485447	0.133178	0.1492229
1075.453	9	0.389241	0.3473646	0.1405517	0.09537009	0.133069	0.1495089
1075.553	9	0.378637	0.3443614	0.1419762	0.09965108	0.13273	0.1460074
1075.653	9	0.391518	0.339925	0.142089	0.09774972	0.133111	0.1438452
1075.753	9	0.378945	0.3291707	0.1432021	0.09803163	0.129258	0.1436548
1075.853	9	0.386414	0.3269341	0.1405145	0.099052	0.129837	0.1464276
1075.953	9	0.382264	0.3298805	0.138245	0.09659891	0.129843	0.1481511
1076.053	9	0.397964	0.3419852	0.1419621	0.09827458	0.128147	0.1488954
1076.153	9	0.381516	0.3469515	0.1406122	0.09676193	0.125005	0.1493033
1076.253	9	0.395292	0.3460215	0.1394148	0.09930288	0.128554	0.1480503
1076.353	9	0.387515	0.3435094	0.1411833	0.1016198	0.128675	0.1487616
1076.453	9	0.388816	0.3354789	0.1406743	0.1035776	0.1289	0.1481611
1076.553	9	0.384618	0.3370997	0.1399863	0.1001608	0.126911	0.1515729
1076.653	9	0.39639	0.3397835	0.1393398	0.1019164	0.125567	0.1532877
1076.753	9	0.392617	0.3441907	0.1422564	0.1034099	0.124389	0.1542872
1076.853	9	0.390074	0.3472736	0.1432152	0.1028365	0.121061	0.1543189
1076.953	9	0.38612	0.3507248	0.1441977	0.1016065	0.123514	0.1549385
1077.053	9	0.37999	0.3484818	0.1420235	0.1039216	0.12121	0.1536548
1077.153	9	0.391178	0.3521926	0.1415561	0.102648	0.118829	0.1554103
1077.253	9	0.398391	0.3513538	0.1380677	0.1003199	0.120045	0.1579689
1077.353	9	0.402867	0.341697	0.1391221	0.1006101	0.120659	0.1544468
1077.453	9	0.388509	0.340773	0.1386902	0.09877679	0.12238	0.1546994
1077.553	9	0.388076	0.3373001	0.1394494	0.09840304	0.119599	0.1544845
1077.653	9	0.389167	0.3389869	0.1382182	0.09643599	0.120941	0.1540584
1077.753	9	0.379929	0.3283692	0.1393491	0.0965822	0.122549	0.1558558
1077.853	9	0.3815	0.3226807	0.1397637	0.1017125	0.123091	0.156996
1077.953	9	0.39005	0.3249658	0.1385808	0.101269	0.121155	0.1546341
1078.053	9	0.399845	0.3231483	0.1369173	0.1015764	0.122823	0.1523024
1078.153	9	0.397567	0.3144112	0.1380666	0.09931906	0.126161	0.1514674
1078.253	9	0.393093	0.3332178	0.1381045	0.10039	0.1249	0.1511655
1078.353	9	0.381734	0.3388418	0.139945	0.1000606	0.124853	0.1489729
1078.453	9	0.386347	0.3429092	0.1380741	0.1028478	0.121861	0.1487629
1078.553	9	0.369889	0.3423688	0.1396635	0.1027894	0.12238	0.1469665
1078.653	9	0.377816	0.3335007	0.1418117	0.1036001	0.125829	0.1487046
1078.753	9	0.384428	0.3326834	0.13797	0.10616	0.127183	0.1527299
1078.853	9	0.387131	0.3356386	0.1373843	0.107486	0.12747	0.1557364
1078.953	9	0.382162	0.3420086	0.13603	0.1073758	0.127156	0.1535704
1079.053	9	0.390266	0.3519859	0.13531	0.1071889	0.125763	0.1535643
1079.237	9.5	0.386855	0.334701	0.1363457	0.1092603	0.12572	0.1513482
1079.337	9.5	0.383867	0.3345335	0.1352065	0.1069989	0.128046	0.1501997
1079.437	9.5	0.398317	0.3397109	0.1304488	0.1084228	0.129567	0.1491695
1079.537	9.5	0.396722	0.3448078	0.1284378	0.1010394	0.133085	0.14238
1079.637	9.5	0.394184	0.3460093	0.1312024	0.1001558	0.134643	0.1466466
1079.737	9.5	0.393545	0.3464311	0.1262258	0.1025573	0.133893	0.1506767
1079.837	9.5	0.399087	0.349444	0.132196	0.1039124	0.133947	0.148842
1079.937	9.5	0.397529	0.3484648	0.1295131	0.09986074	0.135173	0.1468781
1080.037	9.5	0.397569	0.3420766	0.131768	0.1007636	0.134292	0.1497768
1080.137	9.5	0.39434	0.3372358	0.1368212	0.1010018	0.13696	0.1479946
1080.237	9.5	0.391629	0.3357188	0.1354909	0.09971339	0.136325	0.1500109
1080.337	9.5	0.395495	0.335921	0.1308544	0.09943706	0.137491	0.1475786
1080.437	9.5	0.399679	0.3243619	0.1378945	0.09903857	0.136688	0.1477816
1080.537	9.5	0.396992	0.313383	0.1383947	0.09948663	0.136295	0.1485463
1080.637	9.5	0.389627	0.3215113	0.1385757	0.1018598	0.136453	0.1478463
1080.737	9.5	0.38056	0.3224195	0.1389647	0.09968756	0.134751	0.1455966

1080.837	9.5	0.388331	0.3175039	0.1377536	0.09776215	0.134514	0.1458154
1080.937	9.5	0.397429	0.3323023	0.1371475	0.0983087	0.137662	0.1455703
1081.037	9.5	0.383399	0.3449592	0.1390994	0.09801549	0.137621	0.1464613
1081.137	9.5	0.370236	0.3455698	0.1409942	0.09568964	0.137937	0.1480848
1081.237	9.5	0.366922	0.3456805	0.1414943	0.09816131	0.140134	0.1508874
1081.337	9.5	0.366466	0.3339013	0.1391725	0.09678918	0.139084	0.1499862
1081.437	9.5	0.402334	0.3305596	0.1393066	0.09835776	0.135495	0.1488955
1081.537	9.5	0.415146	0.3340457	0.1384147	0.09520315	0.137956	0.1481244
1081.637	9.5	0.399621	0.3432327	0.1401041	0.09480214	0.134316	0.1481253
1081.737	9.5	0.390347	0.3483182	0.1385367	0.09317563	0.131803	0.1501667
1081.837	9.5	0.388368	0.3435756	0.1403189	0.0945679	0.129516	0.1496486
1081.937	9.5	0.388057	0.3449416	0.1399244	0.09643967	0.130218	0.150121
1082.037	9.5	0.38623	0.3346851	0.1392454	0.09912407	0.135514	0.1483191
1082.137	9.5	0.384406	0.3336287	0.1373298	0.100153	0.133158	0.1517191
1082.237	9.5	0.386871	0.3367012	0.1383447	0.0969544	0.133299	0.1500213
1082.337	9.5	0.396683	0.3380225	0.138749	0.09804722	0.136281	0.147854
1082.437	9.5	0.388003	0.3399378	0.1341839	0.100035	0.135041	0.1480158
1082.537	9.5	0.388476	0.3412014	0.1339242	0.1038172	0.131565	0.1476171
1082.637	9.5	0.392889	0.3418726	0.1364586	0.1027269	0.128675	0.1423586
1082.737	9.5	0.391743	0.3434585	0.1376321	0.1039401	0.129075	0.1451909
1082.837	9.5	0.386805	0.3408635	0.1371611	0.1036069	0.128529	0.1443205
1082.937	9.5	0.394514	0.3360311	0.1399922	0.1029806	0.130402	0.1433612
1083.037	9.5	0.389459	0.3309658	0.139632	0.1001764	0.130882	0.1424453
1083.137	9.5	0.386136	0.3336479	0.1409591	0.1021755	0.131259	0.1442801
1083.237	9.5	0.384528	0.3319534	0.139531	0.1069641	0.129323	0.1475286
1083.337	9.5	0.382477	0.3137028	0.1390389	0.1080933	0.129573	0.1475215
1083.437	9.5	0.391438	0.3197311	0.1365526	0.1075016	0.12802	0.1468045
1083.537	9.5	0.386782	0.3219005	0.1374823	0.1080165	0.125889	0.1456992
1083.637	9.5	0.400024	0.3212017	0.1357993	0.1054069	0.125576	0.1453401
1083.737	9.5	0.393617	0.3216226	0.1346638	0.1086585	0.127476	0.1442281
1083.837	9.5	0.393316	0.3383692	0.1349133	0.1038211	0.125412	0.1443234
1083.937	9.5	0.390345	0.3472773	0.1340942	0.1035446	0.122335	0.1464327
1084.037	9.5	0.383978	0.3461972	0.1356385	0.1034352	0.123761	0.1479659
1084.137	9.5	0.381046	0.3408217	0.1345253	0.1049424	0.122356	0.1480257
1084.237	9.5	0.393527	0.3291531	0.1346197	0.1041306	0.121797	0.1505745
1084.337	9.5	0.393471	0.3277883	0.1366352	0.103531	0.12342	0.151851
1084.437	9.5	0.396916	0.3314894	0.1388442	0.1026021	0.126934	0.1512634
1084.537	9.5	0.382715	0.3446203	0.1367187	0.1024802	0.125618	0.1498514
1084.637	9.5	0.378265	0.3462068	0.1359058	0.1031458	0.12486	0.1508516
1084.737	9.5	0.392157	0.3453926	0.1364545	0.1001733	0.122874	0.1509368
1084.837	9.5	0.387991	0.3403463	0.1329905	0.09819083	0.123094	0.1530689
1084.937	9.5	0.387502	0.3327301	0.1357499	0.09834091	0.125185	0.1536536
1085.037	9.5	0.369479	0.3323269	0.1357931	0.09823198	0.126127	0.1544077
1085.137	9.5	0.386497	0.3351668	0.1365955	0.09773674	0.126857	0.1541049
1085.237	9.5	0.388132	0.3371489	0.1353696	0.09909382	0.124999	0.154155
1085.337	9.5	0.386025	0.3381569	0.1347743	0.09958499	0.123809	0.1573371
1085.437	9.5	0.388072	0.3402719	0.1270121	0.09981198	0.126941	0.1559406
1085.537	9.5	0.394347	0.3430481	0.1291335	0.09795893	0.127532	0.1562472
1085.637	9.5	0.390198	0.3417578	0.1286736	0.09821305	0.129762	0.153237
1085.737	9.5	0.387374	0.3380631	0.1317959	0.09774608	0.131311	0.1538415
1085.837	9.5	0.401573	0.3342629	0.1322425	0.1001584	0.132956	0.1566387
1085.937	9.5	0.38947	0.3318554	0.1299605	0.1006518	0.135048	0.155902
1086.037	9.5	0.399037	0.3367884	0.1332491	0.1029406	0.135666	0.1512662
1086.137	9.5	0.402311	0.3221803	0.1346146	0.1045124	0.133499	0.1495376
1086.237	9.5	0.403929	0.3105755	0.1295396	0.1009587	0.133963	0.14928
1086.337	9.5	0.407008	0.3204608	0.1304515	0.1001128	0.135357	0.1504173
1086.437	9.5	0.402877	0.3246323	0.1332251	0.09881072	0.135411	0.1514673
1086.537	9.5	0.39236	0.3151734	0.1332263	0.09978081	0.141166	0.1501508
1086.637	9.5	0.399555	0.3295486	0.1323425	0.1008104	0.14069	0.1478465
1086.737	9.5	0.402185	0.3410927	0.1311608	0.1029617	0.140298	0.1492337
1086.837	9.5	0.394786	0.350173	0.1327574	0.1030787	0.142757	0.1528964
1086.937	9.5	0.396503	0.3505518	0.1315481	0.1032127	0.139931	0.1523017
1087.037	9.5	0.381289	0.3315212	0.1323969	0.09877211	0.139492	0.1478779
1087.137	9.5	0.39776	0.329687	0.132516	0.09732381	0.14066	0.1476925
1087.237	9.5	0.42523	0.3334514	0.1352121	0.09918384	0.139515	0.1460094
1087.337	9.5	0.413694	0.3432875	0.1318711	0.1002247	0.137879	0.1490075
1087.437	9.5	0.39377	0.3491713	0.1331208	0.09915818	0.135604	0.1491412
1087.537	9.5	0.40833	0.347065	0.1325285	0.1007577	0.137528	0.1437159
1087.637	9.5	0.401264	0.3480639	0.1297684	0.09911799	0.139128	0.1466942
1087.737	9.5	0.423176	0.3409214	0.129212	0.1008466	0.136937	0.1497519
1087.837	9.5	0.455204	0.3374967	0.133456	0.09678478	0.136559	0.1504545
1087.937	9.5	0.427518	0.3413078	0.1367182	0.09555549	0.137873	0.1488422
1088.037	9.5	0.404652	0.3434353	0.1407525	0.09515988	0.135544	0.1486448
1088.137	9.5	0.403385	0.3460373	0.143834	0.09723001	0.133712	0.1454731
1088.237	9.5	0.413982	0.3487113	0.1409335	0.09823506	0.131223	0.1488557
1088.337	9.5	0.393095	0.3478876	0.1400929	0.1030788	0.13093	0.147972
1088.437	9.5	0.381457	0.3470453	0.1397259	0.1025751	0.130431	0.1484433
1088.537	9.5	0.39867	0.3444803	0.1393832	0.1009516	0.129883	0.1490554
1088.637	9.5	0.399016	0.3380414	0.1423084	0.09884082	0.129547	0.1506831
1088.737	9.5	0.391512	0.3339895	0.1431267	0.09890309	0.130561	0.146157
1088.837	9.5	0.386711	0.3359948	0.1414959	0.09812155	0.130476	0.1461093
1088.937	9.5	0.396872	0.3293867	0.1418678	0.09785059	0.132528	0.1439318
1089.037	9.5	0.42488	0.3103086	0.1407689	0.1033421	0.133684	0.1469666
1089.137	9.5	0.40026	0.3134423	0.1408754	0.1047567	0.130312	0.1466243
1089.237	9.5	0.418772	0.3165218	0.1402233	0.1061969	0.127326	0.1479632
1089.337	9.5	0.404673	0.3172324	0.1421829	0.1025453	0.125894	0.1477737
1089.437	9.5	0.396843	0.313032	0.1420326	0.1055036	0.126398	0.1485014
1089.537	9.5	0.392712	0.3264465	0.1422745	0.1070737	0.125426	0.1480718
1089.637	9.5	0.37793	0.3505329	0.138122	0.1076082	0.12585	0.148483
1089.737	9.5	0.392446	0.3460725	0.1384777	0.1076275	0.124361	0.152317
1089.837	9.5	0.394862	0.3428444	0.142619	0.1085426	0.124541	0.1500463
1089.937	9.5	0.399553	0.3287966	0.1381035	0.1071319	0.120183	0.148717
1090.037	9.5	0.386132	0.327817	0.1390592	0.1071558	0.120848	0.1464323
1090.137	9.5	0.396445	0.333895	0.1405818	0.1034475	0.123046	0.1472527
1090.237	9.5	0.393678	0.3459461	0.139074	0.1033096	0.119415	0.1481107

1090.337	9.5	0.388245	0.3481551	0.1400172	0.1029829	0.121474	0.1489266
1090.437	9.5	0.386535	0.3456225	0.1429612	0.1048277	0.124195	0.1478419
1090.537	9.5	0.397024	0.3462609	0.145726	0.1030627	0.12174	0.1486532
1090.637	9.5	0.395559	0.3368174	0.1451241	0.1018609	0.118337	0.1422842
1090.737	9.5	0.395855	0.336294	0.1436853	0.1014196	0.117405	0.1436199
1090.837	9.5	0.392736	0.3394067	0.1403036	0.1024636	0.11755	0.1436824
1090.937	9.5	0.386168	0.3419494	0.1382284	0.1047007	0.115395	0.1448453
1091.037	9.5	0.393519	0.3420551	0.1393661	0.1046152	0.115373	0.1443144
1091.137	9.5	0.388282	0.3424202	0.1374588	0.1023209	0.118776	0.1466093
1091.237	9.5	0.38572	0.3432087	0.1385131	0.1020337	0.122085	0.1462756
1091.337	9.5	0.373242	0.3434074	0.1388808	0.1016376	0.120791	0.1434352
1091.437	9.5	0.390679	0.3405229	0.1383317	0.1004609	0.120211	0.1454424
1091.537	9.5	0.390887	0.3346249	0.1391563	0.1019557	0.121229	0.1470734
1091.637	9.5	0.389908	0.3313263	0.1380998	0.1001494	0.122649	0.1480361
1091.737	9.5	0.396609	0.3338865	0.1362289	0.1025273	0.124246	0.1462134
1091.837	9.5	0.389659	0.3246184	0.1361996	0.101354	0.122535	0.1448742
1091.937	9.5	0.389394	0.3114892	0.138761	0.1003024	0.119431	0.1467008
1092.037	9.5	0.388369	0.3071953	0.1377932	0.09765666	0.120427	0.1481639
1092.137	9.5	0.407287	0.3170414	0.1370894	0.0995378	0.1232	0.1502406
1092.237	9.5	0.390724	0.311596	0.1387827	0.09777965	0.12344	0.1524544
1092.337	9.5	0.397686	0.317573	0.1331929	0.1008377	0.126048	0.1529184
1092.437	9.5	0.397406	0.3352961	0.1343187	0.1001734	0.124891	0.1534906
1092.537	9.5	0.392428	0.3513226	0.1339796	0.0969921	0.122347	0.1515223
1092.637	9.5	0.397794	0.3496208	0.1332189	0.09881606	0.12794	0.1535421
1092.737	9.5	0.388176	0.3376103	0.1350594	0.09806955	0.131204	0.1528704
1092.837	9.5	0.381809	0.3282048	0.1353199	0.0953522	0.131492	0.1537877
1092.937	9.5	0.398269	0.3289175	0.1280176	0.09511195	0.132902	0.1551661
1093.037	9.5	0.399369	0.3385199	0.133348	0.09727014	0.132921	0.1562423
1093.137	9.5	0.390833	0.3458959	0.1315049	0.09831795	0.135326	0.1553792
1093.237	9.5	0.397193	0.3459196	0.1321281	0.09882214	0.134228	0.1563535
1093.337	9.5	0.381152	0.3470451	0.1352599	0.09591754	0.134468	0.1577968
1093.437	9.5	0.392843	0.3413558	0.132039	0.09647577	0.137255	0.1560487
1093.537	9.5	0.400485	0.3359742	0.1349421	0.09718679	0.138412	0.1570042
1093.637	9.5	0.39588	0.3345705	0.1366162	0.09754097	0.135576	0.1548187
1093.737	9.5	0.383599	0.3391025	0.1323133	0.09603628	0.134127	0.1543649
1093.837	9.5	0.378728	0.3422579	0.1322003	0.09801109	0.137053	0.1555235
1093.937	9.5	0.37355	0.34264	0.1348083	0.09677561	0.137202	0.1578249
1094.037	9.5	0.387832	0.3441853	0.1359794	0.09877512	0.136493	0.1544421
1094.137	9.5	0.40483	0.3461331	0.1372445	0.09733991	0.136035	0.150384
1094.237	9.5	0.404167	0.3447163	0.136747	0.09547687	0.136585	0.1510624
1094.337	9.5	0.409483	0.3365734	0.1384022	0.09560905	0.133716	0.1473272
1094.437	9.5	0.3939	0.3326541	0.1372267	0.09492691	0.13458	0.1517545
1094.537	9.5	0.388816	0.3349212	0.1375834	0.09652752	0.135852	0.1527545
1094.637	9.5	0.391678	0.3293132	0.1371115	0.09953054	0.137772	0.1504526
1094.737	9.5	0.380418	0.3159147	0.1385107	0.1022685	0.133234	0.150619
1094.837	9.5	0.386002	0.3092232	0.1350627	0.1007656	0.132098	0.1522988
1094.937	9.5	0.386392	0.3211707	0.1354081	0.09994973	0.129304	0.1541615
1095.037	9.5	0.385876	0.3192707	0.1373552	0.1006187	0.130354	0.1527513
1095.137	9.5	0.383559	0.3083964	0.1349634	0.1035748	0.129714	0.1557082
1095.237	9.5	0.391559	0.3314684	0.1351831	0.1022262	0.127146	0.1524841
1095.337	9.5	0.394612	0.3431502	0.1356323	0.1018466	0.13047	0.1525515
1095.437	9.5	0.388644	0.3405305	0.1387599	0.1070399	0.126969	0.1506274
1095.537	9.5	0.39167	0.3403458	0.1411936	0.1108559	0.126298	0.1455987
1095.637	9.5	0.391275	0.3334972	0.141133	0.1089561	0.124794	0.1479945
1095.737	9.5	0.383737	0.3323805	0.1414763	0.1077063	0.123444	0.1509732
1095.837	9.5	0.383989	0.34008	0.1411356	0.1064621	0.12286	0.1525114
1095.937	9.5	0.377251	0.3502828	0.1404111	0.1067772	0.125113	0.1518665
1096.037	9.5	0.389693	0.3533421	0.1396701	0.1078977	0.12636	0.1503017
1096.137	9.5	0.385668	0.3403036	0.1418259	0.106936	0.127036	0.1463207
1096.237	9.5	0.421844	0.3457187	0.1433993	0.107139	0.124468	0.1484476
1096.337	9.5	0.442251	0.3379941	0.1404226	0.1087856	0.126052	0.148256
1096.437	9.5	0.453947	0.3397837	0.1412014	0.1044441	0.123274	0.1474427
1096.537	9.5	0.44828	0.3451073	0.1408604	0.10305	0.123299	0.147459
1096.637	9.5	0.445821	0.3510619	0.1413657	0.1035051	0.11993	0.1488988
1096.737	9.5	0.435855	0.3560953	0.1383219	0.1014218	0.120289	0.1446214
1096.837	9.5	0.44975	0.3583827	0.1396261	0.1012544	0.119116	0.1430604
1096.937	9.5	0.439143	0.3616081	0.138626	0.1019101	0.118436	0.1427452
1097.037	9.5	0.42833	0.3578219	0.1415797	0.1004463	0.121138	0.1431366
1097.137	9.5	0.405778	0.3564793	0.138024	0.09933475	0.120353	0.1431469
1097.237	9.5	0.404805	0.3491736	0.1398025	0.09975567	0.118191	0.1460146
1097.337	9.5	0.433334	0.3429394	0.1402569	0.09878874	0.119841	0.1466692
1097.437	9.5	0.427869	0.3413086	0.1364743	0.09765936	0.118915	0.1463578
1097.537	9.5	0.418413	0.3220549	0.1380256	0.100337	0.117155	0.1450553
1097.637	9.5	0.416933	0.3138483	0.1405309	0.1001298	0.116695	0.1465086
1097.737	9.5	0.420892	0.3113199	0.1382446	0.09755559	0.11865	0.1498657
1097.837	9.5	0.420661	0.3221725	0.1381041	0.09810171	0.118611	0.1471895
1097.937	9.5	0.403449	0.3117728	0.1419121	0.09834854	0.119615	0.1468829
1098.037	9.5	0.392135	0.3270874	0.141454	0.09820052	0.11934	0.1460824
1098.137	9.5	0.394699	0.3562917	0.1415427	0.0982748	0.120952	0.147036
1098.237	9.5	0.390629	0.3566232	0.1394541	0.09531062	0.121191	0.1454
1098.337	9.5	0.389287	0.3539778	0.1397302	0.09503619	0.121509	0.1437213
1098.437	9.5	0.411524	0.3502614	0.1382518	0.09613924	0.12146	0.1446476
1098.537	9.5	0.401302	0.3528363	0.140624	0.09404593	0.121591	0.1456725
1098.637	9.5	0.396186	0.3560465	0.1391295	0.0952258	0.122137	0.1390912
1098.737	9.5	0.397153	0.3633394	0.1361315	0.0971364	0.125303	0.1437604
1098.837	9.5	0.390787	0.371964	0.1375918	0.092973	0.127696	0.1436488
1098.937	9.5	0.398048	0.3590485	0.1360978	0.09295256	0.127822	0.1435282
1099.037	9.5	0.389844	0.3489305	0.1377277	0.09406957	0.128103	0.1449572
1099.137	9.5	0.379927	0.3492652	0.1372817	0.09367818	0.12772	0.1476893
1099.237	9.5	0.396549	0.3511908	0.1356195	0.09535854	0.129277	0.1495423
1099.337	9.5	0.39762	0.3545803	0.1349028	0.09361025	0.130605	0.1472495
1099.437	9.5	0.388753	0.3637348	0.1376017	0.09539067	0.130872	0.1455796
1099.537	9.5	0.40379	0.3683438	0.1363144	0.09593517	0.132925	0.1434771
1099.637	9.5	0.383399	0.3702609	0.1365834	0.09933809	0.134183	0.1452931
1099.737	9.5	0.405242	0.372513	0.1387345	0.09753834	0.136683	0.1433738

1099.837	9.5	0.400557	0.3713956	0.133554	0.09823744	0.137069	0.143966
1099.937	9.5	0.406114	0.3686287	0.134947	0.09861958	0.138855	0.1477335
1100.037	9.5	0.381151	0.3605663	0.1344314	0.09945987	0.138401	0.1473363
1100.137	9.5	0.396249	0.3527199	0.1349633	0.0980052	0.140231	0.1490354
1100.237	9.5	0.406091	0.3463248	0.1356945	0.09722348	0.137917	0.1501877
1100.337	9.5	0.408553	0.3323864	0.1344896	0.09797476	0.136734	0.1532706
1100.437	9.5	0.414225	0.3163413	0.1282866	0.09725685	0.139182	0.1527896
1100.537	9.5	0.403455	0.3076211	0.1307641	0.09666707	0.141763	0.1539175
1100.637	9.5	0.39911	0.3231188	0.1278025	0.09506787	0.138124	0.1549846
1100.737	9.5	0.396678	0.3247612	0.1315944	0.09588926	0.138334	0.1553113
1100.837	9.5	0.398048	0.3141367	0.133063	0.095643	0.136191	0.1591556
1100.937	9.5	0.41737	0.3375439	0.1292948	0.09835692	0.137028	0.1568266
1101.037	9.5	0.39884	0.3481046	0.1342444	0.0992837	0.134796	0.1578455
1101.137	9.5	0.40516	0.3481566	0.1338223	0.09940352	0.134246	0.158311
1101.237	9.5	0.406867	0.3414528	0.1297272	0.09631529	0.137037	0.152219
1101.337	9.5	0.40638	0.3393146	0.1309607	0.0976111	0.138261	0.1508998
1101.437	9.5	0.404664	0.3394808	0.1340115	0.09964968	0.134645	0.1483153
1101.537	9.5	0.411312	0.3444955	0.1348222	0.1025592	0.132488	0.1493165
1101.637	9.5	0.40022	0.3511246	0.1342855	0.1009741	0.130274	0.1497488
1101.737	9.5	0.389249	0.3538693	0.1335928	0.1022895	0.129201	0.1494696
1101.837	9.5	0.394594	0.3432756	0.1370185	0.1019082	0.127314	0.145967
1101.937	9.5	0.40422	0.3422348	0.1368173	0.1027607	0.129056	0.1454888
1102.037	9.5	0.390389	0.3355071	0.13767	0.1015853	0.130456	0.1466289
1102.137	9.5	0.40019	0.335056	0.1371425	0.1033637	0.129008	0.1482451
1102.237	9.5	0.385748	0.3382748	0.1372429	0.1042351	0.129286	0.1486095
1102.337	9.5	0.398185	0.3401631	0.1334074	0.1068799	0.131323	0.1465686
1102.437	9.5	0.392719	0.3431808	0.1314656	0.1075851	0.132383	0.1484223
1102.537	9.5	0.409243	0.3477245	0.1360064	0.1096983	0.134041	0.1518835
1102.637	9.5	0.391608	0.3462937	0.1340029	0.1083097	0.131397	0.1500326
1102.737	9.5	0.409478	0.34519	0.1360602	0.1080303	0.131473	0.1507676
1102.837	9.5	0.409824	0.3457695	0.1372595	0.1069131	0.131183	0.1501958
1102.937	9.5	0.401323	0.3385925	0.1384204	0.1047588	0.129165	0.1523185
1103.037	9.5	0.395616	0.3346463	0.1390786	0.1040139	0.128729	0.1514693
1103.137	9.5	0.412133	0.3353248	0.1393705	0.1031475	0.12799	0.1433724
1103.237	9.5	0.406097	0.3196754	0.1373334	0.10441	0.125543	0.1477027
1103.337	9.5	0.413837	0.309343	0.139676	0.106175	0.122467	0.1520318
1103.437	9.5	0.398251	0.314244	0.1397649	0.1017638	0.122672	0.1516877
1103.537	9.5	0.381875	0.322477	0.1392815	0.1020359	0.122486	0.1498926
1103.637	9.5	0.399521	0.3208697	0.1398652	0.100375	0.124028	0.1485809
1103.737	9.5	0.399117	0.3254003	0.1418637	0.1022564	0.126213	0.1441438
1103.837	9.5	0.389824	0.3447521	0.1407912	0.1036751	0.125326	0.1479953
1103.937	9.5	0.372968	0.3503434	0.1428506	0.1053584	0.123854	0.1471024
1104.037	9.5	0.385665	0.3475469	0.1422529	0.1048207	0.124545	0.1485617
1104.137	9.5	0.384707	0.3348666	0.1435477	0.1046577	0.126755	0.1506093
1104.237	9.5	0.376347	0.3359429	0.1422448	0.1006223	0.124583	0.145169
1104.337	9.5	0.380371	0.3366153	0.1431548	0.101157	0.12227	0.1424954
1104.437	9.5	0.383906	0.3414717	0.1403339	0.1037999	0.124348	0.1443688
1104.537	9.5	0.386482	0.3495446	0.1416682	0.1011188	0.125832	0.1450596
1104.637	9.5	0.386257	0.3469165	0.139762	0.1020136	0.125364	0.1453046
1104.737	9.5	0.403679	0.3452349	0.1392487	0.1022129	0.121648	0.1475181
1104.837	9.5	0.392957	0.3390087	0.1406876	0.1031298	0.121913	0.1486027
1104.937	9.5	0.398288	0.3326524	0.1373116	0.1041029	0.123424	0.1472397
1105.037	9.5	0.396881	0.3331895	0.1381355	0.1067601	0.125222	0.1476576
1105.137	9.5	0.388845	0.3368216	0.1390502	0.1034464	0.125919	0.148405
1105.237	9.5	0.395128	0.3386692	0.1365446	0.1000608	0.124286	0.1503891
1105.337	9.5	0.385132	0.3414774	0.1372592	0.1004249	0.123848	0.1472562
1105.437	9.5	0.382868	0.342665	0.1404435	0.09857254	0.125416	0.1459342
1105.537	9.5	0.395746	0.3429554	0.1403752	0.09996596	0.127227	0.1458655
1105.637	9.5	0.397826	0.3416772	0.1422294	0.09813754	0.127554	0.146458
1105.737	9.5	0.387435	0.3423031	0.1411556	0.1004551	0.131664	0.1457381
1105.837	9.5	0.403051	0.3360515	0.1393285	0.1002177	0.129384	0.1434581
1105.937	9.5	0.401718	0.3334575	0.1355185	0.1008061	0.130545	0.1433975
1106.037	9.5	0.42624	0.3312401	0.1396451	0.1006316	0.130453	0.1380977
1106.137	9.5	0.415795	0.3097569	0.1405435	0.09988626	0.128808	0.1404938
1106.237	9.5	0.444975	0.3125626	0.1397779	0.1009466	0.13145	0.1423617
1106.337	9.5	0.431838	0.3264084	0.1411515	0.1025969	0.133474	0.1443731
1106.437	9.5	0.401639	0.3265215	0.1389954	0.1013336	0.132626	0.1454292
1106.537	9.5	0.409387	0.3185602	0.1380098	0.1015753	0.130731	0.1452489
1106.637	9.5	0.416241	0.3348302	0.1378077	0.09929536	0.131354	0.146834
1106.737	9.5	0.432925	0.347331	0.1374692	0.09745196	0.134695	0.1449713
1106.837	9.5	0.425422	0.3533031	0.1369597	0.09436707	0.138508	0.1435836
1106.937	9.5	0.425286	0.3442368	0.14207	0.09477822	0.136238	0.1431694
1107.037	9.5	0.398956	0.3366674	0.1394341	0.09696227	0.139257	0.1419055
1107.137	9.5	0.394181	0.3334382	0.1393125	0.09873839	0.1415	0.1411996
1107.237	9.5	0.387356	0.3375263	0.1400065	0.1001458	0.139264	0.1441716
1107.337	9.5	0.384445	0.3420275	0.133803	0.1001192	0.138558	0.1448762
1107.437	9.5	0.383748	0.3469473	0.1359226	0.1021336	0.136804	0.1458915
1107.537	9.5	0.38978	0.3449539	0.1369066	0.1007	0.134921	0.1469898
1107.637	9.5	0.388075	0.349245	0.1371934	0.101116	0.137412	0.1478333
1107.737	9.5	0.379934	0.3371259	0.137174	0.1026583	0.137796	0.1505033
1107.837	9.5	0.386183	0.3341534	0.1356153	0.1049374	0.136683	0.1483643
1107.937	9.5	0.397644	0.3360349	0.1279556	0.1046276	0.140078	0.1503153
1108.037	9.5	0.405306	0.3388813	0.130277	0.1055321	0.136392	0.1507126
1108.137	9.5	0.418884	0.3406875	0.1281807	0.1060788	0.134505	0.1506524
1108.237	9.5	0.436851	0.3427522	0.1294579	0.1073749	0.132138	0.1545313
1108.337	9.5	0.440672	0.3411162	0.1314564	0.1052362	0.135809	0.1555016
1108.437	9.5	0.422733	0.3406377	0.1307779	0.1074426	0.134311	0.1519466
1108.537	9.5	0.400071	0.3405981	0.134865	0.1095501	0.134844	0.1523174
1108.637	9.5	0.402124	0.3384099	0.1338592	0.1094235	0.135723	0.1548393
1108.737	9.5	0.399377	0.333075	0.1301727	0.1074087	0.135905	0.1512649
1108.837	9.5	0.427103	0.33236	0.1316947	0.1074388	0.133195	0.1522515
1108.937	9.5	0.424088	0.3212916	0.1320848	0.1046216	0.133699	0.1520385
1109.037	9.5	0.435077	0.3057123	0.1333147	0.1037682	0.133415	0.1528648
1109.137	9.5	0.437304	0.3107287	0.1317908	0.1035252	0.132904	0.1525238
1109.237	9.5	0.444329	0.3275304	0.131759	0.102751	0.130177	0.1514791

1109.337	9.5	0.439833	0.3250367	0.1342448	0.1022869	0.130955	0.1500862
1109.437	9.5	0.44195	0.3233695	0.1332053	0.1042689	0.132905	0.1515318
1109.537	9.5	0.448311	0.3428815	0.1348645	0.1058594	0.128968	0.1515498
1109.637	9.5	0.430038	0.3510303	0.1351681	0.1026273	0.128881	0.1524994
1109.737	9.5	0.422097	0.3465723	0.1331236	0.09885464	0.125537	0.1496276
1109.837	9.5	0.399174	0.340207	0.1327446	0.09896023	0.126547	0.1469717
1109.937	9.5	0.426329	0.3357367	0.1326136	0.09979227	0.123127	0.148527
1110.037	9.5	0.445024	0.3346	0.1332067	0.1006502	0.124495	0.1502007
1110.137	9.5	0.445405	0.3420516	0.1303546	0.100081	0.123468	0.1468964
1110.237	9.5	0.420576	0.3469251	0.1332692	0.103015	0.124548	0.1497234
1110.337	9.5	0.438039	0.3485619	0.1357208	0.1021825	0.125962	0.1463873
1110.437	9.5	0.443977	0.3475513	0.1398097	0.1010782	0.128303	0.1483472
1110.537	9.5	0.426533	0.3458135	0.1376325	0.09895992	0.129574	0.1466815
1110.637	9.5	0.414485	0.3338014	0.1358081	0.0976949	0.126414	0.1430086
1110.737	9.5	0.421726	0.3353631	0.1391741	0.1000303	0.122607	0.1470009
1110.837	9.5	0.428103	0.3375595	0.1365815	0.09982032	0.124198	0.1494191
1110.937	9.5	0.394444	0.3400205	0.1378794	0.09890773	0.12509	0.1485025
1111.037	9.5	0.401466	0.3433787	0.139051	0.1005887	0.124541	0.1468094
1111.137	9.5	0.388514	0.3428771	0.1410561	0.1012612	0.12639	0.1441095
1111.237	9.5	0.399139	0.3429137	0.1424137	0.09953844	0.126153	0.1421442
1111.337	9.5	0.393982	0.3444347	0.1390817	0.1027687	0.128036	0.1447005
1111.437	9.5	0.394366	0.3415939	0.1401264	0.1032197	0.128981	0.1442254
1111.537	9.5	0.394583	0.3365541	0.1410115	0.1013474	0.124507	0.1448546
1111.637	9.5	0.384555	0.3337577	0.1425603	0.1023641	0.12356	0.1474615
1111.737	9.5	0.385277	0.3301524	0.1424178	0.09966251	0.125057	0.1441798
1111.837	9.5	0.394416	0.3088528	0.1447887	0.09836888	0.125859	0.1439785
1111.937	9.5	0.40267	0.3104379	0.1409934	0.09668147	0.127943	0.1441505
1112.037	9.5	0.39774	0.3336904	0.1398285	0.09747086	0.127491	0.1425181
1112.137	9.5	0.413223	0.322936	0.1354415	0.09688446	0.125469	0.1401993
1112.237	9.5	0.384563	0.3257038	0.1352675	0.09844293	0.129773	0.142035
1112.337	9.5	0.38764	0.3389633	0.1365815	0.09887293	0.128781	0.1428453
1112.437	9.5	0.399841	0.3458871	0.1357468	0.09902602	0.127174	0.143608
1112.537	9.5	0.393373	0.3681144	0.1372402	0.1012985	0.128138	0.1447675
1112.637	9.5	0.385779	0.3553026	0.1379125	0.1020479	0.129944	0.1463928
1112.737	9.5	0.374646	0.352381	0.1357667	0.09971981	0.13081	0.1489021
1112.837	9.5	0.371808	0.355043	0.1355191	0.1006602	0.128934	0.1466009
1112.937	9.5	0.373777	0.3588871	0.1375309	0.1016925	0.130893	0.1463998
1113.037	9.5	0.391521	0.3657815	0.1387099	0.101068	0.135534	0.1467121
1113.137	9.5	0.397268	0.3735158	0.1402017	0.09793919	0.134089	0.1458621
1113.237	9.5	0.394882	0.3590031	0.1381343	0.09551965	0.134907	0.1438984
1113.337	9.5	0.397873	0.3556972	0.139356	0.09691563	0.137236	0.142432
1113.437	9.5	0.386755	0.3495036	0.1367159	0.09810242	0.134995	0.1448332
1113.537	9.5	0.383545	0.350039	0.1367303	0.100002	0.137183	0.1411597
1113.637	9.5	0.381972	0.3558962	0.137012	0.1034254	0.135748	0.1431623
1113.737	9.5	0.382207	0.3635278	0.1365995	0.1057249	0.135959	0.1437012
1113.837	9.5	0.380844	0.3695885	0.1368235	0.1003709	0.138577	0.1419657
1113.937	9.5	0.386775	0.3723218	0.1357249	0.09889908	0.139126	0.1417932
1114.037	9.5	0.38092	0.3749287	0.137882	0.1046557	0.140113	0.1438631
1114.137	9.5	0.38944	0.3733412	0.1377415	0.1075671	0.139937	0.1449077
1114.237	9.5	0.38535	0.3725066	0.1393766	0.1080478	0.135735	0.1417806
1114.337	9.5	0.393793	0.364522	0.1393059	0.1100001	0.135097	0.1462552
1114.437	9.5	0.384441	0.3563266	0.1424045	0.1069948	0.135591	0.1458173
1114.537	9.5	0.412966	0.3481849	0.1413882	0.1064522	0.136667	0.1436223
1114.637	9.5	0.395939	0.3381709	0.1406887	0.1063704	0.136902	0.1422681
1114.737	9.5	0.383987	0.3326482	0.1432503	0.1089253	0.135883	0.1442589
1114.837	9.5	0.380371	0.3387058	0.1375763	0.1104796	0.136729	0.1454593
1114.937	9.5	0.378604	0.3427134	0.1379252	0.1116305	0.133275	0.1470141
1115.037	9.5	0.38839	0.3254273	0.1384283	0.1108167	0.133096	0.1516303
1115.137	9.5	0.389565	0.3270869	0.137336	0.1094332	0.136195	0.1537078
1115.237	9.5	0.393435	0.3360885	0.1368423	0.1094876	0.132572	0.1517207
1115.337	9.5	0.395011	0.3503555	0.1349948	0.1073827	0.130461	0.1494974
1115.437	9.5	0.387179	0.3550964	0.1285749	0.1031635	0.12936	0.1527584
1115.537	9.5	0.391642	0.3450128	0.1301572	0.104747	0.129532	0.153818
1115.637	9.5	0.385692	0.3422266	0.1296288	0.1044172	0.128635	0.1536112
1115.737	9.5	0.377898	0.3421601	0.1300776	0.1053428	0.13039	0.1541811
1115.837	9.5	0.38405	0.3490848	0.131813	0.1074387	0.128517	0.1549825
1115.937	9.5	0.384426	0.3574825	0.1303511	0.1059896	0.127839	0.1545257
1116.037	9.5	0.386297	0.3597853	0.1345948	0.1001766	0.129085	0.1545341
1116.137	9.5	0.379569	0.3476095	0.1368158	0.1025718	0.130143	0.1524381
1116.237	9.5	0.381463	0.3500617	0.1327014	0.1012717	0.128993	0.150215
1116.337	9.5	0.382385	0.33895	0.1324306	0.1035486	0.125381	0.1476493
1116.437	9.5	0.374793	0.3416723	0.1349838	0.1019791	0.127479	0.1476475
1116.537	9.5	0.35699	0.3440415	0.1353484	0.09960205	0.125259	0.1516279
1116.637	9.5	0.369123	0.3466567	0.1337249	0.09976266	0.123811	0.1529604
1116.737	9.5	0.377971	0.3477406	0.133148	0.09936933	0.123446	0.1519142
1116.837	9.5	0.379025	0.3518831	0.1350307	0.09941104	0.12455	0.1505485
1116.937	9.5	0.378967	0.3522087	0.1344912	0.09800475	0.12341	0.1520685
1117.037	9.5	0.381399	0.3518001	0.1362552	0.1005531	0.123253	0.1520033
1117.137	9.5	0.380494	0.3506605	0.1356083	0.09900369	0.124252	0.1527244
1117.237	9.5	0.382174	0.3418899	0.1355803	0.1000784	0.124175	0.149968
1117.337	9.5	0.394062	0.3387222	0.1325133	0.09892849	0.123102	0.1480872
1117.437	9.5	0.397453	0.3351219	0.1346645	0.1007707	0.125344	0.1483012
1117.537	9.5	0.400938	0.3314002	0.1369277	0.0998803	0.125649	0.1485894
1117.637	9.5	0.395187	0.3365047	0.1341998	0.1045331	0.126127	0.1479481
1117.737	9.5	0.393046	0.3280855	0.1371354	0.1038333	0.128139	0.1501993
1117.837	9.5	0.392261	0.3279854	0.1373962	0.0988893	0.126595	0.1493637
1117.937	9.5	0.386516	0.3114093	0.1368877	0.1000023	0.126458	0.1503227
1118.037	9.5	0.385705	0.3179937	0.1396856	0.0982937	0.129211	0.1475772
1118.137	9.5	0.389754	0.3368503	0.139477	0.09711518	0.128464	0.1447989
1118.237	9.5	0.395153	0.3560123	0.139504	0.09595662	0.12993	0.1502616
1118.337	9.5	0.389257	0.351832	0.1375035	0.09805549	0.131751	0.1503303
1118.437	9.5	0.397389	0.3396446	0.1403799	0.1002026	0.132862	0.1522669
1118.537	9.5	0.393728	0.3380995	0.138582	0.1031222	0.135546	0.1512519
1118.637	9.5	0.39451	0.3427329	0.1418153	0.1000145	0.134877	0.1499066
1118.737	9.5	0.399368	0.3480451	0.1445402	0.0990326	0.135615	0.1486167

1118.837	9.5	0.396107	0.3566186	0.1427926	0.09830087	0.135956	0.1495668
1118.937	9.5	0.383496	0.350667	0.141654	0.09541512	0.138189	0.1496373
1119.037	9.5	0.371206	0.3540224	0.1417493	0.09579917	0.138478	0.1494946
1119.137	9.5	0.373536	0.3446853	0.1420027	0.1004053	0.138452	0.1492525
1119.237	9.5	0.375134	0.3401461	0.1412586	0.1003833	0.139206	0.1457233
1119.337	9.5	0.389877	0.3419721	0.1434199	0.1010245	0.139985	0.1467743
1119.437	9.5	0.410946	0.3476061	0.1416375	0.1003728	0.137304	0.144814
1119.537	9.5	0.405148	0.3491983	0.1404252	0.09990167	0.13689	0.1438291
1119.637	9.5	0.408055	0.3511815	0.1380317	0.1002719	0.137587	0.14354
1119.737	9.5	0.396756	0.3513339	0.1354577	0.1016468	0.139052	0.1467011
1119.837	9.5	0.403894	0.3531989	0.1382412	0.1036849	0.138537	0.1471728
1119.937	9.5	0.404129	0.3518775	0.136307	0.1054676	0.138404	0.1466818
1120.037	9.5	0.392104	0.3479675	0.1388549	0.105887	0.137941	0.1491413
1120.137	9.5	0.381755	0.3449	0.1411742	0.09994126	0.140562	0.1505954
1120.237	9.5	0.390428	0.3390335	0.1399571	0.09818073	0.139153	0.1522719
1120.337	9.5	0.380584	0.3346339	0.1406131	0.1021512	0.139749	0.1494079
1120.437	9.5	0.387967	0.332034	0.1436313	0.1057665	0.13804	0.1486939
1120.537	9.5	0.385041	0.3233987	0.1445793	0.104225	0.141277	0.1476312
1120.637	9.5	0.391518	0.3372863	0.1453474	0.1080296	0.139154	0.1480895
1120.737	9.5	0.383821	0.309444	0.1417786	0.1070664	0.136355	0.1474137
1120.837	9.5	0.396845	0.3093025	0.1395252	0.103437	0.137296	0.1440127
1120.937	9.5	0.396997	0.325431	0.1369545	0.1031412	0.132843	0.144578
1121.037	9.5	0.385289	0.3435482	0.1377364	0.103519	0.13155	0.1432074
1121.137	9.5	0.380852	0.3610538	0.1360859	0.103107	0.131922	0.1474858
1121.237	9.5	0.379206	0.346233	0.1360457	0.1037394	0.132014	0.1465033
1121.337	9.5	0.390514	0.3413845	0.1385428	0.1052611	0.132548	0.1467293
1121.437	9.5	0.390065	0.3375723	0.1373409	0.1038466	0.131075	0.1468048
1121.537	9.5	0.397522	0.340942	0.137663	0.1020134	0.129027	0.1452485
1121.637	9.5	0.38749	0.3493186	0.1377578	0.1056934	0.12873	0.1489874
1121.737	9.5	0.385504	0.3533108	0.1363944	0.1014816	0.127136	0.1474597
1121.837	9.5	0.397708	0.3458613	0.1361323	0.1004933	0.128878	0.1478822
1121.937	9.5	0.391287	0.354311	0.1405247	0.09968948	0.129216	0.1454737
1122.037	9.5	0.387637	0.3414601	0.1390054	0.0998975	0.128753	0.1445558
1122.137	9.5	0.393349	0.3407272	0.1370957	0.1007346	0.126092	0.1438801
1122.237	9.5	0.395791	0.3414431	0.1364523	0.09987005	0.12462	0.1448465
1122.337	9.5	0.392576	0.3436611	0.1318995	0.09431542	0.123895	0.1471433
1122.437	9.5	0.385905	0.3450072	0.134626	0.0988477	0.122614	0.1499115
1122.537	9.5	0.386571	0.3455019	0.1335404	0.09727482	0.119402	0.1501321
1122.637	9.5	0.390608	0.3458676	0.132286	0.09680893	0.120266	0.1542845
1122.737	9.5	0.387124	0.3454452	0.1330983	0.09522921	0.123833	0.154845
1122.837	9.5	0.373025	0.3451254	0.1333864	0.09849032	0.122393	0.1495456
1122.937	9.5	0.392024	0.3409851	0.1271027	0.09870876	0.117745	0.1518809
1123.037	9.5	0.399479	0.337115	0.1292277	0.09893554	0.119276	0.1557188
1123.137	9.5	0.400031	0.3383361	0.1268542	0.09821408	0.121438	0.1554347
1123.237	9.5	0.393297	0.3329915	0.1281459	0.09864397	0.122835	0.1566281
1123.337	9.5	0.399031	0.3290435	0.129669	0.1015496	0.123864	0.1550382
1123.437	9.5	0.396603	0.3276571	0.1309701	0.1020516	0.123017	0.1503439
1123.537	9.5	0.392837	0.327205	0.1346648	0.1042567	0.126217	0.151189
1123.637	9.5	0.397979	0.3090256	0.1374087	0.1016016	0.124656	0.1525555
1123.737	9.5	0.407775	0.313114	0.1341043	0.1024633	0.125846	0.1514114
1123.837	9.5	0.407125	0.3332089	0.1329537	0.1003474	0.12591	0.1525976
1123.937	9.5	0.398593	0.3515805	0.1341006	0.1016585	0.125313	0.1518825
1124.037	9.5	0.399082	0.3627364	0.1338543	0.1030761	0.126516	0.1503304
1124.137	9.5	0.396284	0.3427976	0.1322532	0.09715367	0.122494	0.150827
1124.237	9.5	0.391474	0.3377925	0.1310324	0.1005794	0.120606	0.1505809
1124.337	9.5	0.389062	0.3375663	0.1347765	0.1011929	0.126376	0.14893
1124.437	9.5	0.386565	0.3417759	0.1346067	0.09854052	0.127222	0.1488638
1124.537	9.5	0.397306	0.3507992	0.1354962	0.09574009	0.126434	0.149043
1124.637	9.5	0.394046	0.3504636	0.1342623	0.09658246	0.125761	0.1501
1124.737	9.5	0.394764	0.3553855	0.1343393	0.0955489	0.125562	0.1496451
1124.837	9.5	0.393714	0.3499608	0.132488	0.09894569	0.130315	0.1473611
1124.937	9.5	0.384786	0.3421043	0.1344793	0.0970037	0.132836	0.14519
1125.037	9.5	0.393751	0.3405447	0.1361894	0.09541241	0.135582	0.1468998
1125.137	9.5	0.392765	0.3412949	0.134267	0.09612041	0.133785	0.1447281
1125.237	9.5	0.386306	0.3418301	0.1357926	0.09522961	0.13747	0.1474182
1125.337	9.5	0.370788	0.3447458	0.1386753	0.09501687	0.14019	0.1462819
1125.437	9.5	0.373797	0.3453611	0.1391943	0.09729134	0.138143	0.1479346
1125.537	9.5	0.374659	0.3462197	0.1378119	0.09737843	0.138153	0.146075
1125.637	9.5	0.388	0.3450022	0.1429628	0.09941038	0.139872	0.140458
1125.737	9.5	0.397343	0.3420483	0.141998	0.098616	0.137451	0.1435593
1125.837	9.5	0.396064	0.3380719	0.1403075	0.09869934	0.13689	0.147892
1125.937	9.5	0.399464	0.3343316	0.1413127	0.09839889	0.13848	0.1502852
1126.037	9.5	0.390827	0.3346545	0.1429524	0.09781896	0.137682	0.1509251
1126.137	9.5	0.387594	0.3258139	0.1441227	0.0985689	0.135361	0.148747
1126.237	9.5	0.393662	0.3321359	0.145603	0.102884	0.133877	0.1459018
1126.337	9.5	0.380143	0.3192145	0.1434583	0.1031286	0.134275	0.1488626
1126.437	9.5	0.379993	0.3099269	0.1442119	0.1018401	0.136055	0.1496902
1126.537	9.5	0.387088	0.3084743	0.1412722	0.1026505	0.137137	0.1491298
1126.637	9.5	0.387023	0.3225013	0.1421701	0.104048	0.137032	0.1505161
1126.737	9.5	0.385044	0.3308969	0.1406928	0.1053149	0.136301	0.1483104
1126.837	9.5	0.390862	0.3497	0.1428815	0.1074899	0.135005	0.1446533
1126.937	9.5	0.398983	0.3504639	0.1388528	0.1094349	0.13443	0.1451994
1127.037	9.5	0.389925	0.3384945	0.1379998	0.1083227	0.129502	0.1446068
1127.137	9.5	0.405957	0.3366413	0.136245	0.1082309	0.132568	0.1423654
1127.237	9.5	0.40456	0.3422301	0.1383472	0.106957	0.13236	0.142592
1127.337	9.5	0.390741	0.3502924	0.1398906	0.1069471	0.127163	0.1439447
1127.437	9.5	0.388892	0.3550436	0.1365433	0.1073099	0.132038	0.1467266
1127.537	9.5	0.383097	0.3498919	0.1373578	0.1066711	0.128643	0.1463962
1127.637	9.5	0.39336	0.3506804	0.138836	0.1068908	0.128052	0.1478893
1127.737	9.5	0.387375	0.3424108	0.138233	0.1081944	0.127774	0.1488298
1127.837	9.5	0.401534	0.3411864	0.1376816	0.1061505	0.126295	0.1474614
1127.937	9.5	0.390635	0.3430602	0.1410656	0.1047932	0.12877	0.14897
1128.037	9.5	0.389125	0.3479959	0.1412246	0.09964605	0.128546	0.1491803
1128.137	9.5	0.395067	0.3526464	0.1416303	0.09866098	0.130149	0.1480357
1128.237	9.5	0.392306	0.3537135	0.1402608	0.1025901	0.129214	0.1467913

1128.337	9.5	0.390378	0.3551457	0.1393886	0.1043264	0.128524	0.1445799
1128.437	9.5	0.391556	0.3573497	0.1360362	0.1044661	0.130681	0.1463618
1128.537	9.5	0.393593	0.3560748	0.1377465	0.1047458	0.131617	0.1419614
1128.637	9.5	0.398743	0.346507	0.1381846	0.1026694	0.129508	0.1431528
1128.737	9.5	0.386522	0.3396989	0.1382272	0.1003456	0.128201	0.1430534
1128.837	9.5	0.385138	0.3339238	0.1373575	0.1007398	0.124631	0.1425822
1128.937	9.5	0.389336	0.3301223	0.133412	0.0983112	0.123487	0.1439199
1129.037	9.5	0.387101	0.3245196	0.1353895	0.09805416	0.126143	0.1436713
1129.137	9.5	0.388003	0.3216062	0.1361239	0.09831554	0.12487	0.1438999
1129.237	9.5	0.382359	0.3243572	0.1362839	0.09843858	0.123374	0.144772
1129.337	9.5	0.398373	0.3028695	0.1366243	0.09844851	0.123732	0.1458526
1129.437	9.5	0.397455	0.3100712	0.1426512	0.09893672	0.122873	0.1435572
1129.537	9.5	0.391906	0.3188301	0.1403018	0.1004032	0.120043	0.1419642
1129.637	9.5	0.398216	0.3320747	0.1404029	0.09976114	0.120431	0.1428051
1129.737	9.5	0.401499	0.3538595	0.1390916	0.09960208	0.119532	0.1455386
1129.837	9.5	0.403061	0.3453748	0.1352852	0.09887283	0.120636	0.1453601
1129.937	9.5	0.397351	0.3384728	0.1368254	0.1009768	0.118695	0.1461655
1130.037	9.5	0.414938	0.3361891	0.137681	0.1030528	0.121102	0.1459489
1130.137	9.5	0.403341	0.3450781	0.1377268	0.09998938	0.121203	0.1488847
1130.237	9.5	0.406303	0.3536132	0.1377341	0.100242	0.12141	0.1511208
1130.337	9.5	0.39656	0.3548981	0.1364477	0.1008293	0.125621	0.1489412
1130.437	9.5	0.392376	0.3476099	0.1304093	0.09782966	0.126056	0.1500361
1130.537	9.5	0.393482	0.348565	0.1314363	0.09464651	0.126165	0.1505072
1130.637	9.5	0.38869	0.3396528	0.1325774	0.09452726	0.126213	0.1503298
1130.737	9.5	0.382758	0.3407152	0.1358642	0.09411974	0.126299	0.152
1130.837	9.5	0.395945	0.344495	0.1350331	0.09353539	0.124717	0.1528515
1130.937	9.5	0.396389	0.348625	0.1318657	0.09183532	0.124756	0.1499705
1131.037	9.5	0.3884	0.351826	0.1357193	0.09343509	0.125681	0.1504627
1131.137	9.5	0.396205	0.3494341	0.1376032	0.09492622	0.128119	0.1505042
1131.237	9.5	0.386522	0.3509623	0.1351005	0.09406023	0.130131	0.1457793
1131.337	9.5	0.391398	0.3513855	0.1364627	0.09427455	0.132083	0.1460071
1131.437	9.5	0.388914	0.3505507	0.1378328	0.09480078	0.132214	0.1455006
1131.537	9.5	0.386327	0.340115	0.1366457	0.09424229	0.1302	0.1468807
1131.637	9.5	0.369578	0.3328665	0.1354909	0.0910482	0.131391	0.1443683
1131.737	9.5	0.36706	0.3323028	0.1360666	0.09342062	0.134007	0.1467211
1131.837	9.5	0.373597	0.3278714	0.1377261	0.09274691	0.130159	0.1446328
1131.937	9.5	0.387149	0.3237578	0.1356238	0.09312142	0.134808	0.1455723
1132.037	9.5	0.396721	0.3289653	0.1351409	0.09076038	0.134175	0.1461402
1132.137	9.5	0.399712	0.3089883	0.1352902	0.09180052	0.135285	0.1486864
1132.237	9.5	0.397907	0.3061347	0.1364418	0.09321719	0.134673	0.1470536
1132.337	9.5	0.392948	0.3172986	0.1342454	0.09249268	0.133111	0.1445239
1132.437	9.5	0.386306	0.3227183	0.1345873	0.09286687	0.133235	0.1444756
1132.537	9.5	0.397251	0.3585359	0.1368852	0.09632146	0.132819	0.1486183
1132.637	9.5	0.386011	0.356015	0.1358106	0.0989446	0.134732	0.1448238
1132.737	9.5	0.381407	0.3359008	0.1353301	0.1007727	0.136281	0.1468843
1132.837	9.5	0.383265	0.332183	0.1355798	0.09790429	0.137449	0.1449307
1132.937	9.5	0.385128	0.3336183	0.1360643	0.097748	0.135869	0.1463878
1133.037	9.5	0.386974	0.3415804	0.1369764	0.09960036	0.135377	0.1464628
1133.137	9.5	0.39157	0.3475158	0.1396341	0.1007473	0.132319	0.1433175
1133.237	9.5	0.394162	0.3470294	0.1375725	0.1026637	0.130994	0.1463053
1133.337	9.5	0.389032	0.3540113	0.139108	0.1043989	0.130366	0.1510641
1133.437	9.5	0.395222	0.3404977	0.1400226	0.1053693	0.124649	0.1513284
1133.537	9.5	0.411964	0.3376479	0.1398626	0.1056797	0.124115	0.1503811
1133.637	9.5	0.413385	0.3363322	0.1410695	0.1067033	0.124321	0.1488646
1133.737	9.5	0.400275	0.3383344	0.1441387	0.1054409	0.12968	0.1455636
1133.837	9.5	0.385281	0.3409812	0.1421326	0.1066914	0.127665	0.1488626
1133.937	9.5	0.39558	0.3422728	0.1437434	0.1053262	0.12986	0.1470065
1134.037	9.5	0.390728	0.3411702	0.1434016	0.1066553	0.126958	0.1462294
1134.137	9.5	0.412152	0.3426663	0.1450877	0.107017	0.124377	0.1494021
1134.237	9.5	0.395005	0.3446348	0.1435695	0.1050604	0.123326	0.1464551
1134.337	9.5	0.401983	0.3397806	0.1444809	0.1029072	0.124386	0.1467861
1134.437	9.5	0.409682	0.3380529	0.1404644	0.1009932	0.128842	0.1451461
1134.537	9.5	0.397762	0.3324122	0.1418407	0.1001146	0.128888	0.1433367
1134.637	9.5	0.403119	0.3294236	0.1400773	0.1002416	0.129786	0.1435902
1134.737	9.5	0.409528	0.3255742	0.140351	0.09860529	0.128961	0.1449902
1134.837	9.5	0.410437	0.3222988	0.1394051	0.098111	0.129832	0.1451262
1134.937	9.5	0.409009	0.3247454	0.1383268	0.100403	0.124661	0.1450003
1135.037	9.5	0.393661	0.3054575	0.1403091	0.09939852	0.12102	0.1459699
1135.137	9.5	0.390963	0.3116647	0.1400987	0.09983364	0.122509	0.1486866
1135.237	9.5	0.398821	0.3265339	0.1389886	0.09658251	0.119462	0.1515548
1135.337	9.5	0.392033	0.3329092	0.139257	0.09609126	0.119934	0.1479231
1135.437	9.5	0.39074	0.3564209	0.140368	0.0973528	0.11728	0.1464082
1135.537	9.5	0.368513	0.3405392	0.140879	0.1005366	0.116901	0.1473176
1135.637	9.5	0.378344	0.3333601	0.1396104	0.09812562	0.119818	0.1480943
1135.737	9.5	0.387188	0.3318064	0.1373134	0.09752768	0.119501	0.1469635
1135.837	9.5	0.376317	0.3390808	0.1381647	0.0969584	0.118559	0.1445612
1135.937	9.5	0.377228	0.3459941	0.1362901	0.09792028	0.119234	0.144708
1136.037	9.5	0.381888	0.3517913	0.1386698	0.09743559	0.119227	0.1415019
1136.137	9.5	0.384899	0.3487643	0.138806	0.09705736	0.120226	0.1418281
1136.237	9.5	0.380683	0.3571499	0.1382828	0.09726634	0.120965	0.1438576
1136.337	9.5	0.404075	0.3445505	0.1384927	0.09884591	0.122824	0.1431642
1136.437	9.5	0.39684	0.3402649	0.1361495	0.09621924	0.125315	0.1446255
1136.537	9.5	0.405153	0.3392297	0.1369468	0.09546383	0.127254	0.1434171
1136.637	9.5	0.395862	0.3409167	0.137375	0.09927879	0.127027	0.1444649
1136.737	9.5	0.387857	0.3421327	0.1371739	0.0963265	0.127003	0.1456348
1136.837	9.5	0.397363	0.343627	0.1390183	0.09591541	0.124135	0.1468168
1136.937	9.5	0.382754	0.3459393	0.1430929	0.09895422	0.126498	0.1461082
1137.037	9.5	0.377498	0.3445076	0.1411267	0.09740124	0.125868	0.1444752
1137.137	9.5	0.390991	0.3435644	0.1398105	0.09570804	0.128718	0.1444944
1137.237	9.5	0.400157	0.3389707	0.1405146	0.09490616	0.130987	0.1469243
1137.337	9.5	0.38611	0.3339347	0.1382109	0.09669292	0.130258	0.1461665
1137.437	9.5	0.397537	0.3320269	0.1386682	0.09702908	0.132409	0.1468671
1137.537	9.5	0.385915	0.3271348	0.1381272	0.09526382	0.13393	0.1484171
1137.637	9.5	0.393283	0.3228443	0.1364502	0.09567104	0.133151	0.1502621
1137.737	9.5	0.393024	0.3232461	0.1342642	0.09602649	0.130387	0.1524002

1137.837	9.5	0.388913	0.3189295	0.1317445	0.09451359	0.135178	0.1493293
1137.937	9.5	0.370647	0.3086919	0.1278143	0.09409135	0.132766	0.1515852
1138.037	9.5	0.365469	0.3235258	0.1299713	0.0973528	0.130922	0.151456
1138.137	9.5	0.380858	0.3414468	0.1290078	0.09604245	0.130742	0.150883
1138.237	9.5	0.385425	0.360108	0.1317068	0.09593744	0.12664	0.1500383
1138.337	9.5	0.392697	0.3628479	0.131269	0.0980446	0.130069	0.1504979
1138.437	9.5	0.390723	0.3384019	0.1297882	0.09641954	0.12899	0.1467172
1138.537	9.5	0.390423	0.3338958	0.1346323	0.09585609	0.128694	0.1501276
1138.637	9.5	0.399857	0.3330038	0.1349601	0.09472125	0.130188	0.1525809
1138.737	9.5	0.386631	0.3411309	0.1298522	0.09663748	0.127266	0.1495776
1138.837	9.5	0.391762	0.346881	0.1309334	0.09830969	0.128967	0.1469549
1138.937	9.5	0.38571	0.3478192	0.1314097	0.09920074	0.127606	0.1457193
1139.037	9.5	0.386485	0.3602685	0.1307317	0.0989861	0.127633	0.147934
1139.191	10	0.389223	0.3411547	0.13082	0.1005756	0.132134	0.1478613
1139.291	10	0.39265	0.3415791	0.1313059	0.100914	0.130505	0.1462807
1139.391	10	0.387413	0.344777	0.1323337	0.1037748	0.13065	0.1464799
1139.491	10	0.387243	0.3461193	0.1307225	0.1046791	0.126361	0.1468786
1139.591	10	0.395867	0.3449751	0.1301367	0.1047003	0.1254	0.1477543
1139.691	10	0.391015	0.3459919	0.1286204	0.1029607	0.127236	0.1478602
1139.791	10	0.380874	0.3453666	0.1286451	0.1031802	0.125106	0.1458115
1139.891	10	0.3798	0.3388559	0.1291065	0.1027845	0.126744	0.1444458
1139.991	10	0.382171	0.3332804	0.1271524	0.1050429	0.12923	0.1459088
1140.091	10	0.386718	0.3295491	0.1275316	0.1043939	0.132021	0.1502095
1140.191	10	0.396163	0.3295886	0.1270144	0.1053291	0.128	0.1483345
1140.291	10	0.391495	0.3187226	0.127208	0.1020586	0.127579	0.1491137
1140.391	10	0.388396	0.3212727	0.1311052	0.1005742	0.129539	0.1465628
1140.491	10	0.395447	0.3224891	0.1328509	0.1007529	0.128536	0.1476791
1140.591	10	0.386743	0.3042137	0.1336794	0.1007112	0.126342	0.1470719
1140.691	10	0.389815	0.309008	0.1393209	0.1001757	0.125742	0.1450018
1140.791	10	0.391613	0.3219375	0.14025	0.1002529	0.12916	0.1478299
1140.891	10	0.397546	0.3461333	0.1404245	0.09961557	0.131432	0.1505622
1140.991	10	0.400371	0.362544	0.1431951	0.1019927	0.130852	0.1487863
1141.091	10	0.385785	0.3379343	0.1444242	0.1007484	0.127731	0.1481482
1141.191	10	0.382784	0.33303	0.1421566	0.09991775	0.127883	0.1493458
1141.291	10	0.395877	0.3336009	0.142555	0.1000088	0.124606	0.1458783
1141.391	10	0.388106	0.3384117	0.1400297	0.1007717	0.124243	0.1464781
1141.491	10	0.368431	0.3466911	0.1405809	0.100046	0.12307	0.1446536
1141.591	10	0.385038	0.34813	0.1425741	0.1003101	0.120841	0.1462612
1141.691	10	0.38891	0.359246	0.1411237	0.09883571	0.120416	0.1482067
1141.791	10	0.380392	0.3512613	0.1392634	0.09842124	0.120583	0.1431496
1141.891	10	0.375544	0.3462263	0.1383488	0.09817845	0.116527	0.1454695
1141.991	10	0.385107	0.3401595	0.139066	0.09860425	0.117715	0.1460548
1142.091	10	0.384115	0.3405802	0.1399772	0.09681471	0.118342	0.1454657
1142.191	10	0.379935	0.3415835	0.1366383	0.09780108	0.121582	0.1435648
1142.291	10	0.413571	0.3434159	0.1394507	0.09672228	0.119545	0.1447292
1142.391	10	0.405232	0.3416769	0.1404902	0.1005205	0.120995	0.1471325
1142.491	10	0.408961	0.342968	0.1411687	0.09865031	0.119784	0.1474164
1142.591	10	0.396194	0.3425041	0.1419602	0.09475592	0.120103	0.1484251
1142.691	10	0.393041	0.3367847	0.1442038	0.09548062	0.121032	0.1473922
1142.791	10	0.396512	0.32882	0.1437539	0.09449245	0.120913	0.1477678
1142.891	10	0.388787	0.3286307	0.1420581	0.09469007	0.1226	0.1470723
1142.991	10	0.381747	0.3291333	0.1395871	0.09243003	0.121537	0.1463134
1143.091	10	0.398698	0.3224358	0.1369478	0.09463087	0.118124	0.1424697
1143.191	10	0.394892	0.3170986	0.1388867	0.09890255	0.113852	0.1451456
1143.291	10	0.397176	0.3145607	0.1389323	0.09751735	0.116881	0.1469346
1143.391	10	0.392391	0.2996357	0.1373734	0.0943201	0.119991	0.147713
1143.491	10	0.384072	0.3115413	0.1376024	0.09444427	0.121554	0.1479898
1143.591	10	0.395088	0.3208837	0.1367945	0.09326547	0.126445	0.1424563
1143.691	10	0.388169	0.3277112	0.136875	0.09514455	0.127051	0.1431851
1143.791	10	0.385251	0.3544158	0.1348787	0.1014921	0.13016	0.1413632
1143.891	10	0.373846	0.3345951	0.1355521	0.0978694	0.129788	0.1391504
1143.991	10	0.369647	0.333109	0.1377529	0.09925717	0.132042	0.1413734
1144.091	10	0.393398	0.3352093	0.1396404	0.09871863	0.131518	0.1418228
1144.191	10	0.398325	0.3421678	0.1378117	0.09740557	0.132103	0.1434554
1144.291	10	0.399865	0.3502208	0.138611	0.09730816	0.135406	0.1428027
1144.391	10	0.396555	0.3493657	0.1366029	0.09551984	0.135546	0.1436936
1144.491	10	0.391743	0.3551126	0.1349292	0.097362	0.131835	0.1465766
1144.591	10	0.387031	0.3509779	0.1343414	0.1008876	0.131062	0.1462434
1144.691	10	0.396431	0.3474967	0.1320852	0.1024365	0.13331	0.1446172
1144.791	10	0.382762	0.3433983	0.1334889	0.09813916	0.130476	0.1459787
1144.891	10	0.379015	0.3437815	0.1302546	0.09868263	0.128522	0.1468364
1144.991	10	0.387162	0.3472347	0.1268988	0.09889261	0.130138	0.1471972
1145.091	10	0.381384	0.3475605	0.1275031	0.1014728	0.127945	0.1485565
1145.191	10	0.39206	0.3471223	0.1285046	0.1010037	0.128152	0.1504493
1145.291	10	0.39231	0.3477346	0.1312763	0.1020583	0.128045	0.1529952
1145.391	10	0.411418	0.3461024	0.1290797	0.1053026	0.129595	0.1495994
1145.491	10	0.400973	0.3375124	0.128053	0.1063259	0.131469	0.1516415
1145.591	10	0.427668	0.3280948	0.1324902	0.1028137	0.130505	0.1539703
1145.691	10	0.401637	0.3309328	0.1324659	0.1032164	0.134465	0.1535729
1145.791	10	0.389155	0.3290418	0.1289952	0.1045118	0.131792	0.1546952
1145.891	10	0.379771	0.3167981	0.1297677	0.104877	0.13158	0.1556925
1145.991	10	0.384746	0.3149894	0.1286257	0.1071155	0.128635	0.152729
1146.091	10	0.38459	0.3106944	0.1266694	0.10664	0.128792	0.1533229
1146.191	10	0.402928	0.2994333	0.1279584	0.1099921	0.128469	0.1556599
1146.291	10	0.388913	0.317156	0.1317415	0.1079386	0.12727	0.1514604
1146.391	10	0.388379	0.3247782	0.1323392	0.105648	0.128608	0.1489435
1146.491	10	0.394931	0.3416321	0.1332036	0.1045032	0.126306	0.1448274
1146.591	10	0.383855	0.3566183	0.1322627	0.1027034	0.125917	0.1446101
1146.691	10	0.382465	0.3348472	0.1357916	0.1027702	0.126633	0.1453504
1146.791	10	0.390078	0.3322152	0.1373415	0.1025907	0.12561	0.1456562
1146.891	10	0.394592	0.3342148	0.1372674	0.09878014	0.125132	0.1434576
1146.991	10	0.397379	0.3422123	0.1379058	0.1031597	0.124176	0.1442657
1147.091	10	0.391842	0.3467082	0.1333404	0.1023313	0.124638	0.1446715
1147.191	10	0.383951	0.3477169	0.1369604	0.1021396	0.125993	0.1470776
1147.291	10	0.394174	0.3562172	0.1407487	0.1018407	0.123393	0.1470057

1147.391	10	0.388812	0.3516347	0.141925	0.1043596	0.121562	0.1444939
1147.491	10	0.371631	0.3471513	0.1404191	0.1054404	0.120223	0.1434597
1147.591	10	0.39175	0.3430586	0.141849	0.1093722	0.12066	0.1459332
1147.691	10	0.394654	0.343377	0.1410535	0.1081796	0.121177	0.1454648
1147.791	10	0.387507	0.3435286	0.1427137	0.1063701	0.122145	0.1482982
1147.891	10	0.380547	0.3458864	0.1417615	0.104073	0.119353	0.1454898
1147.991	10	0.385162	0.3453438	0.1416814	0.1048542	0.119361	0.1470147
1148.091	10	0.386432	0.3440308	0.1429175	0.1040933	0.121051	0.1466635
1148.191	10	0.380244	0.3418757	0.1419514	0.1053916	0.119061	0.1439129
1148.291	10	0.402531	0.3351364	0.1419972	0.104228	0.118968	0.1479368
1148.391	10	0.396207	0.3266526	0.1411944	0.1060678	0.118376	0.1492241
1148.491	10	0.404778	0.3291887	0.1408255	0.1043645	0.119982	0.1486732
1148.591	10	0.395478	0.3280556	0.1399562	0.101483	0.120573	0.1464487
1148.691	10	0.390962	0.3132684	0.1417875	0.0998418	0.122645	0.1464745
1148.791	10	0.390728	0.3171923	0.1394197	0.1002752	0.123139	0.1440613
1148.891	10	0.3878	0.312285	0.1400541	0.1015962	0.124391	0.1466552
1148.991	10	0.384367	0.297428	0.1376987	0.1000823	0.124152	0.1460016
1149.091	10	0.394452	0.318141	0.1405903	0.1032027	0.125303	0.1480558
1149.191	10	0.389729	0.3272918	0.1416755	0.1047868	0.122251	0.1483197
1149.291	10	0.397961	0.3516185	0.138905	0.1056535	0.122899	0.1437672
1149.391	10	0.389165	0.3588323	0.1414099	0.1029413	0.123579	0.1431896
1149.491	10	0.389444	0.3364501	0.1398083	0.1030133	0.118825	0.1424083
1149.591	10	0.40825	0.3333758	0.1400744	0.1017197	0.11974	0.1397644
1149.691	10	0.393616	0.3349801	0.1410678	0.1027819	0.12154	0.1397025
1149.791	10	0.382393	0.3420655	0.1436885	0.103927	0.122475	0.1428254
1149.891	10	0.368959	0.3468585	0.1431522	0.1052672	0.126119	0.1426321
1149.991	10	0.377226	0.3483952	0.1418414	0.1036496	0.129316	0.1439647
1150.091	10	0.393656	0.3570028	0.1406033	0.1026124	0.129794	0.146318
1150.191	10	0.400174	0.3501246	0.1396172	0.1018021	0.128547	0.1488393
1150.291	10	0.398218	0.3451213	0.142181	0.10107	0.128345	0.1516554
1150.391	10	0.397556	0.3432232	0.1423713	0.1004804	0.131971	0.1491458
1150.491	10	0.388594	0.343427	0.13998	0.1023579	0.135527	0.1455906
1150.591	10	0.391003	0.3452871	0.1397858	0.1027905	0.135224	0.1428725
1150.691	10	0.394529	0.3454234	0.1405067	0.1048925	0.134779	0.1458441
1150.791	10	0.383215	0.3474115	0.1386175	0.1007714	0.134675	0.1439734
1150.891	10	0.382812	0.345643	0.1370793	0.09929837	0.133717	0.143866
1150.991	10	0.388385	0.3451586	0.1382233	0.1003254	0.13202	0.143682
1151.091	10	0.383358	0.3386759	0.1392542	0.1030531	0.13238	0.1402433
1151.191	10	0.3935	0.3303944	0.1381963	0.1035035	0.133141	0.1402137
1151.291	10	0.393707	0.3319923	0.1383765	0.1077069	0.135282	0.1410618
1151.391	10	0.411273	0.32894	0.1386329	0.1095323	0.131701	0.1423603
1151.491	10	0.399491	0.3238793	0.1365933	0.1087812	0.131535	0.142547
1151.591	10	0.412826	0.3173774	0.1370745	0.1070943	0.132109	0.140723
1151.691	10	0.404982	0.3087959	0.1368379	0.1064435	0.133987	0.1421186
1151.791	10	0.388168	0.3188573	0.1362322	0.1026292	0.135805	0.1408049
1151.891	10	0.382335	0.3381295	0.1333493	0.1026353	0.133924	0.143924
1151.991	10	0.384439	0.356115	0.132123	0.104485	0.136393	0.1443401
1152.091	10	0.3829	0.3655159	0.1285098	0.105073	0.134762	0.1450928
1152.191	10	0.396151	0.3597104	0.1310984	0.1066127	0.131334	0.145303
1152.291	10	0.386676	0.3485238	0.1296081	0.1018848	0.130713	0.14541
1152.391	10	0.389196	0.3532497	0.1346272	0.1013365	0.126816	0.1440479
1152.491	10	0.395046	0.3603845	0.132147	0.1023743	0.127457	0.144645
1152.591	10	0.383129	0.365501	0.1324189	0.1034362	0.127584	0.1457094
1152.691	10	0.383171	0.3708443	0.1344223	0.1024164	0.125729	0.1466583
1152.791	10	0.387859	0.3533142	0.1301949	0.1002392	0.126422	0.1489237
1152.891	10	0.391559	0.3652727	0.1305128	0.09836078	0.126932	0.1490394
1152.991	10	0.397321	0.3588191	0.1339719	0.1037785	0.126487	0.1510163
1153.091	10	0.388884	0.3570497	0.1328178	0.1033776	0.123942	0.1507182
1153.191	10	0.381827	0.3586709	0.132351	0.1043034	0.122712	0.1516305
1153.291	10	0.393556	0.3640334	0.1333755	0.1028887	0.123678	0.1527218
1153.391	10	0.387183	0.3678063	0.1358016	0.1043112	0.124544	0.1546136
1153.491	10	0.368533	0.3701222	0.1338961	0.1035107	0.127056	0.1539271
1153.591	10	0.389212	0.371308	0.1358422	0.1015629	0.127833	0.1548512
1153.691	10	0.39395	0.3687624	0.1335966	0.1006436	0.128476	0.1559501
1153.791	10	0.388273	0.3619388	0.1354125	0.1001806	0.128218	0.152051
1153.891	10	0.375921	0.3499458	0.1370496	0.1003366	0.125764	0.1495724
1153.991	10	0.388167	0.3403854	0.1398341	0.09960367	0.124968	0.1458177
1154.091	10	0.386746	0.3359948	0.1412061	0.09968581	0.124167	0.1458961
1154.191	10	0.380577	0.3284214	0.1386148	0.09942393	0.123536	0.1452523
1154.291	10	0.40368	0.3248057	0.1381274	0.0966576	0.122416	0.143491
1154.391	10	0.400771	0.3236788	0.1395733	0.09971227	0.120847	0.1403034
1154.491	10	0.408694	0.31628	0.1400116	0.09937714	0.11858	0.1434764
1154.591	10	0.39542	0.3222513	0.1412637	0.09507074	0.119083	0.1444578
1154.691	10	0.392686	0.3324072	0.1397247	0.09531928	0.118753	0.1464443
1154.791	10	0.396354	0.3532706	0.1412504	0.09614261	0.120639	0.1452269
1154.891	10	0.391705	0.3587853	0.1422532	0.09725191	0.120761	0.1433511
1154.991	10	0.381636	0.3527463	0.1408824	0.0957571	0.120809	0.1441907
1155.091	10	0.397684	0.3409413	0.1417109	0.09837854	0.121086	0.1463213
1155.191	10	0.39475	0.3430857	0.1432743	0.1006161	0.118446	0.1454751
1155.291	10	0.39504	0.3489261	0.1417864	0.1016169	0.115306	0.1465391
1155.391	10	0.389991	0.3573085	0.1444452	0.100908	0.117103	0.14575
1155.491	10	0.3811	0.3622573	0.1415083	0.1024071	0.118615	0.1473752
1155.591	10	0.398393	0.3491613	0.1403368	0.1007526	0.121572	0.1455658
1155.691	10	0.39629	0.3642839	0.1422575	0.101113	0.122469	0.1421263
1155.791	10	0.383194	0.3510725	0.143971	0.1022776	0.121824	0.145579
1155.891	10	0.381371	0.3492774	0.1408177	0.1007316	0.124541	0.1498099
1155.991	10	0.420547	0.3505023	0.1376034	0.1023649	0.126723	0.1483658
1156.091	10	0.414542	0.3530791	0.1380934	0.09975854	0.128127	0.1462486
1156.191	10	0.421653	0.3566805	0.1417557	0.1009861	0.128578	0.148523
1156.291	10	0.427487	0.3566869	0.1396089	0.1000814	0.12803	0.1471435
1156.391	10	0.416484	0.3590762	0.139944	0.09909135	0.129451	0.1486911
1156.491	10	0.400202	0.3552872	0.140378	0.1013177	0.132558	0.1478244
1156.591	10	0.399957	0.3507344	0.1386687	0.1035772	0.133808	0.148764
1156.691	10	0.397863	0.3438564	0.1375079	0.1020633	0.133485	0.1483141
1156.791	10	0.390032	0.3350208	0.1408498	0.09999413	0.132546	0.1441564

1156.891	10	0.391734	0.3339456	0.1428807	0.0993364	0.134498	0.1430541
1156.991	10	0.403406	0.3259331	0.1424942	0.1003874	0.131984	0.1441499
1157.091	10	0.393486	0.3176288	0.1395578	0.1021217	0.134677	0.145117
1157.191	10	0.395486	0.3113392	0.139864	0.1044952	0.131667	0.1447955
1157.291	10	0.398408	0.3191685	0.1404019	0.1064967	0.135595	0.1451594
1157.391	10	0.41579	0.3228248	0.1411534	0.1044396	0.135905	0.1444287
1157.491	10	0.402491	0.3250841	0.1422751	0.1036541	0.134149	0.1424786
1157.591	10	0.424066	0.3452895	0.1412386	0.1037464	0.137053	0.1417312
1157.691	10	0.402646	0.3574268	0.1378714	0.1066476	0.135018	0.143135
1157.791	10	0.396542	0.3496597	0.1393074	0.1063339	0.134009	0.1478379
1157.891	10	0.384769	0.3406771	0.1388197	0.1058786	0.133605	0.1460167
1157.991	10	0.387214	0.3383599	0.1381961	0.1090084	0.134348	0.1435631
1158.091	10	0.386994	0.3456239	0.1367195	0.1066984	0.133789	0.1431083
1158.191	10	0.409381	0.3518133	0.1388593	0.106808	0.134633	0.145251
1158.291	10	0.407907	0.3548513	0.13742	0.1047655	0.135786	0.1437811
1158.391	10	0.435629	0.3481605	0.1384244	0.1045893	0.134937	0.143634
1158.491	10	0.430245	0.3617015	0.1373243	0.1055848	0.130366	0.1426148
1158.591	10	0.411634	0.3432727	0.1365118	0.104975	0.133974	0.1405356
1158.691	10	0.402878	0.3412712	0.1368138	0.1045857	0.133409	0.1433558
1158.791	10	0.40255	0.3449713	0.136314	0.1051103	0.132358	0.1426392
1158.891	10	0.40223	0.3467036	0.1360404	0.102929	0.131324	0.1399349
1158.991	10	0.409118	0.3490892	0.1351101	0.1030585	0.12976	0.1421136
1159.091	10	0.389134	0.348985	0.1315367	0.1006442	0.130326	0.1415074
1159.191	10	0.385552	0.3510738	0.1304518	0.1016789	0.126657	0.1434184
1159.291	10	0.399425	0.3483447	0.1299818	0.1020483	0.125782	0.1442924
1159.391	10	0.394925	0.3454708	0.1316217	0.1019955	0.127102	0.1446078
1159.491	10	0.379275	0.3404402	0.1301564	0.09898889	0.126673	0.1435876
1159.591	10	0.392672	0.3342612	0.1257741	0.1029695	0.127067	0.1439534
1159.691	10	0.397338	0.3306613	0.1313281	0.1001477	0.125376	0.1447321
1159.791	10	0.395498	0.3239885	0.1320937	0.1015278	0.124232	0.1458151
1159.891	10	0.383146	0.3128957	0.1288242	0.1000127	0.123827	0.1455394
1159.991	10	0.393792	0.3168667	0.1299819	0.09896401	0.124929	0.1471246
1160.091	10	0.388617	0.3195608	0.1310272	0.09642208	0.122453	0.1459851
1160.191	10	0.380229	0.3178568	0.1297389	0.09562249	0.121446	0.1484479
1160.291	10	0.404848	0.3186501	0.129565	0.09681689	0.120714	0.1511172
1160.391	10	0.398429	0.3455066	0.1311145	0.1012149	0.121144	0.1517166
1160.491	10	0.405897	0.3651052	0.1320518	0.09855836	0.121066	0.1516574
1160.591	10	0.39622	0.3476297	0.1334546	0.09401746	0.122462	0.1530045
1160.691	10	0.390617	0.3364674	0.1329371	0.09501755	0.124682	0.1550442
1160.791	10	0.402667	0.335187	0.1337405	0.0947272	0.126035	0.1551556
1160.891	10	0.394061	0.3428433	0.1330731	0.09858887	0.123766	0.1573678
1160.991	10	0.379301	0.3509542	0.1359907	0.1003637	0.121625	0.1530142
1161.091	10	0.397521	0.3535277	0.1390081	0.100888	0.124145	0.1509389
1161.191	10	0.394469	0.3496546	0.135851	0.09951894	0.123402	0.1504487
1161.291	10	0.399922	0.3598662	0.1329687	0.09578087	0.125109	0.1489886
1161.391	10	0.391284	0.3442787	0.1343326	0.09795865	0.126052	0.1474213
1161.491	10	0.382321	0.3417284	0.1359464	0.09904934	0.124527	0.1466156
1161.591	10	0.40319	0.3444684	0.1355315	0.09776749	0.122681	0.1450285
1161.691	10	0.39201	0.3459379	0.138931	0.09799563	0.122907	0.1449471
1161.791	10	0.382084	0.3468805	0.1391267	0.09906012	0.123256	0.1462156
1161.891	10	0.371017	0.3455378	0.1402343	0.09705655	0.125785	0.1484908
1161.991	10	0.384094	0.348821	0.1402403	0.09850316	0.125858	0.1483807
1162.091	10	0.390874	0.3480858	0.1402941	0.09651995	0.119415	0.1462418
1162.191	10	0.393816	0.3431773	0.1431491	0.09746613	0.117863	0.1469801
1162.291	10	0.394057	0.3375709	0.14257	0.09778864	0.121442	0.1502054
1162.391	10	0.392719	0.3321133	0.1442734	0.09598681	0.122867	0.1459531
1162.491	10	0.389272	0.32939	0.1445882	0.09718184	0.125473	0.1442607
1162.591	10	0.381635	0.3211282	0.1445037	0.09963868	0.129528	0.1440854
1162.691	10	0.397476	0.3079918	0.1433264	0.1007778	0.130471	0.1396413
1162.791	10	0.385685	0.3115111	0.144745	0.09761254	0.134571	0.1405359
1162.891	10	0.386341	0.3212832	0.1401763	0.09544463	0.134071	0.1429144
1162.991	10	0.386926	0.3165025	0.1409767	0.09621282	0.136933	0.1438372
1163.091	10	0.385715	0.3215032	0.1377631	0.1012113	0.135456	0.1464556
1163.191	10	0.394967	0.353118	0.1395208	0.1043321	0.136128	0.1449911
1163.291	10	0.395935	0.3686181	0.1386265	0.1032877	0.136661	0.1438679
1163.391	10	0.416665	0.3406727	0.1355876	0.1015728	0.135988	0.1435187
1163.491	10	0.40479	0.3316945	0.1377378	0.1009804	0.13405	0.1489206
1163.591	10	0.402288	0.3319432	0.1361564	0.1034309	0.136998	0.1480163
1163.691	10	0.3943	0.3394628	0.1355148	0.1052359	0.135655	0.146816
1163.791	10	0.418172	0.3486578	0.1365416	0.1032473	0.134671	0.1470031
1163.891	10	0.41833	0.3504665	0.1394798	0.1020762	0.134119	0.1431758
1163.991	10	0.41752	0.3476432	0.1399561	0.1055242	0.135303	0.1439694
1164.091	10	0.390649	0.3566973	0.1409666	0.1044073	0.132569	0.1425464
1164.191	10	0.410284	0.3435676	0.1396867	0.1045705	0.130281	0.1415533
1164.291	10	0.403916	0.3399903	0.1359415	0.10275	0.133087	0.1423777
1164.391	10	0.448641	0.3428349	0.1368677	0.1057493	0.133027	0.1446137
1164.491	10	0.443253	0.3438313	0.1361589	0.1055098	0.131008	0.1465821
1164.591	10	0.4256	0.3450312	0.1356376	0.1046451	0.1342	0.1448705
1164.691	10	0.408899	0.3424436	0.1336533	0.1052905	0.135052	0.1469122
1164.791	10	0.410627	0.3439017	0.1342374	0.1061038	0.132186	0.148727
1164.891	10	0.406163	0.3452147	0.1357508	0.1014168	0.134084	0.1457767
1164.991	10	0.401868	0.3414885	0.1344928	0.1022715	0.134053	0.1441049
1165.091	10	0.393944	0.3361517	0.1351425	0.09992205	0.131194	0.1416228
1165.191	10	0.400101	0.3278985	0.1375287	0.1031134	0.130067	0.1448172
1165.291	10	0.407308	0.3255288	0.1372362	0.1037262	0.127644	0.1443598
1165.391	10	0.40112	0.3190025	0.1378845	0.1025012	0.129467	0.1448411
1165.491	10	0.386221	0.3131669	0.1387781	0.100002	0.126453	0.1445335
1165.591	10	0.399018	0.3151604	0.1365128	0.1039077	0.126564	0.1425481
1165.691	10	0.402893	0.3231699	0.1363813	0.1021255	0.126797	0.1433713
1165.791	10	0.400227	0.3188856	0.1348408	0.1018962	0.122612	0.1427475
1165.891	10	0.37925	0.3260987	0.1349106	0.1027301	0.121066	0.1424749
1165.991	10	0.392521	0.3519998	0.1341429	0.1027136	0.121248	0.1437042
1166.091	10	0.38805	0.3600237	0.1323575	0.1035945	0.123161	0.1464488
1166.191	10	0.378841	0.342612	0.1260047	0.1051082	0.121322	0.1462435
1166.291	10	0.404822	0.3340818	0.1293252	0.101903	0.121742	0.1455291

1166.391	10	0.397283	0.3354339	0.1282668	0.1040699	0.121046	0.1458101
1166.491	10	0.401866	0.3428774	0.1316326	0.1011912	0.122415	0.1460111
1166.591	10	0.388916	0.3490078	0.1292595	0.09780613	0.119994	0.1433949
1166.691	10	0.386647	0.3486136	0.1282665	0.09676494	0.117836	0.1450492
1166.791	10	0.389986	0.3538359	0.1315929	0.09710561	0.117994	0.1452679
1166.891	10	0.386047	0.3577518	0.1296612	0.0971677	0.118585	0.1456335
1166.991	10	0.378967	0.3461608	0.1298028	0.09705314	0.121023	0.1483856
1167.091	10	0.392147	0.3405308	0.1313154	0.09627043	0.119939	0.1497705
1167.191	10	0.391109	0.3437317	0.1303713	0.09757466	0.119045	0.1509527
1167.291	10	0.394418	0.3438949	0.1292939	0.09771831	0.120179	0.1472783
1167.391	10	0.387531	0.3468037	0.1303891	0.09846369	0.12165	0.1479651
1167.491	10	0.382055	0.3442411	0.1330657	0.1019427	0.121287	0.1498691
1167.591	10	0.401495	0.3436164	0.1322306	0.1005431	0.120128	0.1500599
1167.691	10	0.3955	0.3455673	0.1341761	0.1007313	0.121801	0.1498738
1167.791	10	0.382239	0.3412064	0.1357584	0.1042848	0.123673	0.1507624
1167.891	10	0.372069	0.3341365	0.138235	0.10341	0.123247	0.1523471
1167.991	10	0.378235	0.3274823	0.1386003	0.1021997	0.123946	0.1545499
1168.091	10	0.391031	0.327296	0.1394456	0.09736102	0.121045	0.1520167
1168.191	10	0.397325	0.3219229	0.1414742	0.09755781	0.122798	0.1486924
1168.291	10	0.401641	0.3167763	0.1366564	0.09814547	0.124745	0.1468895
1168.391	10	0.398196	0.321771	0.1357519	0.09654889	0.125205	0.1474835
1168.491	10	0.389275	0.3258174	0.1373481	0.101031	0.125191	0.1468328
1168.591	10	0.382432	0.3339138	0.1388066	0.1046651	0.125665	0.1461091
1168.691	10	0.390867	0.3484103	0.1389693	0.1042684	0.125065	0.1492269
1168.791	10	0.382657	0.3549671	0.13929	0.1010151	0.127004	0.1498713
1168.891	10	0.380923	0.359307	0.1408219	0.1004559	0.129265	0.1510534
1168.991	10	0.385797	0.3515375	0.1426467	0.1008111	0.130978	0.1505097
1169.091	10	0.380258	0.343596	0.1418883	0.09966007	0.134958	0.1471088
1169.191	10	0.393094	0.3444456	0.1442995	0.1025967	0.134151	0.1463984
1169.291	10	0.389692	0.3499703	0.1443214	0.102328	0.135647	0.149702
1169.391	10	0.405057	0.3605152	0.1401528	0.1034559	0.134803	0.1484322
1169.491	10	0.392386	0.3554352	0.1411045	0.1021312	0.132344	0.145538
1169.591	10	0.398349	0.3531863	0.1389182	0.1002356	0.132907	0.1440716
1169.691	10	0.388828	0.3557618	0.1406665	0.1057896	0.134422	0.146121
1169.791	10	0.379118	0.3502255	0.1408723	0.1056622	0.135796	0.1462242
1169.891	10	0.382563	0.3504497	0.1406309	0.1044584	0.137441	0.1471467
1169.991	10	0.379175	0.3583958	0.1375791	0.1056994	0.13454	0.1485081
1170.091	10	0.381262	0.3636674	0.1373578	0.1054044	0.129604	0.1473516
1170.191	10	0.392245	0.3665287	0.137875	0.1068188	0.129998	0.1462611
1170.291	10	0.38451	0.3653176	0.1382956	0.1068121	0.131622	0.1450608
1170.391	10	0.386432	0.3644975	0.1355385	0.1089981	0.133033	0.1446569
1170.491	10	0.387635	0.3624572	0.1356141	0.1075033	0.135067	0.1454862
1170.591	10	0.382684	0.3553337	0.1376026	0.1059085	0.13423	0.1456168
1170.691	10	0.384563	0.3483214	0.1360752	0.1058926	0.136094	0.1467333
1170.791	10	0.383365	0.3408825	0.1382657	0.1055342	0.135634	0.1498813
1170.891	10	0.391429	0.335642	0.1407781	0.1044764	0.132953	0.1469179
1170.991	10	0.39405	0.3262973	0.1426412	0.103087	0.131626	0.1454187
1171.091	10	0.381858	0.3239334	0.1422562	0.1006962	0.132534	0.144291
1171.191	10	0.379662	0.3215133	0.1415724	0.1022951	0.130654	0.14439
1171.291	10	0.39242	0.3227299	0.1408776	0.1052149	0.131529	0.1450669
1171.391	10	0.386579	0.3292437	0.1382404	0.1026095	0.131762	0.1455918
1171.491	10	0.365952	0.339439	0.1389116	0.09912761	0.129088	0.1461944
1171.591	10	0.383941	0.3537883	0.1366605	0.0997046	0.127947	0.1469457
1171.691	10	0.391547	0.3670112	0.1329919	0.09895367	0.127099	0.1485352
1171.791	10	0.385296	0.3490103	0.1323963	0.09925573	0.125689	0.1469692
1171.891	10	0.375369	0.3428872	0.135439	0.09808274	0.125616	0.1480187
1171.991	10	0.382644	0.3446811	0.1352319	0.0942928	0.124845	0.1458477
1172.091	10	0.387352	0.3485622	0.1355373	0.09552224	0.125028	0.145974
1172.191	10	0.373956	0.3570341	0.1371962	0.09812688	0.12537	0.1477061
1172.291	10	0.402085	0.3506745	0.1409148	0.09865301	0.128151	0.1474042
1172.391	10	0.39691	0.3512955	0.1376869	0.1007683	0.12575	0.1471669
1172.491	10	0.410689	0.352318	0.137817	0.100947	0.123553	0.1445948
1172.591	10	0.398222	0.3433115	0.1383396	0.0947745	0.120936	0.139338
1172.691	10	0.398266	0.3397394	0.1343434	0.09470309	0.121697	0.1388168
1172.791	10	0.391252	0.34231	0.1339239	0.09393172	0.121422	0.1403261
1172.891	10	0.391037	0.3468676	0.1324207	0.09580038	0.120962	0.1387666
1172.991	10	0.380756	0.3497642	0.1335492	0.09366643	0.121624	0.1410341
1173.091	10	0.389991	0.3494363	0.1323238	0.09351955	0.121562	0.1439828
1173.191	10	0.390384	0.3508482	0.1309903	0.09484632	0.119932	0.145515
1173.291	10	0.397154	0.3491003	0.1276418	0.09672672	0.120036	0.1442719
1173.391	10	0.389619	0.3398027	0.1313149	0.0967533	0.121586	0.1434947
1173.491	10	0.3918	0.3342886	0.1313309	0.1012201	0.12307	0.1422241
1173.591	10	0.400673	0.3308412	0.1325419	0.1020599	0.123132	0.1412594
1173.691	10	0.392889	0.3294527	0.1301214	0.1016007	0.122057	0.1436759
1173.791	10	0.379688	0.3237487	0.1316482	0.1021805	0.122291	0.1465756
1173.891	10	0.369985	0.3297541	0.1339415	0.1011953	0.122109	0.1474919
1173.991	10	0.377238	0.3356197	0.1286866	0.1023754	0.120908	0.1478786
1174.091	10	0.389907	0.3293217	0.1282729	0.1007478	0.121208	0.1500902
1174.191	10	0.393512	0.3336148	0.1301093	0.09918332	0.122444	0.1504093
1174.291	10	0.400638	0.3399233	0.1301089	0.1004357	0.121957	0.1474148
1174.391	10	0.402773	0.3617951	0.1308789	0.09852607	0.121054	0.1487214
1174.491	10	0.389961	0.3707584	0.1328589	0.09945814	0.121617	0.1475793
1174.591	10	0.382963	0.3457399	0.1347631	0.1015691	0.121521	0.1496164
1174.691	10	0.394609	0.3375507	0.132875	0.1010283	0.124774	0.1491657
1174.791	10	0.384596	0.3398402	0.1347482	0.1012712	0.127978	0.1517913
1174.891	10	0.376479	0.3441103	0.1335488	0.09957409	0.131236	0.1519944
1174.991	10	0.381537	0.3507235	0.1335064	0.1025243	0.1367	0.1532452
1175.091	10	0.3861	0.3499219	0.1358185	0.1044334	0.138136	0.1522551
1175.191	10	0.397445	0.3618958	0.1368968	0.107171	0.137844	0.1513148
1175.291	10	0.39134	0.3523212	0.1385508	0.1064828	0.138549	0.1486706
1175.391	10	0.403429	0.3428867	0.1362525	0.1063646	0.139785	0.1493398
1175.491	10	0.38978	0.3417146	0.1381559	0.1061528	0.141089	0.1481153
1175.591	10	0.397594	0.3420356	0.1383601	0.1085253	0.138121	0.1499212
1175.691	10	0.388357	0.3448004	0.1402067	0.1095469	0.139212	0.1460664
1175.791	10	0.385538	0.3451745	0.139967	0.1075292	0.138627	0.1486168

1175.891	10	0.38713	0.3430071	0.1378961	0.1032509	0.137945	0.1475037
1175.991	10	0.383449	0.3453045	0.1367016	0.1041706	0.137897	0.148736
1176.091	10	0.38146	0.3449639	0.1390721	0.104107	0.134138	0.1470272
1176.191	10	0.394964	0.3373425	0.1396648	0.1041975	0.133187	0.1440471
1176.291	10	0.387588	0.3336971	0.1417487	0.1037598	0.131236	0.1441666
1176.391	10	0.390883	0.3318092	0.1425697	0.1024455	0.131397	0.1488552
1176.491	10	0.393743	0.3267446	0.1381771	0.1040732	0.136638	0.1455116
1176.591	10	0.385028	0.3165535	0.1385953	0.1059098	0.133931	0.1458943
1176.691	10	0.382984	0.3375167	0.1379205	0.1057586	0.135491	0.1456128
1176.791	10	0.385495	0.3282806	0.1381475	0.106172	0.13601	0.1473944
1176.891	10	0.388913	0.3285469	0.1392113	0.1020867	0.133408	0.1463344
1176.991	10	0.394706	0.3333342	0.1374022	0.1046184	0.132201	0.1443195
1177.091	10	0.3818	0.3472941	0.1344727	0.1018483	0.131159	0.1438781
1177.191	10	0.380667	0.3649619	0.1336702	0.1037659	0.129982	0.1450293
1177.291	10	0.392113	0.3668771	0.1350091	0.1035788	0.131251	0.1446057
1177.391	10	0.385932	0.3440835	0.1373902	0.1037819	0.133152	0.1463299
1177.491	10	0.369744	0.3359576	0.1335703	0.1035089	0.131541	0.1444378
1177.591	10	0.384117	0.3372954	0.135947	0.1036115	0.130065	0.1459579
1177.691	10	0.392587	0.3434235	0.1368974	0.1034332	0.130646	0.1462312
1177.791	10	0.39328	0.34856	0.1375859	0.1028675	0.127105	0.1484683
1177.891	10	0.374107	0.3469738	0.1377648	0.1021155	0.125539	0.150166
1177.991	10	0.387362	0.3569603	0.1392335	0.1022654	0.124663	0.1440812
1178.091	10	0.388375	0.3544566	0.1394058	0.09930644	0.126995	0.1446214
1178.191	10	0.377936	0.3445309	0.1384764	0.1002826	0.126962	0.141997
1178.291	10	0.404588	0.3439476	0.1358946	0.09805458	0.129972	0.1426913
1178.391	10	0.398772	0.3458272	0.1347428	0.1012918	0.128059	0.143212
1178.491	10	0.405475	0.3477764	0.1371708	0.1003433	0.127488	0.146046
1178.591	10	0.391066	0.350034	0.13677	0.09603296	0.125636	0.1463479
1178.691	10	0.391209	0.3481156	0.1340942	0.09661445	0.124126	0.143412
1178.791	10	0.395105	0.3469771	0.1345358	0.09428222	0.123273	0.1430703
1178.891	10	0.392308	0.34565	0.1352966	0.09571446	0.12253	0.1457572
1178.991	10	0.38068	0.3389704	0.1366089	0.09338465	0.11969	0.1455634
1179.091	10	0.391262	0.3335519	0.1377486	0.0957845	0.11848	0.1461494
1179.191	10	0.390528	0.3314564	0.1367157	0.09526833	0.120154	0.1448299
1179.291	10	0.396459	0.3248376	0.1390497	0.09477415	0.120895	0.1454839
1179.391	10	0.388534	0.3152393	0.1390567	0.094252	0.119196	0.1444625
1179.491	10	0.389306	0.3289846	0.1352914	0.09437468	0.118509	0.1445377
1179.591	10	0.390697	0.3169774	0.1362231	0.09464709	0.121743	0.1453412
1179.691	10	0.385467	0.3324935	0.1322259	0.09861033	0.123158	0.1419265
1179.791	10	0.390414	0.3347838	0.1330407	0.09686899	0.123616	0.1418039
1179.891	10	0.394231	0.3426615	0.1320211	0.09513602	0.124685	0.1423596
1179.991	10	0.416819	0.3569395	0.131515	0.09777047	0.125182	0.1429804
1180.091	10	0.428429	0.3632073	0.132231	0.09800135	0.124936	0.1442032
1180.191	10	0.423316	0.3420916	0.1307621	0.0969851	0.125489	0.1470325
1180.291	10	0.427213	0.3380967	0.1272685	0.09814481	0.122269	0.1482677
1180.391	10	0.41572	0.3366848	0.1276872	0.09674796	0.119052	0.1470764
1180.491	10	0.402855	0.3453394	0.1270996	0.09784088	0.120077	0.1468818
1180.591	10	0.397332	0.3492012	0.1304539	0.09526613	0.12042	0.1459072
1180.691	10	0.395345	0.3482689	0.1307848	0.09429139	0.122944	0.1431355
1180.791	10	0.389985	0.3569584	0.1292852	0.09327127	0.121216	0.1436479
1180.891	10	0.390809	0.3514086	0.1324888	0.09575219	0.122609	0.1471867
1180.991	10	0.389755	0.342369	0.1318326	0.1012436	0.126255	0.1488202
1181.091	10	0.389425	0.3412874	0.1284639	0.1010484	0.128675	0.1494063
1181.191	10	0.395704	0.3431316	0.1288327	0.1019873	0.129141	0.1508524
1181.291	10	0.393591	0.3439838	0.1290346	0.104957	0.128724	0.1531844
1181.391	10	0.405393	0.3443179	0.1302159	0.1073746	0.128825	0.1507403
1181.491	10	0.390605	0.3414812	0.1313091	0.1076059	0.130701	0.1532369
1181.591	10	0.401084	0.3423217	0.1339475	0.1066032	0.129562	0.1541761
1181.691	10	0.400256	0.3406016	0.1346493	0.1053651	0.132854	0.1547582
1181.791	10	0.389756	0.3355753	0.136206	0.1049675	0.132912	0.1526359
1181.891	10	0.389569	0.3300138	0.1377369	0.1058151	0.131773	0.151692
1181.991	10	0.380273	0.32886	0.1364191	0.1070599	0.132538	0.152332
1182.091	10	0.383265	0.319615	0.1343977	0.1048717	0.129757	0.1544504
1182.191	10	0.398847	0.312753	0.1358116	0.1062464	0.129976	0.1519397
1182.291	10	0.387312	0.3376366	0.1390012	0.1018848	0.129605	0.1496835
1182.391	10	0.392154	0.3260385	0.1380469	0.1005608	0.130141	0.149167
1182.491	10	0.39541	0.3349529	0.1413449	0.09990224	0.132887	0.1483615
1182.591	10	0.384598	0.3475269	0.1421401	0.09962675	0.132257	0.149297
1182.691	10	0.383702	0.358286	0.1409109	0.09867252	0.134874	0.149865
1182.791	10	0.386156	0.3725587	0.141581	0.098155	0.134854	0.1468587
1182.891	10	0.389694	0.3713206	0.1425762	0.09582109	0.132029	0.1478993
1182.991	10	0.393751	0.3420374	0.1408554	0.09761319	0.132219	0.1476733
1183.091	10	0.38431	0.3347478	0.1421064	0.09791104	0.133274	0.1487237
1183.191	10	0.380952	0.3346065	0.1433538	0.09809469	0.131007	0.1466228
1183.291	10	0.389416	0.342654	0.1422738	0.09733616	0.129293	0.1447731
1183.391	10	0.383536	0.34818	0.1446223	0.09725206	0.130809	0.1464482
1183.491	10	0.369398	0.3470063	0.1445758	0.09815636	0.129924	0.1509792
1183.591	10	0.384756	0.3598125	0.1449916	0.09614701	0.126068	0.1500203
1183.691	10	0.392844	0.3517878	0.1430712	0.09600573	0.124311	0.1475181
1183.791	10	0.394672	0.3414474	0.1427759	0.09748786	0.124187	0.1476916
1183.891	10	0.376831	0.3403977	0.1415593	0.09720166	0.12314	0.1469845
1183.991	10	0.393778	0.3424801	0.1426266	0.09622836	0.123171	0.1468553
1184.091	10	0.391137	0.3427005	0.1406579	0.09406262	0.1235	0.1481534
1184.191	10	0.384411	0.3425035	0.142309	0.09480767	0.12467	0.1466427
1184.291	10	0.401906	0.3384501	0.1406065	0.09392565	0.125845	0.1453277
1184.391	10	0.398256	0.3403847	0.1436195	0.0970191	0.123276	0.1443185
1184.491	10	0.403015	0.3403323	0.1435111	0.09582809	0.12211	0.1445847
1184.591	10	0.389741	0.3342655	0.1426073	0.09301692	0.120317	0.1443411
1184.691	10	0.392235	0.3279266	0.1446074	0.0935134	0.117572	0.1479656
1184.791	10	0.395813	0.328454	0.1413531	0.09291727	0.116487	0.1476277
1184.891	10	0.39339	0.3158475	0.1395167	0.09167824	0.115998	0.1481767
1184.991	10	0.382327	0.3119424	0.1420759	0.09240587	0.116536	0.1477879
1185.091	10	0.390485	0.3362736	0.1433346	0.09544089	0.117213	0.1429119
1185.191	10	0.391935	0.3296131	0.1413372	0.09508647	0.117816	0.142486
1185.291	10	0.396187	0.3326159	0.1402093	0.0948056	0.116154	0.1429728

1185.391	10	0.403868	0.3488972	0.1389492	0.09484225	0.116942	0.1440736
1185.491	10	0.401605	0.3567528	0.1360905	0.09460682	0.117696	0.1418733
1185.591	10	0.394557	0.3703351	0.1374137	0.09540902	0.120965	0.1429143
1185.691	10	0.387226	0.3669533	0.1375054	0.09829137	0.122383	0.143966
1185.791	10	0.382769	0.339169	0.1384338	0.1000572	0.121825	0.1450922
1185.891	10	0.370743	0.3336152	0.1383297	0.09998791	0.121899	0.1458105
1185.991	10	0.374025	0.3336048	0.1387377	0.1013692	0.120655	0.1470423
1186.091	10	0.39551	0.3430843	0.1381404	0.09785794	0.121683	0.1466994
1186.191	10	0.392942	0.349999	0.1383351	0.09823865	0.119647	0.1470245
1186.291	10	0.397202	0.3439246	0.1378971	0.09718917	0.113602	0.1470702
1186.391	10	0.394943	0.3545617	0.1406557	0.09825102	0.112087	0.1464084
1186.491	10	0.391313	0.3503293	0.1378809	0.0997337	0.117265	0.1446724
1186.591	10	0.386377	0.3399581	0.1371204	0.1028124	0.118476	0.1436915
1186.691	10	0.391002	0.339123	0.1375372	0.1024774	0.121981	0.1419092
1186.791	10	0.384702	0.3411538	0.1353603	0.1020179	0.12558	0.1398817
1186.891	10	0.381889	0.3430419	0.1353803	0.1042935	0.124408	0.1420995
1186.991	10	0.382908	0.3434032	0.1358161	0.1044723	0.127039	0.1425095
1187.091	10	0.388291	0.3431184	0.1345793	0.1049826	0.126798	0.144185
1187.191	10	0.393965	0.3424754	0.1332323	0.1068791	0.126912	0.1463328
1187.291	10	0.392881	0.3418874	0.1313692	0.108477	0.126987	0.1487902
1187.391	10	0.400915	0.3328351	0.1279311	0.1058864	0.127454	0.1466656
1187.491	10	0.390167	0.3249402	0.1309931	0.1061431	0.127785	0.1468906
1187.591	10	0.39545	0.3291828	0.1306702	0.1063746	0.12889	0.1472836
1187.691	10	0.385695	0.3178815	0.1338228	0.1067422	0.130815	0.1476999
1187.791	10	0.378515	0.3096946	0.1325304	0.1101099	0.131124	0.148166
1187.891	10	0.387461	0.3291233	0.1348506	0.1081081	0.129362	0.1481104
1187.991	10	0.378588	0.3242759	0.1353559	0.1095171	0.131004	0.1484202
1188.091	10	0.378405	0.3304026	0.1302408	0.1071286	0.128325	0.1485232
1188.191	10	0.394605	0.3518092	0.1310848	0.1077849	0.126707	0.1490371
1188.291	10	0.387086	0.3465616	0.1330804	0.105721	0.12599	0.1519686
1188.391	10	0.388225	0.3666549	0.1342588	0.1046766	0.12737	0.1519045
1188.491	10	0.392717	0.3616998	0.1348153	0.1059593	0.130319	0.1509815
1188.591	10	0.382303	0.335977	0.1348616	0.1064529	0.130704	0.1524089
1188.691	10	0.381567	0.3322893	0.1365892	0.1076061	0.131542	0.1534504
1188.791	10	0.388824	0.3335505	0.136396	0.1079828	0.13317	0.1525785
1188.891	10	0.391953	0.3443699	0.1382085	0.1032344	0.1327	0.1539235
1188.991	10	0.395665	0.3496688	0.1387135	0.1035755	0.131794	0.1535269
1189.091	10	0.382824	0.3419304	0.1366813	0.103029	0.13055	0.1534822
1189.191	10	0.380823	0.3653043	0.1386694	0.1038336	0.130641	0.1533788
1189.291	10	0.390766	0.3515824	0.1405182	0.1052126	0.129346	0.1496638
1189.391	10	0.386251	0.341868	0.1405377	0.1023649	0.130097	0.1488379
1189.491	10	0.369103	0.3414825	0.1400626	0.100959	0.127697	0.1467223
1189.591	10	0.387986	0.3440152	0.1401599	0.1004253	0.127634	0.145519
1189.691	10	0.394299	0.3448294	0.1403838	0.09989596	0.124869	0.146097
1189.791	10	0.395183	0.3421097	0.1388475	0.1014825	0.12389	0.1401047
1189.891	10	0.380903	0.3445079	0.1415461	0.09979036	0.125683	0.1421387
1189.991	10	0.402173	0.3420581	0.1404531	0.102256	0.124001	0.1443281
1190.091	10	0.394412	0.3397647	0.1430324	0.1021347	0.124984	0.1459731
1190.191	10	0.383608	0.3328839	0.1433377	0.1024311	0.126825	0.145853
1190.291	10	0.405871	0.3264941	0.1417394	0.1012773	0.129172	0.1440838
1190.391	10	0.402164	0.3276459	0.1442629	0.1037475	0.125487	0.1452596
1190.491	10	0.404272	0.3157654	0.1445344	0.1011714	0.125044	0.1497276
1190.591	10	0.390882	0.3039072	0.1430717	0.09869998	0.124848	0.1480625
1190.691	10	0.393301	0.3199294	0.1447254	0.1002268	0.124748	0.1460013
1190.791	10	0.390862	0.3271559	0.1417131	0.09715457	0.123226	0.1451672
1190.891	10	0.391131	0.3291515	0.1405582	0.09596004	0.122408	0.1472387
1190.991	10	0.383144	0.3515133	0.1423669	0.09389392	0.121964	0.1446455
1191.091	10	0.389765	0.3512101	0.1405268	0.09346188	0.120586	0.1408406
1191.191	10	0.390761	0.3698529	0.1376107	0.09574968	0.118611	0.1426219
1191.291	10	0.395233	0.3587041	0.1362265	0.09635817	0.117534	0.1454933
1191.391	10	0.397294	0.3339558	0.1389177	0.09464987	0.118705	0.1439498
1191.491	10	0.391177	0.33295	0.1415277	0.09435617	0.121238	0.1441786
1191.591	10	0.396367	0.3372892	0.1390077	0.09385827	0.120608	0.1448782
1191.691	10	0.391293	0.3454611	0.1419948	0.09542975	0.120075	0.1463692
1191.791	10	0.382149	0.3514968	0.1410118	0.09593809	0.121652	0.1473657
1191.891	10	0.37109	0.3492858	0.1398596	0.0954605	0.123692	0.1484525
1191.991	10	0.377286	0.3607769	0.1395238	0.09538895	0.122264	0.1473608
1192.091	10	0.408244	0.353745	0.1415503	0.09272275	0.123277	0.1447062
1192.191	10	0.423715	0.3424893	0.1407788	0.09309542	0.123999	0.1440789
1192.291	10	0.426053	0.3419501	0.1410039	0.09249765	0.122884	0.1427059
1192.391	10	0.413166	0.3431498	0.1415348	0.09089679	0.120749	0.1443056
1192.491	10	0.396296	0.3458598	0.1391971	0.09210648	0.121317	0.1420181
1192.591	10	0.385817	0.3437354	0.1383878	0.09296314	0.118307	0.1446674
1192.691	10	0.392819	0.3453533	0.1357175	0.09428021	0.118958	0.1461925
1192.791	10	0.390232	0.3446295	0.1359142	0.09463831	0.120823	0.1459283
1192.891	10	0.38884	0.3403351	0.1361778	0.09562718	0.124651	0.1488746
1192.991	10	0.388001	0.3334994	0.1364848	0.09778883	0.130581	0.1498137
1193.091	10	0.392029	0.3293263	0.1368846	0.09581735	0.129938	0.1501058
1193.191	10	0.392097	0.3313181	0.1374988	0.09760071	0.130109	0.1511397
1193.291	10	0.392144	0.3167183	0.1368492	0.1015427	0.129181	0.1494535
1193.391	10	0.401594	0.3124512	0.1358193	0.1024942	0.128703	0.1477652
1193.491	10	0.390754	0.3290583	0.1374822	0.10206	0.130856	0.1455042
1193.591	10	0.402277	0.3273118	0.1356753	0.1013249	0.131592	0.1422612
1193.691	10	0.404719	0.3376071	0.1356616	0.1057154	0.131444	0.1441772
1193.791	10	0.437828	0.3638006	0.1350769	0.1069764	0.129189	0.1427707
1193.891	10	0.425748	0.3599058	0.1355056	0.1040354	0.131425	0.14288
1193.991	10	0.414783	0.3660578	0.1351292	0.1070321	0.131236	0.1447946
1194.091	10	0.386471	0.3523962	0.1336039	0.1070691	0.128907	0.1438015
1194.191	10	0.403241	0.3485864	0.1340195	0.1079627	0.127247	0.1448709
1194.291	10	0.405779	0.352449	0.1346897	0.1035942	0.126182	0.1473894
1194.391	10	0.409831	0.3584844	0.1317506	0.1053567	0.126771	0.1472136
1194.491	10	0.404239	0.366669	0.1306123	0.1041644	0.129539	0.1475242
1194.591	10	0.394232	0.3620349	0.129769	0.1048999	0.131569	0.1479175
1194.691	10	0.393652	0.3559338	0.1309191	0.1061796	0.130759	0.1458615
1194.791	10	0.393493	0.3610123	0.1323264	0.1069989	0.128836	0.1427271

1194.891	10	0.398075	0.3542555	0.1307704	0.1037612	0.125437	0.143113
1194.991	10	0.392208	0.3567455	0.1340791	0.1051742	0.127116	0.1456834
1195.091	10	0.382174	0.3647124	0.1328139	0.1010716	0.127744	0.1473684
1195.191	10	0.383893	0.3722029	0.1293882	0.1006509	0.129793	0.1465106
1195.291	10	0.393804	0.3768734	0.1311076	0.1034948	0.130351	0.1470109
1195.391	10	0.388151	0.3752182	0.1337312	0.1017674	0.131222	0.1493239
1195.491	10	0.370254	0.3752275	0.1343943	0.1017629	0.12966	0.1480569
1195.591	10	0.388125	0.3711083	0.1358448	0.1023009	0.127838	0.1497475
1195.691	10	0.393315	0.3645893	0.1386772	0.1017114	0.126748	0.1527769
1195.791	10	0.393602	0.3581572	0.1394671	0.1000436	0.125104	0.154799
1195.891	10	0.376301	0.349215	0.1376625	0.09914479	0.125423	0.1548306
1195.991	10	0.392393	0.3408451	0.1368733	0.09877455	0.124529	0.1555005
1196.091	10	0.384611	0.3289149	0.1368694	0.0995312	0.126831	0.1541753
1196.191	10	0.374655	0.3189976	0.1366733	0.1002041	0.127664	0.1545976
1196.291	10	0.399829	0.3275635	0.1393078	0.09733767	0.128817	0.1509767
1196.391	10	0.396663	0.3214639	0.1438832	0.09829683	0.128076	0.1489548
1196.491	10	0.406172	0.3311778	0.1427184	0.09566749	0.126959	0.1487689
1196.591	10	0.390259	0.3468133	0.1422236	0.09563266	0.124465	0.1499903
1196.691	10	0.389966	0.3632629	0.1420539	0.09757581	0.122675	0.1504397
1196.791	10	0.389305	0.3663598	0.1425973	0.09306231	0.119925	0.1497254
1196.891	10	0.389245	0.3495037	0.1419081	0.094791	0.118662	0.1456192
1196.991	10	0.380894	0.3429093	0.1453092	0.09354735	0.116901	0.1472242
1197.091	10	0.388899	0.344053	0.1439374	0.09349333	0.114914	0.1483217
1197.191	10	0.387994	0.3488367	0.145604	0.09691051	0.114516	0.1493699
1197.291	10	0.398359	0.3592441	0.1458479	0.09722775	0.115824	0.1475276
1197.391	10	0.394401	0.3527857	0.1438639	0.09539158	0.115919	0.1458041
1197.491	10	0.388419	0.3550151	0.1450369	0.09709503	0.117638	0.1476738
1197.591	10	0.399902	0.3527761	0.1458829	0.09645554	0.117429	0.1498127
1197.691	10	0.41174	0.3422121	0.1465989	0.09789058	0.118131	0.1488479
1197.791	10	0.393008	0.3412944	0.1443584	0.09858295	0.117242	0.1474052
1197.891	10	0.401381	0.3449292	0.144362	0.09933072	0.120676	0.1473451
1197.991	10	0.404308	0.3471856	0.1425009	0.09757289	0.119934	0.145994
1198.091	10	0.403563	0.3459627	0.141809	0.09603094	0.11856	0.1457639
1198.191	10	0.403222	0.3455993	0.1375915	0.09776988	0.118245	0.1472613
1198.291	10	0.407714	0.3471226	0.1369917	0.09872788	0.11738	0.145992
1198.391	10	0.379762	0.3451047	0.1353058	0.09821637	0.118945	0.1455972
1198.491	10	0.386009	0.3399327	0.1401969	0.09910657	0.124426	0.1432071
1198.591	10	0.385633	0.3348991	0.1404019	0.09834637	0.122794	0.1461021
1198.691	10	0.376353	0.3325419	0.1376798	0.09974223	0.123435	0.1445017
1198.791	10	0.383205	0.3343909	0.1389456	0.09854771	0.12601	0.1474
1198.891	10	0.3805	0.325977	0.1379561	0.09723822	0.125955	0.148225
1198.991	10	0.388514	0.3186122	0.1381316	0.1002657	0.130039	0.1478838
1199.115	10.5	0.387395	0.3397368	0.139782	0.1003174	0.130113	0.1493822
1199.215	10.5	0.39391	0.3502397	0.1404988	0.09882274	0.130687	0.1457072
1199.315	10.5	0.389051	0.3707668	0.1399541	0.1004964	0.128493	0.146479
1199.415	10.5	0.390983	0.367704	0.1389284	0.1027783	0.131953	0.1454771
1199.515	10.5	0.388638	0.342278	0.1378626	0.1052806	0.131204	0.1431831
1199.615	10.5	0.388311	0.3373315	0.1349489	0.1029809	0.133122	0.1431752
1199.715	10.5	0.379596	0.3418647	0.1328373	0.1009636	0.134903	0.1442439
1199.815	10.5	0.388477	0.3474772	0.132262	0.103275	0.135121	0.1447697
1199.915	10.5	0.389608	0.3519055	0.1298116	0.101593	0.134656	0.1457126
1200.015	10.5	0.38908	0.3510317	0.1296849	0.1010147	0.132397	0.145881
1200.115	10.5	0.383576	0.3591663	0.132449	0.09891789	0.129493	0.1478742
1200.215	10.5	0.388162	0.3452565	0.1321054	0.09808129	0.127271	0.1482423
1200.315	10.5	0.376331	0.3389896	0.1311036	0.09912141	0.128187	0.148437
1200.415	10.5	0.382512	0.3409561	0.1314617	0.099886	0.127114	0.1467926
1200.515	10.5	0.386321	0.3418468	0.1316812	0.1000777	0.128686	0.1458458
1200.615	10.5	0.390052	0.3446853	0.1292735	0.0990122	0.128963	0.1465378
1200.715	10.5	0.389122	0.3436459	0.1310444	0.09998074	0.129526	0.1449221
1200.815	10.5	0.380461	0.343201	0.1302308	0.09907987	0.129654	0.1447101
1200.915	10.5	0.383348	0.3418977	0.1314806	0.09661693	0.127426	0.1416233
1201.015	10.5	0.392182	0.3394737	0.1315589	0.09551255	0.127274	0.1443484
1201.115	10.5	0.388259	0.3331306	0.1271454	0.09736678	0.126093	0.1468974
1201.215	10.5	0.373327	0.3283323	0.1270923	0.09739397	0.128082	0.1452264
1201.315	10.5	0.390577	0.3332771	0.1289631	0.09706041	0.129366	0.1439969
1201.415	10.5	0.393606	0.325112	0.1333846	0.09565061	0.131462	0.1457646
1201.515	10.5	0.385291	0.3160172	0.1299734	0.09498593	0.131484	0.1412473
1201.615	10.5	0.391108	0.3329695	0.1271344	0.09414808	0.131148	0.1426916
1201.715	10.5	0.387706	0.3253977	0.1312234	0.09679026	0.128679	0.1441184
1201.815	10.5	0.384141	0.3264962	0.1318439	0.0979743	0.126524	0.1459039
1201.915	10.5	0.397007	0.3423399	0.1302088	0.09822401	0.128752	0.1420565
1202.015	10.5	0.398608	0.3610932	0.13151	0.0972079	0.128245	0.1423547
1202.115	10.5	0.406206	0.3633432	0.1323916	0.09741071	0.128096	0.1429262
1202.215	10.5	0.390345	0.3482382	0.1326809	0.09587564	0.12637	0.14336
1202.315	10.5	0.393249	0.338036	0.1326247	0.09684297	0.128011	0.1449025
1202.415	10.5	0.393327	0.3374779	0.1327541	0.09605752	0.127208	0.1479138
1202.515	10.5	0.391868	0.3402194	0.1313107	0.09518459	0.124988	0.1512269
1202.615	10.5	0.384543	0.3485456	0.133206	0.09368761	0.123337	0.1508978
1202.715	10.5	0.387149	0.3469353	0.1344869	0.09248232	0.121671	0.1510472
1202.815	10.5	0.385423	0.3490988	0.1328359	0.09352203	0.121816	0.1528016
1202.915	10.5	0.396509	0.347504	0.1336082	0.09726503	0.12237	0.1532512
1203.015	10.5	0.392385	0.340833	0.1353852	0.09640676	0.123518	0.1548099
1203.115	10.5	0.395635	0.3408867	0.1357871	0.09697898	0.120553	0.152972
1203.215	10.5	0.396236	0.3426531	0.1357073	0.09491611	0.120253	0.1529576
1203.315	10.5	0.397666	0.3442163	0.137252	0.09551205	0.119897	0.1539651
1203.415	10.5	0.377406	0.3461004	0.1379344	0.09597873	0.120783	0.1515836
1203.515	10.5	0.391756	0.3424156	0.1363846	0.09563157	0.118372	0.1509596
1203.615	10.5	0.385179	0.3438242	0.1383453	0.0965962	0.118339	0.1506664
1203.715	10.5	0.392226	0.3410122	0.1393516	0.09407788	0.120512	0.154321
1203.815	10.5	0.402183	0.3337182	0.1413283	0.09596332	0.12088	0.154641
1203.915	10.5	0.401317	0.328156	0.1442477	0.09498692	0.12477	0.1501078
1204.015	10.5	0.395365	0.3294308	0.1465576	0.09405998	0.123682	0.1506904
1204.115	10.5	0.38141	0.326343	0.1461456	0.09765067	0.121703	0.1512734
1204.215	10.5	0.386127	0.3191062	0.1438699	0.09792463	0.118081	0.1524514
1204.315	10.5	0.384675	0.3261152	0.1438401	0.09608717	0.116156	0.1527774

1204.415	10.5	0.380652	0.3167328	0.1408734	0.09467828	0.114443	0.1499302
1204.515	10.5	0.385162	0.3379884	0.1378451	0.09663333	0.118568	0.1514183
1204.615	10.5	0.384161	0.3411953	0.1384326	0.09833205	0.119974	0.1557145
1204.715	10.5	0.384814	0.3585368	0.1360679	0.09844139	0.123006	0.1572241
1204.815	10.5	0.388458	0.3605023	0.1376152	0.1011435	0.124938	0.1543685
1204.915	10.5	0.391799	0.360186	0.1359885	0.1047735	0.127701	0.1527439
1205.015	10.5	0.387671	0.3404225	0.1382908	0.1032913	0.130061	0.1525752
1205.115	10.5	0.402884	0.3376423	0.1367678	0.1006218	0.130339	0.1515653
1205.215	10.5	0.411472	0.340231	0.1339539	0.1004994	0.130006	0.1481736
1205.315	10.5	0.407464	0.3453912	0.1357313	0.1014159	0.129164	0.1477505
1205.415	10.5	0.383785	0.3495408	0.1346919	0.1015474	0.131427	0.1491436
1205.515	10.5	0.396741	0.3479263	0.13528	0.1038801	0.129622	0.1483445
1205.615	10.5	0.389756	0.3563206	0.1393292	0.1038413	0.130917	0.1470118
1205.715	10.5	0.402587	0.3399846	0.137608	0.1065161	0.130011	0.1470103
1205.815	10.5	0.416687	0.337963	0.1377651	0.1025472	0.132498	0.1490004
1205.915	10.5	0.413825	0.3375103	0.1359965	0.101836	0.131564	0.1508999
1206.015	10.5	0.403692	0.3402236	0.1347962	0.1006003	0.131588	0.1508369
1206.115	10.5	0.393567	0.3424849	0.1350072	0.09980197	0.12986	0.1517315
1206.215	10.5	0.403618	0.3404329	0.1350704	0.1002808	0.130068	0.1503051
1206.315	10.5	0.396947	0.3429799	0.133721	0.1001262	0.128847	0.1483324
1206.415	10.5	0.396727	0.3413973	0.1339058	0.09974527	0.13143	0.148798
1206.515	10.5	0.38805	0.3356479	0.1344943	0.1004591	0.13229	0.1472365
1206.615	10.5	0.380894	0.3318112	0.133428	0.1002128	0.133082	0.1476794
1206.715	10.5	0.389735	0.3323585	0.1317517	0.09948175	0.13231	0.1491288
1206.815	10.5	0.386795	0.3301484	0.1325513	0.1014486	0.133486	0.1503556
1206.915	10.5	0.366602	0.3209825	0.1345084	0.1034217	0.132408	0.1516884
1207.015	10.5	0.38122	0.3219734	0.1345471	0.1036204	0.133417	0.1510482
1207.115	10.5	0.388507	0.3231511	0.1345101	0.1012241	0.130575	0.1499965
1207.215	10.5	0.386913	0.3354222	0.1346757	0.09923235	0.130718	0.1502143
1207.315	10.5	0.38697	0.3296922	0.1339481	0.09966468	0.132078	0.1488214
1207.415	10.5	0.389945	0.3532432	0.1336322	0.100728	0.133148	0.1459779
1207.515	10.5	0.384047	0.371926	0.1335491	0.09916533	0.132756	0.1467864
1207.615	10.5	0.390068	0.3677669	0.1336816	0.1001029	0.132948	0.1462789
1207.715	10.5	0.39983	0.3443428	0.132405	0.09989204	0.130281	0.1461973
1207.815	10.5	0.404931	0.3360962	0.1269524	0.1024317	0.13102	0.1460303
1207.915	10.5	0.393756	0.3378096	0.1269499	0.1009852	0.130528	0.1431892
1208.015	10.5	0.38802	0.3464976	0.1257856	0.1005402	0.12914	0.1430268
1208.115	10.5	0.39772	0.3505242	0.1273379	0.1018624	0.128279	0.1467958
1208.215	10.5	0.38766	0.3481249	0.1252985	0.09838179	0.126248	0.1448649
1208.315	10.5	0.381314	0.3592853	0.1278099	0.09694294	0.127627	0.1434687
1208.415	10.5	0.384577	0.3413063	0.1312797	0.09851144	0.126882	0.1447286
1208.515	10.5	0.388383	0.3409636	0.1267728	0.1002122	0.12656	0.1454935
1208.615	10.5	0.395819	0.3421486	0.1291596	0.1002931	0.124068	0.1431817
1208.715	10.5	0.390078	0.34581	0.1312136	0.09784754	0.122729	0.1444661
1208.815	10.5	0.379312	0.3482674	0.1294288	0.09799635	0.119283	0.1455425
1208.915	10.5	0.386843	0.3447953	0.1301832	0.09663984	0.11564	0.1456444
1209.015	10.5	0.380626	0.3456058	0.1319194	0.09830919	0.115019	0.1442515
1209.115	10.5	0.370222	0.3430136	0.1306266	0.09710617	0.115412	0.1462417
1209.215	10.5	0.38634	0.3404528	0.1295242	0.1004867	0.115081	0.1485603
1209.315	10.5	0.373698	0.3375283	0.1299236	0.1013739	0.113199	0.1496279
1209.415	10.5	0.379124	0.3368473	0.1323761	0.100861	0.112827	0.1494018
1209.515	10.5	0.419704	0.3375947	0.1316415	0.1010084	0.110361	0.1505124
1209.615	10.5	0.42799	0.3251212	0.134293	0.1018356	0.112703	0.1471912
1209.715	10.5	0.420364	0.3282664	0.1353853	0.1025719	0.114813	0.1469703
1209.815	10.5	0.382032	0.3252596	0.1325325	0.10279	0.116955	0.1497356
1209.915	10.5	0.382146	0.3192878	0.1316836	0.1017977	0.118175	0.1504685
1210.015	10.5	0.386797	0.3200579	0.1346321	0.09991153	0.11866	0.1502857
1210.115	10.5	0.386659	0.3501828	0.1357326	0.09844495	0.120065	0.1536672
1210.215	10.5	0.388845	0.3721375	0.1414042	0.09664157	0.122744	0.1524242
1210.315	10.5	0.393291	0.3660624	0.138966	0.09872023	0.120861	0.154348
1210.415	10.5	0.446506	0.350617	0.1412765	0.09967667	0.120051	0.1543057
1210.515	10.5	0.39616	0.3393237	0.142563	0.1015492	0.123451	0.1527912
1210.615	10.5	0.399566	0.338855	0.1432011	0.1038365	0.123265	0.1513298
1210.715	10.5	0.387711	0.3450758	0.1433518	0.108308	0.123587	0.1518133
1210.815	10.5	0.394678	0.3526433	0.1413047	0.1065591	0.127259	0.1507077
1210.915	10.5	0.387592	0.34888	0.1409835	0.1052299	0.125846	0.1527809
1211.015	10.5	0.38356	0.3594584	0.1396624	0.1044676	0.126354	0.1481028
1211.115	10.5	0.378653	0.3487532	0.138055	0.1039876	0.125977	0.1493518
1211.215	10.5	0.385872	0.342938	0.1375642	0.1068397	0.126989	0.1488045
1211.315	10.5	0.382123	0.342342	0.1388064	0.1062973	0.126145	0.1514579
1211.415	10.5	0.39596	0.3470125	0.1353223	0.107522	0.128556	0.1521051
1211.515	10.5	0.386318	0.3490034	0.1354315	0.1043732	0.129252	0.1474827
1211.615	10.5	0.386426	0.3506264	0.1370398	0.1028558	0.131274	0.147326
1211.715	10.5	0.380268	0.3509883	0.138947	0.1035678	0.134439	0.1494605
1211.815	10.5	0.381468	0.3491793	0.1339755	0.1019347	0.13305	0.1492932
1211.915	10.5	0.390644	0.3434274	0.1331125	0.1024624	0.132545	0.1498635
1212.015	10.5	0.385616	0.3381551	0.13275	0.1012264	0.132092	0.1495744
1212.115	10.5	0.393466	0.3327228	0.131645	0.09917307	0.131321	0.1505459
1212.215	10.5	0.392799	0.3331808	0.1343981	0.09830672	0.130616	0.1492033
1212.315	10.5	0.381372	0.3197679	0.1362392	0.09660152	0.130518	0.1478101
1212.415	10.5	0.39688	0.3335742	0.1352796	0.09415435	0.12888	0.1495389
1212.515	10.5	0.391629	0.341149	0.1349233	0.09490223	0.128791	0.1497759
1212.615	10.5	0.377172	0.3187019	0.1344483	0.09515736	0.129474	0.1487801
1212.715	10.5	0.387834	0.3370307	0.1333325	0.09577259	0.131862	0.1497294
1212.815	10.5	0.394736	0.3453432	0.1351876	0.09492182	0.13067	0.147977
1212.915	10.5	0.394166	0.3676481	0.134617	0.09527206	0.127593	0.1478836
1213.015	10.5	0.382459	0.3601403	0.1310618	0.09643891	0.129655	0.1491421
1213.115	10.5	0.390642	0.3565647	0.1317747	0.09710049	0.129877	0.1488928
1213.215	10.5	0.384215	0.3453377	0.1329368	0.09640571	0.128486	0.1498377
1213.315	10.5	0.386501	0.3463828	0.1315687	0.09775895	0.129874	0.1472719
1213.415	10.5	0.405113	0.3523642	0.1309372	0.09854758	0.131146	0.1445661
1213.515	10.5	0.403192	0.3593778	0.1349011	0.1015421	0.131176	0.1430346
1213.615	10.5	0.390124	0.353739	0.1353695	0.1004387	0.130139	0.144899
1213.715	10.5	0.396612	0.3553296	0.1351027	0.09900864	0.129035	0.144008
1213.815	10.5	0.394495	0.3543786	0.1352156	0.09792785	0.128828	0.1453603

1213.915	10.5	0.386767	0.3469708	0.1324085	0.09556273	0.128532	0.1458098
1214.015	10.5	0.386357	0.3473498	0.1327308	0.09321862	0.125985	0.1464375
1214.115	10.5	0.393346	0.352709	0.1329549	0.09437328	0.126912	0.1473756
1214.215	10.5	0.394452	0.3566758	0.1342963	0.09686816	0.127218	0.1492485
1214.315	10.5	0.39681	0.3600405	0.1369108	0.09943901	0.128148	0.147916
1214.415	10.5	0.392374	0.3582239	0.1346891	0.0971212	0.125044	0.1471708
1214.515	10.5	0.394556	0.3606443	0.1306204	0.09699596	0.125783	0.142865
1214.615	10.5	0.413475	0.3539937	0.1284894	0.09423652	0.124091	0.1449729
1214.715	10.5	0.410828	0.3448699	0.1303778	0.09567922	0.122311	0.1443254
1214.815	10.5	0.38672	0.3366429	0.1332435	0.09710241	0.121111	0.1433184
1214.915	10.5	0.383766	0.3343672	0.1289656	0.09793014	0.117574	0.1447712
1215.015	10.5	0.393907	0.3365259	0.1298117	0.0971044	0.116715	0.1426244
1215.115	10.5	0.395478	0.3304687	0.1295302	0.09662759	0.11374	0.1469421
1215.215	10.5	0.428718	0.340499	0.1266382	0.09910289	0.116222	0.1453607
1215.315	10.5	0.43444	0.3210872	0.1303862	0.09948612	0.116741	0.1438715
1215.415	10.5	0.426269	0.3217926	0.133008	0.09843722	0.120531	0.1450528
1215.515	10.5	0.403739	0.3277745	0.1299783	0.09959238	0.115026	0.14595
1215.615	10.5	0.392328	0.3527813	0.1298176	0.09984788	0.116505	0.1456187
1215.715	10.5	0.394077	0.3558581	0.1323236	0.09900194	0.115102	0.1460224
1215.815	10.5	0.388111	0.3646806	0.1293939	0.09791763	0.114198	0.1445913
1215.915	10.5	0.396004	0.3474914	0.1310781	0.09777868	0.114757	0.1444661
1216.015	10.5	0.408152	0.3435341	0.1301297	0.1006562	0.113124	0.144491
1216.115	10.5	0.450666	0.3499113	0.1289007	0.09889132	0.113621	0.1430523
1216.215	10.5	0.40616	0.3555539	0.1309739	0.1015209	0.115752	0.146936
1216.315	10.5	0.418654	0.3582965	0.1343085	0.1014586	0.111241	0.1497046
1216.415	10.5	0.390873	0.3489076	0.1331329	0.1041697	0.112449	0.1498369
1216.515	10.5	0.398448	0.3570225	0.1341076	0.1017545	0.114759	0.1496961
1216.615	10.5	0.390828	0.3402574	0.1359674	0.100824	0.115097	0.1526308
1216.715	10.5	0.389937	0.3410727	0.1361675	0.1015214	0.118948	0.1484701
1216.815	10.5	0.381355	0.3447546	0.1366939	0.1001236	0.120625	0.1479915
1216.915	10.5	0.387102	0.3490619	0.1405104	0.1022244	0.120772	0.1490253
1217.015	10.5	0.380873	0.3516967	0.1401455	0.1053166	0.125999	0.1487642
1217.115	10.5	0.402817	0.3487313	0.1427838	0.1068924	0.123902	0.1480553
1217.215	10.5	0.387901	0.3504123	0.1458133	0.1025945	0.126076	0.1496459
1217.315	10.5	0.40623	0.3477172	0.1464307	0.1021994	0.126953	0.1508536
1217.415	10.5	0.400748	0.3393805	0.1449738	0.1033005	0.128388	0.1541952
1217.515	10.5	0.400006	0.3316571	0.1405641	0.103144	0.128389	0.1517096
1217.615	10.5	0.413126	0.328959	0.142292	0.1033912	0.128601	0.1507818
1217.715	10.5	0.400166	0.3327626	0.137869	0.1018369	0.133406	0.1502416
1217.815	10.5	0.389647	0.3268256	0.1360824	0.09874979	0.133849	0.1517553
1217.915	10.5	0.400789	0.3419224	0.1358073	0.09771926	0.134514	0.1498052
1218.015	10.5	0.387463	0.3209528	0.1339428	0.09782828	0.132445	0.1481886
1218.115	10.5	0.388564	0.3216749	0.1333427	0.09674899	0.12982	0.1460596
1218.215	10.5	0.379307	0.3290716	0.1338094	0.0955965	0.130124	0.1440582
1218.315	10.5	0.366041	0.3504135	0.1373273	0.09455208	0.128154	0.1456486
1218.415	10.5	0.381344	0.3593981	0.1396322	0.0942202	0.126687	0.145985
1218.515	10.5	0.387156	0.3609041	0.1353464	0.09469105	0.129685	0.1442141
1218.615	10.5	0.391895	0.3495467	0.1369242	0.09404597	0.131824	0.1428719
1218.715	10.5	0.378704	0.3409991	0.1334769	0.09630514	0.134966	0.1441533
1218.815	10.5	0.38241	0.3456547	0.1327565	0.09608535	0.13235	0.1472402
1218.915	10.5	0.384999	0.352128	0.1357499	0.09683816	0.127364	0.1460368
1219.015	10.5	0.38868	0.3561822	0.1370257	0.0971033	0.128321	0.1451233
1219.115	10.5	0.433391	0.3498222	0.1368092	0.09994189	0.129612	0.1463019
1219.215	10.5	0.422944	0.3540606	0.136834	0.103544	0.127764	0.1446223
1219.315	10.5	0.381209	0.3408874	0.1384804	0.1013333	0.128671	0.1415162
1219.415	10.5	0.4033	0.3378277	0.1356407	0.09837849	0.130591	0.144305
1219.515	10.5	0.396852	0.3397778	0.1350818	0.0974545	0.129159	0.1465339
1219.615	10.5	0.386898	0.3423423	0.1330377	0.09456006	0.126706	0.1451476
1219.715	10.5	0.382986	0.3461414	0.1328714	0.09385289	0.126015	0.1431444
1219.815	10.5	0.402371	0.3452254	0.1317933	0.09328125	0.125817	0.1429411
1219.915	10.5	0.401015	0.3469066	0.1324194	0.09391402	0.128927	0.1430225
1220.015	10.5	0.397861	0.3447969	0.1335137	0.09538526	0.126506	0.1430394
1220.115	10.5	0.392812	0.339557	0.1341324	0.0928515	0.125046	0.1436588
1220.215	10.5	0.38863	0.3328128	0.1343117	0.09349661	0.124805	0.1432547
1220.315	10.5	0.39541	0.3273896	0.1327209	0.09513053	0.123698	0.1447269
1220.415	10.5	0.403583	0.3279915	0.1342436	0.09727018	0.118824	0.1425958
1220.515	10.5	0.383249	0.3255334	0.1329139	0.09474944	0.116923	0.1421582
1220.615	10.5	0.37815	0.3338776	0.1304969	0.09589332	0.114108	0.1432177
1220.715	10.5	0.392587	0.3275993	0.1320702	0.09522255	0.1109	0.1429371
1220.815	10.5	0.394919	0.3171846	0.130339	0.09283328	0.112341	0.1440682
1220.915	10.5	0.405344	0.3292851	0.1310945	0.09327734	0.116143	0.1455742
1221.015	10.5	0.411428	0.3503093	0.1324629	0.09446283	0.1166	0.1451844
1221.115	10.5	0.407583	0.3609813	0.127058	0.09363946	0.11323	0.1431895
1221.215	10.5	0.391776	0.362239	0.1250476	0.09858987	0.115246	0.1433439
1221.315	10.5	0.382589	0.3459812	0.1241776	0.09984163	0.116625	0.1430417
1221.415	10.5	0.385077	0.334113	0.1272394	0.1004328	0.117511	0.1464651
1221.515	10.5	0.38682	0.3364121	0.1273864	0.1024442	0.112207	0.1449537
1221.615	10.5	0.387054	0.3406385	0.1270811	0.1021047	0.113238	0.1435578
1221.715	10.5	0.393511	0.3486409	0.1312362	0.1036901	0.112992	0.143997
1221.815	10.5	0.427405	0.3452715	0.1280678	0.1030043	0.114944	0.1415762
1221.915	10.5	0.41697	0.3499088	0.1264868	0.1048377	0.117465	0.1460975
1222.015	10.5	0.413655	0.3474036	0.1266985	0.1043945	0.117799	0.1441819
1222.115	10.5	0.393784	0.3388655	0.1292964	0.1035497	0.118079	0.1438661
1222.215	10.5	0.390417	0.3388551	0.1281488	0.1040915	0.118	0.1446177
1222.315	10.5	0.384507	0.3401977	0.1285688	0.1048997	0.113816	0.1426282
1222.415	10.5	0.373562	0.3419848	0.1277941	0.1052502	0.114006	0.1424278
1222.515	10.5	0.379024	0.3417505	0.1279095	0.1050289	0.116637	0.141769
1222.615	10.5	0.385191	0.3412815	0.1294973	0.1060105	0.116486	0.1414209
1222.715	10.5	0.386103	0.341831	0.1299771	0.1083255	0.119078	0.1405395
1222.815	10.5	0.393642	0.3397008	0.1293458	0.1076681	0.122626	0.1407849
1222.915	10.5	0.387268	0.3333539	0.1308576	0.1053378	0.124118	0.1433769
1223.015	10.5	0.40469	0.3292817	0.136533	0.1045348	0.126466	0.1450151
1223.115	10.5	0.394588	0.32742	0.1354854	0.1051907	0.127473	0.1456423
1223.215	10.5	0.392062	0.3234381	0.1362137	0.1027823	0.130256	0.1436329
1223.315	10.5	0.389222	0.3255764	0.1392856	0.1027292	0.130574	0.145362

1223.415	10.5	0.402329	0.3320726	0.1380752	0.102747	0.131053	0.1493874
1223.515	10.5	0.390534	0.3128625	0.1381122	0.1009341	0.130596	0.1519024
1223.615	10.5	0.400308	0.3311646	0.1405597	0.1019119	0.13099	0.1522871
1223.715	10.5	0.391869	0.3406777	0.1403166	0.1010309	0.132304	0.1505556
1223.815	10.5	0.384298	0.3492612	0.142744	0.1000988	0.132092	0.1497101
1223.915	10.5	0.378264	0.355413	0.1435547	0.09942668	0.130187	0.1485768
1224.015	10.5	0.368827	0.3544779	0.144794	0.09738345	0.130143	0.1515955
1224.115	10.5	0.381975	0.3376046	0.1448458	0.09510939	0.127306	0.1494235
1224.215	10.5	0.384461	0.3333238	0.1440558	0.0965476	0.12666	0.1505187
1224.315	10.5	0.392962	0.3392467	0.1434522	0.09429628	0.125617	0.1484585
1224.415	10.5	0.381091	0.3451271	0.1418684	0.09421846	0.124394	0.1485747
1224.515	10.5	0.384676	0.3477926	0.1377534	0.09332187	0.126944	0.1481121
1224.615	10.5	0.380101	0.3460344	0.1399187	0.09764096	0.127579	0.1460039
1224.715	10.5	0.372245	0.3513259	0.1371425	0.09768457	0.127791	0.1443814
1224.815	10.5	0.401316	0.3406523	0.1364781	0.09578906	0.131118	0.1438187
1224.915	10.5	0.399738	0.3377821	0.136409	0.09695083	0.13005	0.1443647
1225.015	10.5	0.392257	0.3388541	0.1387378	0.0974454	0.126883	0.1445442
1225.115	10.5	0.388955	0.3416368	0.1360451	0.09622252	0.127615	0.1414195
1225.215	10.5	0.388796	0.3422665	0.1333209	0.09689445	0.126216	0.1413279
1225.315	10.5	0.382995	0.3401273	0.1336955	0.09269446	0.125591	0.1438048
1225.415	10.5	0.384965	0.3418918	0.1326861	0.09041201	0.128379	0.1426731
1225.515	10.5	0.389677	0.3415673	0.1322983	0.08978059	0.128555	0.1434851
1225.615	10.5	0.395503	0.3366536	0.1367875	0.09223716	0.12988	0.1416946
1225.715	10.5	0.39184	0.3305522	0.139482	0.09465344	0.128912	0.1440949
1225.815	10.5	0.399276	0.3294028	0.1379784	0.09324767	0.125344	0.14328
1225.915	10.5	0.393977	0.3273263	0.1367014	0.09355691	0.122607	0.1436473
1226.015	10.5	0.396319	0.3175963	0.1366542	0.09343765	0.122576	0.1453082
1226.115	10.5	0.403066	0.3316906	0.1358201	0.09560974	0.125243	0.1475647
1226.215	10.5	0.395554	0.316154	0.137202	0.09676851	0.124695	0.1441134
1226.315	10.5	0.383094	0.3160159	0.1351916	0.1008651	0.124108	0.1451876
1226.415	10.5	0.384079	0.3309581	0.1347159	0.1010103	0.123157	0.1448092
1226.515	10.5	0.386004	0.3495258	0.1327264	0.09937567	0.122685	0.1467919
1226.615	10.5	0.406327	0.352741	0.132702	0.09982868	0.121256	0.1463576
1226.715	10.5	0.416455	0.3591301	0.1334256	0.09986801	0.122667	0.149737
1226.815	10.5	0.409647	0.3432332	0.1329581	0.09855705	0.121696	0.1496043
1226.915	10.5	0.392963	0.33382	0.1350337	0.09758603	0.120059	0.1473581
1227.015	10.5	0.380252	0.3388105	0.1336619	0.09680308	0.121341	0.1470556
1227.115	10.5	0.3844	0.3449323	0.1345634	0.09642554	0.120349	0.1459636
1227.215	10.5	0.384406	0.3508502	0.1339024	0.09627996	0.119912	0.1501051
1227.315	10.5	0.381564	0.3463022	0.1302156	0.09637573	0.119395	0.1497286
1227.415	10.5	0.394788	0.3556983	0.1306972	0.09742973	0.119136	0.1495375
1227.515	10.5	0.404894	0.3420976	0.1317131	0.09723507	0.117857	0.1496342
1227.615	10.5	0.428224	0.3393251	0.1345602	0.09877805	0.11807	0.1485524
1227.715	10.5	0.406429	0.3392668	0.134385	0.1007645	0.117529	0.1490388
1227.815	10.5	0.402344	0.3433444	0.1265972	0.1010892	0.11766	0.147291
1227.915	10.5	0.391887	0.3457206	0.1272629	0.0996487	0.117146	0.1458916
1228.015	10.5	0.379608	0.3410695	0.1259405	0.1019077	0.117258	0.1455698
1228.115	10.5	0.378277	0.3423242	0.1315167	0.1035202	0.116199	0.1437818
1228.215	10.5	0.376817	0.3417685	0.130696	0.1035948	0.115353	0.1425036
1228.315	10.5	0.386879	0.3390033	0.1302631	0.1022048	0.112161	0.1437685
1228.415	10.5	0.387204	0.3323596	0.1353002	0.1033015	0.112509	0.1410804
1228.515	10.5	0.396188	0.3288983	0.1304687	0.1030336	0.114946	0.14276
1228.615	10.5	0.388837	0.3273274	0.1303401	0.09877752	0.115707	0.1454602
1228.715	10.5	0.406526	0.3194861	0.1311483	0.0978735	0.120023	0.1432673
1228.815	10.5	0.431847	0.3307988	0.13343	0.09993331	0.122968	0.1439342
1228.915	10.5	0.437961	0.3198417	0.1318104	0.09779877	0.124237	0.1459055
1229.015	10.5	0.429055	0.31324	0.1335311	0.09536095	0.129058	0.1459989
1229.115	10.5	0.417387	0.3272307	0.133421	0.09421264	0.127215	0.1462952
1229.215	10.5	0.412763	0.3490447	0.133489	0.09321296	0.125607	0.1449294
1229.315	10.5	0.408718	0.3593922	0.1325694	0.09879777	0.124565	0.1425333
1229.415	10.5	0.4057	0.3730014	0.1327287	0.09764808	0.129442	0.1411654
1229.515	10.5	0.406117	0.3465157	0.130226	0.09930127	0.130401	0.1420851
1229.615	10.5	0.408264	0.3315569	0.1347638	0.09717475	0.131171	0.1427575
1229.715	10.5	0.397742	0.3353773	0.1360751	0.09439885	0.13104	0.1436949
1229.815	10.5	0.396778	0.3411422	0.1338529	0.09335557	0.13143	0.1457715
1229.915	10.5	0.40162	0.3481514	0.1327586	0.09500108	0.132438	0.1461318
1230.015	10.5	0.41169	0.3471087	0.1359574	0.09509686	0.125848	0.1441251
1230.115	10.5	0.392293	0.3576854	0.1355123	0.09540966	0.126155	0.1472502
1230.215	10.5	0.396872	0.3495899	0.1368111	0.09425697	0.12568	0.1520383
1230.315	10.5	0.385277	0.3445263	0.1350335	0.09519745	0.129872	0.1517454
1230.415	10.5	0.372824	0.3403392	0.1361202	0.09361015	0.131315	0.1509221
1230.515	10.5	0.405	0.3425477	0.1378237	0.09663951	0.133809	0.1519139
1230.615	10.5	0.399355	0.3446969	0.1374412	0.0958228	0.132305	0.1487959
1230.715	10.5	0.396217	0.341417	0.1381838	0.09765135	0.129792	0.1512507
1230.815	10.5	0.387535	0.3409011	0.1402042	0.09649504	0.127398	0.1480496
1230.915	10.5	0.3904	0.3406891	0.1404915	0.09722626	0.129809	0.149292
1231.015	10.5	0.385801	0.3376113	0.1385927	0.09719862	0.127022	0.1483635
1231.115	10.5	0.384868	0.3330851	0.1374526	0.09592627	0.12628	0.1469625
1231.215	10.5	0.38948	0.3273829	0.1367754	0.09306449	0.129458	0.1479228
1231.315	10.5	0.396865	0.3289951	0.1356356	0.09549838	0.130389	0.1434391
1231.415	10.5	0.404965	0.3239714	0.1343569	0.09718803	0.128691	0.1442494
1231.515	10.5	0.40878	0.3265207	0.136008	0.09681239	0.129381	0.1440362
1231.615	10.5	0.395668	0.3278221	0.1362916	0.09437904	0.128895	0.1454156
1231.715	10.5	0.398472	0.3101503	0.1402762	0.09638041	0.125044	0.143268
1231.815	10.5	0.397234	0.3215973	0.1370083	0.09733078	0.126919	0.1408624
1231.915	10.5	0.383654	0.3437565	0.1367054	0.09582034	0.125792	0.1449746
1232.015	10.5	0.374569	0.3522328	0.1357774	0.09577726	0.126554	0.1468738
1232.115	10.5	0.388968	0.3645888	0.1355785	0.09520024	0.124223	0.1417555
1232.215	10.5	0.368078	0.3522668	0.1351496	0.09445083	0.122372	0.1405309
1232.315	10.5	0.389316	0.3326385	0.1385618	0.09462535	0.119759	0.1419903
1232.415	10.5	0.405018	0.3318511	0.1371895	0.09419348	0.119577	0.1429655
1232.515	10.5	0.413877	0.338036	0.1360179	0.09635334	0.118558	0.1434968
1232.615	10.5	0.395272	0.3508751	0.1371681	0.09592731	0.119892	0.1442238
1232.715	10.5	0.377072	0.3490917	0.1371192	0.09492113	0.120429	0.1480118
1232.815	10.5	0.382407	0.3544828	0.1381391	0.09642956	0.117824	0.1476674

1232.915	10.5	0.384198	0.356331	0.1379871	0.09779354	0.119017	0.1468593
1233.015	10.5	0.381154	0.3441861	0.1357964	0.0981562	0.120113	0.1468296
1233.115	10.5	0.393515	0.3395643	0.1349715	0.09969333	0.119114	0.146613
1233.215	10.5	0.395975	0.3422067	0.1358027	0.1011343	0.116781	0.1475743
1233.315	10.5	0.419981	0.347918	0.1334382	0.1028602	0.11737	0.1474281
1233.415	10.5	0.395754	0.3466058	0.1325613	0.1045805	0.11962	0.148497
1233.515	10.5	0.391225	0.3442439	0.1337503	0.1027867	0.119594	0.1464163
1233.615	10.5	0.390679	0.3440693	0.1351498	0.09945421	0.120518	0.1452449
1233.715	10.5	0.381467	0.3450047	0.1349906	0.09948042	0.120235	0.1432581
1233.815	10.5	0.379452	0.3404889	0.1370591	0.1014703	0.121662	0.1430674
1233.915	10.5	0.378161	0.3307498	0.1340885	0.1034456	0.119571	0.1449334
1234.015	10.5	0.37395	0.3307194	0.135835	0.1037364	0.118122	0.1438166
1234.115	10.5	0.385778	0.3256076	0.1351256	0.1042343	0.121058	0.1428779
1234.215	10.5	0.390507	0.3180448	0.1344356	0.1042665	0.123879	0.1435861
1234.315	10.5	0.383516	0.3304188	0.1329868	0.1042239	0.126411	0.1429118
1234.415	10.5	0.388567	0.3126239	0.1309198	0.1013175	0.128795	0.1459637
1234.515	10.5	0.395444	0.3221407	0.1266284	0.1003782	0.132995	0.145268
1234.615	10.5	0.390559	0.3408991	0.1273313	0.1033426	0.133217	0.1447879
1234.715	10.5	0.395537	0.3572467	0.127446	0.1004731	0.131175	0.1416889
1234.815	10.5	0.397708	0.3587838	0.1296761	0.09721961	0.129625	0.1409577
1234.915	10.5	0.392654	0.3618182	0.1282397	0.09547738	0.129881	0.1391336
1235.015	10.5	0.394626	0.342877	0.1321547	0.09333793	0.132441	0.1398346
1235.115	10.5	0.395826	0.3328511	0.132677	0.0908883	0.135809	0.1371197
1235.215	10.5	0.390386	0.3387541	0.1297728	0.09052987	0.135464	0.1418799
1235.315	10.5	0.389388	0.3446398	0.1306683	0.09453879	0.138044	0.1423484
1235.415	10.5	0.378041	0.3484026	0.1326453	0.09830622	0.135737	0.1400787
1235.515	10.5	0.370463	0.3538573	0.1322841	0.09548198	0.134721	0.1434757
1235.615	10.5	0.388492	0.3666146	0.1313886	0.09513143	0.134152	0.1462923
1235.715	10.5	0.399591	0.3496867	0.134408	0.09579652	0.133444	0.1460406
1235.815	10.5	0.395611	0.3449978	0.1323958	0.09599227	0.13266	0.1441283
1235.915	10.5	0.395892	0.3413118	0.1319261	0.09517642	0.128748	0.1435139
1236.015	10.5	0.386807	0.3438251	0.1308316	0.09489661	0.129607	0.1410676
1236.115	10.5	0.378377	0.3456299	0.1301428	0.09517419	0.131847	0.1375111
1236.215	10.5	0.403511	0.3414968	0.1284405	0.09402357	0.134108	0.1421328
1236.315	10.5	0.394856	0.3401026	0.1295863	0.09368247	0.132781	0.1409926
1236.415	10.5	0.402667	0.3431026	0.1311011	0.09703308	0.130074	0.1396258
1236.515	10.5	0.385463	0.3368564	0.1329185	0.09690376	0.129555	0.1436675
1236.615	10.5	0.387693	0.33167	0.1339635	0.09345376	0.130917	0.1462034
1236.715	10.5	0.388209	0.3292105	0.1358006	0.09343697	0.128382	0.1456021
1236.815	10.5	0.38809	0.3315724	0.1374684	0.0927748	0.127694	0.1477684
1236.915	10.5	0.389655	0.3161503	0.1408239	0.09178257	0.129079	0.1511776
1237.015	10.5	0.395672	0.3206368	0.1402529	0.09521683	0.129925	0.1523326
1237.115	10.5	0.395491	0.3243273	0.1419402	0.09642456	0.128931	0.1525282
1237.215	10.5	0.402195	0.3115882	0.1434634	0.0970785	0.130009	0.1520307
1237.315	10.5	0.395632	0.3296755	0.1402232	0.09368075	0.128256	0.1530733
1237.415	10.5	0.398886	0.3491404	0.143622	0.09596002	0.125479	0.1532787
1237.515	10.5	0.399583	0.3533178	0.1432114	0.09904656	0.126503	0.1517673
1237.615	10.5	0.393798	0.3558555	0.1432095	0.1001318	0.127163	0.1506108
1237.715	10.5	0.375264	0.3449731	0.1407487	0.1015014	0.126287	0.1495951
1237.815	10.5	0.394636	0.3333715	0.1398056	0.1010985	0.124046	0.1502892
1237.915	10.5	0.382904	0.3344451	0.1402183	0.1019362	0.121589	0.1515203
1238.015	10.5	0.395879	0.3396705	0.1406387	0.09981109	0.117917	0.1478442
1238.115	10.5	0.413056	0.3471767	0.1396414	0.09984919	0.120046	0.1498923
1238.215	10.5	0.401836	0.3510504	0.1360225	0.1006234	0.121013	0.1483129
1238.315	10.5	0.391163	0.3579409	0.1388164	0.09919601	0.121939	0.1471798
1238.415	10.5	0.3793	0.3533986	0.1386094	0.09882078	0.120291	0.1449666
1238.515	10.5	0.382158	0.3488956	0.1356179	0.1006684	0.114204	0.1417707
1238.615	10.5	0.385317	0.3444179	0.1364867	0.09872609	0.115196	0.1444132
1238.715	10.5	0.3796	0.3446476	0.1351103	0.09951776	0.115792	0.1465967
1238.815	10.5	0.386007	0.3473417	0.1359692	0.1003513	0.11581	0.1429491
1238.915	10.5	0.390597	0.3463358	0.1377334	0.1042305	0.119212	0.1409174
1239.015	10.5	0.415804	0.344603	0.1388252	0.1054127	0.11852	0.1443457
1239.115	10.5	0.401176	0.3462386	0.1392308	0.1073556	0.117898	0.1444234
1239.215	10.5	0.397549	0.3400896	0.1407149	0.1026703	0.115234	0.142578
1239.315	10.5	0.393241	0.333401	0.1400008	0.09899355	0.116794	0.1452459
1239.415	10.5	0.390887	0.3291304	0.1357892	0.1017644	0.121182	0.1466101
1239.515	10.5	0.390109	0.330856	0.1365441	0.1016573	0.125175	0.1471906
1239.615	10.5	0.398581	0.3244126	0.1361174	0.1024956	0.119226	0.147886
1239.715	10.5	0.39229	0.3233162	0.1343547	0.1022903	0.114906	0.1469803
1239.815	10.5	0.386323	0.3284682	0.1353127	0.1062928	0.116638	0.1481306
1239.915	10.5	0.394495	0.3114257	0.1363407	0.1056478	0.119122	0.1456872
1240.015	10.5	0.38952	0.324405	0.134166	0.1042838	0.120711	0.1437083
1240.115	10.5	0.394657	0.3539001	0.1356178	0.100973	0.119256	0.1470951
1240.215	10.5	0.399787	0.3734757	0.1350574	0.09990084	0.117628	0.1450706
1240.315	10.5	0.392862	0.3632873	0.1359593	0.1011397	0.122219	0.1466381
1240.415	10.5	0.399764	0.3572911	0.1347382	0.1013557	0.125894	0.1454605
1240.515	10.5	0.399948	0.3512232	0.1349961	0.09827855	0.127251	0.1441412
1240.615	10.5	0.409704	0.354518	0.1313863	0.1004908	0.129844	0.1447424
1240.715	10.5	0.401383	0.3592607	0.1318558	0.09887125	0.131775	0.1450874
1240.815	10.5	0.394645	0.3680208	0.1302517	0.09971629	0.133306	0.1441553
1240.915	10.5	0.387423	0.356277	0.1297125	0.09876983	0.132106	0.1437988
1241.015	10.5	0.39535	0.3550332	0.131025	0.09568705	0.132457	0.145376
1241.115	10.5	0.386325	0.3605279	0.1269375	0.09549966	0.13172	0.1455652
1241.215	10.5	0.372498	0.3534675	0.1274178	0.09382959	0.130438	0.1444105
1241.315	10.5	0.392597	0.3565781	0.1288334	0.09528607	0.132071	0.1426061
1241.415	10.5	0.399785	0.3621169	0.13249	0.09727852	0.130791	0.1433862
1241.515	10.5	0.39278	0.3668921	0.1302214	0.09822319	0.129474	0.1414925
1241.615	10.5	0.391328	0.3676854	0.1269965	0.09784614	0.125441	0.1422299
1241.715	10.5	0.388647	0.3697554	0.1288332	0.09919989	0.126885	0.1404176
1241.815	10.5	0.381778	0.3681946	0.1275949	0.0967358	0.125692	0.1394852
1241.915	10.5	0.405809	0.3618344	0.1290994	0.09802079	0.127301	0.1386571
1242.015	10.5	0.397723	0.351068	0.1310441	0.09752776	0.128275	0.1374322
1242.115	10.5	0.407363	0.3443271	0.131314	0.1003362	0.127191	0.1397373
1242.215	10.5	0.387752	0.3432566	0.1296395	0.09878569	0.125874	0.1431825
1242.315	10.5	0.388623	0.3294127	0.1308614	0.0967812	0.12656	0.1444908

1242.415	10.5	0.395794	0.3247523	0.1313934	0.09317682	0.126639	0.1429606
1242.515	10.5	0.386877	0.3299097	0.1305019	0.0944791	0.12358	0.1430909
1242.615	10.5	0.386631	0.3177823	0.1304725	0.09421092	0.124149	0.1443204
1242.715	10.5	0.393831	0.3242484	0.1323085	0.09565927	0.123605	0.1425958
1242.815	10.5	0.400568	0.35842	0.128987	0.0966078	0.126698	0.143385
1242.915	10.5	0.403535	0.3726704	0.1352404	0.0985245	0.128526	0.1439406
1243.015	10.5	0.390181	0.3646224	0.1376949	0.09545551	0.126842	0.1457349
1243.115	10.5	0.399327	0.3594183	0.1408915	0.09488748	0.124825	0.1470648
1243.215	10.5	0.399114	0.3495084	0.1383882	0.0929276	0.122892	0.147504
1243.315	10.5	0.390261	0.3483893	0.141839	0.09376984	0.121449	0.1457568
1243.415	10.5	0.371279	0.3526384	0.1420627	0.09583665	0.123631	0.1458409
1243.515	10.5	0.390425	0.3618957	0.1448045	0.09446162	0.121535	0.1470426
1243.615	10.5	0.37554	0.3535407	0.1429662	0.09614841	0.12027	0.1489948
1243.715	10.5	0.391964	0.3571941	0.1423274	0.09497683	0.119266	0.150561
1243.815	10.5	0.40918	0.3568789	0.1430503	0.09205332	0.119158	0.1513137
1243.915	10.5	0.401088	0.3467007	0.1413456	0.09424246	0.115075	0.1488365
1244.015	10.5	0.391384	0.3452152	0.1415175	0.09225143	0.117387	0.1520306
1244.115	10.5	0.380161	0.3479001	0.1414609	0.09471925	0.119262	0.1516338
1244.215	10.5	0.382862	0.3521412	0.1406341	0.09534106	0.121728	0.1498309
1244.315	10.5	0.382517	0.3533801	0.141231	0.09540771	0.120465	0.1492704
1244.415	10.5	0.378861	0.3506596	0.1400998	0.09619533	0.117579	0.1500363
1244.515	10.5	0.37944	0.3540668	0.1378863	0.09809728	0.116873	0.1484296
1244.615	10.5	0.389117	0.3497159	0.1406235	0.09930015	0.116848	0.1478547
1244.715	10.5	0.409105	0.3417589	0.13877	0.09709016	0.113697	0.1468136
1244.815	10.5	0.397153	0.3349737	0.1376982	0.1006695	0.11574	0.1474098
1244.915	10.5	0.397171	0.3364928	0.1381765	0.0990525	0.119971	0.1488552
1245.015	10.5	0.386904	0.333061	0.1418362	0.09579601	0.120828	0.1476481
1245.115	10.5	0.385813	0.3195418	0.140988	0.09942176	0.123767	0.144553
1245.215	10.5	0.382301	0.3460302	0.1400505	0.09792416	0.12488	0.1432112
1245.315	10.5	0.380732	0.3283397	0.1406081	0.09634495	0.121208	0.1438758
1245.415	10.5	0.381293	0.320895	0.1375294	0.09740369	0.117214	0.1436237
1245.515	10.5	0.38436	0.3581096	0.1379361	0.100342	0.119882	0.1456401
1245.615	10.5	0.387553	0.3760744	0.1421635	0.1026241	0.120584	0.1440768
1245.715	10.5	0.394634	0.370364	0.1426526	0.1047968	0.125141	0.1440316
1245.815	10.5	0.391643	0.3660883	0.1421488	0.0990264	0.123124	0.1419758
1245.915	10.5	0.398397	0.3546375	0.1411476	0.0972346	0.124991	0.1431933
1246.015	10.5	0.392709	0.3500406	0.1397482	0.09853124	0.127336	0.1448727
1246.115	10.5	0.397363	0.3546031	0.1406116	0.09616823	0.127863	0.1460718
1246.215	10.5	0.393501	0.3607618	0.1413821	0.09531738	0.128729	0.1444981
1246.315	10.5	0.394741	0.3640722	0.139552	0.09782247	0.126541	0.1445977
1246.415	10.5	0.39356	0.3502961	0.1404371	0.09935893	0.127531	0.1451866
1246.515	10.5	0.393237	0.3630353	0.1391613	0.09822758	0.127971	0.1463297
1246.615	10.5	0.389357	0.3469573	0.1380375	0.09656224	0.127616	0.1420366
1246.715	10.5	0.394888	0.3424753	0.1370216	0.09774364	0.130686	0.1420915
1246.815	10.5	0.390904	0.3433218	0.138554	0.09651788	0.1319	0.1449572
1246.915	10.5	0.379644	0.3477233	0.1401473	0.09369563	0.130632	0.1439309
1247.015	10.5	0.399414	0.3518766	0.1393653	0.09410053	0.131673	0.1442503
1247.115	10.5	0.405106	0.3481143	0.1380682	0.09667976	0.125753	0.1453899
1247.215	10.5	0.403023	0.3532636	0.1376713	0.09641088	0.124873	0.1437262
1247.315	10.5	0.396437	0.3505847	0.1360704	0.09826442	0.12436	0.1411029
1247.415	10.5	0.393948	0.3425484	0.1364698	0.09703955	0.125545	0.1419214
1247.515	10.5	0.387051	0.3349122	0.1365448	0.09731941	0.128739	0.1437295
1247.615	10.5	0.396403	0.3341583	0.1373263	0.09809321	0.128921	0.1436276
1247.715	10.5	0.394679	0.3330766	0.1357012	0.09640449	0.126894	0.1475567
1247.815	10.5	0.401791	0.3223661	0.129342	0.09963752	0.124473	0.1444308
1247.915	10.5	0.386828	0.3313957	0.1325148	0.09784032	0.123638	0.1412557
1248.015	10.5	0.384851	0.3377334	0.13106	0.09633901	0.123076	0.1408353
1248.115	10.5	0.397246	0.3214551	0.1361814	0.09600855	0.122521	0.1394867
1248.215	10.5	0.387952	0.3396971	0.133224	0.09422059	0.121043	0.1409906
1248.315	10.5	0.386374	0.3717989	0.1318677	0.09061499	0.121139	0.143392
1248.415	10.5	0.393642	0.3661903	0.1332183	0.09169095	0.120894	0.1403078
1248.515	10.5	0.400056	0.3579961	0.1313941	0.09394147	0.124469	0.1416124
1248.615	10.5	0.403298	0.3451746	0.1317705	0.09669831	0.123674	0.1399801
1248.715	10.5	0.386479	0.3397327	0.1338449	0.09406244	0.122783	0.138543
1248.815	10.5	0.393935	0.3466717	0.1328076	0.09441199	0.121785	0.1380522
1248.915	10.5	0.393716	0.354324	0.1332418	0.09327536	0.119212	0.1404257
1249.015	10.5	0.390319	0.3591188	0.1347959	0.0971771	0.120218	0.1435507
1249.115	10.5	0.367329	0.3488976	0.1346993	0.0983678	0.11947	0.1420876
1249.215	10.5	0.377435	0.3559473	0.1332958	0.09560456	0.120026	0.1398683
1249.315	10.5	0.379088	0.3487164	0.1336161	0.09761254	0.12099	0.139896
1249.415	10.5	0.377713	0.3410823	0.1338377	0.09621715	0.117945	0.1396318
1249.515	10.5	0.397773	0.3397831	0.1322015	0.09416151	0.116711	0.1443952
1249.615	10.5	0.39249	0.342849	0.1350864	0.09337782	0.117529	0.1450403
1249.715	10.5	0.38582	0.3462676	0.1365794	0.09224811	0.11844	0.1433234
1249.815	10.5	0.383652	0.3432229	0.1355169	0.09298138	0.119757	0.1429277
1249.915	10.5	0.386258	0.347563	0.137046	0.09386515	0.119736	0.1442132
1250.015	10.5	0.386537	0.3485983	0.1377625	0.09425043	0.121537	0.142492
1250.115	10.5	0.381951	0.3428101	0.1385736	0.09630124	0.121771	0.1456943
1250.215	10.5	0.375206	0.3337145	0.1409974	0.09877384	0.120448	0.1471565
1250.315	10.5	0.393011	0.3313152	0.1408961	0.0987651	0.11775	0.1456806
1250.415	10.5	0.396887	0.3324266	0.1389749	0.1002861	0.118739	0.1476774
1250.515	10.5	0.400599	0.3203689	0.1391014	0.1025809	0.1206	0.1475585
1250.615	10.5	0.395815	0.3210602	0.1410688	0.1023514	0.119784	0.1491083
1250.715	10.5	0.382748	0.3412336	0.141312	0.1020581	0.120194	0.1504288
1250.815	10.5	0.387741	0.319811	0.1416789	0.1017211	0.12108	0.1480917
1250.915	10.5	0.382472	0.3245521	0.1429487	0.1014756	0.122191	0.1461588
1251.015	10.5	0.379933	0.3702215	0.1423665	0.1022447	0.119627	0.1430048
1251.115	10.5	0.378397	0.3619539	0.1415891	0.1022674	0.115648	0.1435146
1251.215	10.5	0.383979	0.3558412	0.1401267	0.1022096	0.120981	0.1473248
1251.315	10.5	0.386204	0.3481855	0.1410395	0.1024255	0.122185	0.143896
1251.415	10.5	0.395719	0.3365254	0.1370065	0.1040185	0.123645	0.1436525
1251.515	10.5	0.391604	0.3415116	0.1358085	0.09836899	0.124324	0.1424163
1251.615	10.5	0.396853	0.3459779	0.1355735	0.09610496	0.120686	0.1432686
1251.715	10.5	0.396952	0.3548516	0.1401125	0.09641837	0.123098	0.1439827
1251.815	10.5	0.398223	0.3461871	0.1363715	0.09500845	0.123719	0.1421508

1251.915	10.5	0.385822	0.3485111	0.1376638	0.09595601	0.12485	0.1432465
1252.015	10.5	0.413432	0.3529393	0.1384965	0.0966882	0.125509	0.1470339
1252.115	10.5	0.4136	0.341633	0.1397904	0.09727091	0.128124	0.1430212
1252.215	10.5	0.412053	0.3387389	0.1389403	0.09842064	0.130512	0.1418065
1252.315	10.5	0.411492	0.3399439	0.1433999	0.09662975	0.130792	0.1441037
1252.415	10.5	0.421332	0.3442121	0.1421266	0.09469829	0.13325	0.1437818
1252.515	10.5	0.412264	0.3401782	0.1385084	0.09430677	0.133258	0.1394321
1252.615	10.5	0.404607	0.3446079	0.139441	0.09344471	0.132143	0.1443608
1252.715	10.5	0.417758	0.3451018	0.1380608	0.09421375	0.131713	0.1461756
1252.815	10.5	0.419994	0.3418141	0.1396568	0.09445968	0.125992	0.142299
1252.915	10.5	0.422004	0.334531	0.1422739	0.09566557	0.125908	0.1426409
1253.015	10.5	0.40704	0.3291866	0.1390473	0.09674388	0.126951	0.1434672
1253.115	10.5	0.417337	0.3313489	0.1398196	0.09531219	0.127324	0.1477185
1253.215	10.5	0.40435	0.3233429	0.1410766	0.09575794	0.126655	0.1475507
1253.315	10.5	0.393884	0.314413	0.1374284	0.09786993	0.127997	0.1457198
1253.415	10.5	0.400433	0.3351768	0.136543	0.09763631	0.126221	0.1471771
1253.515	10.5	0.399592	0.3174621	0.1378616	0.1005536	0.121086	0.1447221
1253.615	10.5	0.390279	0.321238	0.140821	0.0984576	0.123808	0.1444723
1253.715	10.5	0.386998	0.3614826	0.1398589	0.09570339	0.124213	0.1432452
1253.815	10.5	0.405109	0.3621429	0.1403362	0.09754971	0.123933	0.1427349
1253.915	10.5	0.401513	0.3594338	0.136409	0.0979101	0.124898	0.1421194
1254.015	10.5	0.38894	0.3509081	0.1371877	0.09626637	0.125912	0.1422405
1254.115	10.5	0.405527	0.336078	0.1361145	0.09389208	0.125075	0.1425849
1254.215	10.5	0.410235	0.3344646	0.1360908	0.09563649	0.125298	0.1441615
1254.315	10.5	0.420207	0.3419833	0.1363507	0.09517752	0.125683	0.1438247
1254.415	10.5	0.396189	0.3490509	0.1360199	0.09142218	0.123694	0.1466887
1254.515	10.5	0.399937	0.3475554	0.131703	0.09279013	0.125124	0.1452577
1254.615	10.5	0.396076	0.3443252	0.1331054	0.09086797	0.121195	0.1456785
1254.715	10.5	0.39305	0.3567354	0.13122	0.09068025	0.121383	0.1449012
1254.815	10.5	0.369791	0.3498108	0.1345992	0.09401346	0.119439	0.1453001
1254.915	10.5	0.379511	0.3408735	0.131052	0.09696312	0.118584	0.1457207
1255.015	10.5	0.395001	0.3396362	0.1310341	0.09871455	0.120443	0.1448866
1255.115	10.5	0.393799	0.3435025	0.1318918	0.09641133	0.119638	0.1400865
1255.215	10.5	0.453942	0.3418236	0.1295607	0.09414695	0.118248	0.1418755
1255.315	10.5	0.446456	0.3378552	0.1310192	0.09324315	0.119152	0.1411643
1255.415	10.5	0.422751	0.3380648	0.1353864	0.0925886	0.117311	0.1410093
1255.515	10.5	0.402922	0.3432252	0.1331032	0.09275793	0.116952	0.1424453
1255.615	10.5	0.396923	0.3379672	0.1333077	0.09418222	0.11574	0.1430914
1255.715	10.5	0.386265	0.3315594	0.1323343	0.09677984	0.11644	0.1426453
1255.815	10.5	0.389673	0.3312182	0.130226	0.09701618	0.114812	0.1445706
1255.915	10.5	0.379786	0.3264835	0.1313496	0.09568574	0.114456	0.1432023
1256.015	10.5	0.393239	0.3165768	0.1315758	0.09858701	0.114058	0.1417828
1256.115	10.5	0.392627	0.3283753	0.1306542	0.1001625	0.11209	0.1433549
1256.215	10.5	0.399139	0.3303666	0.1334484	0.1013557	0.111863	0.1460207
1256.315	10.5	0.395487	0.3245402	0.1346357	0.100869	0.115895	0.1442373
1256.415	10.5	0.380945	0.3486122	0.1354567	0.09923106	0.116565	0.1447344
1256.515	10.5	0.386027	0.3623042	0.1372811	0.09633472	0.119108	0.1480262
1256.615	10.5	0.379729	0.3592608	0.134481	0.1006688	0.119157	0.1498995
1256.715	10.5	0.378222	0.3607332	0.1355413	0.1021182	0.118293	0.1489615
1256.815	10.5	0.378954	0.3407802	0.1375491	0.1010322	0.116471	0.151735
1256.915	10.5	0.38371	0.3343621	0.1399781	0.1027439	0.119265	0.1527458
1257.015	10.5	0.382086	0.3377815	0.1391344	0.1016824	0.122298	0.1504282
1257.115	10.5	0.391775	0.3457454	0.1394853	0.1059119	0.12398	0.1502168
1257.215	10.5	0.387698	0.348759	0.1402356	0.101173	0.122468	0.1493223
1257.315	10.5	0.389283	0.3424254	0.1413448	0.1003379	0.124404	0.1508948
1257.415	10.5	0.395308	0.359476	0.1425203	0.09904451	0.125312	0.1504809
1257.515	10.5	0.413473	0.3544696	0.1399656	0.09983315	0.126505	0.1472602
1257.615	10.5	0.397121	0.3424075	0.1393529	0.1007156	0.128589	0.1470651
1257.715	10.5	0.408443	0.3416077	0.1381986	0.1003968	0.125571	0.1476707
1257.815	10.5	0.415569	0.3434889	0.1383019	0.09923433	0.126888	0.1462126
1257.915	10.5	0.411433	0.3434241	0.1392277	0.1037255	0.128431	0.1464646
1258.015	10.5	0.406536	0.3399357	0.1387649	0.1016525	0.133103	0.1442484
1258.115	10.5	0.426902	0.3440338	0.1381615	0.09819571	0.133132	0.1447093
1258.215	10.5	0.425164	0.3437794	0.1370144	0.09818485	0.135498	0.146432
1258.315	10.5	0.401874	0.3380349	0.1403426	0.09947453	0.134001	0.147595
1258.415	10.5	0.397496	0.3341111	0.1404397	0.09976716	0.131769	0.1452974
1258.515	10.5	0.40841	0.3302891	0.1360362	0.09822962	0.130953	0.1448243
1258.615	10.5	0.396416	0.3260383	0.1383054	0.09835611	0.130971	0.1442221
1258.715	10.5	0.394055	0.3179134	0.1364177	0.09945253	0.127837	0.1468157
1258.815	10.5	0.395182	0.3257897	0.1359971	0.09868101	0.128913	0.1439974
1258.915	10.5	0.403336	0.3297287	0.13806	0.1003119	0.132586	0.1418865
1259.11	11	0.389595	0.3546353	0.1386468	0.1011274	0.129942	0.1421965
1259.21	11	0.388023	0.3584872	0.1380826	0.1000117	0.133294	0.1414946
1259.31	11	0.3901	0.3451185	0.1391045	0.1010966	0.129797	0.139809
1259.41	11	0.386414	0.3358281	0.13784	0.09543926	0.129378	0.1435237
1259.51	11	0.385437	0.3381159	0.1352884	0.09796287	0.126945	0.1430779
1259.61	11	0.410703	0.3441681	0.1368567	0.09667113	0.126678	0.1416598
1259.71	11	0.412382	0.3481204	0.135923	0.09760648	0.12368	0.1412324
1259.81	11	0.413888	0.3435782	0.1338473	0.1013182	0.123502	0.1402927
1259.91	11	0.392604	0.3539397	0.1350327	0.09827654	0.122727	0.1432267
1260.01	11	0.393737	0.3554633	0.1358753	0.09699458	0.123646	0.1423356
1260.11	11	0.399761	0.3465655	0.1361867	0.09498439	0.123485	0.1434398
1260.21	11	0.381023	0.3463833	0.1357795	0.0960045	0.122772	0.1453828
1260.31	11	0.387737	0.3526312	0.1371127	0.09880529	0.123301	0.143599
1260.41	11	0.389532	0.3529338	0.1348967	0.09671377	0.121965	0.1444571
1260.51	11	0.383685	0.348135	0.1352958	0.09510434	0.124204	0.1427412
1260.61	11	0.404858	0.3489667	0.1324121	0.09426526	0.121971	0.1407707
1260.71	11	0.404395	0.3505091	0.1310054	0.09486732	0.121402	0.1419474
1260.81	11	0.398178	0.3440474	0.1291017	0.09264372	0.120778	0.143263
1260.91	11	0.3819	0.3382482	0.1265251	0.09328446	0.118668	0.1445109
1261.01	11	0.384682	0.3353981	0.1287486	0.09532519	0.119039	0.1456056
1261.11	11	0.377638	0.3253664	0.1279667	0.09398278	0.119453	0.1471265
1261.21	11	0.373061	0.3165429	0.1327589	0.09012618	0.116335	0.1459576
1261.31	11	0.377502	0.3351744	0.1302567	0.09359589	0.118067	0.1429626
1261.41	11	0.383319	0.3329383	0.1326824	0.09397877	0.115398	0.1409294

1261.51	11	0.398243	0.321169	0.1342716	0.09523518	0.112436	0.1414169
1261.61	11	0.392868	0.3424224	0.1323583	0.09806373	0.111908	0.1421554
1261.71	11	0.383906	0.3770342	0.1314204	0.09889257	0.114144	0.142908
1261.81	11	0.381427	0.3619713	0.1342617	0.1016927	0.112239	0.1418887
1261.91	11	0.377284	0.3605437	0.1340152	0.100991	0.110526	0.1399236
1262.01	11	0.379588	0.3521065	0.1353627	0.1010194	0.110786	0.1409554
1262.11	11	0.391103	0.3536422	0.1354549	0.1007069	0.114034	0.1427394
1262.21	11	0.387657	0.3604744	0.1334926	0.09810156	0.115979	0.1412198
1262.31	11	0.386203	0.3657125	0.1337056	0.102206	0.115886	0.1418091
1262.41	11	0.389678	0.3645562	0.1336093	0.09908852	0.116041	0.1437667
1262.51	11	0.387352	0.3538753	0.1332817	0.0991554	0.115581	0.1428974
1262.61	11	0.39684	0.3621262	0.1341809	0.09810337	0.111047	0.1426949
1262.71	11	0.391443	0.3545808	0.1372027	0.09708355	0.115495	0.1432359
1262.81	11	0.389912	0.3570096	0.1369911	0.09704591	0.116698	0.1447525
1262.91	11	0.384952	0.363109	0.1379381	0.09725472	0.116622	0.1447921
1263.01	11	0.394587	0.3677041	0.1398038	0.09468371	0.121553	0.1461042
1263.11	11	0.39721	0.3682809	0.1398197	0.09354448	0.120539	0.1464011
1263.21	11	0.396255	0.3652924	0.1414527	0.09629323	0.124604	0.1459323
1263.31	11	0.385602	0.3654128	0.1427708	0.09522533	0.126127	0.1459993
1263.41	11	0.391481	0.3617918	0.1425061	0.09343942	0.12968	0.146153
1263.51	11	0.379588	0.3503413	0.1415037	0.09172155	0.128609	0.1468921
1263.61	11	0.36601	0.3428462	0.1409938	0.09012438	0.131297	0.1488505
1263.71	11	0.386758	0.3392998	0.1418818	0.09005885	0.12992	0.1479585
1263.81	11	0.389383	0.3300263	0.1397339	0.09092484	0.128361	0.1491486
1263.91	11	0.378435	0.3219316	0.1379358	0.09460372	0.129208	0.1510843
1264.01	11	0.378777	0.3485776	0.1393921	0.09589862	0.131858	0.1486127
1264.11	11	0.387495	0.3212596	0.1372841	0.09667679	0.133783	0.1488414
1264.21	11	0.372652	0.3288008	0.1379959	0.09451788	0.132445	0.1485451
1264.31	11	0.393342	0.3599582	0.1363955	0.09516691	0.132506	0.1471067
1264.41	11	0.392468	0.3648066	0.1377499	0.09509512	0.133629	0.1456932
1264.51	11	0.387318	0.3557802	0.1391384	0.09792008	0.132954	0.1482336
1264.61	11	0.387182	0.3493104	0.1419357	0.09687958	0.133916	0.1491862
1264.71	11	0.392547	0.3420904	0.1393773	0.09491409	0.135437	0.1480218
1264.81	11	0.386627	0.3430724	0.1404884	0.09358121	0.133049	0.14444
1264.91	11	0.382214	0.348814	0.1385467	0.09375273	0.13694	0.1434599
1265.01	11	0.405702	0.3594031	0.1374051	0.09298977	0.132612	0.1452402
1265.11	11	0.412952	0.3501426	0.1392567	0.09188335	0.12877	0.1463515
1265.21	11	0.411689	0.3561871	0.138441	0.09361327	0.127704	0.1450567
1265.31	11	0.390745	0.3536451	0.1374921	0.09342257	0.129156	0.1433883
1265.41	11	0.388654	0.3433036	0.1363134	0.09291425	0.127902	0.1461139
1265.51	11	0.399024	0.3417469	0.1353049	0.09209813	0.127775	0.1492045
1265.61	11	0.392515	0.3448947	0.1362489	0.09227038	0.12847	0.1472658
1265.71	11	0.382819	0.3469534	0.1360253	0.09064575	0.128991	0.1437065
1265.81	11	0.392896	0.3456548	0.1361145	0.09385655	0.130564	0.1458813
1265.91	11	0.373337	0.3487278	0.1346433	0.09488384	0.130807	0.1430587
1266.01	11	0.396467	0.3473972	0.1370152	0.09595698	0.13028	0.1430845
1266.11	11	0.402275	0.3442434	0.1374689	0.09525053	0.125483	0.1443525
1266.21	11	0.422721	0.3364076	0.1380027	0.09466067	0.125712	0.1437173
1266.31	11	0.406079	0.3307325	0.1400292	0.09456864	0.12465	0.1419145
1266.41	11	0.383332	0.3331553	0.1395818	0.09505154	0.125156	0.1411984
1266.51	11	0.385593	0.3245906	0.1367419	0.09844487	0.124378	0.1383181
1266.61	11	0.373894	0.3238662	0.1350719	0.09849273	0.121117	0.1423493
1266.71	11	0.373736	0.3425185	0.1311993	0.0981269	0.120321	0.1441016
1266.81	11	0.383539	0.3212123	0.1307432	0.09940627	0.121407	0.1451035
1266.91	11	0.395308	0.3304697	0.1313434	0.09839489	0.118689	0.1459894
1267.01	11	0.405695	0.3641619	0.1316929	0.0986212	0.121226	0.1419044
1267.11	11	0.402357	0.3605306	0.1315002	0.1022381	0.118829	0.1407336
1267.21	11	0.384922	0.3531317	0.1285465	0.105227	0.117389	0.1383873
1267.31	11	0.38244	0.3424498	0.1298773	0.1039423	0.118862	0.1358216
1267.41	11	0.379122	0.3373503	0.126714	0.1036444	0.12015	0.1365153
1267.51	11	0.379006	0.3426952	0.1308278	0.1035572	0.117561	0.1379669
1267.61	11	0.391776	0.3493386	0.1276929	0.1045186	0.117455	0.1395672
1267.71	11	0.389869	0.3525727	0.1315479	0.1063858	0.118467	0.1388538
1267.81	11	0.392815	0.3452305	0.1340865	0.1090238	0.11922	0.1412627
1267.91	11	0.388395	0.3565448	0.130779	0.1078819	0.116929	0.1403301
1268.01	11	0.381218	0.3507365	0.1310823	0.1035384	0.116739	0.1409408
1268.11	11	0.402263	0.3408734	0.1307248	0.09948301	0.117945	0.1410327
1268.21	11	0.409288	0.3405232	0.1308569	0.100491	0.119662	0.1406936
1268.31	11	0.408483	0.3439079	0.1317711	0.0984863	0.11494	0.1393347
1268.41	11	0.390785	0.3447702	0.132539	0.09850879	0.118638	0.1402489
1268.51	11	0.399108	0.3445532	0.1319376	0.09974703	0.121828	0.1401062
1268.61	11	0.393061	0.3464234	0.1319312	0.09845143	0.122687	0.1424359
1268.71	11	0.405351	0.3457166	0.1328785	0.09720166	0.125317	0.1423635
1268.81	11	0.408553	0.3405983	0.1325476	0.09623784	0.125653	0.1414951
1268.91	11	0.401684	0.3317724	0.1326573	0.09576654	0.128127	0.1407804
1269.01	11	0.394867	0.3284916	0.1334357	0.09732838	0.125602	0.1430513
1269.11	11	0.403261	0.3276319	0.1326685	0.09681234	0.130761	0.1443452
1269.21	11	0.403114	0.3158067	0.1372758	0.09585262	0.131375	0.1463331
1269.31	11	0.4045	0.3281621	0.1403455	0.09448746	0.129708	0.142157
1269.41	11	0.389817	0.3315594	0.1402084	0.09719433	0.130844	0.1410965
1269.51	11	0.386567	0.3241234	0.1428455	0.0975129	0.128865	0.1428682
1269.61	11	0.388716	0.3448608	0.1430516	0.09872509	0.130204	0.1433136
1269.71	11	0.380714	0.3646874	0.1417026	0.09809004	0.13166	0.1444795
1269.81	11	0.397736	0.3587704	0.1407862	0.09762421	0.130608	0.1441674
1269.91	11	0.404051	0.3516654	0.1411345	0.09594402	0.131182	0.146197
1270.01	11	0.389688	0.3372514	0.142709	0.09573023	0.129331	0.1469583
1270.11	11	0.387757	0.3321804	0.1404034	0.09455653	0.131009	0.1450933
1270.21	11	0.3954	0.3394648	0.1406256	0.09734661	0.128732	0.146252
1270.31	11	0.386668	0.3469538	0.1382668	0.0949024	0.12834	0.1471062
1270.41	11	0.387196	0.3488426	0.1369579	0.09774771	0.132961	0.1472608
1270.51	11	0.429295	0.343554	0.1368547	0.09729218	0.131822	0.1482385
1270.61	11	0.418566	0.3591231	0.1365554	0.09879773	0.133614	0.1465816
1270.71	11	0.416961	0.3456432	0.1373169	0.1007085	0.130118	0.1447856
1270.81	11	0.391573	0.3393842	0.1371398	0.09926976	0.128594	0.1478163
1270.91	11	0.393067	0.3394045	0.1406837	0.09883679	0.125789	0.1465826

1271.01	11	0.40035	0.3412715	0.1393051	0.09672443	0.127373	0.1431409
1271.11	11	0.386229	0.3424416	0.14049	0.09546912	0.124422	0.143752
1271.21	11	0.387319	0.340291	0.1400604	0.0958154	0.125517	0.1448421
1271.31	11	0.390547	0.3423472	0.1379079	0.09522106	0.126381	0.1453832
1271.41	11	0.38161	0.3405271	0.1363495	0.09561709	0.126979	0.1429827
1271.51	11	0.397369	0.3342374	0.1361213	0.09429833	0.125612	0.144554
1271.61	11	0.405858	0.3275607	0.1375678	0.0940958	0.122723	0.1455793
1271.71	11	0.410523	0.3297241	0.1387119	0.09211478	0.121985	0.1451589
1271.81	11	0.385898	0.3284513	0.1380447	0.09088918	0.120609	0.1428017
1271.91	11	0.386918	0.3117336	0.1391769	0.09174713	0.120314	0.139505
1272.01	11	0.380538	0.3412338	0.1400944	0.09355959	0.119907	0.1396894
1272.11	11	0.378091	0.3313443	0.1393976	0.09366622	0.119904	0.1431455
1272.21	11	0.375149	0.3322569	0.1383478	0.09562863	0.117688	0.1423181
1272.31	11	0.384664	0.3595729	0.1376274	0.09490783	0.116917	0.1404997
1272.41	11	0.392699	0.3597733	0.1364267	0.09790573	0.114815	0.141322
1272.51	11	0.390976	0.3588088	0.1356937	0.09941227	0.113641	0.1417713
1272.61	11	0.388907	0.3465034	0.1361529	0.09893149	0.113742	0.140672
1272.71	11	0.382672	0.3334263	0.1350946	0.1002755	0.117079	0.1439279
1272.81	11	0.378439	0.3333946	0.1330057	0.09833423	0.116603	0.1458958
1272.91	11	0.381002	0.3378608	0.1348157	0.09939301	0.115786	0.142073
1273.01	11	0.391436	0.3469644	0.131431	0.1008765	0.117863	0.1390806
1273.11	11	0.392685	0.3455151	0.1306797	0.1017638	0.114422	0.1392341
1273.21	11	0.392275	0.3502106	0.1284859	0.1028711	0.111563	0.1436449
1273.31	11	0.397701	0.3577914	0.1312057	0.1039365	0.113552	0.1422021
1273.41	11	0.382911	0.3413417	0.1309095	0.1014723	0.1135	0.1407691
1273.51	11	0.392652	0.3386112	0.1270619	0.09857785	0.114056	0.1415695
1273.61	11	0.403043	0.3389487	0.1282101	0.09948201	0.114568	0.137385
1273.71	11	0.399214	0.3425966	0.1265823	0.1006052	0.114415	0.1374142
1273.81	11	0.393824	0.3410229	0.1304246	0.100186	0.114082	0.1391205
1273.91	11	0.433741	0.3424553	0.1288218	0.1015296	0.114918	0.1395038
1274.01	11	0.427327	0.3411093	0.1296113	0.09877387	0.114214	0.14143
1274.11	11	0.42248	0.3358702	0.132634	0.09868337	0.116006	0.1429087
1274.21	11	0.41099	0.3310161	0.129355	0.09500891	0.120791	0.1426218
1274.31	11	0.42032	0.3283153	0.1312943	0.094895	0.120952	0.1430753
1274.41	11	0.398816	0.329555	0.1311943	0.09326974	0.122524	0.1423294
1274.51	11	0.375111	0.3258501	0.1307281	0.09231552	0.12259	0.1452707
1274.61	11	0.393361	0.3195201	0.132063	0.09231155	0.122682	0.1425722
1274.71	11	0.387938	0.3398218	0.1343301	0.09077541	0.122981	0.1439103
1274.81	11	0.388039	0.3267736	0.1349793	0.08920626	0.126772	0.1433024
1274.91	11	0.382481	0.334383	0.1345756	0.09148054	0.126947	0.1430405
1275.01	11	0.381436	0.3626817	0.133344	0.09149725	0.127198	0.1435627
1275.11	11	0.373864	0.3551716	0.1298041	0.09319851	0.128018	0.1412929
1275.21	11	0.397543	0.3577436	0.1284894	0.09459066	0.130226	0.1384405
1275.31	11	0.401562	0.3441303	0.1305773	0.09425457	0.130191	0.1411239
1275.41	11	0.402619	0.3303238	0.1299595	0.09874229	0.131693	0.1418068
1275.51	11	0.39501	0.3328936	0.1328789	0.09684833	0.130267	0.1447188
1275.61	11	0.407387	0.338489	0.1353466	0.09541246	0.129081	0.1460008
1275.71	11	0.391578	0.3457675	0.1380516	0.09261585	0.129863	0.1477711
1275.81	11	0.39138	0.3407423	0.140511	0.09296279	0.129218	0.1476732
1275.91	11	0.41346	0.3584652	0.1406547	0.0946358	0.125856	0.1476548
1276.01	11	0.42177	0.3526029	0.1407397	0.09544493	0.12478	0.1484996
1276.11	11	0.40923	0.3392128	0.1399458	0.09512741	0.126294	0.1464904
1276.21	11	0.393521	0.3371573	0.1413036	0.09534127	0.127376	0.1477219
1276.31	11	0.389578	0.3408447	0.1426722	0.09592436	0.127949	0.1486519
1276.41	11	0.394988	0.3414598	0.1404424	0.09580866	0.125924	0.1495694
1276.51	11	0.388285	0.3387946	0.141726	0.09537949	0.124792	0.151662
1276.61	11	0.379278	0.3394176	0.1393113	0.09403974	0.124992	0.1524485
1276.71	11	0.378307	0.3413555	0.1371458	0.09593599	0.123895	0.1526889
1276.81	11	0.376441	0.3361188	0.1380144	0.09569941	0.122876	0.1512877
1276.91	11	0.392444	0.3295325	0.1373476	0.0928276	0.123173	0.1512458
1277.01	11	0.431858	0.3294683	0.1365598	0.0914242	0.124512	0.1521637
1277.11	11	0.407428	0.3305317	0.1371963	0.09088359	0.125381	0.1529843
1277.21	11	0.398539	0.3162901	0.1393546	0.08927167	0.125764	0.153299
1277.31	11	0.386365	0.322308	0.1378219	0.0896784	0.124707	0.1518965
1277.41	11	0.388467	0.3297306	0.1363124	0.09193519	0.122986	0.1502464
1277.51	11	0.378294	0.3229874	0.1334092	0.09038293	0.12167	0.1486059
1277.61	11	0.377088	0.335412	0.1347788	0.08766744	0.123148	0.1457766
1277.71	11	0.386514	0.3598357	0.1357933	0.08962049	0.121308	0.1442504
1277.81	11	0.386648	0.3531152	0.1370347	0.09039956	0.122244	0.1433569
1277.91	11	0.393754	0.3532563	0.1353338	0.09142371	0.12019	0.1445964
1278.01	11	0.38328	0.3448941	0.1325094	0.09806956	0.1196	0.1429671
1278.11	11	0.383087	0.3385147	0.1338819	0.1002835	0.119713	0.1394384
1278.21	11	0.38573	0.3417565	0.1345736	0.1010359	0.119766	0.1416985
1278.31	11	0.384185	0.3483299	0.1328522	0.09784079	0.119473	0.1425555
1278.41	11	0.379023	0.351178	0.1322353	0.09950503	0.119797	0.1436295
1278.51	11	0.388407	0.3510692	0.1337578	0.0995714	0.120177	0.1433351
1278.61	11	0.390972	0.3633217	0.1351697	0.09652362	0.117941	0.1419348
1278.71	11	0.394472	0.3451332	0.136525	0.09864987	0.117126	0.1431298
1278.81	11	0.388255	0.3432456	0.1341301	0.09865458	0.119575	0.1443905
1278.91	11	0.38055	0.3476588	0.1341632	0.09964745	0.117149	0.1419825
1279.01	11	0.40654	0.3515217	0.1358294	0.09660544	0.117084	0.1417118
1279.11	11	0.415777	0.3529824	0.134574	0.09752945	0.117699	0.1433021
1279.21	11	0.413287	0.3519773	0.1344966	0.0986394	0.116624	0.1407461
1279.31	11	0.403873	0.3521151	0.129965	0.09909489	0.117314	0.1373132
1279.41	11	0.406782	0.3489502	0.1291945	0.09935796	0.11641	0.1405935
1279.51	11	0.389318	0.3407488	0.1286885	0.09841593	0.11859	0.1418666
1279.61	11	0.394131	0.334261	0.1287191	0.100734	0.120173	0.1388419
1279.71	11	0.388797	0.3337068	0.131015	0.09631747	0.118759	0.1387277
1279.81	11	0.405729	0.3331269	0.1292717	0.09393815	0.11701	0.1376627
1279.91	11	0.39368	0.3095589	0.1287802	0.09534385	0.120146	0.1408249
1280.01	11	0.397993	0.3340447	0.128244	0.09542499	0.121747	0.1424531
1280.11	11	0.400967	0.3187781	0.1322442	0.09742164	0.123637	0.1407271
1280.21	11	0.401952	0.3220654	0.1308294	0.09673808	0.123884	0.143264
1280.31	11	0.398063	0.344977	0.129074	0.0957503	0.123861	0.1379462
1280.41	11	0.407271	0.3629582	0.1304872	0.09549186	0.123149	0.1399424

1280.51	11	0.399605	0.3604974	0.1281724	0.09641673	0.12558	0.1411196
1280.61	11	0.397315	0.3443914	0.1301109	0.09724576	0.127493	0.1432423
1280.71	11	0.424395	0.3323373	0.1321194	0.09829953	0.122771	0.144294
1280.81	11	0.435831	0.332887	0.1297127	0.09526856	0.124837	0.1453351
1280.91	11	0.420541	0.3392673	0.1293353	0.09644452	0.126202	0.143493
1281.01	11	0.412175	0.3503787	0.1307571	0.09297493	0.124789	0.1430295
1281.11	11	0.417662	0.3440466	0.1313342	0.09367258	0.125021	0.1438335
1281.21	11	0.405543	0.3519141	0.1344306	0.09201128	0.124704	0.1425751
1281.31	11	0.392328	0.3534296	0.1359441	0.09273671	0.126229	0.1429814
1281.41	11	0.425924	0.3391116	0.1353293	0.09292378	0.127817	0.1403117
1281.51	11	0.4015	0.3376243	0.1321618	0.09262977	0.124704	0.1409984
1281.61	11	0.401903	0.3409511	0.1340707	0.09284743	0.127065	0.1417034
1281.71	11	0.383225	0.3434683	0.1322615	0.0909357	0.125736	0.1425216
1281.81	11	0.387785	0.3426484	0.1313086	0.09118608	0.128038	0.145652
1281.91	11	0.395319	0.3437092	0.1371092	0.09096391	0.12998	0.1435566
1282.01	11	0.386081	0.3437967	0.1428652	0.09175765	0.130309	0.1445283
1282.11	11	0.370422	0.3405828	0.1434277	0.09125044	0.131938	0.1449816
1282.21	11	0.384492	0.3346561	0.1414536	0.09159304	0.12962	0.1439739
1282.31	11	0.386724	0.3279136	0.1411584	0.09122337	0.130169	0.142204
1282.41	11	0.403246	0.3288699	0.1387056	0.09137277	0.128363	0.1397928
1282.51	11	0.406048	0.3212237	0.1376135	0.09135083	0.128134	0.1410615
1282.61	11	0.397559	0.3044972	0.1373001	0.09315991	0.128309	0.1423458
1282.71	11	0.382242	0.3240205	0.1374497	0.09147942	0.128163	0.142745
1282.81	11	0.385552	0.3156331	0.1364323	0.09289499	0.12846	0.1446581
1282.91	11	0.381559	0.3291432	0.1353113	0.0939173	0.131902	0.14485
1283.01	11	0.379312	0.3475361	0.1357646	0.09215771	0.130212	0.1450705
1283.11	11	0.378156	0.3542151	0.1358932	0.09412683	0.12769	0.1466233
1283.21	11	0.383062	0.3556562	0.1348094	0.09366679	0.12514	0.1493588
1283.31	11	0.393752	0.3422418	0.1346061	0.09342582	0.124429	0.1490892
1283.41	11	0.38499	0.3301757	0.1344481	0.09207337	0.12395	0.1488487
1283.51	11	0.384624	0.3336336	0.1379903	0.09526014	0.125479	0.1486687
1283.61	11	0.390233	0.3418403	0.1352307	0.09568533	0.123385	0.1497396
1283.71	11	0.382669	0.3474727	0.1338209	0.09350494	0.120134	0.1482271
1283.81	11	0.386042	0.341688	0.1336571	0.09214544	0.118316	0.1465341
1283.91	11	0.38666	0.3563174	0.1325588	0.09648153	0.119936	0.1447594
1284.01	11	0.390421	0.3461541	0.1328272	0.09929901	0.117919	0.1445445
1284.11	11	0.395167	0.3370946	0.1367204	0.1005023	0.117587	0.1489083
1284.21	11	0.395635	0.3380148	0.1352811	0.1013696	0.119218	0.1458639
1284.31	11	0.383266	0.3405887	0.1356365	0.0997967	0.118955	0.144474
1284.41	11	0.387496	0.3429392	0.1346559	0.09614971	0.12107	0.1444418
1284.51	11	0.392082	0.3377167	0.1330315	0.09430398	0.121323	0.1432986
1284.61	11	0.387016	0.3403054	0.1318536	0.09487609	0.118555	0.1445082
1284.71	11	0.383476	0.3385226	0.132843	0.09372055	0.118636	0.1424978
1284.81	11	0.374426	0.3349396	0.1325569	0.0947128	0.117701	0.1447528
1284.91	11	0.389714	0.3289801	0.1345742	0.09788136	0.115986	0.1464396
1285.01	11	0.392082	0.3279808	0.1343871	0.09840569	0.117572	0.1453961
1285.11	11	0.382252	0.3247972	0.1333593	0.09592688	0.117664	0.1437394
1285.21	11	0.393724	0.3121934	0.1344433	0.09374094	0.119536	0.144803
1285.31	11	0.387091	0.3314767	0.1370635	0.09539487	0.118324	0.1427279
1285.41	11	0.369962	0.32676	0.1372196	0.09531981	0.116377	0.1413818
1285.51	11	0.394004	0.3288255	0.1374436	0.09239062	0.116575	0.1422125
1285.61	11	0.398181	0.3364306	0.1352892	0.09307097	0.120439	0.1423268
1285.71	11	0.388707	0.3607852	0.1332029	0.09091796	0.120892	0.1403288
1285.81	11	0.397173	0.356888	0.1303394	0.09152254	0.122936	0.14126
1285.91	11	0.385738	0.3588884	0.1298442	0.09095489	0.122501	0.1417479
1286.01	11	0.375869	0.3415642	0.1309463	0.09129816	0.121383	0.1414064
1286.11	11	0.393307	0.3371577	0.1293847	0.09253061	0.123276	0.14332
1286.21	11	0.391082	0.3424555	0.126013	0.09223919	0.126314	0.1450405
1286.31	11	0.394523	0.3481088	0.1266841	0.09512714	0.128223	0.1436499
1286.41	11	0.387253	0.3502192	0.1291497	0.09619045	0.124431	0.1424959
1286.51	11	0.391076	0.3507916	0.1304333	0.09206892	0.127448	0.143609
1286.61	11	0.385787	0.3591035	0.1287459	0.09288652	0.128164	0.1424268
1286.71	11	0.386356	0.346338	0.1301797	0.09148053	0.129076	0.1419422
1286.81	11	0.404294	0.3447948	0.1275989	0.09175704	0.131101	0.140971
1286.91	11	0.427475	0.347899	0.1277107	0.0896733	0.128257	0.1416813
1287.01	11	0.402737	0.3533845	0.1287508	0.0917877	0.127595	0.1423974
1287.11	11	0.392195	0.3544091	0.1260434	0.09388925	0.124846	0.1459436
1287.21	11	0.391559	0.3511245	0.1259504	0.09459435	0.125246	0.1430177
1287.31	11	0.406526	0.3536295	0.1283158	0.09496403	0.12496	0.1414308
1287.41	11	0.40576	0.3537384	0.1267053	0.09221626	0.12292	0.1430714
1287.51	11	0.393211	0.346113	0.1283058	0.09176926	0.126778	0.1432251
1287.61	11	0.40031	0.3369629	0.1286461	0.09265	0.127865	0.1414018
1287.71	11	0.385125	0.3322113	0.1284069	0.0911055	0.130992	0.1419972
1287.81	11	0.401133	0.3256835	0.1289738	0.09153164	0.131949	0.1406239
1287.91	11	0.408067	0.3069864	0.1337497	0.08848922	0.131335	0.1438919
1288.01	11	0.41171	0.3222693	0.1353372	0.08885588	0.127622	0.1426596
1288.11	11	0.413188	0.3179623	0.1350569	0.09012843	0.125456	0.1435372
1288.21	11	0.38297	0.3206303	0.1355714	0.0916191	0.127331	0.1413442
1288.31	11	0.38638	0.3448797	0.138284	0.09173588	0.128447	0.1412053
1288.41	11	0.37843	0.3557509	0.1343172	0.09053504	0.130159	0.1409697
1288.51	11	0.37638	0.3602598	0.1343014	0.08766873	0.127714	0.1427415
1288.61	11	0.383721	0.3456302	0.1386642	0.08694275	0.128277	0.1429517
1288.71	11	0.386598	0.3346741	0.1372088	0.09001355	0.130613	0.1413071
1288.81	11	0.401018	0.3339608	0.139038	0.09101368	0.127792	0.1440699
1288.91	11	0.392447	0.3396317	0.1400736	0.09585866	0.125827	0.1453243
1289.01	11	0.392346	0.3485565	0.1376658	0.09761886	0.124413	0.1431533
1289.11	11	0.391477	0.347101	0.1355872	0.09795344	0.125025	0.1451897
1289.21	11	0.385511	0.3562838	0.1367003	0.0955323	0.124911	0.1447882
1289.31	11	0.381437	0.3536975	0.135059	0.09747713	0.121833	0.1424176
1289.41	11	0.397685	0.341581	0.135385	0.09842733	0.119524	0.1457211
1289.51	11	0.3947	0.3412915	0.1362054	0.09701624	0.115323	0.1483068
1289.61	11	0.394568	0.3385504	0.1350434	0.100464	0.118533	0.1475898
1289.71	11	0.391563	0.3426937	0.1323546	0.1000107	0.11962	0.1478013
1289.81	11	0.385607	0.3417332	0.133844	0.09973428	0.118948	0.1463973
1289.91	11	0.394698	0.3412552	0.1331586	0.09776421	0.119747	0.148047

1290.01	11	0.389591	0.3406074	0.1300326	0.09733738	0.114885	0.1478563
1290.11	11	0.390056	0.3414805	0.1301773	0.09457006	0.115864	0.1458276
1290.21	11	0.381793	0.3355715	0.1294178	0.09421472	0.117363	0.1447251
1290.31	11	0.39178	0.3307074	0.1301336	0.09392843	0.117096	0.1436694
1290.41	11	0.41278	0.327741	0.1341034	0.09671131	0.118375	0.1420926
1290.51	11	0.416453	0.3167739	0.1354582	0.09589015	0.121105	0.1425405
1290.61	11	0.3854	0.3090563	0.1363564	0.09498144	0.121152	0.1421933
1290.71	11	0.401067	0.318327	0.1368505	0.09600943	0.120335	0.1439774
1290.81	11	0.386762	0.3205437	0.1333555	0.09746183	0.117685	0.1451366
1290.91	11	0.37788	0.3269012	0.1323017	0.09664752	0.121817	0.1447699
1291.01	11	0.386182	0.3525651	0.1345917	0.09743188	0.12419	0.1403548
1291.11	11	0.389295	0.3570569	0.1340585	0.09704864	0.123093	0.140458
1291.21	11	0.385164	0.3638512	0.132366	0.09615466	0.124576	0.1453951
1291.31	11	0.388905	0.3424568	0.1336246	0.09517811	0.12209	0.1426985
1291.41	11	0.392543	0.3339888	0.1332301	0.0958504	0.124801	0.1425897
1291.51	11	0.382588	0.335505	0.1355263	0.0951904	0.124852	0.1424391
1291.61	11	0.409603	0.3439476	0.1364435	0.09542517	0.127682	0.1396367
1291.71	11	0.407545	0.3475781	0.1341184	0.09286191	0.126605	0.1404113
1291.81	11	0.394063	0.345478	0.1328118	0.09856349	0.125623	0.1436682
1291.91	11	0.395308	0.3593317	0.1312564	0.0962522	0.12443	0.1454872
1292.01	11	0.39132	0.3485597	0.1309797	0.09560604	0.123426	0.1439101
1292.11	11	0.389744	0.339943	0.1302248	0.09447516	0.123107	0.1444349
1292.21	11	0.386638	0.3409941	0.1296933	0.09616227	0.124559	0.141727
1292.31	11	0.418732	0.3429712	0.1308825	0.09474063	0.128044	0.142683
1292.41	11	0.402933	0.3435985	0.1287024	0.09594858	0.129543	0.1424472
1292.51	11	0.398631	0.3421955	0.1244733	0.09739076	0.129471	0.1436346
1292.61	11	0.382665	0.3460038	0.1266728	0.09623923	0.131345	0.1383349
1292.71	11	0.394615	0.3437701	0.1292645	0.09366984	0.126638	0.1389856
1292.81	11	0.404548	0.3401262	0.1303875	0.09484847	0.127971	0.141486
1292.91	11	0.398634	0.3326411	0.1275507	0.09588434	0.130801	0.140988
1293.01	11	0.380631	0.3293798	0.1307635	0.09655605	0.133211	0.1400394
1293.11	11	0.386077	0.3242575	0.128755	0.09705668	0.133732	0.1410134
1293.21	11	0.371924	0.3090868	0.1278014	0.09725493	0.132651	0.1406897
1293.31	11	0.39992	0.3109387	0.1285611	0.09477251	0.128768	0.1414996
1293.41	11	0.416967	0.317987	0.1266867	0.09337825	0.128187	0.1439131
1293.51	11	0.436791	0.3271589	0.1283894	0.09239996	0.129213	0.1418128
1293.61	11	0.389355	0.3333173	0.1294626	0.09090487	0.128524	0.140383
1293.71	11	0.386582	0.3526281	0.1280361	0.09579746	0.127315	0.1402795
1293.81	11	0.380058	0.3619204	0.1283007	0.09381709	0.128026	0.1378776
1293.91	11	0.386423	0.3549852	0.1284353	0.09067478	0.129613	0.1368018
1294.01	11	0.380087	0.3378774	0.1312155	0.09099324	0.127569	0.1376196
1294.11	11	0.387971	0.3277587	0.1328731	0.09144196	0.125434	0.134753
1294.21	11	0.397165	0.3360288	0.1364464	0.09422148	0.122406	0.1384462
1294.31	11	0.393386	0.3465023	0.1362052	0.09778433	0.122008	0.1387467
1294.41	11	0.395675	0.3487863	0.1380661	0.09887923	0.118188	0.1386731
1294.51	11	0.391225	0.3517686	0.1392963	0.09889972	0.118357	0.1389138
1294.61	11	0.385506	0.359671	0.1406578	0.09769268	0.117396	0.1425308
1294.71	11	0.388985	0.3451848	0.1387902	0.09693191	0.115728	0.1419591
1294.81	11	0.385362	0.3385085	0.1390999	0.09731001	0.116079	0.1420905
1294.91	11	0.38791	0.3398447	0.1391019	0.0980909	0.114324	0.1422253
1295.01	11	0.391713	0.3405344	0.1380886	0.09885145	0.113686	0.1392518
1295.11	11	0.391042	0.3418038	0.1399268	0.1006445	0.115873	0.1382079
1295.21	11	0.384127	0.3413965	0.1431485	0.1014428	0.113015	0.1408651
1295.31	11	0.378927	0.3421102	0.1418726	0.09770853	0.114394	0.1427804
1295.41	11	0.394628	0.344805	0.1386629	0.09784498	0.116751	0.1459056
1295.51	11	0.424615	0.3382652	0.1376983	0.1001607	0.115695	0.1467048
1295.61	11	0.413272	0.3320394	0.1368669	0.09832954	0.113662	0.1473423
1295.71	11	0.414794	0.3305607	0.1372476	0.09793485	0.11365	0.1487947
1295.81	11	0.404385	0.319522	0.1387653	0.09921105	0.115169	0.1497347
1295.91	11	0.395149	0.3071908	0.1364174	0.1004444	0.114672	0.1475531
1296.01	11	0.390455	0.316642	0.1346402	0.1005193	0.116384	0.1503319
1296.11	11	0.391648	0.3175116	0.1364739	0.09823675	0.115953	0.1528707
1296.21	11	0.382445	0.327932	0.1363582	0.09951983	0.11583	0.1523824
1296.31	11	0.36799	0.3461311	0.1344999	0.09890977	0.115828	0.1510908
1296.41	11	0.381951	0.3577499	0.1357743	0.09430686	0.123991	0.1472441
1296.51	11	0.387187	0.3636592	0.1324143	0.09679218	0.124032	0.1434238
1296.61	11	0.381733	0.3474476	0.1319208	0.09519193	0.12304	0.1427122
1296.71	11	0.391702	0.329717	0.1369393	0.09563504	0.121716	0.1420243
1296.81	11	0.386091	0.3291647	0.1360833	0.09545755	0.12305	0.1420222
1296.91	11	0.380184	0.3359634	0.1366366	0.09689342	0.125479	0.1428574
1297.01	11	0.392795	0.354222	0.1367378	0.09582925	0.130302	0.1422013
1297.11	11	0.392838	0.3515731	0.1364933	0.09302546	0.133968	0.1429036
1297.21	11	0.405518	0.3627974	0.1348984	0.09565436	0.131827	0.1426904
1297.31	11	0.391061	0.3571617	0.1324861	0.09823098	0.135068	0.1396307
1297.41	11	0.396633	0.3471943	0.1321408	0.09409819	0.135402	0.1407783
1297.51	11	0.40135	0.3481611	0.1334327	0.09414485	0.13446	0.1447348
1297.61	11	0.392604	0.3485951	0.1355862	0.09367961	0.133064	0.143468
1297.71	11	0.402251	0.3500896	0.1361057	0.09396177	0.131234	0.1445713
1297.81	11	0.422773	0.3483499	0.1346065	0.09320697	0.129725	0.1460619
1297.91	11	0.393604	0.3485848	0.1337051	0.09323911	0.127239	0.1434974
1298.01	11	0.39843	0.3454739	0.133205	0.09401454	0.126999	0.1408885
1298.11	11	0.385887	0.3459207	0.1329679	0.09429514	0.127718	0.1425676
1298.21	11	0.40216	0.3378121	0.132952	0.09499293	0.127099	0.1439985
1298.31	11	0.401333	0.3329433	0.1298124	0.09294321	0.129492	0.1417526
1298.41	11	0.375096	0.3298875	0.1308232	0.0933426	0.128762	0.1401514
1298.51	11	0.381883	0.3134732	0.1317596	0.09710666	0.130803	0.1384382
1298.61	11	0.394395	0.3129688	0.1330427	0.0969178	0.12884	0.1381435
1298.71	11	0.388117	0.3192452	0.1329701	0.09602962	0.128405	0.1367888
1298.81	11	0.407096	0.3225222	0.1272059	0.09013088	0.124477	0.1390051
1298.91	11	0.412221	0.3285	0.1282316	0.08727075	0.123485	0.1393344
1299.01	11	0.406209	0.3550978	0.1278814	0.0866475	0.122822	0.1374514
1299.11	11	0.38317	0.3529475	0.1295947	0.08758466	0.123939	0.1388982
1299.21	11	0.381497	0.3628777	0.1282767	0.08837189	0.121736	0.1381281
1299.31	11	0.378487	0.3408636	0.1311293	0.08577625	0.120494	0.139509
1299.41	11	0.378524	0.3292824	0.1319607	0.08664916	0.121856	0.1384494

1299.51	11	0.382984	0.3284251	0.1314511	0.09017901	0.1208	0.1376828
1299.61	11	0.382694	0.3393422	0.132189	0.09126988	0.119619	0.1389897
1299.71	11	0.396801	0.3483855	0.1299797	0.09323046	0.12008	0.1411728
1299.81	11	0.392832	0.3520965	0.1273636	0.09775858	0.119669	0.1402066
1299.91	11	0.401463	0.3635538	0.1285005	0.1016636	0.121134	0.1385105
1300.01	11	0.392465	0.3505903	0.1292118	0.103369	0.115965	0.1371051
1300.11	11	0.390825	0.3413925	0.1285108	0.1022614	0.114396	0.1379091
1300.21	11	0.383224	0.3411546	0.1289646	0.1035572	0.115518	0.1363299
1300.31	11	0.388695	0.3440175	0.1293616	0.1017437	0.116676	0.1387957
1300.41	11	0.385619	0.3460646	0.1302562	0.1008744	0.114247	0.1347007
1300.51	11	0.38993	0.3438974	0.1303981	0.1047341	0.11269	0.1355187
1300.61	11	0.390642	0.3464428	0.1315706	0.1040168	0.114136	0.1376907
1300.71	11	0.380656	0.3452898	0.1344678	0.1000767	0.113919	0.1372948
1300.81	11	0.380524	0.3380971	0.1362603	0.1000775	0.115361	0.1375687
1300.91	11	0.389594	0.3307993	0.1372594	0.1014349	0.11527	0.139774
1301.01	11	0.393144	0.3295622	0.1356614	0.1015048	0.114418	0.1423308
1301.11	11	0.385136	0.3230172	0.1370247	0.1023849	0.112076	0.1424668
1301.21	11	0.387943	0.3037152	0.1367983	0.1018432	0.114763	0.1401628
1301.31	11	0.399363	0.3034417	0.1367908	0.1006417	0.115918	0.1396651
1301.41	11	0.402263	0.312279	0.138399	0.100284	0.118148	0.1395243
1301.51	11	0.384451	0.3174557	0.1396975	0.1007456	0.121946	0.1431403
1301.61	11	0.39256	0.3301701	0.1412488	0.1005798	0.121898	0.144834
1301.71	11	0.386702	0.3606726	0.1376526	0.1008535	0.116692	0.1462917
1301.81	11	0.388703	0.358007	0.1374814	0.1004636	0.116144	0.1483851
1301.91	11	0.390407	0.3563773	0.1380677	0.1008487	0.117189	0.1499022
1302.01	11	0.392628	0.3383575	0.136911	0.1010425	0.116197	0.1477599
1302.11	11	0.390183	0.3245652	0.1374703	0.1007784	0.118017	0.1477424
1302.21	11	0.389041	0.3300324	0.1354431	0.1006442	0.120819	0.1475644
1302.31	11	0.391194	0.3437493	0.1357206	0.1013547	0.122467	0.1449707
1302.41	11	0.376553	0.3493741	0.133774	0.0989722	0.121804	0.1453579
1302.51	11	0.39982	0.3518852	0.1346768	0.09807485	0.125133	0.1446912
1302.61	11	0.405347	0.3602169	0.1320042	0.09844395	0.124883	0.1457276
1302.71	11	0.395618	0.35122	0.135038	0.09983229	0.12677	0.1434089
1302.81	11	0.403037	0.3446558	0.1320662	0.09463659	0.129347	0.1421019
1302.91	11	0.395985	0.3432507	0.1307748	0.09404495	0.129725	0.1409494
1303.01	11	0.389587	0.3450202	0.1333867	0.09270617	0.131814	0.1404848
1303.11	11	0.388214	0.3445958	0.1349165	0.09657709	0.130963	0.139764
1303.21	11	0.419137	0.3451532	0.136058	0.09501901	0.129664	0.1369295
1303.31	11	0.400792	0.3434883	0.1357781	0.09577742	0.129894	0.1383362
1303.41	11	0.403323	0.3423032	0.1364167	0.0971795	0.127889	0.1399676
1303.51	11	0.382904	0.3320942	0.1349461	0.09587236	0.126349	0.1389602
1303.61	11	0.392378	0.3267169	0.1335531	0.09448184	0.123918	0.1382573
1303.71	11	0.399788	0.3254888	0.131763	0.09659784	0.122888	0.1390788
1303.81	11	0.387626	0.3206498	0.1335117	0.09754306	0.124199	0.1442039
1303.91	11	0.367037	0.3005331	0.135586	0.09920948	0.128406	0.1438328
1304.01	11	0.385167	0.3098558	0.1356623	0.09964485	0.126434	0.140874
1304.11	11	0.375098	0.3101051	0.1349247	0.1000418	0.122871	0.1410859
1304.21	11	0.401202	0.3118436	0.1353544	0.09738896	0.120619	0.142137
1304.31	11	0.438792	0.3428878	0.132019	0.09488352	0.12192	0.1376275
1304.41	11	0.42867	0.3561744	0.1315228	0.09575649	0.123848	0.1391466
1304.51	11	0.394961	0.3580706	0.1324334	0.09248618	0.123187	0.1420321
1304.61	11	0.394982	0.3516786	0.128976	0.09542306	0.123753	0.1426034
1304.71	11	0.38467	0.3310564	0.12929	0.09636093	0.126236	0.1412878
1304.81	11	0.393366	0.3227597	0.1298078	0.0935216	0.125797	0.139041
1304.91	11	0.38575	0.3315071	0.1305332	0.09510988	0.123236	0.1408955
1305.01	11	0.393054	0.3465774	0.131414	0.09714363	0.122472	0.1387871
1305.11	11	0.390432	0.3510936	0.1281526	0.09761149	0.120398	0.1402845
1305.21	11	0.391277	0.3554148	0.1280367	0.09953349	0.121704	0.1392505
1305.31	11	0.392343	0.3593894	0.12709	0.1008013	0.119916	0.1380073
1305.41	11	0.390455	0.3477051	0.1289079	0.1011523	0.121634	0.1401958
1305.51	11	0.385213	0.3443941	0.1273272	0.0997603	0.121448	0.1421959
1305.61	11	0.394137	0.3423178	0.1273201	0.1039019	0.118659	0.141839
1305.71	11	0.381572	0.3424073	0.1296072	0.1078819	0.119063	0.1404531
1305.81	11	0.383291	0.3464785	0.1255695	0.1061985	0.11786	0.1406357
1305.91	11	0.389245	0.3474253	0.1262882	0.1067904	0.116389	0.143091
1306.01	11	0.394446	0.3467702	0.1263888	0.1079286	0.118568	0.1441444
1306.11	11	0.383486	0.3414559	0.1245659	0.1074208	0.118712	0.1429994
1306.21	11	0.377162	0.3299523	0.1261805	0.1034197	0.120078	0.1408091
1306.31	11	0.383231	0.3255705	0.1297259	0.1041353	0.119622	0.139284
1306.41	11	0.387293	0.3255942	0.1284521	0.09971628	0.114105	0.1406772
1306.51	11	0.385725	0.3107897	0.1296996	0.09773327	0.112694	0.1399105
1306.61	11	0.380598	0.3047464	0.1299044	0.09832345	0.113487	0.1412182
1306.71	11	0.386874	0.3113371	0.1288293	0.09763305	0.114139	0.1381104
1306.81	11	0.391416	0.3115382	0.1302945	0.09789041	0.116679	0.1390027
1306.91	11	0.390253	0.3173329	0.1347113	0.09822024	0.118511	0.1414974
1307.01	11	0.385839	0.3538046	0.1354252	0.1009373	0.117464	0.138329
1307.11	11	0.377254	0.3517303	0.1350549	0.09965622	0.116215	0.1381345
1307.21	11	0.366469	0.3624175	0.1350129	0.1006323	0.112587	0.1426439
1307.31	11	0.38319	0.3428819	0.1356472	0.09916033	0.116819	0.1418846
1307.41	11	0.385687	0.3233744	0.1386653	0.09927508	0.120404	0.141775
1307.51	11	0.383507	0.3242524	0.139543	0.09908908	0.119516	0.1401424
1307.61	11	0.386836	0.3385077	0.1392442	0.1003197	0.118785	0.1379186
1307.71	11	0.385106	0.3479556	0.1403026	0.1013787	0.11978	0.1398938
1307.81	11	0.379128	0.3511117	0.141775	0.09937231	0.124671	0.1408374
1307.91	11	0.391957	0.3635621	0.1433504	0.09819987	0.126146	0.1423507
1308.01	11	0.396872	0.3605765	0.1392285	0.09748578	0.127749	0.1448198
1308.11	11	0.412028	0.3478729	0.1410483	0.09907032	0.127872	0.146412
1308.21	11	0.397053	0.3442557	0.1396408	0.09611972	0.131273	0.1486572
1308.31	11	0.393544	0.3429742	0.137402	0.09582987	0.130925	0.1468093
1308.41	11	0.394717	0.342705	0.1387662	0.09485065	0.129125	0.146313
1308.51	11	0.393501	0.3445055	0.1350217	0.0938617	0.129531	0.1483166
1308.61	11	0.398748	0.3447343	0.1349602	0.09479689	0.127395	0.1504596
1308.71	11	0.422069	0.3451295	0.1345193	0.09458522	0.127187	0.1477149
1308.81	11	0.388614	0.3371846	0.1381745	0.09494965	0.127924	0.1443376
1308.91	11	0.400104	0.327986	0.1346434	0.1006975	0.126482	0.1471877

1309.01	11	0.386924	0.3261514	0.1365429	0.09782025	0.127003	0.1432558
1309.11	11	0.39807	0.3217562	0.1350402	0.09892586	0.124752	0.1418384
1309.21	11	0.394265	0.3074642	0.1332259	0.09732216	0.1258	0.1437766
1309.31	11	0.376986	0.2999388	0.1333251	0.09982219	0.125882	0.1445649
1309.41	11	0.368742	0.3042816	0.1347385	0.0995195	0.129789	0.1476096
1309.51	11	0.386913	0.3125023	0.1341489	0.09809357	0.12748	0.1441402
1309.61	11	0.382866	0.3236631	0.1356303	0.09716995	0.130153	0.1439336
1309.71	11	0.38915	0.354877	0.1365353	0.09476372	0.129634	0.1439286
1309.81	11	0.39622	0.3456644	0.1366702	0.09355934	0.129959	0.1447166
1309.91	11	0.393812	0.350751	0.1383916	0.09302112	0.1302	0.1431598
1310.01	11	0.387337	0.3344064	0.1371139	0.09613679	0.12868	0.1420291
1310.11	11	0.383825	0.3199436	0.1382052	0.09790638	0.127867	0.1444921
1310.21	11	0.381001	0.3303235	0.136998	0.09705324	0.129246	0.1436328
1310.31	11	0.382572	0.3426213	0.1367052	0.09573342	0.127783	0.1436525
1310.41	11	0.383177	0.3482461	0.1350167	0.09488544	0.125503	0.1434275
1310.51	11	0.389059	0.3526105	0.1348037	0.09828529	0.12615	0.1416644
1310.61	11	0.39167	0.3607979	0.1340656	0.1006276	0.127271	0.1384956
1310.71	11	0.38991	0.3530501	0.1337739	0.0988035	0.123886	0.1386927
1310.81	11	0.394023	0.3469433	0.1347767	0.09817071	0.124519	0.1404818
1310.91	11	0.393647	0.3493049	0.1320104	0.09967127	0.122804	0.1411801
1311.01	11	0.383753	0.3486782	0.1340205	0.09710232	0.120613	0.1418966
1311.11	11	0.387222	0.3492494	0.1333936	0.09802561	0.119262	0.141553
1311.21	11	0.382767	0.3503827	0.1328433	0.09959518	0.119905	0.1418582
1311.31	11	0.389167	0.3494515	0.1320942	0.09999525	0.116924	0.1392774
1311.41	11	0.392809	0.3493543	0.1299423	0.09977028	0.117343	0.1390334
1311.51	11	0.396551	0.3361011	0.1307195	0.09996427	0.11947	0.1409357
1311.61	11	0.381432	0.3265319	0.1294943	0.1027635	0.120792	0.1383908
1311.71	11	0.382251	0.3255236	0.1301731	0.1016474	0.119227	0.1391527
1311.81	11	0.388561	0.316456	0.1297699	0.09938371	0.117861	0.1393234
1311.91	11	0.3907	0.3034319	0.1302395	0.09875422	0.114019	0.1389986
1312.01	11	0.425176	0.3014165	0.131384	0.09915876	0.117153	0.1406626
1312.11	11	0.396213	0.3047645	0.1278488	0.09996583	0.118187	0.1422968
1312.21	11	0.404556	0.3064096	0.1263007	0.0961081	0.116283	0.1441657
1312.31	11	0.427758	0.3360773	0.1280109	0.0963392	0.117766	0.1447018
1312.41	11	0.415248	0.3509043	0.1283166	0.09683523	0.120868	0.1462407
1312.51	11	0.440996	0.352113	0.1301985	0.09794114	0.122918	0.1433408
1312.61	11	0.427534	0.3466048	0.1316157	0.09711335	0.118046	0.1426145
1312.71	11	0.408759	0.3248788	0.1304116	0.09758378	0.117697	0.1402244
1312.81	11	0.416133	0.3196113	0.1316448	0.0971905	0.119263	0.1384114
1312.91	11	0.419154	0.3305879	0.1330574	0.09827828	0.1228	0.1386251
1313.01	11	0.400655	0.343531	0.1320347	0.09854235	0.127567	0.1352743
1313.11	11	0.403719	0.3468446	0.1318725	0.0996976	0.126145	0.1339431
1313.21	11	0.410794	0.3638043	0.1348143	0.09922828	0.128737	0.1381173
1313.31	11	0.384665	0.3607365	0.1364801	0.09735353	0.126748	0.1371417
1313.41	11	0.403292	0.3489453	0.1375	0.09668968	0.130895	0.1376706
1313.51	11	0.401692	0.344637	0.1394531	0.096379	0.131892	0.1378406
1313.61	11	0.399479	0.3437916	0.136906	0.09852054	0.134846	0.1386212
1313.71	11	0.398028	0.3412404	0.1407573	0.09619195	0.135799	0.1367572
1313.81	11	0.395082	0.3422952	0.1388829	0.09508532	0.134467	0.1374747
1313.91	11	0.387462	0.3432906	0.137351	0.09550819	0.13646	0.1380943
1314.01	11	0.389561	0.3404205	0.1378247	0.09570547	0.13657	0.1379774
1314.11	11	0.413042	0.3368634	0.1423099	0.09369953	0.134459	0.141473
1314.21	11	0.408314	0.330111	0.1438838	0.09540668	0.134043	0.141747
1314.31	11	0.400033	0.3245192	0.1387569	0.09687657	0.130977	0.1435022
1314.41	11	0.391314	0.3235706	0.1392289	0.09614229	0.131312	0.1438737
1314.51	11	0.390487	0.3073102	0.139187	0.09748308	0.130621	0.1457775
1314.61	11	0.407156	0.3007202	0.1377503	0.09816082	0.130151	0.1449163
1314.71	11	0.394705	0.3048297	0.1391894	0.09671607	0.132841	0.147012
1314.81	11	0.373493	0.3079026	0.1354952	0.09811043	0.133521	0.148676
1314.91	11	0.381872	0.3178408	0.1348013	0.09887407	0.132392	0.1469146
1315.01	11	0.375418	0.3490157	0.1350492	0.0967363	0.129531	0.148738
1315.11	11	0.388274	0.3435622	0.139411	0.09506334	0.12741	0.1477414
1315.21	11	0.399596	0.3566056	0.1332684	0.09507179	0.129007	0.1489731
1315.31	11	0.399997	0.3383162	0.1323678	0.09542868	0.126672	0.1452406
1315.41	11	0.387121	0.3191703	0.132912	0.09519215	0.123828	0.142534
1315.51	11	0.389436	0.3247263	0.1330537	0.09619318	0.126445	0.1410649
1315.61	11	0.384158	0.3400698	0.134046	0.09548581	0.125872	0.1410947
1315.71	11	0.384118	0.3493795	0.1377229	0.0939635	0.125456	0.1439278
1315.81	11	0.378866	0.3515573	0.1357706	0.09540746	0.124701	0.1421887
1315.91	11	0.389588	0.3698043	0.1355568	0.09376956	0.123915	0.1413036
1316.01	11	0.389056	0.355139	0.1335477	0.09774755	0.124249	0.1409581
1316.11	11	0.387638	0.3457091	0.1340326	0.09636194	0.125815	0.1416425
1316.21	11	0.391876	0.3436784	0.134765	0.100316	0.127319	0.1402634
1316.31	11	0.383161	0.3431823	0.1332642	0.09824336	0.127793	0.1393179
1316.41	11	0.382317	0.3433968	0.1331583	0.09443528	0.128014	0.1417783
1316.51	11	0.386586	0.3456271	0.1313878	0.09652176	0.125376	0.1441844
1316.61	11	0.374346	0.3447866	0.1338731	0.1015353	0.124514	0.1424629
1316.71	11	0.379704	0.3421074	0.1339201	0.0998586	0.12317	0.1422765
1316.81	11	0.385402	0.3343673	0.1326536	0.09740026	0.121035	0.1440543
1316.91	11	0.390089	0.3250319	0.1316149	0.09818391	0.121539	0.1413015
1317.01	11	0.390103	0.3251089	0.1299608	0.1019199	0.120812	0.1406468
1317.11	11	0.377924	0.320786	0.1321287	0.09717426	0.120145	0.1432902
1317.21	11	0.381382	0.3055362	0.1317399	0.09706745	0.119898	0.1402879
1317.31	11	0.382625	0.3018022	0.1323331	0.09702097	0.117853	0.1403242
1317.41	11	0.387705	0.3029631	0.1324668	0.09585787	0.117598	0.1405172
1317.51	11	0.3884	0.3077979	0.1318461	0.09783829	0.119421	0.139103
1317.61	11	0.379376	0.3281342	0.1301513	0.09832159	0.119162	0.1368528
1317.71	11	0.390055	0.3527572	0.1295841	0.09771568	0.115224	0.1386981
1317.81	11	0.391403	0.3404571	0.1294613	0.09579935	0.117485	0.143913
1317.91	11	0.387631	0.3516889	0.1279441	0.0975325	0.11815	0.1408353
1318.01	11	0.383469	0.3325972	0.1305038	0.09941293	0.119447	0.1432307
1318.11	11	0.369723	0.3193565	0.1294439	0.09839001	0.1175	0.1420752
1318.21	11	0.377907	0.3267014	0.1312546	0.09477073	0.114981	0.1411815
1318.31	11	0.38594	0.3425016	0.1330368	0.09498084	0.117385	0.1403027
1318.41	11	0.390684	0.3476141	0.1310042	0.09543262	0.11977	0.1411558

1318.51	11	0.382447	0.3568203	0.1286104	0.09900993	0.120391	0.1401109
1318.61	11	0.39379	0.3712301	0.1262954	0.09822159	0.120509	0.1398657
1318.71	11	0.397092	0.3536834	0.1266149	0.09867116	0.126796	0.1429406
1318.81	11	0.406634	0.3492114	0.1288393	0.095507	0.132129	0.1439675
1318.91	11	0.398035	0.348606	0.1317236	0.09388074	0.132742	0.1422144
1319.073	11.5	0.398149	0.353785	0.1329823	0.09521224	0.130212	0.1413503
1319.173	11.5	0.395125	0.3504215	0.1366544	0.09643625	0.127781	0.139806
1319.273	11.5	0.395843	0.3448812	0.1362377	0.09171791	0.128812	0.1401719
1319.373	11.5	0.409102	0.335382	0.1350502	0.09091091	0.127898	0.1390399
1319.473	11.5	0.405198	0.3259946	0.1350047	0.09025214	0.127838	0.1334546
1319.573	11.5	0.398016	0.324764	0.1373453	0.09284031	0.127861	0.1372527
1319.673	11.5	0.390988	0.31348	0.1375183	0.09675486	0.128607	0.1365408
1319.773	11.5	0.398753	0.2990271	0.1374324	0.09361596	0.129307	0.1354462
1319.873	11.5	0.408728	0.3040686	0.1397349	0.0966798	0.127015	0.1349148
1319.973	11.5	0.3929	0.3096392	0.1398394	0.09687741	0.127921	0.1342549
1320.073	11.5	0.38046	0.3230706	0.141178	0.09367853	0.127197	0.1332986
1320.173	11.5	0.385655	0.3560998	0.1419336	0.09664658	0.127306	0.1333086
1320.273	11.5	0.380867	0.3569631	0.1469375	0.09732968	0.126366	0.1342607
1320.373	11.5	0.392634	0.3585833	0.1482278	0.09698301	0.131043	0.1338743
1320.473	11.5	0.399019	0.3526357	0.1426544	0.09368013	0.130586	0.1380138
1320.573	11.5	0.401859	0.3500489	0.1419873	0.09441262	0.129367	0.1389912
1320.673	11.5	0.390368	0.357568	0.1404498	0.09329722	0.124517	0.1410831
1320.773	11.5	0.382816	0.3649434	0.1396817	0.09344652	0.12436	0.1447662
1320.873	11.5	0.376333	0.3710672	0.1402995	0.09411109	0.12404	0.1447997
1320.973	11.5	0.377435	0.354169	0.1376401	0.09388863	0.123362	0.1431518
1321.073	11.5	0.387103	0.3652055	0.1359025	0.09372489	0.12309	0.1465403
1321.173	11.5	0.385615	0.3589455	0.1363052	0.09414577	0.123335	0.1477751
1321.273	11.5	0.390338	0.3615879	0.136511	0.09342871	0.124127	0.1473227
1321.373	11.5	0.388062	0.3675636	0.1348433	0.09339705	0.122425	0.1481754
1321.473	11.5	0.384063	0.3726857	0.138424	0.0946101	0.121255	0.1476357
1321.573	11.5	0.383137	0.375744	0.1355996	0.09577785	0.122695	0.1483489
1321.673	11.5	0.384913	0.3776511	0.136694	0.09449961	0.122991	0.1448384
1321.773	11.5	0.37789	0.3775118	0.1410408	0.09466093	0.12622	0.1423608
1321.873	11.5	0.379242	0.3764721	0.1383184	0.09569066	0.127137	0.1420176
1321.973	11.5	0.383762	0.3665451	0.1372512	0.09849909	0.12547	0.1428392
1322.073	11.5	0.393549	0.3572009	0.135914	0.09733842	0.124068	0.1443313
1322.173	11.5	0.378132	0.3475123	0.1348037	0.09859255	0.123399	0.1408293
1322.273	11.5	0.380214	0.3252223	0.1364122	0.09558617	0.121479	0.1429635
1322.373	11.5	0.383181	0.3070436	0.135969	0.09336834	0.120473	0.1442077
1322.473	11.5	0.386935	0.3098341	0.1366546	0.09359067	0.119589	0.1443558
1322.573	11.5	0.399596	0.3086691	0.134352	0.09619571	0.122156	0.1405834
1322.673	11.5	0.388242	0.3226972	0.1321709	0.1005516	0.121937	0.140272
1322.773	11.5	0.397482	0.3593315	0.1337231	0.1016768	0.120353	0.1397324
1322.873	11.5	0.396174	0.3648928	0.1345813	0.1019088	0.118902	0.1424071
1322.973	11.5	0.384818	0.3545733	0.1339556	0.09935966	0.122566	0.1418938
1323.073	11.5	0.388905	0.3413885	0.1340808	0.09791993	0.119837	0.139801
1323.173	11.5	0.381211	0.3423465	0.1345761	0.0999621	0.116961	0.1392238
1323.273	11.5	0.372905	0.3475785	0.1316672	0.09746385	0.116202	0.1394963
1323.373	11.5	0.39388	0.355855	0.1329259	0.0974054	0.118363	0.1395484
1323.473	11.5	0.400857	0.362257	0.1319203	0.09906926	0.120142	0.1407198
1323.573	11.5	0.398117	0.349589	0.1332027	0.09985868	0.1189	0.1398174
1323.673	11.5	0.417561	0.3559556	0.1316495	0.09916288	0.117334	0.139737
1323.773	11.5	0.4114	0.3440072	0.1289889	0.09922701	0.122376	0.1416716
1323.873	11.5	0.40377	0.3461658	0.1293004	0.09872517	0.1231	0.1423232
1323.973	11.5	0.420714	0.3495973	0.1307513	0.09710535	0.127597	0.1402408
1324.073	11.5	0.429831	0.352984	0.132492	0.09677898	0.129861	0.1410912
1324.173	11.5	0.411174	0.3567876	0.1331706	0.1007472	0.128193	0.1438537
1324.273	11.5	0.406996	0.3586488	0.1340749	0.09736188	0.127842	0.1420998
1324.373	11.5	0.401473	0.3590996	0.1305514	0.09616973	0.133207	0.1408883
1324.473	11.5	0.393475	0.3573217	0.1271871	0.09560874	0.130057	0.1384744
1324.573	11.5	0.404229	0.3467504	0.1265537	0.09500498	0.128605	0.1372338
1324.673	11.5	0.417831	0.3393664	0.1265614	0.09271437	0.128204	0.1387505
1324.773	11.5	0.397597	0.3381247	0.1263416	0.09415264	0.12871	0.1394277
1324.873	11.5	0.39551	0.332876	0.1291085	0.09458742	0.126796	0.1398597
1324.973	11.5	0.391662	0.3684421	0.1291517	0.09512085	0.125418	0.1409255
1325.073	11.5	0.40185	0.3550284	0.1313421	0.09651888	0.123393	0.145359
1325.173	11.5	0.392306	0.3644865	0.1313841	0.09412855	0.122118	0.146262
1325.273	11.5	0.367997	0.371134	0.1283151	0.09465033	0.120896	0.1445369
1325.373	11.5	0.390723	0.3693295	0.1323231	0.09683642	0.117524	0.1417505
1325.473	11.5	0.399863	0.3598588	0.1336321	0.09565955	0.117516	0.1414544
1325.573	11.5	0.40132	0.3628497	0.1347452	0.09519953	0.119413	0.1402358
1325.673	11.5	0.397039	0.3497064	0.1367386	0.09348923	0.120648	0.1413992
1325.773	11.5	0.400859	0.3515337	0.1363865	0.09478331	0.123075	0.1371358
1325.873	11.5	0.390463	0.3570967	0.1369336	0.09473535	0.124212	0.1387815
1325.973	11.5	0.389985	0.3630895	0.1410351	0.09160452	0.12089	0.1399881
1326.073	11.5	0.39536	0.3681472	0.1433213	0.09527238	0.120101	0.1395818
1326.173	11.5	0.377411	0.3576268	0.1437166	0.09543958	0.120119	0.1396245
1326.273	11.5	0.382215	0.35515	0.1437695	0.09767133	0.122859	0.1422322
1326.373	11.5	0.382099	0.352595	0.1432463	0.0980142	0.123545	0.1428031
1326.473	11.5	0.389658	0.354824	0.1406195	0.1017344	0.125904	0.1424011
1326.573	11.5	0.390396	0.3596516	0.1395923	0.1029283	0.125057	0.1414775
1326.673	11.5	0.388242	0.3636853	0.136388	0.1047837	0.126379	0.1399439
1326.773	11.5	0.381724	0.3657361	0.1346477	0.1042932	0.125393	0.1419748
1326.873	11.5	0.383029	0.366068	0.1367512	0.1027626	0.122755	0.141423
1326.973	11.5	0.378851	0.3680915	0.1345419	0.1030959	0.121473	0.1427387
1327.073	11.5	0.381842	0.3619761	0.13406	0.1044832	0.121891	0.1425426
1327.173	11.5	0.38309	0.3521109	0.1360989	0.1053861	0.119348	0.1420072
1327.273	11.5	0.392211	0.3447109	0.136381	0.1038745	0.120657	0.1416773
1327.373	11.5	0.38635	0.3447362	0.1350026	0.1035478	0.12117	0.1443852
1327.473	11.5	0.379243	0.3504915	0.1386388	0.1003059	0.119477	0.1460729
1327.573	11.5	0.381474	0.3613705	0.1358339	0.09892579	0.116982	0.1458613
1327.673	11.5	0.383885	0.3454883	0.1362095	0.1003894	0.116609	0.1449101
1327.773	11.5	0.397803	0.3635873	0.1377769	0.1023117	0.11516	0.1433545
1327.873	11.5	0.390776	0.3803253	0.1360133	0.104861	0.116667	0.1459417
1327.973	11.5	0.388409	0.3617562	0.1366397	0.1050494	0.114763	0.147424

1328.073	11.5	0.390868	0.3685123	0.1361944	0.1033686	0.11473	0.1454779
1328.173	11.5	0.383038	0.3607216	0.13379	0.0993852	0.114141	0.1430209
1328.273	11.5	0.392533	0.3557379	0.1353753	0.09879087	0.110422	0.143521
1328.373	11.5	0.391584	0.3589257	0.1330242	0.0981762	0.109876	0.1425195
1328.473	11.5	0.385453	0.3655036	0.1326351	0.09921829	0.112012	0.1402065
1328.573	11.5	0.397337	0.3726175	0.133513	0.09913864	0.11361	0.1400697
1328.673	11.5	0.405954	0.378859	0.133976	0.09924871	0.115122	0.1398976
1328.773	11.5	0.394175	0.3608116	0.1316725	0.09636207	0.115211	0.1432571
1328.873	11.5	0.428376	0.3534141	0.1319054	0.09798319	0.115899	0.1422677
1328.973	11.5	0.408721	0.3548836	0.1326721	0.09758317	0.114652	0.1422827
1329.073	11.5	0.405052	0.3593385	0.1322772	0.09739444	0.114546	0.1418217
1329.173	11.5	0.43476	0.3662324	0.1322562	0.09700438	0.116585	0.1454825
1329.273	11.5	0.430427	0.3714502	0.1315202	0.09676248	0.121208	0.1440297
1329.373	11.5	0.406408	0.3765823	0.1313401	0.09914879	0.121375	0.1425476
1329.473	11.5	0.408128	0.3777992	0.1300737	0.09573194	0.121118	0.1434983
1329.573	11.5	0.40953	0.3805684	0.1283478	0.09264492	0.122877	0.1415092
1329.673	11.5	0.395801	0.3767117	0.1274004	0.08975586	0.126385	0.1416356
1329.773	11.5	0.401194	0.366802	0.1239478	0.09013129	0.127707	0.1418581
1329.873	11.5	0.418433	0.3533714	0.1268543	0.08854239	0.12934	0.1435792
1329.973	11.5	0.390831	0.3495402	0.1274459	0.09230548	0.127244	0.1430002
1330.073	11.5	0.395133	0.3504859	0.1269492	0.09343584	0.132044	0.1422113
1330.173	11.5	0.388716	0.3559506	0.1262493	0.09072916	0.131594	0.1394285
1330.273	11.5	0.401322	0.3487632	0.1309922	0.09197221	0.133794	0.1391718
1330.373	11.5	0.397807	0.3597116	0.1291008	0.09499579	0.13367	0.1403522
1330.473	11.5	0.367245	0.3830776	0.1289823	0.09410727	0.132028	0.1438548
1330.573	11.5	0.379201	0.3615509	0.1277778	0.09731586	0.127747	0.1410577
1330.673	11.5	0.387593	0.3646186	0.1276686	0.09761704	0.127575	0.1403702
1330.773	11.5	0.402013	0.3562057	0.1274707	0.09687132	0.124428	0.1383145
1330.873	11.5	0.409105	0.3463208	0.1294288	0.09714391	0.125783	0.1376358
1330.973	11.5	0.433336	0.3495204	0.1284442	0.09582011	0.12445	0.1389123
1331.073	11.5	0.399536	0.3559957	0.1311988	0.09497292	0.127269	0.135869
1331.173	11.5	0.411538	0.363429	0.1326475	0.096708	0.128278	0.1372701
1331.273	11.5	0.409732	0.372999	0.1307013	0.09681207	0.130369	0.1394316
1331.373	11.5	0.420697	0.357398	0.13093	0.09628146	0.127521	0.1424227
1331.473	11.5	0.427824	0.3466764	0.133814	0.09735648	0.12757	0.1399181
1331.573	11.5	0.424797	0.3428361	0.1346662	0.09733676	0.124685	0.1365077
1331.673	11.5	0.413734	0.3437552	0.1342458	0.09517398	0.124907	0.136266
1331.773	11.5	0.416858	0.3475401	0.1372073	0.0918647	0.124189	0.1356597
1331.873	11.5	0.430387	0.3528318	0.1379587	0.0962688	0.125998	0.1374823
1331.973	11.5	0.420596	0.3588578	0.1399014	0.09804657	0.124595	0.1359758
1332.073	11.5	0.397537	0.3614036	0.1395734	0.09808987	0.124625	0.1312039
1332.173	11.5	0.385801	0.3635148	0.141381	0.09835511	0.125549	0.1347986
1332.273	11.5	0.395442	0.3607363	0.143604	0.1002291	0.121815	0.1363359
1332.373	11.5	0.392114	0.3496745	0.1419569	0.0979665	0.120678	0.1353101
1332.473	11.5	0.387945	0.340005	0.1391773	0.09616568	0.119641	0.1364463
1332.573	11.5	0.394636	0.3381045	0.1392627	0.09707671	0.117738	0.1381754
1332.673	11.5	0.384086	0.3431189	0.1379474	0.09656871	0.120024	0.1378989
1332.773	11.5	0.385587	0.3485757	0.1372317	0.09610605	0.119632	0.1374509
1332.873	11.5	0.405088	0.3465934	0.1383243	0.09640215	0.120813	0.1357206
1332.973	11.5	0.455094	0.3554451	0.137302	0.09620698	0.118874	0.1365714
1333.073	11.5	0.445354	0.3802484	0.135627	0.09699924	0.119039	0.1370105
1333.173	11.5	0.444874	0.3566069	0.13436	0.09660096	0.116307	0.1387563
1333.273	11.5	0.440945	0.3616248	0.1350067	0.0975417	0.115785	0.1415354
1333.373	11.5	0.427716	0.3499892	0.1330749	0.09867482	0.117689	0.1426681
1333.473	11.5	0.456161	0.3352584	0.1374813	0.09848935	0.116618	0.1434423
1333.573	11.5	0.448163	0.3355138	0.1345977	0.0968297	0.117874	0.1415527
1333.673	11.5	0.421792	0.3428382	0.1344558	0.09804363	0.117914	0.1451581
1333.773	11.5	0.433361	0.3497462	0.1373987	0.09791195	0.115913	0.1478292
1333.873	11.5	0.432272	0.3610113	0.1368468	0.09512123	0.115204	0.1481914
1333.973	11.5	0.407882	0.3510545	0.1374408	0.09302079	0.113901	0.1479204
1334.073	11.5	0.401723	0.3448631	0.1355016	0.09554099	0.113655	0.1463495
1334.173	11.5	0.411458	0.3413474	0.1345405	0.09177785	0.115531	0.1476655
1334.273	11.5	0.379849	0.340355	0.1356776	0.08944401	0.117294	0.1518484
1334.373	11.5	0.392822	0.3416574	0.1360526	0.08823851	0.115896	0.1487948
1334.473	11.5	0.393218	0.3445587	0.1340467	0.08797605	0.113398	0.1471096
1334.573	11.5	0.386642	0.3497504	0.1341959	0.09463996	0.112989	0.1483902
1334.673	11.5	0.396022	0.351376	0.1348485	0.09332743	0.115444	0.1471947
1334.773	11.5	0.408517	0.353944	0.1347307	0.08977605	0.116855	0.1473397
1334.873	11.5	0.389751	0.3504505	0.1367718	0.08801408	0.119678	0.1449323
1334.973	11.5	0.386325	0.3430224	0.1342528	0.09000164	0.124331	0.1443498
1335.073	11.5	0.413261	0.3378218	0.1330918	0.08831277	0.123913	0.1453505
1335.173	11.5	0.385321	0.3344553	0.1341844	0.09070504	0.125532	0.1456464
1335.273	11.5	0.393933	0.3402476	0.1349384	0.09400966	0.128082	0.1426766
1335.373	11.5	0.382914	0.3450524	0.1344197	0.09212632	0.127112	0.1426393
1335.473	11.5	0.396381	0.3458837	0.1325509	0.09073736	0.127537	0.1463463
1335.573	11.5	0.394041	0.349051	0.1331228	0.08926725	0.13008	0.1465054
1335.673	11.5	0.370972	0.3785791	0.1308167	0.09014588	0.132608	0.1442387
1335.773	11.5	0.37395	0.3560395	0.1276957	0.09553214	0.131858	0.1433728
1335.873	11.5	0.388068	0.3616812	0.1281265	0.09592581	0.130693	0.1405336
1335.973	11.5	0.399485	0.3518817	0.1311067	0.09697215	0.130555	0.1399341
1336.073	11.5	0.426664	0.3384271	0.1314268	0.09059111	0.128672	0.1423686
1336.173	11.5	0.411665	0.3350891	0.1297856	0.09283396	0.126528	0.142293
1336.273	11.5	0.404948	0.337815	0.1310726	0.09508561	0.126671	0.1422416
1336.373	11.5	0.405025	0.3460911	0.1271757	0.09515247	0.126656	0.1417588
1336.473	11.5	0.415463	0.357211	0.1267001	0.09542035	0.126615	0.1372088
1336.573	11.5	0.416717	0.3505707	0.1281499	0.09511212	0.127879	0.1374798
1336.673	11.5	0.443865	0.3461125	0.1263949	0.09314817	0.126482	0.1374012
1336.773	11.5	0.437389	0.3420804	0.1264304	0.09270801	0.126287	0.1392597
1336.873	11.5	0.42694	0.3401385	0.1287508	0.09406481	0.125131	0.1390033
1336.973	11.5	0.427572	0.3401525	0.1299862	0.09635778	0.123052	0.1391041
1337.073	11.5	0.433219	0.3420812	0.1313801	0.09829274	0.122363	0.1373992
1337.173	11.5	0.428282	0.3460446	0.1317944	0.09786832	0.122551	0.1364664
1337.273	11.5	0.401029	0.3498558	0.1304892	0.09707045	0.12129	0.139618
1337.373	11.5	0.396341	0.3500861	0.1316328	0.09616827	0.121756	0.1381886
1337.473	11.5	0.388649	0.3494655	0.1329296	0.09816248	0.120018	0.1402002

1337.573	11.5	0.403807	0.3423389	0.1336818	0.09778412	0.122409	0.1430552
1337.673	11.5	0.396197	0.3371439	0.1342192	0.09725858	0.123317	0.1443496
1337.773	11.5	0.397679	0.3341182	0.1351519	0.09853277	0.122945	0.1441348
1337.873	11.5	0.385488	0.3393476	0.1355692	0.1001473	0.121803	0.1464562
1337.973	11.5	0.385372	0.3391887	0.1357411	0.09855568	0.123171	0.1457665
1338.073	11.5	0.39633	0.3395197	0.1387807	0.09881602	0.120802	0.1464614
1338.173	11.5	0.400591	0.3469287	0.1411605	0.09756085	0.123839	0.143898
1338.273	11.5	0.403875	0.3706071	0.1420522	0.09635963	0.122656	0.1424702
1338.373	11.5	0.390638	0.3456881	0.140656	0.09609104	0.120267	0.136549
1338.473	11.5	0.39918	0.3584744	0.1364652	0.09450637	0.118648	0.1370465
1338.573	11.5	0.401344	0.3500441	0.1371331	0.096879	0.120287	0.1375908
1338.673	11.5	0.38657	0.336397	0.1353207	0.09645445	0.119175	0.1362426
1338.773	11.5	0.376073	0.3322415	0.1338862	0.09906545	0.118759	0.1403531
1338.873	11.5	0.368055	0.335529	0.1332227	0.09916425	0.116626	0.1411925
1338.973	11.5	0.3752	0.3427383	0.131269	0.1008055	0.11584	0.1389037
1339.073	11.5	0.385094	0.35154	0.1310158	0.09880907	0.115922	0.137219
1339.173	11.5	0.383883	0.3511197	0.1316689	0.09526085	0.117397	0.1349067
1339.273	11.5	0.384356	0.3450491	0.1339884	0.0949113	0.115918	0.1354092
1339.373	11.5	0.382057	0.3435028	0.1316796	0.09545009	0.116273	0.1369032
1339.473	11.5	0.373821	0.3385576	0.1347304	0.09592177	0.115228	0.1387408
1339.573	11.5	0.397307	0.3393635	0.1321787	0.09564787	0.114403	0.1412696
1339.673	11.5	0.403994	0.340898	0.1344894	0.09441093	0.116231	0.1435777
1339.773	11.5	0.397607	0.3438489	0.1368235	0.09570166	0.117229	0.1451053
1339.873	11.5	0.412369	0.3448603	0.1360158	0.09448108	0.11902	0.1435187
1339.973	11.5	0.415332	0.3465554	0.1354479	0.09196134	0.114929	0.1436194
1340.073	11.5	0.399819	0.3469102	0.135015	0.09182933	0.115897	0.1469132
1340.173	11.5	0.388039	0.3413318	0.1347144	0.0908394	0.11958	0.1443634
1340.273	11.5	0.413445	0.3360995	0.1354355	0.09041966	0.121536	0.1468483
1340.373	11.5	0.395457	0.3301173	0.1358061	0.08847683	0.122472	0.1490638
1340.473	11.5	0.395048	0.3336934	0.1336005	0.09339684	0.121551	0.1463241
1340.573	11.5	0.386915	0.3339184	0.133073	0.09201597	0.124778	0.1442553
1340.673	11.5	0.399253	0.3338557	0.1334024	0.0901348	0.127314	0.1460934
1340.773	11.5	0.393923	0.3375528	0.1337213	0.08915699	0.129358	0.1462374
1340.873	11.5	0.377422	0.3643308	0.135642	0.0922484	0.128872	0.1463114
1340.973	11.5	0.368692	0.3521446	0.1337506	0.09411411	0.134237	0.144617
1341.073	11.5	0.393469	0.3538207	0.1326827	0.0952985	0.13373	0.1444986
1341.173	11.5	0.393125	0.350315	0.1330588	0.09532929	0.134296	0.1475726
1341.273	11.5	0.394282	0.3352492	0.1300436	0.09301459	0.134032	0.1460497
1341.373	11.5	0.399571	0.3291758	0.1292063	0.09063533	0.130717	0.1443866
1341.473	11.5	0.394108	0.3322106	0.1291321	0.09170762	0.128895	0.1472642
1341.573	11.5	0.397804	0.3417273	0.1290484	0.09137911	0.129068	0.1482449
1341.673	11.5	0.407706	0.3511297	0.1273273	0.08994074	0.127682	0.147484
1341.773	11.5	0.395242	0.3487719	0.1274701	0.08964378	0.127957	0.1455716
1341.873	11.5	0.398532	0.3467775	0.1293624	0.09001721	0.127275	0.1432275
1341.973	11.5	0.401548	0.3474552	0.1303004	0.0903232	0.131177	0.1444297
1342.073	11.5	0.397432	0.3409374	0.1286783	0.09527662	0.129155	0.1441592
1342.173	11.5	0.415705	0.341096	0.1270222	0.09832818	0.130075	0.1447022
1342.273	11.5	0.416682	0.3424724	0.1280401	0.1006822	0.12807	0.1432838
1342.373	11.5	0.417939	0.3429439	0.1247274	0.1008558	0.126047	0.1416585
1342.473	11.5	0.387025	0.3458864	0.1257456	0.09669208	0.125038	0.1415753
1342.573	11.5	0.397452	0.3486141	0.1287631	0.09807824	0.127404	0.1434592
1342.673	11.5	0.382478	0.3465028	0.1290649	0.1012071	0.124521	0.1417219
1342.773	11.5	0.38626	0.3412022	0.1296859	0.1030519	0.124319	0.1442707
1342.873	11.5	0.385318	0.3367088	0.1291684	0.1041119	0.125229	0.142167
1342.973	11.5	0.390387	0.3292914	0.1309332	0.1019337	0.123886	0.1450018
1343.073	11.5	0.385574	0.333708	0.1308	0.1028004	0.122289	0.1444941
1343.173	11.5	0.378401	0.3309797	0.1296761	0.1014676	0.119762	0.1437881
1343.273	11.5	0.379148	0.334942	0.1296015	0.1003746	0.119935	0.1430067
1343.373	11.5	0.388027	0.3348265	0.1311433	0.1000242	0.122572	0.141773
1343.473	11.5	0.392018	0.3617429	0.1317859	0.09960362	0.123428	0.142877
1343.573	11.5	0.380176	0.3438753	0.1345105	0.09800614	0.123242	0.142555
1343.673	11.5	0.397274	0.3558216	0.1362926	0.09622971	0.121685	0.1437691
1343.773	11.5	0.395335	0.3557592	0.1397972	0.09919097	0.122723	0.144657
1343.873	11.5	0.381121	0.3397936	0.1406782	0.09736621	0.119657	0.1439093
1343.973	11.5	0.383229	0.3321919	0.1405297	0.09883034	0.11852	0.1454476
1344.073	11.5	0.379682	0.3301741	0.1428788	0.09820219	0.118999	0.1418211
1344.173	11.5	0.386232	0.3369538	0.1431711	0.09755133	0.116755	0.1427542
1344.273	11.5	0.395562	0.3458256	0.1441913	0.09350898	0.118265	0.1419282
1344.373	11.5	0.39358	0.3488718	0.1440292	0.09117414	0.118529	0.1393743
1344.473	11.5	0.396279	0.3488432	0.1400117	0.09043546	0.117849	0.135842
1344.573	11.5	0.390754	0.3518191	0.1393885	0.09360087	0.117417	0.1382889
1344.673	11.5	0.388193	0.3416682	0.1385473	0.09441325	0.11671	0.1403672
1344.773	11.5	0.398735	0.3411629	0.1370748	0.09064572	0.117324	0.1437372
1344.873	11.5	0.409528	0.3427391	0.1383042	0.09065297	0.116657	0.1416968
1344.973	11.5	0.405824	0.3427027	0.1353819	0.09271936	0.115066	0.1394851
1345.073	11.5	0.40333	0.344336	0.1337976	0.09399645	0.113231	0.1399074
1345.173	11.5	0.406785	0.3473471	0.1361868	0.09079763	0.115889	0.1385519
1345.273	11.5	0.399189	0.3491141	0.1389213	0.08983413	0.113576	0.1400766
1345.373	11.5	0.388105	0.3447781	0.1371451	0.08763388	0.117135	0.1410552
1345.473	11.5	0.410406	0.3385148	0.1402917	0.08949729	0.117736	0.1437823
1345.573	11.5	0.396477	0.3323057	0.1384271	0.09276305	0.119432	0.1464982
1345.673	11.5	0.39547	0.3356279	0.1374157	0.09404077	0.120882	0.1462096
1345.773	11.5	0.389017	0.3342308	0.1387886	0.09378179	0.11964	0.1439789
1345.873	11.5	0.395397	0.3340953	0.1383982	0.09236226	0.12224	0.1467783
1345.973	11.5	0.401036	0.3300498	0.1384519	0.09332211	0.123354	0.1455362
1346.073	11.5	0.374353	0.3585656	0.137087	0.09423123	0.12551	0.1459173
1346.173	11.5	0.367429	0.3410225	0.1362067	0.09683349	0.130061	0.1454725
1346.273	11.5	0.394129	0.3543121	0.1375929	0.09952335	0.131783	0.1465467
1346.373	11.5	0.392104	0.3565306	0.1378522	0.09932806	0.128188	0.1511125
1346.473	11.5	0.391674	0.3425159	0.1383013	0.09470791	0.130362	0.1489029
1346.573	11.5	0.389679	0.3344634	0.1377309	0.09363195	0.131035	0.1494849
1346.673	11.5	0.393076	0.3290158	0.1367006	0.09511395	0.128838	0.1503902
1346.773	11.5	0.390926	0.335824	0.1348857	0.09334302	0.129684	0.1490897
1346.873	11.5	0.394658	0.3444824	0.1351431	0.09439957	0.126891	0.1458126
1346.973	11.5	0.391697	0.3471466	0.1359798	0.09478466	0.128635	0.1449314

1347.073	11.5	0.393037	0.3513446	0.1340552	0.09484728	0.129009	0.1458549
1347.173	11.5	0.397509	0.3527751	0.1370573	0.09910657	0.124513	0.1496714
1347.273	11.5	0.394419	0.3433285	0.1332123	0.09848776	0.123103	0.1470756
1347.373	11.5	0.407006	0.3435005	0.1332337	0.09937125	0.124704	0.1435376
1347.473	11.5	0.41072	0.3436019	0.1336545	0.1002381	0.126491	0.1455363
1347.573	11.5	0.409125	0.3451268	0.1319032	0.101564	0.126722	0.1459679
1347.673	11.5	0.393587	0.3472828	0.1310039	0.1033032	0.128399	0.1442221
1347.773	11.5	0.389038	0.3510613	0.126378	0.1012657	0.12677	0.1441697
1347.873	11.5	0.378179	0.3516843	0.1300718	0.1034473	0.126298	0.1426899
1347.973	11.5	0.383134	0.3473051	0.1319897	0.1036328	0.125087	0.1415087
1348.073	11.5	0.390393	0.3425601	0.1307611	0.1056934	0.124438	0.1427999
1348.173	11.5	0.387611	0.3359301	0.1283222	0.1038663	0.123689	0.1436319
1348.273	11.5	0.38593	0.3350897	0.1298045	0.1010326	0.123864	0.1444246
1348.373	11.5	0.379926	0.3313054	0.1251716	0.1006065	0.121162	0.1429374
1348.473	11.5	0.386471	0.3319891	0.1251996	0.0992737	0.123021	0.141281
1348.573	11.5	0.386628	0.3300249	0.1291892	0.09903894	0.122461	0.1437093
1348.673	11.5	0.401663	0.3530194	0.1298967	0.09530804	0.122872	0.1423786
1348.773	11.5	0.382428	0.3401278	0.1304791	0.09636157	0.120254	0.1434496
1348.873	11.5	0.405621	0.3447361	0.1329142	0.09682942	0.122059	0.1407786
1348.973	11.5	0.395294	0.3569747	0.131704	0.09871334	0.121535	0.1394435
1349.073	11.5	0.384516	0.3455726	0.1323095	0.1004063	0.122896	0.1385475
1349.173	11.5	0.375789	0.3394462	0.1315386	0.09798796	0.11966	0.1389947
1349.273	11.5	0.370129	0.3384544	0.1308343	0.09858184	0.11869	0.140428
1349.373	11.5	0.377275	0.343156	0.1324718	0.09936387	0.117651	0.1405488
1349.473	11.5	0.381803	0.3520703	0.1337124	0.09837154	0.119447	0.1402274
1349.573	11.5	0.386356	0.355512	0.1338315	0.09769776	0.117069	0.1419309
1349.673	11.5	0.379529	0.3470203	0.1346934	0.09483011	0.119943	0.1437415
1349.773	11.5	0.385306	0.3468764	0.1412881	0.09342853	0.120401	0.1421397
1349.873	11.5	0.387456	0.3442919	0.1408412	0.0907238	0.117336	0.1440209
1349.973	11.5	0.388301	0.348776	0.1398946	0.09220333	0.116529	0.142049
1350.073	11.5	0.390972	0.3520896	0.1380924	0.09279245	0.115551	0.1415647
1350.173	11.5	0.40127	0.3574305	0.13983	0.09216315	0.115703	0.1401606
1350.273	11.5	0.398108	0.3613361	0.1432699	0.09294111	0.114009	0.1388642
1350.373	11.5	0.400791	0.3620558	0.1451543	0.09179825	0.114046	0.1377153
1350.473	11.5	0.410842	0.3638824	0.1426631	0.09052246	0.11447	0.1372381
1350.573	11.5	0.396879	0.3608345	0.1417066	0.09010804	0.114039	0.1362179
1350.673	11.5	0.405175	0.3517663	0.1403798	0.09166569	0.112132	0.1396223
1350.773	11.5	0.409498	0.3409341	0.1375908	0.09468217	0.115597	0.142902
1350.873	11.5	0.393812	0.333169	0.1382834	0.0962095	0.119645	0.1415868
1350.973	11.5	0.392253	0.3334048	0.1352785	0.09486897	0.120062	0.1400847
1351.073	11.5	0.388308	0.332966	0.1362527	0.09139422	0.120421	0.1373622
1351.173	11.5	0.400955	0.3310654	0.1376813	0.09137075	0.120778	0.1378627
1351.273	11.5	0.383624	0.340764	0.1390326	0.09143247	0.124124	0.1405437
1351.373	11.5	0.363973	0.3345524	0.1357026	0.09101819	0.123203	0.1413909
1351.473	11.5	0.390887	0.3397688	0.1369558	0.09333352	0.123935	0.1424648
1351.573	11.5	0.3908	0.3579462	0.1352779	0.09639546	0.126304	0.1435159
1351.673	11.5	0.392877	0.3482872	0.1349144	0.09703514	0.128674	0.1425714
1351.773	11.5	0.388613	0.3361453	0.1360143	0.0964867	0.129514	0.142593
1351.873	11.5	0.394732	0.3366042	0.1349491	0.09773607	0.130926	0.146029
1351.973	11.5	0.389671	0.3389633	0.1371337	0.09848288	0.127902	0.1449857
1352.073	11.5	0.385111	0.3489736	0.1369	0.09787319	0.12616	0.1449208
1352.173	11.5	0.392141	0.3551898	0.1351555	0.09793545	0.12617	0.1448201
1352.273	11.5	0.389955	0.3493477	0.134816	0.09495027	0.124947	0.1455769
1352.373	11.5	0.398098	0.347665	0.1333938	0.09370184	0.121954	0.1496211
1352.473	11.5	0.392673	0.3410036	0.1337066	0.09444749	0.121577	0.149323
1352.573	11.5	0.407933	0.3406107	0.1344283	0.09782187	0.123337	0.1476493
1352.673	11.5	0.416846	0.3423307	0.1316472	0.1008844	0.122874	0.1466517
1352.773	11.5	0.40676	0.3469872	0.1313836	0.1007412	0.122798	0.1439273
1352.873	11.5	0.395661	0.3503275	0.135369	0.1018759	0.120349	0.1421458
1352.973	11.5	0.389067	0.3512344	0.1349986	0.1005197	0.121299	0.1444705
1353.073	11.5	0.378247	0.3536757	0.1329485	0.09840188	0.121095	0.1463626
1353.173	11.5	0.382635	0.3502627	0.1355469	0.09684186	0.120151	0.1468257
1353.273	11.5	0.392228	0.3429531	0.131458	0.09727586	0.118917	0.1449093
1353.373	11.5	0.39417	0.3338406	0.1318937	0.1004956	0.120135	0.1420613
1353.473	11.5	0.388493	0.3286915	0.1326548	0.1012364	0.118686	0.1431641
1353.573	11.5	0.377626	0.3330505	0.1322792	0.09938408	0.121773	0.1412198
1353.673	11.5	0.382512	0.3253651	0.1308188	0.09575504	0.120217	0.1404121
1353.773	11.5	0.386388	0.3299883	0.1276571	0.09375095	0.118475	0.1377402
1353.873	11.5	0.415699	0.3386152	0.1306341	0.09240967	0.120493	0.1375354
1353.973	11.5	0.414417	0.3386514	0.1313094	0.09352887	0.120842	0.1348024
1354.073	11.5	0.415391	0.3355214	0.1320723	0.09412999	0.117574	0.1386371
1354.173	11.5	0.413051	0.3638687	0.1289845	0.09582558	0.118864	0.1414672
1354.273	11.5	0.396444	0.3502731	0.1331328	0.0946395	0.1181	0.1402013
1354.373	11.5	0.387444	0.3346386	0.1295951	0.09152485	0.116615	0.1367879
1354.473	11.5	0.384992	0.3298864	0.1266133	0.09513722	0.114873	0.1369933
1354.573	11.5	0.385052	0.3323941	0.128379	0.09525625	0.115034	0.1372284
1354.673	11.5	0.40451	0.3411056	0.1288278	0.09560495	0.111904	0.137397
1354.773	11.5	0.410588	0.3484628	0.1271441	0.09449746	0.112399	0.1380786
1354.873	11.5	0.394829	0.3482141	0.1295211	0.09519186	0.110469	0.1351755
1354.973	11.5	0.405215	0.3520707	0.1306673	0.09528606	0.110748	0.135885
1355.073	11.5	0.403502	0.3391386	0.1306241	0.09442928	0.113136	0.1352857
1355.173	11.5	0.393487	0.3365139	0.1308234	0.09491131	0.112367	0.1356376
1355.273	11.5	0.40346	0.3362449	0.1297953	0.09475604	0.118487	0.1381702
1355.373	11.5	0.403466	0.3398419	0.129782	0.09246298	0.118215	0.1380692
1355.473	11.5	0.390028	0.3434889	0.1345612	0.09289271	0.115692	0.1357976
1355.573	11.5	0.394033	0.3425818	0.1351126	0.09216174	0.116629	0.136663
1355.673	11.5	0.400566	0.3461315	0.1359715	0.09257186	0.118457	0.1375251
1355.773	11.5	0.389575	0.3450662	0.140641	0.09280025	0.117617	0.1354222
1355.873	11.5	0.395666	0.3381664	0.1378611	0.09211855	0.116706	0.1370501
1355.973	11.5	0.416819	0.3305956	0.139273	0.09214522	0.118664	0.1392664
1356.073	11.5	0.391469	0.3268288	0.1389578	0.09472588	0.119888	0.1378019
1356.173	11.5	0.39864	0.3299968	0.1386357	0.09646898	0.117633	0.1388334
1356.273	11.5	0.383746	0.3248556	0.1420422	0.09362257	0.120693	0.1365951
1356.373	11.5	0.403731	0.3268942	0.141751	0.09161207	0.117903	0.1369675
1356.473	11.5	0.390734	0.3356964	0.1398522	0.09005414	0.121125	0.1383508

1356.573	11.5	0.363028	0.3381294	0.1395996	0.09144208	0.124213	0.1376938
1356.673	11.5	0.385101	0.3300824	0.1372231	0.09228782	0.124584	0.1385136
1356.773	11.5	0.384354	0.3559741	0.1353288	0.09462946	0.123533	0.141214
1356.873	11.5	0.391046	0.3515346	0.1367196	0.09401751	0.128936	0.1411667
1356.973	11.5	0.386934	0.3336191	0.1343067	0.09289417	0.129967	0.1409346
1357.073	11.5	0.391731	0.3263074	0.1337701	0.09197894	0.131412	0.1406973
1357.173	11.5	0.390845	0.3270552	0.1359348	0.09100847	0.130001	0.1375566
1357.273	11.5	0.386386	0.3396757	0.1364495	0.09169203	0.128119	0.1395408
1357.373	11.5	0.397209	0.3461871	0.133279	0.09469052	0.128695	0.1427812
1357.473	11.5	0.388908	0.344428	0.1366667	0.09322062	0.124278	0.1470084
1357.573	11.5	0.393051	0.3476319	0.1353666	0.09181287	0.120891	0.1476741
1357.673	11.5	0.395288	0.3402537	0.1373068	0.09246852	0.119015	0.1469607
1357.773	11.5	0.400964	0.3372739	0.139908	0.09675912	0.122019	0.1466089
1357.873	11.5	0.410907	0.3391572	0.138658	0.09786568	0.126378	0.152344
1357.973	11.5	0.40878	0.3399416	0.135761	0.1007888	0.127562	0.1529698
1358.073	11.5	0.402684	0.341758	0.1366953	0.1009382	0.123076	0.152943
1358.173	11.5	0.382878	0.3412519	0.1345732	0.09854367	0.124328	0.1528481
1358.273	11.5	0.381015	0.3439879	0.1358496	0.09757514	0.124959	0.1531589
1358.373	11.5	0.381332	0.3426125	0.1370028	0.09858843	0.124142	0.1537148
1358.473	11.5	0.389734	0.336547	0.1356612	0.09713963	0.123651	0.1483913
1358.573	11.5	0.392078	0.3294308	0.1360426	0.1014987	0.12728	0.1451056
1358.673	11.5	0.387435	0.3263026	0.1348729	0.1017129	0.125652	0.1466142
1358.773	11.5	0.378721	0.3286149	0.1339873	0.1007812	0.129289	0.1450321
1358.873	11.5	0.38072	0.3214847	0.1347176	0.09813876	0.127983	0.1453492
1358.973	11.5	0.382864	0.3263028	0.1354028	0.09788071	0.126371	0.1451093
1359.073	11.5	0.394588	0.3293001	0.1351137	0.09586614	0.124744	0.1464926
1359.173	11.5	0.389473	0.3367391	0.138009	0.09417058	0.127365	0.147171
1359.273	11.5	0.390021	0.3269057	0.1375895	0.09415921	0.127224	0.1450237
1359.373	11.5	0.390364	0.351054	0.1350364	0.09199227	0.126325	0.142587
1359.473	11.5	0.392185	0.3462477	0.1335327	0.09243424	0.123646	0.1430565
1359.573	11.5	0.386903	0.3342482	0.1317477	0.09094225	0.121346	0.1432736
1359.673	11.5	0.383743	0.3300109	0.1294576	0.09132409	0.119929	0.1431783
1359.773	11.5	0.383734	0.3314685	0.1297626	0.09530643	0.117366	0.142115
1359.873	11.5	0.394442	0.3431644	0.1322373	0.09342884	0.116153	0.1402138
1359.973	11.5	0.391789	0.350425	0.1348664	0.09257607	0.116459	0.1410152
1360.073	11.5	0.387758	0.3478006	0.1323369	0.09281059	0.115434	0.1443415
1360.173	11.5	0.395777	0.3470588	0.1308036	0.09553265	0.118155	0.1457444
1360.273	11.5	0.394789	0.3422583	0.1339837	0.0955664	0.118711	0.1426625
1360.373	11.5	0.381995	0.3405035	0.1314157	0.09831178	0.118008	0.142438
1360.473	11.5	0.395721	0.3395339	0.1299564	0.09737651	0.119433	0.1398642
1360.573	11.5	0.401847	0.3427996	0.1315481	0.09493969	0.119563	0.1384557
1360.673	11.5	0.394617	0.3480186	0.131298	0.09742893	0.116819	0.139049
1360.773	11.5	0.397287	0.348902	0.130375	0.09551897	0.116246	0.1401996
1360.873	11.5	0.409493	0.3547591	0.1329196	0.09311648	0.119504	0.1382934
1360.973	11.5	0.398106	0.3497109	0.1310657	0.09054066	0.118388	0.1383642
1361.073	11.5	0.395952	0.3395882	0.1284878	0.08765794	0.113404	0.1379495
1361.173	11.5	0.418568	0.3320261	0.1312595	0.08955235	0.115706	0.1391922
1361.273	11.5	0.389006	0.3270196	0.1291687	0.08946241	0.11977	0.1392256
1361.373	11.5	0.401511	0.3304443	0.1324469	0.09293564	0.11789	0.138858
1361.473	11.5	0.388827	0.3268883	0.1343996	0.09153267	0.116999	0.1415023
1361.573	11.5	0.403676	0.3267932	0.1336643	0.09109093	0.118489	0.1418643
1361.673	11.5	0.396619	0.3249377	0.1319008	0.09240798	0.120673	0.1421568
1361.773	11.5	0.366942	0.3421389	0.1370779	0.09353625	0.122411	0.140177
1361.873	11.5	0.382093	0.3300893	0.1359599	0.0951765	0.123385	0.1418971
1361.973	11.5	0.385621	0.3516141	0.136092	0.09569564	0.123597	0.1403304
1362.073	11.5	0.38614	0.3568935	0.138272	0.09469108	0.123936	0.1389434
1362.173	11.5	0.38857	0.3375576	0.1423463	0.09435586	0.122453	0.1400338
1362.273	11.5	0.393134	0.3313904	0.1425395	0.09621065	0.123965	0.1374861
1362.373	11.5	0.390657	0.3283857	0.1441373	0.09621055	0.125612	0.1360105
1362.473	11.5	0.38763	0.3391737	0.1402996	0.09611665	0.124759	0.1381796
1362.573	11.5	0.396381	0.3479001	0.1385604	0.09598444	0.12581	0.138422
1362.673	11.5	0.387991	0.3468103	0.1371817	0.09548046	0.123309	0.1383644
1362.773	11.5	0.387273	0.3459485	0.1360314	0.09546529	0.123128	0.1429826
1362.873	11.5	0.395847	0.3405676	0.138056	0.09754556	0.121128	0.143015
1362.973	11.5	0.399182	0.3392079	0.1360128	0.09652595	0.121888	0.1435806
1363.073	11.5	0.411434	0.3374444	0.1343725	0.100621	0.126827	0.1449881
1363.173	11.5	0.409289	0.3388097	0.1362112	0.1033269	0.127656	0.1452515
1363.273	11.5	0.408041	0.3428788	0.1358825	0.1042153	0.12564	0.1455622
1363.373	11.5	0.380494	0.3437874	0.1333797	0.1026673	0.123927	0.1452369
1363.473	11.5	0.381622	0.3460823	0.1367025	0.1025662	0.125881	0.145818
1363.573	11.5	0.382015	0.3446361	0.1351691	0.1034534	0.127064	0.1466076
1363.673	11.5	0.386659	0.3408571	0.1352133	0.1032669	0.12713	0.1478535
1363.773	11.5	0.393107	0.3372061	0.1386627	0.1033868	0.125298	0.1463857
1363.873	11.5	0.390048	0.3279918	0.136768	0.1018625	0.123705	0.1479677
1363.973	11.5	0.383166	0.3281925	0.1354656	0.1001146	0.122966	0.14736
1364.073	11.5	0.380292	0.3257464	0.1351577	0.09581383	0.123429	0.1484274
1364.173	11.5	0.383631	0.3252685	0.133258	0.09240981	0.12213	0.1489205
1364.273	11.5	0.394408	0.3239224	0.1363295	0.09287407	0.122263	0.1481171
1364.373	11.5	0.395797	0.3406199	0.1332031	0.09329791	0.119857	0.1487131
1364.473	11.5	0.39286	0.3271992	0.132804	0.096039	0.116922	0.1455332
1364.573	11.5	0.3948	0.3527857	0.1338158	0.09225532	0.116267	0.1461986
1364.673	11.5	0.396527	0.3585459	0.1337678	0.09286923	0.113082	0.1453898
1364.773	11.5	0.387915	0.3410784	0.1315942	0.0915597	0.112475	0.1424026
1364.873	11.5	0.381632	0.3295137	0.132415	0.09178146	0.11181	0.1403677
1364.973	11.5	0.373073	0.3236434	0.1327628	0.09291569	0.113073	0.1376031
1365.073	11.5	0.390006	0.3358573	0.1326925	0.0931123	0.111489	0.1391579
1365.173	11.5	0.393236	0.3468327	0.1333806	0.09478584	0.111771	0.1412391
1365.273	11.5	0.389538	0.3531585	0.1324849	0.09222361	0.111303	0.1412489
1365.373	11.5	0.395242	0.350159	0.1324218	0.09562475	0.114203	0.1393375
1365.473	11.5	0.392179	0.3460084	0.1332515	0.09508409	0.113563	0.1407043
1365.573	11.5	0.386249	0.3440675	0.1308837	0.09578806	0.113712	0.1433468
1365.673	11.5	0.404038	0.342036	0.1292823	0.0969385	0.113706	0.1435056
1365.773	11.5	0.42216	0.3444472	0.126873	0.09505703	0.114498	0.1422575
1365.873	11.5	0.424638	0.3474108	0.1295621	0.09464157	0.114937	0.1406301
1365.973	11.5	0.435984	0.3467438	0.1310395	0.09509627	0.113352	0.137369

1366.073	11.5	0.43418	0.3493788	0.1274386	0.09253929	0.114524	0.1407293
1366.173	11.5	0.423072	0.3479355	0.1241675	0.09270875	0.113288	0.1423057
1366.273	11.5	0.403737	0.3422554	0.1262734	0.09313492	0.112839	0.143673
1366.373	11.5	0.428312	0.3360525	0.1243556	0.09219094	0.114011	0.1429812
1366.473	11.5	0.396424	0.3289019	0.1260493	0.0930826	0.117407	0.1389435
1366.573	11.5	0.399097	0.3299829	0.1274224	0.09646273	0.117128	0.1383487
1366.673	11.5	0.390375	0.3224839	0.1249911	0.09541789	0.115765	0.1384531
1366.773	11.5	0.400799	0.324367	0.1243037	0.09457894	0.114553	0.1409391
1366.873	11.5	0.396824	0.3264777	0.1257549	0.09423357	0.118885	0.1373242
1366.973	11.5	0.377498	0.3401793	0.1251386	0.09500714	0.121316	0.1360644
1367.073	11.5	0.377345	0.3306943	0.1259506	0.09669345	0.121679	0.1358434
1367.173	11.5	0.386374	0.3515247	0.1284736	0.09661192	0.123562	0.1373633
1367.273	11.5	0.38805	0.3662794	0.1273393	0.09472498	0.127335	0.1405985
1367.373	11.5	0.39181	0.3430401	0.1302202	0.09309585	0.128275	0.1406457
1367.473	11.5	0.392044	0.3303646	0.1318437	0.09378804	0.129336	0.1403109
1367.573	11.5	0.392277	0.3234651	0.1305324	0.09339512	0.131869	0.142593
1367.673	11.5	0.390505	0.3358848	0.1310252	0.0938267	0.125748	0.1441787
1367.773	11.5	0.397609	0.3476946	0.1377916	0.09259192	0.123337	0.1407984
1367.873	11.5	0.386326	0.347422	0.1371698	0.09093945	0.124506	0.139544
1367.973	11.5	0.384599	0.3478724	0.1386417	0.09089904	0.121413	0.138359
1368.073	11.5	0.394239	0.3412257	0.1393945	0.09531126	0.120177	0.1385899
1368.173	11.5	0.397649	0.3373976	0.1414634	0.09728194	0.120896	0.1417956
1368.273	11.5	0.413509	0.3388298	0.142617	0.09759754	0.124278	0.1387197
1368.373	11.5	0.414146	0.3408577	0.142381	0.09753264	0.12582	0.1388597
1368.473	11.5	0.407884	0.3440965	0.1386638	0.0997012	0.12547	0.1374629
1368.573	11.5	0.386269	0.3437331	0.1396295	0.1002628	0.121769	0.1362156
1368.673	11.5	0.387862	0.3458145	0.1372658	0.1001957	0.121466	0.1354279
1368.773	11.5	0.382914	0.3473559	0.137062	0.1001179	0.123056	0.1393071
1368.873	11.5	0.38562	0.3446611	0.1399102	0.1008396	0.121578	0.1413093
1368.973	11.5	0.39177	0.3373489	0.1368115	0.1031696	0.119204	0.1415856
1369.073	11.5	0.391469	0.3308921	0.1356272	0.102835	0.119231	0.141251
1369.173	11.5	0.382064	0.3324	0.1366502	0.1028665	0.120754	0.1384935
1369.273	11.5	0.377685	0.3238031	0.1363732	0.09949338	0.119497	0.1371967
1369.373	11.5	0.381458	0.3246547	0.1344088	0.09803961	0.118112	0.1381213
1369.473	11.5	0.39172	0.3226082	0.1369619	0.09825965	0.117875	0.1379452
1369.573	11.5	0.39355	0.3406984	0.1354097	0.1007152	0.118677	0.1419668
1369.673	11.5	0.387597	0.3248284	0.1371554	0.1024901	0.118455	0.1447911
1369.773	11.5	0.392111	0.348917	0.1381636	0.1001549	0.118796	0.1444344
1369.873	11.5	0.393576	0.3650154	0.1343215	0.1002661	0.11672	0.148659
1369.973	11.5	0.389079	0.3465837	0.1334243	0.09784939	0.116448	0.1495083
1370.073	11.5	0.385578	0.3340355	0.1355621	0.09795091	0.117843	0.1497615
1370.173	11.5	0.404538	0.3281658	0.1336913	0.09891051	0.119784	0.1497983
1370.273	11.5	0.398453	0.3385577	0.1341907	0.09748756	0.115653	0.1499198
1370.373	11.5	0.40056	0.3489715	0.1324566	0.09780324	0.115249	0.1516667
1370.473	11.5	0.392425	0.3546884	0.1320743	0.09466861	0.116151	0.1488489
1370.573	11.5	0.406421	0.3526911	0.1341996	0.09752562	0.116694	0.1490812
1370.673	11.5	0.419217	0.3510224	0.1334876	0.09855285	0.114983	0.1507199
1370.773	11.5	0.410353	0.3475505	0.1357289	0.09694604	0.115564	0.1489047
1370.873	11.5	0.423943	0.3501564	0.1369222	0.09701473	0.113936	0.1453777
1370.973	11.5	0.416261	0.3515395	0.1367689	0.0949816	0.113784	0.144434
1371.073	11.5	0.401215	0.3606785	0.1351483	0.09633034	0.113183	0.1455641
1371.173	11.5	0.404801	0.3616795	0.1349213	0.09523595	0.113797	0.1464426
1371.273	11.5	0.411092	0.3636616	0.1309304	0.09392924	0.115321	0.1447078
1371.373	11.5	0.404426	0.3573331	0.1288987	0.09247851	0.114912	0.1436929
1371.473	11.5	0.393155	0.3547567	0.1293424	0.09168097	0.114446	0.1447418
1371.573	11.5	0.411966	0.3446922	0.1277169	0.09189758	0.111428	0.1438469
1371.673	11.5	0.392559	0.3357286	0.1250058	0.09108687	0.114322	0.1445407
1371.773	11.5	0.397537	0.3332391	0.1217293	0.09426161	0.112825	0.142759
1371.873	11.5	0.392118	0.3240364	0.1242776	0.09473215	0.112964	0.1445067
1371.973	11.5	0.39525	0.317863	0.1277896	0.09534242	0.112389	0.1426249
1372.073	11.5	0.399461	0.3270452	0.1262206	0.09785479	0.118327	0.1436183
1372.173	11.5	0.37471	0.3330086	0.1266609	0.09495964	0.119177	0.1437911
1372.273	11.5	0.371065	0.3243048	0.1304061	0.0951945	0.121249	0.1439206
1372.373	11.5	0.385208	0.3397547	0.1273852	0.09427118	0.120403	0.1455842
1372.473	11.5	0.38771	0.3631355	0.1274256	0.09591908	0.12281	0.1408677
1372.573	11.5	0.39469	0.3523332	0.127268	0.09473128	0.123711	0.138736
1372.673	11.5	0.391204	0.3303542	0.1265364	0.09528568	0.120973	0.1360089
1372.773	11.5	0.395427	0.3213437	0.1277433	0.09679057	0.121143	0.1376537
1372.873	11.5	0.387571	0.333351	0.1285513	0.09516823	0.120751	0.1351636
1372.973	11.5	0.395712	0.3456948	0.1272003	0.09984557	0.117317	0.1358316
1373.073	11.5	0.39057	0.3497576	0.1266356	0.1012334	0.116059	0.1356448
1373.173	11.5	0.385506	0.3487899	0.1271385	0.09709477	0.114632	0.1360604
1373.273	11.5	0.392707	0.3509045	0.1251332	0.09667328	0.116162	0.1367467
1373.373	11.5	0.392291	0.3425158	0.1295818	0.09774838	0.118169	0.1362476
1373.473	11.5	0.405278	0.3409194	0.1329917	0.09655353	0.123552	0.1375472
1373.573	11.5	0.414844	0.341394	0.134023	0.09792556	0.120688	0.1403194
1373.673	11.5	0.404937	0.341287	0.1347056	0.1003129	0.119949	0.1415527
1373.773	11.5	0.388389	0.3432835	0.1355256	0.09953729	0.115528	0.1404823
1373.873	11.5	0.389101	0.3445362	0.1334651	0.1005639	0.118543	0.1405559
1373.973	11.5	0.380009	0.3460099	0.1342418	0.09978039	0.118099	0.1403909
1374.073	11.5	0.387508	0.3425854	0.1373699	0.09744936	0.117016	0.1389535
1374.173	11.5	0.40089	0.3356287	0.1396202	0.09935804	0.117498	0.1366339
1374.273	11.5	0.396142	0.3301746	0.1396078	0.1013059	0.117332	0.1364268
1374.373	11.5	0.389809	0.3320246	0.1413148	0.101439	0.115674	0.1373654
1374.473	11.5	0.380093	0.3237459	0.1372987	0.09948664	0.116874	0.1368512
1374.573	11.5	0.38953	0.3178951	0.1373142	0.09582523	0.119283	0.136067
1374.673	11.5	0.393606	0.3248613	0.1368962	0.09565705	0.119876	0.1359732
1374.773	11.5	0.414722	0.335516	0.134199	0.09634741	0.123264	0.1395998
1374.873	11.5	0.389068	0.3253901	0.1354931	0.09642196	0.1204	0.1398234
1374.973	11.5	0.398564	0.3316417	0.1327716	0.09941144	0.122074	0.1399221
1375.073	11.5	0.393704	0.3613555	0.1331614	0.1003629	0.119851	0.1375191
1375.173	11.5	0.393627	0.3535247	0.1345313	0.09825879	0.118044	0.1388274
1375.273	11.5	0.38267	0.3332196	0.1368267	0.09704691	0.118535	0.1387352
1375.373	11.5	0.378437	0.3194463	0.1336267	0.09703148	0.117336	0.1387896
1375.473	11.5	0.386405	0.3316767	0.1363697	0.09814789	0.115386	0.1401873

1375.573	11.5	0.390422	0.3438684	0.1342917	0.09766887	0.118504	0.141343
1375.673	11.5	0.387366	0.3518575	0.1347033	0.09579103	0.117813	0.141416
1375.773	11.5	0.390303	0.348096	0.1350439	0.09482188	0.116368	0.1412075
1375.873	11.5	0.387782	0.3513808	0.1324065	0.09584621	0.114425	0.1454923
1375.973	11.5	0.385459	0.3449961	0.1317516	0.09385619	0.11351	0.146247
1376.073	11.5	0.39294	0.3439195	0.130251	0.09338629	0.11298	0.1483818
1376.173	11.5	0.391776	0.3430248	0.1299772	0.09364469	0.116529	0.1494489
1376.273	11.5	0.400967	0.344655	0.1320439	0.09500834	0.11664	0.1491883
1376.373	11.5	0.401739	0.3499371	0.132396	0.09655081	0.11546	0.1500088
1376.473	11.5	0.411925	0.3466382	0.1315737	0.09310581	0.116106	0.1452611
1376.573	11.5	0.412887	0.3540263	0.1305435	0.09029313	0.115502	0.1455414
1376.673	11.5	0.39256	0.3494954	0.1288708	0.08750381	0.114315	0.1481315
1376.773	11.5	0.398667	0.3358613	0.1281646	0.08765558	0.111866	0.1464614
1376.873	11.5	0.397194	0.3293896	0.1319098	0.08836527	0.116086	0.1453569
1376.973	11.5	0.392061	0.3292388	0.1332439	0.0944536	0.117623	0.1445042
1377.073	11.5	0.393809	0.3294461	0.1315933	0.09449507	0.11558	0.1442591
1377.173	11.5	0.38924	0.3201106	0.1308432	0.09296118	0.116797	0.1457161
1377.273	11.5	0.40512	0.3200608	0.127726	0.09433785	0.118792	0.1433355
1377.373	11.5	0.387862	0.3301081	0.1269619	0.0948268	0.118221	0.1440271
1377.473	11.5	0.369169	0.3272283	0.1258024	0.09339487	0.122059	0.1462138
1377.573	11.5	0.380174	0.339633	0.1250854	0.09342953	0.121131	0.1450039
1377.673	11.5	0.392345	0.3491955	0.1221451	0.09502854	0.120976	0.1427697
1377.773	11.5	0.392656	0.3459688	0.12002	0.09487088	0.120635	0.1430103
1377.873	11.5	0.389915	0.3322315	0.1242816	0.09746571	0.11835	0.1422056
1377.973	11.5	0.397511	0.3214606	0.126564	0.09737588	0.119468	0.1382636
1378.073	11.5	0.391051	0.3266841	0.1282713	0.09502397	0.119343	0.1399198
1378.173	11.5	0.393249	0.339361	0.1247277	0.09972655	0.120444	0.1397323
1378.273	11.5	0.394412	0.3491504	0.1278107	0.1005509	0.122458	0.1386099
1378.373	11.5	0.397877	0.3442658	0.1263706	0.1005018	0.120883	0.1409629
1378.473	11.5	0.412614	0.3501668	0.1246797	0.09838446	0.118668	0.1396808
1378.573	11.5	0.413464	0.3421661	0.1259495	0.09625806	0.118803	0.1387633
1378.673	11.5	0.403789	0.3423433	0.1246572	0.09682205	0.12288	0.1405995
1378.773	11.5	0.388958	0.342685	0.1243335	0.09886562	0.12667	0.1423434
1378.873	11.5	0.388908	0.3447142	0.1281065	0.1006201	0.128111	0.1398202
1379	12	0.38272	0.3497434	0.1287722	0.09915829	0.120804	0.1394518
1379.1	12	0.383786	0.3472426	0.1276911	0.09822793	0.120854	0.1371237
1379.2	12	0.390858	0.3384349	0.1285159	0.09832765	0.121589	0.1365627
1379.3	12	0.387828	0.3294631	0.1279239	0.1010204	0.122103	0.140286
1379.4	12	0.381733	0.3290348	0.129987	0.09984834	0.120622	0.1398183
1379.5	12	0.377252	0.3190306	0.1322137	0.09832992	0.121329	0.1401082
1379.6	12	0.382032	0.3227785	0.1334879	0.09628479	0.122145	0.141202
1379.7	12	0.421102	0.3175044	0.1346084	0.09631836	0.125074	0.1419995
1379.8	12	0.431307	0.3283015	0.1367554	0.09523711	0.122532	0.1430435
1379.9	12	0.44251	0.3213875	0.133705	0.09690217	0.12161	0.1427594
1380	12	0.418625	0.3411964	0.1355617	0.09682379	0.120259	0.142472
1380.1	12	0.412728	0.3515804	0.1364666	0.09811349	0.120871	0.1402116
1380.2	12	0.398506	0.3382746	0.1389847	0.0978841	0.123191	0.1392601
1380.3	12	0.405151	0.3299737	0.1398472	0.09603408	0.121784	0.1367937
1380.4	12	0.407253	0.3253944	0.1365703	0.09643469	0.118327	0.1380402
1380.5	12	0.415043	0.3380248	0.1330898	0.09550451	0.117169	0.1391181
1380.6	12	0.4183	0.3475338	0.133426	0.09728066	0.115702	0.1383158
1380.7	12	0.407206	0.3484958	0.1327843	0.09646331	0.114297	0.1388406
1380.8	12	0.41553	0.3518272	0.1308433	0.09631442	0.115201	0.1403821
1380.9	12	0.396784	0.3499923	0.1339432	0.09462103	0.11734	0.1410454
1381	12	0.392573	0.342116	0.1331448	0.09716181	0.114135	0.1401079
1381.1	12	0.389412	0.3471267	0.1325591	0.09611788	0.112493	0.1386099
1381.2	12	0.39216	0.3484408	0.1341439	0.09743454	0.114256	0.1392248
1381.3	12	0.396742	0.3506457	0.1341536	0.0980421	0.113054	0.1414222
1381.4	12	0.404477	0.3504345	0.1344377	0.09740794	0.113518	0.1419713
1381.5	12	0.402248	0.3514979	0.133685	0.09678667	0.114678	0.1413969
1381.6	12	0.389669	0.3514335	0.130468	0.09337546	0.112691	0.1437016
1381.7	12	0.407246	0.3443222	0.13115	0.09287816	0.114155	0.146298
1381.8	12	0.38574	0.3338072	0.1313882	0.09535086	0.112491	0.1433924
1381.9	12	0.394373	0.3252596	0.1318561	0.09665238	0.112549	0.1466478
1382	12	0.38367	0.3179432	0.1314406	0.09833828	0.112905	0.1479822
1382.1	12	0.406049	0.3171778	0.1324876	0.095408	0.116673	0.1511869
1382.2	12	0.403051	0.3199204	0.1325049	0.09305945	0.120811	0.1532831
1382.3	12	0.383318	0.3099586	0.1323034	0.09516564	0.122032	0.1524304
1382.4	12	0.383538	0.3280357	0.1346142	0.0978964	0.123009	0.1504002
1382.5	12	0.385366	0.3216728	0.134826	0.0985433	0.124328	0.1459115
1382.6	12	0.393969	0.3532476	0.1311326	0.1004161	0.12481	0.1426898
1382.7	12	0.389392	0.3552802	0.131205	0.09786005	0.126168	0.1413073
1382.8	12	0.407762	0.3351085	0.129208	0.09587338	0.127991	0.1412562
1382.9	12	0.397679	0.3228881	0.1265059	0.0948198	0.128649	0.1392805
1383	12	0.393224	0.3290669	0.1267135	0.09555043	0.130649	0.1387476
1383.1	12	0.388271	0.3338837	0.1272017	0.09627119	0.128249	0.1399201
1383.2	12	0.386492	0.344867	0.1250703	0.09706952	0.129876	0.1431486
1383.3	12	0.394701	0.3457993	0.1218633	0.09503546	0.131054	0.1410512
1383.4	12	0.391114	0.3525649	0.1237961	0.09523103	0.12883	0.1402405
1383.5	12	0.407044	0.3428429	0.123922	0.0955327	0.127193	0.1439832
1383.6	12	0.421333	0.3381789	0.1278165	0.09383548	0.122805	0.1447225
1383.7	12	0.408701	0.3383352	0.125976	0.09476655	0.121197	0.1435328
1383.8	12	0.384566	0.3409253	0.1268708	0.09823424	0.119635	0.1402304
1383.9	12	0.384782	0.3441499	0.1255605	0.09906834	0.122057	0.1401151
1384	12	0.382427	0.3445272	0.1253303	0.09802718	0.122531	0.1408544
1384.1	12	0.386275	0.3443676	0.1276664	0.0956648	0.121796	0.1442138
1384.2	12	0.389656	0.3422852	0.1277256	0.09598255	0.12006	0.1443716
1384.3	12	0.387324	0.3340705	0.1274894	0.1027307	0.121575	0.1421954
1384.4	12	0.378761	0.3278674	0.1301372	0.1031686	0.119936	0.1419055
1384.5	12	0.376779	0.3252931	0.1286885	0.09801815	0.119487	0.1403533
1384.6	12	0.380513	0.310206	0.130235	0.09579995	0.118742	0.1397272
1384.7	12	0.382831	0.3169269	0.1315143	0.09421849	0.121384	0.1410956
1384.8	12	0.393178	0.3236137	0.1299197	0.09497499	0.119637	0.1434751
1384.9	12	0.393035	0.3297214	0.13222	0.09675965	0.120287	0.1398197
1385	12	0.3835	0.3229423	0.1298784	0.0958243	0.119554	0.1388965

1385.1	12	0.38509	0.3370942	0.1308687	0.09637795	0.117726	0.1388316
1385.2	12	0.377683	0.3641468	0.1329038	0.09492818	0.114757	0.1383916
1385.3	12	0.377078	0.3481732	0.1334351	0.09284335	0.115642	0.1402821
1385.4	12	0.376352	0.3253924	0.1356706	0.09302856	0.11769	0.1387301
1385.5	12	0.380619	0.3196581	0.1363201	0.0946368	0.117037	0.1378689
1385.6	12	0.384499	0.336814	0.1385477	0.09513544	0.116349	0.1394175
1385.7	12	0.385351	0.3469919	0.1385218	0.09262558	0.112602	0.1401348
1385.8	12	0.394467	0.3471105	0.1405797	0.09712224	0.111127	0.1383238
1385.9	12	0.391205	0.3468083	0.1371708	0.09867103	0.112621	0.1371932
1386	12	0.400415	0.3487319	0.1375161	0.09786057	0.111621	0.1367748
1386.1	12	0.401226	0.3414014	0.1361022	0.09799208	0.114984	0.136328
1386.2	12	0.403072	0.3423504	0.134065	0.09517007	0.11296	0.1374632
1386.3	12	0.412928	0.3423583	0.1348531	0.09527323	0.111045	0.1364692
1386.4	12	0.42756	0.3455226	0.1330953	0.09562294	0.11173	0.1375838
1386.5	12	0.430399	0.3494393	0.1321509	0.09616508	0.112238	0.1377897
1386.6	12	0.405788	0.3550435	0.1371132	0.09196763	0.112353	0.1360508
1386.7	12	0.420143	0.3546599	0.1361604	0.09231975	0.112606	0.1389086
1386.8	12	0.388518	0.3424321	0.1338313	0.09517808	0.112411	0.1402755
1386.9	12	0.398914	0.33549	0.1351448	0.09590509	0.111706	0.1388272
1387	12	0.38297	0.3258468	0.1334404	0.09701382	0.111248	0.1417008
1387.1	12	0.407863	0.3257786	0.1350551	0.09434182	0.111582	0.142305
1387.2	12	0.394487	0.3128599	0.1358238	0.09557999	0.111068	0.1391953
1387.3	12	0.370981	0.3267718	0.1351163	0.09521905	0.11559	0.141138
1387.4	12	0.381814	0.3170239	0.1356943	0.09354419	0.120416	0.1421897
1387.5	12	0.378827	0.3366476	0.1345067	0.09299614	0.120868	0.1414921
1387.6	12	0.385303	0.3260946	0.1332561	0.09282333	0.122163	0.1434972
1387.7	12	0.389692	0.3513004	0.1329654	0.09059805	0.121204	0.1442113
1387.8	12	0.393027	0.3581762	0.1324906	0.09221304	0.125257	0.142841
1387.9	12	0.388256	0.3371509	0.1321875	0.09023432	0.125274	0.1442696
1388	12	0.391837	0.3227197	0.130976	0.08827537	0.128174	0.1481162
1388.1	12	0.385433	0.3284643	0.1314553	0.09130502	0.130291	0.1475525
1388.2	12	0.385233	0.3417406	0.1325599	0.09434897	0.132222	0.1468819
1388.3	12	0.395183	0.3491932	0.1311652	0.09310772	0.129417	0.149368
1388.4	12	0.392206	0.3471408	0.1297573	0.09391033	0.128056	0.1527777
1388.5	12	0.409741	0.3653955	0.1303642	0.09640601	0.127459	0.148785
1388.6	12	0.447987	0.3460317	0.1290348	0.0943825	0.12584	0.1439291
1388.7	12	0.425241	0.3421162	0.1281741	0.09257221	0.126524	0.1438367
1388.8	12	0.385927	0.3436877	0.1279029	0.09363609	0.122745	0.1429512
1388.9	12	0.382475	0.3476395	0.1270203	0.09349085	0.122983	0.1428607
1389	12	0.386316	0.3509464	0.1222238	0.09202824	0.122266	0.1462097
1389.1	12	0.391042	0.3545964	0.1229967	0.09524078	0.122421	0.1452707
1389.2	12	0.388894	0.3566227	0.1226036	0.09421177	0.123735	0.1469859
1389.3	12	0.386255	0.3569285	0.1262181	0.0990725	0.12617	0.1454373
1389.4	12	0.378721	0.3454064	0.122913	0.09875703	0.123222	0.1446447
1389.5	12	0.376293	0.3350164	0.123917	0.09476124	0.12424	0.1472765
1389.6	12	0.378842	0.3322644	0.1229883	0.09337667	0.125846	0.1482254
1389.7	12	0.38867	0.3217909	0.1240519	0.09434743	0.126466	0.1476797
1389.8	12	0.392849	0.3220193	0.1267192	0.0947739	0.122455	0.1446034
1389.9	12	0.390176	0.319954	0.1281361	0.0978807	0.121871	0.1455422
1390	12	0.385792	0.3203254	0.1272654	0.09651324	0.121337	0.1435771
1390.1	12	0.386475	0.3193814	0.128512	0.09546606	0.123252	0.1455391
1390.2	12	0.382302	0.336384	0.1283001	0.09246787	0.122387	0.1461929
1390.3	12	0.386341	0.3513302	0.1298545	0.09203696	0.121817	0.1450355
1390.4	12	0.384908	0.3471963	0.1301786	0.09415475	0.120088	0.145461
1390.5	12	0.385222	0.3425024	0.1287504	0.09220312	0.120992	0.1392551
1390.6	12	0.388216	0.3448251	0.131385	0.09354588	0.120588	0.1389945
1390.7	12	0.397559	0.3554731	0.1313181	0.09139141	0.121447	0.1418857
1390.8	12	0.404862	0.3645824	0.1313405	0.09400103	0.118953	0.1432377
1390.9	12	0.393685	0.3657169	0.1339359	0.09411128	0.119069	0.1386592
1391	12	0.402203	0.3492589	0.1341677	0.09558486	0.118392	0.1395099
1391.1	12	0.410067	0.3531923	0.1377656	0.09592994	0.116865	0.1391129
1391.2	12	0.412041	0.3558202	0.1377385	0.09583139	0.11386	0.1395947
1391.3	12	0.413468	0.3616429	0.1384239	0.09693307	0.11693	0.1409136
1391.4	12	0.409542	0.3665332	0.1391468	0.09297036	0.115613	0.1414146
1391.5	12	0.405288	0.3701219	0.1406127	0.09459213	0.117331	0.1415679
1391.6	12	0.392711	0.3743569	0.1373145	0.09428678	0.116806	0.1449537
1391.7	12	0.41267	0.3728834	0.1361679	0.09378728	0.115225	0.1475131
1391.8	12	0.392137	0.3730613	0.134913	0.09216867	0.114839	0.1447463
1391.9	12	0.400649	0.3653133	0.1342747	0.09270726	0.115946	0.1442523
1392	12	0.381131	0.3530985	0.1355092	0.09541561	0.114774	0.1431235
1392.1	12	0.404666	0.3419148	0.1341102	0.09069379	0.113353	0.1391882
1392.2	12	0.396522	0.3354506	0.1320682	0.09005827	0.113585	0.1363347
1392.3	12	0.371302	0.3176481	0.1341521	0.08998755	0.115052	0.1373094
1392.4	12	0.377662	0.3248479	0.1351212	0.09310158	0.112582	0.1388919
1392.5	12	0.380792	0.322174	0.1334139	0.09585524	0.113173	0.1401378
1392.6	12	0.38522	0.3318568	0.1355957	0.09652713	0.114947	0.1363528
1392.7	12	0.385739	0.3287217	0.1341839	0.09606834	0.116254	0.1377262
1392.8	12	0.394604	0.3505454	0.1353756	0.09584139	0.117397	0.1422627
1392.9	12	0.390128	0.3432353	0.1354914	0.09279705	0.116682	0.1401919
1393	12	0.387216	0.3327926	0.1367292	0.0933171	0.119907	0.1400712
1393.1	12	0.388189	0.3333996	0.1366081	0.09612916	0.12123	0.1387035
1393.2	12	0.389548	0.3354593	0.1357201	0.09388283	0.121891	0.1391448
1393.3	12	0.395974	0.3451872	0.1336164	0.09073235	0.123048	0.1388567
1393.4	12	0.391888	0.3543932	0.1308328	0.09164186	0.126535	0.1398574
1393.5	12	0.40223	0.3504971	0.1317479	0.09447227	0.127678	0.1406589
1393.6	12	0.416218	0.3550884	0.1322575	0.09442123	0.131126	0.1441611
1393.7	12	0.407915	0.3461769	0.1316715	0.09498651	0.130856	0.1421163
1393.8	12	0.38601	0.3472011	0.1330761	0.09402065	0.126868	0.1406159
1393.9	12	0.379246	0.3499811	0.1346357	0.09422742	0.129683	0.1432299
1394	12	0.385662	0.3518942	0.1346118	0.09507015	0.125833	0.141855
1394.1	12	0.391825	0.3565257	0.1325615	0.09467094	0.122482	0.1424857
1394.2	12	0.388472	0.3563412	0.1321971	0.09549723	0.121237	0.1441062
1394.3	12	0.389472	0.3565564	0.1310878	0.09752323	0.121089	0.1443535
1394.4	12	0.382508	0.3515324	0.1324095	0.0976812	0.12398	0.1493006
1394.5	12	0.379227	0.3442761	0.1311215	0.09500988	0.12663	0.145911

1394.6	12	0.380481	0.34066	0.1292873	0.09526987	0.124919	0.1445856
1394.7	12	0.385791	0.3365317	0.1260574	0.09606515	0.122673	0.1423003
1394.8	12	0.392882	0.3312945	0.1244265	0.09559939	0.123025	0.1419671
1394.9	12	0.392303	0.3220865	0.1240056	0.09599423	0.123792	0.1421308
1395	12	0.38755	0.3247402	0.1274428	0.09650314	0.120452	0.1391125
1395.1	12	0.378132	0.3097507	0.1252509	0.09721039	0.119838	0.1386016
1395.2	12	0.378842	0.3267262	0.1273634	0.09588914	0.120676	0.1402677
1395.3	12	0.37699	0.3480913	0.1239546	0.09528344	0.123003	0.1397431
1395.4	12	0.38162	0.3571235	0.1231411	0.0961651	0.125935	0.1401343
1395.5	12	0.387873	0.3493901	0.1255878	0.09719868	0.125278	0.1420992
1395.6	12	0.391705	0.3399537	0.1250267	0.09602346	0.121436	0.1429508
1395.7	12	0.37989	0.3382946	0.1253448	0.09334608	0.119447	0.1440313
1395.8	12	0.383267	0.3428637	0.1268728	0.09467713	0.120933	0.1426872
1395.9	12	0.382688	0.3529526	0.1244771	0.0936253	0.121388	0.144681
1396	12	0.383425	0.3563611	0.1261762	0.09353354	0.118415	0.145182
1396.1	12	0.38194	0.3465555	0.1265529	0.09422529	0.116731	0.1442161
1396.2	12	0.39407	0.3512299	0.1268587	0.09303812	0.117091	0.1440826
1396.3	12	0.393536	0.3437364	0.1321462	0.09694685	0.117945	0.1448006
1396.4	12	0.404674	0.3448521	0.1345577	0.09574625	0.114212	0.1442747
1396.5	12	0.403857	0.348273	0.1340061	0.09416183	0.11571	0.1414503
1396.6	12	0.390204	0.3497555	0.1359214	0.09237839	0.113732	0.1418548
1396.7	12	0.413045	0.3562344	0.1361917	0.09254644	0.114086	0.1423443
1396.8	12	0.392297	0.3590644	0.138719	0.09544854	0.114775	0.1425679
1396.9	12	0.3994	0.3574588	0.1398409	0.09651361	0.113864	0.1413942
1397	12	0.382895	0.354163	0.1404019	0.09634748	0.115899	0.1423661
1397.1	12	0.404298	0.3443837	0.1394536	0.09412046	0.115647	0.1407916
1397.2	12	0.400846	0.3350745	0.1417034	0.09461756	0.112787	0.1404295
1397.3	12	0.373934	0.3377286	0.1408207	0.09541994	0.11092	0.1417385
1397.4	12	0.376145	0.328018	0.1378673	0.09518114	0.11209	0.1423345
1397.5	12	0.379394	0.3345444	0.1348213	0.09147961	0.1104	0.1408182
1397.6	12	0.385424	0.3195139	0.135709	0.09214048	0.109563	0.1410377
1397.7	12	0.386479	0.3257741	0.1366139	0.09058004	0.109636	0.1406521
1397.8	12	0.39105	0.3339416	0.1333462	0.08613472	0.114435	0.1406238
1397.9	12	0.390419	0.3492109	0.1320642	0.08662055	0.118112	0.1417726
1398	12	0.390204	0.3534446	0.1332319	0.08786182	0.120518	0.1413214
1398.1	12	0.389185	0.3400802	0.1363192	0.09338745	0.121018	0.1411844
1398.2	12	0.388727	0.3320633	0.1322752	0.09535255	0.12437	0.1395072
1398.3	12	0.393393	0.3306187	0.134598	0.09689172	0.122592	0.1382826
1398.4	12	0.390876	0.3385693	0.134193	0.09757668	0.125477	0.1370462
1398.5	12	0.398358	0.348957	0.1350118	0.09972078	0.126603	0.1362576
1398.6	12	0.424588	0.3456923	0.1356883	0.09725428	0.13047	0.1375047
1398.7	12	0.412341	0.34897	0.135657	0.09886926	0.129897	0.138758
1398.8	12	0.387629	0.3423023	0.1339226	0.1021867	0.133108	0.1397636
1398.9	12	0.381069	0.3415807	0.1335554	0.09728023	0.132439	0.1396093
1399	12	0.387361	0.3438561	0.1344917	0.09597703	0.129354	0.1380165
1399.1	12	0.386643	0.34682	0.1350621	0.09707214	0.131775	0.1369926
1399.2	12	0.384436	0.3492145	0.1344436	0.09806683	0.129589	0.1360531
1399.3	12	0.387061	0.3508934	0.1351201	0.1001028	0.127352	0.1392632
1399.4	12	0.383089	0.3547238	0.1356301	0.0990743	0.126048	0.1415391
1399.5	12	0.377257	0.3555652	0.1338322	0.096389	0.125785	0.1426769
1399.6	12	0.381427	0.3455867	0.1361658	0.09457912	0.1281	0.144373
1399.7	12	0.386302	0.3405616	0.1368497	0.09467524	0.127054	0.1457909
1399.8	12	0.389506	0.3398605	0.1345444	0.09572672	0.127761	0.1438757
1399.9	12	0.391806	0.3337137	0.1350596	0.09603558	0.124955	0.1438725
1400	12	0.385329	0.3294983	0.132368	0.09432983	0.124457	0.1451126
1400.1	12	0.379597	0.3289729	0.1320225	0.09715308	0.126104	0.1483647
1400.2	12	0.377435	0.3183227	0.1317563	0.09674818	0.12428	0.1510199
1400.3	12	0.39769	0.3286768	0.1309404	0.0953988	0.122663	0.145417
1400.4	12	0.395868	0.3421732	0.128725	0.09394485	0.122672	0.1444025
1400.5	12	0.399034	0.3573956	0.12679	0.09520073	0.120034	0.1455467
1400.6	12	0.397731	0.3523519	0.1269562	0.09541162	0.121845	0.1464696
1400.7	12	0.391468	0.3324177	0.1294514	0.0937425	0.120027	0.1449862
1400.8	12	0.398289	0.3274466	0.1284045	0.09283745	0.119275	0.1433583
1400.9	12	0.387171	0.3327642	0.1269803	0.09140393	0.11976	0.1438374
1401	12	0.391577	0.3434659	0.1278141	0.09168518	0.118542	0.144202
1401.1	12	0.392517	0.3487708	0.1239628	0.09118937	0.11921	0.1391306
1401.2	12	0.398588	0.3471429	0.1262237	0.09159398	0.116718	0.1398286
1401.3	12	0.399659	0.351159	0.12752	0.09382578	0.115548	0.1447176
1401.4	12	0.401209	0.3409178	0.1275585	0.09193444	0.114318	0.1455337
1401.5	12	0.402134	0.3397321	0.1281489	0.09122676	0.112533	0.1432127
1401.6	12	0.389549	0.3413929	0.1268453	0.09230787	0.113233	0.1427524
1401.7	12	0.41069	0.3425294	0.1274847	0.09314442	0.115988	0.1397845
1401.8	12	0.391193	0.3480005	0.1300877	0.09468173	0.114675	0.138967
1401.9	12	0.402397	0.3481569	0.1302619	0.09288731	0.114274	0.1414797
1402	12	0.389295	0.3502015	0.1334144	0.0946269	0.114394	0.1387476
1402.1	12	0.40749	0.3483457	0.1352661	0.09267414	0.112502	0.1393764
1402.2	12	0.399525	0.3401499	0.1367969	0.09204207	0.112379	0.1402973
1402.3	12	0.376988	0.3393201	0.1380509	0.09161327	0.113479	0.141352
1402.4	12	0.37647	0.3374585	0.1369618	0.09332895	0.113422	0.1421949
1402.5	12	0.377075	0.3246904	0.1395247	0.09313013	0.112895	0.1419578
1402.6	12	0.387339	0.3268821	0.1367785	0.09550602	0.112147	0.1399633
1402.7	12	0.387731	0.3146832	0.1375058	0.09382708	0.113315	0.1406836
1402.8	12	0.39019	0.3170846	0.1377905	0.09246742	0.111802	0.1410293
1402.9	12	0.388773	0.3215644	0.1397051	0.08900119	0.10908	0.1418189
1403	12	0.38686	0.3558211	0.1402684	0.08838506	0.113869	0.1420207
1403.1	12	0.389333	0.356057	0.138316	0.09168017	0.11733	0.1414663
1403.2	12	0.387563	0.3398385	0.1377697	0.09185482	0.118137	0.14037
1403.3	12	0.392322	0.3303017	0.1357091	0.09188984	0.121247	0.1419566
1403.4	12	0.389634	0.3281047	0.1377364	0.09371312	0.126346	0.1423835
1403.5	12	0.398555	0.3344323	0.1356359	0.09566855	0.128136	0.1408193
1403.6	12	0.418291	0.3475324	0.1351781	0.09779119	0.128978	0.1396816
1403.7	12	0.407044	0.3469292	0.1353171	0.1007313	0.130782	0.1367605
1403.8	12	0.384926	0.351194	0.1359613	0.09918405	0.134906	0.1366457
1403.9	12	0.3837	0.3456556	0.1342946	0.1007675	0.135163	0.1345381
1404	12	0.38893	0.3403008	0.1379149	0.1000041	0.134162	0.134542

1404.1	12	0.386719	0.3391505	0.1373919	0.1010193	0.133303	0.1334398
1404.2	12	0.386781	0.3431614	0.1363663	0.09775434	0.130404	0.1320509
1404.3	12	0.388501	0.3452328	0.13743	0.1012951	0.127833	0.1361261
1404.4	12	0.384927	0.3466375	0.1391332	0.1005536	0.126267	0.1392116
1404.5	12	0.379552	0.3488234	0.137145	0.09751229	0.124802	0.1387425
1404.6	12	0.38111	0.3449858	0.1347574	0.09492592	0.123556	0.1396583
1404.7	12	0.38631	0.3379765	0.1334414	0.09472246	0.122054	0.137005
1404.8	12	0.391878	0.3313634	0.1328943	0.09717132	0.126193	0.1352039
1404.9	12	0.389923	0.3354276	0.1327537	0.09686711	0.127497	0.1386714
1405	12	0.389883	0.337262	0.1311835	0.0950894	0.127844	0.1434052
1405.1	12	0.380609	0.315082	0.1325034	0.09504529	0.12191	0.1449043
1405.2	12	0.380151	0.3203945	0.1320332	0.09273861	0.12291	0.143624
1405.3	12	0.383141	0.3017657	0.13326	0.09223364	0.122442	0.1422158
1405.4	12	0.38559	0.3115537	0.1344563	0.09155267	0.11979	0.1433291
1405.5	12	0.384764	0.3275	0.1327605	0.09292659	0.117915	0.1437802
1405.6	12	0.390549	0.3551539	0.1345379	0.09338477	0.121239	0.1457146
1405.7	12	0.379275	0.3510347	0.1332432	0.09325511	0.121179	0.1493364
1405.8	12	0.381068	0.3327412	0.1304478	0.09420282	0.122212	0.1497955
1405.9	12	0.376894	0.325405	0.1281843	0.09345789	0.122115	0.1498454
1406	12	0.386531	0.329862	0.12679	0.093529	0.120955	0.1453945
1406.1	12	0.387629	0.3425449	0.1261901	0.09146702	0.119756	0.1453652
1406.2	12	0.401298	0.3486017	0.1272144	0.08949269	0.118593	0.1444842
1406.3	12	0.402559	0.345433	0.1268811	0.09316836	0.120176	0.1441614
1406.4	12	0.411894	0.3519673	0.1281885	0.09302732	0.119185	0.1435349
1406.5	12	0.408382	0.3394923	0.127482	0.09075124	0.11924	0.1422834
1406.6	12	0.392647	0.3383701	0.1281665	0.09075739	0.118397	0.1437638
1406.7	12	0.418569	0.3408642	0.1300589	0.09138092	0.117214	0.1449894
1406.8	12	0.398707	0.3424352	0.1259367	0.092196	0.115853	0.1417778
1406.9	12	0.399025	0.3477488	0.1260642	0.0943557	0.114369	0.1428013
1407	12	0.387644	0.3512828	0.1285044	0.09463165	0.113951	0.1485642
1407.1	12	0.404825	0.3485732	0.1277221	0.09145444	0.114988	0.1459151
1407.2	12	0.395167	0.3464849	0.1299149	0.09115866	0.115421	0.1428767
1407.3	12	0.36619	0.3351617	0.1306281	0.08938408	0.116106	0.1422982
1407.4	12	0.374931	0.331893	0.1331357	0.09024163	0.11526	0.1386447
1407.5	12	0.379731	0.3368498	0.1350209	0.08935152	0.116531	0.139747
1407.6	12	0.390632	0.325708	0.1342144	0.09205351	0.116368	0.1408164
1407.7	12	0.390204	0.319613	0.1347256	0.09143377	0.11532	0.1391615
1407.8	12	0.401916	0.3106799	0.136486	0.09046539	0.114486	0.1407432
1407.9	12	0.399489	0.3067921	0.1358279	0.09047017	0.114824	0.138419
1408	12	0.389798	0.3080195	0.1364951	0.09116808	0.116184	0.1389782
1408.1	12	0.387795	0.3471173	0.1393058	0.09549585	0.116987	0.1392227
1408.2	12	0.390086	0.3555899	0.1405208	0.09746275	0.119661	0.1415095
1408.3	12	0.396112	0.3438415	0.1402571	0.09889222	0.120494	0.1407714
1408.4	12	0.394732	0.3272272	0.1397792	0.09753628	0.118813	0.1417991
1408.5	12	0.402933	0.3262517	0.1405774	0.09842368	0.11942	0.1424595
1408.6	12	0.417452	0.338739	0.1406339	0.09754319	0.12057	0.1432084
1408.7	12	0.410618	0.3520653	0.1421795	0.09911191	0.124392	0.1431012
1408.8	12	0.383005	0.3458995	0.1415214	0.1006208	0.127187	0.1412769
1408.9	12	0.382893	0.3484214	0.1382848	0.09689064	0.126988	0.1408336
1409	12	0.388757	0.3463764	0.1360165	0.09697542	0.128445	0.1416539
1409.1	12	0.382271	0.3388704	0.1369547	0.09833955	0.128235	0.140404
1409.2	12	0.382728	0.3402569	0.1360789	0.0982325	0.127616	0.139575
1409.3	12	0.388564	0.3459576	0.1344996	0.09696691	0.124643	0.1353023
1409.4	12	0.380911	0.3461025	0.1352561	0.09700441	0.12428	0.1349476
1409.5	12	0.382364	0.3471757	0.1371555	0.09790125	0.125688	0.135248
1409.6	12	0.379811	0.3541977	0.1341358	0.09591122	0.126808	0.1342234
1409.7	12	0.385176	0.3515077	0.1367109	0.09373191	0.125384	0.1337244
1409.8	12	0.385994	0.345384	0.1369889	0.09382072	0.124028	0.1370052
1409.9	12	0.393195	0.3390249	0.1367357	0.09587295	0.119796	0.1358947
1410	12	0.389687	0.3404143	0.1364668	0.093921	0.120128	0.1336527
1410.1	12	0.382114	0.3396911	0.1370416	0.09512755	0.121071	0.1341354
1410.2	12	0.381484	0.316173	0.1373028	0.09113335	0.120704	0.1335793
1410.3	12	0.37534	0.3266291	0.1360422	0.09171163	0.119067	0.1339715
1410.4	12	0.377903	0.3126552	0.1370784	0.0942567	0.118619	0.1332417
1410.5	12	0.377006	0.3137007	0.1368591	0.0930724	0.118492	0.1348642
1410.6	12	0.385181	0.3324592	0.1361287	0.09348722	0.119841	0.1384231
1410.7	12	0.377869	0.3617509	0.1363717	0.09319109	0.117506	0.1399673
1410.8	12	0.385536	0.3510681	0.1349481	0.09369271	0.118002	0.1409511
1410.9	12	0.377376	0.3415209	0.1342662	0.09340551	0.120604	0.1401367
1411	12	0.391691	0.3326837	0.1352055	0.09437296	0.121188	0.1383817
1411.1	12	0.392039	0.3365746	0.1366745	0.09475994	0.11964	0.1396197
1411.2	12	0.399544	0.345158	0.1361855	0.09395467	0.119876	0.1428747
1411.3	12	0.398717	0.349221	0.1354713	0.09527028	0.121336	0.1445412
1411.4	12	0.408996	0.3456001	0.1326978	0.09629784	0.121082	0.1434981
1411.5	12	0.40474	0.3554149	0.1320334	0.09397952	0.122089	0.1409736
1411.6	12	0.39129	0.3400232	0.1302383	0.09222709	0.123457	0.1431973
1411.7	12	0.406763	0.3359458	0.1298498	0.09292394	0.126682	0.1422687
1411.8	12	0.390355	0.3403285	0.1297197	0.09274119	0.125577	0.1396339
1411.9	12	0.397716	0.3405985	0.1268069	0.09661381	0.126346	0.1411256
1412	12	0.384171	0.3446279	0.1278406	0.09884658	0.127869	0.1425425
1412.1	12	0.397748	0.3523335	0.1286801	0.09471948	0.132626	0.1418175
1412.2	12	0.396424	0.3569193	0.1285553	0.09180225	0.13125	0.1405004
1412.3	12	0.368806	0.3573551	0.1288673	0.09467752	0.130992	0.1413371
1412.4	12	0.375434	0.3471979	0.1307372	0.09548946	0.129292	0.1425313
1412.5	12	0.376803	0.3331311	0.1289756	0.09680187	0.127193	0.139931
1412.6	12	0.39011	0.3380078	0.1277553	0.09516023	0.127786	0.1386134
1412.7	12	0.389245	0.3322404	0.1308253	0.09579331	0.128668	0.1426982
1412.8	12	0.390224	0.319956	0.128945	0.09397352	0.128575	0.1403666
1412.9	12	0.386798	0.3224811	0.1284068	0.09539645	0.128461	0.1386671
1413	12	0.390061	0.3128469	0.1302102	0.09797089	0.125109	0.1395172
1413.1	12	0.388416	0.3223013	0.1329858	0.1023753	0.123542	0.138818
1413.2	12	0.385169	0.3432409	0.1344986	0.1047731	0.124103	0.1423716
1413.3	12	0.39553	0.35198	0.132908	0.102779	0.122988	0.141275
1413.4	12	0.391755	0.3501126	0.1329553	0.1030595	0.125462	0.1407606
1413.5	12	0.39303	0.3435485	0.1359719	0.1033523	0.126226	0.1420478

1413.6	12	0.437992	0.3436379	0.134877	0.1034758	0.125984	0.140048
1413.7	12	0.425819	0.3481812	0.137264	0.1016478	0.12944	0.1408024
1413.8	12	0.387428	0.356273	0.1397027	0.100754	0.127077	0.1395319
1413.9	12	0.382404	0.3529754	0.1404933	0.1011092	0.127351	0.1411976
1414	12	0.38861	0.3565311	0.1402065	0.1025676	0.129137	0.1398994
1414.1	12	0.387861	0.3501408	0.1407512	0.1022115	0.12805	0.1408318
1414.2	12	0.384819	0.349774	0.1400511	0.1017617	0.124098	0.1398369
1414.3	12	0.394129	0.3557829	0.1410393	0.09972701	0.121841	0.1400818
1414.4	12	0.38665	0.3615313	0.1404137	0.09792437	0.120898	0.1403192
1414.5	12	0.383748	0.3653419	0.1379547	0.09715445	0.116779	0.140886
1414.6	12	0.386002	0.3637131	0.1379053	0.09785779	0.118629	0.1408011
1414.7	12	0.385708	0.3640828	0.1372315	0.09628501	0.115102	0.1423988
1414.8	12	0.392802	0.3612797	0.13714	0.09681574	0.112013	0.1411466
1414.9	12	0.398534	0.3563296	0.1368048	0.09436704	0.110231	0.1421912
1415	12	0.395603	0.3483737	0.1343007	0.094496	0.110018	0.1419245
1415.1	12	0.385025	0.3432284	0.134563	0.09788939	0.112	0.1409824
1415.2	12	0.380833	0.3316135	0.1367268	0.09510856	0.115392	0.139457
1415.3	12	0.374402	0.3131742	0.136363	0.09307183	0.113532	0.1399799
1415.4	12	0.380073	0.3184245	0.1383274	0.09377287	0.115909	0.1365978
1415.5	12	0.389522	0.3098243	0.1375296	0.09339432	0.114069	0.1388226
1415.6	12	0.394357	0.3093827	0.135128	0.09285248	0.115411	0.1394474
1415.7	12	0.396828	0.3268057	0.1351322	0.09091585	0.114494	0.13871
1415.8	12	0.410616	0.3501432	0.13535	0.09402867	0.114615	0.1401989
1415.9	12	0.39996	0.3551373	0.1364329	0.0932468	0.115758	0.1408926
1416	12	0.406598	0.3357551	0.1349235	0.09379484	0.117315	0.1413075
1416.1	12	0.402478	0.3310389	0.1330633	0.09426329	0.115326	0.1401106
1416.2	12	0.407938	0.3360491	0.1327534	0.09193015	0.115996	0.1384737
1416.3	12	0.406442	0.3440356	0.1313184	0.09368499	0.119281	0.140978
1416.4	12	0.410207	0.3515477	0.1295501	0.09135288	0.116927	0.1437647
1416.5	12	0.40856	0.3467239	0.130839	0.08909934	0.1193	0.145177
1416.6	12	0.392241	0.3524478	0.1322919	0.08567351	0.122947	0.1442052
1416.7	12	0.403915	0.3385766	0.1328476	0.08642815	0.122466	0.1440928
1416.8	12	0.389476	0.3385002	0.1366213	0.08779287	0.122199	0.1436008
1416.9	12	0.396527	0.3419236	0.1332721	0.08931568	0.125133	0.1448264
1417	12	0.385777	0.3459775	0.1336794	0.09212525	0.125479	0.1464528
1417.1	12	0.397912	0.3471779	0.1332018	0.08988295	0.12711	0.1473436
1417.2	12	0.399544	0.346125	0.1339633	0.09084015	0.128238	0.1440086
1417.3	12	0.37641	0.3466394	0.1317593	0.09164938	0.129656	0.1427242
1417.4	12	0.373726	0.3430167	0.1299254	0.09364898	0.125079	0.1414398
1417.5	12	0.378277	0.3376569	0.1299329	0.09518272	0.125084	0.1388656
1417.6	12	0.389036	0.3316511	0.1280041	0.09636503	0.124916	0.1389022
1417.7	12	0.388743	0.3263485	0.1281504	0.0965533	0.121257	0.1412452
1417.8	12	0.39858	0.3152807	0.1289786	0.09439808	0.121738	0.1425846
1417.9	12	0.390174	0.3111045	0.1292837	0.09549666	0.122125	0.1416357
1418	12	0.386306	0.3120748	0.1277894	0.09510319	0.12763	0.1411423
1418.1	12	0.383561	0.3032332	0.1322769	0.0962687	0.129151	0.1406539
1418.2	12	0.385228	0.3030121	0.1300412	0.09714001	0.128029	0.1394556
1418.3	12	0.395729	0.3340689	0.1290608	0.09348954	0.123412	0.1379937
1418.4	12	0.393068	0.3633016	0.1313141	0.09392441	0.123543	0.1446207
1418.5	12	0.394757	0.3428243	0.1308167	0.09753554	0.121022	0.1435801
1418.6	12	0.409582	0.3323944	0.1309766	0.1001998	0.119893	0.1425326
1418.7	12	0.40462	0.3321145	0.1294829	0.1001812	0.125013	0.1413756
1418.8	12	0.386015	0.3389439	0.1292525	0.09927328	0.124765	0.1393307
1418.9	12	0.382121	0.3468424	0.1295792	0.1001463	0.123427	0.1396048
1419	12	0.38717	0.3446705	0.1275566	0.1010716	0.124998	0.1413345
1419.1	12	0.38293	0.356642	0.128602	0.1023711	0.124797	0.1419031
1419.2	12	0.382932	0.3459443	0.1346283	0.101609	0.121947	0.1411332
1419.3	12	0.390844	0.3387491	0.1349327	0.09974767	0.121952	0.1365942
1419.4	12	0.384958	0.3389866	0.1355909	0.09891342	0.120649	0.135401
1419.5	12	0.382333	0.3406598	0.1349393	0.09674818	0.120461	0.1405116
1419.6	12	0.376666	0.3421063	0.1360371	0.09559231	0.118642	0.1415739
1419.7	12	0.379922	0.3424971	0.1368147	0.09440605	0.115041	0.1409605
1419.8	12	0.384145	0.3417346	0.1381178	0.09268002	0.114537	0.1418302
1419.9	12	0.379751	0.3412265	0.1419899	0.09388132	0.11549	0.1399355
1420	12	0.38698	0.3382449	0.140431	0.09160554	0.113653	0.1400285
1420.1	12	0.384997	0.3342335	0.1387556	0.09508237	0.115949	0.1372792
1420.2	12	0.380908	0.3308785	0.1365945	0.09409795	0.11751	0.1367842
1420.3	12	0.372372	0.3263456	0.1377156	0.09393647	0.116823	0.136463
1420.4	12	0.378247	0.3072926	0.1380688	0.09445165	0.115989	0.1399952
1420.5	12	0.379236	0.3189441	0.1385228	0.09542657	0.11752	0.1410553
1420.6	12	0.384235	0.3056816	0.1367246	0.09501178	0.118728	0.141301
1420.7	12	0.376221	0.3105645	0.1343148	0.0946909	0.116379	0.1408065
1420.8	12	0.38003	0.3136925	0.1347606	0.0946205	0.115998	0.1358869
1420.9	12	0.374203	0.3371349	0.1361229	0.09246611	0.11664	0.1347311
1421	12	0.385446	0.3527932	0.134767	0.09434506	0.11493	0.1340553
1421.1	12	0.39108	0.3362166	0.134832	0.09338674	0.110756	0.1311563
1421.2	12	0.39827	0.3308491	0.1344419	0.09350247	0.111036	0.1326166
1421.3	12	0.399485	0.333345	0.1328902	0.09491311	0.11672	0.1328265
1421.4	12	0.409158	0.3407954	0.1351725	0.09335247	0.117686	0.1333538
1421.5	12	0.409241	0.3477758	0.1357929	0.09373594	0.122066	0.1332804
1421.6	12	0.392186	0.349403	0.135974	0.09151945	0.119623	0.130496
1421.7	12	0.407675	0.3559032	0.1332971	0.08939729	0.124811	0.1316972
1421.8	12	0.390562	0.3427386	0.1316778	0.08828709	0.128164	0.1315694
1421.9	12	0.397281	0.3394701	0.1323693	0.09126358	0.126673	0.1316357
1422	12	0.385081	0.3404181	0.1321787	0.09349468	0.127509	0.1352297
1422.1	12	0.393688	0.3407665	0.1303966	0.09413445	0.129582	0.1392475
1422.2	12	0.394497	0.3418376	0.1311535	0.09423405	0.133431	0.139629
1422.3	12	0.367189	0.3442953	0.1313143	0.09282017	0.13438	0.1408282
1422.4	12	0.377706	0.3429644	0.130026	0.0949396	0.129812	0.1420717
1422.5	12	0.376317	0.3417174	0.1305732	0.09583149	0.126603	0.1412372
1422.6	12	0.384937	0.3375661	0.1299132	0.09660868	0.127221	0.1412121
1422.7	12	0.388574	0.3308015	0.1296141	0.09485821	0.124804	0.14298
1422.8	12	0.395097	0.3261977	0.1307797	0.09247629	0.125031	0.1445507
1422.9	12	0.394394	0.3209378	0.1293384	0.09233966	0.127628	0.1428139
1423	12	0.389217	0.3223056	0.1280325	0.09268846	0.131782	0.1447437

1423.1	12	0.384318	0.3196762	0.1298839	0.09306402	0.133042	0.1445391
1423.2	12	0.387538	0.3050172	0.1293825	0.09487224	0.130884	0.1451607
1423.3	12	0.388159	0.3113155	0.1273516	0.09397508	0.127291	0.1454414
1423.4	12	0.390341	0.3317929	0.1293142	0.09330221	0.126393	0.142669
1423.5	12	0.38883	0.3463559	0.1300352	0.09601255	0.129068	0.1434669
1423.6	12	0.410983	0.3502729	0.1312825	0.0980001	0.127506	0.1415111
1423.7	12	0.405353	0.3362867	0.1286332	0.09999199	0.128066	0.1408027
1423.8	12	0.387002	0.3331867	0.1309984	0.0993093	0.12807	0.1423817
1423.9	12	0.378847	0.3396377	0.1306154	0.09910122	0.125741	0.143108
1424	12	0.384297	0.349613	0.1285566	0.1036858	0.126274	0.1406568
1424.1	12	0.383144	0.3487475	0.1295501	0.1025599	0.127299	0.1411588
1424.2	12	0.385196	0.3534959	0.1295303	0.1011239	0.123346	0.1394228
1424.3	12	0.392339	0.346983	0.1311153	0.1029545	0.119767	0.1387081
1424.4	12	0.384283	0.3418196	0.1305257	0.1039465	0.117062	0.1394847
1424.5	12	0.38057	0.3396511	0.1285601	0.1009481	0.116295	0.1373197
1424.6	12	0.376926	0.3397126	0.1298468	0.09852376	0.11424	0.1358897
1424.7	12	0.382422	0.3419465	0.1309621	0.09574832	0.112482	0.1352844
1424.8	12	0.389632	0.3441724	0.1308914	0.09698907	0.114198	0.1359839
1424.9	12	0.387063	0.344846	0.1336031	0.09695348	0.113685	0.1360089
1425	12	0.385574	0.3420982	0.1339379	0.09672449	0.114066	0.1341287
1425.1	12	0.381269	0.3388091	0.1347029	0.09797247	0.115292	0.1360496
1425.2	12	0.378467	0.3336917	0.1359937	0.0968548	0.116011	0.1358477
1425.3	12	0.37172	0.3301107	0.1351693	0.09577129	0.114315	0.137099
1425.4	12	0.376813	0.3320892	0.1382165	0.09742152	0.116723	0.1408649
1425.5	12	0.379049	0.3203847	0.1366027	0.1019819	0.117165	0.1414066
1425.6	12	0.384714	0.3248145	0.1369728	0.1011112	0.118385	0.140486
1425.7	12	0.380683	0.31414	0.1372831	0.1013093	0.11723	0.1388445
1425.8	12	0.38548	0.3145248	0.1380508	0.1025305	0.115663	0.1416694
1425.9	12	0.377201	0.3216664	0.1357485	0.1009747	0.115487	0.1389989
1426	12	0.384742	0.3410655	0.1355291	0.1012924	0.116657	0.1388061
1426.1	12	0.39249	0.3579447	0.1344991	0.09951755	0.114748	0.1407063
1426.2	12	0.400141	0.3388097	0.1318447	0.09781989	0.113059	0.1407254
1426.3	12	0.398661	0.3305872	0.1312617	0.09876183	0.116286	0.1402048
1426.4	12	0.407181	0.3314742	0.1302337	0.09540637	0.116138	0.1400036
1426.5	12	0.406088	0.3399982	0.1305718	0.09338929	0.116132	0.1395778
1426.6	12	0.39153	0.3485057	0.1338625	0.09033833	0.114477	0.1397426
1426.7	12	0.407348	0.3514522	0.1325108	0.0909098	0.119927	0.1388176
1426.8	12	0.389541	0.3579039	0.1312672	0.0939529	0.120115	0.1357449
1426.9	12	0.400352	0.3414559	0.1317616	0.09695927	0.126026	0.1360877
1427	12	0.38789	0.3376943	0.1298173	0.09972127	0.132391	0.1353888
1427.1	12	0.394702	0.3392933	0.133333	0.09940878	0.133572	0.1345104
1427.2	12	0.395434	0.3413685	0.13413	0.1000789	0.136602	0.1373328
1427.3	12	0.364431	0.3447604	0.1330458	0.0989573	0.13504	0.1384871
1427.4	12	0.376429	0.3465688	0.1323759	0.09806995	0.132005	0.1403572
1427.5	12	0.378581	0.3450486	0.1312486	0.09767275	0.128506	0.1401501
1427.6	12	0.382934	0.3466051	0.1317154	0.09835208	0.125727	0.1365764
1427.7	12	0.399044	0.3415937	0.1311649	0.09911609	0.123988	0.1370071
1427.8	12	0.393659	0.334865	0.1302264	0.09772986	0.123199	0.1393085
1427.9	12	0.399174	0.3299293	0.1313956	0.09729245	0.122209	0.1407907
1428	12	0.392366	0.3284198	0.1328224	0.09653799	0.122724	0.1404361
1428.1	12	0.382919	0.3233365	0.1315285	0.09844923	0.123954	0.140014
1428.2	12	0.379276	0.3245454	0.1326497	0.09668595	0.127547	0.1386329
1428.3	12	0.391543	0.3127452	0.1328891	0.09551732	0.121674	0.1387119
1428.4	12	0.394401	0.3136987	0.1326715	0.09306617	0.120026	0.1398422
1428.5	12	0.39404	0.3271182	0.1327287	0.09384999	0.120301	0.1438284
1428.6	12	0.410475	0.3520703	0.1317307	0.09606668	0.11934	0.1444613
1428.7	12	0.402169	0.348336	0.1298572	0.09829844	0.121449	0.1454763
1428.8	12	0.385604	0.332085	0.1308963	0.09909432	0.120784	0.1445191
1428.9	12	0.382617	0.327135	0.1289457	0.09902466	0.121654	0.1418453
1429	12	0.383482	0.3372522	0.1281098	0.1014419	0.120924	0.1418956
1429.1	12	0.386116	0.3467021	0.1277935	0.101937	0.119186	0.143252
1429.2	12	0.387477	0.351862	0.1281162	0.100263	0.119026	0.1428189
1429.3	12	0.394994	0.355149	0.129759	0.1021915	0.116735	0.1426718
1429.4	12	0.383614	0.3498821	0.1286305	0.1016187	0.113782	0.144417
1429.5	12	0.377433	0.3407916	0.1293498	0.09865016	0.112454	0.1451756
1429.6	12	0.376852	0.3393077	0.1269285	0.09933277	0.114054	0.1429831
1429.7	12	0.380985	0.3394494	0.1259851	0.09932578	0.113769	0.1385024
1429.8	12	0.388391	0.3409058	0.1255429	0.09939618	0.112471	0.1411418
1429.9	12	0.386823	0.3508122	0.1259601	0.09924973	0.109952	0.1420108
1430	12	0.385498	0.3564053	0.1275207	0.09955876	0.108641	0.1392469
1430.1	12	0.384025	0.350733	0.1300471	0.1005333	0.110015	0.1392556
1430.2	12	0.379699	0.3406777	0.1306385	0.09852417	0.109588	0.1427454
1430.3	12	0.370819	0.3377701	0.1305187	0.09922618	0.11102	0.1394742
1430.4	12	0.377916	0.3346162	0.1308732	0.1012235	0.113431	0.1394229
1430.5	12	0.376611	0.3351429	0.132427	0.09850954	0.114177	0.1390254
1430.6	12	0.383849	0.3179531	0.1356772	0.09979896	0.113997	0.1385665
1430.7	12	0.3795	0.3202164	0.1336363	0.09832482	0.114603	0.1374043
1430.8	12	0.384357	0.3130489	0.1358681	0.09630065	0.115606	0.1371364
1430.9	12	0.375215	0.3042358	0.1371024	0.09710948	0.117638	0.1353106
1431	12	0.383395	0.3092974	0.1352439	0.09647842	0.120259	0.1387271
1431.1	12	0.391681	0.3390256	0.1376723	0.09806196	0.11705	0.1422711
1431.2	12	0.396814	0.36078	0.1355728	0.09701561	0.11626	0.141329
1431.3	12	0.395458	0.3398697	0.135731	0.09813567	0.117463	0.1423834
1431.4	12	0.407951	0.3259802	0.1371655	0.09832301	0.118082	0.1425741
1431.5	12	0.419034	0.3251494	0.1384716	0.09765302	0.119106	0.1410971
1431.6	12	0.408238	0.3403655	0.136629	0.09535276	0.120537	0.1394053
1431.7	12	0.419419	0.351146	0.1350073	0.0954653	0.122913	0.1401885
1431.8	12	0.399387	0.352651	0.1336557	0.09297064	0.123946	0.1407606
1431.9	12	0.409676	0.3567271	0.1295641	0.09083775	0.126509	0.1426907
1432	12	0.396739	0.3470484	0.1297151	0.09531894	0.128245	0.1408587
1432.1	12	0.39981	0.338975	0.1262292	0.09370768	0.131261	0.1409392
1432.2	12	0.401134	0.3376445	0.1257062	0.0964236	0.133074	0.139745
1432.3	12	0.372655	0.3385367	0.1275311	0.09799917	0.133675	0.1388663
1432.4	12	0.373556	0.3427724	0.1267508	0.0980826	0.132723	0.1405759
1432.5	12	0.381586	0.3436466	0.1241432	0.09940872	0.129674	0.1386269

1432.6	12	0.385185	0.3394053	0.1268809	0.1006587	0.129418	0.1383884
1432.7	12	0.396835	0.3434125	0.1258916	0.1018041	0.127911	0.1367849
1432.8	12	0.392321	0.3389804	0.1284511	0.1002597	0.1309	0.1362519
1432.9	12	0.398814	0.332839	0.1351611	0.09885702	0.129991	0.1384321
1433	12	0.39293	0.3312249	0.135661	0.09865864	0.129487	0.1384853
1433.1	12	0.383717	0.330668	0.133089	0.09798736	0.129236	0.1366986
1433.2	12	0.381282	0.3225004	0.1325432	0.09978752	0.126569	0.139856
1433.3	12	0.392699	0.3207045	0.1337541	0.09900085	0.123101	0.1371364
1433.4	12	0.39207	0.3027885	0.1335094	0.10109	0.121274	0.1362921
1433.5	12	0.392984	0.305036	0.1312903	0.1011574	0.123186	0.1408844
1433.6	12	0.409492	0.3165284	0.1318261	0.1011428	0.12303	0.1416252
1433.7	12	0.399575	0.3455208	0.1340669	0.1008582	0.122684	0.142158
1433.8	12	0.384735	0.3531137	0.1345105	0.09940115	0.119397	0.1412038
1433.9	12	0.384187	0.3333362	0.1334737	0.1017211	0.120326	0.1380472
1434	12	0.387596	0.3243108	0.1322514	0.1038176	0.121105	0.1401251
1434.1	12	0.39726	0.3326523	0.1295278	0.1038772	0.120338	0.1427185
1434.2	12	0.396016	0.3476233	0.1325039	0.1023139	0.118965	0.1430305
1434.3	12	0.397572	0.3536342	0.1293604	0.1030536	0.121138	0.140918
1434.4	12	0.38593	0.3531733	0.1283337	0.1006373	0.119749	0.1415525
1434.5	12	0.379021	0.3584623	0.1294415	0.09757964	0.116982	0.1430002
1434.6	12	0.378288	0.3428512	0.1293194	0.09816565	0.116367	0.1440901
1434.7	12	0.3816	0.3382238	0.1261495	0.09936093	0.115665	0.143908
1434.8	12	0.401673	0.3398466	0.1272862	0.09875534	0.118413	0.1432669
1434.9	12	0.402385	0.342288	0.1255302	0.09984434	0.116067	0.1418906
1435	12	0.39073	0.347169	0.127523	0.09850033	0.115534	0.1416172
1435.1	12	0.384436	0.3463166	0.1253765	0.0980708	0.11617	0.140643
1435.2	12	0.379739	0.3462373	0.1288871	0.09646966	0.116321	0.1413358
1435.3	12	0.397446	0.3447033	0.1291719	0.09583315	0.115854	0.1419754
1435.4	12	0.400993	0.3365378	0.1273569	0.0975595	0.114737	0.1394811
1435.5	12	0.400914	0.329407	0.1256519	0.097739	0.114744	0.1399946
1435.6	12	0.40271	0.3360306	0.1274165	0.09723265	0.114668	0.1425635
1435.7	12	0.391893	0.316835	0.1261732	0.0968184	0.115345	0.1394437
1435.8	12	0.395252	0.3217591	0.1259054	0.09697148	0.113561	0.1372502
1435.9	12	0.383303	0.3095248	0.1276364	0.09597905	0.112598	0.1365819
1436	12	0.381722	0.300627	0.1307344	0.09349336	0.115203	0.1349963
1436.1	12	0.392195	0.3040586	0.1328056	0.09288044	0.113895	0.1367384
1436.2	12	0.40583	0.3304363	0.1317965	0.09249965	0.113385	0.1387282
1436.3	12	0.405967	0.350742	0.1325457	0.09354656	0.116447	0.1378517
1436.4	12	0.410997	0.3372498	0.1327826	0.09331175	0.117914	0.1367508
1436.5	12	0.405119	0.3259442	0.1358791	0.09288032	0.120402	0.1356556
1436.6	12	0.391538	0.327133	0.1359567	0.09170369	0.118548	0.135875
1436.7	12	0.406194	0.341046	0.134681	0.0911068	0.118802	0.138087
1436.8	12	0.392368	0.3504981	0.1349405	0.09327044	0.121566	0.1401572
1436.9	12	0.405232	0.3494156	0.1367047	0.09442646	0.121436	0.1386151
1437	12	0.394467	0.3619592	0.1368664	0.09479501	0.122768	0.1393931
1437.1	12	0.401807	0.3478635	0.1381646	0.09228991	0.127001	0.1395399
1437.2	12	0.401241	0.3379651	0.1414897	0.09156665	0.128411	0.1416234
1437.3	12	0.372625	0.3378381	0.1404952	0.09322336	0.129833	0.1417076
1437.4	12	0.372433	0.339545	0.1395507	0.09264923	0.129244	0.1425431
1437.5	12	0.37929	0.3439859	0.1381642	0.09447183	0.128007	0.1429793
1437.6	12	0.389246	0.346262	0.1354022	0.09268424	0.12743	0.1436569
1437.7	12	0.399562	0.3467205	0.136337	0.09251316	0.126456	0.1380661
1437.8	12	0.391594	0.3471503	0.1336094	0.09474728	0.125086	0.1360897
1437.9	12	0.398443	0.3395697	0.132608	0.09171194	0.125544	0.1358481
1438	12	0.393954	0.3320068	0.1329241	0.0897652	0.125651	0.1361483
1438.1	12	0.385803	0.3287123	0.1316754	0.09355873	0.127078	0.1369924
1438.2	12	0.395077	0.3342986	0.1312988	0.09254256	0.127134	0.1344413
1438.3	12	0.414957	0.3184526	0.1338412	0.09168288	0.124209	0.1387796
1438.4	12	0.40349	0.3179392	0.1314102	0.09122165	0.12447	0.1411001
1438.5	12	0.386223	0.2999449	0.1332995	0.09304442	0.12382	0.1406082
1438.6	12	0.386651	0.2993047	0.1355041	0.09711481	0.12202	0.1399983
1438.7	12	0.386275	0.3105204	0.1353126	0.101165	0.12428	0.1412709
1438.8	12	0.392157	0.3427951	0.1342274	0.1018599	0.124969	0.13745
1438.997	12.5	0.387492	0.331375	0.1342306	0.1019408	0.122036	0.1371569
1439.097	12.5	0.389694	0.3480413	0.134946	0.1034302	0.120426	0.1379541
1439.197	12.5	0.381465	0.3523852	0.1345309	0.1025576	0.121402	0.1397332
1439.297	12.5	0.378441	0.3567185	0.1348218	0.09241804	0.122125	0.1405136
1439.397	12.5	0.379212	0.3556169	0.1330329	0.09162483	0.120546	0.1444235
1439.497	12.5	0.39096	0.3388315	0.1326037	0.08677094	0.1181	0.1438587
1439.597	12.5	0.382935	0.3354726	0.1342026	0.08582853	0.115756	0.1415231
1439.697	12.5	0.383325	0.3379294	0.1351795	0.08554776	0.11659	0.1394811
1439.797	12.5	0.390101	0.3452785	0.1354657	0.08740714	0.116448	0.1398413
1439.897	12.5	0.382265	0.3495882	0.1321476	0.09110796	0.115087	0.1421388
1439.997	12.5	0.383773	0.3419411	0.1332506	0.09070873	0.112944	0.1461296
1440.097	12.5	0.387715	0.3415736	0.1319467	0.09214067	0.112403	0.1467711
1440.197	12.5	0.389332	0.3420729	0.1284822	0.09036813	0.113241	0.1455614
1440.297	12.5	0.389389	0.3348267	0.1280172	0.092967	0.112351	0.1450608
1440.397	12.5	0.38117	0.3288186	0.1273277	0.09493975	0.111312	0.1432169
1440.497	12.5	0.396471	0.3427163	0.129323	0.09278734	0.110427	0.1426416
1440.597	12.5	0.385128	0.3116811	0.1247908	0.09418965	0.11152	0.1425081
1440.697	12.5	0.39112	0.3195172	0.1250945	0.0941079	0.111135	0.1415438
1440.797	12.5	0.406608	0.3047373	0.124126	0.09325102	0.113333	0.1392456
1440.897	12.5	0.431931	0.2962184	0.1215392	0.09228788	0.114103	0.1403157
1440.997	12.5	0.442524	0.3030649	0.1257299	0.09543038	0.114581	0.1417929
1441.097	12.5	0.449641	0.3357406	0.1249797	0.09468087	0.113681	0.1412998
1441.197	12.5	0.437936	0.3494576	0.1242674	0.09207611	0.111181	0.1390987
1441.297	12.5	0.420738	0.3337582	0.1233975	0.09433197	0.111765	0.1405241
1441.397	12.5	0.427832	0.3227453	0.127092	0.09927655	0.116843	0.143228
1441.497	12.5	0.411684	0.3298026	0.1306042	0.09988144	0.119741	0.141926
1441.597	12.5	0.406521	0.3450819	0.1312931	0.1028201	0.121841	0.1397158
1441.697	12.5	0.403738	0.3477527	0.1328961	0.1048677	0.120219	0.1390132
1441.797	12.5	0.407949	0.3557531	0.133759	0.1031766	0.121507	0.1393693
1441.897	12.5	0.39696	0.3613511	0.1318283	0.1020161	0.122788	0.1403054
1441.997	12.5	0.376973	0.3441312	0.1353582	0.1019301	0.124693	0.1406
1442.097	12.5	0.380528	0.339465	0.1371615	0.1041698	0.126594	0.1380968

1442.197	12.5	0.393671	0.3401076	0.1378829	0.1037835	0.130567	0.1356549
1442.297	12.5	0.396686	0.3411834	0.1386491	0.1003918	0.1323	0.1344226
1442.397	12.5	0.393145	0.3439041	0.139651	0.0962212	0.133262	0.1374328
1442.497	12.5	0.402048	0.3446106	0.1402586	0.09487249	0.128353	0.1389735
1442.597	12.5	0.392717	0.3441184	0.1417344	0.09335174	0.128733	0.1419185
1442.697	12.5	0.38324	0.3381668	0.1393935	0.09363312	0.128033	0.1410807
1442.797	12.5	0.3863	0.3327054	0.1381334	0.09325698	0.125284	0.13951
1442.897	12.5	0.392408	0.3288294	0.1383844	0.09297682	0.126109	0.1394362
1442.997	12.5	0.391764	0.3390377	0.1369276	0.09336602	0.127184	0.1400575
1443.097	12.5	0.392377	0.3078513	0.1369322	0.09407478	0.128874	0.1415535
1443.197	12.5	0.415953	0.3207129	0.1353624	0.09434182	0.127713	0.140338
1443.297	12.5	0.402912	0.298999	0.1356361	0.09228998	0.127665	0.1388795
1443.397	12.5	0.38458	0.2987683	0.1388811	0.09245579	0.127018	0.1392016
1443.497	12.5	0.38555	0.322173	0.1355084	0.09380811	0.12484	0.1357799
1443.597	12.5	0.385906	0.3442264	0.133706	0.09405922	0.124079	0.1381624
1443.697	12.5	0.39075	0.352077	0.136222	0.09593128	0.125772	0.1401046
1443.797	12.5	0.38978	0.3437313	0.134427	0.09454925	0.126122	0.1373153
1443.897	12.5	0.388301	0.3437256	0.135695	0.09355113	0.12644	0.1388485
1443.997	12.5	0.3818	0.3495766	0.1372187	0.09203822	0.123956	0.1403179
1444.097	12.5	0.378627	0.3608979	0.1365188	0.09414598	0.122935	0.1397212
1444.197	12.5	0.375623	0.3629295	0.1361395	0.08980262	0.12148	0.1408831
1444.297	12.5	0.381588	0.3584962	0.1362032	0.08751206	0.120314	0.1403595
1444.397	12.5	0.37865	0.3601196	0.1363024	0.08665335	0.120097	0.1399685
1444.497	12.5	0.386157	0.3498947	0.1365032	0.08861747	0.116706	0.143263
1444.597	12.5	0.389915	0.3532123	0.1327821	0.08721781	0.114621	0.13975
1444.697	12.5	0.383127	0.362214	0.1322571	0.08903982	0.11514	0.1389162
1444.797	12.5	0.397576	0.367287	0.1321148	0.09117424	0.11474	0.1395372
1444.897	12.5	0.413597	0.3708959	0.1310217	0.08894063	0.113016	0.1380167
1444.997	12.5	0.406727	0.3693176	0.1305362	0.08802203	0.113125	0.1387596
1445.097	12.5	0.404062	0.3682439	0.1292626	0.08902571	0.114315	0.1406319
1445.197	12.5	0.392138	0.3631644	0.1288253	0.09124485	0.117026	0.1425255
1445.297	12.5	0.411716	0.3503258	0.1280541	0.0920971	0.115971	0.1463912
1445.397	12.5	0.397488	0.3412263	0.1283248	0.09043148	0.115681	0.1467994
1445.497	12.5	0.409939	0.3405697	0.1304641	0.09000953	0.114499	0.1471515
1445.597	12.5	0.431957	0.3394341	0.1287083	0.09144317	0.114207	0.1480433
1445.697	12.5	0.414058	0.337488	0.1252425	0.09228656	0.115872	0.1466189
1445.797	12.5	0.406273	0.3626767	0.1246635	0.09327639	0.117919	0.1478414
1445.897	12.5	0.413798	0.3809313	0.1267781	0.09258834	0.11539	0.1480854
1445.997	12.5	0.404833	0.3825167	0.1273646	0.09104362	0.117515	0.1481362
1446.097	12.5	0.391786	0.3835672	0.1239392	0.09433635	0.117121	0.1434964
1446.197	12.5	0.405726	0.3675477	0.1260802	0.09560457	0.114656	0.1421887
1446.297	12.5	0.402378	0.3590651	0.1240972	0.09824852	0.112966	0.1418254
1446.397	12.5	0.394648	0.363666	0.1261733	0.09747843	0.117141	0.1403937
1446.497	12.5	0.397784	0.371394	0.1282306	0.1001732	0.121416	0.1400657
1446.597	12.5	0.39589	0.3782858	0.1300107	0.09980265	0.123442	0.1428346
1446.697	12.5	0.381294	0.3781236	0.1309686	0.1007977	0.124039	0.1429334
1446.797	12.5	0.366368	0.3565746	0.1304154	0.09969219	0.127074	0.144449
1446.897	12.5	0.380137	0.356312	0.1300815	0.1008443	0.128856	0.1410603
1446.997	12.5	0.388807	0.3550853	0.1331709	0.1021869	0.128657	0.1396909
1447.097	12.5	0.391314	0.3613785	0.1323329	0.09962768	0.130073	0.143369
1447.197	12.5	0.393491	0.3686473	0.1344947	0.09417318	0.132823	0.1435201
1447.297	12.5	0.407164	0.3732643	0.1359557	0.09258987	0.131686	0.1414393
1447.397	12.5	0.4045	0.3794672	0.1371305	0.09220234	0.132753	0.1404813
1447.497	12.5	0.388274	0.3785225	0.1401069	0.09230149	0.129985	0.1380738
1447.597	12.5	0.39529	0.3742158	0.138982	0.09116697	0.127756	0.139306
1447.697	12.5	0.397479	0.3639187	0.1390306	0.09360052	0.128374	0.1415557
1447.797	12.5	0.39229	0.349663	0.1406821	0.0948075	0.128226	0.1404909
1447.897	12.5	0.398261	0.3436878	0.1431471	0.09468398	0.127545	0.1401981
1447.997	12.5	0.418279	0.3469876	0.1426803	0.09526554	0.12243	0.1360079
1448.097	12.5	0.408135	0.3629593	0.1402776	0.09380856	0.122815	0.1374124
1448.197	12.5	0.391645	0.3544414	0.13698	0.09349499	0.119584	0.1386584
1448.297	12.5	0.389963	0.3562616	0.1383514	0.0954332	0.122138	0.1399127
1448.397	12.5	0.388553	0.3614463	0.1371449	0.09655668	0.121539	0.13956
1448.497	12.5	0.393797	0.3681961	0.135857	0.09381247	0.121683	0.1426937
1448.597	12.5	0.389401	0.3674877	0.1328168	0.09343093	0.123957	0.1435543
1448.697	12.5	0.390136	0.3525147	0.132223	0.09204684	0.122107	0.1416765
1448.797	12.5	0.384995	0.3416	0.1338031	0.08874022	0.120939	0.1416992
1448.897	12.5	0.382862	0.3418564	0.1351909	0.09014748	0.119771	0.14275
1448.997	12.5	0.379457	0.3501984	0.1321592	0.0874087	0.119919	0.1402304
1449.097	12.5	0.378728	0.3598105	0.1313453	0.08826751	0.120047	0.1447691
1449.197	12.5	0.378872	0.3594558	0.1305746	0.08699501	0.120518	0.1429311
1449.297	12.5	0.390197	0.3497709	0.1312815	0.0909176	0.118498	0.142894
1449.397	12.5	0.390562	0.3440309	0.1340877	0.09213358	0.118282	0.1431282
1449.497	12.5	0.381817	0.3421658	0.1322252	0.09201495	0.11798	0.1410565
1449.597	12.5	0.384212	0.3430167	0.1321595	0.09109304	0.118453	0.138101
1449.697	12.5	0.37846	0.3455091	0.1320142	0.08663408	0.117636	0.1376394
1449.797	12.5	0.381343	0.3493467	0.1342959	0.08797359	0.115798	0.1367258
1449.897	12.5	0.380866	0.3536683	0.1340615	0.08791041	0.113851	0.1381643
1449.997	12.5	0.381122	0.3559292	0.1332467	0.09014469	0.113471	0.1367135
1450.097	12.5	0.381862	0.3509387	0.133734	0.09286696	0.11203	0.1353879
1450.197	12.5	0.374721	0.346278	0.1325245	0.09380682	0.110807	0.1397891
1450.297	12.5	0.383993	0.3370455	0.1318972	0.09381785	0.111455	0.1401189
1450.397	12.5	0.391153	0.3359022	0.1320769	0.0914731	0.112204	0.1411561
1450.497	12.5	0.400765	0.3451907	0.1301588	0.08960234	0.110847	0.1411818
1450.597	12.5	0.404832	0.355628	0.1285158	0.08977828	0.109189	0.1398934
1450.697	12.5	0.41635	0.3507994	0.1276906	0.09364726	0.108973	0.1404191
1450.797	12.5	0.408304	0.3573649	0.1280766	0.09143652	0.109243	0.1402313
1450.897	12.5	0.393589	0.3550982	0.128053	0.09022533	0.109838	0.1400708
1450.997	12.5	0.402534	0.3601489	0.1277814	0.09401973	0.111465	0.1429981
1451.097	12.5	0.386732	0.3605458	0.1264609	0.0962115	0.111624	0.1434449
1451.197	12.5	0.389746	0.3496698	0.1263302	0.09797841	0.110683	0.143356
1451.297	12.5	0.390599	0.3357782	0.1273516	0.09949259	0.110488	0.1456284
1451.397	12.5	0.39271	0.3370497	0.1286414	0.09829309	0.111561	0.1453943
1451.497	12.5	0.380408	0.3458425	0.1260327	0.09765139	0.115018	0.1498271
1451.597	12.5	0.365472	0.3569002	0.1273797	0.09841201	0.117903	0.1496074

1451.697	12.5	0.381034	0.3558099	0.1245957	0.09753344	0.115452	0.1491873
1451.797	12.5	0.387077	0.3498248	0.1250845	0.09725529	0.116656	0.143641
1451.897	12.5	0.388916	0.342352	0.1263103	0.09507248	0.12111	0.1417811
1451.997	12.5	0.39364	0.3368833	0.1253978	0.09236246	0.120644	0.1434272
1452.097	12.5	0.402239	0.3391473	0.1265287	0.09166618	0.122303	0.1450063
1452.197	12.5	0.398708	0.3436224	0.1269906	0.09281833	0.126664	0.1405686
1452.297	12.5	0.38438	0.3486975	0.1278806	0.09376955	0.126961	0.1401788
1452.397	12.5	0.393186	0.3522437	0.1312748	0.09484574	0.126429	0.1416013
1452.497	12.5	0.393124	0.3513069	0.1321572	0.0933223	0.129241	0.1441635
1452.597	12.5	0.386906	0.3487607	0.1326852	0.09201761	0.127336	0.1418896
1452.697	12.5	0.390187	0.3440362	0.1351648	0.09128393	0.127002	0.1408695
1452.797	12.5	0.408761	0.3360405	0.1306987	0.09072044	0.124874	0.1439527
1452.897	12.5	0.401176	0.334105	0.1316316	0.09299438	0.122333	0.14304
1452.997	12.5	0.388388	0.3437477	0.1356351	0.09344994	0.122222	0.1426433
1453.097	12.5	0.388424	0.3509159	0.1394359	0.09313852	0.126862	0.1400439
1453.197	12.5	0.39126	0.3523723	0.1390464	0.09423941	0.127531	0.1360794
1453.297	12.5	0.389103	0.3592308	0.1383194	0.0943632	0.130137	0.1357769
1453.397	12.5	0.388384	0.3542399	0.1379268	0.0934339	0.129135	0.1368301
1453.497	12.5	0.388957	0.3587554	0.1401876	0.08971515	0.127822	0.14064
1453.597	12.5	0.382756	0.3575451	0.1377791	0.08958539	0.126339	0.1401401
1453.697	12.5	0.378263	0.3518846	0.1339345	0.0920241	0.123077	0.1372635
1453.797	12.5	0.380985	0.3327909	0.1328821	0.08864995	0.124234	0.1379261
1453.897	12.5	0.384405	0.3312207	0.1337097	0.08657163	0.123193	0.1383673
1453.997	12.5	0.381783	0.3415664	0.1335694	0.08643548	0.125708	0.140137
1454.097	12.5	0.3851	0.3506154	0.1300339	0.08693062	0.12359	0.1390376
1454.197	12.5	0.390477	0.3542124	0.1282821	0.08632823	0.123648	0.1415137
1454.297	12.5	0.376584	0.3532736	0.1313388	0.09112586	0.124279	0.1403873
1454.397	12.5	0.378964	0.3424561	0.129876	0.09313536	0.123025	0.1384004
1454.497	12.5	0.377585	0.3351403	0.1304805	0.09073691	0.121186	0.1389439
1454.597	12.5	0.386242	0.3380886	0.1330338	0.09277428	0.120587	0.1376139
1454.697	12.5	0.386684	0.3402231	0.1332176	0.09333047	0.119617	0.1378512
1454.797	12.5	0.380943	0.3448405	0.1370774	0.09514	0.118069	0.1387034
1454.897	12.5	0.393202	0.3476805	0.1375989	0.0946424	0.117034	0.1390853
1454.997	12.5	0.38208	0.3477172	0.13532	0.09466149	0.114829	0.1388021
1455.097	12.5	0.394222	0.344373	0.1340845	0.09472126	0.116606	0.1394303
1455.197	12.5	0.402246	0.3425586	0.1324654	0.09372775	0.11586	0.1383572
1455.297	12.5	0.39799	0.3375203	0.1327206	0.09238548	0.113988	0.1380046
1455.397	12.5	0.399129	0.3367617	0.1346474	0.09348179	0.114014	0.140072
1455.497	12.5	0.417743	0.3438473	0.1331567	0.09264861	0.11241	0.1384022
1455.597	12.5	0.408532	0.346199	0.1316624	0.09164702	0.112927	0.1408006
1455.697	12.5	0.398234	0.3492735	0.1317858	0.09371152	0.113496	0.1375098
1455.797	12.5	0.403622	0.3542054	0.1308255	0.09588332	0.112643	0.136769
1455.897	12.5	0.388232	0.3457011	0.1319033	0.1005972	0.113905	0.1401693
1455.997	12.5	0.392935	0.3483726	0.1342468	0.1034315	0.114608	0.1404917
1456.097	12.5	0.391104	0.3538219	0.1332456	0.1034063	0.115355	0.1405964
1456.197	12.5	0.397551	0.3496811	0.1314172	0.09982553	0.11519	0.1397046
1456.297	12.5	0.378926	0.3350085	0.1301359	0.09997544	0.116092	0.1402725
1456.397	12.5	0.367553	0.3304924	0.1286218	0.09810895	0.116245	0.1434313
1456.497	12.5	0.382134	0.3406492	0.1261937	0.09687338	0.117621	0.1467249
1456.597	12.5	0.392298	0.3502766	0.124847	0.1003448	0.120691	0.1468522
1456.697	12.5	0.39006	0.3528542	0.1271121	0.09872512	0.123752	0.1486579
1456.797	12.5	0.395994	0.3553008	0.1277808	0.09188255	0.128047	0.148223
1456.897	12.5	0.396632	0.3464117	0.1278714	0.08973039	0.127749	0.146072
1456.997	12.5	0.401169	0.3400432	0.1265334	0.09044562	0.12674	0.1492906
1457.097	12.5	0.390995	0.3403555	0.1291014	0.09168594	0.12724	0.1497442
1457.197	12.5	0.396761	0.3405382	0.1269146	0.08887354	0.130217	0.1504686
1457.297	12.5	0.394021	0.3484679	0.1261438	0.08969171	0.129925	0.1503696
1457.397	12.5	0.391361	0.3496611	0.1253231	0.09002867	0.13044	0.1512198
1457.497	12.5	0.394546	0.3495947	0.124882	0.09173056	0.128726	0.1474008
1457.597	12.5	0.407423	0.3478215	0.1257378	0.09379151	0.124561	0.1427016
1457.697	12.5	0.404701	0.3456308	0.1272891	0.09400177	0.122697	0.1414256
1457.797	12.5	0.387992	0.3393731	0.128527	0.09413992	0.120373	0.1441351
1457.897	12.5	0.390827	0.3414515	0.132308	0.09354474	0.119204	0.1431962
1457.997	12.5	0.388699	0.3408999	0.1332162	0.09466476	0.119609	0.1438534
1458.097	12.5	0.387249	0.3450605	0.1335915	0.09702174	0.123461	0.1431365
1458.197	12.5	0.390705	0.3467176	0.1327931	0.09940284	0.125454	0.1438991
1458.297	12.5	0.390341	0.345749	0.1330841	0.09766304	0.124403	0.1429739
1458.397	12.5	0.385524	0.3384861	0.1370071	0.0971317	0.121267	0.1401703
1458.497	12.5	0.381206	0.3435732	0.1374404	0.09612364	0.121212	0.1397441
1458.597	12.5	0.385013	0.3508695	0.1403605	0.0916663	0.120256	0.1402842
1458.697	12.5	0.388146	0.3523326	0.1392676	0.08848251	0.118882	0.1419221
1458.797	12.5	0.382887	0.3339442	0.1403029	0.09192388	0.120318	0.1414971
1458.897	12.5	0.389465	0.33194	0.1399751	0.09254137	0.122376	0.1423498
1458.997	12.5	0.389487	0.3402037	0.1416076	0.09195508	0.124076	0.1407195
1459.097	12.5	0.37752	0.3503464	0.1370557	0.09428693	0.126835	0.1394097
1459.197	12.5	0.377333	0.3530306	0.1360598	0.09444801	0.124313	0.1409157
1459.297	12.5	0.376715	0.3545613	0.1351526	0.09295251	0.121082	0.1414955
1459.397	12.5	0.392964	0.3524529	0.1366975	0.0928029	0.121575	0.1400972
1459.497	12.5	0.390752	0.3420916	0.1358471	0.09358752	0.121344	0.1373767
1459.597	12.5	0.396757	0.3385956	0.13412	0.0930763	0.119158	0.134974
1459.697	12.5	0.433212	0.3400031	0.1340324	0.09382698	0.11864	0.1403375
1459.797	12.5	0.417112	0.3477878	0.1351749	0.09383462	0.118856	0.1414174
1459.897	12.5	0.428969	0.3523554	0.1320184	0.09271361	0.119171	0.140596
1459.997	12.5	0.425061	0.3499691	0.1317099	0.0952707	0.118821	0.1387401
1460.097	12.5	0.419444	0.3475811	0.1276703	0.09337043	0.118968	0.1365597
1460.197	12.5	0.416625	0.345565	0.1303205	0.09467848	0.115573	0.1387695
1460.297	12.5	0.425815	0.3358419	0.1345005	0.09602685	0.112539	0.1396985
1460.397	12.5	0.408217	0.3348728	0.1353556	0.09436432	0.113012	0.140211
1460.497	12.5	0.397698	0.3402658	0.1333693	0.0968015	0.115304	0.1393064
1460.597	12.5	0.403382	0.3413321	0.1334145	0.09871333	0.115718	0.1379406
1460.697	12.5	0.392256	0.3485521	0.131976	0.09965508	0.117405	0.136866
1460.797	12.5	0.39439	0.3437611	0.1312127	0.101704	0.116517	0.1382642
1460.897	12.5	0.395124	0.3371833	0.1303455	0.1012822	0.115741	0.1378867
1460.997	12.5	0.399718	0.3403009	0.1324844	0.09751992	0.117526	0.1381451
1461.097	12.5	0.381893	0.3518545	0.1331145	0.09692764	0.117439	0.138474

1461.197	12.5	0.369807	0.3482032	0.1342499	0.09653097	0.116555	0.140953
1461.297	12.5	0.382997	0.3351767	0.1414068	0.09891363	0.117688	0.1418148
1461.397	12.5	0.392332	0.3314025	0.1347945	0.100262	0.121292	0.139138
1461.497	12.5	0.390762	0.3390283	0.1329877	0.09851998	0.122853	0.1401087
1461.597	12.5	0.394854	0.3472284	0.1312055	0.09548637	0.121251	0.1408739
1461.697	12.5	0.402244	0.3524781	0.1304654	0.0940586	0.124232	0.1394312
1461.797	12.5	0.401756	0.3481667	0.1301386	0.09606862	0.124612	0.1422221
1461.897	12.5	0.391532	0.3496617	0.1282201	0.09501486	0.122559	0.1435578
1461.997	12.5	0.400842	0.3383059	0.1272231	0.09441914	0.123643	0.1458388
1462.097	12.5	0.397416	0.3376721	0.1242582	0.0915615	0.126143	0.1427681
1462.197	12.5	0.393566	0.3378553	0.1263199	0.09229483	0.1294	0.1440154
1462.297	12.5	0.396753	0.3451735	0.1280562	0.09283898	0.130253	0.1475148
1462.397	12.5	0.40761	0.3472972	0.1269409	0.09211332	0.1286	0.145012
1462.497	12.5	0.406332	0.3473933	0.1290564	0.0926501	0.126667	0.1453664
1462.597	12.5	0.388836	0.3471802	0.1274079	0.09374738	0.124172	0.146476
1462.697	12.5	0.388731	0.3435622	0.1242695	0.09109491	0.124402	0.1470152
1462.797	12.5	0.391172	0.3363763	0.1273126	0.09138827	0.122566	0.1429526
1462.897	12.5	0.395672	0.3345637	0.128777	0.09491622	0.12178	0.1425226
1462.997	12.5	0.391243	0.339716	0.1294057	0.0950189	0.120526	0.1447054
1463.097	12.5	0.387447	0.3402994	0.1296037	0.09580673	0.122507	0.1440986
1463.197	12.5	0.382523	0.3473938	0.1286893	0.09352799	0.12587	0.1415325
1463.297	12.5	0.382084	0.338393	0.1300692	0.09548606	0.126294	0.1408396
1463.397	12.5	0.382403	0.3305688	0.1306992	0.09421126	0.123222	0.1414765
1463.497	12.5	0.390145	0.3364919	0.1291895	0.09199781	0.122208	0.1408763
1463.597	12.5	0.38146	0.3512075	0.1315834	0.09034409	0.12407	0.1383938
1463.697	12.5	0.387524	0.3511403	0.1355819	0.08843768	0.124909	0.1392553
1463.797	12.5	0.39033	0.3334201	0.1375618	0.08871004	0.123621	0.1416015
1463.897	12.5	0.377206	0.3369984	0.1387735	0.09353355	0.121016	0.1402918
1463.997	12.5	0.382687	0.3411263	0.1365261	0.09265243	0.120653	0.1402673
1464.097	12.5	0.386415	0.3521469	0.1355263	0.09114622	0.121679	0.1411485
1464.197	12.5	0.385622	0.3548265	0.1375929	0.09231269	0.120653	0.1395348
1464.297	12.5	0.386041	0.3499003	0.1426413	0.09406508	0.118181	0.1410884
1464.397	12.5	0.383383	0.3467572	0.1444239	0.09499032	0.118051	0.141552
1464.497	12.5	0.386506	0.3406031	0.1396779	0.09559372	0.118487	0.1407082
1464.597	12.5	0.376695	0.3423611	0.1346595	0.09333675	0.116759	0.1382158
1464.697	12.5	0.391807	0.344795	0.1307202	0.09339242	0.11643	0.1392397
1464.797	12.5	0.396554	0.350961	0.1327996	0.09336437	0.115162	0.1384218
1464.897	12.5	0.390431	0.3552231	0.1360697	0.09330388	0.114631	0.1389759
1464.997	12.5	0.393573	0.354243	0.1363772	0.09715196	0.115065	0.1367154
1465.097	12.5	0.406998	0.3506544	0.1357311	0.09657823	0.113619	0.1377589
1465.197	12.5	0.401167	0.3439966	0.1370808	0.09620947	0.115327	0.1363757
1465.297	12.5	0.39193	0.3349641	0.1347105	0.09440289	0.113358	0.1376939
1465.397	12.5	0.400935	0.3319064	0.1341835	0.09654377	0.112148	0.1383368
1465.497	12.5	0.392723	0.3403324	0.1335927	0.09753282	0.115259	0.1344928
1465.597	12.5	0.396526	0.334836	0.1344983	0.09941547	0.116585	0.13536
1465.697	12.5	0.39683	0.3392896	0.1379039	0.09740328	0.1163	0.1378892
1465.797	12.5	0.401407	0.3352301	0.1366818	0.09502462	0.114229	0.1371979
1465.897	12.5	0.381048	0.3293033	0.1367503	0.09924768	0.113253	0.1377117
1465.997	12.5	0.366502	0.3312326	0.1350299	0.1005497	0.112715	0.1386739
1466.097	12.5	0.384798	0.3485411	0.1349956	0.1001915	0.112847	0.1353172
1466.197	12.5	0.3954	0.3518882	0.1366447	0.1021729	0.112461	0.1360471
1466.297	12.5	0.390246	0.3273111	0.1361444	0.1009242	0.113428	0.1354609
1466.397	12.5	0.395145	0.327546	0.134782	0.09717113	0.11472	0.140708
1466.497	12.5	0.401449	0.334765	0.1364243	0.09548134	0.117625	0.1409129
1466.597	12.5	0.401573	0.344806	0.1356193	0.09302177	0.119626	0.1416148
1466.697	12.5	0.394174	0.3498973	0.135133	0.09366395	0.121007	0.142544
1466.797	12.5	0.401217	0.3495628	0.1340173	0.0974813	0.124091	0.1414289
1466.897	12.5	0.400468	0.3461525	0.1332679	0.0987918	0.123858	0.13698
1466.997	12.5	0.3914	0.3379902	0.1339212	0.09943099	0.123874	0.1349828
1467.097	12.5	0.39923	0.3371195	0.1302615	0.09775295	0.125197	0.1380098
1467.197	12.5	0.409672	0.3387058	0.1295943	0.09670483	0.128251	0.1382641
1467.297	12.5	0.403698	0.3446652	0.1287269	0.0970802	0.128162	0.1370524
1467.397	12.5	0.38366	0.3458983	0.1277928	0.09597404	0.128215	0.1368279
1467.497	12.5	0.385148	0.3453395	0.1274585	0.09514494	0.124752	0.1379878
1467.597	12.5	0.388567	0.3429933	0.126636	0.0968985	0.122803	0.1357426
1467.697	12.5	0.388465	0.3386082	0.1273328	0.09572921	0.12336	0.1360345
1467.797	12.5	0.389685	0.3289977	0.1318983	0.09275756	0.1211	0.1390554
1467.897	12.5	0.383571	0.3283704	0.1309479	0.09360655	0.121259	0.1397577
1467.997	12.5	0.381367	0.3371333	0.1332249	0.09133258	0.123766	0.1417898
1468.097	12.5	0.3795	0.3346645	0.1290779	0.0936051	0.124556	0.1427673
1468.197	12.5	0.382891	0.3397819	0.1273797	0.09261099	0.126695	0.1419074
1468.297	12.5	0.387476	0.3341292	0.1291077	0.09151342	0.126306	0.1375047
1468.397	12.5	0.378889	0.3250811	0.1254435	0.09065443	0.122947	0.1391048
1468.497	12.5	0.388365	0.3322535	0.1268223	0.08866003	0.1212	0.1385867
1468.597	12.5	0.387682	0.3455124	0.1286666	0.08979837	0.121914	0.1378103
1468.697	12.5	0.375401	0.3428291	0.1288489	0.09333839	0.122994	0.1358892
1468.797	12.5	0.372816	0.329149	0.1303552	0.09318401	0.123186	0.1392478
1468.897	12.5	0.37341	0.327861	0.1297081	0.09365369	0.122785	0.140751
1468.997	12.5	0.378909	0.3336825	0.1295124	0.09446998	0.125234	0.1390371
1469.097	12.5	0.382739	0.3459671	0.130666	0.09529935	0.12495	0.1402083
1469.197	12.5	0.375041	0.3519593	0.1292794	0.09866713	0.124805	0.1443571
1469.297	12.5	0.385592	0.3485475	0.1309365	0.09927136	0.121803	0.1438636
1469.397	12.5	0.377824	0.347733	0.1325532	0.09927265	0.122001	0.1427785
1469.497	12.5	0.391605	0.3404419	0.1382814	0.09748413	0.122099	0.142606
1469.597	12.5	0.401852	0.3416236	0.1394312	0.09526139	0.120909	0.1426178
1469.697	12.5	0.402668	0.3440711	0.139232	0.09186109	0.12073	0.1397436
1469.797	12.5	0.404558	0.3488092	0.1408986	0.09393657	0.116389	0.1398221
1469.897	12.5	0.416023	0.3522341	0.1424917	0.09523191	0.117019	0.1409082
1469.997	12.5	0.401914	0.3506918	0.1386707	0.09465449	0.116117	0.1399913
1470.097	12.5	0.393609	0.3489956	0.1377404	0.09430242	0.114844	0.1394355
1470.197	12.5	0.400669	0.3449721	0.1358559	0.09502459	0.115215	0.1393098
1470.297	12.5	0.392255	0.332668	0.136399	0.09814887	0.114455	0.1410149
1470.397	12.5	0.399014	0.3283961	0.1372436	0.1014311	0.113763	0.1404519
1470.497	12.5	0.3972	0.3340504	0.1356505	0.1012979	0.112849	0.1409413
1470.597	12.5	0.404292	0.3329367	0.13653	0.09699654	0.113808	0.1413209

1470.697	12.5	0.37961	0.3393031	0.135358	0.09912723	0.113965	0.1396329
1470.797	12.5	0.364518	0.3323964	0.131444	0.09863205	0.113052	0.1413737
1470.897	12.5	0.38429	0.3216394	0.1350633	0.09812603	0.112678	0.1392943
1470.997	12.5	0.391809	0.3277057	0.1346826	0.09739325	0.114566	0.1388062
1471.097	12.5	0.388315	0.3445993	0.1338947	0.0954266	0.116784	0.1414333
1471.197	12.5	0.392141	0.3534968	0.1377741	0.09266491	0.118593	0.1436288
1471.297	12.5	0.398411	0.3356563	0.1385742	0.09053326	0.117306	0.1435889
1471.397	12.5	0.39968	0.3352447	0.1377916	0.08935012	0.119262	0.1429719
1471.497	12.5	0.392826	0.3459881	0.1365084	0.09257273	0.121801	0.1418931
1471.597	12.5	0.402207	0.3555462	0.1344099	0.09666967	0.125361	0.1402501
1471.697	12.5	0.40208	0.3618292	0.1351439	0.09356654	0.130703	0.1398782
1471.797	12.5	0.391884	0.3455393	0.1334347	0.09124444	0.133863	0.1401692
1471.897	12.5	0.393256	0.3434699	0.1328706	0.09254463	0.130727	0.1418778
1471.997	12.5	0.410944	0.3423369	0.1322025	0.08960824	0.127431	0.1420768
1472.097	12.5	0.404288	0.3460409	0.1324936	0.09184784	0.124787	0.1428841
1472.197	12.5	0.382389	0.3516079	0.1325202	0.09503774	0.123394	0.1455818
1472.297	12.5	0.384572	0.358618	0.1315757	0.09401459	0.121594	0.1443417
1472.397	12.5	0.392485	0.3616738	0.1328736	0.09463304	0.119873	0.1432196
1472.497	12.5	0.390994	0.3591716	0.1327119	0.0947371	0.11709	0.1421511
1472.597	12.5	0.390081	0.3578129	0.1320894	0.09477867	0.117266	0.1420511
1472.697	12.5	0.387668	0.3559482	0.1303549	0.09405998	0.119914	0.1436758
1472.797	12.5	0.38177	0.3383878	0.1275761	0.09328635	0.120258	0.1439778
1472.897	12.5	0.381044	0.3324868	0.1281498	0.09461488	0.121168	0.1463475
1472.997	12.5	0.383027	0.3400104	0.1256336	0.09329379	0.118066	0.1465204
1473.097	12.5	0.387474	0.3385378	0.1272312	0.09248149	0.11936	0.1464616
1473.197	12.5	0.383116	0.3432288	0.130513	0.09032258	0.119592	0.1481764
1473.297	12.5	0.389767	0.3338402	0.1264699	0.09130773	0.119576	0.1477149
1473.397	12.5	0.38803	0.3235688	0.126677	0.0912606	0.122853	0.1467712
1473.497	12.5	0.380154	0.3301988	0.1281628	0.09504252	0.122307	0.1466419
1473.597	12.5	0.372309	0.34681	0.1249331	0.09426913	0.12067	0.1470341
1473.697	12.5	0.375809	0.3491006	0.1261265	0.09445217	0.118363	0.144787
1473.797	12.5	0.379968	0.3288785	0.1276425	0.09323524	0.117498	0.1430545
1473.897	12.5	0.385423	0.3273824	0.1275355	0.09473555	0.117471	0.1445233
1473.997	12.5	0.381467	0.3362103	0.1310264	0.09527428	0.117941	0.1416845
1474.097	12.5	0.392803	0.3451316	0.1278496	0.09456252	0.117865	0.1392087
1474.197	12.5	0.385488	0.3497459	0.1269681	0.09119894	0.117064	0.1399926
1474.297	12.5	0.392263	0.3433229	0.1292565	0.0901743	0.115255	0.1430847
1474.397	12.5	0.396021	0.3399933	0.1259445	0.09246188	0.114126	0.1443326
1474.497	12.5	0.413828	0.338816	0.1269579	0.09009926	0.111773	0.1397652
1474.597	12.5	0.412856	0.3372529	0.1288553	0.0888716	0.111691	0.1394692
1474.697	12.5	0.422109	0.3410507	0.1307582	0.08772998	0.111693	0.1425313
1474.797	12.5	0.412388	0.3452925	0.1335316	0.08782845	0.11015	0.1442716
1474.897	12.5	0.39831	0.3460228	0.1365758	0.08663147	0.10925	0.145557
1474.997	12.5	0.399764	0.3440311	0.1391874	0.08829501	0.112893	0.1461999
1475.097	12.5	0.391602	0.3433717	0.1394628	0.09150272	0.112987	0.141864
1475.197	12.5	0.398675	0.3378736	0.1419667	0.09528914	0.112001	0.142102
1475.297	12.5	0.397889	0.3313794	0.1435496	0.09905349	0.110546	0.1430397
1475.397	12.5	0.402643	0.3301912	0.1407872	0.09836715	0.110083	0.1425817
1475.497	12.5	0.384187	0.3346522	0.1367328	0.09792749	0.110651	0.1408245
1475.597	12.5	0.364237	0.3322239	0.1356488	0.09673711	0.11043	0.1402922
1475.697	12.5	0.388243	0.3387171	0.1338722	0.09746515	0.106944	0.1426119
1475.797	12.5	0.394514	0.3294623	0.1339194	0.09561925	0.108723	0.1447095
1475.897	12.5	0.388858	0.3232259	0.1318434	0.09562701	0.111444	0.1406873
1475.997	12.5	0.389793	0.3278443	0.1333748	0.09340532	0.113562	0.1408168
1476.097	12.5	0.399021	0.3392448	0.1351847	0.09216236	0.115345	0.1410229
1476.197	12.5	0.399412	0.3459836	0.1327097	0.09078561	0.118903	0.1424627
1476.297	12.5	0.393655	0.3311156	0.1328924	0.09077085	0.118307	0.1421641
1476.397	12.5	0.401905	0.3275408	0.1333499	0.09105497	0.118142	0.1413098
1476.497	12.5	0.400965	0.3356462	0.1319773	0.09119622	0.124324	0.142183
1476.597	12.5	0.391094	0.3457915	0.134847	0.09050658	0.127512	0.1426953
1476.697	12.5	0.392948	0.348012	0.1353994	0.08954474	0.128319	0.1403022
1476.797	12.5	0.403318	0.3441379	0.137101	0.09149737	0.125706	0.1404943
1476.897	12.5	0.403951	0.341068	0.135367	0.09117702	0.122047	0.1400557
1476.997	12.5	0.382736	0.335373	0.1345742	0.09365927	0.118865	0.1368931
1477.097	12.5	0.384795	0.3354571	0.1344128	0.09289296	0.119352	0.1376833
1477.197	12.5	0.392423	0.341261	0.133764	0.09420685	0.118001	0.136648
1477.297	12.5	0.391355	0.3458756	0.1338608	0.09344275	0.118414	0.1376296
1477.397	12.5	0.391528	0.3492821	0.1335319	0.09450606	0.118289	0.1376207
1477.497	12.5	0.385438	0.3467656	0.1331142	0.09225061	0.120004	0.1389832
1477.597	12.5	0.380504	0.3473227	0.1350148	0.09066992	0.122505	0.139315
1477.697	12.5	0.380114	0.3402657	0.1358248	0.0937992	0.121583	0.1402706
1477.797	12.5	0.38034	0.3321314	0.1354871	0.08476412	0.12096	0.143337
1477.897	12.5	0.386162	0.3286072	0.1364604	0.08381287	0.121663	0.1446134
1477.997	12.5	0.37874	0.3342084	0.1355661	0.08356416	0.121201	0.1434238
1478.097	12.5	0.384923	0.3303562	0.1347651	0.08396011	0.121125	0.1419725
1478.197	12.5	0.385138	0.3379177	0.1333347	0.08328519	0.1221	0.142395
1478.297	12.5	0.379116	0.3311132	0.1306975	0.08755133	0.119012	0.1428421
1478.397	12.5	0.380293	0.3192019	0.1278497	0.08915391	0.11726	0.1451748
1478.497	12.5	0.386826	0.3203762	0.1299592	0.08909244	0.116892	0.1443612
1478.597	12.5	0.39269	0.3357268	0.1299926	0.0899376	0.11478	0.1441767
1478.697	12.5	0.39265	0.3457642	0.1306354	0.09213543	0.11471	0.1451892
1478.797	12.5	0.390407	0.3312092	0.1287487	0.09550859	0.115664	0.1464204
1478.897	12.5	0.397712	0.3295142	0.1322158	0.09505598	0.117552	0.1473197
1478.997	12.5	0.387047	0.337496	0.1298311	0.09475031	0.11537	0.1461973
1479.097	12.5	0.399467	0.3468937	0.1290019	0.09276898	0.112988	0.1492001
1479.197	12.5	0.404137	0.3510222	0.1295268	0.09061988	0.11337	0.1453538
1479.297	12.5	0.401868	0.3458628	0.1284832	0.08990013	0.113114	0.1444421
1479.397	12.5	0.401085	0.3428106	0.1295821	0.09233477	0.110519	0.1440575
1479.497	12.5	0.421233	0.337629	0.1302966	0.09135858	0.11126	0.1426968
1479.597	12.5	0.432061	0.3411169	0.129218	0.08874776	0.109655	0.1397445
1479.697	12.5	0.406256	0.342995	0.1295286	0.0865927	0.10998	0.1424508
1479.797	12.5	0.407686	0.3502231	0.1316754	0.08801486	0.11485	0.145418
1479.897	12.5	0.402404	0.3515094	0.1324167	0.09275482	0.113769	0.1441346
1479.997	12.5	0.406203	0.3482938	0.1361563	0.09659599	0.114517	0.1420301
1480.097	12.5	0.402244	0.3489152	0.1359866	0.09947255	0.11273	0.1451686

1480.197	12.5	0.415398	0.3405828	0.1371196	0.098065	0.11128	0.1430879
1480.297	12.5	0.395772	0.3311599	0.135252	0.101136	0.111551	0.1412897
1480.397	12.5	0.373856	0.329659	0.1385547	0.1022402	0.111418	0.1405034
1480.497	12.5	0.386865	0.3354212	0.1368241	0.102295	0.109125	0.1376603
1480.597	12.5	0.391568	0.3346057	0.136502	0.1010611	0.111183	0.1392551
1480.697	12.5	0.39051	0.338106	0.1395476	0.09983902	0.112348	0.1422605
1480.797	12.5	0.38993	0.3270834	0.1419396	0.09718094	0.111409	0.1440472
1480.897	12.5	0.402368	0.3181325	0.1380826	0.09608105	0.115869	0.1417754
1480.997	12.5	0.4021	0.3177777	0.1369795	0.09741562	0.114734	0.1379397
1481.097	12.5	0.392772	0.3335716	0.1381366	0.09899651	0.115737	0.1382983
1481.197	12.5	0.400091	0.3423816	0.1382852	0.09907969	0.119831	0.1417331
1481.297	12.5	0.397595	0.3292768	0.1384467	0.09588154	0.12267	0.1421878
1481.397	12.5	0.387934	0.3285609	0.1370183	0.0943554	0.120795	0.1399923
1481.497	12.5	0.394017	0.3348768	0.1350807	0.09395858	0.123837	0.139004
1481.597	12.5	0.404355	0.3481151	0.1362029	0.09290615	0.126593	0.1412033
1481.697	12.5	0.402951	0.3512806	0.1328312	0.09434134	0.124251	0.142069
1481.797	12.5	0.380436	0.3469936	0.1369103	0.09527879	0.122329	0.1404709
1481.897	12.5	0.383733	0.3487295	0.1335906	0.09485148	0.120174	0.1393568
1481.997	12.5	0.389918	0.3402435	0.1338608	0.09751406	0.118915	0.140544
1482.097	12.5	0.389445	0.3430148	0.1356441	0.09500669	0.117903	0.1438682
1482.197	12.5	0.383876	0.3450487	0.1379222	0.09280467	0.117562	0.1418058
1482.297	12.5	0.383764	0.3473511	0.1399052	0.09149393	0.118914	0.1405123
1482.397	12.5	0.380357	0.3513201	0.1370604	0.09078337	0.119654	0.1368895
1482.497	12.5	0.381913	0.3482315	0.1361302	0.09070842	0.121374	0.1328333
1482.597	12.5	0.377232	0.3478827	0.1357572	0.0882505	0.120482	0.131116
1482.697	12.5	0.383224	0.3419851	0.1351613	0.0855366	0.120775	0.1312236
1482.797	12.5	0.377566	0.3316076	0.1345401	0.08690763	0.119104	0.1334866
1482.897	12.5	0.383028	0.329547	0.1346262	0.08647282	0.117415	0.1332616
1482.997	12.5	0.38292	0.3357013	0.1354121	0.08791389	0.118558	0.1364541
1483.097	12.5	0.375757	0.3298173	0.1368061	0.09422856	0.118613	0.1405544
1483.197	12.5	0.385119	0.3360503	0.1350203	0.0935102	0.117895	0.1400143
1483.297	12.5	0.389578	0.3255233	0.1364436	0.09175543	0.11672	0.140065
1483.397	12.5	0.389308	0.3211596	0.1361282	0.09105334	0.115761	0.1376049
1483.497	12.5	0.386247	0.3173137	0.1334522	0.0905243	0.113901	0.1380411
1483.597	12.5	0.382338	0.3241254	0.1329287	0.09327365	0.114311	0.1396033
1483.697	12.5	0.394526	0.3445939	0.1323473	0.09343416	0.115701	0.1403026
1483.797	12.5	0.379437	0.3311713	0.1314759	0.09260127	0.112445	0.14108
1483.897	12.5	0.395797	0.3295626	0.1284239	0.09117075	0.111149	0.1435508
1483.997	12.5	0.412893	0.3412823	0.1286966	0.09134449	0.110107	0.1410759
1484.097	12.5	0.406436	0.3571343	0.1310191	0.09029307	0.110961	0.1414981
1484.197	12.5	0.403963	0.3605624	0.1284273	0.09404784	0.112699	0.1424321
1484.297	12.5	0.412497	0.3474231	0.127938	0.09142346	0.115221	0.1426429
1484.397	12.5	0.407668	0.3494126	0.1290718	0.09190549	0.112769	0.1427365
1484.497	12.5	0.392344	0.3393092	0.1279668	0.09121216	0.113536	0.1438039
1484.597	12.5	0.405013	0.3379826	0.1273771	0.0928714	0.114178	0.1436966
1484.697	12.5	0.400864	0.3432676	0.1261226	0.09561504	0.11634	0.1420637
1484.797	12.5	0.40631	0.34968	0.1268387	0.09974565	0.117578	0.1407134
1484.897	12.5	0.406613	0.352103	0.1280773	0.09939732	0.115374	0.1396648
1484.997	12.5	0.421531	0.3514884	0.1270657	0.09562822	0.114288	0.1392135
1485.097	12.5	0.395185	0.3468293	0.1272364	0.09778889	0.11501	0.1398799
1485.197	12.5	0.374696	0.3438337	0.1296634	0.09869572	0.116058	0.1390869
1485.297	12.5	0.391789	0.3371782	0.131014	0.09475723	0.113194	0.1400071
1485.397	12.5	0.388436	0.3302436	0.1338932	0.09782148	0.115692	0.1380588
1485.497	12.5	0.390501	0.3357313	0.1321739	0.09981342	0.120179	0.1406052
1485.597	12.5	0.388565	0.3294365	0.132908	0.09864479	0.125139	0.1403549
1485.697	12.5	0.401564	0.3437515	0.1358595	0.095672	0.124407	0.1402273
1485.797	12.5	0.397465	0.3355194	0.1400056	0.09459179	0.126624	0.1391391
1485.897	12.5	0.391844	0.3293396	0.1419396	0.09563345	0.126524	0.1379676
1485.997	12.5	0.393876	0.321624	0.1434308	0.09593517	0.123951	0.1353342
1486.097	12.5	0.392358	0.3334477	0.1432533	0.09434934	0.122	0.1371055
1486.197	12.5	0.387324	0.3475918	0.1447581	0.09566803	0.121477	0.1389915
1486.297	12.5	0.391481	0.3299825	0.1424297	0.09503721	0.124415	0.1387664
1486.397	12.5	0.400562	0.3270397	0.1384301	0.09437478	0.127588	0.1384814
1486.497	12.5	0.40091	0.3333579	0.1389883	0.09483816	0.124264	0.1398286
1486.597	12.5	0.382104	0.3474407	0.1386378	0.09489114	0.120998	0.1385623
1486.697	12.5	0.382082	0.3524669	0.1387239	0.09220501	0.121519	0.1390171
1486.797	12.5	0.391293	0.3448458	0.1367697	0.09279367	0.120807	0.1391686
1486.897	12.5	0.38896	0.3448468	0.1339615	0.09556466	0.121417	0.1396563
1486.997	12.5	0.384125	0.3369969	0.1331922	0.09395305	0.120858	0.1386769
1487.097	12.5	0.381856	0.3371083	0.1335925	0.09359964	0.121739	0.1386295
1487.197	12.5	0.380368	0.3392761	0.1329389	0.09395014	0.122087	0.1387811
1487.297	12.5	0.380448	0.3445388	0.1327423	0.09767773	0.121367	0.1384862
1487.397	12.5	0.382578	0.3470104	0.1305253	0.09515721	0.118606	0.1372633
1487.497	12.5	0.386837	0.3451907	0.1324826	0.09407596	0.120384	0.1369741
1487.597	12.5	0.381195	0.3459133	0.1331178	0.09236442	0.119013	0.1376421
1487.697	12.5	0.386404	0.3406393	0.1329412	0.09117834	0.118039	0.1380128
1487.797	12.5	0.387283	0.3338451	0.1335066	0.09051277	0.121322	0.1369434
1487.897	12.5	0.37671	0.3309319	0.1319172	0.09188195	0.120848	0.1333239
1487.997	12.5	0.384847	0.3309571	0.1331909	0.09297487	0.11881	0.1331323
1488.097	12.5	0.391002	0.326314	0.1326848	0.09329271	0.118163	0.1347243
1488.197	12.5	0.389627	0.3406695	0.1310898	0.094471	0.116455	0.1361207
1488.297	12.5	0.387619	0.3318081	0.1299137	0.09535138	0.116234	0.1380779
1488.397	12.5	0.385827	0.3202632	0.1308773	0.09903394	0.114445	0.1394594
1488.497	12.5	0.42998	0.3155807	0.1334649	0.09919707	0.113926	0.1417253
1488.597	12.5	0.432932	0.3278635	0.1338555	0.09862109	0.113782	0.1387852
1488.697	12.5	0.452059	0.3506058	0.1332695	0.0976336	0.112762	0.1382201
1488.797	12.5	0.460827	0.3255517	0.1361464	0.09832314	0.113547	0.1387472
1488.897	12.5	0.452301	0.3218458	0.1338909	0.09868135	0.112277	0.1398215
1488.997	12.5	0.454813	0.3303699	0.1329774	0.09987212	0.110704	0.1437009
1489.097	12.5	0.457361	0.344765	0.1313093	0.09888184	0.113848	0.1435449
1489.197	12.5	0.444426	0.3504277	0.1305493	0.09845531	0.11315	0.1448327
1489.297	12.5	0.4182	0.3514203	0.1276005	0.09544749	0.113503	0.1468852
1489.397	12.5	0.417159	0.3508461	0.1275671	0.09642713	0.11736	0.1461498
1489.497	12.5	0.406944	0.3403348	0.1285459	0.09842495	0.117634	0.1469166
1489.597	12.5	0.404589	0.3373335	0.1314604	0.09861191	0.116242	0.1467321

1489.697	12.5	0.402953	0.3403579	0.1263055	0.09814902	0.116386	0.147285
1489.797	12.5	0.430334	0.3451876	0.1274476	0.09649924	0.114596	0.1485507
1489.897	12.5	0.400374	0.3451559	0.1259015	0.09794594	0.115754	0.1478059
1489.997	12.5	0.376686	0.3470946	0.1259489	0.09880883	0.115819	0.1480868
1490.097	12.5	0.395262	0.3452824	0.1254541	0.09897181	0.11286	0.1448495
1490.197	12.5	0.386998	0.3393565	0.1250533	0.1018718	0.116703	0.1446096
1490.297	12.5	0.395599	0.3351488	0.1260277	0.09908739	0.11923	0.1453679
1490.397	12.5	0.393162	0.3345495	0.1259068	0.09632329	0.123104	0.145103
1490.497	12.5	0.411718	0.3368472	0.12644	0.09676167	0.12782	0.143811
1490.597	12.5	0.406986	0.3238668	0.1288165	0.09729524	0.129907	0.1434243
1490.697	12.5	0.394805	0.347222	0.1303251	0.09691495	0.130117	0.1451906
1490.797	12.5	0.392527	0.3348949	0.1333088	0.09404349	0.129476	0.1428337
1490.897	12.5	0.395709	0.3228556	0.1370605	0.09523432	0.128263	0.1405363
1490.997	12.5	0.388932	0.3165829	0.1352575	0.09322337	0.127196	0.1434487
1491.097	12.5	0.386626	0.3333803	0.1352592	0.09233652	0.129103	0.1420491
1491.197	12.5	0.408706	0.3536974	0.1370689	0.09207077	0.131276	0.1413275
1491.297	12.5	0.401155	0.3303452	0.1359444	0.09211716	0.127652	0.1409076
1491.397	12.5	0.382057	0.3249752	0.1348802	0.09168139	0.122543	0.1405022
1491.497	12.5	0.381251	0.3328034	0.1353578	0.08975053	0.121761	0.1410905
1491.597	12.5	0.389819	0.3441828	0.1383026	0.09135364	0.123239	0.1415847
1491.697	12.5	0.389991	0.353927	0.138275	0.09162605	0.121236	0.1427383
1491.797	12.5	0.386166	0.3521323	0.1339066	0.09368862	0.119703	0.1408277
1491.897	12.5	0.383382	0.3518379	0.1315839	0.09377541	0.122546	0.1408024
1491.997	12.5	0.379446	0.3411301	0.1314916	0.09308325	0.123598	0.1400689
1492.097	12.5	0.380584	0.3412815	0.1323765	0.09430744	0.12201	0.1398603
1492.197	12.5	0.380528	0.3434937	0.1343136	0.09316648	0.121941	0.1433473
1492.297	12.5	0.383569	0.3446649	0.133885	0.09408055	0.122923	0.1405601
1492.397	12.5	0.378195	0.3523122	0.1337821	0.09058756	0.123867	0.1396936
1492.497	12.5	0.384943	0.3491779	0.1350388	0.08924322	0.12254	0.1379026
1492.597	12.5	0.386676	0.3501396	0.132353	0.09044513	0.122704	0.139584
1492.697	12.5	0.378676	0.3425261	0.1327727	0.09337568	0.120698	0.1397427
1492.797	12.5	0.389695	0.33721	0.1306957	0.09302876	0.122461	0.1395326
1492.897	12.5	0.406529	0.3356377	0.1297468	0.08970705	0.123053	0.1391873
1492.997	12.5	0.395795	0.342353	0.131754	0.0895205	0.119414	0.1381334
1493.097	12.5	0.389875	0.3257594	0.1327267	0.08994196	0.118315	0.1341455
1493.197	12.5	0.385916	0.3424109	0.1315876	0.09228113	0.118749	0.1348654
1493.297	12.5	0.395332	0.3328539	0.1296917	0.09362119	0.118962	0.1357587
1493.397	12.5	0.381786	0.32499	0.1318633	0.09309109	0.115362	0.1351303
1493.497	12.5	0.387442	0.3167278	0.132873	0.09024637	0.112397	0.1330228
1493.597	12.5	0.39345	0.3347111	0.1327751	0.09492182	0.115846	0.134939
1493.697	12.5	0.392443	0.3519858	0.1350218	0.0973125	0.115279	0.1396153
1493.797	12.5	0.395975	0.3331793	0.1357345	0.09596814	0.114777	0.1393729
1493.897	12.5	0.414236	0.3256502	0.1343947	0.09393007	0.116441	0.1373613
1493.997	12.5	0.410691	0.3363143	0.1356368	0.09227484	0.114044	0.1419493
1494.097	12.5	0.389566	0.3479177	0.1356878	0.09132556	0.114018	0.1398828
1494.197	12.5	0.403167	0.3561606	0.1368513	0.09395798	0.114787	0.1411719
1494.297	12.5	0.393656	0.3548312	0.1347887	0.09530914	0.113591	0.140221
1494.397	12.5	0.398485	0.3477156	0.1330441	0.09827051	0.11297	0.1414943
1494.497	12.5	0.403431	0.3432451	0.1344846	0.09994561	0.110445	0.143408
1494.597	12.5	0.420269	0.3448194	0.1337638	0.09933742	0.109446	0.1441024
1494.697	12.5	0.393306	0.3548526	0.1330551	0.1015486	0.111919	0.1472325
1494.797	12.5	0.368884	0.3640177	0.1298399	0.1028093	0.111455	0.146791
1494.897	12.5	0.392913	0.3664556	0.1293607	0.1024406	0.109919	0.1442497
1494.997	12.5	0.383911	0.3655694	0.1304183	0.1018657	0.113097	0.1455948
1495.097	12.5	0.389798	0.3570469	0.1290294	0.1025234	0.117348	0.1429155
1495.197	12.5	0.391894	0.351216	0.1286307	0.09868852	0.122994	0.1448813
1495.297	12.5	0.398137	0.3451705	0.1288269	0.09726387	0.123334	0.1427506
1495.397	12.5	0.397129	0.3379424	0.1270654	0.09683317	0.121758	0.1440396
1495.497	12.5	0.390612	0.3365405	0.1278137	0.09630084	0.122463	0.1473031
1495.597	12.5	0.397242	0.3282572	0.1293596	0.09519613	0.124038	0.1455993
1495.697	12.5	0.390003	0.3432519	0.1279068	0.095277	0.125096	0.1442065
1495.797	12.5	0.3953	0.3323711	0.1270612	0.09429874	0.127108	0.1436043
1495.897	12.5	0.383823	0.3221653	0.124094	0.09475368	0.12826	0.1416501
1495.997	12.5	0.403995	0.3186731	0.1269806	0.09400868	0.130686	0.1390549
1496.097	12.5	0.404882	0.3392285	0.1296957	0.09431409	0.131643	0.1406045
1496.197	12.5	0.389092	0.3551592	0.1314906	0.09325662	0.126569	0.1421722
1496.297	12.5	0.387034	0.330454	0.1346441	0.09309762	0.124619	0.1414359
1496.397	12.5	0.394457	0.3256381	0.1361576	0.09293265	0.123671	0.1410268
1496.497	12.5	0.392273	0.331716	0.138848	0.09405727	0.123264	0.1421462
1496.597	12.5	0.389021	0.3440611	0.139966	0.09729606	0.122956	0.1445521
1496.697	12.5	0.383845	0.3483326	0.1387654	0.09647463	0.123352	0.1441512
1496.797	12.5	0.37736	0.3544144	0.1388519	0.09344794	0.125144	0.144629
1496.897	12.5	0.376869	0.3461263	0.1390393	0.0933061	0.124087	0.1442686
1496.997	12.5	0.377315	0.3399911	0.1391292	0.09078	0.121198	0.1427892
1497.097	12.5	0.382545	0.3379994	0.1403056	0.08973361	0.118516	0.1399785
1497.197	12.5	0.380144	0.3432584	0.1384327	0.08656386	0.119862	0.1407572
1497.297	12.5	0.384942	0.345215	0.1369816	0.0880888	0.118787	0.140885
1497.397	12.5	0.384221	0.3440832	0.1355464	0.09052555	0.121487	0.1404628
1497.497	12.5	0.375398	0.3430461	0.1342726	0.09222253	0.120109	0.1395519
1497.597	12.5	0.376501	0.3415597	0.1352075	0.09016093	0.120107	0.140774
1497.697	12.5	0.384052	0.3396598	0.1322711	0.08936331	0.120005	0.1389818
1497.797	12.5	0.382299	0.3378798	0.1316578	0.08802432	0.118794	0.1398938
1497.897	12.5	0.386258	0.3352458	0.1335716	0.08957567	0.117874	0.1401721
1497.997	12.5	0.380595	0.339174	0.1351504	0.09443114	0.116244	0.1399555
1498.097	12.5	0.387859	0.326823	0.1326887	0.096778	0.115355	0.1438262
1498.197	12.5	0.411694	0.342429	0.1315778	0.09739584	0.115228	0.1430314
1498.297	12.5	0.394522	0.3264912	0.1316625	0.09507962	0.114762	0.1411576
1498.397	12.5	0.380742	0.3169823	0.1327439	0.09256777	0.113217	0.1424026
1498.497	12.5	0.394619	0.3148788	0.1336712	0.09000896	0.112601	0.1416751
1498.597	12.5	0.380721	0.3346381	0.1358532	0.09118246	0.11305	0.1406121
1498.697	12.5	0.387044	0.3572698	0.1345907	0.09079638	0.112346	0.1408345
1498.797	12.5	0.389465	0.3317835	0.1300443	0.08933923	0.110572	0.1373865
1498.963	13	0.406727	0.3453338	0.129268	0.09101742	0.110532	0.1368268
1499.063	13	0.396798	0.3467186	0.1307832	0.09703477	0.109999	0.1361054
1499.163	13	0.390883	0.3528754	0.1341636	0.09965296	0.110777	0.1373606

1499.263	13	0.388281	0.3458025	0.1338905	0.102209	0.113371	0.1379702
1499.363	13	0.398217	0.3399256	0.1321022	0.1041737	0.113756	0.1384311
1499.463	13	0.388127	0.3389751	0.1318142	0.102394	0.110905	0.1394054
1499.563	13	0.389089	0.3441538	0.1341396	0.1002471	0.110943	0.1421358
1499.663	13	0.411245	0.3477563	0.1343365	0.09954356	0.110868	0.1419672
1499.763	13	0.392084	0.3448333	0.1361424	0.09362648	0.111214	0.1409808
1499.863	13	0.382736	0.3451418	0.1339113	0.09316771	0.112463	0.1424702
1499.963	13	0.389829	0.3422543	0.1323362	0.09448475	0.115271	0.143426
1500.063	13	0.392452	0.3384919	0.1327695	0.09399868	0.115975	0.1437679
1500.163	13	0.391648	0.3342116	0.1263327	0.09255864	0.120026	0.141639
1500.263	13	0.387125	0.3335163	0.1253251	0.09199867	0.121284	0.1441422
1500.363	13	0.383571	0.3351757	0.1289836	0.09084532	0.126675	0.1446775
1500.463	13	0.385052	0.3289236	0.1261384	0.0900402	0.128226	0.1428772
1500.563	13	0.37984	0.3340949	0.1279796	0.08945129	0.129454	0.1437652
1500.663	13	0.383895	0.3231764	0.126533	0.09126166	0.131693	0.1412477
1500.763	13	0.383413	0.3105632	0.1267846	0.08981993	0.133595	0.1445607
1500.863	13	0.386889	0.3140431	0.1274178	0.08942612	0.132049	0.1463086
1500.963	13	0.383538	0.3471907	0.1288388	0.09094331	0.132344	0.1509186
1501.063	13	0.370811	0.342885	0.1291997	0.09499378	0.128524	0.1461541
1501.163	13	0.376059	0.3255407	0.12639	0.09429912	0.127982	0.1434079
1501.263	13	0.372656	0.3281465	0.1279252	0.0941098	0.125462	0.1434928
1501.363	13	0.376003	0.3367919	0.1271894	0.0944297	0.125736	0.143341
1501.463	13	0.382947	0.3531455	0.1271312	0.09249721	0.121636	0.1405983
1501.563	13	0.3874	0.3484695	0.1299745	0.09084269	0.12485	0.1398668
1501.663	13	0.383504	0.347633	0.1294225	0.08825992	0.125085	0.1420723
1501.763	13	0.378751	0.3372033	0.1326966	0.08802566	0.122079	0.1403576
1501.863	13	0.38455	0.3350905	0.1372215	0.08910172	0.121094	0.138464
1501.963	13	0.402186	0.3374353	0.1375897	0.09021169	0.120955	0.1405762
1502.063	13	0.399185	0.3427366	0.1381891	0.08725046	0.12032	0.1427981
1502.163	13	0.413119	0.3426637	0.1393633	0.08610303	0.11839	0.1432007
1502.263	13	0.41427	0.342115	0.1415443	0.08782066	0.120353	0.1429629
1502.363	13	0.38943	0.3391835	0.1412216	0.09131511	0.118533	0.141201
1502.463	13	0.39861	0.3385083	0.1382029	0.09309789	0.118428	0.1402732
1502.563	13	0.386951	0.3320282	0.1382636	0.09504101	0.118447	0.1404553
1502.663	13	0.399409	0.3274797	0.1366878	0.09301303	0.119887	0.143275
1502.763	13	0.395871	0.3336953	0.1369933	0.09261325	0.120581	0.1405551
1502.863	13	0.424904	0.3212158	0.1365022	0.08935197	0.120956	0.1390606
1502.963	13	0.413647	0.3414544	0.134264	0.08807644	0.120995	0.1415657
1503.063	13	0.38012	0.3313452	0.1324144	0.0893044	0.118174	0.1435515
1503.163	13	0.396482	0.3225622	0.1331811	0.08795718	0.115337	0.1459086
1503.263	13	0.390949	0.3130023	0.1311508	0.08710976	0.114442	0.1423464
1503.363	13	0.385811	0.3297715	0.1337043	0.09016372	0.113857	0.1405821
1503.463	13	0.387584	0.3583426	0.1312425	0.09218045	0.114178	0.1401238
1503.563	13	0.401252	0.3309006	0.1326647	0.0920658	0.1143	0.1401577
1503.663	13	0.39754	0.3222955	0.1337532	0.09599721	0.112697	0.140409
1503.763	13	0.386698	0.3249881	0.1347416	0.09829907	0.112251	0.1396551
1503.863	13	0.386918	0.3430797	0.1339018	0.09818801	0.112339	0.1396677
1503.963	13	0.395503	0.3512802	0.1327814	0.1005695	0.112981	0.1401241
1504.063	13	0.392385	0.3526356	0.1314602	0.099981	0.11459	0.1392941
1504.163	13	0.388598	0.3504995	0.1311791	0.09962323	0.113893	0.140393
1504.263	13	0.403503	0.341295	0.1307367	0.09844697	0.112535	0.1414803
1504.363	13	0.393049	0.3378599	0.1320658	0.09513863	0.113003	0.1402971
1504.463	13	0.381002	0.3432492	0.1331483	0.09493853	0.114991	0.1394223
1504.563	13	0.38356	0.3443982	0.1347123	0.09437035	0.113271	0.1396656
1504.663	13	0.384799	0.3450195	0.1340226	0.09410184	0.118157	0.1399913
1504.763	13	0.385042	0.3431798	0.1352952	0.09498999	0.118843	0.1408893
1504.863	13	0.382444	0.3436221	0.1335048	0.09750321	0.120397	0.1425308
1504.963	13	0.378517	0.3373342	0.1327971	0.09245715	0.122902	0.1451078
1505.063	13	0.378709	0.326814	0.1332258	0.09187892	0.12071	0.1436757
1505.163	13	0.372925	0.3271271	0.1329337	0.09191344	0.121525	0.1436629
1505.263	13	0.384344	0.3322981	0.1318864	0.09290661	0.123934	0.1437436
1505.363	13	0.383014	0.3334548	0.1299143	0.09148643	0.12591	0.1439333
1505.463	13	0.389388	0.3362171	0.1290901	0.0896202	0.130823	0.1473914
1505.563	13	0.38375	0.3267831	0.1326177	0.09387311	0.132848	0.148687
1505.663	13	0.376746	0.3094159	0.1320866	0.09379813	0.131426	0.148698
1505.763	13	0.386317	0.318164	0.1332327	0.09492897	0.128097	0.1480443
1505.863	13	0.390704	0.3491133	0.1319789	0.09656581	0.126131	0.1440479
1505.963	13	0.40198	0.3405339	0.1298748	0.09893028	0.12645	0.1443383
1506.063	13	0.395634	0.3201836	0.1279413	0.09765402	0.123989	0.1455959
1506.163	13	0.416292	0.3232132	0.1279453	0.09446044	0.125583	0.1490214
1506.263	13	0.464955	0.334535	0.1284271	0.09262589	0.123151	0.1465319
1506.363	13	0.426231	0.3529672	0.1293931	0.09226031	0.124206	0.1482377
1506.463	13	0.434589	0.3503461	0.1294485	0.09223377	0.126031	0.146879
1506.563	13	0.433355	0.3468758	0.1328216	0.09306077	0.125676	0.1447666
1506.663	13	0.422883	0.338932	0.1335324	0.09285536	0.123494	0.1450132
1506.763	13	0.434616	0.3354479	0.1338404	0.09001026	0.122305	0.1451411
1506.863	13	0.45066	0.3428061	0.1343098	0.08990808	0.121722	0.1422894
1506.963	13	0.410372	0.3472418	0.1357287	0.09238422	0.122434	0.1437951
1507.063	13	0.410554	0.3472834	0.1384433	0.09577547	0.122251	0.1429697
1507.163	13	0.391494	0.3500471	0.1375654	0.09511163	0.118809	0.1445403
1507.263	13	0.400111	0.3483341	0.1427721	0.09603789	0.118865	0.1440397
1507.363	13	0.401545	0.3434074	0.1427477	0.09662862	0.118598	0.1424134
1507.463	13	0.408154	0.3360366	0.1428027	0.09511418	0.118152	0.1437014
1507.563	13	0.39409	0.3300501	0.1423851	0.09517883	0.120408	0.1457058
1507.663	13	0.373586	0.337898	0.1385651	0.09285182	0.119965	0.1447484
1507.763	13	0.392509	0.328206	0.1352555	0.09182838	0.12062	0.1423766
1507.863	13	0.395855	0.3422901	0.1350836	0.09029623	0.120283	0.1399544
1507.963	13	0.38417	0.3269194	0.1365532	0.09034185	0.119599	0.141777
1508.063	13	0.388543	0.319515	0.1365489	0.09604514	0.116376	0.1440819
1508.163	13	0.398197	0.3145139	0.1363403	0.09561621	0.114289	0.1416894
1508.263	13	0.398344	0.3223999	0.1343624	0.09471703	0.114962	0.1404635
1508.363	13	0.390645	0.3469297	0.134262	0.09432776	0.115816	0.1397129
1508.463	13	0.388822	0.3233771	0.1309768	0.09394554	0.113826	0.1422553
1508.563	13	0.396117	0.3182821	0.1330168	0.09527031	0.113927	0.1443006
1508.663	13	0.394904	0.3289414	0.1322782	0.0929402	0.113152	0.1427544

1508.763	13	0.384022	0.3464618	0.1327054	0.09644084	0.112346	0.1412386
1508.863	13	0.398183	0.3491476	0.1335679	0.09888937	0.111402	0.1410998
1508.963	13	0.393888	0.3513412	0.1357866	0.09186316	0.113438	0.1405046
1509.063	13	0.380216	0.3379757	0.134489	0.09183281	0.114152	0.1415798
1509.163	13	0.388374	0.3397438	0.1332887	0.09481364	0.113141	0.1418266
1509.263	13	0.394358	0.3414232	0.1318865	0.09557381	0.110299	0.143792
1509.363	13	0.38583	0.3462326	0.1313404	0.09290886	0.107359	0.1432434
1509.463	13	0.385544	0.3514055	0.1309346	0.09442466	0.111232	0.1435071
1509.563	13	0.383833	0.3484334	0.1329877	0.09458732	0.1104	0.1448362
1509.663	13	0.380378	0.3474876	0.1324492	0.09379959	0.112897	0.1441376
1509.763	13	0.380572	0.3453386	0.1337037	0.09283445	0.113744	0.1420754
1509.863	13	0.374533	0.3402523	0.1352807	0.09302865	0.115681	0.1415837
1509.963	13	0.380108	0.3292869	0.1336531	0.09416074	0.117212	0.1376311
1510.063	13	0.385841	0.3281877	0.132737	0.09320685	0.122906	0.1351659
1510.163	13	0.385457	0.3342173	0.1327844	0.0934995	0.124705	0.1362836
1510.263	13	0.374645	0.3311749	0.1303641	0.0938945	0.12575	0.1377175
1510.363	13	0.381847	0.3315624	0.1307895	0.09371255	0.12674	0.1394962
1510.463	13	0.382707	0.3140754	0.130859	0.09532835	0.125635	0.1413876
1510.563	13	0.387222	0.3024367	0.1289765	0.09657113	0.126684	0.1425064
1510.663	13	0.378616	0.3107465	0.128927	0.09563605	0.125418	0.1396429
1510.763	13	0.384729	0.343042	0.1311336	0.09414577	0.125697	0.1397224
1510.863	13	0.394731	0.3401792	0.1288715	0.09241954	0.123832	0.136072
1510.963	13	0.376821	0.3198612	0.1292539	0.09255138	0.123618	0.1429596
1511.063	13	0.388029	0.3202195	0.1298609	0.0941034	0.124196	0.1467531
1511.163	13	0.396656	0.3381088	0.1280442	0.09596623	0.123529	0.1482964
1511.263	13	0.391925	0.3509377	0.1268768	0.09471106	0.124311	0.1462937
1511.363	13	0.401157	0.3464941	0.1249819	0.09052058	0.123363	0.1431917
1511.463	13	0.416205	0.3433291	0.1246292	0.09061804	0.124513	0.1454089
1511.563	13	0.397709	0.3317902	0.123377	0.09208095	0.124854	0.1468431
1511.663	13	0.387392	0.3304057	0.1260038	0.09457088	0.123742	0.1492279
1511.763	13	0.397938	0.3343757	0.1295506	0.09399802	0.12253	0.1454495
1511.863	13	0.395095	0.3380252	0.1314483	0.09254257	0.120968	0.1474374
1511.963	13	0.398875	0.3391665	0.1305581	0.09200674	0.117268	0.1441812
1512.063	13	0.408375	0.3379417	0.1318755	0.08880008	0.117306	0.1423754
1512.163	13	0.408025	0.3380061	0.1331902	0.09147937	0.117685	0.1409861
1512.263	13	0.392341	0.3359291	0.1331548	0.09139831	0.117401	0.1403812
1512.363	13	0.384477	0.3259541	0.1345091	0.09049032	0.118393	0.136205
1512.463	13	0.395377	0.3237666	0.139036	0.09026944	0.116913	0.1402736
1512.563	13	0.389949	0.3352442	0.1370092	0.0916798	0.115639	0.1416968
1512.663	13	0.385124	0.3253381	0.1378926	0.09456334	0.113863	0.1418726
1512.763	13	0.39668	0.3367873	0.1371594	0.09365511	0.114108	0.1375133
1512.863	13	0.400611	0.3244578	0.1394311	0.09249686	0.112802	0.135439
1512.963	13	0.390044	0.3068546	0.1371446	0.09041974	0.111551	0.1378085
1513.063	13	0.388654	0.3105559	0.1373515	0.0919169	0.111088	0.1394177
1513.163	13	0.392245	0.3226305	0.1367082	0.09568822	0.111567	0.1363153
1513.263	13	0.394846	0.3477545	0.137303	0.09695812	0.112605	0.1362982
1513.363	13	0.381961	0.3217506	0.1350513	0.09867671	0.112134	0.1367766
1513.463	13	0.395724	0.3174866	0.1335592	0.09625184	0.113465	0.1372775
1513.563	13	0.395286	0.3233326	0.1381139	0.09541493	0.114298	0.1395937
1513.663	13	0.379379	0.3440899	0.1345702	0.0942717	0.112759	0.1391113
1513.763	13	0.382879	0.3494868	0.1352704	0.09523378	0.109499	0.1385716
1513.863	13	0.387311	0.3503994	0.1347781	0.09697402	0.111024	0.1378963
1513.963	13	0.381315	0.3406662	0.1325196	0.09761605	0.111795	0.1379312
1514.063	13	0.384181	0.3388546	0.1355345	0.0974383	0.11602	0.1389237
1514.163	13	0.381621	0.3392392	0.13869	0.09686714	0.112883	0.1367169
1514.263	13	0.378484	0.3461727	0.1417489	0.0967377	0.115537	0.1363958
1514.363	13	0.37844	0.35114	0.1395916	0.095111	0.115814	0.138604
1514.463	13	0.373397	0.3511246	0.1402198	0.09371943	0.118633	0.1379413
1514.563	13	0.378322	0.3501697	0.1418088	0.09203947	0.120708	0.1383778
1514.663	13	0.386855	0.3486366	0.1405904	0.09142718	0.121448	0.1399886
1514.763	13	0.388244	0.3362268	0.1413044	0.09161821	0.121527	0.1397495
1514.863	13	0.377115	0.3326818	0.1391099	0.09254383	0.122231	0.1418225
1514.963	13	0.367012	0.3336133	0.1403059	0.0923102	0.123616	0.1387806
1515.063	13	0.376998	0.3295579	0.1411754	0.09269282	0.12535	0.1385705
1515.163	13	0.375916	0.3385603	0.1384967	0.09311157	0.129051	0.1368967
1515.263	13	0.379107	0.333726	0.1392205	0.09443911	0.130682	0.1343173
1515.363	13	0.382295	0.3221334	0.1380625	0.09242165	0.129737	0.1352187
1515.463	13	0.39007	0.3057325	0.135387	0.09307569	0.125655	0.134044
1515.563	13	0.381554	0.3206351	0.1352252	0.09480159	0.123515	0.1355475
1515.663	13	0.390108	0.3497866	0.1344577	0.09333752	0.120249	0.1363688
1515.763	13	0.395886	0.3341903	0.1339638	0.09588677	0.118802	0.1360914
1515.863	13	0.39235	0.3239463	0.1347077	0.09716648	0.119048	0.137353
1515.963	13	0.398751	0.3263502	0.1368384	0.09591825	0.118675	0.1400125
1516.063	13	0.408967	0.3392757	0.1347505	0.09497203	0.118756	0.1413618
1516.163	13	0.401515	0.3507392	0.1339309	0.09501874	0.115685	0.1404124
1516.263	13	0.386384	0.3462163	0.134622	0.09631627	0.118059	0.1404078
1516.363	13	0.397969	0.3444703	0.1332664	0.09620213	0.118813	0.1398535
1516.463	13	0.393726	0.3360958	0.1345182	0.09445879	0.118282	0.1410571
1516.563	13	0.403257	0.3351243	0.1331487	0.09325147	0.113755	0.1411835
1516.663	13	0.410434	0.3394594	0.1299799	0.09610015	0.114359	0.1427817
1516.763	13	0.413011	0.3397575	0.1294113	0.09335315	0.11468	0.1399813
1516.863	13	0.386867	0.3428982	0.1286709	0.09374914	0.11584	0.1404919
1516.963	13	0.386522	0.3453286	0.128856	0.09179632	0.114607	0.1437182
1517.063	13	0.39047	0.3455431	0.1332795	0.09012617	0.113542	0.1460607
1517.163	13	0.395576	0.3360708	0.1347956	0.09215391	0.114191	0.1472676
1517.263	13	0.381716	0.3335947	0.1363291	0.09541517	0.114657	0.1468272
1517.363	13	0.394546	0.3335145	0.1377974	0.09744252	0.116378	0.1478156
1517.463	13	0.409085	0.3317863	0.1403318	0.09689985	0.115942	0.1455829
1517.563	13	0.392865	0.3302635	0.1410831	0.09737361	0.114782	0.143179
1517.663	13	0.388055	0.3394314	0.1418958	0.09714448	0.115459	0.1430682
1517.763	13	0.390754	0.3227756	0.1411333	0.09674647	0.115292	0.1440361
1517.863	13	0.39631	0.3099869	0.139754	0.09648794	0.116604	0.1433817
1517.963	13	0.385687	0.3089201	0.1392101	0.09809176	0.1164	0.1448417
1518.063	13	0.391447	0.3345664	0.1396074	0.09856492	0.116533	0.1452729
1518.163	13	0.396788	0.3515976	0.1371273	0.09646359	0.116606	0.1423832

1518.263	13	0.383496	0.3209758	0.1365752	0.09625754	0.116044	0.1394968
1518.363	13	0.389048	0.318829	0.1350535	0.096458	0.114606	0.1439242
1518.463	13	0.407181	0.3321214	0.1350041	0.09565923	0.117301	0.1419126
1518.563	13	0.408858	0.3414153	0.134535	0.09624486	0.115501	0.1432662
1518.663	13	0.398513	0.3482997	0.1322885	0.09635923	0.112635	0.1422777
1518.763	13	0.385745	0.3466199	0.1348106	0.09409785	0.116123	0.1424269
1518.863	13	0.380079	0.339217	0.1324668	0.09422762	0.117217	0.1424693
1518.963	13	0.382842	0.3355188	0.1308057	0.09466921	0.11306	0.1431649
1519.063	13	0.3796	0.3332195	0.1297067	0.09547525	0.114124	0.1434944
1519.163	13	0.393168	0.3364177	0.1311903	0.09469152	0.114957	0.1431005
1519.263	13	0.393788	0.3413844	0.1362649	0.0933708	0.116999	0.1399929
1519.363	13	0.391513	0.3398158	0.1383648	0.09533113	0.117669	0.1414267
1519.463	13	0.384068	0.340595	0.1377709	0.09527681	0.117369	0.1454568
1519.563	13	0.371486	0.3386997	0.1357087	0.09413962	0.118534	0.1454333
1519.663	13	0.395541	0.3352515	0.1377072	0.09270437	0.123036	0.1444452
1519.763	13	0.387861	0.3348102	0.1364868	0.09276658	0.125829	0.146185
1519.863	13	0.390429	0.3327001	0.135137	0.09301528	0.125663	0.1450382
1519.963	13	0.400205	0.3280741	0.1365802	0.0891721	0.129764	0.1440122
1520.063	13	0.421858	0.3332403	0.1355476	0.08880056	0.130543	0.1431277
1520.163	13	0.414411	0.3376164	0.1372266	0.0893516	0.129102	0.1426485
1520.263	13	0.409892	0.3162898	0.1379969	0.08930861	0.126183	0.1407993
1520.363	13	0.417117	0.3029203	0.1348601	0.09088697	0.124883	0.1385283
1520.463	13	0.413475	0.3164645	0.1339277	0.09198935	0.122354	0.1364258
1520.563	13	0.412733	0.3488179	0.1326901	0.09118174	0.122103	0.1350898
1520.663	13	0.420045	0.3369955	0.1309633	0.09148447	0.122595	0.1364466
1520.763	13	0.415083	0.3179817	0.1313272	0.09311242	0.124026	0.1359112
1520.863	13	0.38899	0.3211542	0.1316395	0.09303954	0.124282	0.134988
1520.963	13	0.398547	0.3352626	0.1312352	0.09366247	0.124463	0.135456
1521.063	13	0.39308	0.3474863	0.1335968	0.09448566	0.124549	0.1392777
1521.163	13	0.39548	0.350232	0.1339233	0.09280211	0.123863	0.1398274
1521.263	13	0.400434	0.3498574	0.1334643	0.09346444	0.12282	0.1407334
1521.363	13	0.405707	0.3380041	0.1313529	0.09156045	0.121721	0.1402247
1521.463	13	0.383103	0.3376403	0.1308552	0.08998345	0.122204	0.1431365
1521.563	13	0.37887	0.3415634	0.1286941	0.08989682	0.121526	0.1423855
1521.663	13	0.394167	0.346368	0.12682	0.09055112	0.121189	0.1448791
1521.763	13	0.399318	0.3483966	0.1263669	0.09053189	0.120242	0.1468465
1521.863	13	0.387565	0.3524854	0.1268544	0.09270588	0.120818	0.14766
1521.963	13	0.393372	0.3520258	0.1268367	0.09315939	0.118573	0.1458814
1522.063	13	0.401687	0.3442665	0.1289242	0.09440619	0.119256	0.1476291
1522.163	13	0.390083	0.334469	0.1307674	0.09380329	0.119139	0.1492899
1522.263	13	0.389316	0.3330257	0.1309491	0.09579489	0.116487	0.1503406
1522.363	13	0.392357	0.3299772	0.1291175	0.09715043	0.115756	0.1489295
1522.463	13	0.398579	0.3260715	0.1315635	0.100576	0.115294	0.1506452
1522.563	13	0.38747	0.3400039	0.1343557	0.1040371	0.115211	0.1466355
1522.663	13	0.395938	0.3251147	0.1366934	0.1009638	0.116222	0.1419848
1522.763	13	0.396801	0.3106723	0.1383939	0.0980959	0.11545	0.1398718
1522.863	13	0.385108	0.3085447	0.1398215	0.09588636	0.115935	0.1406545
1522.963	13	0.379031	0.334811	0.1372702	0.09649345	0.117007	0.1413559
1523.063	13	0.396381	0.3602349	0.1357127	0.09852891	0.117871	0.1409251
1523.163	13	0.397248	0.3251037	0.1362321	0.09825442	0.118661	0.1417731
1523.263	13	0.387656	0.3166187	0.1392269	0.09402335	0.11612	0.1390882
1523.363	13	0.378595	0.3290828	0.1381379	0.09374177	0.116163	0.1391004
1523.463	13	0.378789	0.3450872	0.1379147	0.09386482	0.115675	0.1412103
1523.563	13	0.37769	0.349452	0.1361612	0.0926645	0.116433	0.138253
1523.663	13	0.376641	0.3519778	0.1373835	0.09119539	0.118061	0.1381014
1523.763	13	0.381874	0.3407679	0.136067	0.09283958	0.116472	0.1361234
1523.863	13	0.389062	0.3351759	0.1338219	0.09182805	0.11908	0.1339661
1523.963	13	0.39751	0.3336739	0.1339256	0.09015375	0.121085	0.1367595
1524.063	13	0.386385	0.33437	0.1316669	0.09279848	0.115509	0.137453
1524.163	13	0.372061	0.3393934	0.1299381	0.09401499	0.115177	0.1378664
1524.263	13	0.387171	0.3384615	0.1280686	0.09200032	0.117627	0.1349432
1524.363	13	0.380231	0.3411186	0.1271568	0.09066597	0.120905	0.1353276
1524.463	13	0.387332	0.3447046	0.1307103	0.09340219	0.123503	0.1390825
1524.563	13	0.384407	0.3368805	0.1321236	0.09233709	0.128109	0.1391701
1524.663	13	0.401032	0.3367082	0.1347139	0.09169592	0.130468	0.1376907
1524.763	13	0.387722	0.3362812	0.1330977	0.09276298	0.131531	0.1383531
1524.863	13	0.383137	0.3352609	0.1335885	0.09220853	0.130308	0.1385392
1524.963	13	0.392105	0.3389869	0.1330283	0.09263285	0.12703	0.1381862
1525.063	13	0.399252	0.3481522	0.1303719	0.09537404	0.125808	0.1379193
1525.163	13	0.39847	0.328963	0.1301382	0.09240933	0.126981	0.1362616
1525.263	13	0.416655	0.3165188	0.1309221	0.09300597	0.124738	0.136306
1525.363	13	0.42392	0.3258947	0.1313129	0.09415478	0.121605	0.1374546
1525.463	13	0.398294	0.3599565	0.1346062	0.09750433	0.123401	0.1362465
1525.563	13	0.397718	0.3452874	0.1326434	0.09791782	0.124863	0.1346507
1525.663	13	0.395418	0.324707	0.1305525	0.09644605	0.125491	0.1333874
1525.763	13	0.398365	0.3233004	0.1299533	0.09507547	0.123206	0.1330068
1525.863	13	0.408346	0.3378095	0.1307046	0.09650502	0.120322	0.1321637
1525.963	13	0.429265	0.348126	0.1293993	0.09475087	0.120395	0.135694
1526.063	13	0.400666	0.3525524	0.1310474	0.09489858	0.119392	0.1366135
1526.163	13	0.386111	0.3564853	0.129603	0.09466396	0.121087	0.1363405
1526.263	13	0.406467	0.3423041	0.129655	0.09309795	0.122779	0.1401813
1526.363	13	0.395118	0.3389084	0.1292691	0.09564371	0.120699	0.141542
1526.463	13	0.385527	0.3446281	0.1297484	0.09728388	0.120364	0.1413762
1526.563	13	0.389944	0.3477946	0.128183	0.09990401	0.119379	0.1398135
1526.663	13	0.40787	0.3476705	0.1309228	0.09959827	0.117591	0.1379302
1526.763	13	0.395887	0.3450798	0.1279936	0.09871341	0.115667	0.1362863
1526.863	13	0.391632	0.3475406	0.1270134	0.09711957	0.114949	0.1408261
1526.963	13	0.393713	0.3432806	0.1249326	0.09972258	0.114659	0.1439721
1527.063	13	0.402103	0.3379082	0.1236939	0.09910743	0.113483	0.1461401
1527.163	13	0.388385	0.3318478	0.1275363	0.09986419	0.11235	0.1436268
1527.263	13	0.393585	0.3344388	0.1278314	0.1016553	0.113876	0.1432723
1527.363	13	0.398907	0.3291656	0.131434	0.09962027	0.113668	0.1453886
1527.463	13	0.38736	0.3426215	0.1327896	0.0972856	0.11193	0.1473283
1527.563	13	0.38092	0.327903	0.1304474	0.09575209	0.111294	0.1462196
1527.663	13	0.390905	0.3157194	0.1317016	0.09537169	0.111318	0.1459281

1527.763	13	0.393179	0.3240144	0.1290922	0.09487744	0.111439	0.1435145
1527.863	13	0.393439	0.3379218	0.1325242	0.09408297	0.110757	0.1397611
1527.963	13	0.383528	0.3537473	0.1348966	0.09595107	0.112162	0.1407909
1528.063	13	0.387909	0.338511	0.1365811	0.09312238	0.113892	0.1407838
1528.163	13	0.386977	0.3372121	0.1359209	0.09309553	0.115276	0.1383337
1528.263	13	0.383508	0.3402389	0.1367349	0.09353961	0.112654	0.137608
1528.363	13	0.388919	0.3561879	0.1395276	0.09184841	0.11288	0.1390741
1528.463	13	0.391157	0.3535878	0.1411735	0.09346187	0.113926	0.1374107
1528.563	13	0.389309	0.3544718	0.1368836	0.09298453	0.113927	0.1371677
1528.663	13	0.389165	0.3487222	0.1362337	0.09377394	0.117873	0.1368742
1528.763	13	0.382397	0.3439773	0.1347308	0.09422617	0.114769	0.1355577
1528.863	13	0.415975	0.3474123	0.1343336	0.09319749	0.113894	0.1388175
1528.963	13	0.422609	0.353361	0.135008	0.09159477	0.119009	0.1385017
1529.063	13	0.422349	0.3591006	0.1335389	0.09287227	0.118458	0.1378596
1529.163	13	0.416966	0.3617367	0.1317558	0.09110134	0.118772	0.1401027
1529.263	13	0.41837	0.3598852	0.1319615	0.09050698	0.120909	0.1399004
1529.363	13	0.403542	0.3556593	0.130284	0.09073494	0.122114	0.13987
1529.463	13	0.38418	0.3547398	0.1305597	0.09117227	0.121476	0.1384015
1529.563	13	0.395012	0.3432797	0.1297777	0.09270486	0.124882	0.136376
1529.663	13	0.406161	0.3384828	0.129117	0.09396617	0.128205	0.1369479
1529.763	13	0.39649	0.3362448	0.1311058	0.0902393	0.125865	0.1395253
1529.863	13	0.403986	0.3417809	0.1293226	0.09064427	0.124245	0.1405074
1529.963	13	0.410903	0.3488032	0.130359	0.09029457	0.121429	0.1406211
1530.063	13	0.390719	0.3219498	0.1295397	0.09083859	0.122673	0.1383624
1530.163	13	0.392146	0.311691	0.1319042	0.09267561	0.124862	0.1358718
1530.263	13	0.392098	0.3374512	0.1296669	0.09231111	0.127647	0.136646
1530.363	13	0.393437	0.3616117	0.1319625	0.0942201	0.127061	0.1370876
1530.463	13	0.394593	0.3495576	0.1325501	0.09503566	0.126562	0.1388133
1530.563	13	0.407588	0.3419473	0.1315101	0.0946264	0.123424	0.1372501
1530.663	13	0.385866	0.344497	0.1331685	0.09484643	0.121937	0.1341645
1530.763	13	0.362379	0.3531308	0.1319831	0.09621467	0.12067	0.1351579
1530.863	13	0.387477	0.3638805	0.1313131	0.09357181	0.118315	0.1334552
1530.963	13	0.38781	0.354826	0.1335614	0.08954273	0.117861	0.1350694
1531.063	13	0.384313	0.3538524	0.1322217	0.09167822	0.119602	0.1354856
1531.163	13	0.391823	0.3467121	0.1307901	0.09784464	0.11905	0.1350807
1531.263	13	0.393977	0.3493421	0.1310934	0.1002042	0.117291	0.1372898
1531.363	13	0.388909	0.3545459	0.1305131	0.09981145	0.116722	0.1378752
1531.463	13	0.382309	0.3587305	0.1309991	0.09769902	0.117431	0.1413117
1531.563	13	0.390688	0.3629296	0.1297443	0.09983809	0.113425	0.1423061
1531.663	13	0.404898	0.361258	0.1310702	0.1013168	0.112338	0.1430526
1531.763	13	0.392041	0.3577508	0.1269109	0.1004692	0.112139	0.1413679
1531.863	13	0.393903	0.3529063	0.1274105	0.09957617	0.11089	0.1410805
1531.963	13	0.395044	0.3459307	0.126154	0.0964794	0.109369	0.1402593
1532.063	13	0.390194	0.342191	0.1269406	0.0913615	0.108275	0.1411039
1532.163	13	0.383999	0.3487437	0.1267127	0.09132773	0.10974	0.1420754
1532.263	13	0.389524	0.3404158	0.1232059	0.09095025	0.109326	0.1443535
1532.363	13	0.394568	0.3493991	0.124738	0.09056248	0.110326	0.1446931
1532.463	13	0.388585	0.3295004	0.1258424	0.09099025	0.110493	0.1439991
1532.563	13	0.386982	0.3144086	0.1275653	0.09149779	0.111748	0.1436453
1532.663	13	0.38311	0.3164668	0.1301799	0.08995833	0.109768	0.1440769
1532.763	13	0.38136	0.3466979	0.1298471	0.09176738	0.106482	0.1441969
1532.863	13	0.382544	0.3532006	0.1321041	0.09120599	0.107612	0.1442926
1532.963	13	0.379911	0.3325142	0.1313416	0.09112663	0.109415	0.1438535
1533.063	13	0.382308	0.3301247	0.1330893	0.0926732	0.109725	0.1404045
1533.163	13	0.388154	0.3375521	0.1374954	0.09194387	0.113402	0.1384324
1533.263	13	0.39171	0.3490805	0.1392718	0.09083382	0.114213	0.1356242
1533.363	13	0.38486	0.3476653	0.1385189	0.09106762	0.117657	0.1365495
1533.463	13	0.382528	0.3547705	0.139881	0.09228232	0.117445	0.1368814
1533.563	13	0.372469	0.3386903	0.1384412	0.09239046	0.119476	0.1379997
1533.663	13	0.372089	0.3352172	0.139239	0.09289062	0.118707	0.1394926
1533.763	13	0.374223	0.3347605	0.1382145	0.09138189	0.120365	0.1383912
1533.863	13	0.391887	0.3398246	0.1367037	0.08936237	0.126967	0.1418515
1533.963	13	0.386991	0.3426754	0.1341012	0.08824687	0.127143	0.1417149
1534.063	13	0.380549	0.3445989	0.1315874	0.08837345	0.126535	0.1408846
1534.163	13	0.390378	0.3450712	0.1304636	0.08941074	0.127281	0.1398407
1534.263	13	0.399777	0.3435253	0.1294405	0.0930234	0.128236	0.1388479
1534.363	13	0.395977	0.3427241	0.1285596	0.09217165	0.128791	0.1372025
1534.463	13	0.404651	0.3316787	0.1319577	0.09171125	0.126185	0.1407516
1534.563	13	0.412906	0.3325741	0.1298349	0.09367221	0.126001	0.1424339
1534.663	13	0.392098	0.3407256	0.133471	0.09506551	0.125378	0.1397018
1534.763	13	0.393584	0.3487307	0.1318501	0.09623478	0.127959	0.1375114
1534.863	13	0.400538	0.3453195	0.1326061	0.09497877	0.128545	0.1390744
1534.963	13	0.399623	0.32548	0.1343716	0.09375961	0.12799	0.1400365
1535.063	13	0.401205	0.3127216	0.1339903	0.09448098	0.124646	0.1400651
1535.163	13	0.405282	0.3323902	0.1323044	0.09462844	0.123772	0.1400962
1535.263	13	0.386266	0.3649749	0.1298671	0.09402912	0.122721	0.1385363
1535.363	13	0.361385	0.3390602	0.1300696	0.0967865	0.119505	0.1369441
1535.463	13	0.384057	0.325601	0.1291534	0.09318635	0.119596	0.1384654
1535.563	13	0.387848	0.3291109	0.1307439	0.09332596	0.117519	0.1390166
1535.663	13	0.383821	0.3405448	0.1288534	0.09709296	0.11954	0.1393136
1535.763	13	0.38993	0.3505101	0.1284396	0.1005859	0.118735	0.1385924
1535.863	13	0.387771	0.3517602	0.129523	0.1009873	0.116932	0.1371784
1535.963	13	0.391972	0.3534441	0.1282786	0.1039292	0.116131	0.1355505
1536.063	13	0.385886	0.333836	0.1267456	0.1036053	0.115019	0.1362344
1536.163	13	0.390155	0.330616	0.1296972	0.1039992	0.114563	0.1352676
1536.263	13	0.398279	0.3356505	0.1271802	0.1025404	0.11227	0.1347697
1536.363	13	0.393204	0.3377489	0.1249157	0.1000714	0.110868	0.1325779
1536.463	13	0.388755	0.3422165	0.1261309	0.09933805	0.110023	0.1321432
1536.563	13	0.393104	0.3425446	0.126258	0.09969393	0.110369	0.1313198
1536.663	13	0.389528	0.3431166	0.1250813	0.09472697	0.109914	0.1358096
1536.763	13	0.381365	0.3415307	0.1241171	0.09080642	0.111541	0.1384777
1536.863	13	0.388409	0.3316054	0.1263215	0.09384316	0.11193	0.138344
1536.963	13	0.388943	0.3280506	0.1264276	0.09564567	0.11122	0.1380032
1537.063	13	0.392222	0.3435956	0.1263531	0.0937512	0.110091	0.1375055
1537.163	13	0.383418	0.338686	0.124572	0.09510194	0.110024	0.1380808

1537.263	13	0.379575	0.3467081	0.1217759	0.09140895	0.110667	0.141709
1537.363	13	0.377487	0.3299998	0.1217051	0.09139393	0.111014	0.1403891
1537.463	13	0.379207	0.3093369	0.1210934	0.09247444	0.111636	0.1407143
1537.563	13	0.378592	0.3116268	0.1229336	0.09432028	0.109316	0.140339
1537.663	13	0.377818	0.3488389	0.1219869	0.09307615	0.110726	0.1412318
1537.763	13	0.393751	0.3546591	0.1262756	0.09174502	0.118184	0.1456402
1537.863	13	0.399362	0.3274281	0.1290667	0.09314078	0.12062	0.148269
1537.963	13	0.389539	0.3292595	0.1307825	0.09381407	0.121941	0.1443156
1538.063	13	0.398581	0.3350807	0.1373565	0.09413313	0.122856	0.1438807
1538.163	13	0.398937	0.3490115	0.1392203	0.09119876	0.12696	0.1447843
1538.263	13	0.379637	0.3469485	0.1409222	0.09336521	0.12565	0.1414697
1538.363	13	0.380411	0.3609807	0.1412747	0.09300283	0.12431	0.1386337
1538.463	13	0.397964	0.3418655	0.1398118	0.09142821	0.12611	0.1392954
1538.563	13	0.399208	0.3356073	0.1388932	0.09079153	0.128418	0.1403856
1538.663	13	0.38766	0.337166	0.1361771	0.09087078	0.131014	0.1406824
1538.763	13	0.393293	0.3427472	0.137429	0.08960255	0.128061	0.139181
1538.863	13	0.401904	0.3451012	0.135681	0.08904454	0.125241	0.139611
1538.963	13	0.396369	0.3466226	0.1337443	0.08895176	0.126545	0.1349358
1539.063	13	0.402041	0.3492793	0.1343412	0.08773383	0.125298	0.1364286
1539.163	13	0.409307	0.3481276	0.1326336	0.08942141	0.125762	0.1391232
1539.263	13	0.395627	0.337413	0.1321586	0.0900337	0.122826	0.1402469
1539.363	13	0.393569	0.3313341	0.1332358	0.09181436	0.123511	0.138696
1539.463	13	0.394887	0.3362623	0.129929	0.09144565	0.12235	0.1393041
1539.563	13	0.394536	0.3374055	0.1285651	0.09035756	0.120345	0.1372002
1539.663	13	0.39857	0.339797	0.1337803	0.09186929	0.118598	0.1379602
1539.763	13	0.409989	0.3411099	0.1299846	0.08991129	0.116323	0.1385002
1539.863	13	0.397438	0.3132623	0.1310924	0.09050719	0.11637	0.1355274
1539.963	13	0.365529	0.3121547	0.1295067	0.0908608	0.114257	0.1340602
1540.063	13	0.383804	0.3310054	0.1279029	0.08889538	0.114153	0.1324617
1540.163	13	0.392154	0.3638166	0.1289285	0.08904224	0.113914	0.1340546
1540.263	13	0.389068	0.3423063	0.1286726	0.09173667	0.116872	0.1358795
1540.363	13	0.391626	0.3347082	0.1300239	0.09411518	0.116633	0.1346509
1540.463	13	0.389482	0.3327894	0.1303346	0.09491023	0.116669	0.1338275
1540.563	13	0.392431	0.3437375	0.1307701	0.09347361	0.118208	0.1333089
1540.663	13	0.380713	0.3519994	0.1281841	0.09335588	0.116561	0.1357263
1540.763	13	0.389263	0.3544896	0.1282706	0.09633653	0.114231	0.1350541
1540.863	13	0.399911	0.3549347	0.1285204	0.09781338	0.111969	0.1340474
1540.963	13	0.394358	0.3376626	0.1282585	0.09642405	0.111105	0.137125
1541.063	13	0.383939	0.3394653	0.1314983	0.0982376	0.108599	0.1357692
1541.163	13	0.394385	0.3449341	0.1325735	0.0979147	0.106849	0.1385435
1541.263	13	0.390569	0.3485549	0.1313329	0.0946078	0.107294	0.1371875
1541.363	13	0.385324	0.3541219	0.1320382	0.09046549	0.107338	0.1345191
1541.463	13	0.385759	0.3552015	0.1299839	0.09098064	0.107918	0.1332933
1541.563	13	0.389869	0.3533984	0.1286129	0.09088327	0.107999	0.1315458
1541.663	13	0.391696	0.3505671	0.1289981	0.09354307	0.106915	0.1312895
1541.763	13	0.386617	0.3406737	0.128866	0.0924316	0.107864	0.1329663
1541.863	13	0.381253	0.3352731	0.1271233	0.09178187	0.105535	0.1337553
1541.963	13	0.378516	0.3424801	0.1290971	0.0923181	0.10504	0.1344718
1542.063	13	0.378657	0.3304822	0.1288204	0.09227623	0.108446	0.1360841
1542.163	13	0.375747	0.3405819	0.1245796	0.09160098	0.109922	0.1364857
1542.263	13	0.378687	0.327741	0.1249845	0.09272991	0.108084	0.1357246
1542.363	13	0.386295	0.3127197	0.1221866	0.09314508	0.113233	0.13657
1542.463	13	0.396303	0.3146084	0.1207451	0.0935447	0.114614	0.1393981
1542.563	13	0.387424	0.3440487	0.1236353	0.09311255	0.11637	0.1370824
1542.663	13	0.37746	0.3557412	0.1234132	0.09329042	0.118499	0.1404937
1542.763	13	0.381336	0.3302034	0.1243289	0.08974224	0.120209	0.1428629
1542.863	13	0.377794	0.330517	0.1243581	0.08821175	0.123567	0.1405077
1542.963	13	0.37822	0.3342005	0.127298	0.0879331	0.125914	0.1454053
1543.063	13	0.386154	0.34498	0.1277154	0.08695779	0.124758	0.1491396
1543.163	13	0.393534	0.3489542	0.1284585	0.08674131	0.125916	0.1484017
1543.263	13	0.384447	0.3622017	0.1348866	0.08484873	0.128857	0.1479695
1543.363	13	0.390622	0.3391264	0.1352239	0.08664395	0.129189	0.1476981
1543.463	13	0.399932	0.3341289	0.1365028	0.08934641	0.12781	0.1444886
1543.563	13	0.398448	0.3339634	0.1394908	0.08977906	0.128485	0.142449
1543.663	13	0.39698	0.3368653	0.1390892	0.09075862	0.128106	0.1414943
1543.763	13	0.410884	0.3418744	0.1411004	0.09327019	0.130218	0.1405969
1543.863	13	0.403948	0.3438586	0.1400749	0.0967644	0.128783	0.1388728
1543.963	13	0.392338	0.3421683	0.1406534	0.09678029	0.130169	0.1396043
1544.063	13	0.398137	0.3402645	0.1397902	0.09608533	0.128749	0.1414659
1544.163	13	0.394506	0.3375682	0.1368552	0.09376786	0.129081	0.1381675
1544.263	13	0.399755	0.33099	0.1357557	0.09051646	0.122717	0.1404611
1544.363	13	0.402859	0.3321677	0.1356834	0.08970493	0.120404	0.1422881
1544.463	13	0.402431	0.3410127	0.1293377	0.08681898	0.123065	0.1438921
1544.563	13	0.370142	0.3357176	0.1319436	0.09014966	0.119146	0.1424011
1544.663	13	0.373241	0.3435391	0.1286767	0.0926109	0.119325	0.1404635
1544.763	13	0.387206	0.3198197	0.1282507	0.08944346	0.117591	0.1401045
1544.863	13	0.393994	0.3049458	0.1331977	0.0896846	0.117885	0.1405133
1544.963	13	0.389466	0.330698	0.1339227	0.08821148	0.116402	0.1387135
1545.063	13	0.390473	0.3676954	0.1343069	0.08993762	0.115708	0.1378253
1545.163	13	0.389699	0.339998	0.1330025	0.0923809	0.113018	0.1370081
1545.263	13	0.383092	0.3244006	0.1347615	0.09203408	0.110385	0.1348919
1545.363	13	0.384863	0.3262195	0.1383454	0.0919136	0.111172	0.1332129
1545.463	13	0.395225	0.3365271	0.1373348	0.0939481	0.110998	0.1323773
1545.563	13	0.39497	0.3456353	0.1345525	0.0974469	0.110295	0.1322195
1545.663	13	0.38377	0.3549431	0.1355468	0.1007275	0.109614	0.1311207
1545.763	13	0.389816	0.3586451	0.1364512	0.09977059	0.109051	0.13102
1545.863	13	0.391929	0.3385006	0.1357902	0.09754762	0.109114	0.1338892
1545.963	13	0.382366	0.3345504	0.1361372	0.09343075	0.110015	0.1339506
1546.063	13	0.38312	0.3387192	0.13558	0.09344424	0.108343	0.1327051
1546.163	13	0.391112	0.3425888	0.1353083	0.09549732	0.108135	0.1371848
1546.263	13	0.391364	0.3459074	0.1364341	0.09433883	0.108213	0.1340632
1546.363	13	0.38565	0.3452017	0.1352858	0.0916706	0.110299	0.1344859
1546.463	13	0.382331	0.342374	0.13618	0.09249148	0.107228	0.1355557
1546.563	13	0.380068	0.3376057	0.1382501	0.09249164	0.104778	0.1377185
1546.663	13	0.378356	0.3327441	0.1362045	0.0923743	0.106469	0.1370014

1546.763	13	0.376173	0.3293354	0.1340256	0.09317079	0.111215	0.1339069
1546.863	13	0.379258	0.340681	0.1317732	0.0929784	0.110686	0.1336672
1546.963	13	0.386149	0.3301036	0.1321722	0.09170972	0.115461	0.1342968
1547.063	13	0.391247	0.3387134	0.1308668	0.09444173	0.115205	0.1340397
1547.163	13	0.389748	0.3321902	0.1290795	0.09242569	0.11795	0.1367628
1547.263	13	0.373252	0.3122266	0.1314063	0.09291844	0.117864	0.1368374
1547.363	13	0.388884	0.3174268	0.1296215	0.09268144	0.117219	0.1387542
1547.463	13	0.380312	0.344577	0.129601	0.09110178	0.112752	0.1409729
1547.563	13	0.374973	0.3537697	0.1306497	0.08955563	0.114307	0.138797
1547.663	13	0.381641	0.3254385	0.1277293	0.08789618	0.116943	0.1385214
1547.763	13	0.416003	0.3286183	0.1280676	0.089427	0.119653	0.1385326
1547.863	13	0.399778	0.3340446	0.1280454	0.09008361	0.124414	0.1420099
1547.963	13	0.40908	0.3456116	0.1272858	0.09086003	0.122353	0.1410051
1548.063	13	0.415892	0.3460391	0.1266316	0.08970144	0.120818	0.1402466
1548.163	13	0.413627	0.3602682	0.1272474	0.09141596	0.120831	0.1464229
1548.263	13	0.408513	0.346935	0.1295178	0.09019806	0.123221	0.1484127
1548.363	13	0.412435	0.3385957	0.130817	0.09168559	0.121967	0.1468804
1548.463	13	0.410229	0.3374018	0.13261	0.0935874	0.122692	0.1454258
1548.563	13	0.394373	0.3413694	0.1337312	0.0977526	0.119858	0.1460071
1548.663	13	0.3994	0.343656	0.1360072	0.09765042	0.122966	0.1428289
1548.763	13	0.397014	0.3474667	0.1368541	0.09588947	0.123428	0.1428049
1548.863	13	0.399871	0.3470337	0.1385306	0.09769142	0.121055	0.1424625
1548.963	13	0.400946	0.3423683	0.1402444	0.09857529	0.115632	0.1431832
1549.063	13	0.411124	0.3346364	0.1396996	0.09785685	0.117511	0.1439537
1549.163	13	0.375109	0.328182	0.1415855	0.09893797	0.117382	0.14334
1549.263	13	0.372095	0.3307004	0.1457453	0.09817249	0.117303	0.1436096
1549.363	13	0.390234	0.3352652	0.1417747	0.09608606	0.116282	0.143578
1549.463	13	0.391288	0.3270075	0.1368761	0.09712058	0.115628	0.1418592
1549.563	13	0.388147	0.3376696	0.1352741	0.09791886	0.11519	0.1476094
1549.663	13	0.395361	0.320096	0.1340529	0.09776289	0.114377	0.1457113
1549.763	13	0.39122	0.307878	0.1338018	0.0983119	0.117765	0.1445424
1549.863	13	0.386056	0.3258774	0.1322332	0.09662665	0.117231	0.1422893
1549.963	13	0.383949	0.3580272	0.1301646	0.09706828	0.114333	0.1416515
1550.063	13	0.390493	0.3381447	0.1318945	0.1017506	0.111205	0.1410585
1550.163	13	0.396757	0.3254812	0.1329564	0.1048715	0.110522	0.1404535
1550.263	13	0.383	0.3283444	0.1330033	0.1063272	0.111864	0.1431508
1550.363	13	0.390072	0.3390503	0.134175	0.1041263	0.109797	0.1390119
1550.463	13	0.395677	0.3461499	0.1316199	0.1029748	0.111589	0.1359804
1550.563	13	0.386396	0.355152	0.1323013	0.0985016	0.110984	0.1319414
1550.663	13	0.38614	0.3628582	0.1316793	0.0985758	0.109731	0.1317004
1550.763	13	0.388583	0.341033	0.1296661	0.09985596	0.108484	0.135221
1550.863	13	0.390818	0.335426	0.1293679	0.09975033	0.109811	0.1367946
1550.963	13	0.38551	0.3377167	0.1292681	0.09939247	0.111318	0.1352803
1551.063	13	0.385452	0.3428508	0.1303485	0.1017487	0.108983	0.1347283
1551.163	13	0.382383	0.3453181	0.1299677	0.09941266	0.107541	0.1329812
1551.263	13	0.379278	0.3448244	0.1275952	0.09775591	0.106983	0.1334838
1551.363	13	0.378869	0.3429672	0.1278228	0.09853276	0.108621	0.1355186
1551.463	13	0.380838	0.3386539	0.1293602	0.09707232	0.109129	0.1333556
1551.563	13	0.387132	0.3317269	0.1302878	0.09680067	0.111394	0.1344784
1551.663	13	0.390513	0.3276637	0.1309038	0.0981239	0.112076	0.1358525
1551.763	13	0.388075	0.3386731	0.1304241	0.0966207	0.112198	0.1372108
1551.863	13	0.379273	0.3253491	0.1307061	0.09643266	0.11544	0.1378449
1551.963	13	0.393108	0.3332534	0.1298656	0.09373746	0.118084	0.1380365
1552.063	13	0.384217	0.3266087	0.1275245	0.09295992	0.11728	0.1348501
1552.163	13	0.377383	0.3074145	0.1277836	0.09484188	0.119485	0.1346561
1552.263	13	0.383554	0.3141628	0.1258603	0.0943097	0.120097	0.1366251
1552.363	13	0.431678	0.3374613	0.1255831	0.09490152	0.125148	0.1377002
1552.463	13	0.409296	0.3602613	0.1277824	0.09441102	0.129395	0.1364064
1552.563	13	0.399221	0.3307769	0.1296729	0.09302135	0.133138	0.1373398
1552.663	13	0.403094	0.3218433	0.1292403	0.09638662	0.1291	0.1361714
1552.763	13	0.406677	0.3304836	0.1309906	0.09666872	0.127426	0.1366049
1552.863	13	0.406008	0.3417506	0.1274171	0.09508213	0.124944	0.1397233
1552.963	13	0.412681	0.3452947	0.1270281	0.09572422	0.125022	0.143653
1553.063	13	0.414872	0.3626286	0.1256946	0.09478373	0.127162	0.1448629
1553.163	13	0.397158	0.3508745	0.1249703	0.09633139	0.125282	0.1437101
1553.263	13	0.401453	0.3409685	0.1282202	0.09656724	0.124496	0.1432378
1553.363	13	0.395567	0.3370209	0.126014	0.09559752	0.12514	0.1454801
1553.463	13	0.400477	0.3399487	0.1275851	0.09226391	0.122219	0.1465506
1553.563	13	0.401122	0.3410642	0.1305026	0.09494544	0.122927	0.1457705
1553.663	13	0.411886	0.3433056	0.131728	0.09466155	0.122086	0.1433534
1553.763	13	0.38565	0.3446958	0.1339106	0.0977321	0.121031	0.1448154
1553.863	13	0.371498	0.3417019	0.136444	0.09989314	0.120698	0.1425867
1553.963	13	0.386776	0.3366005	0.1363294	0.1000491	0.120493	0.1406962
1554.063	13	0.393049	0.3294967	0.1388875	0.1005495	0.122473	0.1427042
1554.163	13	0.391054	0.3336847	0.1374484	0.09957816	0.11742	0.1442922
1554.263	13	0.391888	0.3360275	0.138619	0.09829552	0.116453	0.1406665
1554.363	13	0.388284	0.3330206	0.1411818	0.09649733	0.117089	0.1407233
1554.463	13	0.389084	0.3377362	0.1424141	0.09369802	0.116188	0.1404798
1554.563	13	0.384012	0.3136744	0.1421507	0.09374067	0.115661	0.1389229
1554.663	13	0.38979	0.3036966	0.1381501	0.09502517	0.112412	0.1398692
1554.763	13	0.398262	0.3183318	0.1372721	0.09713765	0.110563	0.1418617
1554.863	13	0.382649	0.3559883	0.1344544	0.09739984	0.11205	0.1392072
1554.963	13	0.387239	0.3382182	0.1357845	0.09853246	0.111802	0.1401074
1555.063	13	0.394098	0.3219613	0.1344367	0.09780215	0.112266	0.1390865
1555.163	13	0.3871	0.3256805	0.1307682	0.0966564	0.111955	0.1408449
1555.263	13	0.38327	0.338896	0.1316293	0.09361227	0.109674	0.1441026
1555.363	13	0.387909	0.3515146	0.1322404	0.09834211	0.110085	0.1438298
1555.463	13	0.389515	0.3542148	0.1304024	0.0988474	0.108923	0.1437645
1555.563	13	0.385828	0.3615431	0.1288216	0.09782785	0.111906	0.1439477
1555.663	13	0.384654	0.3459891	0.1294632	0.09779272	0.115938	0.1431541
1555.763	13	0.383819	0.3379912	0.1313474	0.09489322	0.114144	0.1405132
1555.863	13	0.378843	0.3394935	0.129456	0.09371206	0.112853	0.1376363
1555.963	13	0.379829	0.3392171	0.129218	0.09114279	0.111932	0.1366716
1556.063	13	0.378949	0.3427486	0.1274569	0.09156719	0.112591	0.1386294
1556.163	13	0.386023	0.3429995	0.1273199	0.08923194	0.111114	0.1381582

1556.263	13	0.391179	0.3451971	0.127554	0.09119944	0.113842	0.1372007
1556.363	13	0.390608	0.3395977	0.1269721	0.09450891	0.114454	0.1356966
1556.463	13	0.376392	0.3317018	0.127599	0.0956313	0.118781	0.1340868
1556.563	13	0.387659	0.330568	0.129769	0.09502627	0.119916	0.1377046
1556.663	13	0.387696	0.3355426	0.1304989	0.09239399	0.119402	0.1387631
1556.763	13	0.393768	0.3278258	0.131238	0.09414281	0.119542	0.1367115
1556.863	13	0.395413	0.3328974	0.1293566	0.09327748	0.120681	0.1350839
1556.963	13	0.407528	0.3232647	0.129662	0.09280087	0.120395	0.1346184
1557.063	13	0.397261	0.3052884	0.1307369	0.09146744	0.121872	0.1344594
1557.163	13	0.395391	0.3142123	0.1297799	0.08878947	0.128572	0.1351766
1557.263	13	0.407432	0.341518	0.1278014	0.08877076	0.127538	0.1342249
1557.363	13	0.414466	0.3569835	0.1278392	0.08995166	0.126771	0.1361889
1557.463	13	0.412104	0.326715	0.1257804	0.09075931	0.129252	0.1375145
1557.563	13	0.421299	0.3196326	0.1280133	0.09246707	0.128026	0.1404365
1557.663	13	0.416738	0.3301432	0.1288306	0.09371772	0.12712	0.1387938
1557.763	13	0.397118	0.3488134	0.1278231	0.09552742	0.126486	0.1372148
1557.863	13	0.402544	0.3499515	0.1275627	0.09497926	0.129509	0.1388352
1557.963	13	0.400829	0.3608131	0.1279642	0.09485549	0.130471	0.1392323
1558.063	13	0.40102	0.351704	0.1235407	0.09716932	0.12873	0.1397193
1558.163	13	0.389974	0.3380927	0.1263404	0.09813376	0.127512	0.1425384
1558.263	13	0.369113	0.3372985	0.1254267	0.09969841	0.124654	0.1452692
1558.363	13	0.389455	0.3392603	0.1288992	0.09893127	0.121819	0.1462328
1558.463	13	0.392403	0.3470331	0.131077	0.09592185	0.118793	0.1424457
1558.563	13	0.387943	0.3522048	0.1295035	0.09572168	0.120081	0.1401598
1558.663	13	0.389279	0.3568595	0.1277055	0.09657488	0.122487	0.1406502
1558.763	13	0.383997	0.3453763	0.1296253	0.1005157	0.121675	0.1392838
1558.902	13.5	0.389008	0.3373684	0.1334601	0.09828039	0.119891	0.140499
1559.002	13.5	0.388151	0.3413321	0.134903	0.09975215	0.119208	0.1413535
1559.102	13.5	0.39693	0.3339013	0.137517	0.09903168	0.118023	0.1412601
1559.202	13.5	0.395594	0.3343302	0.1384205	0.09656911	0.116496	0.1402062
1559.302	13.5	0.381735	0.3175028	0.1392297	0.09383154	0.114953	0.1425347
1559.402	13.5	0.396264	0.3085266	0.1369774	0.09459204	0.112799	0.1448145
1559.502	13.5	0.396101	0.3276663	0.1369604	0.09381489	0.113646	0.1411655
1559.602	13.5	0.382919	0.3646192	0.1375482	0.09202757	0.114589	0.1390358
1559.702	13.5	0.388758	0.3395647	0.1393134	0.09306926	0.112145	0.1390155
1559.802	13.5	0.388868	0.3221627	0.139739	0.0936622	0.112528	0.1391135
1559.902	13.5	0.392828	0.3268813	0.1366411	0.09221952	0.112671	0.134695
1560.002	13.5	0.389299	0.3421164	0.1352356	0.09176258	0.110142	0.1368886
1560.102	13.5	0.39192	0.3498353	0.1301627	0.09241156	0.108221	0.1368071
1560.202	13.5	0.383725	0.3583266	0.1270273	0.09238908	0.110405	0.138421
1560.302	13.5	0.378573	0.3581791	0.1272357	0.09135143	0.113895	0.1395913
1560.402	13.5	0.381923	0.3423375	0.1290111	0.09281899	0.112844	0.1389551
1560.502	13.5	0.37928	0.338897	0.1279963	0.09041002	0.112542	0.1419431
1560.602	13.5	0.390532	0.3500551	0.1263904	0.08605658	0.114515	0.1426608
1560.702	13.5	0.392679	0.3525344	0.1242413	0.08848664	0.114036	0.1387733
1560.802	13.5	0.380811	0.3544651	0.1276501	0.08750286	0.109895	0.1382882
1560.902	13.5	0.38857	0.3534544	0.1255866	0.08909585	0.113277	0.1371365
1561.002	13.5	0.388208	0.3587041	0.126356	0.09091464	0.115194	0.1351022
1561.102	13.5	0.372083	0.3468204	0.1248119	0.09476072	0.118438	0.136942
1561.202	13.5	0.377364	0.3399338	0.1261673	0.09530769	0.1189	0.1351005
1561.302	13.5	0.3957	0.3400897	0.1260265	0.09457928	0.121747	0.1350807
1561.402	13.5	0.387839	0.3486734	0.1285705	0.09069192	0.124763	0.1357798
1561.502	13.5	0.393864	0.3360446	0.1311314	0.0894649	0.127747	0.1338408
1561.602	13.5	0.40802	0.3386929	0.1302657	0.09021205	0.128729	0.133737
1561.702	13.5	0.412036	0.319391	0.1323303	0.08891849	0.127883	0.1315065
1561.802	13.5	0.413922	0.3104636	0.1342477	0.08757831	0.128389	0.1320375
1561.902	13.5	0.425824	0.3301377	0.1328785	0.08903801	0.12788	0.1337439
1562.002	13.5	0.409992	0.3617071	0.1313484	0.08714202	0.125724	0.1332066
1562.102	13.5	0.399642	0.3431152	0.1309348	0.08815306	0.126087	0.1336898
1562.202	13.5	0.40671	0.3255198	0.1298336	0.09107062	0.126272	0.1328012
1562.302	13.5	0.396345	0.3268744	0.1302327	0.09150457	0.128001	0.1352441
1562.402	13.5	0.400765	0.3455441	0.1291977	0.0889516	0.127185	0.1340553
1562.502	13.5	0.424274	0.3459172	0.1288029	0.09250727	0.12781	0.1311481
1562.602	13.5	0.43607	0.3568968	0.1296169	0.09539969	0.127286	0.1328215
1562.702	13.5	0.391453	0.3539216	0.1291745	0.09588297	0.125	0.1353236
1562.802	13.5	0.398184	0.3453032	0.1272108	0.09417201	0.124135	0.1361557
1562.902	13.5	0.407066	0.3367757	0.1269988	0.0909356	0.12154	0.1373623
1563.002	13.5	0.392373	0.3403255	0.1259248	0.09344981	0.12092	0.1366733
1563.102	13.5	0.396117	0.3462842	0.1254734	0.09404693	0.120384	0.1378463
1563.202	13.5	0.386811	0.3501856	0.1244767	0.09528298	0.120088	0.1363902
1563.302	13.5	0.398228	0.3527622	0.1257808	0.09684511	0.1196	0.1363283
1563.402	13.5	0.3866	0.3456356	0.1244925	0.09661496	0.120684	0.1400689
1563.502	13.5	0.389502	0.3389087	0.1244901	0.1005451	0.119501	0.144192
1563.602	13.5	0.400668	0.3342558	0.125922	0.09918506	0.117177	0.1469022
1563.702	13.5	0.38836	0.3374717	0.1289237	0.09707545	0.116913	0.1462109
1563.802	13.5	0.401969	0.3349456	0.1284916	0.09897541	0.116844	0.143452
1563.902	13.5	0.401763	0.3255459	0.1293675	0.09959188	0.113069	0.1414034
1564.002	13.5	0.392326	0.3306951	0.1309129	0.09400783	0.110484	0.1408401
1564.102	13.5	0.396843	0.3160054	0.1324908	0.09228951	0.108629	0.1411713
1564.202	13.5	0.407149	0.3077428	0.1332141	0.09261711	0.110133	0.143086
1564.302	13.5	0.408471	0.3245437	0.1334909	0.09317854	0.111143	0.1458894
1564.402	13.5	0.409562	0.3567274	0.1352252	0.09166725	0.113314	0.1435164
1564.502	13.5	0.393857	0.3406795	0.1369653	0.09175193	0.114229	0.1421415
1564.602	13.5	0.392905	0.3265568	0.1386479	0.08891339	0.115164	0.141764
1564.702	13.5	0.379117	0.3310609	0.1350115	0.08900169	0.113581	0.1394339
1564.802	13.5	0.380055	0.3441113	0.134362	0.08920756	0.114522	0.138771
1564.902	13.5	0.385429	0.3492922	0.1344596	0.09020505	0.113997	0.1376476
1565.002	13.5	0.391679	0.3549504	0.1342244	0.08992746	0.11122	0.1383604
1565.102	13.5	0.395191	0.3519063	0.1340591	0.08827372	0.109807	0.1329158
1565.202	13.5	0.382749	0.3437436	0.1315893	0.09035009	0.111451	0.1333371
1565.302	13.5	0.383688	0.3367167	0.1322359	0.09117803	0.113131	0.1358453
1565.402	13.5	0.395398	0.3391784	0.1332676	0.09138741	0.109279	0.1401038
1565.502	13.5	0.383327	0.3408618	0.1313281	0.09093487	0.111865	0.1429042
1565.602	13.5	0.381116	0.3472162	0.131549	0.09360231	0.114328	0.1418405
1565.702	13.5	0.384744	0.3518727	0.1316833	0.09250737	0.117737	0.1413353

1565.802	13.5	0.39157	0.3463473	0.1338495	0.09358215	0.12162	0.1402948
1565.902	13.5	0.381238	0.3373208	0.1328846	0.09384884	0.122856	0.1394726
1566.002	13.5	0.401255	0.3354911	0.1306341	0.09576818	0.122536	0.1360237
1566.102	13.5	0.409164	0.3387692	0.130658	0.09645929	0.122614	0.1311872
1566.202	13.5	0.407575	0.3324153	0.1303723	0.09682184	0.124148	0.1322244
1566.302	13.5	0.41012	0.3238335	0.1315001	0.09360002	0.123696	0.1317997
1566.402	13.5	0.416402	0.3255772	0.1310918	0.09440237	0.123699	0.129792
1566.502	13.5	0.401234	0.3131976	0.1310739	0.09028969	0.124914	0.1318055
1566.602	13.5	0.405104	0.3066711	0.1307705	0.09271204	0.123352	0.13345
1566.702	13.5	0.409996	0.3201403	0.130756	0.09294352	0.123047	0.1320049
1566.802	13.5	0.41513	0.3595748	0.1304323	0.09203361	0.122515	0.1323902
1566.902	13.5	0.417514	0.3399294	0.132976	0.09436929	0.119527	0.1316323
1567.002	13.5	0.415059	0.3231753	0.1349399	0.09437519	0.117725	0.1348301
1567.102	13.5	0.391858	0.3279963	0.1309105	0.09180541	0.12082	0.1353671
1567.202	13.5	0.366842	0.3417872	0.1287692	0.09449103	0.122585	0.1340412
1567.302	13.5	0.386971	0.3464487	0.1286076	0.09009562	0.122197	0.1354058
1567.402	13.5	0.394865	0.3548959	0.1266022	0.09309893	0.120428	0.1349961
1567.502	13.5	0.390221	0.350105	0.1280934	0.09618334	0.121759	0.1364157
1567.602	13.5	0.388391	0.3405563	0.127407	0.09631953	0.118251	0.1374416
1567.702	13.5	0.386005	0.3355336	0.1302666	0.0982144	0.116947	0.1353395
1567.802	13.5	0.385534	0.3385613	0.1294248	0.09753222	0.117365	0.1365306
1567.902	13.5	0.382269	0.3419538	0.1290474	0.09540155	0.116266	0.1375704
1568.002	13.5	0.391886	0.3453273	0.1263141	0.09618603	0.118213	0.1371848
1568.102	13.5	0.388899	0.3450578	0.1228499	0.09847698	0.115891	0.1387245
1568.202	13.5	0.378324	0.3465866	0.1252686	0.09746583	0.114784	0.1398094
1568.302	13.5	0.392727	0.3388202	0.1263995	0.09517882	0.115588	0.1357715
1568.402	13.5	0.389412	0.3363367	0.1279226	0.09413403	0.113394	0.1342973
1568.502	13.5	0.380617	0.3366132	0.1288736	0.09176131	0.111123	0.1341494
1568.602	13.5	0.386868	0.3360898	0.1290477	0.0916826	0.111222	0.1366567
1568.702	13.5	0.386459	0.3219599	0.1287934	0.09272014	0.110816	0.1397023
1568.802	13.5	0.389644	0.3225374	0.1258369	0.09226224	0.109711	0.1434443
1568.902	13.5	0.384619	0.3143616	0.1283237	0.09148715	0.107977	0.1450851
1569.002	13.5	0.391186	0.305008	0.13069	0.09091964	0.108453	0.1455604
1569.102	13.5	0.382022	0.3184991	0.1326789	0.0894903	0.109426	0.146462
1569.202	13.5	0.376846	0.3596289	0.1340041	0.08881584	0.108682	0.1454122
1569.302	13.5	0.374866	0.3378574	0.1353846	0.08740006	0.10939	0.1453118
1569.402	13.5	0.382574	0.3228377	0.1384653	0.08691256	0.10834	0.1456428
1569.502	13.5	0.390133	0.3298953	0.1395343	0.08809879	0.10884	0.145978
1569.602	13.5	0.389519	0.3433716	0.1386274	0.08840475	0.107997	0.1429064
1569.702	13.5	0.381324	0.3485357	0.1372577	0.08695056	0.107538	0.1417943
1569.802	13.5	0.377484	0.3537511	0.135353	0.08963651	0.107431	0.1418589
1569.902	13.5	0.369683	0.3490959	0.1361143	0.08677223	0.107061	0.1414776
1570.002	13.5	0.369656	0.3393915	0.1369795	0.08813238	0.106993	0.1398501
1570.102	13.5	0.376734	0.3348184	0.136707	0.08757326	0.107907	0.1386353
1570.202	13.5	0.390509	0.3419256	0.1338031	0.08896886	0.109446	0.1415845
1570.302	13.5	0.381198	0.3479732	0.1342953	0.08872604	0.108554	0.139982
1570.402	13.5	0.390261	0.3376931	0.1355569	0.0882181	0.112279	0.1394606
1570.502	13.5	0.400906	0.3391004	0.1318747	0.08439013	0.117319	0.1428149
1570.602	13.5	0.401046	0.3416172	0.1322262	0.08654466	0.118118	0.1416084
1570.702	13.5	0.406762	0.3357578	0.1300779	0.08382072	0.117664	0.1414649
1570.802	13.5	0.425277	0.3312687	0.1348358	0.08458437	0.120254	0.1406575
1570.902	13.5	0.429245	0.3316935	0.1353758	0.0878693	0.123626	0.1416899
1571.002	13.5	0.413194	0.3288148	0.1343527	0.08944945	0.124834	0.1442749
1571.102	13.5	0.410722	0.3290657	0.1354533	0.08934705	0.125243	0.1437564
1571.202	13.5	0.398676	0.3217943	0.1335563	0.08845871	0.123003	0.1433035
1571.302	13.5	0.404785	0.3120926	0.1336518	0.09058188	0.124419	0.1406866
1571.402	13.5	0.414906	0.3102324	0.1323615	0.08865511	0.124171	0.1400745
1571.502	13.5	0.408192	0.3161852	0.1319253	0.08373001	0.125056	0.1389225
1571.602	13.5	0.379007	0.3453909	0.1320543	0.08839627	0.12408	0.1392175
1571.702	13.5	0.375658	0.3382523	0.1330134	0.08736829	0.123899	0.1397201
1571.802	13.5	0.389672	0.3369033	0.1342284	0.08644601	0.123343	0.13897
1571.902	13.5	0.387109	0.3362668	0.1356966	0.08764661	0.122043	0.1398563
1572.002	13.5	0.38844	0.3446635	0.1354978	0.0926668	0.121017	0.1383542
1572.102	13.5	0.383181	0.3516621	0.1334801	0.09427387	0.120925	0.1384008
1572.202	13.5	0.389512	0.3584938	0.131384	0.09471541	0.12187	0.1382326
1572.302	13.5	0.384826	0.3526513	0.1288178	0.09634562	0.120897	0.1394741
1572.402	13.5	0.382758	0.3432535	0.1290852	0.09771129	0.122377	0.1367401
1572.502	13.5	0.393702	0.3429191	0.1282284	0.1023501	0.12042	0.1377708
1572.602	13.5	0.37948	0.3468705	0.127801	0.09909616	0.120456	0.1395985
1572.702	13.5	0.382212	0.3474876	0.1297779	0.0979963	0.119928	0.1396094
1572.802	13.5	0.392715	0.353933	0.1274821	0.09362329	0.119359	0.1407245
1572.902	13.5	0.38205	0.3550869	0.1286073	0.08961429	0.118591	0.1395625
1573.002	13.5	0.382871	0.3550434	0.1283567	0.08861674	0.11459	0.138042
1573.102	13.5	0.389134	0.3437401	0.1261321	0.0884962	0.113831	0.1378961
1573.202	13.5	0.389914	0.3428856	0.1278297	0.0869275	0.111802	0.1358663
1573.302	13.5	0.392907	0.3371363	0.1296299	0.08610599	0.111507	0.1378571
1573.402	13.5	0.390108	0.3335461	0.129618	0.08633815	0.111602	0.1408804
1573.502	13.5	0.393217	0.3277215	0.1280516	0.08596729	0.109256	0.1406302
1573.602	13.5	0.374233	0.3254399	0.129153	0.08766527	0.109874	0.140014
1573.702	13.5	0.373324	0.3126214	0.1279471	0.08705235	0.106803	0.1404189
1573.802	13.5	0.380982	0.3101114	0.1286016	0.08908857	0.106636	0.1423166
1573.902	13.5	0.391567	0.3195556	0.135107	0.08856752	0.10912	0.1450074
1574.002	13.5	0.389278	0.3494981	0.1322521	0.08836729	0.108726	0.1468477
1574.102	13.5	0.382197	0.3350616	0.1335189	0.08803504	0.1098	0.1474126
1574.202	13.5	0.382442	0.3323675	0.1322116	0.08791037	0.109521	0.1458879
1574.302	13.5	0.370944	0.3324724	0.1346593	0.08749367	0.108356	0.1464496
1574.402	13.5	0.375606	0.3439066	0.1366883	0.08916809	0.108711	0.1462528
1574.502	13.5	0.377365	0.3501307	0.1372673	0.09149893	0.109652	0.1483053
1574.602	13.5	0.393562	0.3614768	0.1383728	0.0906022	0.107867	0.1515673
1574.702	13.5	0.3872	0.3536257	0.1373208	0.09017212	0.113448	0.1497437
1574.802	13.5	0.38217	0.3393579	0.1366483	0.08904469	0.114472	0.1461827
1574.902	13.5	0.400992	0.3380369	0.1370038	0.08873399	0.113485	0.1458517
1575.002	13.5	0.407155	0.3430789	0.136042	0.08916198	0.116169	0.1446759
1575.102	13.5	0.400553	0.343037	0.1357882	0.08871016	0.118513	0.1430867
1575.202	13.5	0.408997	0.346225	0.1353081	0.08854308	0.11575	0.1414293

1575.302	13.5	0.414911	0.3475053	0.1369506	0.08991038	0.117642	0.1426046
1575.402	13.5	0.398307	0.3426396	0.1381918	0.0901026	0.119649	0.1414685
1575.502	13.5	0.410671	0.3359127	0.1376256	0.09361664	0.120039	0.1384919
1575.602	13.5	0.422392	0.3344961	0.1364931	0.09422331	0.122317	0.1397861
1575.702	13.5	0.416129	0.334326	0.1345451	0.09202854	0.127362	0.1431549
1575.802	13.5	0.424034	0.3328008	0.1357429	0.09135185	0.124453	0.1425059
1575.902	13.5	0.433383	0.329792	0.1338644	0.09217504	0.125034	0.1388439
1576.002	13.5	0.398942	0.3253575	0.1348032	0.09343343	0.123026	0.1369228
1576.102	13.5	0.373118	0.3116307	0.134489	0.09486813	0.121508	0.1404274
1576.202	13.5	0.389701	0.3080655	0.136177	0.09204086	0.120094	0.1431664
1576.302	13.5	0.393117	0.3249153	0.13426	0.09435869	0.124193	0.1418012
1576.402	13.5	0.38989	0.3507557	0.1326516	0.09663899	0.126981	0.1405102
1576.502	13.5	0.389746	0.3310655	0.1309787	0.0964471	0.125273	0.1374926
1576.602	13.5	0.38859	0.3268485	0.1285796	0.09682951	0.123367	0.1386483
1576.702	13.5	0.386314	0.3357506	0.1305475	0.09652639	0.124423	0.1372192
1576.802	13.5	0.384856	0.3456373	0.1310267	0.09446778	0.124846	0.1376064
1576.902	13.5	0.395596	0.3452902	0.1307128	0.09686314	0.122664	0.1360899
1577.002	13.5	0.389033	0.355748	0.1308352	0.1004295	0.122402	0.1377321
1577.102	13.5	0.379543	0.3548152	0.1302101	0.1005013	0.12068	0.1385589
1577.202	13.5	0.39348	0.3390381	0.1306685	0.0981729	0.11798	0.1392976
1577.302	13.5	0.389351	0.3346591	0.1304066	0.09792247	0.117619	0.1370214
1577.402	13.5	0.381912	0.3345323	0.1273459	0.09439847	0.118178	0.1363007
1577.502	13.5	0.384542	0.3383821	0.1285759	0.09414604	0.118018	0.1385589
1577.602	13.5	0.384203	0.337536	0.1291737	0.09643748	0.117351	0.1385525
1577.702	13.5	0.386578	0.3420554	0.1292749	0.09214025	0.116931	0.1381511
1577.802	13.5	0.387341	0.339947	0.1273867	0.0913377	0.11471	0.1399285
1577.902	13.5	0.391423	0.3374197	0.125654	0.08968514	0.113743	0.1388843
1578.002	13.5	0.379079	0.3369414	0.1225476	0.0862035	0.114026	0.1398948
1578.102	13.5	0.373547	0.3367258	0.1239973	0.08743788	0.113996	0.1381511
1578.202	13.5	0.375544	0.3316259	0.1235039	0.08822288	0.112963	0.1345726
1578.302	13.5	0.381327	0.3283381	0.1250253	0.0916677	0.110749	0.1350001
1578.402	13.5	0.390052	0.3246266	0.1279208	0.0899131	0.109412	0.1351619
1578.502	13.5	0.384926	0.3107317	0.1266384	0.08807816	0.112222	0.1338696
1578.602	13.5	0.379004	0.3084732	0.127142	0.08880369	0.115102	0.1331313
1578.702	13.5	0.430512	0.3151794	0.1284265	0.08595119	0.114475	0.1341027
1578.802	13.5	0.429705	0.3425597	0.129316	0.08510566	0.113297	0.1340754
1578.902	13.5	0.425619	0.3306231	0.1325858	0.08729865	0.111216	0.1348535
1579.002	13.5	0.41387	0.3245631	0.1321852	0.0854151	0.109811	0.1371364
1579.102	13.5	0.468281	0.3278691	0.1331557	0.08549299	0.110049	0.1390305
1579.202	13.5	0.445027	0.3451952	0.1356502	0.08444069	0.107847	0.1427585
1579.302	13.5	0.44698	0.349135	0.1336495	0.08442023	0.109721	0.1448977
1579.402	13.5	0.457889	0.3583558	0.1329378	0.0853246	0.110581	0.1439604
1579.502	13.5	0.451394	0.356095	0.1329976	0.08733266	0.108816	0.1447672
1579.602	13.5	0.443751	0.3412328	0.1334607	0.08580496	0.114722	0.1426474
1579.702	13.5	0.438245	0.3393865	0.1321338	0.08538178	0.117993	0.1442925
1579.802	13.5	0.467434	0.3387561	0.1338224	0.08665487	0.118086	0.1453395
1579.902	13.5	0.457754	0.3402481	0.1346075	0.08684742	0.121199	0.149564
1580.002	13.5	0.443179	0.3425424	0.1348027	0.08652842	0.122468	0.1449171
1580.102	13.5	0.417509	0.3458064	0.132323	0.08580342	0.125076	0.138804
1580.202	13.5	0.413486	0.3482519	0.1318639	0.08635881	0.128154	0.1370855
1580.302	13.5	0.417971	0.336138	0.1324654	0.08937944	0.129157	0.1377092
1580.402	13.5	0.393673	0.3324952	0.1342682	0.08998146	0.127171	0.1405592
1580.502	13.5	0.366325	0.3390912	0.1340243	0.09162319	0.126277	0.1378084
1580.602	13.5	0.382085	0.3351526	0.1334488	0.0937814	0.121387	0.1391295
1580.702	13.5	0.388978	0.3286154	0.1325463	0.09190888	0.119372	0.141166
1580.802	13.5	0.385143	0.3253784	0.1332993	0.09351452	0.119601	0.1403474
1580.902	13.5	0.388574	0.3158914	0.1317971	0.09457052	0.12108	0.1383081
1581.002	13.5	0.385135	0.3103707	0.1321613	0.09639458	0.120437	0.1381266
1581.102	13.5	0.391259	0.3194061	0.1317222	0.09666734	0.121071	0.1402914
1581.202	13.5	0.38489	0.346356	0.1321741	0.09627971	0.118105	0.1426659
1581.302	13.5	0.392628	0.3309066	0.1321385	0.09712544	0.118971	0.1413033
1581.402	13.5	0.402167	0.3262347	0.1332324	0.09673971	0.119665	0.1385473
1581.502	13.5	0.378198	0.3287768	0.131788	0.1001075	0.119194	0.1369119
1581.602	13.5	0.387861	0.3362327	0.1294771	0.1005554	0.118157	0.1372078
1581.702	13.5	0.403356	0.3442834	0.1297174	0.09942712	0.118269	0.1351726
1581.802	13.5	0.392872	0.3586238	0.1303967	0.09522169	0.119073	0.1336303
1581.902	13.5	0.40293	0.3523239	0.1305479	0.09360548	0.119376	0.1346608
1582.002	13.5	0.395474	0.340191	0.1309084	0.09281215	0.116832	0.1329127
1582.102	13.5	0.403466	0.3360312	0.1286222	0.09195585	0.116115	0.1331748
1582.202	13.5	0.399876	0.3338118	0.126824	0.09088594	0.115202	0.1327835
1582.302	13.5	0.395716	0.3436383	0.1247618	0.09163285	0.115824	0.132889
1582.402	13.5	0.400217	0.3535738	0.1248355	0.09124429	0.115114	0.1317921
1582.502	13.5	0.379687	0.3503133	0.1250977	0.08997734	0.114431	0.131219
1582.602	13.5	0.37674	0.3400678	0.1251145	0.09016503	0.11417	0.1297399
1582.702	13.5	0.386499	0.3365351	0.1258478	0.08897667	0.112311	0.1316982
1582.802	13.5	0.385189	0.3330484	0.1247724	0.0904036	0.112493	0.1295231
1582.902	13.5	0.389061	0.3345551	0.1247445	0.0936949	0.111237	0.1321198
1583.002	13.5	0.386517	0.3411061	0.1246593	0.09248691	0.108594	0.1312419
1583.102	13.5	0.389298	0.3288751	0.125329	0.09078737	0.110521	0.1330051
1583.202	13.5	0.384938	0.329486	0.1251943	0.09065281	0.110494	0.1359185
1583.302	13.5	0.381593	0.3214022	0.1271967	0.09105736	0.112525	0.1376659
1583.402	13.5	0.374081	0.3184321	0.1287874	0.089412	0.114794	0.1364422
1583.502	13.5	0.381389	0.3285891	0.127871	0.08798964	0.11216	0.134671
1583.602	13.5	0.384989	0.3562072	0.1313851	0.08806372	0.11235	0.134279
1583.702	13.5	0.383536	0.3355634	0.1336392	0.08665796	0.112967	0.1350009
1583.802	13.5	0.390378	0.3241216	0.1302039	0.08721065	0.111408	0.1368974
1583.902	13.5	0.40472	0.3343527	0.1312762	0.0887078	0.111521	0.1375886
1584.002	13.5	0.405034	0.3441255	0.1321959	0.0897373	0.11535	0.138916
1584.102	13.5	0.421655	0.3452903	0.1329114	0.08958519	0.116379	0.1430638
1584.202	13.5	0.423333	0.3584217	0.1355512	0.08941887	0.116025	0.1415926
1584.302	13.5	0.406742	0.3546301	0.1373647	0.08900476	0.121529	0.1418746
1584.402	13.5	0.410535	0.3457603	0.1399812	0.09159847	0.117469	0.1435501
1584.502	13.5	0.397927	0.3390005	0.1376437	0.09276217	0.117943	0.143661
1584.602	13.5	0.394038	0.339713	0.1365731	0.08971552	0.120598	0.1439644
1584.702	13.5	0.412797	0.3463571	0.1360595	0.09211818	0.126548	0.1447661

1584.802	13.5	0.411921	0.35213	0.133231	0.09277131	0.124617	0.1433517
1584.902	13.5	0.382005	0.3501727	0.1321493	0.09134475	0.124736	0.1415992
1585.002	13.5	0.378427	0.3442697	0.1307261	0.09107176	0.122775	0.139921
1585.102	13.5	0.385136	0.3407848	0.1298999	0.08898477	0.123385	0.1380618
1585.202	13.5	0.388161	0.3386702	0.1284352	0.08917185	0.123283	0.1347504
1585.302	13.5	0.389589	0.3374673	0.1289798	0.09380194	0.122618	0.1341556
1585.402	13.5	0.38515	0.345576	0.1308476	0.0939762	0.120778	0.135671
1585.502	13.5	0.388476	0.3325878	0.1287617	0.0956741	0.119785	0.1349114
1585.602	13.5	0.387544	0.3342357	0.1296828	0.09536633	0.12368	0.1374006
1585.702	13.5	0.380318	0.3274852	0.1275502	0.0966595	0.125609	0.1413925
1585.802	13.5	0.391068	0.3208794	0.1305169	0.09760962	0.124967	0.1413018
1585.902	13.5	0.386518	0.3334275	0.1305355	0.09876606	0.125898	0.1383489
1586.002	13.5	0.378092	0.3607035	0.1299607	0.09925652	0.126026	0.1361995
1586.102	13.5	0.39178	0.3434025	0.1288962	0.09644288	0.122053	0.1365601
1586.202	13.5	0.392685	0.3331145	0.1323251	0.09137408	0.120942	0.1377197
1586.302	13.5	0.39263	0.3343291	0.1333202	0.09027434	0.122827	0.1376389
1586.402	13.5	0.392288	0.3429787	0.1337474	0.09140927	0.120866	0.1378079
1586.502	13.5	0.39807	0.3570145	0.129605	0.08979607	0.119743	0.1370961
1586.602	13.5	0.406057	0.3640593	0.1271834	0.09004703	0.11826	0.1362011
1586.702	13.5	0.386535	0.3585224	0.1289005	0.08922129	0.117241	0.1337453
1586.802	13.5	0.392029	0.3418881	0.1307335	0.08610846	0.117077	0.1359136
1586.902	13.5	0.382979	0.3398902	0.1314122	0.08572523	0.116807	0.1358626
1587.002	13.5	0.378682	0.3462471	0.1305229	0.086087	0.115798	0.1368943
1587.102	13.5	0.378146	0.3513888	0.1282869	0.08433264	0.113676	0.1362171
1587.202	13.5	0.386585	0.3520625	0.1299358	0.08653446	0.114583	0.1367233
1587.302	13.5	0.391183	0.355474	0.1296426	0.08744138	0.113132	0.1367903
1587.402	13.5	0.386437	0.351436	0.1264181	0.08974122	0.111112	0.1376335
1587.502	13.5	0.381974	0.3417657	0.1239036	0.08997178	0.111132	0.1380705
1587.602	13.5	0.399468	0.3385295	0.1248824	0.09019895	0.108615	0.1365107
1587.702	13.5	0.395326	0.3372434	0.1278508	0.09010316	0.109234	0.1355954
1587.802	13.5	0.389205	0.34245	0.1281981	0.08969607	0.108411	0.1365487
1587.902	13.5	0.389199	0.3361045	0.1292582	0.08829772	0.107803	0.138238
1588.002	13.5	0.397713	0.3371663	0.1257295	0.08649787	0.107792	0.1351623
1588.102	13.5	0.38294	0.3272358	0.1266417	0.0843819	0.104472	0.1356209
1588.202	13.5	0.390561	0.3252117	0.128983	0.08217883	0.105518	0.1371759
1588.302	13.5	0.391074	0.3337264	0.1300182	0.08320393	0.106532	0.1402009
1588.402	13.5	0.392607	0.3655741	0.1293091	0.08570166	0.107677	0.1397013
1588.502	13.5	0.3966	0.3348934	0.1284847	0.08376005	0.106015	0.1402842
1588.602	13.5	0.408864	0.3272056	0.1274431	0.08342223	0.106955	0.1419225
1588.702	13.5	0.40547	0.3292063	0.1266949	0.08458433	0.105611	0.1395554
1588.802	13.5	0.405808	0.3394669	0.1306512	0.08552819	0.110614	0.1407227
1588.902	13.5	0.392812	0.3453429	0.1355153	0.08554158	0.113038	0.1425178
1589.002	13.5	0.388448	0.3629209	0.1362436	0.08174495	0.113607	0.1410668
1589.102	13.5	0.390961	0.3557635	0.1373311	0.08155558	0.117953	0.1444355
1589.202	13.5	0.400429	0.3431111	0.1355745	0.08454716	0.117802	0.1445286
1589.302	13.5	0.380292	0.3434083	0.1357052	0.08579331	0.119142	0.1458385
1589.402	13.5	0.361891	0.3463913	0.1381854	0.08769827	0.120644	0.1459937
1589.502	13.5	0.379412	0.3538833	0.1389897	0.088726	0.12441	0.1469102
1589.602	13.5	0.38472	0.3529668	0.1397592	0.08895682	0.12414	0.1469257
1589.702	13.5	0.386939	0.3554843	0.1332035	0.09071959	0.122513	0.1477591
1589.802	13.5	0.388292	0.3515506	0.130398	0.09047043	0.11936	0.1445639
1589.902	13.5	0.383	0.3526666	0.1320869	0.09429599	0.118835	0.1428805
1590.002	13.5	0.384675	0.3444428	0.1292963	0.09703547	0.122819	0.1433287
1590.102	13.5	0.378823	0.3423875	0.1298318	0.09755798	0.122982	0.1435225
1590.202	13.5	0.384494	0.341125	0.1281817	0.09901171	0.119894	0.1409508
1590.302	13.5	0.394083	0.3318853	0.1304708	0.09939732	0.121359	0.1387246
1590.402	13.5	0.379365	0.3322381	0.1324062	0.102182	0.121262	0.1369694
1590.502	13.5	0.392284	0.3174391	0.1305789	0.1011087	0.121717	0.1366675
1590.602	13.5	0.398131	0.3172327	0.1317226	0.09756275	0.1214	0.1373191
1590.702	13.5	0.393372	0.3349504	0.128845	0.09456486	0.118337	0.142478
1590.802	13.5	0.398823	0.3670249	0.1303454	0.09428421	0.11637	0.1417929
1590.902	13.5	0.402748	0.3373864	0.131195	0.09496196	0.116777	0.1387786
1591.002	13.5	0.406132	0.3229679	0.1310735	0.09328336	0.11519	0.1357079
1591.102	13.5	0.39433	0.3274337	0.1307472	0.0921149	0.11486	0.1348652
1591.202	13.5	0.388986	0.34084	0.1318079	0.09038596	0.11292	0.1354908
1591.302	13.5	0.385857	0.3433731	0.1340633	0.08867159	0.112887	0.1367476
1591.402	13.5	0.385097	0.3640249	0.1344732	0.08851091	0.111329	0.13585
1591.502	13.5	0.376908	0.3576374	0.1358845	0.09022117	0.109561	0.1336
1591.602	13.5	0.387552	0.340138	0.1339562	0.08895721	0.109883	0.1345463
1591.702	13.5	0.387272	0.3401172	0.1334479	0.08837514	0.112073	0.1331964
1591.802	13.5	0.388582	0.3410564	0.1340211	0.08892329	0.111912	0.1322106
1591.902	13.5	0.379829	0.344913	0.1307693	0.08920059	0.109199	0.1339109
1592.002	13.5	0.375031	0.3456518	0.1307836	0.08973206	0.107792	0.135969
1592.102	13.5	0.369015	0.3465398	0.1302717	0.09040653	0.107746	0.1360317
1592.202	13.5	0.363299	0.3464311	0.1281824	0.09138649	0.108955	0.1356806
1592.302	13.5	0.378205	0.3379391	0.1284589	0.08974685	0.108936	0.1351072
1592.402	13.5	0.391074	0.3407803	0.1267048	0.08778583	0.107989	0.1356905
1592.502	13.5	0.380952	0.339654	0.1257811	0.08659096	0.106441	0.1349062
1592.602	13.5	0.387454	0.3344378	0.1263554	0.08535381	0.108104	0.1332207
1592.702	13.5	0.426892	0.3260093	0.1284083	0.08356614	0.108649	0.1332246
1592.802	13.5	0.436374	0.3278331	0.1260024	0.08355314	0.110862	0.1351409
1592.902	13.5	0.436105	0.3116303	0.125308	0.08401673	0.115628	0.1341969
1593.002	13.5	0.445748	0.3087032	0.1244391	0.08564752	0.115933	0.1342219
1593.102	13.5	0.423538	0.326501	0.1246252	0.08568486	0.114029	0.1368083
1593.202	13.5	0.414901	0.3628758	0.1241324	0.08751974	0.115247	0.1373755
1593.302	13.5	0.404	0.3372952	0.1236573	0.0903514	0.115922	0.1388603
1593.402	13.5	0.393954	0.322451	0.1267549	0.0890168	0.117519	0.1396624
1593.502	13.5	0.39113	0.3268844	0.1287526	0.08793698	0.118836	0.1389975
1593.602	13.5	0.413668	0.3420596	0.1268832	0.08931793	0.120809	0.1409741
1593.702	13.5	0.401696	0.3473642	0.1266784	0.0890965	0.121818	0.1408626
1593.802	13.5	0.37446	0.3610709	0.1281888	0.08892354	0.12462	0.1423445
1593.902	13.5	0.377052	0.3548685	0.1297694	0.08661469	0.122105	0.1440521
1594.002	13.5	0.391832	0.3406265	0.1302087	0.08578165	0.121744	0.1454567
1594.102	13.5	0.390887	0.3408221	0.1337968	0.08764637	0.12419	0.1475305
1594.202	13.5	0.393717	0.3428589	0.1345098	0.09053523	0.120086	0.14407

1594.302	13.5	0.389599	0.3463604	0.1345483	0.09379489	0.122978	0.1445436
1594.402	13.5	0.398166	0.3490831	0.1333688	0.09571837	0.125113	0.147203
1594.502	13.5	0.392431	0.3559071	0.1361927	0.09908538	0.127292	0.1491994
1594.602	13.5	0.394452	0.3513691	0.1376863	0.1001568	0.127411	0.1495977
1594.702	13.5	0.396279	0.3418316	0.1339895	0.1004295	0.124417	0.1510992
1594.802	13.5	0.389311	0.3398247	0.1326615	0.1002164	0.124848	0.1451597
1594.902	13.5	0.38607	0.3425351	0.1351521	0.1024115	0.12253	0.1415016
1595.002	13.5	0.395511	0.3308826	0.1345153	0.1006283	0.12094	0.1408487
1595.102	13.5	0.399043	0.3219084	0.1314503	0.0934803	0.122606	0.1407643
1595.202	13.5	0.398183	0.3220623	0.1316238	0.09110495	0.122659	0.1393064
1595.302	13.5	0.390297	0.3052646	0.1304552	0.08887149	0.1188	0.1398791
1595.402	13.5	0.395088	0.3113031	0.1297699	0.08979288	0.117171	0.1401123
1595.502	13.5	0.399172	0.3196558	0.1274917	0.09116671	0.114617	0.1388631
1595.602	13.5	0.385149	0.3422109	0.127389	0.09059867	0.115773	0.1401284
1595.702	13.5	0.388704	0.333461	0.1266376	0.08932338	0.115988	0.1423459
1595.802	13.5	0.385572	0.3301679	0.1277718	0.08895172	0.114468	0.1399756
1595.902	13.5	0.380012	0.330028	0.1318508	0.08878601	0.113952	0.1393642
1596.002	13.5	0.37789	0.3406616	0.1311916	0.08687446	0.114944	0.1381184
1596.102	13.5	0.383569	0.3489811	0.1279393	0.08879843	0.113813	0.1377876
1596.202	13.5	0.386958	0.3626646	0.1303728	0.08927254	0.113659	0.1401278
1596.302	13.5	0.384586	0.3537446	0.1321763	0.09216499	0.109433	0.1405176
1596.402	13.5	0.371066	0.3445622	0.129714	0.09279474	0.109574	0.1405214
1596.502	13.5	0.386603	0.3454992	0.1285189	0.09331426	0.108253	0.1402427
1596.602	13.5	0.383945	0.3470223	0.1299976	0.09251535	0.10849	0.1396924
1596.702	13.5	0.38076	0.3539564	0.1307798	0.09511322	0.112764	0.1395336
1596.802	13.5	0.397499	0.3563002	0.132621	0.09115488	0.113495	0.1396855
1596.902	13.5	0.419281	0.359923	0.133338	0.08960889	0.113522	0.1407957
1597.002	13.5	0.395717	0.3578811	0.1323721	0.08942547	0.110486	0.1408031
1597.102	13.5	0.408101	0.3460442	0.1293023	0.08613651	0.110555	0.1395971
1597.202	13.5	0.401712	0.343563	0.1272051	0.08641195	0.109904	0.1398727
1597.302	13.5	0.397722	0.3399539	0.1273729	0.08919148	0.113467	0.1401932
1597.402	13.5	0.405255	0.3452187	0.1257767	0.08677997	0.115746	0.1399674
1597.502	13.5	0.412191	0.3246057	0.1251786	0.08828688	0.120466	0.1397154
1597.602	13.5	0.399801	0.3209375	0.1265141	0.08933884	0.123343	0.1377441
1597.702	13.5	0.399668	0.310471	0.1279099	0.09258113	0.123434	0.1361893
1597.802	13.5	0.393359	0.3168867	0.1278822	0.09603462	0.119655	0.1359704
1597.902	13.5	0.388268	0.3459477	0.1289932	0.09496172	0.116617	0.1354048
1598.002	13.5	0.406205	0.36688	0.1253963	0.09366602	0.119912	0.1341709
1598.102	13.5	0.419723	0.3436963	0.1264162	0.09453665	0.123433	0.1334062
1598.202	13.5	0.39238	0.341182	0.1274162	0.09594975	0.12547	0.1332632
1598.302	13.5	0.367518	0.3433189	0.1274802	0.09690481	0.125398	0.1350773
1598.402	13.5	0.38484	0.3526796	0.1276741	0.09454818	0.120857	0.137161
1598.502	13.5	0.390025	0.3590252	0.1259876	0.09276326	0.121223	0.1392716
1598.602	13.5	0.383932	0.3600816	0.128058	0.09098549	0.120153	0.1409031
1598.702	13.5	0.387557	0.3557965	0.1266146	0.09219095	0.120487	0.1383587
1598.802	13.5	0.389399	0.344863	0.1274482	0.0945452	0.124265	0.138654
1598.902	13.5	0.394052	0.3481536	0.1257922	0.09570674	0.124493	0.1411646
1599.002	13.5	0.382936	0.3506781	0.1277052	0.09630559	0.127398	0.1450502
1599.102	13.5	0.392357	0.3572718	0.1297546	0.09884457	0.123977	0.1458544
1599.202	13.5	0.394075	0.3579691	0.1329573	0.1028117	0.124974	0.1469498
1599.302	13.5	0.378199	0.3594312	0.1348716	0.1018973	0.125423	0.1465062
1599.402	13.5	0.389878	0.358772	0.1351679	0.1032934	0.123851	0.1434094
1599.502	13.5	0.400543	0.3458245	0.1335781	0.102859	0.120516	0.1451192
1599.602	13.5	0.392811	0.3418512	0.1344317	0.1013237	0.117551	0.1448894
1599.702	13.5	0.39597	0.3381009	0.134292	0.09675209	0.118074	0.1469484
1599.802	13.5	0.389104	0.3493869	0.1358508	0.09483553	0.115629	0.144012
1599.902	13.5	0.395149	0.3268563	0.1363317	0.09391654	0.116045	0.1424589
1600.002	13.5	0.388771	0.3254062	0.1359738	0.09319563	0.116754	0.1448313
1600.102	13.5	0.390895	0.3125589	0.1326469	0.09291221	0.117148	0.1430687
1600.202	13.5	0.385337	0.3128297	0.1306257	0.09011437	0.117609	0.1410538
1600.302	13.5	0.379676	0.341037	0.1311778	0.08924393	0.113859	0.1433298
1600.402	13.5	0.377739	0.3622912	0.1290112	0.0906527	0.113979	0.1422733
1600.502	13.5	0.379364	0.3350128	0.1267434	0.09178073	0.115214	0.1401793
1600.602	13.5	0.390677	0.3272606	0.1279407	0.09161794	0.113404	0.1408165
1600.702	13.5	0.385769	0.3277044	0.1280622	0.09400234	0.113522	0.1428471
1600.802	13.5	0.382114	0.3418008	0.1294491	0.09385237	0.113332	0.1414804
1600.902	13.5	0.380285	0.3463078	0.1299657	0.09294094	0.1111	0.1409134
1601.002	13.5	0.378289	0.3551876	0.1295805	0.09369142	0.110961	0.1406716
1601.102	13.5	0.369227	0.3452387	0.1303999	0.09385494	0.112603	0.1401515
1601.202	13.5	0.389043	0.3339922	0.1298826	0.09233917	0.111192	0.1410758
1601.302	13.5	0.389535	0.3321958	0.1303485	0.0911386	0.110844	0.1431738
1601.402	13.5	0.378541	0.3351613	0.1299235	0.08859602	0.111489	0.143839
1601.502	13.5	0.385955	0.3406539	0.1313992	0.08927965	0.110658	0.1417607
1601.602	13.5	0.394288	0.3430054	0.1310779	0.08847259	0.111768	0.1431413
1601.702	13.5	0.40103	0.3403304	0.1313214	0.09165888	0.113054	0.1405219
1601.802	13.5	0.402898	0.3396044	0.1331508	0.08989789	0.115749	0.1375079
1601.902	13.5	0.414592	0.3328335	0.1331142	0.0892437	0.118912	0.1383642
1602.002	13.5	0.408847	0.3298156	0.1325517	0.09031838	0.118386	0.1385818
1602.102	13.5	0.399984	0.3301461	0.1306408	0.09216453	0.119324	0.1397718
1602.202	13.5	0.400926	0.3450653	0.1307692	0.09363699	0.117204	0.1406366
1602.302	13.5	0.394237	0.3286143	0.1296007	0.09172308	0.119799	0.1390201
1602.402	13.5	0.393419	0.3254107	0.1270889	0.09110001	0.122039	0.1411828
1602.502	13.5	0.428758	0.3170117	0.126317	0.09362633	0.124621	0.1413794
1602.602	13.5	0.407941	0.3142305	0.1263846	0.09254359	0.129298	0.1392339
1602.702	13.5	0.391351	0.3373513	0.128352	0.09383927	0.126298	0.1400436
1602.802	13.5	0.379312	0.3634087	0.1256855	0.0943874	0.121903	0.138737
1602.902	13.5	0.403321	0.3279787	0.1267725	0.09277173	0.121204	0.1388208
1603.002	13.5	0.392894	0.3215709	0.1243285	0.09206419	0.117262	0.1400577
1603.102	13.5	0.390828	0.3242234	0.1255921	0.09041377	0.116573	0.1413244
1603.202	13.5	0.384352	0.3440286	0.1273751	0.09241711	0.116839	0.1419026
1603.302	13.5	0.392168	0.3498679	0.1293328	0.09414956	0.117671	0.1398658
1603.402	13.5	0.391231	0.356965	0.1300757	0.09370331	0.119233	0.1381176
1603.502	13.5	0.396367	0.3465235	0.1271948	0.09506419	0.121309	0.1365367
1603.602	13.5	0.40477	0.3377737	0.1278087	0.09739623	0.122645	0.1362092
1603.702	13.5	0.38894	0.3345601	0.1269711	0.09753471	0.122275	0.1359968

1603.802	13.5	0.398226	0.3380654	0.1278335	0.09744205	0.121192	0.1390157
1603.902	13.5	0.404164	0.3437245	0.1303178	0.09818015	0.120715	0.1403152
1604.002	13.5	0.39648	0.349286	0.1312759	0.09275543	0.119615	0.1414314
1604.102	13.5	0.401317	0.356265	0.1343317	0.09025463	0.119828	0.1429424
1604.202	13.5	0.400223	0.3463493	0.136794	0.09038854	0.119319	0.1432246
1604.302	13.5	0.406087	0.3389164	0.1373761	0.09256143	0.119085	0.1456606
1604.402	13.5	0.413435	0.33444	0.1386137	0.09469403	0.116023	0.1449867
1604.502	13.5	0.38445	0.3354006	0.1397066	0.09581281	0.115496	0.1450281
1604.602	13.5	0.390833	0.3428079	0.1391418	0.09333774	0.115477	0.1437158
1604.702	13.5	0.385938	0.3406227	0.1365679	0.09570722	0.112273	0.1480879
1604.802	13.5	0.384728	0.3288515	0.136333	0.09444313	0.109469	0.1446498
1604.902	13.5	0.395892	0.3169942	0.1353521	0.0953628	0.11037	0.1404204
1605.002	13.5	0.401447	0.3135425	0.1346785	0.09563624	0.109108	0.1395984
1605.102	13.5	0.391713	0.3366915	0.1309603	0.09454125	0.108706	0.1389309
1605.202	13.5	0.386208	0.3574738	0.1281724	0.09547341	0.109587	0.1373601
1605.302	13.5	0.371524	0.3317873	0.127373	0.09337643	0.109674	0.1365378
1605.402	13.5	0.380709	0.325513	0.1275313	0.09253313	0.111457	0.138738
1605.502	13.5	0.377172	0.3249008	0.1263668	0.0922932	0.112657	0.1398955
1605.602	13.5	0.377305	0.3393945	0.1251391	0.09435296	0.113505	0.1378925
1605.702	13.5	0.398724	0.3435072	0.1239644	0.09218507	0.112341	0.1405051
1605.802	13.5	0.384925	0.3562031	0.1266874	0.09198292	0.112784	0.14255
1605.902	13.5	0.381078	0.3490967	0.1311929	0.09210084	0.111331	0.1415673
1606.002	13.5	0.39997	0.3409532	0.1327049	0.09362904	0.110273	0.1398956
1606.102	13.5	0.403363	0.3414228	0.1286656	0.09461607	0.112488	0.1399683
1606.202	13.5	0.400692	0.3412751	0.1315404	0.09690037	0.115944	0.1423525
1606.302	13.5	0.405789	0.3452565	0.1325427	0.09864089	0.115167	0.1412962
1606.402	13.5	0.412012	0.3475345	0.1314882	0.09812637	0.114354	0.140399
1606.502	13.5	0.400018	0.3468089	0.1334486	0.09885089	0.117466	0.1378653
1606.602	13.5	0.39934	0.3456159	0.1309441	0.09803709	0.120714	0.1390679
1606.702	13.5	0.394112	0.3393018	0.1332271	0.09591234	0.12045	0.1392476
1606.802	13.5	0.389323	0.3335746	0.1358754	0.09407555	0.119251	0.1387123
1606.902	13.5	0.411925	0.3339929	0.1339355	0.09635694	0.120651	0.1379291
1607.002	13.5	0.406847	0.3414172	0.1345392	0.09638685	0.125654	0.1365101
1607.102	13.5	0.388436	0.3348958	0.1326344	0.09640486	0.12603	0.1388967
1607.202	13.5	0.368395	0.3326508	0.1300053	0.09636228	0.12488	0.1375978
1607.302	13.5	0.392463	0.3243823	0.1305424	0.09074359	0.123375	0.1380991
1607.402	13.5	0.394725	0.3202324	0.130063	0.08892877	0.121226	0.1392193
1607.502	13.5	0.384415	0.3465663	0.1288106	0.08927668	0.118522	0.1385514
1607.602	13.5	0.38804	0.3656949	0.1276121	0.09291916	0.11658	0.1370436
1607.702	13.5	0.386843	0.3370216	0.1302007	0.09754051	0.118576	0.1407936
1607.802	13.5	0.393766	0.3283558	0.129561	0.09673504	0.120782	0.1388252
1607.902	13.5	0.38176	0.3296495	0.1283855	0.09819777	0.123143	0.1383004
1608.002	13.5	0.389456	0.3418639	0.1275524	0.1014028	0.120478	0.1383316
1608.102	13.5	0.39237	0.3477891	0.1248083	0.1016058	0.120642	0.134936
1608.202	13.5	0.376684	0.3539859	0.1248113	0.1024489	0.119201	0.1360628
1608.302	13.5	0.392257	0.3540549	0.1263051	0.1040868	0.11834	0.1379761
1608.402	13.5	0.396844	0.3357464	0.1294232	0.1014159	0.118714	0.1373804
1608.502	13.5	0.394204	0.3358042	0.1297426	0.09667917	0.119159	0.1382914
1608.602	13.5	0.393394	0.3421396	0.1287208	0.09664789	0.120853	0.1393194
1608.702	13.5	0.394084	0.3510561	0.1234992	0.09659126	0.118568	0.140011
1608.802	13.5	0.398804	0.348798	0.1241088	0.09550197	0.118199	0.1400222
1608.902	13.5	0.387921	0.347317	0.1254319	0.09695222	0.11811	0.1393649
1609.002	13.5	0.388597	0.3411888	0.124832	0.0949496	0.11779	0.1412954
1609.102	13.5	0.38256	0.3367601	0.1265728	0.09316329	0.116432	0.1446277
1609.202	13.5	0.375875	0.3328501	0.1282551	0.09440938	0.113727	0.1431637
1609.302	13.5	0.370989	0.3380454	0.1310457	0.09450285	0.115654	0.1444058
1609.402	13.5	0.381251	0.3378995	0.1347921	0.093523	0.117748	0.1455888
1609.502	13.5	0.386505	0.3318148	0.1331287	0.09353516	0.116695	0.1449115
1609.602	13.5	0.385283	0.3230256	0.1342358	0.09428175	0.116718	0.1451627
1609.702	13.5	0.372073	0.3127997	0.1343367	0.09406624	0.117752	0.1459289
1609.802	13.5	0.376904	0.3145468	0.1316867	0.09162385	0.116211	0.1436011
1609.902	13.5	0.373018	0.3429102	0.1317947	0.09166097	0.11427	0.1423164
1610.002	13.5	0.36805	0.3657399	0.1309325	0.09369478	0.114078	0.1429144
1610.102	13.5	0.392901	0.3357984	0.1301322	0.0916298	0.113419	0.1400084
1610.202	13.5	0.389735	0.3283296	0.1279593	0.09127604	0.110245	0.1384331
1610.302	13.5	0.379657	0.3284445	0.1295165	0.08918641	0.110538	0.1395527
1610.402	13.5	0.391992	0.3423358	0.1277233	0.09003728	0.11184	0.139503
1610.502	13.5	0.401894	0.3478591	0.1244961	0.0872456	0.111008	0.1361651
1610.602	13.5	0.402312	0.3598252	0.1242055	0.09121995	0.111384	0.1383163
1610.702	13.5	0.40122	0.3549005	0.123013	0.09076699	0.11399	0.1400416
1610.802	13.5	0.430923	0.3370156	0.1267126	0.09022463	0.1176	0.1402434
1610.902	13.5	0.440424	0.3356558	0.1281265	0.08887541	0.12072	0.1402193
1611.002	13.5	0.45661	0.3373889	0.1296244	0.08938093	0.123517	0.1408064
1611.102	13.5	0.427027	0.3422287	0.1291974	0.09099407	0.122516	0.1385124
1611.202	13.5	0.413177	0.3476568	0.130919	0.09156226	0.120185	0.1388907
1611.302	13.5	0.422508	0.3477881	0.1322111	0.08955688	0.12237	0.1391002
1611.402	13.5	0.438842	0.3480388	0.1328783	0.09225266	0.123805	0.1383475
1611.502	13.5	0.432217	0.3398976	0.1317345	0.08934366	0.124993	0.1376572
1611.602	13.5	0.408997	0.3334656	0.1335797	0.09067181	0.127563	0.1375358
1611.702	13.5	0.390959	0.336751	0.1355888	0.09312198	0.124521	0.1377957
1611.802	13.5	0.403229	0.3342036	0.1330536	0.09208566	0.122556	0.1388667
1611.902	13.5	0.382698	0.3236405	0.1324147	0.0945114	0.117182	0.1391876
1612.002	13.5	0.391791	0.3264334	0.1328228	0.09592631	0.118793	0.1393023
1612.102	13.5	0.380987	0.3197133	0.1310269	0.09883729	0.116838	0.1382145
1612.202	13.5	0.393639	0.3161693	0.1296231	0.09920397	0.119667	0.1382961
1612.302	13.5	0.38434	0.3407928	0.1281661	0.1004247	0.121575	0.1402947
1612.402	13.5	0.381845	0.3628435	0.1263858	0.1031888	0.122929	0.143221
1612.502	13.5	0.396425	0.3296387	0.124506	0.1033314	0.123648	0.1419431
1612.602	13.5	0.385292	0.3294547	0.124684	0.1013786	0.126536	0.1394315
1612.702	13.5	0.397303	0.3351516	0.1254018	0.1007399	0.124979	0.1420145
1612.802	13.5	0.403521	0.3435397	0.1245659	0.1009876	0.124249	0.1413478
1612.902	13.5	0.399234	0.3496534	0.12363	0.09986015	0.123571	0.1397046
1613.002	13.5	0.406189	0.3593076	0.1214863	0.0984853	0.122779	0.1370141
1613.102	13.5	0.408814	0.3597299	0.1208955	0.09767159	0.12357	0.1381264
1613.202	13.5	0.401723	0.3377729	0.121479	0.09718802	0.120698	0.1383719

1613.302	13.5	0.400616	0.3327674	0.1222251	0.09744824	0.119853	0.1395119
1613.402	13.5	0.384461	0.3335598	0.1227501	0.09754044	0.120496	0.1388546
1613.502	13.5	0.388322	0.3398834	0.1219688	0.09371392	0.119976	0.139864
1613.602	13.5	0.37912	0.3490693	0.1234483	0.0916344	0.116435	0.1411681
1613.702	13.5	0.376994	0.3447942	0.1233468	0.09067452	0.114849	0.1388661
1613.802	13.5	0.384379	0.3427828	0.1262053	0.09051256	0.113627	0.140192
1613.902	13.5	0.392345	0.339896	0.1263163	0.09179914	0.11391	0.1400809
1614.002	13.5	0.384557	0.3353162	0.1295129	0.09303402	0.112479	0.1414109
1614.102	13.5	0.383826	0.3342299	0.1322978	0.09077427	0.109469	0.1457948
1614.202	13.5	0.383166	0.3329817	0.1366896	0.09177215	0.108526	0.1469573
1614.302	13.5	0.387486	0.3273861	0.1380753	0.09090708	0.112157	0.1449737
1614.402	13.5	0.382648	0.3249702	0.1391105	0.09346767	0.111494	0.1456834
1614.502	13.5	0.386421	0.3156796	0.1376713	0.09170122	0.113665	0.1456274
1614.602	13.5	0.401418	0.3176571	0.1384575	0.08773171	0.114985	0.1421489
1614.702	13.5	0.386149	0.3291663	0.1349567	0.08831249	0.11492	0.1436124
1614.802	13.5	0.385532	0.3572378	0.132664	0.08830309	0.115565	0.1398688
1614.902	13.5	0.410447	0.327747	0.1317411	0.08861257	0.114094	0.138267
1615.002	13.5	0.41517	0.3234726	0.1310171	0.08907465	0.11355	0.1407417
1615.102	13.5	0.414637	0.3256166	0.1303844	0.09045365	0.114669	0.1402038
1615.202	13.5	0.4164	0.3441986	0.1302471	0.08938908	0.114893	0.1382865
1615.302	13.5	0.413873	0.3480406	0.1292171	0.09063015	0.118451	0.1377411
1615.402	13.5	0.397859	0.3574065	0.1289599	0.09030641	0.118623	0.1381021
1615.502	13.5	0.402152	0.355985	0.128382	0.09209774	0.116948	0.134104
1615.602	13.5	0.40809	0.3392571	0.1285392	0.09080435	0.119129	0.1330438
1615.702	13.5	0.401998	0.3338615	0.1279456	0.08774208	0.121898	0.135987
1615.802	13.5	0.427853	0.3353719	0.1304994	0.08997408	0.122894	0.1356099
1615.902	13.5	0.430738	0.3397951	0.1314721	0.09061752	0.124772	0.1350262
1616.002	13.5	0.38717	0.3481154	0.1314628	0.09196547	0.126318	0.1342962
1616.102	13.5	0.37183	0.3442146	0.1298482	0.09107824	0.124209	0.1338297
1616.202	13.5	0.382669	0.3461327	0.1349348	0.09080102	0.122116	0.1357369
1616.302	13.5	0.391043	0.3414427	0.1365793	0.09252264	0.124107	0.1360836
1616.402	13.5	0.384401	0.3339445	0.1372972	0.09396721	0.122795	0.1352741
1616.502	13.5	0.385261	0.3342155	0.1376074	0.09303422	0.121312	0.1355859
1616.602	13.5	0.38602	0.328548	0.1346319	0.09433322	0.12059	0.135347
1616.702	13.5	0.391485	0.3243503	0.1348942	0.09500337	0.124477	0.1376271
1616.802	13.5	0.379235	0.3284962	0.1356875	0.09594336	0.127115	0.1392325
1616.902	13.5	0.388121	0.3156875	0.1360348	0.09755132	0.123605	0.1376602
1617.002	13.5	0.393684	0.3122554	0.1353761	0.09735904	0.122987	0.1379749
1617.102	13.5	0.379026	0.3341257	0.1351996	0.09482673	0.123083	0.1374417
1617.202	13.5	0.395948	0.3665791	0.1338718	0.09875112	0.122654	0.1353887
1617.302	13.5	0.398434	0.3323078	0.1324175	0.09788622	0.124156	0.1340185
1617.402	13.5	0.394624	0.3184746	0.1292318	0.09629082	0.12233	0.1345213
1617.502	13.5	0.39038	0.3293744	0.1262144	0.09554565	0.120211	0.1342663
1617.602	13.5	0.390293	0.3433479	0.1251677	0.09506218	0.118885	0.1333252
1617.702	13.5	0.395171	0.3490643	0.1256407	0.09815604	0.116477	0.1332578
1617.802	13.5	0.388169	0.3573278	0.1238862	0.09587511	0.116863	0.1324953
1617.902	13.5	0.38797	0.3550145	0.1244221	0.09391821	0.115385	0.1354065
1618.002	13.5	0.385065	0.3369956	0.125004	0.0932696	0.115708	0.1335347
1618.102	13.5	0.386591	0.3340633	0.1246467	0.09286319	0.112943	0.1338455
1618.202	13.5	0.38267	0.3419587	0.125886	0.0909899	0.109162	0.1319942
1618.302	13.5	0.371857	0.3506945	0.1274195	0.09160412	0.108211	0.1347796
1618.402	13.5	0.400293	0.3447455	0.1269835	0.09213147	0.112129	0.1371601
1618.502	13.5	0.408271	0.3460752	0.1275701	0.09250566	0.109545	0.1334122
1618.602	13.5	0.409654	0.3514408	0.1285109	0.09333989	0.110241	0.1346012
1618.702	13.5	0.417165	0.3391456	0.1267109	0.09190726	0.112168	0.1370342
1618.9	14	0.399412	0.3341841	0.1292923	0.09173593	0.11358	0.1411068
1619	14	0.387882	0.3327998	0.1302952	0.09107929	0.115342	0.1367264
1619.1	14	0.404745	0.3343058	0.1336447	0.09187397	0.113663	0.1397338
1619.2	14	0.408103	0.3414077	0.1349035	0.09174144	0.110796	0.1412646
1619.3	14	0.403185	0.3339428	0.1342459	0.09401564	0.111098	0.1373482
1619.4	14	0.407365	0.3431873	0.1319701	0.09163819	0.113021	0.1374232
1619.5	14	0.413973	0.3407789	0.1337091	0.0930548	0.110309	0.1393759
1619.6	14	0.404329	0.3359571	0.1348661	0.09300626	0.111863	0.1389118
1619.7	14	0.407169	0.3386605	0.1367701	0.09435407	0.112372	0.1372049
1619.8	14	0.399839	0.3368236	0.1333647	0.09158668	0.113623	0.1402104
1619.9	14	0.391553	0.3425978	0.1322943	0.09098778	0.112016	0.1405852
1620	14	0.40719	0.3437631	0.1310811	0.09057694	0.115336	0.1407191
1620.1	14	0.394971	0.3395923	0.1321956	0.09172042	0.114632	0.1418357
1620.2	14	0.373125	0.3434502	0.1320165	0.09329694	0.118401	0.141573
1620.3	14	0.366922	0.3395925	0.1300014	0.09297838	0.119553	0.1375889
1620.4	14	0.39343	0.3426334	0.1292919	0.09204347	0.121152	0.1361794
1620.5	14	0.38194	0.3494538	0.1283988	0.0928926	0.125866	0.1378228
1620.6	14	0.388325	0.3414321	0.1267882	0.09443358	0.129576	0.1342696
1620.7	14	0.38135	0.3500605	0.1299187	0.09385311	0.126826	0.134478
1620.8	14	0.391729	0.3497666	0.1304773	0.09242189	0.126104	0.1388584
1620.9	14	0.382826	0.3526616	0.1277663	0.09370167	0.121372	0.1372487
1621	14	0.384949	0.3477792	0.1287426	0.09453074	0.119319	0.1371529
1621.1	14	0.394337	0.352072	0.1325371	0.09430081	0.118067	0.1380306
1621.2	14	0.382688	0.3519053	0.1335883	0.09344905	0.122248	0.137049
1621.3	14	0.403384	0.3428479	0.1305293	0.09657378	0.125832	0.1393658
1621.4	14	0.399479	0.3521394	0.1316781	0.09697617	0.124392	0.1382776
1621.5	14	0.394239	0.3550923	0.1316568	0.0966813	0.124001	0.1366251
1621.6	14	0.392976	0.3486603	0.1324446	0.09715128	0.120406	0.1351731
1621.7	14	0.389529	0.3579644	0.1319135	0.09661476	0.119308	0.1372126
1621.8	14	0.39391	0.3569914	0.1333445	0.0990539	0.120791	0.1369368
1621.9	14	0.388218	0.3511061	0.1308325	0.09836508	0.123762	0.1346094
1622	14	0.386294	0.3519557	0.1295137	0.09663714	0.124275	0.1359552
1622.1	14	0.378206	0.3530146	0.129548	0.09541809	0.1213	0.1344197
1622.2	14	0.374748	0.3453239	0.127755	0.09404837	0.120799	0.1321211
1622.3	14	0.371639	0.3454908	0.1264651	0.09343279	0.119836	0.133326
1622.4	14	0.382223	0.346967	0.1261762	0.09263024	0.116227	0.133872
1622.5	14	0.379654	0.3497308	0.1267451	0.09149402	0.117526	0.1370265
1622.6	14	0.381625	0.3481104	0.1236806	0.09252855	0.115518	0.1380223
1622.7	14	0.374893	0.3526592	0.1244981	0.09365368	0.113697	0.1368662
1622.8	14	0.379241	0.3588176	0.1245156	0.09309135	0.113401	0.1373019

1622.9	14	0.381996	0.358504	0.123862	0.0921123	0.112973	0.138976
1623	14	0.383971	0.3638463	0.1260553	0.09264538	0.113259	0.1388342
1623.1	14	0.399985	0.3589882	0.1275903	0.09327691	0.115354	0.1354379
1623.2	14	0.382647	0.3613777	0.1300165	0.09472744	0.113091	0.1347703
1623.3	14	0.381607	0.368913	0.1305647	0.0945567	0.11143	0.1348469
1623.4	14	0.407045	0.3649888	0.1307773	0.09316441	0.109272	0.1345443
1623.5	14	0.406666	0.366	0.1310894	0.09324423	0.109132	0.1363332
1623.6	14	0.406962	0.3582589	0.133752	0.09394736	0.110398	0.1379856
1623.7	14	0.40855	0.3563272	0.1309543	0.08893166	0.107754	0.1372987
1623.8	14	0.412455	0.3590574	0.131959	0.09026368	0.108781	0.1365365
1623.9	14	0.40013	0.3636412	0.1363297	0.09058868	0.109086	0.1372369
1624	14	0.404982	0.3532277	0.1369063	0.09328135	0.10926	0.138398
1624.1	14	0.399305	0.3594779	0.1392323	0.09197751	0.110823	0.1399857
1624.2	14	0.392119	0.3553637	0.1408751	0.09119071	0.112884	0.1405533
1624.3	14	0.402375	0.3512929	0.1389045	0.09139435	0.114746	0.1390067
1624.4	14	0.393957	0.3521247	0.1370216	0.09058849	0.115994	0.1415006
1624.5	14	0.370621	0.353399	0.1339367	0.09348533	0.118693	0.1431921
1624.6	14	0.371259	0.3472344	0.1312969	0.09383315	0.120439	0.1425518
1624.7	14	0.391408	0.3541995	0.1316587	0.09469786	0.12309	0.1412476
1624.8	14	0.383092	0.3466948	0.1309031	0.09685918	0.127395	0.1445102
1624.9	14	0.388278	0.3561085	0.1293233	0.09910164	0.129865	0.1426059
1625	14	0.383275	0.3563596	0.1287044	0.09945895	0.131808	0.1415899
1625.1	14	0.38878	0.3611704	0.1294001	0.0999091	0.125957	0.1419903
1625.2	14	0.380959	0.3542323	0.1297552	0.09926416	0.125544	0.1407264
1625.3	14	0.387577	0.3617789	0.1287215	0.09597909	0.12337	0.1390172
1625.4	14	0.390655	0.3726591	0.1285334	0.09392355	0.123263	0.1397607
1625.5	14	0.379619	0.3548201	0.1316478	0.09443051	0.123068	0.1434175
1625.6	14	0.400916	0.3677045	0.132022	0.09695995	0.126489	0.1404376
1625.7	14	0.400074	0.3715594	0.1297724	0.09588605	0.12835	0.1379196
1625.8	14	0.392839	0.3560975	0.1301127	0.09419986	0.12791	0.1429407
1625.9	14	0.387237	0.3647734	0.1310598	0.0930737	0.123899	0.1399634
1626	14	0.394306	0.3619268	0.1326992	0.09567411	0.122666	0.1387955
1626.1	14	0.393416	0.3620305	0.1316877	0.09853595	0.119221	0.1401498
1626.2	14	0.385972	0.3709769	0.1298392	0.09766934	0.119237	0.1408233
1626.3	14	0.389261	0.3686927	0.1294382	0.09575843	0.122999	0.1423646
1626.4	14	0.374121	0.3648282	0.1303148	0.0933278	0.125472	0.1414573
1626.5	14	0.372167	0.3617434	0.1310743	0.09229543	0.12471	0.1415851
1626.6	14	0.376613	0.3595318	0.1327601	0.09145986	0.125191	0.1395909
1626.7	14	0.382383	0.3571535	0.1305446	0.09080476	0.123099	0.1391995
1626.8	14	0.382085	0.3556478	0.1297798	0.0929493	0.121446	0.142584
1626.9	14	0.379275	0.3591083	0.128973	0.09540895	0.121328	0.1410761
1627	14	0.372635	0.3654975	0.1293428	0.09591081	0.118224	0.1402905
1627.1	14	0.369994	0.3675961	0.1273536	0.09402607	0.115668	0.1380612
1627.2	14	0.366901	0.3681213	0.1276517	0.09137294	0.112531	0.1406828
1627.3	14	0.381643	0.3665433	0.129568	0.09221011	0.113352	0.1395832
1627.4	14	0.384466	0.3586754	0.1270023	0.0906434	0.113585	0.1394171
1627.5	14	0.378875	0.3557744	0.1269665	0.08970705	0.114454	0.1403352
1627.6	14	0.383887	0.3561686	0.125076	0.08999086	0.113018	0.1381257
1627.7	14	0.400721	0.3639993	0.1256964	0.09163395	0.114579	0.137145
1627.8	14	0.40106	0.3622729	0.1259492	0.09376128	0.11471	0.1349913
1627.9	14	0.401976	0.3541608	0.1264859	0.09519663	0.115015	0.1306541
1628	14	0.402627	0.3534186	0.1301949	0.09188657	0.113173	0.1310476
1628.1	14	0.401194	0.3547384	0.1319461	0.09354093	0.112253	0.1302986
1628.2	14	0.402776	0.3580283	0.1311081	0.09620421	0.114154	0.1343901
1628.3	14	0.404128	0.348544	0.1303469	0.09598897	0.116579	0.1330021
1628.4	14	0.396376	0.3542731	0.1303594	0.09268984	0.116121	0.1315199
1628.5	14	0.402703	0.3507931	0.1280401	0.09154087	0.11543	0.1334509
1628.6	14	0.403531	0.3505515	0.1303912	0.09196763	0.115698	0.1322851
1628.7	14	0.387572	0.3597036	0.1352319	0.09139939	0.116613	0.1374342
1628.8	14	0.36599	0.3599063	0.1378403	0.09362347	0.117072	0.1380193
1628.9	14	0.377792	0.3610412	0.1366122	0.09418897	0.11921	0.1363655
1629	14	0.394092	0.3547746	0.1374286	0.09227395	0.119703	0.137926
1629.1	14	0.38487	0.3546192	0.1382431	0.09567938	0.125038	0.1409043
1629.2	14	0.38804	0.3619144	0.1388604	0.09932256	0.127869	0.1434741
1629.3	14	0.385946	0.3659895	0.1368213	0.1021134	0.129098	0.1438406
1629.4	14	0.389099	0.3593059	0.1355814	0.09885786	0.130223	0.1450753
1629.5	14	0.382846	0.3630346	0.1349057	0.09998846	0.129133	0.1447751
1629.6	14	0.393603	0.3556517	0.1342438	0.09744341	0.123196	0.1448004
1629.7	14	0.390297	0.3594108	0.132444	0.09930365	0.123653	0.1420947
1629.8	14	0.386255	0.3488482	0.1328071	0.09875444	0.121342	0.1435577
1629.9	14	0.404762	0.3513957	0.1328252	0.09976821	0.120036	0.1398428
1630	14	0.399263	0.3547074	0.1320187	0.09979635	0.120077	0.1382009
1630.1	14	0.39471	0.3508118	0.1308693	0.09511405	0.122924	0.1385376
1630.2	14	0.395124	0.3475901	0.1286288	0.09448507	0.124909	0.1364047
1630.3	14	0.393876	0.3508853	0.1317935	0.09598515	0.119149	0.1358237
1630.4	14	0.3978	0.3514881	0.133757	0.0970309	0.116796	0.1365324
1630.5	14	0.38309	0.3520849	0.1325763	0.09570413	0.115725	0.1371921
1630.6	14	0.38674	0.3520551	0.134439	0.09418884	0.115146	0.1347284
1630.7	14	0.37537	0.3578221	0.1358954	0.0920654	0.118189	0.1345241
1630.8	14	0.373207	0.3498498	0.1362869	0.09124885	0.120045	0.1365225
1630.9	14	0.379676	0.3490621	0.1356087	0.09057744	0.119617	0.1364476
1631	14	0.382009	0.3474175	0.132951	0.08999921	0.119366	0.1353454
1631.1	14	0.386307	0.3510318	0.1319386	0.09245546	0.116644	0.1376201
1631.2	14	0.383705	0.3520661	0.1354629	0.0925511	0.115932	0.137392
1631.3	14	0.380888	0.339639	0.133932	0.09004863	0.115789	0.1388905
1631.4	14	0.379102	0.3472295	0.1350119	0.09124021	0.117215	0.1372327
1631.5	14	0.361101	0.342113	0.1351015	0.09048425	0.114955	0.1378018
1631.6	14	0.380327	0.3444223	0.1340453	0.09239233	0.112729	0.1353813
1631.7	14	0.381333	0.3506338	0.1338709	0.08919811	0.113265	0.1365384
1631.8	14	0.37399	0.3432133	0.1305016	0.0902038	0.113995	0.1387769
1631.9	14	0.383204	0.3114699	0.1265183	0.08822193	0.113543	0.1404803
1632	14	0.399658	0.33574	0.1263605	0.08753067	0.112254	0.1396457
1632.1	14	0.399755	0.3385903	0.1264168	0.08724868	0.109813	0.1376836
1632.2	14	0.397542	0.3336244	0.1261558	0.09026174	0.110709	0.1395158
1632.3	14	0.410358	0.3393186	0.1253009	0.08609647	0.110068	0.1377816

1632.4	14	0.397501	0.3341301	0.1246039	0.0880639	0.109983	0.1372484
1632.5	14	0.402526	0.3373949	0.1266975	0.08942201	0.111586	0.138875
1632.6	14	0.401905	0.3439887	0.1276774	0.09008338	0.109844	0.1395376
1632.7	14	0.394029	0.3486836	0.1282416	0.08568435	0.108769	0.1363421
1632.8	14	0.397165	0.3556913	0.1296397	0.08357637	0.110894	0.1338001
1632.9	14	0.398751	0.3468551	0.1296237	0.08392428	0.111518	0.1309606
1633	14	0.38223	0.3423126	0.130928	0.08487463	0.113056	0.1342882
1633.1	14	0.364545	0.3442754	0.1327116	0.08931986	0.116654	0.1332168
1633.2	14	0.379766	0.3339707	0.1325833	0.09112639	0.116234	0.1335267
1633.3	14	0.391877	0.3291435	0.1329143	0.09197386	0.116878	0.1318109
1633.4	14	0.386969	0.329644	0.1353093	0.09344875	0.120802	0.1342011
1633.5	14	0.385242	0.33429	0.1354352	0.09430673	0.125957	0.1331426
1633.6	14	0.387109	0.3303902	0.1346355	0.09953617	0.126509	0.1326797
1633.7	14	0.387171	0.3287985	0.1343387	0.09946868	0.126392	0.1336809
1633.8	14	0.382157	0.3374238	0.1342667	0.09853396	0.125993	0.1346077
1633.9	14	0.392628	0.3397882	0.1367652	0.1006445	0.126351	0.1332652
1634	14	0.391648	0.3386706	0.1375766	0.1005157	0.120295	0.1343361
1634.1	14	0.388731	0.3342003	0.1348294	0.09831639	0.119345	0.1383961
1634.2	14	0.399297	0.3424698	0.1329622	0.09865604	0.116844	0.1395841
1634.3	14	0.397885	0.3359638	0.1324733	0.0965068	0.114099	0.1368115
1634.4	14	0.393198	0.3412949	0.1320484	0.09387386	0.111608	0.1369707
1634.5	14	0.394175	0.3452302	0.1316617	0.09463152	0.112195	0.1363021
1634.6	14	0.396024	0.3413267	0.1320126	0.0968906	0.112962	0.1384514
1634.7	14	0.395282	0.3409726	0.1323856	0.09670313	0.115318	0.1383936
1634.8	14	0.378349	0.3395545	0.131506	0.09635097	0.116006	0.1406166
1634.9	14	0.386165	0.3461539	0.1316147	0.09624028	0.115109	0.1377358
1635	14	0.375776	0.3453549	0.1334999	0.09481348	0.113783	0.1333986
1635.1	14	0.374188	0.3403872	0.1341603	0.09541954	0.113678	0.1337753
1635.2	14	0.377587	0.3465724	0.1354418	0.09452635	0.116596	0.1353955
1635.3	14	0.382718	0.3406533	0.1347159	0.09397285	0.119132	0.1352732
1635.4	14	0.387097	0.3406653	0.1358152	0.09406382	0.121246	0.1337629
1635.5	14	0.381202	0.3514269	0.1355931	0.09285127	0.119906	0.1360461
1635.6	14	0.371719	0.3438447	0.1350128	0.0908984	0.118693	0.1344207
1635.7	14	0.372303	0.3478585	0.1320416	0.09092175	0.11784	0.1353923
1635.8	14	0.366648	0.350239	0.1312889	0.08952473	0.119681	0.1358487
1635.9	14	0.383864	0.354768	0.1336424	0.09056876	0.115516	0.1379589
1636	14	0.390794	0.3486398	0.1386355	0.08926292	0.11329	0.1364481
1636.1	14	0.380171	0.3507821	0.1376133	0.09003182	0.114071	0.1353841
1636.2	14	0.393394	0.3489223	0.1364098	0.09066934	0.114383	0.1345621
1636.3	14	0.401767	0.3427438	0.1330676	0.091493	0.114631	0.1350348
1636.4	14	0.404648	0.3580609	0.1310023	0.09255174	0.115861	0.1371357
1636.5	14	0.402744	0.3586462	0.1313185	0.09275123	0.113278	0.136532
1636.6	14	0.4124	0.3548315	0.1288061	0.09175364	0.112724	0.1355008
1636.7	14	0.407197	0.3604494	0.1281213	0.09193329	0.112582	0.1381346
1636.8	14	0.43364	0.3591045	0.1269957	0.09379888	0.11326	0.1387304
1636.9	14	0.417023	0.3527964	0.1266581	0.09461866	0.113428	0.1379555
1637	14	0.399493	0.358463	0.1262443	0.095677	0.113281	0.1357907
1637.1	14	0.41224	0.3587033	0.1272013	0.09449009	0.113634	0.1329776
1637.2	14	0.407547	0.3524674	0.128046	0.09263287	0.113508	0.1322571
1637.3	14	0.38899	0.3508025	0.1281931	0.09215783	0.112061	0.1331589
1637.4	14	0.364594	0.3485363	0.130093	0.09362974	0.11181	0.1348929
1637.5	14	0.381132	0.349319	0.1334431	0.09156814	0.109854	0.1349139
1637.6	14	0.394084	0.3518572	0.1332116	0.09425145	0.112606	0.1330594
1637.7	14	0.388211	0.3573678	0.1320385	0.09658877	0.112218	0.132465
1637.8	14	0.386042	0.3617926	0.1313863	0.1029141	0.115505	0.1351206
1637.9	14	0.39082	0.3652691	0.1293824	0.1057669	0.119943	0.1352491
1638	14	0.38716	0.3627547	0.1322116	0.1044333	0.125245	0.1346532
1638.1	14	0.382065	0.3563181	0.1317366	0.1039257	0.126027	0.1314395
1638.2	14	0.388542	0.3611482	0.130854	0.10057	0.128019	0.1309275
1638.3	14	0.386835	0.3671316	0.1333184	0.1009574	0.13182	0.1290124
1638.4	14	0.398041	0.3651504	0.1328551	0.1006861	0.130252	0.1332377
1638.5	14	0.399081	0.3669975	0.1334188	0.1005691	0.127599	0.1346315
1638.6	14	0.393944	0.361011	0.13272	0.09844039	0.128106	0.132846
1638.7	14	0.393331	0.3594243	0.1347991	0.09587573	0.124969	0.1354328
1638.8	14	0.392278	0.3607305	0.1335992	0.09620547	0.121667	0.1364679
1638.9	14	0.393099	0.3656763	0.1322808	0.09933186	0.121687	0.1380789
1639	14	0.388115	0.3521834	0.1303674	0.1012182	0.120582	0.14039
1639.1	14	0.381668	0.3578883	0.129282	0.1013899	0.12378	0.1422617
1639.2	14	0.382504	0.356549	0.128731	0.09981328	0.123719	0.1438065
1639.3	14	0.377404	0.3536907	0.1281078	0.09815909	0.123746	0.1407119
1639.4	14	0.373647	0.3523966	0.1308039	0.09890878	0.124621	0.143549
1639.5	14	0.379438	0.3528388	0.1278538	0.0965894	0.122516	0.14554
1639.6	14	0.388897	0.3504637	0.1276598	0.09481043	0.125568	0.1452187
1639.7	14	0.386547	0.3584174	0.1262797	0.09359902	0.122691	0.138875
1639.8	14	0.380923	0.3531208	0.1262215	0.09347482	0.12217	0.1401625
1639.9	14	0.387185	0.3596407	0.1311941	0.09311842	0.119713	0.137339
1640	14	0.378254	0.3556199	0.1327089	0.09412197	0.118956	0.1375733
1640.1	14	0.377088	0.3599299	0.132544	0.09333196	0.119142	0.1376078
1640.2	14	0.390344	0.3519365	0.1323364	0.09318475	0.120389	0.1400158
1640.3	14	0.385684	0.3588907	0.131401	0.09223653	0.119367	0.1377396
1640.4	14	0.381003	0.3656506	0.13226	0.0922114	0.115117	0.1372683
1640.5	14	0.39617	0.3482613	0.1319775	0.09067249	0.112618	0.1397308
1640.6	14	0.402859	0.3552142	0.1320993	0.09093766	0.116004	0.1403995
1640.7	14	0.401459	0.3659812	0.1319102	0.09240092	0.115081	0.1394246
1640.8	14	0.400684	0.3546668	0.1311723	0.09036131	0.110829	0.140299
1640.9	14	0.408181	0.3662141	0.1302701	0.09015863	0.111336	0.1404022
1641	14	0.399397	0.3622697	0.1310255	0.09044124	0.11057	0.1415357
1641.1	14	0.405502	0.3596871	0.1292661	0.09207186	0.111546	0.1416149
1641.2	14	0.414818	0.3694177	0.1284573	0.09433807	0.112405	0.1389344
1641.3	14	0.395543	0.3695002	0.1262793	0.09412228	0.109798	0.1385214
1641.4	14	0.417542	0.365856	0.1227686	0.09402715	0.108213	0.1377243
1641.5	14	0.40999	0.3639437	0.120135	0.09382548	0.1093	0.1392176
1641.6	14	0.407262	0.359866	0.1220597	0.09256107	0.111289	0.1383488
1641.7	14	0.378005	0.3570966	0.1232315	0.09549807	0.109143	0.1381361
1641.8	14	0.400842	0.3550006	0.121137	0.09255034	0.109071	0.1370622

1641.9	14	0.391942	0.3569266	0.1223159	0.09501406	0.112847	0.1371966
1642	14	0.386132	0.3636431	0.1224218	0.09466106	0.113266	0.13551
1642.1	14	0.383336	0.3656013	0.1249524	0.09975778	0.112538	0.1351649
1642.2	14	0.388441	0.3665881	0.1269541	0.1017848	0.116286	0.1369508
1642.3	14	0.383194	0.3660461	0.1299899	0.1001922	0.12039	0.1358946
1642.4	14	0.390378	0.359795	0.1311604	0.09920356	0.124598	0.1360639
1642.5	14	0.39501	0.3532288	0.1296209	0.09790497	0.124691	0.137034
1642.6	14	0.389283	0.3553438	0.1302538	0.09638049	0.125135	0.1346356
1642.7	14	0.413385	0.3651175	0.1326817	0.09840088	0.129085	0.1333616
1642.8	14	0.408394	0.3646428	0.132447	0.09738226	0.129391	0.1335687
1642.9	14	0.398895	0.3603474	0.1306372	0.09622805	0.126852	0.1340636
1643	14	0.399347	0.3554435	0.130732	0.09379845	0.125036	0.134013
1643.1	14	0.398692	0.3569377	0.1335379	0.09452674	0.122053	0.1323075
1643.2	14	0.402338	0.3593312	0.1325887	0.09716903	0.120564	0.1341498
1643.3	14	0.391912	0.349886	0.1351663	0.09586563	0.118629	0.1335719
1643.4	14	0.385315	0.356438	0.1358901	0.09771129	0.123309	0.1329646
1643.5	14	0.382464	0.3507984	0.1362565	0.09515087	0.125365	0.1361724
1643.6	14	0.379816	0.3519903	0.1379924	0.09412678	0.123916	0.1397173
1643.7	14	0.374391	0.3601337	0.1352946	0.09403501	0.120968	0.1443053
1643.8	14	0.386041	0.3571661	0.132238	0.09392839	0.119269	0.144649
1643.9	14	0.384445	0.3606263	0.1287251	0.0923479	0.119001	0.1444606
1644	14	0.388305	0.3557189	0.1258329	0.09113007	0.120315	0.145449
1644.1	14	0.369663	0.3549255	0.1257239	0.09131409	0.12141	0.1425389
1644.2	14	0.382101	0.3611714	0.1253187	0.09122052	0.121179	0.1411568
1644.3	14	0.37533	0.3638122	0.1261065	0.0919815	0.118343	0.140273
1644.4	14	0.373351	0.3579118	0.1235518	0.09532012	0.118569	0.1384395
1644.5	14	0.390197	0.3597995	0.1240356	0.09440807	0.116115	0.1361219
1644.6	14	0.390764	0.3559955	0.1242227	0.09047288	0.114805	0.1342802
1644.7	14	0.381434	0.3565967	0.1268849	0.08844703	0.114939	0.1342485
1644.8	14	0.406031	0.3491805	0.1294452	0.08787777	0.113027	0.1339004
1644.9	14	0.404668	0.3518909	0.1297961	0.08655543	0.112221	0.1345799
1645	14	0.407074	0.3542472	0.1306126	0.08698567	0.113073	0.1346566
1645.1	14	0.431673	0.3478203	0.1317643	0.08784274	0.114277	0.1337792
1645.2	14	0.430623	0.3507056	0.1335298	0.08917744	0.114732	0.1346307
1645.3	14	0.421886	0.3559071	0.1330549	0.09034286	0.113181	0.138889
1645.4	14	0.41858	0.3519796	0.1340012	0.09426248	0.111463	0.1381452
1645.5	14	0.431566	0.3564915	0.1346042	0.09407974	0.110126	0.1391093
1645.6	14	0.39683	0.3554544	0.1366867	0.09527526	0.111273	0.1357239
1645.7	14	0.42378	0.3614869	0.1331232	0.09293175	0.111596	0.1356306
1645.8	14	0.416338	0.351319	0.1339933	0.09368368	0.112423	0.1354743
1645.9	14	0.375121	0.3501593	0.132211	0.09334876	0.109676	0.1370925
1646	14	0.368362	0.3529095	0.1308005	0.09504583	0.110207	0.1346169
1646.1	14	0.39297	0.3560184	0.1298361	0.09465736	0.1095	0.1311108
1646.2	14	0.387402	0.349958	0.129658	0.09420168	0.108163	0.130399
1646.3	14	0.382981	0.3468042	0.1270804	0.09592858	0.112363	0.1300978
1646.4	14	0.383812	0.3470117	0.1277626	0.09868194	0.116178	0.1314785
1646.5	14	0.38814	0.3388982	0.1293887	0.1025938	0.118104	0.133957
1646.6	14	0.379087	0.3525902	0.1270023	0.1023288	0.116424	0.1349941
1646.7	14	0.391114	0.3489187	0.1282559	0.09854429	0.116997	0.1354743
1646.8	14	0.3951	0.3327584	0.1273268	0.09979876	0.119872	0.1353948
1646.9	14	0.387554	0.3235159	0.1278254	0.0962088	0.124631	0.1364216
1647	14	0.401479	0.3473336	0.1282701	0.09794572	0.126642	0.137596
1647.1	14	0.40471	0.3406522	0.1301937	0.09717852	0.126427	0.136942
1647.2	14	0.392131	0.3392713	0.130618	0.09594499	0.126018	0.1353865
1647.3	14	0.392323	0.3376324	0.1321274	0.09657389	0.123009	0.1345971
1647.4	14	0.394444	0.3391108	0.1314494	0.09683634	0.122047	0.1340783
1647.5	14	0.401354	0.3437627	0.1287656	0.09731642	0.123772	0.1319284
1647.6	14	0.400895	0.3504915	0.1285476	0.09542375	0.121382	0.1327134
1647.7	14	0.391119	0.3478595	0.1278622	0.09599578	0.11919	0.134942
1647.8	14	0.39543	0.3561576	0.1293205	0.09244741	0.120544	0.1375328
1647.9	14	0.389602	0.3461773	0.1304248	0.09241147	0.123718	0.1355144
1648	14	0.378989	0.3436954	0.1315212	0.09412599	0.124845	0.1366054
1648.1	14	0.39252	0.3472261	0.1336881	0.09309887	0.123917	0.1342183
1648.2	14	0.393417	0.3420123	0.1370324	0.09258316	0.121269	0.1366372
1648.3	14	0.386937	0.3291613	0.1358629	0.09365554	0.118816	0.1376511
1648.4	14	0.370044	0.3343008	0.1343085	0.09088273	0.116956	0.1407909
1648.5	14	0.381193	0.3346073	0.1312231	0.09106688	0.116562	0.141749
1648.6	14	0.378029	0.3419214	0.1310647	0.09108267	0.116204	0.1421394
1648.7	14	0.378135	0.3426766	0.1318685	0.09341208	0.116539	0.1414736
1648.8	14	0.389233	0.3445116	0.1286972	0.0920259	0.11717	0.1456723
1648.9	14	0.378204	0.3415817	0.127023	0.08974369	0.115869	0.1453827
1649	14	0.375068	0.3419833	0.1275177	0.08785454	0.115097	0.1443605
1649.1	14	0.397665	0.3429175	0.1317598	0.08739945	0.114579	0.1441057
1649.2	14	0.403979	0.344557	0.1277324	0.08939371	0.112502	0.1415141
1649.3	14	0.410557	0.3456241	0.1266427	0.09243307	0.11201	0.1393683
1649.4	14	0.419322	0.3497345	0.1280307	0.08894376	0.11253	0.1394063
1649.5	14	0.417627	0.3563057	0.1298922	0.08890309	0.1124	0.1384662
1649.6	14	0.420043	0.3477745	0.1306496	0.08997948	0.112241	0.1380839
1649.7	14	0.424222	0.3505174	0.1302032	0.09277828	0.112375	0.1371403
1649.8	14	0.431191	0.3458945	0.1291161	0.09243309	0.104995	0.1375451
1649.9	14	0.405177	0.3462104	0.1297056	0.09341007	0.105142	0.1367601
1650	14	0.423492	0.3567232	0.1307357	0.09314921	0.106124	0.1361184
1650.1	14	0.407876	0.3531021	0.1306672	0.09184711	0.108925	0.1378369
1650.2	14	0.373136	0.3565387	0.1303713	0.09199383	0.110143	0.1355933
1650.3	14	0.365576	0.358809	0.1277648	0.09519527	0.110225	0.1357528
1650.4	14	0.390997	0.358642	0.1286077	0.09297174	0.111902	0.133643
1650.5	14	0.382183	0.3581318	0.1282203	0.09457217	0.113579	0.1345034
1650.6	14	0.38662	0.3594875	0.1312599	0.1003629	0.116109	0.1345678
1650.7	14	0.380511	0.3509668	0.1283727	0.1008675	0.11917	0.133995
1650.8	14	0.387879	0.3627315	0.1273309	0.1000636	0.119874	0.1366682
1650.9	14	0.379419	0.3640398	0.1285846	0.100073	0.12136	0.1351026
1651	14	0.392552	0.3635342	0.1263768	0.1023265	0.122654	0.1356165
1651.1	14	0.393858	0.3723892	0.1239695	0.1016689	0.119344	0.1370607
1651.2	14	0.384044	0.3659867	0.1234567	0.09931872	0.121545	0.1371329
1651.3	14	0.395775	0.3650073	0.1256343	0.1011513	0.125448	0.1355772

1651.4	14	0.395228	0.3597835	0.1235343	0.09647679	0.124008	0.1360506
1651.5	14	0.389518	0.3618249	0.1253788	0.0940061	0.122887	0.1384576
1651.6	14	0.390199	0.3535045	0.1230006	0.09383024	0.120911	0.13809
1651.7	14	0.386586	0.3519277	0.1235151	0.09435552	0.121167	0.1379087
1651.8	14	0.390615	0.3568353	0.1257708	0.09534798	0.119972	0.1372116
1651.9	14	0.385809	0.3525219	0.1271624	0.09647214	0.120468	0.1364143
1652	14	0.378894	0.3635024	0.128473	0.0974239	0.121618	0.1357156
1652.1	14	0.375922	0.3645653	0.1295039	0.09702279	0.122141	0.1346741
1652.2	14	0.374772	0.3656228	0.1262513	0.09498432	0.12099	0.1359631
1652.3	14	0.370568	0.3562224	0.1262412	0.09580223	0.118888	0.1356919
1652.4	14	0.378964	0.357381	0.1295094	0.09657744	0.117198	0.1364859
1652.5	14	0.3755	0.3629521	0.1298816	0.09734935	0.118627	0.1372735
1652.6	14	0.384796	0.3654141	0.1285148	0.09744134	0.119518	0.1372273
1652.7	14	0.370898	0.3620057	0.1325731	0.09553593	0.119327	0.1384082
1652.8	14	0.375616	0.3566368	0.1357269	0.09457186	0.120505	0.1394
1652.9	14	0.376523	0.357987	0.137256	0.09180126	0.115978	0.1383819
1653	14	0.376986	0.3655078	0.1369269	0.09473369	0.114433	0.1393338
1653.1	14	0.392214	0.3529306	0.1383312	0.09290107	0.114874	0.141041
1653.2	14	0.392647	0.3555808	0.1361191	0.09204558	0.118485	0.1414126
1653.3	14	0.393337	0.3492481	0.1362047	0.09084843	0.11573	0.1432589
1653.4	14	0.411052	0.3445563	0.1337153	0.08869687	0.111437	0.143888
1653.5	14	0.428999	0.3425601	0.1311794	0.08915765	0.11316	0.1411278
1653.6	14	0.445513	0.3446167	0.1279568	0.08973244	0.111089	0.1412382
1653.7	14	0.458649	0.3479075	0.127274	0.08969737	0.109404	0.1419345
1653.8	14	0.452235	0.3468326	0.1265668	0.0900462	0.109055	0.1399059
1653.9	14	0.450829	0.3501297	0.1268399	0.09160119	0.11048	0.1400999
1654	14	0.430683	0.3508151	0.1264564	0.09300402	0.109384	0.1420669
1654.1	14	0.414389	0.3542618	0.1263435	0.09172817	0.111087	0.1365103
1654.2	14	0.405956	0.3537062	0.1245352	0.09271944	0.110265	0.1357026
1654.3	14	0.407987	0.3550739	0.1271478	0.09269171	0.107488	0.1360516
1654.4	14	0.396223	0.3536726	0.1286862	0.09272034	0.108803	0.1365424
1654.5	14	0.392466	0.3504232	0.1258618	0.09341785	0.109451	0.1345807
1654.6	14	0.390615	0.3625051	0.126852	0.09371747	0.107464	0.1352852
1654.7	14	0.396471	0.3494276	0.1303088	0.09322575	0.110186	0.1341126
1654.8	14	0.377583	0.3589426	0.132055	0.093771	0.112363	0.1355099
1654.9	14	0.38468	0.3581606	0.130794	0.09437995	0.116653	0.1377025
1655	14	0.384492	0.3588804	0.1321822	0.09687087	0.118259	0.1371217
1655.1	14	0.392052	0.3633506	0.1325705	0.09825929	0.121334	0.1367778
1655.2	14	0.379301	0.3617581	0.13234	0.09513838	0.125104	0.1371954
1655.3	14	0.39217	0.3588534	0.1297367	0.09753338	0.122562	0.1385871
1655.4	14	0.393935	0.3569898	0.1313585	0.1003692	0.122549	0.13963
1655.5	14	0.388132	0.3517479	0.1296754	0.1013549	0.129064	0.1394746
1655.6	14	0.392315	0.3502065	0.1283285	0.1002268	0.132347	0.1361057
1655.7	14	0.398273	0.3545758	0.1285776	0.09957867	0.128686	0.1338672
1655.8	14	0.387611	0.3627861	0.1267007	0.0980747	0.127287	0.1374226
1655.9	14	0.391259	0.3698857	0.1249313	0.09566691	0.122662	0.1370991
1656	14	0.390592	0.3698978	0.1255703	0.09409981	0.123933	0.1358411
1656.1	14	0.392146	0.3651534	0.1250301	0.09231038	0.121752	0.1377269
1656.2	14	0.382007	0.3533031	0.122091	0.09258825	0.123557	0.1383862
1656.3	14	0.382592	0.3527584	0.122587	0.09402405	0.124479	0.1377299
1656.4	14	0.376779	0.3602746	0.119612	0.0941581	0.125738	0.137749
1656.5	14	0.374522	0.3616401	0.1187132	0.09356868	0.122493	0.1373992
1656.6	14	0.374006	0.3580476	0.1205029	0.09094779	0.121623	0.1373671
1656.7	14	0.382682	0.3564074	0.1233077	0.09145181	0.117072	0.1359192
1656.8	14	0.381449	0.3556442	0.1251978	0.09161089	0.119602	0.1363189
1656.9	14	0.38141	0.3518945	0.1265282	0.09077628	0.119298	0.1361983
1657	14	0.375396	0.3547814	0.125669	0.08993968	0.116721	0.1356962
1657.1	14	0.387895	0.3508799	0.1265908	0.09011012	0.116758	0.1329493
1657.2	14	0.389229	0.3529556	0.1300472	0.09139128	0.114124	0.1337032
1657.3	14	0.393624	0.3608143	0.1336227	0.09193255	0.114536	0.1332005
1657.4	14	0.390669	0.3571765	0.1345124	0.09124564	0.115677	0.1338022
1657.5	14	0.381743	0.3559467	0.1332864	0.0910162	0.114331	0.1336946
1657.6	14	0.386971	0.3607685	0.1310532	0.08842474	0.111703	0.136475
1657.7	14	0.400925	0.3581346	0.1341632	0.08681631	0.110422	0.1362571
1657.8	14	0.403808	0.3657247	0.138128	0.08810179	0.111635	0.1352827
1657.9	14	0.407873	0.3617998	0.1369035	0.08795628	0.111177	0.13995
1658	14	0.420987	0.363895	0.1356992	0.08796265	0.11106	0.1426978
1658.1	14	0.405755	0.3561504	0.1341416	0.08957876	0.108649	0.1446785
1658.2	14	0.416214	0.353733	0.1333768	0.09049466	0.108615	0.1451402
1658.3	14	0.418504	0.3483095	0.1301801	0.08927795	0.111242	0.1427573
1658.4	14	0.418902	0.3525346	0.1282634	0.09059367	0.113317	0.1430647
1658.5	14	0.421913	0.3548713	0.1281945	0.08913442	0.112186	0.1418263
1658.6	14	0.411666	0.350398	0.1265951	0.08786399	0.110219	0.1419195
1658.7	14	0.419567	0.3524146	0.1277271	0.08644734	0.110763	0.1415204
1658.8	14	0.381213	0.3515146	0.1258343	0.08684632	0.112143	0.1403242
1658.9	14	0.379677	0.35627	0.1240203	0.0876704	0.111324	0.1372579
1659	14	0.393464	0.3545294	0.1235471	0.08807284	0.112904	0.1376598
1659.1	14	0.390389	0.3608802	0.1250914	0.09025242	0.113824	0.1365889
1659.2	14	0.390642	0.3525587	0.128463	0.09230867	0.118348	0.1357879
1659.3	14	0.381867	0.3505264	0.1286694	0.09424185	0.116899	0.1372729
1659.4	14	0.381926	0.3536381	0.1307152	0.09476365	0.117591	0.1401947
1659.5	14	0.376655	0.3521336	0.1343148	0.09607783	0.121432	0.138298
1659.6	14	0.391522	0.3369528	0.1357592	0.09781837	0.126047	0.1370281
1659.7	14	0.387109	0.344525	0.1358858	0.09413521	0.126392	0.1372631
1659.8	14	0.394102	0.3435719	0.1340173	0.09084859	0.127461	0.1369421
1659.9	14	0.396627	0.3561833	0.1344116	0.0937998	0.129482	0.1369276
1660	14	0.401227	0.3507487	0.1342022	0.09953574	0.126708	0.135371
1660.1	14	0.390551	0.3192282	0.1323428	0.09572031	0.12529	0.1353565
1660.2	14	0.394057	0.3410493	0.1341212	0.09391277	0.121508	0.1341051
1660.3	14	0.392382	0.3413489	0.1315859	0.09115694	0.121703	0.1358081
1660.4	14	0.395145	0.3373518	0.1302298	0.09306493	0.120908	0.1346828
1660.5	14	0.379525	0.3399782	0.1290769	0.09104154	0.122194	0.13399
1660.6	14	0.382973	0.3397824	0.126129	0.09241129	0.124135	0.1328946
1660.7	14	0.378096	0.3460646	0.1256177	0.09252852	0.122329	0.1334072
1660.8	14	0.376178	0.3542533	0.1266171	0.09172662	0.120253	0.1346611

1660.9	14	0.377904	0.3574397	0.1272224	0.09107874	0.120905	0.1347501
1661	14	0.382892	0.3467882	0.1252655	0.09099738	0.119579	0.1330058
1661.1	14	0.382547	0.3430807	0.1237359	0.08903754	0.121028	0.1370588
1661.2	14	0.377812	0.3484343	0.1218671	0.08874811	0.117843	0.1356615
1661.3	14	0.378536	0.3491153	0.1242814	0.09137101	0.118974	0.1347908
1661.4	14	0.39651	0.3429343	0.1270721	0.09076181	0.115568	0.1345951
1661.5	14	0.400903	0.3463574	0.127606	0.0891327	0.114616	0.133969
1661.6	14	0.408902	0.3386294	0.1286137	0.09055098	0.111919	0.13498
1661.7	14	0.409253	0.3375388	0.1278601	0.08841244	0.112557	0.1332535
1661.8	14	0.399326	0.3417098	0.1257946	0.08854759	0.112409	0.1312147
1661.9	14	0.398195	0.3490189	0.1223865	0.08830777	0.110394	0.1289694
1662	14	0.408694	0.3441972	0.1260551	0.08749829	0.109907	0.130978
1662.1	14	0.409256	0.3473484	0.1266802	0.08884625	0.111984	0.1307959
1662.2	14	0.406737	0.3457964	0.1287564	0.08958953	0.11101	0.1300894
1662.3	14	0.413787	0.3444443	0.1298241	0.09036335	0.109501	0.1310303
1662.4	14	0.404641	0.3438232	0.129531	0.09198095	0.106687	0.1331739
1662.5	14	0.42587	0.3500657	0.1335679	0.09076876	0.10596	0.1334579
1662.6	14	0.433914	0.3498157	0.1383986	0.09017288	0.107214	0.1323605
1662.7	14	0.440814	0.3474981	0.1392259	0.09018187	0.108688	0.1352919
1662.8	14	0.450947	0.3667362	0.135817	0.0893888	0.112315	0.138527
1662.9	14	0.452834	0.3519233	0.135509	0.09034654	0.112929	0.1401957
1663	14	0.427914	0.3540598	0.1359978	0.09193536	0.112954	0.1402336
1663.1	14	0.370008	0.3497378	0.1337154	0.09088849	0.111817	0.1389191
1663.2	14	0.38487	0.3498761	0.1314361	0.09112702	0.103974	0.1361343
1663.3	14	0.392507	0.3623505	0.1282031	0.09010785	0.10408	0.1382108
1663.4	14	0.392474	0.3559848	0.1276432	0.09232579	0.108577	0.1374978
1663.5	14	0.391284	0.3602037	0.1272328	0.09623638	0.111234	0.1369031
1663.6	14	0.384237	0.3624526	0.126526	0.09696691	0.112335	0.1358517
1663.7	14	0.38738	0.3613485	0.1274682	0.09657321	0.114522	0.1322133
1663.8	14	0.381387	0.3608513	0.1259502	0.09487069	0.119661	0.131914
1663.9	14	0.394541	0.3668629	0.1294653	0.09583794	0.124634	0.1329409
1664	14	0.386313	0.3547805	0.1312346	0.09476846	0.122217	0.1313256
1664.1	14	0.390408	0.368317	0.1292832	0.09320799	0.127369	0.1307131
1664.2	14	0.397977	0.3698364	0.1310031	0.0921839	0.129478	0.1314356
1664.3	14	0.403508	0.3693151	0.1319767	0.09443892	0.126238	0.1295038
1664.4	14	0.395866	0.3798268	0.1329132	0.09250285	0.126082	0.1329776
1664.5	14	0.405128	0.3715239	0.1347185	0.09231192	0.123867	0.1354597
1664.6	14	0.399353	0.3706836	0.1327057	0.092074	0.122395	0.1324933
1664.7	14	0.401838	0.362979	0.131862	0.09150722	0.122511	0.1329814
1664.8	14	0.380859	0.3683085	0.1328582	0.09089057	0.123019	0.1318265
1664.9	14	0.3847	0.3603704	0.1317145	0.08965939	0.123443	0.1324072
1665	14	0.380676	0.35897	0.1319274	0.09043119	0.120536	0.1355428
1665.1	14	0.377682	0.3617454	0.1289239	0.09120466	0.118745	0.1362606
1665.2	14	0.386378	0.3589299	0.1270508	0.09008941	0.116412	0.1365575
1665.3	14	0.386509	0.3617364	0.127022	0.09161636	0.116978	0.1371099
1665.4	14	0.384039	0.3625332	0.1243444	0.09142927	0.119144	0.1386538
1665.5	14	0.376933	0.3679811	0.1241906	0.09196023	0.119596	0.1372066
1665.6	14	0.387096	0.3573652	0.1251352	0.09070563	0.1204	0.1368059
1665.7	14	0.383656	0.3574971	0.1241081	0.09002659	0.118012	0.1363489
1665.8	14	0.377428	0.364048	0.123076	0.09205006	0.115185	0.1364163
1665.9	14	0.387694	0.3629392	0.1224151	0.0946701	0.112447	0.1352749
1666	14	0.388569	0.365326	0.1216792	0.09211423	0.113893	0.1329026
1666.1	14	0.383372	0.3594275	0.1214355	0.09229252	0.113979	0.1353554
1666.2	14	0.392544	0.3580783	0.1242702	0.088995	0.110836	0.1369408
1666.3	14	0.445612	0.365539	0.1263848	0.08743946	0.109308	0.1369328
1666.4	14	0.47456	0.3584054	0.12598	0.0895181	0.109685	0.1364579
1666.5	14	0.452199	0.3569251	0.1254931	0.09080069	0.111575	0.1332519
1666.6	14	0.457766	0.3553661	0.1241878	0.09363214	0.11236	0.1305468
1666.7	14	0.462681	0.3532882	0.1199321	0.09421462	0.111281	0.1294864
1666.8	14	0.476298	0.3465248	0.1214743	0.09423717	0.111249	0.1321145
1666.9	14	0.438099	0.3493927	0.1232702	0.09372358	0.110721	0.1312434
1667	14	0.409605	0.3468389	0.1293268	0.09356044	0.111107	0.1329815
1667.1	14	0.413775	0.3520211	0.1298954	0.09284715	0.112235	0.1301432
1667.2	14	0.407951	0.3462734	0.1296401	0.09129433	0.110035	0.1298392
1667.3	14	0.414467	0.355695	0.1304496	0.09101623	0.108654	0.1304924
1667.4	14	0.403053	0.3598236	0.1336876	0.0929995	0.108776	0.1289493
1667.5	14	0.404174	0.3541531	0.1364442	0.09081046	0.109216	0.1313817
1667.6	14	0.402607	0.3582905	0.1367715	0.0908089	0.110608	0.1354242
1667.7	14	0.392274	0.3661368	0.1355156	0.09304046	0.112165	0.1359169
1667.8	14	0.391668	0.3512647	0.1340945	0.0941247	0.112985	0.1356105
1667.9	14	0.390235	0.36681	0.1330779	0.09557292	0.111958	0.1350177
1668	14	0.389937	0.362271	0.1290393	0.09743571	0.113264	0.1348185
1668.1	14	0.383455	0.36603	0.1285893	0.09411026	0.114817	0.1352869
1668.2	14	0.401797	0.3635449	0.1272005	0.09514968	0.118736	0.1374266
1668.3	14	0.388098	0.3652823	0.1290471	0.09544617	0.121045	0.1381117
1668.4	14	0.394793	0.3692701	0.1287739	0.09791485	0.124116	0.1382787
1668.5	14	0.398473	0.3697761	0.1300196	0.09583334	0.127502	0.1358232
1668.6	14	0.401076	0.3682934	0.12825	0.09389566	0.12662	0.1348279
1668.7	14	0.391265	0.3630412	0.1304096	0.09541298	0.125222	0.1347278
1668.8	14	0.394292	0.3544602	0.1328786	0.09348895	0.120238	0.1354918
1668.9	14	0.389807	0.3584292	0.132345	0.09267672	0.117815	0.1369438
1669	14	0.392306	0.3594871	0.1310368	0.08951969	0.11858	0.1389418
1669.1	14	0.37845	0.3681672	0.1322691	0.09011842	0.121589	0.1373421
1669.2	14	0.385018	0.3748944	0.1344604	0.08917597	0.123355	0.1366675
1669.3	14	0.379662	0.3752231	0.1363859	0.08920372	0.121759	0.1359964
1669.4	14	0.374372	0.3726253	0.1355985	0.0903883	0.121241	0.1345957
1669.5	14	0.379691	0.3574131	0.1344577	0.09046199	0.122225	0.1350579
1669.6	14	0.382937	0.3537897	0.1341354	0.08914693	0.123276	0.1355802
1669.7	14	0.382929	0.3641587	0.133637	0.0904073	0.123309	0.135624
1669.8	14	0.376673	0.3647996	0.1336156	0.09169128	0.122949	0.1354829
1669.9	14	0.413618	0.3646581	0.130152	0.09093137	0.123211	0.1361122
1670	14	0.480517	0.3593973	0.1280337	0.09031586	0.120851	0.135897
1670.1	14	0.490746	0.3583311	0.1287172	0.08837473	0.118151	0.1357028
1670.2	14	0.458451	0.3613878	0.128271	0.08914465	0.115361	0.136603
1670.3	14	0.489776	0.3546332	0.1273865	0.08725388	0.115998	0.1361516

1670.4	14	0.495702	0.3549093	0.1281758	0.08825736	0.110481	0.1371126
1670.5	14	0.493426	0.35509	0.1302754	0.08714882	0.1106	0.137812
1670.6	14	0.500391	0.3587897	0.1284813	0.08899734	0.111512	0.1396665
1670.7	14	0.462075	0.3575324	0.1286922	0.09048994	0.11189	0.1403253
1670.8	14	0.437063	0.3571587	0.1266505	0.08980186	0.114325	0.1378583
1670.9	14	0.441652	0.357725	0.126961	0.09261104	0.113382	0.1378085
1671	14	0.411578	0.3557237	0.1278082	0.09328713	0.112757	0.1377196
1671.1	14	0.40871	0.36153	0.1302349	0.09346935	0.11123	0.1361388
1671.2	14	0.403962	0.3650338	0.1317133	0.09313586	0.113153	0.1365295
1671.3	14	0.389745	0.3655591	0.1302538	0.09273748	0.113622	0.1366696
1671.4	14	0.403525	0.3605145	0.129463	0.08968948	0.112451	0.1360992
1671.5	14	0.403776	0.359274	0.1287099	0.08845796	0.112496	0.1352036
1671.6	14	0.376085	0.3499489	0.130255	0.08808409	0.115181	0.1351987
1671.7	14	0.36819	0.3517991	0.1283425	0.08961715	0.115506	0.1330176
1671.8	14	0.390337	0.3551262	0.1299159	0.09017128	0.117199	0.133151
1671.9	14	0.388183	0.3458713	0.1324769	0.08873123	0.117569	0.1331863
1672	14	0.397272	0.3513469	0.1340809	0.08959018	0.120086	0.1339404
1672.1	14	0.391875	0.3541037	0.1368936	0.08823729	0.123981	0.1345256
1672.2	14	0.392474	0.352282	0.1379471	0.09198764	0.124692	0.1333624
1672.3	14	0.37971	0.3507474	0.1388005	0.09341978	0.125216	0.1349206
1672.4	14	0.392902	0.361399	0.1361394	0.09514093	0.124031	0.1371545
1672.5	14	0.398626	0.3548298	0.1359898	0.09521287	0.123239	0.1390083
1672.6	14	0.385699	0.347765	0.136225	0.09398472	0.125752	0.141423
1672.7	14	0.397263	0.348944	0.1345018	0.09422923	0.128361	0.1413341
1672.8	14	0.400589	0.3510886	0.1309977	0.09182499	0.125197	0.1397273
1672.9	14	0.400925	0.3414127	0.1278553	0.08990777	0.122009	0.1404296
1673	14	0.391186	0.3477419	0.1279609	0.0880854	0.122292	0.1396008
1673.1	14	0.395605	0.3422302	0.1280218	0.08970442	0.120518	0.1400396
1673.2	14	0.392745	0.3549419	0.127497	0.09169576	0.118228	0.1393339
1673.3	14	0.386432	0.3581519	0.1260013	0.09156276	0.119899	0.137157
1673.4	14	0.384444	0.3370809	0.1277491	0.09167878	0.123597	0.1352338
1673.5	14	0.385104	0.3261581	0.1271667	0.08956015	0.123487	0.1348658
1673.6	14	0.376326	0.3451486	0.129626	0.08748125	0.121984	0.1344679
1673.7	14	0.37654	0.3424929	0.1285357	0.08580396	0.119877	0.1367488
1673.8	14	0.382466	0.3477414	0.1282994	0.08742665	0.118473	0.1412696
1673.9	14	0.3788	0.3414673	0.128162	0.08801373	0.119599	0.1393421
1674	14	0.385609	0.3424974	0.1289902	0.0886034	0.119521	0.1404987
1674.1	14	0.369624	0.3490192	0.1295508	0.0904019	0.118885	0.1428063
1674.2	14	0.376691	0.3505692	0.1290349	0.09134278	0.11955	0.1411703
1674.3	14	0.379792	0.3520473	0.128461	0.08863281	0.117507	0.1414486
1674.4	14	0.372223	0.345986	0.1307592	0.08778016	0.115868	0.1401487
1674.5	14	0.383269	0.3480206	0.1305651	0.08776005	0.117145	0.139568
1674.6	14	0.390642	0.354393	0.1332959	0.08740832	0.117742	0.1399779
1674.7	14	0.379312	0.3509233	0.1315205	0.0894639	0.114013	0.1418404
1674.8	14	0.398435	0.3529373	0.1294669	0.08882243	0.112683	0.1381755
1674.9	14	0.421428	0.3576917	0.1313013	0.08632074	0.115199	0.1368967
1675	14	0.429566	0.3447763	0.1307275	0.08642264	0.114676	0.1394282
1675.1	14	0.424668	0.3479562	0.1281008	0.08530196	0.114354	0.1391445
1675.2	14	0.440633	0.3486101	0.127914	0.08712047	0.112095	0.1388333
1675.3	14	0.407031	0.3453634	0.1288208	0.08779082	0.109734	0.1366848
1675.4	14	0.418431	0.3416871	0.125477	0.08576095	0.108647	0.137193
1675.5	14	0.414399	0.3480068	0.1264992	0.08573856	0.109028	0.1377482
1675.6	14	0.396939	0.343205	0.1239466	0.08749288	0.112919	0.1365845
1675.7	14	0.407041	0.3480244	0.1270181	0.08694051	0.112336	0.13851
1675.8	14	0.401778	0.3535187	0.1300874	0.0886387	0.112092	0.1376217
1675.9	14	0.373378	0.3507628	0.1337836	0.09009928	0.112375	0.1391471
1676	14	0.372556	0.3433783	0.1324101	0.089603	0.114728	0.1387741
1676.1	14	0.381693	0.351466	0.1327271	0.08784267	0.115627	0.1384822
1676.2	14	0.385406	0.3514293	0.1320962	0.08750134	0.117782	0.1389875
1676.3	14	0.396272	0.3471188	0.1306923	0.08755241	0.118471	0.1363193
1676.4	14	0.38513	0.3556153	0.129996	0.08993243	0.12025	0.136416
1676.5	14	0.386158	0.3454304	0.130885	0.09104647	0.122599	0.1364682
1676.6	14	0.378432	0.3513492	0.1294646	0.09260047	0.123501	0.1387504
1676.7	14	0.387688	0.3605821	0.1303391	0.09282043	0.125411	0.1383124
1676.8	14	0.393174	0.3538254	0.1348934	0.09504097	0.126128	0.1351162
1676.9	14	0.388394	0.36402	0.1356851	0.1005616	0.12703	0.1332802
1677	14	0.391059	0.3693575	0.1380922	0.09899481	0.127321	0.1337439
1677.1	14	0.397607	0.3696857	0.1374764	0.09671502	0.125365	0.1356306
1677.2	14	0.392956	0.3720099	0.1348345	0.09101592	0.121584	0.1359038
1677.3	14	0.392217	0.3698902	0.1323708	0.08982499	0.117471	0.139032
1677.4	14	0.392894	0.3624825	0.1305041	0.08884867	0.118888	0.1395895
1677.5	14	0.389448	0.3681086	0.1312709	0.08884237	0.118579	0.1386329
1677.6	14	0.386588	0.3710669	0.1284578	0.08893365	0.120639	0.1366887
1677.7	14	0.379435	0.3694521	0.1264684	0.08871675	0.122598	0.1364909
1677.8	14	0.383925	0.3724208	0.1291091	0.0910819	0.123066	0.1362476
1677.9	14	0.379454	0.3659202	0.1282659	0.09301496	0.123165	0.1388958
1678	14	0.384581	0.3671384	0.1264859	0.08978702	0.122496	0.1397074
1678.1	14	0.383162	0.365187	0.1262625	0.09183268	0.116916	0.1373788
1678.2	14	0.370993	0.3638734	0.1247969	0.0922978	0.117376	0.1366775
1678.3	14	0.376586	0.3604882	0.1262154	0.08967855	0.11848	0.137634
1678.4	14	0.376018	0.35821	0.1273067	0.08891213	0.117425	0.138218
1678.5	14	0.377322	0.358445	0.1272136	0.0860435	0.11764	0.1368525
1678.6	14	0.403655	0.3592158	0.1253087	0.08552293	0.117101	0.1364328
1678.768	14	0.395956	0.3641621	0.1266831	0.08484647	0.11325	0.1350944
1678.868	14.5	0.395181	0.3672014	0.1256904	0.08399205	0.114468	0.1370686
1678.968	14.5	0.395789	0.3629399	0.1272751	0.08322813	0.116578	0.1383871
1679.068	14.5	0.404891	0.3591402	0.1284235	0.08453614	0.113769	0.1355808
1679.168	14.5	0.405345	0.3636395	0.128452	0.084077	0.112137	0.1358196
1679.268	14.5	0.413808	0.3651595	0.1308109	0.08352789	0.110047	0.1347207
1679.368	14.5	0.399867	0.3690782	0.1337703	0.08636232	0.109004	0.1364914
1679.468	14.5	0.409142	0.363374	0.1344208	0.08688119	0.110581	0.1373373
1679.568	14.5	0.404723	0.3631924	0.130121	0.08570809	0.109703	0.1379934
1679.668	14.5	0.394692	0.3627729	0.1286798	0.08529349	0.110942	0.1364107
1679.768	14.5	0.394664	0.3637358	0.1309406	0.08471263	0.110958	0.1365078
1679.868	14.5	0.402152	0.3535039	0.1286291	0.08725081	0.109318	0.1378685

1679.968	14.5	0.384125	0.3547927	0.126159	0.08795071	0.110354	0.13672
1680.068	14.5	0.371253	0.3522146	0.1266429	0.08841284	0.107319	0.1368727
1680.168	14.5	0.387925	0.3512601	0.1250979	0.08954327	0.107493	0.137067
1680.268	14.5	0.391248	0.3481678	0.1256885	0.09114012	0.107483	0.1375726
1680.368	14.5	0.395743	0.3532209	0.1256013	0.09199583	0.107647	0.1362248
1680.468	14.5	0.386697	0.3569247	0.1258505	0.09062576	0.107858	0.1351672
1680.568	14.5	0.387501	0.3515066	0.123716	0.09358476	0.107912	0.1354672
1680.668	14.5	0.381142	0.3582198	0.1250543	0.0930849	0.110413	0.1343096
1680.768	14.5	0.387597	0.3590285	0.126837	0.09208207	0.109325	0.1346435
1680.868	14.5	0.394664	0.356177	0.1263428	0.0960903	0.111548	0.134927
1680.968	14.5	0.392379	0.3613335	0.1267624	0.1006961	0.115429	0.1333019
1681.068	14.5	0.391482	0.3635475	0.1261001	0.1015843	0.120079	0.1350304
1681.168	14.5	0.401209	0.3587201	0.1289356	0.09744832	0.118202	0.1338764
1681.268	14.5	0.392309	0.3650578	0.128907	0.09446663	0.123993	0.1343236
1681.368	14.5	0.397856	0.3720287	0.130749	0.09551608	0.127833	0.1331663
1681.468	14.5	0.391566	0.3669493	0.1314207	0.09436247	0.128892	0.134808
1681.568	14.5	0.395137	0.3687815	0.1316637	0.09561366	0.127411	0.1345747
1681.668	14.5	0.383163	0.369727	0.1330992	0.09620558	0.123806	0.1325263
1681.768	14.5	0.381861	0.3712546	0.1292894	0.09423397	0.119726	0.1317114
1681.868	14.5	0.382562	0.3730535	0.1277649	0.09266155	0.120876	0.1322926
1681.968	14.5	0.380247	0.3717189	0.1263014	0.09163869	0.124416	0.1354573
1682.068	14.5	0.3822	0.3677314	0.126657	0.09095547	0.124365	0.1360163
1682.168	14.5	0.385999	0.3630659	0.1260318	0.09050696	0.123881	0.1360422
1682.268	14.5	0.382989	0.3612712	0.1221157	0.09096998	0.122352	0.1367778
1682.368	14.5	0.377705	0.3619131	0.1242513	0.09038211	0.121313	0.1372719
1682.468	14.5	0.373513	0.3658271	0.122234	0.09055716	0.120573	0.1393917
1682.568	14.5	0.372703	0.3726669	0.1202241	0.08959839	0.120486	0.1397916
1682.668	14.5	0.375653	0.3807952	0.1205499	0.08885363	0.12047	0.1410475
1682.768	14.5	0.386513	0.3773295	0.1229293	0.09017424	0.118521	0.1417985
1682.868	14.5	0.382562	0.3678326	0.1240972	0.08903953	0.118177	0.1457098
1682.968	14.5	0.383172	0.3550119	0.1244993	0.0907243	0.117441	0.1437805
1683.068	14.5	0.397924	0.3576753	0.1257441	0.088477	0.117366	0.1424014
1683.168	14.5	0.404419	0.3665448	0.1264527	0.08814391	0.116661	0.1398787
1683.268	14.5	0.413915	0.3687129	0.1245331	0.09133274	0.114672	0.1388031
1683.368	14.5	0.410059	0.3606505	0.1244146	0.09054886	0.112626	0.1400588
1683.468	14.5	0.405125	0.3576598	0.1231908	0.09013505	0.110528	0.1410599
1683.568	14.5	0.396572	0.3608079	0.1265296	0.09182173	0.110807	0.140407
1683.668	14.5	0.414441	0.3573371	0.1289619	0.09103571	0.110802	0.1403606
1683.768	14.5	0.409532	0.3566988	0.1288074	0.09000407	0.110577	0.1405848
1683.868	14.5	0.395315	0.3543255	0.1286234	0.08839486	0.110293	0.1407125
1683.968	14.5	0.401549	0.3539655	0.1280225	0.08954526	0.109462	0.1395352
1684.068	14.5	0.39899	0.3607186	0.1297036	0.08830593	0.109312	0.1400342
1684.168	14.5	0.375524	0.3580483	0.1280313	0.09055911	0.109718	0.1387948
1684.268	14.5	0.389315	0.3556174	0.124748	0.09222043	0.110003	0.1373518
1684.368	14.5	0.388984	0.3612792	0.1267496	0.08881651	0.106499	0.1371053
1684.468	14.5	0.387797	0.3604904	0.1283911	0.08699398	0.108559	0.1377954
1684.568	14.5	0.391174	0.3663283	0.1271328	0.09059878	0.108339	0.1370568
1684.668	14.5	0.38302	0.3613076	0.1259913	0.09241032	0.107812	0.1363959
1684.768	14.5	0.384603	0.3650103	0.1242198	0.0944374	0.113186	0.137544
1684.868	14.5	0.384286	0.3593468	0.1242524	0.09336723	0.117952	0.134848
1684.968	14.5	0.38979	0.3563041	0.1228642	0.09222158	0.12039	0.1349645
1685.068	14.5	0.387433	0.3519785	0.1255153	0.09391749	0.117025	0.137157
1685.168	14.5	0.393288	0.3549813	0.1246335	0.09628136	0.117526	0.1371273
1685.268	14.5	0.387858	0.3553303	0.1247734	0.09664748	0.118154	0.1377422
1685.368	14.5	0.395863	0.3501359	0.1226299	0.09688731	0.121103	0.1370059
1685.468	14.5	0.393233	0.3532703	0.1204901	0.09595411	0.121814	0.136517
1685.568	14.5	0.397413	0.3528736	0.124238	0.09433741	0.125459	0.1356237
1685.668	14.5	0.391011	0.3566734	0.1256717	0.09495848	0.126302	0.1333628
1685.768	14.5	0.390897	0.356327	0.1289516	0.09749541	0.123814	0.1309102
1685.868	14.5	0.384836	0.3588932	0.1302786	0.0954095	0.123043	0.1302751
1685.968	14.5	0.381643	0.3532226	0.1306012	0.09056173	0.119827	0.1301842
1686.068	14.5	0.3769	0.3531713	0.1337515	0.09139642	0.11811	0.1321319
1686.168	14.5	0.380175	0.3542697	0.1349712	0.09107558	0.1181	0.1307766
1686.268	14.5	0.388442	0.3533418	0.1348421	0.09051377	0.122557	0.1291769
1686.368	14.5	0.380398	0.3420252	0.1316809	0.09107538	0.124689	0.1304831
1686.468	14.5	0.380228	0.3450615	0.1323198	0.09152998	0.1228	0.131503
1686.568	14.5	0.379943	0.3385557	0.1318314	0.08983289	0.120748	0.1308174
1686.668	14.5	0.384115	0.3500454	0.1297819	0.08869834	0.121905	0.1330965
1686.768	14.5	0.385783	0.3507202	0.1267843	0.08666762	0.120085	0.1350638
1686.868	14.5	0.390064	0.3207653	0.125338	0.08850032	0.119875	0.135242
1686.968	14.5	0.398587	0.3460515	0.1260601	0.08823035	0.118215	0.1369643
1687.068	14.5	0.394686	0.3513285	0.1219594	0.08769263	0.118235	0.1380795
1687.168	14.5	0.405817	0.3457433	0.1216264	0.08712191	0.116059	0.1349297
1687.268	14.5	0.410491	0.3472468	0.1233948	0.08426058	0.112189	0.1361084
1687.368	14.5	0.411009	0.3433801	0.1257729	0.08663372	0.111582	0.1378638
1687.468	14.5	0.450293	0.3461869	0.1264772	0.08730162	0.111198	0.1370609
1687.568	14.5	0.476755	0.3516925	0.124598	0.08664833	0.111056	0.1374642
1687.668	14.5	0.443579	0.3598135	0.1253842	0.08836098	0.109565	0.1383924
1687.768	14.5	0.444399	0.3487022	0.12717	0.08977418	0.10925	0.140112
1687.868	14.5	0.433202	0.3431333	0.1273793	0.09121761	0.110099	0.1400021
1687.968	14.5	0.431671	0.3484039	0.130293	0.08841481	0.10786	0.1414451
1688.068	14.5	0.445969	0.3531185	0.1285497	0.08479609	0.108598	0.141027
1688.168	14.5	0.443607	0.3519651	0.1291853	0.08608843	0.108969	0.1392399
1688.268	14.5	0.407208	0.3605169	0.1329765	0.08682104	0.109272	0.1385507
1688.368	14.5	0.379835	0.3498485	0.1346487	0.08946674	0.110843	0.1361208
1688.468	14.5	0.395584	0.3503736	0.1326503	0.08801582	0.11329	0.1385009
1688.568	14.5	0.38549	0.3579427	0.1287277	0.08601572	0.113306	0.1392427
1688.668	14.5	0.398934	0.3537148	0.1247832	0.08677569	0.111044	0.1393152
1688.768	14.5	0.381853	0.3564342	0.1245701	0.08969448	0.110292	0.1395659
1688.868	14.5	0.38693	0.3507393	0.124504	0.09096583	0.1111	0.140597
1688.968	14.5	0.386168	0.3539514	0.1263038	0.0928368	0.110824	0.1411238
1689.068	14.5	0.394195	0.343038	0.127629	0.0912367	0.116732	0.1401322
1689.168	14.5	0.393764	0.3458987	0.1265763	0.09257517	0.120325	0.1381154
1689.268	14.5	0.391072	0.3552032	0.1255243	0.09394657	0.122004	0.1363238
1689.368	14.5	0.38704	0.3459327	0.1234646	0.09542315	0.121323	0.1348453

1689.468	14.5	0.39904	0.3458235	0.1225614	0.09762233	0.121829	0.1344399
1689.568	14.5	0.391266	0.3634504	0.1251735	0.09502409	0.125303	0.1341387
1689.668	14.5	0.397784	0.3625087	0.129534	0.09401942	0.124531	0.1354764
1689.768	14.5	0.39388	0.3636867	0.1286727	0.09508903	0.125771	0.1344123
1689.868	14.5	0.394175	0.359419	0.1279955	0.09385365	0.128205	0.1343686
1689.968	14.5	0.383925	0.3520369	0.1245188	0.09239139	0.127192	0.1353637
1690.068	14.5	0.384355	0.3615412	0.1258485	0.09147894	0.122701	0.1345272
1690.168	14.5	0.381974	0.353567	0.1287447	0.08937901	0.123411	0.1345539
1690.268	14.5	0.383339	0.3624484	0.1274537	0.0879539	0.120324	0.1347259
1690.368	14.5	0.390215	0.370523	0.1307172	0.09049974	0.118593	0.1338172
1690.468	14.5	0.39315	0.3716597	0.1324492	0.09119991	0.119855	0.1332229
1690.568	14.5	0.3838	0.3703447	0.1348005	0.09322891	0.123443	0.131165
1690.668	14.5	0.37363	0.3740861	0.1357537	0.09234244	0.123332	0.1287344
1690.768	14.5	0.376455	0.3654553	0.1368807	0.09094211	0.120802	0.1292358
1690.868	14.5	0.369515	0.3704771	0.138333	0.09036653	0.117373	0.130803
1690.968	14.5	0.376088	0.3689124	0.1357704	0.09132449	0.120992	0.1288617
1691.068	14.5	0.385246	0.3673804	0.1342072	0.0907199	0.1196	0.1327041
1691.168	14.5	0.392112	0.3746449	0.1337775	0.08968326	0.116869	0.1311157
1691.268	14.5	0.394358	0.3708691	0.1326812	0.09063046	0.117519	0.1311721
1691.368	14.5	0.411474	0.3700734	0.130574	0.08904855	0.116759	0.1308813
1691.468	14.5	0.434266	0.3640102	0.1292734	0.0907665	0.117004	0.1291757
1691.568	14.5	0.45716	0.37272	0.1309436	0.090324	0.116551	0.1337249
1691.668	14.5	0.431349	0.3620368	0.1286341	0.09050819	0.118591	0.1360345
1691.768	14.5	0.41636	0.3566842	0.1287637	0.08978147	0.118031	0.1375273
1691.868	14.5	0.410346	0.3595075	0.1255369	0.09023538	0.11768	0.1396683
1691.968	14.5	0.417856	0.3573675	0.1278948	0.08934017	0.116036	0.1374083
1692.068	14.5	0.401335	0.3635485	0.128818	0.08773784	0.115612	0.1392828
1692.168	14.5	0.396825	0.3613331	0.1275566	0.08609273	0.114298	0.1385278
1692.268	14.5	0.397404	0.3624794	0.1255017	0.08540086	0.114059	0.1412582
1692.368	14.5	0.393454	0.357186	0.1264725	0.08782639	0.113664	0.1423623
1692.468	14.5	0.371207	0.3543435	0.1269152	0.08949561	0.115449	0.1408004
1692.568	14.5	0.38676	0.3627472	0.1279947	0.08825741	0.115365	0.1398353
1692.668	14.5	0.394497	0.3603525	0.1282721	0.08631494	0.116273	0.1367095
1692.768	14.5	0.392384	0.3602773	0.128711	0.08759002	0.115504	0.138685
1692.868	14.5	0.384033	0.3583638	0.130387	0.08716358	0.116508	0.1388986
1692.968	14.5	0.387694	0.3579834	0.1334759	0.09014975	0.11444	0.1376686
1693.068	14.5	0.382545	0.3634242	0.1320277	0.09319238	0.113615	0.139859
1693.168	14.5	0.395705	0.3538644	0.1287457	0.09187649	0.112583	0.138227
1693.268	14.5	0.3927	0.3561021	0.1287213	0.09417093	0.114031	0.1389309
1693.368	14.5	0.391279	0.3537129	0.1290239	0.09509466	0.117589	0.1390024
1693.468	14.5	0.387475	0.3504025	0.1253315	0.09626971	0.118729	0.1379354
1693.568	14.5	0.392868	0.3486537	0.1233844	0.09790055	0.118895	0.1372567
1693.668	14.5	0.393237	0.350839	0.1256037	0.09632211	0.11945	0.1357786
1693.768	14.5	0.399193	0.3543111	0.1231981	0.09498256	0.120202	0.1381543
1693.868	14.5	0.394633	0.3549629	0.124015	0.09198281	0.120483	0.1379457
1693.968	14.5	0.394837	0.3546416	0.1218399	0.09286046	0.119972	0.1373658
1694.068	14.5	0.38613	0.3595218	0.1181886	0.09262606	0.120098	0.1358458
1694.168	14.5	0.38319	0.3620465	0.1168933	0.09197643	0.122293	0.1350617
1694.268	14.5	0.379134	0.3610708	0.1195125	0.08996012	0.125029	0.1368495
1694.368	14.5	0.377022	0.3646863	0.1224485	0.08923896	0.121658	0.1379347
1694.468	14.5	0.380823	0.3668348	0.1263131	0.08943932	0.120998	0.1365698
1694.568	14.5	0.388516	0.3601625	0.1274432	0.0881917	0.118145	0.1367322
1694.668	14.5	0.383484	0.375654	0.1287394	0.0882443	0.117939	0.1347722
1694.768	14.5	0.373642	0.3639184	0.1315793	0.0916846	0.115846	0.1364482
1694.868	14.5	0.370413	0.3699617	0.1266605	0.09171844	0.118419	0.1358516
1694.968	14.5	0.373381	0.3640375	0.1325448	0.09291986	0.119976	0.1351355
1695.068	14.5	0.37753	0.3648608	0.134033	0.09081817	0.118359	0.1355876
1695.168	14.5	0.383655	0.3678185	0.1324598	0.09261653	0.11579	0.1350475
1695.268	14.5	0.38344	0.3683818	0.1343389	0.08940248	0.117096	0.1334926
1695.368	14.5	0.384901	0.3681912	0.1379742	0.08999629	0.116864	0.1318852
1695.468	14.5	0.398696	0.3657602	0.1404556	0.08951636	0.118578	0.1315857
1695.568	14.5	0.413324	0.3607357	0.1372395	0.09034609	0.118777	0.1294876
1695.668	14.5	0.410479	0.3601665	0.1356066	0.09044627	0.121579	0.1300873
1695.768	14.5	0.412223	0.363049	0.1322081	0.08959983	0.119705	0.131278
1695.868	14.5	0.418398	0.3702115	0.130742	0.09013686	0.117568	0.1329709
1695.968	14.5	0.399149	0.3778354	0.1274198	0.09230337	0.117836	0.1335979
1696.068	14.5	0.404961	0.3789054	0.1239085	0.09339593	0.117424	0.1330951
1696.168	14.5	0.39994	0.3716322	0.127793	0.09446678	0.113435	0.1339895
1696.268	14.5	0.396724	0.3576635	0.125515	0.09301879	0.111595	0.1343555
1696.368	14.5	0.406022	0.3541847	0.1256004	0.09019789	0.11391	0.1363134
1696.468	14.5	0.397742	0.3684843	0.1252347	0.08892971	0.1125	0.1383033
1696.568	14.5	0.37032	0.3686527	0.1284231	0.08850303	0.110386	0.1393198
1696.668	14.5	0.376913	0.3599377	0.1289015	0.09081598	0.108887	0.1409472
1696.768	14.5	0.402885	0.3565634	0.1288878	0.09356973	0.109009	0.1395558
1696.868	14.5	0.394541	0.3561771	0.1284651	0.09233247	0.109955	0.1395471
1696.968	14.5	0.392225	0.3546534	0.1289776	0.09265891	0.11326	0.139939
1697.068	14.5	0.382964	0.3535968	0.1290695	0.09338346	0.112555	0.1405171
1697.168	14.5	0.390721	0.3519665	0.1297638	0.09323026	0.111718	0.1396198
1697.268	14.5	0.38952	0.3522834	0.1313483	0.09394194	0.114129	0.1390585
1697.368	14.5	0.391776	0.3586273	0.1311723	0.09480567	0.113068	0.1385645
1697.468	14.5	0.388156	0.3565374	0.1330023	0.09777182	0.113342	0.1382097
1697.568	14.5	0.382456	0.3549257	0.1321817	0.1001505	0.1145	0.1369291
1697.668	14.5	0.387736	0.3614953	0.1315789	0.09980937	0.116954	0.1363087
1697.768	14.5	0.395888	0.3587812	0.1294835	0.09909921	0.117867	0.1364988
1697.868	14.5	0.390614	0.3667918	0.1290524	0.09512604	0.119784	0.1374869
1697.968	14.5	0.396552	0.3640522	0.1278703	0.09314986	0.119697	0.1358329
1698.068	14.5	0.389266	0.3662547	0.1258115	0.09268189	0.119089	0.138174
1698.168	14.5	0.386753	0.3598805	0.126259	0.09104743	0.12145	0.1359759
1698.268	14.5	0.383292	0.3585263	0.1277917	0.08913074	0.121972	0.1372962
1698.368	14.5	0.382531	0.3525406	0.1269036	0.08741383	0.12473	0.1392434
1698.468	14.5	0.376722	0.3541289	0.1279159	0.08504841	0.127797	0.1378896
1698.568	14.5	0.380537	0.353901	0.1291908	0.08606695	0.126886	0.1393481
1698.668	14.5	0.382718	0.3455181	0.1257532	0.08714766	0.122847	0.1418725
1698.768	14.5	0.381644	0.3500571	0.1251177	0.08681479	0.119403	0.1414193
1698.868	14.5	0.379564	0.353835	0.1239332	0.08937532	0.117482	0.1398267

1698.968	14.5	0.372023	0.3592633	0.1246566	0.0904149	0.115643	0.138854
1699.068	14.5	0.378656	0.3572046	0.1271463	0.092337	0.114795	0.1366341
1699.168	14.5	0.385114	0.3639048	0.127443	0.09106638	0.117666	0.1370938
1699.268	14.5	0.382331	0.353101	0.1250303	0.09316435	0.116879	0.1366903
1699.368	14.5	0.391369	0.3525639	0.129283	0.09321135	0.115546	0.1379215
1699.468	14.5	0.391965	0.3515431	0.1276373	0.0921319	0.114967	0.1388007
1699.568	14.5	0.394616	0.3538934	0.1270567	0.08755048	0.118117	0.1394812
1699.668	14.5	0.416706	0.3403128	0.1269938	0.08625589	0.120945	0.1367346
1699.768	14.5	0.41474	0.3519042	0.128855	0.08784407	0.122605	0.1337091
1699.868	14.5	0.409272	0.3419401	0.1317017	0.08789155	0.121346	0.1350761
1699.968	14.5	0.423033	0.3576591	0.1337734	0.08515728	0.122418	0.1345051
1700.068	14.5	0.408278	0.348696	0.1363488	0.08647987	0.118832	0.1330414
1700.168	14.5	0.408113	0.3282695	0.1350957	0.08714165	0.116471	0.1290331
1700.268	14.5	0.401893	0.338568	0.1338966	0.08856396	0.116232	0.1291091
1700.368	14.5	0.396237	0.3500308	0.1343358	0.08736331	0.11399	0.1303298
1700.468	14.5	0.40774	0.3459302	0.1322788	0.08416013	0.110796	0.1328902
1700.568	14.5	0.401523	0.3460822	0.1291283	0.08424658	0.111608	0.1322415
1700.668	14.5	0.379256	0.3461853	0.1295137	0.08282158	0.110904	0.1324073
1700.768	14.5	0.382425	0.3497007	0.1297083	0.08379021	0.108745	0.1314063
1700.868	14.5	0.391458	0.3524491	0.1268562	0.0839721	0.109492	0.1318955
1700.968	14.5	0.393145	0.3565868	0.125782	0.0866532	0.10927	0.1342868
1701.068	14.5	0.39345	0.3521118	0.1261384	0.09059703	0.109862	0.1357816
1701.168	14.5	0.386004	0.3437897	0.126184	0.09387472	0.113422	0.1404059
1701.268	14.5	0.389285	0.3492667	0.1287255	0.09355275	0.113434	0.1391116
1701.368	14.5	0.384451	0.3545484	0.1298759	0.09555282	0.111297	0.1399737
1701.468	14.5	0.398434	0.3525808	0.1291064	0.09490011	0.110597	0.1410446
1701.568	14.5	0.394004	0.3581222	0.1290005	0.09640566	0.111023	0.1387378
1701.668	14.5	0.383089	0.3609672	0.1237298	0.0969452	0.112766	0.1409216
1701.768	14.5	0.386504	0.3506944	0.1221148	0.09687275	0.113521	0.1423257
1701.868	14.5	0.395002	0.3544607	0.1247408	0.09597708	0.114338	0.140279
1701.968	14.5	0.390186	0.3523154	0.1234332	0.09251197	0.116225	0.1390747
1702.068	14.5	0.399112	0.3527167	0.1282002	0.09054122	0.119206	0.139077
1702.168	14.5	0.393962	0.351759	0.1298201	0.09123453	0.119946	0.1359425
1702.268	14.5	0.388855	0.357506	0.1313538	0.09154415	0.122065	0.1378039
1702.368	14.5	0.379398	0.3533043	0.1301431	0.0906	0.120586	0.1394138
1702.468	14.5	0.376898	0.3569129	0.1276013	0.09124633	0.120778	0.1363877
1702.568	14.5	0.379038	0.3527292	0.1269123	0.08807907	0.12192	0.1354925
1702.668	14.5	0.379478	0.3522421	0.1262198	0.08901447	0.120927	0.135736
1702.768	14.5	0.382671	0.3465917	0.1243988	0.09067866	0.120643	0.135574
1702.868	14.5	0.39183	0.3586857	0.1247574	0.09009087	0.120539	0.1381133
1702.968	14.5	0.381486	0.3642415	0.1261582	0.09113166	0.120585	0.1384834
1703.068	14.5	0.37612	0.3645715	0.1250729	0.08888172	0.1184	0.1401169
1703.168	14.5	0.382431	0.3626531	0.1236725	0.08967749	0.11616	0.1411357
1703.268	14.5	0.377434	0.3549662	0.1229012	0.08870867	0.115006	0.1421652
1703.368	14.5	0.379614	0.3644308	0.1252396	0.08728857	0.114919	0.1417362
1703.468	14.5	0.385467	0.3617902	0.1234893	0.08881025	0.115067	0.1407285
1703.568	14.5	0.416411	0.3634079	0.1231325	0.08931231	0.116483	0.1403297
1703.668	14.5	0.419377	0.3653999	0.120928	0.08711096	0.11478	0.1402544
1703.768	14.5	0.439275	0.3673155	0.121498	0.0860879	0.111584	0.1388135
1703.868	14.5	0.436316	0.3632068	0.1230539	0.08797057	0.111741	0.1404928
1703.968	14.5	0.422949	0.3671339	0.1236094	0.08867009	0.112916	0.1404063
1704.068	14.5	0.411389	0.3626548	0.1264417	0.08750716	0.114528	0.140893
1704.168	14.5	0.407086	0.3677402	0.1273929	0.08587083	0.117219	0.1402179
1704.268	14.5	0.393439	0.3647467	0.1310543	0.08468066	0.118385	0.1399366
1704.368	14.5	0.40243	0.3668021	0.134624	0.08660061	0.115652	0.1377474
1704.468	14.5	0.395817	0.3704745	0.1353022	0.08748591	0.112756	0.1369024
1704.568	14.5	0.396317	0.3709545	0.1377829	0.08676431	0.111837	0.1368555
1704.668	14.5	0.402426	0.3639841	0.1360167	0.08646242	0.112178	0.1356548
1704.768	14.5	0.387253	0.3604378	0.1369408	0.08438841	0.110353	0.133856
1704.868	14.5	0.363659	0.3636667	0.1334507	0.08648977	0.110049	0.1338418
1704.968	14.5	0.381154	0.3600153	0.1355488	0.08666518	0.110199	0.1333117
1705.068	14.5	0.394614	0.3560157	0.1347541	0.08757029	0.10924	0.1331363
1705.168	14.5	0.394158	0.3562698	0.1318953	0.09055616	0.110138	0.1335786
1705.268	14.5	0.390965	0.3532521	0.1317649	0.0906574	0.110486	0.135022
1705.368	14.5	0.387241	0.3583397	0.1336153	0.09436496	0.110253	0.1344078
1705.468	14.5	0.385282	0.3618891	0.1331798	0.09941694	0.111778	0.1351791
1705.568	14.5	0.396125	0.36723	0.1326551	0.1004261	0.112917	0.1389151
1705.668	14.5	0.396994	0.3586551	0.130884	0.100644	0.110542	0.1417563
1705.768	14.5	0.384255	0.3584684	0.129158	0.1014215	0.107797	0.144067
1705.868	14.5	0.382794	0.3661571	0.1290006	0.1011916	0.10803	0.1435297
1705.968	14.5	0.389749	0.3603786	0.1316371	0.100973	0.105999	0.1435232
1706.068	14.5	0.388836	0.3629408	0.1299323	0.09849734	0.106789	0.143535
1706.168	14.5	0.391859	0.3611697	0.1295876	0.09540243	0.107322	0.1436357
1706.268	14.5	0.395308	0.3630589	0.1294788	0.09527219	0.112188	0.1394527
1706.368	14.5	0.389271	0.3628079	0.1314558	0.09532748	0.117133	0.143092
1706.468	14.5	0.381766	0.3543507	0.1302085	0.09359396	0.121013	0.1386901
1706.568	14.5	0.378159	0.3545819	0.1290033	0.09446777	0.125851	0.1372115
1706.668	14.5	0.376486	0.3483725	0.1321273	0.0936399	0.12829	0.1364089
1706.768	14.5	0.375263	0.3479079	0.1314849	0.09020291	0.126675	0.1341092
1706.868	14.5	0.37787	0.3446401	0.1325483	0.08904574	0.125907	0.1339791
1706.968	14.5	0.386257	0.349007	0.1313087	0.08861078	0.126349	0.1365682
1707.068	14.5	0.380547	0.3475004	0.1285066	0.09062819	0.128184	0.136775
1707.168	14.5	0.376427	0.3571058	0.1281516	0.09281522	0.124409	0.1370976
1707.268	14.5	0.372262	0.350186	0.1275693	0.09177644	0.123443	0.1373011
1707.368	14.5	0.374954	0.359343	0.125774	0.09041131	0.121934	0.135882
1707.468	14.5	0.377301	0.3591205	0.1277559	0.09010877	0.119816	0.1346173
1707.568	14.5	0.382424	0.3561401	0.1269944	0.09056655	0.121057	0.1346655
1707.668	14.5	0.381416	0.3605131	0.1237955	0.09039273	0.121518	0.1370838
1707.768	14.5	0.38295	0.3648532	0.1243971	0.09089825	0.120873	0.1369227
1707.868	14.5	0.396425	0.3511801	0.1206595	0.09005245	0.120087	0.1366513
1707.968	14.5	0.396712	0.3705373	0.1223724	0.08926946	0.117979	0.1371612
1708.068	14.5	0.403047	0.3634808	0.1220006	0.09320686	0.117827	0.1383363
1708.168	14.5	0.404199	0.3634253	0.1244772	0.09509343	0.117812	0.1367428
1708.268	14.5	0.407744	0.3608012	0.1255523	0.09371872	0.118133	0.1373992
1708.368	14.5	0.395754	0.3635995	0.126066	0.09425325	0.119204	0.1379843

1708.468	14.5	0.403383	0.3703383	0.1255153	0.09431446	0.121202	0.1376728
1708.568	14.5	0.402725	0.3632509	0.1274754	0.09504645	0.121702	0.1411534
1708.668	14.5	0.399396	0.3594266	0.128474	0.09251722	0.117092	0.1382381
1708.768	14.5	0.410451	0.3601741	0.1327804	0.09045023	0.114472	0.1376047
1708.868	14.5	0.397022	0.361996	0.1388522	0.09151117	0.113937	0.14104
1708.968	14.5	0.374216	0.3657244	0.1381516	0.09059788	0.113938	0.1410684
1709.068	14.5	0.37177	0.3737406	0.1398304	0.09012394	0.113253	0.1393708
1709.168	14.5	0.39208	0.3726598	0.1426594	0.09094406	0.113138	0.1392343
1709.268	14.5	0.393658	0.3611418	0.1426661	0.09106154	0.112821	0.1384065
1709.368	14.5	0.394815	0.3545381	0.1411551	0.09505127	0.112353	0.1336691
1709.468	14.5	0.383618	0.3613247	0.1364285	0.09904417	0.111036	0.1337825
1709.568	14.5	0.394389	0.367339	0.13788	0.09714112	0.111556	0.1331034
1709.668	14.5	0.390057	0.361101	0.1366276	0.09481069	0.113696	0.133536
1709.768	14.5	0.394512	0.3568215	0.1347926	0.0943463	0.112066	0.1325652
1709.868	14.5	0.388302	0.3588786	0.1302667	0.09698016	0.110099	0.132488
1709.968	14.5	0.380494	0.3543724	0.128768	0.1001989	0.110021	0.1316447
1710.068	14.5	0.385354	0.3542022	0.129482	0.1012735	0.111425	0.1338106
1710.168	14.5	0.394246	0.3534051	0.1310208	0.1015558	0.110224	0.1381394
1710.268	14.5	0.388264	0.3533764	0.1323256	0.09926596	0.11619	0.1402123
1710.368	14.5	0.39323	0.3558433	0.1306798	0.09772582	0.116691	0.139619
1710.468	14.5	0.386061	0.3550816	0.1327236	0.09847266	0.120602	0.1407716
1710.568	14.5	0.395422	0.3563221	0.1322383	0.09785433	0.122398	0.1396986
1710.668	14.5	0.385482	0.3582485	0.1302883	0.09606476	0.121715	0.1406577
1710.768	14.5	0.380687	0.3605811	0.1312104	0.09549826	0.121647	0.1403632
1710.868	14.5	0.373086	0.3603797	0.1294028	0.09468075	0.120586	0.1386068
1710.968	14.5	0.376948	0.3535406	0.1295509	0.09517516	0.124419	0.1394039
1711.068	14.5	0.375664	0.3562435	0.1316658	0.09633817	0.127212	0.1364177
1711.168	14.5	0.379945	0.3454346	0.12959	0.09593869	0.124915	0.1336913
1711.268	14.5	0.378656	0.3452686	0.130055	0.09688445	0.119532	0.1312115
1711.368	14.5	0.373544	0.3495521	0.1320935	0.09648861	0.119623	0.1313471
1711.468	14.5	0.37542	0.3444399	0.1327637	0.09554151	0.117002	0.1358156
1711.568	14.5	0.376304	0.3534832	0.1294578	0.09210127	0.11761	0.1380139
1711.668	14.5	0.376489	0.3504085	0.12553	0.09247485	0.118538	0.1363107
1711.768	14.5	0.382614	0.3538485	0.1251341	0.09181552	0.119622	0.1380045
1711.868	14.5	0.385394	0.3595567	0.1262039	0.09193813	0.120247	0.138377
1711.968	14.5	0.393322	0.3524795	0.1239289	0.09002548	0.117301	0.1342591
1712.068	14.5	0.399231	0.3486022	0.1220723	0.08909224	0.115405	0.1342669
1712.168	14.5	0.402864	0.3521791	0.1208801	0.09066055	0.115607	0.13203
1712.268	14.5	0.405721	0.3514931	0.1181978	0.09217785	0.11423	0.133856
1712.368	14.5	0.441737	0.3452612	0.120974	0.08841626	0.115508	0.1363797
1712.468	14.5	0.444138	0.3432962	0.1200838	0.08840872	0.117535	0.1367733
1712.568	14.5	0.443528	0.3421564	0.120478	0.0888422	0.117197	0.1356957
1712.668	14.5	0.428965	0.3543219	0.120185	0.09101734	0.113662	0.1345173
1712.768	14.5	0.426104	0.3344775	0.1224297	0.09382	0.11063	0.1350289
1712.868	14.5	0.432074	0.335526	0.1252446	0.09290606	0.111362	0.1342706
1712.968	14.5	0.412815	0.3498826	0.1272286	0.09416387	0.111645	0.1301965
1713.068	14.5	0.395304	0.3442853	0.1269126	0.09500927	0.109713	0.1308821
1713.168	14.5	0.383301	0.3454543	0.1305781	0.09791417	0.106595	0.1309591
1713.268	14.5	0.408392	0.3455854	0.1320187	0.09708098	0.107643	0.131756
1713.368	14.5	0.405499	0.3504792	0.1330898	0.09630959	0.108534	0.1330284
1713.468	14.5	0.3945	0.3502784	0.1342553	0.096381	0.108758	0.1334926
1713.568	14.5	0.392816	0.3506266	0.1349484	0.0972063	0.111445	0.1302699
1713.668	14.5	0.396609	0.3456296	0.1384349	0.09570926	0.110963	0.1281389
1713.768	14.5	0.386186	0.3484848	0.1394557	0.0971517	0.109061	0.1293042
1713.868	14.5	0.396391	0.3492275	0.1404494	0.09568951	0.106382	0.1304302
1713.968	14.5	0.393154	0.3509715	0.1374232	0.09667037	0.106834	0.1281482
1714.068	14.5	0.376776	0.3583089	0.1360031	0.09766276	0.10811	0.128373
1714.168	14.5	0.382388	0.3486535	0.1342277	0.09954473	0.110363	0.127681
1714.268	14.5	0.391261	0.34905	0.131252	0.1008855	0.109963	0.1313761
1714.368	14.5	0.385024	0.3583456	0.1287157	0.1004582	0.112823	0.1331204
1714.468	14.5	0.391214	0.3544837	0.1275497	0.1006022	0.113369	0.1341837
1714.568	14.5	0.390516	0.3495355	0.1268908	0.09856746	0.11576	0.1341496
1714.668	14.5	0.392108	0.3593792	0.1287104	0.0959539	0.115636	0.1320765
1714.768	14.5	0.379939	0.3542181	0.1280766	0.09415846	0.118604	0.1360121
1714.868	14.5	0.381225	0.3628789	0.1275191	0.09403568	0.122663	0.138056
1714.968	14.5	0.37338	0.359295	0.1276405	0.09138623	0.124906	0.1363732
1715.068	14.5	0.374637	0.3529502	0.1285189	0.08933374	0.125215	0.1382176
1715.168	14.5	0.380089	0.3517761	0.1285746	0.09287213	0.12761	0.1372077
1715.268	14.5	0.387091	0.3666258	0.1288738	0.09429946	0.128831	0.1418575
1715.368	14.5	0.376958	0.3585299	0.1298762	0.09415296	0.126304	0.1420055
1715.468	14.5	0.374233	0.360819	0.1295656	0.09420259	0.12362	0.1404606
1715.568	14.5	0.385251	0.3546598	0.1301475	0.09331729	0.122261	0.141826
1715.668	14.5	0.380869	0.3587168	0.1303291	0.09242031	0.125099	0.1412347
1715.768	14.5	0.379676	0.3622211	0.1302147	0.08942571	0.123653	0.1398749
1715.868	14.5	0.387395	0.3604088	0.1312707	0.09056677	0.122178	0.1388272
1715.968	14.5	0.393134	0.3638295	0.1343284	0.08986118	0.122139	0.1365397
1716.068	14.5	0.391507	0.3634095	0.13214	0.0888022	0.121464	0.1360371
1716.168	14.5	0.40997	0.3625434	0.1309407	0.08573947	0.122617	0.1356097
1716.268	14.5	0.407456	0.3686376	0.1300988	0.08419757	0.116248	0.1359204
1716.368	14.5	0.421538	0.3605615	0.131393	0.08758149	0.113129	0.1352733
1716.468	14.5	0.41932	0.3677236	0.1300229	0.09114229	0.114235	0.1356974
1716.568	14.5	0.417926	0.3717178	0.1292336	0.08936403	0.116501	0.134982
1716.668	14.5	0.415446	0.3705547	0.1307143	0.08847389	0.118133	0.1353098
1716.768	14.5	0.415752	0.3727696	0.1331874	0.09021787	0.119739	0.1346672
1716.868	14.5	0.42019	0.365437	0.1292728	0.09297871	0.119875	0.1366714
1716.968	14.5	0.424357	0.3593788	0.128677	0.09336263	0.117146	0.1379763
1717.068	14.5	0.44587	0.367713	0.127093	0.09160679	0.114667	0.1381252
1717.168	14.5	0.444436	0.3617764	0.1256482	0.09407277	0.111731	0.1366651
1717.268	14.5	0.409814	0.3551299	0.1253416	0.09324478	0.11036	0.1334418
1717.368	14.5	0.397455	0.3558208	0.1257872	0.09554885	0.109634	0.1338967
1717.468	14.5	0.410054	0.3570607	0.1253624	0.09495121	0.108802	0.1342613
1717.568	14.5	0.39186	0.3623504	0.1249936	0.09309682	0.108071	0.1327462
1717.668	14.5	0.39184	0.3628282	0.1253821	0.09443935	0.108334	0.1330223
1717.768	14.5	0.385489	0.3456561	0.1257743	0.0942364	0.11185	0.1333138
1717.868	14.5	0.385914	0.3567866	0.1248951	0.09270055	0.111049	0.1345463

1717.968	14.5	0.389854	0.361994	0.129417	0.09595916	0.108629	0.1348156
1718.068	14.5	0.391201	0.3620534	0.1290871	0.09602019	0.111274	0.1379341
1718.168	14.5	0.386009	0.3625922	0.1320884	0.09736424	0.111031	0.1369644
1718.268	14.5	0.377835	0.3601753	0.1355178	0.09798459	0.11251	0.1341849
1718.368	14.5	0.386527	0.3481189	0.1359451	0.09801979	0.111445	0.1345195
1718.468	14.5	0.390665	0.3388428	0.140179	0.09771501	0.108057	0.1343756
1718.568	14.5	0.386455	0.3474894	0.1401703	0.09561992	0.112612	0.1326995
1718.668	14.5	0.394496	0.3523901	0.1342872	0.09315415	0.114296	0.1327254
1718.768	14.5	0.387256	0.3491474	0.1359368	0.09227926	0.11572	0.1334942
1718.868	14.5	0.390249	0.3462068	0.1365115	0.09196625	0.114361	0.1344776
1718.968	14.5	0.380928	0.3490393	0.1364482	0.09334993	0.11477	0.1377275
1719.068	14.5	0.38139	0.3580055	0.1331979	0.09521978	0.118182	0.1370784
1719.168	14.5	0.375338	0.3541319	0.1298595	0.0938406	0.122502	0.136484
1719.268	14.5	0.381904	0.3612707	0.1327342	0.09305072	0.124514	0.1330544
1719.368	14.5	0.383712	0.3604327	0.1303925	0.09240486	0.126619	0.1326377
1719.468	14.5	0.373825	0.3567627	0.1298084	0.09200919	0.124595	0.1348429
1719.568	14.5	0.370272	0.3613953	0.1283645	0.09111018	0.121508	0.1369454
1719.668	14.5	0.383609	0.3651104	0.1295404	0.09047699	0.118614	0.1396782
1719.768	14.5	0.398431	0.3597873	0.1293206	0.0910573	0.11854	0.1383087
1719.868	14.5	0.391853	0.3744267	0.1310256	0.09029571	0.118785	0.1394095
1719.968	14.5	0.393755	0.359794	0.1309675	0.08956316	0.120946	0.1418318
1720.068	14.5	0.394854	0.3665167	0.1291182	0.088162	0.122881	0.1428925
1720.168	14.5	0.405684	0.365204	0.1283087	0.08719876	0.122307	0.1435389
1720.268	14.5	0.405362	0.3709279	0.1313066	0.0836751	0.118872	0.1430638
1720.368	14.5	0.414161	0.3731415	0.1309078	0.08272758	0.11898	0.1391303
1720.468	14.5	0.445093	0.3643114	0.1318274	0.08311956	0.119316	0.137769
1720.568	14.5	0.438115	0.3622449	0.1350185	0.08777528	0.120323	0.1353315
1720.668	14.5	0.468572	0.3603374	0.1335998	0.08638102	0.121474	0.1344504
1720.768	14.5	0.460584	0.3588122	0.1333179	0.08634596	0.120549	0.1366381
1720.868	14.5	0.449885	0.3675604	0.132293	0.08712689	0.117126	0.1380815
1720.968	14.5	0.44334	0.3721796	0.131861	0.08946459	0.117312	0.1371768
1721.068	14.5	0.44668	0.3781276	0.1307125	0.09089708	0.11507	0.1385244
1721.168	14.5	0.458246	0.3769143	0.1299862	0.08788054	0.111094	0.1404077
1721.268	14.5	0.421041	0.3621863	0.1323286	0.08813042	0.108847	0.1387599
1721.368	14.5	0.377998	0.3544043	0.133469	0.08697061	0.110027	0.1373028
1721.468	14.5	0.368442	0.362172	0.1300473	0.08815499	0.110916	0.1368406
1721.568	14.5	0.388389	0.3663034	0.1298269	0.08816487	0.111432	0.1353915
1721.668	14.5	0.389819	0.3621772	0.12805	0.08478922	0.111108	0.1360822
1721.768	14.5	0.399819	0.3579742	0.1224073	0.08706629	0.109933	0.1351068
1721.868	14.5	0.386042	0.3570121	0.1214229	0.0914677	0.109874	0.1343773
1721.968	14.5	0.396283	0.3536784	0.1240602	0.09313861	0.112188	0.1325387
1722.068	14.5	0.387206	0.3541291	0.1273622	0.09338949	0.111915	0.1322293
1722.168	14.5	0.394179	0.3541507	0.1297904	0.09393102	0.112554	0.1335489
1722.268	14.5	0.393354	0.3564907	0.1305418	0.09487106	0.113835	0.1357012
1722.368	14.5	0.379358	0.3565328	0.1293312	0.09428652	0.112006	0.1355128
1722.468	14.5	0.384964	0.3568916	0.1301355	0.09342741	0.111013	0.1340925
1722.568	14.5	0.390592	0.365748	0.1338768	0.09432908	0.10973	0.1333946
1722.668	14.5	0.387503	0.3604495	0.1342745	0.09474664	0.113228	0.1367312
1722.768	14.5	0.398225	0.3667553	0.1343542	0.09359407	0.111171	0.1374389
1722.868	14.5	0.389695	0.363479	0.1373324	0.09202621	0.109667	0.1379762
1722.968	14.5	0.397197	0.3616741	0.1397563	0.09300696	0.11263	0.137305
1723.068	14.5	0.376303	0.3631988	0.1406486	0.09256906	0.111756	0.1374082
1723.168	14.5	0.382313	0.3473155	0.1416041	0.09315784	0.111493	0.135851
1723.268	14.5	0.371839	0.3483711	0.1346885	0.09053024	0.117141	0.136754
1723.368	14.5	0.377908	0.3486702	0.1345883	0.09029873	0.122331	0.1369303
1723.468	14.5	0.386058	0.3465385	0.1354602	0.0892689	0.12637	0.136605
1723.568	14.5	0.384173	0.3539228	0.1350987	0.08991924	0.128596	0.1372342
1723.668	14.5	0.372653	0.3539076	0.1347414	0.08795692	0.126346	0.1357543
1723.768	14.5	0.374114	0.3581629	0.1328283	0.0891604	0.125459	0.1347407
1723.868	14.5	0.384984	0.357943	0.1344348	0.09042371	0.122175	0.1348865
1723.968	14.5	0.384069	0.3567224	0.1307286	0.09024701	0.121813	0.1377579
1724.068	14.5	0.379459	0.3541356	0.1290936	0.08902021	0.124301	0.1382965
1724.168	14.5	0.380134	0.3544125	0.1278456	0.09132823	0.12875	0.1377849
1724.268	14.5	0.39107	0.356543	0.1289452	0.09005474	0.129749	0.1382751
1724.368	14.5	0.388874	0.3443548	0.1304735	0.08749472	0.12688	0.1378821
1724.468	14.5	0.407882	0.350164	0.1289335	0.08612344	0.126076	0.1376095
1724.568	14.5	0.407998	0.3426756	0.1309427	0.08532524	0.124584	0.1372378
1724.668	14.5	0.421634	0.3584733	0.1307326	0.08886495	0.125207	0.1393594
1724.768	14.5	0.41597	0.3505402	0.1288954	0.08749797	0.12838	0.1399427
1724.868	14.5	0.429617	0.3270543	0.1300713	0.08859199	0.125436	0.1411338
1724.968	14.5	0.417101	0.350457	0.1304762	0.08686511	0.122053	0.1364222
1725.068	14.5	0.412046	0.3462751	0.1292805	0.08957808	0.119282	0.1356737
1725.168	14.5	0.419368	0.3441603	0.1299165	0.09062465	0.11786	0.1353748
1725.268	14.5	0.417758	0.349306	0.1291374	0.0891902	0.113981	0.1348885
1725.368	14.5	0.414397	0.3560875	0.130171	0.08577114	0.113205	0.1341105
1725.468	14.5	0.411254	0.3588447	0.1275273	0.08607222	0.110518	0.1349466
1725.568	14.5	0.41201	0.3606766	0.127374	0.08834919	0.109068	0.1325232
1725.668	14.5	0.411363	0.3494842	0.1272845	0.08552836	0.109292	0.1309258
1725.768	14.5	0.400182	0.3484615	0.1268335	0.08306696	0.109481	0.1331501
1725.868	14.5	0.394956	0.3558571	0.1278193	0.08571269	0.109238	0.1342434
1725.968	14.5	0.386738	0.3560722	0.1305341	0.09103378	0.109629	0.134285
1726.068	14.5	0.394949	0.3608864	0.1292133	0.09036613	0.110753	0.1354893
1726.168	14.5	0.388236	0.3611717	0.1294637	0.09225133	0.107946	0.1337447
1726.268	14.5	0.397517	0.3510609	0.1276313	0.09606377	0.107762	0.1357518
1726.368	14.5	0.40048	0.3539149	0.1265652	0.09613235	0.105957	0.1366929
1726.468	14.5	0.384351	0.3547911	0.1265794	0.09493869	0.10765	0.1350733
1726.568	14.5	0.385227	0.3501122	0.1263994	0.09520408	0.109422	0.1352815
1726.668	14.5	0.393388	0.356353	0.1282317	0.09459756	0.109476	0.1373105
1726.768	14.5	0.388019	0.3531851	0.1278356	0.09512657	0.109871	0.135295
1726.868	14.5	0.408756	0.3644164	0.1291918	0.0945424	0.114955	0.1345468
1726.968	14.5	0.406504	0.3640082	0.1313138	0.09333733	0.117009	0.1309052
1727.068	14.5	0.405434	0.3603717	0.13257	0.0925713	0.118437	0.130859
1727.168	14.5	0.392014	0.3568294	0.1328953	0.09124915	0.117771	0.1319339
1727.268	14.5	0.391425	0.3684315	0.1339036	0.09173946	0.115207	0.1335521
1727.368	14.5	0.375288	0.3618469	0.1351364	0.0889585	0.116979	0.1346025

1727.468	14.5	0.37381	0.365265	0.1403723	0.08876184	0.119224	0.1341439
1727.568	14.5	0.399783	0.3568477	0.1424922	0.09076237	0.121761	0.1376212
1727.668	14.5	0.408044	0.3627169	0.1424135	0.09309371	0.123692	0.1353218
1727.768	14.5	0.380896	0.3656412	0.1397592	0.0922163	0.122287	0.135103
1727.868	14.5	0.374102	0.3653342	0.136298	0.0905961	0.123109	0.132073
1727.968	14.5	0.389041	0.3695038	0.1337302	0.09115328	0.120116	0.1316648
1728.068	14.5	0.387233	0.3724607	0.1333405	0.08920641	0.118736	0.132838
1728.168	14.5	0.383485	0.3686175	0.1324897	0.08812788	0.118391	0.1337622
1728.268	14.5	0.387201	0.3707942	0.13245	0.09060576	0.119517	0.1335713
1728.368	14.5	0.400513	0.3646281	0.1302591	0.08921929	0.12124	0.1342413
1728.468	14.5	0.394174	0.3737288	0.1314088	0.08851318	0.117884	0.1343278
1728.568	14.5	0.405835	0.3742635	0.1297398	0.08873425	0.119392	0.13114
1728.668	14.5	0.410449	0.3691202	0.1296311	0.08817415	0.118881	0.1314304
1728.768	14.5	0.430547	0.3750341	0.1277969	0.08915295	0.118347	0.1323598
1728.868	14.5	0.434049	0.3734938	0.1261589	0.08845904	0.118359	0.1359566
1728.968	14.5	0.436906	0.3680812	0.1284834	0.09013134	0.119506	0.1390081
1729.068	14.5	0.416253	0.3738439	0.1310365	0.09204556	0.11832	0.1455259
1729.168	14.5	0.403401	0.3675746	0.1283865	0.09189014	0.115415	0.1438444
1729.268	14.5	0.403196	0.3609531	0.1275171	0.09062355	0.11398	0.1425531
1729.368	14.5	0.39331	0.3587143	0.125101	0.08768651	0.114237	0.1398886
1729.468	14.5	0.401403	0.3608134	0.1251426	0.08751667	0.113778	0.1407973
1729.568	14.5	0.394891	0.3691764	0.1257388	0.08844047	0.111325	0.1373125
1729.668	14.5	0.369085	0.3701453	0.1275878	0.08820222	0.109771	0.1379752
1729.768	14.5	0.371729	0.3502039	0.1325126	0.08803325	0.10712	0.1379033
1729.868	14.5	0.387859	0.3612555	0.1313708	0.08960654	0.10722	0.1346063
1729.968	14.5	0.395942	0.3682723	0.1296814	0.09118016	0.107687	0.1354414
1730.068	14.5	0.399391	0.367301	0.1270812	0.09339716	0.107204	0.1370339
1730.168	14.5	0.383583	0.367545	0.1284282	0.0924155	0.107289	0.1386295
1730.268	14.5	0.394314	0.3650659	0.1274886	0.09136944	0.107152	0.1391479
1730.368	14.5	0.392251	0.359591	0.1249471	0.0929243	0.106937	0.1369273
1730.468	14.5	0.40175	0.3386873	0.1254708	0.09512573	0.111116	0.1349671
1730.568	14.5	0.392983	0.3450577	0.1277452	0.09707928	0.109989	0.1332829
1730.668	14.5	0.383361	0.3507646	0.1256304	0.09470651	0.111297	0.1360751
1730.768	14.5	0.387376	0.3546613	0.1244412	0.09554221	0.113193	0.136819
1730.868	14.5	0.392291	0.3521546	0.1253618	0.09518827	0.109854	0.1379764
1730.968	14.5	0.3905	0.3598395	0.124719	0.09168329	0.11016	0.1374458
1731.068	14.5	0.40444	0.3624353	0.1228614	0.09137162	0.110922	0.1369494
1731.168	14.5	0.398574	0.362687	0.1216906	0.09041034	0.112402	0.1354161
1731.268	14.5	0.397325	0.3635085	0.1249565	0.0921291	0.115919	0.1356748
1731.368	14.5	0.381272	0.3652724	0.1259294	0.08890131	0.120112	0.1322339
1731.468	14.5	0.381013	0.3593853	0.1265564	0.08898626	0.124195	0.1320709
1731.568	14.5	0.37137	0.3625315	0.1264739	0.0884802	0.119755	0.132256
1731.668	14.5	0.379033	0.3704026	0.1250773	0.08702692	0.11784	0.1349066
1731.768	14.5	0.381228	0.3571872	0.1274988	0.08709825	0.122451	0.1355387
1731.868	14.5	0.380414	0.3786633	0.1310267	0.08968911	0.122462	0.1363418
1731.968	14.5	0.37696	0.3632035	0.1332	0.09283622	0.119757	0.1382404
1732.068	14.5	0.370286	0.3668241	0.1340757	0.0908926	0.118295	0.1354784
1732.168	14.5	0.369568	0.3681633	0.1344047	0.09013402	0.116106	0.1350487
1732.268	14.5	0.370125	0.3710776	0.1361772	0.08928094	0.116465	0.1345682
1732.368	14.5	0.375555	0.3759897	0.1365358	0.08883464	0.117078	0.1326061
1732.468	14.5	0.37654	0.3728332	0.1332266	0.08863009	0.119686	0.130997
1732.568	14.5	0.378421	0.3673738	0.1331717	0.08767799	0.117422	0.1319591
1732.668	14.5	0.389007	0.3585234	0.1314813	0.08515293	0.116336	0.1320765
1732.768	14.5	0.397529	0.3591498	0.1295308	0.08522466	0.116056	0.1313376
1732.868	14.5	0.406832	0.3614715	0.1259402	0.08948765	0.115197	0.1304806
1732.968	14.5	0.403315	0.3704168	0.1240771	0.08968919	0.118358	0.1304075
1733.068	14.5	0.414084	0.3769203	0.1278886	0.0880823	0.118944	0.1304959
1733.168	14.5	0.433317	0.3771473	0.1257043	0.09162136	0.117909	0.130351
1733.268	14.5	0.422523	0.3615746	0.1266545	0.09045819	0.115048	0.130879
1733.368	14.5	0.444813	0.3529464	0.1290756	0.09099558	0.114474	0.1320514
1733.468	14.5	0.427999	0.3624734	0.1280911	0.09173841	0.115075	0.138154
1733.568	14.5	0.425202	0.3673489	0.1298717	0.09059168	0.115396	0.1377772
1733.668	14.5	0.418668	0.3666671	0.1291716	0.08858576	0.112326	0.1374451
1733.768	14.5	0.396064	0.3607873	0.1288368	0.08898226	0.110013	0.1346061
1733.868	14.5	0.376995	0.3585236	0.1289035	0.09221671	0.108465	0.1328765
1733.968	14.5	0.3877	0.3556173	0.1297498	0.09261996	0.108721	0.1321191
1734.068	14.5	0.388041	0.3572711	0.1318835	0.0941752	0.111197	0.1326227
1734.168	14.5	0.39655	0.3534035	0.1287622	0.09721284	0.11085	0.1327259
1734.268	14.5	0.38571	0.3599012	0.1291066	0.09718979	0.111124	0.1333258
1734.368	14.5	0.390572	0.3613702	0.1328641	0.09745359	0.111866	0.1346864
1734.468	14.5	0.388094	0.360248	0.131002	0.09887561	0.113534	0.1350048
1734.568	14.5	0.400973	0.3652906	0.1305496	0.1004348	0.11508	0.1344537
1734.668	14.5	0.393799	0.3623614	0.128809	0.1007753	0.112468	0.137091
1734.768	14.5	0.376426	0.3696883	0.127255	0.09888882	0.111194	0.1387932
1734.868	14.5	0.38243	0.3645301	0.1282159	0.1006897	0.112106	0.1379882
1734.968	14.5	0.393453	0.3586054	0.1273911	0.1015595	0.111774	0.1435085
1735.068	14.5	0.384278	0.3627208	0.128072	0.09922402	0.114656	0.1423488
1735.168	14.5	0.394011	0.3529852	0.1288449	0.09628135	0.116508	0.1392767
1735.268	14.5	0.394669	0.3570938	0.126139	0.09460594	0.118628	0.1398196
1735.368	14.5	0.399132	0.3565921	0.1239097	0.09379106	0.11772	0.1403452
1735.468	14.5	0.384837	0.3522144	0.124074	0.09371573	0.118045	0.1404889
1735.568	14.5	0.386449	0.3566762	0.1239369	0.0926968	0.120161	0.1411517
1735.668	14.5	0.376078	0.3535702	0.1228014	0.09113545	0.120048	0.1373758
1735.768	14.5	0.376314	0.3615146	0.1231984	0.08989546	0.120797	0.1362044
1735.868	14.5	0.383955	0.3603099	0.1242839	0.0923942	0.122932	0.1389506
1735.968	14.5	0.389735	0.3607373	0.1255436	0.09209811	0.125011	0.137056
1736.068	14.5	0.376551	0.3537009	0.1248528	0.09304848	0.126253	0.1349639
1736.168	14.5	0.373328	0.352227	0.1259518	0.0924687	0.124937	0.1353204
1736.268	14.5	0.392492	0.3561935	0.1268691	0.09242697	0.120532	0.1357915
1736.368	14.5	0.377726	0.340917	0.1300878	0.09376334	0.121452	0.1339205
1736.468	14.5	0.386696	0.3484368	0.1324409	0.09393569	0.122275	0.1331709
1736.568	14.5	0.396008	0.345854	0.1347468	0.09260676	0.122752	0.1341272
1736.668	14.5	0.40406	0.3603838	0.1345953	0.09140444	0.12199	0.1330136
1736.768	14.5	0.417182	0.3597719	0.1355897	0.09052049	0.121097	0.1321185
1736.868	14.5	0.452778	0.3269085	0.1365892	0.0885364	0.121322	0.1327219

1736.968	14.5	0.474337	0.3450905	0.1404236	0.08949189	0.121311	0.1319242
1737.068	14.5	0.487758	0.3425294	0.1364351	0.09255646	0.121435	0.13094
1737.168	14.5	0.488553	0.3440191	0.1329465	0.09274862	0.12189	0.1330605
1737.268	14.5	0.483284	0.3497031	0.1321262	0.09478055	0.119662	0.1324989
1737.368	14.5	0.473871	0.3552879	0.130893	0.0934149	0.120594	0.1337274
1737.468	14.5	0.458973	0.3582929	0.129859	0.09110901	0.119496	0.1355144
1737.568	14.5	0.440308	0.3650088	0.1303549	0.09065451	0.118322	0.1349497
1737.668	14.5	0.428952	0.3528531	0.1311153	0.09289033	0.114999	0.1326563
1737.768	14.5	0.426429	0.3469918	0.12844	0.09155884	0.111675	0.1348167
1737.868	14.5	0.396452	0.3587412	0.1288655	0.0931994	0.110852	0.1355406
1737.968	14.5	0.372318	0.3624342	0.1318151	0.09484962	0.107857	0.1368799
1738.068	14.5	0.391224	0.3654844	0.132215	0.0971461	0.109292	0.1388782
1738.168	14.5	0.388621	0.3700954	0.1324089	0.09587843	0.110391	0.1398704
1738.268	14.5	0.387482	0.3546979	0.1332602	0.09403791	0.108403	0.141956
1738.368	14.5	0.387436	0.3555814	0.1318583	0.09628342	0.108435	0.1423527
1738.468	14.5	0.395897	0.3577566	0.1312584	0.09886642	0.107883	0.1414061
1738.568	14.5	0.398873	0.3572221	0.1325068	0.09811144	0.108365	0.1379426
1738.706	14.5	0.382072	0.35704	0.1322868	0.09718451	0.110217	0.1384472
1738.806	15	0.380697	0.3577879	0.132606	0.09664246	0.112154	0.1368746
1738.906	15	0.390728	0.36474	0.1324021	0.0987911	0.113584	0.1361389
1739.006	15	0.387041	0.3690374	0.1341347	0.1004394	0.115937	0.1378711
1739.106	15	0.397945	0.3657232	0.135453	0.08669699	0.115924	0.1386579
1739.206	15	0.394004	0.3574023	0.1356287	0.08424421	0.113925	0.1401425
1739.306	15	0.396006	0.3711873	0.1332514	0.08731846	0.113226	0.1409771
1739.406	15	0.383091	0.3680371	0.1311632	0.08454197	0.113973	0.1378242
1739.506	15	0.384391	0.3669384	0.1311562	0.0821504	0.116444	0.136793
1739.606	15	0.375826	0.3598495	0.1307925	0.08368299	0.11797	0.1398538
1739.706	15	0.377391	0.3624887	0.1295615	0.08433931	0.118948	0.1398953
1739.806	15	0.38185	0.3699021	0.129975	0.08554973	0.123117	0.1411815
1739.906	15	0.382152	0.3643162	0.1315607	0.08586063	0.125822	0.1410218
1740.006	15	0.379335	0.3642349	0.127483	0.08718598	0.125362	0.1400089
1740.106	15	0.368418	0.3726111	0.1229025	0.08736009	0.129113	0.1386849
1740.206	15	0.373298	0.3650672	0.1251127	0.08781979	0.126675	0.1393947
1740.306	15	0.380676	0.3755664	0.1256208	0.08858605	0.12411	0.1384947
1740.406	15	0.378276	0.3675674	0.1261374	0.09120424	0.1229	0.1383227
1740.506	15	0.385627	0.3767413	0.125723	0.09404394	0.121428	0.1391628
1740.606	15	0.384661	0.3757642	0.125463	0.09097069	0.118124	0.1352028
1740.706	15	0.391384	0.3719939	0.1294054	0.08881459	0.118915	0.1353663
1740.806	15	0.404862	0.3712998	0.1282756	0.08800992	0.118691	0.1356838
1740.906	15	0.415382	0.3721986	0.1317897	0.09183524	0.117259	0.1350612
1741.006	15	0.419092	0.3710122	0.1343408	0.09561265	0.117246	0.1358583
1741.106	15	0.424671	0.3764606	0.1370514	0.0982058	0.118354	0.1340158
1741.206	15	0.417832	0.3684098	0.1378185	0.1005956	0.11715	0.1354026
1741.306	15	0.402442	0.3635463	0.1391103	0.1024633	0.118449	0.1349197
1741.406	15	0.40496	0.3604813	0.1378567	0.1014534	0.118293	0.133539
1741.506	15	0.393075	0.3624033	0.1318671	0.1000726	0.119776	0.1356627
1741.606	15	0.397727	0.368149	0.1319816	0.1014935	0.11818	0.1361846
1741.706	15	0.39929	0.3703789	0.1317869	0.0990442	0.114604	0.135031
1741.806	15	0.385003	0.358115	0.1292362	0.09705333	0.113432	0.1324164
1741.906	15	0.384507	0.3656054	0.1270813	0.09379398	0.111025	0.1313663
1742.006	15	0.391389	0.3695931	0.1266066	0.09339191	0.110163	0.1361578
1742.106	15	0.391519	0.368135	0.1290603	0.09330496	0.10812	0.1379401
1742.206	15	0.387958	0.3685124	0.1273908	0.09392274	0.108999	0.1391054
1742.306	15	0.387379	0.3691749	0.1274582	0.08989042	0.107911	0.1381559
1742.406	15	0.390605	0.3655096	0.126717	0.091459	0.109069	0.1383742
1742.506	15	0.395851	0.3451042	0.1271477	0.09426713	0.108468	0.1391832
1742.606	15	0.397908	0.3500494	0.1300287	0.09489068	0.108784	0.1417725
1742.706	15	0.382942	0.3570555	0.1280335	0.09165549	0.107828	0.1436657
1742.806	15	0.381604	0.3569318	0.1272718	0.09073903	0.10807	0.1453177
1742.906	15	0.392026	0.3551181	0.1283867	0.09048966	0.110147	0.143365
1743.006	15	0.383361	0.3601092	0.1291522	0.08880132	0.111223	0.1445682
1743.106	15	0.397185	0.3642999	0.129121	0.08697533	0.112788	0.1436433
1743.206	15	0.390115	0.3592361	0.1289241	0.08623284	0.114525	0.1414645
1743.306	15	0.389691	0.3635266	0.1316258	0.08477169	0.113717	0.1423977
1743.406	15	0.377531	0.3685853	0.1311827	0.08350147	0.117305	0.1395505
1743.506	15	0.379696	0.3601513	0.1322778	0.08149783	0.116484	0.1404579
1743.606	15	0.372396	0.3633633	0.1290857	0.08421112	0.118375	0.1409537
1743.706	15	0.377762	0.3703314	0.1267335	0.08695449	0.116224	0.139688
1743.806	15	0.381963	0.3592726	0.1271356	0.08749399	0.115912	0.1401076
1743.906	15	0.377988	0.3781868	0.1250429	0.08872838	0.118879	0.1418446
1744.006	15	0.377958	0.3604708	0.1251267	0.09150665	0.119249	0.1408269
1744.106	15	0.373835	0.3683262	0.1261003	0.09274954	0.12079	0.1381933
1744.206	15	0.375049	0.3668138	0.1226476	0.09064917	0.121151	0.1398363
1744.306	15	0.400378	0.3708248	0.1212423	0.08849492	0.123463	0.1398042
1744.406	15	0.391762	0.3723701	0.1207807	0.09068125	0.125565	0.1393768
1744.506	15	0.39126	0.3684665	0.1205336	0.09447457	0.124051	0.138344
1744.606	15	0.388363	0.3658863	0.1199611	0.09510528	0.124021	0.138422
1744.706	15	0.40111	0.3594331	0.1199823	0.09477318	0.124085	0.1372551
1744.806	15	0.412864	0.3619736	0.1223606	0.09444806	0.123378	0.1373925
1744.906	15	0.418861	0.3658671	0.1227201	0.09421017	0.125298	0.1377703
1745.006	15	0.412884	0.3685038	0.125383	0.09832142	0.124366	0.1366239
1745.106	15	0.412367	0.3797703	0.1236012	0.1015782	0.121228	0.1374322
1745.206	15	0.412342	0.379239	0.1265965	0.09880523	0.12077	0.1373429
1745.306	15	0.39916	0.3648535	0.1304583	0.1003492	0.118681	0.1351379
1745.406	15	0.399661	0.359786	0.1357404	0.1029769	0.11766	0.1340889
1745.506	15	0.391313	0.3697497	0.1401932	0.106974	0.118323	0.1338974
1745.606	15	0.392849	0.36999	0.1384953	0.1032607	0.117115	0.1333653
1745.706	15	0.389685	0.3670748	0.1361491	0.09706814	0.115786	0.132997
1745.806	15	0.368728	0.3638988	0.1381784	0.09467095	0.11703	0.1353796
1745.906	15	0.381326	0.3645363	0.1345971	0.0944459	0.11547	0.1347333
1746.006	15	0.389593	0.3607619	0.1339485	0.09367527	0.111535	0.1363604
1746.106	15	0.390671	0.3615253	0.1342391	0.0946549	0.114653	0.137672
1746.206	15	0.390907	0.3583429	0.1328046	0.09742573	0.11315	0.1356932
1746.306	15	0.388329	0.3634307	0.1300078	0.0949985	0.111927	0.1334257
1746.406	15	0.389435	0.363247	0.1287627	0.09374478	0.111647	0.1323611

1746.506	15	0.399122	0.3630299	0.1290901	0.09462592	0.112819	0.1343828
1746.606	15	0.400089	0.3698032	0.1268431	0.0941643	0.112716	0.1352777
1746.706	15	0.383373	0.3665279	0.1288495	0.09044644	0.11248	0.1362408
1746.806	15	0.382378	0.3722033	0.12793	0.09061033	0.110154	0.1339303
1746.906	15	0.392677	0.3532886	0.129462	0.09095766	0.110297	0.1348515
1747.006	15	0.383455	0.3578473	0.1294385	0.08935138	0.109949	0.1359741
1747.106	15	0.397676	0.3626353	0.1303319	0.08973278	0.110247	0.1373687
1747.206	15	0.393196	0.3509688	0.1259733	0.09002363	0.111315	0.139536
1747.306	15	0.407352	0.353319	0.1239374	0.0878059	0.112555	0.1416237
1747.406	15	0.396693	0.356193	0.1250361	0.08862644	0.112503	0.1445254
1747.506	15	0.403309	0.3503044	0.1254414	0.09006541	0.11523	0.1417406
1747.606	15	0.395491	0.3585959	0.1243666	0.09199712	0.116322	0.1392731
1747.706	15	0.385839	0.359733	0.1258775	0.09226854	0.116558	0.1407749
1747.806	15	0.394558	0.3648759	0.1290742	0.09160595	0.114577	0.1402346
1747.906	15	0.387226	0.3663778	0.1277774	0.0939573	0.116461	0.141463
1748.006	15	0.374602	0.3614649	0.1286693	0.0920043	0.118994	0.1409587
1748.106	15	0.370141	0.3581616	0.1262437	0.09082861	0.12148	0.1388079
1748.206	15	0.368267	0.3586525	0.1257477	0.09182719	0.122113	0.1374758
1748.306	15	0.370131	0.3612052	0.1253767	0.08934804	0.121616	0.1361317
1748.406	15	0.378035	0.3429114	0.1232106	0.09114452	0.126214	0.13765
1748.506	15	0.383296	0.3531058	0.1252865	0.09479342	0.125382	0.1394032
1748.606	15	0.378407	0.3432951	0.1270422	0.0933169	0.12442	0.1382161
1748.706	15	0.39182	0.3591878	0.1251373	0.09472936	0.124131	0.1346437
1748.806	15	0.418044	0.3490446	0.1248528	0.09590198	0.123212	0.1356013
1748.906	15	0.417265	0.3309117	0.123255	0.09707092	0.122314	0.1350104
1749.006	15	0.413038	0.343699	0.1241262	0.09787316	0.126069	0.1346392
1749.106	15	0.413858	0.3462291	0.1252688	0.09756657	0.127084	0.1356931
1749.206	15	0.411518	0.344047	0.1290096	0.09618496	0.122247	0.1360519
1749.306	15	0.402968	0.3498908	0.1300136	0.09751311	0.121229	0.1348244
1749.406	15	0.39831	0.354876	0.131468	0.09413128	0.120821	0.1376525
1749.506	15	0.387143	0.3554488	0.1315821	0.09477782	0.121553	0.136466
1749.606	15	0.39922	0.3599395	0.1330139	0.09640885	0.121996	0.1378972
1749.706	15	0.398833	0.3489014	0.1337787	0.09594765	0.120271	0.1393407
1749.806	15	0.371633	0.351191	0.1372835	0.09705339	0.119262	0.1397594
1749.906	15	0.392193	0.3609065	0.1364487	0.09610634	0.118866	0.1356521
1750.006	15	0.395866	0.3577316	0.1395297	0.09635783	0.120038	0.1342857
1750.106	15	0.397297	0.3593054	0.1390484	0.09624463	0.117149	0.1340807
1750.206	15	0.392808	0.3592066	0.1402816	0.09573707	0.115804	0.1337129
1750.306	15	0.385821	0.3526676	0.1397599	0.09577443	0.115125	0.135469
1750.406	15	0.388503	0.356793	0.1350889	0.09349539	0.115174	0.136718
1750.506	15	0.394497	0.3524042	0.1332765	0.09323362	0.113679	0.1365588
1750.606	15	0.40086	0.3536482	0.1326531	0.09242478	0.113196	0.1352411
1750.706	15	0.386544	0.3572356	0.1309055	0.09113424	0.113642	0.1337491
1750.806	15	0.381479	0.3505949	0.1277909	0.0924114	0.112503	0.1320659
1750.906	15	0.39255	0.3559512	0.1273376	0.09306383	0.110487	0.1309069
1751.006	15	0.383153	0.3610557	0.1286938	0.09254826	0.109076	0.1306452
1751.106	15	0.39413	0.3628645	0.1280074	0.09208053	0.107398	0.130861
1751.206	15	0.391748	0.356201	0.1267969	0.09053043	0.106545	0.1299776
1751.306	15	0.38899	0.3665294	0.1273381	0.08703125	0.106777	0.1330793
1751.406	15	0.386393	0.3651398	0.1292179	0.08452107	0.111425	0.1343291
1751.506	15	0.388201	0.3676708	0.1298832	0.0871672	0.114597	0.1343202
1751.606	15	0.384877	0.3583407	0.127117	0.09002402	0.11482	0.1372846
1751.706	15	0.381285	0.3598976	0.1262527	0.09107478	0.116501	0.1387236
1751.806	15	0.387223	0.3657638	0.1238726	0.09097537	0.116981	0.1410219
1751.906	15	0.383305	0.3644848	0.1230842	0.0911128	0.115584	0.1427301
1752.006	15	0.37621	0.3672895	0.1216344	0.09210403	0.111547	0.1442264
1752.106	15	0.371807	0.3691701	0.1210833	0.09247634	0.110955	0.1444342
1752.206	15	0.37066	0.365057	0.1251839	0.09419366	0.115204	0.1407624
1752.306	15	0.3742	0.3747596	0.124492	0.09171285	0.119302	0.1385665
1752.406	15	0.380009	0.3684704	0.1268487	0.09083707	0.123621	0.1393355
1752.506	15	0.388177	0.3752511	0.1263554	0.09514244	0.124586	0.140555
1752.606	15	0.382597	0.3766727	0.1231489	0.09577673	0.126184	0.1406713
1752.706	15	0.404124	0.3750681	0.1228627	0.09459049	0.125913	0.1379551
1752.806	15	0.415335	0.3739631	0.1204434	0.0965985	0.121914	0.1407006
1752.906	15	0.417579	0.3717386	0.1216002	0.09581347	0.118178	0.1401016
1753.006	15	0.428136	0.3677078	0.1229174	0.09853451	0.118334	0.1384971
1753.106	15	0.418052	0.3721085	0.1209128	0.09766094	0.122539	0.1378395
1753.206	15	0.420101	0.3674161	0.1223603	0.09663503	0.124275	0.1379463
1753.306	15	0.406609	0.362088	0.1213981	0.09567682	0.125878	0.1371069
1753.406	15	0.399192	0.3603125	0.1206046	0.0927339	0.124533	0.138483
1753.506	15	0.391392	0.3577316	0.1208805	0.09399967	0.12132	0.1372428
1753.606	15	0.395126	0.3696549	0.1236834	0.0945064	0.119299	0.1359864
1753.706	15	0.386126	0.3690563	0.1235632	0.09112853	0.1192	0.1360687
1753.806	15	0.366108	0.3462074	0.123549	0.09320176	0.119895	0.1351988
1753.906	15	0.379443	0.3584851	0.1258942	0.09228578	0.119962	0.1356177
1754.006	15	0.387568	0.3676418	0.1304929	0.09225097	0.117594	0.1364309
1754.106	15	0.392693	0.3656214	0.1349909	0.09340156	0.116312	0.1366664
1754.206	15	0.390159	0.3671881	0.1370852	0.09282581	0.117372	0.1366263
1754.306	15	0.385592	0.3679927	0.1358016	0.0928114	0.117851	0.1358244
1754.406	15	0.387096	0.3694448	0.1365974	0.09122635	0.117093	0.1362179
1754.506	15	0.40157	0.3530774	0.1398815	0.09056257	0.117068	0.1371339
1754.606	15	0.400676	0.348797	0.1395626	0.09140184	0.113858	0.1386305
1754.706	15	0.382302	0.3535442	0.137818	0.09138392	0.11063	0.1377773
1754.806	15	0.379197	0.3519746	0.1346576	0.08997274	0.110788	0.1370385
1754.906	15	0.392097	0.3512385	0.1323089	0.08923398	0.10991	0.1390755
1755.006	15	0.383938	0.3589502	0.1319681	0.08857147	0.109979	0.137758
1755.106	15	0.393159	0.3631509	0.1330986	0.0915527	0.107805	0.1384012
1755.206	15	0.390029	0.3591153	0.1311714	0.09003922	0.106211	0.1384516
1755.306	15	0.38796	0.3615805	0.12811	0.08716343	0.107761	0.1390145
1755.406	15	0.388823	0.3644758	0.1292824	0.08516283	0.106829	0.138596
1755.506	15	0.39598	0.3568625	0.1269586	0.0860213	0.10627	0.1373239
1755.606	15	0.391008	0.3595313	0.1268997	0.08923563	0.107596	0.1350236
1755.706	15	0.389075	0.3638455	0.1265095	0.09021246	0.108036	0.1337584
1755.806	15	0.395261	0.3527866	0.1273952	0.08975174	0.108679	0.1347883
1755.906	15	0.388863	0.373523	0.1292078	0.08909876	0.107629	0.139143

1756.006	15	0.380862	0.3585797	0.1282002	0.0877715	0.107574	0.1398692
1756.106	15	0.372467	0.3684658	0.128285	0.08853176	0.108292	0.140732
1756.206	15	0.370673	0.3649753	0.1274019	0.09209798	0.111272	0.141987
1756.306	15	0.373151	0.3676047	0.1274924	0.09099784	0.113505	0.1413871
1756.406	15	0.382936	0.3713783	0.1278932	0.09085698	0.115151	0.1434674
1756.506	15	0.384783	0.3680137	0.1255946	0.09341346	0.119007	0.1459796
1756.606	15	0.381813	0.365096	0.1261884	0.09421244	0.120105	0.1461292
1756.706	15	0.385464	0.3607011	0.1265965	0.09384769	0.123801	0.1450967
1756.806	15	0.398179	0.361248	0.127106	0.0932853	0.123773	0.1447078
1756.906	15	0.40724	0.3647473	0.1269096	0.09402415	0.123025	0.1437344
1757.006	15	0.408089	0.3698868	0.1276701	0.09640478	0.119848	0.1435699
1757.106	15	0.409927	0.3782296	0.1300708	0.09621993	0.11813	0.1425898
1757.206	15	0.411988	0.3764375	0.1297923	0.09588675	0.119637	0.1426592
1757.306	15	0.403127	0.3630071	0.1301617	0.09663422	0.123362	0.1410645
1757.406	15	0.400683	0.3570529	0.1302899	0.09851974	0.123485	0.1419401
1757.506	15	0.393187	0.3674675	0.1291067	0.1025599	0.120958	0.1420519
1757.606	15	0.396292	0.3693043	0.1262723	0.1023333	0.121196	0.1418702
1757.706	15	0.391321	0.361783	0.1271312	0.0979955	0.119267	0.1423211
1757.806	15	0.3642	0.3599729	0.1250775	0.09556477	0.117973	0.1420803
1757.906	15	0.385422	0.3640333	0.1249467	0.09360558	0.119056	0.1383046
1758.006	15	0.391618	0.3582259	0.1270298	0.09116867	0.119242	0.1389173
1758.106	15	0.395902	0.3602788	0.1288413	0.09145385	0.118153	0.1413399
1758.206	15	0.38879	0.3586409	0.1286276	0.09102671	0.117776	0.1416725
1758.306	15	0.385122	0.3607968	0.1325592	0.08878745	0.116843	0.1407761
1758.406	15	0.38502	0.3626856	0.13104	0.08780383	0.11513	0.1408102
1758.506	15	0.400411	0.36185	0.1332122	0.08783503	0.11555	0.1394644
1758.606	15	0.397746	0.3681353	0.1361268	0.09015077	0.110591	0.1384622
1758.706	15	0.381678	0.3686636	0.1355542	0.09049433	0.108734	0.1347165
1758.806	15	0.382191	0.369973	0.1382045	0.08995243	0.110804	0.1345406
1758.906	15	0.394149	0.3628609	0.1416168	0.08593939	0.109372	0.1347687
1759.006	15	0.387564	0.3529255	0.140376	0.08498938	0.110287	0.1364217
1759.106	15	0.395108	0.3639336	0.138941	0.08881736	0.112081	0.1356508
1759.206	15	0.38616	0.3534548	0.1388227	0.09059954	0.109842	0.137237
1759.306	15	0.388832	0.3595536	0.1337999	0.08886086	0.109805	0.1347219
1759.406	15	0.385168	0.3572193	0.1300501	0.08350006	0.109265	0.1337527
1759.506	15	0.39001	0.3514358	0.1314556	0.08195467	0.109255	0.1324584
1759.606	15	0.384838	0.3612378	0.1320048	0.08675171	0.111351	0.133339
1759.706	15	0.38659	0.3614206	0.1280824	0.08929291	0.110957	0.1344631
1759.806	15	0.393689	0.3616977	0.1291369	0.0906574	0.110656	0.1315454
1759.906	15	0.386	0.3672739	0.1278257	0.08740091	0.113238	0.1332654
1760.006	15	0.381754	0.3596463	0.1271779	0.0868828	0.112458	0.1299703
1760.106	15	0.372323	0.3579044	0.1265254	0.08874787	0.113714	0.1281689
1760.206	15	0.373972	0.3598414	0.1259983	0.09272737	0.114268	0.1313478
1760.306	15	0.374746	0.3571351	0.1260783	0.09515303	0.117106	0.1323414
1760.406	15	0.385397	0.3436587	0.1244787	0.09389655	0.119093	0.1325237
1760.506	15	0.386265	0.3520523	0.1242742	0.09417515	0.121622	0.1350594
1760.606	15	0.375846	0.3475126	0.1246978	0.09241297	0.126699	0.1342926
1760.706	15	0.391428	0.3571114	0.1249168	0.09266224	0.128694	0.1351296
1760.806	15	0.400638	0.3445181	0.1247435	0.09372079	0.129137	0.1365364
1760.906	15	0.410565	0.3323587	0.1222354	0.09511917	0.127315	0.1387355
1761.006	15	0.411516	0.3430429	0.1232943	0.09936075	0.124695	0.1401536
1761.106	15	0.412798	0.3431759	0.1284512	0.09897251	0.12096	0.1410132
1761.206	15	0.406737	0.3446056	0.127743	0.0995723	0.119211	0.1417563
1761.306	15	0.39647	0.3486768	0.1275257	0.1021892	0.117701	0.1441617
1761.406	15	0.397369	0.3525134	0.1271928	0.1036191	0.119942	0.143967
1761.506	15	0.39361	0.3543452	0.1265425	0.1053846	0.119071	0.1441734
1761.606	15	0.396748	0.3573089	0.1266859	0.104592	0.119552	0.1420337
1761.706	15	0.394803	0.3474702	0.1237506	0.1002805	0.120519	0.1402842
1761.806	15	0.368535	0.3493566	0.1236753	0.09881546	0.120627	0.1408924
1761.906	15	0.382735	0.3590661	0.1261272	0.09772918	0.118021	0.1425672
1762.006	15	0.3899	0.3547983	0.1227047	0.09737221	0.119761	0.1415871
1762.106	15	0.392548	0.3607539	0.1245729	0.09610394	0.118041	0.1403024
1762.206	15	0.388498	0.361316	0.125273	0.09653516	0.116742	0.1382854
1762.306	15	0.388908	0.3484341	0.1243781	0.09529637	0.115046	0.1383702
1762.406	15	0.390393	0.3557147	0.1262609	0.09383184	0.115879	0.1388003
1762.506	15	0.397234	0.3557594	0.1286776	0.09465342	0.115896	0.1380665
1762.606	15	0.394905	0.3523215	0.1276834	0.09265615	0.116085	0.1354882
1762.706	15	0.38801	0.3554089	0.128746	0.09193406	0.11375	0.136639
1762.806	15	0.384792	0.3520285	0.1306459	0.09260874	0.111	0.1393276
1762.906	15	0.395085	0.3633524	0.1314007	0.09321401	0.111609	0.1373757
1763.006	15	0.393206	0.3658487	0.1309304	0.09400864	0.108287	0.1359369
1763.106	15	0.400915	0.3625829	0.1329103	0.0936951	0.109439	0.1335726
1763.206	15	0.395502	0.355149	0.1365074	0.09267119	0.111541	0.1336534
1763.306	15	0.400308	0.3627818	0.1355396	0.09129906	0.110063	0.1367123
1763.406	15	0.396398	0.3640493	0.1344009	0.08885301	0.111661	0.1349113
1763.506	15	0.406093	0.3684849	0.1394747	0.08888821	0.111212	0.1344327
1763.606	15	0.400947	0.3576407	0.1403695	0.09243467	0.109611	0.1328072
1763.706	15	0.390495	0.3607961	0.1350929	0.09223276	0.107695	0.1343672
1763.806	15	0.405522	0.365171	0.13046	0.09255442	0.106176	0.1340673
1763.906	15	0.401868	0.3634729	0.127542	0.09400676	0.10814	0.1348238
1764.006	15	0.386432	0.3635671	0.1284663	0.09256949	0.108834	0.1352387
1764.106	15	0.376143	0.356165	0.1271679	0.09411407	0.11343	0.1341281
1764.206	15	0.374503	0.3635001	0.1276831	0.09262349	0.116242	0.131619
1764.306	15	0.37262	0.3787591	0.1276785	0.09412695	0.11942	0.1313805
1764.406	15	0.383408	0.3710903	0.1226433	0.09416109	0.118172	0.1322071
1764.506	15	0.391597	0.3736315	0.1231662	0.09861971	0.114874	0.1331532
1764.606	15	0.385571	0.3774269	0.1242171	0.09681612	0.119621	0.1310775
1764.706	15	0.390802	0.3576835	0.1247048	0.09636458	0.117115	0.1347585
1764.806	15	0.401892	0.3711954	0.125639	0.09545625	0.119047	0.1341759
1764.906	15	0.411236	0.3732603	0.1249312	0.0975105	0.120694	0.1334354
1765.006	15	0.410595	0.3673395	0.1248408	0.09952711	0.121059	0.1324861
1765.106	15	0.408907	0.374032	0.124218	0.09892805	0.121109	0.1353022
1765.206	15	0.425185	0.3669585	0.1246526	0.09939833	0.122197	0.1378623
1765.306	15	0.436218	0.3626418	0.1239603	0.1034718	0.123352	0.1408693
1765.406	15	0.441395	0.3595798	0.1221442	0.1035712	0.121885	0.1409102

1765.506	15	0.414618	0.3635752	0.1240133	0.1065371	0.119273	0.1404013
1765.606	15	0.41605	0.3668146	0.1264016	0.1044234	0.120876	0.1444603
1765.706	15	0.407066	0.3703275	0.1253544	0.1000409	0.121494	0.1451172
1765.806	15	0.375513	0.3487997	0.1237465	0.09837333	0.121503	0.1441536
1765.906	15	0.388454	0.3586906	0.1224029	0.09743767	0.122188	0.1436897
1766.006	15	0.392532	0.3639597	0.1257312	0.0974585	0.119331	0.1422688
1766.106	15	0.393042	0.3601936	0.1215182	0.09685856	0.11814	0.1392968
1766.206	15	0.390545	0.3660138	0.1206441	0.09514926	0.116826	0.1389266
1766.306	15	0.389057	0.3669734	0.122831	0.09411441	0.116917	0.1402462
1766.406	15	0.392283	0.3667898	0.1213767	0.09256183	0.114605	0.1407896
1766.506	15	0.393203	0.3493265	0.1212536	0.09180689	0.114468	0.1384083
1766.606	15	0.393479	0.3584845	0.1202263	0.09288916	0.116461	0.1362886
1766.706	15	0.385384	0.3574169	0.1184942	0.09288887	0.115716	0.137756
1766.806	15	0.383222	0.3553125	0.1210517	0.0911448	0.114136	0.1376362
1766.906	15	0.396887	0.3551075	0.1223873	0.09189977	0.11268	0.136534
1767.006	15	0.399684	0.36129	0.1232464	0.09076563	0.113974	0.1368263
1767.106	15	0.403051	0.3622209	0.1219478	0.09251004	0.112899	0.1355325
1767.206	15	0.409747	0.3581737	0.1246099	0.09036655	0.111461	0.1349554
1767.306	15	0.411876	0.3605957	0.125577	0.08844236	0.111273	0.1368495
1767.406	15	0.397106	0.3636813	0.1270905	0.09102177	0.113194	0.1372025
1767.506	15	0.412834	0.3559325	0.1286513	0.09120701	0.111405	0.1365203
1767.606	15	0.404964	0.3570527	0.1278901	0.09423095	0.111713	0.1368075
1767.706	15	0.403767	0.3610022	0.1300158	0.09318781	0.109411	0.1357405
1767.806	15	0.418901	0.3531314	0.1336826	0.08990382	0.108123	0.1366677
1767.906	15	0.40197	0.3745203	0.1365652	0.08737226	0.107808	0.1374087
1768.006	15	0.387285	0.3579428	0.1370969	0.08870076	0.10859	0.1350157
1768.106	15	0.377924	0.3694334	0.136724	0.08985241	0.107426	0.1361582
1768.206	15	0.379363	0.3678769	0.1328098	0.08820654	0.107006	0.1342167
1768.306	15	0.374877	0.3698687	0.1301936	0.09076934	0.112412	0.1380897
1768.406	15	0.380996	0.3722662	0.1304425	0.09272055	0.11696	0.1403651
1768.506	15	0.387961	0.3586599	0.1303762	0.09394209	0.11494	0.1381631
1768.606	15	0.381728	0.3574199	0.129685	0.09349907	0.116857	0.1371606
1768.706	15	0.389064	0.3647756	0.1304544	0.09532665	0.117787	0.1348788
1768.806	15	0.405641	0.3708653	0.1275498	0.09519222	0.119907	0.1341189
1768.906	15	0.412471	0.3770119	0.1266156	0.09896842	0.1214	0.1320078
1769.006	15	0.411627	0.3670063	0.1219773	0.1004073	0.123428	0.1339048
1769.106	15	0.420583	0.35641	0.1224658	0.09774174	0.128365	0.1341051
1769.206	15	0.417883	0.3597868	0.1244627	0.09407009	0.12921	0.1346755
1769.306	15	0.405781	0.366945	0.1268983	0.09656931	0.128121	0.1346215
1769.406	15	0.404615	0.3634997	0.1261815	0.09707647	0.125157	0.1365102
1769.506	15	0.40102	0.3583277	0.1260594	0.09773513	0.125554	0.1380433
1769.606	15	0.396683	0.3601348	0.124843	0.09840314	0.123104	0.1382509
1769.706	15	0.392571	0.3556106	0.1253597	0.09635376	0.110371	0.1393
1769.806	15	0.365342	0.3562981	0.1246656	0.09477768	0.109257	0.1394122
1769.906	15	0.395042	0.3563911	0.1247826	0.0924831	0.113427	0.1390334
1770.006	15	0.406742	0.3640837	0.128796	0.09497579	0.117099	0.1401695
1770.106	15	0.403434	0.3644806	0.1274052	0.09435639	0.117513	0.1424008
1770.206	15	0.399115	0.3656834	0.1271225	0.0945653	0.112227	0.1423876
1770.306	15	0.399695	0.363345	0.1270689	0.09365672	0.1087	0.1435892
1770.406	15	0.398517	0.3682957	0.1283819	0.09278071	0.107396	0.1464691
1770.506	15	0.406778	0.3629032	0.1281927	0.09180371	0.107631	0.1420279
1770.606	15	0.406119	0.3557675	0.1264432	0.09202152	0.106648	0.1394158
1770.706	15	0.384288	0.3635597	0.1264856	0.09236126	0.110627	0.1386097
1770.806	15	0.387984	0.3523796	0.1285689	0.09008238	0.113481	0.1395371
1770.906	15	0.39637	0.358643	0.1281	0.09062885	0.114485	0.1401287
1771.006	15	0.403244	0.3510015	0.1291178	0.08826309	0.114204	0.1408067
1771.106	15	0.421405	0.3538911	0.1278978	0.08905324	0.11429	0.1373379
1771.206	15	0.418284	0.3584168	0.1255109	0.08673456	0.118002	0.1394279
1771.306	15	0.420625	0.3649808	0.1231341	0.08818792	0.122735	0.1405039
1771.406	15	0.393783	0.3642628	0.1254294	0.08762986	0.120912	0.1406997
1771.506	15	0.399918	0.3610038	0.1272637	0.08754961	0.124374	0.1408114
1771.606	15	0.382291	0.3589807	0.1282296	0.08842286	0.12519	0.1411851
1771.706	15	0.393345	0.359782	0.1276103	0.08768038	0.123229	0.1432886
1771.806	15	0.399113	0.3581842	0.1279501	0.08996868	0.121917	0.1437741
1771.906	15	0.381721	0.348183	0.1322072	0.08889036	0.117701	0.1416092
1772.006	15	0.382907	0.3404188	0.1338422	0.08770781	0.120175	0.1411121
1772.106	15	0.373644	0.3553063	0.1360665	0.08556844	0.122687	0.1396819
1772.206	15	0.370738	0.3543652	0.1397336	0.08537976	0.122771	0.1384706
1772.306	15	0.369013	0.3293841	0.1421493	0.0838602	0.12162	0.1382515
1772.406	15	0.3799	0.3443525	0.1457656	0.08665796	0.120784	0.1390177
1772.506	15	0.3861	0.3419424	0.1452539	0.08652757	0.121288	0.1381827
1772.606	15	0.383364	0.3428796	0.1405908	0.08782772	0.121063	0.1361901
1772.706	15	0.390842	0.3484304	0.1356661	0.08790494	0.122838	0.1379975
1772.806	15	0.398648	0.3561016	0.1343559	0.08775625	0.119755	0.1391981
1772.906	15	0.406118	0.3605448	0.132744	0.09068095	0.115939	0.1370478
1773.006	15	0.405213	0.3512623	0.1327082	0.09353791	0.114903	0.1397167
1773.106	15	0.403887	0.3427791	0.1328099	0.09607074	0.11166	0.136901
1773.206	15	0.406347	0.3535676	0.1334742	0.1007367	0.114398	0.133833
1773.306	15	0.402813	0.3548964	0.1298849	0.1014914	0.114815	0.1317908
1773.406	15	0.403765	0.3617836	0.1289964	0.1017832	0.111826	0.1351815
1773.506	15	0.39821	0.3597488	0.1280548	0.1011578	0.109884	0.1353698
1773.606	15	0.395149	0.3484663	0.1298823	0.0978822	0.108421	0.1353814
1773.706	15	0.388948	0.3569791	0.1317463	0.09631015	0.107716	0.1338171
1773.806	15	0.365065	0.3526558	0.1306046	0.09474079	0.106734	0.1353846
1773.906	15	0.380566	0.3487685	0.1298051	0.0950796	0.10613	0.1395079
1774.006	15	0.384793	0.3551874	0.1279357	0.0979557	0.106677	0.1415321
1774.106	15	0.390658	0.3555339	0.1253017	0.09584564	0.106867	0.1425014
1774.206	15	0.389963	0.3691497	0.1255241	0.09241652	0.106632	0.1424024
1774.306	15	0.397453	0.3639745	0.1240665	0.09381483	0.105142	0.1419347
1774.406	15	0.395436	0.3531488	0.1276692	0.09332047	0.106602	0.1432066
1774.506	15	0.394997	0.3647001	0.1306232	0.09195444	0.109241	0.1442005
1774.606	15	0.404509	0.3644077	0.1294037	0.09365782	0.108858	0.1416971
1774.706	15	0.381915	0.3657569	0.1307978	0.09263552	0.109388	0.1418912
1774.806	15	0.386578	0.3556127	0.1307799	0.09182198	0.114724	0.1434803
1774.906	15	0.393651	0.3671381	0.1304507	0.08943031	0.119492	0.140966

1775.006	15	0.393393	0.3627559	0.1275877	0.08765871	0.118258	0.1356577
1775.106	15	0.410177	0.3664747	0.1268975	0.08990066	0.119163	0.1369493
1775.206	15	0.399426	0.3702913	0.1273992	0.08995801	0.119237	0.1371407
1775.306	15	0.397951	0.3598656	0.12581	0.08873312	0.121096	0.1369064
1775.406	15	0.386962	0.3707186	0.1268929	0.08819117	0.121703	0.137428
1775.506	15	0.393998	0.3694183	0.1281584	0.08568571	0.123299	0.1367233
1775.606	15	0.384853	0.3722775	0.1258056	0.08612935	0.120704	0.135034
1775.706	15	0.394433	0.3716537	0.1237917	0.08713962	0.122413	0.1376223
1775.806	15	0.395318	0.3683782	0.1239226	0.08885878	0.12182	0.1380193
1775.906	15	0.382271	0.366699	0.1264387	0.09135142	0.120446	0.1384611
1776.006	15	0.386337	0.3669353	0.1260772	0.09059601	0.120267	0.1365173
1776.106	15	0.371426	0.3662744	0.128406	0.09075548	0.121433	0.1368835
1776.206	15	0.364428	0.3664696	0.1283133	0.09035227	0.123658	0.135549
1776.306	15	0.370062	0.3585198	0.1294791	0.08907289	0.12451	0.1361315
1776.406	15	0.383439	0.3577112	0.1322998	0.09137537	0.126129	0.1324272
1776.506	15	0.38721	0.355159	0.1331281	0.0924761	0.124294	0.134337
1776.606	15	0.379224	0.3631147	0.132851	0.09321412	0.12139	0.1361316
1776.706	15	0.391467	0.3626778	0.1347979	0.09096637	0.120329	0.1347202
1776.806	15	0.411902	0.3521492	0.1337754	0.08885904	0.120192	0.1354503
1776.906	15	0.408375	0.3604531	0.1366127	0.08775853	0.116998	0.1336446
1777.006	15	0.406189	0.3639337	0.1365492	0.09044219	0.115202	0.1352154
1777.106	15	0.407401	0.3661342	0.1306544	0.0905976	0.1148	0.1369359
1777.206	15	0.411805	0.3621895	0.1294324	0.09190562	0.115997	0.1384223
1777.306	15	0.401343	0.3673913	0.1285145	0.0962617	0.117736	0.1389396
1777.406	15	0.400759	0.3577181	0.129183	0.0980027	0.11549	0.1371751
1777.506	15	0.394562	0.3533019	0.1292149	0.1006317	0.112157	0.136312
1777.606	15	0.395642	0.3586926	0.1299085	0.09954991	0.111483	0.1347188
1777.706	15	0.391544	0.3513975	0.1271694	0.09645988	0.111079	0.1340179
1777.806	15	0.364099	0.3524117	0.1291465	0.09669182	0.110759	0.133522
1777.906	15	0.375112	0.3557699	0.1296112	0.09847869	0.110185	0.1326244
1778.006	15	0.383541	0.3649961	0.1306981	0.09813212	0.110558	0.1333494
1778.106	15	0.385888	0.3592536	0.1321634	0.09768351	0.109621	0.1336114
1778.206	15	0.395041	0.362651	0.1311832	0.09771813	0.110083	0.1356291
1778.306	15	0.397848	0.356786	0.1292224	0.09383724	0.109018	0.1341626
1778.406	15	0.39444	0.3580323	0.1283042	0.09036565	0.111407	0.1347411
1778.506	15	0.393298	0.3640285	0.126371	0.0881962	0.110493	0.1335745
1778.606	15	0.407187	0.3581536	0.1279736	0.08831144	0.110782	0.1334031
1778.706	15	0.38161	0.3740698	0.1253435	0.08916619	0.113221	0.139809
1778.806	15	0.386004	0.3608421	0.1260851	0.08920537	0.114425	0.1398028
1778.906	15	0.394101	0.3671038	0.1317451	0.08783114	0.114521	0.1394819
1779.006	15	0.399412	0.3661165	0.1292291	0.08551989	0.113487	0.1389866
1779.106	15	0.410202	0.3712763	0.1280426	0.08887244	0.114957	0.1393816
1779.206	15	0.401956	0.3717271	0.1273657	0.08809245	0.118171	0.1396539
1779.306	15	0.394596	0.3654911	0.1261638	0.08712384	0.118358	0.140684
1779.406	15	0.384708	0.357905	0.1249162	0.08767164	0.122641	0.1366121
1779.506	15	0.393343	0.3578542	0.1254763	0.08697733	0.12571	0.1338659
1779.606	15	0.387282	0.3636285	0.1266456	0.08836442	0.125703	0.13375
1779.706	15	0.388164	0.3722872	0.1277061	0.0896183	0.124997	0.1339446
1779.806	15	0.388918	0.3780319	0.1282422	0.09158318	0.123932	0.1328571
1779.906	15	0.38081	0.3714505	0.1288604	0.09287691	0.121811	0.1342911
1780.006	15	0.384147	0.3574976	0.1270025	0.0905728	0.119181	0.1327464
1780.106	15	0.369999	0.3577534	0.1269425	0.09118662	0.120623	0.1357197
1780.206	15	0.36854	0.3666763	0.1276452	0.09082311	0.121426	0.1384859
1780.306	15	0.375784	0.3645463	0.1284787	0.09134737	0.120288	0.1353144
1780.406	15	0.385334	0.359862	0.1291999	0.09293641	0.118127	0.1362938
1780.506	15	0.388722	0.3596065	0.1287663	0.09344871	0.115881	0.1349971
1780.606	15	0.383665	0.3541532	0.1282322	0.09249396	0.114113	0.1338127
1780.706	15	0.389334	0.357674	0.127241	0.09255194	0.118326	0.1343394
1780.806	15	0.407823	0.3581877	0.1312062	0.0926215	0.118856	0.1317326
1780.906	15	0.41319	0.3633825	0.1341498	0.09399001	0.118199	0.1316065
1781.006	15	0.42312	0.3641844	0.1329937	0.09550087	0.117667	0.1306997
1781.106	15	0.412785	0.3625065	0.1345277	0.09818361	0.114629	0.1336889
1781.206	15	0.417469	0.3650263	0.1343787	0.09838979	0.11727	0.1328164
1781.306	15	0.406282	0.3681204	0.1342398	0.1024838	0.118496	0.1327188
1781.406	15	0.40473	0.3631757	0.135584	0.1028289	0.115094	0.1353051
1781.506	15	0.39464	0.3530818	0.1329287	0.102833	0.115196	0.1375955
1781.606	15	0.398149	0.3658268	0.1307734	0.104488	0.114936	0.1368525
1781.706	15	0.393604	0.3497742	0.1274548	0.0998088	0.114535	0.1348716
1781.806	15	0.364898	0.3566498	0.1272504	0.09772992	0.112966	0.1343607
1781.906	15	0.3798	0.3567596	0.1226436	0.09785962	0.110501	0.1338832
1782.006	15	0.384579	0.3479806	0.120856	0.09758206	0.11219	0.1328938
1782.106	15	0.386573	0.3542179	0.1232852	0.09697768	0.111944	0.1335628
1782.206	15	0.395991	0.3587074	0.1233941	0.0963575	0.11097	0.1321539
1782.306	15	0.401171	0.3586718	0.1242082	0.09448361	0.111785	0.1311312
1782.406	15	0.39763	0.3625014	0.1221252	0.09399084	0.110864	0.1330319
1782.506	15	0.397678	0.3515981	0.1235901	0.09173159	0.113489	0.1352705
1782.606	15	0.413201	0.3571333	0.1260891	0.09060125	0.111933	0.1376807
1782.706	15	0.384241	0.3628201	0.1262488	0.09213636	0.110704	0.1367052
1782.806	15	0.392166	0.3425163	0.1267568	0.09346758	0.109576	0.1367812
1782.906	15	0.397503	0.3467873	0.1228378	0.09356232	0.114402	0.1368399
1783.006	15	0.408432	0.3471384	0.1207892	0.0916267	0.117	0.1375387
1783.106	15	0.422696	0.3553783	0.1224728	0.09093961	0.117097	0.1382506
1783.206	15	0.427874	0.329891	0.1198079	0.08822382	0.119175	0.1406915
1783.306	15	0.417846	0.3416742	0.1251032	0.08915874	0.119394	0.1380212
1783.406	15	0.392635	0.3399881	0.1265104	0.08870802	0.121848	0.1401313
1783.506	15	0.406787	0.341438	0.1278736	0.08756389	0.124253	0.1389364
1783.606	15	0.390176	0.3482755	0.1261514	0.08704174	0.123324	0.1376537
1783.706	15	0.387389	0.3550802	0.1268151	0.0876279	0.121234	0.1360093
1783.806	15	0.402246	0.3600312	0.1264467	0.09110574	0.119129	0.1345746
1783.906	15	0.388349	0.3558791	0.1245192	0.09239714	0.116927	0.1326181
1784.006	15	0.385503	0.345506	0.1223716	0.09254645	0.11506	0.1339367
1784.106	15	0.371414	0.3522037	0.1252744	0.09281638	0.117248	0.1350072
1784.206	15	0.368533	0.3565593	0.1245252	0.09346622	0.11988	0.1323822
1784.306	15	0.37611	0.3576461	0.1291065	0.09148202	0.121771	0.1350927
1784.406	15	0.385524	0.3660998	0.1267317	0.09104419	0.120054	0.1357826

1784.506	15	0.390679	0.3512211	0.123824	0.089492	0.118765	0.1366294
1784.606	15	0.383828	0.3558004	0.1233689	0.09035432	0.118689	0.1355627
1784.706	15	0.390125	0.3538135	0.1240105	0.09183114	0.119602	0.1349851
1784.806	15	0.40746	0.3538187	0.1249795	0.09285626	0.118898	0.1384265
1784.906	15	0.422957	0.3572386	0.1229316	0.09531885	0.117138	0.1385905
1785.006	15	0.416919	0.3586514	0.1227253	0.09807441	0.115594	0.1389692
1785.106	15	0.411641	0.3678884	0.1201923	0.09927542	0.114916	0.1384893
1785.206	15	0.416083	0.3621915	0.1255798	0.09676614	0.114609	0.1369756
1785.306	15	0.404907	0.3541466	0.1308195	0.09875508	0.115548	0.1343244
1785.406	15	0.408788	0.3625295	0.1318801	0.09796832	0.113211	0.1334144
1785.506	15	0.421166	0.3625678	0.1342675	0.09815839	0.111222	0.1348621
1785.606	15	0.442996	0.3657936	0.136316	0.09773555	0.106933	0.1330961
1785.706	15	0.425225	0.3558462	0.1353208	0.09386854	0.108884	0.1337307
1785.806	15	0.413545	0.364444	0.1353436	0.09252205	0.111501	0.1341244
1785.906	15	0.416316	0.3645404	0.1365116	0.09305379	0.111447	0.1354821
1786.006	15	0.417267	0.3710208	0.1343798	0.09121553	0.10966	0.1357444
1786.106	15	0.396534	0.3736809	0.1320138	0.09072152	0.107703	0.1359896
1786.206	15	0.393692	0.3554871	0.1304908	0.08847539	0.108156	0.1372935
1786.306	15	0.396944	0.3710966	0.1302882	0.08698849	0.109271	0.1366551
1786.406	15	0.391905	0.3698949	0.1272028	0.08757018	0.109438	0.1367476
1786.506	15	0.38962	0.3734342	0.1294855	0.08763006	0.113236	0.1346805
1786.606	15	0.397539	0.3728862	0.1285472	0.0878107	0.11177	0.1339403
1786.706	15	0.385528	0.3698482	0.129044	0.08734207	0.111013	0.1329427
1786.806	15	0.387243	0.3673226	0.1288082	0.08753118	0.114108	0.1318433
1786.906	15	0.398207	0.3705537	0.1294729	0.088897	0.115369	0.1329809
1787.006	15	0.39631	0.3693698	0.1284434	0.08573392	0.11373	0.1344294
1787.106	15	0.406317	0.3690827	0.1277745	0.08791839	0.115074	0.1348342
1787.206	15	0.399707	0.3586241	0.1273117	0.08780374	0.116273	0.1341707
1787.306	15	0.399267	0.3608119	0.1274731	0.08437433	0.117613	0.1351605
1787.406	15	0.389643	0.3560852	0.1260363	0.08390334	0.119083	0.1355089
1787.506	15	0.398097	0.3650506	0.124401	0.08474939	0.121955	0.1370328
1787.606	15	0.392684	0.3669233	0.1218431	0.08857612	0.125227	0.1379704
1787.706	15	0.387915	0.3550251	0.1215206	0.08920357	0.122134	0.1375052
1787.806	15	0.399543	0.3618073	0.1251507	0.092135	0.119539	0.1351658
1787.906	15	0.390347	0.3640448	0.1279819	0.09252363	0.117129	0.1346015
1788.006	15	0.380297	0.3680849	0.1273249	0.0933542	0.117035	0.1367164
1788.106	15	0.3777	0.3625805	0.1229615	0.08976368	0.118317	0.1402449
1788.206	15	0.383689	0.3684551	0.1237772	0.09042831	0.118917	0.1413741
1788.306	15	0.388303	0.3648989	0.1238687	0.09046641	0.120054	0.1403733
1788.406	15	0.387874	0.3494233	0.1224832	0.08904503	0.117353	0.1393517
1788.506	15	0.390288	0.3581333	0.1227192	0.08985306	0.116171	0.1396578
1788.606	15	0.387732	0.3576864	0.1249804	0.093263	0.116516	0.1371281
1788.706	15	0.395155	0.3563291	0.1236866	0.09576656	0.119322	0.1368373
1788.806	15	0.420094	0.3587985	0.1237112	0.09412178	0.117971	0.1372729
1788.906	15	0.417704	0.3663356	0.1210971	0.09390073	0.115361	0.1339498
1789.006	15	0.411957	0.3567654	0.1201233	0.0973229	0.113838	0.137628
1789.106	15	0.403782	0.3626804	0.12192	0.09827389	0.114548	0.1388174
1789.206	15	0.416717	0.3607396	0.123064	0.09719781	0.112136	0.1391875
1789.306	15	0.399048	0.3583883	0.125443	0.0980468	0.111445	0.1379163
1789.406	15	0.404368	0.3622149	0.1269216	0.0995852	0.110469	0.1373942
1789.506	15	0.412208	0.3524063	0.1247926	0.0975137	0.108194	0.1376067
1789.606	15	0.449969	0.3760467	0.1254231	0.0937474	0.106479	0.1363121
1789.706	15	0.434577	0.3570437	0.1283657	0.093668	0.106739	0.1377063
1789.806	15	0.415387	0.3660481	0.1327001	0.09501583	0.106926	0.1373024
1789.906	15	0.413035	0.366229	0.1326576	0.09588768	0.10705	0.1366585
1790.006	15	0.421965	0.3696125	0.1360559	0.09601238	0.110237	0.1397205
1790.106	15	0.390387	0.3695477	0.1347152	0.09577563	0.114771	0.1398765
1790.206	15	0.390253	0.3644269	0.1350487	0.0964345	0.11334	0.1364255
1790.306	15	0.396627	0.3561708	0.1364049	0.0930597	0.113152	0.1360811
1790.406	15	0.393149	0.3536119	0.1335474	0.09142787	0.112266	0.1370031
1790.506	15	0.401731	0.3623376	0.1320533	0.09098046	0.114261	0.1364892
1790.606	15	0.402994	0.3697541	0.1308991	0.09328232	0.113854	0.1368457
1790.706	15	0.391691	0.3809066	0.1325845	0.09306204	0.115191	0.1350593
1790.806	15	0.390266	0.3752601	0.1302091	0.09068152	0.111472	0.1365602
1790.906	15	0.397256	0.3585451	0.1288203	0.09178603	0.112403	0.1363441
1791.006	15	0.394336	0.3580215	0.1302689	0.09049345	0.115347	0.1372225
1791.106	15	0.405654	0.3697907	0.1289837	0.08957802	0.116143	0.1321624
1791.206	15	0.400792	0.3666847	0.1275771	0.08781302	0.11585	0.1298839
1791.306	15	0.401486	0.3593052	0.1261333	0.0854106	0.112475	0.1276737
1791.406	15	0.386077	0.3566448	0.1259443	0.08567627	0.116593	0.1330001
1791.506	15	0.395224	0.3531783	0.1260738	0.08380978	0.118831	0.1354373
1791.606	15	0.381532	0.357688	0.1264718	0.08721454	0.122498	0.1355493
1791.706	15	0.388096	0.3568178	0.1249589	0.08804991	0.120951	0.1366459
1791.806	15	0.390574	0.3641816	0.1226481	0.08838177	0.120699	0.1371887
1791.906	15	0.383185	0.3663221	0.1217675	0.08903317	0.119878	0.1405859
1792.006	15	0.387711	0.3631038	0.1229631	0.08974129	0.120939	0.1420094
1792.106	15	0.379346	0.363764	0.1215301	0.08810977	0.122645	0.1437112
1792.206	15	0.39628	0.3693089	0.1245692	0.09157762	0.12233	0.1429781
1792.306	15	0.425548	0.3660972	0.1256327	0.08889367	0.121184	0.1405987
1792.406	15	0.409261	0.3583143	0.1274666	0.09008411	0.118968	0.1405473
1792.506	15	0.409429	0.3673944	0.1231737	0.09216755	0.118627	0.1424631
1792.606	15	0.408775	0.352488	0.1215242	0.09385051	0.117073	0.1425425
1792.706	15	0.413136	0.3572594	0.1214589	0.09693071	0.117243	0.1403857
1792.806	15	0.423951	0.3555216	0.1202875	0.09673713	0.119228	0.1389608
1792.906	15	0.421203	0.3512689	0.1190849	0.09554332	0.117427	0.1410269
1793.006	15	0.417012	0.3596744	0.1197862	0.09687185	0.11501	0.1418933
1793.106	15	0.409604	0.3594752	0.1190535	0.09828965	0.114481	0.1409085
1793.206	15	0.415912	0.3607033	0.1214421	0.09853192	0.114525	0.140752
1793.306	15	0.405662	0.3593807	0.1220616	0.09815708	0.112922	0.1387815
1793.406	15	0.408917	0.3568882	0.1212423	0.09994441	0.111148	0.138851
1793.506	15	0.412486	0.3602156	0.1202597	0.1031523	0.112618	0.1401326
1793.606	15	0.426907	0.3599241	0.1228196	0.1001851	0.110323	0.137065
1793.706	15	0.405505	0.3399548	0.120514	0.09707428	0.108449	0.1345147
1793.806	15	0.398235	0.3437523	0.1221622	0.09849591	0.108319	0.1345192
1793.906	15	0.421487	0.3526012	0.1243083	0.09877937	0.108496	0.1357577

1794.006	15	0.439179	0.3515447	0.1252256	0.09622046	0.110231	0.1378955
1794.106	15	0.396172	0.3260041	0.127297	0.09600174	0.111399	0.1393698
1794.206	15	0.39917	0.3446653	0.1312546	0.09735547	0.109332	0.1391889
1794.306	15	0.402654	0.3428771	0.13397	0.09703317	0.108695	0.1380825
1794.406	15	0.398286	0.3400931	0.1349894	0.09349873	0.110258	0.1397231
1794.506	15	0.401443	0.3478727	0.1377229	0.09521166	0.111802	0.1359807
1794.606	15	0.404828	0.3557409	0.1361803	0.09600621	0.113685	0.1363955
1794.706	15	0.392568	0.3599205	0.1394469	0.09422095	0.116374	0.1332681
1794.806	15	0.392644	0.355457	0.1364567	0.09221527	0.116626	0.133588
1794.906	15	0.401102	0.3487448	0.1330638	0.08870577	0.119168	0.1337948
1795.006	15	0.39626	0.3551567	0.1323368	0.08719756	0.116953	0.1364108
1795.106	15	0.411699	0.3612382	0.1310982	0.08973776	0.119021	0.1360253
1795.206	15	0.404062	0.3616972	0.1311945	0.08897725	0.123779	0.1366598
1795.306	15	0.397438	0.3702427	0.1289181	0.08949254	0.122938	0.1338597
1795.406	15	0.389036	0.3531902	0.1287482	0.08891784	0.122086	0.1339333
1795.506	15	0.397321	0.3567203	0.1271469	0.0881533	0.127021	0.1341553
1795.606	15	0.385949	0.3549008	0.1266145	0.08968498	0.129033	0.131875
1795.706	15	0.393565	0.3544951	0.1256971	0.08992376	0.123176	0.1323127
1795.806	15	0.395741	0.3593765	0.127328	0.09033105	0.118222	0.1328485
1795.906	15	0.384066	0.359034	0.1280479	0.09010203	0.118792	0.1335438
1796.006	15	0.391718	0.3710668	0.1299102	0.08931496	0.119696	0.1336582
1796.106	15	0.379603	0.364455	0.1296676	0.08826849	0.120008	0.1339797
1796.206	15	0.373516	0.3538387	0.1289639	0.08827051	0.118353	0.1339314
1796.306	15	0.385932	0.3647428	0.1276268	0.08844662	0.117784	0.1374375
1796.406	15	0.384856	0.3664448	0.1281192	0.08775728	0.114051	0.1375224
1796.506	15	0.397088	0.3684731	0.1270171	0.09045748	0.113141	0.1410211
1796.606	15	0.39056	0.3576113	0.1263228	0.09131929	0.113927	0.1406801
1796.706	15	0.399534	0.3657717	0.1283252	0.09046968	0.113541	0.1408454
1796.806	15	0.41159	0.3646342	0.1303834	0.0937905	0.115656	0.1426967
1796.906	15	0.416598	0.3717663	0.1300007	0.09912406	0.113923	0.1426414
1797.006	15	0.414393	0.3765956	0.1287854	0.1021786	0.113654	0.1423496
1797.106	15	0.406349	0.3675622	0.1294067	0.1021755	0.116527	0.1406539
1797.206	15	0.415351	0.3743386	0.1279945	0.1047976	0.116481	0.1386479
1797.306	15	0.408344	0.3720586	0.1248613	0.1026819	0.115197	0.137061
1797.406	15	0.429231	0.3797127	0.1246087	0.1022343	0.114068	0.1383835
1797.506	15	0.438974	0.376399	0.1233934	0.1037097	0.112266	0.1375615
1797.606	15	0.44973	0.3757908	0.1219918	0.1009428	0.110375	0.133789
1797.706	15	0.43195	0.372776	0.1231323	0.09458797	0.109869	0.1364782
1797.806	15	0.405785	0.3736759	0.1206149	0.09618656	0.111436	0.1318643
1797.906	15	0.426053	0.3717557	0.1189977	0.0960585	0.110068	0.135742
1798.006	15	0.396583	0.3734232	0.1191691	0.09777827	0.107646	0.1378004
1798.106	15	0.393146	0.362914	0.1212732	0.09687006	0.108062	0.1364675
1798.206	15	0.397905	0.3621652	0.1223849	0.09654525	0.107405	0.1365276
1798.306	15	0.399013	0.3565841	0.1239823	0.09249845	0.107427	0.1389396
1798.406	15	0.391936	0.3679667	0.1238723	0.09178633	0.10934	0.1415223
1798.506	15	0.39332	0.3691648	0.1286181	0.09049542	0.109478	0.1426019
1798.614	15	0.398429	0.3590455	0.1328644	0.09110051	0.108353	0.1426191
1798.714	15.5	0.39553	0.3638634	0.1349748	0.0904326	0.104954	0.1420196
1798.814	15.5	0.403151	0.3669407	0.1394494	0.08842439	0.108451	0.1422569
1798.914	15.5	0.400154	0.368304	0.1404131	0.08784711	0.108588	0.1403644
1799.014	15.5	0.396067	0.3629383	0.1406898	0.08494627	0.110587	0.1396521
1799.114	15.5	0.393878	0.3668264	0.1398109	0.08314893	0.115285	0.139211
1799.214	15.5	0.396666	0.3663521	0.1379997	0.08296202	0.118704	0.1410088
1799.314	15.5	0.389372	0.3513623	0.1338372	0.08377036	0.120187	0.1400654
1799.414	15.5	0.397774	0.3608935	0.1331582	0.08712396	0.123196	0.1382957
1799.514	15.5	0.400045	0.3590095	0.1336429	0.08775979	0.124911	0.1397115
1799.614	15.5	0.389953	0.3596002	0.1324521	0.08688372	0.12703	0.1381318
1799.714	15.5	0.39091	0.36175	0.1303805	0.08765077	0.125022	0.1355935
1799.814	15.5	0.383761	0.3650648	0.1290078	0.08794406	0.122031	0.1320882
1799.914	15.5	0.412803	0.3589972	0.1248524	0.08723897	0.121239	0.1310123
1800.014	15.5	0.445819	0.3651935	0.125553	0.08971261	0.121365	0.1296508
1800.114	15.5	0.432963	0.3622574	0.1250507	0.08952266	0.123708	0.1277735
1800.214	15.5	0.425392	0.3593943	0.1280332	0.09133452	0.122179	0.1263545
1800.314	15.5	0.464661	0.3669999	0.129123	0.09388176	0.120035	0.128947
1800.414	15.5	0.450504	0.3568105	0.1278402	0.09159203	0.117284	0.1309033
1800.514	15.5	0.479203	0.3776675	0.1267299	0.09473363	0.116524	0.1337338
1800.614	15.5	0.452585	0.3600998	0.1274445	0.09721955	0.115749	0.1341281
1800.714	15.5	0.458244	0.3683646	0.1275017	0.1003506	0.116135	0.1371445
1800.814	15.5	0.434983	0.3685021	0.1287521	0.1047698	0.114774	0.1410087
1800.914	15.5	0.424412	0.3729966	0.1282062	0.1038904	0.113244	0.1391318
1801.014	15.5	0.433263	0.370826	0.1300087	0.09805866	0.112089	0.1414579
1801.114	15.5	0.421841	0.3651536	0.1283637	0.09833419	0.112675	0.1395021
1801.214	15.5	0.424443	0.3580407	0.1266939	0.0978703	0.114019	0.1401623
1801.314	15.5	0.407197	0.3573321	0.1290167	0.09214442	0.113918	0.1384252
1801.414	15.5	0.393524	0.3611025	0.1236742	0.09391829	0.111588	0.1417103
1801.514	15.5	0.406488	0.3705172	0.1215238	0.09493797	0.109331	0.1441409
1801.614	15.5	0.424839	0.3808011	0.1239541	0.09439015	0.107353	0.1408848
1801.714	15.5	0.397758	0.375478	0.122508	0.09398985	0.105521	0.140107
1801.814	15.5	0.398173	0.355913	0.1232207	0.09829043	0.104681	0.1385395
1801.914	15.5	0.404677	0.3571511	0.1244484	0.09682501	0.103631	0.137129
1802.014	15.5	0.388334	0.3685122	0.1223084	0.09532838	0.103167	0.1358853
1802.114	15.5	0.404045	0.3662428	0.1213836	0.095574	0.104631	0.1355464
1802.214	15.5	0.394287	0.3594522	0.1239915	0.09330168	0.105009	0.1365722
1802.314	15.5	0.396431	0.3568237	0.1260651	0.09359711	0.104422	0.1389314
1802.414	15.5	0.393301	0.3541599	0.1288658	0.09143147	0.106412	0.1401983
1802.514	15.5	0.400647	0.3581555	0.1296359	0.08993463	0.107035	0.1388396
1802.614	15.5	0.40159	0.3571625	0.1303603	0.08739071	0.106498	0.1371142
1802.714	15.5	0.404703	0.3611509	0.1326324	0.08696815	0.107854	0.1368322
1802.814	15.5	0.39925	0.3647084	0.1347404	0.08758797	0.112691	0.139025
1802.914	15.5	0.395058	0.3626864	0.1361871	0.08785374	0.113719	0.1417147
1803.014	15.5	0.399832	0.3660184	0.1394083	0.08686877	0.112996	0.1417538
1803.114	15.5	0.393423	0.3684438	0.1388828	0.08831947	0.113779	0.1406709
1803.214	15.5	0.394071	0.3662302	0.1388037	0.09137248	0.117335	0.1395749
1803.314	15.5	0.397556	0.355542	0.1369461	0.09199312	0.121215	0.1404438
1803.414	15.5	0.394757	0.3655654	0.1354742	0.09145673	0.12203	0.1390374

1803.514	15.5	0.393799	0.3547017	0.1345707	0.09276744	0.123158	0.1383617
1803.614	15.5	0.372728	0.3587135	0.13283	0.09442584	0.12364	0.1339512
1803.714	15.5	0.381895	0.3596913	0.1312566	0.09265611	0.123265	0.1337176
1803.814	15.5	0.405101	0.3530666	0.1292536	0.09130598	0.121567	0.1325733
1803.914	15.5	0.421936	0.3605894	0.1296374	0.09356477	0.117484	0.1329853
1804.014	15.5	0.4291	0.3598422	0.1275778	0.09198465	0.119642	0.1312632
1804.114	15.5	0.422241	0.3616363	0.1271007	0.09466017	0.123569	0.1320192
1804.214	15.5	0.431923	0.3629943	0.1259956	0.09739733	0.122656	0.1340822
1804.314	15.5	0.438611	0.3559634	0.1240673	0.09739435	0.121386	0.1316764
1804.414	15.5	0.429975	0.35966	0.125003	0.09869303	0.11793	0.1298617
1804.514	15.5	0.425218	0.3594609	0.1262357	0.1004791	0.116121	0.132905
1804.614	15.5	0.427613	0.343758	0.1290409	0.09946702	0.115097	0.1328757
1804.714	15.5	0.425208	0.3439504	0.1286574	0.09946377	0.115867	0.1330275
1804.814	15.5	0.423918	0.357929	0.1276228	0.1010885	0.116365	0.1313285
1804.914	15.5	0.427171	0.3603476	0.1277137	0.09737029	0.114967	0.1289228
1805.014	15.5	0.457114	0.3329233	0.1271741	0.09519713	0.113075	0.1281851
1805.114	15.5	0.453858	0.3511084	0.1279825	0.09462073	0.112854	0.1297139
1805.214	15.5	0.432928	0.3481931	0.1309683	0.09298818	0.114717	0.1295524
1805.314	15.5	0.442406	0.346426	0.1308114	0.0949342	0.112949	0.1317687
1805.414	15.5	0.455304	0.3528158	0.1303203	0.09515676	0.111393	0.1375923
1805.514	15.5	0.414608	0.3585156	0.130706	0.09459136	0.112152	0.1398531
1805.614	15.5	0.402545	0.3623313	0.1297536	0.09542611	0.111266	0.1395871
1805.714	15.5	0.407475	0.3539476	0.127502	0.09552615	0.109921	0.1397365
1805.814	15.5	0.398883	0.3507129	0.1278919	0.09487709	0.113507	0.1387645
1805.914	15.5	0.390202	0.3581181	0.1291072	0.0944742	0.113066	0.1399442
1806.014	15.5	0.404234	0.3597865	0.1263749	0.09381446	0.113216	0.1392859
1806.114	15.5	0.387309	0.3638593	0.1276893	0.09245628	0.115179	0.1332881
1806.214	15.5	0.394923	0.370508	0.1283565	0.09306932	0.116202	0.1332556
1806.314	15.5	0.396442	0.353122	0.1240852	0.0925293	0.115349	0.1358983
1806.414	15.5	0.394722	0.3613663	0.123013	0.09221085	0.112501	0.1342891
1806.514	15.5	0.406324	0.3554321	0.1223773	0.08957879	0.110244	0.1321299
1806.614	15.5	0.402633	0.3574958	0.122367	0.08821331	0.110946	0.1318973
1806.714	15.5	0.399985	0.3610416	0.1262773	0.08890937	0.109656	0.1314377
1806.814	15.5	0.391688	0.3613063	0.1250276	0.08837076	0.111033	0.1365727
1806.914	15.5	0.398672	0.3714735	0.1256252	0.08771939	0.114211	0.1331767
1807.014	15.5	0.388388	0.3648068	0.1285559	0.08934442	0.114591	0.1359497
1807.114	15.5	0.388414	0.3574155	0.1317976	0.09117696	0.118025	0.1377297
1807.214	15.5	0.399896	0.3686672	0.1380071	0.08963636	0.123885	0.1368358
1807.314	15.5	0.388477	0.3624654	0.1400909	0.08993851	0.123732	0.1382372
1807.414	15.5	0.38008	0.3677296	0.1398559	0.08800622	0.125369	0.1412847
1807.514	15.5	0.373437	0.3556131	0.1404355	0.08924783	0.130704	0.141114
1807.614	15.5	0.387071	0.3685844	0.1370938	0.0893525	0.131014	0.1395087
1807.714	15.5	0.418881	0.3661057	0.1309486	0.09097099	0.127711	0.1397818
1807.814	15.5	0.399553	0.3738561	0.1286338	0.09142569	0.126376	0.1380495
1807.914	15.5	0.41083	0.3786949	0.1300599	0.09071659	0.120673	0.1393781
1808.014	15.5	0.419563	0.371317	0.1274908	0.08997381	0.120576	0.1368879
1808.114	15.5	0.43188	0.3786486	0.1246331	0.09297063	0.12013	0.1353336
1808.214	15.5	0.446847	0.3722366	0.1252667	0.09250864	0.12183	0.1371531
1808.314	15.5	0.425663	0.3781414	0.1240408	0.09130149	0.12174	0.1359119
1808.414	15.5	0.41665	0.3772368	0.1245642	0.09552811	0.119675	0.1385648
1808.514	15.5	0.431623	0.3758454	0.125272	0.09769923	0.116202	0.1398961
1808.614	15.5	0.412858	0.3692072	0.126544	0.09763574	0.11521	0.1388631
1808.714	15.5	0.407152	0.3715241	0.127188	0.09822813	0.113665	0.1373718
1808.814	15.5	0.430146	0.369331	0.1285277	0.09565368	0.113131	0.1382461
1808.914	15.5	0.439092	0.3723477	0.1295152	0.0965344	0.113385	0.1322229
1809.014	15.5	0.413799	0.3627468	0.1304694	0.09796644	0.114842	0.1295355
1809.114	15.5	0.391032	0.363377	0.1301317	0.09381736	0.117275	0.1312371
1809.214	15.5	0.398842	0.3569705	0.1272704	0.09225822	0.118301	0.1332598
1809.314	15.5	0.423607	0.3699593	0.1266778	0.0918676	0.115942	0.1339643
1809.414	15.5	0.400202	0.3702713	0.1276099	0.09171914	0.113504	0.1333875
1809.514	15.5	0.402392	0.3582501	0.1272187	0.08933068	0.111956	0.133549
1809.614	15.5	0.394693	0.3653115	0.1274346	0.08808602	0.111377	0.1354788
1809.714	15.5	0.392123	0.368163	0.1265304	0.08991686	0.111312	0.1392841
1809.814	15.5	0.391146	0.3714721	0.1254665	0.09072419	0.110773	0.1414707
1809.914	15.5	0.398507	0.3655015	0.1262754	0.09121554	0.108408	0.1419883
1810.014	15.5	0.388651	0.3673113	0.1235547	0.09032657	0.109196	0.1393362
1810.114	15.5	0.392477	0.3704867	0.1222315	0.08674269	0.111396	0.1387044
1810.214	15.5	0.397346	0.3523664	0.1229966	0.08829071	0.113887	0.1391259
1810.314	15.5	0.397766	0.3581398	0.1231575	0.08725375	0.115081	0.1385967
1810.414	15.5	0.40986	0.3600312	0.123249	0.08762789	0.115385	0.1392246
1810.514	15.5	0.406444	0.3559395	0.1201702	0.08716123	0.115414	0.1353628
1810.614	15.5	0.407892	0.3605017	0.1168231	0.08661512	0.111867	0.1340508
1810.714	15.5	0.400465	0.3660986	0.1154165	0.08576972	0.114003	0.1370965
1810.814	15.5	0.398875	0.3581094	0.1193522	0.08519697	0.114173	0.1368949
1810.914	15.5	0.389522	0.3627913	0.1213153	0.08692022	0.11851	0.1368296
1811.014	15.5	0.399332	0.3608211	0.1229472	0.08780501	0.117821	0.1359736
1811.114	15.5	0.408404	0.3586982	0.1257061	0.08823624	0.117592	0.1362366
1811.214	15.5	0.385537	0.3683096	0.1252756	0.0866381	0.118943	0.1349798
1811.314	15.5	0.384131	0.3537844	0.1265647	0.08817171	0.120786	0.1351881
1811.414	15.5	0.371638	0.3787279	0.1276825	0.08780225	0.120134	0.1344113
1811.514	15.5	0.383005	0.3590375	0.1316691	0.0883342	0.123782	0.1346423
1811.614	15.5	0.383725	0.3661576	0.1368036	0.08820695	0.128984	0.1340372
1811.714	15.5	0.392624	0.3689362	0.139092	0.09000275	0.128482	0.1354466
1811.814	15.5	0.389662	0.374086	0.1395734	0.09200124	0.12468	0.1350248
1811.914	15.5	0.394675	0.3697847	0.1359373	0.09095726	0.121333	0.1359877
1812.014	15.5	0.406767	0.3656031	0.1307082	0.08877806	0.121719	0.1360919
1812.114	15.5	0.414471	0.3584482	0.1276358	0.08814674	0.123821	0.1378786
1812.214	15.5	0.408877	0.3582969	0.1291859	0.08987765	0.124744	0.1352627
1812.314	15.5	0.411827	0.3644367	0.1264999	0.09052516	0.121283	0.135751
1812.414	15.5	0.416882	0.3714865	0.1256116	0.09201577	0.119178	0.1357457
1812.514	15.5	0.410807	0.380022	0.1274792	0.09731361	0.119921	0.1333949
1812.614	15.5	0.41329	0.3775784	0.124519	0.09628619	0.118354	0.1357358
1812.714	15.5	0.413958	0.3574615	0.1251165	0.09701694	0.118802	0.1341763
1812.814	15.5	0.42932	0.3562535	0.1257129	0.09760997	0.117195	0.1363761
1812.914	15.5	0.40557	0.3709283	0.126529	0.09797309	0.115955	0.1365255

1813.014	15.5	0.382488	0.3672763	0.1272744	0.0972854	0.114011	0.1352063
1813.114	15.5	0.408598	0.360167	0.1265799	0.09680869	0.114305	0.132054
1813.214	15.5	0.437409	0.3590365	0.1244241	0.09720712	0.118844	0.131055
1813.314	15.5	0.401425	0.3562295	0.1233712	0.0946439	0.117737	0.1285618
1813.414	15.5	0.400138	0.3608477	0.1265136	0.09256539	0.114951	0.128638
1813.514	15.5	0.397039	0.3574401	0.126936	0.09106798	0.113092	0.1293212
1813.614	15.5	0.396036	0.3628193	0.1257539	0.09062091	0.113752	0.1315208
1813.714	15.5	0.405558	0.3652729	0.1285571	0.09241454	0.112455	0.1306383
1813.814	15.5	0.397471	0.363625	0.125787	0.09244573	0.11324	0.1296176
1813.914	15.5	0.390588	0.3669871	0.1275175	0.09253986	0.114067	0.1311288
1814.014	15.5	0.391845	0.3699378	0.1243292	0.09112555	0.113754	0.1299365
1814.114	15.5	0.401163	0.3673263	0.1240956	0.08982697	0.115063	0.1312371
1814.214	15.5	0.397893	0.3571387	0.1232526	0.09068218	0.116451	0.136705
1814.314	15.5	0.406413	0.3649442	0.1200278	0.08939469	0.116758	0.1396909
1814.414	15.5	0.400065	0.351318	0.1211071	0.08729462	0.118082	0.1400277
1814.514	15.5	0.391851	0.3592897	0.122184	0.08943669	0.117367	0.1425805
1814.614	15.5	0.399793	0.362027	0.121559	0.08608043	0.117457	0.1428869
1814.714	15.5	0.386769	0.34752	0.1231616	0.0848911	0.118866	0.1415479
1814.814	15.5	0.393741	0.3640245	0.121144	0.08681194	0.118815	0.1414607
1814.914	15.5	0.397187	0.3614819	0.1199774	0.08845003	0.117226	0.1377214
1815.014	15.5	0.386706	0.3632174	0.1201462	0.08754264	0.11926	0.1329592
1815.114	15.5	0.388263	0.3682478	0.121629	0.08808426	0.12126	0.1329138
1815.214	15.5	0.379937	0.3608707	0.1217423	0.08965976	0.118593	0.1317478
1815.314	15.5	0.372383	0.3616858	0.123258	0.08801834	0.118498	0.1298212
1815.414	15.5	0.387211	0.363171	0.1233763	0.08735054	0.120929	0.1280264
1815.514	15.5	0.386805	0.3468598	0.1236117	0.09095772	0.122798	0.1289983
1815.614	15.5	0.399025	0.3517009	0.1260664	0.09178933	0.123557	0.1302173
1815.714	15.5	0.388481	0.3456102	0.1298888	0.09396275	0.123499	0.1326416
1815.814	15.5	0.412933	0.3642889	0.1348509	0.09183346	0.121017	0.1316465
1815.914	15.5	0.434227	0.3395812	0.137149	0.09031793	0.121972	0.1338652
1816.014	15.5	0.427837	0.3484891	0.1358867	0.0923365	0.122193	0.1332991
1816.114	15.5	0.416555	0.3491019	0.1369887	0.0913858	0.120862	0.136058
1816.214	15.5	0.447287	0.3467352	0.1348748	0.09421302	0.122767	0.1386155
1816.314	15.5	0.435451	0.3495261	0.1305241	0.09630784	0.119567	0.1408529
1816.414	15.5	0.413323	0.356639	0.1293851	0.09559584	0.117195	0.1389
1816.514	15.5	0.423915	0.3604513	0.1282995	0.09434591	0.116998	0.1388052
1816.614	15.5	0.424036	0.3596776	0.1272374	0.09062867	0.116595	0.1393271
1816.714	15.5	0.42407	0.3520455	0.1257612	0.09414688	0.114906	0.1348571
1816.814	15.5	0.406363	0.3551379	0.1266816	0.0936792	0.112223	0.1365148
1816.914	15.5	0.396364	0.3596017	0.1235403	0.09238999	0.11157	0.1365688
1817.014	15.5	0.414043	0.3545097	0.1250961	0.09349061	0.109887	0.1358445
1817.114	15.5	0.407058	0.3726751	0.1271075	0.09412843	0.112555	0.1335444
1817.214	15.5	0.395056	0.3582648	0.1293733	0.09091923	0.114624	0.1332256
1817.314	15.5	0.394645	0.3571718	0.1291874	0.08841572	0.114859	0.135796
1817.414	15.5	0.393532	0.3581876	0.1274782	0.0860711	0.112447	0.1366027
1817.514	15.5	0.385896	0.3548508	0.1263339	0.08770242	0.110581	0.1369282
1817.614	15.5	0.398811	0.3596802	0.1267865	0.08719645	0.108791	0.1346069
1817.714	15.5	0.384852	0.3564626	0.1279406	0.08730724	0.10819	0.1338448
1817.814	15.5	0.391781	0.3715074	0.1272373	0.08845685	0.106252	0.1322722
1817.914	15.5	0.392483	0.3673283	0.1270128	0.09079349	0.106555	0.1327391
1818.014	15.5	0.398614	0.3614476	0.1300999	0.0899423	0.106236	0.1350462
1818.114	15.5	0.401891	0.3621156	0.1310135	0.09050156	0.108154	0.1330186
1818.214	15.5	0.401459	0.3684237	0.1317455	0.09051076	0.110643	0.129558
1818.314	15.5	0.40121	0.3681315	0.1279826	0.08800504	0.113184	0.1288131
1818.414	15.5	0.3925	0.3600674	0.1267009	0.08660159	0.113709	0.1263193
1818.514	15.5	0.396169	0.3628395	0.1224594	0.0861067	0.11388	0.12764
1818.614	15.5	0.388807	0.3683887	0.120229	0.08751976	0.112627	0.1308297
1818.714	15.5	0.397991	0.3703013	0.1204321	0.09014843	0.114035	0.1336854
1818.814	15.5	0.388947	0.3751252	0.1235577	0.08835274	0.114315	0.1376657
1818.914	15.5	0.382799	0.3744151	0.123529	0.0905055	0.114747	0.137207
1819.014	15.5	0.391462	0.3783822	0.1239681	0.09054947	0.116496	0.1392759
1819.114	15.5	0.380884	0.3769315	0.1222709	0.09134323	0.120713	0.1415767
1819.214	15.5	0.386835	0.3767875	0.1224905	0.09027047	0.118121	0.1422816
1819.314	15.5	0.398929	0.3782007	0.1208475	0.09088689	0.118461	0.1432296
1819.414	15.5	0.389699	0.3751924	0.1209122	0.09376943	0.120925	0.1364644
1819.514	15.5	0.404797	0.3749057	0.121076	0.09548823	0.123059	0.1341581
1819.614	15.5	0.415181	0.3721168	0.1233226	0.09546167	0.123677	0.133111
1819.714	15.5	0.420841	0.3669392	0.1249835	0.09288014	0.122307	0.1310136
1819.814	15.5	0.434249	0.3757517	0.1256168	0.09199735	0.12266	0.1292224
1819.914	15.5	0.451331	0.3642918	0.1272581	0.09288161	0.12182	0.1327628
1820.014	15.5	0.445395	0.3650927	0.128309	0.09615596	0.1213	0.1358459
1820.114	15.5	0.455631	0.3572838	0.1290172	0.09791909	0.122536	0.1360579
1820.214	15.5	0.446741	0.3720087	0.1323659	0.09913251	0.12278	0.1367846
1820.314	15.5	0.440037	0.370681	0.1310367	0.1000682	0.121524	0.1376535
1820.414	15.5	0.434063	0.3595205	0.1325925	0.1012032	0.119087	0.1375831
1820.514	15.5	0.459688	0.367726	0.1293171	0.09998555	0.117563	0.1375769
1820.614	15.5	0.447396	0.370933	0.1292366	0.09738395	0.119013	0.1384767
1820.714	15.5	0.434729	0.3692202	0.130276	0.09655524	0.119433	0.1382232
1820.814	15.5	0.437878	0.369181	0.1307955	0.0972155	0.116301	0.1353493
1820.914	15.5	0.457813	0.3685765	0.1263375	0.09751251	0.114515	0.1347969
1821.014	15.5	0.403134	0.3725266	0.1243977	0.09614763	0.113001	0.1327508
1821.114	15.5	0.398405	0.3535433	0.1276996	0.09501228	0.113572	0.1322324
1821.214	15.5	0.395109	0.3614355	0.1280191	0.0933796	0.115829	0.1305959
1821.314	15.5	0.394968	0.3599329	0.128024	0.09315792	0.117166	0.1337954
1821.414	15.5	0.392565	0.3582885	0.1286797	0.09307221	0.113037	0.1353302
1821.514	15.5	0.398003	0.3619773	0.1296681	0.09042382	0.111463	0.1325615
1821.614	15.5	0.390223	0.3651206	0.1282426	0.09043305	0.109654	0.1322031
1821.714	15.5	0.393812	0.3581159	0.1271697	0.09015067	0.109276	0.135172
1821.814	15.5	0.399125	0.3645791	0.1264935	0.09155449	0.111959	0.1334701
1821.914	15.5	0.398095	0.3639981	0.1246319	0.08976824	0.112072	0.131767
1822.014	15.5	0.408486	0.3583	0.1252473	0.08847026	0.113071	0.1316694
1822.114	15.5	0.401401	0.3642108	0.1248407	0.08776402	0.110527	0.1332604
1822.214	15.5	0.402182	0.3547304	0.1239652	0.08692655	0.114848	0.1324597
1822.314	15.5	0.391958	0.3750824	0.1264281	0.08739336	0.113659	0.1340136
1822.414	15.5	0.397351	0.3606737	0.1264813	0.08864744	0.115122	0.1349204

1822.514	15.5	0.390267	0.3669117	0.1253603	0.08887638	0.115695	0.1355179
1822.614	15.5	0.394794	0.3678212	0.1242977	0.08785528	0.113662	0.1385513
1822.714	15.5	0.392233	0.3731605	0.1233577	0.0876687	0.107999	0.1386967
1822.814	15.5	0.386181	0.3702239	0.1219096	0.0890556	0.1099	0.1413665
1822.914	15.5	0.39054	0.3674769	0.120087	0.09162699	0.113614	0.1429097
1823.014	15.5	0.386064	0.3610997	0.1212558	0.09277763	0.114554	0.1418516
1823.114	15.5	0.388883	0.3573835	0.1216335	0.09270771	0.120085	0.1423378
1823.214	15.5	0.393791	0.361333	0.1205554	0.09285559	0.120794	0.1424767
1823.314	15.5	0.405192	0.3679956	0.1209481	0.09131081	0.119342	0.1430525
1823.414	15.5	0.396774	0.3790461	0.1176561	0.09159493	0.119007	0.1382449
1823.514	15.5	0.396801	0.3809115	0.1206193	0.09169852	0.121046	0.1368242
1823.614	15.5	0.414725	0.3610553	0.1225378	0.09208287	0.122237	0.1366969
1823.714	15.5	0.432103	0.3562383	0.1243002	0.09403844	0.121981	0.1342635
1823.814	15.5	0.421394	0.3693914	0.1249968	0.09275284	0.120942	0.1320035
1823.914	15.5	0.414169	0.3679073	0.1260362	0.09244581	0.120566	0.1305447
1824.014	15.5	0.428917	0.3620949	0.1252323	0.09352297	0.120138	0.1305483
1824.114	15.5	0.414922	0.3569992	0.1284578	0.09532378	0.122478	0.1323115
1824.214	15.5	0.413003	0.3587736	0.1297497	0.09735422	0.12451	0.1317145
1824.314	15.5	0.413289	0.3617131	0.1309866	0.09574548	0.124919	0.131344
1824.414	15.5	0.432423	0.3577813	0.131907	0.100084	0.121739	0.1307385
1824.514	15.5	0.410199	0.3624916	0.1333832	0.1003949	0.121208	0.1355188
1824.614	15.5	0.402657	0.3658476	0.1329444	0.09909865	0.119725	0.1381311
1824.714	15.5	0.413363	0.36436	0.1326543	0.09738506	0.119626	0.1399394
1824.814	15.5	0.435345	0.3686992	0.1304126	0.09712154	0.116642	0.1393589
1824.914	15.5	0.397717	0.3674619	0.1271342	0.09668191	0.114363	0.1376787
1825.014	15.5	0.397165	0.37246	0.1258177	0.09448744	0.114933	0.1361425
1825.114	15.5	0.399269	0.3625784	0.1255648	0.09174196	0.116634	0.1339586
1825.214	15.5	0.395353	0.3620801	0.1218299	0.09169422	0.115607	0.1324449
1825.314	15.5	0.397171	0.3593827	0.1207009	0.09411959	0.116895	0.133178
1825.414	15.5	0.399522	0.3566365	0.1223373	0.09461471	0.113163	0.1341063
1825.514	15.5	0.389288	0.3593463	0.1219514	0.09281692	0.110209	0.1339673
1825.614	15.5	0.392113	0.3506086	0.1222099	0.093131	0.108691	0.1363372
1825.714	15.5	0.399791	0.3582942	0.1194587	0.09147232	0.109312	0.136318
1825.814	15.5	0.397191	0.3570445	0.1209846	0.08855449	0.110206	0.1341727
1825.914	15.5	0.407222	0.3607508	0.1217966	0.0878638	0.111206	0.1329365
1826.014	15.5	0.397793	0.3663942	0.1228799	0.08769944	0.111606	0.1350135
1826.114	15.5	0.393001	0.3587343	0.1231177	0.08458737	0.110075	0.133449
1826.214	15.5	0.393702	0.3546144	0.1250144	0.08418167	0.110403	0.1306348
1826.314	15.5	0.39766	0.3576457	0.1261383	0.08355036	0.110769	0.1290239
1826.414	15.5	0.392079	0.3401308	0.1246293	0.0865965	0.111564	0.1298862
1826.514	15.5	0.395891	0.3504076	0.1245205	0.0873393	0.113139	0.1293922
1826.614	15.5	0.393616	0.3433296	0.1279162	0.08656009	0.112454	0.129302
1826.714	15.5	0.386906	0.361637	0.1270211	0.08638538	0.114026	0.1290332
1826.814	15.5	0.388875	0.3404846	0.1259464	0.08704018	0.115543	0.1322746
1826.914	15.5	0.390505	0.3466791	0.1256195	0.08670929	0.118199	0.1333726
1827.014	15.5	0.425752	0.3521854	0.1259854	0.08751117	0.117275	0.1351791
1827.114	15.5	0.422081	0.348482	0.1213079	0.08966494	0.118751	0.1336109
1827.214	15.5	0.435929	0.3515319	0.1208397	0.0904822	0.120401	0.1381341
1827.314	15.5	0.420944	0.3591541	0.1218817	0.0908826	0.119599	0.1364527
1827.414	15.5	0.462512	0.3609019	0.1233031	0.08976417	0.121952	0.1350448
1827.514	15.5	0.43177	0.3642952	0.1219987	0.08905198	0.123543	0.1350599
1827.614	15.5	0.437361	0.3500951	0.1223529	0.09058148	0.126599	0.1366622
1827.714	15.5	0.444233	0.3547937	0.1203703	0.09435201	0.125111	0.1330088
1827.814	15.5	0.435507	0.3591902	0.1208842	0.09610011	0.122279	0.1314179
1827.914	15.5	0.435791	0.3547041	0.1206092	0.09719928	0.118069	0.1311966
1828.014	15.5	0.426471	0.3725782	0.1224639	0.0964283	0.119297	0.1299631
1828.114	15.5	0.433448	0.3593971	0.1236904	0.09299044	0.121857	0.1309892
1828.214	15.5	0.421944	0.3563218	0.1245045	0.09483545	0.123074	0.1293885
1828.314	15.5	0.429008	0.3582471	0.122547	0.09366411	0.124178	0.1295485
1828.414	15.5	0.405149	0.373413	0.1231639	0.09203176	0.1215	0.1339952
1828.514	15.5	0.387851	0.3684426	0.126156	0.09183041	0.119675	0.1350707
1828.614	15.5	0.405494	0.3573955	0.1296393	0.09405173	0.119636	0.1346074
1828.714	15.5	0.408366	0.3682249	0.1303955	0.09200566	0.12238	0.1313191
1828.814	15.5	0.394672	0.3656654	0.1325149	0.09064662	0.119054	0.1345384
1828.914	15.5	0.392869	0.3694275	0.132294	0.09003939	0.115299	0.1367607
1829.014	15.5	0.393289	0.3586776	0.1309649	0.08660466	0.114032	0.139649
1829.114	15.5	0.391746	0.3722635	0.128493	0.08864557	0.112664	0.1375246
1829.214	15.5	0.398281	0.3702641	0.1272232	0.09075198	0.11388	0.1363583
1829.314	15.5	0.389518	0.3743699	0.1262386	0.0911104	0.114519	0.1357769
1829.414	15.5	0.392253	0.38037	0.1261657	0.09184179	0.111628	0.1337192
1829.514	15.5	0.389932	0.3777924	0.1235693	0.09262379	0.111838	0.1329303
1829.614	15.5	0.39916	0.3772657	0.1237743	0.09058625	0.109344	0.1319288
1829.714	15.5	0.4018	0.3768249	0.1251658	0.08664432	0.109231	0.1323449
1829.814	15.5	0.40721	0.3794086	0.1260312	0.08794868	0.1106	0.1330588
1829.914	15.5	0.4015	0.3787378	0.1265991	0.08651103	0.109892	0.1324896
1830.014	15.5	0.393842	0.3694518	0.1249095	0.08703111	0.108574	0.1312736
1830.114	15.5	0.400405	0.3711902	0.1258454	0.08525845	0.104866	0.1314182
1830.214	15.5	0.393739	0.3740152	0.1268633	0.08589689	0.105476	0.1324991
1830.314	15.5	0.397997	0.368777	0.1255161	0.08763915	0.104339	0.1331523
1830.414	15.5	0.395342	0.3631174	0.1234546	0.08651143	0.107356	0.1320776
1830.514	15.5	0.390442	0.3581674	0.1250243	0.0871971	0.108192	0.129597
1830.614	15.5	0.384692	0.3688587	0.1280323	0.08813267	0.111167	0.129575
1830.714	15.5	0.387427	0.3702042	0.1277865	0.09070305	0.110968	0.1285122
1830.814	15.5	0.384942	0.3610561	0.126913	0.09258357	0.116713	0.1280541
1830.914	15.5	0.385005	0.3694452	0.1303839	0.08867454	0.116005	0.1310115
1831.014	15.5	0.38759	0.3704306	0.1314457	0.08808522	0.116356	0.1329835
1831.114	15.5	0.395873	0.3730665	0.129102	0.08997624	0.122834	0.1363763
1831.214	15.5	0.39062	0.3666792	0.1272122	0.08949023	0.124642	0.1363129
1831.314	15.5	0.409509	0.3720618	0.1256422	0.09109487	0.123306	0.1397439
1831.414	15.5	0.41716	0.359912	0.1256619	0.08892768	0.122736	0.142632
1831.514	15.5	0.410252	0.3598315	0.1254411	0.09112306	0.122473	0.145506
1831.614	15.5	0.411916	0.3571687	0.1263902	0.09487941	0.124751	0.1424999
1831.714	15.5	0.424401	0.3542849	0.1255514	0.09537216	0.127005	0.1391463
1831.814	15.5	0.415892	0.3589635	0.1260027	0.09660356	0.126696	0.1404415
1831.914	15.5	0.410076	0.3602142	0.1267436	0.09740116	0.123756	0.1440859

1832.014	15.5	0.414298	0.3605494	0.1241366	0.09627856	0.121393	0.1372003
1832.114	15.5	0.427807	0.363531	0.1231676	0.09716935	0.120221	0.132353
1832.214	15.5	0.422092	0.3574011	0.1228009	0.09741533	0.120428	0.1325248
1832.314	15.5	0.396754	0.3612667	0.1250122	0.09418881	0.119246	0.1315711
1832.414	15.5	0.387775	0.3603909	0.1243982	0.09250733	0.118347	0.1319692
1832.514	15.5	0.411185	0.3651129	0.1240472	0.09267952	0.117243	0.1309577
1832.614	15.5	0.401743	0.362035	0.1230181	0.09141294	0.117024	0.129126
1832.714	15.5	0.398272	0.3635539	0.1243212	0.08973946	0.116894	0.1345452
1832.814	15.5	0.39478	0.3628266	0.1267369	0.08857756	0.117693	0.1351238
1832.914	15.5	0.393718	0.3677931	0.1269791	0.08575293	0.11651	0.1346745
1833.014	15.5	0.391225	0.3688562	0.1315403	0.08471889	0.112736	0.1333214
1833.114	15.5	0.400828	0.3628314	0.1303429	0.08584327	0.111028	0.1347525
1833.214	15.5	0.385424	0.3561503	0.1334078	0.08724913	0.109359	0.1364532
1833.314	15.5	0.392204	0.3572546	0.132648	0.08731594	0.109474	0.1384601
1833.414	15.5	0.397124	0.3649583	0.1285617	0.08827998	0.113802	0.1388274
1833.514	15.5	0.397335	0.3746845	0.1266671	0.08713831	0.112753	0.1377798
1833.614	15.5	0.411087	0.3780245	0.1274538	0.08595724	0.109133	0.1379398
1833.714	15.5	0.405734	0.3603523	0.1257775	0.08771537	0.112421	0.1384781
1833.814	15.5	0.39529	0.3556355	0.1211649	0.08513262	0.114066	0.1377563
1833.914	15.5	0.389345	0.368665	0.1228265	0.08367755	0.112424	0.1373451
1834.014	15.5	0.402125	0.3666256	0.1223295	0.08687439	0.112842	0.1385428
1834.114	15.5	0.391974	0.3602844	0.1236655	0.08703469	0.110835	0.1359446
1834.214	15.5	0.396016	0.35856	0.1251176	0.08884063	0.109453	0.1385304
1834.314	15.5	0.394978	0.3555544	0.1271995	0.08742275	0.111374	0.1376585
1834.414	15.5	0.388589	0.3595142	0.127014	0.08707243	0.109835	0.1383801
1834.514	15.5	0.388031	0.3575366	0.128585	0.08761357	0.112989	0.138098
1834.614	15.5	0.386882	0.3673994	0.1284363	0.08978906	0.114755	0.1393318
1834.714	15.5	0.382836	0.3641724	0.1287535	0.0920729	0.118581	0.135718
1834.814	15.5	0.395095	0.3653288	0.1287056	0.09095624	0.118348	0.1332368
1834.914	15.5	0.393965	0.3657405	0.1279826	0.08949886	0.115466	0.1345621
1835.014	15.5	0.39717	0.3687411	0.1286581	0.09030818	0.117427	0.1353446
1835.114	15.5	0.395506	0.3593934	0.1299111	0.09105641	0.118367	0.1337912
1835.214	15.5	0.414915	0.3641954	0.1327267	0.09329183	0.120195	0.1319809
1835.314	15.5	0.433621	0.3477399	0.1310217	0.08958302	0.11951	0.1310778
1835.414	15.5	0.422354	0.3577589	0.1311096	0.08732056	0.116315	0.133457
1835.514	15.5	0.422207	0.3527465	0.1287833	0.08806662	0.118563	0.1324131
1835.614	15.5	0.432969	0.3515142	0.1273687	0.09117568	0.121468	0.1349348
1835.714	15.5	0.427404	0.3589007	0.1258389	0.09475944	0.123422	0.1388622
1835.814	15.5	0.413809	0.3612359	0.123777	0.09555666	0.123007	0.1387797
1835.914	15.5	0.415472	0.3685306	0.123581	0.0967007	0.119126	0.1378602
1836.014	15.5	0.431807	0.35831	0.1241897	0.09411088	0.117429	0.1387883
1836.114	15.5	0.407997	0.3583319	0.1253179	0.09238412	0.117579	0.1396687
1836.214	15.5	0.379002	0.3631122	0.1260887	0.0896032	0.117686	0.144709
1836.314	15.5	0.395498	0.3384414	0.1221918	0.09128395	0.11697	0.1409386
1836.414	15.5	0.416166	0.3435533	0.1249682	0.09143914	0.116168	0.1398623
1836.514	15.5	0.394018	0.3507335	0.1274496	0.09066213	0.117185	0.1393595
1836.614	15.5	0.395903	0.3496634	0.1264703	0.09037635	0.11769	0.1382834
1836.714	15.5	0.389195	0.3381219	0.1275205	0.08441748	0.117928	0.1354896
1836.814	15.5	0.393299	0.347579	0.1291877	0.08357354	0.115432	0.1346323
1836.914	15.5	0.393731	0.3181782	0.1280259	0.08687767	0.111441	0.1383338
1837.014	15.5	0.395908	0.3412611	0.1293664	0.09101644	0.109745	0.1390653
1837.114	15.5	0.385885	0.3393379	0.1298992	0.09083515	0.107619	0.1406791
1837.214	15.5	0.393747	0.3616249	0.1308608	0.08960292	0.108498	0.140413
1837.314	15.5	0.397822	0.3596163	0.1347946	0.0890382	0.109485	0.1386143
1837.414	15.5	0.395097	0.3527102	0.1380417	0.08827142	0.10876	0.1394114
1837.514	15.5	0.40686	0.3587883	0.1383913	0.08909032	0.108528	0.1381349
1837.614	15.5	0.400936	0.3578994	0.1350048	0.08789227	0.110928	0.1371581
1837.714	15.5	0.394085	0.3681408	0.1279906	0.08854644	0.112236	0.1347801
1837.814	15.5	0.393421	0.3646904	0.1273659	0.08648507	0.111772	0.1322165
1837.914	15.5	0.398237	0.3553755	0.129005	0.08459403	0.110563	0.1323972
1838.014	15.5	0.393839	0.3570017	0.127136	0.08402909	0.109725	0.1307683
1838.114	15.5	0.394793	0.3562458	0.1235336	0.0867495	0.11388	0.1301538
1838.214	15.5	0.393723	0.3605968	0.1235264	0.08635956	0.114949	0.1336991
1838.314	15.5	0.387266	0.3591591	0.1215802	0.08466918	0.115888	0.1347072
1838.414	15.5	0.389262	0.3730465	0.1217168	0.08664378	0.116923	0.13377
1838.514	15.5	0.381254	0.3675722	0.1231443	0.08710703	0.117058	0.1353346
1838.614	15.5	0.384198	0.3568145	0.1242212	0.08721021	0.11902	0.1339218
1838.714	15.5	0.385921	0.3702781	0.1276281	0.08865223	0.123268	0.135147
1838.814	15.5	0.403502	0.3646373	0.128389	0.089559	0.126372	0.1350075
1838.914	15.5	0.402209	0.3676595	0.1264991	0.08777621	0.129038	0.135073
1839.014	15.5	0.451342	0.3594004	0.1243096	0.08621825	0.12605	0.1290068
1839.114	15.5	0.451715	0.3692581	0.1239275	0.08993275	0.125687	0.1281056
1839.214	15.5	0.44832	0.3690909	0.1232444	0.09197889	0.122916	0.1292301
1839.314	15.5	0.427992	0.3772244	0.1218524	0.09386472	0.11982	0.1298902
1839.414	15.5	0.415474	0.3768182	0.1247693	0.0927283	0.119763	0.1318044
1839.514	15.5	0.424344	0.37564	0.1259166	0.09152467	0.11936	0.1320813
1839.614	15.5	0.417991	0.3708366	0.1262382	0.09217064	0.121699	0.1316513
1839.714	15.5	0.427168	0.378237	0.1264778	0.09025388	0.119786	0.1333359
1839.814	15.5	0.41334	0.3792879	0.1274712	0.08970267	0.116237	0.1357222
1839.914	15.5	0.431184	0.3760687	0.1257796	0.0905908	0.115473	0.1368246
1840.014	15.5	0.414259	0.3685028	0.1233093	0.08892049	0.115924	0.1367946
1840.114	15.5	0.384333	0.3694017	0.1217949	0.08712419	0.117189	0.1387681
1840.214	15.5	0.4054	0.3749012	0.1214637	0.08507254	0.117109	0.1388138
1840.314	15.5	0.413057	0.3688326	0.1208707	0.08699502	0.11405	0.1407304
1840.414	15.5	0.391482	0.3640027	0.1216951	0.08651788	0.111773	0.1413155
1840.514	15.5	0.39446	0.3579604	0.1206223	0.08822656	0.114085	0.1406143
1840.614	15.5	0.391194	0.3690615	0.1179924	0.08569658	0.114262	0.1361923
1840.714	15.5	0.391881	0.3697685	0.1184228	0.08733957	0.112209	0.1366776
1840.814	15.5	0.397761	0.3582708	0.1214041	0.08567575	0.111455	0.1359255
1840.914	15.5	0.391802	0.3624935	0.1227613	0.08837386	0.112096	0.1356004
1841.014	15.5	0.386609	0.3679301	0.1264383	0.08796126	0.110566	0.1359196
1841.114	15.5	0.39124	0.3698619	0.1274037	0.08807544	0.109761	0.1335131
1841.214	15.5	0.395156	0.3680839	0.1271155	0.08893646	0.109261	0.131562
1841.314	15.5	0.397728	0.373355	0.126865	0.08929484	0.108222	0.1374541
1841.414	15.5	0.409087	0.3617359	0.130246	0.08913661	0.108348	0.135956

1841.514	15.5	0.401698	0.3649589	0.1336616	0.08429565	0.111925	0.1366866
1841.614	15.5	0.390527	0.3605451	0.1360472	0.08370273	0.110903	0.1354142
1841.714	15.5	0.396566	0.3575866	0.1344885	0.08342562	0.110949	0.137965
1841.814	15.5	0.392537	0.3612702	0.1337748	0.08066035	0.113715	0.135961
1841.914	15.5	0.394009	0.3662248	0.1321538	0.08275387	0.114228	0.1368532
1842.014	15.5	0.39684	0.3612261	0.1242423	0.08357867	0.115982	0.1362856
1842.114	15.5	0.392344	0.3652796	0.1230985	0.08576071	0.115989	0.132827
1842.214	15.5	0.384818	0.3582013	0.124272	0.08461524	0.115546	0.1327942
1842.314	15.5	0.386357	0.3629426	0.1218161	0.08645328	0.115909	0.1317159
1842.414	15.5	0.385039	0.3634774	0.118875	0.08782105	0.117554	0.1341461
1842.514	15.5	0.390919	0.3668047	0.1208327	0.0864405	0.121025	0.1333058
1842.614	15.5	0.423789	0.365373	0.1196469	0.08538605	0.121116	0.1318964
1842.714	15.5	0.410265	0.3650735	0.1195727	0.08430554	0.121844	0.1321041
1842.814	15.5	0.39233	0.3672113	0.1219703	0.08424959	0.123546	0.1364146
1842.914	15.5	0.408605	0.3720554	0.1238734	0.08611041	0.123268	0.1367122
1843.014	15.5	0.416897	0.371847	0.1250951	0.08524226	0.122722	0.1381425
1843.114	15.5	0.431847	0.3657377	0.125801	0.08566222	0.118406	0.1376098
1843.214	15.5	0.4526	0.3585312	0.1249248	0.08880731	0.116162	0.1374453
1843.314	15.5	0.458872	0.3588839	0.1232227	0.08836778	0.117931	0.1348229
1843.414	15.5	0.453743	0.3669544	0.1253306	0.08947526	0.120711	0.132953
1843.514	15.5	0.431564	0.3780427	0.127017	0.09094051	0.121567	0.131514
1843.614	15.5	0.432152	0.3800915	0.1265517	0.08907891	0.118935	0.1326266
1843.714	15.5	0.428843	0.3595515	0.1300543	0.09075492	0.116737	0.1334259
1843.814	15.5	0.421035	0.3580968	0.1305166	0.09322043	0.115416	0.1321539
1843.914	15.5	0.398157	0.3727446	0.1274336	0.09095063	0.116377	0.1328969
1844.014	15.5	0.397682	0.3698815	0.127241	0.08702136	0.116703	0.1360427
1844.114	15.5	0.435582	0.3614642	0.1272493	0.08563249	0.114717	0.140258
1844.214	15.5	0.416227	0.3609273	0.1278645	0.088237	0.114948	0.1428465
1844.314	15.5	0.395346	0.3552202	0.1254618	0.08954623	0.116472	0.1420653
1844.414	15.5	0.395187	0.3591127	0.1244656	0.08937003	0.115298	0.1426432
1844.514	15.5	0.395501	0.3606639	0.1262858	0.08915821	0.115132	0.141655
1844.614	15.5	0.389697	0.3659735	0.1232487	0.08932002	0.11151	0.1404595
1844.714	15.5	0.400035	0.3642905	0.1219959	0.08777644	0.110701	0.1392712
1844.814	15.5	0.383239	0.3661006	0.1206203	0.08841378	0.109624	0.1373246
1844.914	15.5	0.390901	0.3718263	0.1187736	0.08728839	0.106613	0.1370511
1845.014	15.5	0.394026	0.3722253	0.1190082	0.08639237	0.108536	0.1374823
1845.114	15.5	0.395491	0.3603899	0.1218142	0.08829604	0.110996	0.1349835
1845.214	15.5	0.400839	0.3676456	0.1210193	0.08788231	0.107902	0.1331876
1845.314	15.5	0.404228	0.3524156	0.1228852	0.08750086	0.107854	0.1339521
1845.414	15.5	0.398742	0.3645224	0.1247069	0.08536351	0.108433	0.1346206
1845.514	15.5	0.390607	0.3520446	0.1248592	0.08446564	0.109995	0.1350408
1845.614	15.5	0.394917	0.3629678	0.1248385	0.08332323	0.110304	0.1366924
1845.714	15.5	0.392787	0.3612436	0.1275639	0.0836321	0.112313	0.1349929
1845.814	15.5	0.395764	0.3613344	0.1317994	0.085116	0.11011	0.1355671
1845.914	15.5	0.394963	0.3672217	0.1338629	0.08355024	0.113071	0.1344841
1846.014	15.5	0.386991	0.3577017	0.1363385	0.08285932	0.115919	0.1348393
1846.114	15.5	0.386531	0.3585821	0.1364827	0.08322994	0.113781	0.1353622
1846.214	15.5	0.387787	0.3605882	0.1321751	0.08559509	0.114373	0.1357493
1846.314	15.5	0.379175	0.3444332	0.1260921	0.08439683	0.118059	0.1342662
1846.414	15.5	0.384931	0.3440211	0.1251611	0.08406774	0.121987	0.1347932
1846.514	15.5	0.38911	0.3586411	0.1289443	0.08377837	0.124451	0.136241
1846.614	15.5	0.39304	0.3483821	0.1265303	0.0841563	0.129256	0.1348868
1846.714	15.5	0.39444	0.3436982	0.1247843	0.08483975	0.129057	0.1342416
1846.814	15.5	0.415803	0.3507727	0.125562	0.08583178	0.127721	0.1335925
1846.914	15.5	0.425775	0.3175353	0.1229177	0.08421959	0.126442	0.1341744
1847.014	15.5	0.42377	0.3457436	0.1224466	0.08827369	0.120785	0.1327204
1847.114	15.5	0.428896	0.345666	0.1202021	0.0909728	0.120468	0.1350532
1847.214	15.5	0.428217	0.3621712	0.1243563	0.09130237	0.120895	0.1339909
1847.314	15.5	0.421946	0.3569164	0.1287367	0.08995824	0.121657	0.1343721
1847.414	15.5	0.414947	0.3510669	0.1292233	0.09098426	0.123379	0.1345343
1847.514	15.5	0.421454	0.3576637	0.1265027	0.09063361	0.119468	0.1326452
1847.614	15.5	0.424608	0.3582961	0.1261565	0.094168	0.117424	0.1320488
1847.714	15.5	0.407057	0.3692295	0.1264059	0.09335133	0.117099	0.1287633
1847.814	15.5	0.381535	0.3643707	0.1268263	0.09151884	0.117361	0.1284983
1847.914	15.5	0.391822	0.3555487	0.1259539	0.0903945	0.117696	0.1308449
1848.014	15.5	0.412375	0.3590822	0.1298345	0.09189783	0.115921	0.1325543
1848.114	15.5	0.391317	0.3566871	0.1315347	0.09311735	0.114496	0.1314231
1848.214	15.5	0.396499	0.3605867	0.1316437	0.09376947	0.112805	0.1325955
1848.314	15.5	0.384778	0.3582317	0.1303278	0.09238971	0.113868	0.1351928
1848.414	15.5	0.389848	0.3757547	0.1318351	0.09320422	0.11572	0.1347673
1848.514	15.5	0.387007	0.3674032	0.132606	0.0926148	0.111875	0.1362222
1848.614	15.5	0.394011	0.3575944	0.1299592	0.09151717	0.110713	0.13695
1848.714	15.5	0.379717	0.3720449	0.1272954	0.09179331	0.108593	0.1378442
1848.814	15.5	0.389033	0.366176	0.1288955	0.09129006	0.108602	0.13648
1848.914	15.5	0.390465	0.368109	0.1258679	0.09077208	0.108855	0.1404817
1849.014	15.5	0.392818	0.360647	0.1275892	0.0891073	0.109235	0.1428242
1849.114	15.5	0.402626	0.3731741	0.1260495	0.08697011	0.109612	0.137823
1849.214	15.5	0.399595	0.3719898	0.1216784	0.0837101	0.109886	0.1348285
1849.314	15.5	0.395906	0.3803964	0.1185888	0.08198646	0.108767	0.1341767
1849.414	15.5	0.387289	0.3789655	0.1201512	0.08242355	0.106099	0.1314021
1849.514	15.5	0.398506	0.3820971	0.1191824	0.08421779	0.108208	0.1317841
1849.614	15.5	0.390159	0.3720763	0.1187815	0.08534077	0.109734	0.1334513
1849.714	15.5	0.394256	0.3782401	0.1210808	0.08422849	0.112615	0.1314979
1849.814	15.5	0.393691	0.3783916	0.1233975	0.08645614	0.114983	0.1364438
1849.914	15.5	0.381166	0.378306	0.1244152	0.08509157	0.114626	0.1371965
1850.014	15.5	0.383646	0.3680651	0.1255054	0.08784985	0.114889	0.133815
1850.114	15.5	0.372187	0.368246	0.1303404	0.09022309	0.117023	0.1328431
1850.214	15.5	0.375686	0.3762414	0.1332616	0.08954728	0.117704	0.1317428
1850.314	15.5	0.382002	0.3680479	0.1325839	0.0874702	0.119945	0.1322984
1850.414	15.5	0.389952	0.3650054	0.1321814	0.08919663	0.121435	0.1322089
1850.514	15.5	0.386331	0.3554978	0.1310936	0.08984019	0.125101	0.13401
1850.614	15.5	0.400351	0.3688188	0.127808	0.0902632	0.122561	0.1348607
1850.714	15.5	0.41061	0.3687989	0.128435	0.09081613	0.121446	0.1344021
1850.814	15.5	0.408743	0.3600537	0.1319477	0.09011158	0.118951	0.1337253
1850.914	15.5	0.410821	0.3687176	0.128379	0.09448171	0.114519	0.1342684

1851.014	15.5	0.411284	0.3692921	0.1250245	0.09466379	0.115135	0.1350609
1851.114	15.5	0.421775	0.3694881	0.1247461	0.09689464	0.118164	0.13464
1851.214	15.5	0.417115	0.3690375	0.1243424	0.1007789	0.117855	0.1339399
1851.314	15.5	0.413582	0.3739219	0.1230729	0.0998857	0.113621	0.13348
1851.414	15.5	0.411831	0.361873	0.1212258	0.09970351	0.113074	0.1329967
1851.514	15.5	0.422948	0.3619684	0.1227284	0.09840087	0.11272	0.1304676
1851.614	15.5	0.406712	0.3597901	0.1252892	0.09595515	0.113206	0.1328557
1851.714	15.5	0.39249	0.3570616	0.1250866	0.09604321	0.113851	0.1339448
1851.814	15.5	0.406678	0.3592789	0.1244236	0.09767184	0.113561	0.1361691
1851.914	15.5	0.410659	0.3657895	0.1248675	0.09774059	0.113759	0.1328859
1852.014	15.5	0.388893	0.3615747	0.1258414	0.09561601	0.115486	0.1306795
1852.114	15.5	0.3937	0.3640088	0.1237871	0.09525066	0.113848	0.1292715
1852.214	15.5	0.391175	0.3584805	0.1233929	0.09420048	0.113105	0.1291093
1852.314	15.5	0.392061	0.3631272	0.126511	0.09388182	0.109816	0.130939
1852.414	15.5	0.393059	0.3620936	0.125436	0.09455957	0.109942	0.1287299
1852.514	15.5	0.390759	0.3699698	0.1258817	0.09395721	0.109464	0.1320624
1852.614	15.5	0.383625	0.3634806	0.1254931	0.0926709	0.108383	0.1364165
1852.714	15.5	0.390168	0.3673164	0.1274094	0.09298459	0.107141	0.1387237
1852.814	15.5	0.392918	0.3681902	0.1258772	0.09165772	0.105044	0.142066
1852.914	15.5	0.395467	0.3744982	0.1238786	0.09031595	0.106808	0.1423747
1853.014	15.5	0.402723	0.3724296	0.1228715	0.089048	0.107744	0.143338
1853.114	15.5	0.398124	0.3667725	0.1241633	0.08670954	0.107544	0.1428351
1853.214	15.5	0.391904	0.3598029	0.1211866	0.08611771	0.107865	0.145749
1853.314	15.5	0.394643	0.360763	0.1214168	0.08583777	0.10994	0.1484734
1853.414	15.5	0.395873	0.3679411	0.1202203	0.08528721	0.113051	0.1399198
1853.514	15.5	0.390518	0.3768398	0.1191986	0.08593595	0.110721	0.1375656
1853.614	15.5	0.394143	0.3782038	0.1188305	0.0866257	0.112861	0.1354703
1853.714	15.5	0.389708	0.3639792	0.11889	0.08655097	0.114149	0.1355313
1853.814	15.5	0.385519	0.3587438	0.1185916	0.08559779	0.114043	0.1357608
1853.914	15.5	0.38272	0.3736674	0.1194025	0.08600596	0.114792	0.1369827
1854.014	15.5	0.374262	0.3727444	0.118763	0.08796243	0.119278	0.1338506
1854.114	15.5	0.381071	0.3636861	0.1222245	0.08939034	0.122596	0.134353
1854.214	15.5	0.38558	0.3616666	0.1198688	0.09112564	0.122501	0.1369342
1854.314	15.5	0.398184	0.3578556	0.1254905	0.09315737	0.124041	0.1355264
1854.414	15.5	0.386304	0.3613531	0.1306627	0.09309509	0.12814	0.1328934
1854.514	15.5	0.401522	0.3603101	0.1362532	0.09369905	0.122808	0.1322445
1854.614	15.5	0.40778	0.3637818	0.1372638	0.09310612	0.122337	0.1348723
1854.714	15.5	0.414383	0.3667443	0.1361855	0.09281543	0.121921	0.1351849
1854.814	15.5	0.420371	0.368202	0.1317463	0.0957288	0.116396	0.1350071
1854.914	15.5	0.417641	0.3681185	0.1293396	0.09617345	0.115989	0.1327403
1855.014	15.5	0.423809	0.3758843	0.1308944	0.09575272	0.11935	0.1337852
1855.114	15.5	0.41178	0.361667	0.1320586	0.09622243	0.119236	0.1324554
1855.214	15.5	0.413056	0.3623396	0.1288552	0.09924527	0.114912	0.1329031
1855.314	15.5	0.418425	0.3527011	0.1266032	0.1009466	0.114674	0.1327098
1855.414	15.5	0.420923	0.358973	0.1257818	0.09943648	0.115136	0.1357953
1855.514	15.5	0.396578	0.3564329	0.1237119	0.09452289	0.113907	0.1355854
1855.614	15.5	0.385854	0.3580541	0.1217432	0.09200753	0.112598	0.1342672
1855.714	15.5	0.400285	0.35617	0.1218149	0.09200621	0.111457	0.1325952
1855.814	15.5	0.408786	0.3590637	0.1222063	0.09359699	0.112971	0.131431
1855.914	15.5	0.392987	0.364981	0.1256521	0.09306877	0.111854	0.1340322
1856.014	15.5	0.390204	0.3571634	0.1228745	0.09407871	0.111452	0.1344599
1856.114	15.5	0.390223	0.3558312	0.1207923	0.09282693	0.113025	0.1325571
1856.214	15.5	0.389196	0.3613108	0.1210936	0.09191488	0.11231	0.1297468
1856.314	15.5	0.396847	0.3432009	0.1217061	0.09115691	0.111168	0.1294027
1856.414	15.5	0.383797	0.3438025	0.1218265	0.09257226	0.109334	0.1305287
1856.514	15.5	0.385889	0.3511273	0.1200806	0.09213106	0.111329	0.1287878
1856.614	15.5	0.389177	0.3480633	0.1242788	0.09281227	0.110492	0.127691
1856.714	15.5	0.396311	0.3391972	0.1225288	0.09089559	0.108618	0.1290375
1856.814	15.5	0.398915	0.3508889	0.1215113	0.08956707	0.108719	0.1300218
1856.914	15.5	0.402953	0.329301	0.1219802	0.08852111	0.109436	0.1336608
1857.014	15.5	0.39719	0.3436545	0.123648	0.08575403	0.110526	0.1357941
1857.114	15.5	0.389887	0.3516181	0.1246459	0.08350647	0.109507	0.1413184
1857.214	15.5	0.393546	0.3600478	0.1219607	0.08572763	0.109536	0.1390239
1857.314	15.5	0.392528	0.3580737	0.1208519	0.08580631	0.108602	0.1403541
1857.414	15.5	0.397981	0.3505297	0.1202617	0.08911712	0.107609	0.1403544
1857.514	15.5	0.395398	0.3561377	0.1191817	0.08834328	0.111316	0.1389261
1857.614	15.5	0.389789	0.3578258	0.1188637	0.08895743	0.114584	0.1427114
1857.714	15.5	0.384311	0.366303	0.1190195	0.08974291	0.118007	0.138841
1857.814	15.5	0.377178	0.3614859	0.1186822	0.09090916	0.120032	0.1380402
1857.914	15.5	0.38978	0.3535077	0.121548	0.08938269	0.120291	0.1374548
1858.014	15.5	0.387659	0.358687	0.1216301	0.08798899	0.124538	0.1347523
1858.114	15.5	0.392375	0.3567056	0.1246243	0.09037767	0.123741	0.1336127
1858.214	15.5	0.407757	0.3592724	0.1273053	0.09141278	0.122883	0.1354176
1858.314	15.5	0.42184	0.3574098	0.1286142	0.0927629	0.12049	0.1333571
1858.414	15.5	0.424237	0.374102	0.1293185	0.09421604	0.118574	0.1333739
1858.593	15.5	0.414161	0.3641035	0.1301877	0.09441201	0.119142	0.132253
1858.693	16	0.421957	0.3560739	0.1345606	0.09158224	0.119289	0.1316533
1858.793	16	0.417061	0.3644398	0.1387815	0.09215864	0.116645	0.131792
1858.893	16	0.406172	0.364975	0.1403074	0.09522737	0.118346	0.1333958
1858.993	16	0.413504	0.3675771	0.1390706	0.1013982	0.120827	0.1347183
1859.093	16	0.421618	0.3609348	0.1399183	0.1036407	0.119553	0.1342226
1859.193	16	0.407125	0.3729379	0.1321517	0.1003247	0.120624	0.1317493
1859.293	16	0.388987	0.3708639	0.130282	0.09724279	0.119503	0.1317768
1859.393	16	0.409397	0.3725362	0.1324615	0.09397607	0.117974	0.1330109
1859.493	16	0.414273	0.3767502	0.1325484	0.09217195	0.117358	0.1319955
1859.593	16	0.391743	0.3818235	0.1292826	0.09246362	0.116852	0.132397
1859.693	16	0.391858	0.3742331	0.1266341	0.09298276	0.117177	0.1321059
1859.793	16	0.390253	0.3754883	0.1258581	0.09286064	0.115775	0.1372676
1859.893	16	0.394338	0.3741903	0.1262554	0.09235355	0.114439	0.1367923
1859.993	16	0.404359	0.3630629	0.1262891	0.09303653	0.117093	0.1362687
1860.093	16	0.389081	0.3657826	0.1257453	0.09419866	0.117801	0.13515
1860.193	16	0.388432	0.3702067	0.1236025	0.09207663	0.113892	0.1361142
1860.293	16	0.39135	0.3746886	0.1235016	0.09079083	0.111131	0.134748
1860.393	16	0.39769	0.3688309	0.1254955	0.0903141	0.110562	0.1338852
1860.493	16	0.395939	0.3645082	0.1241866	0.08886737	0.112927	0.130839

1860.593	16	0.400919	0.3579538	0.1251376	0.08885423	0.113328	0.1302088
1860.693	16	0.398197	0.3672774	0.1258769	0.0888112	0.112943	0.1315503
1860.793	16	0.388874	0.366859	0.1279196	0.08639263	0.114472	0.1305757
1860.893	16	0.39896	0.3579947	0.1246921	0.08648756	0.114412	0.1269745
1860.993	16	0.393634	0.3672941	0.1255926	0.08669596	0.115879	0.1282221
1861.093	16	0.395664	0.3661653	0.1274696	0.09094887	0.113321	0.1303672
1861.193	16	0.39409	0.3704594	0.1274413	0.08810157	0.114129	0.1341122
1861.293	16	0.384052	0.3689905	0.125647	0.08895493	0.111279	0.138499
1861.393	16	0.386921	0.3715053	0.1272697	0.0876877	0.110309	0.143366
1861.493	16	0.379307	0.3620009	0.1252999	0.08904191	0.113241	0.1441461
1861.593	16	0.39196	0.3613153	0.1264475	0.08920293	0.117403	0.1448765
1861.693	16	0.412175	0.357551	0.1230626	0.08542757	0.119029	0.1468429
1861.793	16	0.406692	0.3598638	0.1211878	0.08868182	0.119361	0.1438496
1861.893	16	0.392099	0.3624276	0.1212064	0.09171607	0.121061	0.1433451
1861.993	16	0.414756	0.366385	0.1200623	0.09202442	0.122899	0.1447255
1862.093	16	0.424615	0.3618924	0.1181163	0.09303807	0.12395	0.1405131
1862.193	16	0.424629	0.364856	0.11752	0.09040213	0.127932	0.1392728
1862.293	16	0.421111	0.3585512	0.1161381	0.09502975	0.127884	0.1387055
1862.393	16	0.416423	0.3624317	0.1225993	0.09458425	0.121707	0.1371605
1862.493	16	0.416761	0.3653393	0.1255275	0.09424337	0.119455	0.1381388
1862.593	16	0.40508	0.3687797	0.1311994	0.09751173	0.117287	0.1374397
1862.693	16	0.411668	0.3681943	0.1342632	0.1005379	0.115814	0.1390203
1862.793	16	0.428855	0.3699969	0.136578	0.1002651	0.119383	0.1396813
1862.893	16	0.425357	0.3688313	0.1392209	0.1008513	0.12146	0.1371453
1862.993	16	0.401528	0.3750986	0.1395359	0.1000833	0.119728	0.1330969
1863.093	16	0.404437	0.3714395	0.1347676	0.09571671	0.119525	0.1321113
1863.193	16	0.425177	0.3684807	0.1329253	0.09530612	0.119576	0.1351558
1863.293	16	0.404662	0.3603267	0.132413	0.09694217	0.121127	0.1352745
1863.393	16	0.39511	0.3608948	0.1296262	0.0937422	0.121347	0.1346757
1863.493	16	0.390819	0.3673039	0.126477	0.09213959	0.119646	0.1366418
1863.593	16	0.391737	0.3771538	0.127479	0.09363364	0.118779	0.1373918
1863.693	16	0.391649	0.3789095	0.1272983	0.09376705	0.118537	0.1371367
1863.793	16	0.398921	0.362634	0.1272946	0.09417448	0.118633	0.1366966
1863.893	16	0.387395	0.35887	0.1233346	0.09345428	0.118406	0.1379404
1863.993	16	0.39354	0.3745474	0.1210881	0.09401538	0.118532	0.1375128
1864.093	16	0.392882	0.3738717	0.1235338	0.09563834	0.115984	0.1396008
1864.193	16	0.400417	0.3638412	0.123079	0.0937532	0.114473	0.1409729
1864.293	16	0.402538	0.3620345	0.1235763	0.09097849	0.112465	0.144371
1864.393	16	0.39907	0.357713	0.1219178	0.08878478	0.113749	0.1441863
1864.493	16	0.391244	0.3620834	0.1242964	0.08826502	0.112472	0.1444273
1864.593	16	0.394499	0.3602546	0.1228012	0.08840489	0.113166	0.1415255
1864.693	16	0.393867	0.362603	0.1233025	0.08752503	0.110985	0.1402898
1864.793	16	0.388765	0.3640106	0.1237414	0.08900629	0.111588	0.1367277
1864.893	16	0.393579	0.3709638	0.1242916	0.09018432	0.112748	0.1349851
1864.993	16	0.38926	0.3669074	0.1249736	0.0916687	0.116856	0.1337214
1865.093	16	0.382358	0.3753777	0.1258546	0.09243531	0.117094	0.1341515
1865.193	16	0.380254	0.3618255	0.1255535	0.09074017	0.111532	0.1330546
1865.293	16	0.379575	0.3658656	0.1228461	0.09153464	0.111028	0.1346362
1865.393	16	0.387894	0.3535662	0.1202405	0.09241857	0.108539	0.1345772
1865.493	16	0.368262	0.3623812	0.1226123	0.09279893	0.111685	0.1361864
1865.593	16	0.388194	0.358874	0.1223512	0.09480587	0.114332	0.1385661
1865.693	16	0.394227	0.3580765	0.1231321	0.09499848	0.115511	0.1416584
1865.793	16	0.403999	0.3605662	0.1220906	0.09630432	0.120958	0.1405324
1865.893	16	0.404583	0.3657944	0.1185674	0.09647463	0.121844	0.1419407
1865.993	16	0.417136	0.3682063	0.1177819	0.09534959	0.124637	0.1437166
1866.093	16	0.416137	0.3618906	0.117686	0.09591586	0.124876	0.1432624
1866.193	16	0.425106	0.3608316	0.1179704	0.09694672	0.121867	0.1416352
1866.293	16	0.413874	0.3643039	0.1199925	0.09889673	0.118947	0.142839
1866.393	16	0.404809	0.3470454	0.1209569	0.09990939	0.118782	0.1389478
1866.493	16	0.411526	0.3490508	0.1206964	0.1011207	0.117342	0.1394396
1866.593	16	0.420758	0.3513021	0.1231267	0.1015848	0.115724	0.1373014
1866.693	16	0.401354	0.3586347	0.1227598	0.1006159	0.117014	0.1368956
1866.793	16	0.394957	0.3397409	0.1281964	0.0971286	0.116508	0.1380215
1866.893	16	0.405204	0.3557802	0.13185	0.0950297	0.114552	0.1382134
1866.993	16	0.406335	0.3475427	0.1343265	0.09505464	0.114132	0.1364159
1867.093	16	0.426605	0.3366951	0.1354928	0.09758724	0.115089	0.1390366
1867.193	16	0.444606	0.3534486	0.1316909	0.0945707	0.117544	0.1371163
1867.293	16	0.410224	0.3610577	0.1283143	0.09243819	0.118667	0.1342426
1867.393	16	0.404621	0.3638636	0.1313336	0.09177811	0.116908	0.1334977
1867.493	16	0.401795	0.3529874	0.1315678	0.09317015	0.117215	0.1349049
1867.593	16	0.399415	0.3561015	0.1269533	0.09540473	0.114272	0.134775
1867.693	16	0.403551	0.3600905	0.127186	0.09612978	0.114399	0.1344796
1867.793	16	0.406642	0.363921	0.1265804	0.09387875	0.115037	0.1333249
1867.893	16	0.424813	0.3723166	0.1287946	0.09192352	0.113059	0.1322564
1867.993	16	0.42299	0.3537366	0.1256105	0.090919	0.110212	0.135335
1868.093	16	0.406613	0.35891	0.1259626	0.08969339	0.108905	0.1339907
1868.193	16	0.405992	0.3580512	0.1246264	0.08768255	0.108369	0.1331726
1868.293	16	0.400899	0.3583488	0.1241808	0.08737563	0.108526	0.1350528
1868.393	16	0.403979	0.3571014	0.1230268	0.0883221	0.107595	0.1387699
1868.493	16	0.397408	0.37614	0.1229166	0.08658996	0.109896	0.13932
1868.593	16	0.389995	0.3691415	0.1238307	0.08676427	0.110456	0.1390813
1868.693	16	0.392318	0.3594936	0.1239214	0.08786488	0.10899	0.1371319
1868.793	16	0.388344	0.367	0.1249054	0.08704926	0.109402	0.1363217
1868.893	16	0.385331	0.3667677	0.1282057	0.08785557	0.109459	0.136846
1868.993	16	0.376512	0.3703805	0.1246576	0.09066773	0.108755	0.135991
1869.093	16	0.380127	0.3616652	0.1231978	0.09033889	0.1075	0.1331423
1869.193	16	0.389989	0.3743425	0.1248691	0.08712924	0.108391	0.1310075
1869.293	16	0.389346	0.3711968	0.1270363	0.0867855	0.109697	0.1297476
1869.393	16	0.3869	0.3769638	0.1283507	0.08866029	0.112413	0.1306264
1869.493	16	0.391326	0.3812234	0.1271267	0.09037717	0.114814	0.1276197
1869.593	16	0.401818	0.3823111	0.129157	0.08924954	0.117107	0.1276001
1869.693	16	0.403863	0.3764282	0.12874	0.09001277	0.11886	0.1265198
1869.793	16	0.415756	0.3772437	0.1285396	0.09234668	0.120774	0.1275136
1869.893	16	0.433182	0.377154	0.1269258	0.09311134	0.122179	0.1312066
1869.993	16	0.435036	0.3712338	0.1243494	0.09138923	0.122428	0.1357866

1870.093	16	0.430872	0.3673339	0.1248478	0.09653229	0.119851	0.1387979
1870.193	16	0.434132	0.370295	0.123419	0.09929851	0.119851	0.1406318
1870.293	16	0.433371	0.3749237	0.1232846	0.1009035	0.120952	0.1430299
1870.393	16	0.432805	0.3669578	0.1244682	0.1011073	0.118537	0.143038
1870.493	16	0.402632	0.3643966	0.1276735	0.09848042	0.117295	0.1428463
1870.593	16	0.431731	0.357655	0.1279309	0.09173203	0.116869	0.1418528
1870.693	16	0.438739	0.3716174	0.1314123	0.09185416	0.117615	0.1361281
1870.793	16	0.41498	0.3698466	0.1326967	0.09265412	0.117032	0.1361148
1870.893	16	0.45271	0.3606998	0.13467	0.09074312	0.11767	0.1334199
1870.993	16	0.433675	0.3692531	0.1362244	0.08996575	0.118509	0.130598
1871.093	16	0.409754	0.3692672	0.1381301	0.08858012	0.118897	0.1321528
1871.193	16	0.398426	0.3713913	0.1350024	0.08839712	0.119907	0.1319641
1871.293	16	0.405153	0.3680427	0.134618	0.09030459	0.117865	0.1302701
1871.393	16	0.398909	0.3741829	0.1299739	0.08905146	0.118081	0.1344254
1871.493	16	0.404185	0.3638003	0.1291331	0.09082335	0.116042	0.1325195
1871.593	16	0.404089	0.3635568	0.1308415	0.08914281	0.116655	0.1309802
1871.693	16	0.421452	0.3627246	0.1257119	0.08766512	0.116377	0.1316236
1871.793	16	0.41834	0.3604962	0.1257323	0.08685201	0.11279	0.1345678
1871.893	16	0.401072	0.3621136	0.128103	0.08500776	0.110418	0.1335359
1871.993	16	0.399253	0.3676707	0.1253078	0.08552171	0.110298	0.1330191
1872.093	16	0.391663	0.3642546	0.1237997	0.08635668	0.108997	0.1301309
1872.193	16	0.391399	0.3665881	0.1223256	0.08591837	0.109794	0.1304137
1872.293	16	0.396311	0.3596472	0.1241914	0.08485967	0.108566	0.1339992
1872.393	16	0.391018	0.3643125	0.124719	0.08428495	0.107872	0.1345039
1872.493	16	0.393634	0.3633563	0.1240907	0.08538926	0.107354	0.1345592
1872.593	16	0.385593	0.3712103	0.1251981	0.08424953	0.105691	0.1359592
1872.693	16	0.389138	0.3658781	0.1251249	0.08476221	0.106329	0.1390359
1872.793	16	0.376845	0.3702374	0.1243395	0.08783601	0.109782	0.137954
1872.893	16	0.389648	0.3701879	0.1237592	0.08631201	0.109936	0.1389139
1872.993	16	0.400461	0.3764066	0.1258356	0.08511551	0.109729	0.136028
1873.093	16	0.403479	0.3720798	0.1284459	0.08621252	0.111529	0.1363392
1873.193	16	0.393822	0.366995	0.1275831	0.08931226	0.11274	0.1355209
1873.293	16	0.405213	0.3594422	0.1257244	0.08719621	0.113911	0.13392
1873.393	16	0.401353	0.3609862	0.1245862	0.08537074	0.113253	0.1363247
1873.493	16	0.417504	0.3681221	0.1251973	0.08589022	0.110648	0.135793
1873.593	16	0.418008	0.378232	0.1221896	0.08860449	0.114693	0.13583
1873.693	16	0.426537	0.3814422	0.1200657	0.08807375	0.117081	0.1355458
1873.793	16	0.424666	0.3629978	0.1217114	0.08787858	0.115904	0.1345032
1873.893	16	0.424515	0.3576167	0.1224496	0.09068611	0.118162	0.1306518
1873.993	16	0.424001	0.3729258	0.1235858	0.09224286	0.118882	0.1287993
1874.093	16	0.437629	0.3740245	0.1210301	0.09609862	0.117321	0.1297688
1874.193	16	0.413268	0.3639636	0.1180421	0.09423733	0.114306	0.1323902
1874.293	16	0.393876	0.361343	0.1211863	0.09153207	0.114877	0.1351641
1874.393	16	0.411321	0.3572239	0.1200847	0.09017027	0.116756	0.1349194
1874.493	16	0.41298	0.3597637	0.1203405	0.09151491	0.117005	0.137531
1874.593	16	0.420203	0.357801	0.1208271	0.09146138	0.117895	0.1400601
1874.693	16	0.454234	0.3661752	0.119254	0.09112304	0.117548	0.1413714
1874.793	16	0.428475	0.3645222	0.1225568	0.08828877	0.117448	0.1416148
1874.893	16	0.410285	0.3695951	0.1239276	0.09009983	0.115576	0.1390957
1874.993	16	0.404487	0.3689187	0.1238817	0.08948564	0.117703	0.1337691
1875.093	16	0.399685	0.3725924	0.1287274	0.09097742	0.11598	0.1340817
1875.193	16	0.409547	0.3590901	0.1355738	0.08960764	0.114012	0.1340873
1875.293	16	0.400527	0.3643745	0.1368183	0.08966503	0.11522	0.1322037
1875.393	16	0.422741	0.3453447	0.138072	0.09135009	0.118049	0.135586
1875.493	16	0.427339	0.3574089	0.1330934	0.09014665	0.118607	0.1312461
1875.593	16	0.412737	0.3516798	0.1315735	0.0887839	0.11864	0.1296275
1875.693	16	0.410279	0.3573639	0.1330551	0.08678299	0.114349	0.1337434
1875.793	16	0.397652	0.3591244	0.1291538	0.08614352	0.112839	0.1330386
1875.893	16	0.393971	0.3571156	0.1270509	0.08534828	0.111144	0.1322836
1875.993	16	0.396525	0.3634234	0.1294727	0.08673882	0.110308	0.1307101
1876.093	16	0.394566	0.3520249	0.1279628	0.08852943	0.108211	0.132203
1876.193	16	0.401602	0.3596058	0.1283446	0.0858016	0.10673	0.1338724
1876.293	16	0.386657	0.3649282	0.125484	0.08831265	0.105644	0.1348583
1876.393	16	0.391425	0.3455971	0.1268164	0.09032313	0.106693	0.137123
1876.493	16	0.379721	0.3465845	0.125794	0.09235123	0.106919	0.1380682
1876.593	16	0.381773	0.3579417	0.1259489	0.09066916	0.105844	0.1376135
1876.693	16	0.417914	0.3480391	0.1237422	0.08915571	0.108656	0.1376721
1876.793	16	0.430829	0.3474919	0.1251281	0.09095596	0.108872	0.137964
1876.893	16	0.398025	0.3552513	0.1267452	0.09268097	0.111712	0.1366166
1876.993	16	0.409364	0.3364637	0.1235158	0.09020808	0.111491	0.1374846
1877.093	16	0.417464	0.3457785	0.1242104	0.08983029	0.108541	0.1367984
1877.193	16	0.416189	0.3551571	0.1282133	0.09068225	0.106631	0.1391546
1877.293	16	0.41834	0.361526	0.1276126	0.09363279	0.108831	0.1358946
1877.393	16	0.428579	0.3606342	0.1272746	0.09804877	0.110876	0.1343786
1877.493	16	0.426257	0.3519932	0.1258773	0.09799691	0.111606	0.1325437
1877.593	16	0.425152	0.3563255	0.1254431	0.09970142	0.117805	0.1307281
1877.693	16	0.428092	0.3583244	0.12396	0.09716336	0.126033	0.1311885
1877.793	16	0.419559	0.3693641	0.1226566	0.09989503	0.125215	0.1332933
1877.893	16	0.427056	0.3670147	0.1238774	0.1017767	0.122917	0.1338935
1877.993	16	0.388758	0.3547652	0.1214542	0.1000989	0.121308	0.1349781
1878.093	16	0.40241	0.3570331	0.1241129	0.09626514	0.119576	0.1356979
1878.193	16	0.411492	0.35601	0.1210838	0.0980529	0.117137	0.1342698
1878.293	16	0.39941	0.3581265	0.1164262	0.09635062	0.116445	0.1336827
1878.393	16	0.440382	0.3572949	0.1161642	0.09337482	0.118315	0.1319782
1878.493	16	0.440379	0.3764588	0.1184606	0.09347223	0.117544	0.134831
1878.593	16	0.412334	0.3685343	0.1182033	0.09246472	0.118548	0.1377128
1878.693	16	0.401484	0.3573831	0.1174652	0.0907384	0.116374	0.1379706
1878.793	16	0.401556	0.3696049	0.1157259	0.09086114	0.116638	0.1386702
1878.893	16	0.401565	0.3658174	0.1205252	0.09127415	0.117043	0.141123
1878.993	16	0.407462	0.3695967	0.1225151	0.08960855	0.114803	0.1437404
1879.093	16	0.409845	0.3608551	0.1236649	0.08880887	0.11252	0.1427716
1879.193	16	0.427352	0.3728319	0.1286078	0.08695924	0.111831	0.1417035
1879.293	16	0.427943	0.3705242	0.1343754	0.0854392	0.11168	0.1378016
1879.393	16	0.419686	0.3780157	0.1331556	0.0862057	0.113809	0.1359986
1879.493	16	0.408922	0.382866	0.1345375	0.08681249	0.112986	0.1326238

1879.593	16	0.398122	0.38104	0.1354361	0.08556964	0.110111	0.132636
1879.693	16	0.394266	0.3780815	0.1327195	0.08450982	0.108523	0.1339615
1879.793	16	0.398717	0.3753094	0.1320561	0.08597763	0.106999	0.1333716
1879.893	16	0.399646	0.3768613	0.12946	0.09105024	0.106533	0.1349437
1879.993	16	0.395435	0.3729222	0.1268736	0.08995268	0.105115	0.1335737
1880.093	16	0.387333	0.366986	0.1256971	0.09019238	0.106419	0.133588
1880.193	16	0.390449	0.3696312	0.1276703	0.0892638	0.107821	0.1344405
1880.293	16	0.382277	0.3727902	0.1231039	0.08710671	0.108157	0.1327363
1880.393	16	0.396968	0.3679324	0.1217123	0.08743446	0.107414	0.1343793
1880.493	16	0.392917	0.3614131	0.1239766	0.08954845	0.10527	0.1369464
1880.593	16	0.400171	0.358224	0.1238947	0.09201118	0.104714	0.1381409
1880.693	16	0.390463	0.3682866	0.1231924	0.09437062	0.104923	0.1383714
1880.793	16	0.397693	0.3671265	0.1238707	0.09236911	0.107684	0.1372107
1880.893	16	0.40015	0.3592361	0.1255799	0.0902548	0.110547	0.1359594
1880.993	16	0.414685	0.3668528	0.1241596	0.09227001	0.109738	0.1353882
1881.093	16	0.416215	0.3686274	0.122965	0.09326571	0.109934	0.134796
1881.193	16	0.424518	0.3705168	0.1226838	0.0975024	0.110385	0.1356156
1881.293	16	0.420322	0.3665067	0.1261917	0.09688886	0.109582	0.1355521
1881.393	16	0.422954	0.3737251	0.1247326	0.0978481	0.114554	0.1364825
1881.493	16	0.432127	0.3625686	0.1232693	0.09987371	0.116717	0.1309648
1881.593	16	0.443963	0.3625586	0.1218113	0.1006567	0.117163	0.1325949
1881.693	16	0.411089	0.3610423	0.1225643	0.09868237	0.11606	0.1326107
1881.793	16	0.401025	0.3598424	0.1215383	0.09315705	0.117891	0.132427
1881.893	16	0.449386	0.3616008	0.1213664	0.09548423	0.11785	0.1308917
1881.993	16	0.420496	0.3666932	0.1225818	0.09847476	0.115264	0.1313206
1882.093	16	0.422317	0.3652631	0.1228897	0.09624368	0.116207	0.133024
1882.193	16	0.456891	0.3663188	0.1221044	0.09534661	0.115051	0.1309896
1882.293	16	0.433337	0.3581875	0.122949	0.09242126	0.117067	0.133909
1882.393	16	0.414195	0.3650262	0.119959	0.09028916	0.113371	0.1390132
1882.493	16	0.407511	0.3613267	0.1185528	0.09103982	0.111716	0.1416633
1882.593	16	0.397392	0.3738305	0.1197021	0.09136013	0.111332	0.1424864
1882.693	16	0.412354	0.3657976	0.1196156	0.09178079	0.112567	0.140818
1882.793	16	0.403749	0.3703298	0.1233417	0.08927126	0.112465	0.1420472
1882.893	16	0.427077	0.3713973	0.1233188	0.08836213	0.111786	0.1423422
1882.993	16	0.430195	0.3758771	0.1234621	0.08609474	0.112919	0.1403939
1883.093	16	0.425888	0.37286	0.1262763	0.08680824	0.114374	0.1415661
1883.193	16	0.421423	0.3658315	0.1288268	0.08663549	0.11293	0.1396059
1883.293	16	0.402882	0.3605928	0.1309393	0.08404758	0.111035	0.1372744
1883.393	16	0.397799	0.3602869	0.1341463	0.08302444	0.107726	0.1364741
1883.493	16	0.395855	0.3700067	0.1346488	0.08622865	0.103392	0.1355907
1883.593	16	0.396017	0.3809105	0.1331565	0.08898684	0.104359	0.1352663
1883.693	16	0.402361	0.3798078	0.1364424	0.08694559	0.107054	0.1382098
1883.793	16	0.38876	0.3619231	0.1349603	0.08797538	0.10594	0.1359067
1883.893	16	0.395279	0.3575965	0.1352852	0.09105589	0.10571	0.1386028
1883.993	16	0.380633	0.3728654	0.131678	0.09034746	0.104209	0.1380204
1884.093	16	0.384605	0.3700743	0.1278632	0.08695032	0.104929	0.1363498
1884.193	16	0.415046	0.3619692	0.1272548	0.08569325	0.104323	0.134461
1884.293	16	0.41654	0.3615979	0.1293901	0.08666217	0.104881	0.1362697
1884.393	16	0.398732	0.3550133	0.1250001	0.08768234	0.105762	0.1359751
1884.493	16	0.404253	0.3572323	0.1233737	0.09193364	0.108383	0.1363441
1884.593	16	0.401489	0.3593596	0.1222786	0.09073923	0.107861	0.1382538
1884.693	16	0.404398	0.3647021	0.1265229	0.08998604	0.112896	0.1401676
1884.793	16	0.41364	0.3619829	0.1263603	0.09285799	0.113027	0.1372368
1884.893	16	0.418157	0.3678629	0.1269115	0.09383439	0.109152	0.1378373
1884.993	16	0.422313	0.3718054	0.1258467	0.09654354	0.1067	0.1350767
1885.093	16	0.41339	0.3716102	0.1260784	0.1011462	0.111623	0.1324585
1885.193	16	0.417079	0.3591357	0.1252214	0.1021693	0.115264	0.1326749
1885.293	16	0.421002	0.3680314	0.1260055	0.1014047	0.118538	0.1350916
1885.393	16	0.427696	0.3519223	0.1270421	0.1009963	0.119246	0.1352328
1885.493	16	0.388671	0.3623635	0.1278636	0.09582625	0.118253	0.1343942
1885.593	16	0.39337	0.3528059	0.128741	0.09372612	0.119298	0.1324826
1885.693	16	0.408818	0.360238	0.1246522	0.09302298	0.117208	0.1318214
1885.793	16	0.396723	0.3611358	0.1242287	0.0930622	0.116794	0.1308005
1885.893	16	0.437643	0.3593496	0.1266999	0.09136804	0.115022	0.1340705
1885.993	16	0.443311	0.3679743	0.1239541	0.08958232	0.113907	0.1367331
1886.093	16	0.417519	0.3577434	0.1238997	0.08851407	0.115966	0.1390621
1886.193	16	0.403857	0.3604884	0.1247869	0.08907514	0.117342	0.1375819
1886.293	16	0.402498	0.3650186	0.1218985	0.08935253	0.113244	0.1383533
1886.393	16	0.403413	0.3470244	0.1232377	0.08963836	0.115073	0.1357872
1886.493	16	0.407134	0.3474229	0.1226672	0.08631171	0.113643	0.1368591
1886.593	16	0.406937	0.35914	0.1213706	0.08327939	0.114462	0.1370869
1886.693	16	0.425183	0.3502219	0.1230143	0.08375565	0.115369	0.1382803
1886.793	16	0.420712	0.347452	0.1241007	0.08374168	0.116154	0.1379643
1886.893	16	0.411109	0.3548125	0.1242353	0.08753563	0.114671	0.1417052
1886.993	16	0.409326	0.3352804	0.1271715	0.08630897	0.111569	0.1423791
1887.093	16	0.401038	0.3479808	0.1281016	0.08627252	0.110562	0.1432993
1887.193	16	0.393563	0.3552613	0.1266754	0.08775735	0.112528	0.1451147
1887.293	16	0.394538	0.3639246	0.1261121	0.08958629	0.10882	0.1421019
1887.393	16	0.396687	0.3584407	0.1264463	0.08802897	0.108445	0.1395213
1887.493	16	0.397363	0.352116	0.1256689	0.08515453	0.108666	0.1367389
1887.593	16	0.386538	0.3559411	0.1290058	0.08489006	0.109026	0.135869
1887.693	16	0.387642	0.3577388	0.1296487	0.08700335	0.107693	0.1356813
1887.793	16	0.378922	0.3700704	0.1316852	0.08639352	0.105235	0.1357192
1887.893	16	0.383244	0.365279	0.1308169	0.0855578	0.106088	0.1334432
1887.993	16	0.393701	0.3529131	0.1284199	0.08722568	0.107788	0.1346455
1888.093	16	0.406106	0.3553982	0.1291198	0.08490859	0.108437	0.1360172
1888.193	16	0.393551	0.3529915	0.1281215	0.08586918	0.106145	0.1346991
1888.293	16	0.404903	0.3587812	0.1229864	0.08722687	0.108357	0.1330772
1888.393	16	0.413206	0.3643108	0.1212542	0.08734744	0.107558	0.133626
1888.493	16	0.416971	0.3538163	0.1189319	0.0882751	0.104347	0.1328981
1888.593	16	0.42519	0.3729666	0.1204705	0.08910962	0.107352	0.1322479
1888.693	16	0.437379	0.367695	0.1211953	0.09342729	0.107658	0.1337861
1888.793	16	0.427864	0.3603234	0.1240812	0.09443346	0.110192	0.135197
1888.893	16	0.424085	0.3709212	0.1255146	0.09547629	0.111542	0.1367267
1888.993	16	0.418219	0.3718045	0.1255845	0.09544286	0.114721	0.1337429

1889.093	16	0.432905	0.3773125	0.1261038	0.09373791	0.115206	0.1343627
1889.193	16	0.409003	0.3843256	0.1249807	0.09393039	0.119401	0.1324105
1889.293	16	0.389682	0.3821513	0.1249787	0.09141499	0.123246	0.136902
1889.393	16	0.408001	0.3749707	0.1233285	0.09119966	0.122834	0.1343987
1889.493	16	0.416781	0.3765768	0.1241	0.09187237	0.122977	0.1340087
1889.593	16	0.420812	0.376141	0.1285481	0.09309816	0.115362	0.1298504
1889.693	16	0.457941	0.3735533	0.128114	0.09010256	0.114351	0.1299147
1889.793	16	0.438165	0.3677188	0.1243161	0.08883232	0.11562	0.1295108
1889.893	16	0.415603	0.3647104	0.1238477	0.08778342	0.116739	0.1281409
1889.993	16	0.406774	0.3704026	0.1250705	0.08633756	0.115783	0.1298572
1890.093	16	0.394876	0.3618356	0.1263445	0.08884861	0.115681	0.130919
1890.193	16	0.404754	0.3599427	0.1229672	0.08951285	0.115543	0.1325984
1890.293	16	0.398327	0.3604515	0.1243128	0.08928256	0.114892	0.1354719
1890.393	16	0.410679	0.365959	0.1241547	0.0871576	0.113659	0.1387425
1890.493	16	0.412478	0.3586733	0.1231385	0.08431592	0.113593	0.1362613
1890.593	16	0.42141	0.3656314	0.1218725	0.08646632	0.11401	0.1375179
1890.693	16	0.416442	0.3679929	0.1197534	0.08412556	0.114664	0.1363672
1890.793	16	0.399351	0.3676375	0.1191486	0.08319493	0.113158	0.1392543
1890.893	16	0.397881	0.3670016	0.1214719	0.0832428	0.11321	0.1396705
1890.993	16	0.391838	0.3744773	0.1226443	0.08420821	0.112216	0.1411874
1891.093	16	0.39398	0.3576527	0.1284451	0.08529408	0.112918	0.1406577
1891.193	16	0.398113	0.3613433	0.1294721	0.08364463	0.112801	0.140916
1891.293	16	0.389307	0.3608248	0.1321352	0.08601186	0.112947	0.1412769
1891.393	16	0.393638	0.3598286	0.1327082	0.08597132	0.113249	0.1420957
1891.493	16	0.377093	0.3646449	0.1344109	0.08986312	0.11266	0.1371843
1891.593	16	0.378405	0.3608565	0.1380789	0.09080555	0.113578	0.1358125
1891.693	16	0.391268	0.3661875	0.1422123	0.08907326	0.114063	0.1325823
1891.793	16	0.400449	0.3600846	0.142794	0.08900124	0.110784	0.1298809
1891.893	16	0.393202	0.3624135	0.1419976	0.09151925	0.111314	0.1324371
1891.993	16	0.400974	0.3623627	0.1388659	0.09320036	0.11423	0.1324238
1892.093	16	0.40092	0.3751823	0.1342638	0.09141514	0.113178	0.1336477
1892.193	16	0.403123	0.3649991	0.1336441	0.09135466	0.11474	0.1343726
1892.293	16	0.412519	0.3707711	0.1331337	0.09043808	0.112755	0.1340178
1892.393	16	0.419273	0.3725559	0.1295635	0.09308144	0.111854	0.1317051
1892.493	16	0.422077	0.3755679	0.1285916	0.09577317	0.113045	0.1293756
1892.593	16	0.420828	0.3683414	0.1307306	0.09808746	0.116944	0.131373
1892.693	16	0.424882	0.3594111	0.1281949	0.09749208	0.117791	0.1313704
1892.793	16	0.429761	0.3583435	0.126831	0.1003203	0.117453	0.1314372
1892.893	16	0.43097	0.3682391	0.1255101	0.09943483	0.120733	0.1333839
1892.993	16	0.390855	0.3778743	0.127706	0.09575357	0.121629	0.1339808
1893.093	16	0.394108	0.380051	0.1281113	0.0944631	0.118944	0.1348731
1893.193	16	0.412892	0.361239	0.1272306	0.0928263	0.116907	0.1329918
1893.293	16	0.401387	0.3569037	0.1276814	0.09495221	0.116711	0.1325552
1893.393	16	0.441086	0.3727427	0.1285561	0.0936048	0.117084	0.132453
1893.493	16	0.447863	0.3688508	0.1263557	0.0905434	0.117177	0.1302858
1893.593	16	0.420471	0.3596367	0.1265227	0.09055712	0.117355	0.1323572
1893.693	16	0.400655	0.3570395	0.1309069	0.0900518	0.113998	0.1350721
1893.793	16	0.401898	0.3577044	0.1325836	0.08860967	0.11497	0.1342572
1893.893	16	0.396208	0.3546154	0.1329102	0.0884456	0.11534	0.1331264
1893.993	16	0.405632	0.3633141	0.1311142	0.09092841	0.118036	0.1326416
1894.093	16	0.402999	0.364324	0.1284698	0.09026814	0.116835	0.1323193
1894.193	16	0.409399	0.3679128	0.1273486	0.08781419	0.116494	0.1320856
1894.293	16	0.413477	0.3684529	0.1272572	0.08778179	0.117426	0.1339222
1894.393	16	0.412064	0.3734284	0.1266137	0.08724736	0.115742	0.1365411
1894.493	16	0.407174	0.3583976	0.1265288	0.08629921	0.116465	0.1381207
1894.593	16	0.402165	0.3654931	0.1247658	0.08793902	0.114741	0.1391857
1894.693	16	0.390477	0.3531524	0.1262813	0.08762269	0.112277	0.1382687
1894.793	16	0.392902	0.3616893	0.124208	0.08759613	0.108502	0.1374896
1894.893	16	0.399738	0.3519955	0.1218077	0.08772431	0.108002	0.1387493
1894.993	16	0.395397	0.3612968	0.1219679	0.08660299	0.106841	0.1404902
1895.093	16	0.38658	0.3606823	0.1232183	0.08484798	0.108967	0.140882
1895.193	16	0.384681	0.3622863	0.1263139	0.08653912	0.109776	0.1400075
1895.293	16	0.385068	0.3577494	0.1291609	0.08842598	0.110864	0.1401706
1895.393	16	0.386812	0.359847	0.1274301	0.08603106	0.110089	0.1420222
1895.493	16	0.386586	0.3629589	0.1299649	0.08676926	0.109545	0.1453229
1895.593	16	0.403194	0.3453251	0.1295351	0.0862903	0.112189	0.1441951
1895.693	16	0.395046	0.3430334	0.1323038	0.08718067	0.112521	0.1415873
1895.793	16	0.401588	0.3602224	0.1340694	0.08625323	0.108735	0.1404538
1895.893	16	0.402834	0.3407921	0.132651	0.0887999	0.113847	0.1375735
1895.993	16	0.409939	0.3541887	0.1339582	0.09314702	0.117324	0.1352866
1896.093	16	0.411144	0.3491971	0.1356888	0.09408433	0.11732	0.1350631
1896.193	16	0.41855	0.3361339	0.1321104	0.09250898	0.118538	0.1349701
1896.293	16	0.423818	0.3510484	0.1318262	0.08946033	0.119586	0.1376361
1896.393	16	0.421409	0.3590729	0.1317578	0.09208275	0.120474	0.1376286
1896.493	16	0.418244	0.3632624	0.1292854	0.09250658	0.123438	0.1378741
1896.593	16	0.429794	0.3517264	0.1267475	0.09637983	0.123863	0.1364892
1896.693	16	0.398596	0.3561429	0.1290392	0.09528007	0.124062	0.1368559
1896.793	16	0.382378	0.3589318	0.1275352	0.0922304	0.123244	0.1386629
1896.893	16	0.402849	0.3710502	0.1264067	0.09070168	0.123832	0.1386145
1896.993	16	0.400517	0.3644683	0.1244846	0.08759785	0.123253	0.136822
1897.093	16	0.41766	0.3537706	0.1282323	0.08844145	0.120856	0.1383576
1897.193	16	0.44093	0.3537646	0.1291224	0.08916241	0.121141	0.137084
1897.293	16	0.434063	0.3528584	0.1279899	0.08793752	0.121975	0.1372971
1897.393	16	0.407726	0.3569649	0.1267727	0.08789185	0.120103	0.1363283
1897.493	16	0.407658	0.366876	0.1258492	0.08770025	0.119153	0.1346171
1897.593	16	0.392892	0.3716721	0.1257766	0.08690601	0.118427	0.1351406
1897.693	16	0.401749	0.358358	0.1262037	0.08673126	0.116874	0.1356474
1897.793	16	0.397182	0.3670019	0.1279801	0.08760011	0.117846	0.1342067
1897.893	16	0.410663	0.3668961	0.1297754	0.08584235	0.116309	0.1337502
1897.993	16	0.407167	0.3660083	0.129664	0.08819628	0.115575	0.1343992
1898.093	16	0.411492	0.3623407	0.1254919	0.08971326	0.112712	0.1352709
1898.193	16	0.412208	0.3718023	0.1228402	0.08573698	0.112898	0.1334397
1898.293	16	0.398123	0.376444	0.1234319	0.08524956	0.112866	0.1318029
1898.393	16	0.398277	0.3831045	0.1236527	0.08538527	0.10934	0.1300811
1898.493	16	0.392404	0.380212	0.1236177	0.08497955	0.107552	0.1325633

1898.593	16	0.398488	0.3835515	0.1232307	0.08621118	0.109285	0.1324793
1898.693	16	0.401438	0.3741532	0.1219982	0.08670166	0.108158	0.1320129
1898.793	16	0.389804	0.3767467	0.122948	0.08834253	0.108231	0.1307438
1898.893	16	0.385166	0.3728469	0.1227362	0.08773156	0.108565	0.1327022
1898.993	16	0.381683	0.3648842	0.1214593	0.09158893	0.109273	0.1383597
1899.093	16	0.388185	0.365887	0.1213463	0.09111723	0.112753	0.1398339
1899.193	16	0.389844	0.371265	0.1232447	0.08911772	0.112991	0.136697
1899.293	16	0.400789	0.3614826	0.1239672	0.08894446	0.111504	0.138487
1899.393	16	0.396872	0.3610449	0.1247798	0.09052219	0.113475	0.1412891
1899.493	16	0.403142	0.3576904	0.1229548	0.08868457	0.112578	0.1425054
1899.593	16	0.402083	0.367377	0.1238387	0.08806008	0.114272	0.1439274
1899.693	16	0.41036	0.3617823	0.1279932	0.08767438	0.116839	0.1426921
1899.793	16	0.429388	0.3628406	0.1288167	0.08661713	0.119133	0.1416254
1899.893	16	0.423142	0.3684215	0.1324199	0.09109597	0.118624	0.1402508
1899.993	16	0.434272	0.3692932	0.1341856	0.09215235	0.114929	0.138748
1900.093	16	0.430315	0.3629811	0.1325585	0.0943726	0.115715	0.1385851
1900.193	16	0.425518	0.3692772	0.131867	0.09481465	0.117381	0.1386101
1900.293	16	0.431641	0.3616125	0.1296388	0.09576161	0.122029	0.1357787
1900.393	16	0.429197	0.3574999	0.1261878	0.09334815	0.124727	0.137642
1900.493	16	0.38822	0.3583062	0.1267791	0.09123689	0.121085	0.1396561
1900.593	16	0.395542	0.35782	0.1275592	0.08806402	0.120834	0.1363251
1900.693	16	0.408311	0.3602268	0.1248477	0.08584933	0.120753	0.1328743
1900.793	16	0.396049	0.3638338	0.1275209	0.08630676	0.120791	0.1331544
1900.893	16	0.437892	0.3633759	0.126872	0.08759262	0.119127	0.1338181
1900.993	16	0.433305	0.3618745	0.1244111	0.0889815	0.120186	0.1327659
1901.093	16	0.424116	0.3574497	0.1206548	0.08771493	0.121087	0.1306702
1901.193	16	0.398281	0.3619363	0.1218265	0.08815493	0.119953	0.1320691
1901.293	16	0.401663	0.3601257	0.121601	0.08870208	0.118115	0.1316085
1901.393	16	0.394039	0.3651429	0.1215094	0.08628249	0.117959	0.1313677
1901.493	16	0.402162	0.3540353	0.1227001	0.08512115	0.118937	0.1334976
1901.593	16	0.397856	0.3642835	0.1238468	0.08760623	0.117533	0.1337516
1901.693	16	0.405337	0.3729632	0.1244203	0.08932856	0.117993	0.1346739
1901.793	16	0.406891	0.3703854	0.1241292	0.08917121	0.117422	0.1381958
1901.893	16	0.403241	0.3629631	0.1221704	0.09223538	0.118057	0.1369767
1901.993	16	0.401589	0.3554324	0.1271987	0.08838935	0.119062	0.133989
1902.093	16	0.398938	0.3597999	0.1276763	0.08748428	0.116258	0.1316426
1902.193	16	0.392207	0.3709309	0.1286759	0.08692165	0.112406	0.1324277
1902.293	16	0.392194	0.3790743	0.1269044	0.08782852	0.111289	0.1332232
1902.393	16	0.396129	0.3684093	0.1294201	0.08558776	0.110362	0.1318789
1902.493	16	0.393372	0.3563598	0.1270783	0.08581657	0.10908	0.1331325
1902.593	16	0.386466	0.3653341	0.1244788	0.0830086	0.108125	0.136835
1902.693	16	0.390731	0.3717811	0.1253544	0.08501125	0.10591	0.137388
1902.793	16	0.388432	0.3614416	0.1241981	0.08804728	0.109514	0.1355668
1902.893	16	0.398107	0.3583614	0.1205638	0.08769517	0.110868	0.1372181
1902.993	16	0.419958	0.3526141	0.1192255	0.08566851	0.11123	0.1376307
1903.093	16	0.412123	0.3547416	0.1180858	0.08443648	0.109912	0.1364014
1903.193	16	0.400041	0.3587044	0.1194869	0.0875928	0.10931	0.1387548
1903.293	16	0.405986	0.3620502	0.1219786	0.08700334	0.110389	0.1402416
1903.393	16	0.400885	0.3641765	0.123959	0.08453505	0.113218	0.1420932
1903.493	16	0.416644	0.3662264	0.1263407	0.08893147	0.115987	0.1456695
1903.593	16	0.422077	0.3738905	0.1269003	0.08812236	0.119191	0.14687
1903.693	16	0.424645	0.3642562	0.1265848	0.09010874	0.121013	0.1469508
1903.793	16	0.417786	0.3569454	0.1294733	0.09103222	0.119291	0.1450533
1903.893	16	0.42244	0.3559944	0.1289082	0.09254779	0.117389	0.1392471
1903.993	16	0.422456	0.3562613	0.1309944	0.09609412	0.116248	0.1385249
1904.093	16	0.437766	0.35328	0.1313094	0.09845456	0.121382	0.1362005
1904.193	16	0.404477	0.3570791	0.1327982	0.0950994	0.123473	0.1349759
1904.293	16	0.382737	0.3586672	0.1333472	0.08995891	0.122591	0.1361677
1904.393	16	0.402628	0.3593737	0.1324892	0.09157604	0.12256	0.1362395
1904.493	16	0.40099	0.3608148	0.1294076	0.0932247	0.119602	0.1362074
1904.593	16	0.419335	0.3571628	0.1272601	0.09127448	0.119218	0.1389233
1904.693	16	0.448903	0.3593554	0.1271221	0.09337305	0.118955	0.1403797
1904.793	16	0.43361	0.3493921	0.1244485	0.09245249	0.120192	0.1386598
1904.893	16	0.412068	0.3424523	0.1242619	0.08974526	0.120433	0.1366298
1904.993	16	0.404811	0.3464808	0.1252253	0.0843625	0.12059	0.1358273
1905.093	16	0.393382	0.3540768	0.1231318	0.08669568	0.119278	0.1377727
1905.193	16	0.395922	0.3400845	0.1229257	0.08652129	0.117133	0.1321881
1905.293	16	0.39677	0.3518685	0.1222282	0.08600555	0.117526	0.1313295
1905.393	16	0.410631	0.3360925	0.1232523	0.08658095	0.115787	0.135011
1905.493	16	0.406664	0.3488654	0.124251	0.08682912	0.116606	0.1359454
1905.593	16	0.405624	0.3549317	0.1229302	0.08865051	0.117449	0.137227
1905.693	16	0.411229	0.3661115	0.1227003	0.08369858	0.115787	0.134456
1905.793	16	0.401082	0.3508951	0.1244071	0.08441734	0.114906	0.1334107
1905.893	16	0.397061	0.3528592	0.1259832	0.08426692	0.112693	0.1355159
1905.993	16	0.391633	0.3539516	0.1259498	0.08449858	0.109548	0.1358928
1906.093	16	0.395351	0.3627833	0.1284278	0.08455521	0.110437	0.1348753
1906.193	16	0.396343	0.371393	0.1277455	0.08377311	0.109213	0.1332508
1906.293	16	0.38757	0.3521299	0.1309754	0.08521064	0.107423	0.1321277
1906.393	16	0.392177	0.354227	0.1298129	0.08699913	0.106895	0.1312308
1906.493	16	0.382481	0.3534627	0.129199	0.08628221	0.107518	0.1293334
1906.593	16	0.391377	0.3554868	0.1297415	0.08636801	0.110642	0.1322799
1906.693	16	0.393116	0.3592686	0.128484	0.08656167	0.111452	0.1332048
1906.793	16	0.403938	0.37357	0.1267255	0.08776955	0.10966	0.1327755
1906.893	16	0.393188	0.3628986	0.1277429	0.08913714	0.108634	0.1325523
1906.993	16	0.401014	0.3589642	0.1261845	0.08955332	0.110372	0.1317756
1907.093	16	0.399766	0.3726705	0.1254928	0.08989905	0.11228	0.1337519
1907.193	16	0.404054	0.3686146	0.1229237	0.08884672	0.113626	0.137923
1907.293	16	0.421343	0.3581691	0.1200067	0.08906437	0.116577	0.1392003
1907.393	16	0.414716	0.3745611	0.1189012	0.09531374	0.115041	0.1417676
1907.493	16	0.420577	0.3726529	0.1185443	0.09842102	0.116682	0.1408239
1907.593	16	0.417655	0.3801689	0.1220504	0.09905746	0.120807	0.1438147
1907.693	16	0.420407	0.3809084	0.1250144	0.1026625	0.120325	0.1448089
1907.793	16	0.419891	0.3863308	0.1254925	0.1048651	0.12032	0.1445203
1907.893	16	0.429781	0.3724319	0.131863	0.1032054	0.122183	0.1476214
1907.993	16	0.384818	0.3745537	0.132595	0.09746319	0.120497	0.1412972

1908.093	16	0.39386	0.3778355	0.1300725	0.09323977	0.121294	0.1368412
1908.193	16	0.442996	0.3670752	0.1328554	0.0922971	0.12006	0.1378009
1908.293	16	0.425994	0.3642106	0.1340087	0.09220303	0.118054	0.1352577
1908.393	16	0.442824	0.3691762	0.1361357	0.09107544	0.118081	0.1359031
1908.493	16	0.429644	0.3683597	0.1390091	0.09207714	0.121254	0.1366388
1908.593	16	0.419373	0.3594792	0.1336174	0.0901838	0.12263	0.1366948
1908.693	16	0.398015	0.3567726	0.1303071	0.09073292	0.120599	0.1395228
1908.793	16	0.407409	0.3664205	0.1301779	0.08909081	0.11922	0.1394297
1908.893	16	0.390844	0.3683873	0.130761	0.08784853	0.117992	0.1381424
1908.993	16	0.40137	0.3597818	0.1280467	0.08738477	0.119674	0.1356688
1909.093	16	0.398475	0.3702975	0.1277363	0.08606402	0.117976	0.1367785
1909.193	16	0.406845	0.3646473	0.1255514	0.08621583	0.117479	0.1338845
1909.293	16	0.404844	0.3665546	0.1252081	0.08816031	0.116697	0.1353356
1909.393	16	0.406725	0.3672474	0.1241087	0.08754476	0.113447	0.1311864
1909.493	16	0.406325	0.3689045	0.1249158	0.08578628	0.11373	0.1333348
1909.593	16	0.399681	0.3582678	0.1261302	0.08817506	0.113121	0.1355011
1909.693	16	0.390956	0.3543682	0.1257288	0.08898983	0.109234	0.1357234
1909.793	16	0.392101	0.3539127	0.1227213	0.08818229	0.10787	0.1374288
1909.893	16	0.393176	0.3595097	0.1223702	0.09054682	0.108746	0.1350176
1909.993	16	0.391081	0.3638685	0.1233339	0.08956455	0.109008	0.1361953
1910.093	16	0.390242	0.3605449	0.1232552	0.08697228	0.110692	0.1375709
1910.193	16	0.391783	0.3610392	0.1248096	0.08659388	0.108338	0.1341418
1910.293	16	0.386394	0.3559518	0.1287487	0.08796836	0.106542	0.1328045
1910.393	16	0.396587	0.3595302	0.1276664	0.08696938	0.109841	0.1329204
1910.493	16	0.393358	0.3515663	0.1302483	0.08577975	0.107883	0.1295474
1910.593	16	0.399928	0.373548	0.1299994	0.08675217	0.10688	0.1277064
1910.693	16	0.403282	0.3576692	0.130754	0.08689097	0.108374	0.1269184
1910.793	16	0.409541	0.3652208	0.1289892	0.08423158	0.112086	0.1277762
1910.893	16	0.405013	0.3718281	0.1253478	0.08533013	0.109679	0.1297651
1910.993	16	0.412225	0.369527	0.127271	0.08484049	0.114206	0.1332789
1911.093	16	0.410214	0.3632731	0.1242452	0.08490521	0.114849	0.132185
1911.193	16	0.415556	0.355983	0.1247078	0.08979902	0.11116	0.1293518
1911.293	16	0.418259	0.3575194	0.1226368	0.0932902	0.108544	0.1337831
1911.393	16	0.418734	0.3672078	0.1212604	0.1000316	0.115476	0.1374965
1911.493	16	0.411672	0.3759667	0.1210074	0.1008212	0.11804	0.1402821
1911.593	16	0.43085	0.3704521	0.1237187	0.1022893	0.120757	0.144083
1911.693	16	0.395694	0.3575002	0.1233792	0.09905058	0.121844	0.1431233
1911.793	16	0.3785	0.3630745	0.1243585	0.09497266	0.118592	0.1458263
1911.893	16	0.401155	0.3703529	0.1227244	0.09259623	0.119241	0.1475042
1911.993	16	0.393638	0.3597862	0.1217753	0.09138069	0.117041	0.1480526
1912.093	16	0.412649	0.3589653	0.1262095	0.08965584	0.117872	0.1438386
1912.193	16	0.432892	0.3541432	0.1293838	0.09266984	0.117798	0.1372131
1912.293	16	0.427158	0.3570629	0.1286465	0.08839346	0.121166	0.1384631
1912.393	16	0.407707	0.3587646	0.1305584	0.08667707	0.121891	0.1381093
1912.493	16	0.407264	0.3618542	0.134756	0.08730675	0.118018	0.1374414
1912.593	16	0.393642	0.3624751	0.1365401	0.08721835	0.117109	0.1365024
1912.693	16	0.394851	0.3651783	0.13698	0.08650351	0.118161	0.1344145
1912.793	16	0.400498	0.3727573	0.1340247	0.08607735	0.121798	0.1341163
1912.893	16	0.406353	0.3646435	0.1344188	0.08583871	0.118612	0.1363058
1912.993	16	0.404327	0.3538569	0.1331966	0.08550838	0.118933	0.1369457
1913.093	16	0.404613	0.35674	0.130285	0.0849289	0.116119	0.1327954
1913.193	16	0.413098	0.3547973	0.1303354	0.08467199	0.114527	0.1353166
1913.293	16	0.400259	0.3539711	0.1314165	0.08588429	0.110766	0.1361646
1913.393	16	0.396503	0.3507698	0.1319211	0.08550073	0.107387	0.136436
1913.493	16	0.388071	0.3570391	0.12952	0.08588603	0.10508	0.1350378
1913.593	16	0.391643	0.3584583	0.1277903	0.08641015	0.103865	0.1357484
1913.693	16	0.391782	0.3620314	0.1266994	0.08688041	0.104252	0.1354265
1913.793	16	0.38532	0.3554735	0.1260089	0.08738448	0.106306	0.1347647
1913.893	16	0.393562	0.3540795	0.1271906	0.08723348	0.105714	0.1378773
1913.993	16	0.37899	0.3517838	0.1277543	0.08734855	0.106992	0.1340235
1914.093	16	0.38969	0.3364925	0.127786	0.0869711	0.108949	0.1348183
1914.193	16	0.395043	0.3390878	0.1260551	0.08780476	0.109435	0.1343413
1914.293	16	0.395906	0.354814	0.1269112	0.08885063	0.109579	0.1360482
1914.393	16	0.393937	0.3296995	0.1295467	0.09097563	0.110487	0.1338318
1914.493	16	0.396563	0.3495404	0.1319863	0.08781798	0.11118	0.1339282
1914.593	16	0.401904	0.3444953	0.1338102	0.08635635	0.114074	0.1323342
1914.693	16	0.4005	0.3392861	0.1293414	0.08623186	0.113693	0.1336089
1914.793	16	0.417628	0.3539346	0.1306295	0.08939753	0.117656	0.1339681
1914.893	16	0.412305	0.3602369	0.1287251	0.09332395	0.114847	0.1341393
1914.993	16	0.422635	0.3574484	0.1266172	0.09691574	0.114654	0.1375702
1915.093	16	0.426159	0.3525287	0.1265356	0.09858244	0.117664	0.1377248
1915.193	16	0.421982	0.3567955	0.1275134	0.09974736	0.119665	0.1367293
1915.293	16	0.423327	0.3564016	0.1245377	0.1015985	0.120451	0.1357444
1915.393	16	0.432376	0.3751235	0.1266311	0.1028866	0.121814	0.1360317
1915.493	16	0.390041	0.3562014	0.1226334	0.09862593	0.120514	0.1376344
1915.593	16	0.394266	0.3556398	0.1197863	0.09150943	0.122224	0.1394566
1915.693	16	0.411019	0.3531129	0.1190185	0.08967066	0.119142	0.142603
1915.793	16	0.404296	0.354103	0.1193667	0.09044915	0.116327	0.139842
1915.893	16	0.444991	0.3551805	0.1207137	0.08813735	0.117141	0.1388734
1915.993	16	0.428993	0.372886	0.1238716	0.08821346	0.116489	0.1424681
1916.093	16	0.415341	0.3670171	0.1225228	0.08723522	0.118091	0.1453047
1916.193	16	0.39244	0.3545813	0.1272689	0.0874846	0.11741	0.1448595
1916.293	16	0.400015	0.3744771	0.1271366	0.08694164	0.115476	0.14108
1916.393	16	0.389444	0.3672171	0.1304803	0.08734346	0.116487	0.1388245
1916.493	16	0.399727	0.3612255	0.1354944	0.08786687	0.117557	0.1368499
1916.593	16	0.394011	0.3667063	0.134717	0.08706772	0.118187	0.1349112
1916.693	16	0.402794	0.3705852	0.1353101	0.08671302	0.115762	0.1360047
1916.793	16	0.403492	0.3758604	0.1378921	0.08498957	0.113266	0.1358637
1916.893	16	0.410331	0.3838746	0.134975	0.08519758	0.113366	0.1376546
1916.993	16	0.406074	0.3817641	0.1338475	0.08505726	0.11518	0.1384243
1917.093	16	0.400418	0.3752996	0.1332788	0.0853431	0.114419	0.1379119
1917.193	16	0.391153	0.3747549	0.1292964	0.08539641	0.109742	0.133975
1917.293	16	0.390847	0.3756246	0.127668	0.08526092	0.109337	0.1331026
1917.393	16	0.393266	0.3707258	0.1287675	0.08889364	0.107292	0.1346453
1917.493	16	0.391099	0.3646668	0.1260593	0.08804866	0.10772	0.1346551

1917.593	16	0.388501	0.3616636	0.1237083	0.08874985	0.106346	0.135187
1917.693	16	0.392032	0.3696664	0.127172	0.08897583	0.105898	0.1325876
1917.793	16	0.38943	0.3608133	0.1265823	0.08717071	0.107572	0.1374295
1917.893	16	0.397067	0.3591654	0.1280094	0.08737321	0.107047	0.1370246
1917.993	16	0.393706	0.3637663	0.1264302	0.08851754	0.103919	0.1354378
1918.093	16	0.401748	0.3681117	0.1269983	0.08807778	0.101956	0.1361621
1918.193	16	0.403743	0.3582086	0.1265763	0.09026178	0.10575	0.1373451
1918.293	16	0.406659	0.3675421	0.1240855	0.08902581	0.107783	0.1367944
1918.393	16	0.424704	0.3680388	0.1230726	0.08626772	0.111042	0.1371401
1918.535	16	0.423095	0.365853	0.1255867	0.08568078	0.113741	0.1377119
1918.635	16.5	0.428742	0.3679373	0.1300875	0.08729781	0.115534	0.1393207
1918.735	16.5	0.42226	0.3698971	0.1328418	0.09454963	0.114715	0.1361786
1918.835	16.5	0.425599	0.3558303	0.1308149	0.09537178	0.114438	0.1334595
1918.935	16.5	0.44316	0.3576892	0.1313342	0.0955976	0.116894	0.134622
1919.035	16.5	0.401604	0.3585115	0.1313889	0.09302552	0.117136	0.1372295
1919.135	16.5	0.394163	0.3587408	0.1275561	0.08882138	0.118721	0.1362954
1919.235	16.5	0.404932	0.3652226	0.127389	0.08569971	0.119918	0.1347143
1919.335	16.5	0.397165	0.3607768	0.1288074	0.0852417	0.116429	0.1349214
1919.435	16.5	0.430765	0.3654608	0.1272749	0.08797352	0.116823	0.1350386
1919.535	16.5	0.429708	0.3569399	0.1263976	0.08971677	0.115484	0.133174
1919.635	16.5	0.420945	0.3638801	0.1240751	0.08915867	0.113919	0.1335897
1919.735	16.5	0.395455	0.3557219	0.1224094	0.09007408	0.115806	0.138555
1919.835	16.5	0.401577	0.3800609	0.1336467	0.08956198	0.116655	0.1421616
1919.935	16.5	0.390629	0.3606471	0.135588	0.0910451	0.115576	0.1392243
1920.035	16.5	0.402523	0.3675635	0.1349465	0.09131714	0.115719	0.1381879
1920.135	16.5	0.397632	0.3707512	0.1373157	0.09073393	0.114395	0.1378257
1920.235	16.5	0.401027	0.3705194	0.1334614	0.0911034	0.114767	0.1397807
1920.335	16.5	0.404323	0.3670033	0.1314475	0.08851669	0.116511	0.1423384
1920.435	16.5	0.409637	0.3567755	0.1328704	0.08726971	0.115568	0.1434337
1920.535	16.5	0.40179	0.3590283	0.1283731	0.08476684	0.114724	0.1416522
1920.635	16.5	0.396499	0.3676035	0.1245982	0.08587471	0.113573	0.1409692
1920.735	16.5	0.389282	0.3756986	0.1261721	0.08742227	0.114484	0.1370355
1920.835	16.5	0.390863	0.3746695	0.1243804	0.08874571	0.112607	0.1364085
1920.935	16.5	0.389812	0.3587615	0.1244327	0.08739614	0.111542	0.1390621
1921.035	16.5	0.388222	0.3578275	0.1267167	0.08583418	0.109654	0.1400404
1921.135	16.5	0.397344	0.3712879	0.1273205	0.08906195	0.107056	0.1397336
1921.235	16.5	0.388386	0.3620768	0.1289366	0.08983608	0.108681	0.1385428
1921.335	16.5	0.406924	0.3578141	0.1288424	0.0886089	0.107919	0.1360371
1921.435	16.5	0.407631	0.3565302	0.1284761	0.08837254	0.108562	0.1356297
1921.535	16.5	0.401036	0.3561004	0.1276568	0.08647925	0.110394	0.1371618
1921.635	16.5	0.39112	0.3533145	0.1250003	0.08607144	0.109901	0.1385899
1921.735	16.5	0.397145	0.3597382	0.1276237	0.08681407	0.111995	0.1379017
1921.835	16.5	0.398778	0.3617165	0.1309329	0.08387942	0.111518	0.1360519
1921.935	16.5	0.402376	0.3672407	0.1319007	0.08359834	0.110931	0.1384298
1922.035	16.5	0.412834	0.3697653	0.1331207	0.08353084	0.109765	0.138231
1922.135	16.5	0.40821	0.3709653	0.132096	0.08775438	0.113858	0.1375003
1922.235	16.5	0.413079	0.3554153	0.1334512	0.09119865	0.115926	0.1372085
1922.335	16.5	0.417537	0.3618625	0.1314648	0.09422906	0.117942	0.1338398
1922.435	16.5	0.420909	0.3559961	0.1305967	0.09788465	0.117281	0.1335565
1922.535	16.5	0.433003	0.3596901	0.1320044	0.09966426	0.116857	0.1369442
1922.635	16.5	0.406934	0.3490572	0.1309894	0.09759934	0.116821	0.1367395
1922.735	16.5	0.39182	0.3579862	0.1299111	0.09262855	0.118014	0.1359041
1922.835	16.5	0.402764	0.361005	0.1283248	0.09132431	0.122335	0.136941
1922.935	16.5	0.393468	0.3666573	0.1231543	0.09161007	0.128143	0.1366515
1923.035	16.5	0.424924	0.3559292	0.1211989	0.09241693	0.126586	0.1332102
1923.135	16.5	0.424139	0.3572235	0.1226525	0.08980283	0.126492	0.1315097
1923.235	16.5	0.40765	0.3579255	0.1242147	0.08729434	0.123571	0.1289754
1923.335	16.5	0.389465	0.342015	0.1258038	0.08526029	0.118966	0.1308259
1923.435	16.5	0.401339	0.3427829	0.1258948	0.08326356	0.117253	0.1334203
1923.535	16.5	0.391205	0.3577116	0.1281312	0.0861249	0.117752	0.1339322
1923.635	16.5	0.400681	0.3315253	0.1291671	0.08829752	0.120434	0.134649
1923.735	16.5	0.395678	0.3540047	0.1302206	0.09065524	0.117897	0.1349614
1923.835	16.5	0.404083	0.3476782	0.1329978	0.09195565	0.11854	0.1366675
1923.935	16.5	0.404018	0.3391907	0.1360338	0.09145261	0.120959	0.1366549
1924.035	16.5	0.404956	0.3540194	0.1360563	0.09032656	0.123497	0.1392142
1924.135	16.5	0.403531	0.3600731	0.1381421	0.08558211	0.121	0.1400259
1924.235	16.5	0.39765	0.3590617	0.1341269	0.08432387	0.120048	0.1412216
1924.335	16.5	0.391288	0.3516854	0.132806	0.08315111	0.118746	0.1391726
1924.435	16.5	0.389886	0.3561865	0.1319412	0.0842127	0.115619	0.1409766
1924.535	16.5	0.391326	0.3571626	0.1277505	0.08433197	0.113861	0.1464086
1924.635	16.5	0.389292	0.3748336	0.1281445	0.08087861	0.109743	0.1436006
1924.735	16.5	0.393769	0.3590307	0.1277305	0.08151758	0.109041	0.1412963
1924.835	16.5	0.392454	0.3551332	0.1272264	0.08478709	0.109105	0.1376738
1924.935	16.5	0.397868	0.3528094	0.1258476	0.08511093	0.107279	0.1359722
1925.035	16.5	0.404074	0.3534047	0.1256034	0.0858163	0.107973	0.1371503
1925.135	16.5	0.390682	0.3563829	0.1268667	0.08505402	0.108151	0.1353288
1925.235	16.5	0.394985	0.3720188	0.1280646	0.0865884	0.110801	0.1363931
1925.335	16.5	0.397304	0.3694511	0.1273464	0.08898766	0.113516	0.137364
1925.435	16.5	0.394939	0.3578101	0.1279811	0.08662653	0.110927	0.1348546
1925.535	16.5	0.396105	0.3699972	0.1273453	0.08392259	0.107629	0.1349466
1925.635	16.5	0.409588	0.3670398	0.1288592	0.08682404	0.110808	0.1340723
1925.735	16.5	0.408539	0.3665018	0.1259034	0.08906636	0.110485	0.1337742
1925.835	16.5	0.410299	0.3640105	0.1291316	0.09201336	0.107952	0.1348763
1925.935	16.5	0.415235	0.3707674	0.1317882	0.09293102	0.109926	0.1351514
1926.035	16.5	0.413366	0.3797569	0.1335607	0.0962393	0.113375	0.1355453
1926.135	16.5	0.415665	0.3833641	0.1309312	0.09613505	0.114524	0.1372969
1926.235	16.5	0.417121	0.3815361	0.1322697	0.0974679	0.114916	0.1361483
1926.335	16.5	0.383763	0.3836211	0.1295676	0.09185088	0.114013	0.1374031
1926.435	16.5	0.391158	0.3734333	0.1269318	0.08671955	0.117023	0.1330647
1926.535	16.5	0.393321	0.3759356	0.1260399	0.0871944	0.118637	0.1307788
1926.635	16.5	0.409537	0.3720706	0.1262926	0.08678271	0.122439	0.132057
1926.735	16.5	0.437334	0.3643229	0.1260198	0.0865804	0.125307	0.1337782
1926.835	16.5	0.421939	0.361554	0.1229264	0.08677474	0.123074	0.1356684
1926.935	16.5	0.403904	0.3709254	0.1202442	0.08565617	0.124743	0.1355624
1927.035	16.5	0.394191	0.3618651	0.1192337	0.08618066	0.120206	0.133859

1927.135	16.5	0.397446	0.3612987	0.1190025	0.08789404	0.11447	0.1340973
1927.235	16.5	0.393749	0.3577513	0.1209865	0.08669864	0.115953	0.1339041
1927.335	16.5	0.403072	0.3672557	0.1214975	0.08955464	0.119466	0.1350657
1927.435	16.5	0.398251	0.3594164	0.1217816	0.09001859	0.117677	0.1337074
1927.535	16.5	0.400468	0.36208	0.1235565	0.09012824	0.116633	0.1325618
1927.635	16.5	0.403861	0.3677302	0.1270224	0.09083481	0.115894	0.1339712
1927.735	16.5	0.408621	0.3705175	0.1258572	0.08871265	0.115687	0.1366525
1927.835	16.5	0.399696	0.3650892	0.1289099	0.0891119	0.116955	0.1338018
1927.935	16.5	0.393004	0.3700496	0.132537	0.08573614	0.118033	0.1342008
1928.035	16.5	0.391581	0.3583905	0.1340327	0.08486608	0.115484	0.1366161
1928.135	16.5	0.388473	0.3560734	0.1358945	0.08799417	0.11299	0.138026
1928.235	16.5	0.392319	0.3589768	0.132184	0.08379508	0.112204	0.139731
1928.335	16.5	0.390073	0.3580695	0.1305439	0.083855	0.111431	0.1405876
1928.435	16.5	0.396054	0.3618993	0.1291323	0.08595991	0.110337	0.1401846
1928.535	16.5	0.386294	0.3619708	0.1261942	0.08854312	0.109175	0.1416946
1928.635	16.5	0.404072	0.3660441	0.1248237	0.08580392	0.106993	0.1481349
1928.735	16.5	0.406179	0.3622217	0.1253899	0.08336076	0.107512	0.1460828
1928.835	16.5	0.389319	0.3578638	0.1260307	0.08499974	0.106919	0.1427885
1928.935	16.5	0.386884	0.363124	0.1273955	0.08657412	0.107165	0.1411757
1929.035	16.5	0.396065	0.3684649	0.1275534	0.08598159	0.10765	0.1370532
1929.135	16.5	0.394462	0.3650056	0.1303801	0.08510923	0.108005	0.1361861
1929.235	16.5	0.400349	0.3675023	0.1291915	0.08637184	0.107408	0.1368143
1929.335	16.5	0.414392	0.3691802	0.1284324	0.09103665	0.105425	0.1376283
1929.435	16.5	0.411962	0.3737756	0.1271054	0.09551591	0.106169	0.1404543
1929.535	16.5	0.412188	0.3678876	0.1251078	0.09580675	0.104786	0.1407949
1929.635	16.5	0.415178	0.3609014	0.1246463	0.09949824	0.1087	0.1408738
1929.735	16.5	0.409503	0.3556607	0.125824	0.1016119	0.109896	0.1364947
1929.835	16.5	0.430352	0.3631414	0.1284994	0.09955422	0.111478	0.1388456
1929.935	16.5	0.391021	0.3734587	0.1279813	0.09561717	0.111907	0.1361625
1930.035	16.5	0.388407	0.3771803	0.1258086	0.09045925	0.11448	0.1354392
1930.135	16.5	0.395303	0.3636201	0.1251923	0.09019145	0.114006	0.1325018
1930.235	16.5	0.395552	0.3552451	0.1252429	0.08970512	0.112162	0.1367094
1930.335	16.5	0.437849	0.3667905	0.1235629	0.09164386	0.112428	0.1371772
1930.435	16.5	0.427581	0.3675573	0.1186539	0.09232993	0.115993	0.1360984
1930.535	16.5	0.433515	0.3612745	0.1192319	0.09222821	0.119068	0.1369027
1930.635	16.5	0.395906	0.3596527	0.1213466	0.09175508	0.117987	0.1368583
1930.735	16.5	0.410448	0.3519455	0.1218626	0.08986358	0.120142	0.1375082
1930.835	16.5	0.397378	0.3524779	0.1193633	0.08657613	0.117776	0.1369608
1930.935	16.5	0.405484	0.3568708	0.1154868	0.0899128	0.114667	0.1386293
1931.035	16.5	0.394308	0.362441	0.1171594	0.08881708	0.114824	0.1396067
1931.135	16.5	0.401599	0.367001	0.1213119	0.09094604	0.11672	0.1382712
1931.235	16.5	0.402962	0.3620122	0.1221817	0.09088475	0.116536	0.1352633
1931.335	16.5	0.40441	0.3721024	0.1260133	0.08846675	0.116243	0.134345
1931.435	16.5	0.401045	0.3596572	0.1237277	0.08720446	0.116261	0.1332123
1931.535	16.5	0.398305	0.3621587	0.1250605	0.08817572	0.115693	0.1326326
1931.635	16.5	0.389088	0.3519301	0.1279034	0.08875763	0.113339	0.136154
1931.735	16.5	0.390327	0.3605306	0.1299237	0.08874758	0.113896	0.1354848
1931.835	16.5	0.39307	0.3496912	0.1319258	0.08702599	0.114866	0.1332486
1931.935	16.5	0.386722	0.3575624	0.1346655	0.08541019	0.113306	0.1337421
1932.035	16.5	0.391117	0.3587865	0.1352931	0.08864228	0.1134	0.1342929
1932.135	16.5	0.385856	0.3605211	0.1357635	0.08921049	0.111332	0.1355776
1932.235	16.5	0.400412	0.3594989	0.1332057	0.08997274	0.108297	0.1399678
1932.335	16.5	0.405682	0.3577184	0.1311692	0.0893793	0.105706	0.1396802
1932.435	16.5	0.397458	0.3602574	0.1280743	0.09167477	0.105332	0.1400452
1932.535	16.5	0.393386	0.3456049	0.126779	0.09067104	0.106161	0.1413374
1932.635	16.5	0.405089	0.3430564	0.1247841	0.08941462	0.108373	0.1429024
1932.735	16.5	0.405531	0.3511533	0.1216825	0.08731851	0.106692	0.1478294
1932.835	16.5	0.402556	0.3466052	0.1206247	0.08504924	0.108061	0.1443011
1932.935	16.5	0.415697	0.3447411	0.1228335	0.0889687	0.11003	0.1420863
1933.035	16.5	0.409744	0.3502825	0.1244228	0.09496865	0.10995	0.1441467
1933.135	16.5	0.412646	0.3375181	0.1290762	0.09506913	0.110179	0.1411006
1933.235	16.5	0.418568	0.3501671	0.1301418	0.09714768	0.111679	0.1397784
1933.335	16.5	0.413079	0.3575218	0.129732	0.1003723	0.111288	0.1408117
1933.435	16.5	0.422988	0.3667352	0.1290562	0.1026983	0.111762	0.1375483
1933.535	16.5	0.415997	0.3504005	0.1276639	0.1023585	0.111608	0.1383562
1933.635	16.5	0.386211	0.3526384	0.1272165	0.09661927	0.111627	0.140669
1933.735	16.5	0.396486	0.3585685	0.1273493	0.09473725	0.109293	0.1389476
1933.835	16.5	0.398531	0.3665655	0.1305453	0.09502834	0.110041	0.1349386
1933.935	16.5	0.42705	0.3716614	0.1309391	0.09298421	0.113672	0.1356723
1934.035	16.5	0.456809	0.3547787	0.1330246	0.09196065	0.110756	0.1341338
1934.135	16.5	0.438057	0.3540787	0.1324468	0.09167037	0.112831	0.1354092
1934.235	16.5	0.405281	0.3538727	0.1298767	0.08907352	0.112917	0.1360615
1934.335	16.5	0.409437	0.3578556	0.128146	0.09010452	0.113989	0.1369424
1934.435	16.5	0.396707	0.3636971	0.1228421	0.0897393	0.11494	0.1401461
1934.535	16.5	0.399141	0.3756117	0.1230889	0.08881897	0.116981	0.1402019
1934.635	16.5	0.398417	0.3649555	0.1219012	0.08801024	0.113032	0.1387987
1934.735	16.5	0.401172	0.3623771	0.1243697	0.08851494	0.115347	0.1365205
1934.835	16.5	0.408063	0.3690079	0.1209761	0.0880105	0.115256	0.1365626
1934.935	16.5	0.402491	0.369832	0.1176304	0.08823167	0.11285	0.1371441
1935.035	16.5	0.403613	0.3591488	0.1177792	0.08911067	0.114235	0.136406
1935.135	16.5	0.399327	0.3745022	0.1188227	0.08723813	0.112777	0.1340702
1935.235	16.5	0.394135	0.3762244	0.1218645	0.08680633	0.111256	0.1336272
1935.335	16.5	0.391747	0.3830344	0.1266456	0.08801683	0.11386	0.1334824
1935.435	16.5	0.393233	0.3796991	0.1286698	0.0885847	0.115149	0.1345241
1935.535	16.5	0.390675	0.3874036	0.1314792	0.08836611	0.115251	0.1309748
1935.635	16.5	0.390957	0.3721132	0.1325666	0.08608639	0.113925	0.1303956
1935.735	16.5	0.391933	0.3735564	0.1319229	0.08526965	0.115575	0.1346904
1935.835	16.5	0.39389	0.375993	0.1321104	0.08787411	0.114828	0.1377501
1935.935	16.5	0.409137	0.365596	0.1330191	0.08926121	0.111278	0.1356137
1936.035	16.5	0.390766	0.3627024	0.1365501	0.08937817	0.109618	0.1326672
1936.135	16.5	0.401195	0.3675433	0.1354499	0.09116123	0.108559	0.1343488
1936.235	16.5	0.397413	0.3664043	0.1306943	0.09100407	0.10915	0.1351859
1936.335	16.5	0.401506	0.3594506	0.1298313	0.08985547	0.109218	0.1361189
1936.435	16.5	0.395594	0.3543486	0.1290131	0.08779111	0.109627	0.1375179
1936.535	16.5	0.406091	0.3625985	0.1267418	0.08951707	0.110017	0.1393481

1936.635	16.5	0.41487	0.3667717	0.1253373	0.09302079	0.111943	0.1407055
1936.735	16.5	0.412399	0.3568635	0.1250007	0.09335048	0.110601	0.1393315
1936.835	16.5	0.407494	0.3661115	0.1225708	0.09387373	0.107845	0.1416159
1936.935	16.5	0.415012	0.3605144	0.1221145	0.09664679	0.107615	0.1420799
1937.035	16.5	0.416795	0.3623804	0.1191329	0.09838932	0.109432	0.1399381
1937.135	16.5	0.428834	0.3637823	0.1233085	0.09714541	0.113726	0.1384603
1937.235	16.5	0.395284	0.3657379	0.1268869	0.09351009	0.112946	0.1382812
1937.335	16.5	0.40454	0.3532802	0.1284726	0.09068911	0.11309	0.1366884
1937.435	16.5	0.408911	0.35514	0.1288229	0.09253125	0.115218	0.1409113
1937.535	16.5	0.418429	0.3566158	0.1268238	0.09330625	0.115002	0.1409241
1937.635	16.5	0.44667	0.3613512	0.1268516	0.09117712	0.114883	0.1383307
1937.735	16.5	0.414403	0.366165	0.1263206	0.09030236	0.114076	0.1409616
1937.835	16.5	0.403955	0.3643497	0.12921	0.09212023	0.119218	0.1394891
1937.935	16.5	0.391771	0.3636268	0.127827	0.09219011	0.123281	0.1353568
1938.035	16.5	0.399503	0.3576926	0.12879	0.09098535	0.1219	0.1304888
1938.135	16.5	0.399154	0.3655325	0.1272183	0.0900245	0.121007	0.1340942
1938.235	16.5	0.404041	0.3516451	0.1275207	0.08688352	0.11671	0.1357522
1938.335	16.5	0.400774	0.3775516	0.1267743	0.0854405	0.115537	0.1367141
1938.435	16.5	0.403251	0.3608374	0.123043	0.08572797	0.114617	0.1374933
1938.535	16.5	0.40286	0.3691584	0.1247377	0.08382361	0.114909	0.1373652
1938.635	16.5	0.404782	0.3736503	0.123019	0.08299571	0.114501	0.1363608
1938.735	16.5	0.399791	0.3706001	0.1232945	0.08041627	0.113118	0.137111
1938.835	16.5	0.395828	0.3673448	0.1222517	0.0789285	0.114249	0.134103
1938.935	16.5	0.389928	0.3560575	0.1190528	0.07974663	0.11259	0.1327416
1939.035	16.5	0.391158	0.359314	0.1176036	0.08330697	0.114616	0.1340327
1939.135	16.5	0.391904	0.3675136	0.1181816	0.07968579	0.112684	0.1364058
1939.235	16.5	0.387982	0.3781826	0.1191039	0.08111354	0.11431	0.1376635
1939.335	16.5	0.395187	0.3733709	0.1198114	0.08461052	0.112127	0.1368157
1939.435	16.5	0.386981	0.3581849	0.1232806	0.0833598	0.110783	0.1348652
1939.535	16.5	0.404935	0.3591353	0.1295167	0.08511554	0.114399	0.1344352
1939.635	16.5	0.396985	0.3700507	0.1333711	0.08671086	0.110724	0.1328169
1939.735	16.5	0.396119	0.3623057	0.1322561	0.08588374	0.10752	0.1328898
1939.835	16.5	0.391944	0.3585797	0.1323601	0.08777744	0.106132	0.1383907
1939.935	16.5	0.397369	0.3539018	0.1327314	0.08703453	0.106484	0.1382575
1940.035	16.5	0.392644	0.3544053	0.1355035	0.09046775	0.106203	0.1378829
1940.135	16.5	0.400804	0.35544	0.1377912	0.09127384	0.103514	0.1405988
1940.235	16.5	0.405479	0.3610131	0.1333095	0.09456671	0.105304	0.1417623
1940.335	16.5	0.406967	0.3623194	0.1324803	0.09918137	0.109175	0.1430232
1940.435	16.5	0.411095	0.3655993	0.1310676	0.097431	0.109395	0.1395778
1940.535	16.5	0.415544	0.3727592	0.1289093	0.0972302	0.110688	0.1399694
1940.635	16.5	0.407042	0.3690668	0.1265212	0.09709909	0.108628	0.1379749
1940.735	16.5	0.431739	0.3555462	0.1265447	0.09588507	0.109332	0.1329692
1940.835	16.5	0.396931	0.3590015	0.1270704	0.09427688	0.112975	0.1329836
1940.935	16.5	0.407275	0.3577864	0.1277397	0.09134497	0.115635	0.1327849
1941.035	16.5	0.409865	0.3566695	0.1255534	0.0878076	0.113828	0.134553
1941.135	16.5	0.411233	0.3549306	0.1272624	0.08574144	0.116672	0.1369738
1941.235	16.5	0.439333	0.3576635	0.1276612	0.08589687	0.119801	0.1363069
1941.335	16.5	0.445798	0.3598341	0.1278666	0.08407121	0.117337	0.1317531
1941.435	16.5	0.432435	0.3654372	0.1311275	0.08479822	0.116231	0.129003
1941.535	16.5	0.402134	0.3574945	0.1312636	0.08338077	0.114053	0.1299386
1941.635	16.5	0.410739	0.3590092	0.1307449	0.08337229	0.117542	0.1315703
1941.735	16.5	0.399999	0.3545596	0.1298974	0.08334176	0.117914	0.1319986
1941.835	16.5	0.407549	0.3417571	0.1330255	0.0849314	0.11791	0.1329508
1941.935	16.5	0.398916	0.3460235	0.1319842	0.08545978	0.11709	0.1354589
1942.035	16.5	0.39978	0.3591265	0.1315918	0.08577082	0.115899	0.1343052
1942.135	16.5	0.404754	0.3333211	0.1290051	0.0832818	0.117	0.1340104
1942.235	16.5	0.40601	0.3541256	0.1284087	0.08528104	0.116169	0.1334308
1942.335	16.5	0.403017	0.3451139	0.1259151	0.08638787	0.115939	0.1331833
1942.435	16.5	0.400563	0.3429767	0.1220336	0.08434989	0.112757	0.1335277
1942.535	16.5	0.388474	0.3556481	0.1224696	0.08338761	0.113336	0.1369436
1942.635	16.5	0.39398	0.3626294	0.1248651	0.08664484	0.112153	0.1342921
1942.735	16.5	0.395642	0.3566973	0.1234902	0.08852848	0.110127	0.1316184
1942.835	16.5	0.38803	0.3515986	0.1208713	0.08696816	0.111649	0.133128
1942.935	16.5	0.392006	0.3562052	0.1170786	0.08523475	0.114883	0.1320274
1943.035	16.5	0.391647	0.3578769	0.118015	0.08638797	0.116706	0.1321104
1943.135	16.5	0.399293	0.3781551	0.1189085	0.08790161	0.115866	0.1318753
1943.235	16.5	0.399982	0.3576711	0.1184649	0.08747607	0.115675	0.1323144
1943.335	16.5	0.386248	0.3553713	0.1216237	0.08629054	0.114661	0.1305999
1943.435	16.5	0.396986	0.3541923	0.1200546	0.08694744	0.110933	0.1330711
1943.535	16.5	0.39793	0.3556313	0.1210192	0.08790996	0.110123	0.1336452
1943.635	16.5	0.395652	0.3558793	0.1267613	0.08854396	0.108192	0.1343988
1943.735	16.5	0.392315	0.3738537	0.1283454	0.08523303	0.108195	0.1369601
1943.835	16.5	0.403033	0.3671501	0.1296051	0.08686297	0.106867	0.1383838
1943.935	16.5	0.400866	0.3561358	0.1297869	0.09248175	0.108199	0.1393178
1944.035	16.5	0.410714	0.3768638	0.1317537	0.09363764	0.108966	0.1374741
1944.135	16.5	0.411912	0.3651774	0.133999	0.09798545	0.109194	0.1406778
1944.235	16.5	0.412768	0.3606135	0.1275483	0.09790177	0.10799	0.1412429
1944.335	16.5	0.42117	0.3675616	0.1245757	0.09796868	0.106071	0.1414993
1944.435	16.5	0.417585	0.3728756	0.1234748	0.09624422	0.108831	0.1352317
1944.535	16.5	0.396307	0.3790486	0.1243283	0.09235395	0.109039	0.1357809
1944.635	16.5	0.396124	0.3853146	0.121943	0.09220786	0.113732	0.1360237
1944.735	16.5	0.393624	0.3820858	0.1221671	0.09173469	0.114717	0.1346801
1944.835	16.5	0.420247	0.3732427	0.1206388	0.0907006	0.113317	0.1339192
1944.935	16.5	0.430549	0.3745775	0.1208322	0.09098829	0.110911	0.1328873
1945.035	16.5	0.40565	0.3748523	0.1223455	0.09010357	0.115264	0.1324704
1945.135	16.5	0.393051	0.3711698	0.12715	0.08887771	0.11843	0.133119
1945.235	16.5	0.392759	0.3653186	0.1281252	0.08732691	0.121154	0.1322189
1945.335	16.5	0.400681	0.3608499	0.1250205	0.08734166	0.119521	0.1293654
1945.435	16.5	0.401381	0.3697874	0.1229137	0.08859155	0.124257	0.12887
1945.535	16.5	0.404626	0.3621855	0.1228243	0.08990711	0.123799	0.1331737
1945.635	16.5	0.402632	0.3589946	0.1257661	0.08920111	0.119133	0.1331766
1945.735	16.5	0.402862	0.3629674	0.1260964	0.08751847	0.115824	0.1342281
1945.835	16.5	0.399854	0.3684947	0.1265834	0.08647734	0.114942	0.1360041
1945.935	16.5	0.40873	0.3582827	0.1227182	0.08719897	0.116214	0.1352683
1946.035	16.5	0.399193	0.3672302	0.1240241	0.08660256	0.118344	0.1358745

1946.135	16.5	0.392155	0.3647822	0.1211736	0.08407456	0.116771	0.1348238
1946.235	16.5	0.38997	0.3625191	0.1220901	0.0851573	0.115319	0.1348639
1946.335	16.5	0.393735	0.3660881	0.1235282	0.08614462	0.116646	0.1355898
1946.435	16.5	0.392316	0.3703423	0.1215729	0.08563013	0.118076	0.1376344
1946.535	16.5	0.389415	0.3546355	0.1211758	0.08891688	0.119392	0.138731
1946.635	16.5	0.393396	0.3569292	0.1218864	0.09114249	0.119105	0.13835
1946.735	16.5	0.388971	0.3583984	0.1204396	0.0926393	0.119411	0.136122
1946.835	16.5	0.400532	0.3608517	0.1193629	0.09175999	0.118943	0.1350307
1946.935	16.5	0.385948	0.3662571	0.1164252	0.08920017	0.116468	0.1350542
1947.035	16.5	0.391697	0.3620904	0.116554	0.08939004	0.115509	0.1334835
1947.135	16.5	0.388752	0.365525	0.1186898	0.09102985	0.113011	0.1299989
1947.235	16.5	0.405891	0.3569874	0.1198467	0.09256081	0.112145	0.1331683
1947.335	16.5	0.401794	0.3637908	0.1212128	0.09138758	0.110088	0.1325029
1947.435	16.5	0.407769	0.3546262	0.1217886	0.09258231	0.109858	0.1320676
1947.535	16.5	0.421207	0.3799884	0.1267343	0.09161803	0.10833	0.1327122
1947.635	16.5	0.419255	0.3606568	0.1300258	0.09207106	0.107242	0.1346736
1947.735	16.5	0.419742	0.3693481	0.1317822	0.09625944	0.108512	0.1331522
1947.835	16.5	0.424783	0.3734525	0.1344157	0.1011825	0.1091	0.1325275
1947.935	16.5	0.421794	0.3712083	0.135908	0.1029301	0.10781	0.1348098
1948.035	16.5	0.438032	0.3674484	0.1371892	0.1004618	0.108805	0.1372749
1948.135	16.5	0.40162	0.3561335	0.1367218	0.09371849	0.109005	0.1397207
1948.235	16.5	0.404916	0.359055	0.1330035	0.08983173	0.109205	0.1397094
1948.335	16.5	0.39624	0.3758408	0.1292614	0.08960588	0.108836	0.1386923
1948.435	16.5	0.404739	0.3584237	0.1269749	0.0939509	0.109962	0.1342556
1948.535	16.5	0.454878	0.3590698	0.1238201	0.09385138	0.112296	0.1358001
1948.635	16.5	0.411614	0.3685105	0.1198353	0.09286501	0.113688	0.1336157
1948.735	16.5	0.403108	0.3603817	0.1212542	0.09222373	0.112386	0.1320719
1948.835	16.5	0.389821	0.3599325	0.121016	0.09203863	0.112473	0.1343201
1948.935	16.5	0.402324	0.351232	0.124964	0.09315327	0.112175	0.1319162
1949.035	16.5	0.395061	0.3503765	0.1236614	0.09296844	0.113976	0.1326077
1949.135	16.5	0.410059	0.3561568	0.1257724	0.09385669	0.122181	0.1353317
1949.235	16.5	0.402851	0.3609484	0.1275791	0.09307806	0.129945	0.1338893
1949.335	16.5	0.404165	0.3651673	0.1287717	0.0886694	0.126458	0.1293582
1949.435	16.5	0.402458	0.364158	0.1260947	0.08754951	0.120603	0.1287225
1949.535	16.5	0.405596	0.3720456	0.1242785	0.08742107	0.11543	0.1321609
1949.635	16.5	0.396309	0.3542891	0.1234282	0.08867875	0.115533	0.1329028
1949.735	16.5	0.395553	0.3592016	0.1231736	0.08972009	0.114452	0.1330193
1949.835	16.5	0.386306	0.3549204	0.1267592	0.09060269	0.11355	0.1311913
1949.935	16.5	0.391244	0.3545259	0.1251793	0.09155092	0.113714	0.1331793
1950.035	16.5	0.393143	0.3498161	0.1266139	0.08897309	0.112577	0.1346673
1950.135	16.5	0.387564	0.3555829	0.1252886	0.08914706	0.11152	0.135031
1950.235	16.5	0.393551	0.3595238	0.1252052	0.08716624	0.110807	0.1322281
1950.335	16.5	0.378448	0.3603356	0.1247847	0.08887304	0.113862	0.1330531
1950.435	16.5	0.398051	0.3586295	0.1217599	0.08549205	0.112593	0.1336467
1950.535	16.5	0.39315	0.3582409	0.1233449	0.08359495	0.112938	0.1352101
1950.635	16.5	0.385506	0.3402756	0.125579	0.08400016	0.110502	0.1344109
1950.735	16.5	0.387399	0.3412067	0.1239669	0.08683716	0.109486	0.1323523
1950.835	16.5	0.393505	0.3541525	0.1215281	0.08794802	0.107116	0.1315092
1950.935	16.5	0.388803	0.3324015	0.1182804	0.08708562	0.105293	0.1311554
1951.035	16.5	0.396537	0.3521816	0.1177853	0.08708423	0.108487	0.1318419
1951.135	16.5	0.403455	0.3430087	0.1178709	0.08996572	0.109195	0.1291078
1951.235	16.5	0.408833	0.342908	0.1175181	0.09326756	0.10983	0.1294408
1951.335	16.5	0.422464	0.3546395	0.1230917	0.09741051	0.112463	0.1294044
1951.435	16.5	0.422179	0.3643879	0.1237855	0.1007402	0.114301	0.1325761
1951.535	16.5	0.416627	0.3506769	0.1240415	0.1011107	0.11139	0.1320035
1951.635	16.5	0.432046	0.3523236	0.1283369	0.0998724	0.10869	0.1329445
1951.735	16.5	0.40259	0.3559739	0.1275121	0.09532121	0.111073	0.1329478
1951.835	16.5	0.40549	0.3689894	0.1300065	0.09206405	0.111303	0.1332063
1951.935	16.5	0.39969	0.3641259	0.1353532	0.08872991	0.108998	0.1335518
1952.035	16.5	0.403425	0.3548412	0.1370344	0.08750603	0.111383	0.136237
1952.135	16.5	0.457578	0.3530172	0.1366812	0.08829297	0.112711	0.1416118
1952.235	16.5	0.440461	0.3536476	0.1311366	0.09046703	0.118216	0.142847
1952.335	16.5	0.416693	0.3553766	0.131053	0.09075316	0.119307	0.1434346
1952.435	16.5	0.393192	0.3724495	0.1285456	0.09266984	0.116733	0.1376567
1952.535	16.5	0.409599	0.3668319	0.1249989	0.09084497	0.117412	0.1373396
1952.635	16.5	0.403602	0.3560352	0.1229204	0.08846904	0.126462	0.1358255
1952.735	16.5	0.41052	0.3719086	0.1220431	0.08721093	0.125933	0.1345728
1952.835	16.5	0.40288	0.3675089	0.1186478	0.08636974	0.124956	0.1356406
1952.935	16.5	0.403782	0.3563358	0.121625	0.08499458	0.120907	0.1343314
1953.035	16.5	0.407762	0.3721377	0.1229357	0.08213972	0.120339	0.1326532
1953.135	16.5	0.402185	0.374994	0.1251602	0.0829898	0.120385	0.1355373
1953.235	16.5	0.403049	0.3816694	0.1265898	0.08289164	0.119503	0.1343212
1953.335	16.5	0.402208	0.3779516	0.1257906	0.08014926	0.119416	0.1318754
1953.435	16.5	0.387632	0.3797396	0.1234673	0.07988507	0.117783	0.1314115
1953.535	16.5	0.390342	0.371978	0.1233883	0.08166943	0.117072	0.1366963
1953.635	16.5	0.392678	0.3726931	0.1252354	0.08231885	0.115953	0.1372837
1953.735	16.5	0.383928	0.3687449	0.125432	0.08206317	0.116889	0.1383715
1953.835	16.5	0.390069	0.3638467	0.1295591	0.08231659	0.117706	0.138388
1953.935	16.5	0.384481	0.3616473	0.1297911	0.08144636	0.116417	0.1379491
1954.035	16.5	0.39268	0.3684239	0.1304006	0.08125809	0.114832	0.1363059
1954.135	16.5	0.398425	0.3597869	0.1262924	0.0816267	0.115979	0.134807
1954.235	16.5	0.385845	0.3560945	0.1259386	0.08210553	0.113768	0.1320944
1954.335	16.5	0.392526	0.3657652	0.124453	0.08198071	0.111535	0.1321383
1954.435	16.5	0.391633	0.366997	0.1208648	0.08061749	0.107737	0.1303283
1954.535	16.5	0.390545	0.3588081	0.1237322	0.08166452	0.106104	0.1351115
1954.635	16.5	0.388782	0.3636963	0.1232731	0.08302584	0.108207	0.1339689
1954.735	16.5	0.396106	0.3646038	0.1224244	0.08693498	0.109239	0.1314228
1954.835	16.5	0.399171	0.3626485	0.1214134	0.08926225	0.107376	0.1306302
1954.935	16.5	0.405366	0.3682672	0.1206482	0.09437734	0.109217	0.1307639
1955.035	16.5	0.413093	0.3515464	0.1188318	0.09697757	0.112418	0.1292817
1955.135	16.5	0.412482	0.3533027	0.1201436	0.1013958	0.11335	0.1297363
1955.235	16.5	0.414692	0.3568743	0.1212391	0.1036345	0.114263	0.1298811
1955.335	16.5	0.410283	0.3591875	0.1245183	0.1013587	0.11352	0.1291369
1955.435	16.5	0.382226	0.3645401	0.1256043	0.09596097	0.112462	0.1305981
1955.535	16.5	0.390993	0.3612689	0.1263782	0.09395085	0.116105	0.1302914

1955.635	16.5	0.393849	0.3617395	0.1304836	0.09328156	0.117242	0.1292562
1955.735	16.5	0.425544	0.356723	0.1304449	0.09454298	0.11657	0.1293223
1955.835	16.5	0.4466	0.3604406	0.1326808	0.09315336	0.118045	0.1320177
1955.935	16.5	0.423267	0.36826	0.1357744	0.09223945	0.119947	0.1350563
1956.035	16.5	0.412844	0.3626075	0.138035	0.09105801	0.121778	0.1365819
1956.135	16.5	0.402195	0.3678312	0.1383407	0.09073008	0.117714	0.1392495
1956.235	16.5	0.404913	0.3650393	0.1307169	0.09015278	0.12234	0.1407468
1956.335	16.5	0.397245	0.3698035	0.1290938	0.08971652	0.125084	0.1391052
1956.435	16.5	0.407304	0.365173	0.1275648	0.08938158	0.122058	0.1332633
1956.535	16.5	0.41064	0.3535799	0.1251865	0.09000729	0.117956	0.1311935
1956.635	16.5	0.404825	0.3588517	0.1223326	0.0878913	0.117323	0.1326169
1956.735	16.5	0.396556	0.3694024	0.1224923	0.08650249	0.119865	0.1293006
1956.835	16.5	0.404976	0.3771868	0.1203283	0.08616351	0.118284	0.132496
1956.935	16.5	0.397684	0.3664994	0.118561	0.08453125	0.118961	0.1319672
1957.035	16.5	0.395757	0.3558126	0.1195088	0.08530344	0.116298	0.1334231
1957.135	16.5	0.387001	0.366394	0.122145	0.08560435	0.117111	0.1367114
1957.235	16.5	0.393156	0.3638912	0.1245677	0.08743341	0.114673	0.1374887
1957.335	16.5	0.392702	0.3566544	0.1249228	0.08463585	0.113784	0.1345802
1957.435	16.5	0.389074	0.3569025	0.124543	0.08456101	0.112774	0.132322
1957.535	16.5	0.397279	0.3550216	0.1218355	0.08716021	0.112747	0.1354314
1957.635	16.5	0.381759	0.3519139	0.1255849	0.08806881	0.114131	0.1331841
1957.735	16.5	0.398994	0.3591553	0.1256792	0.08797508	0.113498	0.1351518
1957.835	16.5	0.387525	0.3616734	0.1283808	0.08586672	0.112083	0.1349882
1957.935	16.5	0.396357	0.3657679	0.1288893	0.08637009	0.112163	0.1348732
1958.035	16.5	0.399301	0.3703207	0.127556	0.08665849	0.109375	0.1327541
1958.135	16.5	0.404867	0.3609338	0.1238854	0.08348224	0.106805	0.1325654
1958.235	16.5	0.404698	0.3579369	0.1242973	0.08087619	0.106595	0.1324996
1958.335	16.5	0.40921	0.3506013	0.1233712	0.0838367	0.108723	0.1339922
1958.435	16.5	0.414204	0.3619381	0.1189792	0.08501546	0.108083	0.1335323
1958.535	16.5	0.420821	0.3487741	0.1216383	0.08916132	0.106302	0.1359867
1958.635	16.5	0.430225	0.3549921	0.121787	0.09342603	0.10706	0.134232
1958.735	16.5	0.446573	0.3576693	0.1193704	0.09907334	0.108057	0.1319765
1958.835	16.5	0.441494	0.3625635	0.11761	0.1039395	0.108406	0.1337854
1958.935	16.5	0.450598	0.3541679	0.1182071	0.1042577	0.11039	0.1356792
1959.035	16.5	0.402217	0.3552079	0.1174314	0.1001337	0.11278	0.1361896
1959.135	16.5	0.409005	0.3506262	0.1191575	0.09565213	0.113816	0.1351402
1959.235	16.5	0.40646	0.340139	0.1233643	0.09717089	0.108892	0.1386148
1959.335	16.5	0.411929	0.3429311	0.1272838	0.09519808	0.109854	0.1365557
1959.435	16.5	0.46203	0.3536391	0.1266833	0.09506951	0.10926	0.1359052
1959.535	16.5	0.432229	0.3349651	0.1300338	0.09640847	0.109693	0.1359249
1959.635	16.5	0.419893	0.3495943	0.131492	0.09493639	0.114653	0.1361183
1959.735	16.5	0.399589	0.3354945	0.1297397	0.09201194	0.116464	0.1330376
1959.835	16.5	0.40828	0.3484793	0.1342347	0.09027968	0.115814	0.1384891
1959.935	16.5	0.399684	0.3538361	0.1343544	0.08823183	0.120339	0.1408269
1960.035	16.5	0.414406	0.3580682	0.1363792	0.08726092	0.116861	0.1426317
1960.135	16.5	0.407457	0.3499252	0.1385452	0.08797502	0.115875	0.1437201
1960.235	16.5	0.404106	0.3521869	0.1325381	0.08715925	0.115397	0.142762
1960.335	16.5	0.401411	0.3544795	0.1299181	0.08462854	0.114022	0.1440197
1960.435	16.5	0.405814	0.3715087	0.1284864	0.08226259	0.114689	0.1360007
1960.535	16.5	0.398894	0.3561015	0.1254779	0.08218619	0.118783	0.1325384
1960.635	16.5	0.400258	0.3539469	0.124657	0.08256967	0.116717	0.134261
1960.735	16.5	0.386672	0.3510711	0.1241618	0.08151861	0.118603	0.1333323
1960.835	16.5	0.389807	0.3539788	0.1202438	0.08468848	0.116737	0.1343158
1960.935	16.5	0.390373	0.3587683	0.1215542	0.08558591	0.116596	0.1328686
1961.035	16.5	0.382864	0.3717369	0.1216415	0.08684048	0.117108	0.1347422
1961.135	16.5	0.394547	0.3616873	0.1253973	0.08548401	0.116761	0.1402903
1961.235	16.5	0.383711	0.3617052	0.1261232	0.08598966	0.115098	0.1379455
1961.335	16.5	0.398597	0.3644399	0.1284994	0.08805885	0.111194	0.1352471
1961.435	16.5	0.39973	0.3644021	0.1274808	0.08578021	0.11009	0.1313428
1961.535	16.5	0.389011	0.3615471	0.1261129	0.08559597	0.109303	0.1328605
1961.635	16.5	0.394511	0.3697695	0.1271843	0.08691526	0.107568	0.1323897
1961.735	16.5	0.411236	0.3776914	0.1244007	0.0880845	0.1071	0.1309955
1961.835	16.5	0.411881	0.3834603	0.1288367	0.08377615	0.105414	0.1310444
1961.935	16.5	0.404634	0.3795098	0.1301374	0.08059465	0.106101	0.1300033
1962.035	16.5	0.409629	0.3721227	0.129836	0.0846883	0.10728	0.1299492
1962.135	16.5	0.407611	0.3709906	0.126678	0.08785365	0.107174	0.1310482
1962.235	16.5	0.418572	0.3733548	0.1262595	0.09103073	0.106702	0.1303063
1962.335	16.5	0.436453	0.3631413	0.1239223	0.09262583	0.110066	0.1330283
1962.435	16.5	0.446527	0.3597875	0.1218059	0.09635421	0.110508	0.1344635
1962.535	16.5	0.452336	0.3664109	0.1222145	0.09570075	0.10909	0.1338118
1962.635	16.5	0.43365	0.3597842	0.1231898	0.09570122	0.111109	0.1329112
1962.735	16.5	0.407427	0.3612051	0.1219466	0.09460289	0.113255	0.1320168
1962.835	16.5	0.400027	0.3566769	0.1223337	0.09167135	0.115661	0.1328724
1962.935	16.5	0.399499	0.3650151	0.1207974	0.09158921	0.114081	0.1332154
1963.035	16.5	0.458904	0.3584346	0.1197368	0.09195371	0.111622	0.1342946
1963.135	16.5	0.450243	0.3646251	0.1213749	0.09453045	0.110952	0.1328562
1963.235	16.5	0.414077	0.3652668	0.1201731	0.09353925	0.109571	0.1356202
1963.335	16.5	0.392809	0.3621971	0.1212591	0.09049371	0.113709	0.135713
1963.435	16.5	0.396921	0.366276	0.1173892	0.08912661	0.11715	0.1318811
1963.535	16.5	0.397984	0.3648317	0.120771	0.09016671	0.122871	0.1292784
1963.635	16.5	0.413655	0.3547489	0.1263748	0.08885413	0.119483	0.1334276
1963.735	16.5	0.413633	0.3549365	0.1293803	0.08806808	0.114491	0.1346865
1963.835	16.5	0.409362	0.3549496	0.1319351	0.08781552	0.114655	0.1378993
1963.935	16.5	0.405432	0.3567598	0.1340422	0.08390742	0.115442	0.1396057
1964.035	16.5	0.402416	0.3619947	0.132885	0.08522324	0.114978	0.1406445
1964.135	16.5	0.402377	0.3627179	0.1310017	0.08569074	0.116107	0.1415982
1964.235	16.5	0.402257	0.3590675	0.1264032	0.0861357	0.116708	0.1427291
1964.335	16.5	0.390279	0.3567293	0.1258699	0.08525312	0.11633	0.1433233
1964.435	16.5	0.388368	0.3560176	0.127251	0.08705314	0.113074	0.1376125
1964.535	16.5	0.388197	0.3755874	0.1242379	0.08873385	0.114761	0.1339605
1964.635	16.5	0.383974	0.3585545	0.1223434	0.08924164	0.112657	0.1331571
1964.735	16.5	0.389938	0.3662096	0.1208462	0.0865901	0.113542	0.1345866
1964.835	16.5	0.390258	0.3686538	0.1197168	0.08521257	0.11067	0.1353376
1964.935	16.5	0.395281	0.3670197	0.1219634	0.08586296	0.114425	0.1359146
1965.035	16.5	0.404777	0.3619114	0.1213482	0.08458989	0.113393	0.1376761

1965.135	16.5	0.386666	0.3527853	0.1233196	0.08592262	0.113497	0.1375545
1965.235	16.5	0.396038	0.357882	0.1277519	0.0870226	0.109063	0.1338837
1965.335	16.5	0.40543	0.3692642	0.1284461	0.08748955	0.108271	0.1291925
1965.435	16.5	0.416093	0.3764116	0.1280639	0.084892	0.107101	0.1297581
1965.535	16.5	0.404301	0.3632933	0.1257115	0.08413888	0.106768	0.131477
1965.635	16.5	0.419007	0.355197	0.1257582	0.08704907	0.108965	0.1344371
1965.735	16.5	0.419681	0.3664865	0.1256184	0.08947095	0.108357	0.1334171
1965.835	16.5	0.423768	0.36228	0.1274847	0.09218896	0.108091	0.1333335
1965.935	16.5	0.438153	0.3556371	0.1249622	0.09297369	0.111444	0.1326684
1966.035	16.5	0.458496	0.3552762	0.1251841	0.09737775	0.112343	0.1336493
1966.135	16.5	0.451089	0.3534853	0.1251595	0.1039792	0.110028	0.1329339
1966.235	16.5	0.439101	0.3528917	0.1250177	0.1023619	0.111426	0.1336859
1966.335	16.5	0.415157	0.3594071	0.1252643	0.09847053	0.112088	0.1371145
1966.435	16.5	0.429653	0.3636446	0.12226	0.09457006	0.115025	0.1375021
1966.535	16.5	0.430634	0.3637014	0.1216476	0.09265392	0.117174	0.1399596
1966.635	16.5	0.454534	0.3683952	0.1215086	0.09135131	0.114657	0.1367438
1966.735	16.5	0.481214	0.354691	0.1198507	0.09447151	0.114792	0.1349347
1966.835	16.5	0.456583	0.3596766	0.1188545	0.09256944	0.116416	0.1318003
1966.935	16.5	0.434298	0.3502893	0.1167553	0.09108342	0.11656	0.1330251
1967.035	16.5	0.41546	0.361005	0.1168032	0.09116426	0.116115	0.1353503
1967.135	16.5	0.424861	0.3458157	0.1164306	0.08753584	0.117855	0.1327323
1967.235	16.5	0.409512	0.353434	0.1156177	0.08787494	0.122259	0.1347954
1967.335	16.5	0.416982	0.3559402	0.1168271	0.08811081	0.118111	0.1321249
1967.435	16.5	0.4199	0.3617625	0.1166586	0.08855654	0.116429	0.1317215
1967.535	16.5	0.406032	0.3515496	0.1179673	0.08723933	0.114044	0.1284552
1967.635	16.5	0.399334	0.3545342	0.120404	0.08693797	0.115835	0.1306486
1967.735	16.5	0.408734	0.3433121	0.1254407	0.08634859	0.112434	0.1308165
1967.835	16.5	0.402592	0.3408255	0.1312552	0.08619533	0.114609	0.1339806
1967.935	16.5	0.399618	0.3473716	0.1359755	0.08320039	0.112263	0.1336494
1968.035	16.5	0.387156	0.3402986	0.1382425	0.08160686	0.1139	0.136338
1968.135	16.5	0.390322	0.3439031	0.1366702	0.08635879	0.113528	0.135901
1968.235	16.5	0.390263	0.3447096	0.1295262	0.08836217	0.116644	0.1391769
1968.335	16.5	0.384544	0.3348138	0.1269701	0.08914661	0.119149	0.140457
1968.435	16.5	0.398291	0.3488086	0.1280364	0.08889442	0.118425	0.1356902
1968.535	16.5	0.385207	0.3551757	0.1257837	0.09110199	0.118145	0.1314759
1968.635	16.5	0.409638	0.3542942	0.1222253	0.0907174	0.115199	0.1309978
1968.735	16.5	0.392312	0.3500927	0.1226684	0.08853645	0.114904	0.1309363
1968.835	16.5	0.400766	0.354626	0.1206317	0.08954354	0.112491	0.1322583
1968.935	16.5	0.403711	0.3563532	0.1222319	0.08873418	0.109087	0.1311356
1969.035	16.5	0.408137	0.3725277	0.1179811	0.08628442	0.108302	0.1320029
1969.135	16.5	0.394328	0.3530474	0.1230037	0.08556258	0.106614	0.133785
1969.235	16.5	0.399277	0.3510272	0.1273143	0.08698642	0.109344	0.1317189
1969.335	16.5	0.40323	0.3500005	0.1267063	0.08829349	0.109238	0.1296095
1969.435	16.5	0.407316	0.35426	0.1263421	0.08999389	0.109313	0.1313686
1969.535	16.5	0.424373	0.362487	0.1283098	0.09114758	0.107587	0.1330966
1969.635	16.5	0.439431	0.3700643	0.1277311	0.09098046	0.10744	0.1325569
1969.735	16.5	0.446698	0.3581572	0.1263089	0.09348523	0.106197	0.1315665
1969.835	16.5	0.448183	0.3669234	0.1254994	0.09381519	0.106379	0.1297158
1969.935	16.5	0.408416	0.3644119	0.1266823	0.09250626	0.107305	0.1315096
1970.035	16.5	0.402633	0.362119	0.1296153	0.09159618	0.112355	0.1294446
1970.135	16.5	0.401305	0.3630672	0.1283348	0.08879184	0.112713	0.1283723
1970.235	16.5	0.404478	0.3705835	0.1287812	0.087529	0.115643	0.1282042
1970.335	16.5	0.454119	0.3780415	0.126034	0.08680335	0.116177	0.1292781
1970.435	16.5	0.422222	0.3812186	0.1247526	0.08592859	0.119606	0.1305078
1970.535	16.5	0.400743	0.3812152	0.1260921	0.08338126	0.123352	0.1297682
1970.635	16.5	0.389535	0.3712373	0.12633	0.08442857	0.11957	0.1293388
1970.735	16.5	0.39326	0.3698136	0.1240903	0.08550784	0.118362	0.1256647
1970.835	16.5	0.397567	0.3711246	0.1216043	0.08565531	0.119766	0.1261636
1970.935	16.5	0.419561	0.3598775	0.1191874	0.08690447	0.118132	0.1281221
1971.035	16.5	0.408353	0.3597994	0.1197748	0.08828828	0.11774	0.1280506
1971.135	16.5	0.40472	0.3669238	0.1229544	0.08944795	0.117487	0.1284295
1971.235	16.5	0.399997	0.3584384	0.1232858	0.08802782	0.117119	0.1281238
1971.335	16.5	0.406778	0.3607773	0.1231493	0.08749186	0.118347	0.1262178
1971.435	16.5	0.405799	0.3579638	0.1191409	0.08686752	0.118154	0.1268246
1971.535	16.5	0.403287	0.3667489	0.1206104	0.08525488	0.114856	0.1282639
1971.635	16.5	0.389931	0.356268	0.1232257	0.0836559	0.111978	0.1284324
1971.735	16.5	0.3886	0.3655913	0.1259836	0.08576855	0.111545	0.1296854
1971.835	16.5	0.390755	0.3632606	0.1283511	0.08950756	0.111333	0.1336332
1971.935	16.5	0.383615	0.3601159	0.1306742	0.09012157	0.113644	0.1358697
1972.035	16.5	0.39078	0.3653957	0.1341812	0.08893863	0.113831	0.1342914
1972.135	16.5	0.380946	0.3614071	0.1370689	0.08963647	0.116497	0.1331608
1972.235	16.5	0.393776	0.3529017	0.1303226	0.09126905	0.118211	0.1342731
1972.335	16.5	0.400787	0.3544618	0.1285892	0.09095646	0.114846	0.1357417
1972.435	16.5	0.390802	0.3532076	0.1303989	0.08844558	0.110309	0.1322418
1972.535	16.5	0.390339	0.3575782	0.1258421	0.09038816	0.106748	0.1301924
1972.635	16.5	0.396107	0.3573505	0.1217798	0.09343154	0.104778	0.1311822
1972.735	16.5	0.40515	0.363099	0.1220073	0.09305628	0.101946	0.1300497
1972.835	16.5	0.399019	0.3556719	0.1211261	0.09443575	0.102546	0.1300892
1972.935	16.5	0.40716	0.3578288	0.1235694	0.09767322	0.103342	0.1279361
1973.035	16.5	0.404876	0.3511165	0.1218933	0.1005602	0.102375	0.126466
1973.135	16.5	0.420694	0.3762173	0.1257334	0.100603	0.104224	0.1283338
1973.235	16.5	0.444271	0.3583702	0.1285664	0.101716	0.105252	0.1258868
1973.335	16.5	0.45617	0.3646904	0.1254775	0.1016163	0.104217	0.1261943
1973.435	16.5	0.458005	0.3681159	0.1252218	0.09962926	0.105812	0.1273968
1973.535	16.5	0.436393	0.3664618	0.1272607	0.09972925	0.106528	0.1308468
1973.635	16.5	0.402664	0.3588493	0.1287439	0.09864186	0.104667	0.1320077
1973.735	16.5	0.39948	0.3508836	0.1269452	0.09722053	0.109894	0.1307921
1973.835	16.5	0.399928	0.3604449	0.1281588	0.09594897	0.107766	0.1283522
1973.935	16.5	0.44592	0.37126	0.1278291	0.09426551	0.110887	0.1318235
1974.035	16.5	0.450873	0.3764242	0.1284864	0.09670356	0.110037	0.1337589
1974.135	16.5	0.416661	0.3594306	0.1262231	0.0970355	0.11077	0.1315916
1974.235	16.5	0.397568	0.3548422	0.1263551	0.09499702	0.115051	0.1288959
1974.335	16.5	0.398217	0.3664727	0.1261336	0.09084689	0.115022	0.128616
1974.435	16.5	0.398926	0.3586845	0.1232105	0.09131979	0.114598	0.1316239
1974.535	16.5	0.412873	0.3569402	0.1235379	0.09448794	0.116872	0.1341097

1974.635	16.5	0.421454	0.3529202	0.1262102	0.09442841	0.117533	0.131594
1974.735	16.5	0.416803	0.3515242	0.1244673	0.09268481	0.116021	0.1299235
1974.835	16.5	0.404392	0.3548447	0.1234965	0.09043466	0.114252	0.1304475
1974.935	16.5	0.402524	0.3578373	0.1229618	0.0871558	0.113088	0.1301818
1975.035	16.5	0.410915	0.3648091	0.1204116	0.08814133	0.11368	0.1293799
1975.135	16.5	0.402524	0.356409	0.1204718	0.08839606	0.113441	0.1284577
1975.235	16.5	0.394795	0.3644228	0.1190164	0.0870384	0.111842	0.1316919
1975.335	16.5	0.386939	0.3513428	0.1188415	0.08930046	0.109209	0.1305926
1975.435	16.5	0.387132	0.3594986	0.1176912	0.09122202	0.108934	0.1280762
1975.535	16.5	0.387173	0.3511353	0.1196536	0.09169976	0.112948	0.1271052
1975.635	16.5	0.385917	0.3561813	0.125071	0.0907139	0.114077	0.1288515
1975.735	16.5	0.388861	0.3462203	0.1280772	0.09059847	0.115236	0.1323986
1975.835	16.5	0.388766	0.3514943	0.13413	0.09145387	0.115347	0.1370183
1975.935	16.5	0.402525	0.3528291	0.1377923	0.08904245	0.114974	0.1378927
1976.035	16.5	0.386213	0.3566711	0.1375975	0.08789501	0.114444	0.1397588
1976.135	16.5	0.394394	0.3510658	0.1380873	0.08803454	0.110531	0.1375037
1976.235	16.5	0.393425	0.3531001	0.130725	0.086123	0.107557	0.1401106
1976.335	16.5	0.414033	0.3380058	0.1282393	0.08390373	0.1073	0.1414988
1976.435	16.5	0.406207	0.3385175	0.1297038	0.08160376	0.109286	0.1377835
1976.535	16.5	0.412709	0.3476958	0.1274783	0.0851717	0.10977	0.1384055
1976.635	16.5	0.410913	0.3348964	0.126629	0.08775781	0.108405	0.1369364
1976.735	16.5	0.423457	0.3452515	0.12683	0.09134445	0.110473	0.1340053
1976.835	16.5	0.445369	0.3419023	0.1253282	0.09451118	0.113096	0.1356875
1976.935	16.5	0.459961	0.3342227	0.1256235	0.09949822	0.111973	0.1357088
1977.035	16.5	0.458112	0.3477914	0.124108	0.09978358	0.109532	0.1364304
1977.135	16.5	0.455661	0.3564744	0.1281227	0.1023825	0.112852	0.1364831
1977.235	16.5	0.396242	0.3507216	0.1296606	0.0996955	0.108938	0.1350663
1977.335	16.5	0.39279	0.3493914	0.1269679	0.09524373	0.11098	0.1332783
1977.435	16.5	0.409428	0.3528351	0.1275282	0.09245543	0.111839	0.1316169
1977.535	16.5	0.424886	0.3604279	0.1255009	0.09200119	0.111076	0.135264
1977.635	16.5	0.467902	0.3640694	0.1270812	0.0910219	0.111374	0.1371631
1977.735	16.5	0.42685	0.3514274	0.1261763	0.09105821	0.111831	0.1356393
1977.835	16.5	0.414442	0.3480953	0.1274634	0.08585097	0.115748	0.1356039
1977.935	16.5	0.427262	0.3497456	0.1275258	0.08402999	0.121026	0.1373839
1978.035	16.5	0.422252	0.3522137	0.1294178	0.08282543	0.123108	0.1382883
1978.135	16.5	0.407047	0.3692466	0.1273362	0.08427002	0.128008	0.1362366
1978.235	16.5	0.39915	0.3646545	0.1275516	0.08648301	0.125305	0.1338625
1978.335	16.5	0.405635	0.3533747	0.1261564	0.08514208	0.124687	0.1329568
1978.441	16.5	0.400063	0.371898	0.1262897	0.08643989	0.121687	0.1328963
1978.541	17	0.394999	0.3657365	0.1277266	0.08661819	0.120956	0.1322097
1978.641	17	0.387535	0.3544699	0.1266649	0.08426996	0.121953	0.1300089
1978.741	17	0.385719	0.366953	0.1252221	0.08448073	0.121322	0.1298323
1978.841	17	0.388665	0.3727418	0.1239057	0.08164787	0.117223	0.1297326
1978.941	17	0.387226	0.3803829	0.1226339	0.08099654	0.117118	0.129099
1979.041	17	0.387711	0.3790216	0.1208075	0.08080779	0.114487	0.1315218
1979.141	17	0.393139	0.3809305	0.1227347	0.08412074	0.115249	0.1315987
1979.241	17	0.403989	0.3688351	0.1244491	0.08565304	0.114864	0.130689
1979.341	17	0.385501	0.3705088	0.1235961	0.08316312	0.114774	0.1291861
1979.441	17	0.397148	0.3656297	0.1256099	0.08463494	0.113773	0.1278515
1979.541	17	0.400797	0.3599688	0.1266079	0.08631115	0.113485	0.1311488
1979.641	17	0.411036	0.3564349	0.130118	0.0880186	0.114025	0.1328877
1979.741	17	0.396079	0.3661864	0.1369585	0.08794045	0.11404	0.1361659
1979.841	17	0.405479	0.3592037	0.1391573	0.08930843	0.11054	0.1399769
1979.941	17	0.409611	0.3556151	0.1369272	0.09010705	0.110109	0.1381334
1980.041	17	0.4334	0.3619663	0.1295	0.09230797	0.109895	0.1376517
1980.141	17	0.456964	0.3651656	0.1281018	0.09225996	0.110042	0.1394808
1980.241	17	0.455612	0.3569137	0.132937	0.09632987	0.107962	0.1406012
1980.341	17	0.450634	0.3663813	0.1302052	0.09679411	0.108574	0.1418048
1980.441	17	0.415648	0.3631361	0.1266582	0.1001106	0.109525	0.1376983
1980.541	17	0.398773	0.3627756	0.1252408	0.09743024	0.109688	0.1384784
1980.641	17	0.412935	0.3690304	0.1239868	0.09344591	0.109278	0.1381848
1980.741	17	0.411175	0.3566245	0.1264289	0.09360933	0.108333	0.1361769
1980.841	17	0.453393	0.3509825	0.1251791	0.09122508	0.10827	0.1347153
1980.941	17	0.434228	0.3523782	0.1274639	0.09143141	0.108143	0.1327501
1981.041	17	0.415822	0.3527399	0.1272092	0.09212723	0.114919	0.1324435
1981.141	17	0.399806	0.3586856	0.1257492	0.08843003	0.116227	0.1346836
1981.241	17	0.408629	0.3569265	0.1256426	0.08776465	0.117067	0.1332795
1981.341	17	0.413398	0.3586219	0.1260119	0.08647774	0.11895	0.1336776
1981.441	17	0.421622	0.3525697	0.1252925	0.08626378	0.123561	0.1328308
1981.541	17	0.420971	0.3564043	0.1264708	0.08396613	0.126029	0.1346579
1981.641	17	0.406107	0.3554	0.1273221	0.0841781	0.125451	0.133113
1981.741	17	0.40047	0.3649414	0.1292857	0.08272447	0.127965	0.1310918
1981.841	17	0.404336	0.3608901	0.1282682	0.08227848	0.125661	0.1330895
1981.941	17	0.40192	0.3626596	0.1286216	0.08257435	0.125587	0.1369048
1982.041	17	0.398258	0.3693575	0.1286805	0.08200058	0.124191	0.1380789
1982.141	17	0.387593	0.3612199	0.125248	0.08222177	0.124202	0.1367171
1982.241	17	0.385389	0.351392	0.1256732	0.08515275	0.123385	0.13423
1982.341	17	0.391192	0.3529366	0.1268828	0.08480808	0.124842	0.133263
1982.441	17	0.383981	0.3630025	0.1252992	0.08614035	0.122544	0.1318989
1982.541	17	0.389463	0.3745214	0.1223131	0.08618905	0.122188	0.1318794
1982.641	17	0.386224	0.3715244	0.1185639	0.08755548	0.118458	0.1300898
1982.741	17	0.4042	0.3532829	0.1188853	0.08635201	0.117274	0.1317044
1982.841	17	0.388997	0.357949	0.1215351	0.08685677	0.117149	0.1321945
1982.941	17	0.395343	0.3630729	0.1208209	0.08668111	0.117195	0.130705
1983.041	17	0.395294	0.355711	0.1225913	0.08690555	0.117779	0.1320602
1983.141	17	0.394604	0.3535907	0.1246273	0.08777899	0.118756	0.1317526
1983.241	17	0.397577	0.3490569	0.1280189	0.0865541	0.11691	0.1345736
1983.341	17	0.400628	0.3483197	0.1270161	0.08841631	0.114473	0.1333083
1983.441	17	0.406388	0.3555131	0.1255439	0.08934858	0.110203	0.131248
1983.541	17	0.421581	0.3593956	0.1272399	0.09194077	0.108668	0.1301662
1983.641	17	0.4455	0.3618903	0.1310166	0.09055373	0.109591	0.1311687
1983.741	17	0.448339	0.3624025	0.1334077	0.09334163	0.109367	0.1350357
1983.841	17	0.447271	0.360342	0.1336139	0.09669214	0.110843	0.1362881
1983.941	17	0.423547	0.3506819	0.1298282	0.09835871	0.111161	0.1369996
1984.041	17	0.393622	0.3513477	0.1292707	0.09774319	0.110027	0.1376499

1984.141	17	0.404082	0.3534954	0.1302252	0.09355757	0.111542	0.137303
1984.241	17	0.407836	0.347377	0.1271337	0.09105633	0.112227	0.1372068
1984.341	17	0.434332	0.351475	0.124403	0.09143556	0.11088	0.1381582
1984.441	17	0.436962	0.3505389	0.1223749	0.08947762	0.113191	0.1363849
1984.541	17	0.41924	0.3568337	0.1231463	0.08959773	0.112333	0.1365564
1984.641	17	0.406274	0.3507207	0.1229199	0.08822096	0.1129	0.1353571
1984.741	17	0.403615	0.3511902	0.1272127	0.08624661	0.112926	0.1325091
1984.841	17	0.40888	0.3502005	0.129423	0.08486631	0.114963	0.1327702
1984.941	17	0.416148	0.334054	0.1274304	0.08685339	0.118759	0.1328799
1985.041	17	0.412955	0.3364087	0.1265119	0.0864296	0.119338	0.1331774
1985.141	17	0.417532	0.3506368	0.1259738	0.08452918	0.121434	0.133985
1985.241	17	0.40536	0.3269399	0.1249769	0.08577915	0.119649	0.1309718
1985.341	17	0.405396	0.3475882	0.1248311	0.08495553	0.120864	0.1301886
1985.441	17	0.404426	0.3348836	0.1293362	0.08798521	0.122017	0.1287599
1985.541	17	0.402174	0.3415042	0.1305662	0.08895218	0.122327	0.1307377
1985.641	17	0.390849	0.3489627	0.1305171	0.08454473	0.121512	0.1318395
1985.741	17	0.3861	0.358951	0.1269368	0.08739271	0.121355	0.1300335
1985.841	17	0.390664	0.34422	0.1265438	0.09171178	0.116793	0.1309527
1985.941	17	0.385136	0.348956	0.1250734	0.0886094	0.116682	0.1321559
1986.041	17	0.391599	0.3519555	0.1243741	0.0904408	0.114098	0.1323702
1986.141	17	0.386521	0.3675534	0.1245941	0.09028735	0.115819	0.1315626
1986.241	17	0.396935	0.3564485	0.1258067	0.08989801	0.115588	0.1302485
1986.341	17	0.396404	0.3519111	0.1246735	0.08799747	0.113738	0.131434
1986.441	17	0.394267	0.3487686	0.1224264	0.08686534	0.113516	0.1334371
1986.541	17	0.398213	0.3504695	0.1178646	0.09043791	0.113366	0.1354005
1986.641	17	0.408217	0.3524237	0.1167095	0.09316773	0.113892	0.1343838
1986.741	17	0.409971	0.3695069	0.1170799	0.09270897	0.115545	0.131581
1986.841	17	0.409012	0.3625144	0.1186335	0.09128628	0.115355	0.1300624
1986.941	17	0.417361	0.3556276	0.1210845	0.09401258	0.113573	0.1305477
1987.041	17	0.430515	0.3689058	0.1201245	0.09277645	0.108851	0.1296313
1987.141	17	0.452843	0.3642435	0.1197547	0.09324884	0.105675	0.1261648
1987.241	17	0.467157	0.3540992	0.1174008	0.09700664	0.106494	0.128186
1987.341	17	0.470197	0.367311	0.1245281	0.09742165	0.10702	0.1270924
1987.441	17	0.469486	0.3757564	0.1285872	0.09602614	0.105894	0.1264021
1987.541	17	0.424517	0.3819828	0.1323078	0.09638624	0.106473	0.1250255
1987.641	17	0.424754	0.3774898	0.1375371	0.09244047	0.106964	0.1254025
1987.741	17	0.432731	0.3725221	0.1339042	0.09083527	0.111669	0.1286441
1987.841	17	0.435647	0.3698148	0.1291487	0.09196009	0.11218	0.1325992
1987.941	17	0.471883	0.3688862	0.1308224	0.08984225	0.109794	0.1347064
1988.041	17	0.43046	0.363949	0.1296029	0.09296376	0.110692	0.1375271
1988.141	17	0.41744	0.3565106	0.1276642	0.09137432	0.110781	0.1393853
1988.241	17	0.40218	0.3614067	0.1272153	0.09298432	0.112815	0.1413046
1988.341	17	0.412443	0.3611079	0.1244098	0.09469602	0.113682	0.141073
1988.441	17	0.421728	0.357475	0.1239649	0.09273577	0.114413	0.1365672
1988.541	17	0.422845	0.3517421	0.123674	0.09082812	0.11493	0.1364785
1988.641	17	0.424445	0.3625619	0.1256465	0.08902116	0.117672	0.1365635
1988.741	17	0.398678	0.3580137	0.1267213	0.08709943	0.116355	0.1340055
1988.841	17	0.396937	0.3601078	0.128394	0.08605611	0.114932	0.129725
1988.941	17	0.408662	0.3646425	0.1268672	0.08219246	0.113605	0.1296871
1989.041	17	0.401601	0.361665	0.1250219	0.08382129	0.120957	0.1297241
1989.141	17	0.398007	0.3638969	0.1246666	0.08342244	0.119351	0.1337588
1989.241	17	0.391278	0.3652135	0.1249243	0.08225329	0.122149	0.1335076
1989.341	17	0.38606	0.3511882	0.1273935	0.08406952	0.116913	0.1326006
1989.441	17	0.387271	0.3499545	0.1271297	0.08260728	0.118098	0.1332657
1989.541	17	0.384341	0.349768	0.1286621	0.08584507	0.122529	0.1324827
1989.641	17	0.39131	0.3525922	0.1261574	0.08772001	0.123819	0.1336543
1989.741	17	0.38935	0.3574401	0.1267821	0.08627835	0.120831	0.1317606
1989.841	17	0.402011	0.355327	0.1254328	0.08540232	0.120366	0.1337269
1989.941	17	0.392866	0.3536502	0.1240798	0.08748746	0.116301	0.136597
1990.041	17	0.400235	0.3510536	0.1260257	0.08817296	0.114113	0.1361541
1990.141	17	0.398745	0.3535454	0.1237688	0.09023081	0.115772	0.1351435
1990.241	17	0.400978	0.3701307	0.1240629	0.09023142	0.117631	0.1338709
1990.341	17	0.393189	0.3530535	0.1227877	0.09189074	0.118865	0.1360256
1990.441	17	0.401333	0.3617103	0.1234318	0.09511627	0.119597	0.136236
1990.541	17	0.409774	0.3643613	0.1237501	0.09736577	0.116282	0.1347922
1990.641	17	0.441412	0.363047	0.1240343	0.09987433	0.114693	0.1342995
1990.741	17	0.449528	0.356392	0.122433	0.09928617	0.108713	0.1324438
1990.841	17	0.458939	0.3484497	0.120792	0.1009969	0.108908	0.1332812
1990.941	17	0.462391	0.3526534	0.1237983	0.09935916	0.106115	0.1330064
1991.041	17	0.415548	0.365096	0.1283616	0.09795464	0.105645	0.1306045
1991.141	17	0.401298	0.3735867	0.1333024	0.09292035	0.106704	0.1295998
1991.241	17	0.41088	0.3595847	0.1348113	0.09162495	0.105529	0.1333105
1991.341	17	0.422465	0.3509732	0.136499	0.09143819	0.110518	0.1299877
1991.441	17	0.448352	0.3605103	0.138545	0.09178387	0.111759	0.1274049
1991.541	17	0.435406	0.3578961	0.139792	0.09338254	0.109552	0.1278741
1991.641	17	0.422347	0.3514156	0.1367446	0.09203611	0.10899	0.1288116
1991.741	17	0.406145	0.3520971	0.1349202	0.09185662	0.110373	0.1309341
1991.841	17	0.416493	0.3489727	0.1328922	0.09147665	0.10994	0.1363326
1991.941	17	0.424633	0.3477542	0.1304072	0.09270891	0.109155	0.1405338
1992.041	17	0.432934	0.3554046	0.1280108	0.09157669	0.109504	0.1408311
1992.141	17	0.427248	0.3583904	0.126728	0.08972433	0.110734	0.1378958
1992.241	17	0.417102	0.3580674	0.1240696	0.08758381	0.11148	0.1362635
1992.341	17	0.408054	0.3617338	0.1259017	0.08549817	0.110937	0.1399177
1992.441	17	0.411699	0.3544109	0.1243455	0.08541447	0.114281	0.1389841
1992.541	17	0.405164	0.3529096	0.1272791	0.08465739	0.120152	0.1379248
1992.641	17	0.394066	0.3459815	0.1285813	0.08509914	0.121451	0.1357033
1992.741	17	0.390635	0.356234	0.1283266	0.08449648	0.123395	0.1313893
1992.841	17	0.38568	0.3419117	0.1305129	0.08747286	0.125229	0.1308309
1992.941	17	0.39013	0.3487335	0.1283017	0.08824815	0.121222	0.1310645
1993.041	17	0.383492	0.3498878	0.1294541	0.09061281	0.117514	0.1332497
1993.141	17	0.389144	0.3585069	0.1309958	0.09256826	0.11788	0.1363042
1993.241	17	0.386288	0.3478442	0.1350683	0.09193626	0.119409	0.1360957
1993.341	17	0.404895	0.3507903	0.1355318	0.09160704	0.118484	0.1351225
1993.441	17	0.395804	0.3412516	0.1315464	0.09142751	0.12124	0.1346182
1993.541	17	0.408003	0.3341239	0.1285238	0.09101725	0.120428	0.1362545

1993.641	17	0.398012	0.3414062	0.1271696	0.0912073	0.116501	0.1383452
1993.741	17	0.409797	0.3424875	0.1248382	0.09399065	0.11659	0.1333746
1993.841	17	0.403236	0.3387438	0.1228672	0.0930673	0.116925	0.1331369
1993.941	17	0.415804	0.3409218	0.1228191	0.09554277	0.11478	0.1314581
1994.041	17	0.424143	0.3319172	0.1214116	0.09712935	0.113385	0.1307229
1994.141	17	0.442529	0.3457723	0.1213159	0.1013995	0.113897	0.1304373
1994.241	17	0.460341	0.3495426	0.1200639	0.1000821	0.112114	0.1302599
1994.341	17	0.459208	0.3510854	0.1181809	0.1004915	0.108491	0.1309986
1994.441	17	0.467564	0.3453358	0.1163315	0.09906383	0.10733	0.131888
1994.541	17	0.450701	0.3503874	0.1159451	0.09932734	0.106293	0.1342576
1994.641	17	0.419002	0.3509786	0.1173249	0.09685557	0.107192	0.133245
1994.741	17	0.433541	0.3688902	0.1185067	0.09360144	0.108972	0.1333187
1994.841	17	0.436032	0.348031	0.1233513	0.09042946	0.109273	0.1314613
1994.941	17	0.457131	0.3476067	0.1253864	0.09333482	0.109419	0.1328831
1995.041	17	0.459785	0.3460487	0.1298152	0.09432773	0.107921	0.1337389
1995.141	17	0.43888	0.3485736	0.1332555	0.09433516	0.109764	0.1344799
1995.241	17	0.434127	0.3571606	0.1333367	0.0923075	0.109518	0.1375221
1995.341	17	0.429548	0.3655576	0.138055	0.09168569	0.110227	0.1344269
1995.441	17	0.424275	0.3554089	0.1381014	0.08967972	0.111109	0.131297
1995.541	17	0.436717	0.3623616	0.1342046	0.08850716	0.115371	0.1300066
1995.641	17	0.42761	0.3623272	0.1339733	0.08822287	0.117809	0.13186
1995.741	17	0.421086	0.3577844	0.1320782	0.08790345	0.114232	0.1343288
1995.841	17	0.40296	0.3602492	0.1281705	0.08653443	0.110984	0.1394726
1995.941	17	0.408301	0.366888	0.1271245	0.08512004	0.110819	0.1382247
1996.041	17	0.403084	0.3770541	0.124954	0.08566277	0.11491	0.1370635
1996.141	17	0.398219	0.3790675	0.1244337	0.08577614	0.12095	0.1364524
1996.241	17	0.397415	0.3789113	0.123845	0.08474366	0.120478	0.1348176
1996.341	17	0.387217	0.3681078	0.1260592	0.08357187	0.119829	0.1377623
1996.441	17	0.393438	0.3674808	0.1291361	0.08677101	0.119314	0.1358793
1996.541	17	0.383879	0.3693962	0.1295701	0.08688087	0.118021	0.136451
1996.641	17	0.383513	0.3556807	0.1308723	0.08556367	0.120684	0.1364082
1996.741	17	0.382315	0.3581932	0.1310483	0.08661011	0.120211	0.1341547
1996.841	17	0.398357	0.3659283	0.1287023	0.08816768	0.119333	0.1334445
1996.941	17	0.395788	0.3558999	0.1274052	0.0881492	0.118801	0.1318494
1997.041	17	0.392865	0.3567285	0.1302063	0.08713049	0.118368	0.1321829
1997.141	17	0.388184	0.3539924	0.1335967	0.08880595	0.120095	0.1357182
1997.241	17	0.391845	0.3622966	0.1313765	0.08882288	0.11874	0.1362081
1997.341	17	0.384531	0.3447981	0.1276247	0.08863638	0.118835	0.1355412
1997.441	17	0.393055	0.3514131	0.1266904	0.08942094	0.120755	0.1340897
1997.541	17	0.400913	0.3590336	0.1265653	0.09001561	0.115982	0.1357106
1997.641	17	0.421978	0.357436	0.1255595	0.09326355	0.113166	0.1364309
1997.741	17	0.444778	0.3614524	0.1265447	0.09269474	0.113013	0.1336887
1997.841	17	0.45355	0.3594419	0.1260981	0.09454822	0.113875	0.1343045
1997.941	17	0.471509	0.3505116	0.1242131	0.09308539	0.110995	0.1343002
1998.041	17	0.456877	0.3493331	0.1234046	0.09243282	0.107176	0.1319563
1998.141	17	0.418643	0.3491787	0.1225103	0.09249793	0.104988	0.1304921
1998.241	17	0.416762	0.3520609	0.1214462	0.09145735	0.102964	0.1295486
1998.341	17	0.430106	0.3535082	0.1214914	0.0916652	0.106563	0.1301697
1998.441	17	0.441718	0.3559953	0.1190727	0.09129848	0.107878	0.1302532
1998.541	17	0.460358	0.3507667	0.1227408	0.09137426	0.105868	0.1318859
1998.641	17	0.431405	0.3542419	0.1225323	0.09185003	0.105268	0.1281665
1998.741	17	0.419567	0.3502205	0.1248461	0.08886282	0.106672	0.1268371
1998.841	17	0.407035	0.3738141	0.1219166	0.08763263	0.10582	0.1261544
1998.941	17	0.406247	0.3526556	0.1273712	0.08788621	0.105604	0.1261582
1999.041	17	0.419223	0.3624275	0.127481	0.08682266	0.106971	0.1249297
1999.141	17	0.417055	0.3635294	0.1296177	0.08544753	0.109113	0.1252015
1999.241	17	0.428142	0.3619571	0.1334378	0.08546828	0.115035	0.1265057
1999.341	17	0.405636	0.3532235	0.1334412	0.0847192	0.113337	0.1337624
1999.441	17	0.400563	0.3480014	0.1312363	0.08348117	0.112371	0.134021
1999.541	17	0.405733	0.3544779	0.131648	0.08328176	0.113709	0.1325467
1999.641	17	0.403075	0.367025	0.128848	0.08425514	0.116186	0.1325916
1999.741	17	0.392915	0.3730848	0.1268976	0.08407831	0.119207	0.1340682
1999.841	17	0.39272	0.3547298	0.1260583	0.08690982	0.121103	0.134143
1999.941	17	0.391687	0.3517321	0.1242205	0.08760022	0.124037	0.1404271
2000.041	17	0.386496	0.3626626	0.1238677	0.08701739	0.121951	0.1393523
2000.141	17	0.386874	0.3558652	0.1253259	0.08669863	0.123192	0.1392376
2000.241	17	0.386821	0.3530715	0.1286198	0.08989508	0.118739	0.136035
2000.341	17	0.391761	0.3500873	0.1291552	0.09116458	0.117664	0.1362001
2000.441	17	0.403209	0.3475086	0.1284731	0.09011528	0.12056	0.1336485
2000.541	17	0.39181	0.3485889	0.1287935	0.08861604	0.121712	0.1306927
2000.641	17	0.391544	0.3546851	0.1263422	0.08911788	0.119918	0.1294246
2000.741	17	0.394741	0.3624298	0.1256588	0.09154117	0.12025	0.1309679
2000.841	17	0.389442	0.3549313	0.1236111	0.09229092	0.116295	0.1338491
2000.941	17	0.388224	0.3596583	0.1279213	0.08982571	0.11425	0.1346979
2001.041	17	0.398946	0.349049	0.125363	0.09178671	0.115342	0.1316551
2001.141	17	0.408576	0.3548535	0.1245513	0.09374449	0.114981	0.13225
2001.241	17	0.437024	0.3481574	0.1235503	0.09240686	0.115935	0.132485
2001.341	17	0.4354	0.3544542	0.1241386	0.09271324	0.113211	0.1351119
2001.441	17	0.454843	0.3418393	0.1245898	0.09258446	0.111743	0.1343066
2001.541	17	0.461521	0.3475878	0.1255492	0.0929573	0.11122	0.134841
2001.641	17	0.412219	0.3497654	0.1260395	0.09200833	0.107615	0.1350694
2001.741	17	0.39641	0.3576113	0.1245361	0.09091422	0.105227	0.1330287
2001.841	17	0.422276	0.348792	0.1239398	0.08898278	0.103668	0.1317029
2001.941	17	0.434926	0.3514078	0.1217014	0.09029752	0.10574	0.1279885
2002.041	17	0.466257	0.3365689	0.1176141	0.09418731	0.106593	0.1262667
2002.141	17	0.434789	0.3371123	0.1165869	0.0962194	0.108219	0.1266979
2002.241	17	0.441802	0.3443544	0.1189079	0.09368712	0.109322	0.1307167
2002.341	17	0.436033	0.336957	0.1196142	0.09089907	0.109613	0.1272078
2002.441	17	0.426232	0.3417641	0.1214875	0.09088051	0.109852	0.1288745
2002.541	17	0.433487	0.3384793	0.1207894	0.09165529	0.109795	0.1306055
2002.641	17	0.438729	0.3320828	0.1242511	0.08974425	0.11098	0.1296416
2002.741	17	0.437434	0.3451812	0.1282268	0.09095804	0.112174	0.1288092
2002.841	17	0.419218	0.3498658	0.1337367	0.09232188	0.113736	0.1289229
2002.941	17	0.40176	0.3501666	0.1327266	0.09102228	0.115214	0.1290669
2003.041	17	0.405038	0.3456597	0.1369595	0.08999641	0.113534	0.1312532

2003.141	17	0.403307	0.3493191	0.1354073	0.08882901	0.112682	0.1275914
2003.241	17	0.395772	0.3506515	0.1313855	0.08691349	0.116846	0.1274294
2003.341	17	0.399437	0.3665452	0.1298079	0.08619723	0.117541	0.1305464
2003.441	17	0.387319	0.3463274	0.1289298	0.08906323	0.122355	0.1325417
2003.541	17	0.392441	0.3465615	0.1283212	0.08670364	0.121414	0.1364786
2003.641	17	0.385929	0.3447697	0.1250863	0.08831167	0.117009	0.1360824
2003.741	17	0.388161	0.3474745	0.1279669	0.0863684	0.118829	0.1397222
2003.841	17	0.385822	0.3610614	0.1287797	0.08784264	0.118638	0.1396607
2003.941	17	0.3997	0.3633763	0.1276662	0.08992278	0.117348	0.1374211
2004.041	17	0.391377	0.3531196	0.1282166	0.09128537	0.117394	0.1376281
2004.141	17	0.392294	0.3658489	0.1309825	0.09086316	0.11605	0.1355407
2004.241	17	0.392801	0.3611816	0.1307528	0.09051209	0.114521	0.1308259
2004.341	17	0.389364	0.3555348	0.1301508	0.0913965	0.112382	0.128024
2004.441	17	0.388602	0.3611086	0.1302398	0.09146631	0.11049	0.1271366
2004.541	17	0.398255	0.3678486	0.1292544	0.09001368	0.106608	0.1286739
2004.641	17	0.40337	0.3763631	0.1278833	0.09122998	0.108237	0.1294394
2004.741	17	0.429026	0.3769777	0.1266651	0.09213787	0.113555	0.1346323
2004.841	17	0.43253	0.3814328	0.1317791	0.09319104	0.11484	0.1335429
2004.941	17	0.447247	0.3665828	0.1294243	0.09637696	0.11288	0.1311982
2005.041	17	0.473767	0.368924	0.1287631	0.09605118	0.114872	0.1303989
2005.141	17	0.435561	0.3647318	0.1268424	0.09576541	0.114231	0.1319291
2005.241	17	0.412311	0.3557589	0.1250738	0.0925296	0.108121	0.1321292
2005.341	17	0.421832	0.3558856	0.1226601	0.09019607	0.107356	0.1308723
2005.441	17	0.433336	0.3660332	0.1229441	0.08653428	0.105508	0.1337273
2005.541	17	0.45877	0.3559006	0.1241402	0.08846554	0.106345	0.1345249
2005.641	17	0.453002	0.3530929	0.1220128	0.08980048	0.108201	0.1353165
2005.741	17	0.436808	0.3590186	0.1225908	0.09289957	0.108273	0.134864
2005.841	17	0.425268	0.3617417	0.1213427	0.0879304	0.10614	0.1326779
2005.941	17	0.423787	0.3462105	0.1194286	0.0867447	0.108067	0.1329964
2006.041	17	0.42149	0.3610959	0.1186393	0.0885117	0.110152	0.1323809
2006.141	17	0.436063	0.359351	0.1196381	0.0870435	0.108145	0.1301439
2006.241	17	0.425923	0.3597691	0.1228056	0.08685976	0.108842	0.1289093
2006.341	17	0.422042	0.3625001	0.1203622	0.08711392	0.108877	0.1301201
2006.441	17	0.409658	0.3554351	0.1216886	0.08420948	0.111694	0.1297466
2006.541	17	0.406604	0.3478357	0.1252674	0.08377972	0.114301	0.1274229
2006.641	17	0.401844	0.3506306	0.1277266	0.0820369	0.112626	0.1306325
2006.741	17	0.395764	0.3495787	0.1318374	0.08179066	0.111052	0.1282286
2006.841	17	0.401881	0.3530322	0.1350056	0.08068134	0.110848	0.132507
2006.941	17	0.388529	0.3513388	0.1394618	0.08372394	0.113223	0.1318103
2007.041	17	0.389594	0.3564627	0.139048	0.08425332	0.114873	0.1309189
2007.141	17	0.388265	0.3483455	0.1321902	0.0829513	0.119053	0.1360938
2007.241	17	0.386822	0.3552244	0.1326631	0.08347056	0.119744	0.1393207
2007.341	17	0.381728	0.3482495	0.1307577	0.08578321	0.120981	0.1386324
2007.441	17	0.397562	0.3688502	0.1276793	0.08988518	0.121644	0.1397843
2007.541	17	0.399311	0.3545756	0.1241692	0.08789621	0.119789	0.1409222
2007.641	17	0.39508	0.3606241	0.1235987	0.0859877	0.119219	0.1440828
2007.741	17	0.393453	0.3627862	0.1234498	0.08427241	0.120045	0.1440487
2007.841	17	0.391824	0.3593911	0.1232811	0.08357466	0.121931	0.1433267
2007.941	17	0.386014	0.351714	0.1244321	0.08347709	0.118566	0.1388299
2008.041	17	0.39339	0.3465629	0.1275267	0.08546727	0.115616	0.1362057
2008.141	17	0.401566	0.3538422	0.127002	0.08723807	0.115486	0.1344393
2008.241	17	0.424825	0.3502842	0.1256289	0.08784988	0.116005	0.1332519
2008.341	17	0.444175	0.3582895	0.1236686	0.08997924	0.119095	0.1325275
2008.441	17	0.455877	0.3533534	0.1236316	0.09501997	0.11979	0.1350224
2008.541	17	0.485136	0.3503879	0.1243733	0.09909719	0.120451	0.1340091
2008.641	17	0.454442	0.3461155	0.1258522	0.1000084	0.118232	0.1376323
2008.741	17	0.413759	0.342348	0.1267438	0.09819954	0.115728	0.1358178
2008.841	17	0.436427	0.352637	0.1262453	0.09345958	0.112578	0.1353309
2008.941	17	0.430316	0.3584848	0.1248191	0.09070348	0.109321	0.1334895
2009.041	17	0.439639	0.3562265	0.1257122	0.09166522	0.107602	0.1353761
2009.141	17	0.466069	0.3570352	0.124524	0.09230497	0.106757	0.134013
2009.241	17	0.446688	0.3464419	0.1252181	0.0912588	0.106127	0.1320564
2009.341	17	0.432904	0.3532616	0.1244866	0.08853399	0.107765	0.1333536
2009.441	17	0.430267	0.3478904	0.1272845	0.0872595	0.121989	0.1357699
2009.541	17	0.420125	0.3462646	0.1265202	0.08649108	0.123263	0.1332087
2009.641	17	0.447365	0.3461522	0.12439	0.08520816	0.121854	0.1304925
2009.741	17	0.434195	0.348397	0.1242603	0.08465365	0.121202	0.127035
2009.841	17	0.443117	0.3538637	0.1224289	0.08356228	0.122598	0.1278237
2009.941	17	0.413377	0.34834	0.1211164	0.08290821	0.11952	0.1276488
2010.041	17	0.409341	0.3469411	0.1214104	0.08389512	0.11974	0.1272822
2010.141	17	0.407746	0.3432232	0.1211906	0.08237013	0.117606	0.1273605
2010.241	17	0.401085	0.3324727	0.1201611	0.08193311	0.117255	0.1270338
2010.341	17	0.399689	0.3418844	0.1233202	0.08142237	0.118795	0.1285589
2010.441	17	0.394048	0.3356425	0.1276554	0.08121484	0.116981	0.1280749
2010.541	17	0.386759	0.340822	0.1326766	0.08338145	0.114333	0.1286095
2010.641	17	0.389908	0.3383425	0.1331419	0.08618507	0.114004	0.1280525
2010.741	17	0.387573	0.3321371	0.1331997	0.0886435	0.115773	0.1287284
2010.841	17	0.378236	0.3446745	0.1331124	0.09294222	0.113292	0.1296496
2010.941	17	0.392119	0.3516869	0.1325428	0.09329208	0.111984	0.1305311
2011.041	17	0.402044	0.3428335	0.1280816	0.09356266	0.108952	0.1314442
2011.141	17	0.395804	0.3465134	0.1313299	0.09304062	0.108178	0.1337187
2011.241	17	0.402517	0.3458601	0.1292608	0.09285543	0.106932	0.1363252
2011.341	17	0.407347	0.365303	0.1267816	0.09412163	0.106845	0.1351015
2011.441	17	0.398328	0.3494025	0.1271895	0.09278163	0.105648	0.134187
2011.541	17	0.399694	0.3464672	0.1272344	0.0938709	0.107652	0.1346111
2011.641	17	0.403035	0.3437491	0.1275437	0.09689341	0.108741	0.1355322
2011.741	17	0.424346	0.346485	0.1254337	0.1008053	0.110198	0.1386031
2011.841	17	0.449117	0.3573597	0.1282454	0.1000015	0.112221	0.1374037
2011.941	17	0.446748	0.3633772	0.1293494	0.1012376	0.11132	0.1369233
2012.041	17	0.464558	0.3509847	0.1283554	0.1022006	0.114322	0.1355284
2012.141	17	0.462855	0.3654069	0.1290299	0.1036473	0.112236	0.1350534
2012.241	17	0.406319	0.3609116	0.1284953	0.1023516	0.111873	0.1342423
2012.341	17	0.401198	0.3486782	0.1286795	0.09930395	0.115201	0.1313459
2012.441	17	0.422103	0.3615012	0.1255235	0.09597562	0.116109	0.132075
2012.541	17	0.435036	0.3698789	0.1295163	0.09617526	0.119738	0.134275

2012.641	17	0.460147	0.3782559	0.1319402	0.09352405	0.120615	0.1324977
2012.741	17	0.427168	0.3746905	0.1298348	0.09529714	0.121808	0.1336642
2012.841	17	0.416896	0.3691899	0.1266059	0.09329356	0.12077	0.1336871
2012.941	17	0.421576	0.3667751	0.127789	0.09417158	0.116869	0.1338105
2013.041	17	0.413259	0.3674598	0.1252002	0.09521528	0.116241	0.1313249
2013.141	17	0.429336	0.3560306	0.1239088	0.09553844	0.114908	0.1327727
2013.241	17	0.430052	0.3553056	0.1239951	0.09484702	0.114904	0.1318927
2013.341	17	0.43442	0.3642966	0.1242869	0.09136715	0.116898	0.1305047
2013.441	17	0.420565	0.355822	0.127252	0.09120924	0.115721	0.1291568
2013.541	17	0.40585	0.350679	0.1262228	0.08805724	0.11526	0.1280169
2013.641	17	0.408635	0.3597379	0.1226469	0.08596142	0.112942	0.1266476
2013.741	17	0.403852	0.3588926	0.1197466	0.08645681	0.110364	0.1284773
2013.841	17	0.397015	0.3475483	0.121743	0.08719319	0.113759	0.1301429
2013.941	17	0.396713	0.3613915	0.1230849	0.08760612	0.115822	0.1266891
2014.041	17	0.389478	0.3600503	0.1223687	0.08954482	0.112059	0.1250604
2014.141	17	0.391331	0.3623299	0.1183122	0.08956733	0.109075	0.1263001
2014.241	17	0.391868	0.3569109	0.1213144	0.09030655	0.110289	0.1291573
2014.341	17	0.383042	0.3481629	0.1266533	0.09095411	0.108247	0.1274979
2014.441	17	0.388838	0.348187	0.1322347	0.09139973	0.106643	0.1276157
2014.541	17	0.398617	0.3477947	0.131558	0.09235574	0.105228	0.1317309
2014.641	17	0.395454	0.3533697	0.1351125	0.08754995	0.105713	0.1341369
2014.741	17	0.396134	0.3516394	0.1335185	0.0886241	0.10493	0.1350719
2014.841	17	0.395631	0.3531194	0.1329704	0.08880543	0.105538	0.1323847
2014.941	17	0.38689	0.3481515	0.1292314	0.08697905	0.10705	0.1351709
2015.041	17	0.387621	0.3506963	0.1313583	0.08585487	0.10844	0.137475
2015.141	17	0.399973	0.3596113	0.1286435	0.08721727	0.108696	0.138923
2015.241	17	0.405945	0.3540532	0.1274872	0.08883533	0.108738	0.1392012
2015.341	17	0.433764	0.3585798	0.127399	0.09001403	0.108982	0.1407957
2015.441	17	0.43312	0.3605416	0.1235322	0.09033306	0.108281	0.1384967
2015.541	17	0.438809	0.3587848	0.1235914	0.09045187	0.10786	0.1414594
2015.641	17	0.461698	0.3514392	0.1244027	0.09419837	0.10636	0.1435319
2015.741	17	0.412732	0.3433663	0.1256285	0.09572141	0.106632	0.1373857
2015.841	17	0.38928	0.3524892	0.1281488	0.09290087	0.103231	0.1376413
2015.941	17	0.407483	0.3621033	0.1282834	0.08982869	0.106587	0.1378576
2016.041	17	0.413409	0.3698068	0.1284123	0.09100188	0.110864	0.1366661
2016.141	17	0.445599	0.3499326	0.1241863	0.09089703	0.109628	0.1365109
2016.241	17	0.444202	0.3506144	0.1242644	0.09065896	0.112075	0.1349769
2016.341	17	0.435196	0.3551876	0.1227858	0.09378344	0.11506	0.1385649
2016.441	17	0.444152	0.3524952	0.1274519	0.09046934	0.117385	0.1397501
2016.541	17	0.440849	0.348866	0.1294434	0.09028308	0.118087	0.1360288
2016.641	17	0.426209	0.3438712	0.129132	0.09064448	0.116814	0.1328811
2016.741	17	0.429992	0.3425454	0.1275153	0.09083024	0.118099	0.1338084
2016.841	17	0.42376	0.3523258	0.1267453	0.08849324	0.120606	0.1315361
2016.941	17	0.422577	0.3589524	0.124848	0.08871739	0.122619	0.1321351
2017.041	17	0.413032	0.3522802	0.1248563	0.08909529	0.119807	0.1350877
2017.141	17	0.407941	0.3556075	0.1250869	0.08770552	0.11797	0.1375982
2017.241	17	0.404454	0.345799	0.1223441	0.08619007	0.115499	0.1351248
2017.341	17	0.395204	0.350638	0.1221903	0.08639972	0.116504	0.1359459
2017.441	17	0.392317	0.3471788	0.1198941	0.08532917	0.119419	0.1333827
2017.541	17	0.385768	0.3458496	0.1194701	0.08753204	0.117325	0.1321644
2017.641	17	0.394701	0.3452137	0.1215972	0.0879453	0.116572	0.130581
2017.741	17	0.385562	0.3465993	0.1238755	0.08547858	0.115344	0.1335663
2017.841	17	0.386437	0.3518717	0.1258203	0.08378786	0.114527	0.1327248
2017.941	17	0.383739	0.3459443	0.1269686	0.08739854	0.112598	0.1311889
2018.041	17	0.398869	0.3478221	0.1289202	0.08866027	0.109697	0.1305334
2018.141	17	0.397092	0.3421861	0.1312663	0.0891423	0.108695	0.1303007
2018.241	17	0.399089	0.3308569	0.131645	0.0917147	0.107266	0.1296249
2018.341	17	0.400159	0.3405575	0.1339232	0.09331319	0.104991	0.1288769
2018.441	17	0.395496	0.3339601	0.1321766	0.09226598	0.108739	0.1309342
2018.541	17	0.390417	0.3407643	0.1353986	0.09474381	0.109472	0.133718
2018.641	17	0.395448	0.336092	0.1360853	0.09523778	0.11059	0.131649
2018.741	17	0.402412	0.332191	0.1312255	0.09718979	0.109927	0.1311764
2018.841	17	0.42514	0.3443777	0.128491	0.0989432	0.110435	0.1329722
2018.941	17	0.434213	0.3528935	0.1304899	0.0980799	0.109095	0.1339426
2019.041	17	0.449012	0.3416602	0.1274072	0.09789551	0.110956	0.1410509
2019.141	17	0.461519	0.3458631	0.1255195	0.09883514	0.113525	0.1429566
2019.241	17	0.451348	0.3469145	0.1245814	0.09878691	0.117031	0.1416325
2019.341	17	0.410326	0.3649163	0.1232154	0.09679483	0.114614	0.14208
2019.441	17	0.4196	0.3497727	0.1239081	0.09029635	0.116746	0.1394324
2019.541	17	0.421765	0.346521	0.1235125	0.08962756	0.121483	0.1371793
2019.641	17	0.442184	0.3428834	0.1266499	0.09033978	0.11978	0.134563
2019.741	17	0.451502	0.345476	0.1292592	0.09012236	0.121255	0.1353061
2019.841	17	0.424132	0.3576458	0.1286702	0.08990309	0.119916	0.1335368
2019.941	17	0.409839	0.361047	0.1256574	0.08920462	0.120853	0.1333964
2020.041	17	0.406322	0.348953	0.1238182	0.08851805	0.121646	0.1324356
2020.141	17	0.409487	0.3637265	0.1243414	0.08842295	0.117422	0.1349474
2020.241	17	0.429567	0.3580857	0.1250382	0.08686179	0.117513	0.137633
2020.341	17	0.418464	0.3482318	0.1291556	0.08590099	0.116141	0.1367482
2020.441	17	0.417057	0.3586124	0.1297932	0.0842186	0.115735	0.1335368
2020.541	17	0.404286	0.3720987	0.1275845	0.08291751	0.112698	0.131309
2020.641	17	0.40718	0.3792509	0.1282554	0.0829369	0.11145	0.1306837
2020.741	17	0.402671	0.3730767	0.1250443	0.08262583	0.111883	0.1321325
2020.841	17	0.397117	0.3672456	0.1245925	0.08384717	0.114067	0.1329357
2020.941	17	0.392011	0.3659031	0.1254319	0.08359524	0.11437	0.1342606
2021.041	17	0.391464	0.3663043	0.1275489	0.08459809	0.113212	0.1339033
2021.141	17	0.387913	0.3552099	0.1257403	0.0858597	0.11056	0.1327797
2021.241	17	0.382406	0.3542323	0.1256533	0.08623673	0.111817	0.1324012
2021.341	17	0.381108	0.3630178	0.124042	0.08676627	0.110239	0.1323045
2021.441	17	0.378674	0.3569344	0.1211041	0.08716723	0.107644	0.1334959
2021.541	17	0.389185	0.3479022	0.1205972	0.0874387	0.105787	0.1361743
2021.641	17	0.397748	0.3582127	0.1211077	0.08808769	0.109306	0.1349708
2021.741	17	0.392099	0.3582567	0.1214864	0.08765224	0.109087	0.1329153
2021.841	17	0.390359	0.3476222	0.1203026	0.086762	0.106752	0.130437
2021.941	17	0.394101	0.3599793	0.1244932	0.09077343	0.106165	0.128681
2022.041	17	0.385994	0.3577726	0.1319423	0.09233919	0.106304	0.1251036

2022.141	17	0.391392	0.3596989	0.1349034	0.09195684	0.106138	0.1282882
2022.241	17	0.403676	0.3580551	0.1358982	0.09446347	0.108697	0.1309293
2022.341	17	0.416043	0.3473702	0.1378583	0.09970069	0.106803	0.1299843
2022.441	17	0.421714	0.3459422	0.1357327	0.09998307	0.104659	0.1269173
2022.541	17	0.43035	0.3475173	0.1329028	0.1000773	0.105488	0.1262355
2022.641	17	0.447633	0.3514034	0.1285928	0.1008183	0.107906	0.1257807
2022.741	17	0.448654	0.3503615	0.1281743	0.1019925	0.109245	0.1284281
2022.841	17	0.407951	0.3524139	0.1266994	0.09761751	0.108322	0.1305956
2022.941	17	0.401258	0.3467291	0.1258656	0.09168491	0.11106	0.1328561
2023.041	17	0.423468	0.3499528	0.1259177	0.08701622	0.114173	0.1384481
2023.141	17	0.436595	0.3589744	0.1235074	0.08838677	0.115897	0.1406273
2023.241	17	0.453383	0.3529882	0.1233082	0.08854	0.11252	0.1392726
2023.341	17	0.448553	0.3566135	0.1232875	0.08850148	0.111627	0.138792
2023.441	17	0.431796	0.3587937	0.1243294	0.08474874	0.110959	0.1375614
2023.541	17	0.445137	0.3574306	0.126742	0.08402029	0.113576	0.1382174
2023.641	17	0.42878	0.3503066	0.1247575	0.08452677	0.115908	0.1360452
2023.741	17	0.449776	0.3439898	0.1251173	0.08317989	0.116124	0.1347856
2023.841	17	0.444089	0.3540495	0.1225349	0.08498742	0.116589	0.1331273
2023.941	17	0.448993	0.3652158	0.1207305	0.08853821	0.118422	0.1329364
2024.041	17	0.423749	0.3690456	0.1230415	0.08745792	0.117711	0.1335908
2024.141	17	0.418214	0.3474502	0.1245898	0.08469814	0.116607	0.135774
2024.241	17	0.412883	0.3525957	0.1261895	0.08320998	0.115959	0.1332724
2024.341	17	0.402397	0.3546845	0.1267314	0.08113702	0.116198	0.1304279
2024.441	17	0.393091	0.353695	0.1248016	0.07873959	0.116146	0.1311488
2024.541	17	0.400163	0.3493486	0.1259564	0.07869364	0.115433	0.1315675
2024.641	17	0.388513	0.3445969	0.1252256	0.08413558	0.113917	0.13289
2024.741	17	0.383311	0.3451101	0.1238437	0.08398814	0.112625	0.134422
2024.841	17	0.382689	0.3536783	0.1250759	0.08353437	0.112293	0.1332912
2024.941	17	0.377407	0.3586186	0.1230435	0.08539672	0.11282	0.1330747
2025.041	17	0.382972	0.3507194	0.1217904	0.08707932	0.109634	0.1344052
2025.141	17	0.398202	0.3564818	0.1206292	0.08737296	0.107465	0.1342306
2025.241	17	0.395209	0.3464993	0.1191681	0.08808155	0.106155	0.1334297
2025.341	17	0.395746	0.3504712	0.1179201	0.08873597	0.106421	0.132036
2025.441	17	0.395217	0.3471148	0.1181736	0.09129136	0.104804	0.1329636
2025.541	17	0.392179	0.3436251	0.121748	0.09166036	0.103002	0.1307024
2025.641	17	0.392481	0.3454211	0.1242259	0.09403638	0.10134	0.131704
2025.741	17	0.404124	0.3451363	0.1282366	0.1000899	0.104629	0.1317513
2025.841	17	0.406825	0.3535464	0.1332486	0.1033396	0.105214	0.1285925
2025.941	17	0.429082	0.3419635	0.1358047	0.1057715	0.10334	0.1283628
2026.041	17	0.435288	0.3481171	0.1368084	0.1056969	0.104083	0.1268262
2026.141	17	0.460063	0.33719	0.1364621	0.1064053	0.107705	0.1274977
2026.241	17	0.463451	0.3314444	0.1392493	0.1042874	0.109011	0.1266592
2026.341	17	0.414261	0.3423979	0.1425436	0.1015693	0.110533	0.1254358
2026.441	17	0.400164	0.330149	0.1355477	0.09685281	0.106533	0.1255009
2026.541	17	0.412968	0.3436356	0.1293661	0.09174146	0.106579	0.1261217
2026.641	17	0.413559	0.3327827	0.1281906	0.08953778	0.110909	0.1295694
2026.741	17	0.442401	0.3331878	0.1291053	0.08931935	0.115196	0.1310809
2026.841	17	0.437331	0.3440404	0.1260493	0.09432139	0.11688	0.131812
2026.941	17	0.414205	0.3517505	0.1243733	0.09275673	0.119129	0.1312654
2027.041	17	0.405209	0.3407044	0.124384	0.09042496	0.119478	0.1313775
2027.141	17	0.407376	0.3460765	0.1272885	0.0908668	0.118058	0.1338393
2027.241	17	0.4093	0.3465959	0.1261984	0.0905614	0.113661	0.1383112
2027.341	17	0.417854	0.3652609	0.1258976	0.09021389	0.113164	0.1370316
2027.441	17	0.413745	0.3469459	0.1260324	0.08864087	0.115539	0.1347857
2027.541	17	0.413534	0.3456665	0.1260063	0.08833405	0.115258	0.1309242
2027.641	17	0.406852	0.3402137	0.1254575	0.08657294	0.11277	0.1311756
2027.741	17	0.398705	0.3447708	0.1250139	0.08557849	0.111179	0.1323334
2027.841	17	0.401561	0.3570583	0.1274299	0.08408244	0.11013	0.1323644
2027.941	17	0.395391	0.3603145	0.1266014	0.08383334	0.113048	0.1353751
2028.041	17	0.39303	0.3488564	0.1287406	0.08276834	0.112573	0.1354009
2028.141	17	0.387609	0.3630276	0.1272671	0.08289409	0.109566	0.1342562
2028.241	17	0.384051	0.3572192	0.1265085	0.08087759	0.110356	0.1370207
2028.341	17	0.383502	0.3479894	0.124012	0.08377814	0.110541	0.1358227
2028.441	17	0.381858	0.3583169	0.1248215	0.08458652	0.109854	0.1325786
2028.541	17	0.382525	0.370179	0.1221029	0.08498227	0.111369	0.1339684
2028.641	17	0.392958	0.3777831	0.1210406	0.0868056	0.10821	0.1347765
2028.741	17	0.385314	0.3726237	0.1225515	0.08696421	0.105869	0.1340093
2028.841	17	0.396367	0.365939	0.1221241	0.08841821	0.105095	0.1323044
2028.941	17	0.400072	0.3650307	0.1233064	0.09026057	0.103614	0.1326441
2029.041	17	0.393353	0.3651843	0.1220462	0.08949767	0.104219	0.1310868
2029.141	17	0.392029	0.3539066	0.1197019	0.08706609	0.102352	0.1311457
2029.241	17	0.401542	0.3532755	0.1175349	0.08670568	0.104247	0.1321639
2029.341	17	0.406303	0.362105	0.1166642	0.08852603	0.105279	0.1344084
2029.441	17	0.424539	0.3564894	0.1179253	0.0942187	0.104377	0.1329851
2029.541	17	0.432638	0.347061	0.1205011	0.09762632	0.105272	0.1348516
2029.641	17	0.443103	0.3581819	0.1212356	0.09865143	0.107981	0.1323903
2029.741	17	0.457758	0.3562544	0.1234441	0.1005876	0.109165	0.1322403
2029.841	17	0.43847	0.349161	0.1250635	0.09896085	0.112391	0.1322149
2029.941	17	0.401845	0.3594309	0.131002	0.09505434	0.111309	0.1307323
2030.041	17	0.413686	0.3589374	0.1319529	0.0894085	0.11147	0.1319686
2030.141	17	0.428543	0.3586206	0.1363942	0.08756988	0.116246	0.1307511
2030.241	17	0.444646	0.3566343	0.1379181	0.0871555	0.116772	0.1317429
2030.341	17	0.432135	0.3466542	0.1328441	0.08744469	0.115293	0.1320323
2030.441	17	0.418751	0.34611	0.130117	0.08705936	0.117509	0.1330605
2030.541	17	0.419893	0.3468433	0.1283546	0.08550266	0.115476	0.1349885
2030.641	17	0.433718	0.3511633	0.1247874	0.08105358	0.114228	0.1383618
2030.741	17	0.416338	0.3496836	0.1241839	0.08330529	0.113703	0.1368792
2030.841	17	0.429723	0.3521287	0.1273089	0.0858855	0.111699	0.136528
2030.941	17	0.425539	0.3472198	0.1234935	0.08674461	0.109173	0.1374712
2031.041	17	0.425891	0.3495964	0.1239943	0.08601999	0.112282	0.1355156
2031.141	17	0.407307	0.3568109	0.125855	0.08592555	0.112472	0.136739
2031.241	17	0.404096	0.3534553	0.12811	0.08473174	0.113495	0.1343182
2031.341	17	0.403076	0.3564656	0.1282184	0.08345588	0.113875	0.1317477
2031.441	17	0.395082	0.3590459	0.1253665	0.08214732	0.11433	0.1317629
2031.541	17	0.391852	0.3565808	0.1249114	0.08283305	0.113966	0.1334876

2031.641	17	0.387362	0.3498534	0.1224594	0.08239007	0.111741	0.1327252
2031.741	17	0.381348	0.3428239	0.1268833	0.08377575	0.110006	0.1331413
2031.841	17	0.381761	0.3505622	0.1259001	0.0829286	0.111084	0.1374492
2031.941	17	0.378042	0.3622171	0.1281444	0.08448142	0.111312	0.1343158
2032.041	17	0.376279	0.3697258	0.128686	0.08399063	0.111241	0.1353826
2032.141	17	0.383173	0.3500935	0.1268546	0.08318409	0.111811	0.1359119
2032.241	17	0.39016	0.3519681	0.1258399	0.08309185	0.108815	0.1348597
2032.341	17	0.382954	0.3570633	0.1263267	0.08357491	0.108065	0.1342114
2032.441	17	0.384594	0.3522032	0.1242543	0.08113141	0.108892	0.1308739
2032.541	17	0.38951	0.3499625	0.1239613	0.08190316	0.109835	0.1303516
2032.641	17	0.388928	0.3435245	0.1272584	0.08050614	0.1095	0.1346826
2032.741	17	0.395435	0.3430981	0.1254024	0.08129106	0.109993	0.1302846
2032.841	17	0.407153	0.3521405	0.124242	0.08049093	0.11138	0.1310135
2032.941	17	0.415029	0.3543495	0.1201227	0.08025204	0.109548	0.1315313
2033.041	17	0.426404	0.3529251	0.1154258	0.08462984	0.109809	0.130506
2033.141	17	0.429814	0.3549651	0.1171795	0.08746774	0.109043	0.1294435
2033.241	17	0.448439	0.3461513	0.1167632	0.09053653	0.112765	0.1296329
2033.341	17	0.447857	0.3494147	0.1160671	0.0947273	0.114797	0.1281143
2033.441	17	0.399557	0.3453295	0.1146371	0.09385893	0.118415	0.1285384
2033.541	17	0.399153	0.3458821	0.1161289	0.09047019	0.115152	0.1304891
2033.641	17	0.411292	0.3428353	0.1220189	0.0877234	0.116213	0.1300948
2033.741	17	0.425277	0.3434922	0.1263222	0.09015614	0.114644	0.1320465
2033.841	17	0.45106	0.3481744	0.1305008	0.08707073	0.116795	0.1319863
2033.941	17	0.436531	0.345723	0.1339397	0.08620957	0.121587	0.1337568
2034.041	17	0.43411	0.3428524	0.1349302	0.085558	0.119704	0.1340738
2034.141	17	0.436906	0.3403755	0.1372763	0.08380183	0.120811	0.1334308
2034.241	17	0.429421	0.3295189	0.1336171	0.08234753	0.122548	0.1314646
2034.341	17	0.434054	0.3396369	0.1299763	0.08489172	0.122531	0.1322814
2034.441	17	0.430272	0.3342565	0.1288169	0.08745877	0.120695	0.1357207
2034.541	17	0.434683	0.3406869	0.1268165	0.08770056	0.120259	0.1364076
2034.641	17	0.421535	0.3336757	0.1238446	0.08602636	0.117029	0.1366759
2034.741	17	0.412463	0.3320802	0.1252449	0.08707704	0.115643	0.1380873
2034.841	17	0.405225	0.3434482	0.1233007	0.08362767	0.113986	0.1380604
2034.941	17	0.397331	0.3501739	0.1237278	0.08458213	0.116236	0.1377519
2035.041	17	0.390389	0.3395542	0.1221767	0.08473143	0.117639	0.1349783
2035.141	17	0.39299	0.3438736	0.1230791	0.08834989	0.117716	0.1320474
2035.241	17	0.383275	0.3462767	0.1230008	0.08665884	0.116739	0.1335354
2035.341	17	0.379016	0.3623908	0.1244382	0.08681989	0.113701	0.1363253
2035.441	17	0.381954	0.345589	0.125305	0.08770897	0.109455	0.1372302
2035.541	17	0.381214	0.346169	0.1250718	0.08683199	0.108212	0.1361888
2035.641	17	0.38298	0.3428911	0.1253895	0.08664174	0.109076	0.1353702
2035.741	17	0.390946	0.3440998	0.126072	0.08619998	0.106907	0.1382139
2035.841	17	0.381759	0.3562684	0.1279159	0.08503917	0.105934	0.138028
2035.941	17	0.385453	0.3594589	0.1276352	0.08639458	0.103421	0.1381922
2036.041	17	0.391558	0.3484899	0.1236423	0.08568728	0.104829	0.1373937
2036.141	17	0.39468	0.3629562	0.1236933	0.08853495	0.104548	0.1386653
2036.241	17	0.396415	0.3565214	0.1233265	0.09169546	0.107325	0.137089
2036.341	17	0.408275	0.345924	0.1196392	0.09423389	0.107289	0.1347362
2036.441	17	0.417472	0.3575987	0.1179583	0.09514328	0.107809	0.1353171
2036.541	17	0.427776	0.3684191	0.1204048	0.0987445	0.108323	0.1376849
2036.641	17	0.439792	0.3774914	0.1211162	0.09978906	0.108062	0.1371651
2036.741	17	0.460841	0.3718977	0.1200787	0.1007497	0.108931	0.1348281
2036.841	17	0.461342	0.3659283	0.1206614	0.0996892	0.112684	0.1340838
2036.941	17	0.414673	0.365208	0.1191483	0.09724308	0.112506	0.1331256
2037.041	17	0.415725	0.3642937	0.1191512	0.09552682	0.109529	0.1342719
2037.141	17	0.439096	0.3517693	0.1193171	0.09461042	0.11435	0.1331612
2037.241	17	0.440038	0.3517248	0.1244349	0.09429137	0.114793	0.1331794
2037.341	17	0.460823	0.3639168	0.1254072	0.09277593	0.114855	0.1328461
2037.441	17	0.468747	0.3529521	0.1300528	0.09390434	0.120086	0.1314284
2037.541	17	0.451666	0.3493406	0.1319336	0.0927844	0.119869	0.1306896
2037.641	17	0.443377	0.3569226	0.1358133	0.0926431	0.119441	0.1316275
2037.741	17	0.457242	0.3574156	0.1356664	0.0893685	0.119041	0.1316633
2037.841	17	0.45632	0.3465988	0.1345714	0.08836833	0.116443	0.1326498
2037.941	17	0.435264	0.3575757	0.1351056	0.08973923	0.117257	0.1309445
2038.041	17	0.426081	0.3572702	0.1350396	0.0899618	0.11653	0.130121
2038.141	17	0.412871	0.3603838	0.1290438	0.08689314	0.11721	0.1302858
2038.241	17	0.406082	0.3568793	0.1280917	0.08630028	0.117551	0.1321435
2038.414	17	0.399125	0.3451052	0.130588	0.08706622	0.116232	0.137548
2038.514	17.5	0.403806	0.3433614	0.1283911	0.0852731	0.114564	0.1371884
2038.614	17.5	0.394324	0.3439846	0.1272713	0.08502541	0.118074	0.1386134
2038.714	17.5	0.379818	0.3467501	0.123501	0.08523031	0.118036	0.1388763
2038.814	17.5	0.380361	0.3468646	0.1222115	0.08502511	0.117842	0.1401427
2038.914	17.5	0.379431	0.3510732	0.1222333	0.08504518	0.116065	0.1420959
2039.014	17.5	0.382249	0.3461899	0.1255332	0.08312174	0.113961	0.1385846
2039.114	17.5	0.392932	0.3484899	0.1267339	0.08245476	0.115266	0.1360122
2039.214	17.5	0.386169	0.3508059	0.127727	0.08090336	0.113148	0.1350627
2039.314	17.5	0.38778	0.3530446	0.128791	0.08346869	0.110822	0.1346228
2039.414	17.5	0.392327	0.3551168	0.1270318	0.0808972	0.109337	0.1346525
2039.514	17.5	0.390529	0.3582425	0.127997	0.08072598	0.107493	0.1345726
2039.614	17.5	0.39453	0.3547543	0.1279347	0.0861558	0.105492	0.1378679
2039.714	17.5	0.409477	0.3499741	0.1256592	0.09262712	0.104168	0.1353945
2039.814	17.5	0.413658	0.3422443	0.1277346	0.09821016	0.105425	0.1338066
2039.914	17.5	0.429093	0.3493123	0.1242921	0.09930378	0.107764	0.1334377
2040.014	17.5	0.437024	0.3599498	0.1229336	0.1006174	0.109163	0.1329105
2040.114	17.5	0.456747	0.368892	0.1244977	0.09792706	0.109948	0.1309993
2040.214	17.5	0.440294	0.3497564	0.1238455	0.09939366	0.109081	0.1292171
2040.314	17.5	0.392245	0.3504561	0.1233331	0.0931187	0.108807	0.1304692
2040.414	17.5	0.402281	0.3568656	0.1195757	0.0918874	0.108052	0.1323624
2040.514	17.5	0.416529	0.3519168	0.1175788	0.09132227	0.111286	0.1306998
2040.614	17.5	0.431573	0.350408	0.1151834	0.08974523	0.112503	0.128921
2040.714	17.5	0.442698	0.3446421	0.1143467	0.09007255	0.115491	0.131549
2040.814	17.5	0.429223	0.3413454	0.1164094	0.09031349	0.118768	0.132265
2040.914	17.5	0.409926	0.3527706	0.1200411	0.09088679	0.120231	0.1339481
2041.014	17.5	0.410536	0.3552398	0.1203038	0.08657281	0.121627	0.1353954
2041.114	17.5	0.410668	0.3547781	0.1202358	0.08549484	0.123414	0.1355228

2041.214	17.5	0.41966	0.3539675	0.1217725	0.0865722	0.117841	0.1355499
2041.314	17.5	0.412602	0.3457859	0.1268623	0.08836698	0.116587	0.1322731
2041.414	17.5	0.410601	0.3507863	0.1298859	0.08852152	0.118254	0.13055
2041.514	17.5	0.412065	0.3470977	0.1329957	0.0885405	0.116514	0.1310922
2041.614	17.5	0.406322	0.3469308	0.1310579	0.08814198	0.117977	0.1341646
2041.714	17.5	0.397024	0.3433591	0.1246007	0.08541548	0.119933	0.1351154
2041.814	17.5	0.392637	0.3444102	0.1257374	0.08548569	0.118701	0.13462
2041.914	17.5	0.395977	0.3501903	0.1251069	0.08766513	0.119983	0.1333375
2042.014	17.5	0.397534	0.3468368	0.1230521	0.08784445	0.119263	0.1324505
2042.114	17.5	0.386634	0.3452007	0.12482	0.0894383	0.122322	0.1330344
2042.214	17.5	0.384959	0.3409513	0.1241619	0.09071948	0.122016	0.1355211
2042.314	17.5	0.383799	0.3285976	0.1252545	0.09217474	0.118957	0.1419254
2042.414	17.5	0.383708	0.3371344	0.1260875	0.09331165	0.117865	0.1437913
2042.514	17.5	0.395846	0.3352005	0.1283467	0.0931901	0.114622	0.1431258
2042.614	17.5	0.387369	0.3355071	0.1307029	0.09161174	0.109825	0.1434813
2042.714	17.5	0.395874	0.33391	0.130749	0.09101027	0.105744	0.1410547
2042.814	17.5	0.403253	0.3298228	0.1302281	0.08991665	0.105739	0.1408569
2042.914	17.5	0.396205	0.3418311	0.1285249	0.08785719	0.102969	0.1368101
2043.014	17.5	0.390908	0.3486596	0.1273349	0.0869952	0.104108	0.1361287
2043.114	17.5	0.407117	0.3395116	0.1263728	0.08644048	0.104375	0.1357529
2043.214	17.5	0.414531	0.3425423	0.1285558	0.0903995	0.104821	0.1333321
2043.314	17.5	0.420255	0.3434171	0.1287126	0.09010877	0.10467	0.1344025
2043.414	17.5	0.431586	0.3588813	0.1258037	0.09273475	0.104231	0.1356518
2043.514	17.5	0.451726	0.3456796	0.1252287	0.09389756	0.103986	0.137202
2043.614	17.5	0.452976	0.3438282	0.1250831	0.09042224	0.104512	0.1363909
2043.714	17.5	0.401709	0.3399886	0.1234225	0.0893908	0.107219	0.1343618
2043.814	17.5	0.418398	0.3406879	0.1220759	0.08673936	0.108402	0.1345319
2043.914	17.5	0.428726	0.3525263	0.1228367	0.08628311	0.114115	0.135709
2044.014	17.5	0.432904	0.3586204	0.1203572	0.08612551	0.115433	0.1365076
2044.114	17.5	0.453945	0.3468985	0.1194193	0.08838777	0.112942	0.1344572
2044.214	17.5	0.449791	0.3606114	0.1154071	0.08659333	0.112592	0.1330387
2044.314	17.5	0.438027	0.3549045	0.1142558	0.08714285	0.114459	0.1350916
2044.414	17.5	0.447677	0.3449601	0.1138093	0.08822889	0.117167	0.1337135
2044.514	17.5	0.439635	0.3553821	0.116386	0.08864505	0.117183	0.1344283
2044.614	17.5	0.449175	0.3654559	0.1207776	0.08987619	0.120783	0.1353131
2044.714	17.5	0.449314	0.3758464	0.1195773	0.08930965	0.118068	0.1354287
2044.814	17.5	0.437204	0.3710079	0.1208525	0.08928469	0.117127	0.1349732
2044.914	17.5	0.414206	0.3640652	0.1244045	0.08907822	0.114503	0.1359113
2045.014	17.5	0.417016	0.3634125	0.1312842	0.08896764	0.114909	0.1330144
2045.114	17.5	0.40215	0.362882	0.1355578	0.0891092	0.117711	0.1332308
2045.214	17.5	0.390144	0.3517122	0.1387189	0.08788169	0.114668	0.132769
2045.314	17.5	0.394466	0.351081	0.1371086	0.08670621	0.114497	0.1310252
2045.414	17.5	0.405527	0.3615329	0.1294578	0.08894065	0.116745	0.1320603
2045.514	17.5	0.390481	0.3524784	0.1294733	0.08721346	0.118425	0.131347
2045.614	17.5	0.382657	0.3475394	0.1254915	0.09024792	0.120893	0.1311005
2045.714	17.5	0.381988	0.3594657	0.1237171	0.09056988	0.121577	0.1298944
2045.814	17.5	0.380164	0.3555628	0.1199188	0.09186415	0.11871	0.1292195
2045.914	17.5	0.39248	0.3503009	0.1207246	0.09126966	0.118245	0.1322629
2046.014	17.5	0.391066	0.3589502	0.1207112	0.09048206	0.115983	0.1366087
2046.114	17.5	0.393874	0.3568207	0.1209067	0.0874542	0.111383	0.137265
2046.214	17.5	0.393239	0.3595923	0.1202892	0.08505739	0.10796	0.1406371
2046.314	17.5	0.390647	0.353351	0.1201861	0.0854503	0.108684	0.1396014
2046.414	17.5	0.390967	0.3425721	0.1245283	0.08676751	0.106023	0.1417476
2046.514	17.5	0.400988	0.3433723	0.1238344	0.08810864	0.105585	0.140269
2046.614	17.5	0.409121	0.3430679	0.1261835	0.09154916	0.105955	0.1396648
2046.714	17.5	0.430635	0.3467789	0.1269408	0.09355591	0.109576	0.1396033
2046.814	17.5	0.435956	0.3458276	0.1259824	0.09568704	0.108086	0.1359881
2046.914	17.5	0.443941	0.3477115	0.1300356	0.09479578	0.106474	0.1355426
2047.014	17.5	0.451935	0.3439857	0.1277954	0.09785209	0.108719	0.1367709
2047.114	17.5	0.42323	0.3449267	0.1277726	0.09641451	0.10911	0.1365266
2047.214	17.5	0.401849	0.3543342	0.1248683	0.09426226	0.108879	0.1382217
2047.314	17.5	0.425157	0.3492761	0.1256681	0.09447011	0.109712	0.1370472
2047.414	17.5	0.422982	0.3551412	0.1250049	0.09413704	0.110182	0.1393335
2047.514	17.5	0.441221	0.3571219	0.1263602	0.09248851	0.108896	0.1383968
2047.614	17.5	0.432791	0.3532872	0.1274485	0.09165116	0.10828	0.1363235
2047.714	17.5	0.421198	0.3479787	0.1239612	0.09209971	0.108009	0.133524
2047.814	17.5	0.411254	0.3397616	0.1225031	0.09221131	0.11256	0.1336922
2047.914	17.5	0.40614	0.3480026	0.1200346	0.09046488	0.11596	0.1326297
2048.014	17.5	0.418041	0.3588178	0.1152475	0.09040664	0.117478	0.1300768
2048.114	17.5	0.413994	0.367321	0.1158013	0.08989235	0.119244	0.1310856
2048.214	17.5	0.410978	0.3484474	0.1159613	0.08733733	0.118584	0.1352081
2048.314	17.5	0.411028	0.3497217	0.1164815	0.08781892	0.117072	0.1328121
2048.414	17.5	0.425268	0.352204	0.1156365	0.0860901	0.115698	0.1325522
2048.514	17.5	0.403179	0.3493885	0.1166788	0.08510862	0.117225	0.1305963
2048.614	17.5	0.394679	0.3485494	0.1208858	0.08637481	0.115795	0.1323719
2048.714	17.5	0.393211	0.3417569	0.1260835	0.08716835	0.120947	0.1328
2048.814	17.5	0.397543	0.3401097	0.1327078	0.08784501	0.118485	0.1363749
2048.914	17.5	0.393964	0.3514395	0.1349601	0.08817356	0.116679	0.1338797
2049.014	17.5	0.38662	0.3562732	0.1327271	0.09035798	0.117696	0.1340833
2049.114	17.5	0.384503	0.3503353	0.1327779	0.09297621	0.116858	0.1347142
2049.214	17.5	0.380678	0.3513131	0.1298702	0.09617794	0.119224	0.1357006
2049.314	17.5	0.388238	0.3433464	0.1291374	0.09346156	0.116334	0.1333961
2049.414	17.5	0.389627	0.3497004	0.1302663	0.09116573	0.118522	0.1333759
2049.514	17.5	0.382664	0.3458782	0.1264949	0.08664272	0.121375	0.1327699
2049.614	17.5	0.383423	0.3430715	0.1258072	0.08663882	0.120366	0.130061
2049.714	17.5	0.390675	0.3429361	0.1225005	0.08427706	0.118111	0.1308387
2049.814	17.5	0.387689	0.3444081	0.1239856	0.08440846	0.112357	0.1299112
2049.914	17.5	0.394175	0.3507975	0.1215367	0.08509624	0.110239	0.1322105
2050.014	17.5	0.410853	0.3444496	0.124194	0.08749957	0.108403	0.1367635
2050.114	17.5	0.417949	0.3449528	0.1235731	0.09326356	0.108186	0.1382437
2050.214	17.5	0.419672	0.3379107	0.1225489	0.0968584	0.110363	0.1391331
2050.314	17.5	0.440811	0.3274506	0.1241342	0.09869455	0.111048	0.1393401
2050.414	17.5	0.456797	0.337704	0.1240887	0.09934875	0.110571	0.1412757
2050.514	17.5	0.42085	0.3294435	0.1245676	0.09791961	0.110129	0.145074
2050.614	17.5	0.38741	0.339016	0.1236442	0.09495074	0.111012	0.1420308

2050.714	17.5	0.409941	0.3320277	0.1256997	0.09506393	0.108389	0.138747
2050.814	17.5	0.413534	0.3273248	0.1262526	0.09463357	0.1074	0.1358369
2050.914	17.5	0.430082	0.3393351	0.1263031	0.0944676	0.109718	0.134676
2051.014	17.5	0.429307	0.3467043	0.1250635	0.09036747	0.110513	0.1360595
2051.114	17.5	0.418221	0.3370772	0.1237117	0.08919782	0.110828	0.1348512
2051.214	17.5	0.405028	0.3417831	0.1231797	0.08761659	0.112423	0.1333113
2051.314	17.5	0.411925	0.3406914	0.1220867	0.08770566	0.112758	0.1346852
2051.414	17.5	0.420642	0.3582545	0.1204736	0.08954443	0.11335	0.1331074
2051.514	17.5	0.427586	0.3429381	0.1201712	0.08995084	0.116544	0.1352358
2051.614	17.5	0.431542	0.3415402	0.1199212	0.08685702	0.119003	0.1352026
2051.714	17.5	0.42954	0.3382001	0.1171259	0.08386278	0.117517	0.1366841
2051.814	17.5	0.426645	0.3386344	0.1171413	0.08404746	0.117711	0.1351865
2051.914	17.5	0.412266	0.3504463	0.118378	0.08387133	0.117286	0.1339875
2052.014	17.5	0.400272	0.3567914	0.1187656	0.08427185	0.117692	0.1352379
2052.114	17.5	0.394999	0.345019	0.119891	0.08715082	0.118123	0.136218
2052.214	17.5	0.397836	0.3582428	0.1157146	0.08764559	0.11968	0.1337964
2052.314	17.5	0.394434	0.3538441	0.1193078	0.08890585	0.117324	0.1317557
2052.414	17.5	0.383351	0.3443866	0.1202008	0.08908316	0.116364	0.1304947
2052.514	17.5	0.382616	0.3523291	0.1253351	0.08925147	0.115886	0.1316567
2052.614	17.5	0.377845	0.3631453	0.1301558	0.08966146	0.115706	0.132173
2052.714	17.5	0.379776	0.373422	0.1324125	0.08774976	0.118155	0.1314172
2052.814	17.5	0.390863	0.3708562	0.1327704	0.08694699	0.120836	0.1325023
2052.914	17.5	0.380675	0.3638359	0.1276163	0.0855011	0.118974	0.1324754
2053.014	17.5	0.383141	0.3616952	0.1256144	0.08513315	0.116723	0.1324111
2053.114	17.5	0.386033	0.3611295	0.1278693	0.08474046	0.11503	0.1334061
2053.214	17.5	0.385739	0.3507119	0.1261682	0.08465364	0.112094	0.1340272
2053.314	17.5	0.392037	0.3488061	0.1238584	0.08710032	0.110741	0.1340498
2053.414	17.5	0.399547	0.3602746	0.1228573	0.08946021	0.108846	0.1334554
2053.514	17.5	0.413941	0.3511694	0.1214177	0.09098043	0.106192	0.1341374
2053.614	17.5	0.418886	0.3461221	0.1219688	0.0962799	0.106344	0.1323935
2053.714	17.5	0.430309	0.3568497	0.125256	0.09650759	0.106303	0.1344433
2053.814	17.5	0.436881	0.3532907	0.1279922	0.09891728	0.106235	0.1343558
2053.914	17.5	0.433812	0.3478339	0.127716	0.09707117	0.10861	0.1353203
2054.014	17.5	0.398687	0.357634	0.1261396	0.09462091	0.109585	0.1341034
2054.114	17.5	0.404735	0.3566834	0.127326	0.09225643	0.11015	0.1326121
2054.214	17.5	0.421967	0.3576972	0.1272044	0.09293364	0.108814	0.1342671
2054.314	17.5	0.429994	0.3523585	0.12612	0.09242184	0.108005	0.1333849
2054.414	17.5	0.440721	0.3423567	0.1289358	0.09030043	0.110045	0.1362569
2054.514	17.5	0.454365	0.3440966	0.1294051	0.08824103	0.11313	0.1326445
2054.614	17.5	0.43969	0.3435351	0.1293359	0.08858168	0.116937	0.1331852
2054.714	17.5	0.4551	0.3482678	0.1281025	0.08847114	0.118651	0.133627
2054.814	17.5	0.449746	0.3470806	0.128752	0.08850085	0.119103	0.1346645
2054.914	17.5	0.461817	0.3470303	0.1265521	0.08565414	0.117175	0.135854
2055.014	17.5	0.448725	0.3439996	0.1248481	0.08820505	0.116105	0.1373606
2055.114	17.5	0.452117	0.3450858	0.1234432	0.08691261	0.121066	0.1389934
2055.214	17.5	0.450006	0.358567	0.1190272	0.08349755	0.120511	0.1369689
2055.314	17.5	0.43979	0.3490363	0.1202582	0.08024042	0.118552	0.1340609
2055.414	17.5	0.411593	0.35478	0.1187515	0.07999685	0.116993	0.1336368
2055.514	17.5	0.404403	0.3571717	0.1163592	0.08116464	0.116875	0.1322968
2055.614	17.5	0.409405	0.3560105	0.1175394	0.08356328	0.11465	0.1324672
2055.714	17.5	0.406285	0.3461252	0.115977	0.08708806	0.112544	0.1320657
2055.814	17.5	0.38719	0.3397886	0.1176163	0.08711115	0.118429	0.1341962
2055.914	17.5	0.382871	0.3515831	0.1206816	0.08809187	0.119212	0.1343584
2056.014	17.5	0.379333	0.3632443	0.123856	0.08903096	0.116308	0.1307635
2056.114	17.5	0.378812	0.3665413	0.1248369	0.09073323	0.117067	0.1290998
2056.214	17.5	0.389973	0.3481906	0.1257704	0.09165154	0.118047	0.1274261
2056.314	17.5	0.381527	0.3523032	0.1298499	0.09002462	0.11772	0.1290735
2056.414	17.5	0.379466	0.350872	0.1309988	0.09121765	0.115719	0.1304315
2056.514	17.5	0.383977	0.3479107	0.1346121	0.09116708	0.113257	0.1311004
2056.614	17.5	0.382177	0.3483832	0.1326561	0.091962	0.112891	0.1315984
2056.714	17.5	0.388444	0.3410801	0.1256976	0.0904137	0.115454	0.1320025
2056.814	17.5	0.397809	0.342319	0.1279419	0.09190367	0.112602	0.1299844
2056.914	17.5	0.409226	0.3517903	0.126817	0.09590196	0.111072	0.1302864
2057.014	17.5	0.421923	0.3556136	0.1246677	0.09883941	0.10962	0.1328871
2057.114	17.5	0.42157	0.3478126	0.1238073	0.09967443	0.109753	0.1316325
2057.214	17.5	0.436113	0.3517929	0.1218641	0.09772477	0.107284	0.1328672
2057.314	17.5	0.446932	0.3426495	0.1239908	0.09832765	0.109141	0.1327578
2057.414	17.5	0.406074	0.3481669	0.1227384	0.09446262	0.109829	0.1320319
2057.514	17.5	0.401581	0.3455976	0.1254322	0.09161248	0.109812	0.1321455
2057.614	17.5	0.416817	0.3434118	0.130357	0.09032021	0.107988	0.1338705
2057.714	17.5	0.425705	0.3428454	0.1283637	0.0906635	0.107298	0.1363871
2057.814	17.5	0.432186	0.3440449	0.127024	0.09167633	0.107649	0.1378508
2057.914	17.5	0.42514	0.3508011	0.1293789	0.08893622	0.106429	0.1389122
2058.014	17.5	0.415162	0.342536	0.1300331	0.08831784	0.106315	0.1411294
2058.114	17.5	0.422738	0.3450162	0.129106	0.08949332	0.109047	0.1412439
2058.214	17.5	0.426784	0.3377728	0.1322699	0.0910692	0.111084	0.139125
2058.314	17.5	0.436313	0.3278283	0.1309946	0.08963419	0.113681	0.1380593
2058.414	17.5	0.422018	0.3374159	0.1324837	0.08700453	0.116814	0.1350017
2058.514	17.5	0.421908	0.3281635	0.1323123	0.08700625	0.119412	0.1347081
2058.614	17.5	0.413667	0.3363168	0.1289049	0.08642518	0.124035	0.1315486
2058.714	17.5	0.421054	0.3314902	0.1263501	0.08539525	0.125046	0.1291106
2058.814	17.5	0.413617	0.3282907	0.1256422	0.08456237	0.118868	0.1327039
2058.914	17.5	0.404011	0.3396722	0.1257603	0.08488768	0.119876	0.1345838
2059.014	17.5	0.398825	0.3482366	0.1223259	0.08862459	0.120103	0.1361183
2059.114	17.5	0.399938	0.3365949	0.121295	0.0912179	0.118923	0.1335876
2059.214	17.5	0.392789	0.3414581	0.1192914	0.09287569	0.117266	0.1325999
2059.314	17.5	0.385133	0.3403562	0.1166318	0.09472448	0.11773	0.132449
2059.414	17.5	0.38434	0.359086	0.1194435	0.09435882	0.115584	0.1318661
2059.514	17.5	0.379048	0.3420342	0.1213609	0.09378349	0.114992	0.1344074
2059.614	17.5	0.384862	0.3410702	0.124566	0.09540547	0.114579	0.1325456
2059.714	17.5	0.390029	0.3377344	0.1212313	0.09383255	0.116046	0.1312641
2059.814	17.5	0.386445	0.3409151	0.1238737	0.0929776	0.119193	0.1296095
2059.914	17.5	0.384024	0.3532331	0.1272908	0.09195307	0.117678	0.1312059
2060.014	17.5	0.385465	0.3559106	0.1349522	0.08890465	0.11707	0.1312283
2060.114	17.5	0.397133	0.3439889	0.1349399	0.09021526	0.115998	0.1310392

2060.214	17.5	0.402027	0.3569967	0.1355883	0.09044682	0.114564	0.1314187
2060.314	17.5	0.419139	0.3527286	0.1368202	0.09209643	0.114517	0.1318593
2060.414	17.5	0.417373	0.344451	0.1308113	0.09540683	0.110131	0.1305556
2060.514	17.5	0.418177	0.3510534	0.1292079	0.09835953	0.108434	0.1292752
2060.614	17.5	0.431895	0.3658786	0.128894	0.09988725	0.110055	0.1303381
2060.714	17.5	0.446136	0.3756187	0.1247037	0.09788045	0.108911	0.1321668
2060.814	17.5	0.414712	0.3700711	0.1242477	0.09455397	0.110327	0.1302968
2060.914	17.5	0.392156	0.3624642	0.1229734	0.09160701	0.108935	0.1315647
2061.014	17.5	0.430135	0.3612364	0.121759	0.09399858	0.108852	0.1340655
2061.114	17.5	0.42158	0.3597508	0.1212606	0.09445453	0.108708	0.1340329
2061.214	17.5	0.441642	0.3495089	0.1210148	0.09570047	0.10909	0.1357402
2061.314	17.5	0.44364	0.3471115	0.1244646	0.09468816	0.11028	0.1380494
2061.414	17.5	0.444014	0.3578898	0.1265726	0.09052557	0.112799	0.1389306
2061.514	17.5	0.431514	0.3533081	0.1293128	0.08992235	0.113589	0.1390548
2061.614	17.5	0.429481	0.3449244	0.1274025	0.09016837	0.112502	0.1373897
2061.714	17.5	0.417977	0.3563252	0.1255834	0.08955723	0.110583	0.13681
2061.814	17.5	0.418044	0.3551479	0.1260356	0.08833277	0.110075	0.1361948
2061.914	17.5	0.43077	0.3484544	0.1348317	0.08772371	0.115687	0.1322805
2062.014	17.5	0.421793	0.3560672	0.1348207	0.08572661	0.118447	0.1322991
2062.114	17.5	0.421595	0.3570804	0.1338781	0.08423456	0.12056	0.1305714
2062.214	17.5	0.410191	0.3598688	0.1324521	0.08496908	0.122148	0.1318585
2062.314	17.5	0.408525	0.3543592	0.1318177	0.08564592	0.119799	0.133816
2062.414	17.5	0.402374	0.344933	0.1286138	0.08648266	0.121602	0.1332867
2062.514	17.5	0.400673	0.3428546	0.1285697	0.0879757	0.1213	0.1358107
2062.614	17.5	0.395274	0.3434708	0.1271298	0.08674944	0.117831	0.1347033
2062.714	17.5	0.387123	0.3473592	0.1236679	0.08784172	0.119727	0.1335173
2062.814	17.5	0.383398	0.3477232	0.1249111	0.08750054	0.119409	0.1321528
2062.914	17.5	0.379826	0.3493561	0.1225681	0.08728261	0.117863	0.1332536
2063.014	17.5	0.382087	0.3449249	0.1207517	0.08822063	0.117296	0.1337443
2063.114	17.5	0.387946	0.3458073	0.1224418	0.08949436	0.116346	0.1342759
2063.214	17.5	0.383753	0.3509118	0.1223648	0.09009875	0.116706	0.1344739
2063.314	17.5	0.383099	0.3527011	0.1235282	0.09401165	0.117213	0.134813
2063.414	17.5	0.386626	0.3557603	0.1238778	0.09203868	0.115895	0.1334824
2063.514	17.5	0.384623	0.3581746	0.1255633	0.0900237	0.114492	0.1332374
2063.614	17.5	0.392242	0.3556629	0.1304151	0.09270519	0.112167	0.1306907
2063.714	17.5	0.409582	0.350498	0.1318709	0.09499829	0.113968	0.1291797
2063.814	17.5	0.420114	0.3390792	0.1341849	0.09579047	0.112178	0.1314342
2063.914	17.5	0.419102	0.347627	0.1331212	0.09433702	0.108734	0.1325159
2064.014	17.5	0.419775	0.3604178	0.1340125	0.09515063	0.106718	0.1311443
2064.114	17.5	0.43747	0.3680077	0.1324405	0.09729277	0.105146	0.1328447
2064.214	17.5	0.434564	0.348519	0.1267446	0.09696389	0.105589	0.131633
2064.314	17.5	0.395238	0.3482731	0.1259144	0.09202584	0.105549	0.1318278
2064.414	17.5	0.412544	0.3527167	0.1259522	0.08998185	0.103489	0.1316912
2064.514	17.5	0.41878	0.3474498	0.1228442	0.09085974	0.104731	0.1335195
2064.614	17.5	0.437267	0.3473281	0.1247028	0.09218805	0.106422	0.1344542
2064.714	17.5	0.434252	0.3416104	0.1228615	0.092338	0.105395	0.1331931
2064.814	17.5	0.425698	0.3409116	0.1234778	0.09102973	0.10652	0.1342542
2064.914	17.5	0.407631	0.3510533	0.1232898	0.08937021	0.106189	0.1349546
2065.014	17.5	0.429605	0.3525175	0.1237984	0.08875821	0.105063	0.1353025
2065.114	17.5	0.431467	0.3483141	0.1262099	0.08867394	0.107573	0.1394636
2065.214	17.5	0.436537	0.3516219	0.1246499	0.08874709	0.117416	0.1427184
2065.314	17.5	0.442311	0.3441243	0.1254268	0.087755	0.121389	0.141603
2065.414	17.5	0.447332	0.3499477	0.1275464	0.08438797	0.12124	0.1408881
2065.514	17.5	0.433754	0.3450823	0.1294618	0.08334279	0.119418	0.1400366
2065.614	17.5	0.435778	0.3441256	0.1307529	0.08152623	0.11894	0.1387634
2065.714	17.5	0.410564	0.3438431	0.1326076	0.08340818	0.123018	0.1367623
2065.814	17.5	0.407176	0.3432999	0.1295311	0.08571537	0.122483	0.1344783
2065.914	17.5	0.408256	0.3496174	0.1291956	0.09010938	0.118805	0.1343798
2066.014	17.5	0.399325	0.3434048	0.1287171	0.08895617	0.119415	0.1341436
2066.114	17.5	0.389939	0.3448056	0.1299613	0.08755513	0.117024	0.1356489
2066.214	17.5	0.389948	0.3394743	0.1278565	0.09014037	0.117613	0.1363032
2066.314	17.5	0.380904	0.3303361	0.1265865	0.0916414	0.118877	0.1347323
2066.414	17.5	0.381147	0.3367476	0.1264145	0.09194031	0.120012	0.1347215
2066.514	17.5	0.390695	0.329998	0.1251811	0.09174185	0.116118	0.1319858
2066.614	17.5	0.380215	0.3314122	0.1245755	0.09264206	0.115158	0.1301008
2066.714	17.5	0.385729	0.3307506	0.1211161	0.09400334	0.114489	0.1316975
2066.814	17.5	0.384348	0.3265742	0.1199533	0.09286873	0.117504	0.1318649
2066.914	17.5	0.386522	0.3374915	0.1206309	0.09060315	0.116984	0.1306762
2067.014	17.5	0.394149	0.3453554	0.1221885	0.09307902	0.116602	0.1319173
2067.114	17.5	0.402819	0.3364253	0.1231435	0.09487011	0.115601	0.1263892
2067.214	17.5	0.41719	0.3401061	0.1207426	0.0986059	0.113574	0.1281141
2067.314	17.5	0.416993	0.3415622	0.1236623	0.09958597	0.114191	0.1256105
2067.414	17.5	0.423902	0.3569595	0.1280334	0.1002165	0.111283	0.1261628
2067.514	17.5	0.443828	0.3426857	0.1338952	0.1016336	0.109551	0.1276255
2067.614	17.5	0.445192	0.339243	0.1348845	0.09975452	0.106389	0.1283228
2067.714	17.5	0.392768	0.3357121	0.1344708	0.09565046	0.107069	0.128887
2067.814	17.5	0.394629	0.3390613	0.1353592	0.09183749	0.106545	0.1294921
2067.914	17.5	0.419534	0.3486579	0.127198	0.09141701	0.106012	0.126785
2068.014	17.5	0.422632	0.3543816	0.1252392	0.09101145	0.108078	0.1278874
2068.114	17.5	0.44515	0.3412312	0.1257946	0.09026456	0.108427	0.1313293
2068.214	17.5	0.445274	0.3423337	0.1242902	0.08836462	0.109202	0.1322899
2068.314	17.5	0.432199	0.349513	0.1245103	0.08957192	0.106599	0.1310731
2068.414	17.5	0.447219	0.3646455	0.1238375	0.09210037	0.108384	0.130713
2068.514	17.5	0.44317	0.3740737	0.1232836	0.09061886	0.107803	0.1267243
2068.614	17.5	0.455051	0.3718589	0.1222983	0.09128923	0.108026	0.1278306
2068.714	17.5	0.4552	0.359661	0.1217481	0.08942835	0.112112	0.1301834
2068.814	17.5	0.443381	0.3590222	0.1243692	0.08523702	0.115554	0.1327468
2068.914	17.5	0.432721	0.3537716	0.125478	0.0803244	0.116078	0.1348526
2069.014	17.5	0.430043	0.3451963	0.1243677	0.0817882	0.118396	0.1341968
2069.114	17.5	0.420752	0.3554959	0.1215298	0.08230414	0.12003	0.1332231
2069.214	17.5	0.404198	0.3483988	0.1201459	0.08439212	0.123466	0.1335796
2069.314	17.5	0.411137	0.3487655	0.1218444	0.08652695	0.125264	0.1346312
2069.414	17.5	0.404122	0.3520064	0.1260897	0.08960728	0.121102	0.1312756
2069.514	17.5	0.399577	0.3547141	0.125315	0.0872971	0.120432	0.1318654
2069.614	17.5	0.391019	0.3468154	0.124448	0.08731663	0.121492	0.1300352

2069.714	17.5	0.385952	0.3539636	0.1224269	0.08801903	0.123438	0.1303461
2069.814	17.5	0.384931	0.3550021	0.1234124	0.08740512	0.120398	0.1330631
2069.914	17.5	0.395837	0.3576528	0.1216062	0.08585759	0.119247	0.1310246
2070.014	17.5	0.388772	0.3518413	0.1203557	0.08476729	0.119144	0.1322764
2070.114	17.5	0.396019	0.3416368	0.1200943	0.08232687	0.122454	0.1312624
2070.214	17.5	0.390555	0.3414606	0.1193947	0.08552974	0.119453	0.132144
2070.314	17.5	0.382213	0.3439145	0.1210986	0.08609857	0.117435	0.1312606
2070.414	17.5	0.406638	0.348367	0.1180559	0.08560398	0.114269	0.1323858
2070.514	17.5	0.416957	0.3467559	0.116159	0.08515159	0.114671	0.1331743
2070.614	17.5	0.421092	0.3426943	0.1166516	0.08613067	0.114959	0.1309936
2070.714	17.5	0.429841	0.3476503	0.11627	0.08754481	0.111791	0.132714
2070.814	17.5	0.43186	0.3442474	0.116658	0.0912766	0.1094	0.1317892
2070.914	17.5	0.443038	0.3531597	0.1176968	0.09377079	0.107906	0.1303565
2071.014	17.5	0.450787	0.3535311	0.1200176	0.09608842	0.108018	0.1288516
2071.114	17.5	0.406669	0.3576764	0.1227872	0.09484257	0.106336	0.1312996
2071.214	17.5	0.387612	0.3540391	0.1275379	0.08941552	0.107013	0.1298373
2071.314	17.5	0.422585	0.3464688	0.1272036	0.08863196	0.111218	0.1323564
2071.414	17.5	0.415974	0.3372755	0.1250608	0.08750261	0.109659	0.1297601
2071.514	17.5	0.432078	0.3472483	0.1267321	0.08477449	0.10837	0.1320733
2071.614	17.5	0.432965	0.3632976	0.1281546	0.08454919	0.107645	0.1340598
2071.714	17.5	0.409682	0.3599655	0.1241009	0.08430416	0.109415	0.1326958
2071.814	17.5	0.402811	0.3426557	0.1245415	0.08391901	0.10986	0.131046
2071.914	17.5	0.413937	0.3543916	0.1219645	0.08381746	0.108288	0.1333773
2072.014	17.5	0.417151	0.346133	0.1194186	0.08276497	0.112203	0.1343245
2072.114	17.5	0.413896	0.3470086	0.1206165	0.08320943	0.112597	0.1343748
2072.214	17.5	0.42063	0.3395874	0.1194521	0.08136435	0.113564	0.1330256
2072.314	17.5	0.414467	0.3385959	0.1207685	0.08179715	0.11306	0.1364228
2072.414	17.5	0.413922	0.344399	0.1200762	0.08167221	0.116302	0.1393369
2072.514	17.5	0.402562	0.3487639	0.1197091	0.08081406	0.118662	0.1409787
2072.614	17.5	0.400116	0.3497156	0.1214556	0.0818082	0.117785	0.1416278
2072.714	17.5	0.402654	0.3510239	0.1202211	0.08183129	0.115028	0.1417284
2072.814	17.5	0.398116	0.3413137	0.1210952	0.08201825	0.115226	0.1398349
2072.914	17.5	0.396535	0.3478333	0.1221296	0.08206423	0.114548	0.14185
2073.014	17.5	0.391661	0.3448674	0.1233826	0.08438559	0.115637	0.141226
2073.114	17.5	0.38964	0.3398236	0.1249498	0.08710739	0.119778	0.136509
2073.214	17.5	0.385249	0.3414569	0.1256947	0.08883286	0.117012	0.1338191
2073.314	17.5	0.391312	0.3414861	0.1238422	0.08691461	0.117265	0.1335934
2073.414	17.5	0.394774	0.3467759	0.1227341	0.08543406	0.117735	0.1332949
2073.514	17.5	0.390692	0.3441886	0.1237272	0.08625732	0.118372	0.1349351
2073.614	17.5	0.389906	0.3427423	0.1250664	0.08921161	0.118746	0.1347052
2073.714	17.5	0.393455	0.3267305	0.1228124	0.08988049	0.117426	0.1352437
2073.814	17.5	0.404083	0.3306406	0.121729	0.08947997	0.113969	0.1354368
2073.914	17.5	0.410794	0.3359353	0.1203753	0.08998384	0.115018	0.1354112
2074.014	17.5	0.41817	0.3298555	0.1186316	0.0898997	0.112495	0.1345111
2074.114	17.5	0.426801	0.3294545	0.1177829	0.09303771	0.110124	0.1335537
2074.214	17.5	0.432464	0.3275952	0.1164315	0.09135201	0.10978	0.1341532
2074.314	17.5	0.432655	0.336813	0.1151229	0.09289176	0.107291	0.131633
2074.414	17.5	0.443209	0.3452332	0.1141254	0.09443366	0.105007	0.1305138
2074.514	17.5	0.42321	0.338245	0.1158509	0.09293469	0.10338	0.1338134
2074.614	17.5	0.386577	0.344245	0.117219	0.089968	0.104973	0.1317765
2074.714	17.5	0.410941	0.3451288	0.1212484	0.08864813	0.106974	0.1318718
2074.814	17.5	0.416419	0.3526024	0.125876	0.09051725	0.107213	0.1310351
2074.914	17.5	0.437824	0.3441192	0.1262857	0.08874826	0.106186	0.1352238
2075.014	17.5	0.435653	0.3377934	0.1308357	0.08803199	0.106552	0.1343184
2075.114	17.5	0.442441	0.3405239	0.1303584	0.08600056	0.106116	0.1349151
2075.214	17.5	0.424466	0.3462704	0.1299311	0.08841907	0.106868	0.1322139
2075.314	17.5	0.460078	0.3561801	0.1319456	0.08863325	0.10632	0.1303787
2075.414	17.5	0.451116	0.344105	0.1280127	0.08717325	0.10831	0.1304383
2075.514	17.5	0.459484	0.3560815	0.1247177	0.08495749	0.110853	0.1305167
2075.614	17.5	0.451296	0.3494025	0.1266456	0.0854884	0.114874	0.1304549
2075.714	17.5	0.451796	0.3441626	0.1218587	0.08508074	0.117133	0.1334135
2075.814	17.5	0.445788	0.3497943	0.1199392	0.08630941	0.116888	0.1340582
2075.914	17.5	0.437833	0.3662946	0.1203924	0.08512887	0.119181	0.1336915
2076.014	17.5	0.41324	0.3728821	0.1198484	0.08604534	0.118419	0.1334045
2076.114	17.5	0.412928	0.3723707	0.1215791	0.08466218	0.118283	0.1360612
2076.214	17.5	0.41332	0.3588858	0.1218985	0.08670286	0.117394	0.1367737
2076.314	17.5	0.405047	0.3592816	0.1234713	0.08680122	0.115818	0.1388329
2076.414	17.5	0.404298	0.3521732	0.1250353	0.08893599	0.117547	0.1397633
2076.514	17.5	0.397748	0.3457094	0.1242095	0.08931872	0.119334	0.1382324
2076.614	17.5	0.385153	0.3551132	0.1261761	0.09053608	0.119836	0.1410991
2076.714	17.5	0.394278	0.3488567	0.1240626	0.08990873	0.116535	0.1397578
2076.814	17.5	0.402002	0.3485655	0.1227188	0.08619514	0.116457	0.1392153
2076.914	17.5	0.390967	0.3514805	0.12459	0.08832587	0.116039	0.1376006
2077.014	17.5	0.402582	0.35495	0.1230813	0.0920829	0.117912	0.1356911
2077.114	17.5	0.397472	0.3449911	0.1209452	0.09478121	0.117723	0.1321394
2077.214	17.5	0.404617	0.3542162	0.1204635	0.09341501	0.115136	0.1309843
2077.314	17.5	0.419833	0.3533587	0.1227602	0.09377947	0.114155	0.133783
2077.414	17.5	0.424918	0.3578311	0.1199878	0.09295368	0.114387	0.1340339
2077.514	17.5	0.435847	0.3505395	0.118242	0.09292412	0.111715	0.1345684
2077.614	17.5	0.434015	0.3409507	0.117231	0.09233839	0.10804	0.1317574
2077.714	17.5	0.427694	0.3415774	0.1168511	0.09181411	0.105842	0.1335601
2077.814	17.5	0.450408	0.3438149	0.1192223	0.09316215	0.105343	0.1357771
2077.914	17.5	0.444	0.3480244	0.1183486	0.09207631	0.106022	0.1336936
2078.014	17.5	0.398755	0.3479569	0.1161615	0.09009071	0.107773	0.1357348
2078.114	17.5	0.412044	0.3442575	0.1156384	0.08884466	0.106011	0.1340914
2078.214	17.5	0.43668	0.3467302	0.1176229	0.08994889	0.107333	0.1345996
2078.314	17.5	0.434465	0.3475863	0.1187087	0.08907855	0.106948	0.13592
2078.414	17.5	0.443958	0.3512695	0.1171234	0.0884878	0.107476	0.1340615
2078.514	17.5	0.44339	0.3552002	0.1158937	0.08654521	0.111001	0.1322265
2078.614	17.5	0.420842	0.3553627	0.1208706	0.08614325	0.10908	0.1321945
2078.714	17.5	0.422597	0.3534817	0.1209609	0.08540409	0.110101	0.1324226
2078.814	17.5	0.427849	0.3423549	0.1221949	0.08679099	0.11212	0.1331869
2078.914	17.5	0.442401	0.3403876	0.1237604	0.08790814	0.113908	0.1327926
2079.014	17.5	0.432868	0.3500419	0.1275875	0.08901501	0.115617	0.132868
2079.114	17.5	0.427474	0.3657303	0.1284486	0.08855378	0.115961	0.1312691

2079.214	17.5	0.432047	0.3558257	0.1244792	0.08512107	0.118005	0.1290213
2079.314	17.5	0.429717	0.3451286	0.1258615	0.08518744	0.121542	0.1279068
2079.414	17.5	0.416662	0.3544143	0.1265285	0.08542611	0.122213	0.1287439
2079.514	17.5	0.399205	0.3447911	0.1228608	0.08539463	0.121054	0.1293627
2079.614	17.5	0.40326	0.3463773	0.1225165	0.0884434	0.120264	0.1309062
2079.714	17.5	0.404503	0.339581	0.1190318	0.08853839	0.118975	0.1305156
2079.814	17.5	0.393793	0.3393863	0.1175648	0.09178567	0.117806	0.1300132
2079.914	17.5	0.399271	0.3491547	0.1165913	0.09223442	0.119931	0.1334805
2080.014	17.5	0.388689	0.3525215	0.1186249	0.09296028	0.119544	0.1350884
2080.114	17.5	0.399899	0.3487448	0.1197067	0.09392856	0.116494	0.1383712
2080.214	17.5	0.409866	0.3514231	0.1214878	0.092009	0.116097	0.1392582
2080.314	17.5	0.392935	0.3428154	0.1247021	0.09231039	0.117137	0.1401871
2080.414	17.5	0.407777	0.3472572	0.1238647	0.09298217	0.119432	0.1415281
2080.514	17.5	0.39598	0.3482689	0.1234737	0.09334761	0.120071	0.142493
2080.614	17.5	0.398607	0.3386598	0.1242304	0.09091279	0.116167	0.1371094
2080.714	17.5	0.420781	0.3403186	0.1283702	0.09282786	0.115385	0.1342173
2080.814	17.5	0.413973	0.3428214	0.1268794	0.09416037	0.113824	0.1305677
2080.914	17.5	0.416805	0.3484047	0.1274492	0.09365285	0.113925	0.1309937
2081.014	17.5	0.428664	0.3458203	0.1283692	0.09508123	0.110411	0.1329924
2081.114	17.5	0.439388	0.3440512	0.1268349	0.09798526	0.109207	0.1330415
2081.214	17.5	0.44009	0.3290358	0.1242313	0.09822064	0.105742	0.1365021
2081.314	17.5	0.445741	0.3328622	0.1220699	0.09723443	0.104988	0.1355544
2081.414	17.5	0.406591	0.333747	0.1179599	0.09310201	0.105053	0.1316313
2081.514	17.5	0.390955	0.328413	0.1192065	0.09057965	0.106407	0.1313734
2081.614	17.5	0.415294	0.3332871	0.1204242	0.09023743	0.107008	0.1312947
2081.714	17.5	0.417668	0.3270167	0.118878	0.08859018	0.105961	0.1349623
2081.814	17.5	0.441503	0.337847	0.1197584	0.08848505	0.106443	0.1338125
2081.914	17.5	0.444999	0.3451222	0.1200265	0.08890221	0.105839	0.1334287
2082.014	17.5	0.423005	0.3378039	0.120154	0.09034718	0.109069	0.1340891
2082.114	17.5	0.421376	0.3470497	0.1188192	0.08994678	0.109437	0.1337953
2082.214	17.5	0.433717	0.3433195	0.1186096	0.08955034	0.110087	0.1313639
2082.314	17.5	0.434222	0.353847	0.1207778	0.08676155	0.110164	0.132935
2082.414	17.5	0.433223	0.3448586	0.1218229	0.08746795	0.113509	0.1318184
2082.514	17.5	0.463311	0.3390539	0.1274636	0.08797999	0.116747	0.1301551
2082.614	17.5	0.445527	0.3405875	0.1312468	0.08466119	0.117114	0.1309845
2082.714	17.5	0.435031	0.3459641	0.1346809	0.08238783	0.117601	0.1296882
2082.814	17.5	0.411506	0.3565894	0.1344361	0.08208582	0.123326	0.1309925
2082.914	17.5	0.402337	0.3474087	0.1276406	0.08296121	0.121675	0.1326351
2083.014	17.5	0.398252	0.3566028	0.124433	0.08539835	0.116521	0.1314472
2083.114	17.5	0.399381	0.3513679	0.1250052	0.08586058	0.114322	0.1313686
2083.214	17.5	0.398096	0.3452281	0.1238868	0.08640475	0.116427	0.1337833
2083.314	17.5	0.395533	0.3532045	0.1185395	0.08820886	0.117953	0.133477
2083.414	17.5	0.392508	0.3682167	0.1202778	0.09001449	0.11701	0.1356237
2083.514	17.5	0.389336	0.3724107	0.1179479	0.09173809	0.117217	0.1373775
2083.614	17.5	0.397492	0.3731789	0.119151	0.08872054	0.116872	0.1387609
2083.714	17.5	0.405439	0.3594058	0.1177596	0.08711316	0.118153	0.1384703
2083.814	17.5	0.407555	0.3591358	0.1228122	0.08831659	0.120778	0.139498
2083.914	17.5	0.391741	0.3527733	0.1246678	0.08877654	0.123896	0.1391861
2084.014	17.5	0.394904	0.34751	0.1267783	0.08892572	0.121751	0.139265
2084.114	17.5	0.417619	0.3561551	0.1267448	0.0929473	0.117673	0.1418997
2084.214	17.5	0.416951	0.3512373	0.1243819	0.09379716	0.116431	0.140467
2084.314	17.5	0.420097	0.3481156	0.1253194	0.09166577	0.1155	0.1395079
2084.414	17.5	0.418111	0.3565288	0.1278668	0.09220162	0.113702	0.1368521
2084.514	17.5	0.429618	0.3554963	0.1288115	0.09080338	0.111559	0.1343952
2084.614	17.5	0.429285	0.3480468	0.1289573	0.09356386	0.108676	0.1299026
2084.714	17.5	0.445171	0.355846	0.1266833	0.09705439	0.109601	0.1285589
2084.814	17.5	0.411451	0.3552267	0.1271905	0.09520277	0.108139	0.1282588
2084.914	17.5	0.397168	0.3581468	0.1243736	0.09077771	0.107627	0.1297402
2085.014	17.5	0.422573	0.3486231	0.1234309	0.0887891	0.108037	0.1347698
2085.114	17.5	0.426611	0.3420903	0.1229869	0.08906433	0.109789	0.1358097
2085.214	17.5	0.442998	0.3418126	0.1200307	0.08834668	0.109607	0.1356306
2085.314	17.5	0.427849	0.3432082	0.1195712	0.08870933	0.108208	0.1329704
2085.414	17.5	0.429635	0.3477041	0.1185287	0.08617883	0.10919	0.1333372
2085.514	17.5	0.413807	0.34891	0.1177393	0.08482246	0.11098	0.1329935
2085.614	17.5	0.42059	0.3445536	0.1161592	0.08717722	0.111939	0.1338101
2085.714	17.5	0.428514	0.3459891	0.1147576	0.08593401	0.115607	0.1328363
2085.814	17.5	0.439181	0.3497508	0.11648	0.08868004	0.116346	0.1312154
2085.914	17.5	0.434222	0.3492351	0.1163758	0.08870835	0.114509	0.1323413
2086.014	17.5	0.42149	0.3545984	0.117397	0.08750845	0.116861	0.1333536
2086.114	17.5	0.43381	0.3541367	0.1205209	0.08399695	0.120427	0.1302686
2086.214	17.5	0.428964	0.3541722	0.1247274	0.0820002	0.120641	0.1300476
2086.314	17.5	0.410477	0.3429703	0.1302229	0.08241951	0.12112	0.1324462
2086.414	17.5	0.397439	0.3394288	0.1286562	0.08247374	0.117633	0.1333441
2086.514	17.5	0.400719	0.3493018	0.130463	0.08582228	0.116199	0.1321416
2086.614	17.5	0.39676	0.3632071	0.1315751	0.08491656	0.113184	0.1331101
2086.714	17.5	0.397694	0.3545091	0.1258231	0.08787193	0.113386	0.1321749
2086.814	17.5	0.396205	0.3443317	0.1273395	0.08894362	0.11629	0.1327572
2086.914	17.5	0.390905	0.3529082	0.1261869	0.08795169	0.116569	0.1324299
2087.014	17.5	0.402967	0.3470067	0.1231623	0.08620051	0.114867	0.1315091
2087.114	17.5	0.412287	0.3464236	0.1204513	0.08641	0.112732	0.1334899
2087.214	17.5	0.412617	0.3388583	0.1213038	0.09036642	0.114869	0.1299966
2087.314	17.5	0.410516	0.339815	0.1221974	0.09024056	0.118044	0.1298896
2087.414	17.5	0.396615	0.348039	0.1228098	0.08974597	0.11742	0.1299816
2087.514	17.5	0.415799	0.3523089	0.1255089	0.08989365	0.118502	0.1316475
2087.614	17.5	0.428643	0.3467092	0.1276681	0.09356707	0.117057	0.1328599
2087.714	17.5	0.427261	0.3508	0.1278774	0.09725522	0.117183	0.1331674
2087.814	17.5	0.434179	0.343119	0.1290429	0.09820631	0.11371	0.1321655
2087.914	17.5	0.432262	0.3460918	0.127238	0.09770238	0.111045	0.1351182
2088.014	17.5	0.432971	0.3481691	0.1292014	0.09765516	0.110401	0.1387969
2088.114	17.5	0.447717	0.3398221	0.1307776	0.09726544	0.109345	0.1376248
2088.214	17.5	0.433676	0.3446469	0.1335957	0.09552644	0.109329	0.1334222
2088.314	17.5	0.399655	0.3461922	0.1324347	0.09095318	0.109206	0.1313772
2088.414	17.5	0.411064	0.3487817	0.1300325	0.0865848	0.11111	0.1312666
2088.514	17.5	0.421569	0.3454553	0.1288799	0.08929121	0.112593	0.1306393
2088.614	17.5	0.432684	0.3434413	0.1286381	0.09074042	0.113942	0.1297315

2088.714	17.5	0.4435	0.3286228	0.1267523	0.09230489	0.112285	0.1359097
2088.814	17.5	0.453224	0.3344688	0.1277638	0.09191663	0.110359	0.1357782
2088.914	17.5	0.429382	0.3303438	0.1301053	0.08972186	0.111673	0.1315365
2089.014	17.5	0.450611	0.3356229	0.127015	0.09081049	0.114723	0.13097
2089.114	17.5	0.447313	0.3317412	0.1239012	0.09115423	0.115756	0.1331652
2089.214	17.5	0.467878	0.3292134	0.1207492	0.09018213	0.111436	0.1345401
2089.314	17.5	0.455411	0.3360731	0.1196438	0.09198037	0.112896	0.1364646
2089.414	17.5	0.463192	0.3457801	0.1193399	0.09056025	0.113727	0.1377971
2089.514	17.5	0.447127	0.3392607	0.1193697	0.08791306	0.112606	0.138211
2089.614	17.5	0.441818	0.346087	0.117327	0.08233014	0.117395	0.1375161
2089.714	17.5	0.424254	0.3467872	0.1136935	0.08373374	0.121227	0.139652
2089.814	17.5	0.407767	0.3522052	0.1123306	0.08339429	0.119919	0.1377204
2089.914	17.5	0.413374	0.3473118	0.114121	0.08605619	0.118431	0.135284
2090.014	17.5	0.401088	0.3385847	0.1190656	0.08786621	0.116757	0.1362851
2090.114	17.5	0.400384	0.3411331	0.1254252	0.08958902	0.115985	0.1365021
2090.214	17.5	0.410541	0.3464126	0.1274123	0.08904795	0.118434	0.1384182
2090.314	17.5	0.400569	0.3557592	0.1312706	0.08956403	0.119184	0.1388468
2090.414	17.5	0.402224	0.3474587	0.1281495	0.09036081	0.118758	0.1384068
2090.514	17.5	0.411962	0.3561703	0.1265376	0.08965722	0.11932	0.1353167
2090.614	17.5	0.407189	0.3527269	0.1281257	0.09121152	0.11851	0.1348503
2090.714	17.5	0.417016	0.3450229	0.126695	0.09042305	0.119548	0.1387831
2090.814	17.5	0.396534	0.3505283	0.1240143	0.09164099	0.118924	0.1375963
2090.914	17.5	0.406411	0.3661386	0.1230133	0.09078901	0.118257	0.1350285
2091.014	17.5	0.438455	0.3738946	0.1204034	0.09616844	0.116792	0.1336044
2091.114	17.5	0.42525	0.3729025	0.1232905	0.09981553	0.115588	0.1359259
2091.214	17.5	0.43177	0.3605224	0.1257194	0.101082	0.112494	0.1365289
2091.314	17.5	0.433665	0.3596022	0.1280795	0.1010623	0.110985	0.1384108
2091.414	17.5	0.438123	0.3535477	0.12785	0.1003812	0.108915	0.1402109
2091.514	17.5	0.444064	0.346015	0.129093	0.1003777	0.109021	0.1400192
2091.614	17.5	0.446437	0.3549109	0.128296	0.09819434	0.109027	0.1423673
2091.714	17.5	0.398296	0.3514692	0.125604	0.09399315	0.109006	0.1404177
2091.814	17.5	0.396595	0.3510563	0.1240782	0.09283954	0.110641	0.1407186
2091.914	17.5	0.418619	0.3520662	0.1281819	0.09242961	0.111941	0.1399584
2092.014	17.5	0.428035	0.3574372	0.1277321	0.09429976	0.110675	0.138047
2092.114	17.5	0.438167	0.3470351	0.1267288	0.09399219	0.109249	0.1343977
2092.214	17.5	0.437856	0.35388	0.124392	0.09174909	0.109341	0.1329242
2092.314	17.5	0.424741	0.3509796	0.1246148	0.09054659	0.110542	0.1341041
2092.414	17.5	0.432992	0.3573178	0.1214651	0.09119546	0.113061	0.136697
2092.514	17.5	0.422092	0.3508956	0.1213735	0.09084395	0.114636	0.1377815
2092.614	17.5	0.424402	0.3404596	0.1209487	0.08983833	0.114419	0.1342371
2092.714	17.5	0.420478	0.3394023	0.1205	0.09228671	0.112704	0.1317055
2092.814	17.5	0.430592	0.3438575	0.1197145	0.09082301	0.113942	0.1321544
2092.914	17.5	0.430335	0.3492709	0.1179935	0.08677782	0.114598	0.13399
2093.014	17.5	0.425848	0.3493816	0.1163325	0.08539032	0.113223	0.1362772
2093.114	17.5	0.407371	0.3454008	0.1168393	0.08723271	0.114493	0.1355687
2093.214	17.5	0.402648	0.3483046	0.1164663	0.08683515	0.116608	0.1345469
2093.314	17.5	0.400069	0.3459941	0.1172674	0.08892135	0.11277	0.1341169
2093.414	17.5	0.397912	0.3538526	0.1173347	0.0888484	0.110656	0.1355444
2093.514	17.5	0.392683	0.3538314	0.1181762	0.09164357	0.112163	0.1330248
2093.614	17.5	0.399132	0.3542393	0.1216834	0.09211537	0.115211	0.1307231
2093.714	17.5	0.39862	0.3551067	0.1238501	0.09262223	0.115947	0.1307169
2093.814	17.5	0.402047	0.3446388	0.1292125	0.09059981	0.116033	0.1306451
2093.914	17.5	0.404506	0.3360635	0.129498	0.08881651	0.116361	0.1300896
2094.014	17.5	0.403523	0.3481689	0.1311042	0.08953688	0.11526	0.1290846
2094.114	17.5	0.406337	0.3618855	0.1331427	0.09141266	0.116528	0.1268947
2094.214	17.5	0.408839	0.3545876	0.1268232	0.09195382	0.11443	0.1264522
2094.314	17.5	0.410169	0.3436714	0.1278005	0.09127232	0.110198	0.1262294
2094.414	17.5	0.433657	0.3534445	0.1271954	0.08982703	0.107187	0.1281536
2094.514	17.5	0.425491	0.3462096	0.1248673	0.09233686	0.107461	0.1304132
2094.614	17.5	0.421912	0.3462182	0.1232155	0.09600376	0.108786	0.1315618
2094.714	17.5	0.434084	0.3387567	0.1244304	0.09888247	0.107174	0.1322289
2094.814	17.5	0.443731	0.3401718	0.1241063	0.1016293	0.107871	0.1330339
2094.914	17.5	0.434708	0.3463138	0.1235333	0.09977424	0.105724	0.1347044
2095.014	17.5	0.435974	0.349566	0.1247683	0.09956614	0.106525	0.1379174
2095.114	17.5	0.398887	0.3476244	0.1264174	0.09610348	0.104006	0.1395496
2095.214	17.5	0.396855	0.3483606	0.1260775	0.0921106	0.103516	0.1413656
2095.314	17.5	0.425698	0.341761	0.127403	0.09064297	0.103689	0.1440455
2095.414	17.5	0.424041	0.3479965	0.1245217	0.09387945	0.104606	0.1422509
2095.514	17.5	0.429706	0.3450758	0.1272374	0.09130945	0.104494	0.1442155
2095.614	17.5	0.426201	0.339085	0.1274697	0.08890285	0.106472	0.1405715
2095.714	17.5	0.41132	0.3432889	0.1313384	0.08649556	0.107309	0.1370384
2095.814	17.5	0.413618	0.3451388	0.1280432	0.08626249	0.111331	0.1347624
2095.914	17.5	0.413924	0.3474284	0.1258511	0.08734716	0.111376	0.1337368
2096.014	17.5	0.429401	0.3459529	0.1250383	0.08621214	0.111995	0.1331355
2096.114	17.5	0.426823	0.3449898	0.1248644	0.08760324	0.11052	0.1317536
2096.214	17.5	0.425569	0.3256595	0.1228727	0.0854386	0.113477	0.1346054
2096.314	17.5	0.419789	0.3328192	0.1223192	0.0836441	0.114862	0.1338003
2096.414	17.5	0.421899	0.3353499	0.121294	0.08278673	0.116318	0.1325164
2096.514	17.5	0.407235	0.3323327	0.1199559	0.07908445	0.11665	0.132942
2096.614	17.5	0.400168	0.3342344	0.1187517	0.08146758	0.117405	0.1313498
2096.714	17.5	0.39619	0.328232	0.1173142	0.08173226	0.1143	0.1321745
2096.814	17.5	0.396601	0.3376541	0.1169374	0.0846774	0.112348	0.1320272
2096.914	17.5	0.392388	0.3464902	0.1192117	0.08290713	0.113439	0.134713
2097.014	17.5	0.401163	0.3381956	0.1197944	0.08504459	0.116726	0.134158
2097.114	17.5	0.403992	0.346446	0.1194308	0.08595823	0.119098	0.134317
2097.214	17.5	0.401401	0.3441051	0.1201984	0.08749607	0.119899	0.134811
2097.314	17.5	0.4104	0.3538642	0.118969	0.08696207	0.117071	0.1321632
2097.414	17.5	0.410805	0.346949	0.1202128	0.08754658	0.116724	0.1305969
2097.514	17.5	0.419656	0.3375023	0.1247356	0.08798097	0.118807	0.1317718
2097.614	17.5	0.412667	0.3401507	0.1299438	0.08595471	0.117378	0.1327432
2097.714	17.5	0.403592	0.3463392	0.1349788	0.08745612	0.116451	0.132228
2097.814	17.5	0.420252	0.3545301	0.1374839	0.09068657	0.114213	0.1305172
2097.914	17.5	0.430233	0.3459987	0.1346694	0.08851582	0.112699	0.1284667
2098.014	17.5	0.442764	0.355414	0.1319921	0.08971869	0.111776	0.1290591
2098.114	17.5	0.442707	0.3493842	0.1335939	0.09230932	0.111283	0.1300669

2098.214	17.5	0.448627	0.3433205	0.1319133	0.09558862	0.112802	0.1332462
2098.356	17.5	0.436319	0.3496836	0.1282773	0.09460007	0.109667	0.1331427
2098.456	18	0.456441	0.3656411	0.1278297	0.09344634	0.107941	0.1325886
2098.556	18	0.452883	0.3723049	0.1266415	0.0921779	0.109401	0.134121
2098.656	18	0.469467	0.3718261	0.1259888	0.08203828	0.108643	0.1365559
2098.756	18	0.459639	0.3586234	0.1249584	0.08345131	0.107994	0.1355085
2098.856	18	0.458667	0.3585756	0.1294871	0.08169635	0.107785	0.1380966
2098.956	18	0.443741	0.3529592	0.1298286	0.08265033	0.107317	0.136833
2099.056	18	0.439439	0.3489564	0.1311393	0.08243062	0.106513	0.1382391
2099.156	18	0.41256	0.3547733	0.1290448	0.08386353	0.10747	0.1383254
2099.256	18	0.411257	0.3516359	0.1322602	0.08572599	0.107628	0.1376732
2099.356	18	0.409145	0.3520653	0.1315361	0.08550147	0.11161	0.1418596
2099.456	18	0.400849	0.3529243	0.1324802	0.08523423	0.111016	0.1380172
2099.556	18	0.404879	0.3552054	0.129717	0.08735283	0.11148	0.1334732
2099.656	18	0.406473	0.3479255	0.1264582	0.08677987	0.117809	0.1313408
2099.756	18	0.406738	0.3533238	0.1238484	0.08739825	0.118378	0.1334374
2099.856	18	0.416529	0.3518456	0.1237941	0.08805224	0.11491	0.1327619
2099.956	18	0.403923	0.3588982	0.1260156	0.08999069	0.119372	0.1334033
2100.056	18	0.422573	0.3523095	0.1218543	0.08904287	0.12026	0.1338644
2100.156	18	0.400097	0.3427747	0.1224315	0.0915366	0.120125	0.1326397
2100.256	18	0.412443	0.3420535	0.1214701	0.09405869	0.118199	0.132548
2100.356	18	0.438307	0.3443004	0.1215157	0.09885269	0.117258	0.1312973
2100.456	18	0.430696	0.3512613	0.1208673	0.09937785	0.115277	0.1323809
2100.556	18	0.437385	0.3495574	0.1197457	0.09750934	0.118484	0.1326386
2100.656	18	0.448472	0.3464782	0.1222764	0.09241442	0.118129	0.1347547
2100.756	18	0.441256	0.3501496	0.1219972	0.08855328	0.118338	0.1342638
2100.856	18	0.450322	0.3443957	0.1191217	0.08910654	0.11793	0.1337237
2100.956	18	0.442207	0.3590927	0.1154914	0.08894569	0.116705	0.1318902
2101.056	18	0.405307	0.3517745	0.1217651	0.09084642	0.116073	0.1298149
2101.156	18	0.407278	0.3549639	0.1264632	0.08960186	0.116025	0.128632
2101.256	18	0.419908	0.3539904	0.1288589	0.08814283	0.110104	0.1303825
2101.356	18	0.42342	0.3480923	0.1351956	0.08680224	0.108272	0.1297397
2101.456	18	0.432336	0.3377611	0.1352015	0.08672378	0.1104	0.1292304
2101.556	18	0.435231	0.3465713	0.13341	0.08680854	0.110483	0.1288781
2101.656	18	0.416533	0.3619581	0.1323525	0.08747313	0.106491	0.1272739
2101.756	18	0.425177	0.3585996	0.1309411	0.08763551	0.104843	0.124321
2101.856	18	0.416136	0.3425262	0.1271829	0.08558733	0.103759	0.1258572
2101.956	18	0.430047	0.3537658	0.1253028	0.08050894	0.104241	0.1272048
2102.056	18	0.422161	0.3466151	0.1219873	0.08019143	0.104152	0.1312092
2102.156	18	0.426756	0.3475426	0.1197904	0.07914083	0.106417	0.1314241
2102.256	18	0.422943	0.3397192	0.1242868	0.08081661	0.107779	0.1317274
2102.356	18	0.419238	0.3404577	0.1284418	0.08159138	0.107273	0.1277609
2102.456	18	0.405933	0.3462556	0.1274425	0.08491851	0.106191	0.1308659
2102.556	18	0.39818	0.3484497	0.1254564	0.08582222	0.105299	0.1311314
2102.656	18	0.399676	0.3479791	0.1218194	0.08648881	0.106827	0.1341462
2102.756	18	0.396277	0.3489272	0.1230556	0.08645117	0.112146	0.1365054
2102.856	18	0.403766	0.3410927	0.1264553	0.08690284	0.112773	0.1373773
2102.956	18	0.407229	0.347547	0.1286863	0.08726188	0.111328	0.1364416
2103.056	18	0.401679	0.3442939	0.1283997	0.08808775	0.112637	0.1385723
2103.156	18	0.411557	0.3382649	0.1254805	0.08843642	0.116184	0.1351107
2103.256	18	0.41071	0.3434412	0.1255957	0.087708	0.119338	0.1333677
2103.356	18	0.425577	0.3440364	0.1235738	0.09077887	0.119363	0.1338134
2103.456	18	0.401151	0.347423	0.1208319	0.09558142	0.11887	0.1334123
2103.556	18	0.408055	0.3476121	0.1223143	0.09708466	0.119625	0.1336335
2103.656	18	0.419855	0.3449228	0.122119	0.09746987	0.119954	0.1328115
2103.756	18	0.429922	0.3245912	0.1215818	0.09706657	0.115506	0.133396
2103.856	18	0.418971	0.3341797	0.1169667	0.09568901	0.112315	0.1329956
2103.956	18	0.420981	0.336751	0.1159655	0.09652945	0.113479	0.132068
2104.056	18	0.427903	0.3327603	0.1173501	0.09077783	0.115143	0.1314872
2104.156	18	0.426558	0.3347206	0.1207136	0.08900043	0.112414	0.1301071
2104.256	18	0.448105	0.3285912	0.1183281	0.08966284	0.111284	0.1307941
2104.356	18	0.405586	0.3411891	0.1204981	0.08907118	0.112957	0.1309036
2104.456	18	0.404784	0.3481154	0.1240991	0.08855068	0.116264	0.1297774
2104.556	18	0.420845	0.3398384	0.1213956	0.0861307	0.115774	0.1338833
2104.656	18	0.417937	0.3478244	0.1222837	0.08697789	0.115197	0.1325968
2104.756	18	0.439633	0.3439477	0.1267914	0.08837759	0.112306	0.1334472
2104.856	18	0.448284	0.3550309	0.1264968	0.0865513	0.112332	0.1319765
2104.956	18	0.434953	0.3466949	0.1297918	0.0849764	0.110414	0.1315211
2105.056	18	0.438465	0.3384145	0.1297487	0.08496997	0.108426	0.1306693
2105.156	18	0.447279	0.3416863	0.1294073	0.0856088	0.107276	0.1311921
2105.256	18	0.452345	0.3453836	0.1303992	0.08341023	0.102701	0.1296843
2105.356	18	0.45738	0.3546485	0.127478	0.08106332	0.104581	0.1302581
2105.456	18	0.456022	0.3478206	0.1257163	0.0800665	0.105894	0.1315001
2105.556	18	0.452211	0.355812	0.123318	0.0838843	0.10662	0.1321688
2105.656	18	0.436444	0.3496886	0.1202489	0.08707437	0.105539	0.1311511
2105.756	18	0.428075	0.342881	0.121225	0.08849694	0.106059	0.1339528
2105.856	18	0.407642	0.3489559	0.1225984	0.08795851	0.104335	0.1345361
2105.956	18	0.410156	0.3648198	0.1252358	0.08949857	0.106085	0.1330396
2106.056	18	0.406855	0.3716145	0.1268989	0.08954215	0.106995	0.13341
2106.156	18	0.406915	0.3711397	0.1266862	0.08859889	0.109178	0.136888
2106.256	18	0.410592	0.359157	0.1236537	0.09023851	0.111225	0.1401415
2106.356	18	0.408332	0.3577784	0.1251763	0.08811938	0.113669	0.1394504
2106.456	18	0.417441	0.3520245	0.1272602	0.08797128	0.112689	0.1380723
2106.556	18	0.42058	0.3479272	0.1304642	0.0874991	0.110355	0.137593
2106.656	18	0.418551	0.3562258	0.1274253	0.08936104	0.109844	0.1399399
2106.756	18	0.419713	0.3512942	0.1270443	0.09104083	0.114296	0.1392158
2106.856	18	0.412317	0.3494204	0.1274559	0.09116291	0.118371	0.1410739
2106.956	18	0.425245	0.3506227	0.1254013	0.09246577	0.118859	0.1391052
2107.056	18	0.440317	0.3534179	0.1230212	0.09346078	0.118094	0.134498
2107.156	18	0.439556	0.345558	0.1203031	0.09123767	0.113404	0.1325887
2107.256	18	0.443919	0.3538134	0.1213843	0.09233133	0.113973	0.1318654
2107.356	18	0.444978	0.3534687	0.1198347	0.09020949	0.114342	0.1333295
2107.456	18	0.437437	0.3598589	0.1193918	0.08821714	0.115907	0.1314046
2107.556	18	0.45252	0.3515343	0.1185019	0.0867585	0.117231	0.1296015
2107.656	18	0.434295	0.3401003	0.1170951	0.08950125	0.115018	0.1292619

2107.756	18	0.407612	0.3409073	0.1180797	0.09065066	0.115053	0.1275826
2107.856	18	0.418958	0.3451186	0.1156716	0.08778592	0.115961	0.1279751
2107.956	18	0.431731	0.3507135	0.1166242	0.08779626	0.115804	0.1277053
2108.056	18	0.445864	0.3493346	0.1138416	0.0873467	0.112466	0.1292498
2108.156	18	0.436029	0.3466888	0.1138141	0.08782215	0.111014	0.1271968
2108.256	18	0.442703	0.3492374	0.1170571	0.08711419	0.111362	0.130619
2108.356	18	0.433991	0.3484344	0.1258614	0.08610581	0.108084	0.1315874
2108.456	18	0.446992	0.3528721	0.1290253	0.08278415	0.107443	0.1306942
2108.556	18	0.435999	0.3500944	0.1309873	0.08410898	0.105951	0.131224
2108.656	18	0.45189	0.3520275	0.1336521	0.0814658	0.105621	0.1308646
2108.756	18	0.441729	0.3536645	0.1297123	0.0808427	0.10531	0.1304141
2108.856	18	0.438967	0.3443082	0.1298878	0.08255409	0.104157	0.1310536
2108.956	18	0.42799	0.3386738	0.1293694	0.08404442	0.106027	0.1293846
2109.056	18	0.41631	0.3482434	0.1237346	0.08351419	0.10661	0.1285545
2109.156	18	0.407897	0.3627854	0.1240876	0.08598665	0.107019	0.1284984
2109.256	18	0.404725	0.3542491	0.1221397	0.09028109	0.106516	0.127613
2109.356	18	0.403546	0.3434117	0.1250171	0.0900907	0.105957	0.1279496
2109.456	18	0.402798	0.3557695	0.1244232	0.08841788	0.106804	0.1291345
2109.556	18	0.408163	0.3456789	0.1284743	0.08768765	0.110934	0.1324156
2109.656	18	0.406433	0.3480385	0.1292219	0.08424651	0.113312	0.131994
2109.756	18	0.404407	0.340489	0.1294014	0.08641857	0.111741	0.1301134
2109.856	18	0.409481	0.3417405	0.127804	0.08712543	0.110554	0.1325103
2109.956	18	0.404475	0.3469906	0.1255771	0.0917549	0.113142	0.1339364
2110.056	18	0.42956	0.3482943	0.126391	0.09401844	0.112853	0.1349888
2110.156	18	0.408229	0.3489045	0.1284667	0.0958241	0.116646	0.1377224
2110.256	18	0.418901	0.3502666	0.131135	0.09875317	0.119973	0.139376
2110.356	18	0.436348	0.3423325	0.1307734	0.09799782	0.116339	0.1392058
2110.456	18	0.415751	0.3457405	0.1280929	0.09732319	0.114302	0.1386468
2110.556	18	0.429932	0.3438399	0.1284854	0.09754641	0.113537	0.1399613
2110.656	18	0.444887	0.3393874	0.124999	0.09672062	0.111557	0.1365999
2110.756	18	0.443125	0.3429453	0.1246205	0.09253767	0.112768	0.1326225
2110.856	18	0.447189	0.3438517	0.1246448	0.09163046	0.114453	0.1320024
2110.956	18	0.441242	0.3472269	0.1237383	0.09438094	0.116252	0.1308983
2111.056	18	0.407253	0.3489695	0.124371	0.09365146	0.11409	0.1320561
2111.156	18	0.407406	0.3451855	0.1197052	0.09195218	0.116212	0.1333002
2111.256	18	0.430765	0.3286498	0.1184017	0.0890199	0.116309	0.1320862
2111.356	18	0.434148	0.3357092	0.1209661	0.08737294	0.116986	0.1312326
2111.456	18	0.436507	0.3375492	0.1242803	0.087536	0.11466	0.1285125
2111.556	18	0.435677	0.3353793	0.1248265	0.08576082	0.112606	0.1288768
2111.656	18	0.42223	0.3353502	0.1241213	0.0843856	0.11095	0.1296613
2111.756	18	0.429994	0.3301695	0.1236752	0.08326875	0.109374	0.1328869
2111.856	18	0.44754	0.3401888	0.1244242	0.08201534	0.10745	0.132281
2111.956	18	0.461563	0.3465775	0.1290832	0.08068904	0.104857	0.1348596
2112.056	18	0.448326	0.3408675	0.1348853	0.08131837	0.104005	0.1322642
2112.156	18	0.448264	0.3489884	0.1367569	0.08249597	0.105178	0.130543
2112.256	18	0.451545	0.3457312	0.1390352	0.08447786	0.103749	0.1310655
2112.356	18	0.449131	0.3540086	0.1332637	0.08541878	0.103346	0.1310186
2112.456	18	0.426352	0.3486118	0.1305114	0.08565529	0.104436	0.1275108
2112.556	18	0.409963	0.3398562	0.1297098	0.08713167	0.105801	0.1322758
2112.656	18	0.409113	0.3422475	0.1263596	0.08831813	0.105048	0.1352472
2112.756	18	0.401102	0.3470462	0.1240052	0.08728302	0.106009	0.1331738
2112.856	18	0.40813	0.3549319	0.1231736	0.08955038	0.106776	0.1315921
2112.956	18	0.407609	0.347378	0.1234553	0.09049468	0.106705	0.1305076
2113.056	18	0.405762	0.3564204	0.1226454	0.09061395	0.115116	0.1321524
2113.156	18	0.41387	0.3503076	0.1239469	0.08973261	0.116586	0.1325919
2113.256	18	0.412234	0.3447584	0.126471	0.08756356	0.115213	0.1331098
2113.356	18	0.434272	0.3511534	0.1245945	0.0886398	0.115589	0.133772
2113.456	18	0.415064	0.3664442	0.1290394	0.08586228	0.114346	0.1315583
2113.556	18	0.42119	0.3705494	0.1292639	0.0865766	0.114134	0.1296438
2113.656	18	0.436528	0.3713702	0.1290902	0.08907819	0.115874	0.1314197
2113.756	18	0.449893	0.3584193	0.129137	0.09109998	0.117155	0.1346414
2113.856	18	0.452104	0.3578219	0.1313869	0.09271749	0.115029	0.1354887
2113.956	18	0.456548	0.352632	0.1310605	0.09072477	0.115938	0.1367252
2114.056	18	0.443124	0.3474758	0.1302205	0.08733438	0.115753	0.1344503
2114.156	18	0.436944	0.3574395	0.128985	0.08430492	0.11785	0.1356818
2114.256	18	0.454871	0.3519154	0.1281253	0.08676414	0.116735	0.137907
2114.356	18	0.416327	0.3509345	0.1296931	0.08685484	0.116432	0.1375891
2114.456	18	0.411748	0.3509191	0.1288739	0.08570479	0.116464	0.1367539
2114.556	18	0.435481	0.3536208	0.1291537	0.0839389	0.114727	0.1356068
2114.656	18	0.435518	0.3447995	0.1277348	0.08350112	0.114707	0.1307763
2114.756	18	0.449595	0.3547463	0.1280075	0.08373426	0.119312	0.1304553
2114.856	18	0.447027	0.3551647	0.1270361	0.08251152	0.117607	0.1304729
2114.956	18	0.441138	0.3601134	0.1268694	0.08237181	0.116551	0.1298653
2115.056	18	0.43935	0.3504749	0.12684	0.08190025	0.115861	0.1289821
2115.156	18	0.450405	0.3422773	0.1275775	0.08277577	0.112993	0.1285814
2115.256	18	0.451041	0.3431008	0.124693	0.07995391	0.111592	0.1262332
2115.356	18	0.457003	0.3462605	0.1263286	0.07871254	0.109673	0.1280888
2115.456	18	0.455543	0.3503704	0.12762	0.07835605	0.107728	0.1292254
2115.556	18	0.446747	0.3494665	0.1272809	0.08098843	0.106252	0.1312475
2115.656	18	0.436449	0.3479646	0.1296818	0.08397356	0.10657	0.1325755
2115.756	18	0.414692	0.3487648	0.1302131	0.08477531	0.10528	0.1323882
2115.856	18	0.401803	0.3519474	0.1326622	0.08392274	0.105384	0.1322357
2115.956	18	0.403068	0.3513634	0.1301153	0.0850109	0.107562	0.1325946
2116.056	18	0.399184	0.3536073	0.1255538	0.08395568	0.108036	0.1302331
2116.156	18	0.400603	0.3541064	0.1251418	0.08172058	0.106507	0.1282317
2116.256	18	0.403288	0.3532323	0.1233314	0.08327177	0.108132	0.1290523
2116.356	18	0.40305	0.3424714	0.1214535	0.08228347	0.11008	0.1303847
2116.456	18	0.414998	0.3412166	0.1227595	0.08235228	0.114855	0.1310129
2116.556	18	0.427172	0.3504824	0.1227161	0.08011812	0.116399	0.1322473
2116.656	18	0.4266	0.3639275	0.1242031	0.08365573	0.116121	0.1317768
2116.756	18	0.431284	0.3532609	0.1257559	0.08632417	0.114985	0.1312686
2116.856	18	0.422531	0.3459123	0.1282977	0.08867402	0.115202	0.1328012
2116.956	18	0.427453	0.3538199	0.1262814	0.08880702	0.11356	0.1340155
2117.056	18	0.430146	0.347125	0.1259367	0.09027258	0.113962	0.135764
2117.156	18	0.429556	0.3469273	0.1275698	0.09059715	0.116757	0.1320366

2117.256	18	0.432572	0.3416211	0.1287416	0.09107371	0.118946	0.1321842
2117.356	18	0.439365	0.3419372	0.1262841	0.08735511	0.120269	0.1329563
2117.456	18	0.433982	0.3483007	0.1311117	0.08569857	0.118477	0.1360019
2117.556	18	0.439091	0.3518116	0.1304145	0.08535606	0.116595	0.1372383
2117.656	18	0.424676	0.347414	0.127316	0.08498982	0.116995	0.1406124
2117.756	18	0.407195	0.3516252	0.1269725	0.08321925	0.118682	0.1425987
2117.856	18	0.4232	0.3412788	0.1248401	0.08293169	0.1199	0.1440847
2117.956	18	0.441226	0.3456866	0.1231108	0.08582387	0.118826	0.1421151
2118.056	18	0.444881	0.3481446	0.1228438	0.08488917	0.11746	0.1389256
2118.156	18	0.431237	0.3381779	0.1222805	0.0843715	0.119182	0.134949
2118.256	18	0.425191	0.3433487	0.1203639	0.08322509	0.116761	0.1361266
2118.356	18	0.415765	0.3454587	0.1203615	0.08522615	0.117022	0.1335951
2118.456	18	0.420403	0.346456	0.1194333	0.08433314	0.114537	0.134146
2118.556	18	0.436218	0.3484905	0.1193161	0.08437229	0.114259	0.134323
2118.656	18	0.429665	0.3442617	0.120915	0.08241476	0.111907	0.1320149
2118.756	18	0.435324	0.3293625	0.1251256	0.08098109	0.105854	0.1342339
2118.856	18	0.424134	0.3358322	0.1226542	0.08017069	0.106113	0.1324874
2118.956	18	0.433	0.3379134	0.121796	0.08387152	0.105666	0.1303361
2119.056	18	0.414326	0.3361549	0.1249036	0.08306752	0.105961	0.1305204
2119.156	18	0.410811	0.3359836	0.1299394	0.08620013	0.109241	0.1292814
2119.256	18	0.396255	0.328701	0.1361442	0.08634555	0.111673	0.1310846
2119.356	18	0.392747	0.3423725	0.1358986	0.08724589	0.109378	0.1302505
2119.456	18	0.396248	0.346809	0.1343154	0.0867788	0.110266	0.1325176
2119.556	18	0.40042	0.3418625	0.1334025	0.08756762	0.108778	0.1332034
2119.656	18	0.400933	0.3499797	0.1269382	0.08655431	0.109717	0.128456
2119.756	18	0.407345	0.3461131	0.1267036	0.0865147	0.107594	0.130862
2119.856	18	0.414719	0.3564219	0.1267229	0.08403865	0.11031	0.133067
2119.956	18	0.410342	0.3498	0.1233797	0.08545829	0.112842	0.1327972
2120.056	18	0.412159	0.3423911	0.1235306	0.09047765	0.115544	0.1321023
2120.156	18	0.413312	0.343721	0.1220426	0.09140325	0.114344	0.1312955
2120.256	18	0.414259	0.3470014	0.1234405	0.09280429	0.117511	0.1306427
2120.356	18	0.433814	0.3559795	0.1229224	0.09263903	0.116791	0.1317804
2120.456	18	0.436672	0.3495465	0.1249238	0.09356011	0.117192	0.133583
2120.556	18	0.443321	0.3562009	0.1261686	0.09391474	0.116813	0.133642
2120.656	18	0.434049	0.3509462	0.1268322	0.09145772	0.117124	0.1327794
2120.756	18	0.437416	0.3454612	0.1272026	0.08911633	0.11551	0.1322391
2120.856	18	0.447521	0.3524675	0.1256591	0.08738849	0.11523	0.1320436
2120.956	18	0.445686	0.3665797	0.1264247	0.08774818	0.116579	0.1334138
2121.056	18	0.41169	0.3715571	0.1283229	0.09031648	0.119131	0.1350091
2121.156	18	0.422077	0.3710983	0.1299759	0.09390929	0.121076	0.1358531
2121.256	18	0.429339	0.3591453	0.1297081	0.09276974	0.120267	0.1359161
2121.356	18	0.44236	0.3587632	0.1278336	0.09091383	0.119554	0.1371826
2121.456	18	0.450923	0.353097	0.1276387	0.09053935	0.116838	0.1373091
2121.556	18	0.447982	0.3477004	0.1246401	0.09079921	0.116157	0.1387184
2121.656	18	0.442237	0.3571113	0.123177	0.08931813	0.115041	0.1336192
2121.756	18	0.452864	0.3509689	0.1245599	0.08827747	0.113976	0.1313998
2121.856	18	0.460991	0.3503691	0.125404	0.08465412	0.113075	0.130166
2121.956	18	0.466843	0.3503853	0.1254762	0.08225384	0.115507	0.1313986
2122.056	18	0.458471	0.3536555	0.1237102	0.08361251	0.115032	0.1298777
2122.156	18	0.46873	0.3442903	0.1219441	0.08401063	0.110761	0.128756
2122.256	18	0.455145	0.3525737	0.1208262	0.08576021	0.109716	0.132469
2122.356	18	0.439102	0.3555229	0.1219623	0.08617988	0.108582	0.130212
2122.456	18	0.429963	0.3585074	0.1220227	0.08566812	0.108839	0.1295492
2122.556	18	0.412846	0.3501063	0.1219387	0.0845271	0.108418	0.127846
2122.656	18	0.414264	0.3426549	0.1221009	0.08861278	0.10796	0.1258404
2122.756	18	0.403666	0.3434071	0.1241738	0.0892099	0.108379	0.1271931
2122.856	18	0.407285	0.3447376	0.1286836	0.0881207	0.10834	0.1239992
2122.956	18	0.403696	0.3500694	0.1311154	0.08683363	0.107948	0.1237724
2123.056	18	0.408994	0.3494242	0.133141	0.08194871	0.107199	0.1244036
2123.156	18	0.422348	0.3480096	0.1342509	0.0807673	0.107775	0.1238589
2123.256	18	0.415506	0.3505767	0.1296965	0.08089633	0.108789	0.1232669
2123.356	18	0.426198	0.347975	0.1251353	0.08621261	0.11087	0.1211525
2123.456	18	0.416319	0.3547266	0.1258159	0.09111202	0.113009	0.1238502
2123.556	18	0.420617	0.3539653	0.1256806	0.09402705	0.113012	0.127775
2123.656	18	0.44216	0.3543513	0.1221572	0.09693456	0.115466	0.1296973
2123.756	18	0.445403	0.3535282	0.1226105	0.09777518	0.116461	0.1290931
2123.856	18	0.44376	0.3459589	0.1234896	0.09523018	0.114095	0.1282036
2123.956	18	0.439957	0.3395955	0.1219373	0.09541505	0.118032	0.1288905
2124.056	18	0.444435	0.3486961	0.1211996	0.09029495	0.118891	0.1294963
2124.156	18	0.443122	0.3617696	0.1242556	0.08712483	0.117438	0.1342023
2124.256	18	0.450866	0.3571883	0.1231646	0.08999199	0.115644	0.134598
2124.356	18	0.423683	0.3468067	0.1254903	0.08897223	0.117219	0.1337386
2124.456	18	0.416979	0.3563917	0.1278597	0.08992726	0.115888	0.1312673
2124.556	18	0.432302	0.3475898	0.1279556	0.08713201	0.117565	0.1315742
2124.656	18	0.43604	0.3502654	0.1262234	0.08816192	0.117924	0.1327929
2124.756	18	0.447422	0.3434882	0.1294326	0.08862386	0.117833	0.1347681
2124.856	18	0.438661	0.3427737	0.1260794	0.08871118	0.117737	0.136878
2124.956	18	0.442469	0.3474403	0.1239107	0.08496013	0.117823	0.1384003
2125.056	18	0.435956	0.3523976	0.1240236	0.08240727	0.118502	0.1370658
2125.156	18	0.443439	0.3495664	0.1230788	0.08140428	0.115278	0.1376292
2125.256	18	0.434275	0.3525144	0.1208619	0.0783906	0.113108	0.1354351
2125.356	18	0.430745	0.3424105	0.1208856	0.07739081	0.11327	0.1360779
2125.456	18	0.427105	0.3477694	0.1202828	0.07889839	0.113498	0.1334763
2125.556	18	0.417864	0.3474427	0.1202408	0.07997215	0.111269	0.1337819
2125.656	18	0.417896	0.3386236	0.1191838	0.08026779	0.1099	0.131348
2125.756	18	0.418335	0.3419414	0.1183114	0.08232459	0.109451	0.1311448
2125.856	18	0.40505	0.3448792	0.1158646	0.08567804	0.108752	0.1325941
2125.956	18	0.403484	0.3472255	0.1173438	0.08671115	0.108431	0.1327409
2126.056	18	0.399853	0.347583	0.1191197	0.08798931	0.109103	0.1346563
2126.156	18	0.399066	0.345926	0.1206409	0.08789767	0.107994	0.132643
2126.256	18	0.403892	0.3296773	0.1217455	0.08751356	0.107501	0.1284548
2126.356	18	0.401188	0.3380665	0.1248342	0.08804733	0.108863	0.129025
2126.456	18	0.414669	0.335399	0.126488	0.0863941	0.106447	0.128463
2126.556	18	0.425663	0.3377753	0.1302572	0.08530181	0.106775	0.1280873
2126.656	18	0.427038	0.3363023	0.1347923	0.08905853	0.108662	0.1312389

2126.756	18	0.426299	0.3295971	0.1372213	0.0903455	0.113033	0.1314379
2126.856	18	0.431051	0.3429173	0.1339677	0.09133267	0.114339	0.1303972
2126.956	18	0.437299	0.3483195	0.1258799	0.09180597	0.111805	0.1287071
2127.056	18	0.434713	0.3426868	0.127188	0.09223619	0.110048	0.1302397
2127.156	18	0.433056	0.3519986	0.1262392	0.09122774	0.114749	0.1322958
2127.256	18	0.426838	0.3466328	0.1208857	0.09167086	0.113072	0.1325825
2127.356	18	0.4309	0.357008	0.1221335	0.08822909	0.114277	0.1317684
2127.456	18	0.430293	0.3508236	0.1191686	0.08488365	0.117172	0.1302541
2127.556	18	0.438012	0.3429843	0.1204328	0.08706031	0.115932	0.131249
2127.656	18	0.419702	0.3435984	0.117963	0.08813789	0.113639	0.1312355
2127.756	18	0.402405	0.3462097	0.1211918	0.09014356	0.113463	0.1318497
2127.856	18	0.415843	0.3557844	0.1234125	0.08741292	0.11549	0.1345638
2127.956	18	0.433529	0.3517524	0.1240779	0.08741926	0.115816	0.1370428
2128.056	18	0.432122	0.3558587	0.1254071	0.08818623	0.116966	0.1375391
2128.156	18	0.438596	0.3514854	0.1281404	0.08947625	0.116157	0.1421999
2128.256	18	0.445216	0.3652178	0.1270216	0.08677231	0.11646	0.142505
2128.356	18	0.409887	0.3699613	0.1292223	0.08701728	0.116321	0.142983
2128.456	18	0.440769	0.3707403	0.1297176	0.08270451	0.116588	0.1415591
2128.556	18	0.442989	0.3575038	0.1283572	0.0804935	0.113289	0.140724
2128.656	18	0.465151	0.356834	0.1250548	0.07994361	0.111435	0.1400694
2128.756	18	0.463137	0.3498894	0.1257636	0.08232892	0.111401	0.1409601
2128.856	18	0.47004	0.3490428	0.1241049	0.08174219	0.112285	0.1377735
2128.956	18	0.460411	0.3586734	0.1250753	0.08350358	0.112875	0.1364501
2129.056	18	0.444031	0.3523839	0.1260687	0.08413054	0.109418	0.1361585
2129.156	18	0.428568	0.3476785	0.1230635	0.08631039	0.109665	0.1330634
2129.256	18	0.416096	0.3535365	0.1228902	0.08741553	0.106408	0.1337806
2129.356	18	0.410717	0.3439839	0.1225648	0.08888171	0.107597	0.1327841
2129.456	18	0.408952	0.3509626	0.1211005	0.08805682	0.107813	0.1339289
2129.556	18	0.408714	0.3529173	0.1224657	0.08617011	0.111725	0.1354747
2129.656	18	0.39921	0.3545778	0.1233658	0.08349138	0.112166	0.1367791
2129.756	18	0.412909	0.3513791	0.1256329	0.08205246	0.110519	0.1355681
2129.856	18	0.43509	0.3417346	0.1264017	0.08166254	0.109255	0.1343386
2129.956	18	0.433639	0.3419754	0.1262214	0.08230954	0.109142	0.1321152
2130.056	18	0.433533	0.3446178	0.1258515	0.0888806	0.11167	0.132958
2130.156	18	0.421939	0.3489381	0.1257813	0.08854442	0.111016	0.1322832
2130.256	18	0.436364	0.3501118	0.126196	0.08674066	0.114128	0.1315819
2130.356	18	0.457155	0.3476081	0.1275964	0.08766727	0.115943	0.1330119
2130.456	18	0.453774	0.3441685	0.128857	0.08686051	0.114075	0.1313927
2130.556	18	0.457916	0.3588172	0.1259442	0.08939272	0.116572	0.130079
2130.656	18	0.457611	0.3462234	0.1267158	0.08769094	0.11968	0.132938
2130.756	18	0.45896	0.3533472	0.1271207	0.08507766	0.116086	0.1317339
2130.856	18	0.466351	0.3534431	0.1243994	0.08556248	0.116876	0.1301561
2130.956	18	0.460569	0.348897	0.1248252	0.08725077	0.114711	0.1336401
2131.056	18	0.429902	0.3389897	0.1252018	0.08857692	0.113153	0.1316073
2131.156	18	0.438167	0.347344	0.1235335	0.08750883	0.113792	0.1327202
2131.256	18	0.445354	0.359496	0.1245981	0.08674023	0.115556	0.1313695
2131.356	18	0.451964	0.3560078	0.1254214	0.08954043	0.114122	0.1303799
2131.456	18	0.461142	0.3453956	0.1276962	0.08662294	0.113539	0.1324383
2131.556	18	0.455465	0.3513506	0.1268763	0.08653329	0.113439	0.1339886
2131.656	18	0.451203	0.348491	0.1265652	0.08826364	0.113668	0.1321029
2131.756	18	0.457509	0.345246	0.1266999	0.08769142	0.113386	0.1332188
2131.856	18	0.457073	0.3424311	0.1282182	0.08521461	0.111899	0.1346345
2131.956	18	0.466808	0.3431523	0.1281098	0.08268522	0.112926	0.1367393
2132.056	18	0.445517	0.3497635	0.1329979	0.08104958	0.110535	0.1359218
2132.156	18	0.455993	0.3488461	0.1321663	0.0816076	0.10974	0.1385859
2132.256	18	0.424747	0.3502458	0.128427	0.08320772	0.108526	0.1408744
2132.356	18	0.430203	0.3418741	0.1278691	0.08273278	0.106807	0.1408108
2132.456	18	0.423217	0.3447494	0.1262076	0.08475824	0.106897	0.1399199
2132.556	18	0.409122	0.3430952	0.1247441	0.08746153	0.105466	0.1360047
2132.656	18	0.409551	0.3412554	0.1254751	0.08797114	0.106084	0.1351142
2132.756	18	0.402489	0.3401593	0.1233803	0.08655153	0.105545	0.1347624
2132.856	18	0.408505	0.3434273	0.1212313	0.0866968	0.106214	0.1354632
2132.956	18	0.400235	0.3424282	0.120406	0.08563238	0.107575	0.1334773
2133.056	18	0.406073	0.3443554	0.118685	0.08399522	0.107447	0.1326117
2133.156	18	0.425907	0.3440793	0.1180842	0.08224766	0.107053	0.1329776
2133.256	18	0.42547	0.3217056	0.1190158	0.08278251	0.10831	0.1341188
2133.356	18	0.434068	0.337823	0.1221101	0.08715446	0.11189	0.1358014
2133.456	18	0.415474	0.3276813	0.1217625	0.08707564	0.114236	0.1355098
2133.556	18	0.411747	0.3412964	0.1226306	0.09223064	0.117853	0.1361544
2133.656	18	0.437075	0.3282677	0.1206202	0.09431493	0.120697	0.1357573
2133.756	18	0.433463	0.3402686	0.1275303	0.09737808	0.116534	0.1339419
2133.856	18	0.42147	0.3454023	0.1317619	0.09747934	0.112981	0.1359619
2133.956	18	0.415606	0.3431422	0.1340118	0.09427904	0.114707	0.1362888
2134.056	18	0.421086	0.3503573	0.1327519	0.08872459	0.117258	0.1349996
2134.156	18	0.433538	0.3468331	0.1308094	0.08663483	0.119656	0.1333548
2134.256	18	0.440714	0.3609353	0.127184	0.08913171	0.116766	0.1284415
2134.356	18	0.414222	0.3535326	0.1298078	0.08731553	0.113645	0.128249
2134.456	18	0.404566	0.3458461	0.1289789	0.08705027	0.112939	0.1286672
2134.556	18	0.429286	0.3470519	0.1252465	0.08634441	0.112462	0.1301737
2134.656	18	0.433091	0.3522326	0.1267799	0.08693884	0.11366	0.1295192
2134.756	18	0.441906	0.3562315	0.1241521	0.08829682	0.113712	0.1288356
2134.856	18	0.441019	0.3509413	0.1249725	0.08759031	0.116353	0.1277696
2134.956	18	0.428682	0.3562095	0.1244814	0.08606248	0.117944	0.1275986
2135.056	18	0.41203	0.3508004	0.1235439	0.08483419	0.117181	0.1278959
2135.156	18	0.419896	0.3506553	0.1221149	0.08041399	0.113241	0.1285261
2135.256	18	0.423579	0.3574139	0.1196283	0.07917222	0.112425	0.1279344
2135.356	18	0.423223	0.3696017	0.1189184	0.07825819	0.112341	0.1300851
2135.456	18	0.437559	0.367981	0.1194415	0.07655532	0.110831	0.1337666
2135.556	18	0.435697	0.3613084	0.1190763	0.07816233	0.107079	0.1350117
2135.656	18	0.44084	0.3605193	0.1229191	0.08082059	0.105925	0.1366288
2135.756	18	0.434369	0.3535445	0.1231971	0.08272441	0.106463	0.1383078
2135.856	18	0.411507	0.3484561	0.1236033	0.08569384	0.107118	0.1409421
2135.956	18	0.405587	0.355333	0.1228898	0.08673958	0.108079	0.1417029
2136.056	18	0.40198	0.3506214	0.121643	0.08718681	0.106934	0.1430674
2136.156	18	0.403342	0.3487339	0.1180216	0.08926489	0.106534	0.1430082

2136.256	18	0.402476	0.3492513	0.1168371	0.09052762	0.105352	0.1390662
2136.356	18	0.4032	0.3481916	0.118973	0.08991512	0.104942	0.1396485
2136.456	18	0.412378	0.3452563	0.1197985	0.08937755	0.105653	0.1388256
2136.556	18	0.423814	0.3527556	0.1193628	0.09060474	0.10819	0.137902
2136.656	18	0.422214	0.3532358	0.1178167	0.08892991	0.111615	0.1348636
2136.756	18	0.417733	0.3538836	0.1201071	0.08984163	0.113826	0.1344039
2136.856	18	0.413985	0.3465357	0.1201787	0.09092402	0.111321	0.1331614
2136.956	18	0.421678	0.3399067	0.1211109	0.09263591	0.114303	0.1343114
2137.056	18	0.432671	0.3415416	0.120811	0.0944986	0.117574	0.1334277
2137.156	18	0.423571	0.3492839	0.1217364	0.09693491	0.116589	0.1344783
2137.256	18	0.429783	0.3451831	0.1172782	0.09490369	0.11844	0.1342672
2137.356	18	0.432519	0.344832	0.1171655	0.09083254	0.117645	0.1343855
2137.456	18	0.431483	0.346306	0.1240577	0.08759766	0.118112	0.1326606
2137.556	18	0.452244	0.3452642	0.1263148	0.08501946	0.115326	0.1324559
2137.656	18	0.442769	0.3526006	0.1267705	0.084824	0.116191	0.1330428
2137.756	18	0.430172	0.3514315	0.1279734	0.08526444	0.11309	0.1337902
2137.856	18	0.433886	0.3538525	0.1248963	0.08448783	0.111907	0.1313594
2137.956	18	0.436661	0.3527489	0.1250536	0.08682166	0.111876	0.1320089
2138.056	18	0.450147	0.3410398	0.1260911	0.0842033	0.111201	0.133879
2138.156	18	0.450677	0.3406318	0.123571	0.08774123	0.113781	0.1327294
2138.256	18	0.45078	0.3536554	0.1241916	0.08665431	0.114146	0.1304319
2138.356	18	0.440727	0.3627176	0.1232056	0.08699195	0.113673	0.1303714
2138.456	18	0.468393	0.3454304	0.1239744	0.08690728	0.113203	0.1282989
2138.556	18	0.450452	0.3484285	0.1208653	0.08547845	0.109816	0.1293625
2138.656	18	0.480195	0.3470218	0.1203807	0.08461953	0.111139	0.1302745
2138.756	18	0.470389	0.3468368	0.122567	0.0816417	0.109569	0.1333511
2138.856	18	0.478427	0.3444307	0.1217644	0.08107818	0.107576	0.1343962
2138.956	18	0.470782	0.3426398	0.1236427	0.08248948	0.107087	0.1329245
2139.056	18	0.451011	0.345673	0.1245962	0.07976848	0.105387	0.1355722
2139.156	18	0.432395	0.349764	0.1240353	0.08145603	0.104563	0.1364044
2139.256	18	0.414416	0.3458395	0.1259346	0.08388631	0.102264	0.1377678
2139.356	18	0.414916	0.3494749	0.1277156	0.083757	0.104259	0.1400397
2139.456	18	0.414381	0.3409307	0.1267274	0.08588493	0.106792	0.141319
2139.556	18	0.413133	0.3411666	0.1245589	0.08535439	0.107035	0.1401171
2139.656	18	0.408386	0.349447	0.1248035	0.08852286	0.107246	0.1423375
2139.756	18	0.402789	0.335367	0.1250008	0.09160163	0.109953	0.1442789
2139.856	18	0.429114	0.3418448	0.1244186	0.08682753	0.111091	0.1403726
2139.956	18	0.429161	0.3439336	0.1229875	0.08769661	0.114788	0.1390158
2140.056	18	0.437672	0.3400254	0.1214581	0.08911483	0.113109	0.1364606
2140.156	18	0.43035	0.3457594	0.1200424	0.09150644	0.110284	0.1396498
2140.256	18	0.445461	0.3321108	0.1172363	0.09353971	0.11063	0.1407037
2140.356	18	0.467994	0.3284446	0.1166627	0.09504221	0.112165	0.1365634
2140.456	18	0.46367	0.3398983	0.1171905	0.0959712	0.115398	0.136806
2140.556	18	0.458473	0.3284754	0.1172939	0.0969215	0.117863	0.1361406
2140.656	18	0.461294	0.3357404	0.1201187	0.09193037	0.121467	0.1369404
2140.756	18	0.450099	0.3261012	0.1178125	0.08855046	0.116779	0.1366831
2140.856	18	0.459392	0.3393859	0.1197661	0.08528586	0.114002	0.1347111
2140.956	18	0.456698	0.3447932	0.1193458	0.08375259	0.115932	0.1347814
2141.056	18	0.427427	0.3426815	0.1243889	0.08464801	0.116987	0.1350229
2141.156	18	0.427757	0.3483386	0.1261605	0.08457726	0.118444	0.1385768
2141.256	18	0.444356	0.3538841	0.125541	0.08563708	0.117407	0.1370615
2141.356	18	0.445815	0.3528442	0.1248195	0.08372207	0.115439	0.138693
2141.456	18	0.455623	0.3496474	0.1235497	0.08535568	0.114072	0.1347567
2141.556	18	0.455506	0.3413242	0.1247045	0.08476219	0.115149	0.1357135
2141.656	18	0.445824	0.3445571	0.1272074	0.08276235	0.117294	0.1368599
2141.756	18	0.454769	0.3552981	0.1260819	0.08113308	0.116813	0.1354846
2141.856	18	0.451452	0.3542093	0.1248354	0.08276325	0.113743	0.1345745
2141.956	18	0.452657	0.3516166	0.1220102	0.08224516	0.115508	0.1324044
2142.056	18	0.453209	0.3526798	0.1217294	0.08515681	0.114337	0.1313794
2142.156	18	0.466057	0.3465381	0.1218709	0.08474869	0.111059	0.1326756
2142.256	18	0.43257	0.35093	0.1220119	0.0860475	0.109068	0.1324703
2142.356	18	0.411018	0.364611	0.1241221	0.08581184	0.105343	0.1342932
2142.456	18	0.416675	0.3698203	0.123762	0.08577316	0.106737	0.1358248
2142.556	18	0.40563	0.3667526	0.1216235	0.08768242	0.105861	0.132681
2142.656	18	0.408648	0.3565776	0.1245257	0.08933832	0.105707	0.1310293
2142.756	18	0.402942	0.3547476	0.1255339	0.08840286	0.105987	0.1325222
2142.856	18	0.411365	0.3487173	0.1266594	0.08780952	0.10816	0.1340512
2142.956	18	0.399472	0.3474548	0.131888	0.08779614	0.109512	0.1345652
2143.056	18	0.401981	0.3571672	0.1300693	0.08737285	0.110664	0.1363408
2143.156	18	0.414416	0.3496746	0.125953	0.08657785	0.109194	0.1386572
2143.256	18	0.426899	0.345993	0.1257842	0.08826328	0.112859	0.1391644
2143.356	18	0.439739	0.3508096	0.1241148	0.08896252	0.11495	0.1437968
2143.456	18	0.430401	0.3447949	0.1212533	0.08852074	0.113063	0.1424352
2143.556	18	0.426855	0.349257	0.1197289	0.09103739	0.114093	0.1378185
2143.656	18	0.440335	0.3516754	0.1179862	0.09227706	0.11463	0.1371636
2143.756	18	0.434175	0.3549343	0.119077	0.09549629	0.111918	0.1375785
2143.856	18	0.431264	0.3503776	0.1169248	0.09777635	0.115356	0.1382431
2143.956	18	0.420793	0.3401794	0.1167088	0.09625634	0.119241	0.1370941
2144.056	18	0.426916	0.3409623	0.1170526	0.08967385	0.119658	0.1394818
2144.156	18	0.435772	0.3441478	0.1178588	0.08698121	0.117046	0.1387847
2144.256	18	0.444136	0.3460595	0.1172809	0.08738539	0.112868	0.1374391
2144.356	18	0.420983	0.3458153	0.1170298	0.08637887	0.114927	0.1349494
2144.456	18	0.404305	0.344284	0.1178695	0.08559606	0.11984	0.1350665
2144.556	18	0.434747	0.343054	0.1179296	0.08304689	0.119478	0.1318658
2144.656	18	0.42956	0.3575386	0.1201634	0.08545318	0.117078	0.1286029
2144.756	18	0.434192	0.3422369	0.1246207	0.08576957	0.116708	0.1300384
2144.856	18	0.454373	0.3502619	0.1256183	0.08639675	0.11574	0.1330662
2144.956	18	0.442861	0.3519877	0.1251208	0.08561817	0.116271	0.1327302
2145.056	18	0.434199	0.3463026	0.1282888	0.08527195	0.116799	0.1319467
2145.156	18	0.435315	0.3362425	0.1267939	0.08586267	0.115044	0.1319783
2145.256	18	0.434175	0.344931	0.1304464	0.08410775	0.11239	0.1320465
2145.356	18	0.432437	0.3564338	0.1267165	0.08304863	0.113826	0.1337873
2145.456	18	0.447046	0.3563915	0.1237352	0.0831513	0.113329	0.1307358
2145.556	18	0.438562	0.3431539	0.1239973	0.08477471	0.111497	0.131735
2145.656	18	0.441449	0.3538397	0.1211799	0.08715793	0.108248	0.13207

2145.756	18	0.43338	0.343652	0.1229004	0.08737084	0.109694	0.131257
2145.856	18	0.412316	0.3443139	0.1251884	0.08821876	0.109839	0.1321931
2145.956	18	0.405874	0.3416788	0.1270665	0.08906247	0.111918	0.1327166
2146.056	18	0.406624	0.3421279	0.1258953	0.08963884	0.109962	0.1331716
2146.156	18	0.404216	0.3476429	0.124625	0.08864418	0.110807	0.1341657
2146.256	18	0.400412	0.3463019	0.122865	0.09105638	0.111121	0.1348632
2146.356	18	0.39823	0.3487675	0.1230118	0.08967977	0.107644	0.1325142
2146.456	18	0.412308	0.343161	0.120565	0.09037142	0.109807	0.1318029
2146.556	18	0.429207	0.3375315	0.124445	0.09042025	0.110592	0.1341153
2146.656	18	0.420353	0.3391716	0.1239867	0.09106229	0.114037	0.133957
2146.756	18	0.416444	0.3412372	0.1263953	0.09080656	0.114892	0.1374191
2146.856	18	0.412956	0.3376614	0.1238724	0.09174877	0.112987	0.1388513
2146.956	18	0.421748	0.3380993	0.1249933	0.09103806	0.113465	0.1422285
2147.056	18	0.437573	0.3402367	0.1244709	0.08947676	0.114007	0.1415556
2147.156	18	0.429558	0.3423289	0.1239186	0.08609092	0.115301	0.1393311
2147.256	18	0.430962	0.3425608	0.1239583	0.08958339	0.117126	0.1382102
2147.356	18	0.426623	0.3232586	0.1217405	0.08768747	0.119674	0.1369147
2147.456	18	0.432603	0.3377087	0.120916	0.08616973	0.118498	0.1373293
2147.556	18	0.453421	0.328187	0.1213107	0.08410647	0.116083	0.1388763
2147.656	18	0.438582	0.3372087	0.1204239	0.08303215	0.116482	0.1375061
2147.756	18	0.426592	0.3268472	0.1215967	0.08461495	0.119076	0.1374662
2147.856	18	0.436225	0.3341528	0.1224772	0.08385479	0.118902	0.1344118
2147.956	18	0.449539	0.3399215	0.1214289	0.08338808	0.117063	0.1354816
2148.056	18	0.454214	0.3409477	0.121431	0.08400319	0.115537	0.1366906
2148.156	18	0.452205	0.3451973	0.1216569	0.08406533	0.116401	0.1330378
2148.256	18	0.451347	0.3460273	0.1217312	0.08324903	0.115766	0.1290817
2148.356	18	0.429271	0.3605173	0.1227827	0.0840323	0.114637	0.128848
2148.456	18	0.453937	0.3472053	0.1258511	0.08234864	0.114903	0.131755
2148.556	18	0.440678	0.3440107	0.1276699	0.08308912	0.112658	0.1303554
2148.656	18	0.46621	0.343945	0.1311557	0.07961732	0.112343	0.1287242
2148.756	18	0.468613	0.3469835	0.1285144	0.08015853	0.111308	0.1283752
2148.856	18	0.465386	0.3534037	0.1284474	0.0798326	0.107533	0.1278421
2148.956	18	0.463009	0.3474039	0.1282158	0.08193836	0.107236	0.1284208
2149.056	18	0.438443	0.3554043	0.1246014	0.08466358	0.105667	0.1277098
2149.156	18	0.427578	0.3481635	0.121806	0.08731382	0.105233	0.1282819
2149.256	18	0.414969	0.3484356	0.1177881	0.08818448	0.101657	0.1284526
2149.356	18	0.414319	0.3535131	0.1182064	0.08704674	0.103391	0.128761
2149.456	18	0.411087	0.3651153	0.1177451	0.08796181	0.102821	0.1278328
2149.556	18	0.413993	0.3661818	0.1237244	0.08784346	0.106353	0.1295459
2149.656	18	0.402743	0.3596061	0.1262867	0.09222328	0.105261	0.1312223
2149.756	18	0.410749	0.358572	0.1250299	0.09431255	0.104043	0.1328638
2149.856	18	0.429616	0.3514383	0.1254207	0.09393393	0.103788	0.1336627
2149.956	18	0.439819	0.3489663	0.1270507	0.09212857	0.110322	0.134065
2150.056	18	0.443626	0.3494749	0.1268937	0.09301408	0.114059	0.1329645
2150.156	18	0.437164	0.3495755	0.1271058	0.09522772	0.112821	0.1339207
2150.256	18	0.43562	0.3474358	0.1278981	0.09490592	0.111921	0.1365254
2150.356	18	0.461515	0.3456401	0.1271404	0.09315844	0.11463	0.1405351
2150.456	18	0.463347	0.3482876	0.1260463	0.09318656	0.117549	0.1417809
2150.556	18	0.457515	0.3426684	0.127291	0.09431653	0.12159	0.1427599
2150.656	18	0.472163	0.3468906	0.1246416	0.0943192	0.123151	0.1416375
2150.756	18	0.462841	0.351184	0.1256001	0.09147119	0.118068	0.1384774
2150.856	18	0.467336	0.3567868	0.1262248	0.08942994	0.11741	0.1384342
2150.956	18	0.458825	0.3458693	0.1277904	0.0886827	0.11584	0.1375835
2151.056	18	0.429575	0.3382973	0.1258018	0.08751547	0.113118	0.1374679
2151.156	18	0.433809	0.3403628	0.1240872	0.08853205	0.114508	0.1374159
2151.256	18	0.44403	0.3464389	0.1214324	0.09076642	0.111967	0.1367043
2151.356	18	0.437061	0.3441748	0.120298	0.09157264	0.110943	0.1376149
2151.456	18	0.446386	0.3449248	0.1207428	0.09090065	0.111223	0.1373401
2151.556	18	0.448387	0.3446035	0.1202432	0.09250419	0.112086	0.1363829
2151.656	18	0.43674	0.3415288	0.1204845	0.09266084	0.112649	0.1366923
2151.756	18	0.445002	0.3547336	0.1240548	0.09123401	0.11509	0.138004
2151.856	18	0.445027	0.3481653	0.1228064	0.08854824	0.113044	0.1365644
2151.956	18	0.444318	0.351238	0.1277257	0.08461441	0.110596	0.1376403
2152.056	18	0.442287	0.3513989	0.1312056	0.08445502	0.112571	0.1378206
2152.156	18	0.43541	0.341558	0.1319408	0.08306661	0.109757	0.1370963
2152.256	18	0.417173	0.3387513	0.1335549	0.08465655	0.108048	0.1374608
2152.356	18	0.414882	0.3477083	0.1288274	0.08670485	0.106737	0.1367066
2152.456	18	0.417663	0.3616382	0.1261232	0.08792651	0.10599	0.1363656
2152.556	18	0.405193	0.3489677	0.1272451	0.08818927	0.106146	0.136506
2152.656	18	0.407902	0.3461482	0.1248784	0.08802485	0.106985	0.1363671
2152.756	18	0.400033	0.3472177	0.1225594	0.08827699	0.107958	0.1349232
2152.856	18	0.410371	0.3429912	0.1214508	0.08806798	0.108273	0.1312578
2152.956	18	0.400643	0.3443507	0.1212098	0.09107552	0.109253	0.1303051
2153.056	18	0.403443	0.3424735	0.1205634	0.09191068	0.106624	0.131333
2153.156	18	0.420371	0.3443588	0.1209239	0.09267388	0.106372	0.1318661
2153.256	18	0.433998	0.3492839	0.1232444	0.09377103	0.108858	0.1316384
2153.356	18	0.442352	0.349472	0.12103	0.09267601	0.11223	0.131303
2153.456	18	0.42891	0.3478893	0.1226262	0.09102155	0.112786	0.1347614
2153.556	18	0.427264	0.3411258	0.1205828	0.09125895	0.112279	0.1373881
2153.656	18	0.431668	0.3435888	0.1205311	0.0927516	0.114208	0.1394183
2153.756	18	0.432226	0.3458083	0.120625	0.09803177	0.11641	0.1394019
2153.856	18	0.426283	0.3355628	0.1257535	0.09698825	0.11988	0.137033
2153.956	18	0.416256	0.3397466	0.1246592	0.0952419	0.12464	0.1404374
2154.056	18	0.426162	0.3396953	0.1246854	0.09176635	0.124112	0.1415234
2154.156	18	0.4418	0.3401059	0.1259723	0.09031206	0.121181	0.1445558
2154.256	18	0.448347	0.3424249	0.1219079	0.09030098	0.117045	0.1459827
2154.356	18	0.426491	0.3334259	0.1203098	0.08724099	0.117802	0.1427411
2154.456	18	0.415779	0.3235586	0.1209728	0.08805586	0.119393	0.1410994
2154.556	18	0.435454	0.3368007	0.1210374	0.08705152	0.119422	0.1387174
2154.656	18	0.433211	0.3257126	0.1228385	0.08761688	0.115917	0.1387653
2154.756	18	0.431095	0.3348491	0.1204307	0.08859754	0.114174	0.1394741
2154.856	18	0.443058	0.3237929	0.119851	0.08906274	0.113371	0.1364231
2154.956	18	0.434514	0.3368748	0.1196064	0.0887778	0.116686	0.1383688
2155.056	18	0.417138	0.3416122	0.1187162	0.08981637	0.119783	0.137586
2155.156	18	0.424564	0.3384974	0.1209746	0.08914412	0.120383	0.1337579

2155.256	18	0.432986	0.3470767	0.12056	0.08757915	0.117442	0.1312624
2155.356	18	0.433729	0.3468865	0.1209317	0.08833209	0.115934	0.1302957
2155.456	18	0.444447	0.3544242	0.1211032	0.0860581	0.11322	0.1308173
2155.556	18	0.43775	0.3488774	0.123043	0.08592249	0.109547	0.1302571
2155.656	18	0.429466	0.3403783	0.1268028	0.08592161	0.107914	0.1300968
2155.756	18	0.419973	0.3422025	0.1273434	0.08653037	0.106665	0.1307622
2155.856	18	0.411782	0.3501886	0.1279153	0.08741301	0.104649	0.1315628
2155.956	18	0.401548	0.351887	0.1289592	0.08863334	0.106298	0.1305241
2156.056	18	0.400779	0.3487842	0.1240377	0.08642182	0.10756	0.130157
2156.156	18	0.401334	0.3494129	0.1249312	0.08725444	0.107051	0.1324972
2156.256	18	0.404536	0.3464554	0.1248978	0.08687246	0.108462	0.1343451
2156.356	18	0.400741	0.3502208	0.1194024	0.08895078	0.10704	0.1308896
2156.456	18	0.418882	0.3566097	0.1192494	0.08789887	0.105868	0.1301285
2156.556	18	0.430054	0.3685991	0.1154006	0.08684908	0.10638	0.131423
2156.656	18	0.435339	0.3646002	0.1186774	0.08681329	0.110377	0.128681
2156.756	18	0.440048	0.356738	0.1218651	0.08916127	0.110133	0.1318237
2156.856	18	0.429159	0.3557418	0.1237335	0.09203858	0.110736	0.1323905
2156.956	18	0.443213	0.3497346	0.1249595	0.09335145	0.111089	0.1309329
2157.056	18	0.4497	0.3458592	0.1258916	0.09309831	0.111008	0.1284484
2157.156	18	0.443882	0.3551592	0.1249553	0.09640446	0.11121	0.1301198
2157.256	18	0.454933	0.3474305	0.1235937	0.09639119	0.117198	0.1348262
2157.356	18	0.445023	0.3429193	0.1232753	0.09331036	0.121336	0.1340172
2157.456	18	0.448897	0.349147	0.1261688	0.0905625	0.120794	0.1377535
2157.556	18	0.466535	0.3468365	0.1258054	0.0887491	0.116489	0.1386566
2157.656	18	0.438907	0.3491742	0.1261497	0.08735595	0.114809	0.1394819
2157.756	18	0.434636	0.3515181	0.1248383	0.08594744	0.113212	0.1441008
2157.856	18	0.443342	0.3554733	0.1246852	0.08461299	0.112114	0.1451859
2157.956	18	0.447934	0.3531731	0.1231006	0.08528572	0.112028	0.1436721
2158.056	18	0.447533	0.3437332	0.1203323	0.08430776	0.111115	0.1390206
2158.156	18	0.443796	0.3390246	0.1207877	0.08495775	0.109844	0.1355746
2158.267	18	0.463016	0.340342	0.1201727	0.08549035	0.108956	0.1325236
2158.367	18.5	0.433845	0.3472454	0.1200545	0.08618071	0.111126	0.1350084
2158.467	18.5	0.467916	0.3457804	0.1183136	0.08373515	0.114378	0.1315978
2158.567	18.5	0.4753	0.3454224	0.1164597	0.08624946	0.113882	0.1313176
2158.667	18.5	0.47737	0.3469772	0.1139177	0.08431166	0.113162	0.131557
2158.767	18.5	0.473824	0.348705	0.1117518	0.08461571	0.111096	0.1308202
2158.867	18.5	0.452493	0.3480886	0.1145455	0.08327324	0.106864	0.1292841
2158.967	18.5	0.423291	0.3514168	0.1208542	0.08642662	0.103069	0.1316582
2159.067	18.5	0.422458	0.3495418	0.1251857	0.08678841	0.10215	0.1306756
2159.167	18.5	0.4232	0.3491063	0.1272267	0.08617123	0.102684	0.1317442
2159.267	18.5	0.417866	0.3397298	0.1308562	0.08521532	0.101553	0.1329617
2159.367	18.5	0.412996	0.3405283	0.1317656	0.08591968	0.102156	0.1369151
2159.467	18.5	0.407454	0.3524237	0.1331755	0.08767068	0.103625	0.1365629
2159.567	18.5	0.414806	0.3612078	0.1262979	0.09057919	0.10493	0.1355604
2159.667	18.5	0.430801	0.3442039	0.129014	0.08847929	0.104925	0.1331484
2159.767	18.5	0.437837	0.3494812	0.1233803	0.08833044	0.103448	0.1331702
2159.867	18.5	0.438304	0.347115	0.1230682	0.08686395	0.105729	0.1363137
2159.967	18.5	0.430377	0.346189	0.1187014	0.08866879	0.110307	0.1337073
2160.067	18.5	0.44072	0.3416719	0.1186156	0.08914335	0.110787	0.133688
2160.167	18.5	0.458812	0.3394778	0.1190789	0.0917453	0.11024	0.1330012
2160.267	18.5	0.4504	0.3470372	0.1222255	0.09070229	0.111739	0.131614
2160.367	18.5	0.437838	0.3490928	0.1239082	0.09222905	0.112467	0.1302831
2160.467	18.5	0.438984	0.3423855	0.1267709	0.09095506	0.112951	0.1289856
2160.567	18.5	0.44923	0.3477687	0.1282351	0.08987661	0.120182	0.1292596
2160.667	18.5	0.448178	0.3396197	0.1289755	0.09042756	0.124403	0.1257395
2160.767	18.5	0.421773	0.3394597	0.1270164	0.09139702	0.123071	0.1257123
2160.867	18.5	0.424839	0.3451559	0.1293014	0.09156747	0.119935	0.1294587
2160.967	18.5	0.438994	0.3359184	0.1266774	0.09140161	0.118698	0.1316951
2161.067	18.5	0.433913	0.3411007	0.1234631	0.09293877	0.117305	0.1315878
2161.167	18.5	0.440598	0.3414755	0.1205858	0.09229001	0.115463	0.1316705
2161.267	18.5	0.450795	0.3366383	0.1212005	0.09251254	0.116238	0.1342036
2161.367	18.5	0.433351	0.3432898	0.1204328	0.09310007	0.114246	0.1392184
2161.467	18.5	0.437239	0.3225713	0.1177922	0.09260189	0.111137	0.1447342
2161.567	18.5	0.434889	0.3275712	0.1186266	0.09099675	0.110229	0.1475831
2161.667	18.5	0.430061	0.3320159	0.118899	0.09127767	0.111837	0.1446811
2161.767	18.5	0.429228	0.3315885	0.116905	0.08832155	0.114171	0.1415236
2161.867	18.5	0.429476	0.3292251	0.1147114	0.08432607	0.114698	0.1412204
2161.967	18.5	0.413776	0.328119	0.1148072	0.0858814	0.116108	0.1410618
2162.067	18.5	0.405267	0.334774	0.1147391	0.08577195	0.113456	0.1398202
2162.167	18.5	0.412178	0.341796	0.1173938	0.08970931	0.109701	0.1388455
2162.267	18.5	0.404053	0.3425505	0.1174684	0.09141459	0.107913	0.1376109
2162.367	18.5	0.407057	0.3437899	0.1220346	0.09007946	0.107078	0.1380959
2162.467	18.5	0.400831	0.3596046	0.1235637	0.08912989	0.107129	0.1390304
2162.567	18.5	0.406082	0.3449718	0.1244392	0.08778369	0.108303	0.1380602
2162.667	18.5	0.397831	0.3453232	0.1274656	0.08854812	0.107627	0.1403934
2162.767	18.5	0.407966	0.3410454	0.1295543	0.09207865	0.108808	0.1404932
2162.867	18.5	0.424727	0.3436923	0.134189	0.09192996	0.109836	0.1400582
2162.967	18.5	0.427506	0.3531018	0.1276563	0.09029225	0.111001	0.138887
2163.067	18.5	0.439666	0.3488678	0.1250389	0.08956149	0.111329	0.1385889
2163.167	18.5	0.435223	0.3524352	0.12754	0.09127735	0.11176	0.1381114
2163.267	18.5	0.439262	0.3484482	0.1256433	0.09227621	0.111904	0.1363871
2163.367	18.5	0.44483	0.3434988	0.123613	0.09198506	0.113675	0.133707
2163.467	18.5	0.432543	0.3507778	0.1218207	0.09520513	0.114942	0.1337557
2163.567	18.5	0.448844	0.3635542	0.1219357	0.09659202	0.11493	0.1339682
2163.667	18.5	0.441572	0.3655226	0.1200369	0.09283611	0.117878	0.1320144
2163.767	18.5	0.446554	0.3638379	0.1210788	0.09161114	0.118098	0.1326197
2163.867	18.5	0.454404	0.3548088	0.1241561	0.09239484	0.118871	0.1329996
2163.967	18.5	0.434806	0.3540989	0.1251053	0.09361498	0.120893	0.1312766
2164.067	18.5	0.424454	0.346516	0.1265668	0.09239247	0.121737	0.1324829
2164.167	18.5	0.432748	0.3464781	0.129153	0.08941306	0.118912	0.1323914
2164.267	18.5	0.442795	0.3544145	0.129628	0.08935839	0.114748	0.1312311
2164.367	18.5	0.444479	0.3468216	0.1313231	0.09164985	0.114606	0.1294719
2164.467	18.5	0.446882	0.3475949	0.1304476	0.09185109	0.115798	0.1287057
2164.567	18.5	0.448943	0.3488495	0.1316991	0.09080627	0.117296	0.1343222
2164.667	18.5	0.432941	0.3457696	0.127923	0.09160276	0.115324	0.1343419

2164.767	18.5	0.450985	0.348097	0.1284658	0.0880487	0.110408	0.1358266
2164.867	18.5	0.442005	0.3527099	0.125069	0.09081897	0.111342	0.1375059
2164.967	18.5	0.464961	0.3545496	0.1227542	0.08833137	0.11071	0.1452753
2165.067	18.5	0.469332	0.3475211	0.1219938	0.08788441	0.111181	0.1465066
2165.167	18.5	0.473315	0.3381906	0.1218604	0.08544914	0.113686	0.1470638
2165.267	18.5	0.460755	0.3401827	0.1198424	0.0860671	0.112507	0.1448692
2165.367	18.5	0.447179	0.3446918	0.1157253	0.08696286	0.111776	0.1425583
2165.467	18.5	0.419001	0.3442262	0.1128947	0.08765968	0.110022	0.1417229
2165.567	18.5	0.42368	0.3468173	0.1134306	0.08754656	0.107846	0.1417363
2165.667	18.5	0.412251	0.3440925	0.1138848	0.08851349	0.1079	0.1426519
2165.767	18.5	0.416168	0.3410784	0.1130929	0.08714172	0.103874	0.1412404
2165.867	18.5	0.414604	0.3579752	0.1151514	0.0872046	0.102814	0.142038
2165.967	18.5	0.408577	0.3462618	0.121194	0.08888744	0.103773	0.1397889
2166.067	18.5	0.417818	0.3480237	0.1244249	0.08885531	0.104626	0.1402568
2166.167	18.5	0.440124	0.3492153	0.1312733	0.08491154	0.105277	0.1397098
2166.267	18.5	0.440766	0.3437748	0.1319908	0.08725255	0.106489	0.1394394
2166.367	18.5	0.435948	0.3367245	0.136842	0.09068593	0.105621	0.1377501
2166.467	18.5	0.43688	0.3435337	0.1343467	0.0950817	0.104757	0.1325478
2166.567	18.5	0.444953	0.3561454	0.1265045	0.09804067	0.106309	0.1330886
2166.667	18.5	0.44451	0.3513443	0.1259465	0.1016201	0.108612	0.1348965
2166.767	18.5	0.450886	0.3424714	0.1230215	0.1001723	0.107144	0.1353146
2166.867	18.5	0.434644	0.34925	0.1201567	0.0975145	0.103886	0.1357755
2166.967	18.5	0.443467	0.3434207	0.1206277	0.09266827	0.107791	0.1336796
2167.067	18.5	0.450482	0.3427474	0.1189616	0.09022053	0.113085	0.1339417
2167.167	18.5	0.453288	0.3400553	0.1205003	0.09246504	0.11087	0.1339094
2167.267	18.5	0.42538	0.3418847	0.1215609	0.09058335	0.116129	0.1344959
2167.367	18.5	0.441285	0.345932	0.1234195	0.0902374	0.120618	0.1343335
2167.467	18.5	0.441384	0.3420543	0.1250598	0.09107345	0.121386	0.1334284
2167.567	18.5	0.439568	0.3470871	0.1242384	0.08904603	0.119549	0.1340187
2167.667	18.5	0.445335	0.3390253	0.1247647	0.08992737	0.117696	0.1341254
2167.767	18.5	0.448114	0.3403507	0.1238524	0.08934499	0.116647	0.1328558
2167.867	18.5	0.438734	0.3407886	0.1242274	0.08901881	0.116493	0.134017
2167.967	18.5	0.450934	0.3369713	0.1274082	0.08966591	0.115648	0.1349171
2168.067	18.5	0.439747	0.339478	0.1249276	0.08491022	0.117714	0.1334471
2168.167	18.5	0.439432	0.3400428	0.124354	0.08139629	0.114692	0.1344495
2168.267	18.5	0.439342	0.3350005	0.1243776	0.08297224	0.116775	0.1354064
2168.367	18.5	0.438696	0.3392785	0.1214837	0.08077846	0.118077	0.1369555
2168.467	18.5	0.42509	0.3379531	0.1188193	0.08449634	0.117995	0.1362771
2168.567	18.5	0.41239	0.3207282	0.117865	0.08585312	0.112892	0.1399216
2168.667	18.5	0.4131	0.3327907	0.1170819	0.08666921	0.112048	0.1409725
2168.767	18.5	0.40821	0.3240342	0.1183759	0.08658148	0.112063	0.1410026
2168.867	18.5	0.411787	0.3353422	0.1176406	0.08678474	0.110751	0.1440144
2168.967	18.5	0.408333	0.3223	0.115428	0.08459833	0.11166	0.1417358
2169.067	18.5	0.415971	0.3353586	0.1169757	0.08486784	0.109828	0.139082
2169.167	18.5	0.402329	0.3398392	0.1186691	0.08639933	0.110976	0.1382111
2169.267	18.5	0.418063	0.3392223	0.1182451	0.08865535	0.112754	0.1387242
2169.367	18.5	0.436472	0.3457516	0.1178847	0.08622723	0.112689	0.1375316
2169.467	18.5	0.442076	0.34699	0.1215393	0.08363278	0.109598	0.1360737
2169.567	18.5	0.443264	0.3565376	0.1261448	0.08451583	0.109254	0.1319746
2169.667	18.5	0.431495	0.350341	0.1290237	0.08756285	0.10966	0.1319728
2169.767	18.5	0.434862	0.3422289	0.1332757	0.0914609	0.110993	0.1349112
2169.867	18.5	0.435921	0.3455802	0.1367969	0.09374883	0.112641	0.1350393
2169.967	18.5	0.435141	0.3525952	0.135589	0.09717716	0.115368	0.1344936
2170.067	18.5	0.445397	0.3537417	0.1278444	0.1004481	0.115171	0.1356492
2170.167	18.5	0.448426	0.3483672	0.1290971	0.09437504	0.114253	0.1348471
2170.267	18.5	0.450347	0.3511986	0.1275191	0.09122037	0.114814	0.1363152
2170.367	18.5	0.460738	0.3489396	0.1247992	0.08989656	0.117085	0.1363052
2170.467	18.5	0.431704	0.3518033	0.1255718	0.09277072	0.120181	0.1343474
2170.567	18.5	0.426325	0.3568021	0.1235218	0.09294459	0.121718	0.1317618
2170.667	18.5	0.447635	0.368527	0.1232401	0.09263939	0.124246	0.1319505
2170.767	18.5	0.428625	0.3643729	0.1247051	0.09070609	0.118263	0.132965
2170.867	18.5	0.43934	0.3564559	0.1270362	0.08957083	0.114233	0.1346467
2170.967	18.5	0.43871	0.3541779	0.128516	0.09109224	0.116469	0.1331492
2171.067	18.5	0.434351	0.3485759	0.1297052	0.08630532	0.118359	0.1320724
2171.167	18.5	0.427748	0.3443601	0.13041	0.08860347	0.117041	0.1321799
2171.267	18.5	0.444203	0.3536844	0.1306245	0.08529225	0.117128	0.1314957
2171.367	18.5	0.433552	0.3478259	0.128857	0.08361584	0.118354	0.1311935
2171.467	18.5	0.46159	0.3431253	0.1317334	0.08404218	0.116346	0.1323779
2171.567	18.5	0.476061	0.3493003	0.129122	0.08537812	0.114462	0.1325951
2171.667	18.5	0.476132	0.3487331	0.1265535	0.08536696	0.114334	0.1338702
2171.767	18.5	0.464815	0.3476868	0.1261061	0.08619945	0.112562	0.1365439
2171.867	18.5	0.455043	0.3498552	0.1223633	0.08643571	0.113742	0.135799
2171.967	18.5	0.427629	0.3517671	0.1194393	0.08681665	0.115316	0.1376308
2172.067	18.5	0.433052	0.3513069	0.1181474	0.08867568	0.114918	0.1383326
2172.167	18.5	0.417257	0.342034	0.1186466	0.08855241	0.113058	0.1395337
2172.267	18.5	0.424476	0.3369676	0.1196925	0.08868214	0.111323	0.1401345
2172.367	18.5	0.412326	0.3386766	0.1176942	0.08960499	0.10877	0.1403614
2172.467	18.5	0.409003	0.3461077	0.116318	0.0922442	0.108996	0.1394094
2172.567	18.5	0.422789	0.3442791	0.1163477	0.09102684	0.105932	0.1399613
2172.667	18.5	0.441326	0.3449014	0.1168856	0.08939832	0.107145	0.13722
2172.767	18.5	0.437263	0.3475038	0.1213626	0.09048571	0.107211	0.1362495
2172.867	18.5	0.430777	0.3473601	0.1218328	0.08951418	0.107808	0.1373461
2172.967	18.5	0.44206	0.349593	0.122944	0.09101022	0.10773	0.1378896
2173.067	18.5	0.462387	0.3498231	0.122744	0.09244999	0.106859	0.1380803
2173.167	18.5	0.461945	0.3465039	0.1243381	0.09246341	0.111717	0.1382379
2173.267	18.5	0.455012	0.3483468	0.1272273	0.09556981	0.111915	0.1364892
2173.367	18.5	0.470661	0.3385986	0.1285899	0.09610716	0.113749	0.1379637
2173.467	18.5	0.462085	0.3394544	0.1330137	0.09434953	0.116058	0.1400415
2173.567	18.5	0.458945	0.350301	0.127256	0.09277624	0.117661	0.1398332
2173.667	18.5	0.456368	0.3584045	0.1248543	0.0954059	0.120603	0.1389116
2173.767	18.5	0.429595	0.3434366	0.1238455	0.09356786	0.116797	0.137944
2173.867	18.5	0.450092	0.3468051	0.1201882	0.09312069	0.115121	0.1381161
2173.967	18.5	0.449457	0.3458759	0.1194068	0.09157758	0.118999	0.1376419
2174.067	18.5	0.453878	0.3455229	0.1179348	0.08683955	0.12336	0.137548
2174.167	18.5	0.455686	0.3406866	0.1188722	0.08676698	0.124199	0.1354486

2174.267	18.5	0.456765	0.3398714	0.1183421	0.0864808	0.124939	0.1311598
2174.367	18.5	0.449118	0.3463199	0.1229595	0.08626898	0.122055	0.1295297
2174.467	18.5	0.464638	0.3464162	0.1244016	0.08810143	0.122295	0.1309263
2174.567	18.5	0.443123	0.3429203	0.1228045	0.0853738	0.122984	0.1315886
2174.667	18.5	0.456985	0.3466824	0.1219345	0.08519454	0.122647	0.1331216
2174.767	18.5	0.469246	0.3387095	0.1236974	0.08190759	0.120903	0.1325206
2174.867	18.5	0.451338	0.3354208	0.1240919	0.08143129	0.116515	0.1317849
2174.967	18.5	0.434285	0.3443582	0.1272366	0.08519419	0.115248	0.1295762
2175.067	18.5	0.425592	0.3344819	0.1271309	0.0869059	0.113723	0.1293746
2175.167	18.5	0.41302	0.3398705	0.1253373	0.0889217	0.111735	0.1310533
2175.267	18.5	0.415045	0.3410327	0.1220352	0.08898859	0.107945	0.1331666
2175.367	18.5	0.420201	0.3331334	0.1214967	0.08807956	0.109788	0.1331529
2175.467	18.5	0.413933	0.3415818	0.1198363	0.08761528	0.108674	0.1390836
2175.567	18.5	0.422096	0.325564	0.1197363	0.08779445	0.106808	0.1412502
2175.667	18.5	0.408524	0.3251271	0.1208587	0.08865472	0.106764	0.1401313
2175.767	18.5	0.422883	0.3363064	0.1183294	0.08902738	0.107124	0.1381772
2175.867	18.5	0.433545	0.3289332	0.1161194	0.09014378	0.107694	0.1375143
2175.967	18.5	0.438526	0.3296285	0.1129817	0.08848414	0.109742	0.1379749
2176.067	18.5	0.438975	0.3228755	0.1122753	0.08924212	0.108917	0.1443847
2176.167	18.5	0.430013	0.334759	0.1140225	0.09323726	0.109819	0.1435956
2176.267	18.5	0.443809	0.3434361	0.1158616	0.09680769	0.110812	0.1409771
2176.367	18.5	0.441398	0.342477	0.1180003	0.09830534	0.111153	0.1370374
2176.467	18.5	0.428075	0.3457108	0.1219384	0.0966162	0.111128	0.1338383
2176.567	18.5	0.42648	0.357226	0.1208049	0.09512247	0.108747	0.1348374
2176.667	18.5	0.431039	0.3510202	0.1205581	0.09297606	0.111887	0.1351459
2176.767	18.5	0.438898	0.3490189	0.1212006	0.09271917	0.110599	0.1361692
2176.867	18.5	0.450505	0.3414901	0.1256179	0.09251228	0.110567	0.1354412
2176.967	18.5	0.423711	0.3459361	0.1310494	0.09214731	0.112341	0.1324507
2177.067	18.5	0.408542	0.3559069	0.1307179	0.09217532	0.116837	0.1340988
2177.167	18.5	0.437954	0.3505249	0.1284322	0.09000819	0.118329	0.1343422
2177.267	18.5	0.445175	0.3522938	0.1284174	0.08959569	0.121394	0.1372697
2177.367	18.5	0.444898	0.351225	0.1240506	0.08835765	0.122767	0.1372734
2177.467	18.5	0.452422	0.344108	0.1229031	0.08910999	0.120662	0.1354345
2177.567	18.5	0.436402	0.3486277	0.1233207	0.08721947	0.119378	0.1327795
2177.667	18.5	0.426005	0.3620748	0.1207836	0.08813373	0.120643	0.1322874
2177.767	18.5	0.426298	0.3659581	0.1203287	0.08528092	0.122074	0.1308545
2177.867	18.5	0.429349	0.3627198	0.1230529	0.08418538	0.120063	0.129004
2177.967	18.5	0.428995	0.3526579	0.1248659	0.08723876	0.123092	0.1290806
2178.067	18.5	0.429397	0.3502461	0.1245138	0.08857336	0.122161	0.1301208
2178.167	18.5	0.418402	0.342823	0.1203568	0.08854361	0.12051	0.1277163
2178.267	18.5	0.413875	0.3437524	0.1204299	0.08823162	0.117642	0.1285055
2178.367	18.5	0.415105	0.352542	0.1208021	0.08953372	0.116926	0.1302554
2178.467	18.5	0.40223	0.3442563	0.1220505	0.09081468	0.116925	0.1330113
2178.567	18.5	0.407342	0.3431422	0.1262688	0.09012884	0.114424	0.1325836
2178.667	18.5	0.40335	0.3476365	0.1256561	0.08748563	0.114252	0.1308557
2178.767	18.5	0.417448	0.3450266	0.1233781	0.08698466	0.115703	0.1306611
2178.867	18.5	0.409506	0.3456352	0.1255839	0.08826914	0.111462	0.1321565
2178.967	18.5	0.408079	0.3503637	0.122435	0.09171844	0.110695	0.1309895
2179.067	18.5	0.421209	0.3498949	0.1227764	0.09275162	0.108412	0.1334599
2179.167	18.5	0.438232	0.3492691	0.1235175	0.09318812	0.108368	0.1340763
2179.267	18.5	0.425476	0.3391263	0.124605	0.09485681	0.108707	0.134107
2179.367	18.5	0.424966	0.33693	0.1250553	0.09861527	0.110251	0.1390427
2179.467	18.5	0.432491	0.3402772	0.1204698	0.09951861	0.110474	0.1418582
2179.567	18.5	0.442847	0.3462122	0.1206958	0.0994378	0.107064	0.1442662
2179.667	18.5	0.451456	0.3467303	0.1212869	0.09928855	0.105326	0.1403021
2179.767	18.5	0.448249	0.3446796	0.1196581	0.09854178	0.106394	0.1380956
2179.867	18.5	0.461782	0.3438637	0.1206251	0.09451651	0.107765	0.1344946
2179.967	18.5	0.453548	0.3562501	0.1209945	0.08786107	0.108419	0.1349683
2180.067	18.5	0.46113	0.3408869	0.1247553	0.08684816	0.111443	0.1342098
2180.167	18.5	0.460438	0.3467337	0.1268975	0.08594283	0.110879	0.1354153
2180.267	18.5	0.430718	0.3481768	0.1315139	0.08592375	0.110423	0.1340961
2180.367	18.5	0.455411	0.3449844	0.1329233	0.08857248	0.112135	0.1289534
2180.467	18.5	0.44572	0.3374596	0.1318261	0.08930492	0.110478	0.1292794
2180.567	18.5	0.452686	0.3431685	0.1315937	0.08922537	0.112267	0.131796
2180.667	18.5	0.450164	0.3519	0.1276	0.08987141	0.115004	0.1319662
2180.767	18.5	0.451929	0.3547798	0.1289474	0.0899805	0.114817	0.1334389
2180.867	18.5	0.444241	0.3412249	0.1286311	0.08944557	0.117177	0.1330301
2180.967	18.5	0.464893	0.3507706	0.1268323	0.08841631	0.119117	0.131181
2181.067	18.5	0.434242	0.342699	0.1262438	0.08663726	0.120746	0.1301512
2181.167	18.5	0.458112	0.3429685	0.1238945	0.08518858	0.119958	0.1300355
2181.267	18.5	0.472593	0.3406812	0.1227253	0.08714811	0.120181	0.1301374
2181.367	18.5	0.481001	0.3409844	0.123554	0.08752929	0.119112	0.1310832
2181.467	18.5	0.477859	0.3478464	0.1251929	0.08665808	0.118823	0.1309197
2181.567	18.5	0.462947	0.3455513	0.1258987	0.0864413	0.114653	0.1316869
2181.667	18.5	0.438156	0.3464368	0.1279196	0.08736576	0.114455	0.134389
2181.767	18.5	0.431556	0.3432468	0.1282275	0.09077675	0.11529	0.1319713
2181.867	18.5	0.425978	0.3362445	0.1314779	0.08914757	0.11415	0.131508
2181.967	18.5	0.421843	0.3372795	0.1313217	0.08721426	0.112507	0.1317951
2182.067	18.5	0.42474	0.3377142	0.1359491	0.08687699	0.111897	0.133255
2182.167	18.5	0.411113	0.3366514	0.1322769	0.08663871	0.11198	0.1353335
2182.267	18.5	0.419079	0.3350795	0.1291107	0.08648459	0.108584	0.1323919
2182.367	18.5	0.434043	0.3372151	0.1298916	0.08600157	0.107718	0.1329266
2182.467	18.5	0.443279	0.3371692	0.1287345	0.08666027	0.108916	0.1381472
2182.567	18.5	0.438973	0.3373181	0.1266152	0.08939234	0.107014	0.1374185
2182.667	18.5	0.445259	0.3211779	0.1251469	0.08988836	0.108594	0.1377262
2182.767	18.5	0.463627	0.3339646	0.1232499	0.09517954	0.10798	0.1404521
2182.867	18.5	0.473477	0.3289154	0.1216565	0.09720826	0.106476	0.1431503
2182.967	18.5	0.448581	0.3277603	0.1180356	0.09937515	0.105619	0.1436362
2183.067	18.5	0.457578	0.3270187	0.1171341	0.09781601	0.105849	0.1425124
2183.167	18.5	0.447124	0.3306211	0.1157138	0.09197585	0.1079	0.1378932
2183.267	18.5	0.445279	0.3355973	0.1164596	0.0893608	0.110639	0.135292
2183.367	18.5	0.446459	0.3420951	0.121766	0.08905862	0.115925	0.1336409
2183.467	18.5	0.419942	0.3448054	0.1228246	0.08803119	0.117308	0.1372104
2183.567	18.5	0.423689	0.3462586	0.1243699	0.08960705	0.115146	0.1391636
2183.667	18.5	0.450349	0.361977	0.1231867	0.08723687	0.118079	0.1370751

2183.767	18.5	0.435659	0.3473698	0.1287013	0.08861592	0.116423	0.1387824
2183.867	18.5	0.44564	0.3438651	0.1341453	0.08852195	0.116278	0.1360949
2183.967	18.5	0.451776	0.3442821	0.1364219	0.08775762	0.117531	0.134986
2184.067	18.5	0.444471	0.3487323	0.1371174	0.08609784	0.119307	0.1349317
2184.167	18.5	0.441009	0.3528726	0.1293301	0.08529912	0.116813	0.1329015
2184.267	18.5	0.434625	0.3463957	0.128156	0.08415571	0.115976	0.131149
2184.367	18.5	0.424351	0.3551503	0.1291319	0.08282053	0.117967	0.1298279
2184.467	18.5	0.429519	0.347718	0.1244802	0.08305799	0.119833	0.1305217
2184.567	18.5	0.425403	0.3460883	0.1225817	0.08312576	0.119594	0.1290122
2184.667	18.5	0.412243	0.3517182	0.121051	0.08253478	0.11835	0.1279615
2184.767	18.5	0.402225	0.3614522	0.1187696	0.08190683	0.119719	0.1255337
2184.867	18.5	0.411719	0.3636214	0.1173212	0.08715954	0.119412	0.1244035
2184.967	18.5	0.404939	0.3580618	0.1187241	0.0896856	0.119063	0.1263631
2185.067	18.5	0.405361	0.3541684	0.1222758	0.09017722	0.118017	0.124881
2185.167	18.5	0.4102	0.3469641	0.1251546	0.08917619	0.115517	0.1246692
2185.267	18.5	0.418198	0.3440205	0.1241765	0.08858922	0.113256	0.1260913
2185.367	18.5	0.408608	0.3446565	0.1243844	0.08983612	0.113043	0.1277947
2185.467	18.5	0.412673	0.347556	0.1220118	0.08915362	0.111948	0.1288168
2185.567	18.5	0.425699	0.3444841	0.1253034	0.08806948	0.109043	0.12971
2185.667	18.5	0.439588	0.3450933	0.1276521	0.08980814	0.108496	0.1307903
2185.767	18.5	0.430245	0.3482302	0.1267147	0.08902344	0.10717	0.1310637
2185.867	18.5	0.4261	0.3446561	0.1250132	0.0957937	0.103786	0.1323237
2185.967	18.5	0.43293	0.3472453	0.1226973	0.09644541	0.105352	0.1342378
2186.067	18.5	0.436961	0.3492184	0.1210838	0.09967876	0.107758	0.1359103
2186.167	18.5	0.423911	0.3515875	0.1205754	0.09821914	0.109244	0.1344451
2186.267	18.5	0.427792	0.344343	0.1190199	0.09739495	0.108809	0.133033
2186.367	18.5	0.428784	0.3356839	0.121365	0.09388101	0.106538	0.1350853
2186.467	18.5	0.437202	0.3391957	0.1224143	0.08832721	0.110449	0.1374197
2186.567	18.5	0.451332	0.3468016	0.1208781	0.08602807	0.110547	0.1393728
2186.667	18.5	0.432025	0.3474865	0.1188897	0.08538081	0.112824	0.1365705
2186.767	18.5	0.415084	0.3467944	0.1177996	0.08814666	0.114293	0.1349175
2186.867	18.5	0.435684	0.34767	0.1180771	0.08782567	0.113421	0.13332
2186.967	18.5	0.449018	0.3454032	0.1185559	0.08750974	0.117589	0.1334967
2187.067	18.5	0.44277	0.3555458	0.1197614	0.0870897	0.117877	0.1335634
2187.167	18.5	0.444325	0.3471807	0.1200678	0.08721602	0.114375	0.1321426
2187.267	18.5	0.4493	0.3477745	0.1258016	0.08363614	0.115257	0.1336617
2187.367	18.5	0.430071	0.3485399	0.1285274	0.08454779	0.118951	0.1313061
2187.467	18.5	0.42521	0.3418541	0.1315176	0.08451123	0.117179	0.127463
2187.567	18.5	0.417201	0.3382197	0.1321168	0.08462074	0.117089	0.1261096
2187.667	18.5	0.426059	0.3455595	0.125017	0.08139289	0.114965	0.127071
2187.767	18.5	0.438827	0.3575254	0.122183	0.08234634	0.117759	0.1278957
2187.867	18.5	0.456084	0.3480313	0.1240764	0.08266982	0.11842	0.1279641
2187.967	18.5	0.438227	0.3413953	0.1230107	0.08153449	0.11468	0.1292289
2188.067	18.5	0.430998	0.3429676	0.1216985	0.08235672	0.112896	0.1294503
2188.167	18.5	0.416857	0.3412927	0.1231744	0.08463793	0.110182	0.1290525
2188.267	18.5	0.418499	0.3453618	0.1210036	0.08500979	0.109036	0.1286088
2188.367	18.5	0.413464	0.3481863	0.1219746	0.083892	0.109885	0.1291128
2188.467	18.5	0.415777	0.342996	0.1226819	0.08320757	0.108207	0.12851
2188.567	18.5	0.415866	0.3451853	0.1239333	0.08437332	0.107884	0.1316395
2188.667	18.5	0.41325	0.3347946	0.1236932	0.0826413	0.108526	0.1346565
2188.767	18.5	0.419891	0.3359946	0.1228484	0.08196285	0.107919	0.1334684
2188.867	18.5	0.433379	0.3371693	0.1229734	0.08176786	0.107468	0.1331231
2188.967	18.5	0.436667	0.3369195	0.1236322	0.08654959	0.105603	0.1348714
2189.067	18.5	0.436838	0.3369104	0.1253843	0.09223758	0.105243	0.1336245
2189.167	18.5	0.441147	0.3359647	0.1281569	0.09359663	0.105963	0.1355768
2189.267	18.5	0.462423	0.3390268	0.1268401	0.09614913	0.108331	0.1343822
2189.367	18.5	0.475354	0.3353039	0.12441	0.09517375	0.107842	0.1349941
2189.467	18.5	0.453596	0.3247934	0.1265313	0.09402199	0.106389	0.1349624
2189.567	18.5	0.465808	0.3359919	0.1230087	0.09332453	0.107334	0.1400125
2189.667	18.5	0.467381	0.3236831	0.1235484	0.09232055	0.109028	0.1374189
2189.767	18.5	0.471875	0.3324583	0.1234641	0.08879063	0.111449	0.1354602
2189.867	18.5	0.466501	0.3223217	0.1207217	0.08911487	0.109481	0.1349928
2189.967	18.5	0.436584	0.3365538	0.120394	0.08910208	0.114851	0.1356152
2190.067	18.5	0.428365	0.3380692	0.1201928	0.08966836	0.121926	0.1366276
2190.167	18.5	0.457139	0.3415419	0.1194438	0.08687533	0.123583	0.1370292
2190.267	18.5	0.440381	0.3452279	0.1193478	0.08805823	0.124945	0.1327661
2190.367	18.5	0.453466	0.3572342	0.1197501	0.08798756	0.123383	0.1333618
2190.467	18.5	0.463085	0.3474801	0.117724	0.08854429	0.122161	0.1324264
2190.567	18.5	0.451315	0.3472714	0.1213719	0.08643197	0.118503	0.133668
2190.667	18.5	0.447507	0.3449905	0.1249851	0.08417687	0.118453	0.133775
2190.767	18.5	0.441582	0.3501827	0.1287546	0.08300155	0.122557	0.1376043
2190.867	18.5	0.440254	0.3533083	0.1302376	0.08062161	0.122134	0.1393242
2190.967	18.5	0.462594	0.3484811	0.1317291	0.08209269	0.119996	0.1333804
2191.067	18.5	0.460056	0.3514322	0.1280424	0.08185213	0.11679	0.1310713
2191.167	18.5	0.444721	0.34757	0.1254634	0.08515195	0.116421	0.1339804
2191.267	18.5	0.436065	0.3502185	0.1209703	0.08497715	0.116817	0.1319726
2191.367	18.5	0.435653	0.3556573	0.1231849	0.08689555	0.117814	0.1309946
2191.467	18.5	0.42076	0.3660406	0.1244975	0.08770912	0.116275	0.1327165
2191.567	18.5	0.41581	0.3646481	0.1202474	0.08895463	0.11544	0.1349615
2191.667	18.5	0.412367	0.3541753	0.1232478	0.08837185	0.115358	0.1351139
2191.767	18.5	0.421738	0.3505924	0.1221623	0.0887485	0.112286	0.133336
2191.867	18.5	0.414634	0.3440028	0.1234227	0.09024547	0.107585	0.1324179
2191.967	18.5	0.423513	0.3445468	0.1249299	0.09043802	0.106227	0.1345144
2192.067	18.5	0.43084	0.3494717	0.1279887	0.08851654	0.105608	0.1335696
2192.167	18.5	0.439108	0.3431014	0.127262	0.09012616	0.105748	0.1327641
2192.267	18.5	0.430959	0.3467997	0.1267456	0.09171446	0.10643	0.134069
2192.367	18.5	0.432517	0.3487549	0.1254439	0.09234912	0.108096	0.1315021
2192.467	18.5	0.448534	0.3466724	0.1261059	0.0926089	0.108091	0.1338784
2192.567	18.5	0.446915	0.3491507	0.1281151	0.09576231	0.109197	0.1352802
2192.667	18.5	0.438529	0.3481665	0.1327501	0.09462313	0.110042	0.1343722
2192.767	18.5	0.44279	0.3473587	0.1327724	0.09415603	0.109773	0.1360106
2192.867	18.5	0.434617	0.343401	0.1292355	0.09229577	0.11031	0.1364946
2192.967	18.5	0.44546	0.3411224	0.1303349	0.08987823	0.115215	0.1362328
2193.067	18.5	0.450265	0.3416079	0.1283906	0.08764307	0.114034	0.1408246
2193.167	18.5	0.425195	0.3498672	0.1270518	0.08456022	0.11461	0.1392727

2193.267	18.5	0.408223	0.3519187	0.1250294	0.08746728	0.116908	0.141393
2193.367	18.5	0.434645	0.3495532	0.1230475	0.08679677	0.114486	0.1394467
2193.467	18.5	0.436587	0.3472216	0.1228287	0.08895056	0.117915	0.1414075
2193.567	18.5	0.439669	0.3595717	0.1204634	0.08884133	0.121677	0.1444001
2193.667	18.5	0.44655	0.3481206	0.1167368	0.09047169	0.119667	0.1441787
2193.767	18.5	0.449535	0.3504269	0.1159025	0.08791965	0.120152	0.1401318
2193.867	18.5	0.427045	0.3514449	0.1178365	0.08601901	0.119666	0.13654
2193.967	18.5	0.426377	0.345933	0.120706	0.08231656	0.119043	0.13579
2194.067	18.5	0.419103	0.3416398	0.1198947	0.08236607	0.119803	0.1365272
2194.167	18.5	0.410821	0.3481448	0.1238655	0.08057913	0.120066	0.1358446
2194.267	18.5	0.417428	0.3607053	0.1271489	0.08317339	0.119969	0.1334394
2194.367	18.5	0.410311	0.3495426	0.1296126	0.0820922	0.117673	0.1364373
2194.467	18.5	0.404534	0.346868	0.1363088	0.08405111	0.117965	0.1334639
2194.567	18.5	0.405995	0.3475603	0.1412244	0.08125953	0.118106	0.1340065
2194.667	18.5	0.394225	0.3495765	0.1393702	0.08233842	0.115784	0.1348528
2194.767	18.5	0.399487	0.3450672	0.1322952	0.08327886	0.114537	0.1347357
2194.867	18.5	0.401856	0.344533	0.1349407	0.08191401	0.113083	0.1328973
2194.967	18.5	0.417947	0.3492953	0.132696	0.08293767	0.112389	0.1338461
2195.067	18.5	0.400202	0.3489169	0.1277359	0.08469936	0.109218	0.135232
2195.167	18.5	0.408157	0.3491841	0.126085	0.08555347	0.110179	0.1341656
2195.267	18.5	0.415162	0.3410672	0.1230599	0.08509632	0.107427	0.1354233
2195.367	18.5	0.42758	0.3408391	0.1246482	0.08242198	0.106424	0.1373298
2195.467	18.5	0.423052	0.342799	0.1279586	0.0889241	0.105246	0.1375038
2195.567	18.5	0.4264	0.3367386	0.1312951	0.09199309	0.105192	0.1351168
2195.667	18.5	0.433067	0.3408564	0.1317132	0.0932157	0.108199	0.1344576
2195.767	18.5	0.44502	0.3421213	0.1307951	0.09051767	0.107871	0.132437
2195.867	18.5	0.440697	0.3364518	0.131438	0.09110326	0.108097	0.1320781
2195.967	18.5	0.449648	0.3435771	0.1322284	0.09533957	0.110704	0.1344532
2196.067	18.5	0.447602	0.3313616	0.131046	0.09460067	0.108589	0.135057
2196.167	18.5	0.450431	0.3259481	0.1374484	0.090651	0.110545	0.1348258
2196.267	18.5	0.456406	0.3317423	0.1347034	0.08677363	0.110809	0.1348892
2196.367	18.5	0.451457	0.3289445	0.1322703	0.0844624	0.113478	0.1368519
2196.467	18.5	0.431491	0.3314976	0.1286651	0.08549412	0.119717	0.138824
2196.567	18.5	0.448487	0.3307334	0.127897	0.08788417	0.118817	0.139476
2196.667	18.5	0.460254	0.3369401	0.1254779	0.08791366	0.115311	0.1407874
2196.767	18.5	0.449393	0.341956	0.1264296	0.08830522	0.114144	0.139254
2196.867	18.5	0.459186	0.3460688	0.1253906	0.08842827	0.117336	0.1390704
2196.967	18.5	0.463105	0.3474894	0.1259133	0.08646012	0.11754	0.1373015
2197.067	18.5	0.446049	0.3575949	0.1250805	0.08326211	0.115361	0.1383082
2197.167	18.5	0.441623	0.3509348	0.1222332	0.08392297	0.115388	0.1413336
2197.267	18.5	0.421291	0.3437159	0.1214404	0.08503658	0.116015	0.1352821
2197.367	18.5	0.433985	0.3479218	0.1208387	0.08191052	0.117208	0.1344963
2197.467	18.5	0.457541	0.3557965	0.121466	0.08109794	0.11659	0.1329475
2197.567	18.5	0.455621	0.3537846	0.1238372	0.07998502	0.114098	0.1331965
2197.667	18.5	0.481129	0.3497626	0.1260578	0.08150651	0.114167	0.1343201
2197.767	18.5	0.453038	0.352088	0.1293017	0.08312434	0.113819	0.1342451
2197.867	18.5	0.447398	0.3443382	0.1341988	0.08584008	0.114477	0.1370494
2197.967	18.5	0.424665	0.350556	0.1374871	0.08504195	0.112676	0.1344277
2198.067	18.5	0.420599	0.3608919	0.138855	0.08520351	0.114279	0.1323215
2198.167	18.5	0.415658	0.3631566	0.1411463	0.08599731	0.115336	0.13192
2198.267	18.5	0.42266	0.3585415	0.1309059	0.08625221	0.111266	0.1347142
2198.367	18.5	0.41533	0.3558611	0.129759	0.08698738	0.108653	0.1338697
2198.467	18.5	0.4201	0.3479466	0.1291993	0.08632807	0.105436	0.1314427
2198.567	18.5	0.430004	0.3457813	0.1260576	0.08613321	0.104641	0.1330858
2198.667	18.5	0.435157	0.3502863	0.1245841	0.08770954	0.104516	0.1320774
2198.767	18.5	0.446649	0.3479949	0.1228605	0.09026261	0.104996	0.1321732
2198.867	18.5	0.45114	0.3444726	0.1234444	0.09101564	0.106604	0.1320114
2198.967	18.5	0.470099	0.3492005	0.1241975	0.09432589	0.10847	0.1313308
2199.067	18.5	0.474319	0.3482498	0.1263473	0.09520189	0.109806	0.1340812
2199.167	18.5	0.450712	0.3480518	0.1272031	0.09314219	0.110082	0.133057
2199.267	18.5	0.464479	0.3518669	0.1304628	0.09307668	0.111939	0.1320056
2199.367	18.5	0.458478	0.3509402	0.1314807	0.09158184	0.113324	0.1303456
2199.467	18.5	0.460151	0.3496417	0.1320585	0.08875188	0.113373	0.1294947
2199.567	18.5	0.465011	0.3427709	0.1309333	0.08921504	0.113709	0.1298152
2199.667	18.5	0.439918	0.3409585	0.1338234	0.08913139	0.113804	0.1324403
2199.767	18.5	0.427185	0.3482406	0.1344203	0.09022662	0.114095	0.1315048
2199.867	18.5	0.451487	0.3499069	0.1313519	0.08897825	0.117517	0.1300963
2199.967	18.5	0.434238	0.3491853	0.128773	0.08863859	0.119706	0.129976
2200.067	18.5	0.444219	0.3490353	0.1298547	0.08706314	0.122329	0.1320461
2200.167	18.5	0.456534	0.3490083	0.1247376	0.08629051	0.120599	0.1355345
2200.267	18.5	0.45167	0.354357	0.1260278	0.08600677	0.120252	0.1371415
2200.367	18.5	0.440352	0.3519223	0.1262123	0.08665143	0.118521	0.1410885
2200.467	18.5	0.429295	0.3494891	0.1246795	0.08493994	0.119017	0.1398488
2200.567	18.5	0.42481	0.3485234	0.1246844	0.08379104	0.120899	0.1453516
2200.667	18.5	0.427929	0.3412211	0.1208228	0.08574887	0.123185	0.1459429
2200.767	18.5	0.428197	0.3446705	0.1192573	0.085315	0.123782	0.1390763
2200.867	18.5	0.414174	0.3514422	0.1203819	0.08760465	0.120428	0.1362601
2200.967	18.5	0.406573	0.3564517	0.1211837	0.08873139	0.117303	0.1346247
2201.067	18.5	0.409926	0.3450978	0.1190106	0.08742369	0.114161	0.1323589
2201.167	18.5	0.399731	0.3495954	0.1220867	0.08691538	0.115397	0.1327581
2201.267	18.5	0.402167	0.3459437	0.1214851	0.08695874	0.114883	0.1305365
2201.367	18.5	0.40194	0.3483298	0.1261556	0.08615284	0.115936	0.1301665
2201.467	18.5	0.417893	0.3454029	0.1281678	0.08531919	0.113945	0.1364897
2201.567	18.5	0.406365	0.3458461	0.1323867	0.09010786	0.110973	0.1314619
2201.667	18.5	0.416153	0.3520775	0.1394684	0.09110478	0.107675	0.1300379
2201.767	18.5	0.422366	0.3488712	0.1358118	0.09048057	0.104644	0.1268964
2201.867	18.5	0.438218	0.3479616	0.1301186	0.08881743	0.10439	0.131326
2201.967	18.5	0.433096	0.3395692	0.1312956	0.09293721	0.104585	0.1287998
2202.067	18.5	0.429651	0.3395275	0.131328	0.09760708	0.104674	0.1323989
2202.167	18.5	0.436647	0.3470128	0.1280167	0.09953618	0.1044	0.1313576
2202.267	18.5	0.442476	0.3367741	0.12655	0.1001194	0.107308	0.1316577
2202.367	18.5	0.432989	0.3399029	0.1249649	0.09863815	0.107873	0.1306579
2202.467	18.5	0.430365	0.3404725	0.1213904	0.099206	0.110242	0.128182
2202.567	18.5	0.428592	0.3352315	0.1236588	0.09388379	0.112853	0.1305676
2202.667	18.5	0.427799	0.3421446	0.1232148	0.09093488	0.114681	0.1323077

2202.767	18.5	0.443243	0.3247671	0.1256498	0.09011808	0.1131	0.1296323
2202.867	18.5	0.434787	0.3309471	0.1247782	0.0892117	0.115276	0.1287494
2202.967	18.5	0.414557	0.3237855	0.1257134	0.08775353	0.117781	0.1259563
2203.067	18.5	0.428116	0.3310057	0.1259401	0.08687054	0.121606	0.1248118
2203.167	18.5	0.44037	0.3234898	0.1261654	0.08902351	0.121266	0.1251603
2203.267	18.5	0.446113	0.3337291	0.1337654	0.08695982	0.121736	0.1251296
2203.367	18.5	0.461843	0.3375641	0.131513	0.08665958	0.124475	0.1281214
2203.467	18.5	0.463671	0.3366102	0.1289825	0.0848038	0.122668	0.1298575
2203.567	18.5	0.438447	0.3465366	0.1282699	0.08701346	0.11978	0.1317866
2203.667	18.5	0.442576	0.3490129	0.1255593	0.08532032	0.121324	0.1342803
2203.767	18.5	0.416523	0.34941	0.1249081	0.08535292	0.123948	0.1384069
2203.867	18.5	0.421522	0.3483127	0.1247709	0.08399232	0.123859	0.1416347
2203.967	18.5	0.430498	0.3436824	0.1234792	0.0852279	0.121458	0.1401483
2204.067	18.5	0.430249	0.3479807	0.1200589	0.0835849	0.118027	0.1436651
2204.167	18.5	0.427706	0.354702	0.1183192	0.08592628	0.117841	0.141442
2204.267	18.5	0.427726	0.3457143	0.1160969	0.08559512	0.11918	0.1415852
2204.367	18.5	0.421944	0.3507935	0.1159284	0.08739798	0.116073	0.1350129
2204.467	18.5	0.415188	0.3474768	0.113745	0.08816742	0.112783	0.1345245
2204.567	18.5	0.411931	0.3461237	0.1196959	0.08781233	0.114627	0.1333688
2204.667	18.5	0.411452	0.3516753	0.1227388	0.08617888	0.1152	0.1340084
2204.767	18.5	0.416505	0.3619803	0.1285154	0.08777119	0.113066	0.1351799
2204.867	18.5	0.408508	0.3620468	0.1301682	0.09174873	0.110729	0.1336031
2204.967	18.5	0.428096	0.3550569	0.1366707	0.09287639	0.1086	0.134798
2205.067	18.5	0.425456	0.3509448	0.1399482	0.08987839	0.106556	0.1324584
2205.167	18.5	0.436633	0.3458571	0.1410466	0.09209677	0.107259	0.1328663
2205.267	18.5	0.443106	0.3433949	0.1367274	0.09520317	0.108938	0.1321334
2205.367	18.5	0.437848	0.351667	0.128969	0.09803741	0.108288	0.134505
2205.467	18.5	0.454532	0.3465931	0.1274352	0.09866022	0.108655	0.1359811
2205.567	18.5	0.478714	0.3446555	0.1265249	0.100731	0.110397	0.1371519
2205.667	18.5	0.451609	0.3486802	0.1237181	0.101341	0.110007	0.1363504
2205.767	18.5	0.462245	0.34612	0.1213704	0.09980533	0.111381	0.1350489
2205.867	18.5	0.454088	0.3477325	0.1184403	0.09414298	0.114091	0.1339536
2205.967	18.5	0.468796	0.3510848	0.1185474	0.09153037	0.1177	0.1329567
2206.067	18.5	0.465995	0.3547822	0.1206166	0.09031422	0.119616	0.1295962
2206.167	18.5	0.439772	0.3463774	0.120389	0.08763979	0.119125	0.1290372
2206.267	18.5	0.431045	0.341299	0.1214881	0.08678667	0.120149	0.1274216
2206.367	18.5	0.46546	0.3398337	0.1243604	0.08584234	0.120115	0.1294737
2206.467	18.5	0.444165	0.3479361	0.1251505	0.0866373	0.117427	0.1323137
2206.567	18.5	0.449344	0.3483838	0.1248509	0.08699386	0.12195	0.1323572
2206.667	18.5	0.462876	0.3470395	0.1250212	0.0857557	0.121615	0.1341608
2206.767	18.5	0.454045	0.3495131	0.1324418	0.08589014	0.119682	0.1342412
2206.867	18.5	0.445572	0.349274	0.1316074	0.08603767	0.11925	0.1337696
2206.967	18.5	0.426115	0.3493074	0.1295281	0.08548767	0.122094	0.1328587
2207.067	18.5	0.419729	0.3504463	0.1265464	0.08429941	0.121996	0.1323799
2207.167	18.5	0.433482	0.3517868	0.1226722	0.08465177	0.121596	0.1346552
2207.267	18.5	0.450991	0.3478566	0.1222075	0.08611717	0.117456	0.1376153
2207.367	18.5	0.443482	0.3386694	0.1219518	0.08589342	0.11809	0.1387517
2207.467	18.5	0.43609	0.3438594	0.1210137	0.08778585	0.116431	0.1364736
2207.567	18.5	0.443567	0.3540106	0.1211998	0.0876886	0.114655	0.1358541
2207.667	18.5	0.421516	0.3539243	0.1206554	0.08609243	0.111338	0.1380512
2207.767	18.5	0.416781	0.3424557	0.1186388	0.08492529	0.10981	0.1399348
2207.867	18.5	0.411128	0.3470421	0.1193416	0.08613322	0.110532	0.1355515
2207.967	18.5	0.421701	0.3445893	0.1206003	0.0875102	0.111141	0.1364096
2208.067	18.5	0.404908	0.3464905	0.1215125	0.08949432	0.108643	0.1365112
2208.167	18.5	0.421904	0.3439694	0.1181459	0.09118378	0.106984	0.1366774
2208.267	18.5	0.421013	0.3470838	0.1251196	0.09272505	0.106449	0.1362228
2208.367	18.5	0.436388	0.3509739	0.1320315	0.08815169	0.107331	0.135096
2208.467	18.5	0.436211	0.3448103	0.1321118	0.08932163	0.108761	0.1384424
2208.567	18.5	0.439166	0.3470404	0.1343535	0.08889973	0.109204	0.135514
2208.667	18.5	0.454028	0.338924	0.1359668	0.09123179	0.109783	0.1322504
2208.767	18.5	0.449721	0.3379131	0.1383575	0.09387796	0.109151	0.132292
2208.867	18.5	0.432677	0.3413337	0.1305242	0.09599942	0.110552	0.1328702
2208.967	18.5	0.442228	0.3407467	0.1277298	0.09678742	0.111259	0.1328366
2209.067	18.5	0.435507	0.3376612	0.1250948	0.09140694	0.112782	0.1341498
2209.167	18.5	0.436203	0.3358978	0.1226452	0.0877764	0.115631	0.1360653
2209.267	18.5	0.456302	0.3388907	0.1220329	0.08736996	0.114656	0.1352959
2209.367	18.5	0.436927	0.3381277	0.1209048	0.08800582	0.110863	0.1351383
2209.467	18.5	0.423811	0.3250448	0.1213568	0.08612358	0.11121	0.1347379
2209.567	18.5	0.439467	0.3352511	0.1220422	0.08673842	0.114592	0.1351369
2209.667	18.5	0.444087	0.3220486	0.1222541	0.08757653	0.118188	0.1331577
2209.767	18.5	0.452713	0.3306483	0.1205659	0.08725499	0.122293	0.1317849
2209.867	18.5	0.454278	0.3213676	0.1204923	0.08793511	0.118266	0.1296594
2209.967	18.5	0.460108	0.3360569	0.1237009	0.08705816	0.117632	0.1303572
2210.067	18.5	0.44214	0.3399975	0.1265058	0.08713915	0.118413	0.1303496
2210.167	18.5	0.434058	0.3410406	0.1236449	0.08654056	0.119972	0.1299673
2210.267	18.5	0.410802	0.3457155	0.1286137	0.08601245	0.121329	0.1291439
2210.367	18.5	0.411088	0.3556684	0.1267898	0.08309074	0.120965	0.1280396
2210.467	18.5	0.411638	0.3481517	0.1283966	0.08457296	0.12196	0.129325
2210.567	18.5	0.414314	0.3466417	0.126496	0.08310559	0.120272	0.1280801
2210.667	18.5	0.399461	0.3453169	0.1254479	0.08647104	0.119469	0.1330997
2210.767	18.5	0.404026	0.3502659	0.1204887	0.08703172	0.117173	0.1350611
2210.867	18.5	0.398716	0.3526349	0.1193498	0.08846376	0.112153	0.1325116
2210.967	18.5	0.397126	0.3466574	0.1239203	0.08791202	0.111669	0.1288893
2211.067	18.5	0.398361	0.3500651	0.1234249	0.08662513	0.110436	0.1274511
2211.167	18.5	0.407357	0.3459126	0.1228679	0.08661623	0.109187	0.1306962
2211.267	18.5	0.410028	0.3482362	0.1210365	0.08864067	0.108399	0.1339691
2211.367	18.5	0.405467	0.3529368	0.1192676	0.08959451	0.107168	0.1323245
2211.467	18.5	0.41949	0.3653545	0.1192652	0.08964201	0.104474	0.1313621
2211.567	18.5	0.426958	0.3617535	0.1180824	0.0885792	0.106587	0.1279765
2211.667	18.5	0.434336	0.3523228	0.1159194	0.08928773	0.106837	0.1274091
2211.767	18.5	0.43849	0.351509	0.1162387	0.08936968	0.105141	0.1283793
2211.867	18.5	0.441445	0.3443346	0.1206133	0.09223004	0.106725	0.1265084
2211.967	18.5	0.443874	0.3450107	0.1235788	0.09366261	0.107079	0.1295759
2212.067	18.5	0.455932	0.3512042	0.1321937	0.09747034	0.107999	0.1301851
2212.167	18.5	0.440509	0.3459434	0.1372443	0.1002137	0.108246	0.1290944

2212.267	18.5	0.442978	0.3473992	0.138431	0.09798396	0.107486	0.1285264
2212.367	18.5	0.443988	0.3486587	0.1315858	0.09074183	0.110052	0.1289482
2212.467	18.5	0.468931	0.3485852	0.127763	0.08678967	0.115165	0.130127
2212.567	18.5	0.46614	0.3493345	0.1284505	0.08755051	0.115615	0.1299862
2212.667	18.5	0.43214	0.3542145	0.1254055	0.08892757	0.114028	0.1316666
2212.767	18.5	0.432043	0.3556949	0.1221007	0.08826759	0.114375	0.1330205
2212.867	18.5	0.467526	0.3438565	0.1212715	0.08703646	0.114802	0.1316102
2212.967	18.5	0.453638	0.340391	0.117932	0.08715945	0.11876	0.1316693
2213.067	18.5	0.455331	0.3408904	0.1189279	0.0861659	0.122284	0.1302183
2213.167	18.5	0.468338	0.3471563	0.1223705	0.08680708	0.119591	0.1320905
2213.267	18.5	0.45433	0.3481505	0.1230766	0.08859771	0.117056	0.1329888
2213.367	18.5	0.442887	0.3486143	0.1242742	0.08764593	0.11543	0.1308164
2213.467	18.5	0.422576	0.3461936	0.1258499	0.0854405	0.117344	0.1315103
2213.567	18.5	0.417747	0.3550366	0.126211	0.08694035	0.117353	0.1310558
2213.667	18.5	0.434436	0.3466618	0.1247822	0.08757329	0.117348	0.1328713
2213.767	18.5	0.453467	0.3490084	0.1271524	0.08658513	0.117668	0.1339333
2213.867	18.5	0.475584	0.351523	0.1309464	0.08670817	0.116626	0.1344735
2213.967	18.5	0.465263	0.3464659	0.1317324	0.0876302	0.116301	0.1338966
2214.067	18.5	0.445625	0.3382159	0.1270696	0.08860291	0.114732	0.1326966
2214.167	18.5	0.414267	0.3453988	0.1259005	0.09052494	0.112492	0.132768
2214.267	18.5	0.41658	0.3584968	0.1243613	0.08860361	0.112073	0.1358659
2214.367	18.5	0.412261	0.3506942	0.1252654	0.08713064	0.110354	0.1357468
2214.467	18.5	0.417234	0.3447332	0.1254628	0.08909047	0.109814	0.1356221
2214.567	18.5	0.409253	0.347712	0.1245818	0.09096141	0.107205	0.1336541
2214.667	18.5	0.420256	0.3486307	0.1241307	0.09107292	0.105664	0.13748
2214.767	18.5	0.425529	0.3458627	0.123791	0.09079899	0.103148	0.1391907
2214.867	18.5	0.443167	0.3413294	0.1227627	0.09245913	0.103488	0.137786
2214.967	18.5	0.440579	0.3475265	0.1230502	0.09420516	0.103624	0.1335715
2215.067	18.5	0.438729	0.3487236	0.1204834	0.09682493	0.104526	0.1326769
2215.167	18.5	0.459318	0.3462968	0.1177351	0.09635616	0.105667	0.1317488
2215.267	18.5	0.468529	0.3426484	0.1167907	0.09451692	0.107719	0.1336234
2215.367	18.5	0.466073	0.3394382	0.1212974	0.09837453	0.107016	0.132062
2215.467	18.5	0.451401	0.3400815	0.1243723	0.1010221	0.108425	0.133839
2215.567	18.5	0.454924	0.3383667	0.1278459	0.09361432	0.108886	0.1364827
2215.667	18.5	0.453773	0.3398542	0.1303883	0.09154145	0.111959	0.135596
2215.767	18.5	0.462863	0.3405645	0.1348761	0.0879509	0.113695	0.133515
2215.867	18.5	0.441056	0.3363179	0.1344832	0.08699735	0.115716	0.1326725
2215.967	18.5	0.427881	0.342137	0.1273771	0.08688572	0.117577	0.1335529
2216.067	18.5	0.442753	0.3323117	0.1283754	0.08531301	0.117487	0.1318659
2216.167	18.5	0.452532	0.3236455	0.1249551	0.08358298	0.118686	0.1327973
2216.267	18.5	0.453976	0.3335797	0.1208795	0.08455273	0.121542	0.1354133
2216.367	18.5	0.459254	0.3255685	0.1220479	0.08505354	0.121999	0.1344406
2216.467	18.5	0.454431	0.3288707	0.1211475	0.08438438	0.119935	0.1355142
2216.567	18.5	0.440033	0.3289555	0.1178929	0.08604158	0.118033	0.135455
2216.667	18.5	0.431485	0.3370933	0.1201363	0.08451461	0.118214	0.134849
2216.767	18.5	0.408565	0.3412409	0.1214137	0.0835129	0.11958	0.1354418
2216.867	18.5	0.414714	0.3452685	0.1220987	0.08122499	0.119617	0.1318725
2216.967	18.5	0.417302	0.3495879	0.1226124	0.08099935	0.11938	0.1338827
2217.067	18.5	0.43304	0.3589374	0.1243657	0.0816124	0.115547	0.1341022
2217.167	18.5	0.464237	0.3533365	0.1275395	0.08176091	0.115398	0.1329256
2217.267	18.5	0.456749	0.3458425	0.1264836	0.08369698	0.116592	0.1316528
2217.367	18.5	0.423779	0.3500492	0.130577	0.08610892	0.113491	0.1334639
2217.467	18.5	0.4185	0.3547153	0.1306542	0.08533067	0.110169	0.1301501
2217.567	18.5	0.419136	0.3536752	0.1297218	0.08535859	0.109807	0.1295427
2217.667	18.5	0.428929	0.3499368	0.1285931	0.08311319	0.112262	0.1292969
2217.767	18.5	0.438985	0.3497682	0.1280418	0.08378265	0.112025	0.1335974
2217.867	18.5	0.434313	0.3426985	0.1267173	0.08721006	0.110508	0.1365805
2217.967	18.5	0.434828	0.3488084	0.1267029	0.08884893	0.109115	0.1352734
2218.067	18.5	0.440706	0.3608896	0.1269724	0.08757164	0.109104	0.1356528
2218.245	18.5	0.443694	0.3641413	0.1260952	0.09119502	0.104801	0.1373988
2218.345	19	0.444839	0.3586718	0.1213078	0.09560485	0.1034	0.1412346
2218.445	19	0.438175	0.3548198	0.1225953	0.09530629	0.103655	0.1412946
2218.545	19	0.43981	0.3476972	0.1220521	0.09429885	0.105123	0.1391169
2218.645	19	0.434937	0.3456061	0.121309	0.08753575	0.107422	0.1339106
2218.745	19	0.462603	0.3476044	0.1226358	0.08542997	0.106063	0.1313998
2218.845	19	0.44374	0.3455904	0.1194924	0.08608197	0.10591	0.1314482
2218.945	19	0.427194	0.3442704	0.1221952	0.0863917	0.106879	0.1334795
2219.045	19	0.452272	0.3463919	0.1264723	0.08494379	0.11295	0.1329958
2219.145	19	0.457502	0.3478639	0.1289193	0.08625425	0.114721	0.1364648
2219.245	19	0.456043	0.3512146	0.1319933	0.08611054	0.116452	0.1381084
2219.345	19	0.465443	0.3537232	0.1324312	0.08589689	0.116934	0.1365952
2219.445	19	0.460822	0.3545247	0.1312826	0.08594111	0.120413	0.1339761
2219.545	19	0.439862	0.3499188	0.1222877	0.08676416	0.12182	0.133365
2219.645	19	0.427533	0.3419476	0.122211	0.0833952	0.121255	0.1339561
2219.745	19	0.40923	0.3400009	0.1233684	0.08144583	0.121358	0.1315138
2219.845	19	0.416946	0.3440332	0.1232531	0.08220946	0.120336	0.1346985
2219.945	19	0.43981	0.3457829	0.1221935	0.08288436	0.12071	0.1337296
2220.045	19	0.457699	0.346459	0.1248267	0.08118579	0.120724	0.1324023
2220.145	19	0.461016	0.3468576	0.1252573	0.08109289	0.120885	0.1333696
2220.245	19	0.455173	0.3445747	0.1240302	0.08102807	0.119104	0.1351261
2220.345	19	0.426619	0.3522837	0.1267885	0.08377777	0.116901	0.1393077
2220.445	19	0.420921	0.3514822	0.1251353	0.08545147	0.116296	0.1385574
2220.545	19	0.415036	0.3499862	0.1261133	0.08492047	0.119027	0.1366311
2220.645	19	0.426108	0.3512836	0.1307046	0.0832215	0.116931	0.1353715
2220.745	19	0.41214	0.3415341	0.1269065	0.08391605	0.112116	0.1332618
2220.845	19	0.431934	0.3426473	0.1252895	0.08556965	0.112971	0.1321153
2220.945	19	0.434274	0.3514051	0.1285359	0.08514247	0.113286	0.1302373
2221.045	19	0.447706	0.357687	0.1269162	0.08652634	0.113857	0.1315844
2221.145	19	0.444408	0.3467276	0.1221168	0.08993388	0.112072	0.1289099
2221.245	19	0.440454	0.3513325	0.1209307	0.09336682	0.111973	0.1306471
2221.345	19	0.46206	0.3459715	0.1216732	0.09348246	0.108988	0.1339314
2221.445	19	0.47159	0.3474925	0.1222019	0.0942257	0.107117	0.1369724
2221.545	19	0.45335	0.3462411	0.1191826	0.09359446	0.104724	0.139273
2221.645	19	0.458492	0.3439488	0.1195959	0.09486339	0.106424	0.140294
2221.745	19	0.453617	0.350014	0.1191796	0.08998886	0.108823	0.1400715

2221.845	19	0.48574	0.3458587	0.1182386	0.08610502	0.109419	0.1447402
2221.945	19	0.474225	0.348715	0.1196335	0.08568326	0.109349	0.1450779
2222.045	19	0.44533	0.3394096	0.1227292	0.08846382	0.108779	0.1382424
2222.145	19	0.438565	0.3390641	0.1290101	0.09203614	0.109432	0.1332659
2222.245	19	0.469194	0.3464747	0.129621	0.09136422	0.110863	0.1291097
2222.345	19	0.44958	0.3355061	0.1308907	0.09009751	0.109038	0.1283464
2222.445	19	0.449593	0.3376768	0.132313	0.0892833	0.111127	0.1311267
2222.545	19	0.458551	0.3402842	0.1336815	0.0872993	0.113586	0.1306086
2222.645	19	0.459538	0.3345096	0.1301536	0.08434884	0.117332	0.1307888
2222.745	19	0.441271	0.342593	0.1256454	0.08680823	0.121346	0.1303255
2222.845	19	0.409618	0.3241109	0.1268101	0.08573856	0.123618	0.1289671
2222.945	19	0.413556	0.328011	0.1223891	0.08231988	0.121745	0.128604
2223.045	19	0.423426	0.3283427	0.1204282	0.08322924	0.118558	0.1284421
2223.145	19	0.435673	0.3311539	0.119398	0.0855095	0.119911	0.1289124
2223.245	19	0.444967	0.330088	0.1188912	0.08650751	0.119547	0.1288106
2223.345	19	0.441112	0.334727	0.1184621	0.08526849	0.11976	0.1312213
2223.445	19	0.42435	0.3404163	0.1216479	0.08847064	0.119773	0.133309
2223.545	19	0.410863	0.340232	0.122904	0.08900569	0.118753	0.1329881
2223.645	19	0.409726	0.3487779	0.1205319	0.08794751	0.115707	0.1340852
2223.745	19	0.41493	0.3516829	0.1227733	0.08708226	0.112767	0.1307505
2223.845	19	0.416686	0.3524951	0.1231819	0.08656079	0.116869	0.1299828
2223.945	19	0.40983	0.3509245	0.1233671	0.08652508	0.116676	0.130615
2224.045	19	0.423192	0.3452682	0.1267872	0.08572348	0.114516	0.1325681
2224.145	19	0.429566	0.350916	0.124625	0.08345361	0.114503	0.133014
2224.245	19	0.439794	0.3552181	0.1246512	0.08509832	0.112086	0.1328379
2224.345	19	0.434629	0.3477927	0.1243285	0.08850662	0.109392	0.1310797
2224.445	19	0.449167	0.3486392	0.1219396	0.08832707	0.108467	0.131307
2224.545	19	0.460224	0.3457048	0.1198925	0.0893292	0.107828	0.1323653
2224.645	19	0.45494	0.3448222	0.1182477	0.09294174	0.107735	0.1338308
2224.745	19	0.448889	0.3504984	0.1176002	0.09436531	0.108471	0.132374
2224.845	19	0.446469	0.3621223	0.1180061	0.09308933	0.109585	0.1321018
2224.945	19	0.446129	0.3608995	0.1169719	0.08684277	0.111266	0.1345715
2225.045	19	0.466921	0.3548208	0.1172644	0.08597241	0.111983	0.1396297
2225.145	19	0.451961	0.3504165	0.1186048	0.08776738	0.1115	0.142178
2225.245	19	0.430269	0.3447431	0.1188676	0.08893352	0.112229	0.1384096
2225.345	19	0.454812	0.3421276	0.1198921	0.08682725	0.112227	0.1397192
2225.445	19	0.459842	0.349167	0.1214011	0.08689538	0.112845	0.1394088
2225.545	19	0.452736	0.3448648	0.1261498	0.08591592	0.114328	0.1408067
2225.645	19	0.462755	0.3457178	0.126206	0.08659682	0.116593	0.1388614
2225.745	19	0.466938	0.3462091	0.1302831	0.08484282	0.121547	0.1354291
2225.845	19	0.446415	0.3483022	0.1347159	0.08443398	0.121278	0.1324284
2225.945	19	0.433213	0.350303	0.1310855	0.08360051	0.120559	0.1370079
2226.045	19	0.411349	0.3548666	0.1290853	0.08338434	0.123543	0.138943
2226.145	19	0.411593	0.3571667	0.123094	0.08318043	0.124978	0.1384536
2226.245	19	0.42241	0.3458995	0.1245653	0.08403111	0.121978	0.1392
2226.345	19	0.437854	0.3409703	0.1236141	0.08306111	0.12005	0.1365751
2226.445	19	0.463838	0.3395378	0.1204341	0.08537051	0.121411	0.1337812
2226.545	19	0.462654	0.3464693	0.1194129	0.08453739	0.12022	0.1344884
2226.645	19	0.428625	0.3459933	0.1188458	0.08525138	0.121885	0.1328854
2226.745	19	0.423747	0.3476568	0.1175362	0.08707827	0.12403	0.1302986
2226.845	19	0.418075	0.3478849	0.1176238	0.09004863	0.121084	0.1314456
2226.945	19	0.428514	0.3524103	0.1187186	0.08704628	0.119403	0.1334122
2227.045	19	0.42278	0.3458453	0.1204591	0.08793952	0.119993	0.1338604
2227.145	19	0.429178	0.3502882	0.1199012	0.08813073	0.117173	0.1335002
2227.245	19	0.429896	0.3527733	0.1193082	0.08792773	0.116907	0.1312609
2227.345	19	0.445572	0.3489343	0.1217601	0.08757716	0.115465	0.1320643
2227.445	19	0.447229	0.3391702	0.1247952	0.08995555	0.114487	0.1319951
2227.545	19	0.441631	0.3442934	0.1248935	0.09351796	0.111491	0.1308093
2227.645	19	0.453959	0.3547975	0.1241277	0.095534	0.109271	0.1336368
2227.745	19	0.461537	0.3541923	0.1214325	0.09553328	0.108668	0.1317079
2227.845	19	0.465923	0.3470596	0.1212609	0.09567825	0.108994	0.1290802
2227.945	19	0.453499	0.3494574	0.1219522	0.09621973	0.109005	0.1314257
2228.045	19	0.456046	0.3464757	0.1213025	0.0922167	0.108089	0.1310315
2228.145	19	0.468268	0.3464613	0.1198938	0.08885095	0.10879	0.1304435
2228.245	19	0.46978	0.3416926	0.1180255	0.08690538	0.110266	0.130153
2228.345	19	0.450348	0.3443088	0.1164397	0.08465709	0.109994	0.1307721
2228.445	19	0.437411	0.3488619	0.1167426	0.08679045	0.111263	0.1331599
2228.545	19	0.470042	0.3413947	0.1163964	0.08471873	0.111306	0.1351398
2228.645	19	0.45003	0.344527	0.1199555	0.08460144	0.113656	0.1374789
2228.745	19	0.456268	0.3363424	0.1251743	0.08748627	0.113866	0.1397227
2228.845	19	0.455787	0.3381904	0.1250601	0.08712438	0.1149	0.140211
2228.945	19	0.457725	0.3394204	0.1265769	0.08718221	0.115312	0.1354943
2229.045	19	0.445736	0.3372661	0.1279259	0.08655883	0.11449	0.1367738
2229.145	19	0.420893	0.3359668	0.130589	0.08357498	0.113442	0.1356581
2229.245	19	0.407825	0.3338081	0.1342216	0.08151042	0.11858	0.1349217
2229.345	19	0.419241	0.3401453	0.1383785	0.08178797	0.121939	0.1356103
2229.445	19	0.442258	0.3340579	0.1371929	0.07892023	0.121398	0.1363202
2229.545	19	0.460099	0.3237463	0.1283965	0.07901724	0.118552	0.1396526
2229.645	19	0.446926	0.3316473	0.1292462	0.07843675	0.117982	0.1395957
2229.745	19	0.435691	0.3219109	0.1300713	0.07971118	0.118247	0.1401204
2229.845	19	0.416619	0.3321251	0.1262824	0.08284677	0.118811	0.1372566
2229.945	19	0.413908	0.3233133	0.1241697	0.0871862	0.119423	0.1364324
2230.045	19	0.411039	0.3370404	0.1214225	0.08687291	0.119263	0.1346128
2230.145	19	0.42116	0.3395293	0.1212685	0.08496933	0.118036	0.1327855
2230.245	19	0.415601	0.3415877	0.1211792	0.08837324	0.117744	0.1345575
2230.345	19	0.42884	0.3448425	0.1219346	0.09198538	0.117513	0.1331746
2230.445	19	0.42802	0.3563917	0.1216598	0.09040513	0.117216	0.134054
2230.545	19	0.437791	0.3463413	0.1198138	0.09165714	0.11776	0.133561
2230.645	19	0.438747	0.3461398	0.1196821	0.09571462	0.116914	0.1333713
2230.745	19	0.443078	0.3449555	0.1200181	0.0947042	0.113818	0.131176
2230.845	19	0.453032	0.3488452	0.1238224	0.09755423	0.109172	0.1311793
2230.945	19	0.452762	0.3492784	0.1262477	0.09742142	0.107383	0.1331173
2231.045	19	0.449601	0.344263	0.1243999	0.09491046	0.106798	0.1329249
2231.145	19	0.453535	0.344181	0.1206815	0.09277201	0.109817	0.1323157
2231.245	19	0.444375	0.3413947	0.1195545	0.08783222	0.10988	0.132336

2231.345	19	0.461439	0.3419697	0.1184622	0.08803913	0.11051	0.1317754
2231.445	19	0.447964	0.3471491	0.1186233	0.08859102	0.111719	0.1318953
2231.545	19	0.425018	0.3594766	0.1189448	0.08891142	0.111102	0.1288494
2231.645	19	0.444711	0.3568626	0.1191765	0.09002544	0.110218	0.1310659
2231.745	19	0.453741	0.3480758	0.120769	0.08913484	0.110119	0.1315446
2231.845	19	0.453017	0.3465684	0.1196113	0.09084172	0.111529	0.1327544
2231.945	19	0.457686	0.3417211	0.1180631	0.08874901	0.113899	0.1325405
2232.045	19	0.46096	0.3406787	0.1202391	0.08783838	0.111378	0.1340297
2232.145	19	0.452043	0.3469262	0.1190911	0.08794054	0.113358	0.1352541
2232.245	19	0.438562	0.3446064	0.1204498	0.08783718	0.117021	0.1323294
2232.345	19	0.410643	0.3438754	0.1252203	0.08360168	0.116944	0.131106
2232.445	19	0.410071	0.3462203	0.1265627	0.08247918	0.119655	0.1355344
2232.545	19	0.414389	0.3497532	0.1283511	0.0822169	0.123098	0.1385601
2232.645	19	0.42977	0.3505289	0.1314239	0.08346123	0.119339	0.1363486
2232.745	19	0.422503	0.3541969	0.1365027	0.0846951	0.117108	0.1344058
2232.845	19	0.427754	0.3507889	0.1378888	0.08368206	0.115945	0.1318936
2232.945	19	0.412912	0.3413387	0.1286839	0.0844387	0.116189	0.1322382
2233.045	19	0.410978	0.3385324	0.1258938	0.08556402	0.116279	0.1313186
2233.145	19	0.410903	0.3380625	0.1256695	0.08775666	0.116521	0.1308515
2233.245	19	0.416458	0.3426445	0.1224499	0.08586033	0.11527	0.1336365
2233.345	19	0.411713	0.3474693	0.1219172	0.08704016	0.112574	0.1315546
2233.445	19	0.419367	0.3470368	0.1195381	0.08989152	0.111928	0.1299644
2233.545	19	0.426403	0.3431581	0.1191105	0.0898235	0.112842	0.1304109
2233.645	19	0.432723	0.355698	0.1170881	0.08713771	0.114502	0.1302981
2233.745	19	0.438887	0.3461517	0.1188169	0.09024251	0.113607	0.1313564
2233.845	19	0.441799	0.3500876	0.1199581	0.09384225	0.112967	0.1313075
2233.945	19	0.444705	0.3509595	0.1210483	0.09214032	0.112731	0.135138
2234.045	19	0.461944	0.343912	0.1245704	0.09235419	0.109995	0.1349423
2234.145	19	0.471979	0.337624	0.1242007	0.09469331	0.10897	0.1349177
2234.245	19	0.454767	0.3463578	0.1229729	0.09583599	0.107306	0.1323212
2234.345	19	0.458391	0.3573239	0.125471	0.09469534	0.106718	0.1338143
2234.445	19	0.472463	0.350291	0.1254968	0.09037066	0.105896	0.1328663
2234.545	19	0.475942	0.3470436	0.1235579	0.08588807	0.108829	0.1344065
2234.645	19	0.451593	0.35017	0.1200274	0.08490756	0.108949	0.1371731
2234.745	19	0.435488	0.347467	0.1188293	0.08459748	0.109769	0.1366439
2234.845	19	0.471637	0.3427536	0.1174869	0.0849684	0.108768	0.1345246
2234.945	19	0.449119	0.3390897	0.1187301	0.08430357	0.10908	0.1316838
2235.045	19	0.456089	0.344603	0.117747	0.08229367	0.107509	0.1325035
2235.145	19	0.456915	0.3461027	0.118718	0.08334985	0.110943	0.1330204
2235.245	19	0.46211	0.3449682	0.1175492	0.08293035	0.112525	0.1306299
2235.345	19	0.444121	0.3391021	0.1187316	0.08557583	0.113644	0.1272055
2235.445	19	0.423491	0.3395478	0.1190447	0.08377106	0.116556	0.1271931
2235.545	19	0.401263	0.3395218	0.1215863	0.08251725	0.119961	0.1312158
2235.645	19	0.409979	0.3365492	0.1241275	0.08113263	0.118776	0.1327711
2235.745	19	0.431521	0.33854	0.1245392	0.08152182	0.118848	0.1333605
2235.845	19	0.44573	0.3376901	0.1228686	0.08157112	0.118106	0.1373154
2235.945	19	0.463831	0.3344903	0.1280894	0.0822099	0.11765	0.1384019
2236.045	19	0.45245	0.3438789	0.1369898	0.07981732	0.114436	0.1391512
2236.145	19	0.42933	0.3340752	0.1371163	0.08161287	0.118143	0.1387662
2236.245	19	0.425813	0.3285704	0.1387694	0.08365835	0.117994	0.1378192
2236.345	19	0.427676	0.3348179	0.1338284	0.08276634	0.121452	0.1329023
2236.445	19	0.434872	0.3278483	0.1279988	0.08140212	0.121654	0.1314007
2236.545	19	0.422399	0.3302262	0.1262613	0.08286366	0.120445	0.1320451
2236.645	19	0.434772	0.3273383	0.1234561	0.08440257	0.120068	0.131322
2236.745	19	0.436492	0.3341489	0.1218536	0.08335931	0.118751	0.1322934
2236.845	19	0.446783	0.339631	0.1209147	0.08390747	0.116583	0.1331554
2236.945	19	0.449935	0.3436391	0.1183237	0.08896902	0.114213	0.1347554
2237.045	19	0.445611	0.3455947	0.1183398	0.09216696	0.11337	0.1367545
2237.145	19	0.449817	0.3562805	0.119986	0.09269682	0.113019	0.1336512
2237.245	19	0.457741	0.3496626	0.1220921	0.09732717	0.111414	0.1320112
2237.345	19	0.450529	0.3436093	0.1224036	0.09837868	0.106747	0.1293102
2237.445	19	0.458026	0.347531	0.123695	0.09665408	0.106262	0.1309016
2237.545	19	0.450493	0.3509762	0.1239352	0.09243023	0.108485	0.134121
2237.645	19	0.463485	0.3486392	0.1260806	0.09036001	0.108968	0.1341443
2237.745	19	0.456791	0.3444488	0.1289348	0.08633129	0.109393	0.1339919
2237.845	19	0.440918	0.3436968	0.1288035	0.08626802	0.11067	0.1311029
2237.945	19	0.444916	0.3392176	0.1238944	0.08587568	0.108053	0.1329116
2238.045	19	0.462516	0.341283	0.1225385	0.08448812	0.1068	0.1331199
2238.145	19	0.450823	0.3544775	0.1235245	0.08427223	0.1067	0.1332097
2238.245	19	0.456534	0.3570789	0.1225013	0.08396637	0.106576	0.1295177
2238.345	19	0.458477	0.3531338	0.1204352	0.08419834	0.105209	0.1284442
2238.445	19	0.456714	0.3471604	0.119489	0.08215886	0.111782	0.1312599
2238.545	19	0.446201	0.3443643	0.1200803	0.0804558	0.112644	0.130606
2238.645	19	0.403152	0.3422507	0.1185793	0.07758898	0.116477	0.1333116
2238.745	19	0.40869	0.3406083	0.1193815	0.07939195	0.123225	0.1346671
2238.845	19	0.406347	0.3419084	0.1199617	0.07763355	0.122131	0.1309175
2238.945	19	0.426277	0.3446572	0.1204919	0.07816741	0.123118	0.1280455
2239.045	19	0.422216	0.3444913	0.1238992	0.07776974	0.125118	0.1303714
2239.145	19	0.424525	0.3472297	0.1234305	0.08182225	0.122785	0.1288978
2239.245	19	0.418511	0.3504848	0.12357	0.08203038	0.121889	0.1304458
2239.345	19	0.409682	0.3518273	0.1247859	0.08297708	0.120591	0.1318343
2239.445	19	0.408911	0.3510431	0.1303802	0.08193828	0.121763	0.1328231
2239.545	19	0.417202	0.3486431	0.1341554	0.0783365	0.121934	0.1348343
2239.645	19	0.418131	0.3413003	0.1351328	0.07924434	0.12145	0.1305619
2239.745	19	0.417522	0.3366702	0.1337516	0.08151531	0.119498	0.1299032
2239.845	19	0.432726	0.3380502	0.1243695	0.08113685	0.11855	0.1312782
2239.945	19	0.431268	0.3427074	0.1244163	0.08202959	0.119022	0.1327854
2240.045	19	0.438123	0.3482178	0.1243218	0.08451154	0.117832	0.1346852
2240.145	19	0.439491	0.3457217	0.1220965	0.0871684	0.117278	0.1335451
2240.245	19	0.446896	0.341479	0.1229512	0.08728381	0.116008	0.1322843
2240.345	19	0.449246	0.3558	0.1182943	0.08997798	0.114462	0.1310462
2240.445	19	0.462441	0.3479549	0.1171101	0.08993137	0.112329	0.1295784
2240.545	19	0.448472	0.349339	0.1182333	0.09262228	0.111209	0.1276498
2240.645	19	0.459694	0.3504331	0.11914	0.09177329	0.109446	0.1287207
2240.745	19	0.452227	0.3417224	0.1217916	0.088474	0.108394	0.1291508

2240.845	19	0.461237	0.3398808	0.1218072	0.08645232	0.108411	0.1284097
2240.945	19	0.443796	0.3493069	0.1222846	0.08613232	0.107816	0.1255566
2241.045	19	0.423856	0.3564444	0.1229545	0.08178701	0.108607	0.1241013
2241.145	19	0.452875	0.3447635	0.1247977	0.08232122	0.105674	0.1253975
2241.245	19	0.452561	0.3500899	0.1241212	0.08353383	0.10541	0.1262011
2241.345	19	0.448354	0.346267	0.1222053	0.08380937	0.104888	0.1289702
2241.445	19	0.454141	0.3437215	0.1203354	0.08335926	0.104995	0.1273696
2241.545	19	0.458167	0.3409331	0.1246836	0.08348575	0.106083	0.1268842
2241.645	19	0.440556	0.338432	0.1238169	0.08582707	0.10927	0.1291145
2241.745	19	0.435268	0.3425297	0.1226651	0.08398978	0.117009	0.1306722
2241.845	19	0.400015	0.3410483	0.1218703	0.08401496	0.11889	0.1344522
2241.945	19	0.403409	0.3443035	0.1237031	0.0811537	0.120432	0.1350496
2242.045	19	0.414199	0.3350748	0.1226248	0.0814544	0.122471	0.1331272
2242.145	19	0.429235	0.3366844	0.1205189	0.08146639	0.121306	0.1348034
2242.245	19	0.437724	0.3437459	0.1201774	0.08431153	0.123827	0.1384862
2242.345	19	0.437883	0.3326919	0.1179668	0.0857007	0.121588	0.1388615
2242.445	19	0.430293	0.3349974	0.1176784	0.08593948	0.118639	0.1378905
2242.545	19	0.421247	0.3394428	0.1170425	0.08694652	0.113763	0.1361006
2242.645	19	0.421431	0.3327751	0.1211238	0.08667388	0.117651	0.1339505
2242.745	19	0.429563	0.3418512	0.1231036	0.08477239	0.119902	0.1341191
2242.845	19	0.421127	0.3223119	0.1293591	0.08707967	0.122652	0.1355252
2242.945	19	0.428476	0.3286877	0.1355095	0.08947581	0.123324	0.1362549
2243.045	19	0.427194	0.324832	0.1385423	0.09020323	0.121399	0.1335418
2243.145	19	0.441865	0.3269959	0.136536	0.08853627	0.118598	0.1315242
2243.245	19	0.448732	0.3269614	0.1257631	0.08886601	0.118478	0.1325319
2243.345	19	0.445816	0.3319122	0.1239557	0.09143864	0.118076	0.131962
2243.445	19	0.455461	0.3380755	0.1238588	0.09506776	0.119934	0.1331307
2243.545	19	0.458157	0.338644	0.1206883	0.09449928	0.120031	0.1330911
2243.645	19	0.460807	0.3442287	0.1213822	0.09338703	0.118446	0.1343127
2243.745	19	0.454401	0.3473754	0.1163471	0.09350575	0.115075	0.1331069
2243.845	19	0.456077	0.3503039	0.1159485	0.09072482	0.11295	0.1306095
2243.945	19	0.469779	0.3485751	0.1181183	0.08943267	0.110987	0.1281683
2244.045	19	0.460934	0.343663	0.1190685	0.09056126	0.109624	0.1303705
2244.145	19	0.441162	0.3464406	0.1196813	0.08841491	0.111707	0.1314235
2244.245	19	0.432824	0.3515678	0.1174178	0.08763643	0.113185	0.1296172
2244.345	19	0.464476	0.3446523	0.1171065	0.08684702	0.112348	0.1297674
2244.445	19	0.446254	0.3452464	0.1179727	0.08373425	0.112745	0.129442
2244.545	19	0.45084	0.3394477	0.120275	0.0853186	0.112516	0.131159
2244.645	19	0.456875	0.3384068	0.1267649	0.08569624	0.112353	0.1333654
2244.745	19	0.456026	0.3429251	0.1234661	0.08579774	0.11396	0.1330146
2244.845	19	0.446198	0.3547015	0.1213357	0.08476468	0.117156	0.1297562
2244.945	19	0.41087	0.3540498	0.1223909	0.0833122	0.116793	0.1302138
2245.045	19	0.405158	0.3485276	0.1205469	0.08223103	0.115563	0.1313855
2245.145	19	0.409538	0.3445119	0.1191989	0.0803654	0.116678	0.1321493
2245.245	19	0.420547	0.3391213	0.1191594	0.08130293	0.118835	0.1323805
2245.345	19	0.431146	0.3357266	0.119844	0.08371906	0.120499	0.1320621
2245.445	19	0.424171	0.3439505	0.117642	0.08746617	0.124796	0.1305096
2245.545	19	0.428617	0.3430912	0.1150669	0.08697998	0.127252	0.1304604
2245.645	19	0.415525	0.3419219	0.1118563	0.08652867	0.122407	0.1347255
2245.745	19	0.409593	0.3450892	0.1111376	0.08674173	0.11705	0.1382268
2245.845	19	0.416604	0.3473746	0.1121844	0.0863357	0.119215	0.1425813
2245.945	19	0.421836	0.3464537	0.1115834	0.08665698	0.123843	0.1406509
2246.045	19	0.416577	0.3530678	0.1122144	0.08654866	0.12333	0.1392412
2246.145	19	0.430759	0.353476	0.1169472	0.08827171	0.123453	0.1389528
2246.245	19	0.426792	0.3452261	0.1203302	0.08938774	0.12423	0.1407637
2246.345	19	0.441738	0.3355103	0.1264427	0.08976901	0.124442	0.1387447
2246.445	19	0.440972	0.3349958	0.1314806	0.09146073	0.121115	0.1379402
2246.545	19	0.452418	0.341643	0.136269	0.09683854	0.119537	0.135084
2246.645	19	0.460787	0.3413683	0.1286804	0.0988485	0.11718	0.1373085
2246.745	19	0.466794	0.3433043	0.1262714	0.09697537	0.117924	0.1358081
2246.845	19	0.453823	0.3451273	0.1276098	0.09491551	0.119209	0.1364315
2246.945	19	0.461535	0.3478938	0.1258791	0.09529758	0.115466	0.1331592
2247.045	19	0.452705	0.3446739	0.1239953	0.09300374	0.10977	0.1334087
2247.145	19	0.466714	0.3475415	0.1225357	0.09042601	0.108092	0.1318953
2247.245	19	0.449752	0.3483292	0.1199843	0.08980205	0.106972	0.1305165
2247.345	19	0.427104	0.3452266	0.1186574	0.08770677	0.106045	0.1305086
2247.445	19	0.453907	0.3372734	0.1213342	0.08818033	0.106606	0.1320381
2247.545	19	0.453412	0.3429564	0.1210186	0.08843674	0.108104	0.1323129
2247.645	19	0.448726	0.3520557	0.1205945	0.08853601	0.108238	0.1320628
2247.745	19	0.439234	0.3525327	0.1212704	0.08965341	0.107977	0.1307862
2247.845	19	0.455256	0.345565	0.1230785	0.08933531	0.106846	0.1306539
2247.945	19	0.446626	0.3495538	0.1215603	0.08804859	0.10729	0.1313614
2248.045	19	0.437275	0.3360897	0.1276269	0.08531516	0.105956	0.1337109
2248.145	19	0.398244	0.3408888	0.1278218	0.08571026	0.108223	0.1325373
2248.245	19	0.405062	0.3379678	0.1246711	0.08426934	0.110879	0.1310544
2248.345	19	0.409117	0.3399678	0.1239294	0.08243316	0.113788	0.128877
2248.445	19	0.426865	0.330793	0.1228309	0.08422354	0.117684	0.1269976
2248.545	19	0.42374	0.3361358	0.1209789	0.08471531	0.120696	0.1300441
2248.645	19	0.429079	0.3333378	0.1219542	0.08317602	0.128689	0.1303998
2248.745	19	0.42616	0.3340201	0.1216331	0.08442525	0.13128	0.1301706
2248.845	19	0.412542	0.3362227	0.1221216	0.08746418	0.132128	0.1328176
2248.945	19	0.416494	0.3297174	0.1206007	0.0883551	0.126047	0.1301753
2249.045	19	0.424141	0.3386202	0.1177444	0.08694428	0.120412	0.1297596
2249.145	19	0.418564	0.3184555	0.1154119	0.08731682	0.122304	0.1295632
2249.245	19	0.424997	0.3256132	0.1145518	0.08749072	0.107053	0.1329333
2249.345	19	0.42949	0.3216197	0.1176254	0.08714123	0.107018	0.1344156
2249.445	19	0.434918	0.3265239	0.1180254	0.08656338	0.108866	0.1349805
2249.545	19	0.448335	0.3202572	0.119421	0.08922721	0.110413	0.1346536
2249.645	19	0.443351	0.3342008	0.1216165	0.09390537	0.108692	0.1350512
2249.745	19	0.455611	0.338523	0.1263471	0.09868344	0.109918	0.1332427
2249.845	19	0.450218	0.3419971	0.1287385	0.09808477	0.111189	0.1340379
2249.945	19	0.464697	0.3459491	0.1321291	0.0984991	0.111324	0.1306769
2250.045	19	0.447362	0.3554201	0.1310861	0.1011666	0.111732	0.1306103
2250.145	19	0.452217	0.3487444	0.1264125	0.09827565	0.111614	0.1300811
2250.245	19	0.464402	0.3442991	0.1252963	0.0930372	0.112613	0.1307477

2250.345	19	0.454126	0.3451836	0.125233	0.08791222	0.117597	0.1322976
2250.445	19	0.442836	0.3517918	0.1222216	0.08750506	0.123845	0.1326851
2250.545	19	0.434603	0.3460727	0.1213695	0.08407047	0.128574	0.1348042
2250.645	19	0.46677	0.345908	0.1186444	0.08234381	0.127141	0.135218
2250.745	19	0.444609	0.3389385	0.1205317	0.08398023	0.120397	0.1347965
2250.845	19	0.447158	0.3397606	0.1231437	0.08507623	0.117759	0.1327205
2250.945	19	0.455244	0.3440441	0.1247038	0.08869156	0.116265	0.1328154
2251.045	19	0.454675	0.3574195	0.1233678	0.08797798	0.11429	0.1325977
2251.145	19	0.444092	0.3541587	0.1251625	0.0880013	0.114722	0.1333231
2251.245	19	0.417677	0.3485519	0.1251234	0.08591104	0.11445	0.1326389
2251.345	19	0.397727	0.3444223	0.1245433	0.08528291	0.11524	0.1325933
2251.445	19	0.40749	0.3392364	0.1280788	0.08303455	0.116605	0.1314758
2251.545	19	0.420044	0.339523	0.1286515	0.08479432	0.115275	0.1288164
2251.645	19	0.423834	0.3449405	0.125621	0.08768797	0.113434	0.1293083
2251.745	19	0.426131	0.3426977	0.1236623	0.08895954	0.114418	0.1271857
2251.845	19	0.43542	0.3443049	0.1232934	0.08693931	0.114306	0.129553
2251.945	19	0.425605	0.3459018	0.1206444	0.08891923	0.111051	0.1290764
2252.045	19	0.407924	0.3491125	0.1197821	0.09064341	0.107437	0.128625
2252.145	19	0.420988	0.351618	0.1206644	0.08944224	0.1062	0.1329412
2252.245	19	0.430558	0.3528298	0.1196693	0.09023733	0.103882	0.1324283
2252.345	19	0.415831	0.3480016	0.1186154	0.08797071	0.105693	0.1344558
2252.445	19	0.422903	0.3368281	0.1205127	0.08742706	0.106652	0.1355118
2252.545	19	0.418349	0.3342639	0.1200613	0.08653337	0.107319	0.1365362
2252.645	19	0.436653	0.3402048	0.1203698	0.08707274	0.106216	0.1365019
2252.745	19	0.444909	0.3400752	0.1208982	0.09162692	0.109037	0.1386654
2252.845	19	0.445003	0.3456039	0.1185877	0.09649187	0.108207	0.1408653
2252.945	19	0.451974	0.3448078	0.1217103	0.1000991	0.109327	0.1426756
2253.045	19	0.462301	0.3521254	0.1223341	0.1015874	0.106604	0.1409519
2253.145	19	0.459495	0.3429156	0.1270765	0.102572	0.109246	0.1370065
2253.245	19	0.455331	0.3489831	0.1278697	0.1027999	0.110755	0.1384325
2253.345	19	0.448859	0.346857	0.1291472	0.09496897	0.107659	0.1347957
2253.445	19	0.456418	0.3394306	0.1298835	0.09001602	0.10851	0.1334637
2253.545	19	0.450634	0.3404838	0.1251204	0.08948093	0.10853	0.1336308
2253.645	19	0.430264	0.3473532	0.1226904	0.08861429	0.111148	0.1331377
2253.745	19	0.440699	0.3546564	0.1207304	0.08585536	0.115457	0.1344314
2253.845	19	0.461163	0.3434268	0.1184889	0.08300004	0.112965	0.1329664
2253.945	19	0.446854	0.350726	0.1187225	0.08428592	0.110481	0.1313345
2254.045	19	0.451618	0.3429364	0.115504	0.0870994	0.113034	0.13012
2254.145	19	0.448262	0.3411421	0.1165579	0.08754782	0.114909	0.1301195
2254.245	19	0.446495	0.3400124	0.1186955	0.08729245	0.11488	0.1304964
2254.345	19	0.439949	0.3353361	0.1220345	0.08649686	0.11634	0.1319389
2254.445	19	0.402239	0.3405523	0.1207035	0.08443627	0.115241	0.1314908
2254.545	19	0.40571	0.3370986	0.1223028	0.08266164	0.113934	0.1297157
2254.645	19	0.406786	0.3397072	0.1206729	0.08413536	0.115754	0.1304505
2254.745	19	0.426738	0.3294187	0.1203338	0.08445157	0.11731	0.1319071
2254.845	19	0.421988	0.334663	0.1207626	0.08646554	0.1156	0.1338565
2254.945	19	0.420376	0.3325486	0.1279948	0.08681309	0.114232	0.1327753
2255.045	19	0.422985	0.3340182	0.1262892	0.08841262	0.115449	0.1305463
2255.145	19	0.410004	0.332712	0.1213315	0.08958327	0.11216	0.1295965
2255.245	19	0.408403	0.3286993	0.1222923	0.09012978	0.107834	0.128211
2255.345	19	0.416699	0.3384994	0.1195201	0.08693987	0.105934	0.1282208
2255.445	19	0.413372	0.320333	0.1191605	0.08533406	0.103604	0.1283704
2255.545	19	0.415083	0.3216158	0.1196214	0.08416385	0.104956	0.1318573
2255.645	19	0.418875	0.3212323	0.1157504	0.08640328	0.103796	0.1351304
2255.745	19	0.423925	0.3219132	0.1141949	0.08680985	0.105485	0.1354854
2255.845	19	0.442751	0.3231114	0.1134137	0.08714151	0.105962	0.1342031
2255.945	19	0.438893	0.3311735	0.1130295	0.08969447	0.105489	0.1333035
2256.045	19	0.451287	0.337796	0.1125952	0.09095211	0.106154	0.1346118
2256.145	19	0.449399	0.3408321	0.113845	0.09221053	0.107388	0.1365883
2256.245	19	0.471713	0.3471341	0.1143021	0.09227895	0.108419	0.1373653
2256.345	19	0.450001	0.3565953	0.1191775	0.09445274	0.111723	0.1386274
2256.445	19	0.448995	0.3481919	0.1212964	0.09403509	0.113657	0.1386134
2256.545	19	0.463989	0.3497748	0.1202927	0.0893682	0.112618	0.1340837
2256.645	19	0.469593	0.347056	0.1242226	0.08779574	0.110682	0.1335037
2256.745	19	0.447363	0.350923	0.1281339	0.08755197	0.109513	0.1337022
2256.845	19	0.431688	0.3501604	0.1323303	0.08317322	0.113728	0.1323275
2256.945	19	0.476937	0.3474203	0.1272599	0.08150537	0.12026	0.1328459
2257.045	19	0.45361	0.3448858	0.1289795	0.08131836	0.120268	0.1315352
2257.145	19	0.449629	0.3397914	0.1262106	0.08231767	0.11933	0.1341801
2257.245	19	0.458764	0.343107	0.1215565	0.08267935	0.119321	0.1327971
2257.345	19	0.455957	0.3541916	0.12042	0.08035699	0.118371	0.1320061
2257.445	19	0.442956	0.3552866	0.1184943	0.08031395	0.116016	0.1324529
2257.545	19	0.435109	0.3509948	0.1176507	0.07844484	0.114185	0.1314648
2257.645	19	0.401629	0.3456551	0.1165272	0.08054207	0.111978	0.1307377
2257.745	19	0.411979	0.3383366	0.1185367	0.08148127	0.112004	0.1317337
2257.845	19	0.418849	0.33768	0.1215081	0.0793594	0.114945	0.1340196
2257.945	19	0.422889	0.3452457	0.1216431	0.07922758	0.114026	0.1309651
2258.045	19	0.420585	0.3426398	0.1236946	0.08160844	0.113212	0.1300978
2258.145	19	0.430457	0.3432	0.1221977	0.08096569	0.113843	0.130245
2258.245	19	0.429384	0.3458312	0.120558	0.08368913	0.113876	0.1306865
2258.345	19	0.41372	0.3482162	0.1270835	0.08826246	0.110309	0.132264
2258.445	19	0.419103	0.3493532	0.1244843	0.08558434	0.10744	0.1315611
2258.545	19	0.424525	0.3516232	0.121287	0.08435493	0.106055	0.1281451
2258.645	19	0.412935	0.347667	0.1223068	0.08377247	0.108831	0.1281093
2258.745	19	0.422264	0.3379906	0.1216468	0.08433171	0.108508	0.1269982
2258.845	19	0.418131	0.336154	0.1205894	0.08724348	0.108446	0.1290817
2258.945	19	0.433825	0.3390216	0.1210848	0.08623242	0.108766	0.1330548
2259.045	19	0.438746	0.3403268	0.1194243	0.08854894	0.112132	0.1334384
2259.145	19	0.441326	0.3431266	0.1211829	0.09534659	0.111666	0.1330482
2259.245	19	0.441376	0.3470898	0.1213331	0.1022514	0.111298	0.1331228
2259.345	19	0.455413	0.3474597	0.1204807	0.1027395	0.109555	0.1332153
2259.445	19	0.463593	0.3450347	0.1194657	0.1032081	0.113316	0.134358
2259.545	19	0.450115	0.351143	0.1192207	0.101748	0.113256	0.1359476
2259.645	19	0.445512	0.3471539	0.1183978	0.09412203	0.113151	0.138419
2259.745	19	0.445557	0.3460775	0.1167888	0.09124171	0.118335	0.1414524

2259.845	19	0.444236	0.3379942	0.1203241	0.0900042	0.121026	0.1413948
2259.945	19	0.416843	0.3437486	0.1229978	0.09278432	0.121028	0.1379074
2260.045	19	0.424822	0.3551052	0.1280814	0.09058727	0.124016	0.1345517
2260.145	19	0.459625	0.348641	0.1285603	0.08848147	0.123332	0.1325861
2260.245	19	0.441181	0.349202	0.1331	0.08615182	0.122239	0.1330758
2260.345	19	0.451378	0.345506	0.129648	0.08640002	0.120032	0.1328818
2260.445	19	0.452557	0.3434144	0.1262916	0.08558422	0.122411	0.1353289
2260.545	19	0.445246	0.341811	0.1260563	0.085251	0.120955	0.138359
2260.645	19	0.437103	0.3370404	0.1224272	0.08440255	0.118653	0.1366463
2260.745	19	0.408355	0.3416897	0.1191633	0.08163176	0.117152	0.1335556
2260.845	19	0.407832	0.3386129	0.116538	0.08120137	0.115702	0.1331037
2260.945	19	0.410109	0.3404911	0.1183792	0.0806162	0.116368	0.1284521
2261.045	19	0.421337	0.331966	0.1159065	0.07950625	0.114749	0.1283676
2261.145	19	0.426219	0.3317164	0.1156409	0.08135512	0.11342	0.1292387
2261.245	19	0.419592	0.3352012	0.1164601	0.08050193	0.113531	0.1304971
2261.345	19	0.42392	0.3328608	0.1188094	0.08002703	0.112397	0.1296859
2261.445	19	0.419168	0.3336105	0.1189964	0.08252028	0.11071	0.130479
2261.545	19	0.40925	0.3287758	0.1200126	0.08434079	0.1077	0.1310979
2261.645	19	0.417428	0.3391068	0.1198939	0.08384646	0.106033	0.1292358
2261.745	19	0.416938	0.3276311	0.1227129	0.08373114	0.105678	0.1305646
2261.845	19	0.410821	0.3210793	0.1227533	0.08366977	0.104622	0.1302835
2261.945	19	0.419866	0.3232802	0.1205427	0.08337194	0.104631	0.1296998
2262.045	19	0.421785	0.3237964	0.1182795	0.08205315	0.106916	0.1299593
2262.145	19	0.447516	0.3260985	0.1176282	0.08248099	0.10835	0.1291806
2262.245	19	0.43406	0.3310061	0.1172655	0.086493	0.108123	0.1319234
2262.345	19	0.444304	0.3369994	0.1184067	0.09229375	0.106918	0.132835
2262.445	19	0.443752	0.3411442	0.1191305	0.0959589	0.108009	0.1306392
2262.545	19	0.466824	0.3490435	0.1198353	0.09931475	0.108644	0.1328943
2262.645	19	0.4541	0.3531168	0.1174436	0.1014672	0.113232	0.1353243
2262.745	19	0.443538	0.3512826	0.1156508	0.09789436	0.114761	0.1366513
2262.845	19	0.447815	0.3508794	0.115562	0.08900417	0.115115	0.1348921
2262.945	19	0.469614	0.3484335	0.1160861	0.08660651	0.114713	0.1351901
2263.045	19	0.445119	0.3515738	0.1149348	0.08734494	0.116085	0.1361652
2263.145	19	0.426534	0.3532004	0.1149682	0.08754135	0.120663	0.1390751
2263.245	19	0.458968	0.3486248	0.1212099	0.083688	0.128225	0.1390976
2263.345	19	0.451484	0.3473317	0.1222333	0.08278263	0.124023	0.1377374
[truncated: 664,879 more chars]
